# Cytochrome P450 Monooxygenase CYP139 Family Involved in the Synthesis of Secondary Metabolites in 824 Mycobacterial Species

**DOI:** 10.3390/ijms20112690

**Published:** 2019-05-31

**Authors:** Puleng Rosinah Syed, Wanping Chen, David R. Nelson, Abidemi Paul Kappo, Jae-Hyuk Yu, Rajshekhar Karpoormath, Khajamohiddin Syed

**Affiliations:** 1 Department of Pharmaceutical Chemistry, College of Health Sciences, University of KwaZulu-Natal, Durban 4000, South Africa; prosinah@gmail.com; 2 College of Food Science and Technology, Huazhong Agricultural University, Wuhan 430070, China; chenwanping@mail.hzau.edu.cn; 3 Department of Microbiology, Immunology and Biochemistry, University of Tennessee Health Science Center, Memphis, TN 38163, USA; drnelson1@gmail.com; 4 Department of Biochemistry and Microbiology, Faculty of Science and Agriculture, University of Zululand, KwaDlangezwa 3886, South Africa; KappoA@unizulu.ac.za; 5 Department of Bacteriology, University of Wisconsin-Madison, 3155 MSB, 1550 Linden Drive, Madison, WI 53706, USA; jyu1@wisc.edu; 6 Department of Systems Biotechnology, Konkuk University, Seoul 05029, Korea

**Keywords:** biosynthetic gene clusters, cytochrome P450 monooxygenase, CYP139A1, genome data mining, host metabolism, *Mycobacterium tuberculosis*, polyketides, secondary metabolites, tuberculosis

## Abstract

Tuberculosis (TB) is one of the top infectious diseases causing numerous human deaths in the world. Despite enormous efforts, the physiology of the causative agent, *Mycobacterium tuberculosis*, is poorly understood. To contribute to better understanding the physiological capacity of these microbes, we have carried out extensive in silico analyses of the 1111 mycobacterial species genomes focusing on revealing the role of the orphan cytochrome P450 monooxygenase (CYP) CYP139 family. We have found that CYP139 members are present in 894 species belonging to three mycobacterial groups: *M. tuberculosis* complex (850-species), *Mycobacterium avium* complex (34-species), and non-tuberculosis mycobacteria (10-species), with all CYP139 members belonging to the subfamily “A”. CYP139 members have unique amino acid patterns at the CXG motif. Amino acid conservation analysis placed this family in the 8th among CYP families belonging to different biological domains and kingdoms. Biosynthetic gene cluster analyses have revealed that 92% of CYP139As might be associated with producing different secondary metabolites. Such enhanced secondary metabolic potentials with the involvement of CYP139A members might have provided mycobacterial species with advantageous traits in diverse niches competing with other microbial or viral agents, and might help these microbes infect hosts by interfering with the hosts’ metabolism and immune system.

## 1. Introduction

Tuberculosis (TB), a prehistoric disease, remains one of the top 10 causes of death and the leading cause from a single infectious agent, *Mycobacterium tuberculosis*, despite global efforts in disease control programs during the past 20 years [1]. TB is a global disease, found in every country in the world [1]. It became mankind’s oldest and worst enemy owing to its widespread nature across the world and developing resistance to known and available drugs [1]. In 2017, 10 million people developed TB, and an estimated 1.3 million deaths among human immunodeficiency virus (HIV)-negative people and an additional 300,000 deaths from TB among HIV-positive people occurred [1]. The latest data from the Statistics South Africa show that TB is one of the top killers in South Africa [2], suggesting an urgent need to understand *M. tuberculosis* physiology to be able to come up with novel drugs and drug targets.

Despite living in the most advanced medicine era, TB remains a major threat to human health [1]. After 21 years of *M. tuberculosis* genome sequencing [3], to date its physiology is poorly understood and many proteins remain orphans. Genome sequencing analysis of *M. tuberculosis* H37Rv revealed the presence of 20 cytochrome P450 monooxygenases (CYPs/P450s) in its genome [3]. P450s are mixed function oxidoreductases ubiquitously distributed across the biological kingdoms [4]. P450s are well known for their role in essential cellular anabolic and catabolic processes.

Among 20 P450s, to date, the role of only six *M. tuberculosis* H37Rv P450s in its physiology have been elucidated [5]. CYP51B1, highly conserved P450 family across microbes, has been found to catalyse the 14α-demethylation of lanosterol [6,7,8]; CYP121A1 catalyses oxidative crosslinking of the two tyrosines in a cyclodipeptide [9]; CYP125A1 and CYP142A1 catalyse the 26-hydroxylation of cholesterol and cholest-4-en-3-one [10,11]; CYP124A1 catalyses the terminal hydroxylation of methyl-branched hydrocarbons such as those of phytanic acid and farnesol [12], cholesterol and related sterols [10,13], and vitamin D_3_ and CYP128A1 is involved in oxidation of menaquinone MK9 [14].

Among *M. tuberculosis* H37Rv P450s, the *CYP139A1* gene was found downstream of polyketide synthase genes (*pks10*, *pks7*, *pks8*, *pks17*, *pks9* and *pks11*) and situated next to macrolide transport protein [15,16]. Two of the polyketide synthases, *pks7* and *pks8*, were found to be essential for the survival of *M. tuberculosis* [17,18]. Polyketide synthases along with other genes were found to be part of biosynthetic gene clusters (BGCs). As per Medema et al. [19], a BGC can be defined as a physically clustered group of two or more genes in a particular genome that together encode a biosynthetic pathway for the production of a specialised metabolite (including its chemical variants). Bacteria, fungi and plants are known to possess different types of BGCs producing a variety of secondary metabolites that are beneficial to humans. Among the genes that are part of a BGC, P450s play a key role in contributing to the diversity of a secondary metabolite owing to their regio and stereo-specific oxidation [20]. Recently, comprehensive comparative analysis of P450s and those associated with secondary metabolism revealed a large number of P450s involved in the production of secondary metabolites in different bacterial species [21,22].

Based on *CYP139A1* location, this P450 is assumed to be involved in oxidative tailoring of the macrolide structure. In the latest study, involving comprehensive comparative analysis of P450s in bacterial species belonging to the genera *Mycobacterium* and *Streptomyces*, CYP139 P450s were found to be dominantly located in different secondary metabolite BGCs [22]. This strongly indicates that CYP139 P450s are possibly involved in the synthesis of secondary metabolites. This study is aimed at using an in silico approach to unravel the CYP139 P450 family’s role in mycobacterial species physiology.

## 2. Results and Discussion

### 2.1. CYP139 P450s Are Present Only in Certain Mycobacterial Category Species

**Figure 1:**
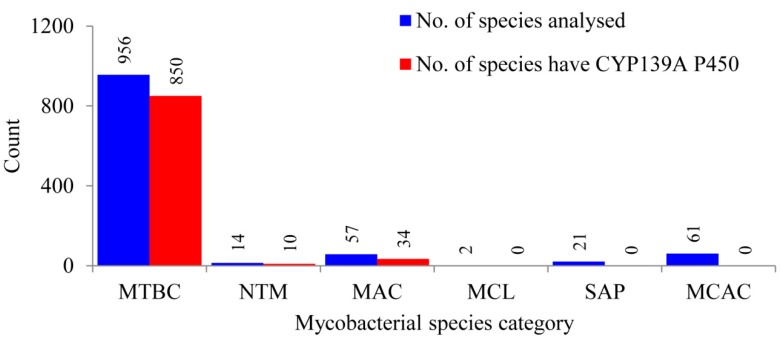
Comparative analysis of CYP139A P450s in species belonging to six different mycobacterial categories. Abbreviations: MTBC, *Mycobacterium tuberculosis* complex; MAV, *M. avium* complex; NTM, non-tuberculosis mycobacteria; MCL, *Mycobacterium* causing leprosy; SAP, Saprophytes and MCAC, *Mycobacterium chelonae-abscessus* complex. Information on mycobacterial species and CYP139A P450s is presented in Supplementary Tables S1 and S2, respectively.

Comprehensive comparative analysis of CYP139 P450s in 1111 mycobacterial species belonging to six different categories (Table S1) revealed that CYP139 P450s are present in 894 mycobacterial species belonging to three categories, namely the *Mycobacterium tuberculosis* complex (MTBC), *M. avium* complex (MAV) and non-tuberculosis mycobacteria (NTM) (Figure 1 and Table S2). This phenomenon of identifying CYP139 P450s only in these three mycobacterial categories was also observed previously when 60 mycobacterial species were analysed [23]. Results from this study, which involved such a large data set, not only supported, but also confirmed that mycobacterial species belonging to categories such as *Mycobacterium* causing leprosy (MCL), Saprophytes (SAP) and the *Mycobacterium chelonae-abscessus* complex (MCAC) do not have CYP139 P450s in their genomes, as seen in Figure 1. Interestingly, not all mycobacterial species of MTBC, NTM and MAC categories have CYP139 P450 (Figure 1). Among 956 mycobacterial species, only 850 mycobacterial species of MTBC have CYP139 P450; 10 of 14 and 34 of 57 mycobacterial species of NTM and MAC, respectively, have this P450 (Figure 1 and Table S2). A detailed analysis of CYP139 P450s along with species names and protein ID is presented in Table S2 and the CYP139 P450 sequences are presented in Supplementary Dataset 1.

**Figure 2:**
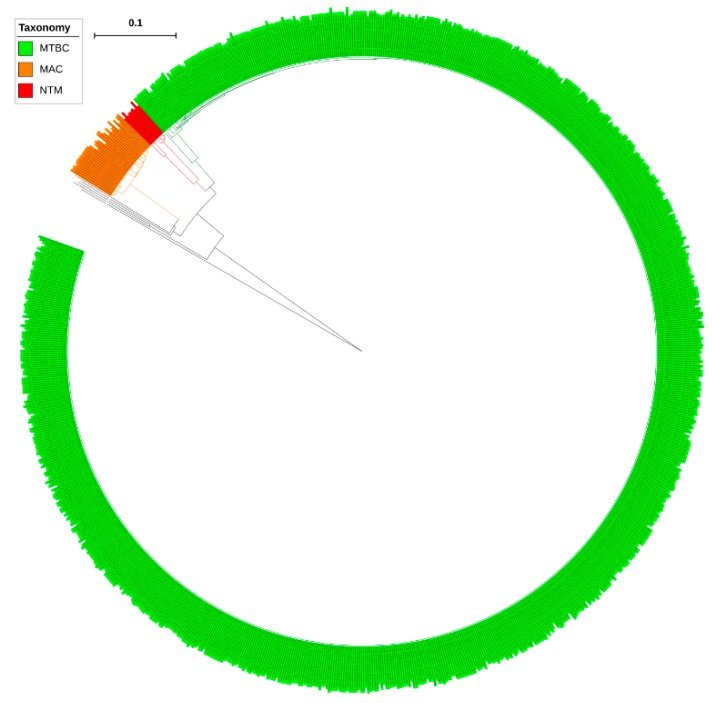
Phylogenetic analysis of CYP139A P450s. Different mycobacterial categories were indicated in different colours. CYP51B1 from *Mycobacterium tuberculosis* H37Rv is used as an outgroup. Abbreviations: MTBC, *Mycobacterium tuberculosis* complex; MAV, *M. avium* complex; NTM, non-tuberculosis mycobacteria. A high-resolution phylogenetic tree is provided in Supplementary Figure S1.

Analysis of *CYP139* P450s in the genomes of mycobacterial species revealed that only a single copy of the *CYP139* P450 gene is present in all mycobacterial species (Table S2). Furthermore, P450 subfamily analysis revealed that all CYP139 P450s found in 894 mycobacterial species belong to the subfamily “A” (Figure 2). Phylogenetic analysis of CYP139A P450s revealed that CYP139A P450s grouped per their mycobacterial category, indicating after speciation CYP139A P450s were subjected to amino acid changes specific to their category (Figure 1), similar to what was observed for other P450s described elsewhere [23,24]. However, four CYP139A P450s belonging to *M. genavense* ATCC 51234 and *Mycobacterium sp*. JDM601 of NTM and *Mycobacterium sp*. UM CSW and *M. avium avium* Env 77 of MAC were aligned separately, suggesting that these CYP139A P450s had deviated from their counterparts (Figure 2). Percentage identity among CYP139 P450s further confirmed that CYP139A P450s from these species have a low percentage identity with their counterparts (Supplementary Dataset 2). CYP139A P450s of *Mycobacterium* sp. UM CSW and *M. avium avium* Env 77 have an average of ~77% and ~63% identity, whereas CYP139A P450s of *M. genavense* ATCC 51234 and *Mycobacterium* sp. JDM601 have an average of 75% and 60% with their counterparts (Supplementary Dataset 2) suggesting these P450s have been subjected to significant amino acid changes. The phenomenon of P450s not grouping with their counterpart species was also observed in fungal species, where CYP53D1 has been subjected to extensive amino acid changes [24], the same as what was observed for the four CYP139A P450s identified in this study. Determining the effect of these amino acid changes on functional specificity of four CYP139A P450s, if any, will be interesting future work.

### 2.2. CYP139 P450 Family Ranked among Top 10 P450 Families

Ranking of P450 families belonging to different biological kingdoms, based on the number of conserved amino acids in their protein sequence, placed the CYP139 P450 family in the twelfth rank [23,25]. While ranking the CYP139 P450 family, only 54 CYP139A P450s were used [23,25]. Identification of quite a large number of CYP139A P450s in this study necessitated re-analysis of the ranking of this P450 family. In order to identify the conservation rank, CYP139A P450s were subjected to PROfile Multiple Alignment with Local Structures and 3D constraints (PROMALS3D) [26] analysis (Supplementary Dataset 3). PROMALS3D analysis revealed the presence of 165 amino acids invariantly conserved in CYP139 P450s (Table 1). Comparative analysis with other P450 families from different biological kingdoms revealed that the CYP139 P450 family now occupies the eighth rank compared to the twelfth rank as assigned previously (Table 1).

### 2.3. CYP139 Family Has Unique Amino Acid Patterns at CXG Motif

In a study by Syed and Mashele [28], analysis of the P450 signature motifs, EXXR and CXG, among different P450 families led to the discovery of amino acid patterns characteristic of a P450 family. The authors proposed that “during the divergence of P450 families from a common ancestor, these amino acids patterns evolved and are retained in each P450 family as a signature of that family” [28]. However, in that study, the CYP139 P450 family is not included. Furthermore, identification of a large number of CYP139A P450s, in this study, gives us an opportunity to identify CYP139 P450 family characteristic amino acid patterns at EXXR and CXG motifs, if any.

**Figure 3:**
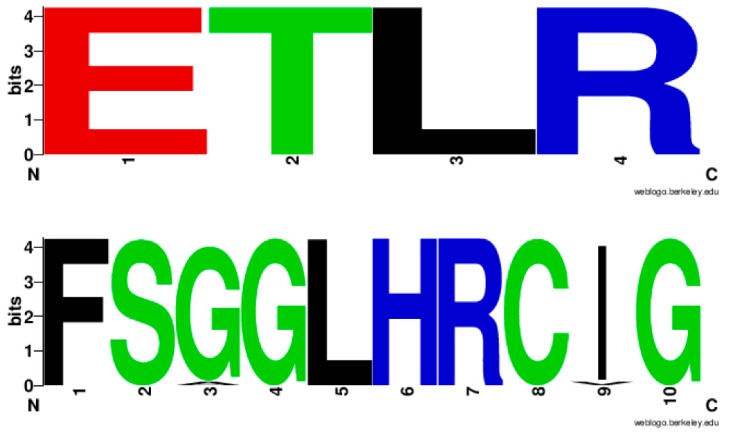
Analysis of amino acid patterns at the EXXR and CXG motif in CYP139 P450 family. In total 894 CYP139 P450 sequences were analysed for EXXR and CXG signature sequences.

Analysis of EXXR and CXG motifs in 894 CYP139A P450s revealed that the CYP139 P450 family EXXR domain is absolutely conserved with amino acid patterns E-T-L-R, whereas, eight amino acids are invariantly conserved in CXG motifs with amino acid patterns of F-S-G(96%)/A(4%)-G-L-H-R-C-I(96%)/V(4%)-G (Figure 3). It is interesting to note that the CYP139 P450 family EXXR motif amino acid pattern absolutely matched with the CYP5 family [28] and amino acid patterns at the CXG motif were unique and not matched with any P450 families described in the literature [25,28,29]. The CYP139 P450 family amino acid patterns at the EXXR and CXG motifs further strongly support the above hypothesis proposed by Syed and Mashele [28].

### 2.4. Most CYP139A P450s Are Part of Secondary Metabolite Biosynthetic Gene Clusters

**Figure 4:**
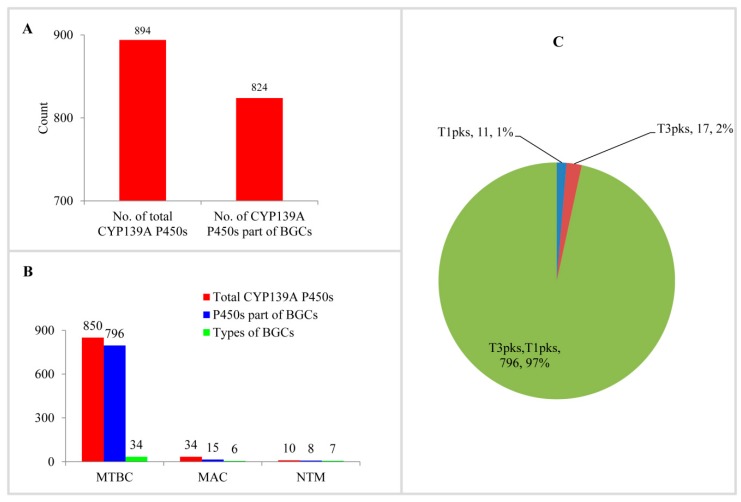
CYP139A P450s secondary metabolite BGCs analysis in mycobacterial species. (**A**) Analysis of CYP139A P450s that are part of BGCs. (**B**) Comparative analysis of CYP139A P450s that are part of BGCs and types of BGCs in different mycobacterial categories. Abbreviations: MTBC, *Mycobacterium tuberculosis* complex; MAV, *M. avium* complex; NTM, non-tuberculosis mycobacteria. (**C**) Comparative analysis of CYP139A P450 cluster types. The type of cluster and the number of CYP139A P450s and their percentage in the total number of P450s were presented in the figure. Abbreviation: T1pks, Type 1 polyketide synthase; T2pks, Type 2 polyketide synthase.

Analysis of CYP139A P450s as part of secondary metabolite BGCs in mycobacterial species revealed that most of the CYP139A P450s are part of different BGCs (Figure 4A and Table S2). Among 894 CYP139A P450s, 824 CYP139A P450s (92%) were found to be part of secondary metabolic BGCs (Figure 4A). This means 70 CYP139A P450s were not found to be part of any secondary metabolite BGCs. Comparison of CYP139A P450s that are part of BGCs in three categories revealed that most of the CYP139A P450s in MTBC and NTM species were part of BGCs, compared to species of MAC, where fewer than half of CYP139A P450s were part of secondary metabolite BGCs (Figure 4B).

**Figure 5:**
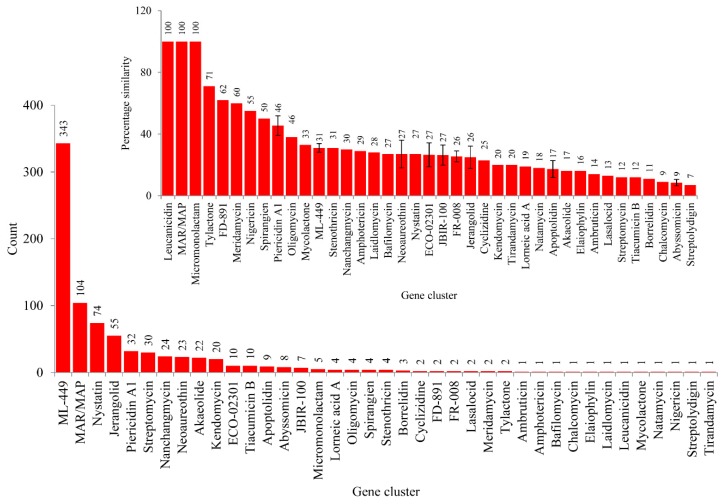
CYP139A P450 gene cluster analysis in mycobacterial species. The 39 gene clusters were presented with their standard abbreviated names as per anti-SMASH. The number next to bars represents the number of CYP139A P450s that is part of that gene cluster. The inset figure shows the percentage identity of CYP139A P450 gene clusters to the known gene clusters available at anti-SMASH. The number next to the bars represents the percentage identity. For some gene clusters the percentage identity is represented with standard deviation (indicated with bars).

Analysis of secondary metabolite BGCs revealed that CYP139A P450s were part of only three different cluster types (Figure 4C and Table S2). Among three different cluster types, CYP139A P450s were found to be present dominantly as part of Type 3-Type 1 polyketide synthase (T3PKS-T1PKS) (97%) compared to T3 PKS (2%) and T1 PKS (1%) (Figure 4C and Table S2). There were 796 CYP139A P450s found to be part of T3PKS-T1PKS, followed by 17 and 11 CYP139 P450s found to be part of T3 PKS and T1 PKS, respectively (Figure 4C and Table S2). Analysis of gene clusters revealed that 824 CYP139A P450s were part of 39 different gene clusters (Figure 4). There were 34 CYP139A P450 gene clusters found in MTBC species, followed by seven gene clusters in NTM species and six gene clusters in MAC (Figure 4B). Among different gene clusters, ML-449 was dominant, with 349 CYP139A P450s followed by methylated alkyl-resorcinol/methylated acyl-phloroglucinol (MAR/MAP) with 104 CYP139A P450s, Nystatin with 74 CYP139A P450s and Jerangolid with 55 CYP139A P450s (Figure 5). Among 39 gene clusters only 11 gene clusters were found to have 10 or more CYP139A P450s (Figure 5). Analysis of DNA sequence percentage identity between CYP139A P450 gene clusters compared to known gene clusters revealed that some of the gene clusters have 100% identity, such as Leucanicidin, MAR/MAP and Micromonolactam (Figure 5), indicating CYP139A P450s are indeed involved in the synthesis of these secondary metabolites.

### 2.5. CYP139A P450s Involved in the Synthesis of Secondary Metabolites in Mycobacterial Species

Comprehensive comparative analysis of CYP139A P450s secondary BGCs in mycobacterial species revealed that CYP139A P450s are indeed involved in the synthesis of different secondary metabolites, as 92% of CYP139A P450s were found to be part of secondary metabolite BGCs (Figure 4 and Figure 5 and Table S2). To understand the role of CYP139A P450s in mycobacterial species’ physiology well, a functional comparison of CYP139A P450s gene clusters’ homolog secondary metabolites was carried out (Table 2). As shown in Table 2, it is clear that CYP139A P450s are involved in the production of chemicals that have antibacterial, antifungal, antiviral and antitumor properties. Interestingly, some of these metabolites in fact showed antimycobacterial activity (Table 2). This indicates that CYP139A P450s are possibly helping mycobacterial species to kill other bacteria, including other mycobacterial species, thus gaining the upper hand in the niche area for their survival. It is interesting to note that CYP139A P450s are present only in MTBC, NTM and MAC categories, but not present in SAP, MCAC or MCL. This necessitates understanding its role in mycobacterial species when they are surviving in hosts such as humans or other animals. In this direction, analysis of some secondary metabolite functions pointed out that some secondary metabolites are certainly helping mycobacterial species to survive in their hosts. For example, MAR/MAP BGC products are found to be part of the cell envelope in *M. marinum*, possibly complicating its access to host immune system or drug actions [30]; Akaeolide has cytotoxic activity against fibroblasts, suggesting it may play a role in tissue weakening in the host [31]; JBIR-100 exhibits cytotoxic activities and inhibition of proton pumps such as vacuolar-type ATPases (V-ATPases) activities and is thus linked with an increasing number of diseases such as osteopetrosis, male infertility and renal acidosis [32,33]. Lorneic acid A inhibits phosphodiesterase PDE5 blocking the degradation of cGMP [34] and thus it might be playing a role in pulmonary hypertension. Meridamycin has been found to bind FK506-binding proteins (FKBP12) [35]. FKBP12 proteins play a key role in regulating fundamental aspects of cell biology and have been found to be critical in mice survival [36]. Nigericin inhibits the Golgi functions in eukaryotic cells and is a well-known activator of the NLRP3 inflammasome [37,38,39], indicating bacterial infection. One secondary metabolite, namely mycolactone, a lipid-like toxin with cytotoxic, immunosuppressive and tissue necrosis activity, has been shown to be involved in the development of Buruli ulcer by *M. ulcerans* [40].

## 3. Materials and Methods

### 3.1. Mycobacterial Species and Genome Databases

In total, 1111 mycobacterial species genomes that are available for public use (as of 12 June 2018) at Integrated Microbial Genomes & Microbiomes (IMG/M) [68] were used in the study (Table S1). Mycobacterial species used in the study, along with their name, genome ID and individual genome database links, were presented in Table S1.

### 3.2. Genome Data Mining and Annotation of CYP139 P450s

The *M. tuberculosis* H37Rv CYP139A1 (Rv1666c) P450 sequence has been blasted with the default settings against individual mycobacterial species genomes at IMG/M [68]. However, each time, only 20 mycobacterial species were selected for BLAST analysis. The hit proteins with more than 40% identity were selected and then subjected to BLAST analysis at the P450 BLAST server (https://ksyed.weebly.com/p450-blast.html) to identify the homolog P450. Hit proteins were then grouped into families and subfamilies based on the International Cytochrome P450 Nomenclature criteria, i.e., P450s showing >40% identity were assigned to the same P450 family and P450s that showed >55% identity were grouped under the same P450 subfamily [69,70,71]. Protein with more than 90% identity considered as ortholog and assigned the same subfamily number.

### 3.3. Phylogenetic Analysis of CYP139A P450s

The phylogenetic tree of CYP139 family members was built with *M. tuberculosis* CYP51B1 (Rv0764c) protein as outgroup. First, the protein sequences were aligned by MAFFT v6.864 [72], embedded on the Trex web serve [73]. Then, the alignments were automatically subjected to infer the best tree by the Trex web server with its embedded weighting procedure. Finally, the tree was visualised and colored by iTOL (http://itol.embl.de/about.cgi) [74].

### 3.4. Analysis of Homology and Amino Acid Conservation

Analysis of percentage identity among CYP139A P450s from species belonging to MAC and NTM categories was carried out as described elsewhere [23,29]. Briefly, the percentage identity between CYP139 P450s was determined using the Clustal Omega [75]. The Clustal Omega percentage identity matrix was downloaded and pasted into an Excel sheet by converting the text into a column option.

Amino acid conservation among CYP139A P450s was carried out following the method described elsewhere [23,25,29]. Briefly, CYP139 P450s were subjected to PROMALS3D [26] to identify invariantly conserved amino acids [27]. The conservation index follows numbers from 5–9, where 9 is the invariantly conserved amino acid across the sequences. The total number of conserved residues indicated by number 9 was recorded. The conserved nature of the CYP139 family was compared to other P450 families from different biological kingdoms, as reported elsewhere [23,25].

### 3.5. Generation of EXXR and CXG Sequence Logo

CYP139 P450 family EXXR and CXG sequence logos were generated following the method described elsewhere [25,28,29]. Briefly, CYP139 P450 sequences were aligned using ClustalW multiple alignments using MEGA7 [76]. After sequence alignment the EXXR and CXG region amino acids (4 and 10 amino acids, respectively), were selected and entered in the WebLogo program (http://weblogo.berkeley.edu/logo.cgi). As a selection parameter, the image format was selected as PNG (bitmap) at 300 dpi resolution. The percentage predominance of amino acids at particular positions is calculated considering the total number of amino acids as 100%. The generated EXXR and CXG logos were used for analysis and compared to the different P450 family EXXR and CXG logos that have been published and are available to the public [25,28,29].

### 3.6. Identification of CYP139 P450 Secondary Metabolite BGCs

BGCs listed on the IMG/M [68] website for each of the mycobacterial species were manually searched for the presence of CYP139 P450s using the protein ID. The BGCs that have CYP139 P450 were selected for further study. The listed BGCs at IMG/M are general [68] and in order to identify the specific type of BGCs, the selected BGCs genome sequences were subjected to secondary metabolite BGCs analysis, as described elsewhere [21]. Briefly, the individual BGC genome sequences downloaded from IMG/M [68] were submitted to anti-SMASH [77]. The type of BGC, percentage similarity to a known cluster and the cluster name were noted. Standard BGC abbreviation terminology developed by anti-SMASH [77] was used in the study.

## 4. Conclusions

The advancement of genome sequencing and bioinformatics tools helps significantly in understanding the role of orphan proteins in organisms. This study is an attempt to utilize the availability of quite a large number of mycobacterial species genome sequences and different bioinformatics tools to understand the role of the orphan CYP139 family in mycobacterial species. This study revealed that the CYP139 family indeed plays a role in the synthesis of secondary metabolites in mycobacterial species. Based on the functions of homolog CYP139 P450 gene clusters’ secondary metabolites, it can be assumed that these metabolites indeed help mycobacterial species to survive in the host, being part of the cell envelope and inhibiting fibroblast, thus causing tissue weakening and causing ulcers via tissue necrosis. The metabolites that exhibit antibacterial (including antimycobacterial), antifungal and antiviral properties certainly help mycobacterial species to gain the upper hand in the niche area compared to those agents. It would be interesting to determine the roles of CYP139A P450s that are not part of gene clusters. Predictions made in the study are based on the functions of homolog secondary metabolites. However, wet laboratory biosynthesis and functional analysis of secondary metabolites should be carried out to understand the role of these metabolites in mycobacterial physiology. Study results can be used as a reference for future experimental studies.

## Figures and Tables

**Table 1:** Comparative amino acid conservation analysis of CYP139 P450 family with top 10 ranked P450 families [23,25]. The conservation index score is obtained as described in the section on materials and methods, following the procedure described elsewhere [27]. The conservation score (5–9) obtained via PROMALS3D is presented in the table, where the number 9 indicates invariantly conserved amino acids in P450 members. P450 families were arranged from the highest to the lowest number of amino acids conserved. CYP139 P450 family is indicated in bold.

P450 Family	Number of Member P450s	Kingdom	PROMALS3D Conservation Index					Rank (Highest to Lowest Conservation)
			5	6	7	8	9	
CYP141	29	Bacteria	0	0	0	0	389	1
CYP51	50	Bacteria	11	102	0	0	264	2
CYP137	38	Bacteria	145	0	0	0	251	3
CYP121	34	Bacteria	0	0	0	0	233	4
CYP132	39	Bacteria	175	0	0	0	217	5
CYP5619	23	Stramenopila (oomycetes)	118	38	170	0	199	6
CYP124	71	Bacteria	52	35	59	0	170	7
**CYP139**	**894**	**Bacteria**	**0**	**127**	**0**	**0**	**165**	**8 (formerly 12)**
CYP188	67	Bacteria	62	0	100	0	141	9
CYP123	74	Bacteria	62	0	82	0	137	10

**Table 2:** Functional analysis of homolog CYP139A P450 gene clusters.

Gene Cluster	Function	Reference
ML-449	Macrolactam antifungal-antibiotic production.	[41]
MAR/MAP	Synthesis of methylated alkyl-resorcinol and methylated acyl-phloroglucinol products found to be part of cell envelope in *M. marinum*.	[30]
Nystatin	Polyene antifungal antibiotic.	[42]
Jerangolid	Antifungal polyketide.	[43]
Piericidin A1	A member of α-pyridone antibiotics, exhibits various biological activities such as antimicrobial, antifungal, and antitumour properties and possesses potent respiration-inhibitory activity against insects owing to its competitive binding capacity to mitochondrial complex I.	[44]
Streptomycin	Antibiotic used to treat bacterial infections, including tuberculosis.	[45]
Nanchangmycin	A polyether ionophore antibiotic produced by *Streptomyces nanchangensis* NS3226 that has insecticidal and in vitro antibacterial properties. Nanchangmycin exhibits antiviral properties against the Zika virus.	[46,47,48]
Neoaureothin	Neoaureothin is an unusual chain-extended analog of aureothin. It was first reported as a co-metabolite of neoantimycin in *Streptomyces orinoci*. It has been reported to have anti-HIV and antifungal activity.	[49]
Akaeolide	A carbocyclic polyketide with moderate antimicrobial activity and cytotoxicity to rat fibroblasts.	[31]
Kendomycin	Macrolide antibiotic with antibacterial activity.	[50]
ECO-02301	Antifungal agent.	[51]
Tiacumicin B	Macrolide antibiotic, which is used for the treatment of *Clostridium difficile* infections.	[52,53]
Apoptolidin	Macrolide antibiotic well known as apoptosis inducer and inhibitor of F0F1-ATPase. It is a promising new therapeutic lead that exhibits remarkable selectivity against cancer cells relative to normal cells.	[54,55,56]
Abyssomicin	A novel spirotetronate polyketide Class I antimicrobial. The biological activity of abyssomicins includes their antimicrobial activity against Gram-positive bacteria and mycobacteria, antitumour properties, latent HIV reactivator, anti-HIV and HIV replication inducer properties	[57]
JBIR-100	A new 16-membered tetraene macrolide from the *Streptomyces* species. Its structure is identical to TS155-2, which is an inhibitor of the thrombin-induced calcium influx. It exhibits cytotoxic and V-ATPases inhibition activities. V-ATPases are ubiquitous proton pumps present in the endomembrane system of all eukaryotic cells and in the plasma membranes of many animal cells that have been correlated with an increasing number of diseases such as osteopetrosis, male infertility and renal acidosis.	[32,33]
Micromonolactam	A new polyene macrolactam antibiotic	[58]
Lorneic acid A	It has a fatty acid-like structure in which a benzene ring is embedded. It inhibits phosphodiesterases (PDE) with selectivity toward PDE5, thus, blocking the degradation of cGMP and having a possible linkage to pulmonary hypertension	[34]
Leucanicidin	A potent nematocide and insecticide macrolide	[59]
Oligomycin	A natural antibiotic that inhibits mitochondrial ATP synthase, thus affecting the electron transport chain.	[60]
Spirangien	Highly cytotoxic and antifungal spiroketal	[61]
Stenothricin	A peptide antibiotic inhibiting bacterial cell wall synthesis	[62]
Borrelidin	A small molecule nitrile-containing macrolide, which is an inhibitor of bacterial and eukaryal threonyl-tRNA synthetase. It exhibits among others antibacterial and anti-angiogenesis activities, suppresses growth and induces apoptosis in malignant acute lymphoblastic leukemia cells.	[63,64]
FD-891	Profoundly blocked both perforin- and FasL-dependent cytotoxicity by cytotoxic T lymphocytes—immunosuppressive.	[65]
FR-008	Macrolide antibiotic with antifungal activity.	[66]
Meridamycin	A 27-membered macrolide that acts as non-immunosuppressive FK506-binding proteins (FKBP12) ligand.	[35]
Ambruticin	Antifungal polyketide	[67]
Nigericin	Nigericin acts as an H^+^, K^+^, Pb^2+^ ionophore. Most commonly it is an antiporter of H^+^ and K^+^. In the past nigericin was used as an antibiotic active against Gram-positive bacteria. It inhibits Golgi functions in eukaryotic cells. Its ability to induce K^+^ efflux also makes it a potent activator of the NLRP3 inflammasome.	[37,38,39]
Mycolactone	Lipid-like toxin with cytotoxic, immunosuppressive and tissue necrosis activity. It plays a key role in the development of Buruli ulcer by *M. ulcerans*.	[40]

## Supplementary Materials

Supplementary materials can be found at https://www.mdpi.com/1422-0067/20/11/2690/s1.
