# Supplementary material for: Cytochrome P450 Monooxygenase CYP139 Family Involved in the Synthesis of Secondary Metabolites in 824 Mycobacterial Species
Source: Int J Mol Sci. 2019 May 31;20(11):2690. doi: 10.3390/ijms20112690 (PMC6600245; doi:10.3390/ijms20112690)
Supplement: Supplementary file 1 [file ijms-20-02690-s001.zip › Supplementary Information/Supplementary Dataset 1.docx]

*Article*

**Cytochrome P450 monooxygenase CYP139 family involved in the synthesis of secondary metabolites in 824 mycobacterial species**

**Puleng Rosinah Syed ^1^, Wanping Chen ^2^, David R Nelson ^3^, Abidemi Paul Kappo ^4^, Jae-Hyuk Yu ^5,6^, Rajshekhar Karpoormath ^1^*, Khajamohiddin Syed ^4,^***

^1^ Department of Pharmaceutical Chemistry, College of Health Sciences, University of KwaZulu-Natal, Durban 4000, South Africa; prosinah@gmail.com (P.R.S.); Karpoormath@ukzn.ac.za (R.K.)

^2^ College of Food Science and Technology, Huazhong Agricultural University, Wuhan 430070, Hubei Province, China; chenwanping@mail.hzau.edu.cn

^3^ Department of Microbiology, Immunology and Biochemistry, University of Tennessee Health Science Center, Memphis, TN, 38163; drnelson1@gmail.com

^4^ Department of Biochemistry and Microbiology, Faculty of Science and Agriculture, University of Zululand, KwaDlangezwa 3886, South Africa; KappoA@unizulu.ac.za (A.P.K.); khajamohiddinsyed@gmail.com (K.S.)

^5^ Department of Bacteriology, University of Wisconsin-Madison, 3155 MSB, 1550 Linden Drive, Madison, WI 53706, USA; [jyu1@wisc.edu](mailto:jyu1@wisc.edu)

^6^ Department of Systems Biotechnology, Konkuk University, Seoul, 05029, Republic of Korea

**Supplementary Dataset 1: CYP139A P450 sequences identified and annotated in mycobacterial species.** Each CYP139A P450 is presented with its gene ID from Integrated Microbial Genomes and Microbiomes [1] in parenthesis followed by species name.

>CYP139A1(2576388909)Mycobacterium tuberculosis TKK_04_0003

MRYPLGEALLALYRWRGPLINAGVGGHGYTYLLGAEANRFVFANADAFSWSQTFESLVPV

DGPTALIVSDGADHRRRRSVVAPGLRHHHVQRYVATMVSNIDTVIDGWQPGQRLDIYQEL

RSAVRRSTAESLFGQRLAVHSDFLGEQLQPLLDLTRRPPQVMRLQQRVNSPGWRRAMAAR

KRIDDLIDAQIADARTAPRPDDHMLTTLISGCSEEGTTLSDNEIRDSIVSLITAGYETTS

GALAWAIYALLTVPGTWESAASEVARVLGGRVPAADDLSALTYLNGVVHETLRLYSPGVI

SARRVLRDLWFDGHRIRAGRLLIFSAYVTHRLPEIWPEPTEFRPLRWDPNAADYRKPAPH

EFIPFSGGLHRCIGAVMATTEMTVILARLVARAMLQLPAQRTHRIRAANFAALRPWPGLT

VEIRKSAPAQ

>CYP139A1(2577593438)Mycobacterium tuberculosis TKK_05MA_0033

MRYPLGEALLALYRWRGPLINAGVGGHGYTYLLGAEANRFVFANADAFSWSQTFESLVPV

DGPTALIVSDGADHRRRRSVVAPGLRHHHVQRYVATMVSNIDTVIDGWQPGQRLDIYQEL

RSAVRRSTAESLFGQRLAVHSDFLGEQLQPLLDLTRRPPQVMRLQQRVNSPGWRRAMAAR

KRIDDLIDAQIADARTAPRPDDHMLTTLISGCSEEGTTLSDNEIRDSIVSLITAGYETTS

GALAWAIYALLTVPGTWESAASEVARVLGGRVPAADDLSALTYLNGVVHETLRLYSPGVI

SARRVLRDLWFDGHRIRAGRLLIFSAYVTHRLPEIWPEPTEFRPLRWDPNAADYRKPAPH

EFIPFSGGLHRCIGAVMATTEMTVILARLVARAMLQLPAQRTHRIRAANFAALRPWPGLT

VEIRKSAPAQ

>CYP139A1(2577803488)Mycobacterium tuberculosis M1283

MRYPLGEALLALYRWRGPLINAGVGGHGYTYLLGAEANRFVFANADAFSWSQTFESLVPV

DGPTALIVSDGADHRRRRSVVAPGLRHHHVQRYVATMVSNIDTVIDGWQPGQRLDIYQEL

RSAVRRSTAESLFGQRLAVHSDFLGEQLQPLLDLTRRPPQVMRLQQRVNSPGWRRAMAAR

KRIDDLIDAQIADARTAPRPDDHMLTTLISGCSEEGTTLSDNEIRDSIVSLITAGYETTS

GALAWAIYALLTVPGTWESAASEVARVLGGRVPAADDLSALTYLNGVVHETLRLYSPGVI

SARRVLRDLWFDGHRIRAGRLLIFSAYVTHRLPEIWPEPTEFRPLRWDPNAADYRKPAPH

EFIPFSGGLHRCIGAVMATTEMTVILARLVARAMLQLPAQRTHRIRAANFAALRPWPGLT

VEIRKSAPAQ

>CYP139A1(2581355094)Mycobacterium bovisWt 21419

MRYPLGEALLALYRWRGPLINAGVGGHGYTYLLGAEANRFVFANADAFSWSQTFESLVPV

DGPTALIVSDGADHRRRRSVVAPGLRHHHVQRYVATMVSNIDTVIDGWQPGQRLDIYQEL

RSAVRRSTAESLFGQRLAVHSDFLGEQLQPLLDLTRRPPQVMRLQQRVNSPGWRRAMAAR

KRIDDLIDAQIADARTAPRPDDHMLTTLISGCSEEGTTLSDNEIRDSIVSLITAGYETTS

GALAWAIYALLTVPGTWESAASEVARVLGGRVPAADDLSALTYLNGVVHETLRLYSPGVI

SARRVLRDLWFDGHRIRAGRLLIFSAYVTHRLPEIWPEPTEFRPLRWDPNAADYRKPAPH

EFIPFSGGLHRCIGAVMATTEMTVILARLVARAMLQLPAQRTHRIRAANFAALRPWPGLT

VEIRKSAPAQ

>CYP139A1(2584983051)Mycobacterium tuberculosis M1415

MRYPLGEALLALYRWRGPLINAGVGGHGYTYLLGAEANRFVFANADAFSWSQTFESLVPV

DGPTALIVSDGADHRRRRSVVAPGLRHHHVQRYVATMVSNIDTVIDGWQPGQRLDIYQEL

RSAVRRSTAESLFGQRLAVHSDFLGEQLQPLLDLTRRPPQVMRLQQRVNSPGWRRAMAAR

KRIDDLIDAQIADARTAPRPDDHMLTTLISGCSEEGTTLSDNEIRDSIVSLITAGYETTS

GALAWAIYALLTVPGTWESAASEVARVLGGRVPAADDLSALTYLNGVVHETLRLYSPGVI

SARRVLRDLWFDGHRIRAGRLLIFSAYVTHRLPEIWPEPTEFRPLRWDPNAADYRKPAPH

EFIPFSGGLHRCIGAVMATTEMTVILARLVARAMLQLPAQRTHRIRAANFAALRPWPGLT

VEIRKSAPAQ

>CYP139A1(2584987406)Mycobacterium tuberculosis TRUG0117

MRYPLGEALLALYRWRGPLINAGVGGHGYTYLLGAEANRFVFANADAFSWSQTFESLVPV

DGPTALIVSDGADHRRRRSVVAPGLRHHHVQRYVATMVSNIDTVIDGWQPGQRLDIYQEL

RSAVRRSTAESLFGQRLAVHSDFLGEQLQPLLDLTRRPPQVMRLQQRVNSPGWRRAMAAR

KRIDDLIDAQIADARTAPRPDDHMLTTLISGCSEEGTTLSDNEIRDSIVSLITAGYETTS

GALAWAIYALLTVPGTWESAASEVARVLGGRVPAADDLSALTYLNGVVHETLRLYSPGVI

SARRVLRDLWFDGHRIRAGRLLIFSAYVTHRLPEIWPEPTEFRPLRWDPNAADYRKPAPH

EFIPFSGGLHRCIGAVMATTEMTVILARLVARAMLQLPAQRTHRIRAANFAALRPWPGLT

VEIRKSAPAQ

>CYP139A1(2589032800)Mycobacterium tuberculosis TBR5

MRYPLGEALLALYRWRGPLINAGVGGHGYTYLLGAEANRFVFANADAFSWSQTFESLVPV

DGPTALIVSDGADHRRRRSVVAPGLRHHHVQRYVATMVSNIDTVIDGWQPGQRLDIYQEL

RSAVRRSTAESLFGQRLAVHSDFLGEQLQPLLDLTRRPPQVMRLQQRVNSPGWRRAMAAR

KRIDDLIDAQIADARTAPRPDDHMLTTLISGCSEEGTTLSDNEIRDSIVSLITAGYETTS

GALAWAIYALLTVPGTWESAASEVARVLGGRVPAADDLSALTYLNGVVHETLRLYSPGVI

SARRVLRDLWFDGHRIRAGRLLIFSAYVTHRLPEIWPEPTEFRPLRWDPNAADYRKPAPH

EFIPFSGGLHRCIGAVMATTEMTVILARLVARAMLQLPAQRTHRIRAANFAALRPWPGLT

VEIRKSAPAQ

>CYP139A1(2592403099)Mycobacterium tuberculosis TKK_02_0022

MRYPLGEALLALYRWRGPLINAGVGGHGYTYLLGAEANRFVFANADAFSWSQTFESLVPV

DGPTALIVSDGADHRRRRSVVAPGLRHHHVQRYVATMVSNIDTVIDGWQPGQRLDIYQEL

RSAVRRSTAESLFGQRLAVHSDFLGEQLQPLLDLTRRPPQVMRLQQRVNSPGWRRAMAAR

KRIDDLIDAQIADARTAPRPDDHMLTTLISGCSEEGTTLSDNEIRDSIVSLITAGYETTS

GALAWAIYALLTVPGTWESAASEVARVLGGRVPAADDLSALTYLNGVVHETLRLYSPGVI

SARRVLRDLWFDGHRIRAGRLLIFSAYVTHRLPEIWPEPTEFRPLRWDPNAADYRKPAPH

EFIPFSGGLHRCIGAVMATTEMTVILARLVARAMLQLPAQRTHRIRAANFAALRPWPGLT

VEIRKSAPAQ

>CYP139A1(2592422247)Mycobacterium tuberculosis TKK_02_0016

MRYPLGEALLALYRWRGPLINAGVGGHGYTYLLGAEANRFVFANADAFSWSQTFESLVPV

DGPTALIVSDGADHRRRRSVVAPGLRHHHVQRYVATMVSNIDTVIDGWQPGQRLDIYQEL

RSAVRRSTAESLFGQRLAVHSDFLGEQLQPLLDLTRRPPQVMRLQQRVNSPGWRRAMAAR

KRIDDLIDAQIADARTAPRPDDHMLTTLISGCSEEGTTLSDNEIRDSIVSLITAGYETTS

GALAWAIYALLTVPGTWESAASEVARVLGGRVPAADDLSALTYLNGVVHETLRLYSPGVI

SARRVLRDLWFDGHRIRAGRLLIFSAYVTHRLPEIWPEPTEFRPLRWDPNAADYRKPAPH

EFIPFSGGLHRCIGAVMATTEMTVILARLVARAMLQLPAQRTHRIRAANFAALRPWPGLT

VEIRKSAPAQ

>CYP139A1(643734506)*Mycobacterium bovis*BCG str. Tokyo 172

MRYPLGEALLALYRWRGPLINAGVGGHGYTYLLGAEANRFVFANADAFSWSQTFESLVPV

DGPTALIVSDGADHRRRRSVVAPGLRHHHVQRYVATMVSNIDTVIDGWQPGQRLDIYQEL

RSAVRRSTAESLFGQRLAVHSDFLGEQLQPLLDLTRRPPQVMRLQQRVNSPGWRRAMAAR

KRIDDLIDAQIADARTAPRPDDHMLTTLISGCSEEGTTLSDNEIRDSIVSLITAGYETTS

GALAWAIYALLTVPGTWESAASEVARVLGGRVPAADDLSALTYLNGVVHETLRLYSPGVI

SARRVLRDLWFDGHRIRAGRLLIFSAYVTHRLPEIWPEPTEFRPLRWDPNAADYRKPAPH

EFIPFSGGLHRCIGAVMATTEMTVILARLVARAMLQLPAQRTHRIRAANFAALRPWPGLT

VEIRKSAPAQ

>CYP139A1(648335985)Mycobacterium tuberculosis KZN R506

MRYPLGEALLALYRWRGPLINAGVGGHGYTYLLGAEANRFVFANADAFSWSQTFESLVPV

DGPTALIVSDGADHRRRRSVVAPGLRHHHVQRYVATMVSNIDTVIDGWQPGQRLDIYQEL

RSAVRRSTAESLFGQRLAVHSDFLGEQLQPLLDLTRRPPQVMRLQQRVNSPGWRRAMAAR

KRIDDLIDAQIADARTAPRPDDHMLTTLISGCSEEGTTLSDNEIRDSIVSLITAGYETTS

GALAWAIYALLTVPGTWESAASEVARVLGGRVPAADDLSALTYLNGVVHETLRLYSPGVI

SARRVLRDLWFDGHRIRAGRLLIFSAYVTHRLPEIWPEPTEFRPLRWDPNAADYRKPAPH

EFIPFSGGLHRCIGAVMATTEMTVILARLVARAMLQLPAQRTHRIRAANFAALRPWPGLT

VEIRKSAPAQ

>CYP139A1(2574754194)Mycobacterium tuberculosis MAL020131

MRYPLGEALLALYRWRGPLINAGVGGHGYTYLLGAEANRFVFANADAFSWSQTFESLVPV

DGPTALIVSDGADHRRRRSVVAPGLRHHHVQRYVATMVSNIDTVIDGWQPGQRLDIYQEL

RSAVRRSTAESLFGQRLAVHSDFLGEQLQPLLDLTRRPPQVMRLQQRVNSPGWRRAMAAR

KRIDDLIDAQIADARTAPRPDDHMLTTLISGCSEEGTTLSDNEIRDSIVSLITAGYETTS

GALAWAIYALLTVPGTWESAASEVARVLGGRVPAADDLSALTYLNGVVHETLRLYSPGVI

SARRVLRDLWFDGHRIRAGRLLIFSAYVTHRLPEIWPEPTEFRPLRWDPNAADYRKPAPH

EFIPFSGGLHRCIGAVMATTEMTVILARLVARAMLQLPAQRTHRIRAANFAALRPWPGLT

VEIRKSAPAQ

>CYP139A1(2575447433)Mycobacterium tuberculosis TKK-01-0077

MRYPLGEALLALYRWRGPLINAGVGGHGYTYLLGAEANRFVFANADAFSWSQTFESLVPV

DGPTALIVSDGADHRRRRSVVAPGLRHHHVQRYVATMVSNIDTVIDGWQPGQRLDIYQEL

RSAVRRSTAESLFGQRLAVHSDFLGEQLQPLLDLTRRPPQVMRLQQRVNSPGWRRAMAAR

KRIDDLIDAQIADARTAPRPDDHMLTTLISGCSEEGTTLSDNEIRDSIVSLITAGYETTS

GALAWAIYALLTVPGTWESAASEVARVLGGRVPAADDLSALTYLNGVVHETLRLYSPGVI

SARRVLRDLWFDGHRIRAGRLLIFSAYVTHRLPEIWPEPTEFRPLRWDPNAADYRKPAPH

EFIPFSGGLHRCIGAVMATTEMTVILARLVARAMLQLPAQRTHRIRAANFAALRPWPGLT

VEIRKSAPAQ

>CYP139A1(2575938969)*Mycobacterium africanum* MAL010111

MRYPLGEALLALYRWRGPLINAGVGGHGYTYLLGAEANRFVFANADAFSWSQTFESLVPV

DGPTALIVSDGADHRRRRSVVAPGLRHHHVQRYVATMVSNIDTVIDGWQPGQRLDIYQEL

RSAVRRSTAESLFGQRLAVHSDFLGEQLQPLLDLTRRPPQVMRLQQRVNSPGWRRAMAAR

KRIDDLIDAQIADARTAPRPDDHMLTTLISGCSEEGTTLSDNEIRDSIVSLITAGYETTS

GALAWAIYALLTVPGTWESAASEVARVLGGRVPAADDLSALTYLNGVVHETLRLYSPGVI

SARRVLRDLWFDGHRIRAGRLLIFSAYVTHRLPEIWPEPTEFRPLRWDPNAADYRKPAPH

EFIPFSGGLHRCIGAVMATTEMTVILARLVARAMLQLPAQRTHRIRAANFAALRPWPGLT

VEIRKSAPAQ

>CYP139A1(2576477081)Mycobacterium tuberculosis OFXR-15

MRYPLGEALLALYRWRGPLINAGVGGHGYTYLLGAEANRFVFANADAFSWSQTFESLVPV

DGPTALIVSDGADHRRRRSVVAPGLRHHHVQRYVATMVSNIDTVIDGWQPGQRLDIYQEL

RSAVRRSTAESLFGQRLAVHSDFLGEQLQPLLDLTRRPPQVMRLQQRVNSPGWRRAMAAR

KRIDDLIDAQIADARTAPRPDDHMLTTLISGCSEEGTTLSDNEIRDSIVSLITAGYETTS

GALAWAIYALLTVPGTWESAASEVARVLGGRVPAADDLSALTYLNGVVHETLRLYSPGVI

SARRVLRDLWFDGHRIRAGRLLIFSAYVTHRLPEIWPEPTEFRPLRWDPNAADYRKPAPH

EFIPFSGGLHRCIGAVMATTEMTVILARLVARAMLQLPAQRTHRIRAANFAALRPWPGLT

VEIRKSAPAQ

>CYP139A1(2576601719)Mycobacterium tuberculosis MAL020179

MRYPLGEALLALYRWRGPLINAGVGGHGYTYLLGAEANRFVFANADAFSWSQTFESLVPV

DGPTALIVSDGADHRRRRSVVAPGLRHHHVQRYVATMVSNIDTVIDGWQPGQRLDIYQEL

RSAVRRSTAESLFGQRLAVHSDFLGEQLQPLLDLTRRPPQVMRLQQRVNSPGWRRAMAAR

KRIDDLIDAQIADARTAPRPDDHMLTTLISGCSEEGTTLSDNEIRDSIVSLITAGYETTS

GALAWAIYALLTVPGTWESAASEVARVLGGRVPAADDLSALTYLNGVVHETLRLYSPGVI

SARRVLRDLWFDGHRIRAGRLLIFSAYVTHRLPEIWPEPTEFRPLRWDPNAADYRKPAPH

EFIPFSGGLHRCIGAVMATTEMTVILARLVARAMLQLPAQRTHRIRAANFAALRPWPGLT

VEIRKSAPAQ

>CYP139A1(2577098384)Mycobacterium tuberculosis BTB12-449

MRYPLGEALLALYRWRGPLINAGVGGHGYTYLLGAEANRFVFANADAFSWSQTFESLVPV

DGPTALIVSDGADHRRRRSVVAPGLRHHHVQRYVATMVSNIDTVIDGWQPGQRLDIYQEL

RSAVRRSTAESLFGQRLAVHSDFLGEQLQPLLDLTRRPPQVMRLQQRVNSPGWRRAMAAR

KRIDDLIDAQIADARTAPRPDDHMLTTLISGCSEEGTTLSDNEIRDSIVSLITAGYETTS

GALAWAIYALLTVPGTWESAASEVARVLGGRVPAADDLSALTYLNGVVHETLRLYSPGVI

SARRVLRDLWFDGHRIRAGRLLIFSAYVTHRLPEIWPEPTEFRPLRWDPNAADYRKPAPH

EFIPFSGGLHRCIGAVMATTEMTVILARLVARAMLQLPAQRTHRIRAANFAALRPWPGLT

VEIRKSAPAQ

>CYP139A1(2578107196)Mycobacterium tuberculosis BTB06-001

MRYPLGEALLALYRWRGPLINAGVGGHGYTYLLGAEANRFVFANADAFSWSQTFESLVPV

DGPTALIVSDGADHRRRRSVVAPGLRHHHVQRYVATMVSNIDTVIDGWQPGQRLDIYQEL

RSAVRRSTAESLFGQRLAVHSDFLGEQLQPLLDLTRRPPQVMRLQQRVNSPGWRRAMAAR

KRIDDLIDAQIADARTAPRPDDHMLTTLISGCSEEGTTLSDNEIRDSIVSLITAGYETTS

GALAWAIYALLTVPGTWESAASEVARVLGGRVPAADDLSALTYLNGVVHETLRLYSPGVI

SARRVLRDLWFDGHRIRAGRLLIFSAYVTHRLPEIWPEPTEFRPLRWDPNAADYRKPAPH

EFIPFSGGLHRCIGAVMATTEMTVILARLVARAMLQLPAQRTHRIRAANFAALRPWPGLT

VEIRKSAPAQ

>CYP139A1(2584883084)Mycobacterium tuberculosis XTB13-198

MRYPLGEALLALYRWRGPLINAGVGGHGYTYLLGAEANRFVFANADAFSWSQTFESLVPV

DGPTALIVSDGADHRRRRSVVAPGLRHHHVQRYVATMVSNIDTVIDGWQPGQRLDIYQEL

RSAVRRSTAESLFGQRLAVHSDFLGEQLQPLLDLTRRPPQVMRLQQRVNSPGWRRAMAAR

KRIDDLIDAQIADARTAPRPDDHMLTTLISGCSEEGTTLSDNEIRDSIVSLITAGYETTS

GALAWAIYALLTVPGTWESAASEVARVLGGRVPAADDLSALTYLNGVVHETLRLYSPGVI

SARRVLRDLWFDGHRIRAGRLLIFSAYVTHRLPEIWPEPTEFRPLRWDPNAADYRKPAPH

EFIPFSGGLHRCIGAVMATTEMTVILARLVARAMLQLPAQRTHRIRAANFAALRPWPGLT

VEIRKSAPAQ

>CYP139A1(2588974834)Mycobacterium tuberculosis TKK-01-0008

MRYPLGEALLALYRWRGPLINAGVGGHGYTYLLGAEANRFVFANADAFSWSQTFESLVPV

DGPTALIVSDGADHRRRRSVVAPGLRHHHVQRYVATMVSNIDTVIDGWQPGQRLDIYQEL

RSAVRRSTAESLFGQRLAVHSDFLGEQLQPLLDLTRRPPQVMRLQQRVNSPGWRRAMAAR

KRIDDLIDAQIADARTAPRPDDHMLTTLISGCSEEGTTLSDNEIRDSIVSLITAGYETTS

GALAWAIYALLTVPGTWESAASEVARVLGGRVPAADDLSALTYLNGVVHETLRLYSPGVI

SARRVLRDLWFDGHRIRAGRLLIFSAYVTHRLPEIWPEPTEFRPLRWDPNAADYRKPAPH

EFIPFSGGLHRCIGAVMATTEMTVILARLVARAMLQLPAQRTHRIRAANFAALRPWPGLT

VEIRKSAPAQ

>CYP139A1(2589056454)Mycobacterium tuberculosis TBR29

MRYPLGEALLALYRWRGPLINAGVGGHGYTYLLGAEANRFVFANADAFSWSQTFESLVPV

DGPTALIVSDGADHRRRRSVVAPGLRHHHVQRYVATMVSNIDTVIDGWQPGQRLDIYQEL

RSAVRRSTAESLFGQRLAVHSDFLGEQLQPLLDLTRRPPQVMRLQQRVNSPGWRRAMAAR

KRIDDLIDAQIADARTAPRPDDHMLTTLISGCSEEGTTLSDNEIRDSIVSLITAGYETTS

GALAWAIYALLTVPGTWESAASEVARVLGGRVPAADDLSALTYLNGVVHETLRLYSPGVI

SARRVLRDLWFDGHRIRAGRLLIFSAYVTHRLPEIWPEPTEFRPLRWDPNAADYRKPAPH

EFIPFSGGLHRCIGAVMATTEMTVILARLVARAMLQLPAQRTHRIRAANFAALRPWPGLT

VEIRKSAPAQ

>CYP139A1(2589161189)Mycobacterium tuberculosis OFXR-4

MRYPLGEALLALYRWRGPLINAGVGGHGYTYLLGAEANRFVFANADAFSWSQTFESLVPV

DGPTALIVSDGADHRRRRSVVAPGLRHHHVQRYVATMVSNIDTVIDGWQPGQRLDIYQEL

RSAVRRSTAESLFGQRLAVHSDFLGEQLQPLLDLTRRPPQVMRLQQRVNSPGWRRAMAAR

KRIDDLIDAQIADARTAPRPDDHMLTTLISGCSEEGTTLSDNEIRDSIVSLITAGYETTS

GALAWAIYALLTVPGTWESAASEVARVLGGRVPAADDLSALTYLNGVVHETLRLYSPGVI

SARRVLRDLWFDGHRIRAGRLLIFSAYVTHRLPEIWPEPTEFRPLRWDPNAADYRKPAPH

EFIPFSGGLHRCIGAVMATTEMTVILARLVARAMLQLPAQRTHRIRAANFAALRPWPGLT

VEIRKSAPAQ

>CYP139A1(2590374347)Mycobacterium tuberculosis OFXR-21

MRYPLGEALLALYRWRGPLINAGVGGHGYTYLLGAEANRFVFANADAFSWSQTFESLVPV

DGPTALIVSDGADHRRRRSVVAPGLRHHHVQRYVATMVSNIDTVIDGWQPGQRLDIYQEL

RSAVRRSTAESLFGQRLAVHSDFLGEQLQPLLDLTRRPPQVMRLQQRVNSPGWRRAMAAR

KRIDDLIDAQIADARTAPRPDDHMLTTLISGCSEEGTTLSDNEIRDSIVSLITAGYETTS

GALAWAIYALLTVPGTWESAASEVARVLGGRVPAADDLSALTYLNGVVHETLRLYSPGVI

SARRVLRDLWFDGHRIRAGRLLIFSAYVTHRLPEIWPEPTEFRPLRWDPNAADYRKPAPH

EFIPFSGGLHRCIGAVMATTEMTVILARLVARAMLQLPAQRTHRIRAANFAALRPWPGLT

VEIRKSAPAQ

>CYP139A1(646018681)Mycobacterium tuberculosis K85

MRYPLGEALLALYRWRGPLINAGVGGHGYTYLLGAEANRFVFANADAFSWSQTFESLVPV

DGPTALIVSDGADHRRRRSVVAPGLRHHHVQRYVATMVSNIDTVIDGWQPGQRLDIYQEL

RSAVRRSTAESLFGQRLAVHSDFLGEQLQPLLDLTRRPPQVMRLQQRVNSPGWRRAMAAR

KRIDDLIDAQIADARTAPRPDDHMLTTLISGCSEEGTTLSDNEIRDSIVSLITAGYETTS

GALAWAIYALLTVPGTWESAASEVARVLGGRVPAADDLSALTYLNGVVHETLRLYSPGVI

SARRVLRDLWFDGHRIRAGRLLIFSAYVTHRLPEIWPEPTEFRPLRWDPNAADYRKPAPH

EFIPFSGGLHRCIGAVMATTEMTVILARLVARAMLQLPAQRTHRIRAANFAALRPWPGLT

VEIRKSAPAQ

>CYP139A1(2575060404)Mycobacterium tuberculosis XTB13-209

MRYPLGEALLALYRWRGPLINAGVGGHGYTYLLGAEANRFVFANADAFSWSQTFESLVPV

DGPTALIVSDGADHRRRRSVVAPGLRHHHVQRYVATMVSNIDTVIDGWQPGQRLDIYQEL

RSAVRRSTAESLFGQRLAVHSDFLGEQLQPLLDLTRRPPQVMRLQQRVNSPGWRRAMAAR

KRIDDLIDAQIADARTAPRPDDHMLTTLISGCSEEGTTLSDNEIRDSIVSLITAGYETTS

GALAWAIYALLTVPGTWESAASEVARVLGGRVPAADDLSALTYLNGVVHETLRLYSPGVI

SARRVLRDLWFDGHRIRAGRLLIFSAYVTHRLPEIWPEPTEFRPLRWDPNAADYRKPAPH

EFIPFSGGLHRCIGAVMATTEMTVILARLVARAMLQLPAQRTHRIRAANFAALRPWPGLT

VEIRKSAPAQ

>CYP139A1(2576105631)Mycobacterium tuberculosis TRUG0037

MRYPLGEALLALYRWRGPLINAGVGGHGYTYLLGAEANRFVFANADAFSWSQTFESLVPV

DGPTALIVSDGADHRRRRSVVAPGLRHHHVQRYVATMVSNIDTVIDGWQPGQRLDIYQEL

RSAVRRSTAESLFGQRLAVHSDFLGEQLQPLLDLTRRPPQVMRLQQRVNSPGWRRAMAAR

KRIDDLIDAQIADARTAPRPDDHMLTTLISGCSEEGTTLSDNEIRDSIVSLITAGYETTS

GALAWAIYALLTVPGTWESAASEVARVLGGRVPAADDLSALTYLNGVVHETLRLYSPGVI

SARRVLRDLWFDGHRIRAGRLLIFSAYVTHRLPEIWPEPTEFRPLRWDPNAADYRKPAPH

EFIPFSGGLHRCIGAVMATTEMTVILARLVARAMLQLPAQRTHRIRAANFAALRPWPGLT

VEIRKSAPAQ

>CYP139A1(2576247251)Mycobacterium tuberculosis TKK_04_0103

MRYPLGEALLALYRWRGPLINAGVGGHGYTYLLGAEANRFVFANADAFSWSQTFESLVPV

DGPTALIVSDGADHRRRRSVVAPGLRHHHVQRYVATMVSNIDTVIDGWQPGQRLDIYQEL

RSAVRRSTAESLFGQRLAVHSDFLGEQLQPLLDLTRRPPQVMRLQQRVNSPGWRRAMAAR

KRIDDLIDAQIADARTAPRPDDHMLTTLISGCSEEGTTLSDNEIRDSIVSLITAGYETTS

GALAWAIYALLTVPGTWESAASEVARVLGGRVPAADDLSALTYLNGVVHETLRLYSPGVI

SARRVLRDLWFDGHRIRAGRLLIFSAYVTHRLPEIWPEPTEFRPLRWDPNAADYRKPAPH

EFIPFSGGLHRCIGAVMATTEMTVILARLVARAMLQLPAQRTHRIRAANFAALRPWPGLT

VEIRKSAPAQ

>CYP139A1(2576981010)Mycobacterium tuberculosis TB_RSA149

MRYPLGEALLALYRWRGPLINAGVGGHGYTYLLGAEANRFVFANADAFSWSQTFESLVPV

DGPTALIVSDGADHRRRRSVVAPGLRHHHVQRYVATMVSNIDTVIDGWQPGQRLDIYQEL

RSAVRRSTAESLFGQRLAVHSDFLGEQLQPLLDLTRRPPQVMRLQQRVNSPGWRRAMAAR

KRIDDLIDAQIADARTAPRPDDHMLTTLISGCSEEGTTLSDNEIRDSIVSLITAGYETTS

GALAWAIYALLTVPGTWESAASEVARVLGGRVPAADDLSALTYLNGVVHETLRLYSPGVI

SARRVLRDLWFDGHRIRAGRLLIFSAYVTHRLPEIWPEPTEFRPLRWDPNAADYRKPAPH

EFIPFSGGLHRCIGAVMATTEMTVILARLVARAMLQLPAQRTHRIRAANFAALRPWPGLT

VEIRKSAPAQ

>CYP139A1(2577093117)Mycobacterium tuberculosis CPHL_A

MRYPLGEALLALYRWRGPLINAGVGGHGYTYLLGAEANRFVFANADAFSWSQTFESLVPV

DGPTALIVSDGADHRRRRSVVAPGLRHHHVQRYVATMVSNIDTVIDGWQPGQRLDIYQEL

RSAVRRSTAESLFGQRLAVHSDFLGEQLQPLLDLTRRPPQVMRLQQRVNSPGWRRAMAAR

KRIDDLIDAQIADARTAPRPDDHMLTTLISGCSEEGTTLSDNEIRDSIVSLITAGYETTS

GALAWAIYALLTVPGTWESAASEVARVLGGRVPAADDLSALTYLNGVVHETLRLYSPGVI

SARRVLRDLWFDGHRIRAGRLLIFSAYVTHRLPEIWPEPTEFRPLRWDPNAADYRKPAPH

EFIPFSGGLHRCIGAVMATTEMTVILARLVARAMLQLPAQRTHRIRAANFAALRPWPGLT

VEIRKSAPAQ

>CYP139A1(2577198903)Mycobacterium tuberculosis MD15974

MRYPLGEALLALYRWRGPLINAGVGGHGYTYLLGAEANRFVFANADAFSWSQTFESLVPV

DGPTALIVSDGADHRRRRSVVAPGLRHHHVQRYVATMVSNIDTVIDGWQPGQRLDIYQEL

RSAVRRSTAESLFGQRLAVHSDFLGEQLQPLLDLTRRPPQVMRLQQRVNSPGWRRAMAAR

KRIDDLIDAQIADARTAPRPDDHMLTTLISGCSEEGTTLSDNEIRDSIVSLITAGYETTS

GALAWAIYALLTVPGTWESAASEVARVLGGRVPAADDLSALTYLNGVVHETLRLYSPGVI

SARRVLRDLWFDGHRIRAGRLLIFSAYVTHRLPEIWPEPTEFRPLRWDPNAADYRKPAPH

EFIPFSGGLHRCIGAVMATTEMTVILARLVARAMLQLPAQRTHRIRAANFAALRPWPGLT

VEIRKSAPAQ

>CYP139A1(2577516047)Mycobacterium tuberculosis TKK_04_0132

MRYPLGEALLALYRWRGPLINAGVGGHGYTYLLGAEANRFVFANADAFSWSQTFESLVPV

DGPTALIVSDGADHRRRRSVVAPGLRHHHVQRYVATMVSNIDTVIDGWQPGQRLDIYQEL

RSAVRRSTAESLFGQRLAVHSDFLGEQLQPLLDLTRRPPQVMRLQQRVNSPGWRRAMAAR

KRIDDLIDAQIADARTAPRPDDHMLTTLISGCSEEGTTLSDNEIRDSIVSLITAGYETTS

GALAWAIYALLTVPGTWESAASEVARVLGGRVPAADDLSALTYLNGVVHETLRLYSPGVI

SARRVLRDLWFDGHRIRAGRLLIFSAYVTHRLPEIWPEPTEFRPLRWDPNAADYRKPAPH

EFIPFSGGLHRCIGAVMATTEMTVILARLVARAMLQLPAQRTHRIRAANFAALRPWPGLT

VEIRKSAPAQ

>CYP139A1(2578213104)Mycobacterium tuberculosis XTB13-255

MRYPLGEALLALYRWRGPLINAGVGGHGYTYLLGAEANRFVFANADAFSWSQTFESLVPV

DGPTALIVSDGADHRRRRSVVAPGLRHHHVQRYVATMVSNIDTVIDGWQPGQRLDIYQEL

RSAVRRSTAESLFGQRLAVHSDFLGEQLQPLLDLTRRPPQVMRLQQRVNSPGWRRAMAAR

KRIDDLIDAQIADARTAPRPDDHMLTTLISGCSEEGTTLSDNEIRDSIVSLITAGYETTS

GALAWAIYALLTVPGTWESAASEVARVLGGRVPAADDLSALTYLNGVVHETLRLYSPGVI

SARRVLRDLWFDGHRIRAGRLLIFSAYVTHRLPEIWPEPTEFRPLRWDPNAADYRKPAPH

EFIPFSGGLHRCIGAVMATTEMTVILARLVARAMLQLPAQRTHRIRAANFAALRPWPGLT

VEIRKSAPAQ

>CYP139A1(2584711251)Mycobacterium tuberculosis TB_RSA01

MRYPLGEALLALYRWRGPLINAGVGGHGYTYLLGAEANRFVFANADAFSWSQTFESLVPV

DGPTALIVSDGADHRRRRSVVAPGLRHHHVQRYVATMVSNIDTVIDGWQPGQRLDIYQEL

RSAVRRSTAESLFGQRLAVHSDFLGEQLQPLLDLTRRPPQVMRLQQRVNSPGWRRAMAAR

KRIDDLIDAQIADARTAPRPDDHMLTTLISGCSEEGTTLSDNEIRDSIVSLITAGYETTS

GALAWAIYALLTVPGTWESAASEVARVLGGRVPAADDLSALTYLNGVVHETLRLYSPGVI

SARRVLRDLWFDGHRIRAGRLLIFSAYVTHRLPEIWPEPTEFRPLRWDPNAADYRKPAPH

EFIPFSGGLHRCIGAVMATTEMTVILARLVARAMLQLPAQRTHRIRAANFAALRPWPGLT

VEIRKSAPAQ

>CYP139A1(2584816678)Mycobacterium tuberculosis XTB13-113

MRYPLGEALLALYRWRGPLINAGVGGHGYTYLLGAEANRFVFANADAFSWSQTFESLVPV

DGPTALIVSDGADHRRRRSVVAPGLRHHHVQRYVATMVSNIDTVIDGWQPGQRLDIYQEL

RSAVRRSTAESLFGQRLAVHSDFLGEQLQPLLDLTRRPPQVMRLQQRVNSPGWRRAMAAR

KRIDDLIDAQIADARTAPRPDDHMLTTLISGCSEEGTTLSDNEIRDSIVSLITAGYETTS

GALAWAIYALLTVPGTWESAASEVARVLGGRVPAADDLSALTYLNGVVHETLRLYSPGVI

SARRVLRDLWFDGHRIRAGRLLIFSAYVTHRLPEIWPEPTEFRPLRWDPNAADYRKPAPH

EFIPFSGGLHRCIGAVMATTEMTVILARLVARAMLQLPAQRTHRIRAANFAALRPWPGLT

VEIRKSAPAQ

>CYP139A1(2589068752)Mycobacterium tuberculosis TBR35

MRYPLGEALLALYRWRGPLINAGVGGHGYTYLLGAEANRFVFANADAFSWSQTFESLVPV

DGPTALIVSDGADHRRRRSVVAPGLRHHHVQRYVATMVSNIDTVIDGWQPGQRLDIYQEL

RSAVRRSTAESLFGQRLAVHSDFLGEQLQPLLDLTRRPPQVMRLQQRVNSPGWRRAMAAR

KRIDDLIDAQIADARTAPRPDDHMLTTLISGCSEEGTTLSDNEIRDSIVSLITAGYETTS

GALAWAIYALLTVPGTWESAASEVARVLGGRVPAADDLSALTYLNGVVHETLRLYSPGVI

SARRVLRDLWFDGHRIRAGRLLIFSAYVTHRLPEIWPEPTEFRPLRWDPNAADYRKPAPH

EFIPFSGGLHRCIGAVMATTEMTVILARLVARAMLQLPAQRTHRIRAANFAALRPWPGLT

VEIRKSAPAQ

>CYP139A1(2589604293)Mycobacterium tuberculosis TKK-01-0050

MRYPLGEALLALYRWRGPLINAGVGGHGYTYLLGAEANRFVFANADAFSWSQTFESLVPV

DGPTALIVSDGADHRRRRSVVAPGLRHHHVQRYVATMVSNIDTVIDGWQPGQRLDIYQEL

RSAVRRSTAESLFGQRLAVHSDFLGEQLQPLLDLTRRPPQVMRLQQRVNSPGWRRAMAAR

KRIDDLIDAQIADARTAPRPDDHMLTTLISGCSEEGTTLSDNEIRDSIVSLITAGYETTS

GALAWAIYALLTVPGTWESAASEVARVLGGRVPAADDLSALTYLNGVVHETLRLYSPGVI

SARRVLRDLWFDGHRIRAGRLLIFSAYVTHRLPEIWPEPTEFRPLRWDPNAADYRKPAPH

EFIPFSGGLHRCIGAVMATTEMTVILARLVARAMLQLPAQRTHRIRAANFAALRPWPGLT

VEIRKSAPAQ

>CYP139A1(643028176)Mycobacterium tuberculosis T92

MRYPLGEALLALYRWRGPLINAGVGGHGYTYLLGAEANRFVFANADAFSWSQTFESLVPV

DGPTALIVSDGADHRRRRSVVAPGLRHHHVQRYVATMVSNIDTVIDGWQPGQRLDIYQEL

RSAVRRSTAESLFGQRLAVHSDFLGEQLQPLLDLTRRPPQVMRLQQRVNSPGWRRAMAAR

KRIDDLIDAQIADARTAPRPDDHMLTTLISGCSEEGTTLSDNEIRDSIVSLITAGYETTS

GALAWAIYALLTVPGTWESAASEVARVLGGRVPAADDLSALTYLNGVVHETLRLYSPGVI

SARRVLRDLWFDGHRIRAGRLLIFSAYVTHRLPEIWPEPTEFRPLRWDPNAADYRKPAPH

EFIPFSGGLHRCIGAVMATTEMTVILARLVARAMLQLPAQRTHRIRAANFAALRPWPGLT

VEIRKSAPAQ

>CYP139A1(648476944)Mycobacterium tuberculosis SUMu009

MRYPLGEALLALYRWRGPLINAGVGGHGYTYLLGAEANRFVFANADAFSWSQTFESLVPV

DGPTALIVSDGADHRRRRSVVAPGLRHHHVQRYVATMVSNIDTVIDGWQPGQRLDIYQEL

RSAVRRSTAESLFGQRLAVHSDFLGEQLQPLLDLTRRPPQVMRLQQRVNSPGWRRAMAAR

KRIDDLIDAQIADARTAPRPDDHMLTTLISGCSEEGTTLSDNEIRDSIVSLITAGYETTS

GALAWAIYALLTVPGTWESAASEVARVLGGRVPAADDLSALTYLNGVVHETLRLYSPGVI

SARRVLRDLWFDGHRIRAGRLLIFSAYVTHRLPEIWPEPTEFRPLRWDPNAADYRKPAPH

EFIPFSGGLHRCIGAVMATTEMTVILARLVARAMLQLPAQRTHRIRAANFAALRPWPGLT

VEIRKSAPAQ

>CYP139A1(2511736071)Mycobacterium tuberculosis CTRI-2

MRYPLGEALLALYRWRGPLINAGVGGHGYTYLLGAEANRFVFANADAFSWSQTFESLVPV

DGPTALIVSDGADHRRRRSVVAPGLRHHHVQRYVATMVSNIDTVIDGWQPGQRLDIYQEL

RSAVRRSTAESLFGQRLAVHSDFLGEQLQPLLDLTRRPPQVMRLQQRVNSPGWRRAMAAR

KRIDDLIDAQIADARTAPRPDDHMLTTLISGCSEEGTTLSDNEIRDSIVSLITAGYETTS

GALAWAIYALLTVPGTWESAASEVARVLGGRVPAADDLSALTYLNGVVHETLRLYSPGVI

SARRVLRDLWFDGHRIRAGRLLIFSAYVTHRLPEIWPEPTEFRPLRWDPNAADYRKPAPH

EFIPFSGGLHRCIGAVMATTEMTVILARLVARAMLQLPAQRTHRIRAANFAALRPWPGLT

VEIRKSAPAQ

>CYP139A1(2546206123)Mycobacterium tuberculosis EAI5/NITR206

MRYPLGEALLALYRWRGPLINAGVGGHGYTYLLGAEANRFVFANADAFSWSQTFESLVPV

DGPTALIVSDGADHRRRRSVVAPGLRHHHVQRYVATMVSNIDTVIDGWQPGQRLDIYQEL

RSAVRRSTAESLFGQRLAVHSDFLGEQLQPLLDLTRRPPQVMRLQQRVNSPGWRRAMAAR

KRIDDLIDAQIADARTAPRPDDHMLTTLISGCSEEGTTLSDNEIRDSIVSLITAGYETTS

GALAWAIYALLTVPGTWESAASEVARVLGGRVPAADDLSALTYLNGVVHETLRLYSPGVI

SARRVLRDLWFDGHRIRAGRLLIFSAYVTHRLPEIWPEPTEFRPLRWDPNAADYRKPAPH

EFIPFSGGLHRCIGAVMATTEMTVILARLVARAMLQLPAQRTHRIRAANFAALRPWPGLT

VEIRKSAPAQ

>CYP139A1(2574780327)Mycobacterium tuberculosis KT-0086

MRYPLGEALLALYRWRGPLINAGVGGHGYTYLLGAEANRFVFANADAFSWSQTFESLVPV

DGPTALIVSDGADHRRRRSVVAPGLRHHHVQRYVATMVSNIDTVIDGWQPGQRLDIYQEL

RSAVRRSTAESLFGQRLAVHSDFLGEQLQPLLDLTRRPPQVMRLQQRVNSPGWRRAMAAR

KRIDDLIDAQIADARTAPRPDDHMLTTLISGCSEEGTTLSDNEIRDSIVSLITAGYETTS

GALAWAIYALLTVPGTWESAASEVARVLGGRVPAADDLSALTYLNGVVHETLRLYSPGVI

SARRVLRDLWFDGHRIRAGRLLIFSAYVTHRLPEIWPEPTEFRPLRWDPNAADYRKPAPH

EFIPFSGGLHRCIGAVMATTEMTVILARLVARAMLQLPAQRTHRIRAANFAALRPWPGLT

VEIRKSAPAQ

>CYP139A1(2575978404)Mycobacterium tuberculosis TBR7

MRYPLGEALLALYRWRGPLINAGVGGHGYTYLLGAEANRFVFANADAFSWSQTFESLVPV

DGPTALIVSDGADHRRRRSVVAPGLRHHHVQRYVATMVSNIDTVIDGWQPGQRLDIYQEL

RSAVRRSTAESLFGQRLAVHSDFLGEQLQPLLDLTRRPPQVMRLQQRVNSPGWRRAMAAR

KRIDDLIDAQIADARTAPRPDDHMLTTLISGCSEEGTTLSDNEIRDSIVSLITAGYETTS

GALAWAIYALLTVPGTWESAASEVARVLGGRVPAADDLSALTYLNGVVHETLRLYSPGVI

SARRVLRDLWFDGHRIRAGRLLIFSAYVTHRLPEIWPEPTEFRPLRWDPNAADYRKPAPH

EFIPFSGGLHRCIGAVMATTEMTVILARLVARAMLQLPAQRTHRIRAANFAALRPWPGLT

VEIRKSAPAQ

>CYP139A1(2576675825)Mycobacterium tuberculosis TRUG0101

MRYPLGEALLALYRWRGPLINAGVGGHGYTYLLGAEANRFVFANADAFSWSQTFESLVPV

DGPTALIVSDGADHRRRRSVVAPGLRHHHVQRYVATMVSNIDTVIDGWQPGQRLDIYQEL

RSAVRRSTAESLFGQRLAVHSDFLGEQLQPLLDLTRRPPQVMRLQQRVNSPGWRRAMAAR

KRIDDLIDAQIADARTAPRPDDHMLTTLISGCSEEGTTLSDNEIRDSIVSLITAGYETTS

GALAWAIYALLTVPGTWESAASEVARVLGGRVPAADDLSALTYLNGVVHETLRLYSPGVI

SARRVLRDLWFDGHRIRAGRLLIFSAYVTHRLPEIWPEPTEFRPLRWDPNAADYRKPAPH

EFIPFSGGLHRCIGAVMATTEMTVILARLVARAMLQLPAQRTHRIRAANFAALRPWPGLT

VEIRKSAPAQ

>CYP139A1(2576947708)Mycobacterium tuberculosis BTB03-144

MRYPLGEALLALYRWRGPLINAGVGGHGYTYLLGAEANRFVFANADAFSWSQTFESLVPV

DGPTALIVSDGADHRRRRSVVAPGLRHHHVQRYVATMVSNIDTVIDGWQPGQRLDIYQEL

RSAVRRSTAESLFGQRLAVHSDFLGEQLQPLLDLTRRPPQVMRLQQRVNSPGWRRAMAAR

KRIDDLIDAQIADARTAPRPDDHMLTTLISGCSEEGTTLSDNEIRDSIVSLITAGYETTS

GALAWAIYALLTVPGTWESAASEVARVLGGRVPAADDLSALTYLNGVVHETLRLYSPGVI

SARRVLRDLWFDGHRIRAGRLLIFSAYVTHRLPEIWPEPTEFRPLRWDPNAADYRKPAPH

EFIPFSGGLHRCIGAVMATTEMTVILARLVARAMLQLPAQRTHRIRAANFAALRPWPGLT

VEIRKSAPAQ

>CYP139A1(2577400922)Mycobacterium tuberculosis TB_RSA120

MRYPLGEALLALYRWRGPLINAGVGGHGYTYLLGAEANRFVFANADAFSWSQTFESLVPV

DGPTALIVSDGADHRRRRSVVAPGLRHHHVQRYVATMVSNIDTVIDGWQPGQRLDIYQEL

RSAVRRSTAESLFGQRLAVHSDFLGEQLQPLLDLTRRPPQVMRLQQRVNSPGWRRAMAAR

KRIDDLIDAQIADARTAPRPDDHMLTTLISGCSEEGTTLSDNEIRDSIVSLITAGYETTS

GALAWAIYALLTVPGTWESAASEVARVLGGRVPAADDLSALTYLNGVVHETLRLYSPGVI

SARRVLRDLWFDGHRIRAGRLLIFSAYVTHRLPEIWPEPTEFRPLRWDPNAADYRKPAPH

EFIPFSGGLHRCIGAVMATTEMTVILARLVARAMLQLPAQRTHRIRAANFAALRPWPGLT

VEIRKSAPAQ

>CYP139A1(2577893113)Mycobacterium tuberculosis GM 1503

MRYPLGEALLALYRWRGPLINAGVGGHGYTYLLGAEANRFVFANADAFSWSQTFESLVPV

DGPTALIVSDGADHRRRRSVVAPGLRHHHVQRYVATMVSNIDTVIDGWQPGQRLDIYQEL

RSAVRRSTAESLFGQRLAVHSDFLGEQLQPLLDLTRRPPQVMRLQQRVNSPGWRRAMAAR

KRIDDLIDAQIADARTAPRPDDHMLTTLISGCSEEGTTLSDNEIRDSIVSLITAGYETTS

GALAWAIYALLTVPGTWESAASEVARVLGGRVPAADDLSALTYLNGVVHETLRLYSPGVI

SARRVLRDLWFDGHRIRAGRLLIFSAYVTHRLPEIWPEPTEFRPLRWDPNAADYRKPAPH

EFIPFSGGLHRCIGAVMATTEMTVILARLVARAMLQLPAQRTHRIRAANFAALRPWPGLT

VEIRKSAPAQ

>CYP139A1(2584759228)Mycobacterium tuberculosis TKK_04_0082

MRYPLGEALLALYRWRGPLINAGVGGHGYTYLLGAEANRFVFANADAFSWSQTFESLVPV

DGPTALIVSDGADHRRRRSVVAPGLRHHHVQRYVATMVSNIDTVIDGWQPGQRLDIYQEL

RSAVRRSTAESLFGQRLAVHSDFLGEQLQPLLDLTRRPPQVMRLQQRVNSPGWRRAMAAR

KRIDDLIDAQIADARTAPRPDDHMLTTLISGCSEEGTTLSDNEIRDSIVSLITAGYETTS

GALAWAIYALLTVPGTWESAASEVARVLGGRVPAADDLSALTYLNGVVHETLRLYSPGVI

SARRVLRDLWFDGHRIRAGRLLIFSAYVTHRLPEIWPEPTEFRPLRWDPNAADYRKPAPH

EFIPFSGGLHRCIGAVMATTEMTVILARLVARAMLQLPAQRTHRIRAANFAALRPWPGLT

VEIRKSAPAQ

>CYP139A1(2584801008)Mycobacterium tuberculosis TKK_02_0002

MRYPLGEALLALYRWRGPLINAGVGGHGYTYLLGAEANRFVFANADAFSWSQTFESLVPV

DGPTALIVSDGADHRRRRSVVAPGLRHHHVQRYVATMVSNIDTVIDGWQPGQRLDIYQEL

RSAVRRSTAESLFGQRLAVHSDFLGEQLQPLLDLTRRPPQVMRLQQRVNSPGWRRAMAAR

KRIDDLIDAQIADARTAPRPDDHMLTTLISGCSEEGTTLSDNEIRDSIVSLITAGYETTS

GALAWAIYALLTVPGTWESAASEVARVLGGRVPAADDLSALTYLNGVVHETLRLYSPGVI

SARRVLRDLWFDGHRIRAGRLLIFSAYVTHRLPEIWPEPTEFRPLRWDPNAADYRKPAPH

EFIPFSGGLHRCIGAVMATTEMTVILARLVARAMLQLPAQRTHRIRAANFAALRPWPGLT

VEIRKSAPAQ

>CYP139A1(2584946269)Mycobacterium tuberculosis MD15050

MRYPLGEALLALYRWRGPLINAGVGGHGYTYLLGAEANRFVFANADAFSWSQTFESLVPV

DGPTALIVSDGADHRRRRSVVAPGLRHHHVQRYVATMVSNIDTVIDGWQPGQRLDIYQEL

RSAVRRSTAESLFGQRLAVHSDFLGEQLQPLLDLTRRPPQVMRLQQRVNSPGWRRAMAAR

KRIDDLIDAQIADARTAPRPDDHMLTTLISGCSEEGTTLSDNEIRDSIVSLITAGYETTS

GALAWAIYALLTVPGTWESAASEVARVLGGRVPAADDLSALTYLNGVVHETLRLYSPGVI

SARRVLRDLWFDGHRIRAGRLLIFSAYVTHRLPEIWPEPTEFRPLRWDPNAADYRKPAPH

EFIPFSGGLHRCIGAVMATTEMTVILARLVARAMLQLPAQRTHRIRAANFAALRPWPGLT

VEIRKSAPAQ

>CYP139A1(2589125802)Mycobacterium tuberculosis TBR75

MRYPLGEALLALYRWRGPLINAGVGGHGYTYLLGAEANRFVFANADAFSWSQTFESLVPV

DGPTALIVSDGADHRRRRSVVAPGLRHHHVQRYVATMVSNIDTVIDGWQPGQRLDIYQEL

RSAVRRSTAESLFGQRLAVHSDFLGEQLQPLLDLTRRPPQVMRLQQRVNSPGWRRAMAAR

KRIDDLIDAQIADARTAPRPDDHMLTTLISGCSEEGTTLSDNEIRDSIVSLITAGYETTS

GALAWAIYALLTVPGTWESAASEVARVLGGRVPAADDLSALTYLNGVVHETLRLYSPGVI

SARRVLRDLWFDGHRIRAGRLLIFSAYVTHRLPEIWPEPTEFRPLRWDPNAADYRKPAPH

EFIPFSGGLHRCIGAVMATTEMTVILARLVARAMLQLPAQRTHRIRAANFAALRPWPGLT

VEIRKSAPAQ

>CYP139A1(2589654545)Mycobacterium tuberculosis TKK-01-0056

MRYPLGEALLALYRWRGPLINAGVGGHGYTYLLGAEANRFVFANADAFSWSQTFESLVPV

DGPTALIVSDGADHRRRRSVVAPGLRHHHVQRYVATMVSNIDTVIDGWQPGQRLDIYQEL

RSAVRRSTAESLFGQRLAVHSDFLGEQLQPLLDLTRRPPQVMRLQQRVNSPGWRRAMAAR

KRIDDLIDAQIADARTAPRPDDHMLTTLISGCSEEGTTLSDNEIRDSIVSLITAGYETTS

GALAWAIYALLTVPGTWESAASEVARVLGGRVPAADDLSALTYLNGVVHETLRLYSPGVI

SARRVLRDLWFDGHRIRAGRLLIFSAYVTHRLPEIWPEPTEFRPLRWDPNAADYRKPAPH

EFIPFSGGLHRCIGAVMATTEMTVILARLVARAMLQLPAQRTHRIRAANFAALRPWPGLT

VEIRKSAPAQ

>CYP139A1(2590190898)Mycobacterium tuberculosis MAL020208

MRYPLGEALLALYRWRGPLINAGVGGHGYTYLLGAEANRFVFANADAFSWSQTFESLVPV

DGPTALIVSDGADHRRRRSVVAPGLRHHHVQRYVATMVSNIDTVIDGWQPGQRLDIYQEL

RSAVRRSTAESLFGQRLAVHSDFLGEQLQPLLDLTRRPPQVMRLQQRVNSPGWRRAMAAR

KRIDDLIDAQIADARTAPRPDDHMLTTLISGCSEEGTTLSDNEIRDSIVSLITAGYETTS

GALAWAIYALLTVPGTWESAASEVARVLGGRVPAADDLSALTYLNGVVHETLRLYSPGVI

SARRVLRDLWFDGHRIRAGRLLIFSAYVTHRLPEIWPEPTEFRPLRWDPNAADYRKPAPH

EFIPFSGGLHRCIGAVMATTEMTVILARLVARAMLQLPAQRTHRIRAANFAALRPWPGLT

VEIRKSAPAQ

>CYP139A1(2592267285)Mycobacterium tuberculosis TKK_04_0005

MRYPLGEALLALYRWRGPLINAGVGGHGYTYLLGAEANRFVFANADAFSWSQTFESLVPV

DGPTALIVSDGADHRRRRSVVAPGLRHHHVQRYVATMVSNIDTVIDGWQPGQRLDIYQEL

RSAVRRSTAESLFGQRLAVHSDFLGEQLQPLLDLTRRPPQVMRLQQRVNSPGWRRAMAAR

KRIDDLIDAQIADARTAPRPDDHMLTTLISGCSEEGTTLSDNEIRDSIVSLITAGYETTS

GALAWAIYALLTVPGTWESAASEVARVLGGRVPAADDLSALTYLNGVVHETLRLYSPGVI

SARRVLRDLWFDGHRIRAGRLLIFSAYVTHRLPEIWPEPTEFRPLRWDPNAADYRKPAPH

EFIPFSGGLHRCIGAVMATTEMTVILARLVARAMLQLPAQRTHRIRAANFAALRPWPGLT

VEIRKSAPAQ

>CYP139A1(2592579018)Mycobacterium tuberculosis TKK_04_0029

MRYPLGEALLALYRWRGPLINAGVGGHGYTYLLGAEANRFVFANADAFSWSQTFESLVPV

DGPTALIVSDGADHRRRRSVVAPGLRHHHVQRYVATMVSNIDTVIDGWQPGQRLDIYQEL

RSAVRRSTAESLFGQRLAVHSDFLGEQLQPLLDLTRRPPQVMRLQQRVNSPGWRRAMAAR

KRIDDLIDAQIADARTAPRPDDHMLTTLISGCSEEGTTLSDNEIRDSIVSLITAGYETTS

GALAWAIYALLTVPGTWESAASEVARVLGGRVPAADDLSALTYLNGVVHETLRLYSPGVI

SARRVLRDLWFDGHRIRAGRLLIFSAYVTHRLPEIWPEPTEFRPLRWDPNAADYRKPAPH

EFIPFSGGLHRCIGAVMATTEMTVILARLVARAMLQLPAQRTHRIRAANFAALRPWPGLT

VEIRKSAPAQ

>CYP139A1(2574803240)Mycobacterium tuberculosis TKK_03_0026

MRYPLGEALLALYRWRGPLINAGVGGHGYTYLLGAEANRFVFANADAFSWSQTFESLVPV

DGPTALIVSDGADHRRRRSVVAPGLRHHHVQRYVATMVSNIDTVIDGWQPGQRLDIYQEL

RSAVRRSTAESLFGQRLAVHSDFLGEQLQPLLDLTRRPPQVMRLQQRVNSPGWRRAMAAR

KRIDDLIDAQIADARTAPRPDDHMLTTLISGCSEEGTTLSDNEIRDSIVSLITAGYETTS

GALAWAIYALLTVPGTWESAASEVARVLGGRVPAADDLSALTYLNGVVHETLRLYSPGVI

SARRVLRDLWFDGHRIRAGRLLIFSAYVTHRLPEIWPEPTEFRPLRWDPNAADYRKPAPH

EFIPFSGGLHRCIGAVMATTEMTVILARLVARAMLQLPAQRTHRIRAANFAALRPWPGLT

VEIRKSAPAQ

>CYP139A1(2575138339)Mycobacterium tuberculosis MD17902

MRYPLGEALLALYRWRGPLINAGVGGHGYTYLLGAEANRFVFANADAFSWSQTFESLVPV

DGPTALIVSDGADHRRRRSVVAPGLRHHHVQRYVATMVSNIDTVIDGWQPGQRLDIYQEL

RSAVRRSTAESLFGQRLAVHSDFLGEQLQPLLDLTRRPPQVMRLQQRVNSPGWRRAMAAR

KRIDDLIDAQIADARTAPRPDDHMLTTLISGCSEEGTTLSDNEIRDSIVSLITAGYETTS

GALAWAIYALLTVPGTWESAASEVARVLGGRVPAADDLSALTYLNGVVHETLRLYSPGVI

SARRVLRDLWFDGHRIRAGRLLIFSAYVTHRLPEIWPEPTEFRPLRWDPNAADYRKPAPH

EFIPFSGGLHRCIGAVMATTEMTVILARLVARAMLQLPAQRTHRIRAANFAALRPWPGLT

VEIRKSAPAQ

>CYP139A1(2575935659)Mycobacterium tuberculosis TKK_04_0044

MRYPLGEALLALYRWRGPLINAGVGGHGYTYLLGAEANRFVFANADAFSWSQTFESLVPV

DGPTALIVSDGADHRRRRSVVAPGLRHHHVQRYVATMVSNIDTVIDGWQPGQRLDIYQEL

RSAVRRSTAESLFGQRLAVHSDFLGEQLQPLLDLTRRPPQVMRLQQRVNSPGWRRAMAAR

KRIDDLIDAQIADARTAPRPDDHMLTTLISGCSEEGTTLSDNEIRDSIVSLITAGYETTS

GALAWAIYALLTVPGTWESAASEVARVLGGRVPAADDLSALTYLNGVVHETLRLYSPGVI

SARRVLRDLWFDGHRIRAGRLLIFSAYVTHRLPEIWPEPTEFRPLRWDPNAADYRKPAPH

EFIPFSGGLHRCIGAVMATTEMTVILARLVARAMLQLPAQRTHRIRAANFAALRPWPGLT

VEIRKSAPAQ

>CYP139A1(2576703024)Mycobacterium tuberculosis MAL020144

MRYPLGEALLALYRWRGPLINAGVGGHGYTYLLGAEANRFVFANADAFSWSQTFESLVPV

DGPTALIVSDGADHRRRRSVVAPGLRHHHVQRYVATMVSNIDTVIDGWQPGQRLDIYQEL

RSAVRRSTAESLFGQRLAVHSDFLGEQLQPLLDLTRRPPQVMRLQQRVNSPGWRRAMAAR

KRIDDLIDAQIADARTAPRPDDHMLTTLISGCSEEGTTLSDNEIRDSIVSLITAGYETTS

GALAWAIYALLTVPGTWESAASEVARVLGGRVPAADDLSALTYLNGVVHETLRLYSPGVI

SARRVLRDLWFDGHRIRAGRLLIFSAYVTHRLPEIWPEPTEFRPLRWDPNAADYRKPAPH

EFIPFSGGLHRCIGAVMATTEMTVILARLVARAMLQLPAQRTHRIRAANFAALRPWPGLT

VEIRKSAPAQ

>CYP139A1(2577143911)Mycobacterium tuberculosis 2094HD

MRYPLGEALLALYRWRGPLINAGVGGHGYTYLLGAEANRFVFANADAFSWSQTFESLVPV

DGPTALIVSDGADHRRRRSVVAPGLRHHHVQRYVATMVSNIDTVIDGWQPGQRLDIYQEL

RSAVRRSTAESLFGQRLAVHSDFLGEQLQPLLDLTRRPPQVMRLQQRVNSPGWRRAMAAR

KRIDDLIDAQIADARTAPRPDDHMLTTLISGCSEEGTTLSDNEIRDSIVSLITAGYETTS

GALAWAIYALLTVPGTWESAASEVARVLGGRVPAADDLSALTYLNGVVHETLRLYSPGVI

SARRVLRDLWFDGHRIRAGRLLIFSAYVTHRLPEIWPEPTEFRPLRWDPNAADYRKPAPH

EFIPFSGGLHRCIGAVMATTEMTVILARLVARAMLQLPAQRTHRIRAANFAALRPWPGLT

VEIRKSAPAQ

>CYP139A1(2577175183)*Mycobacterium africanum* MAL010102

MRYPLGEALLALYRWRGPLINAGVGGHGYTYLLGAEANRFVFANADAFSWSQTFESLVPV

DGPTALIVSDGADHRRRRSVVAPGLRHHHVQRYVATMVSNIDTVIDGWQPGQRLDIYQEL

RSAVRRSTAESLFGQRLAVHSDFLGEQLQPLLDLTRRPPQVMRLQQRVNSPGWRRAMAAR

KRIDDLIDAQIADARTAPRPDDHMLTTLISGCSEEGTTLSDNEIRDSIVSLITAGYETTS

GALAWAIYALLTVPGTWESAASEVARVLGGRVPAADDLSALTYLNGVVHETLRLYSPGVI

SARRVLRDLWFDGHRIRAGRLLIFSAYVTHRLPEIWPEPTEFRPLRWDPNAADYRKPAPH

EFIPFSGGLHRCIGAVMATTEMTVILARLVARAMLQLPAQRTHRIRAANFAALRPWPGLT

VEIRKSAPAQ

>CYP139A1(2577879644)Mycobacterium tuberculosis MD18478

MRYPLGEALLALYRWRGPLINAGVGGHGYTYLLGAEANRFVFANADAFSWSQTFESLVPV

DGPTALIVSDGADHRRRRSVVAPGLRHHHVQRYVATMVSNIDTVIDGWQPGQRLDIYQEL

RSAVRRSTAESLFGQRLAVHSDFLGEQLQPLLDLTRRPPQVMRLQQRVNSPGWRRAMAAR

KRIDDLIDAQIADARTAPRPDDHMLTTLISGCSEEGTTLSDNEIRDSIVSLITAGYETTS

GALAWAIYALLTVPGTWESAASEVARVLGGRVPAADDLSALTYLNGVVHETLRLYSPGVI

SARRVLRDLWFDGHRIRAGRLLIFSAYVTHRLPEIWPEPTEFRPLRWDPNAADYRKPAPH

EFIPFSGGLHRCIGAVMATTEMTVILARLVARAMLQLPAQRTHRIRAANFAALRPWPGLT

VEIRKSAPAQ

>CYP139A1(2577954418)Mycobacterium tuberculosis 1615

MRYPLGEALLALYRWRGPLINAGVGGHGYTYLLGAEANRFVFANADAFSWSQTFESLVPV

DGPTALIVSDGADHRRRRSVVAPGLRHHHVQRYVATMVSNIDTVIDGWQPGQRLDIYQEL

RSAVRRSTAESLFGQRLAVHSDFLGEQLQPLLDLTRRPPQVMRLQQRVNSPGWRRAMAAR

KRIDDLIDAQIADARTAPRPDDHMLTTLISGCSEEGTTLSDNEIRDSIVSLITAGYETTS

GALAWAIYALLTVPGTWESAASEVARVLGGRVPAADDLSALTYLNGVVHETLRLYSPGVI

SARRVLRDLWFDGHRIRAGRLLIFSAYVTHRLPEIWPEPTEFRPLRWDPNAADYRKPAPH

EFIPFSGGLHRCIGAVMATTEMTVILARLVARAMLQLPAQRTHRIRAANFAALRPWPGLT

VEIRKSAPAQ

>CYP139A1(2584625495)Mycobacterium tuberculosis TKK-01-0064

MRYPLGEALLALYRWRGPLINAGVGGHGYTYLLGAEANRFVFANADAFSWSQTFESLVPV

DGPTALIVSDGADHRRRRSVVAPGLRHHHVQRYVATMVSNIDTVIDGWQPGQRLDIYQEL

RSAVRRSTAESLFGQRLAVHSDFLGEQLQPLLDLTRRPPQVMRLQQRVNSPGWRRAMAAR

KRIDDLIDAQIADARTAPRPDDHMLTTLISGCSEEGTTLSDNEIRDSIVSLITAGYETTS

GALAWAIYALLTVPGTWESAASEVARVLGGRVPAADDLSALTYLNGVVHETLRLYSPGVI

SARRVLRDLWFDGHRIRAGRLLIFSAYVTHRLPEIWPEPTEFRPLRWDPNAADYRKPAPH

EFIPFSGGLHRCIGAVMATTEMTVILARLVARAMLQLPAQRTHRIRAANFAALRPWPGLT

VEIRKSAPAQ

>CYP139A1(2589526877)Mycobacterium tuberculosis TKK-01-0022

MRYPLGEALLALYRWRGPLINAGVGGHGYTYLLGAEANRFVFANADAFSWSQTFESLVPV

DGPTALIVSDGADHRRRRSVVAPGLRHHHVQRYVATMVSNIDTVIDGWQPGQRLDIYQEL

RSAVRRSTAESLFGQRLAVHSDFLGEQLQPLLDLTRRPPQVMRLQQRVNSPGWRRAMAAR

KRIDDLIDAQIADARTAPRPDDHMLTTLISGCSEEGTTLSDNEIRDSIVSLITAGYETTS

GALAWAIYALLTVPGTWESAASEVARVLGGRVPAADDLSALTYLNGVVHETLRLYSPGVI

SARRVLRDLWFDGHRIRAGRLLIFSAYVTHRLPEIWPEPTEFRPLRWDPNAADYRKPAPH

EFIPFSGGLHRCIGAVMATTEMTVILARLVARAMLQLPAQRTHRIRAANFAALRPWPGLT

VEIRKSAPAQ

>CYP139A1(2589711853)Mycobacterium tuberculosis TKK-01-0083

MRYPLGEALLALYRWRGPLINAGVGGHGYTYLLGAEANRFVFANADAFSWSQTFESLVPV

DGPTALIVSDGADHRRRRSVVAPGLRHHHVQRYVATMVSNIDTVIDGWQPGQRLDIYQEL

RSAVRRSTAESLFGQRLAVHSDFLGEQLQPLLDLTRRPPQVMRLQQRVNSPGWRRAMAAR

KRIDDLIDAQIADARTAPRPDDHMLTTLISGCSEEGTTLSDNEIRDSIVSLITAGYETTS

GALAWAIYALLTVPGTWESAASEVARVLGGRVPAADDLSALTYLNGVVHETLRLYSPGVI

SARRVLRDLWFDGHRIRAGRLLIFSAYVTHRLPEIWPEPTEFRPLRWDPNAADYRKPAPH

EFIPFSGGLHRCIGAVMATTEMTVILARLVARAMLQLPAQRTHRIRAANFAALRPWPGLT

VEIRKSAPAQ

>CYP139A1(2590113796)Mycobacterium tuberculosis MAL020156

MRYPLGEALLALYRWRGPLINAGVGGHGYTYLLGAEANRFVFANADAFSWSQTFESLVPV

DGPTALIVSDGADHRRRRSVVAPGLRHHHVQRYVATMVSNIDTVIDGWQPGQRLDIYQEL

RSAVRRSTAESLFGQRLAVHSDFLGEQLQPLLDLTRRPPQVMRLQQRVNSPGWRRAMAAR

KRIDDLIDAQIADARTAPRPDDHMLTTLISGCSEEGTTLSDNEIRDSIVSLITAGYETTS

GALAWAIYALLTVPGTWESAASEVARVLGGRVPAADDLSALTYLNGVVHETLRLYSPGVI

SARRVLRDLWFDGHRIRAGRLLIFSAYVTHRLPEIWPEPTEFRPLRWDPNAADYRKPAPH

EFIPFSGGLHRCIGAVMATTEMTVILARLVARAMLQLPAQRTHRIRAANFAALRPWPGLT

VEIRKSAPAQ

>CYP139A1(2592324422)Mycobacterium tuberculosis TKK_03_0020

MRYPLGEALLALYRWRGPLINAGVGGHGYTYLLGAEANRFVFANADAFSWSQTFESLVPV

DGPTALIVSDGADHRRRRSVVAPGLRHHHVQRYVATMVSNIDTVIDGWQPGQRLDIYQEL

RSAVRRSTAESLFGQRLAVHSDFLGEQLQPLLDLTRRPPQVMRLQQRVNSPGWRRAMAAR

KRIDDLIDAQIADARTAPRPDDHMLTTLISGCSEEGTTLSDNEIRDSIVSLITAGYETTS

GALAWAIYALLTVPGTWESAASEVARVLGGRVPAADDLSALTYLNGVVHETLRLYSPGVI

SARRVLRDLWFDGHRIRAGRLLIFSAYVTHRLPEIWPEPTEFRPLRWDPNAADYRKPAPH

EFIPFSGGLHRCIGAVMATTEMTVILARLVARAMLQLPAQRTHRIRAANFAALRPWPGLT

VEIRKSAPAQ

>CYP139A1(2592337997)Mycobacterium tuberculosis TKK_02_0073

MRYPLGEALLALYRWRGPLINAGVGGHGYTYLLGAEANRFVFANADAFSWSQTFESLVPV

DGPTALIVSDGADHRRRRSVVAPGLRHHHVQRYVATMVSNIDTVIDGWQPGQRLDIYQEL

RSAVRRSTAESLFGQRLAVHSDFLGEQLQPLLDLTRRPPQVMRLQQRVNSPGWRRAMAAR

KRIDDLIDAQIADARTAPRPDDHMLTTLISGCSEEGTTLSDNEIRDSIVSLITAGYETTS

GALAWAIYALLTVPGTWESAASEVARVLGGRVPAADDLSALTYLNGVVHETLRLYSPGVI

SARRVLRDLWFDGHRIRAGRLLIFSAYVTHRLPEIWPEPTEFRPLRWDPNAADYRKPAPH

EFIPFSGGLHRCIGAVMATTEMTVILARLVARAMLQLPAQRTHRIRAANFAALRPWPGLT

VEIRKSAPAQ

>CYP139A1(2592373455)Mycobacterium tuberculosis TKK_02_0046

MRYPLGEALLALYRWRGPLINAGVGGHGYTYLLGAEANRFVFANADAFSWSQTFESLVPV

DGPTALIVSDGADHRRRRSVVAPGLRHHHVQRYVATMVSNIDTVIDGWQPGQRLDIYQEL

RSAVRRSTAESLFGQRLAVHSDFLGEQLQPLLDLTRRPPQVMRLQQRVNSPGWRRAMAAR

KRIDDLIDAQIADARTAPRPDDHMLTTLISGCSEEGTTLSDNEIRDSIVSLITAGYETTS

GALAWAIYALLTVPGTWESAASEVARVLGGRVPAADDLSALTYLNGVVHETLRLYSPGVI

SARRVLRDLWFDGHRIRAGRLLIFSAYVTHRLPEIWPEPTEFRPLRWDPNAADYRKPAPH

EFIPFSGGLHRCIGAVMATTEMTVILARLVARAMLQLPAQRTHRIRAANFAALRPWPGLT

VEIRKSAPAQ

>CYP139A1(2592445915)Mycobacterium tuberculosis SK-C

MRYPLGEALLALYRWRGPLINAGVGGHGYTYLLGAEANRFVFANADAFSWSQTFESLVPV

DGPTALIVSDGADHRRRRSVVAPGLRHHHVQRYVATMVSNIDTVIDGWQPGQRLDIYQEL

RSAVRRSTAESLFGQRLAVHSDFLGEQLQPLLDLTRRPPQVMRLQQRVNSPGWRRAMAAR

KRIDDLIDAQIADARTAPRPDDHMLTTLISGCSEEGTTLSDNEIRDSIVSLITAGYETTS

GALAWAIYALLTVPGTWESAASEVARVLGGRVPAADDLSALTYLNGVVHETLRLYSPGVI

SARRVLRDLWFDGHRIRAGRLLIFSAYVTHRLPEIWPEPTEFRPLRWDPNAADYRKPAPH

EFIPFSGGLHRCIGAVMATTEMTVILARLVARAMLQLPAQRTHRIRAANFAALRPWPGLT

VEIRKSAPAQ

>CYP139A1(2575023271)Mycobacterium tuberculosis H3361

MRYPLGEALLALYRWRGPLINAGVGGHGYTYLLGAEANRFVFANADAFSWSQTFESLVPV

DGPTALIVSDGADHRRRRSVVAPGLRHHHVQRYVATMVSNIDTVIDGWQPGQRLDIYQEL

RSAVRRSTAESLFGQRLAVHSDFLGEQLQPLLDLTRRPPQVMRLQQRVNSPGWRRAMAAR

KRIDDLIDAQIADARTAPRPDDHMLTTLISGCSEEGTTLSDNEIRDSIVSLITAGYETTS

GALAWAIYALLTVPGTWESAASEVARVLGGRVPAADDLSALTYLNGVVHETLRLYSPGVI

SARRVLRDLWFDGHRIRAGRLLIFSAYVTHRLPEIWPEPTEFRPLRWDPNAADYRKPAPH

EFIPFSGGLHRCIGAVMATTEMTVILARLVARAMLQLPAQRTHRIRAANFAALRPWPGLT

VEIRKSAPAQ

>CYP139A1(2575619239)Mycobacterium tuberculosis OFXR-7

MRYPLGEALLALYRWRGPLINAGVGGHGYTYLLGAEANRFVFANADAFSWSQTFESLVPV

DGPTALIVSDGADHRRRRSVVAPGLRHHHVQRYVATMVSNIDTVIDGWQPGQRLDIYQEL

RSAVRRSTAESLFGQRLAVHSDFLGEQLQPLLDLTRRPPQVMRLQQRVNSPGWRRAMAAR

KRIDDLIDAQIADARTAPRPDDHMLTTLISGCSEEGTTLSDNEIRDSIVSLITAGYETTS

GALAWAIYALLTVPGTWESAASEVARVLGGRVPAADDLSALTYLNGVVHETLRLYSPGVI

SARRVLRDLWFDGHRIRAGRLLIFSAYVTHRLPEIWPEPTEFRPLRWDPNAADYRKPAPH

EFIPFSGGLHRCIGAVMATTEMTVILARLVARAMLQLPAQRTHRIRAANFAALRPWPGLT

VEIRKSAPAQ

>CYP139A1(2575786887)Mycobacterium tuberculosis TKK-01-0019

MRYPLGEALLALYRWRGPLINAGVGGHGYTYLLGAEANRFVFANADAFSWSQTFESLVPV

DGPTALIVSDGADHRRRRSVVAPGLRHHHVQRYVATMVSNIDTVIDGWQPGQRLDIYQEL

RSAVRRSTAESLFGQRLAVHSDFLGEQLQPLLDLTRRPPQVMRLQQRVNSPGWRRAMAAR

KRIDDLIDAQIADARTAPRPDDHMLTTLISGCSEEGTTLSDNEIRDSIVSLITAGYETTS

GALAWAIYALLTVPGTWESAASEVARVLGGRVPAADDLSALTYLNGVVHETLRLYSPGVI

SARRVLRDLWFDGHRIRAGRLLIFSAYVTHRLPEIWPEPTEFRPLRWDPNAADYRKPAPH

EFIPFSGGLHRCIGAVMATTEMTVILARLVARAMLQLPAQRTHRIRAANFAALRPWPGLT

VEIRKSAPAQ

>CYP139A1(2576882264)Mycobacterium tuberculosis TB_RSA163

MRYPLGEALLALYRWRGPLINAGVGGHGYTYLLGAEANRFVFANADAFSWSQTFESLVPV

DGPTALIVSDGADHRRRRSVVAPGLRHHHVQRYVATMVSNIDTVIDGWQPGQRLDIYQEL

RSAVRRSTAESLFGQRLAVHSDFLGEQLQPLLDLTRRPPQVMRLQQRVNSPGWRRAMAAR

KRIDDLIDAQIADARTAPRPDDHMLTTLISGCSEEGTTLSDNEIRDSIVSLITAGYETTS

GALAWAIYALLTVPGTWESAASEVARVLGGRVPAADDLSALTYLNGVVHETLRLYSPGVI

SARRVLRDLWFDGHRIRAGRLLIFSAYVTHRLPEIWPEPTEFRPLRWDPNAADYRKPAPH

EFIPFSGGLHRCIGAVMATTEMTVILARLVARAMLQLPAQRTHRIRAANFAALRPWPGLT

VEIRKSAPAQ

>CYP139A1(2577215885)Mycobacterium tuberculosis TKK_05MA_0012

MRYPLGEALLALYRWRGPLINAGVGGHGYTYLLGAEANRFVFANADAFSWSQTFESLVPV

DGPTALIVSDGADHRRRRSVVAPGLRHHHVQRYVATMVSNIDTVIDGWQPGQRLDIYQEL

RSAVRRSTAESLFGQRLAVHSDFLGEQLQPLLDLTRRPPQVMRLQQRVNSPGWRRAMAAR

KRIDDLIDAQIADARTAPRPDDHMLTTLISGCSEEGTTLSDNEIRDSIVSLITAGYETTS

GALAWAIYALLTVPGTWESAASEVARVLGGRVPAADDLSALTYLNGVVHETLRLYSPGVI

SARRVLRDLWFDGHRIRAGRLLIFSAYVTHRLPEIWPEPTEFRPLRWDPNAADYRKPAPH

EFIPFSGGLHRCIGAVMATTEMTVILARLVARAMLQLPAQRTHRIRAANFAALRPWPGLT

VEIRKSAPAQ

>CYP139A1(2577627248)Mycobacterium tuberculosis XTB13-161

MRYPLGEALLALYRWRGPLINAGVGGHGYTYLLGAEANRFVFANADAFSWSQTFESLVPV

DGPTALIVSDGADHRRRRSVVAPGLRHHHVQRYVATMVSNIDTVIDGWQPGQRLDIYQEL

RSAVRRSTAESLFGQRLAVHSDFLGEQLQPLLDLTRRPPQVMRLQQRVNSPGWRRAMAAR

KRIDDLIDAQIADARTAPRPDDHMLTTLISGCSEEGTTLSDNEIRDSIVSLITAGYETTS

GALAWAIYALLTVPGTWESAASEVARVLGGRVPAADDLSALTYLNGVVHETLRLYSPGVI

SARRVLRDLWFDGHRIRAGRLLIFSAYVTHRLPEIWPEPTEFRPLRWDPNAADYRKPAPH

EFIPFSGGLHRCIGAVMATTEMTVILARLVARAMLQLPAQRTHRIRAANFAALRPWPGLT

VEIRKSAPAQ

>CYP139A1(2577923998)Mycobacterium tuberculosis NRITLD14

MRYPLGEALLALYRWRGPLINAGVGGHGYTYLLGAEANRFVFANADAFSWSQTFESLVPV

DGPTALIVSDGADHRRRRSVVAPGLRHHHVQRYVATMVSNIDTVIDGWQPGQRLDIYQEL

RSAVRRSTAESLFGQRLAVHSDFLGEQLQPLLDLTRRPPQVMRLQQRVNSPGWRRAMAAR

KRIDDLIDAQIADARTAPRPDDHMLTTLISGCSEEGTTLSDNEIRDSIVSLITAGYETTS

GALAWAIYALLTVPGTWESAASEVARVLGGRVPAADDLSALTYLNGVVHETLRLYSPGVI

SARRVLRDLWFDGHRIRAGRLLIFSAYVTHRLPEIWPEPTEFRPLRWDPNAADYRKPAPH

EFIPFSGGLHRCIGAVMATTEMTVILARLVARAMLQLPAQRTHRIRAANFAALRPWPGLT

VEIRKSAPAQ

>CYP139A1(2583735989)*Mycobacterium africanum* MAL020130

MRYPLGEALLALYRWRGPLINAGVGGHGYTYLLGAEANRFVFANADAFSWSQTFESLVPV

DGPTALIVSDGADHRRRRSVVAPGLRHHHVQRYVATMVSNIDTVIDGWQPGQRLDIYQEL

RSAVRRSTAESLFGQRLAVHSDFLGEQLQPLLDLTRRPPQVMRLQQRVNSPGWRRAMAAR

KRIDDLIDAQIADARTAPRPDDHMLTTLISGCSEEGTTLSDNEIRDSIVSLITAGYETTS

GALAWAIYALLTVPGTWESAASEVARVLGGRVPAADDLSALTYLNGVVHETLRLYSPGVI

SARRVLRDLWFDGHRIRAGRLLIFSAYVTHRLPEIWPEPTEFRPLRWDPNAADYRKPAPH

EFIPFSGGLHRCIGAVMATTEMTVILARLVARAMLQLPAQRTHRIRAANFAALRPWPGLT

VEIRKSAPAQ

>CYP139A1(2584003651)Mycobacterium bovisWt 21231

MRYPLGEALLALYRWRGPLINAGVGGHGYTYLLGAEANRFVFANADAFSWSQTFESLVPV

DGPTALIVSDGADHRRRRSVVAPGLRHHHVQRYVATMVSNIDTVIDGWQPGQRLDIYQEL

RSAVRRSTAESLFGQRLAVHSDFLGEQLQPLLDLTRRPPQVMRLQQRVNSPGWRRAMAAR

KRIDDLIDAQIADARTAPRPDDHMLTTLISGCSEEGTTLSDNEIRDSIVSLITAGYETTS

GALAWAIYALLTVPGTWESAASEVARVLGGRVPAADDLSALTYLNGVVHETLRLYSPGVI

SARRVLRDLWFDGHRIRAGRLLIFSAYVTHRLPEIWPEPTEFRPLRWDPNAADYRKPAPH

EFIPFSGGLHRCIGAVMATTEMTVILARLVARAMLQLPAQRTHRIRAANFAALRPWPGLT

VEIRKSAPAQ

>CYP139A1(2584623655)Mycobacterium tuberculosis M1340

MRYPLGEALLALYRWRGPLINAGVGGHGYTYLLGAEANRFVFANADAFSWSQTFESLVPV

DGPTALIVSDGADHRRRRSVVAPGLRHHHVQRYVATMVSNIDTVIDGWQPGQRLDIYQEL

RSAVRRSTAESLFGQRLAVHSDFLGEQLQPLLDLTRRPPQVMRLQQRVNSPGWRRAMAAR

KRIDDLIDAQIADARTAPRPDDHMLTTLISGCSEEGTTLSDNEIRDSIVSLITAGYETTS

GALAWAIYALLTVPGTWESAASEVARVLGGRVPAADDLSALTYLNGVVHETLRLYSPGVI

SARRVLRDLWFDGHRIRAGRLLIFSAYVTHRLPEIWPEPTEFRPLRWDPNAADYRKPAPH

EFIPFSGGLHRCIGAVMATTEMTVILARLVARAMLQLPAQRTHRIRAANFAALRPWPGLT

VEIRKSAPAQ

>CYP139A1(2584739857)Mycobacterium tuberculosis XTB13-092

MRYPLGEALLALYRWRGPLINAGVGGHGYTYLLGAEANRFVFANADAFSWSQTFESLVPV

DGPTALIVSDGADHRRRRSVVAPGLRHHHVQRYVATMVSNIDTVIDGWQPGQRLDIYQEL

RSAVRRSTAESLFGQRLAVHSDFLGEQLQPLLDLTRRPPQVMRLQQRVNSPGWRRAMAAR

KRIDDLIDAQIADARTAPRPDDHMLTTLISGCSEEGTTLSDNEIRDSIVSLITAGYETTS

GALAWAIYALLTVPGTWESAASEVARVLGGRVPAADDLSALTYLNGVVHETLRLYSPGVI

SARRVLRDLWFDGHRIRAGRLLIFSAYVTHRLPEIWPEPTEFRPLRWDPNAADYRKPAPH

EFIPFSGGLHRCIGAVMATTEMTVILARLVARAMLQLPAQRTHRIRAANFAALRPWPGLT

VEIRKSAPAQ

>CYP139A1(2584858071)Mycobacterium tuberculosis TKK_04_0045

MRYPLGEALLALYRWRGPLINAGVGGHGYTYLLGAEANRFVFANADAFSWSQTFESLVPV

DGPTALIVSDGADHRRRRSVVAPGLRHHHVQRYVATMVSNIDTVIDGWQPGQRLDIYQEL

RSAVRRSTAESLFGQRLAVHSDFLGEQLQPLLDLTRRPPQVMRLQQRVNSPGWRRAMAAR

KRIDDLIDAQIADARTAPRPDDHMLTTLISGCSEEGTTLSDNEIRDSIVSLITAGYETTS

GALAWAIYALLTVPGTWESAASEVARVLGGRVPAADDLSALTYLNGVVHETLRLYSPGVI

SARRVLRDLWFDGHRIRAGRLLIFSAYVTHRLPEIWPEPTEFRPLRWDPNAADYRKPAPH

EFIPFSGGLHRCIGAVMATTEMTVILARLVARAMLQLPAQRTHRIRAANFAALRPWPGLT

VEIRKSAPAQ

>CYP139A1(2584928246)Mycobacterium tuberculosis M2346

MRYPLGEALLALYRWRGPLINAGVGGHGYTYLLGAEANRFVFANADAFSWSQTFESLVPV

DGPTALIVSDGADHRRRRSVVAPGLRHHHVQRYVATMVSNIDTVIDGWQPGQRLDIYQEL

RSAVRRSTAESLFGQRLAVHSDFLGEQLQPLLDLTRRPPQVMRLQQRVNSPGWRRAMAAR

KRIDDLIDAQIADARTAPRPDDHMLTTLISGCSEEGTTLSDNEIRDSIVSLITAGYETTS

GALAWAIYALLTVPGTWESAASEVARVLGGRVPAADDLSALTYLNGVVHETLRLYSPGVI

SARRVLRDLWFDGHRIRAGRLLIFSAYVTHRLPEIWPEPTEFRPLRWDPNAADYRKPAPH

EFIPFSGGLHRCIGAVMATTEMTVILARLVARAMLQLPAQRTHRIRAANFAALRPWPGLT

VEIRKSAPAQ

>CYP139A1(2589040293)Mycobacterium tuberculosis TBR11

MRYPLGEALLALYRWRGPLINAGVGGHGYTYLLGAEANRFVFANADAFSWSQTFESLVPV

DGPTALIVSDGADHRRRRSVVAPGLRHHHVQRYVATMVSNIDTVIDGWQPGQRLDIYQEL

RSAVRRSTAESLFGQRLAVHSDFLGEQLQPLLDLTRRPPQVMRLQQRVNSPGWRRAMAAR

KRIDDLIDAQIADARTAPRPDDHMLTTLISGCSEEGTTLSDNEIRDSIVSLITAGYETTS

GALAWAIYALLTVPGTWESAASEVARVLGGRVPAADDLSALTYLNGVVHETLRLYSPGVI

SARRVLRDLWFDGHRIRAGRLLIFSAYVTHRLPEIWPEPTEFRPLRWDPNAADYRKPAPH

EFIPFSGGLHRCIGAVMATTEMTVILARLVARAMLQLPAQRTHRIRAANFAALRPWPGLT

VEIRKSAPAQ

>CYP139A1(2589658610)Mycobacterium tuberculosis TKK-01-0054

MRYPLGEALLALYRWRGPLINAGVGGHGYTYLLGAEANRFVFANADAFSWSQTFESLVPV

DGPTALIVSDGADHRRRRSVVAPGLRHHHVQRYVATMVSNIDTVIDGWQPGQRLDIYQEL

RSAVRRSTAESLFGQRLAVHSDFLGEQLQPLLDLTRRPPQVMRLQQRVNSPGWRRAMAAR

KRIDDLIDAQIADARTAPRPDDHMLTTLISGCSEEGTTLSDNEIRDSIVSLITAGYETTS

GALAWAIYALLTVPGTWESAASEVARVLGGRVPAADDLSALTYLNGVVHETLRLYSPGVI

SARRVLRDLWFDGHRIRAGRLLIFSAYVTHRLPEIWPEPTEFRPLRWDPNAADYRKPAPH

EFIPFSGGLHRCIGAVMATTEMTVILARLVARAMLQLPAQRTHRIRAANFAALRPWPGLT

VEIRKSAPAQ

>CYP139A1(2590162679)Mycobacterium tuberculosis MAL020196

MRYPLGEALLALYRWRGPLINAGVGGHGYTYLLGAEANRFVFANADAFSWSQTFESLVPV

DGPTALIVSDGADHRRRRSVVAPGLRHHHVQRYVATMVSNIDTVIDGWQPGQRLDIYQEL

RSAVRRSTAESLFGQRLAVHSDFLGEQLQPLLDLTRRPPQVMRLQQRVNSPGWRRAMAAR

KRIDDLIDAQIADARTAPRPDDHMLTTLISGCSEEGTTLSDNEIRDSIVSLITAGYETTS

GALAWAIYALLTVPGTWESAASEVARVLGGRVPAADDLSALTYLNGVVHETLRLYSPGVI

SARRVLRDLWFDGHRIRAGRLLIFSAYVTHRLPEIWPEPTEFRPLRWDPNAADYRKPAPH

EFIPFSGGLHRCIGAVMATTEMTVILARLVARAMLQLPAQRTHRIRAANFAALRPWPGLT

VEIRKSAPAQ

>CYP139A1(2590243884)Mycobacterium tuberculosis KT-0035

MRYPLGEALLALYRWRGPLINAGVGGHGYTYLLGAEANRFVFANADAFSWSQTFESLVPV

DGPTALIVSDGADHRRRRSVVAPGLRHHHVQRYVATMVSNIDTVIDGWQPGQRLDIYQEL

RSAVRRSTAESLFGQRLAVHSDFLGEQLQPLLDLTRRPPQVMRLQQRVNSPGWRRAMAAR

KRIDDLIDAQIADARTAPRPDDHMLTTLISGCSEEGTTLSDNEIRDSIVSLITAGYETTS

GALAWAIYALLTVPGTWESAASEVARVLGGRVPAADDLSALTYLNGVVHETLRLYSPGVI

SARRVLRDLWFDGHRIRAGRLLIFSAYVTHRLPEIWPEPTEFRPLRWDPNAADYRKPAPH

EFIPFSGGLHRCIGAVMATTEMTVILARLVARAMLQLPAQRTHRIRAANFAALRPWPGLT

VEIRKSAPAQ

>CYP139A1(2590531681)Mycobacterium tuberculosis KT-0084

MRYPLGEALLALYRWRGPLINAGVGGHGYTYLLGAEANRFVFANADAFSWSQTFESLVPV

DGPTALIVSDGADHRRRRSVVAPGLRHHHVQRYVATMVSNIDTVIDGWQPGQRLDIYQEL

RSAVRRSTAESLFGQRLAVHSDFLGEQLQPLLDLTRRPPQVMRLQQRVNSPGWRRAMAAR

KRIDDLIDAQIADARTAPRPDDHMLTTLISGCSEEGTTLSDNEIRDSIVSLITAGYETTS

GALAWAIYALLTVPGTWESAASEVARVLGGRVPAADDLSALTYLNGVVHETLRLYSPGVI

SARRVLRDLWFDGHRIRAGRLLIFSAYVTHRLPEIWPEPTEFRPLRWDPNAADYRKPAPH

EFIPFSGGLHRCIGAVMATTEMTVILARLVARAMLQLPAQRTHRIRAANFAALRPWPGLT

VEIRKSAPAQ

>CYP139A1(2574614619)Mycobacterium tuberculosis TB_RSA111

MRYPLGEALLALYRWRGPLINAGVGGHGYTYLLGAEANRFVFANADAFSWSQTFESLVPV

DGPTALIVSDGADHRRRRSVVAPGLRHHHVQRYVATMVSNIDTVIDGWQPGQRLDIYQEL

RSAVRRSTAESLFGQRLAVHSDFLGEQLQPLLDLTRRPPQVMRLQQRVNSPGWRRAMAAR

KRIDDLIDAQIADARTAPRPDDHMLTTLISGCSEEGTTLSDNEIRDSIVSLITAGYETTS

GALAWAIYALLTVPGTWESAASEVARVLGGRVPAADDLSALTYLNGVVHETLRLYSPGVI

SARRVLRDLWFDGHRIRAGRLLIFSAYVTHRLPEIWPEPTEFRPLRWDPNAADYRKPAPH

EFIPFSGGLHRCIGAVMATTEMTVILARLVARAMLQLPAQRTHRIRAANFAALRPWPGLT

VEIRKSAPAQ

>CYP139A1(2575016195)Mycobacterium tuberculosis BTB12-001

MRYPLGEALLALYRWRGPLINAGVGGHGYTYLLGAEANRFVFANADAFSWSQTFESLVPV

DGPTALIVSDGADHRRRRSVVAPGLRHHHVQRYVATMVSNIDTVIDGWQPGQRLDIYQEL

RSAVRRSTAESLFGQRLAVHSDFLGEQLQPLLDLTRRPPQVMRLQQRVNSPGWRRAMAAR

KRIDDLIDAQIADARTAPRPDDHMLTTLISGCSEEGTTLSDNEIRDSIVSLITAGYETTS

GALAWAIYALLTVPGTWESAASEVARVLGGRVPAADDLSALTYLNGVVHETLRLYSPGVI

SARRVLRDLWFDGHRIRAGRLLIFSAYVTHRLPEIWPEPTEFRPLRWDPNAADYRKPAPH

EFIPFSGGLHRCIGAVMATTEMTVILARLVARAMLQLPAQRTHRIRAANFAALRPWPGLT

VEIRKSAPAQ

>CYP139A1(2575426848)Mycobacterium tuberculosis TB_RSA118

MRYPLGEALLALYRWRGPLINAGVGGHGYTYLLGAEANRFVFANADAFSWSQTFESLVPV

DGPTALIVSDGADHRRRRSVVAPGLRHHHVQRYVATMVSNIDTVIDGWQPGQRLDIYQEL

RSAVRRSTAESLFGQRLAVHSDFLGEQLQPLLDLTRRPPQVMRLQQRVNSPGWRRAMAAR

KRIDDLIDAQIADARTAPRPDDHMLTTLISGCSEEGTTLSDNEIRDSIVSLITAGYETTS

GALAWAIYALLTVPGTWESAASEVARVLGGRVPAADDLSALTYLNGVVHETLRLYSPGVI

SARRVLRDLWFDGHRIRAGRLLIFSAYVTHRLPEIWPEPTEFRPLRWDPNAADYRKPAPH

EFIPFSGGLHRCIGAVMATTEMTVILARLVARAMLQLPAQRTHRIRAANFAALRPWPGLT

VEIRKSAPAQ

>CYP139A1(2576630848)Mycobacterium tuberculosis TB_RSA59

MRYPLGEALLALYRWRGPLINAGVGGHGYTYLLGAEANRFVFANADAFSWSQTFESLVPV

DGPTALIVSDGADHRRRRSVVAPGLRHHHVQRYVATMVSNIDTVIDGWQPGQRLDIYQEL

RSAVRRSTAESLFGQRLAVHSDFLGEQLQPLLDLTRRPPQVMRLQQRVNSPGWRRAMAAR

KRIDDLIDAQIADARTAPRPDDHMLTTLISGCSEEGTTLSDNEIRDSIVSLITAGYETTS

GALAWAIYALLTVPGTWESAASEVARVLGGRVPAADDLSALTYLNGVVHETLRLYSPGVI

SARRVLRDLWFDGHRIRAGRLLIFSAYVTHRLPEIWPEPTEFRPLRWDPNAADYRKPAPH

EFIPFSGGLHRCIGAVMATTEMTVILARLVARAMLQLPAQRTHRIRAANFAALRPWPGLT

VEIRKSAPAQ

>CYP139A1(2577468911)Mycobacterium tuberculosis NRITLD56

MRYPLGEALLALYRWRGPLINAGVGGHGYTYLLGAEANRFVFANADAFSWSQTFESLVPV

DGPTALIVSDGADHRRRRSVVAPGLRHHHVQRYVATMVSNIDTVIDGWQPGQRLDIYQEL

RSAVRRSTAESLFGQRLAVHSDFLGEQLQPLLDLTRRPPQVMRLQQRVNSPGWRRAMAAR

KRIDDLIDAQIADARTAPRPDDHMLTTLISGCSEEGTTLSDNEIRDSIVSLITAGYETTS

GALAWAIYALLTVPGTWESAASEVARVLGGRVPAADDLSALTYLNGVVHETLRLYSPGVI

SARRVLRDLWFDGHRIRAGRLLIFSAYVTHRLPEIWPEPTEFRPLRWDPNAADYRKPAPH

EFIPFSGGLHRCIGAVMATTEMTVILARLVARAMLQLPAQRTHRIRAANFAALRPWPGLT

VEIRKSAPAQ

>CYP139A1(2577974906)Mycobacterium tuberculosis XTB13-214

MRYPLGEALLALYRWRGPLINAGVGGHGYTYLLGAEANRFVFANADAFSWSQTFESLVPV

DGPTALIVSDGADHRRRRSVVAPGLRHHHVQRYVATMVSNIDTVIDGWQPGQRLDIYQEL

RSAVRRSTAESLFGQRLAVHSDFLGEQLQPLLDLTRRPPQVMRLQQRVNSPGWRRAMAAR

KRIDDLIDAQIADARTAPRPDDHMLTTLISGCSEEGTTLSDNEIRDSIVSLITAGYETTS

GALAWAIYALLTVPGTWESAASEVARVLGGRVPAADDLSALTYLNGVVHETLRLYSPGVI

SARRVLRDLWFDGHRIRAGRLLIFSAYVTHRLPEIWPEPTEFRPLRWDPNAADYRKPAPH

EFIPFSGGLHRCIGAVMATTEMTVILARLVARAMLQLPAQRTHRIRAANFAALRPWPGLT

VEIRKSAPAQ

>CYP139A1(2578062326)Mycobacterium tuberculosis BTB09-565

MRYPLGEALLALYRWRGPLINAGVGGHGYTYLLGAEANRFVFANADAFSWSQTFESLVPV

DGPTALIVSDGADHRRRRSVVAPGLRHHHVQRYVATMVSNIDTVIDGWQPGQRLDIYQEL

RSAVRRSTAESLFGQRLAVHSDFLGEQLQPLLDLTRRPPQVMRLQQRVNSPGWRRAMAAR

KRIDDLIDAQIADARTAPRPDDHMLTTLISGCSEEGTTLSDNEIRDSIVSLITAGYETTS

GALAWAIYALLTVPGTWESAASEVARVLGGRVPAADDLSALTYLNGVVHETLRLYSPGVI

SARRVLRDLWFDGHRIRAGRLLIFSAYVTHRLPEIWPEPTEFRPLRWDPNAADYRKPAPH

EFIPFSGGLHRCIGAVMATTEMTVILARLVARAMLQLPAQRTHRIRAANFAALRPWPGLT

VEIRKSAPAQ

>CYP139A1(2584836956)Mycobacterium tuberculosis TKK_04_0038

MRYPLGEALLALYRWRGPLINAGVGGHGYTYLLGAEANRFVFANADAFSWSQTFESLVPV

DGPTALIVSDGADHRRRRSVVAPGLRHHHVQRYVATMVSNIDTVIDGWQPGQRLDIYQEL

RSAVRRSTAESLFGQRLAVHSDFLGEQLQPLLDLTRRPPQVMRLQQRVNSPGWRRAMAAR

KRIDDLIDAQIADARTAPRPDDHMLTTLISGCSEEGTTLSDNEIRDSIVSLITAGYETTS

GALAWAIYALLTVPGTWESAASEVARVLGGRVPAADDLSALTYLNGVVHETLRLYSPGVI

SARRVLRDLWFDGHRIRAGRLLIFSAYVTHRLPEIWPEPTEFRPLRWDPNAADYRKPAPH

EFIPFSGGLHRCIGAVMATTEMTVILARLVARAMLQLPAQRTHRIRAANFAALRPWPGLT

VEIRKSAPAQ

>CYP139A1(2584998642)Mycobacterium tuberculosis KT-0109

MRYPLGEALLALYRWRGPLINAGVGGHGYTYLLGAEANRFVFANADAFSWSQTFESLVPV

DGPTALIVSDGADHRRRRSVVAPGLRHHHVQRYVATMVSNIDTVIDGWQPGQRLDIYQEL

RSAVRRSTAESLFGQRLAVHSDFLGEQLQPLLDLTRRPPQVMRLQQRVNSPGWRRAMAAR

KRIDDLIDAQIADARTAPRPDDHMLTTLISGCSEEGTTLSDNEIRDSIVSLITAGYETTS

GALAWAIYALLTVPGTWESAASEVARVLGGRVPAADDLSALTYLNGVVHETLRLYSPGVI

SARRVLRDLWFDGHRIRAGRLLIFSAYVTHRLPEIWPEPTEFRPLRWDPNAADYRKPAPH

EFIPFSGGLHRCIGAVMATTEMTVILARLVARAMLQLPAQRTHRIRAANFAALRPWPGLT

VEIRKSAPAQ

>CYP139A1(2589130082)Mycobacterium tuberculosis TBR80

MRYPLGEALLALYRWRGPLINAGVGGHGYTYLLGAEANRFVFANADAFSWSQTFESLVPV

DGPTALIVSDGADHRRRRSVVAPGLRHHHVQRYVATMVSNIDTVIDGWQPGQRLDIYQEL

RSAVRRSTAESLFGQRLAVHSDFLGEQLQPLLDLTRRPPQVMRLQQRVNSPGWRRAMAAR

KRIDDLIDAQIADARTAPRPDDHMLTTLISGCSEEGTTLSDNEIRDSIVSLITAGYETTS

GALAWAIYALLTVPGTWESAASEVARVLGGRVPAADDLSALTYLNGVVHETLRLYSPGVI

SARRVLRDLWFDGHRIRAGRLLIFSAYVTHRLPEIWPEPTEFRPLRWDPNAADYRKPAPH

EFIPFSGGLHRCIGAVMATTEMTVILARLVARAMLQLPAQRTHRIRAANFAALRPWPGLT

VEIRKSAPAQ

>CYP139A1(2589592089)Mycobacterium tuberculosis TKK-01-0034

MRYPLGEALLALYRWRGPLINAGVGGHGYTYLLGAEANRFVFANADAFSWSQTFESLVPV

DGPTALIVSDGADHRRRRSVVAPGLRHHHVQRYVATMVSNIDTVIDGWQPGQRLDIYQEL

RSAVRRSTAESLFGQRLAVHSDFLGEQLQPLLDLTRRPPQVMRLQQRVNSPGWRRAMAAR

KRIDDLIDAQIADARTAPRPDDHMLTTLISGCSEEGTTLSDNEIRDSIVSLITAGYETTS

GALAWAIYALLTVPGTWESAASEVARVLGGRVPAADDLSALTYLNGVVHETLRLYSPGVI

SARRVLRDLWFDGHRIRAGRLLIFSAYVTHRLPEIWPEPTEFRPLRWDPNAADYRKPAPH

EFIPFSGGLHRCIGAVMATTEMTVILARLVARAMLQLPAQRTHRIRAANFAALRPWPGLT

VEIRKSAPAQ

>CYP139A1(2590052832)Mycobacterium tuberculosis MAL010130

MRYPLGEALLALYRWRGPLINAGVGGHGYTYLLGAEANRFVFANADAFSWSQTFESLVPV

DGPTALIVSDGADHRRRRSVVAPGLRHHHVQRYVATMVSNIDTVIDGWQPGQRLDIYQEL

RSAVRRSTAESLFGQRLAVHSDFLGEQLQPLLDLTRRPPQVMRLQQRVNSPGWRRAMAAR

KRIDDLIDAQIADARTAPRPDDHMLTTLISGCSEEGTTLSDNEIRDSIVSLITAGYETTS

GALAWAIYALLTVPGTWESAASEVARVLGGRVPAADDLSALTYLNGVVHETLRLYSPGVI

SARRVLRDLWFDGHRIRAGRLLIFSAYVTHRLPEIWPEPTEFRPLRWDPNAADYRKPAPH

EFIPFSGGLHRCIGAVMATTEMTVILARLVARAMLQLPAQRTHRIRAANFAALRPWPGLT

VEIRKSAPAQ

>CYP139A1(2590505688)Mycobacterium tuberculosis KT-0102

MRYPLGEALLALYRWRGPLINAGVGGHGYTYLLGAEANRFVFANADAFSWSQTFESLVPV

DGPTALIVSDGADHRRRRSVVAPGLRHHHVQRYVATMVSNIDTVIDGWQPGQRLDIYQEL

RSAVRRSTAESLFGQRLAVHSDFLGEQLQPLLDLTRRPPQVMRLQQRVNSPGWRRAMAAR

KRIDDLIDAQIADARTAPRPDDHMLTTLISGCSEEGTTLSDNEIRDSIVSLITAGYETTS

GALAWAIYALLTVPGTWESAASEVARVLGGRVPAADDLSALTYLNGVVHETLRLYSPGVI

SARRVLRDLWFDGHRIRAGRLLIFSAYVTHRLPEIWPEPTEFRPLRWDPNAADYRKPAPH

EFIPFSGGLHRCIGAVMATTEMTVILARLVARAMLQLPAQRTHRIRAANFAALRPWPGLT

VEIRKSAPAQ

>CYP139A1(2592353030)Mycobacterium tuberculosis TKK_02_0067

MRYPLGEALLALYRWRGPLINAGVGGHGYTYLLGAEANRFVFANADAFSWSQTFESLVPV

DGPTALIVSDGADHRRRRSVVAPGLRHHHVQRYVATMVSNIDTVIDGWQPGQRLDIYQEL

RSAVRRSTAESLFGQRLAVHSDFLGEQLQPLLDLTRRPPQVMRLQQRVNSPGWRRAMAAR

KRIDDLIDAQIADARTAPRPDDHMLTTLISGCSEEGTTLSDNEIRDSIVSLITAGYETTS

GALAWAIYALLTVPGTWESAASEVARVLGGRVPAADDLSALTYLNGVVHETLRLYSPGVI

SARRVLRDLWFDGHRIRAGRLLIFSAYVTHRLPEIWPEPTEFRPLRWDPNAADYRKPAPH

EFIPFSGGLHRCIGAVMATTEMTVILARLVARAMLQLPAQRTHRIRAANFAALRPWPGLT

VEIRKSAPAQ

>CYP139A1(637026884)Mycobacterium tuberculosis H37Rv

MRYPLGEALLALYRWRGPLINAGVGGHGYTYLLGAEANRFVFANADAFSWSQTFESLVPV

DGPTALIVSDGADHRRRRSVVAPGLRHHHVQRYVATMVSNIDTVIDGWQPGQRLDIYQEL

RSAVRRSTAESLFGQRLAVHSDFLGEQLQPLLDLTRRPPQVMRLQQRVNSPGWRRAMAAR

KRIDDLIDAQIADARTAPRPDDHMLTTLISGCSEEGTTLSDNEIRDSIVSLITAGYETTS

GALAWAIYALLTVPGTWESAASEVARVLGGRVPAADDLSALTYLNGVVHETLRLYSPGVI

SARRVLRDLWFDGHRIRAGRLLIFSAYVTHRLPEIWPEPTEFRPLRWDPNAADYRKPAPH

EFIPFSGGLHRCIGAVMATTEMTVILARLVARAMLQLPAQRTHRIRAANFAALRPWPGLT

VEIRKSAPAQ

>CYP139A1(2574886309)Mycobacterium tuberculosis TBR65

MRYPLGEALLALYRWRGPLINAGVGGHGYTYLLGAEANRFVFANADAFSWSQTFESLVPV

DGPTALIVSDGADHRRRRSVVAPGLRHHHVQRYVATMVSNIDTVIDGWQPGQRLDIYQEL

RSAVRRSTAESLFGQRLAVHSDFLGEQLQPLLDLTRRPPQVMRLQQRVNSPGWRRAMAAR

KRIDDLIDAQIADARTAPRPDDHMLTTLISGCSEEGTTLSDNEIRDSIVSLITAGYETTS

GALAWAIYALLTVPGTWESAASEVARVLGGRVPAADDLSALTYLNGVVHETLRLYSPGVI

SARRVLRDLWFDGHRIRAGRLLIFSAYVTHRLPEIWPEPTEFRPLRWDPNAADYRKPAPH

EFIPFSGGLHRCIGAVMATTEMTVILARLVARAMLQLPAQRTHRIRAANFAALRPWPGLT

VEIRKSAPAQ

>CYP139A1(2575295342)Mycobacterium tuberculosis TB_RSA148

MRYPLGEALLALYRWRGPLINAGVGGHGYTYLLGAEANRFVFANADAFSWSQTFESLVPV

DGPTALIVSDGADHRRRRSVVAPGLRHHHVQRYVATMVSNIDTVIDGWQPGQRLDIYQEL

RSAVRRSTAESLFGQRLAVHSDFLGEQLQPLLDLTRRPPQVMRLQQRVNSPGWRRAMAAR

KRIDDLIDAQIADARTAPRPDDHMLTTLISGCSEEGTTLSDNEIRDSIVSLITAGYETTS

GALAWAIYALLTVPGTWESAASEVARVLGGRVPAADDLSALTYLNGVVHETLRLYSPGVI

SARRVLRDLWFDGHRIRAGRLLIFSAYVTHRLPEIWPEPTEFRPLRWDPNAADYRKPAPH

EFIPFSGGLHRCIGAVMATTEMTVILARLVARAMLQLPAQRTHRIRAANFAALRPWPGLT

VEIRKSAPAQ

>CYP139A1(2575942274)Mycobacterium tuberculosis XTB13-200

MRYPLGEALLALYRWRGPLINAGVGGHGYTYLLGAEANRFVFANADAFSWSQTFESLVPV

DGPTALIVSDGADHRRRRSVVAPGLRHHHVQRYVATMVSNIDTVIDGWQPGQRLDIYQEL

RSAVRRSTAESLFGQRLAVHSDFLGEQLQPLLDLTRRPPQVMRLQQRVNSPGWRRAMAAR

KRIDDLIDAQIADARTAPRPDDHMLTTLISGCSEEGTTLSDNEIRDSIVSLITAGYETTS

GALAWAIYALLTVPGTWESAASEVARVLGGRVPAADDLSALTYLNGVVHETLRLYSPGVI

SARRVLRDLWFDGHRIRAGRLLIFSAYVTHRLPEIWPEPTEFRPLRWDPNAADYRKPAPH

EFIPFSGGLHRCIGAVMATTEMTVILARLVARAMLQLPAQRTHRIRAANFAALRPWPGLT

VEIRKSAPAQ

>CYP139A1(2576123248)Mycobacterium tuberculosis M1906

MRYPLGEALLALYRWRGPLINAGVGGHGYTYLLGAEANRFVFANADAFSWSQTFESLVPV

DGPTALIVSDGADHRRRRSVVAPGLRHHHVQRYVATMVSNIDTVIDGWQPGQRLDIYQEL

RSAVRRSTAESLFGQRLAVHSDFLGEQLQPLLDLTRRPPQVMRLQQRVNSPGWRRAMAAR

KRIDDLIDAQIADARTAPRPDDHMLTTLISGCSEEGTTLSDNEIRDSIVSLITAGYETTS

GALAWAIYALLTVPGTWESAASEVARVLGGRVPAADDLSALTYLNGVVHETLRLYSPGVI

SARRVLRDLWFDGHRIRAGRLLIFSAYVTHRLPEIWPEPTEFRPLRWDPNAADYRKPAPH

EFIPFSGGLHRCIGAVMATTEMTVILARLVARAMLQLPAQRTHRIRAANFAALRPWPGLT

VEIRKSAPAQ

>CYP139A1(2576712596)Mycobacterium tuberculosis KT-0056

MRYPLGEALLALYRWRGPLINAGVGGHGYTYLLGAEANRFVFANADAFSWSQTFESLVPV

DGPTALIVSDGADHRRRRSVVAPGLRHHHVQRYVATMVSNIDTVIDGWQPGQRLDIYQEL

RSAVRRSTAESLFGQRLAVHSDFLGEQLQPLLDLTRRPPQVMRLQQRVNSPGWRRAMAAR

KRIDDLIDAQIADARTAPRPDDHMLTTLISGCSEEGTTLSDNEIRDSIVSLITAGYETTS

GALAWAIYALLTVPGTWESAASEVARVLGGRVPAADDLSALTYLNGVVHETLRLYSPGVI

SARRVLRDLWFDGHRIRAGRLLIFSAYVTHRLPEIWPEPTEFRPLRWDPNAADYRKPAPH

EFIPFSGGLHRCIGAVMATTEMTVILARLVARAMLQLPAQRTHRIRAANFAALRPWPGLT

VEIRKSAPAQ

>CYP139A1(2577856904)Mycobacterium tuberculosis TKK_05SA_0014

MRYPLGEALLALYRWRGPLINAGVGGHGYTYLLGAEANRFVFANADAFSWSQTFESLVPV

DGPTALIVSDGADHRRRRSVVAPGLRHHHVQRYVATMVSNIDTVIDGWQPGQRLDIYQEL

RSAVRRSTAESLFGQRLAVHSDFLGEQLQPLLDLTRRPPQVMRLQQRVNSPGWRRAMAAR

KRIDDLIDAQIADARTAPRPDDHMLTTLISGCSEEGTTLSDNEIRDSIVSLITAGYETTS

GALAWAIYALLTVPGTWESAASEVARVLGGRVPAADDLSALTYLNGVVHETLRLYSPGVI

SARRVLRDLWFDGHRIRAGRLLIFSAYVTHRLPEIWPEPTEFRPLRWDPNAADYRKPAPH

EFIPFSGGLHRCIGAVMATTEMTVILARLVARAMLQLPAQRTHRIRAANFAALRPWPGLT

VEIRKSAPAQ

>CYP139A1(2578013153)Mycobacterium tuberculosis TB_RSA32

MRYPLGEALLALYRWRGPLINAGVGGHGYTYLLGAEANRFVFANADAFSWSQTFESLVPV

DGPTALIVSDGADHRRRRSVVAPGLRHHHVQRYVATMVSNIDTVIDGWQPGQRLDIYQEL

RSAVRRSTAESLFGQRLAVHSDFLGEQLQPLLDLTRRPPQVMRLQQRVNSPGWRRAMAAR

KRIDDLIDAQIADARTAPRPDDHMLTTLISGCSEEGTTLSDNEIRDSIVSLITAGYETTS

GALAWAIYALLTVPGTWESAASEVARVLGGRVPAADDLSALTYLNGVVHETLRLYSPGVI

SARRVLRDLWFDGHRIRAGRLLIFSAYVTHRLPEIWPEPTEFRPLRWDPNAADYRKPAPH

EFIPFSGGLHRCIGAVMATTEMTVILARLVARAMLQLPAQRTHRIRAANFAALRPWPGLT

VEIRKSAPAQ

>CYP139A1(2578182623)Mycobacterium tuberculosis TKK-01-0025

MRYPLGEALLALYRWRGPLINAGVGGHGYTYLLGAEANRFVFANADAFSWSQTFESLVPV

DGPTALIVSDGADHRRRRSVVAPGLRHHHVQRYVATMVSNIDTVIDGWQPGQRLDIYQEL

RSAVRRSTAESLFGQRLAVHSDFLGEQLQPLLDLTRRPPQVMRLQQRVNSPGWRRAMAAR

KRIDDLIDAQIADARTAPRPDDHMLTTLISGCSEEGTTLSDNEIRDSIVSLITAGYETTS

GALAWAIYALLTVPGTWESAASEVARVLGGRVPAADDLSALTYLNGVVHETLRLYSPGVI

SARRVLRDLWFDGHRIRAGRLLIFSAYVTHRLPEIWPEPTEFRPLRWDPNAADYRKPAPH

EFIPFSGGLHRCIGAVMATTEMTVILARLVARAMLQLPAQRTHRIRAANFAALRPWPGLT

VEIRKSAPAQ

>CYP139A1(2580771058)Mycobacterium bovisMr 4387

MRYPLGEALLALYRWRGPLINAGVGGHGYTYLLGAEANRFVFANADAFSWSQTFESLVPV

DGPTALIVSDGADHRRRRSVVAPGLRHHHVQRYVATMVSNIDTVIDGWQPGQRLDIYQEL

RSAVRRSTAESLFGQRLAVHSDFLGEQLQPLLDLTRRPPQVMRLQQRVNSPGWRRAMAAR

KRIDDLIDAQIADARTAPRPDDHMLTTLISGCSEEGTTLSDNEIRDSIVSLITAGYETTS

GALAWAIYALLTVPGTWESAASEVARVLGGRVPAADDLSALTYLNGVVHETLRLYSPGVI

SARRVLRDLWFDGHRIRAGRLLIFSAYVTHRLPEIWPEPTEFRPLRWDPNAADYRKPAPH

EFIPFSGGLHRCIGAVMATTEMTVILARLVARAMLQLPAQRTHRIRAANFAALRPWPGLT

VEIRKSAPAQ

>CYP139A1(2584641128)Mycobacterium tuberculosis XTB13-088

MRYPLGEALLALYRWRGPLINAGVGGHGYTYLLGAEANRFVFANADAFSWSQTFESLVPV

DGPTALIVSDGADHRRRRSVVAPGLRHHHVQRYVATMVSNIDTVIDGWQPGQRLDIYQEL

RSAVRRSTAESLFGQRLAVHSDFLGEQLQPLLDLTRRPPQVMRLQQRVNSPGWRRAMAAR

KRIDDLIDAQIADARTAPRPDDHMLTTLISGCSEEGTTLSDNEIRDSIVSLITAGYETTS

GALAWAIYALLTVPGTWESAASEVARVLGGRVPAADDLSALTYLNGVVHETLRLYSPGVI

SARRVLRDLWFDGHRIRAGRLLIFSAYVTHRLPEIWPEPTEFRPLRWDPNAADYRKPAPH

EFIPFSGGLHRCIGAVMATTEMTVILARLVARAMLQLPAQRTHRIRAANFAALRPWPGLT

VEIRKSAPAQ

>CYP139A1(2584649146)Mycobacterium tuberculosis M2203

MRYPLGEALLALYRWRGPLINAGVGGHGYTYLLGAEANRFVFANADAFSWSQTFESLVPV

DGPTALIVSDGADHRRRRSVVAPGLRHHHVQRYVATMVSNIDTVIDGWQPGQRLDIYQEL

RSAVRRSTAESLFGQRLAVHSDFLGEQLQPLLDLTRRPPQVMRLQQRVNSPGWRRAMAAR

KRIDDLIDAQIADARTAPRPDDHMLTTLISGCSEEGTTLSDNEIRDSIVSLITAGYETTS

GALAWAIYALLTVPGTWESAASEVARVLGGRVPAADDLSALTYLNGVVHETLRLYSPGVI

SARRVLRDLWFDGHRIRAGRLLIFSAYVTHRLPEIWPEPTEFRPLRWDPNAADYRKPAPH

EFIPFSGGLHRCIGAVMATTEMTVILARLVARAMLQLPAQRTHRIRAANFAALRPWPGLT

VEIRKSAPAQ

>CYP139A1(2584660713)Mycobacterium tuberculosis TRUG0072

MRYPLGEALLALYRWRGPLINAGVGGHGYTYLLGAEANRFVFANADAFSWSQTFESLVPV

DGPTALIVSDGADHRRRRSVVAPGLRHHHVQRYVATMVSNIDTVIDGWQPGQRLDIYQEL

RSAVRRSTAESLFGQRLAVHSDFLGEQLQPLLDLTRRPPQVMRLQQRVNSPGWRRAMAAR

KRIDDLIDAQIADARTAPRPDDHMLTTLISGCSEEGTTLSDNEIRDSIVSLITAGYETTS

GALAWAIYALLTVPGTWESAASEVARVLGGRVPAADDLSALTYLNGVVHETLRLYSPGVI

SARRVLRDLWFDGHRIRAGRLLIFSAYVTHRLPEIWPEPTEFRPLRWDPNAADYRKPAPH

EFIPFSGGLHRCIGAVMATTEMTVILARLVARAMLQLPAQRTHRIRAANFAALRPWPGLT

VEIRKSAPAQ

>CYP139A1(2584967006)Mycobacterium tuberculosis TKK_04_0002

MRYPLGEALLALYRWRGPLINAGVGGHGYTYLLGAEANRFVFANADAFSWSQTFESLVPV

DGPTALIVSDGADHRRRRSVVAPGLRHHHVQRYVATMVSNIDTVIDGWQPGQRLDIYQEL

RSAVRRSTAESLFGQRLAVHSDFLGEQLQPLLDLTRRPPQVMRLQQRVNSPGWRRAMAAR

KRIDDLIDAQIADARTAPRPDDHMLTTLISGCSEEGTTLSDNEIRDSIVSLITAGYETTS

GALAWAIYALLTVPGTWESAASEVARVLGGRVPAADDLSALTYLNGVVHETLRLYSPGVI

SARRVLRDLWFDGHRIRAGRLLIFSAYVTHRLPEIWPEPTEFRPLRWDPNAADYRKPAPH

EFIPFSGGLHRCIGAVMATTEMTVILARLVARAMLQLPAQRTHRIRAANFAALRPWPGLT

VEIRKSAPAQ

>CYP139A1(2584970624)Mycobacterium tuberculosis BTB12-384

MRYPLGEALLALYRWRGPLINAGVGGHGYTYLLGAEANRFVFANADAFSWSQTFESLVPV

DGPTALIVSDGADHRRRRSVVAPGLRHHHVQRYVATMVSNIDTVIDGWQPGQRLDIYQEL

RSAVRRSTAESLFGQRLAVHSDFLGEQLQPLLDLTRRPPQVMRLQQRVNSPGWRRAMAAR

KRIDDLIDAQIADARTAPRPDDHMLTTLISGCSEEGTTLSDNEIRDSIVSLITAGYETTS

GALAWAIYALLTVPGTWESAASEVARVLGGRVPAADDLSALTYLNGVVHETLRLYSPGVI

SARRVLRDLWFDGHRIRAGRLLIFSAYVTHRLPEIWPEPTEFRPLRWDPNAADYRKPAPH

EFIPFSGGLHRCIGAVMATTEMTVILARLVARAMLQLPAQRTHRIRAANFAALRPWPGLT

VEIRKSAPAQ

>CYP139A1(2589053585)Mycobacterium tuberculosis TBR23

MRYPLGEALLALYRWRGPLINAGVGGHGYTYLLGAEANRFVFANADAFSWSQTFESLVPV

DGPTALIVSDGADHRRRRSVVAPGLRHHHVQRYVATMVSNIDTVIDGWQPGQRLDIYQEL

RSAVRRSTAESLFGQRLAVHSDFLGEQLQPLLDLTRRPPQVMRLQQRVNSPGWRRAMAAR

KRIDDLIDAQIADARTAPRPDDHMLTTLISGCSEEGTTLSDNEIRDSIVSLITAGYETTS

GALAWAIYALLTVPGTWESAASEVARVLGGRVPAADDLSALTYLNGVVHETLRLYSPGVI

SARRVLRDLWFDGHRIRAGRLLIFSAYVTHRLPEIWPEPTEFRPLRWDPNAADYRKPAPH

EFIPFSGGLHRCIGAVMATTEMTVILARLVARAMLQLPAQRTHRIRAANFAALRPWPGLT

VEIRKSAPAQ

>CYP139A1(2589498327)Mycobacterium tuberculosis TKK-01-0007

MRYPLGEALLALYRWRGPLINAGVGGHGYTYLLGAEANRFVFANADAFSWSQTFESLVPV

DGPTALIVSDGADHRRRRSVVAPGLRHHHVQRYVATMVSNIDTVIDGWQPGQRLDIYQEL

RSAVRRSTAESLFGQRLAVHSDFLGEQLQPLLDLTRRPPQVMRLQQRVNSPGWRRAMAAR

KRIDDLIDAQIADARTAPRPDDHMLTTLISGCSEEGTTLSDNEIRDSIVSLITAGYETTS

GALAWAIYALLTVPGTWESAASEVARVLGGRVPAADDLSALTYLNGVVHETLRLYSPGVI

SARRVLRDLWFDGHRIRAGRLLIFSAYVTHRLPEIWPEPTEFRPLRWDPNAADYRKPAPH

EFIPFSGGLHRCIGAVMATTEMTVILARLVARAMLQLPAQRTHRIRAANFAALRPWPGLT

VEIRKSAPAQ

>CYP139A1(2589563529)Mycobacterium tuberculosis TKK-01-0028

MRYPLGEALLALYRWRGPLINAGVGGHGYTYLLGAEANRFVFANADAFSWSQTFESLVPV

DGPTALIVSDGADHRRRRSVVAPGLRHHHVQRYVATMVSNIDTVIDGWQPGQRLDIYQEL

RSAVRRSTAESLFGQRLAVHSDFLGEQLQPLLDLTRRPPQVMRLQQRVNSPGWRRAMAAR

KRIDDLIDAQIADARTAPRPDDHMLTTLISGCSEEGTTLSDNEIRDSIVSLITAGYETTS

GALAWAIYALLTVPGTWESAASEVARVLGGRVPAADDLSALTYLNGVVHETLRLYSPGVI

SARRVLRDLWFDGHRIRAGRLLIFSAYVTHRLPEIWPEPTEFRPLRWDPNAADYRKPAPH

EFIPFSGGLHRCIGAVMATTEMTVILARLVARAMLQLPAQRTHRIRAANFAALRPWPGLT

VEIRKSAPAQ

>CYP139A1(2590377218)Mycobacterium tuberculosis OFXR-20

MRYPLGEALLALYRWRGPLINAGVGGHGYTYLLGAEANRFVFANADAFSWSQTFESLVPV

DGPTALIVSDGADHRRRRSVVAPGLRHHHVQRYVATMVSNIDTVIDGWQPGQRLDIYQEL

RSAVRRSTAESLFGQRLAVHSDFLGEQLQPLLDLTRRPPQVMRLQQRVNSPGWRRAMAAR

KRIDDLIDAQIADARTAPRPDDHMLTTLISGCSEEGTTLSDNEIRDSIVSLITAGYETTS

GALAWAIYALLTVPGTWESAASEVARVLGGRVPAADDLSALTYLNGVVHETLRLYSPGVI

SARRVLRDLWFDGHRIRAGRLLIFSAYVTHRLPEIWPEPTEFRPLRWDPNAADYRKPAPH

EFIPFSGGLHRCIGAVMATTEMTVILARLVARAMLQLPAQRTHRIRAANFAALRPWPGLT

VEIRKSAPAQ

>CYP139A1(2592283614)Mycobacterium tuberculosis TKK_03_0045

MRYPLGEALLALYRWRGPLINAGVGGHGYTYLLGAEANRFVFANADAFSWSQTFESLVPV

DGPTALIVSDGADHRRRRSVVAPGLRHHHVQRYVATMVSNIDTVIDGWQPGQRLDIYQEL

RSAVRRSTAESLFGQRLAVHSDFLGEQLQPLLDLTRRPPQVMRLQQRVNSPGWRRAMAAR

KRIDDLIDAQIADARTAPRPDDHMLTTLISGCSEEGTTLSDNEIRDSIVSLITAGYETTS

GALAWAIYALLTVPGTWESAASEVARVLGGRVPAADDLSALTYLNGVVHETLRLYSPGVI

SARRVLRDLWFDGHRIRAGRLLIFSAYVTHRLPEIWPEPTEFRPLRWDPNAADYRKPAPH

EFIPFSGGLHRCIGAVMATTEMTVILARLVARAMLQLPAQRTHRIRAANFAALRPWPGLT

VEIRKSAPAQ

>CYP139A1(2592319784)Mycobacterium tuberculosis TKK_03_0022

MRYPLGEALLALYRWRGPLINAGVGGHGYTYLLGAEANRFVFANADAFSWSQTFESLVPV

DGPTALIVSDGADHRRRRSVVAPGLRHHHVQRYVATMVSNIDTVIDGWQPGQRLDIYQEL

RSAVRRSTAESLFGQRLAVHSDFLGEQLQPLLDLTRRPPQVMRLQQRVNSPGWRRAMAAR

KRIDDLIDAQIADARTAPRPDDHMLTTLISGCSEEGTTLSDNEIRDSIVSLITAGYETTS

GALAWAIYALLTVPGTWESAASEVARVLGGRVPAADDLSALTYLNGVVHETLRLYSPGVI

SARRVLRDLWFDGHRIRAGRLLIFSAYVTHRLPEIWPEPTEFRPLRWDPNAADYRKPAPH

EFIPFSGGLHRCIGAVMATTEMTVILARLVARAMLQLPAQRTHRIRAANFAALRPWPGLT

VEIRKSAPAQ

>CYP139A1(2575106637)Mycobacterium tuberculosis 16955

MRYPLGEALLALYRWRGPLINAGVGGHGYTYLLGAEANRFVFANADAFSWSQTFESLVPV

DGPTALIVSDGADHRRRRSVVAPGLRHHHVQRYVATMVSNIDTVIDGWQPGQRLDIYQEL

RSAVRRSTAESLFGQRLAVHSDFLGEQLQPLLDLTRRPPQVMRLQQRVNSPGWRRAMAAR

KRIDDLIDAQIADARTAPRPDDHMLTTLISGCSEEGTTLSDNEIRDSIVSLITAGYETTS

GALAWAIYALLTVPGTWESAASEVARVLGGRVPAADDLSALTYLNGVVHETLRLYSPGVI

SARRVLRDLWFDGHRIRAGRLLIFSAYVTHRLPEIWPEPTEFRPLRWDPNAADYRKPAPH

EFIPFSGGLHRCIGAVMATTEMTVILARLVARAMLQLPAQRTHRIRAANFAALRPWPGLT

VEIRKSAPAQ

>CYP139A1(2575157076)Mycobacterium tuberculosis BTB08-362

MRYPLGEALLALYRWRGPLINAGVGGHGYTYLLGAEANRFVFANADAFSWSQTFESLVPV

DGPTALIVSDGADHRRRRSVVAPGLRHHHVQRYVATMVSNIDTVIDGWQPGQRLDIYQEL

RSAVRRSTAESLFGQRLAVHSDFLGEQLQPLLDLTRRPPQVMRLQQRVNSPGWRRAMAAR

KRIDDLIDAQIADARTAPRPDDHMLTTLISGCSEEGTTLSDNEIRDSIVSLITAGYETTS

GALAWAIYALLTVPGTWESAASEVARVLGGRVPAADDLSALTYLNGVVHETLRLYSPGVI

SARRVLRDLWFDGHRIRAGRLLIFSAYVTHRLPEIWPEPTEFRPLRWDPNAADYRKPAPH

EFIPFSGGLHRCIGAVMATTEMTVILARLVARAMLQLPAQRTHRIRAANFAALRPWPGLT

VEIRKSAPAQ

>CYP139A1(2575361778)Mycobacterium tuberculosis M1449

MRYPLGEALLALYRWRGPLINAGVGGHGYTYLLGAEANRFVFANADAFSWSQTFESLVPV

DGPTALIVSDGADHRRRRSVVAPGLRHHHVQRYVATMVSNIDTVIDGWQPGQRLDIYQEL

RSAVRRSTAESLFGQRLAVHSDFLGEQLQPLLDLTRRPPQVMRLQQRVNSPGWRRAMAAR

KRIDDLIDAQIADARTAPRPDDHMLTTLISGCSEEGTTLSDNEIRDSIVSLITAGYETTS

GALAWAIYALLTVPGTWESAASEVARVLGGRVPAADDLSALTYLNGVVHETLRLYSPGVI

SARRVLRDLWFDGHRIRAGRLLIFSAYVTHRLPEIWPEPTEFRPLRWDPNAADYRKPAPH

EFIPFSGGLHRCIGAVMATTEMTVILARLVARAMLQLPAQRTHRIRAANFAALRPWPGLT

VEIRKSAPAQ

>CYP139A1(2576009184)Mycobacterium tuberculosis BTB04-452

MRYPLGEALLALYRWRGPLINAGVGGHGYTYLLGAEANRFVFANADAFSWSQTFESLVPV

DGPTALIVSDGADHRRRRSVVAPGLRHHHVQRYVATMVSNIDTVIDGWQPGQRLDIYQEL

RSAVRRSTAESLFGQRLAVHSDFLGEQLQPLLDLTRRPPQVMRLQQRVNSPGWRRAMAAR

KRIDDLIDAQIADARTAPRPDDHMLTTLISGCSEEGTTLSDNEIRDSIVSLITAGYETTS

GALAWAIYALLTVPGTWESAASEVARVLGGRVPAADDLSALTYLNGVVHETLRLYSPGVI

SARRVLRDLWFDGHRIRAGRLLIFSAYVTHRLPEIWPEPTEFRPLRWDPNAADYRKPAPH

EFIPFSGGLHRCIGAVMATTEMTVILARLVARAMLQLPAQRTHRIRAANFAALRPWPGLT

VEIRKSAPAQ

>CYP139A1(2576566954)Mycobacterium tuberculosis M1008

MRYPLGEALLALYRWRGPLINAGVGGHGYTYLLGAEANRFVFANADAFSWSQTFESLVPV

DGPTALIVSDGADHRRRRSVVAPGLRHHHVQRYVATMVSNIDTVIDGWQPGQRLDIYQEL

RSAVRRSTAESLFGQRLAVHSDFLGEQLQPLLDLTRRPPQVMRLQQRVNSPGWRRAMAAR

KRIDDLIDAQIADARTAPRPDDHMLTTLISGCSEEGTTLSDNEIRDSIVSLITAGYETTS

GALAWAIYALLTVPGTWESAASEVARVLGGRVPAADDLSALTYLNGVVHETLRLYSPGVI

SARRVLRDLWFDGHRIRAGRLLIFSAYVTHRLPEIWPEPTEFRPLRWDPNAADYRKPAPH

EFIPFSGGLHRCIGAVMATTEMTVILARLVARAMLQLPAQRTHRIRAANFAALRPWPGLT

VEIRKSAPAQ

>CYP139A1(2576731741)Mycobacterium tuberculosis M2131

MRYPLGEALLALYRWRGPLINAGVGGHGYTYLLGAEANRFVFANADAFSWSQTFESLVPV

DGPTALIVSDGADHRRRRSVVAPGLRHHHVQRYVATMVSNIDTVIDGWQPGQRLDIYQEL

RSAVRRSTAESLFGQRLAVHSDFLGEQLQPLLDLTRRPPQVMRLQQRVNSPGWRRAMAAR

KRIDDLIDAQIADARTAPRPDDHMLTTLISGCSEEGTTLSDNEIRDSIVSLITAGYETTS

GALAWAIYALLTVPGTWESAASEVARVLGGRVPAADDLSALTYLNGVVHETLRLYSPGVI

SARRVLRDLWFDGHRIRAGRLLIFSAYVTHRLPEIWPEPTEFRPLRWDPNAADYRKPAPH

EFIPFSGGLHRCIGAVMATTEMTVILARLVARAMLQLPAQRTHRIRAANFAALRPWPGLT

VEIRKSAPAQ

>CYP139A1(2581562358)*Mycobacterium africanum* MAL020148

MRYPLGEALLALYRWRGPLINAGVGGHGYTYLLGAEANRFVFANADAFSWSQTFESLVPV

DGPTALIVSDGADHRRRRSVVAPGLRHHHVQRYVATMVSNIDTVIDGWQPGQRLDIYQEL

RSAVRRSTAESLFGQRLAVHSDFLGEQLQPLLDLTRRPPQVMRLQQRVNSPGWRRAMAAR

KRIDDLIDAQIADARTAPRPDDHMLTTLISGCSEEGTTLSDNEIRDSIVSLITAGYETTS

GALAWAIYALLTVPGTWESAASEVARVLGGRVPAADDLSALTYLNGVVHETLRLYSPGVI

SARRVLRDLWFDGHRIRAGRLLIFSAYVTHRLPEIWPEPTEFRPLRWDPNAADYRKPAPH

EFIPFSGGLHRCIGAVMATTEMTVILARLVARAMLQLPAQRTHRIRAANFAALRPWPGLT

VEIRKSAPAQ

>CYP139A1(2584107430)*Mycobacterium bovis*Kc 32216

MRYPLGEALLALYRWRGPLINAGVGGHGYTYLLGAEANRFVFANADAFSWSQTFESLVPV

DGPTALIVSDGADHRRRRSVVAPGLRHHHVQRYVATMVSNIDTVIDGWQPGQRLDIYQEL

RSAVRRSTAESLFGQRLAVHSDFLGEQLQPLLDLTRRPPQVMRLQQRVNSPGWRRAMAAR

KRIDDLIDAQIADARTAPRPDDHMLTTLISGCSEEGTTLSDNEIRDSIVSLITAGYETTS

GALAWAIYALLTVPGTWESAASEVARVLGGRVPAADDLSALTYLNGVVHETLRLYSPGVI

SARRVLRDLWFDGHRIRAGRLLIFSAYVTHRLPEIWPEPTEFRPLRWDPNAADYRKPAPH

EFIPFSGGLHRCIGAVMATTEMTVILARLVARAMLQLPAQRTHRIRAANFAALRPWPGLT

VEIRKSAPAQ

>CYP139A1(2590025444)Mycobacterium tuberculosis MAL010088

MRYPLGEALLALYRWRGPLINAGVGGHGYTYLLGAEANRFVFANADAFSWSQTFESLVPV

DGPTALIVSDGADHRRRRSVVAPGLRHHHVQRYVATMVSNIDTVIDGWQPGQRLDIYQEL

RSAVRRSTAESLFGQRLAVHSDFLGEQLQPLLDLTRRPPQVMRLQQRVNSPGWRRAMAAR

KRIDDLIDAQIADARTAPRPDDHMLTTLISGCSEEGTTLSDNEIRDSIVSLITAGYETTS

GALAWAIYALLTVPGTWESAASEVARVLGGRVPAADDLSALTYLNGVVHETLRLYSPGVI

SARRVLRDLWFDGHRIRAGRLLIFSAYVTHRLPEIWPEPTEFRPLRWDPNAADYRKPAPH

EFIPFSGGLHRCIGAVMATTEMTVILARLVARAMLQLPAQRTHRIRAANFAALRPWPGLT

VEIRKSAPAQ

>CYP139A1(2590040558)Mycobacterium tuberculosis MAL010105

MRYPLGEALLALYRWRGPLINAGVGGHGYTYLLGAEANRFVFANADAFSWSQTFESLVPV

DGPTALIVSDGADHRRRRSVVAPGLRHHHVQRYVATMVSNIDTVIDGWQPGQRLDIYQEL

RSAVRRSTAESLFGQRLAVHSDFLGEQLQPLLDLTRRPPQVMRLQQRVNSPGWRRAMAAR

KRIDDLIDAQIADARTAPRPDDHMLTTLISGCSEEGTTLSDNEIRDSIVSLITAGYETTS

GALAWAIYALLTVPGTWESAASEVARVLGGRVPAADDLSALTYLNGVVHETLRLYSPGVI

SARRVLRDLWFDGHRIRAGRLLIFSAYVTHRLPEIWPEPTEFRPLRWDPNAADYRKPAPH

EFIPFSGGLHRCIGAVMATTEMTVILARLVARAMLQLPAQRTHRIRAANFAALRPWPGLT

VEIRKSAPAQ

>CYP139A1(2590214646)Mycobacterium tuberculosis KT-0058

MRYPLGEALLALYRWRGPLINAGVGGHGYTYLLGAEANRFVFANADAFSWSQTFESLVPV

DGPTALIVSDGADHRRRRSVVAPGLRHHHVQRYVATMVSNIDTVIDGWQPGQRLDIYQEL

RSAVRRSTAESLFGQRLAVHSDFLGEQLQPLLDLTRRPPQVMRLQQRVNSPGWRRAMAAR

KRIDDLIDAQIADARTAPRPDDHMLTTLISGCSEEGTTLSDNEIRDSIVSLITAGYETTS

GALAWAIYALLTVPGTWESAASEVARVLGGRVPAADDLSALTYLNGVVHETLRLYSPGVI

SARRVLRDLWFDGHRIRAGRLLIFSAYVTHRLPEIWPEPTEFRPLRWDPNAADYRKPAPH

EFIPFSGGLHRCIGAVMATTEMTVILARLVARAMLQLPAQRTHRIRAANFAALRPWPGLT

VEIRKSAPAQ

>CYP139A1(2590223019)Mycobacterium tuberculosis KT-0048

MRYPLGEALLALYRWRGPLINAGVGGHGYTYLLGAEANRFVFANADAFSWSQTFESLVPV

DGPTALIVSDGADHRRRRSVVAPGLRHHHVQRYVATMVSNIDTVIDGWQPGQRLDIYQEL

RSAVRRSTAESLFGQRLAVHSDFLGEQLQPLLDLTRRPPQVMRLQQRVNSPGWRRAMAAR

KRIDDLIDAQIADARTAPRPDDHMLTTLISGCSEEGTTLSDNEIRDSIVSLITAGYETTS

GALAWAIYALLTVPGTWESAASEVARVLGGRVPAADDLSALTYLNGVVHETLRLYSPGVI

SARRVLRDLWFDGHRIRAGRLLIFSAYVTHRLPEIWPEPTEFRPLRWDPNAADYRKPAPH

EFIPFSGGLHRCIGAVMATTEMTVILARLVARAMLQLPAQRTHRIRAANFAALRPWPGLT

VEIRKSAPAQ

>CYP139A1(2590266966)Mycobacterium tuberculosis KT-0016

MRYPLGEALLALYRWRGPLINAGVGGHGYTYLLGAEANRFVFANADAFSWSQTFESLVPV

DGPTALIVSDGADHRRRRSVVAPGLRHHHVQRYVATMVSNIDTVIDGWQPGQRLDIYQEL

RSAVRRSTAESLFGQRLAVHSDFLGEQLQPLLDLTRRPPQVMRLQQRVNSPGWRRAMAAR

KRIDDLIDAQIADARTAPRPDDHMLTTLISGCSEEGTTLSDNEIRDSIVSLITAGYETTS

GALAWAIYALLTVPGTWESAASEVARVLGGRVPAADDLSALTYLNGVVHETLRLYSPGVI

SARRVLRDLWFDGHRIRAGRLLIFSAYVTHRLPEIWPEPTEFRPLRWDPNAADYRKPAPH

EFIPFSGGLHRCIGAVMATTEMTVILARLVARAMLQLPAQRTHRIRAANFAALRPWPGLT

VEIRKSAPAQ

>CYP139A1(647086307)Mycobacterium tuberculosis KZN 4207

MRYPLGEALLALYRWRGPLINAGVGGHGYTYLLGAEANRFVFANADAFSWSQTFESLVPV

DGPTALIVSDGADHRRRRSVVAPGLRHHHVQRYVATMVSNIDTVIDGWQPGQRLDIYQEL

RSAVRRSTAESLFGQRLAVHSDFLGEQLQPLLDLTRRPPQVMRLQQRVNSPGWRRAMAAR

KRIDDLIDAQIADARTAPRPDDHMLTTLISGCSEEGTTLSDNEIRDSIVSLITAGYETTS

GALAWAIYALLTVPGTWESAASEVARVLGGRVPAADDLSALTYLNGVVHETLRLYSPGVI

SARRVLRDLWFDGHRIRAGRLLIFSAYVTHRLPEIWPEPTEFRPLRWDPNAADYRKPAPH

EFIPFSGGLHRCIGAVMATTEMTVILARLVARAMLQLPAQRTHRIRAANFAALRPWPGLT

VEIRKSAPAQ

>CYP139A1(2574726119)Mycobacterium tuberculosis TKK_02_0015

MRYPLGEALLALYRWRGPLINAGVGGHGYTYLLGAEANRFVFANADAFSWSQTFESLVPV

DGPTALIVSDGADHRRRRSVVAPGLRHHHVQRYVATMVSNIDTVIDGWQPGQRLDIYQEL

RSAVRRSTAESLFGQRLAVHSDFLGEQLQPLLDLTRRPPQVMRLQQRVNSPGWRRAMAAR

KRIDDLIDAQIADARTAPRPDDHMLTTLISGCSEEGTTLSDNEIRDSIVSLITAGYETTS

GALAWAIYALLTVPGTWESAASEVARVLGGRVPAADDLSALTYLNGVVHETLRLYSPGVI

SARRVLRDLWFDGHRIRAGRLLIFSAYVTHRLPEIWPEPTEFRPLRWDPNAADYRKPAPH

EFIPFSGGLHRCIGAVMATTEMTVILARLVARAMLQLPAQRTHRIRAANFAALRPWPGLT

VEIRKSAPAQ

>CYP139A1(2574757270)Mycobacterium tuberculosis T46

MRYPLGEALLALYRWRGPLINAGVGGHGYTYLLGAEANRFVFANADAFSWSQTFESLVPV

DGPTALIVSDGADHRRRRSVVAPGLRHHHVQRYVATMVSNIDTVIDGWQPGQRLDIYQEL

RSAVRRSTAESLFGQRLAVHSDFLGEQLQPLLDLTRRPPQVMRLQQRVNSPGWRRAMAAR

KRIDDLIDAQIADARTAPRPDDHMLTTLISGCSEEGTTLSDNEIRDSIVSLITAGYETTS

GALAWAIYALLTVPGTWESAASEVARVLGGRVPAADDLSALTYLNGVVHETLRLYSPGVI

SARRVLRDLWFDGHRIRAGRLLIFSAYVTHRLPEIWPEPTEFRPLRWDPNAADYRKPAPH

EFIPFSGGLHRCIGAVMATTEMTVILARLVARAMLQLPAQRTHRIRAANFAALRPWPGLT

VEIRKSAPAQ

>CYP139A1(2575280304)Mycobacterium tuberculosis MAL010133

MRYPLGEALLALYRWRGPLINAGVGGHGYTYLLGAEANRFVFANADAFSWSQTFESLVPV

DGPTALIVSDGADHRRRRSVVAPGLRHHHVQRYVATMVSNIDTVIDGWQPGQRLDIYQEL

RSAVRRSTAESLFGQRLAVHSDFLGEQLQPLLDLTRRPPQVMRLQQRVNSPGWRRAMAAR

KRIDDLIDAQIADARTAPRPDDHMLTTLISGCSEEGTTLSDNEIRDSIVSLITAGYETTS

GALAWAIYALLTVPGTWESAASEVARVLGGRVPAADDLSALTYLNGVVHETLRLYSPGVI

SARRVLRDLWFDGHRIRAGRLLIFSAYVTHRLPEIWPEPTEFRPLRWDPNAADYRKPAPH

EFIPFSGGLHRCIGAVMATTEMTVILARLVARAMLQLPAQRTHRIRAANFAALRPWPGLT

VEIRKSAPAQ

>CYP139A1(2575601683)Mycobacterium tuberculosis TKK_05SA_0012

MRYPLGEALLALYRWRGPLINAGVGGHGYTYLLGAEANRFVFANADAFSWSQTFESLVPV

DGPTALIVSDGADHRRRRSVVAPGLRHHHVQRYVATMVSNIDTVIDGWQPGQRLDIYQEL

RSAVRRSTAESLFGQRLAVHSDFLGEQLQPLLDLTRRPPQVMRLQQRVNSPGWRRAMAAR

KRIDDLIDAQIADARTAPRPDDHMLTTLISGCSEEGTTLSDNEIRDSIVSLITAGYETTS

GALAWAIYALLTVPGTWESAASEVARVLGGRVPAADDLSALTYLNGVVHETLRLYSPGVI

SARRVLRDLWFDGHRIRAGRLLIFSAYVTHRLPEIWPEPTEFRPLRWDPNAADYRKPAPH

EFIPFSGGLHRCIGAVMATTEMTVILARLVARAMLQLPAQRTHRIRAANFAALRPWPGLT

VEIRKSAPAQ

>CYP139A1(2576158036)Mycobacterium tuberculosis TB_RSA83

MRYPLGEALLALYRWRGPLINAGVGGHGYTYLLGAEANRFVFANADAFSWSQTFESLVPV

DGPTALIVSDGADHRRRRSVVAPGLRHHHVQRYVATMVSNIDTVIDGWQPGQRLDIYQEL

RSAVRRSTAESLFGQRLAVHSDFLGEQLQPLLDLTRRPPQVMRLQQRVNSPGWRRAMAAR

KRIDDLIDAQIADARTAPRPDDHMLTTLISGCSEEGTTLSDNEIRDSIVSLITAGYETTS

GALAWAIYALLTVPGTWESAASEVARVLGGRVPAADDLSALTYLNGVVHETLRLYSPGVI

SARRVLRDLWFDGHRIRAGRLLIFSAYVTHRLPEIWPEPTEFRPLRWDPNAADYRKPAPH

EFIPFSGGLHRCIGAVMATTEMTVILARLVARAMLQLPAQRTHRIRAANFAALRPWPGLT

VEIRKSAPAQ

>CYP139A1(2577689111)Mycobacterium tuberculosis TB_RSA51

MRYPLGEALLALYRWRGPLINAGVGGHGYTYLLGAEANRFVFANADAFSWSQTFESLVPV

DGPTALIVSDGADHRRRRSVVAPGLRHHHVQRYVATMVSNIDTVIDGWQPGQRLDIYQEL

RSAVRRSTAESLFGQRLAVHSDFLGEQLQPLLDLTRRPPQVMRLQQRVNSPGWRRAMAAR

KRIDDLIDAQIADARTAPRPDDHMLTTLISGCSEEGTTLSDNEIRDSIVSLITAGYETTS

GALAWAIYALLTVPGTWESAASEVARVLGGRVPAADDLSALTYLNGVVHETLRLYSPGVI

SARRVLRDLWFDGHRIRAGRLLIFSAYVTHRLPEIWPEPTEFRPLRWDPNAADYRKPAPH

EFIPFSGGLHRCIGAVMATTEMTVILARLVARAMLQLPAQRTHRIRAANFAALRPWPGLT

VEIRKSAPAQ

>CYP139A1(2577751179)Mycobacterium tuberculosis TKK-01-0068

MRYPLGEALLALYRWRGPLINAGVGGHGYTYLLGAEANRFVFANADAFSWSQTFESLVPV

DGPTALIVSDGADHRRRRSVVAPGLRHHHVQRYVATMVSNIDTVIDGWQPGQRLDIYQEL

RSAVRRSTAESLFGQRLAVHSDFLGEQLQPLLDLTRRPPQVMRLQQRVNSPGWRRAMAAR

KRIDDLIDAQIADARTAPRPDDHMLTTLISGCSEEGTTLSDNEIRDSIVSLITAGYETTS

GALAWAIYALLTVPGTWESAASEVARVLGGRVPAADDLSALTYLNGVVHETLRLYSPGVI

SARRVLRDLWFDGHRIRAGRLLIFSAYVTHRLPEIWPEPTEFRPLRWDPNAADYRKPAPH

EFIPFSGGLHRCIGAVMATTEMTVILARLVARAMLQLPAQRTHRIRAANFAALRPWPGLT

VEIRKSAPAQ

>CYP139A1(2577845812)Mycobacterium tuberculosis XTB13-199

MRYPLGEALLALYRWRGPLINAGVGGHGYTYLLGAEANRFVFANADAFSWSQTFESLVPV

DGPTALIVSDGADHRRRRSVVAPGLRHHHVQRYVATMVSNIDTVIDGWQPGQRLDIYQEL

RSAVRRSTAESLFGQRLAVHSDFLGEQLQPLLDLTRRPPQVMRLQQRVNSPGWRRAMAAR

KRIDDLIDAQIADARTAPRPDDHMLTTLISGCSEEGTTLSDNEIRDSIVSLITAGYETTS

GALAWAIYALLTVPGTWESAASEVARVLGGRVPAADDLSALTYLNGVVHETLRLYSPGVI

SARRVLRDLWFDGHRIRAGRLLIFSAYVTHRLPEIWPEPTEFRPLRWDPNAADYRKPAPH

EFIPFSGGLHRCIGAVMATTEMTVILARLVARAMLQLPAQRTHRIRAANFAALRPWPGLT

VEIRKSAPAQ

>CYP139A1(2577900964)Mycobacterium tuberculosis TB_RSA138

MRYPLGEALLALYRWRGPLINAGVGGHGYTYLLGAEANRFVFANADAFSWSQTFESLVPV

DGPTALIVSDGADHRRRRSVVAPGLRHHHVQRYVATMVSNIDTVIDGWQPGQRLDIYQEL

RSAVRRSTAESLFGQRLAVHSDFLGEQLQPLLDLTRRPPQVMRLQQRVNSPGWRRAMAAR

KRIDDLIDAQIADARTAPRPDDHMLTTLISGCSEEGTTLSDNEIRDSIVSLITAGYETTS

GALAWAIYALLTVPGTWESAASEVARVLGGRVPAADDLSALTYLNGVVHETLRLYSPGVI

SARRVLRDLWFDGHRIRAGRLLIFSAYVTHRLPEIWPEPTEFRPLRWDPNAADYRKPAPH

EFIPFSGGLHRCIGAVMATTEMTVILARLVARAMLQLPAQRTHRIRAANFAALRPWPGLT

VEIRKSAPAQ

>CYP139A1(2577988240)Mycobacterium tuberculosis TB_RSA132

MRYPLGEALLALYRWRGPLINAGVGGHGYTYLLGAEANRFVFANADAFSWSQTFESLVPV

DGPTALIVSDGADHRRRRSVVAPGLRHHHVQRYVATMVSNIDTVIDGWQPGQRLDIYQEL

RSAVRRSTAESLFGQRLAVHSDFLGEQLQPLLDLTRRPPQVMRLQQRVNSPGWRRAMAAR

KRIDDLIDAQIADARTAPRPDDHMLTTLISGCSEEGTTLSDNEIRDSIVSLITAGYETTS

GALAWAIYALLTVPGTWESAASEVARVLGGRVPAADDLSALTYLNGVVHETLRLYSPGVI

SARRVLRDLWFDGHRIRAGRLLIFSAYVTHRLPEIWPEPTEFRPLRWDPNAADYRKPAPH

EFIPFSGGLHRCIGAVMATTEMTVILARLVARAMLQLPAQRTHRIRAANFAALRPWPGLT

VEIRKSAPAQ

>CYP139A1(2578237814)Mycobacterium tuberculosis XTB13-123

MRYPLGEALLALYRWRGPLINAGVGGHGYTYLLGAEANRFVFANADAFSWSQTFESLVPV

DGPTALIVSDGADHRRRRSVVAPGLRHHHVQRYVATMVSNIDTVIDGWQPGQRLDIYQEL

RSAVRRSTAESLFGQRLAVHSDFLGEQLQPLLDLTRRPPQVMRLQQRVNSPGWRRAMAAR

KRIDDLIDAQIADARTAPRPDDHMLTTLISGCSEEGTTLSDNEIRDSIVSLITAGYETTS

GALAWAIYALLTVPGTWESAASEVARVLGGRVPAADDLSALTYLNGVVHETLRLYSPGVI

SARRVLRDLWFDGHRIRAGRLLIFSAYVTHRLPEIWPEPTEFRPLRWDPNAADYRKPAPH

EFIPFSGGLHRCIGAVMATTEMTVILARLVARAMLQLPAQRTHRIRAANFAALRPWPGLT

VEIRKSAPAQ

>CYP139A1(2584703088)Mycobacterium tuberculosis TKK_04_0072

MRYPLGEALLALYRWRGPLINAGVGGHGYTYLLGAEANRFVFANADAFSWSQTFESLVPV

DGPTALIVSDGADHRRRRSVVAPGLRHHHVQRYVATMVSNIDTVIDGWQPGQRLDIYQEL

RSAVRRSTAESLFGQRLAVHSDFLGEQLQPLLDLTRRPPQVMRLQQRVNSPGWRRAMAAR

KRIDDLIDAQIADARTAPRPDDHMLTTLISGCSEEGTTLSDNEIRDSIVSLITAGYETTS

GALAWAIYALLTVPGTWESAASEVARVLGGRVPAADDLSALTYLNGVVHETLRLYSPGVI

SARRVLRDLWFDGHRIRAGRLLIFSAYVTHRLPEIWPEPTEFRPLRWDPNAADYRKPAPH

EFIPFSGGLHRCIGAVMATTEMTVILARLVARAMLQLPAQRTHRIRAANFAALRPWPGLT

VEIRKSAPAQ

>CYP139A1(2584776403)Mycobacterium tuberculosis TKK_04_0129

MRYPLGEALLALYRWRGPLINAGVGGHGYTYLLGAEANRFVFANADAFSWSQTFESLVPV

DGPTALIVSDGADHRRRRSVVAPGLRHHHVQRYVATMVSNIDTVIDGWQPGQRLDIYQEL

RSAVRRSTAESLFGQRLAVHSDFLGEQLQPLLDLTRRPPQVMRLQQRVNSPGWRRAMAAR

KRIDDLIDAQIADARTAPRPDDHMLTTLISGCSEEGTTLSDNEIRDSIVSLITAGYETTS

GALAWAIYALLTVPGTWESAASEVARVLGGRVPAADDLSALTYLNGVVHETLRLYSPGVI

SARRVLRDLWFDGHRIRAGRLLIFSAYVTHRLPEIWPEPTEFRPLRWDPNAADYRKPAPH

EFIPFSGGLHRCIGAVMATTEMTVILARLVARAMLQLPAQRTHRIRAANFAALRPWPGLT

VEIRKSAPAQ

>CYP139A1(2584785697)Mycobacterium tuberculosis TKK_05MA_0009

MRYPLGEALLALYRWRGPLINAGVGGHGYTYLLGAEANRFVFANADAFSWSQTFESLVPV

DGPTALIVSDGADHRRRRSVVAPGLRHHHVQRYVATMVSNIDTVIDGWQPGQRLDIYQEL

RSAVRRSTAESLFGQRLAVHSDFLGEQLQPLLDLTRRPPQVMRLQQRVNSPGWRRAMAAR

KRIDDLIDAQIADARTAPRPDDHMLTTLISGCSEEGTTLSDNEIRDSIVSLITAGYETTS

GALAWAIYALLTVPGTWESAASEVARVLGGRVPAADDLSALTYLNGVVHETLRLYSPGVI

SARRVLRDLWFDGHRIRAGRLLIFSAYVTHRLPEIWPEPTEFRPLRWDPNAADYRKPAPH

EFIPFSGGLHRCIGAVMATTEMTVILARLVARAMLQLPAQRTHRIRAANFAALRPWPGLT

VEIRKSAPAQ

>CYP139A1(2584898862)Mycobacterium tuberculosis TKK-01-0065

MRYPLGEALLALYRWRGPLINAGVGGHGYTYLLGAEANRFVFANADAFSWSQTFESLVPV

DGPTALIVSDGADHRRRRSVVAPGLRHHHVQRYVATMVSNIDTVIDGWQPGQRLDIYQEL

RSAVRRSTAESLFGQRLAVHSDFLGEQLQPLLDLTRRPPQVMRLQQRVNSPGWRRAMAAR

KRIDDLIDAQIADARTAPRPDDHMLTTLISGCSEEGTTLSDNEIRDSIVSLITAGYETTS

GALAWAIYALLTVPGTWESAASEVARVLGGRVPAADDLSALTYLNGVVHETLRLYSPGVI

SARRVLRDLWFDGHRIRAGRLLIFSAYVTHRLPEIWPEPTEFRPLRWDPNAADYRKPAPH

EFIPFSGGLHRCIGAVMATTEMTVILARLVARAMLQLPAQRTHRIRAANFAALRPWPGLT

VEIRKSAPAQ

>CYP139A1(2584906709)Mycobacterium tuberculosis TKK_04_0042

MRYPLGEALLALYRWRGPLINAGVGGHGYTYLLGAEANRFVFANADAFSWSQTFESLVPV

DGPTALIVSDGADHRRRRSVVAPGLRHHHVQRYVATMVSNIDTVIDGWQPGQRLDIYQEL

RSAVRRSTAESLFGQRLAVHSDFLGEQLQPLLDLTRRPPQVMRLQQRVNSPGWRRAMAAR

KRIDDLIDAQIADARTAPRPDDHMLTTLISGCSEEGTTLSDNEIRDSIVSLITAGYETTS

GALAWAIYALLTVPGTWESAASEVARVLGGRVPAADDLSALTYLNGVVHETLRLYSPGVI

SARRVLRDLWFDGHRIRAGRLLIFSAYVTHRLPEIWPEPTEFRPLRWDPNAADYRKPAPH

EFIPFSGGLHRCIGAVMATTEMTVILARLVARAMLQLPAQRTHRIRAANFAALRPWPGLT

VEIRKSAPAQ

>CYP139A1(2589082097)Mycobacterium tuberculosis TBR44

MRYPLGEALLALYRWRGPLINAGVGGHGYTYLLGAEANRFVFANADAFSWSQTFESLVPV

DGPTALIVSDGADHRRRRSVVAPGLRHHHVQRYVATMVSNIDTVIDGWQPGQRLDIYQEL

RSAVRRSTAESLFGQRLAVHSDFLGEQLQPLLDLTRRPPQVMRLQQRVNSPGWRRAMAAR

KRIDDLIDAQIADARTAPRPDDHMLTTLISGCSEEGTTLSDNEIRDSIVSLITAGYETTS

GALAWAIYALLTVPGTWESAASEVARVLGGRVPAADDLSALTYLNGVVHETLRLYSPGVI

SARRVLRDLWFDGHRIRAGRLLIFSAYVTHRLPEIWPEPTEFRPLRWDPNAADYRKPAPH

EFIPFSGGLHRCIGAVMATTEMTVILARLVARAMLQLPAQRTHRIRAANFAALRPWPGLT

VEIRKSAPAQ

>CYP139A1(2589142105)Mycobacterium tuberculosis OFXR-2

MRYPLGEALLALYRWRGPLINAGVGGHGYTYLLGAEANRFVFANADAFSWSQTFESLVPV

DGPTALIVSDGADHRRRRSVVAPGLRHHHVQRYVATMVSNIDTVIDGWQPGQRLDIYQEL

RSAVRRSTAESLFGQRLAVHSDFLGEQLQPLLDLTRRPPQVMRLQQRVNSPGWRRAMAAR

KRIDDLIDAQIADARTAPRPDDHMLTTLISGCSEEGTTLSDNEIRDSIVSLITAGYETTS

GALAWAIYALLTVPGTWESAASEVARVLGGRVPAADDLSALTYLNGVVHETLRLYSPGVI

SARRVLRDLWFDGHRIRAGRLLIFSAYVTHRLPEIWPEPTEFRPLRWDPNAADYRKPAPH

EFIPFSGGLHRCIGAVMATTEMTVILARLVARAMLQLPAQRTHRIRAANFAALRPWPGLT

VEIRKSAPAQ

>CYP139A1(2589707360)Mycobacterium tuberculosis TKK-01-0082

MRYPLGEALLALYRWRGPLINAGVGGHGYTYLLGAEANRFVFANADAFSWSQTFESLVPV

DGPTALIVSDGADHRRRRSVVAPGLRHHHVQRYVATMVSNIDTVIDGWQPGQRLDIYQEL

RSAVRRSTAESLFGQRLAVHSDFLGEQLQPLLDLTRRPPQVMRLQQRVNSPGWRRAMAAR

KRIDDLIDAQIADARTAPRPDDHMLTTLISGCSEEGTTLSDNEIRDSIVSLITAGYETTS

GALAWAIYALLTVPGTWESAASEVARVLGGRVPAADDLSALTYLNGVVHETLRLYSPGVI

SARRVLRDLWFDGHRIRAGRLLIFSAYVTHRLPEIWPEPTEFRPLRWDPNAADYRKPAPH

EFIPFSGGLHRCIGAVMATTEMTVILARLVARAMLQLPAQRTHRIRAANFAALRPWPGLT

VEIRKSAPAQ

>CYP139A1(2590142346)Mycobacterium tuberculosis MAL020192

MRYPLGEALLALYRWRGPLINAGVGGHGYTYLLGAEANRFVFANADAFSWSQTFESLVPV

DGPTALIVSDGADHRRRRSVVAPGLRHHHVQRYVATMVSNIDTVIDGWQPGQRLDIYQEL

RSAVRRSTAESLFGQRLAVHSDFLGEQLQPLLDLTRRPPQVMRLQQRVNSPGWRRAMAAR

KRIDDLIDAQIADARTAPRPDDHMLTTLISGCSEEGTTLSDNEIRDSIVSLITAGYETTS

GALAWAIYALLTVPGTWESAASEVARVLGGRVPAADDLSALTYLNGVVHETLRLYSPGVI

SARRVLRDLWFDGHRIRAGRLLIFSAYVTHRLPEIWPEPTEFRPLRWDPNAADYRKPAPH

EFIPFSGGLHRCIGAVMATTEMTVILARLVARAMLQLPAQRTHRIRAANFAALRPWPGLT

VEIRKSAPAQ

>CYP139A1(2592254166)Mycobacterium tuberculosis TKK_04_0008

MRYPLGEALLALYRWRGPLINAGVGGHGYTYLLGAEANRFVFANADAFSWSQTFESLVPV

DGPTALIVSDGADHRRRRSVVAPGLRHHHVQRYVATMVSNIDTVIDGWQPGQRLDIYQEL

RSAVRRSTAESLFGQRLAVHSDFLGEQLQPLLDLTRRPPQVMRLQQRVNSPGWRRAMAAR

KRIDDLIDAQIADARTAPRPDDHMLTTLISGCSEEGTTLSDNEIRDSIVSLITAGYETTS

GALAWAIYALLTVPGTWESAASEVARVLGGRVPAADDLSALTYLNGVVHETLRLYSPGVI

SARRVLRDLWFDGHRIRAGRLLIFSAYVTHRLPEIWPEPTEFRPLRWDPNAADYRKPAPH

EFIPFSGGLHRCIGAVMATTEMTVILARLVARAMLQLPAQRTHRIRAANFAALRPWPGLT

VEIRKSAPAQ

>CYP139A1(2592348241)Mycobacterium tuberculosis TKK_02_0068

MRYPLGEALLALYRWRGPLINAGVGGHGYTYLLGAEANRFVFANADAFSWSQTFESLVPV

DGPTALIVSDGADHRRRRSVVAPGLRHHHVQRYVATMVSNIDTVIDGWQPGQRLDIYQEL

RSAVRRSTAESLFGQRLAVHSDFLGEQLQPLLDLTRRPPQVMRLQQRVNSPGWRRAMAAR

KRIDDLIDAQIADARTAPRPDDHMLTTLISGCSEEGTTLSDNEIRDSIVSLITAGYETTS

GALAWAIYALLTVPGTWESAASEVARVLGGRVPAADDLSALTYLNGVVHETLRLYSPGVI

SARRVLRDLWFDGHRIRAGRLLIFSAYVTHRLPEIWPEPTEFRPLRWDPNAADYRKPAPH

EFIPFSGGLHRCIGAVMATTEMTVILARLVARAMLQLPAQRTHRIRAANFAALRPWPGLT

VEIRKSAPAQ

>CYP139A1(2592364277)Mycobacterium tuberculosis TKK_02_0061

MRYPLGEALLALYRWRGPLINAGVGGHGYTYLLGAEANRFVFANADAFSWSQTFESLVPV

DGPTALIVSDGADHRRRRSVVAPGLRHHHVQRYVATMVSNIDTVIDGWQPGQRLDIYQEL

RSAVRRSTAESLFGQRLAVHSDFLGEQLQPLLDLTRRPPQVMRLQQRVNSPGWRRAMAAR

KRIDDLIDAQIADARTAPRPDDHMLTTLISGCSEEGTTLSDNEIRDSIVSLITAGYETTS

GALAWAIYALLTVPGTWESAASEVARVLGGRVPAADDLSALTYLNGVVHETLRLYSPGVI

SARRVLRDLWFDGHRIRAGRLLIFSAYVTHRLPEIWPEPTEFRPLRWDPNAADYRKPAPH

EFIPFSGGLHRCIGAVMATTEMTVILARLVARAMLQLPAQRTHRIRAANFAALRPWPGLT

VEIRKSAPAQ

>CYP139A1(2592537861)Mycobacterium tuberculosis TKK_04_0048

MRYPLGEALLALYRWRGPLINAGVGGHGYTYLLGAEANRFVFANADAFSWSQTFESLVPV

DGPTALIVSDGADHRRRRSVVAPGLRHHHVQRYVATMVSNIDTVIDGWQPGQRLDIYQEL

RSAVRRSTAESLFGQRLAVHSDFLGEQLQPLLDLTRRPPQVMRLQQRVNSPGWRRAMAAR

KRIDDLIDAQIADARTAPRPDDHMLTTLISGCSEEGTTLSDNEIRDSIVSLITAGYETTS

GALAWAIYALLTVPGTWESAASEVARVLGGRVPAADDLSALTYLNGVVHETLRLYSPGVI

SARRVLRDLWFDGHRIRAGRLLIFSAYVTHRLPEIWPEPTEFRPLRWDPNAADYRKPAPH

EFIPFSGGLHRCIGAVMATTEMTVILARLVARAMLQLPAQRTHRIRAANFAALRPWPGLT

VEIRKSAPAQ

>CYP139A1(2546454904)Mycobacterium tuberculosis GuangZ0019

MRYPLGEALLALYRWRGPLINAGVGGHGYTYLLGAEANRFVFANADAFSWSQTFESLVPV

DGPTALIVSDGADHRRRRSVVAPGLRHHHVQRYVATMVSNIDTVIDGWQPGQRLDIYQEL

RSAVRRSTAESLFGQRLAVHSDFLGEQLQPLLDLTRRPPQVMRLQQRVNSPGWRRAMAAR

KRIDDLIDAQIADARTAPRPDDHMLTTLISGCSEEGTTLSDNEIRDSIVSLITAGYETTS

GALAWAIYALLTVPGTWESAASEVARVLGGRVPAADDLSALTYLNGVVHETLRLYSPGVI

SARRVLRDLWFDGHRIRAGRLLIFSAYVTHRLPEIWPEPTEFRPLRWDPNAADYRKPAPH

EFIPFSGGLHRCIGAVMATTEMTVILARLVARAMLQLPAQRTHRIRAANFAALRPWPGLT

VEIRKSAPAQ

>CYP139A1(2577218717)Mycobacterium tuberculosis M2248

MRYPLGEALLALYRWRGPLINAGVGGHGYTYLLGAEANRFVFANADAFSWSQTFESLVPV

DGPTALIVSDGADHRRRRSVVAPGLRHHHVQRYVATMVSNIDTVIDGWQPGQRLDIYQEL

RSAVRRSTAESLFGQRLAVHSDFLGEQLQPLLDLTRRPPQVMRLQQRVNSPGWRRAMAAR

KRIDDLIDAQIADARTAPRPDDHMLTTLISGCSEEGTTLSDNEIRDSIVSLITAGYETTS

GALAWAIYALLTVPGTWESAASEVARVLGGRVPAADDLSALTYLNGVVHETLRLYSPGVI

SARRVLRDLWFDGHRIRAGRLLIFSAYVTHRLPEIWPEPTEFRPLRWDPNAADYRKPAPH

EFIPFSGGLHRCIGAVMATTEMTVILARLVARAMLQLPAQRTHRIRAANFAALRPWPGLT

VEIRKSAPAQ

>CYP139A1(2577720788)Mycobacterium tuberculosis M2479

MRYPLGEALLALYRWRGPLINAGVGGHGYTYLLGAEANRFVFANADAFSWSQTFESLVPV

DGPTALIVSDGADHRRRRSVVAPGLRHHHVQRYVATMVSNIDTVIDGWQPGQRLDIYQEL

RSAVRRSTAESLFGQRLAVHSDFLGEQLQPLLDLTRRPPQVMRLQQRVNSPGWRRAMAAR

KRIDDLIDAQIADARTAPRPDDHMLTTLISGCSEEGTTLSDNEIRDSIVSLITAGYETTS

GALAWAIYALLTVPGTWESAASEVARVLGGRVPAADDLSALTYLNGVVHETLRLYSPGVI

SARRVLRDLWFDGHRIRAGRLLIFSAYVTHRLPEIWPEPTEFRPLRWDPNAADYRKPAPH

EFIPFSGGLHRCIGAVMATTEMTVILARLVARAMLQLPAQRTHRIRAANFAALRPWPGLT

VEIRKSAPAQ

>CYP139A1(2581366557)*Mycobacterium africanum* MAL020173

MRYPLGEALLALYRWRGPLINAGVGGHGYTYLLGAEANRFVFANADAFSWSQTFESLVPV

DGPTALIVSDGADHRRRRSVVAPGLRHHHVQRYVATMVSNIDTVIDGWQPGQRLDIYQEL

RSAVRRSTAESLFGQRLAVHSDFLGEQLQPLLDLTRRPPQVMRLQQRVNSPGWRRAMAAR

KRIDDLIDAQIADARTAPRPDDHMLTTLISGCSEEGTTLSDNEIRDSIVSLITAGYETTS

GALAWAIYALLTVPGTWESAASEVARVLGGRVPAADDLSALTYLNGVVHETLRLYSPGVI

SARRVLRDLWFDGHRIRAGRLLIFSAYVTHRLPEIWPEPTEFRPLRWDPNAADYRKPAPH

EFIPFSGGLHRCIGAVMATTEMTVILARLVARAMLQLPAQRTHRIRAANFAALRPWPGLT

VEIRKSAPAQ

>CYP139A1(2584638962)Mycobacterium tuberculosis 51628

MRYPLGEALLALYRWRGPLINAGVGGHGYTYLLGAEANRFVFANADAFSWSQTFESLVPV

DGPTALIVSDGADHRRRRSVVAPGLRHHHVQRYVATMVSNIDTVIDGWQPGQRLDIYQEL

RSAVRRSTAESLFGQRLAVHSDFLGEQLQPLLDLTRRPPQVMRLQQRVNSPGWRRAMAAR

KRIDDLIDAQIADARTAPRPDDHMLTTLISGCSEEGTTLSDNEIRDSIVSLITAGYETTS

GALAWAIYALLTVPGTWESAASEVARVLGGRVPAADDLSALTYLNGVVHETLRLYSPGVI

SARRVLRDLWFDGHRIRAGRLLIFSAYVTHRLPEIWPEPTEFRPLRWDPNAADYRKPAPH

EFIPFSGGLHRCIGAVMATTEMTVILARLVARAMLQLPAQRTHRIRAANFAALRPWPGLT

VEIRKSAPAQ

>CYP139A1(2590154524)Mycobacterium tuberculosis MAL020187

MRYPLGEALLALYRWRGPLINAGVGGHGYTYLLGAEANRFVFANADAFSWSQTFESLVPV

DGPTALIVSDGADHRRRRSVVAPGLRHHHVQRYVATMVSNIDTVIDGWQPGQRLDIYQEL

RSAVRRSTAESLFGQRLAVHSDFLGEQLQPLLDLTRRPPQVMRLQQRVNSPGWRRAMAAR

KRIDDLIDAQIADARTAPRPDDHMLTTLISGCSEEGTTLSDNEIRDSIVSLITAGYETTS

GALAWAIYALLTVPGTWESAASEVARVLGGRVPAADDLSALTYLNGVVHETLRLYSPGVI

SARRVLRDLWFDGHRIRAGRLLIFSAYVTHRLPEIWPEPTEFRPLRWDPNAADYRKPAPH

EFIPFSGGLHRCIGAVMATTEMTVILARLVARAMLQLPAQRTHRIRAANFAALRPWPGLT

VEIRKSAPAQ

>CYP139A1(2590260165)Mycobacterium tuberculosis KT-0022

MRYPLGEALLALYRWRGPLINAGVGGHGYTYLLGAEANRFVFANADAFSWSQTFESLVPV

DGPTALIVSDGADHRRRRSVVAPGLRHHHVQRYVATMVSNIDTVIDGWQPGQRLDIYQEL

RSAVRRSTAESLFGQRLAVHSDFLGEQLQPLLDLTRRPPQVMRLQQRVNSPGWRRAMAAR

KRIDDLIDAQIADARTAPRPDDHMLTTLISGCSEEGTTLSDNEIRDSIVSLITAGYETTS

GALAWAIYALLTVPGTWESAASEVARVLGGRVPAADDLSALTYLNGVVHETLRLYSPGVI

SARRVLRDLWFDGHRIRAGRLLIFSAYVTHRLPEIWPEPTEFRPLRWDPNAADYRKPAPH

EFIPFSGGLHRCIGAVMATTEMTVILARLVARAMLQLPAQRTHRIRAANFAALRPWPGLT

VEIRKSAPAQ

>CYP139A1(2590539845)Mycobacterium tuberculosis KT-0080

MRYPLGEALLALYRWRGPLINAGVGGHGYTYLLGAEANRFVFANADAFSWSQTFESLVPV

DGPTALIVSDGADHRRRRSVVAPGLRHHHVQRYVATMVSNIDTVIDGWQPGQRLDIYQEL

RSAVRRSTAESLFGQRLAVHSDFLGEQLQPLLDLTRRPPQVMRLQQRVNSPGWRRAMAAR

KRIDDLIDAQIADARTAPRPDDHMLTTLISGCSEEGTTLSDNEIRDSIVSLITAGYETTS

GALAWAIYALLTVPGTWESAASEVARVLGGRVPAADDLSALTYLNGVVHETLRLYSPGVI

SARRVLRDLWFDGHRIRAGRLLIFSAYVTHRLPEIWPEPTEFRPLRWDPNAADYRKPAPH

EFIPFSGGLHRCIGAVMATTEMTVILARLVARAMLQLPAQRTHRIRAANFAALRPWPGLT

VEIRKSAPAQ

>CYP139A1(641783198)Mycobacterium tuberculosis Haarlem

MRYPLGEALLALYRWRGPLINAGVGGHGYTYLLGAEANRFVFANADAFSWSQTFESLVPV

DGPTALIVSDGADHRRRRSVVAPGLRHHHVQRYVATMVSNIDTVIDGWQPGQRLDIYQEL

RSAVRRSTAESLFGQRLAVHSDFLGEQLQPLLDLTRRPPQVMRLQQRVNSPGWRRAMAAR

KRIDDLIDAQIADARTAPRPDDHMLTTLISGCSEEGTTLSDNEIRDSIVSLITAGYETTS

GALAWAIYALLTVPGTWESAASEVARVLGGRVPAADDLSALTYLNGVVHETLRLYSPGVI

SARRVLRDLWFDGHRIRAGRLLIFSAYVTHRLPEIWPEPTEFRPLRWDPNAADYRKPAPH

EFIPFSGGLHRCIGAVMATTEMTVILARLVARAMLQLPAQRTHRIRAANFAALRPWPGLT

VEIRKSAPAQ

>CYP139A1(643031783)Mycobacterium tuberculosis EAS054

MRYPLGEALLALYRWRGPLINAGVGGHGYTYLLGAEANRFVFANADAFSWSQTFESLVPV

DGPTALIVSDGADHRRRRSVVAPGLRHHHVQRYVATMVSNIDTVIDGWQPGQRLDIYQEL

RSAVRRSTAESLFGQRLAVHSDFLGEQLQPLLDLTRRPPQVMRLQQRVNSPGWRRAMAAR

KRIDDLIDAQIADARTAPRPDDHMLTTLISGCSEEGTTLSDNEIRDSIVSLITAGYETTS

GALAWAIYALLTVPGTWESAASEVARVLGGRVPAADDLSALTYLNGVVHETLRLYSPGVI

SARRVLRDLWFDGHRIRAGRLLIFSAYVTHRLPEIWPEPTEFRPLRWDPNAADYRKPAPH

EFIPFSGGLHRCIGAVMATTEMTVILARLVARAMLQLPAQRTHRIRAANFAALRPWPGLT

VEIRKSAPAQ

>CYP139A1(651039004)*Mycobacterium canettii* CIPT 140010059

MRYPLGEALLALYRWRGPLINAGVGGHGYTYLLGAEANRFVFANADAFSWSQTFESLVPV

DGPTALIVSDGADHRRRRSVVAPGLRHHHVQRYVATMVSNIDTVIDGWQPGQRLDIYQEL

RSAVRRSTAESLFGQRLAVHSDFLGEQLQPLLDLTRRPPQVMRLQQRVNSPGWRRAMAAR

KRIDDLIDAQIADARTAPRPDDHMLTTLISGCSEEGTTLSDNEIRDSIVSLITAGYETTS

GALAWAIYALLTVPGTWESAASEVARVLGGRVPAADDLSALTYLNGVVHETLRLYSPGVI

SARRVLRDLWFDGHRIRAGRLLIFSAYVTHRLPEIWPEPTEFRPLRWDPNAADYRKPAPH

EFIPFSGGLHRCIGAVMATTEMTVILARLVARAMLQLPAQRTHRIRAANFAALRPWPGLT

VEIRKSAPAQ

>CYP139A1(2574843285)Mycobacterium tuberculosis TKK_04_0157

MRYPLGEALLALYRWRGPLINAGVGGHGYTYLLGAEANRFVFANADAFSWSQTFESLVPV

DGPTALIVSDGADHRRRRSVVAPGLRHHHVQRYVATMVSNIDTVIDGWQPGQRLDIYQEL

RSAVRRSTAESLFGQRLAVHSDFLGEQLQPLLDLTRRPPQVMRLQQRVNSPGWRRAMAAR

KRIDDLIDAQIADARTAPRPDDHMLTTLISGCSEEGTTLSDNEIRDSIVSLITAGYETTS

GALAWAIYALLTVPGTWESAASEVARVLGGRVPAADDLSALTYLNGVVHETLRLYSPGVI

SARRVLRDLWFDGHRIRAGRLLIFSAYVTHRLPEIWPEPTEFRPLRWDPNAADYRKPAPH

EFIPFSGGLHRCIGAVMATTEMTVILARLVARAMLQLPAQRTHRIRAANFAALRPWPGLT

VEIRKSAPAQ

>CYP139A1(2575542837)Mycobacterium tuberculosis TKK_04_0021

MRYPLGEALLALYRWRGPLINAGVGGHGYTYLLGAEANRFVFANADAFSWSQTFESLVPV

DGPTALIVSDGADHRRRRSVVAPGLRHHHVQRYVATMVSNIDTVIDGWQPGQRLDIYQEL

RSAVRRSTAESLFGQRLAVHSDFLGEQLQPLLDLTRRPPQVMRLQQRVNSPGWRRAMAAR

KRIDDLIDAQIADARTAPRPDDHMLTTLISGCSEEGTTLSDNEIRDSIVSLITAGYETTS

GALAWAIYALLTVPGTWESAASEVARVLGGRVPAADDLSALTYLNGVVHETLRLYSPGVI

SARRVLRDLWFDGHRIRAGRLLIFSAYVTHRLPEIWPEPTEFRPLRWDPNAADYRKPAPH

EFIPFSGGLHRCIGAVMATTEMTVILARLVARAMLQLPAQRTHRIRAANFAALRPWPGLT

VEIRKSAPAQ

>CYP139A1(2575709449)Mycobacterium tuberculosis NRITLD44

MRYPLGEALLALYRWRGPLINAGVGGHGYTYLLGAEANRFVFANADAFSWSQTFESLVPV

DGPTALIVSDGADHRRRRSVVAPGLRHHHVQRYVATMVSNIDTVIDGWQPGQRLDIYQEL

RSAVRRSTAESLFGQRLAVHSDFLGEQLQPLLDLTRRPPQVMRLQQRVNSPGWRRAMAAR

KRIDDLIDAQIADARTAPRPDDHMLTTLISGCSEEGTTLSDNEIRDSIVSLITAGYETTS

GALAWAIYALLTVPGTWESAASEVARVLGGRVPAADDLSALTYLNGVVHETLRLYSPGVI

SARRVLRDLWFDGHRIRAGRLLIFSAYVTHRLPEIWPEPTEFRPLRWDPNAADYRKPAPH

EFIPFSGGLHRCIGAVMATTEMTVILARLVARAMLQLPAQRTHRIRAANFAALRPWPGLT

VEIRKSAPAQ

>CYP139A1(2576051820)Mycobacterium tuberculosis TB_RSA67

MRYPLGEALLALYRWRGPLINAGVGGHGYTYLLGAEANRFVFANADAFSWSQTFESLVPV

DGPTALIVSDGADHRRRRSVVAPGLRHHHVQRYVATMVSNIDTVIDGWQPGQRLDIYQEL

RSAVRRSTAESLFGQRLAVHSDFLGEQLQPLLDLTRRPPQVMRLQQRVNSPGWRRAMAAR

KRIDDLIDAQIADARTAPRPDDHMLTTLISGCSEEGTTLSDNEIRDSIVSLITAGYETTS

GALAWAIYALLTVPGTWESAASEVARVLGGRVPAADDLSALTYLNGVVHETLRLYSPGVI

SARRVLRDLWFDGHRIRAGRLLIFSAYVTHRLPEIWPEPTEFRPLRWDPNAADYRKPAPH

EFIPFSGGLHRCIGAVMATTEMTVILARLVARAMLQLPAQRTHRIRAANFAALRPWPGLT

VEIRKSAPAQ

>CYP139A1(2578094092)Mycobacterium tuberculosis NRITLD15

MRYPLGEALLALYRWRGPLINAGVGGHGYTYLLGAEANRFVFANADAFSWSQTFESLVPV

DGPTALIVSDGADHRRRRSVVAPGLRHHHVQRYVATMVSNIDTVIDGWQPGQRLDIYQEL

RSAVRRSTAESLFGQRLAVHSDFLGEQLQPLLDLTRRPPQVMRLQQRVNSPGWRRAMAAR

KRIDDLIDAQIADARTAPRPDDHMLTTLISGCSEEGTTLSDNEIRDSIVSLITAGYETTS

GALAWAIYALLTVPGTWESAASEVARVLGGRVPAADDLSALTYLNGVVHETLRLYSPGVI

SARRVLRDLWFDGHRIRAGRLLIFSAYVTHRLPEIWPEPTEFRPLRWDPNAADYRKPAPH

EFIPFSGGLHRCIGAVMATTEMTVILARLVARAMLQLPAQRTHRIRAANFAALRPWPGLT

VEIRKSAPAQ

>CYP139A1(2584617586)Mycobacterium tuberculosis TRUG0116

MRYPLGEALLALYRWRGPLINAGVGGHGYTYLLGAEANRFVFANADAFSWSQTFESLVPV

DGPTALIVSDGADHRRRRSVVAPGLRHHHVQRYVATMVSNIDTVIDGWQPGQRLDIYQEL

RSAVRRSTAESLFGQRLAVHSDFLGEQLQPLLDLTRRPPQVMRLQQRVNSPGWRRAMAAR

KRIDDLIDAQIADARTAPRPDDHMLTTLISGCSEEGTTLSDNEIRDSIVSLITAGYETTS

GALAWAIYALLTVPGTWESAASEVARVLGGRVPAADDLSALTYLNGVVHETLRLYSPGVI

SARRVLRDLWFDGHRIRAGRLLIFSAYVTHRLPEIWPEPTEFRPLRWDPNAADYRKPAPH

EFIPFSGGLHRCIGAVMATTEMTVILARLVARAMLQLPAQRTHRIRAANFAALRPWPGLT

VEIRKSAPAQ

>CYP139A1(2584694835)Mycobacterium tuberculosis TKK_04_0066

MRYPLGEALLALYRWRGPLINAGVGGHGYTYLLGAEANRFVFANADAFSWSQTFESLVPV

DGPTALIVSDGADHRRRRSVVAPGLRHHHVQRYVATMVSNIDTVIDGWQPGQRLDIYQEL

RSAVRRSTAESLFGQRLAVHSDFLGEQLQPLLDLTRRPPQVMRLQQRVNSPGWRRAMAAR

KRIDDLIDAQIADARTAPRPDDHMLTTLISGCSEEGTTLSDNEIRDSIVSLITAGYETTS

GALAWAIYALLTVPGTWESAASEVARVLGGRVPAADDLSALTYLNGVVHETLRLYSPGVI

SARRVLRDLWFDGHRIRAGRLLIFSAYVTHRLPEIWPEPTEFRPLRWDPNAADYRKPAPH

EFIPFSGGLHRCIGAVMATTEMTVILARLVARAMLQLPAQRTHRIRAANFAALRPWPGLT

VEIRKSAPAQ

>CYP139A1(2584822720)Mycobacterium tuberculosis XTB13-238

MRYPLGEALLALYRWRGPLINAGVGGHGYTYLLGAEANRFVFANADAFSWSQTFESLVPV

DGPTALIVSDGADHRRRRSVVAPGLRHHHVQRYVATMVSNIDTVIDGWQPGQRLDIYQEL

RSAVRRSTAESLFGQRLAVHSDFLGEQLQPLLDLTRRPPQVMRLQQRVNSPGWRRAMAAR

KRIDDLIDAQIADARTAPRPDDHMLTTLISGCSEEGTTLSDNEIRDSIVSLITAGYETTS

GALAWAIYALLTVPGTWESAASEVARVLGGRVPAADDLSALTYLNGVVHETLRLYSPGVI

SARRVLRDLWFDGHRIRAGRLLIFSAYVTHRLPEIWPEPTEFRPLRWDPNAADYRKPAPH

EFIPFSGGLHRCIGAVMATTEMTVILARLVARAMLQLPAQRTHRIRAANFAALRPWPGLT

VEIRKSAPAQ

>CYP139A1(2584931720)Mycobacterium tuberculosis MD16577

MRYPLGEALLALYRWRGPLINAGVGGHGYTYLLGAEANRFVFANADAFSWSQTFESLVPV

DGPTALIVSDGADHRRRRSVVAPGLRHHHVQRYVATMVSNIDTVIDGWQPGQRLDIYQEL

RSAVRRSTAESLFGQRLAVHSDFLGEQLQPLLDLTRRPPQVMRLQQRVNSPGWRRAMAAR

KRIDDLIDAQIADARTAPRPDDHMLTTLISGCSEEGTTLSDNEIRDSIVSLITAGYETTS

GALAWAIYALLTVPGTWESAASEVARVLGGRVPAADDLSALTYLNGVVHETLRLYSPGVI

SARRVLRDLWFDGHRIRAGRLLIFSAYVTHRLPEIWPEPTEFRPLRWDPNAADYRKPAPH

EFIPFSGGLHRCIGAVMATTEMTVILARLVARAMLQLPAQRTHRIRAANFAALRPWPGLT

VEIRKSAPAQ

>CYP139A1(2589165303)Mycobacterium tuberculosis OFXR-16

MRYPLGEALLALYRWRGPLINAGVGGHGYTYLLGAEANRFVFANADAFSWSQTFESLVPV

DGPTALIVSDGADHRRRRSVVAPGLRHHHVQRYVATMVSNIDTVIDGWQPGQRLDIYQEL

RSAVRRSTAESLFGQRLAVHSDFLGEQLQPLLDLTRRPPQVMRLQQRVNSPGWRRAMAAR

KRIDDLIDAQIADARTAPRPDDHMLTTLISGCSEEGTTLSDNEIRDSIVSLITAGYETTS

GALAWAIYALLTVPGTWESAASEVARVLGGRVPAADDLSALTYLNGVVHETLRLYSPGVI

SARRVLRDLWFDGHRIRAGRLLIFSAYVTHRLPEIWPEPTEFRPLRWDPNAADYRKPAPH

EFIPFSGGLHRCIGAVMATTEMTVILARLVARAMLQLPAQRTHRIRAANFAALRPWPGLT

VEIRKSAPAQ

>CYP139A1(2589608377)Mycobacterium tuberculosis TKK-01-0049

MRYPLGEALLALYRWRGPLINAGVGGHGYTYLLGAEANRFVFANADAFSWSQTFESLVPV

DGPTALIVSDGADHRRRRSVVAPGLRHHHVQRYVATMVSNIDTVIDGWQPGQRLDIYQEL

RSAVRRSTAESLFGQRLAVHSDFLGEQLQPLLDLTRRPPQVMRLQQRVNSPGWRRAMAAR

KRIDDLIDAQIADARTAPRPDDHMLTTLISGCSEEGTTLSDNEIRDSIVSLITAGYETTS

GALAWAIYALLTVPGTWESAASEVARVLGGRVPAADDLSALTYLNGVVHETLRLYSPGVI

SARRVLRDLWFDGHRIRAGRLLIFSAYVTHRLPEIWPEPTEFRPLRWDPNAADYRKPAPH

EFIPFSGGLHRCIGAVMATTEMTVILARLVARAMLQLPAQRTHRIRAANFAALRPWPGLT

VEIRKSAPAQ

>CYP139A1(2589638476)Mycobacterium tuberculosis TKK-01-0060

MRYPLGEALLALYRWRGPLINAGVGGHGYTYLLGAEANRFVFANADAFSWSQTFESLVPV

DGPTALIVSDGADHRRRRSVVAPGLRHHHVQRYVATMVSNIDTVIDGWQPGQRLDIYQEL

RSAVRRSTAESLFGQRLAVHSDFLGEQLQPLLDLTRRPPQVMRLQQRVNSPGWRRAMAAR

KRIDDLIDAQIADARTAPRPDDHMLTTLISGCSEEGTTLSDNEIRDSIVSLITAGYETTS

GALAWAIYALLTVPGTWESAASEVARVLGGRVPAADDLSALTYLNGVVHETLRLYSPGVI

SARRVLRDLWFDGHRIRAGRLLIFSAYVTHRLPEIWPEPTEFRPLRWDPNAADYRKPAPH

EFIPFSGGLHRCIGAVMATTEMTVILARLVARAMLQLPAQRTHRIRAANFAALRPWPGLT

VEIRKSAPAQ

>CYP139A1(2589687166)Mycobacterium tuberculosis TKK-01-0066

MRYPLGEALLALYRWRGPLINAGVGGHGYTYLLGAEANRFVFANADAFSWSQTFESLVPV

DGPTALIVSDGADHRRRRSVVAPGLRHHHVQRYVATMVSNIDTVIDGWQPGQRLDIYQEL

RSAVRRSTAESLFGQRLAVHSDFLGEQLQPLLDLTRRPPQVMRLQQRVNSPGWRRAMAAR

KRIDDLIDAQIADARTAPRPDDHMLTTLISGCSEEGTTLSDNEIRDSIVSLITAGYETTS

GALAWAIYALLTVPGTWESAASEVARVLGGRVPAADDLSALTYLNGVVHETLRLYSPGVI

SARRVLRDLWFDGHRIRAGRLLIFSAYVTHRLPEIWPEPTEFRPLRWDPNAADYRKPAPH

EFIPFSGGLHRCIGAVMATTEMTVILARLVARAMLQLPAQRTHRIRAANFAALRPWPGLT

VEIRKSAPAQ

>CYP139A1(2590174897)Mycobacterium tuberculosis MAL020201

MRYPLGEALLALYRWRGPLINAGVGGHGYTYLLGAEANRFVFANADAFSWSQTFESLVPV

DGPTALIVSDGADHRRRRSVVAPGLRHHHVQRYVATMVSNIDTVIDGWQPGQRLDIYQEL

RSAVRRSTAESLFGQRLAVHSDFLGEQLQPLLDLTRRPPQVMRLQQRVNSPGWRRAMAAR

KRIDDLIDAQIADARTAPRPDDHMLTTLISGCSEEGTTLSDNEIRDSIVSLITAGYETTS

GALAWAIYALLTVPGTWESAASEVARVLGGRVPAADDLSALTYLNGVVHETLRLYSPGVI

SARRVLRDLWFDGHRIRAGRLLIFSAYVTHRLPEIWPEPTEFRPLRWDPNAADYRKPAPH

EFIPFSGGLHRCIGAVMATTEMTVILARLVARAMLQLPAQRTHRIRAANFAALRPWPGLT

VEIRKSAPAQ

>CYP139A1(2590370532)Mycobacterium tuberculosis OFXR-22

MRYPLGEALLALYRWRGPLINAGVGGHGYTYLLGAEANRFVFANADAFSWSQTFESLVPV

DGPTALIVSDGADHRRRRSVVAPGLRHHHVQRYVATMVSNIDTVIDGWQPGQRLDIYQEL

RSAVRRSTAESLFGQRLAVHSDFLGEQLQPLLDLTRRPPQVMRLQQRVNSPGWRRAMAAR

KRIDDLIDAQIADARTAPRPDDHMLTTLISGCSEEGTTLSDNEIRDSIVSLITAGYETTS

GALAWAIYALLTVPGTWESAASEVARVLGGRVPAADDLSALTYLNGVVHETLRLYSPGVI

SARRVLRDLWFDGHRIRAGRLLIFSAYVTHRLPEIWPEPTEFRPLRWDPNAADYRKPAPH

EFIPFSGGLHRCIGAVMATTEMTVILARLVARAMLQLPAQRTHRIRAANFAALRPWPGLT

VEIRKSAPAQ

>CYP139A1(2592328029)Mycobacterium tuberculosis TKK_03_0018

MRYPLGEALLALYRWRGPLINAGVGGHGYTYLLGAEANRFVFANADAFSWSQTFESLVPV

DGPTALIVSDGADHRRRRSVVAPGLRHHHVQRYVATMVSNIDTVIDGWQPGQRLDIYQEL

RSAVRRSTAESLFGQRLAVHSDFLGEQLQPLLDLTRRPPQVMRLQQRVNSPGWRRAMAAR

KRIDDLIDAQIADARTAPRPDDHMLTTLISGCSEEGTTLSDNEIRDSIVSLITAGYETTS

GALAWAIYALLTVPGTWESAASEVARVLGGRVPAADDLSALTYLNGVVHETLRLYSPGVI

SARRVLRDLWFDGHRIRAGRLLIFSAYVTHRLPEIWPEPTEFRPLRWDPNAADYRKPAPH

EFIPFSGGLHRCIGAVMATTEMTVILARLVARAMLQLPAQRTHRIRAANFAALRPWPGLT

VEIRKSAPAQ

>CYP139A1(2592426321)Mycobacterium tuberculosis TKK_02_0013

MRYPLGEALLALYRWRGPLINAGVGGHGYTYLLGAEANRFVFANADAFSWSQTFESLVPV

DGPTALIVSDGADHRRRRSVVAPGLRHHHVQRYVATMVSNIDTVIDGWQPGQRLDIYQEL

RSAVRRSTAESLFGQRLAVHSDFLGEQLQPLLDLTRRPPQVMRLQQRVNSPGWRRAMAAR

KRIDDLIDAQIADARTAPRPDDHMLTTLISGCSEEGTTLSDNEIRDSIVSLITAGYETTS

GALAWAIYALLTVPGTWESAASEVARVLGGRVPAADDLSALTYLNGVVHETLRLYSPGVI

SARRVLRDLWFDGHRIRAGRLLIFSAYVTHRLPEIWPEPTEFRPLRWDPNAADYRKPAPH

EFIPFSGGLHRCIGAVMATTEMTVILARLVARAMLQLPAQRTHRIRAANFAALRPWPGLT

VEIRKSAPAQ

>CYP139A1(2575094331)Mycobacterium tuberculosis KT-0051

MRYPLGEALLALYRWRGPLINAGVGGHGYTYLLGAEANRFVFANADAFSWSQTFESLVPV

DGPTALIVSDGADHRRRRSVVAPGLRHHHVQRYVATMVSNIDTVIDGWQPGQRLDIYQEL

RSAVRRSTAESLFGQRLAVHSDFLGEQLQPLLDLTRRPPQVMRLQQRVNSPGWRRAMAAR

KRIDDLIDAQIADARTAPRPDDHMLTTLISGCSEEGTTLSDNEIRDSIVSLITAGYETTS

GALAWAIYALLTVPGTWESAASEVARVLGGRVPAADDLSALTYLNGVVHETLRLYSPGVI

SARRVLRDLWFDGHRIRAGRLLIFSAYVTHRLPEIWPEPTEFRPLRWDPNAADYRKPAPH

EFIPFSGGLHRCIGAVMATTEMTVILARLVARAMLQLPAQRTHRIRAANFAALRPWPGLT

VEIRKSAPAQ

>CYP139A1(2576101264)Mycobacterium tuberculosis KT-0072

MRYPLGEALLALYRWRGPLINAGVGGHGYTYLLGAEANRFVFANADAFSWSQTFESLVPV

DGPTALIVSDGADHRRRRSVVAPGLRHHHVQRYVATMVSNIDTVIDGWQPGQRLDIYQEL

RSAVRRSTAESLFGQRLAVHSDFLGEQLQPLLDLTRRPPQVMRLQQRVNSPGWRRAMAAR

KRIDDLIDAQIADARTAPRPDDHMLTTLISGCSEEGTTLSDNEIRDSIVSLITAGYETTS

GALAWAIYALLTVPGTWESAASEVARVLGGRVPAADDLSALTYLNGVVHETLRLYSPGVI

SARRVLRDLWFDGHRIRAGRLLIFSAYVTHRLPEIWPEPTEFRPLRWDPNAADYRKPAPH

EFIPFSGGLHRCIGAVMATTEMTVILARLVARAMLQLPAQRTHRIRAANFAALRPWPGLT

VEIRKSAPAQ

>CYP139A1(2576927477)Mycobacterium tuberculosis M1913

MRYPLGEALLALYRWRGPLINAGVGGHGYTYLLGAEANRFVFANADAFSWSQTFESLVPV

DGPTALIVSDGADHRRRRSVVAPGLRHHHVQRYVATMVSNIDTVIDGWQPGQRLDIYQEL

RSAVRRSTAESLFGQRLAVHSDFLGEQLQPLLDLTRRPPQVMRLQQRVNSPGWRRAMAAR

KRIDDLIDAQIADARTAPRPDDHMLTTLISGCSEEGTTLSDNEIRDSIVSLITAGYETTS

GALAWAIYALLTVPGTWESAASEVARVLGGRVPAADDLSALTYLNGVVHETLRLYSPGVI

SARRVLRDLWFDGHRIRAGRLLIFSAYVTHRLPEIWPEPTEFRPLRWDPNAADYRKPAPH

EFIPFSGGLHRCIGAVMATTEMTVILARLVARAMLQLPAQRTHRIRAANFAALRPWPGLT

VEIRKSAPAQ

>CYP139A1(2577009596)Mycobacterium tuberculosis M1418

MRYPLGEALLALYRWRGPLINAGVGGHGYTYLLGAEANRFVFANADAFSWSQTFESLVPV

DGPTALIVSDGADHRRRRSVVAPGLRHHHVQRYVATMVSNIDTVIDGWQPGQRLDIYQEL

RSAVRRSTAESLFGQRLAVHSDFLGEQLQPLLDLTRRPPQVMRLQQRVNSPGWRRAMAAR

KRIDDLIDAQIADARTAPRPDDHMLTTLISGCSEEGTTLSDNEIRDSIVSLITAGYETTS

GALAWAIYALLTVPGTWESAASEVARVLGGRVPAADDLSALTYLNGVVHETLRLYSPGVI

SARRVLRDLWFDGHRIRAGRLLIFSAYVTHRLPEIWPEPTEFRPLRWDPNAADYRKPAPH

EFIPFSGGLHRCIGAVMATTEMTVILARLVARAMLQLPAQRTHRIRAANFAALRPWPGLT

VEIRKSAPAQ

>CYP139A1(2577884547)Mycobacterium tuberculosis M1017

MRYPLGEALLALYRWRGPLINAGVGGHGYTYLLGAEANRFVFANADAFSWSQTFESLVPV

DGPTALIVSDGADHRRRRSVVAPGLRHHHVQRYVATMVSNIDTVIDGWQPGQRLDIYQEL

RSAVRRSTAESLFGQRLAVHSDFLGEQLQPLLDLTRRPPQVMRLQQRVNSPGWRRAMAAR

KRIDDLIDAQIADARTAPRPDDHMLTTLISGCSEEGTTLSDNEIRDSIVSLITAGYETTS

GALAWAIYALLTVPGTWESAASEVARVLGGRVPAADDLSALTYLNGVVHETLRLYSPGVI

SARRVLRDLWFDGHRIRAGRLLIFSAYVTHRLPEIWPEPTEFRPLRWDPNAADYRKPAPH

EFIPFSGGLHRCIGAVMATTEMTVILARLVARAMLQLPAQRTHRIRAANFAALRPWPGLT

VEIRKSAPAQ

>CYP139A1(2577997123)Mycobacterium tuberculosis KT-0027

MRYPLGEALLALYRWRGPLINAGVGGHGYTYLLGAEANRFVFANADAFSWSQTFESLVPV

DGPTALIVSDGADHRRRRSVVAPGLRHHHVQRYVATMVSNIDTVIDGWQPGQRLDIYQEL

RSAVRRSTAESLFGQRLAVHSDFLGEQLQPLLDLTRRPPQVMRLQQRVNSPGWRRAMAAR

KRIDDLIDAQIADARTAPRPDDHMLTTLISGCSEEGTTLSDNEIRDSIVSLITAGYETTS

GALAWAIYALLTVPGTWESAASEVARVLGGRVPAADDLSALTYLNGVVHETLRLYSPGVI

SARRVLRDLWFDGHRIRAGRLLIFSAYVTHRLPEIWPEPTEFRPLRWDPNAADYRKPAPH

EFIPFSGGLHRCIGAVMATTEMTVILARLVARAMLQLPAQRTHRIRAANFAALRPWPGLT

VEIRKSAPAQ

>CYP139A1(2578155745)Mycobacterium tuberculosis M1025

MRYPLGEALLALYRWRGPLINAGVGGHGYTYLLGAEANRFVFANADAFSWSQTFESLVPV

DGPTALIVSDGADHRRRRSVVAPGLRHHHVQRYVATMVSNIDTVIDGWQPGQRLDIYQEL

RSAVRRSTAESLFGQRLAVHSDFLGEQLQPLLDLTRRPPQVMRLQQRVNSPGWRRAMAAR

KRIDDLIDAQIADARTAPRPDDHMLTTLISGCSEEGTTLSDNEIRDSIVSLITAGYETTS

GALAWAIYALLTVPGTWESAASEVARVLGGRVPAADDLSALTYLNGVVHETLRLYSPGVI

SARRVLRDLWFDGHRIRAGRLLIFSAYVTHRLPEIWPEPTEFRPLRWDPNAADYRKPAPH

EFIPFSGGLHRCIGAVMATTEMTVILARLVARAMLQLPAQRTHRIRAANFAALRPWPGLT

VEIRKSAPAQ

>CYP139A1(2581807696)*Mycobacterium africanum* MAL010123

MRYPLGEALLALYRWRGPLINAGVGGHGYTYLLGAEANRFVFANADAFSWSQTFESLVPV

DGPTALIVSDGADHRRRRSVVAPGLRHHHVQRYVATMVSNIDTVIDGWQPGQRLDIYQEL

RSAVRRSTAESLFGQRLAVHSDFLGEQLQPLLDLTRRPPQVMRLQQRVNSPGWRRAMAAR

KRIDDLIDAQIADARTAPRPDDHMLTTLISGCSEEGTTLSDNEIRDSIVSLITAGYETTS

GALAWAIYALLTVPGTWESAASEVARVLGGRVPAADDLSALTYLNGVVHETLRLYSPGVI

SARRVLRDLWFDGHRIRAGRLLIFSAYVTHRLPEIWPEPTEFRPLRWDPNAADYRKPAPH

EFIPFSGGLHRCIGAVMATTEMTVILARLVARAMLQLPAQRTHRIRAANFAALRPWPGLT

VEIRKSAPAQ

>CYP139A1(2582018155)*Mycobacterium africanum* MAL010071

MRYPLGEALLALYRWRGPLINAGVGGHGYTYLLGAEANRFVFANADAFSWSQTFESLVPV

DGPTALIVSDGADHRRRRSVVAPGLRHHHVQRYVATMVSNIDTVIDGWQPGQRLDIYQEL

RSAVRRSTAESLFGQRLAVHSDFLGEQLQPLLDLTRRPPQVMRLQQRVNSPGWRRAMAAR

KRIDDLIDAQIADARTAPRPDDHMLTTLISGCSEEGTTLSDNEIRDSIVSLITAGYETTS

GALAWAIYALLTVPGTWESAASEVARVLGGRVPAADDLSALTYLNGVVHETLRLYSPGVI

SARRVLRDLWFDGHRIRAGRLLIFSAYVTHRLPEIWPEPTEFRPLRWDPNAADYRKPAPH

EFIPFSGGLHRCIGAVMATTEMTVILARLVARAMLQLPAQRTHRIRAANFAALRPWPGLT

VEIRKSAPAQ

>CYP139A1(2589154518)Mycobacterium tuberculosis OFXR-11

MRYPLGEALLALYRWRGPLINAGVGGHGYTYLLGAEANRFVFANADAFSWSQTFESLVPV

DGPTALIVSDGADHRRRRSVVAPGLRHHHVQRYVATMVSNIDTVIDGWQPGQRLDIYQEL

RSAVRRSTAESLFGQRLAVHSDFLGEQLQPLLDLTRRPPQVMRLQQRVNSPGWRRAMAAR

KRIDDLIDAQIADARTAPRPDDHMLTTLISGCSEEGTTLSDNEIRDSIVSLITAGYETTS

GALAWAIYALLTVPGTWESAASEVARVLGGRVPAADDLSALTYLNGVVHETLRLYSPGVI

SARRVLRDLWFDGHRIRAGRLLIFSAYVTHRLPEIWPEPTEFRPLRWDPNAADYRKPAPH

EFIPFSGGLHRCIGAVMATTEMTVILARLVARAMLQLPAQRTHRIRAANFAALRPWPGLT

VEIRKSAPAQ

>CYP139A1(2590109466)Mycobacterium tuberculosis MAL020152

MRYPLGEALLALYRWRGPLINAGVGGHGYTYLLGAEANRFVFANADAFSWSQTFESLVPV

DGPTALIVSDGADHRRRRSVVAPGLRHHHVQRYVATMVSNIDTVIDGWQPGQRLDIYQEL

RSAVRRSTAESLFGQRLAVHSDFLGEQLQPLLDLTRRPPQVMRLQQRVNSPGWRRAMAAR

KRIDDLIDAQIADARTAPRPDDHMLTTLISGCSEEGTTLSDNEIRDSIVSLITAGYETTS

GALAWAIYALLTVPGTWESAASEVARVLGGRVPAADDLSALTYLNGVVHETLRLYSPGVI

SARRVLRDLWFDGHRIRAGRLLIFSAYVTHRLPEIWPEPTEFRPLRWDPNAADYRKPAPH

EFIPFSGGLHRCIGAVMATTEMTVILARLVARAMLQLPAQRTHRIRAANFAALRPWPGLT

VEIRKSAPAQ

>CYP139A1(2590195268)Mycobacterium tuberculosis MAL020209

MRYPLGEALLALYRWRGPLINAGVGGHGYTYLLGAEANRFVFANADAFSWSQTFESLVPV

DGPTALIVSDGADHRRRRSVVAPGLRHHHVQRYVATMVSNIDTVIDGWQPGQRLDIYQEL

RSAVRRSTAESLFGQRLAVHSDFLGEQLQPLLDLTRRPPQVMRLQQRVNSPGWRRAMAAR

KRIDDLIDAQIADARTAPRPDDHMLTTLISGCSEEGTTLSDNEIRDSIVSLITAGYETTS

GALAWAIYALLTVPGTWESAASEVARVLGGRVPAADDLSALTYLNGVVHETLRLYSPGVI

SARRVLRDLWFDGHRIRAGRLLIFSAYVTHRLPEIWPEPTEFRPLRWDPNAADYRKPAPH

EFIPFSGGLHRCIGAVMATTEMTVILARLVARAMLQLPAQRTHRIRAANFAALRPWPGLT

VEIRKSAPAQ

>CYP139A1(2590198349)Mycobacterium tuberculosis MAL020211

MRYPLGEALLALYRWRGPLINAGVGGHGYTYLLGAEANRFVFANADAFSWSQTFESLVPV

DGPTALIVSDGADHRRRRSVVAPGLRHHHVQRYVATMVSNIDTVIDGWQPGQRLDIYQEL

RSAVRRSTAESLFGQRLAVHSDFLGEQLQPLLDLTRRPPQVMRLQQRVNSPGWRRAMAAR

KRIDDLIDAQIADARTAPRPDDHMLTTLISGCSEEGTTLSDNEIRDSIVSLITAGYETTS

GALAWAIYALLTVPGTWESAASEVARVLGGRVPAADDLSALTYLNGVVHETLRLYSPGVI

SARRVLRDLWFDGHRIRAGRLLIFSAYVTHRLPEIWPEPTEFRPLRWDPNAADYRKPAPH

EFIPFSGGLHRCIGAVMATTEMTVILARLVARAMLQLPAQRTHRIRAANFAALRPWPGLT

VEIRKSAPAQ

>CYP139A1(2574799840)Mycobacterium tuberculosis TKK_05MA_0020

MRYPLGEALLALYRWRGPLINAGVGGHGYTYLLGAEANRFVFANADAFSWSQTFESLVPV

DGPTALIVSDGADHRRRRSVVAPGLRHHHVQRYVATMVSNIDTVIDGWQPGQRLDIYQEL

RSAVRRSTAESLFGQRLAVHSDFLGEQLQPLLDLTRRPPQVMRLQQRVNSPGWRRAMAAR

KRIDDLIDAQIADARTAPRPDDHMLTTLISGCSEEGTTLSDNEIRDSIVSLITAGYETTS

GALAWAIYALLTVPGTWESAASEVARVLGGRVPAADDLSALTYLNGVVHETLRLYSPGVI

SARRVLRDLWFDGHRIRAGRLLIFSAYVTHRLPEIWPEPTEFRPLRWDPNAADYRKPAPH

EFIPFSGGLHRCIGAVMATTEMTVILARLVARAMLQLPAQRTHRIRAANFAALRPWPGLT

VEIRKSAPAQ

>CYP139A1(2576399693)Mycobacterium tuberculosis TB_RSA195

MRYPLGEALLALYRWRGPLINAGVGGHGYTYLLGAEANRFVFANADAFSWSQTFESLVPV

DGPTALIVSDGADHRRRRSVVAPGLRHHHVQRYVATMVSNIDTVIDGWQPGQRLDIYQEL

RSAVRRSTAESLFGQRLAVHSDFLGEQLQPLLDLTRRPPQVMRLQQRVNSPGWRRAMAAR

KRIDDLIDAQIADARTAPRPDDHMLTTLISGCSEEGTTLSDNEIRDSIVSLITAGYETTS

GALAWAIYALLTVPGTWESAASEVARVLGGRVPAADDLSALTYLNGVVHETLRLYSPGVI

SARRVLRDLWFDGHRIRAGRLLIFSAYVTHRLPEIWPEPTEFRPLRWDPNAADYRKPAPH

EFIPFSGGLHRCIGAVMATTEMTVILARLVARAMLQLPAQRTHRIRAANFAALRPWPGLT

VEIRKSAPAQ

>CYP139A1(2577024745)Mycobacterium tuberculosis TKK-01-0091

MRYPLGEALLALYRWRGPLINAGVGGHGYTYLLGAEANRFVFANADAFSWSQTFESLVPV

DGPTALIVSDGADHRRRRSVVAPGLRHHHVQRYVATMVSNIDTVIDGWQPGQRLDIYQEL

RSAVRRSTAESLFGQRLAVHSDFLGEQLQPLLDLTRRPPQVMRLQQRVNSPGWRRAMAAR

KRIDDLIDAQIADARTAPRPDDHMLTTLISGCSEEGTTLSDNEIRDSIVSLITAGYETTS

GALAWAIYALLTVPGTWESAASEVARVLGGRVPAADDLSALTYLNGVVHETLRLYSPGVI

SARRVLRDLWFDGHRIRAGRLLIFSAYVTHRLPEIWPEPTEFRPLRWDPNAADYRKPAPH

EFIPFSGGLHRCIGAVMATTEMTVILARLVARAMLQLPAQRTHRIRAANFAALRPWPGLT

VEIRKSAPAQ

>CYP139A1(2577872587)Mycobacterium tuberculosis TB_RSA07

MRYPLGEALLALYRWRGPLINAGVGGHGYTYLLGAEANRFVFANADAFSWSQTFESLVPV

DGPTALIVSDGADHRRRRSVVAPGLRHHHVQRYVATMVSNIDTVIDGWQPGQRLDIYQEL

RSAVRRSTAESLFGQRLAVHSDFLGEQLQPLLDLTRRPPQVMRLQQRVNSPGWRRAMAAR

KRIDDLIDAQIADARTAPRPDDHMLTTLISGCSEEGTTLSDNEIRDSIVSLITAGYETTS

GALAWAIYALLTVPGTWESAASEVARVLGGRVPAADDLSALTYLNGVVHETLRLYSPGVI

SARRVLRDLWFDGHRIRAGRLLIFSAYVTHRLPEIWPEPTEFRPLRWDPNAADYRKPAPH

EFIPFSGGLHRCIGAVMATTEMTVILARLVARAMLQLPAQRTHRIRAANFAALRPWPGLT

VEIRKSAPAQ

>CYP139A1(2579813772)Mycobacterium tuberculosis TBR10

MRYPLGEALLALYRWRGPLINAGVGGHGYTYLLGAEANRFVFANADAFSWSQTFESLVPV

DGPTALIVSDGADHRRRRSVVAPGLRHHHVQRYVATMVSNIDTVIDGWQPGQRLDIYQEL

RSAVRRSTAESLFGQRLAVHSDFLGEQLQPLLDLTRRPPQVMRLQQRVNSPGWRRAMAAR

KRIDDLIDAQIADARTAPRPDDHMLTTLISGCSEEGTTLSDNEIRDSIVSLITAGYETTS

GALAWAIYALLTVPGTWESAASEVARVLGGRVPAADDLSALTYLNGVVHETLRLYSPGVI

SARRVLRDLWFDGHRIRAGRLLIFSAYVTHRLPEIWPEPTEFRPLRWDPNAADYRKPAPH

EFIPFSGGLHRCIGAVMATTEMTVILARLVARAMLQLPAQRTHRIRAANFAALRPWPGLT

VEIRKSAPAQ

>CYP139A1(2584772421)Mycobacterium tuberculosis TRUG0080

MRYPLGEALLALYRWRGPLINAGVGGHGYTYLLGAEANRFVFANADAFSWSQTFESLVPV

DGPTALIVSDGADHRRRRSVVAPGLRHHHVQRYVATMVSNIDTVIDGWQPGQRLDIYQEL

RSAVRRSTAESLFGQRLAVHSDFLGEQLQPLLDLTRRPPQVMRLQQRVNSPGWRRAMAAR

KRIDDLIDAQIADARTAPRPDDHMLTTLISGCSEEGTTLSDNEIRDSIVSLITAGYETTS

GALAWAIYALLTVPGTWESAASEVARVLGGRVPAADDLSALTYLNGVVHETLRLYSPGVI

SARRVLRDLWFDGHRIRAGRLLIFSAYVTHRLPEIWPEPTEFRPLRWDPNAADYRKPAPH

EFIPFSGGLHRCIGAVMATTEMTVILARLVARAMLQLPAQRTHRIRAANFAALRPWPGLT

VEIRKSAPAQ

>CYP139A1(2584893836)Mycobacterium tuberculosis TB_RSA107

MRYPLGEALLALYRWRGPLINAGVGGHGYTYLLGAEANRFVFANADAFSWSQTFESLVPV

DGPTALIVSDGADHRRRRSVVAPGLRHHHVQRYVATMVSNIDTVIDGWQPGQRLDIYQEL

RSAVRRSTAESLFGQRLAVHSDFLGEQLQPLLDLTRRPPQVMRLQQRVNSPGWRRAMAAR

KRIDDLIDAQIADARTAPRPDDHMLTTLISGCSEEGTTLSDNEIRDSIVSLITAGYETTS

GALAWAIYALLTVPGTWESAASEVARVLGGRVPAADDLSALTYLNGVVHETLRLYSPGVI

SARRVLRDLWFDGHRIRAGRLLIFSAYVTHRLPEIWPEPTEFRPLRWDPNAADYRKPAPH

EFIPFSGGLHRCIGAVMATTEMTVILARLVARAMLQLPAQRTHRIRAANFAALRPWPGLT

VEIRKSAPAQ

>CYP139A1(2589026697)Mycobacterium tuberculosis TBR8

MRYPLGEALLALYRWRGPLINAGVGGHGYTYLLGAEANRFVFANADAFSWSQTFESLVPV

DGPTALIVSDGADHRRRRSVVAPGLRHHHVQRYVATMVSNIDTVIDGWQPGQRLDIYQEL

RSAVRRSTAESLFGQRLAVHSDFLGEQLQPLLDLTRRPPQVMRLQQRVNSPGWRRAMAAR

KRIDDLIDAQIADARTAPRPDDHMLTTLISGCSEEGTTLSDNEIRDSIVSLITAGYETTS

GALAWAIYALLTVPGTWESAASEVARVLGGRVPAADDLSALTYLNGVVHETLRLYSPGVI

SARRVLRDLWFDGHRIRAGRLLIFSAYVTHRLPEIWPEPTEFRPLRWDPNAADYRKPAPH

EFIPFSGGLHRCIGAVMATTEMTVILARLVARAMLQLPAQRTHRIRAANFAALRPWPGLT

VEIRKSAPAQ

>CYP139A1(2589514641)Mycobacterium tuberculosis TKK-01-0003

MRYPLGEALLALYRWRGPLINAGVGGHGYTYLLGAEANRFVFANADAFSWSQTFESLVPV

DGPTALIVSDGADHRRRRSVVAPGLRHHHVQRYVATMVSNIDTVIDGWQPGQRLDIYQEL

RSAVRRSTAESLFGQRLAVHSDFLGEQLQPLLDLTRRPPQVMRLQQRVNSPGWRRAMAAR

KRIDDLIDAQIADARTAPRPDDHMLTTLISGCSEEGTTLSDNEIRDSIVSLITAGYETTS

GALAWAIYALLTVPGTWESAASEVARVLGGRVPAADDLSALTYLNGVVHETLRLYSPGVI

SARRVLRDLWFDGHRIRAGRLLIFSAYVTHRLPEIWPEPTEFRPLRWDPNAADYRKPAPH

EFIPFSGGLHRCIGAVMATTEMTVILARLVARAMLQLPAQRTHRIRAANFAALRPWPGLT

VEIRKSAPAQ

>CYP139A1(2589547247)Mycobacterium tuberculosis TKK-01-0027

MRYPLGEALLALYRWRGPLINAGVGGHGYTYLLGAEANRFVFANADAFSWSQTFESLVPV

DGPTALIVSDGADHRRRRSVVAPGLRHHHVQRYVATMVSNIDTVIDGWQPGQRLDIYQEL

RSAVRRSTAESLFGQRLAVHSDFLGEQLQPLLDLTRRPPQVMRLQQRVNSPGWRRAMAAR

KRIDDLIDAQIADARTAPRPDDHMLTTLISGCSEEGTTLSDNEIRDSIVSLITAGYETTS

GALAWAIYALLTVPGTWESAASEVARVLGGRVPAADDLSALTYLNGVVHETLRLYSPGVI

SARRVLRDLWFDGHRIRAGRLLIFSAYVTHRLPEIWPEPTEFRPLRWDPNAADYRKPAPH

EFIPFSGGLHRCIGAVMATTEMTVILARLVARAMLQLPAQRTHRIRAANFAALRPWPGLT

VEIRKSAPAQ

>CYP139A1(2589620637)Mycobacterium tuberculosis TKK-01-0045

MRYPLGEALLALYRWRGPLINAGVGGHGYTYLLGAEANRFVFANADAFSWSQTFESLVPV

DGPTALIVSDGADHRRRRSVVAPGLRHHHVQRYVATMVSNIDTVIDGWQPGQRLDIYQEL

RSAVRRSTAESLFGQRLAVHSDFLGEQLQPLLDLTRRPPQVMRLQQRVNSPGWRRAMAAR

KRIDDLIDAQIADARTAPRPDDHMLTTLISGCSEEGTTLSDNEIRDSIVSLITAGYETTS

GALAWAIYALLTVPGTWESAASEVARVLGGRVPAADDLSALTYLNGVVHETLRLYSPGVI

SARRVLRDLWFDGHRIRAGRLLIFSAYVTHRLPEIWPEPTEFRPLRWDPNAADYRKPAPH

EFIPFSGGLHRCIGAVMATTEMTVILARLVARAMLQLPAQRTHRIRAANFAALRPWPGLT

VEIRKSAPAQ

>CYP139A1(2592230560)Mycobacterium tuberculosis TKK_04_0020

MRYPLGEALLALYRWRGPLINAGVGGHGYTYLLGAEANRFVFANADAFSWSQTFESLVPV

DGPTALIVSDGADHRRRRSVVAPGLRHHHVQRYVATMVSNIDTVIDGWQPGQRLDIYQEL

RSAVRRSTAESLFGQRLAVHSDFLGEQLQPLLDLTRRPPQVMRLQQRVNSPGWRRAMAAR

KRIDDLIDAQIADARTAPRPDDHMLTTLISGCSEEGTTLSDNEIRDSIVSLITAGYETTS

GALAWAIYALLTVPGTWESAASEVARVLGGRVPAADDLSALTYLNGVVHETLRLYSPGVI

SARRVLRDLWFDGHRIRAGRLLIFSAYVTHRLPEIWPEPTEFRPLRWDPNAADYRKPAPH

EFIPFSGGLHRCIGAVMATTEMTVILARLVARAMLQLPAQRTHRIRAANFAALRPWPGLT

VEIRKSAPAQ

>CYP139A1(2592242791)Mycobacterium tuberculosis TKK_04_0015

MRYPLGEALLALYRWRGPLINAGVGGHGYTYLLGAEANRFVFANADAFSWSQTFESLVPV

DGPTALIVSDGADHRRRRSVVAPGLRHHHVQRYVATMVSNIDTVIDGWQPGQRLDIYQEL

RSAVRRSTAESLFGQRLAVHSDFLGEQLQPLLDLTRRPPQVMRLQQRVNSPGWRRAMAAR

KRIDDLIDAQIADARTAPRPDDHMLTTLISGCSEEGTTLSDNEIRDSIVSLITAGYETTS

GALAWAIYALLTVPGTWESAASEVARVLGGRVPAADDLSALTYLNGVVHETLRLYSPGVI

SARRVLRDLWFDGHRIRAGRLLIFSAYVTHRLPEIWPEPTEFRPLRWDPNAADYRKPAPH

EFIPFSGGLHRCIGAVMATTEMTVILARLVARAMLQLPAQRTHRIRAANFAALRPWPGLT

VEIRKSAPAQ

>CYP139A1(2592299949)Mycobacterium tuberculosis TKK_03_0033

MRYPLGEALLALYRWRGPLINAGVGGHGYTYLLGAEANRFVFANADAFSWSQTFESLVPV

DGPTALIVSDGADHRRRRSVVAPGLRHHHVQRYVATMVSNIDTVIDGWQPGQRLDIYQEL

RSAVRRSTAESLFGQRLAVHSDFLGEQLQPLLDLTRRPPQVMRLQQRVNSPGWRRAMAAR

KRIDDLIDAQIADARTAPRPDDHMLTTLISGCSEEGTTLSDNEIRDSIVSLITAGYETTS

GALAWAIYALLTVPGTWESAASEVARVLGGRVPAADDLSALTYLNGVVHETLRLYSPGVI

SARRVLRDLWFDGHRIRAGRLLIFSAYVTHRLPEIWPEPTEFRPLRWDPNAADYRKPAPH

EFIPFSGGLHRCIGAVMATTEMTVILARLVARAMLQLPAQRTHRIRAANFAALRPWPGLT

VEIRKSAPAQ

>CYP139A1(2592377452)Mycobacterium tuberculosis TKK_02_0039

MRYPLGEALLALYRWRGPLINAGVGGHGYTYLLGAEANRFVFANADAFSWSQTFESLVPV

DGPTALIVSDGADHRRRRSVVAPGLRHHHVQRYVATMVSNIDTVIDGWQPGQRLDIYQEL

RSAVRRSTAESLFGQRLAVHSDFLGEQLQPLLDLTRRPPQVMRLQQRVNSPGWRRAMAAR

KRIDDLIDAQIADARTAPRPDDHMLTTLISGCSEEGTTLSDNEIRDSIVSLITAGYETTS

GALAWAIYALLTVPGTWESAASEVARVLGGRVPAADDLSALTYLNGVVHETLRLYSPGVI

SARRVLRDLWFDGHRIRAGRLLIFSAYVTHRLPEIWPEPTEFRPLRWDPNAADYRKPAPH

EFIPFSGGLHRCIGAVMATTEMTVILARLVARAMLQLPAQRTHRIRAANFAALRPWPGLT

VEIRKSAPAQ

>CYP139A1(2592405959)Mycobacterium tuberculosis TKK_02_0021

MRYPLGEALLALYRWRGPLINAGVGGHGYTYLLGAEANRFVFANADAFSWSQTFESLVPV

DGPTALIVSDGADHRRRRSVVAPGLRHHHVQRYVATMVSNIDTVIDGWQPGQRLDIYQEL

RSAVRRSTAESLFGQRLAVHSDFLGEQLQPLLDLTRRPPQVMRLQQRVNSPGWRRAMAAR

KRIDDLIDAQIADARTAPRPDDHMLTTLISGCSEEGTTLSDNEIRDSIVSLITAGYETTS

GALAWAIYALLTVPGTWESAASEVARVLGGRVPAADDLSALTYLNGVVHETLRLYSPGVI

SARRVLRDLWFDGHRIRAGRLLIFSAYVTHRLPEIWPEPTEFRPLRWDPNAADYRKPAPH

EFIPFSGGLHRCIGAVMATTEMTVILARLVARAMLQLPAQRTHRIRAANFAALRPWPGLT

VEIRKSAPAQ

>CYP139A1(2592558321)Mycobacterium tuberculosis TKK_04_0037

MRYPLGEALLALYRWRGPLINAGVGGHGYTYLLGAEANRFVFANADAFSWSQTFESLVPV

DGPTALIVSDGADHRRRRSVVAPGLRHHHVQRYVATMVSNIDTVIDGWQPGQRLDIYQEL

RSAVRRSTAESLFGQRLAVHSDFLGEQLQPLLDLTRRPPQVMRLQQRVNSPGWRRAMAAR

KRIDDLIDAQIADARTAPRPDDHMLTTLISGCSEEGTTLSDNEIRDSIVSLITAGYETTS

GALAWAIYALLTVPGTWESAASEVARVLGGRVPAADDLSALTYLNGVVHETLRLYSPGVI

SARRVLRDLWFDGHRIRAGRLLIFSAYVTHRLPEIWPEPTEFRPLRWDPNAADYRKPAPH

EFIPFSGGLHRCIGAVMATTEMTVILARLVARAMLQLPAQRTHRIRAANFAALRPWPGLT

VEIRKSAPAQ

>CYP139A1(648456112)Mycobacterium tuberculosis SUMu004

MRYPLGEALLALYRWRGPLINAGVGGHGYTYLLGAEANRFVFANADAFSWSQTFESLVPV

DGPTALIVSDGADHRRRRSVVAPGLRHHHVQRYVATMVSNIDTVIDGWQPGQRLDIYQEL

RSAVRRSTAESLFGQRLAVHSDFLGEQLQPLLDLTRRPPQVMRLQQRVNSPGWRRAMAAR

KRIDDLIDAQIADARTAPRPDDHMLTTLISGCSEEGTTLSDNEIRDSIVSLITAGYETTS

GALAWAIYALLTVPGTWESAASEVARVLGGRVPAADDLSALTYLNGVVHETLRLYSPGVI

SARRVLRDLWFDGHRIRAGRLLIFSAYVTHRLPEIWPEPTEFRPLRWDPNAADYRKPAPH

EFIPFSGGLHRCIGAVMATTEMTVILARLVARAMLQLPAQRTHRIRAANFAALRPWPGLT

VEIRKSAPAQ

>CYP139A1(2575561335)Mycobacterium tuberculosis M1213

MRYPLGEALLALYRWRGPLINAGVGGHGYTYLLGAEANRFVFANADAFSWSQTFESLVPV

DGPTALIVSDGADHRRRRSVVAPGLRHHHVQRYVATMVSNIDTVIDGWQPGQRLDIYQEL

RSAVRRSTAESLFGQRLAVHSDFLGEQLQPLLDLTRRPPQVMRLQQRVNSPGWRRAMAAR

KRIDDLIDAQIADARTAPRPDDHMLTTLISGCSEEGTTLSDNEIRDSIVSLITAGYETTS

GALAWAIYALLTVPGTWESAASEVARVLGGRVPAADDLSALTYLNGVVHETLRLYSPGVI

SARRVLRDLWFDGHRIRAGRLLIFSAYVTHRLPEIWPEPTEFRPLRWDPNAADYRKPAPH

EFIPFSGGLHRCIGAVMATTEMTVILARLVARAMLQLPAQRTHRIRAANFAALRPWPGLT

VEIRKSAPAQ

>CYP139A1(2575869049)Mycobacterium tuberculosis KT-0034

MRYPLGEALLALYRWRGPLINAGVGGHGYTYLLGAEANRFVFANADAFSWSQTFESLVPV

DGPTALIVSDGADHRRRRSVVAPGLRHHHVQRYVATMVSNIDTVIDGWQPGQRLDIYQEL

RSAVRRSTAESLFGQRLAVHSDFLGEQLQPLLDLTRRPPQVMRLQQRVNSPGWRRAMAAR

KRIDDLIDAQIADARTAPRPDDHMLTTLISGCSEEGTTLSDNEIRDSIVSLITAGYETTS

GALAWAIYALLTVPGTWESAASEVARVLGGRVPAADDLSALTYLNGVVHETLRLYSPGVI

SARRVLRDLWFDGHRIRAGRLLIFSAYVTHRLPEIWPEPTEFRPLRWDPNAADYRKPAPH

EFIPFSGGLHRCIGAVMATTEMTVILARLVARAMLQLPAQRTHRIRAANFAALRPWPGLT

VEIRKSAPAQ

>CYP139A1(2576250927)Mycobacterium tuberculosis KT-0110

MRYPLGEALLALYRWRGPLINAGVGGHGYTYLLGAEANRFVFANADAFSWSQTFESLVPV

DGPTALIVSDGADHRRRRSVVAPGLRHHHVQRYVATMVSNIDTVIDGWQPGQRLDIYQEL

RSAVRRSTAESLFGQRLAVHSDFLGEQLQPLLDLTRRPPQVMRLQQRVNSPGWRRAMAAR

KRIDDLIDAQIADARTAPRPDDHMLTTLISGCSEEGTTLSDNEIRDSIVSLITAGYETTS

GALAWAIYALLTVPGTWESAASEVARVLGGRVPAADDLSALTYLNGVVHETLRLYSPGVI

SARRVLRDLWFDGHRIRAGRLLIFSAYVTHRLPEIWPEPTEFRPLRWDPNAADYRKPAPH

EFIPFSGGLHRCIGAVMATTEMTVILARLVARAMLQLPAQRTHRIRAANFAALRPWPGLT

VEIRKSAPAQ

>CYP139A1(2577075963)Mycobacterium tuberculosis BTB03-012

MRYPLGEALLALYRWRGPLINAGVGGHGYTYLLGAEANRFVFANADAFSWSQTFESLVPV

DGPTALIVSDGADHRRRRSVVAPGLRHHHVQRYVATMVSNIDTVIDGWQPGQRLDIYQEL

RSAVRRSTAESLFGQRLAVHSDFLGEQLQPLLDLTRRPPQVMRLQQRVNSPGWRRAMAAR

KRIDDLIDAQIADARTAPRPDDHMLTTLISGCSEEGTTLSDNEIRDSIVSLITAGYETTS

GALAWAIYALLTVPGTWESAASEVARVLGGRVPAADDLSALTYLNGVVHETLRLYSPGVI

SARRVLRDLWFDGHRIRAGRLLIFSAYVTHRLPEIWPEPTEFRPLRWDPNAADYRKPAPH

EFIPFSGGLHRCIGAVMATTEMTVILARLVARAMLQLPAQRTHRIRAANFAALRPWPGLT

VEIRKSAPAQ

>CYP139A1(2577655387)Mycobacterium tuberculosis KT-0070

MRYPLGEALLALYRWRGPLINAGVGGHGYTYLLGAEANRFVFANADAFSWSQTFESLVPV

DGPTALIVSDGADHRRRRSVVAPGLRHHHVQRYVATMVSNIDTVIDGWQPGQRLDIYQEL

RSAVRRSTAESLFGQRLAVHSDFLGEQLQPLLDLTRRPPQVMRLQQRVNSPGWRRAMAAR

KRIDDLIDAQIADARTAPRPDDHMLTTLISGCSEEGTTLSDNEIRDSIVSLITAGYETTS

GALAWAIYALLTVPGTWESAASEVARVLGGRVPAADDLSALTYLNGVVHETLRLYSPGVI

SARRVLRDLWFDGHRIRAGRLLIFSAYVTHRLPEIWPEPTEFRPLRWDPNAADYRKPAPH

EFIPFSGGLHRCIGAVMATTEMTVILARLVARAMLQLPAQRTHRIRAANFAALRPWPGLT

VEIRKSAPAQ

>CYP139A1(2579808474)Mycobacterium tuberculosis M995

MRYPLGEALLALYRWRGPLINAGVGGHGYTYLLGAEANRFVFANADAFSWSQTFESLVPV

DGPTALIVSDGADHRRRRSVVAPGLRHHHVQRYVATMVSNIDTVIDGWQPGQRLDIYQEL

RSAVRRSTAESLFGQRLAVHSDFLGEQLQPLLDLTRRPPQVMRLQQRVNSPGWRRAMAAR

KRIDDLIDAQIADARTAPRPDDHMLTTLISGCSEEGTTLSDNEIRDSIVSLITAGYETTS

GALAWAIYALLTVPGTWESAASEVARVLGGRVPAADDLSALTYLNGVVHETLRLYSPGVI

SARRVLRDLWFDGHRIRAGRLLIFSAYVTHRLPEIWPEPTEFRPLRWDPNAADYRKPAPH

EFIPFSGGLHRCIGAVMATTEMTVILARLVARAMLQLPAQRTHRIRAANFAALRPWPGLT

VEIRKSAPAQ

>CYP139A1(2580939152)*Mycobacterium africanum* MAL010079

MRYPLGEALLALYRWRGPLINAGVGGHGYTYLLGAEANRFVFANADAFSWSQTFESLVPV

DGPTALIVSDGADHRRRRSVVAPGLRHHHVQRYVATMVSNIDTVIDGWQPGQRLDIYQEL

RSAVRRSTAESLFGQRLAVHSDFLGEQLQPLLDLTRRPPQVMRLQQRVNSPGWRRAMAAR

KRIDDLIDAQIADARTAPRPDDHMLTTLISGCSEEGTTLSDNEIRDSIVSLITAGYETTS

GALAWAIYALLTVPGTWESAASEVARVLGGRVPAADDLSALTYLNGVVHETLRLYSPGVI

SARRVLRDLWFDGHRIRAGRLLIFSAYVTHRLPEIWPEPTEFRPLRWDPNAADYRKPAPH

EFIPFSGGLHRCIGAVMATTEMTVILARLVARAMLQLPAQRTHRIRAANFAALRPWPGLT

VEIRKSAPAQ

>CYP139A1(2581510874)*Mycobacterium africanum* MAL010084

MRYPLGEALLALYRWRGPLINAGVGGHGYTYLLGAEANRFVFANADAFSWSQTFESLVPV

DGPTALIVSDGADHRRRRSVVAPGLRHHHVQRYVATMVSNIDTVIDGWQPGQRLDIYQEL

RSAVRRSTAESLFGQRLAVHSDFLGEQLQPLLDLTRRPPQVMRLQQRVNSPGWRRAMAAR

KRIDDLIDAQIADARTAPRPDDHMLTTLISGCSEEGTTLSDNEIRDSIVSLITAGYETTS

GALAWAIYALLTVPGTWESAASEVARVLGGRVPAADDLSALTYLNGVVHETLRLYSPGVI

SARRVLRDLWFDGHRIRAGRLLIFSAYVTHRLPEIWPEPTEFRPLRWDPNAADYRKPAPH

EFIPFSGGLHRCIGAVMATTEMTVILARLVARAMLQLPAQRTHRIRAANFAALRPWPGLT

VEIRKSAPAQ

>CYP139A1(2590048710)Mycobacterium tuberculosis MAL010124

MRYPLGEALLALYRWRGPLINAGVGGHGYTYLLGAEANRFVFANADAFSWSQTFESLVPV

DGPTALIVSDGADHRRRRSVVAPGLRHHHVQRYVATMVSNIDTVIDGWQPGQRLDIYQEL

RSAVRRSTAESLFGQRLAVHSDFLGEQLQPLLDLTRRPPQVMRLQQRVNSPGWRRAMAAR

KRIDDLIDAQIADARTAPRPDDHMLTTLISGCSEEGTTLSDNEIRDSIVSLITAGYETTS

GALAWAIYALLTVPGTWESAASEVARVLGGRVPAADDLSALTYLNGVVHETLRLYSPGVI

SARRVLRDLWFDGHRIRAGRLLIFSAYVTHRLPEIWPEPTEFRPLRWDPNAADYRKPAPH

EFIPFSGGLHRCIGAVMATTEMTVILARLVARAMLQLPAQRTHRIRAANFAALRPWPGLT

VEIRKSAPAQ

>CYP139A1(2590237321)Mycobacterium tuberculosis KT-0043

MRYPLGEALLALYRWRGPLINAGVGGHGYTYLLGAEANRFVFANADAFSWSQTFESLVPV

DGPTALIVSDGADHRRRRSVVAPGLRHHHVQRYVATMVSNIDTVIDGWQPGQRLDIYQEL

RSAVRRSTAESLFGQRLAVHSDFLGEQLQPLLDLTRRPPQVMRLQQRVNSPGWRRAMAAR

KRIDDLIDAQIADARTAPRPDDHMLTTLISGCSEEGTTLSDNEIRDSIVSLITAGYETTS

GALAWAIYALLTVPGTWESAASEVARVLGGRVPAADDLSALTYLNGVVHETLRLYSPGVI

SARRVLRDLWFDGHRIRAGRLLIFSAYVTHRLPEIWPEPTEFRPLRWDPNAADYRKPAPH

EFIPFSGGLHRCIGAVMATTEMTVILARLVARAMLQLPAQRTHRIRAANFAALRPWPGLT

VEIRKSAPAQ

>CYP139A1(2574784397)Mycobacterium tuberculosis TB_RSA194

MRYPLGEALLALYRWRGPLINAGVGGHGYTYLLGAEANRFVFANADAFSWSQTFESLVPV

DGPTALIVSDGADHRRRRSVVAPGLRHHHVQRYVATMVSNIDTVIDGWQPGQRLDIYQEL

RSAVRRSTAESLFGQRLAVHSDFLGEQLQPLLDLTRRPPQVMRLQQRVNSPGWRRAMAAR

KRIDDLIDAQIADARTAPRPDDHMLTTLISGCSEEGTTLSDNEIRDSIVSLITAGYETTS

GALAWAIYALLTVPGTWESAASEVARVLGGRVPAADDLSALTYLNGVVHETLRLYSPGVI

SARRVLRDLWFDGHRIRAGRLLIFSAYVTHRLPEIWPEPTEFRPLRWDPNAADYRKPAPH

EFIPFSGGLHRCIGAVMATTEMTVILARLVARAMLQLPAQRTHRIRAANFAALRPWPGLT

VEIRKSAPAQ

>CYP139A1(2574860198)Mycobacterium tuberculosis TKK_02_0071

MRYPLGEALLALYRWRGPLINAGVGGHGYTYLLGAEANRFVFANADAFSWSQTFESLVPV

DGPTALIVSDGADHRRRRSVVAPGLRHHHVQRYVATMVSNIDTVIDGWQPGQRLDIYQEL

RSAVRRSTAESLFGQRLAVHSDFLGEQLQPLLDLTRRPPQVMRLQQRVNSPGWRRAMAAR

KRIDDLIDAQIADARTAPRPDDHMLTTLISGCSEEGTTLSDNEIRDSIVSLITAGYETTS

GALAWAIYALLTVPGTWESAASEVARVLGGRVPAADDLSALTYLNGVVHETLRLYSPGVI

SARRVLRDLWFDGHRIRAGRLLIFSAYVTHRLPEIWPEPTEFRPLRWDPNAADYRKPAPH

EFIPFSGGLHRCIGAVMATTEMTVILARLVARAMLQLPAQRTHRIRAANFAALRPWPGLT

VEIRKSAPAQ

>CYP139A1(2574872651)Mycobacterium tuberculosis TB_RSA68

MRYPLGEALLALYRWRGPLINAGVGGHGYTYLLGAEANRFVFANADAFSWSQTFESLVPV

DGPTALIVSDGADHRRRRSVVAPGLRHHHVQRYVATMVSNIDTVIDGWQPGQRLDIYQEL

RSAVRRSTAESLFGQRLAVHSDFLGEQLQPLLDLTRRPPQVMRLQQRVNSPGWRRAMAAR

KRIDDLIDAQIADARTAPRPDDHMLTTLISGCSEEGTTLSDNEIRDSIVSLITAGYETTS

GALAWAIYALLTVPGTWESAASEVARVLGGRVPAADDLSALTYLNGVVHETLRLYSPGVI

SARRVLRDLWFDGHRIRAGRLLIFSAYVTHRLPEIWPEPTEFRPLRWDPNAADYRKPAPH

EFIPFSGGLHRCIGAVMATTEMTVILARLVARAMLQLPAQRTHRIRAANFAALRPWPGLT

VEIRKSAPAQ

>CYP139A1(2575185207)Mycobacterium tuberculosis MD19964

MRYPLGEALLALYRWRGPLINAGVGGHGYTYLLGAEANRFVFANADAFSWSQTFESLVPV

DGPTALIVSDGADHRRRRSVVAPGLRHHHVQRYVATMVSNIDTVIDGWQPGQRLDIYQEL

RSAVRRSTAESLFGQRLAVHSDFLGEQLQPLLDLTRRPPQVMRLQQRVNSPGWRRAMAAR

KRIDDLIDAQIADARTAPRPDDHMLTTLISGCSEEGTTLSDNEIRDSIVSLITAGYETTS

GALAWAIYALLTVPGTWESAASEVARVLGGRVPAADDLSALTYLNGVVHETLRLYSPGVI

SARRVLRDLWFDGHRIRAGRLLIFSAYVTHRLPEIWPEPTEFRPLRWDPNAADYRKPAPH

EFIPFSGGLHRCIGAVMATTEMTVILARLVARAMLQLPAQRTHRIRAANFAALRPWPGLT

VEIRKSAPAQ

>CYP139A1(2576316581)Mycobacterium tuberculosis XTB13-143

MRYPLGEALLALYRWRGPLINAGVGGHGYTYLLGAEANRFVFANADAFSWSQTFESLVPV

DGPTALIVSDGADHRRRRSVVAPGLRHHHVQRYVATMVSNIDTVIDGWQPGQRLDIYQEL

RSAVRRSTAESLFGQRLAVHSDFLGEQLQPLLDLTRRPPQVMRLQQRVNSPGWRRAMAAR

KRIDDLIDAQIADARTAPRPDDHMLTTLISGCSEEGTTLSDNEIRDSIVSLITAGYETTS

GALAWAIYALLTVPGTWESAASEVARVLGGRVPAADDLSALTYLNGVVHETLRLYSPGVI

SARRVLRDLWFDGHRIRAGRLLIFSAYVTHRLPEIWPEPTEFRPLRWDPNAADYRKPAPH

EFIPFSGGLHRCIGAVMATTEMTVILARLVARAMLQLPAQRTHRIRAANFAALRPWPGLT

VEIRKSAPAQ

>CYP139A1(2576932272)Mycobacterium tuberculosis TKK_03_0158

MRYPLGEALLALYRWRGPLINAGVGGHGYTYLLGAEANRFVFANADAFSWSQTFESLVPV

DGPTALIVSDGADHRRRRSVVAPGLRHHHVQRYVATMVSNIDTVIDGWQPGQRLDIYQEL

RSAVRRSTAESLFGQRLAVHSDFLGEQLQPLLDLTRRPPQVMRLQQRVNSPGWRRAMAAR

KRIDDLIDAQIADARTAPRPDDHMLTTLISGCSEEGTTLSDNEIRDSIVSLITAGYETTS

GALAWAIYALLTVPGTWESAASEVARVLGGRVPAADDLSALTYLNGVVHETLRLYSPGVI

SARRVLRDLWFDGHRIRAGRLLIFSAYVTHRLPEIWPEPTEFRPLRWDPNAADYRKPAPH

EFIPFSGGLHRCIGAVMATTEMTVILARLVARAMLQLPAQRTHRIRAANFAALRPWPGLT

VEIRKSAPAQ

>CYP139A1(2577632297)Mycobacterium tuberculosis MD17517

MRYPLGEALLALYRWRGPLINAGVGGHGYTYLLGAEANRFVFANADAFSWSQTFESLVPV

DGPTALIVSDGADHRRRRSVVAPGLRHHHVQRYVATMVSNIDTVIDGWQPGQRLDIYQEL

RSAVRRSTAESLFGQRLAVHSDFLGEQLQPLLDLTRRPPQVMRLQQRVNSPGWRRAMAAR

KRIDDLIDAQIADARTAPRPDDHMLTTLISGCSEEGTTLSDNEIRDSIVSLITAGYETTS

GALAWAIYALLTVPGTWESAASEVARVLGGRVPAADDLSALTYLNGVVHETLRLYSPGVI

SARRVLRDLWFDGHRIRAGRLLIFSAYVTHRLPEIWPEPTEFRPLRWDPNAADYRKPAPH

EFIPFSGGLHRCIGAVMATTEMTVILARLVARAMLQLPAQRTHRIRAANFAALRPWPGLT

VEIRKSAPAQ

>CYP139A1(2577641910)Mycobacterium tuberculosis TB_RSA70

MRYPLGEALLALYRWRGPLINAGVGGHGYTYLLGAEANRFVFANADAFSWSQTFESLVPV

DGPTALIVSDGADHRRRRSVVAPGLRHHHVQRYVATMVSNIDTVIDGWQPGQRLDIYQEL

RSAVRRSTAESLFGQRLAVHSDFLGEQLQPLLDLTRRPPQVMRLQQRVNSPGWRRAMAAR

KRIDDLIDAQIADARTAPRPDDHMLTTLISGCSEEGTTLSDNEIRDSIVSLITAGYETTS

GALAWAIYALLTVPGTWESAASEVARVLGGRVPAADDLSALTYLNGVVHETLRLYSPGVI

SARRVLRDLWFDGHRIRAGRLLIFSAYVTHRLPEIWPEPTEFRPLRWDPNAADYRKPAPH

EFIPFSGGLHRCIGAVMATTEMTVILARLVARAMLQLPAQRTHRIRAANFAALRPWPGLT

VEIRKSAPAQ

>CYP139A1(2584609065)Mycobacterium tuberculosis TBR51

MRYPLGEALLALYRWRGPLINAGVGGHGYTYLLGAEANRFVFANADAFSWSQTFESLVPV

DGPTALIVSDGADHRRRRSVVAPGLRHHHVQRYVATMVSNIDTVIDGWQPGQRLDIYQEL

RSAVRRSTAESLFGQRLAVHSDFLGEQLQPLLDLTRRPPQVMRLQQRVNSPGWRRAMAAR

KRIDDLIDAQIADARTAPRPDDHMLTTLISGCSEEGTTLSDNEIRDSIVSLITAGYETTS

GALAWAIYALLTVPGTWESAASEVARVLGGRVPAADDLSALTYLNGVVHETLRLYSPGVI

SARRVLRDLWFDGHRIRAGRLLIFSAYVTHRLPEIWPEPTEFRPLRWDPNAADYRKPAPH

EFIPFSGGLHRCIGAVMATTEMTVILARLVARAMLQLPAQRTHRIRAANFAALRPWPGLT

VEIRKSAPAQ

>CYP139A1(2584674477)Mycobacterium tuberculosis TKK_03_0040

MRYPLGEALLALYRWRGPLINAGVGGHGYTYLLGAEANRFVFANADAFSWSQTFESLVPV

DGPTALIVSDGADHRRRRSVVAPGLRHHHVQRYVATMVSNIDTVIDGWQPGQRLDIYQEL

RSAVRRSTAESLFGQRLAVHSDFLGEQLQPLLDLTRRPPQVMRLQQRVNSPGWRRAMAAR

KRIDDLIDAQIADARTAPRPDDHMLTTLISGCSEEGTTLSDNEIRDSIVSLITAGYETTS

GALAWAIYALLTVPGTWESAASEVARVLGGRVPAADDLSALTYLNGVVHETLRLYSPGVI

SARRVLRDLWFDGHRIRAGRLLIFSAYVTHRLPEIWPEPTEFRPLRWDPNAADYRKPAPH

EFIPFSGGLHRCIGAVMATTEMTVILARLVARAMLQLPAQRTHRIRAANFAALRPWPGLT

VEIRKSAPAQ

>CYP139A1(2584806904)Mycobacterium tuberculosis TB_RSA104

MRYPLGEALLALYRWRGPLINAGVGGHGYTYLLGAEANRFVFANADAFSWSQTFESLVPV

DGPTALIVSDGADHRRRRSVVAPGLRHHHVQRYVATMVSNIDTVIDGWQPGQRLDIYQEL

RSAVRRSTAESLFGQRLAVHSDFLGEQLQPLLDLTRRPPQVMRLQQRVNSPGWRRAMAAR

KRIDDLIDAQIADARTAPRPDDHMLTTLISGCSEEGTTLSDNEIRDSIVSLITAGYETTS

GALAWAIYALLTVPGTWESAASEVARVLGGRVPAADDLSALTYLNGVVHETLRLYSPGVI

SARRVLRDLWFDGHRIRAGRLLIFSAYVTHRLPEIWPEPTEFRPLRWDPNAADYRKPAPH

EFIPFSGGLHRCIGAVMATTEMTVILARLVARAMLQLPAQRTHRIRAANFAALRPWPGLT

VEIRKSAPAQ

>CYP139A1(2584849650)Mycobacterium tuberculosis TRUG0083

MRYPLGEALLALYRWRGPLINAGVGGHGYTYLLGAEANRFVFANADAFSWSQTFESLVPV

DGPTALIVSDGADHRRRRSVVAPGLRHHHVQRYVATMVSNIDTVIDGWQPGQRLDIYQEL

RSAVRRSTAESLFGQRLAVHSDFLGEQLQPLLDLTRRPPQVMRLQQRVNSPGWRRAMAAR

KRIDDLIDAQIADARTAPRPDDHMLTTLISGCSEEGTTLSDNEIRDSIVSLITAGYETTS

GALAWAIYALLTVPGTWESAASEVARVLGGRVPAADDLSALTYLNGVVHETLRLYSPGVI

SARRVLRDLWFDGHRIRAGRLLIFSAYVTHRLPEIWPEPTEFRPLRWDPNAADYRKPAPH

EFIPFSGGLHRCIGAVMATTEMTVILARLVARAMLQLPAQRTHRIRAANFAALRPWPGLT

VEIRKSAPAQ

>CYP139A1(2584854188)Mycobacterium tuberculosis TRUG0124

MRYPLGEALLALYRWRGPLINAGVGGHGYTYLLGAEANRFVFANADAFSWSQTFESLVPV

DGPTALIVSDGADHRRRRSVVAPGLRHHHVQRYVATMVSNIDTVIDGWQPGQRLDIYQEL

RSAVRRSTAESLFGQRLAVHSDFLGEQLQPLLDLTRRPPQVMRLQQRVNSPGWRRAMAAR

KRIDDLIDAQIADARTAPRPDDHMLTTLISGCSEEGTTLSDNEIRDSIVSLITAGYETTS

GALAWAIYALLTVPGTWESAASEVARVLGGRVPAADDLSALTYLNGVVHETLRLYSPGVI

SARRVLRDLWFDGHRIRAGRLLIFSAYVTHRLPEIWPEPTEFRPLRWDPNAADYRKPAPH

EFIPFSGGLHRCIGAVMATTEMTVILARLVARAMLQLPAQRTHRIRAANFAALRPWPGLT

VEIRKSAPAQ

>CYP139A1(2589100068)Mycobacterium tuberculosis TBR56

MRYPLGEALLALYRWRGPLINAGVGGHGYTYLLGAEANRFVFANADAFSWSQTFESLVPV

DGPTALIVSDGADHRRRRSVVAPGLRHHHVQRYVATMVSNIDTVIDGWQPGQRLDIYQEL

RSAVRRSTAESLFGQRLAVHSDFLGEQLQPLLDLTRRPPQVMRLQQRVNSPGWRRAMAAR

KRIDDLIDAQIADARTAPRPDDHMLTTLISGCSEEGTTLSDNEIRDSIVSLITAGYETTS

GALAWAIYALLTVPGTWESAASEVARVLGGRVPAADDLSALTYLNGVVHETLRLYSPGVI

SARRVLRDLWFDGHRIRAGRLLIFSAYVTHRLPEIWPEPTEFRPLRWDPNAADYRKPAPH

EFIPFSGGLHRCIGAVMATTEMTVILARLVARAMLQLPAQRTHRIRAANFAALRPWPGLT

VEIRKSAPAQ

>CYP139A1(2589666655)Mycobacterium tuberculosis TKK-01-0076

MRYPLGEALLALYRWRGPLINAGVGGHGYTYLLGAEANRFVFANADAFSWSQTFESLVPV

DGPTALIVSDGADHRRRRSVVAPGLRHHHVQRYVATMVSNIDTVIDGWQPGQRLDIYQEL

RSAVRRSTAESLFGQRLAVHSDFLGEQLQPLLDLTRRPPQVMRLQQRVNSPGWRRAMAAR

KRIDDLIDAQIADARTAPRPDDHMLTTLISGCSEEGTTLSDNEIRDSIVSLITAGYETTS

GALAWAIYALLTVPGTWESAASEVARVLGGRVPAADDLSALTYLNGVVHETLRLYSPGVI

SARRVLRDLWFDGHRIRAGRLLIFSAYVTHRLPEIWPEPTEFRPLRWDPNAADYRKPAPH

EFIPFSGGLHRCIGAVMATTEMTVILARLVARAMLQLPAQRTHRIRAANFAALRPWPGLT

VEIRKSAPAQ

>CYP139A1(2589703490)Mycobacterium tuberculosis TKK-01-0088

MRYPLGEALLALYRWRGPLINAGVGGHGYTYLLGAEANRFVFANADAFSWSQTFESLVPV

DGPTALIVSDGADHRRRRSVVAPGLRHHHVQRYVATMVSNIDTVIDGWQPGQRLDIYQEL

RSAVRRSTAESLFGQRLAVHSDFLGEQLQPLLDLTRRPPQVMRLQQRVNSPGWRRAMAAR

KRIDDLIDAQIADARTAPRPDDHMLTTLISGCSEEGTTLSDNEIRDSIVSLITAGYETTS

GALAWAIYALLTVPGTWESAASEVARVLGGRVPAADDLSALTYLNGVVHETLRLYSPGVI

SARRVLRDLWFDGHRIRAGRLLIFSAYVTHRLPEIWPEPTEFRPLRWDPNAADYRKPAPH

EFIPFSGGLHRCIGAVMATTEMTVILARLVARAMLQLPAQRTHRIRAANFAALRPWPGLT

VEIRKSAPAQ

>CYP139A1(2589724111)Mycobacterium tuberculosis TKK-01-0086

MRYPLGEALLALYRWRGPLINAGVGGHGYTYLLGAEANRFVFANADAFSWSQTFESLVPV

DGPTALIVSDGADHRRRRSVVAPGLRHHHVQRYVATMVSNIDTVIDGWQPGQRLDIYQEL

RSAVRRSTAESLFGQRLAVHSDFLGEQLQPLLDLTRRPPQVMRLQQRVNSPGWRRAMAAR

KRIDDLIDAQIADARTAPRPDDHMLTTLISGCSEEGTTLSDNEIRDSIVSLITAGYETTS

GALAWAIYALLTVPGTWESAASEVARVLGGRVPAADDLSALTYLNGVVHETLRLYSPGVI

SARRVLRDLWFDGHRIRAGRLLIFSAYVTHRLPEIWPEPTEFRPLRWDPNAADYRKPAPH

EFIPFSGGLHRCIGAVMATTEMTVILARLVARAMLQLPAQRTHRIRAANFAALRPWPGLT

VEIRKSAPAQ

>CYP139A1(2590117900)Mycobacterium tuberculosis MAL020157

MRYPLGEALLALYRWRGPLINAGVGGHGYTYLLGAEANRFVFANADAFSWSQTFESLVPV

DGPTALIVSDGADHRRRRSVVAPGLRHHHVQRYVATMVSNIDTVIDGWQPGQRLDIYQEL

RSAVRRSTAESLFGQRLAVHSDFLGEQLQPLLDLTRRPPQVMRLQQRVNSPGWRRAMAAR

KRIDDLIDAQIADARTAPRPDDHMLTTLISGCSEEGTTLSDNEIRDSIVSLITAGYETTS

GALAWAIYALLTVPGTWESAASEVARVLGGRVPAADDLSALTYLNGVVHETLRLYSPGVI

SARRVLRDLWFDGHRIRAGRLLIFSAYVTHRLPEIWPEPTEFRPLRWDPNAADYRKPAPH

EFIPFSGGLHRCIGAVMATTEMTVILARLVARAMLQLPAQRTHRIRAANFAALRPWPGLT

VEIRKSAPAQ

>CYP139A1(2590134192)Mycobacterium tuberculosis MAL020186

MRYPLGEALLALYRWRGPLINAGVGGHGYTYLLGAEANRFVFANADAFSWSQTFESLVPV

DGPTALIVSDGADHRRRRSVVAPGLRHHHVQRYVATMVSNIDTVIDGWQPGQRLDIYQEL

RSAVRRSTAESLFGQRLAVHSDFLGEQLQPLLDLTRRPPQVMRLQQRVNSPGWRRAMAAR

KRIDDLIDAQIADARTAPRPDDHMLTTLISGCSEEGTTLSDNEIRDSIVSLITAGYETTS

GALAWAIYALLTVPGTWESAASEVARVLGGRVPAADDLSALTYLNGVVHETLRLYSPGVI

SARRVLRDLWFDGHRIRAGRLLIFSAYVTHRLPEIWPEPTEFRPLRWDPNAADYRKPAPH

EFIPFSGGLHRCIGAVMATTEMTVILARLVARAMLQLPAQRTHRIRAANFAALRPWPGLT

VEIRKSAPAQ

>CYP139A1(2590166763)Mycobacterium tuberculosis MAL020199

MRYPLGEALLALYRWRGPLINAGVGGHGYTYLLGAEANRFVFANADAFSWSQTFESLVPV

DGPTALIVSDGADHRRRRSVVAPGLRHHHVQRYVATMVSNIDTVIDGWQPGQRLDIYQEL

RSAVRRSTAESLFGQRLAVHSDFLGEQLQPLLDLTRRPPQVMRLQQRVNSPGWRRAMAAR

KRIDDLIDAQIADARTAPRPDDHMLTTLISGCSEEGTTLSDNEIRDSIVSLITAGYETTS

GALAWAIYALLTVPGTWESAASEVARVLGGRVPAADDLSALTYLNGVVHETLRLYSPGVI

SARRVLRDLWFDGHRIRAGRLLIFSAYVTHRLPEIWPEPTEFRPLRWDPNAADYRKPAPH

EFIPFSGGLHRCIGAVMATTEMTVILARLVARAMLQLPAQRTHRIRAANFAALRPWPGLT

VEIRKSAPAQ

>CYP139A1(2590356674)Mycobacterium tuberculosis OFXR-29

MRYPLGEALLALYRWRGPLINAGVGGHGYTYLLGAEANRFVFANADAFSWSQTFESLVPV

DGPTALIVSDGADHRRRRSVVAPGLRHHHVQRYVATMVSNIDTVIDGWQPGQRLDIYQEL

RSAVRRSTAESLFGQRLAVHSDFLGEQLQPLLDLTRRPPQVMRLQQRVNSPGWRRAMAAR

KRIDDLIDAQIADARTAPRPDDHMLTTLISGCSEEGTTLSDNEIRDSIVSLITAGYETTS

GALAWAIYALLTVPGTWESAASEVARVLGGRVPAADDLSALTYLNGVVHETLRLYSPGVI

SARRVLRDLWFDGHRIRAGRLLIFSAYVTHRLPEIWPEPTEFRPLRWDPNAADYRKPAPH

EFIPFSGGLHRCIGAVMATTEMTVILARLVARAMLQLPAQRTHRIRAANFAALRPWPGLT

VEIRKSAPAQ

>CYP139A1(2549410800)Mycobacterium tuberculosis OSDD504

RGPLINAGVGGHGYTYLLGAEANRFVFANADAFSWSQTFESLVPVDGPTALIVSDGADHR

RRRSVVAPGLRHHHVQRYVATMVSNIDTVIDGWQPGQRLDIYQELRSAVRRSTAESLFGQ

RLAVHSDFLGEQLQPLLDLTRRPPQVMRLQQRVNSPGWRRAMAARKRIDDLIDAQIADAR

TAPRPDDHMLTTLISGCSEEGTTLSDNEIRDSIVSLITAGYETTSGALAWAIYALLTVPG

TWESAASEVARVLGGRVPAADDLSALTYLNGVVHETLRLYSPGVISARRVLRDLWFDGHR

IRAGRLLIFSAYVTHRLPEIWPEPTEFRPLRWDPNAADYRKPAPHEFIPFSGGLHRCIGA

VMATTEMTVILARLVARAMLQLPAQRTHRIRAANFAALRPWPGLTVEIRKSAPAQ

>CYP139A1(2575655283)Mycobacterium tuberculosis 3280CJ

MRYPLGEALLALYRWRGPLINAGVGGHGYTYLLGAEANRFVFANADAFSWSQTFESLVPV

DGPTALIVSDGADHRRRRSVVAPGLRHHHVQRYVATMVSNIDTVIDGWQPGQRLDIYQEL

RSAVRRSTAESLFGQRLAVHSDFLGEQLQPLLDLTRRPPQVMRLQQRVNSPGWRRAMAAR

KRIDDLIDAQIADARTAPRPDDHMLTTLISGCSEEGTTLSDNEIRDSIVSLITAGYETTS

GALAWAIYALLTVPGTWESAASEVARVLGGRVPAADDLSALTYLNGVVHETLRLYSPGVI

SARRVLRDLWFDGHRIRAGRLLIFSAYVTHRLPEIWPEPTEFRPLRWDPNAADYRKPAPH

EFIPFSGGLHRCIGAVMATTEMTVILARLVARAMLQLPAQRTHRIRAANFAALRPWPGLT

VEIRKSAPAQ

>CYP139A1(2576373809)Mycobacterium tuberculosis 2541MS

MRYPLGEALLALYRWRGPLINAGVGGHGYTYLLGAEANRFVFANADAFSWSQTFESLVPV

DGPTALIVSDGADHRRRRSVVAPGLRHHHVQRYVATMVSNIDTVIDGWQPGQRLDIYQEL

RSAVRRSTAESLFGQRLAVHSDFLGEQLQPLLDLTRRPPQVMRLQQRVNSPGWRRAMAAR

KRIDDLIDAQIADARTAPRPDDHMLTTLISGCSEEGTTLSDNEIRDSIVSLITAGYETTS

GALAWAIYALLTVPGTWESAASEVARVLGGRVPAADDLSALTYLNGVVHETLRLYSPGVI

SARRVLRDLWFDGHRIRAGRLLIFSAYVTHRLPEIWPEPTEFRPLRWDPNAADYRKPAPH

EFIPFSGGLHRCIGAVMATTEMTVILARLVARAMLQLPAQRTHRIRAANFAALRPWPGLT

VEIRKSAPAQ

>CYP139A1(2576609942)Mycobacterium tuberculosis BTB08-148

MRYPLGEALLALYRWRGPLINAGVGGHGYTYLLGAEANRFVFANADAFSWSQTFESLVPV

DGPTALIVSDGADHRRRRSVVAPGLRHHHVQRYVATMVSNIDTVIDGWQPGQRLDIYQEL

RSAVRRSTAESLFGQRLAVHSDFLGEQLQPLLDLTRRPPQVMRLQQRVNSPGWRRAMAAR

KRIDDLIDAQIADARTAPRPDDHMLTTLISGCSEEGTTLSDNEIRDSIVSLITAGYETTS

GALAWAIYALLTVPGTWESAASEVARVLGGRVPAADDLSALTYLNGVVHETLRLYSPGVI

SARRVLRDLWFDGHRIRAGRLLIFSAYVTHRLPEIWPEPTEFRPLRWDPNAADYRKPAPH

EFIPFSGGLHRCIGAVMATTEMTVILARLVARAMLQLPAQRTHRIRAANFAALRPWPGLT

VEIRKSAPAQ

>CYP139A1(2576684060)Mycobacterium tuberculosis BTB12-400

MRYPLGEALLALYRWRGPLINAGVGGHGYTYLLGAEANRFVFANADAFSWSQTFESLVPV

DGPTALIVSDGADHRRRRSVVAPGLRHHHVQRYVATMVSNIDTVIDGWQPGQRLDIYQEL

RSAVRRSTAESLFGQRLAVHSDFLGEQLQPLLDLTRRPPQVMRLQQRVNSPGWRRAMAAR

KRIDDLIDAQIADARTAPRPDDHMLTTLISGCSEEGTTLSDNEIRDSIVSLITAGYETTS

GALAWAIYALLTVPGTWESAASEVARVLGGRVPAADDLSALTYLNGVVHETLRLYSPGVI

SARRVLRDLWFDGHRIRAGRLLIFSAYVTHRLPEIWPEPTEFRPLRWDPNAADYRKPAPH

EFIPFSGGLHRCIGAVMATTEMTVILARLVARAMLQLPAQRTHRIRAANFAALRPWPGLT

VEIRKSAPAQ

>CYP139A1(2577110129)Mycobacterium tuberculosis KT-0024

MRYPLGEALLALYRWRGPLINAGVGGHGYTYLLGAEANRFVFANADAFSWSQTFESLVPV

DGPTALIVSDGADHRRRRSVVAPGLRHHHVQRYVATMVSNIDTVIDGWQPGQRLDIYQEL

RSAVRRSTAESLFGQRLAVHSDFLGEQLQPLLDLTRRPPQVMRLQQRVNSPGWRRAMAAR

KRIDDLIDAQIADARTAPRPDDHMLTTLISGCSEEGTTLSDNEIRDSIVSLITAGYETTS

GALAWAIYALLTVPGTWESAASEVARVLGGRVPAADDLSALTYLNGVVHETLRLYSPGVI

SARRVLRDLWFDGHRIRAGRLLIFSAYVTHRLPEIWPEPTEFRPLRWDPNAADYRKPAPH

EFIPFSGGLHRCIGAVMATTEMTVILARLVARAMLQLPAQRTHRIRAANFAALRPWPGLT

VEIRKSAPAQ

>CYP139A1(2577733463)Mycobacterium tuberculosis KT-0100

MRYPLGEALLALYRWRGPLINAGVGGHGYTYLLGAEANRFVFANADAFSWSQTFESLVPV

DGPTALIVSDGADHRRRRSVVAPGLRHHHVQRYVATMVSNIDTVIDGWQPGQRLDIYQEL

RSAVRRSTAESLFGQRLAVHSDFLGEQLQPLLDLTRRPPQVMRLQQRVNSPGWRRAMAAR

KRIDDLIDAQIADARTAPRPDDHMLTTLISGCSEEGTTLSDNEIRDSIVSLITAGYETTS

GALAWAIYALLTVPGTWESAASEVARVLGGRVPAADDLSALTYLNGVVHETLRLYSPGVI

SARRVLRDLWFDGHRIRAGRLLIFSAYVTHRLPEIWPEPTEFRPLRWDPNAADYRKPAPH

EFIPFSGGLHRCIGAVMATTEMTVILARLVARAMLQLPAQRTHRIRAANFAALRPWPGLT

VEIRKSAPAQ

>CYP139A1(2578170996)Mycobacterium tuberculosis BTB11-236

MRYPLGEALLALYRWRGPLINAGVGGHGYTYLLGAEANRFVFANADAFSWSQTFESLVPV

DGPTALIVSDGADHRRRRSVVAPGLRHHHVQRYVATMVSNIDTVIDGWQPGQRLDIYQEL

RSAVRRSTAESLFGQRLAVHSDFLGEQLQPLLDLTRRPPQVMRLQQRVNSPGWRRAMAAR

KRIDDLIDAQIADARTAPRPDDHMLTTLISGCSEEGTTLSDNEIRDSIVSLITAGYETTS

GALAWAIYALLTVPGTWESAASEVARVLGGRVPAADDLSALTYLNGVVHETLRLYSPGVI

SARRVLRDLWFDGHRIRAGRLLIFSAYVTHRLPEIWPEPTEFRPLRWDPNAADYRKPAPH

EFIPFSGGLHRCIGAVMATTEMTVILARLVARAMLQLPAQRTHRIRAANFAALRPWPGLT

VEIRKSAPAQ

>CYP139A1(2583731958)*Mycobacterium africanum* MAL020135

MRYPLGEALLALYRWRGPLINAGVGGHGYTYLLGAEANRFVFANADAFSWSQTFESLVPV

DGPTALIVSDGADHRRRRSVVAPGLRHHHVQRYVATMVSNIDTVIDGWQPGQRLDIYQEL

RSAVRRSTAESLFGQRLAVHSDFLGEQLQPLLDLTRRPPQVMRLQQRVNSPGWRRAMAAR

KRIDDLIDAQIADARTAPRPDDHMLTTLISGCSEEGTTLSDNEIRDSIVSLITAGYETTS

GALAWAIYALLTVPGTWESAASEVARVLGGRVPAADDLSALTYLNGVVHETLRLYSPGVI

SARRVLRDLWFDGHRIRAGRLLIFSAYVTHRLPEIWPEPTEFRPLRWDPNAADYRKPAPH

EFIPFSGGLHRCIGAVMATTEMTVILARLVARAMLQLPAQRTHRIRAANFAALRPWPGLT

VEIRKSAPAQ

>CYP139A1(2590523531)Mycobacterium tuberculosis KT-0089

MRYPLGEALLALYRWRGPLINAGVGGHGYTYLLGAEANRFVFANADAFSWSQTFESLVPV

DGPTALIVSDGADHRRRRSVVAPGLRHHHVQRYVATMVSNIDTVIDGWQPGQRLDIYQEL

RSAVRRSTAESLFGQRLAVHSDFLGEQLQPLLDLTRRPPQVMRLQQRVNSPGWRRAMAAR

KRIDDLIDAQIADARTAPRPDDHMLTTLISGCSEEGTTLSDNEIRDSIVSLITAGYETTS

GALAWAIYALLTVPGTWESAASEVARVLGGRVPAADDLSALTYLNGVVHETLRLYSPGVI

SARRVLRDLWFDGHRIRAGRLLIFSAYVTHRLPEIWPEPTEFRPLRWDPNAADYRKPAPH

EFIPFSGGLHRCIGAVMATTEMTVILARLVARAMLQLPAQRTHRIRAANFAALRPWPGLT

VEIRKSAPAQ

>CYP139A1(2590526411)Mycobacterium tuberculosis KT-0085

MRYPLGEALLALYRWRGPLINAGVGGHGYTYLLGAEANRFVFANADAFSWSQTFESLVPV

DGPTALIVSDGADHRRRRSVVAPGLRHHHVQRYVATMVSNIDTVIDGWQPGQRLDIYQEL

RSAVRRSTAESLFGQRLAVHSDFLGEQLQPLLDLTRRPPQVMRLQQRVNSPGWRRAMAAR

KRIDDLIDAQIADARTAPRPDDHMLTTLISGCSEEGTTLSDNEIRDSIVSLITAGYETTS

GALAWAIYALLTVPGTWESAASEVARVLGGRVPAADDLSALTYLNGVVHETLRLYSPGVI

SARRVLRDLWFDGHRIRAGRLLIFSAYVTHRLPEIWPEPTEFRPLRWDPNAADYRKPAPH

EFIPFSGGLHRCIGAVMATTEMTVILARLVARAMLQLPAQRTHRIRAANFAALRPWPGLT

VEIRKSAPAQ

>CYP139A1(2590552289)Mycobacterium tuberculosis KT-0077

MRYPLGEALLALYRWRGPLINAGVGGHGYTYLLGAEANRFVFANADAFSWSQTFESLVPV

DGPTALIVSDGADHRRRRSVVAPGLRHHHVQRYVATMVSNIDTVIDGWQPGQRLDIYQEL

RSAVRRSTAESLFGQRLAVHSDFLGEQLQPLLDLTRRPPQVMRLQQRVNSPGWRRAMAAR

KRIDDLIDAQIADARTAPRPDDHMLTTLISGCSEEGTTLSDNEIRDSIVSLITAGYETTS

GALAWAIYALLTVPGTWESAASEVARVLGGRVPAADDLSALTYLNGVVHETLRLYSPGVI

SARRVLRDLWFDGHRIRAGRLLIFSAYVTHRLPEIWPEPTEFRPLRWDPNAADYRKPAPH

EFIPFSGGLHRCIGAVMATTEMTVILARLVARAMLQLPAQRTHRIRAANFAALRPWPGLT

VEIRKSAPAQ

>CYP139A1(2575205306)Mycobacterium tuberculosis TKK-01-0090

MRYPLGEALLALYRWRGPLINAGVGGHGYTYLLGAEANRFVFANADAFSWSQTFESLVPV

DGPTALIVSDGADHRRRRSVVAPGLRHHHVQRYVATMVSNIDTVIDGWQPGQRLDIYQEL

RSAVRRSTAESLFGQRLAVHSDFLGEQLQPLLDLTRRPPQVMRLQQRVNSPGWRRAMAAR

KRIDDLIDAQIADARTAPRPDDHMLTTLISGCSEEGTTLSDNEIRDSIVSLITAGYETTS

GALAWAIYALLTVPGTWESAASEVARVLGGRVPAADDLSALTYLNGVVHETLRLYSPGVI

SARRVLRDLWFDGHRIRAGRLLIFSAYVTHRLPEIWPEPTEFRPLRWDPNAADYRKPAPH

EFIPFSGGLHRCIGAVMATTEMTVILARLVARAMLQLPAQRTHRIRAANFAALRPWPGLT

VEIRKSAPAQ

>CYP139A1(2575230562)Mycobacterium tuberculosis TBR55

MRYPLGEALLALYRWRGPLINAGVGGHGYTYLLGAEANRFVFANADAFSWSQTFESLVPV

DGPTALIVSDGADHRRRRSVVAPGLRHHHVQRYVATMVSNIDTVIDGWQPGQRLDIYQEL

RSAVRRSTAESLFGQRLAVHSDFLGEQLQPLLDLTRRPPQVMRLQQRVNSPGWRRAMAAR

KRIDDLIDAQIADARTAPRPDDHMLTTLISGCSEEGTTLSDNEIRDSIVSLITAGYETTS

GALAWAIYALLTVPGTWESAASEVARVLGGRVPAADDLSALTYLNGVVHETLRLYSPGVI

SARRVLRDLWFDGHRIRAGRLLIFSAYVTHRLPEIWPEPTEFRPLRWDPNAADYRKPAPH

EFIPFSGGLHRCIGAVMATTEMTVILARLVARAMLQLPAQRTHRIRAANFAALRPWPGLT

VEIRKSAPAQ

>CYP139A1(2575304538)Mycobacterium tuberculosis UG-C

MRYPLGEALLALYRWRGPLINAGVGGHGYTYLLGAEANRFVFANADAFSWSQTFESLVPV

DGPTALIVSDGADHRRRRSVVAPGLRHHHVQRYVATMVSNIDTVIDGWQPGQRLDIYQEL

RSAVRRSTAESLFGQRLAVHSDFLGEQLQPLLDLTRRPPQVMRLQQRVNSPGWRRAMAAR

KRIDDLIDAQIADARTAPRPDDHMLTTLISGCSEEGTTLSDNEIRDSIVSLITAGYETTS

GALAWAIYALLTVPGTWESAASEVARVLGGRVPAADDLSALTYLNGVVHETLRLYSPGVI

SARRVLRDLWFDGHRIRAGRLLIFSAYVTHRLPEIWPEPTEFRPLRWDPNAADYRKPAPH

EFIPFSGGLHRCIGAVMATTEMTVILARLVARAMLQLPAQRTHRIRAANFAALRPWPGLT

VEIRKSAPAQ

>CYP139A1(2575476916)Mycobacterium tuberculosis TB_RSA90

MRYPLGEALLALYRWRGPLINAGVGGHGYTYLLGAEANRFVFANADAFSWSQTFESLVPV

DGPTALIVSDGADHRRRRSVVAPGLRHHHVQRYVATMVSNIDTVIDGWQPGQRLDIYQEL

RSAVRRSTAESLFGQRLAVHSDFLGEQLQPLLDLTRRPPQVMRLQQRVNSPGWRRAMAAR

KRIDDLIDAQIADARTAPRPDDHMLTTLISGCSEEGTTLSDNEIRDSIVSLITAGYETTS

GALAWAIYALLTVPGTWESAASEVARVLGGRVPAADDLSALTYLNGVVHETLRLYSPGVI

SARRVLRDLWFDGHRIRAGRLLIFSAYVTHRLPEIWPEPTEFRPLRWDPNAADYRKPAPH

EFIPFSGGLHRCIGAVMATTEMTVILARLVARAMLQLPAQRTHRIRAANFAALRPWPGLT

VEIRKSAPAQ

>CYP139A1(2575790354)Mycobacterium tuberculosis TKK_05SA_0048

MRYPLGEALLALYRWRGPLINAGVGGHGYTYLLGAEANRFVFANADAFSWSQTFESLVPV

DGPTALIVSDGADHRRRRSVVAPGLRHHHVQRYVATMVSNIDTVIDGWQPGQRLDIYQEL

RSAVRRSTAESLFGQRLAVHSDFLGEQLQPLLDLTRRPPQVMRLQQRVNSPGWRRAMAAR

KRIDDLIDAQIADARTAPRPDDHMLTTLISGCSEEGTTLSDNEIRDSIVSLITAGYETTS

GALAWAIYALLTVPGTWESAASEVARVLGGRVPAADDLSALTYLNGVVHETLRLYSPGVI

SARRVLRDLWFDGHRIRAGRLLIFSAYVTHRLPEIWPEPTEFRPLRWDPNAADYRKPAPH

EFIPFSGGLHRCIGAVMATTEMTVILARLVARAMLQLPAQRTHRIRAANFAALRPWPGLT

VEIRKSAPAQ

>CYP139A1(2576077839)Mycobacterium tuberculosis TKK_04_0024

MRYPLGEALLALYRWRGPLINAGVGGHGYTYLLGAEANRFVFANADAFSWSQTFESLVPV

DGPTALIVSDGADHRRRRSVVAPGLRHHHVQRYVATMVSNIDTVIDGWQPGQRLDIYQEL

RSAVRRSTAESLFGQRLAVHSDFLGEQLQPLLDLTRRPPQVMRLQQRVNSPGWRRAMAAR

KRIDDLIDAQIADARTAPRPDDHMLTTLISGCSEEGTTLSDNEIRDSIVSLITAGYETTS

GALAWAIYALLTVPGTWESAASEVARVLGGRVPAADDLSALTYLNGVVHETLRLYSPGVI

SARRVLRDLWFDGHRIRAGRLLIFSAYVTHRLPEIWPEPTEFRPLRWDPNAADYRKPAPH

EFIPFSGGLHRCIGAVMATTEMTVILARLVARAMLQLPAQRTHRIRAANFAALRPWPGLT

VEIRKSAPAQ

>CYP139A1(2576163842)Mycobacterium tuberculosis TB_RSA140

MRYPLGEALLALYRWRGPLINAGVGGHGYTYLLGAEANRFVFANADAFSWSQTFESLVPV

DGPTALIVSDGADHRRRRSVVAPGLRHHHVQRYVATMVSNIDTVIDGWQPGQRLDIYQEL

RSAVRRSTAESLFGQRLAVHSDFLGEQLQPLLDLTRRPPQVMRLQQRVNSPGWRRAMAAR

KRIDDLIDAQIADARTAPRPDDHMLTTLISGCSEEGTTLSDNEIRDSIVSLITAGYETTS

GALAWAIYALLTVPGTWESAASEVARVLGGRVPAADDLSALTYLNGVVHETLRLYSPGVI

SARRVLRDLWFDGHRIRAGRLLIFSAYVTHRLPEIWPEPTEFRPLRWDPNAADYRKPAPH

EFIPFSGGLHRCIGAVMATTEMTVILARLVARAMLQLPAQRTHRIRAANFAALRPWPGLT

VEIRKSAPAQ

>CYP139A1(2577313382)Mycobacterium tuberculosis TRUG0085

MRYPLGEALLALYRWRGPLINAGVGGHGYTYLLGAEANRFVFANADAFSWSQTFESLVPV

DGPTALIVSDGADHRRRRSVVAPGLRHHHVQRYVATMVSNIDTVIDGWQPGQRLDIYQEL

RSAVRRSTAESLFGQRLAVHSDFLGEQLQPLLDLTRRPPQVMRLQQRVNSPGWRRAMAAR

KRIDDLIDAQIADARTAPRPDDHMLTTLISGCSEEGTTLSDNEIRDSIVSLITAGYETTS

GALAWAIYALLTVPGTWESAASEVARVLGGRVPAADDLSALTYLNGVVHETLRLYSPGVI

SARRVLRDLWFDGHRIRAGRLLIFSAYVTHRLPEIWPEPTEFRPLRWDPNAADYRKPAPH

EFIPFSGGLHRCIGAVMATTEMTVILARLVARAMLQLPAQRTHRIRAANFAALRPWPGLT

VEIRKSAPAQ

>CYP139A1(2577519304)Mycobacterium tuberculosis MD15212

MRYPLGEALLALYRWRGPLINAGVGGHGYTYLLGAEANRFVFANADAFSWSQTFESLVPV

DGPTALIVSDGADHRRRRSVVAPGLRHHHVQRYVATMVSNIDTVIDGWQPGQRLDIYQEL

RSAVRRSTAESLFGQRLAVHSDFLGEQLQPLLDLTRRPPQVMRLQQRVNSPGWRRAMAAR

KRIDDLIDAQIADARTAPRPDDHMLTTLISGCSEEGTTLSDNEIRDSIVSLITAGYETTS

GALAWAIYALLTVPGTWESAASEVARVLGGRVPAADDLSALTYLNGVVHETLRLYSPGVI

SARRVLRDLWFDGHRIRAGRLLIFSAYVTHRLPEIWPEPTEFRPLRWDPNAADYRKPAPH

EFIPFSGGLHRCIGAVMATTEMTVILARLVARAMLQLPAQRTHRIRAANFAALRPWPGLT

VEIRKSAPAQ

>CYP139A1(2577816400)Mycobacterium tuberculosis TKK_03_0031

MRYPLGEALLALYRWRGPLINAGVGGHGYTYLLGAEANRFVFANADAFSWSQTFESLVPV

DGPTALIVSDGADHRRRRSVVAPGLRHHHVQRYVATMVSNIDTVIDGWQPGQRLDIYQEL

RSAVRRSTAESLFGQRLAVHSDFLGEQLQPLLDLTRRPPQVMRLQQRVNSPGWRRAMAAR

KRIDDLIDAQIADARTAPRPDDHMLTTLISGCSEEGTTLSDNEIRDSIVSLITAGYETTS

GALAWAIYALLTVPGTWESAASEVARVLGGRVPAADDLSALTYLNGVVHETLRLYSPGVI

SARRVLRDLWFDGHRIRAGRLLIFSAYVTHRLPEIWPEPTEFRPLRWDPNAADYRKPAPH

EFIPFSGGLHRCIGAVMATTEMTVILARLVARAMLQLPAQRTHRIRAANFAALRPWPGLT

VEIRKSAPAQ

>CYP139A1(2584670398)Mycobacterium tuberculosis TKK_03_0059

MRYPLGEALLALYRWRGPLINAGVGGHGYTYLLGAEANRFVFANADAFSWSQTFESLVPV

DGPTALIVSDGADHRRRRSVVAPGLRHHHVQRYVATMVSNIDTVIDGWQPGQRLDIYQEL

RSAVRRSTAESLFGQRLAVHSDFLGEQLQPLLDLTRRPPQVMRLQQRVNSPGWRRAMAAR

KRIDDLIDAQIADARTAPRPDDHMLTTLISGCSEEGTTLSDNEIRDSIVSLITAGYETTS

GALAWAIYALLTVPGTWESAASEVARVLGGRVPAADDLSALTYLNGVVHETLRLYSPGVI

SARRVLRDLWFDGHRIRAGRLLIFSAYVTHRLPEIWPEPTEFRPLRWDPNAADYRKPAPH

EFIPFSGGLHRCIGAVMATTEMTVILARLVARAMLQLPAQRTHRIRAANFAALRPWPGLT

VEIRKSAPAQ

>CYP139A1(2584841030)Mycobacterium tuberculosis TKK_04_0060

MRYPLGEALLALYRWRGPLINAGVGGHGYTYLLGAEANRFVFANADAFSWSQTFESLVPV

DGPTALIVSDGADHRRRRSVVAPGLRHHHVQRYVATMVSNIDTVIDGWQPGQRLDIYQEL

RSAVRRSTAESLFGQRLAVHSDFLGEQLQPLLDLTRRPPQVMRLQQRVNSPGWRRAMAAR

KRIDDLIDAQIADARTAPRPDDHMLTTLISGCSEEGTTLSDNEIRDSIVSLITAGYETTS

GALAWAIYALLTVPGTWESAASEVARVLGGRVPAADDLSALTYLNGVVHETLRLYSPGVI

SARRVLRDLWFDGHRIRAGRLLIFSAYVTHRLPEIWPEPTEFRPLRWDPNAADYRKPAPH

EFIPFSGGLHRCIGAVMATTEMTVILARLVARAMLQLPAQRTHRIRAANFAALRPWPGLT

VEIRKSAPAQ

>CYP139A1(2584865918)Mycobacterium tuberculosis TB_RSA46

MRYPLGEALLALYRWRGPLINAGVGGHGYTYLLGAEANRFVFANADAFSWSQTFESLVPV

DGPTALIVSDGADHRRRRSVVAPGLRHHHVQRYVATMVSNIDTVIDGWQPGQRLDIYQEL

RSAVRRSTAESLFGQRLAVHSDFLGEQLQPLLDLTRRPPQVMRLQQRVNSPGWRRAMAAR

KRIDDLIDAQIADARTAPRPDDHMLTTLISGCSEEGTTLSDNEIRDSIVSLITAGYETTS

GALAWAIYALLTVPGTWESAASEVARVLGGRVPAADDLSALTYLNGVVHETLRLYSPGVI

SARRVLRDLWFDGHRIRAGRLLIFSAYVTHRLPEIWPEPTEFRPLRWDPNAADYRKPAPH

EFIPFSGGLHRCIGAVMATTEMTVILARLVARAMLQLPAQRTHRIRAANFAALRPWPGLT

VEIRKSAPAQ

>CYP139A1(2589588224)Mycobacterium tuberculosis TKK-01-0043

MRYPLGEALLALYRWRGPLINAGVGGHGYTYLLGAEANRFVFANADAFSWSQTFESLVPV

DGPTALIVSDGADHRRRRSVVAPGLRHHHVQRYVATMVSNIDTVIDGWQPGQRLDIYQEL

RSAVRRSTAESLFGQRLAVHSDFLGEQLQPLLDLTRRPPQVMRLQQRVNSPGWRRAMAAR

KRIDDLIDAQIADARTAPRPDDHMLTTLISGCSEEGTTLSDNEIRDSIVSLITAGYETTS

GALAWAIYALLTVPGTWESAASEVARVLGGRVPAADDLSALTYLNGVVHETLRLYSPGVI

SARRVLRDLWFDGHRIRAGRLLIFSAYVTHRLPEIWPEPTEFRPLRWDPNAADYRKPAPH

EFIPFSGGLHRCIGAVMATTEMTVILARLVARAMLQLPAQRTHRIRAANFAALRPWPGLT

VEIRKSAPAQ

>CYP139A1(2589624713)Mycobacterium tuberculosis TKK-01-0057

MRYPLGEALLALYRWRGPLINAGVGGHGYTYLLGAEANRFVFANADAFSWSQTFESLVPV

DGPTALIVSDGADHRRRRSVVAPGLRHHHVQRYVATMVSNIDTVIDGWQPGQRLDIYQEL

RSAVRRSTAESLFGQRLAVHSDFLGEQLQPLLDLTRRPPQVMRLQQRVNSPGWRRAMAAR

KRIDDLIDAQIADARTAPRPDDHMLTTLISGCSEEGTTLSDNEIRDSIVSLITAGYETTS

GALAWAIYALLTVPGTWESAASEVARVLGGRVPAADDLSALTYLNGVVHETLRLYSPGVI

SARRVLRDLWFDGHRIRAGRLLIFSAYVTHRLPEIWPEPTEFRPLRWDPNAADYRKPAPH

EFIPFSGGLHRCIGAVMATTEMTVILARLVARAMLQLPAQRTHRIRAANFAALRPWPGLT

VEIRKSAPAQ

>CYP139A1(2589736180)Mycobacterium tuberculosis TKK-01-0093

MRYPLGEALLALYRWRGPLINAGVGGHGYTYLLGAEANRFVFANADAFSWSQTFESLVPV

DGPTALIVSDGADHRRRRSVVAPGLRHHHVQRYVATMVSNIDTVIDGWQPGQRLDIYQEL

RSAVRRSTAESLFGQRLAVHSDFLGEQLQPLLDLTRRPPQVMRLQQRVNSPGWRRAMAAR

KRIDDLIDAQIADARTAPRPDDHMLTTLISGCSEEGTTLSDNEIRDSIVSLITAGYETTS

GALAWAIYALLTVPGTWESAASEVARVLGGRVPAADDLSALTYLNGVVHETLRLYSPGVI

SARRVLRDLWFDGHRIRAGRLLIFSAYVTHRLPEIWPEPTEFRPLRWDPNAADYRKPAPH

EFIPFSGGLHRCIGAVMATTEMTVILARLVARAMLQLPAQRTHRIRAANFAALRPWPGLT

VEIRKSAPAQ

>CYP139A1(2590032388)Mycobacterium tuberculosis MAL010108

MRYPLGEALLALYRWRGPLINAGVGGHGYTYLLGAEANRFVFANADAFSWSQTFESLVPV

DGPTALIVSDGADHRRRRSVVAPGLRHHHVQRYVATMVSNIDTVIDGWQPGQRLDIYQEL

RSAVRRSTAESLFGQRLAVHSDFLGEQLQPLLDLTRRPPQVMRLQQRVNSPGWRRAMAAR

KRIDDLIDAQIADARTAPRPDDHMLTTLISGCSEEGTTLSDNEIRDSIVSLITAGYETTS

GALAWAIYALLTVPGTWESAASEVARVLGGRVPAADDLSALTYLNGVVHETLRLYSPGVI

SARRVLRDLWFDGHRIRAGRLLIFSAYVTHRLPEIWPEPTEFRPLRWDPNAADYRKPAPH

EFIPFSGGLHRCIGAVMATTEMTVILARLVARAMLQLPAQRTHRIRAANFAALRPWPGLT

VEIRKSAPAQ

>CYP139A1(2590501611)Mycobacterium tuberculosis KT-0104

MRYPLGEALLALYRWRGPLINAGVGGHGYTYLLGAEANRFVFANADAFSWSQTFESLVPV

DGPTALIVSDGADHRRRRSVVAPGLRHHHVQRYVATMVSNIDTVIDGWQPGQRLDIYQEL

RSAVRRSTAESLFGQRLAVHSDFLGEQLQPLLDLTRRPPQVMRLQQRVNSPGWRRAMAAR

KRIDDLIDAQIADARTAPRPDDHMLTTLISGCSEEGTTLSDNEIRDSIVSLITAGYETTS

GALAWAIYALLTVPGTWESAASEVARVLGGRVPAADDLSALTYLNGVVHETLRLYSPGVI

SARRVLRDLWFDGHRIRAGRLLIFSAYVTHRLPEIWPEPTEFRPLRWDPNAADYRKPAPH

EFIPFSGGLHRCIGAVMATTEMTVILARLVARAMLQLPAQRTHRIRAANFAALRPWPGLT

VEIRKSAPAQ

>CYP139A1(2592312197)Mycobacterium tuberculosis TKK_03_0027

MRYPLGEALLALYRWRGPLINAGVGGHGYTYLLGAEANRFVFANADAFSWSQTFESLVPV

DGPTALIVSDGADHRRRRSVVAPGLRHHHVQRYVATMVSNIDTVIDGWQPGQRLDIYQEL

RSAVRRSTAESLFGQRLAVHSDFLGEQLQPLLDLTRRPPQVMRLQQRVNSPGWRRAMAAR

KRIDDLIDAQIADARTAPRPDDHMLTTLISGCSEEGTTLSDNEIRDSIVSLITAGYETTS

GALAWAIYALLTVPGTWESAASEVARVLGGRVPAADDLSALTYLNGVVHETLRLYSPGVI

SARRVLRDLWFDGHRIRAGRLLIFSAYVTHRLPEIWPEPTEFRPLRWDPNAADYRKPAPH

EFIPFSGGLHRCIGAVMATTEMTVILARLVARAMLQLPAQRTHRIRAANFAALRPWPGLT

VEIRKSAPAQ

>CYP139A1(2592435284)Mycobacterium tuberculosis TKK_02_0006

MRYPLGEALLALYRWRGPLINAGVGGHGYTYLLGAEANRFVFANADAFSWSQTFESLVPV

DGPTALIVSDGADHRRRRSVVAPGLRHHHVQRYVATMVSNIDTVIDGWQPGQRLDIYQEL

RSAVRRSTAESLFGQRLAVHSDFLGEQLQPLLDLTRRPPQVMRLQQRVNSPGWRRAMAAR

KRIDDLIDAQIADARTAPRPDDHMLTTLISGCSEEGTTLSDNEIRDSIVSLITAGYETTS

GALAWAIYALLTVPGTWESAASEVARVLGGRVPAADDLSALTYLNGVVHETLRLYSPGVI

SARRVLRDLWFDGHRIRAGRLLIFSAYVTHRLPEIWPEPTEFRPLRWDPNAADYRKPAPH

EFIPFSGGLHRCIGAVMATTEMTVILARLVARAMLQLPAQRTHRIRAANFAALRPWPGLT

VEIRKSAPAQ

>CYP139A1(2511811274)*Mycobacterium bovis*BCG Mexico

MRYPLGEALLALYRWRGPLINAGVGGHGYTYLLGAEANRFVFANADAFSWSQTFESLVPV

DGPTALIVSDGADHRRRRSVVAPGLRHHHVQRYVATMVSNIDTVIDGWQPGQRLDIYQEL

RSAVRRSTAESLFGQRLAVHSDFLGEQLQPLLDLTRRPPQVMRLQQRVNSPGWRRAMAAR

KRIDDLIDAQIADARTAPRPDDHMLTTLISGCSEEGTTLSDNEIRDSIVSLITAGYETTS

GALAWAIYALLTVPGTWESAASEVARVLGGRVPAADDLSALTYLNGVVHETLRLYSPGVI

SARRVLRDLWFDGHRIRAGRLLIFSAYVTHRLPEIWPEPTEFRPLRWDPNAADYRKPAPH

EFIPFSGGLHRCIGAVMATTEMTVILARLVARAMLQLPAQRTHRIRAANFAALRPWPGLT

VEIRKSAPAQ

>CYP139A1(2546202077)Mycobacterium tuberculosis CAS/NITR204

MRYPLGEALLALYRWRGPLINAGVGGHGYTYLLGAEANRFVFANADAFSWSQTFESLVPV

DGPTALIVSDGADHRRRRSVVAPGLRHHHVQRYVATMVSNIDTVIDGWQPGQRLDIYQEL

RSAVRRSTAESLFGQRLAVHSDFLGEQLQPLLDLTRRPPQVMRLQQRVNSPGWRRAMAAR

KRIDDLIDAQIADARTAPRPDDHMLTTLISGCSEEGTTLSDNEIRDSIVSLITAGYETTS

GALAWAIYALLTVPGTWESAASEVARVLGGRVPAADDLSALTYLNGVVHETLRLYSPGVI

SARRVLRDLWFDGHRIRAGRLLIFSAYVTHRLPEIWPEPTEFRPLRWDPNAADYRKPAPH

EFIPFSGGLHRCIGAVMATTEMTVILARLVARAMLQLPAQRTHRIRAANFAALRPWPGLT

VEIRKSAPAQ

>CYP139A1(2574693694)Mycobacterium tuberculosis BTB07-206

MRYPLGEALLALYRWRGPLINAGVGGHGYTYLLGAEANRFVFANADAFSWSQTFESLVPV

DGPTALIVSDGADHRRRRSVVAPGLRHHHVQRYVATMVSNIDTVIDGWQPGQRLDIYQEL

RSAVRRSTAESLFGQRLAVHSDFLGEQLQPLLDLTRRPPQVMRLQQRVNSPGWRRAMAAR

KRIDDLIDAQIADARTAPRPDDHMLTTLISGCSEEGTTLSDNEIRDSIVSLITAGYETTS

GALAWAIYALLTVPGTWESAASEVARVLGGRVPAADDLSALTYLNGVVHETLRLYSPGVI

SARRVLRDLWFDGHRIRAGRLLIFSAYVTHRLPEIWPEPTEFRPLRWDPNAADYRKPAPH

EFIPFSGGLHRCIGAVMATTEMTVILARLVARAMLQLPAQRTHRIRAANFAALRPWPGLT

VEIRKSAPAQ

>CYP139A1(2574968392)*Mycobacterium africanum* MAL010100

MRYPLGEALLALYRWRGPLINAGVGGHGYTYLLGAEANRFVFANADAFSWSQTFESLVPV

DGPTALIVSDGADHRRRRSVVAPGLRHHHVQRYVATMVSNIDTVIDGWQPGQRLDIYQEL

RSAVRRSTAESLFGQRLAVHSDFLGEQLQPLLDLTRRPPQVMRLQQRVNSPGWRRAMAAR

KRIDDLIDAQIADARTAPRPDDHMLTTLISGCSEEGTTLSDNEIRDSIVSLITAGYETTS

GALAWAIYALLTVPGTWESAASEVARVLGGRVPAADDLSALTYLNGVVHETLRLYSPGVI

SARRVLRDLWFDGHRIRAGRLLIFSAYVTHRLPEIWPEPTEFRPLRWDPNAADYRKPAPH

EFIPFSGGLHRCIGAVMATTEMTVILARLVARAMLQLPAQRTHRIRAANFAALRPWPGLT

VEIRKSAPAQ

>CYP139A1(2577060340)Mycobacterium tuberculosis BTB12-314

MRYPLGEALLALYRWRGPLINAGVGGHGYTYLLGAEANRFVFANADAFSWSQTFESLVPV

DGPTALIVSDGADHRRRRSVVAPGLRHHHVQRYVATMVSNIDTVIDGWQPGQRLDIYQEL

RSAVRRSTAESLFGQRLAVHSDFLGEQLQPLLDLTRRPPQVMRLQQRVNSPGWRRAMAAR

KRIDDLIDAQIADARTAPRPDDHMLTTLISGCSEEGTTLSDNEIRDSIVSLITAGYETTS

GALAWAIYALLTVPGTWESAASEVARVLGGRVPAADDLSALTYLNGVVHETLRLYSPGVI

SARRVLRDLWFDGHRIRAGRLLIFSAYVTHRLPEIWPEPTEFRPLRWDPNAADYRKPAPH

EFIPFSGGLHRCIGAVMATTEMTVILARLVARAMLQLPAQRTHRIRAANFAALRPWPGLT

VEIRKSAPAQ

>CYP139A1(2577454945)Mycobacterium tuberculosis BTB07-034

MRYPLGEALLALYRWRGPLINAGVGGHGYTYLLGAEANRFVFANADAFSWSQTFESLVPV

DGPTALIVSDGADHRRRRSVVAPGLRHHHVQRYVATMVSNIDTVIDGWQPGQRLDIYQEL

RSAVRRSTAESLFGQRLAVHSDFLGEQLQPLLDLTRRPPQVMRLQQRVNSPGWRRAMAAR

KRIDDLIDAQIADARTAPRPDDHMLTTLISGCSEEGTTLSDNEIRDSIVSLITAGYETTS

GALAWAIYALLTVPGTWESAASEVARVLGGRVPAADDLSALTYLNGVVHETLRLYSPGVI

SARRVLRDLWFDGHRIRAGRLLIFSAYVTHRLPEIWPEPTEFRPLRWDPNAADYRKPAPH

EFIPFSGGLHRCIGAVMATTEMTVILARLVARAMLQLPAQRTHRIRAANFAALRPWPGLT

VEIRKSAPAQ

>CYP139A1(2580123929)*Mycobacterium bovis*Kc 9614

MRYPLGEALLALYRWRGPLINAGVGGHGYTYLLGAEANRFVFANADAFSWSQTFESLVPV

DGPTALIVSDGADHRRRRSVVAPGLRHHHVQRYVATMVSNIDTVIDGWQPGQRLDIYQEL

RSAVRRSTAESLFGQRLAVHSDFLGEQLQPLLDLTRRPPQVMRLQQRVNSPGWRRAMAAR

KRIDDLIDAQIADARTAPRPDDHMLTTLISGCSEEGTTLSDNEIRDSIVSLITAGYETTS

GALAWAIYALLTVPGTWESAASEVARVLGGRVPAADDLSALTYLNGVVHETLRLYSPGVI

SARRVLRDLWFDGHRIRAGRLLIFSAYVTHRLPEIWPEPTEFRPLRWDPNAADYRKPAPH

EFIPFSGGLHRCIGAVMATTEMTVILARLVARAMLQLPAQRTHRIRAANFAALRPWPGLT

VEIRKSAPAQ

>CYP139A1(2581930746)*Mycobacterium africanum* MAL020107

MRYPLGEALLALYRWRGPLINAGVGGHGYTYLLGAEANRFVFANADAFSWSQTFESLVPV

DGPTALIVSDGADHRRRRSVVAPGLRHHHVQRYVATMVSNIDTVIDGWQPGQRLDIYQEL

RSAVRRSTAESLFGQRLAVHSDFLGEQLQPLLDLTRRPPQVMRLQQRVNSPGWRRAMAAR

KRIDDLIDAQIADARTAPRPDDHMLTTLISGCSEEGTTLSDNEIRDSIVSLITAGYETTS

GALAWAIYALLTVPGTWESAASEVARVLGGRVPAADDLSALTYLNGVVHETLRLYSPGVI

SARRVLRDLWFDGHRIRAGRLLIFSAYVTHRLPEIWPEPTEFRPLRWDPNAADYRKPAPH

EFIPFSGGLHRCIGAVMATTEMTVILARLVARAMLQLPAQRTHRIRAANFAALRPWPGLT

VEIRKSAPAQ

>CYP139A1(2584196432)*Mycobacterium africanum* K85

MRYPLGEALLALYRWRGPLINAGVGGHGYTYLLGAEANRFVFANADAFSWSQTFESLVPV

DGPTALIVSDGADHRRRRSVVAPGLRHHHVQRYVATMVSNIDTVIDGWQPGQRLDIYQEL

RSAVRRSTAESLFGQRLAVHSDFLGEQLQPLLDLTRRPPQVMRLQQRVNSPGWRRAMAAR

KRIDDLIDAQIADARTAPRPDDHMLTTLISGCSEEGTTLSDNEIRDSIVSLITAGYETTS

GALAWAIYALLTVPGTWESAASEVARVLGGRVPAADDLSALTYLNGVVHETLRLYSPGVI

SARRVLRDLWFDGHRIRAGRLLIFSAYVTHRLPEIWPEPTEFRPLRWDPNAADYRKPAPH

EFIPFSGGLHRCIGAVMATTEMTVILARLVARAMLQLPAQRTHRIRAANFAALRPWPGLT

VEIRKSAPAQ

>CYP139A1(2584726832)Mycobacterium tuberculosis 2091HD

MRYPLGEALLALYRWRGPLINAGVGGHGYTYLLGAEANRFVFANADAFSWSQTFESLVPV

DGPTALIVSDGADHRRRRSVVAPGLRHHHVQRYVATMVSNIDTVIDGWQPGQRLDIYQEL

RSAVRRSTAESLFGQRLAVHSDFLGEQLQPLLDLTRRPPQVMRLQQRVNSPGWRRAMAAR

KRIDDLIDAQIADARTAPRPDDHMLTTLISGCSEEGTTLSDNEIRDSIVSLITAGYETTS

GALAWAIYALLTVPGTWESAASEVARVLGGRVPAADDLSALTYLNGVVHETLRLYSPGVI

SARRVLRDLWFDGHRIRAGRLLIFSAYVTHRLPEIWPEPTEFRPLRWDPNAADYRKPAPH

EFIPFSGGLHRCIGAVMATTEMTVILARLVARAMLQLPAQRTHRIRAANFAALRPWPGLT

VEIRKSAPAQ

>CYP139A1(2584911080)Mycobacterium tuberculosis KT-0011

MRYPLGEALLALYRWRGPLINAGVGGHGYTYLLGAEANRFVFANADAFSWSQTFESLVPV

DGPTALIVSDGADHRRRRSVVAPGLRHHHVQRYVATMVSNIDTVIDGWQPGQRLDIYQEL

RSAVRRSTAESLFGQRLAVHSDFLGEQLQPLLDLTRRPPQVMRLQQRVNSPGWRRAMAAR

KRIDDLIDAQIADARTAPRPDDHMLTTLISGCSEEGTTLSDNEIRDSIVSLITAGYETTS

GALAWAIYALLTVPGTWESAASEVARVLGGRVPAADDLSALTYLNGVVHETLRLYSPGVI

SARRVLRDLWFDGHRIRAGRLLIFSAYVTHRLPEIWPEPTEFRPLRWDPNAADYRKPAPH

EFIPFSGGLHRCIGAVMATTEMTVILARLVARAMLQLPAQRTHRIRAANFAALRPWPGLT

VEIRKSAPAQ

>CYP139A1(2584962435)Mycobacterium tuberculosis H2438

MRYPLGEALLALYRWRGPLINAGVGGHGYTYLLGAEANRFVFANADAFSWSQTFESLVPV

DGPTALIVSDGADHRRRRSVVAPGLRHHHVQRYVATMVSNIDTVIDGWQPGQRLDIYQEL

RSAVRRSTAESLFGQRLAVHSDFLGEQLQPLLDLTRRPPQVMRLQQRVNSPGWRRAMAAR

KRIDDLIDAQIADARTAPRPDDHMLTTLISGCSEEGTTLSDNEIRDSIVSLITAGYETTS

GALAWAIYALLTVPGTWESAASEVARVLGGRVPAADDLSALTYLNGVVHETLRLYSPGVI

SARRVLRDLWFDGHRIRAGRLLIFSAYVTHRLPEIWPEPTEFRPLRWDPNAADYRKPAPH

EFIPFSGGLHRCIGAVMATTEMTVILARLVARAMLQLPAQRTHRIRAANFAALRPWPGLT

VEIRKSAPAQ

>CYP139A1(2584995097)Mycobacterium tuberculosis M2129

MRYPLGEALLALYRWRGPLINAGVGGHGYTYLLGAEANRFVFANADAFSWSQTFESLVPV

DGPTALIVSDGADHRRRRSVVAPGLRHHHVQRYVATMVSNIDTVIDGWQPGQRLDIYQEL

RSAVRRSTAESLFGQRLAVHSDFLGEQLQPLLDLTRRPPQVMRLQQRVNSPGWRRAMAAR

KRIDDLIDAQIADARTAPRPDDHMLTTLISGCSEEGTTLSDNEIRDSIVSLITAGYETTS

GALAWAIYALLTVPGTWESAASEVARVLGGRVPAADDLSALTYLNGVVHETLRLYSPGVI

SARRVLRDLWFDGHRIRAGRLLIFSAYVTHRLPEIWPEPTEFRPLRWDPNAADYRKPAPH

EFIPFSGGLHRCIGAVMATTEMTVILARLVARAMLQLPAQRTHRIRAANFAALRPWPGLT

VEIRKSAPAQ

>CYP139A1(640602381)Mycobacterium tuberculosis H37Ra

MRYPLGEALLALYRWRGPLINAGVGGHGYTYLLGAEANRFVFANADAFSWSQTFESLVPV

DGPTALIVSDGADHRRRRSVVAPGLRHHHVQRYVATMVSNIDTVIDGWQPGQRLDIYQEL

RSAVRRSTAESLFGQRLAVHSDFLGEQLQPLLDLTRRPPQVMRLQQRVNSPGWRRAMAAR

KRIDDLIDAQIADARTAPRPDDHMLTTLISGCSEEGTTLSDNEIRDSIVSLITAGYETTS

GALAWAIYALLTVPGTWESAASEVARVLGGRVPAADDLSALTYLNGVVHETLRLYSPGVI

SARRVLRDLWFDGHRIRAGRLLIFSAYVTHRLPEIWPEPTEFRPLRWDPNAADYRKPAPH

EFIPFSGGLHRCIGAVMATTEMTVILARLVARAMLQLPAQRTHRIRAANFAALRPWPGLT

VEIRKSAPAQ

>CYP139A1(643019022)Mycobacterium tuberculosis 94_M4241A

MRYPLGEALLALYRWRGPLINAGVGGHGYTYLLGAEANRFVFANADAFSWSQTFESLVPV

DGPTALIVSDGADHRRRRSVVAPGLRHHHVQRYVATMVSNIDTVIDGWQPGQRLDIYQEL

RSAVRRSTAESLFGQRLAVHSDFLGEQLQPLLDLTRRPPQVMRLQQRVNSPGWRRAMAAR

KRIDDLIDAQIADARTAPRPDDHMLTTLISGCSEEGTTLSDNEIRDSIVSLITAGYETTS

GALAWAIYALLTVPGTWESAASEVARVLGGRVPAADDLSALTYLNGVVHETLRLYSPGVI

SARRVLRDLWFDGHRIRAGRLLIFSAYVTHRLPEIWPEPTEFRPLRWDPNAADYRKPAPH

EFIPFSGGLHRCIGAVMATTEMTVILARLVARAMLQLPAQRTHRIRAANFAALRPWPGLT

VEIRKSAPAQ

>CYP139A1(2573562450)Mycobacterium tuberculosis INS_XDR

MRYPLGEALLALYRWRGPLINAGVGGHGYTYLLGAEANRFVFANADAFSWSQTFESLVPV

DGPTALIVSDGADHRRRRSVVAPGLRHHHVQRYVATMVSNIDTVIDGWQPGQRLDIYQEL

RSAVRRSTAESLFGQRLAVHSDFLGEQLQPLLDLTRRPPQVMRLQQRVNSPGWRRAMAAR

KRIDDLIDAQIADARTAPRPDDHMLTTLISGCSEEGTTLSDNEIRDSIVSLITAGYETTS

GALAWAIYALLTVPGTWESAASEVARVLGGRVPAADDLSALTYLNGVVHETLRLYSPGVI

SARRVLRDLWFDGHRIRAGRLLIFSAYVTHRLPEIWPEPTEFRPLRWDPNAADYRKPAPH

EFIPFSGGLHRCIGAVMATTEMTVILARLVARAMLQLPAQRTHRIRAANFAALRPWPGLT

VEIRKSAPAQ

>CYP139A1(2575255926)Mycobacterium tuberculosis KT-0004

MRYPLGEALLALYRWRGPLINAGVGGHGYTYLLGAEANRFVFANADAFSWSQTFESLVPV

DGPTALIVSDGADHRRRRSVVAPGLRHHHVQRYVATMVSNIDTVIDGWQPGQRLDIYQEL

RSAVRRSTAESLFGQRLAVHSDFLGEQLQPLLDLTRRPPQVMRLQQRVNSPGWRRAMAAR

KRIDDLIDAQIADARTAPRPDDHMLTTLISGCSEEGTTLSDNEIRDSIVSLITAGYETTS

GALAWAIYALLTVPGTWESAASEVARVLGGRVPAADDLSALTYLNGVVHETLRLYSPGVI

SARRVLRDLWFDGHRIRAGRLLIFSAYVTHRLPEIWPEPTEFRPLRWDPNAADYRKPAPH

EFIPFSGGLHRCIGAVMATTEMTVILARLVARAMLQLPAQRTHRIRAANFAALRPWPGLT

VEIRKSAPAQ

>CYP139A1(2577905307)Mycobacterium tuberculosis M2085

MRYPLGEALLALYRWRGPLINAGVGGHGYTYLLGAEANRFVFANADAFSWSQTFESLVPV

DGPTALIVSDGADHRRRRSVVAPGLRHHHVQRYVATMVSNIDTVIDGWQPGQRLDIYQEL

RSAVRRSTAESLFGQRLAVHSDFLGEQLQPLLDLTRRPPQVMRLQQRVNSPGWRRAMAAR

KRIDDLIDAQIADARTAPRPDDHMLTTLISGCSEEGTTLSDNEIRDSIVSLITAGYETTS

GALAWAIYALLTVPGTWESAASEVARVLGGRVPAADDLSALTYLNGVVHETLRLYSPGVI

SARRVLRDLWFDGHRIRAGRLLIFSAYVTHRLPEIWPEPTEFRPLRWDPNAADYRKPAPH

EFIPFSGGLHRCIGAVMATTEMTVILARLVARAMLQLPAQRTHRIRAANFAALRPWPGLT

VEIRKSAPAQ

>CYP139A1(2579818167)Mycobacterium tuberculosis M1787

MRYPLGEALLALYRWRGPLINAGVGGHGYTYLLGAEANRFVFANADAFSWSQTFESLVPV

DGPTALIVSDGADHRRRRSVVAPGLRHHHVQRYVATMVSNIDTVIDGWQPGQRLDIYQEL

RSAVRRSTAESLFGQRLAVHSDFLGEQLQPLLDLTRRPPQVMRLQQRVNSPGWRRAMAAR

KRIDDLIDAQIADARTAPRPDDHMLTTLISGCSEEGTTLSDNEIRDSIVSLITAGYETTS

GALAWAIYALLTVPGTWESAASEVARVLGGRVPAADDLSALTYLNGVVHETLRLYSPGVI

SARRVLRDLWFDGHRIRAGRLLIFSAYVTHRLPEIWPEPTEFRPLRWDPNAADYRKPAPH

EFIPFSGGLHRCIGAVMATTEMTVILARLVARAMLQLPAQRTHRIRAANFAALRPWPGLT

VEIRKSAPAQ

>CYP139A1(2582001898)*Mycobacterium africanum* MAL010128

MRYPLGEALLALYRWRGPLINAGVGGHGYTYLLGAEANRFVFANADAFSWSQTFESLVPV

DGPTALIVSDGADHRRRRSVVAPGLRHHHVQRYVATMVSNIDTVIDGWQPGQRLDIYQEL

RSAVRRSTAESLFGQRLAVHSDFLGEQLQPLLDLTRRPPQVMRLQQRVNSPGWRRAMAAR

KRIDDLIDAQIADARTAPRPDDHMLTTLISGCSEEGTTLSDNEIRDSIVSLITAGYETTS

GALAWAIYALLTVPGTWESAASEVARVLGGRVPAADDLSALTYLNGVVHETLRLYSPGVI

SARRVLRDLWFDGHRIRAGRLLIFSAYVTHRLPEIWPEPTEFRPLRWDPNAADYRKPAPH

EFIPFSGGLHRCIGAVMATTEMTVILARLVARAMLQLPAQRTHRIRAANFAALRPWPGLT

VEIRKSAPAQ

>CYP139A1(2588538927)Mycobacterium tuberculosis K

MRYPLGEALLALYRWRGPLINAGVGGHGYTYLLGAEANRFVFANADAFSWSQTFESLVPV

DGPTALIVSDGADHRRRRSVVAPGLRHHHVQRYVATMVSNIDTVIDGWQPGQRLDIYQEL

RSAVRRSTAESLFGQRLAVHSDFLGEQLQPLLDLTRRPPQVMRLQQRVNSPGWRRAMAAR

KRIDDLIDAQIADARTAPRPDDHMLTTLISGCSEEGTTLSDNEIRDSIVSLITAGYETTS

GALAWAIYALLTVPGTWESAASEVARVLGGRVPAADDLSALTYLNGVVHETLRLYSPGVI

SARRVLRDLWFDGHRIRAGRLLIFSAYVTHRLPEIWPEPTEFRPLRWDPNAADYRKPAPH

EFIPFSGGLHRCIGAVMATTEMTVILARLVARAMLQLPAQRTHRIRAANFAALRPWPGLT

VEIRKSAPAQ

>CYP139A1(2590073317)Mycobacterium tuberculosis MAL020102

MRYPLGEALLALYRWRGPLINAGVGGHGYTYLLGAEANRFVFANADAFSWSQTFESLVPV

DGPTALIVSDGADHRRRRSVVAPGLRHHHVQRYVATMVSNIDTVIDGWQPGQRLDIYQEL

RSAVRRSTAESLFGQRLAVHSDFLGEQLQPLLDLTRRPPQVMRLQQRVNSPGWRRAMAAR

KRIDDLIDAQIADARTAPRPDDHMLTTLISGCSEEGTTLSDNEIRDSIVSLITAGYETTS

GALAWAIYALLTVPGTWESAASEVARVLGGRVPAADDLSALTYLNGVVHETLRLYSPGVI

SARRVLRDLWFDGHRIRAGRLLIFSAYVTHRLPEIWPEPTEFRPLRWDPNAADYRKPAPH

EFIPFSGGLHRCIGAVMATTEMTVILARLVARAMLQLPAQRTHRIRAANFAALRPWPGLT

VEIRKSAPAQ

>CYP139A1(2590075971)Mycobacterium tuberculosis MAL010134

MRYPLGEALLALYRWRGPLINAGVGGHGYTYLLGAEANRFVFANADAFSWSQTFESLVPV

DGPTALIVSDGADHRRRRSVVAPGLRHHHVQRYVATMVSNIDTVIDGWQPGQRLDIYQEL

RSAVRRSTAESLFGQRLAVHSDFLGEQLQPLLDLTRRPPQVMRLQQRVNSPGWRRAMAAR

KRIDDLIDAQIADARTAPRPDDHMLTTLISGCSEEGTTLSDNEIRDSIVSLITAGYETTS

GALAWAIYALLTVPGTWESAASEVARVLGGRVPAADDLSALTYLNGVVHETLRLYSPGVI

SARRVLRDLWFDGHRIRAGRLLIFSAYVTHRLPEIWPEPTEFRPLRWDPNAADYRKPAPH

EFIPFSGGLHRCIGAVMATTEMTVILARLVARAMLQLPAQRTHRIRAANFAALRPWPGLT

VEIRKSAPAQ

>CYP139A1(2590211264)Mycobacterium tuberculosis KT-0063

MRYPLGEALLALYRWRGPLINAGVGGHGYTYLLGAEANRFVFANADAFSWSQTFESLVPV

DGPTALIVSDGADHRRRRSVVAPGLRHHHVQRYVATMVSNIDTVIDGWQPGQRLDIYQEL

RSAVRRSTAESLFGQRLAVHSDFLGEQLQPLLDLTRRPPQVMRLQQRVNSPGWRRAMAAR

KRIDDLIDAQIADARTAPRPDDHMLTTLISGCSEEGTTLSDNEIRDSIVSLITAGYETTS

GALAWAIYALLTVPGTWESAASEVARVLGGRVPAADDLSALTYLNGVVHETLRLYSPGVI

SARRVLRDLWFDGHRIRAGRLLIFSAYVTHRLPEIWPEPTEFRPLRWDPNAADYRKPAPH

EFIPFSGGLHRCIGAVMATTEMTVILARLVARAMLQLPAQRTHRIRAANFAALRPWPGLT

VEIRKSAPAQ

>CYP139A1(2590249475)Mycobacterium tuberculosis KT-0028

MRYPLGEALLALYRWRGPLINAGVGGHGYTYLLGAEANRFVFANADAFSWSQTFESLVPV

DGPTALIVSDGADHRRRRSVVAPGLRHHHVQRYVATMVSNIDTVIDGWQPGQRLDIYQEL

RSAVRRSTAESLFGQRLAVHSDFLGEQLQPLLDLTRRPPQVMRLQQRVNSPGWRRAMAAR

KRIDDLIDAQIADARTAPRPDDHMLTTLISGCSEEGTTLSDNEIRDSIVSLITAGYETTS

GALAWAIYALLTVPGTWESAASEVARVLGGRVPAADDLSALTYLNGVVHETLRLYSPGVI

SARRVLRDLWFDGHRIRAGRLLIFSAYVTHRLPEIWPEPTEFRPLRWDPNAADYRKPAPH

EFIPFSGGLHRCIGAVMATTEMTVILARLVARAMLQLPAQRTHRIRAANFAALRPWPGLT

VEIRKSAPAQ

>CYP139A1(2590279314)Mycobacterium tuberculosis KT-0008

MRYPLGEALLALYRWRGPLINAGVGGHGYTYLLGAEANRFVFANADAFSWSQTFESLVPV

DGPTALIVSDGADHRRRRSVVAPGLRHHHVQRYVATMVSNIDTVIDGWQPGQRLDIYQEL

RSAVRRSTAESLFGQRLAVHSDFLGEQLQPLLDLTRRPPQVMRLQQRVNSPGWRRAMAAR

KRIDDLIDAQIADARTAPRPDDHMLTTLISGCSEEGTTLSDNEIRDSIVSLITAGYETTS

GALAWAIYALLTVPGTWESAASEVARVLGGRVPAADDLSALTYLNGVVHETLRLYSPGVI

SARRVLRDLWFDGHRIRAGRLLIFSAYVTHRLPEIWPEPTEFRPLRWDPNAADYRKPAPH

EFIPFSGGLHRCIGAVMATTEMTVILARLVARAMLQLPAQRTHRIRAANFAALRPWPGLT

VEIRKSAPAQ

>CYP139A1(2590559932)Mycobacterium tuberculosis KT-0071

MRYPLGEALLALYRWRGPLINAGVGGHGYTYLLGAEANRFVFANADAFSWSQTFESLVPV

DGPTALIVSDGADHRRRRSVVAPGLRHHHVQRYVATMVSNIDTVIDGWQPGQRLDIYQEL

RSAVRRSTAESLFGQRLAVHSDFLGEQLQPLLDLTRRPPQVMRLQQRVNSPGWRRAMAAR

KRIDDLIDAQIADARTAPRPDDHMLTTLISGCSEEGTTLSDNEIRDSIVSLITAGYETTS

GALAWAIYALLTVPGTWESAASEVARVLGGRVPAADDLSALTYLNGVVHETLRLYSPGVI

SARRVLRDLWFDGHRIRAGRLLIFSAYVTHRLPEIWPEPTEFRPLRWDPNAADYRKPAPH

EFIPFSGGLHRCIGAVMATTEMTVILARLVARAMLQLPAQRTHRIRAANFAALRPWPGLT

VEIRKSAPAQ

>CYP139A1(640606444)Mycobacterium tuberculosis F11

MRYPLGEALLALYRWRGPLINAGVGGHGYTYLLGAEANRFVFANADAFSWSQTFESLVPV

DGPTALIVSDGADHRRRRSVVAPGLRHHHVQRYVATMVSNIDTVIDGWQPGQRLDIYQEL

RSAVRRSTAESLFGQRLAVHSDFLGEQLQPLLDLTRRPPQVMRLQQRVNSPGWRRAMAAR

KRIDDLIDAQIADARTAPRPDDHMLTTLISGCSEEGTTLSDNEIRDSIVSLITAGYETTS

GALAWAIYALLTVPGTWESAASEVARVLGGRVPAADDLSALTYLNGVVHETLRLYSPGVI

SARRVLRDLWFDGHRIRAGRLLIFSAYVTHRLPEIWPEPTEFRPLRWDPNAADYRKPAPH

EFIPFSGGLHRCIGAVMATTEMTVILARLVARAMLQLPAQRTHRIRAANFAALRPWPGLT

VEIRKSAPAQ

>CYP139A1(651025167)*Mycobacterium africanum* GM041182

MRYPLGEALLALYRWRGPLINAGVGGHGYTYLLGAEANRFVFANADAFSWSQTFESLVPV

DGPTALIVSDGADHRRRRSVVAPGLRHHHVQRYVATMVSNIDTVIDGWQPGQRLDIYQEL

RSAVRRSTAESLFGQRLAVHSDFLGEQLQPLLDLTRRPPQVMRLQQRVNSPGWRRAMAAR

KRIDDLIDAQIADARTAPRPDDHMLTTLISGCSEEGTTLSDNEIRDSIVSLITAGYETTS

GALAWAIYALLTVPGTWESAASEVARVLGGRVPAADDLSALTYLNGVVHETLRLYSPGVI

SARRVLRDLWFDGHRIRAGRLLIFSAYVTHRLPEIWPEPTEFRPLRWDPNAADYRKPAPH

EFIPFSGGLHRCIGAVMATTEMTVILARLVARAMLQLPAQRTHRIRAANFAALRPWPGLT

VEIRKSAPAQ

>CYP139A1(2574876573)Mycobacterium tuberculosis TBR58

MRYPLGEALLALYRWRGPLINAGVGGHGYTYLLGAEANRFVFANADAFSWSQTFESLVPV

DGPTALIVSDGADHRRRRSVVAPGLRHHHVQRYVATMVSNIDTVIDGWQPGQRLDIYQEL

RSAVRRSTAESLFGQRLAVHSDFLGEQLQPLLDLTRRPPQVMRLQQRVNSPGWRRAMAAR

KRIDDLIDAQIADARTAPRPDDHMLTTLISGCSEEGTTLSDNEIRDSIVSLITAGYETTS

GALAWAIYALLTVPGTWESAASEVARVLGGRVPAADDLSALTYLNGVVHETLRLYSPGVI

SARRVLRDLWFDGHRIRAGRLLIFSAYVTHRLPEIWPEPTEFRPLRWDPNAADYRKPAPH

EFIPFSGGLHRCIGAVMATTEMTVILARLVARAMLQLPAQRTHRIRAANFAALRPWPGLT

VEIRKSAPAQ

>CYP139A1(2575028161)Mycobacterium tuberculosis TKK_05SA_0058

MRYPLGEALLALYRWRGPLINAGVGGHGYTYLLGAEANRFVFANADAFSWSQTFESLVPV

DGPTALIVSDGADHRRRRSVVAPGLRHHHVQRYVATMVSNIDTVIDGWQPGQRLDIYQEL

RSAVRRSTAESLFGQRLAVHSDFLGEQLQPLLDLTRRPPQVMRLQQRVNSPGWRRAMAAR

KRIDDLIDAQIADARTAPRPDDHMLTTLISGCSEEGTTLSDNEIRDSIVSLITAGYETTS

GALAWAIYALLTVPGTWESAASEVARVLGGRVPAADDLSALTYLNGVVHETLRLYSPGVI

SARRVLRDLWFDGHRIRAGRLLIFSAYVTHRLPEIWPEPTEFRPLRWDPNAADYRKPAPH

EFIPFSGGLHRCIGAVMATTEMTVILARLVARAMLQLPAQRTHRIRAANFAALRPWPGLT

VEIRKSAPAQ

>CYP139A1(2575888575)Mycobacterium tuberculosis TBR41

MRYPLGEALLALYRWRGPLINAGVGGHGYTYLLGAEANRFVFANADAFSWSQTFESLVPV

DGPTALIVSDGADHRRRRSVVAPGLRHHHVQRYVATMVSNIDTVIDGWQPGQRLDIYQEL

RSAVRRSTAESLFGQRLAVHSDFLGEQLQPLLDLTRRPPQVMRLQQRVNSPGWRRAMAAR

KRIDDLIDAQIADARTAPRPDDHMLTTLISGCSEEGTTLSDNEIRDSIVSLITAGYETTS

GALAWAIYALLTVPGTWESAASEVARVLGGRVPAADDLSALTYLNGVVHETLRLYSPGVI

SARRVLRDLWFDGHRIRAGRLLIFSAYVTHRLPEIWPEPTEFRPLRWDPNAADYRKPAPH

EFIPFSGGLHRCIGAVMATTEMTVILARLVARAMLQLPAQRTHRIRAANFAALRPWPGLT

VEIRKSAPAQ

>CYP139A1(2576708817)Mycobacterium tuberculosis TKK_03_0090

MRYPLGEALLALYRWRGPLINAGVGGHGYTYLLGAEANRFVFANADAFSWSQTFESLVPV

DGPTALIVSDGADHRRRRSVVAPGLRHHHVQRYVATMVSNIDTVIDGWQPGQRLDIYQEL

RSAVRRSTAESLFGQRLAVHSDFLGEQLQPLLDLTRRPPQVMRLQQRVNSPGWRRAMAAR

KRIDDLIDAQIADARTAPRPDDHMLTTLISGCSEEGTTLSDNEIRDSIVSLITAGYETTS

GALAWAIYALLTVPGTWESAASEVARVLGGRVPAADDLSALTYLNGVVHETLRLYSPGVI

SARRVLRDLWFDGHRIRAGRLLIFSAYVTHRLPEIWPEPTEFRPLRWDPNAADYRKPAPH

EFIPFSGGLHRCIGAVMATTEMTVILARLVARAMLQLPAQRTHRIRAANFAALRPWPGLT

VEIRKSAPAQ

>CYP139A1(2577386815)Mycobacterium tuberculosis TKK_04_0054

MRYPLGEALLALYRWRGPLINAGVGGHGYTYLLGAEANRFVFANADAFSWSQTFESLVPV

DGPTALIVSDGADHRRRRSVVAPGLRHHHVQRYVATMVSNIDTVIDGWQPGQRLDIYQEL

RSAVRRSTAESLFGQRLAVHSDFLGEQLQPLLDLTRRPPQVMRLQQRVNSPGWRRAMAAR

KRIDDLIDAQIADARTAPRPDDHMLTTLISGCSEEGTTLSDNEIRDSIVSLITAGYETTS

GALAWAIYALLTVPGTWESAASEVARVLGGRVPAADDLSALTYLNGVVHETLRLYSPGVI

SARRVLRDLWFDGHRIRAGRLLIFSAYVTHRLPEIWPEPTEFRPLRWDPNAADYRKPAPH

EFIPFSGGLHRCIGAVMATTEMTVILARLVARAMLQLPAQRTHRIRAANFAALRPWPGLT

VEIRKSAPAQ

>CYP139A1(2578040087)Mycobacterium tuberculosis TKK_05SA_0017

MRYPLGEALLALYRWRGPLINAGVGGHGYTYLLGAEANRFVFANADAFSWSQTFESLVPV

DGPTALIVSDGADHRRRRSVVAPGLRHHHVQRYVATMVSNIDTVIDGWQPGQRLDIYQEL

RSAVRRSTAESLFGQRLAVHSDFLGEQLQPLLDLTRRPPQVMRLQQRVNSPGWRRAMAAR

KRIDDLIDAQIADARTAPRPDDHMLTTLISGCSEEGTTLSDNEIRDSIVSLITAGYETTS

GALAWAIYALLTVPGTWESAASEVARVLGGRVPAADDLSALTYLNGVVHETLRLYSPGVI

SARRVLRDLWFDGHRIRAGRLLIFSAYVTHRLPEIWPEPTEFRPLRWDPNAADYRKPAPH

EFIPFSGGLHRCIGAVMATTEMTVILARLVARAMLQLPAQRTHRIRAANFAALRPWPGLT

VEIRKSAPAQ

>CYP139A1(2584645880)Mycobacterium tuberculosis TKK-01-0038

MRYPLGEALLALYRWRGPLINAGVGGHGYTYLLGAEANRFVFANADAFSWSQTFESLVPV

DGPTALIVSDGADHRRRRSVVAPGLRHHHVQRYVATMVSNIDTVIDGWQPGQRLDIYQEL

RSAVRRSTAESLFGQRLAVHSDFLGEQLQPLLDLTRRPPQVMRLQQRVNSPGWRRAMAAR

KRIDDLIDAQIADARTAPRPDDHMLTTLISGCSEEGTTLSDNEIRDSIVSLITAGYETTS

GALAWAIYALLTVPGTWESAASEVARVLGGRVPAADDLSALTYLNGVVHETLRLYSPGVI

SARRVLRDLWFDGHRIRAGRLLIFSAYVTHRLPEIWPEPTEFRPLRWDPNAADYRKPAPH

EFIPFSGGLHRCIGAVMATTEMTVILARLVARAMLQLPAQRTHRIRAANFAALRPWPGLT

VEIRKSAPAQ

>CYP139A1(2584659187)Mycobacterium tuberculosis XTB13-194

MRYPLGEALLALYRWRGPLINAGVGGHGYTYLLGAEANRFVFANADAFSWSQTFESLVPV

DGPTALIVSDGADHRRRRSVVAPGLRHHHVQRYVATMVSNIDTVIDGWQPGQRLDIYQEL

RSAVRRSTAESLFGQRLAVHSDFLGEQLQPLLDLTRRPPQVMRLQQRVNSPGWRRAMAAR

KRIDDLIDAQIADARTAPRPDDHMLTTLISGCSEEGTTLSDNEIRDSIVSLITAGYETTS

GALAWAIYALLTVPGTWESAASEVARVLGGRVPAADDLSALTYLNGVVHETLRLYSPGVI

SARRVLRDLWFDGHRIRAGRLLIFSAYVTHRLPEIWPEPTEFRPLRWDPNAADYRKPAPH

EFIPFSGGLHRCIGAVMATTEMTVILARLVARAMLQLPAQRTHRIRAANFAALRPWPGLT

VEIRKSAPAQ

>CYP139A1(2584718757)Mycobacterium tuberculosis XTB13-082

MRYPLGEALLALYRWRGPLINAGVGGHGYTYLLGAEANRFVFANADAFSWSQTFESLVPV

DGPTALIVSDGADHRRRRSVVAPGLRHHHVQRYVATMVSNIDTVIDGWQPGQRLDIYQEL

RSAVRRSTAESLFGQRLAVHSDFLGEQLQPLLDLTRRPPQVMRLQQRVNSPGWRRAMAAR

KRIDDLIDAQIADARTAPRPDDHMLTTLISGCSEEGTTLSDNEIRDSIVSLITAGYETTS

GALAWAIYALLTVPGTWESAASEVARVLGGRVPAADDLSALTYLNGVVHETLRLYSPGVI

SARRVLRDLWFDGHRIRAGRLLIFSAYVTHRLPEIWPEPTEFRPLRWDPNAADYRKPAPH

EFIPFSGGLHRCIGAVMATTEMTVILARLVARAMLQLPAQRTHRIRAANFAALRPWPGLT

VEIRKSAPAQ

>CYP139A1(2589064595)Mycobacterium tuberculosis TBR40

MRYPLGEALLALYRWRGPLINAGVGGHGYTYLLGAEANRFVFANADAFSWSQTFESLVPV

DGPTALIVSDGADHRRRRSVVAPGLRHHHVQRYVATMVSNIDTVIDGWQPGQRLDIYQEL

RSAVRRSTAESLFGQRLAVHSDFLGEQLQPLLDLTRRPPQVMRLQQRVNSPGWRRAMAAR

KRIDDLIDAQIADARTAPRPDDHMLTTLISGCSEEGTTLSDNEIRDSIVSLITAGYETTS

GALAWAIYALLTVPGTWESAASEVARVLGGRVPAADDLSALTYLNGVVHETLRLYSPGVI

SARRVLRDLWFDGHRIRAGRLLIFSAYVTHRLPEIWPEPTEFRPLRWDPNAADYRKPAPH

EFIPFSGGLHRCIGAVMATTEMTVILARLVARAMLQLPAQRTHRIRAANFAALRPWPGLT

VEIRKSAPAQ

>CYP139A1(2589113575)Mycobacterium tuberculosis TBR66

MRYPLGEALLALYRWRGPLINAGVGGHGYTYLLGAEANRFVFANADAFSWSQTFESLVPV

DGPTALIVSDGADHRRRRSVVAPGLRHHHVQRYVATMVSNIDTVIDGWQPGQRLDIYQEL

RSAVRRSTAESLFGQRLAVHSDFLGEQLQPLLDLTRRPPQVMRLQQRVNSPGWRRAMAAR

KRIDDLIDAQIADARTAPRPDDHMLTTLISGCSEEGTTLSDNEIRDSIVSLITAGYETTS

GALAWAIYALLTVPGTWESAASEVARVLGGRVPAADDLSALTYLNGVVHETLRLYSPGVI

SARRVLRDLWFDGHRIRAGRLLIFSAYVTHRLPEIWPEPTEFRPLRWDPNAADYRKPAPH

EFIPFSGGLHRCIGAVMATTEMTVILARLVARAMLQLPAQRTHRIRAANFAALRPWPGLT

VEIRKSAPAQ

>CYP139A1(2589559459)Mycobacterium tuberculosis TKK-01-0029

MRYPLGEALLALYRWRGPLINAGVGGHGYTYLLGAEANRFVFANADAFSWSQTFESLVPV

DGPTALIVSDGADHRRRRSVVAPGLRHHHVQRYVATMVSNIDTVIDGWQPGQRLDIYQEL

RSAVRRSTAESLFGQRLAVHSDFLGEQLQPLLDLTRRPPQVMRLQQRVNSPGWRRAMAAR

KRIDDLIDAQIADARTAPRPDDHMLTTLISGCSEEGTTLSDNEIRDSIVSLITAGYETTS

GALAWAIYALLTVPGTWESAASEVARVLGGRVPAADDLSALTYLNGVVHETLRLYSPGVI

SARRVLRDLWFDGHRIRAGRLLIFSAYVTHRLPEIWPEPTEFRPLRWDPNAADYRKPAPH

EFIPFSGGLHRCIGAVMATTEMTVILARLVARAMLQLPAQRTHRIRAANFAALRPWPGLT

VEIRKSAPAQ

>CYP139A1(2589575466)Mycobacterium tuberculosis TKK-01-0039

MRYPLGEALLALYRWRGPLINAGVGGHGYTYLLGAEANRFVFANADAFSWSQTFESLVPV

DGPTALIVSDGADHRRRRSVVAPGLRHHHVQRYVATMVSNIDTVIDGWQPGQRLDIYQEL

RSAVRRSTAESLFGQRLAVHSDFLGEQLQPLLDLTRRPPQVMRLQQRVNSPGWRRAMAAR

KRIDDLIDAQIADARTAPRPDDHMLTTLISGCSEEGTTLSDNEIRDSIVSLITAGYETTS

GALAWAIYALLTVPGTWESAASEVARVLGGRVPAADDLSALTYLNGVVHETLRLYSPGVI

SARRVLRDLWFDGHRIRAGRLLIFSAYVTHRLPEIWPEPTEFRPLRWDPNAADYRKPAPH

EFIPFSGGLHRCIGAVMATTEMTVILARLVARAMLQLPAQRTHRIRAANFAALRPWPGLT

VEIRKSAPAQ

>CYP139A1(2589595990)Mycobacterium tuberculosis TKK-01-0044

MRYPLGEALLALYRWRGPLINAGVGGHGYTYLLGAEANRFVFANADAFSWSQTFESLVPV

DGPTALIVSDGADHRRRRSVVAPGLRHHHVQRYVATMVSNIDTVIDGWQPGQRLDIYQEL

RSAVRRSTAESLFGQRLAVHSDFLGEQLQPLLDLTRRPPQVMRLQQRVNSPGWRRAMAAR

KRIDDLIDAQIADARTAPRPDDHMLTTLISGCSEEGTTLSDNEIRDSIVSLITAGYETTS

GALAWAIYALLTVPGTWESAASEVARVLGGRVPAADDLSALTYLNGVVHETLRLYSPGVI

SARRVLRDLWFDGHRIRAGRLLIFSAYVTHRLPEIWPEPTEFRPLRWDPNAADYRKPAPH

EFIPFSGGLHRCIGAVMATTEMTVILARLVARAMLQLPAQRTHRIRAANFAALRPWPGLT

VEIRKSAPAQ

>CYP139A1(2592259141)Mycobacterium tuberculosis TKK_04_0007

MRYPLGEALLALYRWRGPLINAGVGGHGYTYLLGAEANRFVFANADAFSWSQTFESLVPV

DGPTALIVSDGADHRRRRSVVAPGLRHHHVQRYVATMVSNIDTVIDGWQPGQRLDIYQEL

RSAVRRSTAESLFGQRLAVHSDFLGEQLQPLLDLTRRPPQVMRLQQRVNSPGWRRAMAAR

KRIDDLIDAQIADARTAPRPDDHMLTTLISGCSEEGTTLSDNEIRDSIVSLITAGYETTS

GALAWAIYALLTVPGTWESAASEVARVLGGRVPAADDLSALTYLNGVVHETLRLYSPGVI

SARRVLRDLWFDGHRIRAGRLLIFSAYVTHRLPEIWPEPTEFRPLRWDPNAADYRKPAPH

EFIPFSGGLHRCIGAVMATTEMTVILARLVARAMLQLPAQRTHRIRAANFAALRPWPGLT

VEIRKSAPAQ

>CYP139A1(2592280664)Mycobacterium tuberculosis TKK_03_0081

MRYPLGEALLALYRWRGPLINAGVGGHGYTYLLGAEANRFVFANADAFSWSQTFESLVPV

DGPTALIVSDGADHRRRRSVVAPGLRHHHVQRYVATMVSNIDTVIDGWQPGQRLDIYQEL

RSAVRRSTAESLFGQRLAVHSDFLGEQLQPLLDLTRRPPQVMRLQQRVNSPGWRRAMAAR

KRIDDLIDAQIADARTAPRPDDHMLTTLISGCSEEGTTLSDNEIRDSIVSLITAGYETTS

GALAWAIYALLTVPGTWESAASEVARVLGGRVPAADDLSALTYLNGVVHETLRLYSPGVI

SARRVLRDLWFDGHRIRAGRLLIFSAYVTHRLPEIWPEPTEFRPLRWDPNAADYRKPAPH

EFIPFSGGLHRCIGAVMATTEMTVILARLVARAMLQLPAQRTHRIRAANFAALRPWPGLT

VEIRKSAPAQ

>CYP139A1(2592389699)Mycobacterium tuberculosis TKK_02_0033

MRYPLGEALLALYRWRGPLINAGVGGHGYTYLLGAEANRFVFANADAFSWSQTFESLVPV

DGPTALIVSDGADHRRRRSVVAPGLRHHHVQRYVATMVSNIDTVIDGWQPGQRLDIYQEL

RSAVRRSTAESLFGQRLAVHSDFLGEQLQPLLDLTRRPPQVMRLQQRVNSPGWRRAMAAR

KRIDDLIDAQIADARTAPRPDDHMLTTLISGCSEEGTTLSDNEIRDSIVSLITAGYETTS

GALAWAIYALLTVPGTWESAASEVARVLGGRVPAADDLSALTYLNGVVHETLRLYSPGVI

SARRVLRDLWFDGHRIRAGRLLIFSAYVTHRLPEIWPEPTEFRPLRWDPNAADYRKPAPH

EFIPFSGGLHRCIGAVMATTEMTVILARLVARAMLQLPAQRTHRIRAANFAALRPWPGLT

VEIRKSAPAQ

>CYP139A1(2592438630)Mycobacterium tuberculosis TKK_02_0003

MRYPLGEALLALYRWRGPLINAGVGGHGYTYLLGAEANRFVFANADAFSWSQTFESLVPV

DGPTALIVSDGADHRRRRSVVAPGLRHHHVQRYVATMVSNIDTVIDGWQPGQRLDIYQEL

RSAVRRSTAESLFGQRLAVHSDFLGEQLQPLLDLTRRPPQVMRLQQRVNSPGWRRAMAAR

KRIDDLIDAQIADARTAPRPDDHMLTTLISGCSEEGTTLSDNEIRDSIVSLITAGYETTS

GALAWAIYALLTVPGTWESAASEVARVLGGRVPAADDLSALTYLNGVVHETLRLYSPGVI

SARRVLRDLWFDGHRIRAGRLLIFSAYVTHRLPEIWPEPTEFRPLRWDPNAADYRKPAPH

EFIPFSGGLHRCIGAVMATTEMTVILARLVARAMLQLPAQRTHRIRAANFAALRPWPGLT

VEIRKSAPAQ

>CYP139A1(2592553482)Mycobacterium tuberculosis TKK_04_0039

MRYPLGEALLALYRWRGPLINAGVGGHGYTYLLGAEANRFVFANADAFSWSQTFESLVPV

DGPTALIVSDGADHRRRRSVVAPGLRHHHVQRYVATMVSNIDTVIDGWQPGQRLDIYQEL

RSAVRRSTAESLFGQRLAVHSDFLGEQLQPLLDLTRRPPQVMRLQQRVNSPGWRRAMAAR

KRIDDLIDAQIADARTAPRPDDHMLTTLISGCSEEGTTLSDNEIRDSIVSLITAGYETTS

GALAWAIYALLTVPGTWESAASEVARVLGGRVPAADDLSALTYLNGVVHETLRLYSPGVI

SARRVLRDLWFDGHRIRAGRLLIFSAYVTHRLPEIWPEPTEFRPLRWDPNAADYRKPAPH

EFIPFSGGLHRCIGAVMATTEMTVILARLVARAMLQLPAQRTHRIRAANFAALRPWPGLT

VEIRKSAPAQ

>CYP139A1(2592570555)Mycobacterium tuberculosis TKK_04_0031

MRYPLGEALLALYRWRGPLINAGVGGHGYTYLLGAEANRFVFANADAFSWSQTFESLVPV

DGPTALIVSDGADHRRRRSVVAPGLRHHHVQRYVATMVSNIDTVIDGWQPGQRLDIYQEL

RSAVRRSTAESLFGQRLAVHSDFLGEQLQPLLDLTRRPPQVMRLQQRVNSPGWRRAMAAR

KRIDDLIDAQIADARTAPRPDDHMLTTLISGCSEEGTTLSDNEIRDSIVSLITAGYETTS

GALAWAIYALLTVPGTWESAASEVARVLGGRVPAADDLSALTYLNGVVHETLRLYSPGVI

SARRVLRDLWFDGHRIRAGRLLIFSAYVTHRLPEIWPEPTEFRPLRWDPNAADYRKPAPH

EFIPFSGGLHRCIGAVMATTEMTVILARLVARAMLQLPAQRTHRIRAANFAALRPWPGLT

VEIRKSAPAQ

>CYP139A1(2575252379)Mycobacterium tuberculosis M1481

MRYPLGEALLALYRWRGPLINAGVGGHGYTYLLGAEANRFVFANADAFSWSQTFESLVPV

DGPTALIVSDGADHRRRRSVVAPGLRHHHVQRYVATMVSNIDTVIDGWQPGQRLDIYQEL

RSAVRRSTAESLFGQRLAVHSDFLGEQLQPLLDLTRRPPQVMRLQQRVNSPGWRRAMAAR

KRIDDLIDAQIADARTAPRPDDHMLTTLISGCSEEGTTLSDNEIRDSIVSLITAGYETTS

GALAWAIYALLTVPGTWESAASEVARVLGGRVPAADDLSALTYLNGVVHETLRLYSPGVI

SARRVLRDLWFDGHRIRAGRLLIFSAYVTHRLPEIWPEPTEFRPLRWDPNAADYRKPAPH

EFIPFSGGLHRCIGAVMATTEMTVILARLVARAMLQLPAQRTHRIRAANFAALRPWPGLT

VEIRKSAPAQ

>CYP139A1(2575520720)Mycobacterium tuberculosis BTB11-001

MRYPLGEALLALYRWRGPLINAGVGGHGYTYLLGAEANRFVFANADAFSWSQTFESLVPV

DGPTALIVSDGADHRRRRSVVAPGLRHHHVQRYVATMVSNIDTVIDGWQPGQRLDIYQEL

RSAVRRSTAESLFGQRLAVHSDFLGEQLQPLLDLTRRPPQVMRLQQRVNSPGWRRAMAAR

KRIDDLIDAQIADARTAPRPDDHMLTTLISGCSEEGTTLSDNEIRDSIVSLITAGYETTS

GALAWAIYALLTVPGTWESAASEVARVLGGRVPAADDLSALTYLNGVVHETLRLYSPGVI

SARRVLRDLWFDGHRIRAGRLLIFSAYVTHRLPEIWPEPTEFRPLRWDPNAADYRKPAPH

EFIPFSGGLHRCIGAVMATTEMTVILARLVARAMLQLPAQRTHRIRAANFAALRPWPGLT

VEIRKSAPAQ

>CYP139A1(2575705222)*Mycobacterium africanum* MAL010137

MRYPLGEALLALYRWRGPLINAGVGGHGYTYLLGAEANRFVFANADAFSWSQTFESLVPV

DGPTALIVSDGADHRRRRSVVAPGLRHHHVQRYVATMVSNIDTVIDGWQPGQRLDIYQEL

RSAVRRSTAESLFGQRLAVHSDFLGEQLQPLLDLTRRPPQVMRLQQRVNSPGWRRAMAAR

KRIDDLIDAQIADARTAPRPDDHMLTTLISGCSEEGTTLSDNEIRDSIVSLITAGYETTS

GALAWAIYALLTVPGTWESAASEVARVLGGRVPAADDLSALTYLNGVVHETLRLYSPGVI

SARRVLRDLWFDGHRIRAGRLLIFSAYVTHRLPEIWPEPTEFRPLRWDPNAADYRKPAPH

EFIPFSGGLHRCIGAVMATTEMTVILARLVARAMLQLPAQRTHRIRAANFAALRPWPGLT

VEIRKSAPAQ

>CYP139A1(2575964273)Mycobacterium tuberculosis KT-0040

MRYPLGEALLALYRWRGPLINAGVGGHGYTYLLGAEANRFVFANADAFSWSQTFESLVPV

DGPTALIVSDGADHRRRRSVVAPGLRHHHVQRYVATMVSNIDTVIDGWQPGQRLDIYQEL

RSAVRRSTAESLFGQRLAVHSDFLGEQLQPLLDLTRRPPQVMRLQQRVNSPGWRRAMAAR

KRIDDLIDAQIADARTAPRPDDHMLTTLISGCSEEGTTLSDNEIRDSIVSLITAGYETTS

GALAWAIYALLTVPGTWESAASEVARVLGGRVPAADDLSALTYLNGVVHETLRLYSPGVI

SARRVLRDLWFDGHRIRAGRLLIFSAYVTHRLPEIWPEPTEFRPLRWDPNAADYRKPAPH

EFIPFSGGLHRCIGAVMATTEMTVILARLVARAMLQLPAQRTHRIRAANFAALRPWPGLT

VEIRKSAPAQ

>CYP139A1(2577195360)Mycobacterium tuberculosis MAL020120

MRYPLGEALLALYRWRGPLINAGVGGHGYTYLLGAEANRFVFANADAFSWSQTFESLVPV

DGPTALIVSDGADHRRRRSVVAPGLRHHHVQRYVATMVSNIDTVIDGWQPGQRLDIYQEL

RSAVRRSTAESLFGQRLAVHSDFLGEQLQPLLDLTRRPPQVMRLQQRVNSPGWRRAMAAR

KRIDDLIDAQIADARTAPRPDDHMLTTLISGCSEEGTTLSDNEIRDSIVSLITAGYETTS

GALAWAIYALLTVPGTWESAASEVARVLGGRVPAADDLSALTYLNGVVHETLRLYSPGVI

SARRVLRDLWFDGHRIRAGRLLIFSAYVTHRLPEIWPEPTEFRPLRWDPNAADYRKPAPH

EFIPFSGGLHRCIGAVMATTEMTVILARLVARAMLQLPAQRTHRIRAANFAALRPWPGLT

VEIRKSAPAQ

>CYP139A1(2581587072)Mycobacterium bovisBz 31150

MRYPLGEALLALYRWRGPLINAGVGGHGYTYLLGAEANRFVFANADAFSWSQTFESLVPV

DGPTALIVSDGADHRRRRSVVAPGLRHHHVQRYVATMVSNIDTVIDGWQPGQRLDIYQEL

RSAVRRSTAESLFGQRLAVHSDFLGEQLQPLLDLTRRPPQVMRLQQRVNSPGWRRAMAAR

KRIDDLIDAQIADARTAPRPDDHMLTTLISGCSEEGTTLSDNEIRDSIVSLITAGYETTS

GALAWAIYALLTVPGTWESAASEVARVLGGRVPAADDLSALTYLNGVVHETLRLYSPGVI

SARRVLRDLWFDGHRIRAGRLLIFSAYVTHRLPEIWPEPTEFRPLRWDPNAADYRKPAPH

EFIPFSGGLHRCIGAVMATTEMTVILARLVARAMLQLPAQRTHRIRAANFAALRPWPGLT

VEIRKSAPAQ

>CYP139A1(2584633235)Mycobacterium tuberculosis M1956

MRYPLGEALLALYRWRGPLINAGVGGHGYTYLLGAEANRFVFANADAFSWSQTFESLVPV

DGPTALIVSDGADHRRRRSVVAPGLRHHHVQRYVATMVSNIDTVIDGWQPGQRLDIYQEL

RSAVRRSTAESLFGQRLAVHSDFLGEQLQPLLDLTRRPPQVMRLQQRVNSPGWRRAMAAR

KRIDDLIDAQIADARTAPRPDDHMLTTLISGCSEEGTTLSDNEIRDSIVSLITAGYETTS

GALAWAIYALLTVPGTWESAASEVARVLGGRVPAADDLSALTYLNGVVHETLRLYSPGVI

SARRVLRDLWFDGHRIRAGRLLIFSAYVTHRLPEIWPEPTEFRPLRWDPNAADYRKPAPH

EFIPFSGGLHRCIGAVMATTEMTVILARLVARAMLQLPAQRTHRIRAANFAALRPWPGLT

VEIRKSAPAQ

>CYP139A1(2584732271)Mycobacterium tuberculosis M1703

MRYPLGEALLALYRWRGPLINAGVGGHGYTYLLGAEANRFVFANADAFSWSQTFESLVPV

DGPTALIVSDGADHRRRRSVVAPGLRHHHVQRYVATMVSNIDTVIDGWQPGQRLDIYQEL

RSAVRRSTAESLFGQRLAVHSDFLGEQLQPLLDLTRRPPQVMRLQQRVNSPGWRRAMAAR

KRIDDLIDAQIADARTAPRPDDHMLTTLISGCSEEGTTLSDNEIRDSIVSLITAGYETTS

GALAWAIYALLTVPGTWESAASEVARVLGGRVPAADDLSALTYLNGVVHETLRLYSPGVI

SARRVLRDLWFDGHRIRAGRLLIFSAYVTHRLPEIWPEPTEFRPLRWDPNAADYRKPAPH

EFIPFSGGLHRCIGAVMATTEMTVILARLVARAMLQLPAQRTHRIRAANFAALRPWPGLT

VEIRKSAPAQ

>CYP139A1(2584791771)Mycobacterium tuberculosis 1010SM

MRYPLGEALLALYRWRGPLINAGVGGHGYTYLLGAEANRFVFANADAFSWSQTFESLVPV

DGPTALIVSDGADHRRRRSVVAPGLRHHHVQRYVATMVSNIDTVIDGWQPGQRLDIYQEL

RSAVRRSTAESLFGQRLAVHSDFLGEQLQPLLDLTRRPPQVMRLQQRVNSPGWRRAMAAR

KRIDDLIDAQIADARTAPRPDDHMLTTLISGCSEEGTTLSDNEIRDSIVSLITAGYETTS

GALAWAIYALLTVPGTWESAASEVARVLGGRVPAADDLSALTYLNGVVHETLRLYSPGVI

SARRVLRDLWFDGHRIRAGRLLIFSAYVTHRLPEIWPEPTEFRPLRWDPNAADYRKPAPH

EFIPFSGGLHRCIGAVMATTEMTVILARLVARAMLQLPAQRTHRIRAANFAALRPWPGLT

VEIRKSAPAQ

>CYP139A1(2589148607)Mycobacterium tuberculosis OFXR-5

MRYPLGEALLALYRWRGPLINAGVGGHGYTYLLGAEANRFVFANADAFSWSQTFESLVPV

DGPTALIVSDGADHRRRRSVVAPGLRHHHVQRYVATMVSNIDTVIDGWQPGQRLDIYQEL

RSAVRRSTAESLFGQRLAVHSDFLGEQLQPLLDLTRRPPQVMRLQQRVNSPGWRRAMAAR

KRIDDLIDAQIADARTAPRPDDHMLTTLISGCSEEGTTLSDNEIRDSIVSLITAGYETTS

GALAWAIYALLTVPGTWESAASEVARVLGGRVPAADDLSALTYLNGVVHETLRLYSPGVI

SARRVLRDLWFDGHRIRAGRLLIFSAYVTHRLPEIWPEPTEFRPLRWDPNAADYRKPAPH

EFIPFSGGLHRCIGAVMATTEMTVILARLVARAMLQLPAQRTHRIRAANFAALRPWPGLT

VEIRKSAPAQ

>CYP139A1(2590150448)Mycobacterium tuberculosis MAL020194

MRYPLGEALLALYRWRGPLINAGVGGHGYTYLLGAEANRFVFANADAFSWSQTFESLVPV

DGPTALIVSDGADHRRRRSVVAPGLRHHHVQRYVATMVSNIDTVIDGWQPGQRLDIYQEL

RSAVRRSTAESLFGQRLAVHSDFLGEQLQPLLDLTRRPPQVMRLQQRVNSPGWRRAMAAR

KRIDDLIDAQIADARTAPRPDDHMLTTLISGCSEEGTTLSDNEIRDSIVSLITAGYETTS

GALAWAIYALLTVPGTWESAASEVARVLGGRVPAADDLSALTYLNGVVHETLRLYSPGVI

SARRVLRDLWFDGHRIRAGRLLIFSAYVTHRLPEIWPEPTEFRPLRWDPNAADYRKPAPH

EFIPFSGGLHRCIGAVMATTEMTVILARLVARAMLQLPAQRTHRIRAANFAALRPWPGLT

VEIRKSAPAQ

>CYP139A1(2590231653)Mycobacterium tuberculosis KT-0045

MRYPLGEALLALYRWRGPLINAGVGGHGYTYLLGAEANRFVFANADAFSWSQTFESLVPV

DGPTALIVSDGADHRRRRSVVAPGLRHHHVQRYVATMVSNIDTVIDGWQPGQRLDIYQEL

RSAVRRSTAESLFGQRLAVHSDFLGEQLQPLLDLTRRPPQVMRLQQRVNSPGWRRAMAAR

KRIDDLIDAQIADARTAPRPDDHMLTTLISGCSEEGTTLSDNEIRDSIVSLITAGYETTS

GALAWAIYALLTVPGTWESAASEVARVLGGRVPAADDLSALTYLNGVVHETLRLYSPGVI

SARRVLRDLWFDGHRIRAGRLLIFSAYVTHRLPEIWPEPTEFRPLRWDPNAADYRKPAPH

EFIPFSGGLHRCIGAVMATTEMTVILARLVARAMLQLPAQRTHRIRAANFAALRPWPGLT

VEIRKSAPAQ

>CYP139A1(2590283895)Mycobacterium tuberculosis KT-0007

MRYPLGEALLALYRWRGPLINAGVGGHGYTYLLGAEANRFVFANADAFSWSQTFESLVPV

DGPTALIVSDGADHRRRRSVVAPGLRHHHVQRYVATMVSNIDTVIDGWQPGQRLDIYQEL

RSAVRRSTAESLFGQRLAVHSDFLGEQLQPLLDLTRRPPQVMRLQQRVNSPGWRRAMAAR

KRIDDLIDAQIADARTAPRPDDHMLTTLISGCSEEGTTLSDNEIRDSIVSLITAGYETTS

GALAWAIYALLTVPGTWESAASEVARVLGGRVPAADDLSALTYLNGVVHETLRLYSPGVI

SARRVLRDLWFDGHRIRAGRLLIFSAYVTHRLPEIWPEPTEFRPLRWDPNAADYRKPAPH

EFIPFSGGLHRCIGAVMATTEMTVILARLVARAMLQLPAQRTHRIRAANFAALRPWPGLT

VEIRKSAPAQ

>CYP139A1(645120373)Mycobacterium tuberculosis KZN 605

MRYPLGEALLALYRWRGPLINAGVGGHGYTYLLGAEANRFVFANADAFSWSQTFESLVPV

DGPTALIVSDGADHRRRRSVVAPGLRHHHVQRYVATMVSNIDTVIDGWQPGQRLDIYQEL

RSAVRRSTAESLFGQRLAVHSDFLGEQLQPLLDLTRRPPQVMRLQQRVNSPGWRRAMAAR

KRIDDLIDAQIADARTAPRPDDHMLTTLISGCSEEGTTLSDNEIRDSIVSLITAGYETTS

GALAWAIYALLTVPGTWESAASEVARVLGGRVPAADDLSALTYLNGVVHETLRLYSPGVI

SARRVLRDLWFDGHRIRAGRLLIFSAYVTHRLPEIWPEPTEFRPLRWDPNAADYRKPAPH

EFIPFSGGLHRCIGAVMATTEMTVILARLVARAMLQLPAQRTHRIRAANFAALRPWPGLT

VEIRKSAPAQ

>CYP139A1(2574734007)Mycobacterium tuberculosis TKK_04_0080

MRYPLGEALLALYRWRGPLINAGVGGHGYTYLLGAEANRFVFANADAFSWSQTFESLVPV

DGPTALIVSDGADHRRRRSVVAPGLRHHHVQRYVATMVSNIDTVIDGWQPGQRLDIYQEL

RSAVRRSTAESLFGQRLAVHSDFLGEQLQPLLDLTRRPPQVMRLQQRVNSPGWRRAMAAR

KRIDDLIDAQIADARTAPRPDDHMLTTLISGCSEEGTTLSDNEIRDSIVSLITAGYETTS

GALAWAIYALLTVPGTWESAASEVARVLGGRVPAADDLSALTYLNGVVHETLRLYSPGVI

SARRVLRDLWFDGHRIRAGRLLIFSAYVTHRLPEIWPEPTEFRPLRWDPNAADYRKPAPH

EFIPFSGGLHRCIGAVMATTEMTVILARLVARAMLQLPAQRTHRIRAANFAALRPWPGLT

VEIRKSAPAQ

>CYP139A1(2575099885)Mycobacterium tuberculosis XTB13-251

MRYPLGEALLALYRWRGPLINAGVGGHGYTYLLGAEANRFVFANADAFSWSQTFESLVPV

DGPTALIVSDGADHRRRRSVVAPGLRHHHVQRYVATMVSNIDTVIDGWQPGQRLDIYQEL

RSAVRRSTAESLFGQRLAVHSDFLGEQLQPLLDLTRRPPQVMRLQQRVNSPGWRRAMAAR

KRIDDLIDAQIADARTAPRPDDHMLTTLISGCSEEGTTLSDNEIRDSIVSLITAGYETTS

GALAWAIYALLTVPGTWESAASEVARVLGGRVPAADDLSALTYLNGVVHETLRLYSPGVI

SARRVLRDLWFDGHRIRAGRLLIFSAYVTHRLPEIWPEPTEFRPLRWDPNAADYRKPAPH

EFIPFSGGLHRCIGAVMATTEMTVILARLVARAMLQLPAQRTHRIRAANFAALRPWPGLT

VEIRKSAPAQ

>CYP139A1(2575132657)Mycobacterium tuberculosis TKK_04_0019

MRYPLGEALLALYRWRGPLINAGVGGHGYTYLLGAEANRFVFANADAFSWSQTFESLVPV

DGPTALIVSDGADHRRRRSVVAPGLRHHHVQRYVATMVSNIDTVIDGWQPGQRLDIYQEL

RSAVRRSTAESLFGQRLAVHSDFLGEQLQPLLDLTRRPPQVMRLQQRVNSPGWRRAMAAR

KRIDDLIDAQIADARTAPRPDDHMLTTLISGCSEEGTTLSDNEIRDSIVSLITAGYETTS

GALAWAIYALLTVPGTWESAASEVARVLGGRVPAADDLSALTYLNGVVHETLRLYSPGVI

SARRVLRDLWFDGHRIRAGRLLIFSAYVTHRLPEIWPEPTEFRPLRWDPNAADYRKPAPH

EFIPFSGGLHRCIGAVMATTEMTVILARLVARAMLQLPAQRTHRIRAANFAALRPWPGLT

VEIRKSAPAQ

>CYP139A1(2575465536)Mycobacterium tuberculosis OFXR-8

MRYPLGEALLALYRWRGPLINAGVGGHGYTYLLGAEANRFVFANADAFSWSQTFESLVPV

DGPTALIVSDGADHRRRRSVVAPGLRHHHVQRYVATMVSNIDTVIDGWQPGQRLDIYQEL

RSAVRRSTAESLFGQRLAVHSDFLGEQLQPLLDLTRRPPQVMRLQQRVNSPGWRRAMAAR

KRIDDLIDAQIADARTAPRPDDHMLTTLISGCSEEGTTLSDNEIRDSIVSLITAGYETTS

GALAWAIYALLTVPGTWESAASEVARVLGGRVPAADDLSALTYLNGVVHETLRLYSPGVI

SARRVLRDLWFDGHRIRAGRLLIFSAYVTHRLPEIWPEPTEFRPLRWDPNAADYRKPAPH

EFIPFSGGLHRCIGAVMATTEMTVILARLVARAMLQLPAQRTHRIRAANFAALRPWPGLT

VEIRKSAPAQ

>CYP139A1(2576665707)Mycobacterium tuberculosis TKK_04_0117

MRYPLGEALLALYRWRGPLINAGVGGHGYTYLLGAEANRFVFANADAFSWSQTFESLVPV

DGPTALIVSDGADHRRRRSVVAPGLRHHHVQRYVATMVSNIDTVIDGWQPGQRLDIYQEL

RSAVRRSTAESLFGQRLAVHSDFLGEQLQPLLDLTRRPPQVMRLQQRVNSPGWRRAMAAR

KRIDDLIDAQIADARTAPRPDDHMLTTLISGCSEEGTTLSDNEIRDSIVSLITAGYETTS

GALAWAIYALLTVPGTWESAASEVARVLGGRVPAADDLSALTYLNGVVHETLRLYSPGVI

SARRVLRDLWFDGHRIRAGRLLIFSAYVTHRLPEIWPEPTEFRPLRWDPNAADYRKPAPH

EFIPFSGGLHRCIGAVMATTEMTVILARLVARAMLQLPAQRTHRIRAANFAALRPWPGLT

VEIRKSAPAQ

>CYP139A1(2576698280)Mycobacterium tuberculosis XTB13-081

MRYPLGEALLALYRWRGPLINAGVGGHGYTYLLGAEANRFVFANADAFSWSQTFESLVPV

DGPTALIVSDGADHRRRRSVVAPGLRHHHVQRYVATMVSNIDTVIDGWQPGQRLDIYQEL

RSAVRRSTAESLFGQRLAVHSDFLGEQLQPLLDLTRRPPQVMRLQQRVNSPGWRRAMAAR

KRIDDLIDAQIADARTAPRPDDHMLTTLISGCSEEGTTLSDNEIRDSIVSLITAGYETTS

GALAWAIYALLTVPGTWESAASEVARVLGGRVPAADDLSALTYLNGVVHETLRLYSPGVI

SARRVLRDLWFDGHRIRAGRLLIFSAYVTHRLPEIWPEPTEFRPLRWDPNAADYRKPAPH

EFIPFSGGLHRCIGAVMATTEMTVILARLVARAMLQLPAQRTHRIRAANFAALRPWPGLT

VEIRKSAPAQ

>CYP139A1(2576759003)Mycobacterium tuberculosis TBR37

MRYPLGEALLALYRWRGPLINAGVGGHGYTYLLGAEANRFVFANADAFSWSQTFESLVPV

DGPTALIVSDGADHRRRRSVVAPGLRHHHVQRYVATMVSNIDTVIDGWQPGQRLDIYQEL

RSAVRRSTAESLFGQRLAVHSDFLGEQLQPLLDLTRRPPQVMRLQQRVNSPGWRRAMAAR

KRIDDLIDAQIADARTAPRPDDHMLTTLISGCSEEGTTLSDNEIRDSIVSLITAGYETTS

GALAWAIYALLTVPGTWESAASEVARVLGGRVPAADDLSALTYLNGVVHETLRLYSPGVI

SARRVLRDLWFDGHRIRAGRLLIFSAYVTHRLPEIWPEPTEFRPLRWDPNAADYRKPAPH

EFIPFSGGLHRCIGAVMATTEMTVILARLVARAMLQLPAQRTHRIRAANFAALRPWPGLT

VEIRKSAPAQ

>CYP139A1(2577673179)Mycobacterium tuberculosis TKK_03_0083

MRYPLGEALLALYRWRGPLINAGVGGHGYTYLLGAEANRFVFANADAFSWSQTFESLVPV

DGPTALIVSDGADHRRRRSVVAPGLRHHHVQRYVATMVSNIDTVIDGWQPGQRLDIYQEL

RSAVRRSTAESLFGQRLAVHSDFLGEQLQPLLDLTRRPPQVMRLQQRVNSPGWRRAMAAR

KRIDDLIDAQIADARTAPRPDDHMLTTLISGCSEEGTTLSDNEIRDSIVSLITAGYETTS

GALAWAIYALLTVPGTWESAASEVARVLGGRVPAADDLSALTYLNGVVHETLRLYSPGVI

SARRVLRDLWFDGHRIRAGRLLIFSAYVTHRLPEIWPEPTEFRPLRWDPNAADYRKPAPH

EFIPFSGGLHRCIGAVMATTEMTVILARLVARAMLQLPAQRTHRIRAANFAALRPWPGLT

VEIRKSAPAQ

>CYP139A1(2577936335)Mycobacterium tuberculosis TKK_03_0118

MRYPLGEALLALYRWRGPLINAGVGGHGYTYLLGAEANRFVFANADAFSWSQTFESLVPV

DGPTALIVSDGADHRRRRSVVAPGLRHHHVQRYVATMVSNIDTVIDGWQPGQRLDIYQEL

RSAVRRSTAESLFGQRLAVHSDFLGEQLQPLLDLTRRPPQVMRLQQRVNSPGWRRAMAAR

KRIDDLIDAQIADARTAPRPDDHMLTTLISGCSEEGTTLSDNEIRDSIVSLITAGYETTS

GALAWAIYALLTVPGTWESAASEVARVLGGRVPAADDLSALTYLNGVVHETLRLYSPGVI

SARRVLRDLWFDGHRIRAGRLLIFSAYVTHRLPEIWPEPTEFRPLRWDPNAADYRKPAPH

EFIPFSGGLHRCIGAVMATTEMTVILARLVARAMLQLPAQRTHRIRAANFAALRPWPGLT

VEIRKSAPAQ

>CYP139A1(2578008273)Mycobacterium tuberculosis TKK-01-0026

MRYPLGEALLALYRWRGPLINAGVGGHGYTYLLGAEANRFVFANADAFSWSQTFESLVPV

DGPTALIVSDGADHRRRRSVVAPGLRHHHVQRYVATMVSNIDTVIDGWQPGQRLDIYQEL

RSAVRRSTAESLFGQRLAVHSDFLGEQLQPLLDLTRRPPQVMRLQQRVNSPGWRRAMAAR

KRIDDLIDAQIADARTAPRPDDHMLTTLISGCSEEGTTLSDNEIRDSIVSLITAGYETTS

GALAWAIYALLTVPGTWESAASEVARVLGGRVPAADDLSALTYLNGVVHETLRLYSPGVI

SARRVLRDLWFDGHRIRAGRLLIFSAYVTHRLPEIWPEPTEFRPLRWDPNAADYRKPAPH

EFIPFSGGLHRCIGAVMATTEMTVILARLVARAMLQLPAQRTHRIRAANFAALRPWPGLT

VEIRKSAPAQ

>CYP139A1(2578073775)Mycobacterium tuberculosis TB_RSA74

MRYPLGEALLALYRWRGPLINAGVGGHGYTYLLGAEANRFVFANADAFSWSQTFESLVPV

DGPTALIVSDGADHRRRRSVVAPGLRHHHVQRYVATMVSNIDTVIDGWQPGQRLDIYQEL

RSAVRRSTAESLFGQRLAVHSDFLGEQLQPLLDLTRRPPQVMRLQQRVNSPGWRRAMAAR

KRIDDLIDAQIADARTAPRPDDHMLTTLISGCSEEGTTLSDNEIRDSIVSLITAGYETTS

GALAWAIYALLTVPGTWESAASEVARVLGGRVPAADDLSALTYLNGVVHETLRLYSPGVI

SARRVLRDLWFDGHRIRAGRLLIFSAYVTHRLPEIWPEPTEFRPLRWDPNAADYRKPAPH

EFIPFSGGLHRCIGAVMATTEMTVILARLVARAMLQLPAQRTHRIRAANFAALRPWPGLT

VEIRKSAPAQ

>CYP139A1(2584960744)Mycobacterium tuberculosis XTB13-290

MRYPLGEALLALYRWRGPLINAGVGGHGYTYLLGAEANRFVFANADAFSWSQTFESLVPV

DGPTALIVSDGADHRRRRSVVAPGLRHHHVQRYVATMVSNIDTVIDGWQPGQRLDIYQEL

RSAVRRSTAESLFGQRLAVHSDFLGEQLQPLLDLTRRPPQVMRLQQRVNSPGWRRAMAAR

KRIDDLIDAQIADARTAPRPDDHMLTTLISGCSEEGTTLSDNEIRDSIVSLITAGYETTS

GALAWAIYALLTVPGTWESAASEVARVLGGRVPAADDLSALTYLNGVVHETLRLYSPGVI

SARRVLRDLWFDGHRIRAGRLLIFSAYVTHRLPEIWPEPTEFRPLRWDPNAADYRKPAPH

EFIPFSGGLHRCIGAVMATTEMTVILARLVARAMLQLPAQRTHRIRAANFAALRPWPGLT

VEIRKSAPAQ

>CYP139A1(2588992251)Mycobacterium tuberculosis TKK-01-0069

MRYPLGEALLALYRWRGPLINAGVGGHGYTYLLGAEANRFVFANADAFSWSQTFESLVPV

DGPTALIVSDGADHRRRRSVVAPGLRHHHVQRYVATMVSNIDTVIDGWQPGQRLDIYQEL

RSAVRRSTAESLFGQRLAVHSDFLGEQLQPLLDLTRRPPQVMRLQQRVNSPGWRRAMAAR

KRIDDLIDAQIADARTAPRPDDHMLTTLISGCSEEGTTLSDNEIRDSIVSLITAGYETTS

GALAWAIYALLTVPGTWESAASEVARVLGGRVPAADDLSALTYLNGVVHETLRLYSPGVI

SARRVLRDLWFDGHRIRAGRLLIFSAYVTHRLPEIWPEPTEFRPLRWDPNAADYRKPAPH

EFIPFSGGLHRCIGAVMATTEMTVILARLVARAMLQLPAQRTHRIRAANFAALRPWPGLT

VEIRKSAPAQ

>CYP139A1(2589077872)Mycobacterium tuberculosis TBR43

MRYPLGEALLALYRWRGPLINAGVGGHGYTYLLGAEANRFVFANADAFSWSQTFESLVPV

DGPTALIVSDGADHRRRRSVVAPGLRHHHVQRYVATMVSNIDTVIDGWQPGQRLDIYQEL

RSAVRRSTAESLFGQRLAVHSDFLGEQLQPLLDLTRRPPQVMRLQQRVNSPGWRRAMAAR

KRIDDLIDAQIADARTAPRPDDHMLTTLISGCSEEGTTLSDNEIRDSIVSLITAGYETTS

GALAWAIYALLTVPGTWESAASEVARVLGGRVPAADDLSALTYLNGVVHETLRLYSPGVI

SARRVLRDLWFDGHRIRAGRLLIFSAYVTHRLPEIWPEPTEFRPLRWDPNAADYRKPAPH

EFIPFSGGLHRCIGAVMATTEMTVILARLVARAMLQLPAQRTHRIRAANFAALRPWPGLT

VEIRKSAPAQ

>CYP139A1(2589518744)Mycobacterium tuberculosis TKK-01-0013

MRYPLGEALLALYRWRGPLINAGVGGHGYTYLLGAEANRFVFANADAFSWSQTFESLVPV

DGPTALIVSDGADHRRRRSVVAPGLRHHHVQRYVATMVSNIDTVIDGWQPGQRLDIYQEL

RSAVRRSTAESLFGQRLAVHSDFLGEQLQPLLDLTRRPPQVMRLQQRVNSPGWRRAMAAR

KRIDDLIDAQIADARTAPRPDDHMLTTLISGCSEEGTTLSDNEIRDSIVSLITAGYETTS

GALAWAIYALLTVPGTWESAASEVARVLGGRVPAADDLSALTYLNGVVHETLRLYSPGVI

SARRVLRDLWFDGHRIRAGRLLIFSAYVTHRLPEIWPEPTEFRPLRWDPNAADYRKPAPH

EFIPFSGGLHRCIGAVMATTEMTVILARLVARAMLQLPAQRTHRIRAANFAALRPWPGLT

VEIRKSAPAQ

>CYP139A1(2589583955)Mycobacterium tuberculosis TKK-01-0042

MRYPLGEALLALYRWRGPLINAGVGGHGYTYLLGAEANRFVFANADAFSWSQTFESLVPV

DGPTALIVSDGADHRRRRSVVAPGLRHHHVQRYVATMVSNIDTVIDGWQPGQRLDIYQEL

RSAVRRSTAESLFGQRLAVHSDFLGEQLQPLLDLTRRPPQVMRLQQRVNSPGWRRAMAAR

KRIDDLIDAQIADARTAPRPDDHMLTTLISGCSEEGTTLSDNEIRDSIVSLITAGYETTS

GALAWAIYALLTVPGTWESAASEVARVLGGRVPAADDLSALTYLNGVVHETLRLYSPGVI

SARRVLRDLWFDGHRIRAGRLLIFSAYVTHRLPEIWPEPTEFRPLRWDPNAADYRKPAPH

EFIPFSGGLHRCIGAVMATTEMTVILARLVARAMLQLPAQRTHRIRAANFAALRPWPGLT

VEIRKSAPAQ

>CYP139A1(2589646609)Mycobacterium tuberculosis TKK-01-0063

MRYPLGEALLALYRWRGPLINAGVGGHGYTYLLGAEANRFVFANADAFSWSQTFESLVPV

DGPTALIVSDGADHRRRRSVVAPGLRHHHVQRYVATMVSNIDTVIDGWQPGQRLDIYQEL

RSAVRRSTAESLFGQRLAVHSDFLGEQLQPLLDLTRRPPQVMRLQQRVNSPGWRRAMAAR

KRIDDLIDAQIADARTAPRPDDHMLTTLISGCSEEGTTLSDNEIRDSIVSLITAGYETTS

GALAWAIYALLTVPGTWESAASEVARVLGGRVPAADDLSALTYLNGVVHETLRLYSPGVI

SARRVLRDLWFDGHRIRAGRLLIFSAYVTHRLPEIWPEPTEFRPLRWDPNAADYRKPAPH

EFIPFSGGLHRCIGAVMATTEMTVILARLVARAMLQLPAQRTHRIRAANFAALRPWPGLT

VEIRKSAPAQ

>CYP139A1(2592316279)Mycobacterium tuberculosis TKK_03_0025

MRYPLGEALLALYRWRGPLINAGVGGHGYTYLLGAEANRFVFANADAFSWSQTFESLVPV

DGPTALIVSDGADHRRRRSVVAPGLRHHHVQRYVATMVSNIDTVIDGWQPGQRLDIYQEL

RSAVRRSTAESLFGQRLAVHSDFLGEQLQPLLDLTRRPPQVMRLQQRVNSPGWRRAMAAR

KRIDDLIDAQIADARTAPRPDDHMLTTLISGCSEEGTTLSDNEIRDSIVSLITAGYETTS

GALAWAIYALLTVPGTWESAASEVARVLGGRVPAADDLSALTYLNGVVHETLRLYSPGVI

SARRVLRDLWFDGHRIRAGRLLIFSAYVTHRLPEIWPEPTEFRPLRWDPNAADYRKPAPH

EFIPFSGGLHRCIGAVMATTEMTVILARLVARAMLQLPAQRTHRIRAANFAALRPWPGLT

VEIRKSAPAQ

>CYP139A1(2592546111)Mycobacterium tuberculosis TKK_04_0046

MRYPLGEALLALYRWRGPLINAGVGGHGYTYLLGAEANRFVFANADAFSWSQTFESLVPV

DGPTALIVSDGADHRRRRSVVAPGLRHHHVQRYVATMVSNIDTVIDGWQPGQRLDIYQEL

RSAVRRSTAESLFGQRLAVHSDFLGEQLQPLLDLTRRPPQVMRLQQRVNSPGWRRAMAAR

KRIDDLIDAQIADARTAPRPDDHMLTTLISGCSEEGTTLSDNEIRDSIVSLITAGYETTS

GALAWAIYALLTVPGTWESAASEVARVLGGRVPAADDLSALTYLNGVVHETLRLYSPGVI

SARRVLRDLWFDGHRIRAGRLLIFSAYVTHRLPEIWPEPTEFRPLRWDPNAADYRKPAPH

EFIPFSGGLHRCIGAVMATTEMTVILARLVARAMLQLPAQRTHRIRAANFAALRPWPGLT

VEIRKSAPAQ

>CYP139A1(2511553315)Mycobacterium tuberculosis KZN 4207(DS)

MRYPLGEALLALYRWRGPLINAGVGGHGYTYLLGAEANRFVFANADAFSWSQTFESLVPV

DGPTALIVSDGADHRRRRSVVAPGLRHHHVQRYVATMVSNIDTVIDGWQPGQRLDIYQEL

RSAVRRSTAESLFGQRLAVHSDFLGEQLQPLLDLTRRPPQVMRLQQRVNSPGWRRAMAAR

KRIDDLIDAQIADARTAPRPDDHMLTTLISGCSEEGTTLSDNEIRDSIVSLITAGYETTS

GALAWAIYALLTVPGTWESAASEVARVLGGRVPAADDLSALTYLNGVVHETLRLYSPGVI

SARRVLRDLWFDGHRIRAGRLLIFSAYVTHRLPEIWPEPTEFRPLRWDPNAADYRKPAPH

EFIPFSGGLHRCIGAVMATTEMTVILARLVARAMLQLPAQRTHRIRAANFAALRPWPGLT

VEIRKSAPAQ

>CYP139A1(2574949227)Mycobacterium tuberculosis KT-0042

MRYPLGEALLALYRWRGPLINAGVGGHGYTYLLGAEANRFVFANADAFSWSQTFESLVPV

DGPTALIVSDGADHRRRRSVVAPGLRHHHVQRYVATMVSNIDTVIDGWQPGQRLDIYQEL

RSAVRRSTAESLFGQRLAVHSDFLGEQLQPLLDLTRRPPQVMRLQQRVNSPGWRRAMAAR

KRIDDLIDAQIADARTAPRPDDHMLTTLISGCSEEGTTLSDNEIRDSIVSLITAGYETTS

GALAWAIYALLTVPGTWESAASEVARVLGGRVPAADDLSALTYLNGVVHETLRLYSPGVI

SARRVLRDLWFDGHRIRAGRLLIFSAYVTHRLPEIWPEPTEFRPLRWDPNAADYRKPAPH

EFIPFSGGLHRCIGAVMATTEMTVILARLVARAMLQLPAQRTHRIRAANFAALRPWPGLT

VEIRKSAPAQ

>CYP139A1(2575533703)Mycobacterium tuberculosis MAL020162

MRYPLGEALLALYRWRGPLINAGVGGHGYTYLLGAEANRFVFANADAFSWSQTFESLVPV

DGPTALIVSDGADHRRRRSVVAPGLRHHHVQRYVATMVSNIDTVIDGWQPGQRLDIYQEL

RSAVRRSTAESLFGQRLAVHSDFLGEQLQPLLDLTRRPPQVMRLQQRVNSPGWRRAMAAR

KRIDDLIDAQIADARTAPRPDDHMLTTLISGCSEEGTTLSDNEIRDSIVSLITAGYETTS

GALAWAIYALLTVPGTWESAASEVARVLGGRVPAADDLSALTYLNGVVHETLRLYSPGVI

SARRVLRDLWFDGHRIRAGRLLIFSAYVTHRLPEIWPEPTEFRPLRWDPNAADYRKPAPH

EFIPFSGGLHRCIGAVMATTEMTVILARLVARAMLQLPAQRTHRIRAANFAALRPWPGLT

VEIRKSAPAQ

>CYP139A1(2576015739)Mycobacterium tuberculosis M1444

MRYPLGEALLALYRWRGPLINAGVGGHGYTYLLGAEANRFVFANADAFSWSQTFESLVPV

DGPTALIVSDGADHRRRRSVVAPGLRHHHVQRYVATMVSNIDTVIDGWQPGQRLDIYQEL

RSAVRRSTAESLFGQRLAVHSDFLGEQLQPLLDLTRRPPQVMRLQQRVNSPGWRRAMAAR

KRIDDLIDAQIADARTAPRPDDHMLTTLISGCSEEGTTLSDNEIRDSIVSLITAGYETTS

GALAWAIYALLTVPGTWESAASEVARVLGGRVPAADDLSALTYLNGVVHETLRLYSPGVI

SARRVLRDLWFDGHRIRAGRLLIFSAYVTHRLPEIWPEPTEFRPLRWDPNAADYRKPAPH

EFIPFSGGLHRCIGAVMATTEMTVILARLVARAMLQLPAQRTHRIRAANFAALRPWPGLT

VEIRKSAPAQ

>CYP139A1(2576644886)Mycobacterium tuberculosis BTB03-143

MRYPLGEALLALYRWRGPLINAGVGGHGYTYLLGAEANRFVFANADAFSWSQTFESLVPV

DGPTALIVSDGADHRRRRSVVAPGLRHHHVQRYVATMVSNIDTVIDGWQPGQRLDIYQEL

RSAVRRSTAESLFGQRLAVHSDFLGEQLQPLLDLTRRPPQVMRLQQRVNSPGWRRAMAAR

KRIDDLIDAQIADARTAPRPDDHMLTTLISGCSEEGTTLSDNEIRDSIVSLITAGYETTS

GALAWAIYALLTVPGTWESAASEVARVLGGRVPAADDLSALTYLNGVVHETLRLYSPGVI

SARRVLRDLWFDGHRIRAGRLLIFSAYVTHRLPEIWPEPTEFRPLRWDPNAADYRKPAPH

EFIPFSGGLHRCIGAVMATTEMTVILARLVARAMLQLPAQRTHRIRAANFAALRPWPGLT

VEIRKSAPAQ

>CYP139A1(2577497204)Mycobacterium tuberculosis M2137

MRYPLGEALLALYRWRGPLINAGVGGHGYTYLLGAEANRFVFANADAFSWSQTFESLVPV

DGPTALIVSDGADHRRRRSVVAPGLRHHHVQRYVATMVSNIDTVIDGWQPGQRLDIYQEL

RSAVRRSTAESLFGQRLAVHSDFLGEQLQPLLDLTRRPPQVMRLQQRVNSPGWRRAMAAR

KRIDDLIDAQIADARTAPRPDDHMLTTLISGCSEEGTTLSDNEIRDSIVSLITAGYETTS

GALAWAIYALLTVPGTWESAASEVARVLGGRVPAADDLSALTYLNGVVHETLRLYSPGVI

SARRVLRDLWFDGHRIRAGRLLIFSAYVTHRLPEIWPEPTEFRPLRWDPNAADYRKPAPH

EFIPFSGGLHRCIGAVMATTEMTVILARLVARAMLQLPAQRTHRIRAANFAALRPWPGLT

VEIRKSAPAQ

>CYP139A1(2580744928)*Mycobacterium bovis*D 4155]

MRYPLGEALLALYRWRGPLINAGVGGHGYTYLLGAEANRFVFANADAFSWSQTFESLVPV

DGPTALIVSDGADHRRRRSVVAPGLRHHHVQRYVATMVSNIDTVIDGWQPGQRLDIYQEL

RSAVRRSTAESLFGQRLAVHSDFLGEQLQPLLDLTRRPPQVMRLQQRVNSPGWRRAMAAR

KRIDDLIDAQIADARTAPRPDDHMLTTLISGCSEEGTTLSDNEIRDSIVSLITAGYETTS

GALAWAIYALLTVPGTWESAASEVARVLGGRVPAADDLSALTYLNGVVHETLRLYSPGVI

SARRVLRDLWFDGHRIRAGRLLIFSAYVTHRLPEIWPEPTEFRPLRWDPNAADYRKPAPH

EFIPFSGGLHRCIGAVMATTEMTVILARLVARAMLQLPAQRTHRIRAANFAALRPWPGLT

VEIRKSAPAQ

>CYP139A1(2583727889)*Mycobacterium africanum* MAL010136

MRYPLGEALLALYRWRGPLINAGVGGHGYTYLLGAEANRFVFANADAFSWSQTFESLVPV

DGPTALIVSDGADHRRRRSVVAPGLRHHHVQRYVATMVSNIDTVIDGWQPGQRLDIYQEL

RSAVRRSTAESLFGQRLAVHSDFLGEQLQPLLDLTRRPPQVMRLQQRVNSPGWRRAMAAR

KRIDDLIDAQIADARTAPRPDDHMLTTLISGCSEEGTTLSDNEIRDSIVSLITAGYETTS

GALAWAIYALLTVPGTWESAASEVARVLGGRVPAADDLSALTYLNGVVHETLRLYSPGVI

SARRVLRDLWFDGHRIRAGRLLIFSAYVTHRLPEIWPEPTEFRPLRWDPNAADYRKPAPH

EFIPFSGGLHRCIGAVMATTEMTVILARLVARAMLQLPAQRTHRIRAANFAALRPWPGLT

VEIRKSAPAQ

>CYP139A1(2590064965)Mycobacterium tuberculosis MAL020132

MRYPLGEALLALYRWRGPLINAGVGGHGYTYLLGAEANRFVFANADAFSWSQTFESLVPV

DGPTALIVSDGADHRRRRSVVAPGLRHHHVQRYVATMVSNIDTVIDGWQPGQRLDIYQEL

RSAVRRSTAESLFGQRLAVHSDFLGEQLQPLLDLTRRPPQVMRLQQRVNSPGWRRAMAAR

KRIDDLIDAQIADARTAPRPDDHMLTTLISGCSEEGTTLSDNEIRDSIVSLITAGYETTS

GALAWAIYALLTVPGTWESAASEVARVLGGRVPAADDLSALTYLNGVVHETLRLYSPGVI

SARRVLRDLWFDGHRIRAGRLLIFSAYVTHRLPEIWPEPTEFRPLRWDPNAADYRKPAPH

EFIPFSGGLHRCIGAVMATTEMTVILARLVARAMLQLPAQRTHRIRAANFAALRPWPGLT

VEIRKSAPAQ

>CYP139A1(2590121989)Mycobacterium tuberculosis MAL020174

MRYPLGEALLALYRWRGPLINAGVGGHGYTYLLGAEANRFVFANADAFSWSQTFESLVPV

DGPTALIVSDGADHRRRRSVVAPGLRHHHVQRYVATMVSNIDTVIDGWQPGQRLDIYQEL

RSAVRRSTAESLFGQRLAVHSDFLGEQLQPLLDLTRRPPQVMRLQQRVNSPGWRRAMAAR

KRIDDLIDAQIADARTAPRPDDHMLTTLISGCSEEGTTLSDNEIRDSIVSLITAGYETTS

GALAWAIYALLTVPGTWESAASEVARVLGGRVPAADDLSALTYLNGVVHETLRLYSPGVI

SARRVLRDLWFDGHRIRAGRLLIFSAYVTHRLPEIWPEPTEFRPLRWDPNAADYRKPAPH

EFIPFSGGLHRCIGAVMATTEMTVILARLVARAMLQLPAQRTHRIRAANFAALRPWPGLT

VEIRKSAPAQ

>CYP139A1(2590294228)Mycobacterium tuberculosis KT-0002

MRYPLGEALLALYRWRGPLINAGVGGHGYTYLLGAEANRFVFANADAFSWSQTFESLVPV

DGPTALIVSDGADHRRRRSVVAPGLRHHHVQRYVATMVSNIDTVIDGWQPGQRLDIYQEL

RSAVRRSTAESLFGQRLAVHSDFLGEQLQPLLDLTRRPPQVMRLQQRVNSPGWRRAMAAR

KRIDDLIDAQIADARTAPRPDDHMLTTLISGCSEEGTTLSDNEIRDSIVSLITAGYETTS

GALAWAIYALLTVPGTWESAASEVARVLGGRVPAADDLSALTYLNGVVHETLRLYSPGVI

SARRVLRDLWFDGHRIRAGRLLIFSAYVTHRLPEIWPEPTEFRPLRWDPNAADYRKPAPH

EFIPFSGGLHRCIGAVMATTEMTVILARLVARAMLQLPAQRTHRIRAANFAALRPWPGLT

VEIRKSAPAQ

>CYP139A1(2590499050)Mycobacterium tuberculosis KT-0106

MRYPLGEALLALYRWRGPLINAGVGGHGYTYLLGAEANRFVFANADAFSWSQTFESLVPV

DGPTALIVSDGADHRRRRSVVAPGLRHHHVQRYVATMVSNIDTVIDGWQPGQRLDIYQEL

RSAVRRSTAESLFGQRLAVHSDFLGEQLQPLLDLTRRPPQVMRLQQRVNSPGWRRAMAAR

KRIDDLIDAQIADARTAPRPDDHMLTTLISGCSEEGTTLSDNEIRDSIVSLITAGYETTS

GALAWAIYALLTVPGTWESAASEVARVLGGRVPAADDLSALTYLNGVVHETLRLYSPGVI

SARRVLRDLWFDGHRIRAGRLLIFSAYVTHRLPEIWPEPTEFRPLRWDPNAADYRKPAPH

EFIPFSGGLHRCIGAVMATTEMTVILARLVARAMLQLPAQRTHRIRAANFAALRPWPGLT

VEIRKSAPAQ

>CYP139A1(2590519464)Mycobacterium tuberculosis KT-0092

MRYPLGEALLALYRWRGPLINAGVGGHGYTYLLGAEANRFVFANADAFSWSQTFESLVPV

DGPTALIVSDGADHRRRRSVVAPGLRHHHVQRYVATMVSNIDTVIDGWQPGQRLDIYQEL

RSAVRRSTAESLFGQRLAVHSDFLGEQLQPLLDLTRRPPQVMRLQQRVNSPGWRRAMAAR

KRIDDLIDAQIADARTAPRPDDHMLTTLISGCSEEGTTLSDNEIRDSIVSLITAGYETTS

GALAWAIYALLTVPGTWESAASEVARVLGGRVPAADDLSALTYLNGVVHETLRLYSPGVI

SARRVLRDLWFDGHRIRAGRLLIFSAYVTHRLPEIWPEPTEFRPLRWDPNAADYRKPAPH

EFIPFSGGLHRCIGAVMATTEMTVILARLVARAMLQLPAQRTHRIRAANFAALRPWPGLT

VEIRKSAPAQ

>CYP139A1(2590548005)Mycobacterium tuberculosis KT-0078

MRYPLGEALLALYRWRGPLINAGVGGHGYTYLLGAEANRFVFANADAFSWSQTFESLVPV

DGPTALIVSDGADHRRRRSVVAPGLRHHHVQRYVATMVSNIDTVIDGWQPGQRLDIYQEL

RSAVRRSTAESLFGQRLAVHSDFLGEQLQPLLDLTRRPPQVMRLQQRVNSPGWRRAMAAR

KRIDDLIDAQIADARTAPRPDDHMLTTLISGCSEEGTTLSDNEIRDSIVSLITAGYETTS

GALAWAIYALLTVPGTWESAASEVARVLGGRVPAADDLSALTYLNGVVHETLRLYSPGVI

SARRVLRDLWFDGHRIRAGRLLIFSAYVTHRLPEIWPEPTEFRPLRWDPNAADYRKPAPH

EFIPFSGGLHRCIGAVMATTEMTVILARLVARAMLQLPAQRTHRIRAANFAALRPWPGLT

VEIRKSAPAQ

>CYP139A1(637139034)*Mycobacterium bovis*AF2122/97

MRYPLGEALLALYRWRGPLINAGVGGHGYTYLLGAEANRFVFANADAFSWSQTFESLVPV

DGPTALIVSDGADHRRRRSVVAPGLRHHHVQRYVATMVSNIDTVIDGWQPGQRLDIYQEL

RSAVRRSTAESLFGQRLAVHSDFLGEQLQPLLDLTRRPPQVMRLQQRVNSPGWRRAMAAR

KRIDDLIDAQIADARTAPRPDDHMLTTLISGCSEEGTTLSDNEIRDSIVSLITAGYETTS

GALAWAIYALLTVPGTWESAASEVARVLGGRVPAADDLSALTYLNGVVHETLRLYSPGVI

SARRVLRDLWFDGHRIRAGRLLIFSAYVTHRLPEIWPEPTEFRPLRWDPNAADYRKPAPH

EFIPFSGGLHRCIGAVMATTEMTVILARLVARAMLQLPAQRTHRIRAANFAALRPWPGLT

VEIRKSAPAQ

>CYP139A1(639830617)*Mycobacterium bovis*BCG str. Pasteur 1173P2

MRYPLGEALLALYRWRGPLINAGVGGHGYTYLLGAEANRFVFANADAFSWSQTFESLVPV

DGPTALIVSDGADHRRRRSVVAPGLRHHHVQRYVATMVSNIDTVIDGWQPGQRLDIYQEL

RSAVRRSTAESLFGQRLAVHSDFLGEQLQPLLDLTRRPPQVMRLQQRVNSPGWRRAMAAR

KRIDDLIDAQIADARTAPRPDDHMLTTLISGCSEEGTTLSDNEIRDSIVSLITAGYETTS

GALAWAIYALLTVPGTWESAASEVARVLGGRVPAADDLSALTYLNGVVHETLRLYSPGVI

SARRVLRDLWFDGHRIRAGRLLIFSAYVTHRLPEIWPEPTEFRPLRWDPNAADYRKPAPH

EFIPFSGGLHRCIGAVMATTEMTVILARLVARAMLQLPAQRTHRIRAANFAALRPWPGLT

VEIRKSAPAQ

>CYP139A1(2574640348)Mycobacterium tuberculosis TKK_04_0120

MRYPLGEALLALYRWRGPLINAGVGGHGYTYLLGAEANRFVFANADAFSWSQTFESLVPV

DGPTALIVSDGADHRRRRSVVAPGLRHHHVQRYVATMVSNIDTVIDGWQPGQRLDIYQEL

RSAVRRSTAESLFGQRLAVHSDFLGEQLQPLLDLTRRPPQVMRLQQRVNSPGWRRAMAAR

KRIDDLIDAQIADARTAPRPDDHMLTTLISGCSEEGTTLSDNEIRDSIVSLITAGYETTS

GALAWAIYALLTVPGTWESAASEVARVLGGRVPAADDLSALTYLNGVVHETLRLYSPGVI

SARRVLRDLWFDGHRIRAGRLLIFSAYVTHRLPEIWPEPTEFRPLRWDPNAADYRKPAPH

EFIPFSGGLHRCIGAVMATTEMTVILARLVARAMLQLPAQRTHRIRAANFAALRPWPGLT

VEIRKSAPAQ

>CYP139A1(2574794413)Mycobacterium tuberculosis XTB13-114

MRYPLGEALLALYRWRGPLINAGVGGHGYTYLLGAEANRFVFANADAFSWSQTFESLVPV

DGPTALIVSDGADHRRRRSVVAPGLRHHHVQRYVATMVSNIDTVIDGWQPGQRLDIYQEL

RSAVRRSTAESLFGQRLAVHSDFLGEQLQPLLDLTRRPPQVMRLQQRVNSPGWRRAMAAR

KRIDDLIDAQIADARTAPRPDDHMLTTLISGCSEEGTTLSDNEIRDSIVSLITAGYETTS

GALAWAIYALLTVPGTWESAASEVARVLGGRVPAADDLSALTYLNGVVHETLRLYSPGVI

SARRVLRDLWFDGHRIRAGRLLIFSAYVTHRLPEIWPEPTEFRPLRWDPNAADYRKPAPH

EFIPFSGGLHRCIGAVMATTEMTVILARLVARAMLQLPAQRTHRIRAANFAALRPWPGLT

VEIRKSAPAQ

>CYP139A1(2574987296)Mycobacterium tuberculosis MD18498

MRYPLGEALLALYRWRGPLINAGVGGHGYTYLLGAEANRFVFANADAFSWSQTFESLVPV

DGPTALIVSDGADHRRRRSVVAPGLRHHHVQRYVATMVSNIDTVIDGWQPGQRLDIYQEL

RSAVRRSTAESLFGQRLAVHSDFLGEQLQPLLDLTRRPPQVMRLQQRVNSPGWRRAMAAR

KRIDDLIDAQIADARTAPRPDDHMLTTLISGCSEEGTTLSDNEIRDSIVSLITAGYETTS

GALAWAIYALLTVPGTWESAASEVARVLGGRVPAADDLSALTYLNGVVHETLRLYSPGVI

SARRVLRDLWFDGHRIRAGRLLIFSAYVTHRLPEIWPEPTEFRPLRWDPNAADYRKPAPH

EFIPFSGGLHRCIGAVMATTEMTVILARLVARAMLQLPAQRTHRIRAANFAALRPWPGLT

VEIRKSAPAQ

>CYP139A1(2575084753)Mycobacterium tuberculosis MD17888

MRYPLGEALLALYRWRGPLINAGVGGHGYTYLLGAEANRFVFANADAFSWSQTFESLVPV

DGPTALIVSDGADHRRRRSVVAPGLRHHHVQRYVATMVSNIDTVIDGWQPGQRLDIYQEL

RSAVRRSTAESLFGQRLAVHSDFLGEQLQPLLDLTRRPPQVMRLQQRVNSPGWRRAMAAR

KRIDDLIDAQIADARTAPRPDDHMLTTLISGCSEEGTTLSDNEIRDSIVSLITAGYETTS

GALAWAIYALLTVPGTWESAASEVARVLGGRVPAADDLSALTYLNGVVHETLRLYSPGVI

SARRVLRDLWFDGHRIRAGRLLIFSAYVTHRLPEIWPEPTEFRPLRWDPNAADYRKPAPH

EFIPFSGGLHRCIGAVMATTEMTVILARLVARAMLQLPAQRTHRIRAANFAALRPWPGLT

VEIRKSAPAQ

>CYP139A1(2575627312)Mycobacterium tuberculosis TB_RSA134

MRYPLGEALLALYRWRGPLINAGVGGHGYTYLLGAEANRFVFANADAFSWSQTFESLVPV

DGPTALIVSDGADHRRRRSVVAPGLRHHHVQRYVATMVSNIDTVIDGWQPGQRLDIYQEL

RSAVRRSTAESLFGQRLAVHSDFLGEQLQPLLDLTRRPPQVMRLQQRVNSPGWRRAMAAR

KRIDDLIDAQIADARTAPRPDDHMLTTLISGCSEEGTTLSDNEIRDSIVSLITAGYETTS

GALAWAIYALLTVPGTWESAASEVARVLGGRVPAADDLSALTYLNGVVHETLRLYSPGVI

SARRVLRDLWFDGHRIRAGRLLIFSAYVTHRLPEIWPEPTEFRPLRWDPNAADYRKPAPH

EFIPFSGGLHRCIGAVMATTEMTVILARLVARAMLQLPAQRTHRIRAANFAALRPWPGLT

VEIRKSAPAQ

>CYP139A1(2576321347)Mycobacterium tuberculosis TB_RSA76

MRYPLGEALLALYRWRGPLINAGVGGHGYTYLLGAEANRFVFANADAFSWSQTFESLVPV

DGPTALIVSDGADHRRRRSVVAPGLRHHHVQRYVATMVSNIDTVIDGWQPGQRLDIYQEL

RSAVRRSTAESLFGQRLAVHSDFLGEQLQPLLDLTRRPPQVMRLQQRVNSPGWRRAMAAR

KRIDDLIDAQIADARTAPRPDDHMLTTLISGCSEEGTTLSDNEIRDSIVSLITAGYETTS

GALAWAIYALLTVPGTWESAASEVARVLGGRVPAADDLSALTYLNGVVHETLRLYSPGVI

SARRVLRDLWFDGHRIRAGRLLIFSAYVTHRLPEIWPEPTEFRPLRWDPNAADYRKPAPH

EFIPFSGGLHRCIGAVMATTEMTVILARLVARAMLQLPAQRTHRIRAANFAALRPWPGLT

VEIRKSAPAQ

>CYP139A1(2576378950)Mycobacterium tuberculosis TKK_04_0051

MRYPLGEALLALYRWRGPLINAGVGGHGYTYLLGAEANRFVFANADAFSWSQTFESLVPV

DGPTALIVSDGADHRRRRSVVAPGLRHHHVQRYVATMVSNIDTVIDGWQPGQRLDIYQEL

RSAVRRSTAESLFGQRLAVHSDFLGEQLQPLLDLTRRPPQVMRLQQRVNSPGWRRAMAAR

KRIDDLIDAQIADARTAPRPDDHMLTTLISGCSEEGTTLSDNEIRDSIVSLITAGYETTS

GALAWAIYALLTVPGTWESAASEVARVLGGRVPAADDLSALTYLNGVVHETLRLYSPGVI

SARRVLRDLWFDGHRIRAGRLLIFSAYVTHRLPEIWPEPTEFRPLRWDPNAADYRKPAPH

EFIPFSGGLHRCIGAVMATTEMTVILARLVARAMLQLPAQRTHRIRAANFAALRPWPGLT

VEIRKSAPAQ

>CYP139A1(2576735552)Mycobacterium tuberculosis SK-B

MRYPLGEALLALYRWRGPLINAGVGGHGYTYLLGAEANRFVFANADAFSWSQTFESLVPV

DGPTALIVSDGADHRRRRSVVAPGLRHHHVQRYVATMVSNIDTVIDGWQPGQRLDIYQEL

RSAVRRSTAESLFGQRLAVHSDFLGEQLQPLLDLTRRPPQVMRLQQRVNSPGWRRAMAAR

KRIDDLIDAQIADARTAPRPDDHMLTTLISGCSEEGTTLSDNEIRDSIVSLITAGYETTS

GALAWAIYALLTVPGTWESAASEVARVLGGRVPAADDLSALTYLNGVVHETLRLYSPGVI

SARRVLRDLWFDGHRIRAGRLLIFSAYVTHRLPEIWPEPTEFRPLRWDPNAADYRKPAPH

EFIPFSGGLHRCIGAVMATTEMTVILARLVARAMLQLPAQRTHRIRAANFAALRPWPGLT

VEIRKSAPAQ

>CYP139A1(2576940434)Mycobacterium tuberculosis TKK_04_0040

MRYPLGEALLALYRWRGPLINAGVGGHGYTYLLGAEANRFVFANADAFSWSQTFESLVPV

DGPTALIVSDGADHRRRRSVVAPGLRHHHVQRYVATMVSNIDTVIDGWQPGQRLDIYQEL

RSAVRRSTAESLFGQRLAVHSDFLGEQLQPLLDLTRRPPQVMRLQQRVNSPGWRRAMAAR

KRIDDLIDAQIADARTAPRPDDHMLTTLISGCSEEGTTLSDNEIRDSIVSLITAGYETTS

GALAWAIYALLTVPGTWESAASEVARVLGGRVPAADDLSALTYLNGVVHETLRLYSPGVI

SARRVLRDLWFDGHRIRAGRLLIFSAYVTHRLPEIWPEPTEFRPLRWDPNAADYRKPAPH

EFIPFSGGLHRCIGAVMATTEMTVILARLVARAMLQLPAQRTHRIRAANFAALRPWPGLT

VEIRKSAPAQ

>CYP139A1(2577812038)Mycobacterium tuberculosis TB_RSA21

MRYPLGEALLALYRWRGPLINAGVGGHGYTYLLGAEANRFVFANADAFSWSQTFESLVPV

DGPTALIVSDGADHRRRRSVVAPGLRHHHVQRYVATMVSNIDTVIDGWQPGQRLDIYQEL

RSAVRRSTAESLFGQRLAVHSDFLGEQLQPLLDLTRRPPQVMRLQQRVNSPGWRRAMAAR

KRIDDLIDAQIADARTAPRPDDHMLTTLISGCSEEGTTLSDNEIRDSIVSLITAGYETTS

GALAWAIYALLTVPGTWESAASEVARVLGGRVPAADDLSALTYLNGVVHETLRLYSPGVI

SARRVLRDLWFDGHRIRAGRLLIFSAYVTHRLPEIWPEPTEFRPLRWDPNAADYRKPAPH

EFIPFSGGLHRCIGAVMATTEMTVILARLVARAMLQLPAQRTHRIRAANFAALRPWPGLT

VEIRKSAPAQ

>CYP139A1(2577861791)Mycobacterium tuberculosis TKK-01-0037

MRYPLGEALLALYRWRGPLINAGVGGHGYTYLLGAEANRFVFANADAFSWSQTFESLVPV

DGPTALIVSDGADHRRRRSVVAPGLRHHHVQRYVATMVSNIDTVIDGWQPGQRLDIYQEL

RSAVRRSTAESLFGQRLAVHSDFLGEQLQPLLDLTRRPPQVMRLQQRVNSPGWRRAMAAR

KRIDDLIDAQIADARTAPRPDDHMLTTLISGCSEEGTTLSDNEIRDSIVSLITAGYETTS

GALAWAIYALLTVPGTWESAASEVARVLGGRVPAADDLSALTYLNGVVHETLRLYSPGVI

SARRVLRDLWFDGHRIRAGRLLIFSAYVTHRLPEIWPEPTEFRPLRWDPNAADYRKPAPH

EFIPFSGGLHRCIGAVMATTEMTVILARLVARAMLQLPAQRTHRIRAANFAALRPWPGLT

VEIRKSAPAQ

>CYP139A1(2578053379)Mycobacterium tuberculosis TB_RSA78

MRYPLGEALLALYRWRGPLINAGVGGHGYTYLLGAEANRFVFANADAFSWSQTFESLVPV

DGPTALIVSDGADHRRRRSVVAPGLRHHHVQRYVATMVSNIDTVIDGWQPGQRLDIYQEL

RSAVRRSTAESLFGQRLAVHSDFLGEQLQPLLDLTRRPPQVMRLQQRVNSPGWRRAMAAR

KRIDDLIDAQIADARTAPRPDDHMLTTLISGCSEEGTTLSDNEIRDSIVSLITAGYETTS

GALAWAIYALLTVPGTWESAASEVARVLGGRVPAADDLSALTYLNGVVHETLRLYSPGVI

SARRVLRDLWFDGHRIRAGRLLIFSAYVTHRLPEIWPEPTEFRPLRWDPNAADYRKPAPH

EFIPFSGGLHRCIGAVMATTEMTVILARLVARAMLQLPAQRTHRIRAANFAALRPWPGLT

VEIRKSAPAQ

>CYP139A1(2584860925)Mycobacterium tuberculosis TKK_05SA_0042

MRYPLGEALLALYRWRGPLINAGVGGHGYTYLLGAEANRFVFANADAFSWSQTFESLVPV

DGPTALIVSDGADHRRRRSVVAPGLRHHHVQRYVATMVSNIDTVIDGWQPGQRLDIYQEL

RSAVRRSTAESLFGQRLAVHSDFLGEQLQPLLDLTRRPPQVMRLQQRVNSPGWRRAMAAR

KRIDDLIDAQIADARTAPRPDDHMLTTLISGCSEEGTTLSDNEIRDSIVSLITAGYETTS

GALAWAIYALLTVPGTWESAASEVARVLGGRVPAADDLSALTYLNGVVHETLRLYSPGVI

SARRVLRDLWFDGHRIRAGRLLIFSAYVTHRLPEIWPEPTEFRPLRWDPNAADYRKPAPH

EFIPFSGGLHRCIGAVMATTEMTVILARLVARAMLQLPAQRTHRIRAANFAALRPWPGLT

VEIRKSAPAQ

>CYP139A1(2584936325)Mycobacterium tuberculosis XTB13-110

MRYPLGEALLALYRWRGPLINAGVGGHGYTYLLGAEANRFVFANADAFSWSQTFESLVPV

DGPTALIVSDGADHRRRRSVVAPGLRHHHVQRYVATMVSNIDTVIDGWQPGQRLDIYQEL

RSAVRRSTAESLFGQRLAVHSDFLGEQLQPLLDLTRRPPQVMRLQQRVNSPGWRRAMAAR

KRIDDLIDAQIADARTAPRPDDHMLTTLISGCSEEGTTLSDNEIRDSIVSLITAGYETTS

GALAWAIYALLTVPGTWESAASEVARVLGGRVPAADDLSALTYLNGVVHETLRLYSPGVI

SARRVLRDLWFDGHRIRAGRLLIFSAYVTHRLPEIWPEPTEFRPLRWDPNAADYRKPAPH

EFIPFSGGLHRCIGAVMATTEMTVILARLVARAMLQLPAQRTHRIRAANFAALRPWPGLT

VEIRKSAPAQ

>CYP139A1(2584939524)Mycobacterium tuberculosis TKK_02_0014

MRYPLGEALLALYRWRGPLINAGVGGHGYTYLLGAEANRFVFANADAFSWSQTFESLVPV

DGPTALIVSDGADHRRRRSVVAPGLRHHHVQRYVATMVSNIDTVIDGWQPGQRLDIYQEL

RSAVRRSTAESLFGQRLAVHSDFLGEQLQPLLDLTRRPPQVMRLQQRVNSPGWRRAMAAR

KRIDDLIDAQIADARTAPRPDDHMLTTLISGCSEEGTTLSDNEIRDSIVSLITAGYETTS

GALAWAIYALLTVPGTWESAASEVARVLGGRVPAADDLSALTYLNGVVHETLRLYSPGVI

SARRVLRDLWFDGHRIRAGRLLIFSAYVTHRLPEIWPEPTEFRPLRWDPNAADYRKPAPH

EFIPFSGGLHRCIGAVMATTEMTVILARLVARAMLQLPAQRTHRIRAANFAALRPWPGLT

VEIRKSAPAQ

>CYP139A1(2589106145)Mycobacterium tuberculosis TBR53

MRYPLGEALLALYRWRGPLINAGVGGHGYTYLLGAEANRFVFANADAFSWSQTFESLVPV

DGPTALIVSDGADHRRRRSVVAPGLRHHHVQRYVATMVSNIDTVIDGWQPGQRLDIYQEL

RSAVRRSTAESLFGQRLAVHSDFLGEQLQPLLDLTRRPPQVMRLQQRVNSPGWRRAMAAR

KRIDDLIDAQIADARTAPRPDDHMLTTLISGCSEEGTTLSDNEIRDSIVSLITAGYETTS

GALAWAIYALLTVPGTWESAASEVARVLGGRVPAADDLSALTYLNGVVHETLRLYSPGVI

SARRVLRDLWFDGHRIRAGRLLIFSAYVTHRLPEIWPEPTEFRPLRWDPNAADYRKPAPH

EFIPFSGGLHRCIGAVMATTEMTVILARLVARAMLQLPAQRTHRIRAANFAALRPWPGLT

VEIRKSAPAQ

>CYP139A1(2589543202)Mycobacterium tuberculosis TKK-01-0024

MRYPLGEALLALYRWRGPLINAGVGGHGYTYLLGAEANRFVFANADAFSWSQTFESLVPV

DGPTALIVSDGADHRRRRSVVAPGLRHHHVQRYVATMVSNIDTVIDGWQPGQRLDIYQEL

RSAVRRSTAESLFGQRLAVHSDFLGEQLQPLLDLTRRPPQVMRLQQRVNSPGWRRAMAAR

KRIDDLIDAQIADARTAPRPDDHMLTTLISGCSEEGTTLSDNEIRDSIVSLITAGYETTS

GALAWAIYALLTVPGTWESAASEVARVLGGRVPAADDLSALTYLNGVVHETLRLYSPGVI

SARRVLRDLWFDGHRIRAGRLLIFSAYVTHRLPEIWPEPTEFRPLRWDPNAADYRKPAPH

EFIPFSGGLHRCIGAVMATTEMTVILARLVARAMLQLPAQRTHRIRAANFAALRPWPGLT

VEIRKSAPAQ

>CYP139A1(2589642534)Mycobacterium tuberculosis TKK-01-0062

MRYPLGEALLALYRWRGPLINAGVGGHGYTYLLGAEANRFVFANADAFSWSQTFESLVPV

DGPTALIVSDGADHRRRRSVVAPGLRHHHVQRYVATMVSNIDTVIDGWQPGQRLDIYQEL

RSAVRRSTAESLFGQRLAVHSDFLGEQLQPLLDLTRRPPQVMRLQQRVNSPGWRRAMAAR

KRIDDLIDAQIADARTAPRPDDHMLTTLISGCSEEGTTLSDNEIRDSIVSLITAGYETTS

GALAWAIYALLTVPGTWESAASEVARVLGGRVPAADDLSALTYLNGVVHETLRLYSPGVI

SARRVLRDLWFDGHRIRAGRLLIFSAYVTHRLPEIWPEPTEFRPLRWDPNAADYRKPAPH

EFIPFSGGLHRCIGAVMATTEMTVILARLVARAMLQLPAQRTHRIRAANFAALRPWPGLT

VEIRKSAPAQ

>CYP139A1(2589691243)Mycobacterium tuberculosis TKK-01-0081

MRYPLGEALLALYRWRGPLINAGVGGHGYTYLLGAEANRFVFANADAFSWSQTFESLVPV

DGPTALIVSDGADHRRRRSVVAPGLRHHHVQRYVATMVSNIDTVIDGWQPGQRLDIYQEL

RSAVRRSTAESLFGQRLAVHSDFLGEQLQPLLDLTRRPPQVMRLQQRVNSPGWRRAMAAR

KRIDDLIDAQIADARTAPRPDDHMLTTLISGCSEEGTTLSDNEIRDSIVSLITAGYETTS

GALAWAIYALLTVPGTWESAASEVARVLGGRVPAADDLSALTYLNGVVHETLRLYSPGVI

SARRVLRDLWFDGHRIRAGRLLIFSAYVTHRLPEIWPEPTEFRPLRWDPNAADYRKPAPH

EFIPFSGGLHRCIGAVMATTEMTVILARLVARAMLQLPAQRTHRIRAANFAALRPWPGLT

VEIRKSAPAQ

>CYP139A1(2589695547)Mycobacterium tuberculosis TKK-01-0089

MRYPLGEALLALYRWRGPLINAGVGGHGYTYLLGAEANRFVFANADAFSWSQTFESLVPV

DGPTALIVSDGADHRRRRSVVAPGLRHHHVQRYVATMVSNIDTVIDGWQPGQRLDIYQEL

RSAVRRSTAESLFGQRLAVHSDFLGEQLQPLLDLTRRPPQVMRLQQRVNSPGWRRAMAAR

KRIDDLIDAQIADARTAPRPDDHMLTTLISGCSEEGTTLSDNEIRDSIVSLITAGYETTS

GALAWAIYALLTVPGTWESAASEVARVLGGRVPAADDLSALTYLNGVVHETLRLYSPGVI

SARRVLRDLWFDGHRIRAGRLLIFSAYVTHRLPEIWPEPTEFRPLRWDPNAADYRKPAPH

EFIPFSGGLHRCIGAVMATTEMTVILARLVARAMLQLPAQRTHRIRAANFAALRPWPGLT

VEIRKSAPAQ

>CYP139A1(2592222422)Mycobacterium tuberculosis TKK_04_0023

MRYPLGEALLALYRWRGPLINAGVGGHGYTYLLGAEANRFVFANADAFSWSQTFESLVPV

DGPTALIVSDGADHRRRRSVVAPGLRHHHVQRYVATMVSNIDTVIDGWQPGQRLDIYQEL

RSAVRRSTAESLFGQRLAVHSDFLGEQLQPLLDLTRRPPQVMRLQQRVNSPGWRRAMAAR

KRIDDLIDAQIADARTAPRPDDHMLTTLISGCSEEGTTLSDNEIRDSIVSLITAGYETTS

GALAWAIYALLTVPGTWESAASEVARVLGGRVPAADDLSALTYLNGVVHETLRLYSPGVI

SARRVLRDLWFDGHRIRAGRLLIFSAYVTHRLPEIWPEPTEFRPLRWDPNAADYRKPAPH

EFIPFSGGLHRCIGAVMATTEMTVILARLVARAMLQLPAQRTHRIRAANFAALRPWPGLT

VEIRKSAPAQ

>CYP139A1(648446923)Mycobacterium tuberculosis SUMu002

MRYPLGEALLALYRWRGPLINAGVGGHGYTYLLGAEANRFVFANADAFSWSQTFESLVPV

DGPTALIVSDGADHRRRRSVVAPGLRHHHVQRYVATMVSNIDTVIDGWQPGQRLDIYQEL

RSAVRRSTAESLFGQRLAVHSDFLGEQLQPLLDLTRRPPQVMRLQQRVNSPGWRRAMAAR

KRIDDLIDAQIADARTAPRPDDHMLTTLISGCSEEGTTLSDNEIRDSIVSLITAGYETTS

GALAWAIYALLTVPGTWESAASEVARVLGGRVPAADDLSALTYLNGVVHETLRLYSPGVI

SARRVLRDLWFDGHRIRAGRLLIFSAYVTHRLPEIWPEPTEFRPLRWDPNAADYRKPAPH

EFIPFSGGLHRCIGAVMATTEMTVILARLVARAMLQLPAQRTHRIRAANFAALRPWPGLT

VEIRKSAPAQ

>CYP139A1(648469578)Mycobacterium tuberculosis SUMu007

MRYPLGEALLALYRWRGPLINAGVGGHGYTYLLGAEANRFVFANADAFSWSQTFESLVPV

DGPTALIVSDGADHRRRRSVVAPGLRHHHVQRYVATMVSNIDTVIDGWQPGQRLDIYQEL

RSAVRRSTAESLFGQRLAVHSDFLGEQLQPLLDLTRRPPQVMRLQQRVNSPGWRRAMAAR

KRIDDLIDAQIADARTAPRPDDHMLTTLISGCSEEGTTLSDNEIRDSIVSLITAGYETTS

GALAWAIYALLTVPGTWESAASEVARVLGGRVPAADDLSALTYLNGVVHETLRLYSPGVI

SARRVLRDLWFDGHRIRAGRLLIFSAYVTHRLPEIWPEPTEFRPLRWDPNAADYRKPAPH

EFIPFSGGLHRCIGAVMATTEMTVILARLVARAMLQLPAQRTHRIRAANFAALRPWPGLT

VEIRKSAPAQ

>CYP139A1(2546188127)Mycobacterium tuberculosis Beijing/NITR203

MRYPLGEALLALYRWRGPLINAGVGGHGYTYLLGAEANRFVFANADAFSWSQTFESLVPV

DGPTALIVSDGADHRRRRSVVAPGLRHHHVQRYVATMVSNIDTVIDGWQPGQRLDIYQEL

RSAVRRSTAESLFGQRLAVHSDFLGEQLQPLLDLTRRPPQVMRLQQRVNSPGWRRAMAAR

KRIDDLIDAQIADARTAPRPDDHMLTTLISGCSEEGTTLSDNEIRDSIVSLITAGYETTS

GALAWAIYALLTVPGTWESAASEVARVLGGRVPAADDLSALTYLNGVVHETLRLYSPGVI

SARRVLRDLWFDGHRIRAGRLLIFSAYVTHRLPEIWPEPTEFRPLRWDPNAADYRKPAPH

EFIPFSGGLHRCIGAVMATTEMTVILARLVARAMLQLPAQRTHRIRAANFAALRPWPGLT

VEIRKSAPAQ

>CYP139A1(2574773054)Mycobacterium tuberculosis M1848

MRYPLGEALLALYRWRGPLINAGVGGHGYTYLLGAEANRFVFANADAFSWSQTFESLVPV

DGPTALIVSDGADHRRRRSVVAPGLRHHHVQRYVATMVSNIDTVIDGWQPGQRLDIYQEL

RSAVRRSTAESLFGQRLAVHSDFLGEQLQPLLDLTRRPPQVMRLQQRVNSPGWRRAMAAR

KRIDDLIDAQIADARTAPRPDDHMLTTLISGCSEEGTTLSDNEIRDSIVSLITAGYETTS

GALAWAIYALLTVPGTWESAASEVARVLGGRVPAADDLSALTYLNGVVHETLRLYSPGVI

SARRVLRDLWFDGHRIRAGRLLIFSAYVTHRLPEIWPEPTEFRPLRWDPNAADYRKPAPH

EFIPFSGGLHRCIGAVMATTEMTVILARLVARAMLQLPAQRTHRIRAANFAALRPWPGLT

VEIRKSAPAQ

>CYP139A1(2574854728)Mycobacterium tuberculosis M2402

MRYPLGEALLALYRWRGPLINAGVGGHGYTYLLGAEANRFVFANADAFSWSQTFESLVPV

DGPTALIVSDGADHRRRRSVVAPGLRHHHVQRYVATMVSNIDTVIDGWQPGQRLDIYQEL

RSAVRRSTAESLFGQRLAVHSDFLGEQLQPLLDLTRRPPQVMRLQQRVNSPGWRRAMAAR

KRIDDLIDAQIADARTAPRPDDHMLTTLISGCSEEGTTLSDNEIRDSIVSLITAGYETTS

GALAWAIYALLTVPGTWESAASEVARVLGGRVPAADDLSALTYLNGVVHETLRLYSPGVI

SARRVLRDLWFDGHRIRAGRLLIFSAYVTHRLPEIWPEPTEFRPLRWDPNAADYRKPAPH

EFIPFSGGLHRCIGAVMATTEMTVILARLVARAMLQLPAQRTHRIRAANFAALRPWPGLT

VEIRKSAPAQ

>CYP139A1(2575984510)Mycobacterium tuberculosis M2136

MRYPLGEALLALYRWRGPLINAGVGGHGYTYLLGAEANRFVFANADAFSWSQTFESLVPV

DGPTALIVSDGADHRRRRSVVAPGLRHHHVQRYVATMVSNIDTVIDGWQPGQRLDIYQEL

RSAVRRSTAESLFGQRLAVHSDFLGEQLQPLLDLTRRPPQVMRLQQRVNSPGWRRAMAAR

KRIDDLIDAQIADARTAPRPDDHMLTTLISGCSEEGTTLSDNEIRDSIVSLITAGYETTS

GALAWAIYALLTVPGTWESAASEVARVLGGRVPAADDLSALTYLNGVVHETLRLYSPGVI

SARRVLRDLWFDGHRIRAGRLLIFSAYVTHRLPEIWPEPTEFRPLRWDPNAADYRKPAPH

EFIPFSGGLHRCIGAVMATTEMTVILARLVARAMLQLPAQRTHRIRAANFAALRPWPGLT

VEIRKSAPAQ

>CYP139A1(2576293562)Mycobacterium tuberculosis BTB10-308

MRYPLGEALLALYRWRGPLINAGVGGHGYTYLLGAEANRFVFANADAFSWSQTFESLVPV

DGPTALIVSDGADHRRRRSVVAPGLRHHHVQRYVATMVSNIDTVIDGWQPGQRLDIYQEL

RSAVRRSTAESLFGQRLAVHSDFLGEQLQPLLDLTRRPPQVMRLQQRVNSPGWRRAMAAR

KRIDDLIDAQIADARTAPRPDDHMLTTLISGCSEEGTTLSDNEIRDSIVSLITAGYETTS

GALAWAIYALLTVPGTWESAASEVARVLGGRVPAADDLSALTYLNGVVHETLRLYSPGVI

SARRVLRDLWFDGHRIRAGRLLIFSAYVTHRLPEIWPEPTEFRPLRWDPNAADYRKPAPH

EFIPFSGGLHRCIGAVMATTEMTVILARLVARAMLQLPAQRTHRIRAANFAALRPWPGLT

VEIRKSAPAQ

>CYP139A1(2576717872)Mycobacterium tuberculosis BTB11-214

MRYPLGEALLALYRWRGPLINAGVGGHGYTYLLGAEANRFVFANADAFSWSQTFESLVPV

DGPTALIVSDGADHRRRRSVVAPGLRHHHVQRYVATMVSNIDTVIDGWQPGQRLDIYQEL

RSAVRRSTAESLFGQRLAVHSDFLGEQLQPLLDLTRRPPQVMRLQQRVNSPGWRRAMAAR

KRIDDLIDAQIADARTAPRPDDHMLTTLISGCSEEGTTLSDNEIRDSIVSLITAGYETTS

GALAWAIYALLTVPGTWESAASEVARVLGGRVPAADDLSALTYLNGVVHETLRLYSPGVI

SARRVLRDLWFDGHRIRAGRLLIFSAYVTHRLPEIWPEPTEFRPLRWDPNAADYRKPAPH

EFIPFSGGLHRCIGAVMATTEMTVILARLVARAMLQLPAQRTHRIRAANFAALRPWPGLT

VEIRKSAPAQ

>CYP139A1(2578189802)Mycobacterium tuberculosis KT-0096

MRYPLGEALLALYRWRGPLINAGVGGHGYTYLLGAEANRFVFANADAFSWSQTFESLVPV

DGPTALIVSDGADHRRRRSVVAPGLRHHHVQRYVATMVSNIDTVIDGWQPGQRLDIYQEL

RSAVRRSTAESLFGQRLAVHSDFLGEQLQPLLDLTRRPPQVMRLQQRVNSPGWRRAMAAR

KRIDDLIDAQIADARTAPRPDDHMLTTLISGCSEEGTTLSDNEIRDSIVSLITAGYETTS

GALAWAIYALLTVPGTWESAASEVARVLGGRVPAADDLSALTYLNGVVHETLRLYSPGVI

SARRVLRDLWFDGHRIRAGRLLIFSAYVTHRLPEIWPEPTEFRPLRWDPNAADYRKPAPH

EFIPFSGGLHRCIGAVMATTEMTVILARLVARAMLQLPAQRTHRIRAANFAALRPWPGLT

VEIRKSAPAQ

>CYP139A1(2584890677)Mycobacterium tuberculosis BTB12-206

MRYPLGEALLALYRWRGPLINAGVGGHGYTYLLGAEANRFVFANADAFSWSQTFESLVPV

DGPTALIVSDGADHRRRRSVVAPGLRHHHVQRYVATMVSNIDTVIDGWQPGQRLDIYQEL

RSAVRRSTAESLFGQRLAVHSDFLGEQLQPLLDLTRRPPQVMRLQQRVNSPGWRRAMAAR

KRIDDLIDAQIADARTAPRPDDHMLTTLISGCSEEGTTLSDNEIRDSIVSLITAGYETTS

GALAWAIYALLTVPGTWESAASEVARVLGGRVPAADDLSALTYLNGVVHETLRLYSPGVI

SARRVLRDLWFDGHRIRAGRLLIFSAYVTHRLPEIWPEPTEFRPLRWDPNAADYRKPAPH

EFIPFSGGLHRCIGAVMATTEMTVILARLVARAMLQLPAQRTHRIRAANFAALRPWPGLT

VEIRKSAPAQ

>CYP139A1(2590070071)Mycobacterium tuberculosis MAL020110

MRYPLGEALLALYRWRGPLINAGVGGHGYTYLLGAEANRFVFANADAFSWSQTFESLVPV

DGPTALIVSDGADHRRRRSVVAPGLRHHHVQRYVATMVSNIDTVIDGWQPGQRLDIYQEL

RSAVRRSTAESLFGQRLAVHSDFLGEQLQPLLDLTRRPPQVMRLQQRVNSPGWRRAMAAR

KRIDDLIDAQIADARTAPRPDDHMLTTLISGCSEEGTTLSDNEIRDSIVSLITAGYETTS

GALAWAIYALLTVPGTWESAASEVARVLGGRVPAADDLSALTYLNGVVHETLRLYSPGVI

SARRVLRDLWFDGHRIRAGRLLIFSAYVTHRLPEIWPEPTEFRPLRWDPNAADYRKPAPH

EFIPFSGGLHRCIGAVMATTEMTVILARLVARAMLQLPAQRTHRIRAANFAALRPWPGLT

VEIRKSAPAQ

>CYP139A1(2590169218)Mycobacterium tuberculosis MAL020200

MRYPLGEALLALYRWRGPLINAGVGGHGYTYLLGAEANRFVFANADAFSWSQTFESLVPV

DGPTALIVSDGADHRRRRSVVAPGLRHHHVQRYVATMVSNIDTVIDGWQPGQRLDIYQEL

RSAVRRSTAESLFGQRLAVHSDFLGEQLQPLLDLTRRPPQVMRLQQRVNSPGWRRAMAAR

KRIDDLIDAQIADARTAPRPDDHMLTTLISGCSEEGTTLSDNEIRDSIVSLITAGYETTS

GALAWAIYALLTVPGTWESAASEVARVLGGRVPAADDLSALTYLNGVVHETLRLYSPGVI

SARRVLRDLWFDGHRIRAGRLLIFSAYVTHRLPEIWPEPTEFRPLRWDPNAADYRKPAPH

EFIPFSGGLHRCIGAVMATTEMTVILARLVARAMLQLPAQRTHRIRAANFAALRPWPGLT

VEIRKSAPAQ

>CYP139A1(644880084)Mycobacterium tuberculosis KZN 1435

MRYPLGEALLALYRWRGPLINAGVGGHGYTYLLGAEANRFVFANADAFSWSQTFESLVPV

DGPTALIVSDGADHRRRRSVVAPGLRHHHVQRYVATMVSNIDTVIDGWQPGQRLDIYQEL

RSAVRRSTAESLFGQRLAVHSDFLGEQLQPLLDLTRRPPQVMRLQQRVNSPGWRRAMAAR

KRIDDLIDAQIADARTAPRPDDHMLTTLISGCSEEGTTLSDNEIRDSIVSLITAGYETTS

GALAWAIYALLTVPGTWESAASEVARVLGGRVPAADDLSALTYLNGVVHETLRLYSPGVI

SARRVLRDLWFDGHRIRAGRLLIFSAYVTHRLPEIWPEPTEFRPLRWDPNAADYRKPAPH

EFIPFSGGLHRCIGAVMATTEMTVILARLVARAMLQLPAQRTHRIRAANFAALRPWPGLT

VEIRKSAPAQ

>CYP139A1(2574905673)Mycobacterium tuberculosis TRUG0098

MRYPLGEALLALYRWRGPLINAGVGGHGYTYLLGAEANRFVFANADAFSWSQTFESLVPV

DGPTALIVSDGADHRRRRSVVAPGLRHHHVQRYVATMVSNIDTVIDGWQPGQRLDIYQEL

RSAVRRSTAESLFGQRLAVHSDFLGEQLQPLLDLTRRPPQVMRLQQRVNSPGWRRAMAAR

KRIDDLIDAQIADARTAPRPDDHMLTTLISGCSEEGTTLSDNEIRDSIVSLITAGYETTS

GALAWAIYALLTVPGTWESAASEVARVLGGRVPAADDLSALTYLNGVVHETLRLYSPGVI

SARRVLRDLWFDGHRIRAGRLLIFSAYVTHRLPEIWPEPTEFRPLRWDPNAADYRKPAPH

EFIPFSGGLHRCIGAVMATTEMTVILARLVARAMLQLPAQRTHRIRAANFAALRPWPGLT

VEIRKSAPAQ

>CYP139A1(2575285636)Mycobacterium tuberculosis TRUG0070

MRYPLGEALLALYRWRGPLINAGVGGHGYTYLLGAEANRFVFANADAFSWSQTFESLVPV

DGPTALIVSDGADHRRRRSVVAPGLRHHHVQRYVATMVSNIDTVIDGWQPGQRLDIYQEL

RSAVRRSTAESLFGQRLAVHSDFLGEQLQPLLDLTRRPPQVMRLQQRVNSPGWRRAMAAR

KRIDDLIDAQIADARTAPRPDDHMLTTLISGCSEEGTTLSDNEIRDSIVSLITAGYETTS

GALAWAIYALLTVPGTWESAASEVARVLGGRVPAADDLSALTYLNGVVHETLRLYSPGVI

SARRVLRDLWFDGHRIRAGRLLIFSAYVTHRLPEIWPEPTEFRPLRWDPNAADYRKPAPH

EFIPFSGGLHRCIGAVMATTEMTVILARLVARAMLQLPAQRTHRIRAANFAALRPWPGLT

VEIRKSAPAQ

>CYP139A1(2575508785)Mycobacterium tuberculosis TKK-01-0075

MRYPLGEALLALYRWRGPLINAGVGGHGYTYLLGAEANRFVFANADAFSWSQTFESLVPV

DGPTALIVSDGADHRRRRSVVAPGLRHHHVQRYVATMVSNIDTVIDGWQPGQRLDIYQEL

RSAVRRSTAESLFGQRLAVHSDFLGEQLQPLLDLTRRPPQVMRLQQRVNSPGWRRAMAAR

KRIDDLIDAQIADARTAPRPDDHMLTTLISGCSEEGTTLSDNEIRDSIVSLITAGYETTS

GALAWAIYALLTVPGTWESAASEVARVLGGRVPAADDLSALTYLNGVVHETLRLYSPGVI

SARRVLRDLWFDGHRIRAGRLLIFSAYVTHRLPEIWPEPTEFRPLRWDPNAADYRKPAPH

EFIPFSGGLHRCIGAVMATTEMTVILARLVARAMLQLPAQRTHRIRAANFAALRPWPGLT

VEIRKSAPAQ

>CYP139A1(2576585830)Mycobacterium tuberculosis TB_RSA97

MRYPLGEALLALYRWRGPLINAGVGGHGYTYLLGAEANRFVFANADAFSWSQTFESLVPV

DGPTALIVSDGADHRRRRSVVAPGLRHHHVQRYVATMVSNIDTVIDGWQPGQRLDIYQEL

RSAVRRSTAESLFGQRLAVHSDFLGEQLQPLLDLTRRPPQVMRLQQRVNSPGWRRAMAAR

KRIDDLIDAQIADARTAPRPDDHMLTTLISGCSEEGTTLSDNEIRDSIVSLITAGYETTS

GALAWAIYALLTVPGTWESAASEVARVLGGRVPAADDLSALTYLNGVVHETLRLYSPGVI

SARRVLRDLWFDGHRIRAGRLLIFSAYVTHRLPEIWPEPTEFRPLRWDPNAADYRKPAPH

EFIPFSGGLHRCIGAVMATTEMTVILARLVARAMLQLPAQRTHRIRAANFAALRPWPGLT

VEIRKSAPAQ

>CYP139A1(2584979253)Mycobacterium tuberculosis TKK_04_0067

MRYPLGEALLALYRWRGPLINAGVGGHGYTYLLGAEANRFVFANADAFSWSQTFESLVPV

DGPTALIVSDGADHRRRRSVVAPGLRHHHVQRYVATMVSNIDTVIDGWQPGQRLDIYQEL

RSAVRRSTAESLFGQRLAVHSDFLGEQLQPLLDLTRRPPQVMRLQQRVNSPGWRRAMAAR

KRIDDLIDAQIADARTAPRPDDHMLTTLISGCSEEGTTLSDNEIRDSIVSLITAGYETTS

GALAWAIYALLTVPGTWESAASEVARVLGGRVPAADDLSALTYLNGVVHETLRLYSPGVI

SARRVLRDLWFDGHRIRAGRLLIFSAYVTHRLPEIWPEPTEFRPLRWDPNAADYRKPAPH

EFIPFSGGLHRCIGAVMATTEMTVILARLVARAMLQLPAQRTHRIRAANFAALRPWPGLT

VEIRKSAPAQ

>CYP139A1(2588982991)Mycobacterium tuberculosis TKK-01-0023

MRYPLGEALLALYRWRGPLINAGVGGHGYTYLLGAEANRFVFANADAFSWSQTFESLVPV

DGPTALIVSDGADHRRRRSVVAPGLRHHHVQRYVATMVSNIDTVIDGWQPGQRLDIYQEL

RSAVRRSTAESLFGQRLAVHSDFLGEQLQPLLDLTRRPPQVMRLQQRVNSPGWRRAMAAR

KRIDDLIDAQIADARTAPRPDDHMLTTLISGCSEEGTTLSDNEIRDSIVSLITAGYETTS

GALAWAIYALLTVPGTWESAASEVARVLGGRVPAADDLSALTYLNGVVHETLRLYSPGVI

SARRVLRDLWFDGHRIRAGRLLIFSAYVTHRLPEIWPEPTEFRPLRWDPNAADYRKPAPH

EFIPFSGGLHRCIGAVMATTEMTVILARLVARAMLQLPAQRTHRIRAANFAALRPWPGLT

VEIRKSAPAQ

>CYP139A1(2589048312)Mycobacterium tuberculosis TBR24

MRYPLGEALLALYRWRGPLINAGVGGHGYTYLLGAEANRFVFANADAFSWSQTFESLVPV

DGPTALIVSDGADHRRRRSVVAPGLRHHHVQRYVATMVSNIDTVIDGWQPGQRLDIYQEL

RSAVRRSTAESLFGQRLAVHSDFLGEQLQPLLDLTRRPPQVMRLQQRVNSPGWRRAMAAR

KRIDDLIDAQIADARTAPRPDDHMLTTLISGCSEEGTTLSDNEIRDSIVSLITAGYETTS

GALAWAIYALLTVPGTWESAASEVARVLGGRVPAADDLSALTYLNGVVHETLRLYSPGVI

SARRVLRDLWFDGHRIRAGRLLIFSAYVTHRLPEIWPEPTEFRPLRWDPNAADYRKPAPH

EFIPFSGGLHRCIGAVMATTEMTVILARLVARAMLQLPAQRTHRIRAANFAALRPWPGLT

VEIRKSAPAQ

>CYP139A1(2589060525)Mycobacterium tuberculosis TBR30

MRYPLGEALLALYRWRGPLINAGVGGHGYTYLLGAEANRFVFANADAFSWSQTFESLVPV

DGPTALIVSDGADHRRRRSVVAPGLRHHHVQRYVATMVSNIDTVIDGWQPGQRLDIYQEL

RSAVRRSTAESLFGQRLAVHSDFLGEQLQPLLDLTRRPPQVMRLQQRVNSPGWRRAMAAR

KRIDDLIDAQIADARTAPRPDDHMLTTLISGCSEEGTTLSDNEIRDSIVSLITAGYETTS

GALAWAIYALLTVPGTWESAASEVARVLGGRVPAADDLSALTYLNGVVHETLRLYSPGVI

SARRVLRDLWFDGHRIRAGRLLIFSAYVTHRLPEIWPEPTEFRPLRWDPNAADYRKPAPH

EFIPFSGGLHRCIGAVMATTEMTVILARLVARAMLQLPAQRTHRIRAANFAALRPWPGLT

VEIRKSAPAQ

>CYP139A1(2589502409)Mycobacterium tuberculosis TKK-01-0005

MRYPLGEALLALYRWRGPLINAGVGGHGYTYLLGAEANRFVFANADAFSWSQTFESLVPV

DGPTALIVSDGADHRRRRSVVAPGLRHHHVQRYVATMVSNIDTVIDGWQPGQRLDIYQEL

RSAVRRSTAESLFGQRLAVHSDFLGEQLQPLLDLTRRPPQVMRLQQRVNSPGWRRAMAAR

KRIDDLIDAQIADARTAPRPDDHMLTTLISGCSEEGTTLSDNEIRDSIVSLITAGYETTS

GALAWAIYALLTVPGTWESAASEVARVLGGRVPAADDLSALTYLNGVVHETLRLYSPGVI

SARRVLRDLWFDGHRIRAGRLLIFSAYVTHRLPEIWPEPTEFRPLRWDPNAADYRKPAPH

EFIPFSGGLHRCIGAVMATTEMTVILARLVARAMLQLPAQRTHRIRAANFAALRPWPGLT

VEIRKSAPAQ

>CYP139A1(2589572815)Mycobacterium tuberculosis TKK-01-0036

MRYPLGEALLALYRWRGPLINAGVGGHGYTYLLGAEANRFVFANADAFSWSQTFESLVPV

DGPTALIVSDGADHRRRRSVVAPGLRHHHVQRYVATMVSNIDTVIDGWQPGQRLDIYQEL

RSAVRRSTAESLFGQRLAVHSDFLGEQLQPLLDLTRRPPQVMRLQQRVNSPGWRRAMAAR

KRIDDLIDAQIADARTAPRPDDHMLTTLISGCSEEGTTLSDNEIRDSIVSLITAGYETTS

GALAWAIYALLTVPGTWESAASEVARVLGGRVPAADDLSALTYLNGVVHETLRLYSPGVI

SARRVLRDLWFDGHRIRAGRLLIFSAYVTHRLPEIWPEPTEFRPLRWDPNAADYRKPAPH

EFIPFSGGLHRCIGAVMATTEMTVILARLVARAMLQLPAQRTHRIRAANFAALRPWPGLT

VEIRKSAPAQ

>CYP139A1(2590181544)Mycobacterium tuberculosis MAL020205

MRYPLGEALLALYRWRGPLINAGVGGHGYTYLLGAEANRFVFANADAFSWSQTFESLVPV

DGPTALIVSDGADHRRRRSVVAPGLRHHHVQRYVATMVSNIDTVIDGWQPGQRLDIYQEL

RSAVRRSTAESLFGQRLAVHSDFLGEQLQPLLDLTRRPPQVMRLQQRVNSPGWRRAMAAR

KRIDDLIDAQIADARTAPRPDDHMLTTLISGCSEEGTTLSDNEIRDSIVSLITAGYETTS

GALAWAIYALLTVPGTWESAASEVARVLGGRVPAADDLSALTYLNGVVHETLRLYSPGVI

SARRVLRDLWFDGHRIRAGRLLIFSAYVTHRLPEIWPEPTEFRPLRWDPNAADYRKPAPH

EFIPFSGGLHRCIGAVMATTEMTVILARLVARAMLQLPAQRTHRIRAANFAALRPWPGLT

VEIRKSAPAQ

>CYP139A1(2592234638)Mycobacterium tuberculosis TKK_04_0018

MRYPLGEALLALYRWRGPLINAGVGGHGYTYLLGAEANRFVFANADAFSWSQTFESLVPV

DGPTALIVSDGADHRRRRSVVAPGLRHHHVQRYVATMVSNIDTVIDGWQPGQRLDIYQEL

RSAVRRSTAESLFGQRLAVHSDFLGEQLQPLLDLTRRPPQVMRLQQRVNSPGWRRAMAAR

KRIDDLIDAQIADARTAPRPDDHMLTTLISGCSEEGTTLSDNEIRDSIVSLITAGYETTS

GALAWAIYALLTVPGTWESAASEVARVLGGRVPAADDLSALTYLNGVVHETLRLYSPGVI

SARRVLRDLWFDGHRIRAGRLLIFSAYVTHRLPEIWPEPTEFRPLRWDPNAADYRKPAPH

EFIPFSGGLHRCIGAVMATTEMTVILARLVARAMLQLPAQRTHRIRAANFAALRPWPGLT

VEIRKSAPAQ

>CYP139A1(2592271382)Mycobacterium tuberculosis TKK_04_0001

MRYPLGEALLALYRWRGPLINAGVGGHGYTYLLGAEANRFVFANADAFSWSQTFESLVPV

DGPTALIVSDGADHRRRRSVVAPGLRHHHVQRYVATMVSNIDTVIDGWQPGQRLDIYQEL

RSAVRRSTAESLFGQRLAVHSDFLGEQLQPLLDLTRRPPQVMRLQQRVNSPGWRRAMAAR

KRIDDLIDAQIADARTAPRPDDHMLTTLISGCSEEGTTLSDNEIRDSIVSLITAGYETTS

GALAWAIYALLTVPGTWESAASEVARVLGGRVPAADDLSALTYLNGVVHETLRLYSPGVI

SARRVLRDLWFDGHRIRAGRLLIFSAYVTHRLPEIWPEPTEFRPLRWDPNAADYRKPAPH

EFIPFSGGLHRCIGAVMATTEMTVILARLVARAMLQLPAQRTHRIRAANFAALRPWPGLT

VEIRKSAPAQ

>CYP139A1(2592295853)Mycobacterium tuberculosis TKK_03_0034

MRYPLGEALLALYRWRGPLINAGVGGHGYTYLLGAEANRFVFANADAFSWSQTFESLVPV

DGPTALIVSDGADHRRRRSVVAPGLRHHHVQRYVATMVSNIDTVIDGWQPGQRLDIYQEL

RSAVRRSTAESLFGQRLAVHSDFLGEQLQPLLDLTRRPPQVMRLQQRVNSPGWRRAMAAR

KRIDDLIDAQIADARTAPRPDDHMLTTLISGCSEEGTTLSDNEIRDSIVSLITAGYETTS

GALAWAIYALLTVPGTWESAASEVARVLGGRVPAADDLSALTYLNGVVHETLRLYSPGVI

SARRVLRDLWFDGHRIRAGRLLIFSAYVTHRLPEIWPEPTEFRPLRWDPNAADYRKPAPH

EFIPFSGGLHRCIGAVMATTEMTVILARLVARAMLQLPAQRTHRIRAANFAALRPWPGLT

VEIRKSAPAQ

>CYP139A1(2592344179)Mycobacterium tuberculosis TKK_02_0069

MRYPLGEALLALYRWRGPLINAGVGGHGYTYLLGAEANRFVFANADAFSWSQTFESLVPV

DGPTALIVSDGADHRRRRSVVAPGLRHHHVQRYVATMVSNIDTVIDGWQPGQRLDIYQEL

RSAVRRSTAESLFGQRLAVHSDFLGEQLQPLLDLTRRPPQVMRLQQRVNSPGWRRAMAAR

KRIDDLIDAQIADARTAPRPDDHMLTTLISGCSEEGTTLSDNEIRDSIVSLITAGYETTS

GALAWAIYALLTVPGTWESAASEVARVLGGRVPAADDLSALTYLNGVVHETLRLYSPGVI

SARRVLRDLWFDGHRIRAGRLLIFSAYVTHRLPEIWPEPTEFRPLRWDPNAADYRKPAPH

EFIPFSGGLHRCIGAVMATTEMTVILARLVARAMLQLPAQRTHRIRAANFAALRPWPGLT

VEIRKSAPAQ

>CYP139A1(2592357110)Mycobacterium tuberculosis TKK_02_0063

MRYPLGEALLALYRWRGPLINAGVGGHGYTYLLGAEANRFVFANADAFSWSQTFESLVPV

DGPTALIVSDGADHRRRRSVVAPGLRHHHVQRYVATMVSNIDTVIDGWQPGQRLDIYQEL

RSAVRRSTAESLFGQRLAVHSDFLGEQLQPLLDLTRRPPQVMRLQQRVNSPGWRRAMAAR

KRIDDLIDAQIADARTAPRPDDHMLTTLISGCSEEGTTLSDNEIRDSIVSLITAGYETTS

GALAWAIYALLTVPGTWESAASEVARVLGGRVPAADDLSALTYLNGVVHETLRLYSPGVI

SARRVLRDLWFDGHRIRAGRLLIFSAYVTHRLPEIWPEPTEFRPLRWDPNAADYRKPAPH

EFIPFSGGLHRCIGAVMATTEMTVILARLVARAMLQLPAQRTHRIRAANFAALRPWPGLT

VEIRKSAPAQ

>CYP139A1(643035957)Mycobacterium tuberculosis T85

MRYPLGEALLALYRWRGPLINAGVGGHGYTYLLGAEANRFVFANADAFSWSQTFESLVPV

DGPTALIVSDGADHRRRRSVVAPGLRHHHVQRYVATMVSNIDTVIDGWQPGQRLDIYQEL

RSAVRRSTAESLFGQRLAVHSDFLGEQLQPLLDLTRRPPQVMRLQQRVNSPGWRRAMAAR

KRIDDLIDAQIADARTAPRPDDHMLTTLISGCSEEGTTLSDNEIRDSIVSLITAGYETTS

GALAWAIYALLTVPGTWESAASEVARVLGGRVPAADDLSALTYLNGVVHETLRLYSPGVI

SARRVLRDLWFDGHRIRAGRLLIFSAYVTHRLPEIWPEPTEFRPLRWDPNAADYRKPAPH

EFIPFSGGLHRCIGAVMATTEMTVILARLVARAMLQLPAQRTHRIRAANFAALRPWPGLT

VEIRKSAPAQ

>CYP139A1(2575162612)Mycobacterium tuberculosis 2483AR

MRYPLGEALLALYRWRGPLINAGVGGHGYTYLLGAEANRFVFANADAFSWSQTFESLVPV

DGPTALIVSDGADHRRRRSVVAPGLRHHHVQRYVATMVSNIDTVIDGWQPGQRLDIYQEL

RSAVRRSTAESLFGQRLAVHSDFLGEQLQPLLDLTRRPPQVMRLQQRVNSPGWRRAMAAR

KRIDDLIDAQIADARTAPRPDDHMLTTLISGCSEEGTTLSDNEIRDSIVSLITAGYETTS

GALAWAIYALLTVPGTWESAASEVARVLGGRVPAADDLSALTYLNGVVHETLRLYSPGVI

SARRVLRDLWFDGHRIRAGRLLIFSAYVTHRLPEIWPEPTEFRPLRWDPNAADYRKPAPH

EFIPFSGGLHRCIGAVMATTEMTVILARLVARAMLQLPAQRTHRIRAANFAALRPWPGLT

VEIRKSAPAQ

>CYP139A1(2575198589)*Mycobacterium africanum* MAL010112

MRYPLGEALLALYRWRGPLINAGVGGHGYTYLLGAEANRFVFANADAFSWSQTFESLVPV

DGPTALIVSDGADHRRRRSVVAPGLRHHHVQRYVATMVSNIDTVIDGWQPGQRLDIYQEL

RSAVRRSTAESLFGQRLAVHSDFLGEQLQPLLDLTRRPPQVMRLQQRVNSPGWRRAMAAR

KRIDDLIDAQIADARTAPRPDDHMLTTLISGCSEEGTTLSDNEIRDSIVSLITAGYETTS

GALAWAIYALLTVPGTWESAASEVARVLGGRVPAADDLSALTYLNGVVHETLRLYSPGVI

SARRVLRDLWFDGHRIRAGRLLIFSAYVTHRLPEIWPEPTEFRPLRWDPNAADYRKPAPH

EFIPFSGGLHRCIGAVMATTEMTVILARLVARAMLQLPAQRTHRIRAANFAALRPWPGLT

VEIRKSAPAQ

>CYP139A1(2575418829)Mycobacterium tuberculosis KT-0108

MRYPLGEALLALYRWRGPLINAGVGGHGYTYLLGAEANRFVFANADAFSWSQTFESLVPV

DGPTALIVSDGADHRRRRSVVAPGLRHHHVQRYVATMVSNIDTVIDGWQPGQRLDIYQEL

RSAVRRSTAESLFGQRLAVHSDFLGEQLQPLLDLTRRPPQVMRLQQRVNSPGWRRAMAAR

KRIDDLIDAQIADARTAPRPDDHMLTTLISGCSEEGTTLSDNEIRDSIVSLITAGYETTS

GALAWAIYALLTVPGTWESAASEVARVLGGRVPAADDLSALTYLNGVVHETLRLYSPGVI

SARRVLRDLWFDGHRIRAGRLLIFSAYVTHRLPEIWPEPTEFRPLRWDPNAADYRKPAPH

EFIPFSGGLHRCIGAVMATTEMTVILARLVARAMLQLPAQRTHRIRAANFAALRPWPGLT

VEIRKSAPAQ

>CYP139A1(2576172216)Mycobacterium tuberculosis MD16277

MRYPLGEALLALYRWRGPLINAGVGGHGYTYLLGAEANRFVFANADAFSWSQTFESLVPV

DGPTALIVSDGADHRRRRSVVAPGLRHHHVQRYVATMVSNIDTVIDGWQPGQRLDIYQEL

RSAVRRSTAESLFGQRLAVHSDFLGEQLQPLLDLTRRPPQVMRLQQRVNSPGWRRAMAAR

KRIDDLIDAQIADARTAPRPDDHMLTTLISGCSEEGTTLSDNEIRDSIVSLITAGYETTS

GALAWAIYALLTVPGTWESAASEVARVLGGRVPAADDLSALTYLNGVVHETLRLYSPGVI

SARRVLRDLWFDGHRIRAGRLLIFSAYVTHRLPEIWPEPTEFRPLRWDPNAADYRKPAPH

EFIPFSGGLHRCIGAVMATTEMTVILARLVARAMLQLPAQRTHRIRAANFAALRPWPGLT

VEIRKSAPAQ

>CYP139A1(2576534922)Mycobacterium tuberculosis OFXR-13

MRYPLGEALLALYRWRGPLINAGVGGHGYTYLLGAEANRFVFANADAFSWSQTFESLVPV

DGPTALIVSDGADHRRRRSVVAPGLRHHHVQRYVATMVSNIDTVIDGWQPGQRLDIYQEL

RSAVRRSTAESLFGQRLAVHSDFLGEQLQPLLDLTRRPPQVMRLQQRVNSPGWRRAMAAR

KRIDDLIDAQIADARTAPRPDDHMLTTLISGCSEEGTTLSDNEIRDSIVSLITAGYETTS

GALAWAIYALLTVPGTWESAASEVARVLGGRVPAADDLSALTYLNGVVHETLRLYSPGVI

SARRVLRDLWFDGHRIRAGRLLIFSAYVTHRLPEIWPEPTEFRPLRWDPNAADYRKPAPH

EFIPFSGGLHRCIGAVMATTEMTVILARLVARAMLQLPAQRTHRIRAANFAALRPWPGLT

VEIRKSAPAQ

>CYP139A1(2577185436)Mycobacterium tuberculosis BTB05-285

MRYPLGEALLALYRWRGPLINAGVGGHGYTYLLGAEANRFVFANADAFSWSQTFESLVPV

DGPTALIVSDGADHRRRRSVVAPGLRHHHVQRYVATMVSNIDTVIDGWQPGQRLDIYQEL

RSAVRRSTAESLFGQRLAVHSDFLGEQLQPLLDLTRRPPQVMRLQQRVNSPGWRRAMAAR

KRIDDLIDAQIADARTAPRPDDHMLTTLISGCSEEGTTLSDNEIRDSIVSLITAGYETTS

GALAWAIYALLTVPGTWESAASEVARVLGGRVPAADDLSALTYLNGVVHETLRLYSPGVI

SARRVLRDLWFDGHRIRAGRLLIFSAYVTHRLPEIWPEPTEFRPLRWDPNAADYRKPAPH

EFIPFSGGLHRCIGAVMATTEMTVILARLVARAMLQLPAQRTHRIRAANFAALRPWPGLT

VEIRKSAPAQ

>CYP139A1(2577236838)Mycobacterium tuberculosis M2508

MRYPLGEALLALYRWRGPLINAGVGGHGYTYLLGAEANRFVFANADAFSWSQTFESLVPV

DGPTALIVSDGADHRRRRSVVAPGLRHHHVQRYVATMVSNIDTVIDGWQPGQRLDIYQEL

RSAVRRSTAESLFGQRLAVHSDFLGEQLQPLLDLTRRPPQVMRLQQRVNSPGWRRAMAAR

KRIDDLIDAQIADARTAPRPDDHMLTTLISGCSEEGTTLSDNEIRDSIVSLITAGYETTS

GALAWAIYALLTVPGTWESAASEVARVLGGRVPAADDLSALTYLNGVVHETLRLYSPGVI

SARRVLRDLWFDGHRIRAGRLLIFSAYVTHRLPEIWPEPTEFRPLRWDPNAADYRKPAPH

EFIPFSGGLHRCIGAVMATTEMTVILARLVARAMLQLPAQRTHRIRAANFAALRPWPGLT

VEIRKSAPAQ

>CYP139A1(2577373328)Mycobacterium tuberculosis OFXR-33

MRYPLGEALLALYRWRGPLINAGVGGHGYTYLLGAEANRFVFANADAFSWSQTFESLVPV

DGPTALIVSDGADHRRRRSVVAPGLRHHHVQRYVATMVSNIDTVIDGWQPGQRLDIYQEL

RSAVRRSTAESLFGQRLAVHSDFLGEQLQPLLDLTRRPPQVMRLQQRVNSPGWRRAMAAR

KRIDDLIDAQIADARTAPRPDDHMLTTLISGCSEEGTTLSDNEIRDSIVSLITAGYETTS

GALAWAIYALLTVPGTWESAASEVARVLGGRVPAADDLSALTYLNGVVHETLRLYSPGVI

SARRVLRDLWFDGHRIRAGRLLIFSAYVTHRLPEIWPEPTEFRPLRWDPNAADYRKPAPH

EFIPFSGGLHRCIGAVMATTEMTVILARLVARAMLQLPAQRTHRIRAANFAALRPWPGLT

VEIRKSAPAQ

>CYP139A1(2578099005)Mycobacterium tuberculosis BTB07-325

MRYPLGEALLALYRWRGPLINAGVGGHGYTYLLGAEANRFVFANADAFSWSQTFESLVPV

DGPTALIVSDGADHRRRRSVVAPGLRHHHVQRYVATMVSNIDTVIDGWQPGQRLDIYQEL

RSAVRRSTAESLFGQRLAVHSDFLGEQLQPLLDLTRRPPQVMRLQQRVNSPGWRRAMAAR

KRIDDLIDAQIADARTAPRPDDHMLTTLISGCSEEGTTLSDNEIRDSIVSLITAGYETTS

GALAWAIYALLTVPGTWESAASEVARVLGGRVPAADDLSALTYLNGVVHETLRLYSPGVI

SARRVLRDLWFDGHRIRAGRLLIFSAYVTHRLPEIWPEPTEFRPLRWDPNAADYRKPAPH

EFIPFSGGLHRCIGAVMATTEMTVILARLVARAMLQLPAQRTHRIRAANFAALRPWPGLT

VEIRKSAPAQ

>CYP139A1(2580366102)*Mycobacterium africanum* MAL010120

MRYPLGEALLALYRWRGPLINAGVGGHGYTYLLGAEANRFVFANADAFSWSQTFESLVPV

DGPTALIVSDGADHRRRRSVVAPGLRHHHVQRYVATMVSNIDTVIDGWQPGQRLDIYQEL

RSAVRRSTAESLFGQRLAVHSDFLGEQLQPLLDLTRRPPQVMRLQQRVNSPGWRRAMAAR

KRIDDLIDAQIADARTAPRPDDHMLTTLISGCSEEGTTLSDNEIRDSIVSLITAGYETTS

GALAWAIYALLTVPGTWESAASEVARVLGGRVPAADDLSALTYLNGVVHETLRLYSPGVI

SARRVLRDLWFDGHRIRAGRLLIFSAYVTHRLPEIWPEPTEFRPLRWDPNAADYRKPAPH

EFIPFSGGLHRCIGAVMATTEMTVILARLVARAMLQLPAQRTHRIRAANFAALRPWPGLT

VEIRKSAPAQ

>CYP139A1(2580467795)*Mycobacterium africanum* MAL020185

MRYPLGEALLALYRWRGPLINAGVGGHGYTYLLGAEANRFVFANADAFSWSQTFESLVPV

DGPTALIVSDGADHRRRRSVVAPGLRHHHVQRYVATMVSNIDTVIDGWQPGQRLDIYQEL

RSAVRRSTAESLFGQRLAVHSDFLGEQLQPLLDLTRRPPQVMRLQQRVNSPGWRRAMAAR

KRIDDLIDAQIADARTAPRPDDHMLTTLISGCSEEGTTLSDNEIRDSIVSLITAGYETTS

GALAWAIYALLTVPGTWESAASEVARVLGGRVPAADDLSALTYLNGVVHETLRLYSPGVI

SARRVLRDLWFDGHRIRAGRLLIFSAYVTHRLPEIWPEPTEFRPLRWDPNAADYRKPAPH

EFIPFSGGLHRCIGAVMATTEMTVILARLVARAMLQLPAQRTHRIRAANFAALRPWPGLT

VEIRKSAPAQ

>CYP139A1(2584613792)Mycobacterium tuberculosis 44503

MRYPLGEALLALYRWRGPLINAGVGGHGYTYLLGAEANRFVFANADAFSWSQTFESLVPV

DGPTALIVSDGADHRRRRSVVAPGLRHHHVQRYVATMVSNIDTVIDGWQPGQRLDIYQEL

RSAVRRSTAESLFGQRLAVHSDFLGEQLQPLLDLTRRPPQVMRLQQRVNSPGWRRAMAAR

KRIDDLIDAQIADARTAPRPDDHMLTTLISGCSEEGTTLSDNEIRDSIVSLITAGYETTS

GALAWAIYALLTVPGTWESAASEVARVLGGRVPAADDLSALTYLNGVVHETLRLYSPGVI

SARRVLRDLWFDGHRIRAGRLLIFSAYVTHRLPEIWPEPTEFRPLRWDPNAADYRKPAPH

EFIPFSGGLHRCIGAVMATTEMTVILARLVARAMLQLPAQRTHRIRAANFAALRPWPGLT

VEIRKSAPAQ

>CYP139A1(2584681396)Mycobacterium tuberculosis KT-0041

MRYPLGEALLALYRWRGPLINAGVGGHGYTYLLGAEANRFVFANADAFSWSQTFESLVPV

DGPTALIVSDGADHRRRRSVVAPGLRHHHVQRYVATMVSNIDTVIDGWQPGQRLDIYQEL

RSAVRRSTAESLFGQRLAVHSDFLGEQLQPLLDLTRRPPQVMRLQQRVNSPGWRRAMAAR

KRIDDLIDAQIADARTAPRPDDHMLTTLISGCSEEGTTLSDNEIRDSIVSLITAGYETTS

GALAWAIYALLTVPGTWESAASEVARVLGGRVPAADDLSALTYLNGVVHETLRLYSPGVI

SARRVLRDLWFDGHRIRAGRLLIFSAYVTHRLPEIWPEPTEFRPLRWDPNAADYRKPAPH

EFIPFSGGLHRCIGAVMATTEMTVILARLVARAMLQLPAQRTHRIRAANFAALRPWPGLT

VEIRKSAPAQ

>CYP139A1(2584913651)Mycobacterium tuberculosis M1700

MRYPLGEALLALYRWRGPLINAGVGGHGYTYLLGAEANRFVFANADAFSWSQTFESLVPV

DGPTALIVSDGADHRRRRSVVAPGLRHHHVQRYVATMVSNIDTVIDGWQPGQRLDIYQEL

RSAVRRSTAESLFGQRLAVHSDFLGEQLQPLLDLTRRPPQVMRLQQRVNSPGWRRAMAAR

KRIDDLIDAQIADARTAPRPDDHMLTTLISGCSEEGTTLSDNEIRDSIVSLITAGYETTS

GALAWAIYALLTVPGTWESAASEVARVLGGRVPAADDLSALTYLNGVVHETLRLYSPGVI

SARRVLRDLWFDGHRIRAGRLLIFSAYVTHRLPEIWPEPTEFRPLRWDPNAADYRKPAPH

EFIPFSGGLHRCIGAVMATTEMTVILARLVARAMLQLPAQRTHRIRAANFAALRPWPGLT

VEIRKSAPAQ

>CYP139A1(2584942354)Mycobacterium tuberculosis MD15956

MRYPLGEALLALYRWRGPLINAGVGGHGYTYLLGAEANRFVFANADAFSWSQTFESLVPV

DGPTALIVSDGADHRRRRSVVAPGLRHHHVQRYVATMVSNIDTVIDGWQPGQRLDIYQEL

RSAVRRSTAESLFGQRLAVHSDFLGEQLQPLLDLTRRPPQVMRLQQRVNSPGWRRAMAAR

KRIDDLIDAQIADARTAPRPDDHMLTTLISGCSEEGTTLSDNEIRDSIVSLITAGYETTS

GALAWAIYALLTVPGTWESAASEVARVLGGRVPAADDLSALTYLNGVVHETLRLYSPGVI

SARRVLRDLWFDGHRIRAGRLLIFSAYVTHRLPEIWPEPTEFRPLRWDPNAADYRKPAPH

EFIPFSGGLHRCIGAVMATTEMTVILARLVARAMLQLPAQRTHRIRAANFAALRPWPGLT

VEIRKSAPAQ

>CYP139A1(2590093450)Mycobacterium tuberculosis MAL020142

MRYPLGEALLALYRWRGPLINAGVGGHGYTYLLGAEANRFVFANADAFSWSQTFESLVPV

DGPTALIVSDGADHRRRRSVVAPGLRHHHVQRYVATMVSNIDTVIDGWQPGQRLDIYQEL

RSAVRRSTAESLFGQRLAVHSDFLGEQLQPLLDLTRRPPQVMRLQQRVNSPGWRRAMAAR

KRIDDLIDAQIADARTAPRPDDHMLTTLISGCSEEGTTLSDNEIRDSIVSLITAGYETTS

GALAWAIYALLTVPGTWESAASEVARVLGGRVPAADDLSALTYLNGVVHETLRLYSPGVI

SARRVLRDLWFDGHRIRAGRLLIFSAYVTHRLPEIWPEPTEFRPLRWDPNAADYRKPAPH

EFIPFSGGLHRCIGAVMATTEMTVILARLVARAMLQLPAQRTHRIRAANFAALRPWPGLT

VEIRKSAPAQ

>CYP139A1(2590207004)Mycobacterium tuberculosis OFXR-1

MRYPLGEALLALYRWRGPLINAGVGGHGYTYLLGAEANRFVFANADAFSWSQTFESLVPV

DGPTALIVSDGADHRRRRSVVAPGLRHHHVQRYVATMVSNIDTVIDGWQPGQRLDIYQEL

RSAVRRSTAESLFGQRLAVHSDFLGEQLQPLLDLTRRPPQVMRLQQRVNSPGWRRAMAAR

KRIDDLIDAQIADARTAPRPDDHMLTTLISGCSEEGTTLSDNEIRDSIVSLITAGYETTS

GALAWAIYALLTVPGTWESAASEVARVLGGRVPAADDLSALTYLNGVVHETLRLYSPGVI

SARRVLRDLWFDGHRIRAGRLLIFSAYVTHRLPEIWPEPTEFRPLRWDPNAADYRKPAPH

EFIPFSGGLHRCIGAVMATTEMTVILARLVARAMLQLPAQRTHRIRAANFAALRPWPGLT

VEIRKSAPAQ

>CYP139A1(2590276448)Mycobacterium tuberculosis KT-0014

MRYPLGEALLALYRWRGPLINAGVGGHGYTYLLGAEANRFVFANADAFSWSQTFESLVPV

DGPTALIVSDGADHRRRRSVVAPGLRHHHVQRYVATMVSNIDTVIDGWQPGQRLDIYQEL

RSAVRRSTAESLFGQRLAVHSDFLGEQLQPLLDLTRRPPQVMRLQQRVNSPGWRRAMAAR

KRIDDLIDAQIADARTAPRPDDHMLTTLISGCSEEGTTLSDNEIRDSIVSLITAGYETTS

GALAWAIYALLTVPGTWESAASEVARVLGGRVPAADDLSALTYLNGVVHETLRLYSPGVI

SARRVLRDLWFDGHRIRAGRLLIFSAYVTHRLPEIWPEPTEFRPLRWDPNAADYRKPAPH

EFIPFSGGLHRCIGAVMATTEMTVILARLVARAMLQLPAQRTHRIRAANFAALRPWPGLT

VEIRKSAPAQ

>CYP139A1(2575455196)Mycobacterium tuberculosis TKK_03_0109

MRYPLGEALLALYRWRGPLINAGVGGHGYTYLLGAEANRFVFANADAFSWSQTFESLVPV

DGPTALIVSDGADHRRRRSVVAPGLRHHHVQRYVATMVSNIDTVIDGWQPGQRLDIYQEL

RSAVRRSTAESLFGQRLAVHSDFLGEQLQPLLDLTRRPPQVMRLQQRVNSPGWRRAMAAR

KRIDDLIDAQIADARTAPRPDDHMLTTLISGCSEEGTTLSDNEIRDSIVSLITAGYETTS

GALAWAIYALLTVPGTWESAASEVARVLGGRVPAADDLSALTYLNGVVHETLRLYSPGVI

SARRVLRDLWFDGHRIRAGRLLIFSAYVTHRLPEIWPEPTEFRPLRWDPNAADYRKPAPH

EFIPFSGGLHRCIGAVMATTEMTVILARLVARAMLQLPAQRTHRIRAANFAALRPWPGLT

VEIRKSAPAQ

>CYP139A1(2575920420)Mycobacterium tuberculosis TKK_04_0064

MRYPLGEALLALYRWRGPLINAGVGGHGYTYLLGAEANRFVFANADAFSWSQTFESLVPV

DGPTALIVSDGADHRRRRSVVAPGLRHHHVQRYVATMVSNIDTVIDGWQPGQRLDIYQEL

RSAVRRSTAESLFGQRLAVHSDFLGEQLQPLLDLTRRPPQVMRLQQRVNSPGWRRAMAAR

KRIDDLIDAQIADARTAPRPDDHMLTTLISGCSEEGTTLSDNEIRDSIVSLITAGYETTS

GALAWAIYALLTVPGTWESAASEVARVLGGRVPAADDLSALTYLNGVVHETLRLYSPGVI

SARRVLRDLWFDGHRIRAGRLLIFSAYVTHRLPEIWPEPTEFRPLRWDPNAADYRKPAPH

EFIPFSGGLHRCIGAVMATTEMTVILARLVARAMLQLPAQRTHRIRAANFAALRPWPGLT

VEIRKSAPAQ

>CYP139A1(2575998368)Mycobacterium tuberculosis XTB13-094

MRYPLGEALLALYRWRGPLINAGVGGHGYTYLLGAEANRFVFANADAFSWSQTFESLVPV

DGPTALIVSDGADHRRRRSVVAPGLRHHHVQRYVATMVSNIDTVIDGWQPGQRLDIYQEL

RSAVRRSTAESLFGQRLAVHSDFLGEQLQPLLDLTRRPPQVMRLQQRVNSPGWRRAMAAR

KRIDDLIDAQIADARTAPRPDDHMLTTLISGCSEEGTTLSDNEIRDSIVSLITAGYETTS

GALAWAIYALLTVPGTWESAASEVARVLGGRVPAADDLSALTYLNGVVHETLRLYSPGVI

SARRVLRDLWFDGHRIRAGRLLIFSAYVTHRLPEIWPEPTEFRPLRWDPNAADYRKPAPH

EFIPFSGGLHRCIGAVMATTEMTVILARLVARAMLQLPAQRTHRIRAANFAALRPWPGLT

VEIRKSAPAQ

>CYP139A1(2576583109)Mycobacterium tuberculosis TB_RSA82

MRYPLGEALLALYRWRGPLINAGVGGHGYTYLLGAEANRFVFANADAFSWSQTFESLVPV

DGPTALIVSDGADHRRRRSVVAPGLRHHHVQRYVATMVSNIDTVIDGWQPGQRLDIYQEL

RSAVRRSTAESLFGQRLAVHSDFLGEQLQPLLDLTRRPPQVMRLQQRVNSPGWRRAMAAR

KRIDDLIDAQIADARTAPRPDDHMLTTLISGCSEEGTTLSDNEIRDSIVSLITAGYETTS

GALAWAIYALLTVPGTWESAASEVARVLGGRVPAADDLSALTYLNGVVHETLRLYSPGVI

SARRVLRDLWFDGHRIRAGRLLIFSAYVTHRLPEIWPEPTEFRPLRWDPNAADYRKPAPH

EFIPFSGGLHRCIGAVMATTEMTVILARLVARAMLQLPAQRTHRIRAANFAALRPWPGLT

VEIRKSAPAQ

>CYP139A1(2576886343)Mycobacterium tuberculosis TB_RSA09

MRYPLGEALLALYRWRGPLINAGVGGHGYTYLLGAEANRFVFANADAFSWSQTFESLVPV

DGPTALIVSDGADHRRRRSVVAPGLRHHHVQRYVATMVSNIDTVIDGWQPGQRLDIYQEL

RSAVRRSTAESLFGQRLAVHSDFLGEQLQPLLDLTRRPPQVMRLQQRVNSPGWRRAMAAR

KRIDDLIDAQIADARTAPRPDDHMLTTLISGCSEEGTTLSDNEIRDSIVSLITAGYETTS

GALAWAIYALLTVPGTWESAASEVARVLGGRVPAADDLSALTYLNGVVHETLRLYSPGVI

SARRVLRDLWFDGHRIRAGRLLIFSAYVTHRLPEIWPEPTEFRPLRWDPNAADYRKPAPH

EFIPFSGGLHRCIGAVMATTEMTVILARLVARAMLQLPAQRTHRIRAANFAALRPWPGLT

VEIRKSAPAQ

>CYP139A1(2576986616)Mycobacterium tuberculosis TRUG0040

MRYPLGEALLALYRWRGPLINAGVGGHGYTYLLGAEANRFVFANADAFSWSQTFESLVPV

DGPTALIVSDGADHRRRRSVVAPGLRHHHVQRYVATMVSNIDTVIDGWQPGQRLDIYQEL

RSAVRRSTAESLFGQRLAVHSDFLGEQLQPLLDLTRRPPQVMRLQQRVNSPGWRRAMAAR

KRIDDLIDAQIADARTAPRPDDHMLTTLISGCSEEGTTLSDNEIRDSIVSLITAGYETTS

GALAWAIYALLTVPGTWESAASEVARVLGGRVPAADDLSALTYLNGVVHETLRLYSPGVI

SARRVLRDLWFDGHRIRAGRLLIFSAYVTHRLPEIWPEPTEFRPLRWDPNAADYRKPAPH

EFIPFSGGLHRCIGAVMATTEMTVILARLVARAMLQLPAQRTHRIRAANFAALRPWPGLT

VEIRKSAPAQ

>CYP139A1(2577256179)Mycobacterium tuberculosis TKK-01-0079

MRYPLGEALLALYRWRGPLINAGVGGHGYTYLLGAEANRFVFANADAFSWSQTFESLVPV

DGPTALIVSDGADHRRRRSVVAPGLRHHHVQRYVATMVSNIDTVIDGWQPGQRLDIYQEL

RSAVRRSTAESLFGQRLAVHSDFLGEQLQPLLDLTRRPPQVMRLQQRVNSPGWRRAMAAR

KRIDDLIDAQIADARTAPRPDDHMLTTLISGCSEEGTTLSDNEIRDSIVSLITAGYETTS

GALAWAIYALLTVPGTWESAASEVARVLGGRVPAADDLSALTYLNGVVHETLRLYSPGVI

SARRVLRDLWFDGHRIRAGRLLIFSAYVTHRLPEIWPEPTEFRPLRWDPNAADYRKPAPH

EFIPFSGGLHRCIGAVMATTEMTVILARLVARAMLQLPAQRTHRIRAANFAALRPWPGLT

VEIRKSAPAQ

>CYP139A1(2577876445)Mycobacterium tuberculosis XTB13-162

MRYPLGEALLALYRWRGPLINAGVGGHGYTYLLGAEANRFVFANADAFSWSQTFESLVPV

DGPTALIVSDGADHRRRRSVVAPGLRHHHVQRYVATMVSNIDTVIDGWQPGQRLDIYQEL

RSAVRRSTAESLFGQRLAVHSDFLGEQLQPLLDLTRRPPQVMRLQQRVNSPGWRRAMAAR

KRIDDLIDAQIADARTAPRPDDHMLTTLISGCSEEGTTLSDNEIRDSIVSLITAGYETTS

GALAWAIYALLTVPGTWESAASEVARVLGGRVPAADDLSALTYLNGVVHETLRLYSPGVI

SARRVLRDLWFDGHRIRAGRLLIFSAYVTHRLPEIWPEPTEFRPLRWDPNAADYRKPAPH

EFIPFSGGLHRCIGAVMATTEMTVILARLVARAMLQLPAQRTHRIRAANFAALRPWPGLT

VEIRKSAPAQ

>CYP139A1(2579798813)Mycobacterium tuberculosis TB_RSA64

MRYPLGEALLALYRWRGPLINAGVGGHGYTYLLGAEANRFVFANADAFSWSQTFESLVPV

DGPTALIVSDGADHRRRRSVVAPGLRHHHVQRYVATMVSNIDTVIDGWQPGQRLDIYQEL

RSAVRRSTAESLFGQRLAVHSDFLGEQLQPLLDLTRRPPQVMRLQQRVNSPGWRRAMAAR

KRIDDLIDAQIADARTAPRPDDHMLTTLISGCSEEGTTLSDNEIRDSIVSLITAGYETTS

GALAWAIYALLTVPGTWESAASEVARVLGGRVPAADDLSALTYLNGVVHETLRLYSPGVI

SARRVLRDLWFDGHRIRAGRLLIFSAYVTHRLPEIWPEPTEFRPLRWDPNAADYRKPAPH

EFIPFSGGLHRCIGAVMATTEMTVILARLVARAMLQLPAQRTHRIRAANFAALRPWPGLT

VEIRKSAPAQ

>CYP139A1(2584715344)Mycobacterium tuberculosis TB_RSA166

MRYPLGEALLALYRWRGPLINAGVGGHGYTYLLGAEANRFVFANADAFSWSQTFESLVPV

DGPTALIVSDGADHRRRRSVVAPGLRHHHVQRYVATMVSNIDTVIDGWQPGQRLDIYQEL

RSAVRRSTAESLFGQRLAVHSDFLGEQLQPLLDLTRRPPQVMRLQQRVNSPGWRRAMAAR

KRIDDLIDAQIADARTAPRPDDHMLTTLISGCSEEGTTLSDNEIRDSIVSLITAGYETTS

GALAWAIYALLTVPGTWESAASEVARVLGGRVPAADDLSALTYLNGVVHETLRLYSPGVI

SARRVLRDLWFDGHRIRAGRLLIFSAYVTHRLPEIWPEPTEFRPLRWDPNAADYRKPAPH

EFIPFSGGLHRCIGAVMATTEMTVILARLVARAMLQLPAQRTHRIRAANFAALRPWPGLT

VEIRKSAPAQ

>CYP139A1(2588659602)Mycobacterium tuberculosis T67

MRYPLGEALLALYRWRGPLINAGVGGHGYTYLLGAEANRFVFANADAFSWSQTFESLVPV

DGPTALIVSDGADHRRRRSVVAPGLRHHHVQRYVATMVSNIDTVIDGWQPGQRLDIYQEL

RSAVRRSTAESLFGQRLAVHSDFLGEQLQPLLDLTRRPPQVMRLQQRVNSPGWRRAMAAR

KRIDDLIDAQIADARTAPRPDDHMLTTLISGCSEEGTTLSDNEIRDSIVSLITAGYETTS

GALAWAIYALLTVPGTWESAASEVARVLGGRVPAADDLSALTYLNGVVHETLRLYSPGVI

SARRVLRDLWFDGHRIRAGRLLIFSAYVTHRLPEIWPEPTEFRPLRWDPNAADYRKPAPH

EFIPFSGGLHRCIGAVMATTEMTVILARLVARAMLQLPAQRTHRIRAANFAALRPWPGLT

VEIRKSAPAQ

>CYP139A1(2589089021)Mycobacterium tuberculosis TBR48

MRYPLGEALLALYRWRGPLINAGVGGHGYTYLLGAEANRFVFANADAFSWSQTFESLVPV

DGPTALIVSDGADHRRRRSVVAPGLRHHHVQRYVATMVSNIDTVIDGWQPGQRLDIYQEL

RSAVRRSTAESLFGQRLAVHSDFLGEQLQPLLDLTRRPPQVMRLQQRVNSPGWRRAMAAR

KRIDDLIDAQIADARTAPRPDDHMLTTLISGCSEEGTTLSDNEIRDSIVSLITAGYETTS

GALAWAIYALLTVPGTWESAASEVARVLGGRVPAADDLSALTYLNGVVHETLRLYSPGVI

SARRVLRDLWFDGHRIRAGRLLIFSAYVTHRLPEIWPEPTEFRPLRWDPNAADYRKPAPH

EFIPFSGGLHRCIGAVMATTEMTVILARLVARAMLQLPAQRTHRIRAANFAALRPWPGLT

VEIRKSAPAQ

>CYP139A1(2589093094)Mycobacterium tuberculosis TBR49

MRYPLGEALLALYRWRGPLINAGVGGHGYTYLLGAEANRFVFANADAFSWSQTFESLVPV

DGPTALIVSDGADHRRRRSVVAPGLRHHHVQRYVATMVSNIDTVIDGWQPGQRLDIYQEL

RSAVRRSTAESLFGQRLAVHSDFLGEQLQPLLDLTRRPPQVMRLQQRVNSPGWRRAMAAR

KRIDDLIDAQIADARTAPRPDDHMLTTLISGCSEEGTTLSDNEIRDSIVSLITAGYETTS

GALAWAIYALLTVPGTWESAASEVARVLGGRVPAADDLSALTYLNGVVHETLRLYSPGVI

SARRVLRDLWFDGHRIRAGRLLIFSAYVTHRLPEIWPEPTEFRPLRWDPNAADYRKPAPH

EFIPFSGGLHRCIGAVMATTEMTVILARLVARAMLQLPAQRTHRIRAANFAALRPWPGLT

VEIRKSAPAQ

>CYP139A1(2589535085)Mycobacterium tuberculosis TKK-01-0016

MRYPLGEALLALYRWRGPLINAGVGGHGYTYLLGAEANRFVFANADAFSWSQTFESLVPV

DGPTALIVSDGADHRRRRSVVAPGLRHHHVQRYVATMVSNIDTVIDGWQPGQRLDIYQEL

RSAVRRSTAESLFGQRLAVHSDFLGEQLQPLLDLTRRPPQVMRLQQRVNSPGWRRAMAAR

KRIDDLIDAQIADARTAPRPDDHMLTTLISGCSEEGTTLSDNEIRDSIVSLITAGYETTS

GALAWAIYALLTVPGTWESAASEVARVLGGRVPAADDLSALTYLNGVVHETLRLYSPGVI

SARRVLRDLWFDGHRIRAGRLLIFSAYVTHRLPEIWPEPTEFRPLRWDPNAADYRKPAPH

EFIPFSGGLHRCIGAVMATTEMTVILARLVARAMLQLPAQRTHRIRAANFAALRPWPGLT

VEIRKSAPAQ

>CYP139A1(2589663797)Mycobacterium tuberculosis TKK-01-0074

MRYPLGEALLALYRWRGPLINAGVGGHGYTYLLGAEANRFVFANADAFSWSQTFESLVPV

DGPTALIVSDGADHRRRRSVVAPGLRHHHVQRYVATMVSNIDTVIDGWQPGQRLDIYQEL

RSAVRRSTAESLFGQRLAVHSDFLGEQLQPLLDLTRRPPQVMRLQQRVNSPGWRRAMAAR

KRIDDLIDAQIADARTAPRPDDHMLTTLISGCSEEGTTLSDNEIRDSIVSLITAGYETTS

GALAWAIYALLTVPGTWESAASEVARVLGGRVPAADDLSALTYLNGVVHETLRLYSPGVI

SARRVLRDLWFDGHRIRAGRLLIFSAYVTHRLPEIWPEPTEFRPLRWDPNAADYRKPAPH

EFIPFSGGLHRCIGAVMATTEMTVILARLVARAMLQLPAQRTHRIRAANFAALRPWPGLT

VEIRKSAPAQ

>CYP139A1(2589671952)Mycobacterium tuberculosis TKK-01-0073

MRYPLGEALLALYRWRGPLINAGVGGHGYTYLLGAEANRFVFANADAFSWSQTFESLVPV

DGPTALIVSDGADHRRRRSVVAPGLRHHHVQRYVATMVSNIDTVIDGWQPGQRLDIYQEL

RSAVRRSTAESLFGQRLAVHSDFLGEQLQPLLDLTRRPPQVMRLQQRVNSPGWRRAMAAR

KRIDDLIDAQIADARTAPRPDDHMLTTLISGCSEEGTTLSDNEIRDSIVSLITAGYETTS

GALAWAIYALLTVPGTWESAASEVARVLGGRVPAADDLSALTYLNGVVHETLRLYSPGVI

SARRVLRDLWFDGHRIRAGRLLIFSAYVTHRLPEIWPEPTEFRPLRWDPNAADYRKPAPH

EFIPFSGGLHRCIGAVMATTEMTVILARLVARAMLQLPAQRTHRIRAANFAALRPWPGLT

VEIRKSAPAQ

>CYP139A1(2589719807)Mycobacterium tuberculosis TKK-01-0087

MRYPLGEALLALYRWRGPLINAGVGGHGYTYLLGAEANRFVFANADAFSWSQTFESLVPV

DGPTALIVSDGADHRRRRSVVAPGLRHHHVQRYVATMVSNIDTVIDGWQPGQRLDIYQEL

RSAVRRSTAESLFGQRLAVHSDFLGEQLQPLLDLTRRPPQVMRLQQRVNSPGWRRAMAAR

KRIDDLIDAQIADARTAPRPDDHMLTTLISGCSEEGTTLSDNEIRDSIVSLITAGYETTS

GALAWAIYALLTVPGTWESAASEVARVLGGRVPAADDLSALTYLNGVVHETLRLYSPGVI

SARRVLRDLWFDGHRIRAGRLLIFSAYVTHRLPEIWPEPTEFRPLRWDPNAADYRKPAPH

EFIPFSGGLHRCIGAVMATTEMTVILARLVARAMLQLPAQRTHRIRAANFAALRPWPGLT

VEIRKSAPAQ

>CYP139A1(2592246876)Mycobacterium tuberculosis TKK_04_0014

MRYPLGEALLALYRWRGPLINAGVGGHGYTYLLGAEANRFVFANADAFSWSQTFESLVPV

DGPTALIVSDGADHRRRRSVVAPGLRHHHVQRYVATMVSNIDTVIDGWQPGQRLDIYQEL

RSAVRRSTAESLFGQRLAVHSDFLGEQLQPLLDLTRRPPQVMRLQQRVNSPGWRRAMAAR

KRIDDLIDAQIADARTAPRPDDHMLTTLISGCSEEGTTLSDNEIRDSIVSLITAGYETTS

GALAWAIYALLTVPGTWESAASEVARVLGGRVPAADDLSALTYLNGVVHETLRLYSPGVI

SARRVLRDLWFDGHRIRAGRLLIFSAYVTHRLPEIWPEPTEFRPLRWDPNAADYRKPAPH

EFIPFSGGLHRCIGAVMATTEMTVILARLVARAMLQLPAQRTHRIRAANFAALRPWPGLT

VEIRKSAPAQ

>CYP139A1(2592381635)Mycobacterium tuberculosis TKK_02_0036

MRYPLGEALLALYRWRGPLINAGVGGHGYTYLLGAEANRFVFANADAFSWSQTFESLVPV

DGPTALIVSDGADHRRRRSVVAPGLRHHHVQRYVATMVSNIDTVIDGWQPGQRLDIYQEL

RSAVRRSTAESLFGQRLAVHSDFLGEQLQPLLDLTRRPPQVMRLQQRVNSPGWRRAMAAR

KRIDDLIDAQIADARTAPRPDDHMLTTLISGCSEEGTTLSDNEIRDSIVSLITAGYETTS

GALAWAIYALLTVPGTWESAASEVARVLGGRVPAADDLSALTYLNGVVHETLRLYSPGVI

SARRVLRDLWFDGHRIRAGRLLIFSAYVTHRLPEIWPEPTEFRPLRWDPNAADYRKPAPH

EFIPFSGGLHRCIGAVMATTEMTVILARLVARAMLQLPAQRTHRIRAANFAALRPWPGLT

VEIRKSAPAQ

>CYP139A1(2592410032)Mycobacterium tuberculosis TKK_02_0020

MRYPLGEALLALYRWRGPLINAGVGGHGYTYLLGAEANRFVFANADAFSWSQTFESLVPV

DGPTALIVSDGADHRRRRSVVAPGLRHHHVQRYVATMVSNIDTVIDGWQPGQRLDIYQEL

RSAVRRSTAESLFGQRLAVHSDFLGEQLQPLLDLTRRPPQVMRLQQRVNSPGWRRAMAAR

KRIDDLIDAQIADARTAPRPDDHMLTTLISGCSEEGTTLSDNEIRDSIVSLITAGYETTS

GALAWAIYALLTVPGTWESAASEVARVLGGRVPAADDLSALTYLNGVVHETLRLYSPGVI

SARRVLRDLWFDGHRIRAGRLLIFSAYVTHRLPEIWPEPTEFRPLRWDPNAADYRKPAPH

EFIPFSGGLHRCIGAVMATTEMTVILARLVARAMLQLPAQRTHRIRAANFAALRPWPGLT

VEIRKSAPAQ

>CYP139A1(2592414205)Mycobacterium tuberculosis TKK_02_0018

MRYPLGEALLALYRWRGPLINAGVGGHGYTYLLGAEANRFVFANADAFSWSQTFESLVPV

DGPTALIVSDGADHRRRRSVVAPGLRHHHVQRYVATMVSNIDTVIDGWQPGQRLDIYQEL

RSAVRRSTAESLFGQRLAVHSDFLGEQLQPLLDLTRRPPQVMRLQQRVNSPGWRRAMAAR

KRIDDLIDAQIADARTAPRPDDHMLTTLISGCSEEGTTLSDNEIRDSIVSLITAGYETTS

GALAWAIYALLTVPGTWESAASEVARVLGGRVPAADDLSALTYLNGVVHETLRLYSPGVI

SARRVLRDLWFDGHRIRAGRLLIFSAYVTHRLPEIWPEPTEFRPLRWDPNAADYRKPAPH

EFIPFSGGLHRCIGAVMATTEMTVILARLVARAMLQLPAQRTHRIRAANFAALRPWPGLT

VEIRKSAPAQ

>CYP139A1(2592566477)Mycobacterium tuberculosis TKK_04_0034

MRYPLGEALLALYRWRGPLINAGVGGHGYTYLLGAEANRFVFANADAFSWSQTFESLVPV

DGPTALIVSDGADHRRRRSVVAPGLRHHHVQRYVATMVSNIDTVIDGWQPGQRLDIYQEL

RSAVRRSTAESLFGQRLAVHSDFLGEQLQPLLDLTRRPPQVMRLQQRVNSPGWRRAMAAR

KRIDDLIDAQIADARTAPRPDDHMLTTLISGCSEEGTTLSDNEIRDSIVSLITAGYETTS

GALAWAIYALLTVPGTWESAASEVARVLGGRVPAADDLSALTYLNGVVHETLRLYSPGVI

SARRVLRDLWFDGHRIRAGRLLIFSAYVTHRLPEIWPEPTEFRPLRWDPNAADYRKPAPH

EFIPFSGGLHRCIGAVMATTEMTVILARLVARAMLQLPAQRTHRIRAANFAALRPWPGLT

VEIRKSAPAQ

>CYP139A1(2575647889)Mycobacterium tuberculosis BTB11-343

MRYPLGEALLALYRWRGPLINAGVGGHGYTYLLGAEANRFVFANADAFSWSQTFESLVPV

DGPTALIVSDGADHRRRRSVVAPGLRHHHVQRYVATMVSNIDTVIDGWQPGQRLDIYQEL

RSAVRRSTAESLFGQRLAVHSDFLGEQLQPLLDLTRRPPQVMRLQQRVNSPGWRRAMAAR

KRIDDLIDAQIADARTAPRPDDHMLTTLISGCSEEGTTLSDNEIRDSIVSLITAGYETTS

GALAWAIYALLTVPGTWESAASEVARVLGGRVPAADDLSALTYLNGVVHETLRLYSPGVI

SARRVLRDLWFDGHRIRAGRLLIFSAYVTHRLPEIWPEPTEFRPLRWDPNAADYRKPAPH

EFIPFSGGLHRCIGAVMATTEMTVILARLVARAMLQLPAQRTHRIRAANFAALRPWPGLT

VEIRKSAPAQ

>CYP139A1(2577429481)Mycobacterium tuberculosis 49375

MRYPLGEALLALYRWRGPLINAGVGGHGYTYLLGAEANRFVFANADAFSWSQTFESLVPV

DGPTALIVSDGADHRRRRSVVAPGLRHHHVQRYVATMVSNIDTVIDGWQPGQRLDIYQEL

RSAVRRSTAESLFGQRLAVHSDFLGEQLQPLLDLTRRPPQVMRLQQRVNSPGWRRAMAAR

KRIDDLIDAQIADARTAPRPDDHMLTTLISGCSEEGTTLSDNEIRDSIVSLITAGYETTS

GALAWAIYALLTVPGTWESAASEVARVLGGRVPAADDLSALTYLNGVVHETLRLYSPGVI

SARRVLRDLWFDGHRIRAGRLLIFSAYVTHRLPEIWPEPTEFRPLRWDPNAADYRKPAPH

EFIPFSGGLHRCIGAVMATTEMTVILARLVARAMLQLPAQRTHRIRAANFAALRPWPGLT

VEIRKSAPAQ

>CYP139A1(2578084434)Mycobacterium tuberculosis KT-0001

MRYPLGEALLALYRWRGPLINAGVGGHGYTYLLGAEANRFVFANADAFSWSQTFESLVPV

DGPTALIVSDGADHRRRRSVVAPGLRHHHVQRYVATMVSNIDTVIDGWQPGQRLDIYQEL

RSAVRRSTAESLFGQRLAVHSDFLGEQLQPLLDLTRRPPQVMRLQQRVNSPGWRRAMAAR

KRIDDLIDAQIADARTAPRPDDHMLTTLISGCSEEGTTLSDNEIRDSIVSLITAGYETTS

GALAWAIYALLTVPGTWESAASEVARVLGGRVPAADDLSALTYLNGVVHETLRLYSPGVI

SARRVLRDLWFDGHRIRAGRLLIFSAYVTHRLPEIWPEPTEFRPLRWDPNAADYRKPAPH

EFIPFSGGLHRCIGAVMATTEMTVILARLVARAMLQLPAQRTHRIRAANFAALRPWPGLT

VEIRKSAPAQ

>CYP139A1(2580301723)*Mycobacterium bovis*B2 7505

MRYPLGEALLALYRWRGPLINAGVGGHGYTYLLGAEANRFVFANADAFSWSQTFESLVPV

DGPTALIVSDGADHRRRRSVVAPGLRHHHVQRYVATMVSNIDTVIDGWQPGQRLDIYQEL

RSAVRRSTAESLFGQRLAVHSDFLGEQLQPLLDLTRRPPQVMRLQQRVNSPGWRRAMAAR

KRIDDLIDAQIADARTAPRPDDHMLTTLISGCSEEGTTLSDNEIRDSIVSLITAGYETTS

GALAWAIYALLTVPGTWESAASEVARVLGGRVPAADDLSALTYLNGVVHETLRLYSPGVI

SARRVLRDLWFDGHRIRAGRLLIFSAYVTHRLPEIWPEPTEFRPLRWDPNAADYRKPAPH

EFIPFSGGLHRCIGAVMATTEMTVILARLVARAMLQLPAQRTHRIRAANFAALRPWPGLT

VEIRKSAPAQ

>CYP139A1(2581901399)*Mycobacterium africanum* MAL010129

MRYPLGEALLALYRWRGPLINAGVGGHGYTYLLGAEANRFVFANADAFSWSQTFESLVPV

DGPTALIVSDGADHRRRRSVVAPGLRHHHVQRYVATMVSNIDTVIDGWQPGQRLDIYQEL

RSAVRRSTAESLFGQRLAVHSDFLGEQLQPLLDLTRRPPQVMRLQQRVNSPGWRRAMAAR

KRIDDLIDAQIADARTAPRPDDHMLTTLISGCSEEGTTLSDNEIRDSIVSLITAGYETTS

GALAWAIYALLTVPGTWESAASEVARVLGGRVPAADDLSALTYLNGVVHETLRLYSPGVI

SARRVLRDLWFDGHRIRAGRLLIFSAYVTHRLPEIWPEPTEFRPLRWDPNAADYRKPAPH

EFIPFSGGLHRCIGAVMATTEMTVILARLVARAMLQLPAQRTHRIRAANFAALRPWPGLT

VEIRKSAPAQ

>CYP139A1(2584689961)Mycobacterium tuberculosis M1961

MRYPLGEALLALYRWRGPLINAGVGGHGYTYLLGAEANRFVFANADAFSWSQTFESLVPV

DGPTALIVSDGADHRRRRSVVAPGLRHHHVQRYVATMVSNIDTVIDGWQPGQRLDIYQEL

RSAVRRSTAESLFGQRLAVHSDFLGEQLQPLLDLTRRPPQVMRLQQRVNSPGWRRAMAAR

KRIDDLIDAQIADARTAPRPDDHMLTTLISGCSEEGTTLSDNEIRDSIVSLITAGYETTS

GALAWAIYALLTVPGTWESAASEVARVLGGRVPAADDLSALTYLNGVVHETLRLYSPGVI

SARRVLRDLWFDGHRIRAGRLLIFSAYVTHRLPEIWPEPTEFRPLRWDPNAADYRKPAPH

EFIPFSGGLHRCIGAVMATTEMTVILARLVARAMLQLPAQRTHRIRAANFAALRPWPGLT

VEIRKSAPAQ

>CYP139A1(2584748012)Mycobacterium tuberculosis BTB13-206

MRYPLGEALLALYRWRGPLINAGVGGHGYTYLLGAEANRFVFANADAFSWSQTFESLVPV

DGPTALIVSDGADHRRRRSVVAPGLRHHHVQRYVATMVSNIDTVIDGWQPGQRLDIYQEL

RSAVRRSTAESLFGQRLAVHSDFLGEQLQPLLDLTRRPPQVMRLQQRVNSPGWRRAMAAR

KRIDDLIDAQIADARTAPRPDDHMLTTLISGCSEEGTTLSDNEIRDSIVSLITAGYETTS

GALAWAIYALLTVPGTWESAASEVARVLGGRVPAADDLSALTYLNGVVHETLRLYSPGVI

SARRVLRDLWFDGHRIRAGRLLIFSAYVTHRLPEIWPEPTEFRPLRWDPNAADYRKPAPH

EFIPFSGGLHRCIGAVMATTEMTVILARLVARAMLQLPAQRTHRIRAANFAALRPWPGLT

VEIRKSAPAQ

>CYP139A1(2584878425)Mycobacterium tuberculosis KT-0098

MRYPLGEALLALYRWRGPLINAGVGGHGYTYLLGAEANRFVFANADAFSWSQTFESLVPV

DGPTALIVSDGADHRRRRSVVAPGLRHHHVQRYVATMVSNIDTVIDGWQPGQRLDIYQEL

RSAVRRSTAESLFGQRLAVHSDFLGEQLQPLLDLTRRPPQVMRLQQRVNSPGWRRAMAAR

KRIDDLIDAQIADARTAPRPDDHMLTTLISGCSEEGTTLSDNEIRDSIVSLITAGYETTS

GALAWAIYALLTVPGTWESAASEVARVLGGRVPAADDLSALTYLNGVVHETLRLYSPGVI

SARRVLRDLWFDGHRIRAGRLLIFSAYVTHRLPEIWPEPTEFRPLRWDPNAADYRKPAPH

EFIPFSGGLHRCIGAVMATTEMTVILARLVARAMLQLPAQRTHRIRAANFAALRPWPGLT

VEIRKSAPAQ

>CYP139A1(2584990570)Mycobacterium tuberculosis BTB07-254

MRYPLGEALLALYRWRGPLINAGVGGHGYTYLLGAEANRFVFANADAFSWSQTFESLVPV

DGPTALIVSDGADHRRRRSVVAPGLRHHHVQRYVATMVSNIDTVIDGWQPGQRLDIYQEL

RSAVRRSTAESLFGQRLAVHSDFLGEQLQPLLDLTRRPPQVMRLQQRVNSPGWRRAMAAR

KRIDDLIDAQIADARTAPRPDDHMLTTLISGCSEEGTTLSDNEIRDSIVSLITAGYETTS

GALAWAIYALLTVPGTWESAASEVARVLGGRVPAADDLSALTYLNGVVHETLRLYSPGVI

SARRVLRDLWFDGHRIRAGRLLIFSAYVTHRLPEIWPEPTEFRPLRWDPNAADYRKPAPH

EFIPFSGGLHRCIGAVMATTEMTVILARLVARAMLQLPAQRTHRIRAANFAALRPWPGLT

VEIRKSAPAQ

>CYP139A1(2590014899)Mycobacterium tuberculosis MAL010080

MRYPLGEALLALYRWRGPLINAGVGGHGYTYLLGAEANRFVFANADAFSWSQTFESLVPV

DGPTALIVSDGADHRRRRSVVAPGLRHHHVQRYVATMVSNIDTVIDGWQPGQRLDIYQEL

RSAVRRSTAESLFGQRLAVHSDFLGEQLQPLLDLTRRPPQVMRLQQRVNSPGWRRAMAAR

KRIDDLIDAQIADARTAPRPDDHMLTTLISGCSEEGTTLSDNEIRDSIVSLITAGYETTS

GALAWAIYALLTVPGTWESAASEVARVLGGRVPAADDLSALTYLNGVVHETLRLYSPGVI

SARRVLRDLWFDGHRIRAGRLLIFSAYVTHRLPEIWPEPTEFRPLRWDPNAADYRKPAPH

EFIPFSGGLHRCIGAVMATTEMTVILARLVARAMLQLPAQRTHRIRAANFAALRPWPGLT

VEIRKSAPAQ

>CYP139A1(2590313607)Mycobacterium tuberculosis Haarlem

MRYPLGEALLALYRWRGPLINAGVGGHGYTYLLGAEANRFVFANADAFSWSQTFESLVPV

DGPTALIVSDGADHRRRRSVVAPGLRHHHVQRYVATMVSNIDTVIDGWQPGQRLDIYQEL

RSAVRRSTAESLFGQRLAVHSDFLGEQLQPLLDLTRRPPQVMRLQQRVNSPGWRRAMAAR

KRIDDLIDAQIADARTAPRPDDHMLTTLISGCSEEGTTLSDNEIRDSIVSLITAGYETTS

GALAWAIYALLTVPGTWESAASEVARVLGGRVPAADDLSALTYLNGVVHETLRLYSPGVI

SARRVLRDLWFDGHRIRAGRLLIFSAYVTHRLPEIWPEPTEFRPLRWDPNAADYRKPAPH

EFIPFSGGLHRCIGAVMATTEMTVILARLVARAMLQLPAQRTHRIRAANFAALRPWPGLT

VEIRKSAPAQ

>CYP139A1(2590563570)Mycobacterium tuberculosis KT-0064

MRYPLGEALLALYRWRGPLINAGVGGHGYTYLLGAEANRFVFANADAFSWSQTFESLVPV

DGPTALIVSDGADHRRRRSVVAPGLRHHHVQRYVATMVSNIDTVIDGWQPGQRLDIYQEL

RSAVRRSTAESLFGQRLAVHSDFLGEQLQPLLDLTRRPPQVMRLQQRVNSPGWRRAMAAR

KRIDDLIDAQIADARTAPRPDDHMLTTLISGCSEEGTTLSDNEIRDSIVSLITAGYETTS

GALAWAIYALLTVPGTWESAASEVARVLGGRVPAADDLSALTYLNGVVHETLRLYSPGVI

SARRVLRDLWFDGHRIRAGRLLIFSAYVTHRLPEIWPEPTEFRPLRWDPNAADYRKPAPH

EFIPFSGGLHRCIGAVMATTEMTVILARLVARAMLQLPAQRTHRIRAANFAALRPWPGLT

VEIRKSAPAQ

>CYP139A1(2574682482)Mycobacterium tuberculosis MAL020181

MRYPLGEALLALYRWRGPLINAGVGGHGYTYLLGAEANRFVFANADAFSWSQTFESLVPV

DGPTALIVSDGADHRRRRSVVAPGLRHHHVQRYVATMVSNIDTVIDGWQPGQRLDIYQEL

RSAVRRSTAESLFGQRLAVHSDFLGEQLQPLLDLTRRPPQVMRLQQRVNSPGWRRAMAAR

KRIDDLIDAQIADARTAPRPDDHMLTTLISGCSEEGTTLSDNEIRDSIVSLITAGYETTS

GALAWAIYALLTVPGTWESAASEVARVLGGRVPAADDLSALTYLNGVVHETLRLYSPGVI

SARRVLRDLWFDGHRIRAGRLLIFSAYVTHRLPEIWPEPTEFRPLRWDPNAADYRKPAPH

EFIPFSGGLHRCIGAVMATTEMTVILARLVARAMLQLPAQRTHRIRAANFAALRPWPGLT

VEIRKSAPAQ

>CYP139A1(2574937709)Mycobacterium tuberculosis TB_RSA173

MRYPLGEALLALYRWRGPLINAGVGGHGYTYLLGAEANRFVFANADAFSWSQTFESLVPV

DGPTALIVSDGADHRRRRSVVAPGLRHHHVQRYVATMVSNIDTVIDGWQPGQRLDIYQEL

RSAVRRSTAESLFGQRLAVHSDFLGEQLQPLLDLTRRPPQVMRLQQRVNSPGWRRAMAAR

KRIDDLIDAQIADARTAPRPDDHMLTTLISGCSEEGTTLSDNEIRDSIVSLITAGYETTS

GALAWAIYALLTVPGTWESAASEVARVLGGRVPAADDLSALTYLNGVVHETLRLYSPGVI

SARRVLRDLWFDGHRIRAGRLLIFSAYVTHRLPEIWPEPTEFRPLRWDPNAADYRKPAPH

EFIPFSGGLHRCIGAVMATTEMTVILARLVARAMLQLPAQRTHRIRAANFAALRPWPGLT

VEIRKSAPAQ

>CYP139A1(2575051511)Mycobacterium tuberculosis MD13878

MRYPLGEALLALYRWRGPLINAGVGGHGYTYLLGAEANRFVFANADAFSWSQTFESLVPV

DGPTALIVSDGADHRRRRSVVAPGLRHHHVQRYVATMVSNIDTVIDGWQPGQRLDIYQEL

RSAVRRSTAESLFGQRLAVHSDFLGEQLQPLLDLTRRPPQVMRLQQRVNSPGWRRAMAAR

KRIDDLIDAQIADARTAPRPDDHMLTTLISGCSEEGTTLSDNEIRDSIVSLITAGYETTS

GALAWAIYALLTVPGTWESAASEVARVLGGRVPAADDLSALTYLNGVVHETLRLYSPGVI

SARRVLRDLWFDGHRIRAGRLLIFSAYVTHRLPEIWPEPTEFRPLRWDPNAADYRKPAPH

EFIPFSGGLHRCIGAVMATTEMTVILARLVARAMLQLPAQRTHRIRAANFAALRPWPGLT

VEIRKSAPAQ

>CYP139A1(2575142685)Mycobacterium tuberculosis TB_RSA174

MRYPLGEALLALYRWRGPLINAGVGGHGYTYLLGAEANRFVFANADAFSWSQTFESLVPV

DGPTALIVSDGADHRRRRSVVAPGLRHHHVQRYVATMVSNIDTVIDGWQPGQRLDIYQEL

RSAVRRSTAESLFGQRLAVHSDFLGEQLQPLLDLTRRPPQVMRLQQRVNSPGWRRAMAAR

KRIDDLIDAQIADARTAPRPDDHMLTTLISGCSEEGTTLSDNEIRDSIVSLITAGYETTS

GALAWAIYALLTVPGTWESAASEVARVLGGRVPAADDLSALTYLNGVVHETLRLYSPGVI

SARRVLRDLWFDGHRIRAGRLLIFSAYVTHRLPEIWPEPTEFRPLRWDPNAADYRKPAPH

EFIPFSGGLHRCIGAVMATTEMTVILARLVARAMLQLPAQRTHRIRAANFAALRPWPGLT

VEIRKSAPAQ

>CYP139A1(2575538950)Mycobacterium tuberculosis TKK-01-0011

MRYPLGEALLALYRWRGPLINAGVGGHGYTYLLGAEANRFVFANADAFSWSQTFESLVPV

DGPTALIVSDGADHRRRRSVVAPGLRHHHVQRYVATMVSNIDTVIDGWQPGQRLDIYQEL

RSAVRRSTAESLFGQRLAVHSDFLGEQLQPLLDLTRRPPQVMRLQQRVNSPGWRRAMAAR

KRIDDLIDAQIADARTAPRPDDHMLTTLISGCSEEGTTLSDNEIRDSIVSLITAGYETTS

GALAWAIYALLTVPGTWESAASEVARVLGGRVPAADDLSALTYLNGVVHETLRLYSPGVI

SARRVLRDLWFDGHRIRAGRLLIFSAYVTHRLPEIWPEPTEFRPLRWDPNAADYRKPAPH

EFIPFSGGLHRCIGAVMATTEMTVILARLVARAMLQLPAQRTHRIRAANFAALRPWPGLT

VEIRKSAPAQ

>CYP139A1(2575858812)Mycobacterium tuberculosis TKK_03_0042

MRYPLGEALLALYRWRGPLINAGVGGHGYTYLLGAEANRFVFANADAFSWSQTFESLVPV

DGPTALIVSDGADHRRRRSVVAPGLRHHHVQRYVATMVSNIDTVIDGWQPGQRLDIYQEL

RSAVRRSTAESLFGQRLAVHSDFLGEQLQPLLDLTRRPPQVMRLQQRVNSPGWRRAMAAR

KRIDDLIDAQIADARTAPRPDDHMLTTLISGCSEEGTTLSDNEIRDSIVSLITAGYETTS

GALAWAIYALLTVPGTWESAASEVARVLGGRVPAADDLSALTYLNGVVHETLRLYSPGVI

SARRVLRDLWFDGHRIRAGRLLIFSAYVTHRLPEIWPEPTEFRPLRWDPNAADYRKPAPH

EFIPFSGGLHRCIGAVMATTEMTVILARLVARAMLQLPAQRTHRIRAANFAALRPWPGLT

VEIRKSAPAQ

>CYP139A1(2576059958)Mycobacterium tuberculosis TB_RSA199

MRYPLGEALLALYRWRGPLINAGVGGHGYTYLLGAEANRFVFANADAFSWSQTFESLVPV

DGPTALIVSDGADHRRRRSVVAPGLRHHHVQRYVATMVSNIDTVIDGWQPGQRLDIYQEL

RSAVRRSTAESLFGQRLAVHSDFLGEQLQPLLDLTRRPPQVMRLQQRVNSPGWRRAMAAR

KRIDDLIDAQIADARTAPRPDDHMLTTLISGCSEEGTTLSDNEIRDSIVSLITAGYETTS

GALAWAIYALLTVPGTWESAASEVARVLGGRVPAADDLSALTYLNGVVHETLRLYSPGVI

SARRVLRDLWFDGHRIRAGRLLIFSAYVTHRLPEIWPEPTEFRPLRWDPNAADYRKPAPH

EFIPFSGGLHRCIGAVMATTEMTVILARLVARAMLQLPAQRTHRIRAANFAALRPWPGLT

VEIRKSAPAQ

>CYP139A1(2576471218)Mycobacterium tuberculosis OFXR-3

MRYPLGEALLALYRWRGPLINAGVGGHGYTYLLGAEANRFVFANADAFSWSQTFESLVPV

DGPTALIVSDGADHRRRRSVVAPGLRHHHVQRYVATMVSNIDTVIDGWQPGQRLDIYQEL

RSAVRRSTAESLFGQRLAVHSDFLGEQLQPLLDLTRRPPQVMRLQQRVNSPGWRRAMAAR

KRIDDLIDAQIADARTAPRPDDHMLTTLISGCSEEGTTLSDNEIRDSIVSLITAGYETTS

GALAWAIYALLTVPGTWESAASEVARVLGGRVPAADDLSALTYLNGVVHETLRLYSPGVI

SARRVLRDLWFDGHRIRAGRLLIFSAYVTHRLPEIWPEPTEFRPLRWDPNAADYRKPAPH

EFIPFSGGLHRCIGAVMATTEMTVILARLVARAMLQLPAQRTHRIRAANFAALRPWPGLT

VEIRKSAPAQ

>CYP139A1(2576497080)Mycobacterium tuberculosis TKK_04_0140

MRYPLGEALLALYRWRGPLINAGVGGHGYTYLLGAEANRFVFANADAFSWSQTFESLVPV

DGPTALIVSDGADHRRRRSVVAPGLRHHHVQRYVATMVSNIDTVIDGWQPGQRLDIYQEL

RSAVRRSTAESLFGQRLAVHSDFLGEQLQPLLDLTRRPPQVMRLQQRVNSPGWRRAMAAR

KRIDDLIDAQIADARTAPRPDDHMLTTLISGCSEEGTTLSDNEIRDSIVSLITAGYETTS

GALAWAIYALLTVPGTWESAASEVARVLGGRVPAADDLSALTYLNGVVHETLRLYSPGVI

SARRVLRDLWFDGHRIRAGRLLIFSAYVTHRLPEIWPEPTEFRPLRWDPNAADYRKPAPH

EFIPFSGGLHRCIGAVMATTEMTVILARLVARAMLQLPAQRTHRIRAANFAALRPWPGLT

VEIRKSAPAQ

>CYP139A1(2577038023)Mycobacterium tuberculosis TB_RSA178

MRYPLGEALLALYRWRGPLINAGVGGHGYTYLLGAEANRFVFANADAFSWSQTFESLVPV

DGPTALIVSDGADHRRRRSVVAPGLRHHHVQRYVATMVSNIDTVIDGWQPGQRLDIYQEL

RSAVRRSTAESLFGQRLAVHSDFLGEQLQPLLDLTRRPPQVMRLQQRVNSPGWRRAMAAR

KRIDDLIDAQIADARTAPRPDDHMLTTLISGCSEEGTTLSDNEIRDSIVSLITAGYETTS

GALAWAIYALLTVPGTWESAASEVARVLGGRVPAADDLSALTYLNGVVHETLRLYSPGVI

SARRVLRDLWFDGHRIRAGRLLIFSAYVTHRLPEIWPEPTEFRPLRWDPNAADYRKPAPH

EFIPFSGGLHRCIGAVMATTEMTVILARLVARAMLQLPAQRTHRIRAANFAALRPWPGLT

VEIRKSAPAQ

>CYP139A1(2577203844)Mycobacterium tuberculosis TB_RSA77

MRYPLGEALLALYRWRGPLINAGVGGHGYTYLLGAEANRFVFANADAFSWSQTFESLVPV

DGPTALIVSDGADHRRRRSVVAPGLRHHHVQRYVATMVSNIDTVIDGWQPGQRLDIYQEL

RSAVRRSTAESLFGQRLAVHSDFLGEQLQPLLDLTRRPPQVMRLQQRVNSPGWRRAMAAR

KRIDDLIDAQIADARTAPRPDDHMLTTLISGCSEEGTTLSDNEIRDSIVSLITAGYETTS

GALAWAIYALLTVPGTWESAASEVARVLGGRVPAADDLSALTYLNGVVHETLRLYSPGVI

SARRVLRDLWFDGHRIRAGRLLIFSAYVTHRLPEIWPEPTEFRPLRWDPNAADYRKPAPH

EFIPFSGGLHRCIGAVMATTEMTVILARLVARAMLQLPAQRTHRIRAANFAALRPWPGLT

VEIRKSAPAQ

>CYP139A1(2577322951)Mycobacterium tuberculosis TKK-01-0047

MRYPLGEALLALYRWRGPLINAGVGGHGYTYLLGAEANRFVFANADAFSWSQTFESLVPV

DGPTALIVSDGADHRRRRSVVAPGLRHHHVQRYVATMVSNIDTVIDGWQPGQRLDIYQEL

RSAVRRSTAESLFGQRLAVHSDFLGEQLQPLLDLTRRPPQVMRLQQRVNSPGWRRAMAAR

KRIDDLIDAQIADARTAPRPDDHMLTTLISGCSEEGTTLSDNEIRDSIVSLITAGYETTS

GALAWAIYALLTVPGTWESAASEVARVLGGRVPAADDLSALTYLNGVVHETLRLYSPGVI

SARRVLRDLWFDGHRIRAGRLLIFSAYVTHRLPEIWPEPTEFRPLRWDPNAADYRKPAPH

EFIPFSGGLHRCIGAVMATTEMTVILARLVARAMLQLPAQRTHRIRAANFAALRPWPGLT

VEIRKSAPAQ

>CYP139A1(2577422627)Mycobacterium tuberculosis MD16265

MRYPLGEALLALYRWRGPLINAGVGGHGYTYLLGAEANRFVFANADAFSWSQTFESLVPV

DGPTALIVSDGADHRRRRSVVAPGLRHHHVQRYVATMVSNIDTVIDGWQPGQRLDIYQEL

RSAVRRSTAESLFGQRLAVHSDFLGEQLQPLLDLTRRPPQVMRLQQRVNSPGWRRAMAAR

KRIDDLIDAQIADARTAPRPDDHMLTTLISGCSEEGTTLSDNEIRDSIVSLITAGYETTS

GALAWAIYALLTVPGTWESAASEVARVLGGRVPAADDLSALTYLNGVVHETLRLYSPGVI

SARRVLRDLWFDGHRIRAGRLLIFSAYVTHRLPEIWPEPTEFRPLRWDPNAADYRKPAPH

EFIPFSGGLHRCIGAVMATTEMTVILARLVARAMLQLPAQRTHRIRAANFAALRPWPGLT

VEIRKSAPAQ

>CYP139A1(2577551091)Mycobacterium tuberculosis TB_RSA127

MRYPLGEALLALYRWRGPLINAGVGGHGYTYLLGAEANRFVFANADAFSWSQTFESLVPV

DGPTALIVSDGADHRRRRSVVAPGLRHHHVQRYVATMVSNIDTVIDGWQPGQRLDIYQEL

RSAVRRSTAESLFGQRLAVHSDFLGEQLQPLLDLTRRPPQVMRLQQRVNSPGWRRAMAAR

KRIDDLIDAQIADARTAPRPDDHMLTTLISGCSEEGTTLSDNEIRDSIVSLITAGYETTS

GALAWAIYALLTVPGTWESAASEVARVLGGRVPAADDLSALTYLNGVVHETLRLYSPGVI

SARRVLRDLWFDGHRIRAGRLLIFSAYVTHRLPEIWPEPTEFRPLRWDPNAADYRKPAPH

EFIPFSGGLHRCIGAVMATTEMTVILARLVARAMLQLPAQRTHRIRAANFAALRPWPGLT

VEIRKSAPAQ

>CYP139A1(2584755863)Mycobacterium tuberculosis XTB13-086

MRYPLGEALLALYRWRGPLINAGVGGHGYTYLLGAEANRFVFANADAFSWSQTFESLVPV

DGPTALIVSDGADHRRRRSVVAPGLRHHHVQRYVATMVSNIDTVIDGWQPGQRLDIYQEL

RSAVRRSTAESLFGQRLAVHSDFLGEQLQPLLDLTRRPPQVMRLQQRVNSPGWRRAMAAR

KRIDDLIDAQIADARTAPRPDDHMLTTLISGCSEEGTTLSDNEIRDSIVSLITAGYETTS

GALAWAIYALLTVPGTWESAASEVARVLGGRVPAADDLSALTYLNGVVHETLRLYSPGVI

SARRVLRDLWFDGHRIRAGRLLIFSAYVTHRLPEIWPEPTEFRPLRWDPNAADYRKPAPH

EFIPFSGGLHRCIGAVMATTEMTVILARLVARAMLQLPAQRTHRIRAANFAALRPWPGLT

VEIRKSAPAQ

>CYP139A1(2589117553)Mycobacterium tuberculosis TBR60

MRYPLGEALLALYRWRGPLINAGVGGHGYTYLLGAEANRFVFANADAFSWSQTFESLVPV

DGPTALIVSDGADHRRRRSVVAPGLRHHHVQRYVATMVSNIDTVIDGWQPGQRLDIYQEL

RSAVRRSTAESLFGQRLAVHSDFLGEQLQPLLDLTRRPPQVMRLQQRVNSPGWRRAMAAR

KRIDDLIDAQIADARTAPRPDDHMLTTLISGCSEEGTTLSDNEIRDSIVSLITAGYETTS

GALAWAIYALLTVPGTWESAASEVARVLGGRVPAADDLSALTYLNGVVHETLRLYSPGVI

SARRVLRDLWFDGHRIRAGRLLIFSAYVTHRLPEIWPEPTEFRPLRWDPNAADYRKPAPH

EFIPFSGGLHRCIGAVMATTEMTVILARLVARAMLQLPAQRTHRIRAANFAALRPWPGLT

VEIRKSAPAQ

>CYP139A1(2589121735)Mycobacterium tuberculosis TBR74

MRYPLGEALLALYRWRGPLINAGVGGHGYTYLLGAEANRFVFANADAFSWSQTFESLVPV

DGPTALIVSDGADHRRRRSVVAPGLRHHHVQRYVATMVSNIDTVIDGWQPGQRLDIYQEL

RSAVRRSTAESLFGQRLAVHSDFLGEQLQPLLDLTRRPPQVMRLQQRVNSPGWRRAMAAR

KRIDDLIDAQIADARTAPRPDDHMLTTLISGCSEEGTTLSDNEIRDSIVSLITAGYETTS

GALAWAIYALLTVPGTWESAASEVARVLGGRVPAADDLSALTYLNGVVHETLRLYSPGVI

SARRVLRDLWFDGHRIRAGRLLIFSAYVTHRLPEIWPEPTEFRPLRWDPNAADYRKPAPH

EFIPFSGGLHRCIGAVMATTEMTVILARLVARAMLQLPAQRTHRIRAANFAALRPWPGLT

VEIRKSAPAQ

>CYP139A1(2589506393)Mycobacterium tuberculosis TKK-01-0004

MRYPLGEALLALYRWRGPLINAGVGGHGYTYLLGAEANRFVFANADAFSWSQTFESLVPV

DGPTALIVSDGADHRRRRSVVAPGLRHHHVQRYVATMVSNIDTVIDGWQPGQRLDIYQEL

RSAVRRSTAESLFGQRLAVHSDFLGEQLQPLLDLTRRPPQVMRLQQRVNSPGWRRAMAAR

KRIDDLIDAQIADARTAPRPDDHMLTTLISGCSEEGTTLSDNEIRDSIVSLITAGYETTS

GALAWAIYALLTVPGTWESAASEVARVLGGRVPAADDLSALTYLNGVVHETLRLYSPGVI

SARRVLRDLWFDGHRIRAGRLLIFSAYVTHRLPEIWPEPTEFRPLRWDPNAADYRKPAPH

EFIPFSGGLHRCIGAVMATTEMTVILARLVARAMLQLPAQRTHRIRAANFAALRPWPGLT

VEIRKSAPAQ

>CYP139A1(2590044637)Mycobacterium tuberculosis MAL010117

MRYPLGEALLALYRWRGPLINAGVGGHGYTYLLGAEANRFVFANADAFSWSQTFESLVPV

DGPTALIVSDGADHRRRRSVVAPGLRHHHVQRYVATMVSNIDTVIDGWQPGQRLDIYQEL

RSAVRRSTAESLFGQRLAVHSDFLGEQLQPLLDLTRRPPQVMRLQQRVNSPGWRRAMAAR

KRIDDLIDAQIADARTAPRPDDHMLTTLISGCSEEGTTLSDNEIRDSIVSLITAGYETTS

GALAWAIYALLTVPGTWESAASEVARVLGGRVPAADDLSALTYLNGVVHETLRLYSPGVI

SARRVLRDLWFDGHRIRAGRLLIFSAYVTHRLPEIWPEPTEFRPLRWDPNAADYRKPAPH

EFIPFSGGLHRCIGAVMATTEMTVILARLVARAMLQLPAQRTHRIRAANFAALRPWPGLT

VEIRKSAPAQ

>CYP139A1(2590137275)Mycobacterium tuberculosis MAL020195

MRYPLGEALLALYRWRGPLINAGVGGHGYTYLLGAEANRFVFANADAFSWSQTFESLVPV

DGPTALIVSDGADHRRRRSVVAPGLRHHHVQRYVATMVSNIDTVIDGWQPGQRLDIYQEL

RSAVRRSTAESLFGQRLAVHSDFLGEQLQPLLDLTRRPPQVMRLQQRVNSPGWRRAMAAR

KRIDDLIDAQIADARTAPRPDDHMLTTLISGCSEEGTTLSDNEIRDSIVSLITAGYETTS

GALAWAIYALLTVPGTWESAASEVARVLGGRVPAADDLSALTYLNGVVHETLRLYSPGVI

SARRVLRDLWFDGHRIRAGRLLIFSAYVTHRLPEIWPEPTEFRPLRWDPNAADYRKPAPH

EFIPFSGGLHRCIGAVMATTEMTVILARLVARAMLQLPAQRTHRIRAANFAALRPWPGLT

VEIRKSAPAQ

>CYP139A1(2590345757)Mycobacterium tuberculosis OFXR-32

MRYPLGEALLALYRWRGPLINAGVGGHGYTYLLGAEANRFVFANADAFSWSQTFESLVPV

DGPTALIVSDGADHRRRRSVVAPGLRHHHVQRYVATMVSNIDTVIDGWQPGQRLDIYQEL

RSAVRRSTAESLFGQRLAVHSDFLGEQLQPLLDLTRRPPQVMRLQQRVNSPGWRRAMAAR

KRIDDLIDAQIADARTAPRPDDHMLTTLISGCSEEGTTLSDNEIRDSIVSLITAGYETTS

GALAWAIYALLTVPGTWESAASEVARVLGGRVPAADDLSALTYLNGVVHETLRLYSPGVI

SARRVLRDLWFDGHRIRAGRLLIFSAYVTHRLPEIWPEPTEFRPLRWDPNAADYRKPAPH

EFIPFSGGLHRCIGAVMATTEMTVILARLVARAMLQLPAQRTHRIRAANFAALRPWPGLT

VEIRKSAPAQ

>CYP139A1(2592275464)Mycobacterium tuberculosis TKK_03_0082

MRYPLGEALLALYRWRGPLINAGVGGHGYTYLLGAEANRFVFANADAFSWSQTFESLVPV

DGPTALIVSDGADHRRRRSVVAPGLRHHHVQRYVATMVSNIDTVIDGWQPGQRLDIYQEL

RSAVRRSTAESLFGQRLAVHSDFLGEQLQPLLDLTRRPPQVMRLQQRVNSPGWRRAMAAR

KRIDDLIDAQIADARTAPRPDDHMLTTLISGCSEEGTTLSDNEIRDSIVSLITAGYETTS

GALAWAIYALLTVPGTWESAASEVARVLGGRVPAADDLSALTYLNGVVHETLRLYSPGVI

SARRVLRDLWFDGHRIRAGRLLIFSAYVTHRLPEIWPEPTEFRPLRWDPNAADYRKPAPH

EFIPFSGGLHRCIGAVMATTEMTVILARLVARAMLQLPAQRTHRIRAANFAALRPWPGLT

VEIRKSAPAQ

>CYP139A1(2575515927)Mycobacterium tuberculosis M1221

MRYPLGEALLALYRWRGPLINAGVGGHGYTYLLGAEANRFVFANADAFSWSQTFESLVPV

DGPTALIVSDGADHRRRRSVVAPGLRHHHVQRYVATMVSNIDTVIDGWQPGQRLDIYQEL

RSAVRRSTAESLFGQRLAVHSDFLGEQLQPLLDLTRRPPQVMRLQQRVNSPGWRRAMAAR

KRIDDLIDAQIADARTAPRPDDHMLTTLISGCSEEGTTLSDNEIRDSIVSLITAGYETTS

GALAWAIYALLTVPGTWESAASEVARVLGGRVPAADDLSALTYLNGVVHETLRLYSPGVI

SARRVLRDLWFDGHRIRAGRLLIFSAYVTHRLPEIWPEPTEFRPLRWDPNAADYRKPAPH

EFIPFSGGLHRCIGAVMATTEMTVILARLVARAMLQLPAQRTHRIRAANFAALRPWPGLT

VEIRKSAPAQ

>CYP139A1(2576459372)Mycobacterium tuberculosis MAL010109

MRYPLGEALLALYRWRGPLINAGVGGHGYTYLLGAEANRFVFANADAFSWSQTFESLVPV

DGPTALIVSDGADHRRRRSVVAPGLRHHHVQRYVATMVSNIDTVIDGWQPGQRLDIYQEL

RSAVRRSTAESLFGQRLAVHSDFLGEQLQPLLDLTRRPPQVMRLQQRVNSPGWRRAMAAR

KRIDDLIDAQIADARTAPRPDDHMLTTLISGCSEEGTTLSDNEIRDSIVSLITAGYETTS

GALAWAIYALLTVPGTWESAASEVARVLGGRVPAADDLSALTYLNGVVHETLRLYSPGVI

SARRVLRDLWFDGHRIRAGRLLIFSAYVTHRLPEIWPEPTEFRPLRWDPNAADYRKPAPH

EFIPFSGGLHRCIGAVMATTEMTVILARLVARAMLQLPAQRTHRIRAANFAALRPWPGLT

VEIRKSAPAQ

>CYP139A1(2576522568)Mycobacterium tuberculosis KT-0037

MRYPLGEALLALYRWRGPLINAGVGGHGYTYLLGAEANRFVFANADAFSWSQTFESLVPV

DGPTALIVSDGADHRRRRSVVAPGLRHHHVQRYVATMVSNIDTVIDGWQPGQRLDIYQEL

RSAVRRSTAESLFGQRLAVHSDFLGEQLQPLLDLTRRPPQVMRLQQRVNSPGWRRAMAAR

KRIDDLIDAQIADARTAPRPDDHMLTTLISGCSEEGTTLSDNEIRDSIVSLITAGYETTS

GALAWAIYALLTVPGTWESAASEVARVLGGRVPAADDLSALTYLNGVVHETLRLYSPGVI

SARRVLRDLWFDGHRIRAGRLLIFSAYVTHRLPEIWPEPTEFRPLRWDPNAADYRKPAPH

EFIPFSGGLHRCIGAVMATTEMTVILARLVARAMLQLPAQRTHRIRAANFAALRPWPGLT

VEIRKSAPAQ

>CYP139A1(2576559538)Mycobacterium tuberculosis 1173CS

MRYPLGEALLALYRWRGPLINAGVGGHGYTYLLGAEANRFVFANADAFSWSQTFESLVPV

DGPTALIVSDGADHRRRRSVVAPGLRHHHVQRYVATMVSNIDTVIDGWQPGQRLDIYQEL

RSAVRRSTAESLFGQRLAVHSDFLGEQLQPLLDLTRRPPQVMRLQQRVNSPGWRRAMAAR

KRIDDLIDAQIADARTAPRPDDHMLTTLISGCSEEGTTLSDNEIRDSIVSLITAGYETTS

GALAWAIYALLTVPGTWESAASEVARVLGGRVPAADDLSALTYLNGVVHETLRLYSPGVI

SARRVLRDLWFDGHRIRAGRLLIFSAYVTHRLPEIWPEPTEFRPLRWDPNAADYRKPAPH

EFIPFSGGLHRCIGAVMATTEMTVILARLVARAMLQLPAQRTHRIRAANFAALRPWPGLT

VEIRKSAPAQ

>CYP139A1(2577302440)Mycobacterium tuberculosis M2006

MRYPLGEALLALYRWRGPLINAGVGGHGYTYLLGAEANRFVFANADAFSWSQTFESLVPV

DGPTALIVSDGADHRRRRSVVAPGLRHHHVQRYVATMVSNIDTVIDGWQPGQRLDIYQEL

RSAVRRSTAESLFGQRLAVHSDFLGEQLQPLLDLTRRPPQVMRLQQRVNSPGWRRAMAAR

KRIDDLIDAQIADARTAPRPDDHMLTTLISGCSEEGTTLSDNEIRDSIVSLITAGYETTS

GALAWAIYALLTVPGTWESAASEVARVLGGRVPAADDLSALTYLNGVVHETLRLYSPGVI

SARRVLRDLWFDGHRIRAGRLLIFSAYVTHRLPEIWPEPTEFRPLRWDPNAADYRKPAPH

EFIPFSGGLHRCIGAVMATTEMTVILARLVARAMLQLPAQRTHRIRAANFAALRPWPGLT

VEIRKSAPAQ

>CYP139A1(2577448440)Mycobacterium tuberculosis MD15855

MRYPLGEALLALYRWRGPLINAGVGGHGYTYLLGAEANRFVFANADAFSWSQTFESLVPV

DGPTALIVSDGADHRRRRSVVAPGLRHHHVQRYVATMVSNIDTVIDGWQPGQRLDIYQEL

RSAVRRSTAESLFGQRLAVHSDFLGEQLQPLLDLTRRPPQVMRLQQRVNSPGWRRAMAAR

KRIDDLIDAQIADARTAPRPDDHMLTTLISGCSEEGTTLSDNEIRDSIVSLITAGYETTS

GALAWAIYALLTVPGTWESAASEVARVLGGRVPAADDLSALTYLNGVVHETLRLYSPGVI

SARRVLRDLWFDGHRIRAGRLLIFSAYVTHRLPEIWPEPTEFRPLRWDPNAADYRKPAPH

EFIPFSGGLHRCIGAVMATTEMTVILARLVARAMLQLPAQRTHRIRAANFAALRPWPGLT

VEIRKSAPAQ

>CYP139A1(2577462548)Mycobacterium tuberculosis 02_1987

MRYPLGEALLALYRWRGPLINAGVGGHGYTYLLGAEANRFVFANADAFSWSQTFESLVPV

DGPTALIVSDGADHRRRRSVVAPGLRHHHVQRYVATMVSNIDTVIDGWQPGQRLDIYQEL

RSAVRRSTAESLFGQRLAVHSDFLGEQLQPLLDLTRRPPQVMRLQQRVNSPGWRRAMAAR

KRIDDLIDAQIADARTAPRPDDHMLTTLISGCSEEGTTLSDNEIRDSIVSLITAGYETTS

GALAWAIYALLTVPGTWESAASEVARVLGGRVPAADDLSALTYLNGVVHETLRLYSPGVI

SARRVLRDLWFDGHRIRAGRLLIFSAYVTHRLPEIWPEPTEFRPLRWDPNAADYRKPAPH

EFIPFSGGLHRCIGAVMATTEMTVILARLVARAMLQLPAQRTHRIRAANFAALRPWPGLT

VEIRKSAPAQ

>CYP139A1(2584678567)Mycobacterium tuberculosis BTB07-246

MRYPLGEALLALYRWRGPLINAGVGGHGYTYLLGAEANRFVFANADAFSWSQTFESLVPV

DGPTALIVSDGADHRRRRSVVAPGLRHHHVQRYVATMVSNIDTVIDGWQPGQRLDIYQEL

RSAVRRSTAESLFGQRLAVHSDFLGEQLQPLLDLTRRPPQVMRLQQRVNSPGWRRAMAAR

KRIDDLIDAQIADARTAPRPDDHMLTTLISGCSEEGTTLSDNEIRDSIVSLITAGYETTS

GALAWAIYALLTVPGTWESAASEVARVLGGRVPAADDLSALTYLNGVVHETLRLYSPGVI

SARRVLRDLWFDGHRIRAGRLLIFSAYVTHRLPEIWPEPTEFRPLRWDPNAADYRKPAPH

EFIPFSGGLHRCIGAVMATTEMTVILARLVARAMLQLPAQRTHRIRAANFAALRPWPGLT

VEIRKSAPAQ

>CYP139A1(2584763258)Mycobacterium tuberculosis MAL020145

MRYPLGEALLALYRWRGPLINAGVGGHGYTYLLGAEANRFVFANADAFSWSQTFESLVPV

DGPTALIVSDGADHRRRRSVVAPGLRHHHVQRYVATMVSNIDTVIDGWQPGQRLDIYQEL

RSAVRRSTAESLFGQRLAVHSDFLGEQLQPLLDLTRRPPQVMRLQQRVNSPGWRRAMAAR

KRIDDLIDAQIADARTAPRPDDHMLTTLISGCSEEGTTLSDNEIRDSIVSLITAGYETTS

GALAWAIYALLTVPGTWESAASEVARVLGGRVPAADDLSALTYLNGVVHETLRLYSPGVI

SARRVLRDLWFDGHRIRAGRLLIFSAYVTHRLPEIWPEPTEFRPLRWDPNAADYRKPAPH

EFIPFSGGLHRCIGAVMATTEMTVILARLVARAMLQLPAQRTHRIRAANFAALRPWPGLT

VEIRKSAPAQ

>CYP139A1(2584949783)Mycobacterium tuberculosis MD15597

MRYPLGEALLALYRWRGPLINAGVGGHGYTYLLGAEANRFVFANADAFSWSQTFESLVPV

DGPTALIVSDGADHRRRRSVVAPGLRHHHVQRYVATMVSNIDTVIDGWQPGQRLDIYQEL

RSAVRRSTAESLFGQRLAVHSDFLGEQLQPLLDLTRRPPQVMRLQQRVNSPGWRRAMAAR

KRIDDLIDAQIADARTAPRPDDHMLTTLISGCSEEGTTLSDNEIRDSIVSLITAGYETTS

GALAWAIYALLTVPGTWESAASEVARVLGGRVPAADDLSALTYLNGVVHETLRLYSPGVI

SARRVLRDLWFDGHRIRAGRLLIFSAYVTHRLPEIWPEPTEFRPLRWDPNAADYRKPAPH

EFIPFSGGLHRCIGAVMATTEMTVILARLVARAMLQLPAQRTHRIRAANFAALRPWPGLT

VEIRKSAPAQ

>CYP139A1(2589146274)Mycobacterium tuberculosis OFXR-14

MRYPLGEALLALYRWRGPLINAGVGGHGYTYLLGAEANRFVFANADAFSWSQTFESLVPV

DGPTALIVSDGADHRRRRSVVAPGLRHHHVQRYVATMVSNIDTVIDGWQPGQRLDIYQEL

RSAVRRSTAESLFGQRLAVHSDFLGEQLQPLLDLTRRPPQVMRLQQRVNSPGWRRAMAAR

KRIDDLIDAQIADARTAPRPDDHMLTTLISGCSEEGTTLSDNEIRDSIVSLITAGYETTS

GALAWAIYALLTVPGTWESAASEVARVLGGRVPAADDLSALTYLNGVVHETLRLYSPGVI

SARRVLRDLWFDGHRIRAGRLLIFSAYVTHRLPEIWPEPTEFRPLRWDPNAADYRKPAPH

EFIPFSGGLHRCIGAVMATTEMTVILARLVARAMLQLPAQRTHRIRAANFAALRPWPGLT

VEIRKSAPAQ

>CYP139A1(2590089378)Mycobacterium tuberculosis MAL020141

MRYPLGEALLALYRWRGPLINAGVGGHGYTYLLGAEANRFVFANADAFSWSQTFESLVPV

DGPTALIVSDGADHRRRRSVVAPGLRHHHVQRYVATMVSNIDTVIDGWQPGQRLDIYQEL

RSAVRRSTAESLFGQRLAVHSDFLGEQLQPLLDLTRRPPQVMRLQQRVNSPGWRRAMAAR

KRIDDLIDAQIADARTAPRPDDHMLTTLISGCSEEGTTLSDNEIRDSIVSLITAGYETTS

GALAWAIYALLTVPGTWESAASEVARVLGGRVPAADDLSALTYLNGVVHETLRLYSPGVI

SARRVLRDLWFDGHRIRAGRLLIFSAYVTHRLPEIWPEPTEFRPLRWDPNAADYRKPAPH

EFIPFSGGLHRCIGAVMATTEMTVILARLVARAMLQLPAQRTHRIRAANFAALRPWPGLT

VEIRKSAPAQ

>CYP139A1(2590288661)Mycobacterium tuberculosis KT-0006

MRYPLGEALLALYRWRGPLINAGVGGHGYTYLLGAEANRFVFANADAFSWSQTFESLVPV

DGPTALIVSDGADHRRRRSVVAPGLRHHHVQRYVATMVSNIDTVIDGWQPGQRLDIYQEL

RSAVRRSTAESLFGQRLAVHSDFLGEQLQPLLDLTRRPPQVMRLQQRVNSPGWRRAMAAR

KRIDDLIDAQIADARTAPRPDDHMLTTLISGCSEEGTTLSDNEIRDSIVSLITAGYETTS

GALAWAIYALLTVPGTWESAASEVARVLGGRVPAADDLSALTYLNGVVHETLRLYSPGVI

SARRVLRDLWFDGHRIRAGRLLIFSAYVTHRLPEIWPEPTEFRPLRWDPNAADYRKPAPH

EFIPFSGGLHRCIGAVMATTEMTVILARLVARAMLQLPAQRTHRIRAANFAALRPWPGLT

VEIRKSAPAQ

>CYP139A1(2590513765)Mycobacterium tuberculosis KT-0094

MRYPLGEALLALYRWRGPLINAGVGGHGYTYLLGAEANRFVFANADAFSWSQTFESLVPV

DGPTALIVSDGADHRRRRSVVAPGLRHHHVQRYVATMVSNIDTVIDGWQPGQRLDIYQEL

RSAVRRSTAESLFGQRLAVHSDFLGEQLQPLLDLTRRPPQVMRLQQRVNSPGWRRAMAAR

KRIDDLIDAQIADARTAPRPDDHMLTTLISGCSEEGTTLSDNEIRDSIVSLITAGYETTS

GALAWAIYALLTVPGTWESAASEVARVLGGRVPAADDLSALTYLNGVVHETLRLYSPGVI

SARRVLRDLWFDGHRIRAGRLLIFSAYVTHRLPEIWPEPTEFRPLRWDPNAADYRKPAPH

EFIPFSGGLHRCIGAVMATTEMTVILARLVARAMLQLPAQRTHRIRAANFAALRPWPGLT

VEIRKSAPAQ

>CYP139A1(643045086)Mycobacterium tuberculosis GM 1503

MRYPLGEALLALYRWRGPLINAGVGGHGYTYLLGAEANRFVFANADAFSWSQTFESLVPV

DGPTALIVSDGADHRRRRSVVAPGLRHHHVQRYVATMVSNIDTVIDGWQPGQRLDIYQEL

RSAVRRSTAESLFGQRLAVHSDFLGEQLQPLLDLTRRPPQVMRLQQRVNSPGWRRAMAAR

KRIDDLIDAQIADARTAPRPDDHMLTTLISGCSEEGTTLSDNEIRDSIVSLITAGYETTS

GALAWAIYALLTVPGTWESAASEVARVLGGRVPAADDLSALTYLNGVVHETLRLYSPGVI

SARRVLRDLWFDGHRIRAGRLLIFSAYVTHRLPEIWPEPTEFRPLRWDPNAADYRKPAPH

EFIPFSGGLHRCIGAVMATTEMTVILARLVARAMLQLPAQRTHRIRAANFAALRPWPGLT

VEIRKSAPAQ

>CYP139A1(646014426)Mycobacterium tuberculosis CPHL_A

MRYPLGEALLALYRWRGPLINAGVGGHGYTYLLGAEANRFVFANADAFSWSQTFESLVPV

DGPTALIVSDGADHRRRRSVVAPGLRHHHVQRYVATMVSNIDTVIDGWQPGQRLDIYQEL

RSAVRRSTAESLFGQRLAVHSDFLGEQLQPLLDLTRRPPQVMRLQQRVNSPGWRRAMAAR

KRIDDLIDAQIADARTAPRPDDHMLTTLISGCSEEGTTLSDNEIRDSIVSLITAGYETTS

GALAWAIYALLTVPGTWESAASEVARVLGGRVPAADDLSALTYLNGVVHETLRLYSPGVI

SARRVLRDLWFDGHRIRAGRLLIFSAYVTHRLPEIWPEPTEFRPLRWDPNAADYRKPAPH

EFIPFSGGLHRCIGAVMATTEMTVILARLVARAMLQLPAQRTHRIRAANFAALRPWPGLT

VEIRKSAPAQ

>CYP139A1(2574985446)Mycobacterium tuberculosis TKK_05SA_0016

MRYPLGEALLALYRWRGPLINAGVGGHGYTYLLGAEANRFVFANADAFSWSQTFESLVPV

DGPTALIVSDGADHRRRRSVVAPGLRHHHVQRYVATMVSNIDTVIDGWQPGQRLDIYQEL

RSAVRRSTAESLFGQRLAVHSDFLGEQLQPLLDLTRRPPQVMRLQQRVNSPGWRRAMAAR

KRIDDLIDAQIADARTAPRPDDHMLTTLISGCSEEGTTLSDNEIRDSIVSLITAGYETTS

GALAWAIYALLTVPGTWESAASEVARVLGGRVPAADDLSALTYLNGVVHETLRLYSPGVI

SARRVLRDLWFDGHRIRAGRLLIFSAYVTHRLPEIWPEPTEFRPLRWDPNAADYRKPAPH

EFIPFSGGLHRCIGAVMATTEMTVILARLVARAMLQLPAQRTHRIRAANFAALRPWPGLT

VEIRKSAPAQ

>CYP139A1(2575103505)Mycobacterium tuberculosis TKK_03_0156

MRYPLGEALLALYRWRGPLINAGVGGHGYTYLLGAEANRFVFANADAFSWSQTFESLVPV

DGPTALIVSDGADHRRRRSVVAPGLRHHHVQRYVATMVSNIDTVIDGWQPGQRLDIYQEL

RSAVRRSTAESLFGQRLAVHSDFLGEQLQPLLDLTRRPPQVMRLQQRVNSPGWRRAMAAR

KRIDDLIDAQIADARTAPRPDDHMLTTLISGCSEEGTTLSDNEIRDSIVSLITAGYETTS

GALAWAIYALLTVPGTWESAASEVARVLGGRVPAADDLSALTYLNGVVHETLRLYSPGVI

SARRVLRDLWFDGHRIRAGRLLIFSAYVTHRLPEIWPEPTEFRPLRWDPNAADYRKPAPH

EFIPFSGGLHRCIGAVMATTEMTVILARLVARAMLQLPAQRTHRIRAANFAALRPWPGLT

VEIRKSAPAQ

>CYP139A1(2575806159)Mycobacterium tuberculosis TKK_03_0099

MRYPLGEALLALYRWRGPLINAGVGGHGYTYLLGAEANRFVFANADAFSWSQTFESLVPV

DGPTALIVSDGADHRRRRSVVAPGLRHHHVQRYVATMVSNIDTVIDGWQPGQRLDIYQEL

RSAVRRSTAESLFGQRLAVHSDFLGEQLQPLLDLTRRPPQVMRLQQRVNSPGWRRAMAAR

KRIDDLIDAQIADARTAPRPDDHMLTTLISGCSEEGTTLSDNEIRDSIVSLITAGYETTS

GALAWAIYALLTVPGTWESAASEVARVLGGRVPAADDLSALTYLNGVVHETLRLYSPGVI

SARRVLRDLWFDGHRIRAGRLLIFSAYVTHRLPEIWPEPTEFRPLRWDPNAADYRKPAPH

EFIPFSGGLHRCIGAVMATTEMTVILARLVARAMLQLPAQRTHRIRAANFAALRPWPGLT

VEIRKSAPAQ

>CYP139A1(2576156796)Mycobacterium tuberculosis TBR50

MRYPLGEALLALYRWRGPLINAGVGGHGYTYLLGAEANRFVFANADAFSWSQTFESLVPV

DGPTALIVSDGADHRRRRSVVAPGLRHHHVQRYVATMVSNIDTVIDGWQPGQRLDIYQEL

RSAVRRSTAESLFGQRLAVHSDFLGEQLQPLLDLTRRPPQVMRLQQRVNSPGWRRAMAAR

KRIDDLIDAQIADARTAPRPDDHMLTTLISGCSEEGTTLSDNEIRDSIVSLITAGYETTS

GALAWAIYALLTVPGTWESAASEVARVLGGRVPAADDLSALTYLNGVVHETLRLYSPGVI

SARRVLRDLWFDGHRIRAGRLLIFSAYVTHRLPEIWPEPTEFRPLRWDPNAADYRKPAPH

EFIPFSGGLHRCIGAVMATTEMTVILARLVARAMLQLPAQRTHRIRAANFAALRPWPGLT

VEIRKSAPAQ

>CYP139A1(2576936333)Mycobacterium tuberculosis TKK_03_0116

MRYPLGEALLALYRWRGPLINAGVGGHGYTYLLGAEANRFVFANADAFSWSQTFESLVPV

DGPTALIVSDGADHRRRRSVVAPGLRHHHVQRYVATMVSNIDTVIDGWQPGQRLDIYQEL

RSAVRRSTAESLFGQRLAVHSDFLGEQLQPLLDLTRRPPQVMRLQQRVNSPGWRRAMAAR

KRIDDLIDAQIADARTAPRPDDHMLTTLISGCSEEGTTLSDNEIRDSIVSLITAGYETTS

GALAWAIYALLTVPGTWESAASEVARVLGGRVPAADDLSALTYLNGVVHETLRLYSPGVI

SARRVLRDLWFDGHRIRAGRLLIFSAYVTHRLPEIWPEPTEFRPLRWDPNAADYRKPAPH

EFIPFSGGLHRCIGAVMATTEMTVILARLVARAMLQLPAQRTHRIRAANFAALRPWPGLT

VEIRKSAPAQ

>CYP139A1(2577030827)Mycobacterium tuberculosis TKK_05SA_0019

MRYPLGEALLALYRWRGPLINAGVGGHGYTYLLGAEANRFVFANADAFSWSQTFESLVPV

DGPTALIVSDGADHRRRRSVVAPGLRHHHVQRYVATMVSNIDTVIDGWQPGQRLDIYQEL

RSAVRRSTAESLFGQRLAVHSDFLGEQLQPLLDLTRRPPQVMRLQQRVNSPGWRRAMAAR

KRIDDLIDAQIADARTAPRPDDHMLTTLISGCSEEGTTLSDNEIRDSIVSLITAGYETTS

GALAWAIYALLTVPGTWESAASEVARVLGGRVPAADDLSALTYLNGVVHETLRLYSPGVI

SARRVLRDLWFDGHRIRAGRLLIFSAYVTHRLPEIWPEPTEFRPLRWDPNAADYRKPAPH

EFIPFSGGLHRCIGAVMATTEMTVILARLVARAMLQLPAQRTHRIRAANFAALRPWPGLT

VEIRKSAPAQ

>CYP139A1(2577381729)Mycobacterium tuberculosis TKK_05SA_0050

MRYPLGEALLALYRWRGPLINAGVGGHGYTYLLGAEANRFVFANADAFSWSQTFESLVPV

DGPTALIVSDGADHRRRRSVVAPGLRHHHVQRYVATMVSNIDTVIDGWQPGQRLDIYQEL

RSAVRRSTAESLFGQRLAVHSDFLGEQLQPLLDLTRRPPQVMRLQQRVNSPGWRRAMAAR

KRIDDLIDAQIADARTAPRPDDHMLTTLISGCSEEGTTLSDNEIRDSIVSLITAGYETTS

GALAWAIYALLTVPGTWESAASEVARVLGGRVPAADDLSALTYLNGVVHETLRLYSPGVI

SARRVLRDLWFDGHRIRAGRLLIFSAYVTHRLPEIWPEPTEFRPLRWDPNAADYRKPAPH

EFIPFSGGLHRCIGAVMATTEMTVILARLVARAMLQLPAQRTHRIRAANFAALRPWPGLT

VEIRKSAPAQ

>CYP139A1(2584698776)Mycobacterium tuberculosis XTB13-252

MRYPLGEALLALYRWRGPLINAGVGGHGYTYLLGAEANRFVFANADAFSWSQTFESLVPV

DGPTALIVSDGADHRRRRSVVAPGLRHHHVQRYVATMVSNIDTVIDGWQPGQRLDIYQEL

RSAVRRSTAESLFGQRLAVHSDFLGEQLQPLLDLTRRPPQVMRLQQRVNSPGWRRAMAAR

KRIDDLIDAQIADARTAPRPDDHMLTTLISGCSEEGTTLSDNEIRDSIVSLITAGYETTS

GALAWAIYALLTVPGTWESAASEVARVLGGRVPAADDLSALTYLNGVVHETLRLYSPGVI

SARRVLRDLWFDGHRIRAGRLLIFSAYVTHRLPEIWPEPTEFRPLRWDPNAADYRKPAPH

EFIPFSGGLHRCIGAVMATTEMTVILARLVARAMLQLPAQRTHRIRAANFAALRPWPGLT

VEIRKSAPAQ

>CYP139A1(2584825730)Mycobacterium tuberculosis TKK-01-0032

MRYPLGEALLALYRWRGPLINAGVGGHGYTYLLGAEANRFVFANADAFSWSQTFESLVPV

DGPTALIVSDGADHRRRRSVVAPGLRHHHVQRYVATMVSNIDTVIDGWQPGQRLDIYQEL

RSAVRRSTAESLFGQRLAVHSDFLGEQLQPLLDLTRRPPQVMRLQQRVNSPGWRRAMAAR

KRIDDLIDAQIADARTAPRPDDHMLTTLISGCSEEGTTLSDNEIRDSIVSLITAGYETTS

GALAWAIYALLTVPGTWESAASEVARVLGGRVPAADDLSALTYLNGVVHETLRLYSPGVI

SARRVLRDLWFDGHRIRAGRLLIFSAYVTHRLPEIWPEPTEFRPLRWDPNAADYRKPAPH

EFIPFSGGLHRCIGAVMATTEMTVILARLVARAMLQLPAQRTHRIRAANFAALRPWPGLT

VEIRKSAPAQ

>CYP139A1(2584885951)Mycobacterium tuberculosis T92

MRYPLGEALLALYRWRGPLINAGVGGHGYTYLLGAEANRFVFANADAFSWSQTFESLVPV

DGPTALIVSDGADHRRRRSVVAPGLRHHHVQRYVATMVSNIDTVIDGWQPGQRLDIYQEL

RSAVRRSTAESLFGQRLAVHSDFLGEQLQPLLDLTRRPPQVMRLQQRVNSPGWRRAMAAR

KRIDDLIDAQIADARTAPRPDDHMLTTLISGCSEEGTTLSDNEIRDSIVSLITAGYETTS

GALAWAIYALLTVPGTWESAASEVARVLGGRVPAADDLSALTYLNGVVHETLRLYSPGVI

SARRVLRDLWFDGHRIRAGRLLIFSAYVTHRLPEIWPEPTEFRPLRWDPNAADYRKPAPH

EFIPFSGGLHRCIGAVMATTEMTVILARLVARAMLQLPAQRTHRIRAANFAALRPWPGLT

VEIRKSAPAQ

>CYP139A1(2584902930)Mycobacterium tuberculosis TB_RSA62

MRYPLGEALLALYRWRGPLINAGVGGHGYTYLLGAEANRFVFANADAFSWSQTFESLVPV

DGPTALIVSDGADHRRRRSVVAPGLRHHHVQRYVATMVSNIDTVIDGWQPGQRLDIYQEL

RSAVRRSTAESLFGQRLAVHSDFLGEQLQPLLDLTRRPPQVMRLQQRVNSPGWRRAMAAR

KRIDDLIDAQIADARTAPRPDDHMLTTLISGCSEEGTTLSDNEIRDSIVSLITAGYETTS

GALAWAIYALLTVPGTWESAASEVARVLGGRVPAADDLSALTYLNGVVHETLRLYSPGVI

SARRVLRDLWFDGHRIRAGRLLIFSAYVTHRLPEIWPEPTEFRPLRWDPNAADYRKPAPH

EFIPFSGGLHRCIGAVMATTEMTVILARLVARAMLQLPAQRTHRIRAANFAALRPWPGLT

VEIRKSAPAQ

>CYP139A1(2588995218)Mycobacterium tuberculosis TKK-01-0080

MRYPLGEALLALYRWRGPLINAGVGGHGYTYLLGAEANRFVFANADAFSWSQTFESLVPV

DGPTALIVSDGADHRRRRSVVAPGLRHHHVQRYVATMVSNIDTVIDGWQPGQRLDIYQEL

RSAVRRSTAESLFGQRLAVHSDFLGEQLQPLLDLTRRPPQVMRLQQRVNSPGWRRAMAAR

KRIDDLIDAQIADARTAPRPDDHMLTTLISGCSEEGTTLSDNEIRDSIVSLITAGYETTS

GALAWAIYALLTVPGTWESAASEVARVLGGRVPAADDLSALTYLNGVVHETLRLYSPGVI

SARRVLRDLWFDGHRIRAGRLLIFSAYVTHRLPEIWPEPTEFRPLRWDPNAADYRKPAPH

EFIPFSGGLHRCIGAVMATTEMTVILARLVARAMLQLPAQRTHRIRAANFAALRPWPGLT

VEIRKSAPAQ

>CYP139A1(2589139119)Mycobacterium tuberculosis TBR76

MRYPLGEALLALYRWRGPLINAGVGGHGYTYLLGAEANRFVFANADAFSWSQTFESLVPV

DGPTALIVSDGADHRRRRSVVAPGLRHHHVQRYVATMVSNIDTVIDGWQPGQRLDIYQEL

RSAVRRSTAESLFGQRLAVHSDFLGEQLQPLLDLTRRPPQVMRLQQRVNSPGWRRAMAAR

KRIDDLIDAQIADARTAPRPDDHMLTTLISGCSEEGTTLSDNEIRDSIVSLITAGYETTS

GALAWAIYALLTVPGTWESAASEVARVLGGRVPAADDLSALTYLNGVVHETLRLYSPGVI

SARRVLRDLWFDGHRIRAGRLLIFSAYVTHRLPEIWPEPTEFRPLRWDPNAADYRKPAPH

EFIPFSGGLHRCIGAVMATTEMTVILARLVARAMLQLPAQRTHRIRAANFAALRPWPGLT

VEIRKSAPAQ

>CYP139A1(2592238712)Mycobacterium tuberculosis TKK_04_0017

MRYPLGEALLALYRWRGPLINAGVGGHGYTYLLGAEANRFVFANADAFSWSQTFESLVPV

DGPTALIVSDGADHRRRRSVVAPGLRHHHVQRYVATMVSNIDTVIDGWQPGQRLDIYQEL

RSAVRRSTAESLFGQRLAVHSDFLGEQLQPLLDLTRRPPQVMRLQQRVNSPGWRRAMAAR

KRIDDLIDAQIADARTAPRPDDHMLTTLISGCSEEGTTLSDNEIRDSIVSLITAGYETTS

GALAWAIYALLTVPGTWESAASEVARVLGGRVPAADDLSALTYLNGVVHETLRLYSPGVI

SARRVLRDLWFDGHRIRAGRLLIFSAYVTHRLPEIWPEPTEFRPLRWDPNAADYRKPAPH

EFIPFSGGLHRCIGAVMATTEMTVILARLVARAMLQLPAQRTHRIRAANFAALRPWPGLT

VEIRKSAPAQ

>CYP139A1(2592261699)Mycobacterium tuberculosis TKK_04_0006

MRYPLGEALLALYRWRGPLINAGVGGHGYTYLLGAEANRFVFANADAFSWSQTFESLVPV

DGPTALIVSDGADHRRRRSVVAPGLRHHHVQRYVATMVSNIDTVIDGWQPGQRLDIYQEL

RSAVRRSTAESLFGQRLAVHSDFLGEQLQPLLDLTRRPPQVMRLQQRVNSPGWRRAMAAR

KRIDDLIDAQIADARTAPRPDDHMLTTLISGCSEEGTTLSDNEIRDSIVSLITAGYETTS

GALAWAIYALLTVPGTWESAASEVARVLGGRVPAADDLSALTYLNGVVHETLRLYSPGVI

SARRVLRDLWFDGHRIRAGRLLIFSAYVTHRLPEIWPEPTEFRPLRWDPNAADYRKPAPH

EFIPFSGGLHRCIGAVMATTEMTVILARLVARAMLQLPAQRTHRIRAANFAALRPWPGLT

VEIRKSAPAQ

>CYP139A1(2592393538)Mycobacterium tuberculosis TKK_02_0027

MRYPLGEALLALYRWRGPLINAGVGGHGYTYLLGAEANRFVFANADAFSWSQTFESLVPV

DGPTALIVSDGADHRRRRSVVAPGLRHHHVQRYVATMVSNIDTVIDGWQPGQRLDIYQEL

RSAVRRSTAESLFGQRLAVHSDFLGEQLQPLLDLTRRPPQVMRLQQRVNSPGWRRAMAAR

KRIDDLIDAQIADARTAPRPDDHMLTTLISGCSEEGTTLSDNEIRDSIVSLITAGYETTS

GALAWAIYALLTVPGTWESAASEVARVLGGRVPAADDLSALTYLNGVVHETLRLYSPGVI

SARRVLRDLWFDGHRIRAGRLLIFSAYVTHRLPEIWPEPTEFRPLRWDPNAADYRKPAPH

EFIPFSGGLHRCIGAVMATTEMTVILARLVARAMLQLPAQRTHRIRAANFAALRPWPGLT

VEIRKSAPAQ

>CYP139A1(2592397895)Mycobacterium tuberculosis TKK_02_0025

MRYPLGEALLALYRWRGPLINAGVGGHGYTYLLGAEANRFVFANADAFSWSQTFESLVPV

DGPTALIVSDGADHRRRRSVVAPGLRHHHVQRYVATMVSNIDTVIDGWQPGQRLDIYQEL

RSAVRRSTAESLFGQRLAVHSDFLGEQLQPLLDLTRRPPQVMRLQQRVNSPGWRRAMAAR

KRIDDLIDAQIADARTAPRPDDHMLTTLISGCSEEGTTLSDNEIRDSIVSLITAGYETTS

GALAWAIYALLTVPGTWESAASEVARVLGGRVPAADDLSALTYLNGVVHETLRLYSPGVI

SARRVLRDLWFDGHRIRAGRLLIFSAYVTHRLPEIWPEPTEFRPLRWDPNAADYRKPAPH

EFIPFSGGLHRCIGAVMATTEMTVILARLVARAMLQLPAQRTHRIRAANFAALRPWPGLT

VEIRKSAPAQ

>CYP139A1(2592549349)Mycobacterium tuberculosis TKK_04_0043

MRYPLGEALLALYRWRGPLINAGVGGHGYTYLLGAEANRFVFANADAFSWSQTFESLVPV

DGPTALIVSDGADHRRRRSVVAPGLRHHHVQRYVATMVSNIDTVIDGWQPGQRLDIYQEL

RSAVRRSTAESLFGQRLAVHSDFLGEQLQPLLDLTRRPPQVMRLQQRVNSPGWRRAMAAR

KRIDDLIDAQIADARTAPRPDDHMLTTLISGCSEEGTTLSDNEIRDSIVSLITAGYETTS

GALAWAIYALLTVPGTWESAASEVARVLGGRVPAADDLSALTYLNGVVHETLRLYSPGVI

SARRVLRDLWFDGHRIRAGRLLIFSAYVTHRLPEIWPEPTEFRPLRWDPNAADYRKPAPH

EFIPFSGGLHRCIGAVMATTEMTVILARLVARAMLQLPAQRTHRIRAANFAALRPWPGLT

VEIRKSAPAQ

>CYP139A1(648464907)Mycobacterium tuberculosis SUMu006

MRYPLGEALLALYRWRGPLINAGVGGHGYTYLLGAEANRFVFANADAFSWSQTFESLVPV

DGPTALIVSDGADHRRRRSVVAPGLRHHHVQRYVATMVSNIDTVIDGWQPGQRLDIYQEL

RSAVRRSTAESLFGQRLAVHSDFLGEQLQPLLDLTRRPPQVMRLQQRVNSPGWRRAMAAR

KRIDDLIDAQIADARTAPRPDDHMLTTLISGCSEEGTTLSDNEIRDSIVSLITAGYETTS

GALAWAIYALLTVPGTWESAASEVARVLGGRVPAADDLSALTYLNGVVHETLRLYSPGVI

SARRVLRDLWFDGHRIRAGRLLIFSAYVTHRLPEIWPEPTEFRPLRWDPNAADYRKPAPH

EFIPFSGGLHRCIGAVMATTEMTVILARLVARAMLQLPAQRTHRIRAANFAALRPWPGLT

VEIRKSAPAQ

>CYP139A1(648481186)Mycobacterium tuberculosis SUMu010

MRYPLGEALLALYRWRGPLINAGVGGHGYTYLLGAEANRFVFANADAFSWSQTFESLVPV

DGPTALIVSDGADHRRRRSVVAPGLRHHHVQRYVATMVSNIDTVIDGWQPGQRLDIYQEL

RSAVRRSTAESLFGQRLAVHSDFLGEQLQPLLDLTRRPPQVMRLQQRVNSPGWRRAMAAR

KRIDDLIDAQIADARTAPRPDDHMLTTLISGCSEEGTTLSDNEIRDSIVSLITAGYETTS

GALAWAIYALLTVPGTWESAASEVARVLGGRVPAADDLSALTYLNGVVHETLRLYSPGVI

SARRVLRDLWFDGHRIRAGRLLIFSAYVTHRLPEIWPEPTEFRPLRWDPNAADYRKPAPH

EFIPFSGGLHRCIGAVMATTEMTVILARLVARAMLQLPAQRTHRIRAANFAALRPWPGLT

VEIRKSAPAQ

>CYP139A1(2574586310)Mycobacterium tuberculosis MD16553

MRYPLGEALLALYRWRGPLINAGVGGHGYTYLLGAEANRFVFANADAFSWSQTFESLVPV

DGPTALIVSDGADHRRRRSVVAPGLRHHHVQRYVATMVSNIDTVIDGWQPGQRLDIYQEL

RSAVRRSTAESLFGQRLAVHSDFLGEQLQPLLDLTRRPPQVMRLQQRVNSPGWRRAMAAR

KRIDDLIDAQIADARTAPRPDDHMLTTLISGCSEEGTTLSDNEIRDSIVSLITAGYETTS

GALAWAIYALLTVPGTWESAASEVARVLGGRVPAADDLSALTYLNGVVHETLRLYSPGVI

SARRVLRDLWFDGHRIRAGRLLIFSAYVTHRLPEIWPEPTEFRPLRWDPNAADYRKPAPH

EFIPFSGGLHRCIGAVMATTEMTVILARLVARAMLQLPAQRTHRIRAANFAALRPWPGLT

VEIRKSAPAQ

>CYP139A1(2574663269)Mycobacterium tuberculosis M1438

MRYPLGEALLALYRWRGPLINAGVGGHGYTYLLGAEANRFVFANADAFSWSQTFESLVPV

DGPTALIVSDGADHRRRRSVVAPGLRHHHVQRYVATMVSNIDTVIDGWQPGQRLDIYQEL

RSAVRRSTAESLFGQRLAVHSDFLGEQLQPLLDLTRRPPQVMRLQQRVNSPGWRRAMAAR

KRIDDLIDAQIADARTAPRPDDHMLTTLISGCSEEGTTLSDNEIRDSIVSLITAGYETTS

GALAWAIYALLTVPGTWESAASEVARVLGGRVPAADDLSALTYLNGVVHETLRLYSPGVI

SARRVLRDLWFDGHRIRAGRLLIFSAYVTHRLPEIWPEPTEFRPLRWDPNAADYRKPAPH

EFIPFSGGLHRCIGAVMATTEMTVILARLVARAMLQLPAQRTHRIRAANFAALRPWPGLT

VEIRKSAPAQ

>CYP139A1(2574880930)Mycobacterium tuberculosis BTB13-128

MRYPLGEALLALYRWRGPLINAGVGGHGYTYLLGAEANRFVFANADAFSWSQTFESLVPV

DGPTALIVSDGADHRRRRSVVAPGLRHHHVQRYVATMVSNIDTVIDGWQPGQRLDIYQEL

RSAVRRSTAESLFGQRLAVHSDFLGEQLQPLLDLTRRPPQVMRLQQRVNSPGWRRAMAAR

KRIDDLIDAQIADARTAPRPDDHMLTTLISGCSEEGTTLSDNEIRDSIVSLITAGYETTS

GALAWAIYALLTVPGTWESAASEVARVLGGRVPAADDLSALTYLNGVVHETLRLYSPGVI

SARRVLRDLWFDGHRIRAGRLLIFSAYVTHRLPEIWPEPTEFRPLRWDPNAADYRKPAPH

EFIPFSGGLHRCIGAVMATTEMTVILARLVARAMLQLPAQRTHRIRAANFAALRPWPGLT

VEIRKSAPAQ

>CYP139A1(2574911407)Mycobacterium tuberculosis KT-0069

MRYPLGEALLALYRWRGPLINAGVGGHGYTYLLGAEANRFVFANADAFSWSQTFESLVPV

DGPTALIVSDGADHRRRRSVVAPGLRHHHVQRYVATMVSNIDTVIDGWQPGQRLDIYQEL

RSAVRRSTAESLFGQRLAVHSDFLGEQLQPLLDLTRRPPQVMRLQQRVNSPGWRRAMAAR

KRIDDLIDAQIADARTAPRPDDHMLTTLISGCSEEGTTLSDNEIRDSIVSLITAGYETTS

GALAWAIYALLTVPGTWESAASEVARVLGGRVPAADDLSALTYLNGVVHETLRLYSPGVI

SARRVLRDLWFDGHRIRAGRLLIFSAYVTHRLPEIWPEPTEFRPLRWDPNAADYRKPAPH

EFIPFSGGLHRCIGAVMATTEMTVILARLVARAMLQLPAQRTHRIRAANFAALRPWPGLT

VEIRKSAPAQ

>CYP139A1(2575345775)Mycobacterium tuberculosis M2343

MRYPLGEALLALYRWRGPLINAGVGGHGYTYLLGAEANRFVFANADAFSWSQTFESLVPV

DGPTALIVSDGADHRRRRSVVAPGLRHHHVQRYVATMVSNIDTVIDGWQPGQRLDIYQEL

RSAVRRSTAESLFGQRLAVHSDFLGEQLQPLLDLTRRPPQVMRLQQRVNSPGWRRAMAAR

KRIDDLIDAQIADARTAPRPDDHMLTTLISGCSEEGTTLSDNEIRDSIVSLITAGYETTS

GALAWAIYALLTVPGTWESAASEVARVLGGRVPAADDLSALTYLNGVVHETLRLYSPGVI

SARRVLRDLWFDGHRIRAGRLLIFSAYVTHRLPEIWPEPTEFRPLRWDPNAADYRKPAPH

EFIPFSGGLHRCIGAVMATTEMTVILARLVARAMLQLPAQRTHRIRAANFAALRPWPGLT

VEIRKSAPAQ

>CYP139A1(2575468742)Mycobacterium tuberculosis KT-0091

MRYPLGEALLALYRWRGPLINAGVGGHGYTYLLGAEANRFVFANADAFSWSQTFESLVPV

DGPTALIVSDGADHRRRRSVVAPGLRHHHVQRYVATMVSNIDTVIDGWQPGQRLDIYQEL

RSAVRRSTAESLFGQRLAVHSDFLGEQLQPLLDLTRRPPQVMRLQQRVNSPGWRRAMAAR

KRIDDLIDAQIADARTAPRPDDHMLTTLISGCSEEGTTLSDNEIRDSIVSLITAGYETTS

GALAWAIYALLTVPGTWESAASEVARVLGGRVPAADDLSALTYLNGVVHETLRLYSPGVI

SARRVLRDLWFDGHRIRAGRLLIFSAYVTHRLPEIWPEPTEFRPLRWDPNAADYRKPAPH

EFIPFSGGLHRCIGAVMATTEMTVILARLVARAMLQLPAQRTHRIRAANFAALRPWPGLT

VEIRKSAPAQ

>CYP139A1(2576199760)Mycobacterium tuberculosis KT-0003

MRYPLGEALLALYRWRGPLINAGVGGHGYTYLLGAEANRFVFANADAFSWSQTFESLVPV

DGPTALIVSDGADHRRRRSVVAPGLRHHHVQRYVATMVSNIDTVIDGWQPGQRLDIYQEL

RSAVRRSTAESLFGQRLAVHSDFLGEQLQPLLDLTRRPPQVMRLQQRVNSPGWRRAMAAR

KRIDDLIDAQIADARTAPRPDDHMLTTLISGCSEEGTTLSDNEIRDSIVSLITAGYETTS

GALAWAIYALLTVPGTWESAASEVARVLGGRVPAADDLSALTYLNGVVHETLRLYSPGVI

SARRVLRDLWFDGHRIRAGRLLIFSAYVTHRLPEIWPEPTEFRPLRWDPNAADYRKPAPH

EFIPFSGGLHRCIGAVMATTEMTVILARLVARAMLQLPAQRTHRIRAANFAALRPWPGLT

VEIRKSAPAQ

>CYP139A1(2577069926)Mycobacterium tuberculosis M2113

MRYPLGEALLALYRWRGPLINAGVGGHGYTYLLGAEANRFVFANADAFSWSQTFESLVPV

DGPTALIVSDGADHRRRRSVVAPGLRHHHVQRYVATMVSNIDTVIDGWQPGQRLDIYQEL

RSAVRRSTAESLFGQRLAVHSDFLGEQLQPLLDLTRRPPQVMRLQQRVNSPGWRRAMAAR

KRIDDLIDAQIADARTAPRPDDHMLTTLISGCSEEGTTLSDNEIRDSIVSLITAGYETTS

GALAWAIYALLTVPGTWESAASEVARVLGGRVPAADDLSALTYLNGVVHETLRLYSPGVI

SARRVLRDLWFDGHRIRAGRLLIFSAYVTHRLPEIWPEPTEFRPLRWDPNAADYRKPAPH

EFIPFSGGLHRCIGAVMATTEMTVILARLVARAMLQLPAQRTHRIRAANFAALRPWPGLT

VEIRKSAPAQ

>CYP139A1(2577613318)Mycobacterium tuberculosis M2128

MRYPLGEALLALYRWRGPLINAGVGGHGYTYLLGAEANRFVFANADAFSWSQTFESLVPV

DGPTALIVSDGADHRRRRSVVAPGLRHHHVQRYVATMVSNIDTVIDGWQPGQRLDIYQEL

RSAVRRSTAESLFGQRLAVHSDFLGEQLQPLLDLTRRPPQVMRLQQRVNSPGWRRAMAAR

KRIDDLIDAQIADARTAPRPDDHMLTTLISGCSEEGTTLSDNEIRDSIVSLITAGYETTS

GALAWAIYALLTVPGTWESAASEVARVLGGRVPAADDLSALTYLNGVVHETLRLYSPGVI

SARRVLRDLWFDGHRIRAGRLLIFSAYVTHRLPEIWPEPTEFRPLRWDPNAADYRKPAPH

EFIPFSGGLHRCIGAVMATTEMTVILARLVARAMLQLPAQRTHRIRAANFAALRPWPGLT

VEIRKSAPAQ

>CYP139A1(2577684900)Mycobacterium tuberculosis H2398

MRYPLGEALLALYRWRGPLINAGVGGHGYTYLLGAEANRFVFANADAFSWSQTFESLVPV

DGPTALIVSDGADHRRRRSVVAPGLRHHHVQRYVATMVSNIDTVIDGWQPGQRLDIYQEL

RSAVRRSTAESLFGQRLAVHSDFLGEQLQPLLDLTRRPPQVMRLQQRVNSPGWRRAMAAR

KRIDDLIDAQIADARTAPRPDDHMLTTLISGCSEEGTTLSDNEIRDSIVSLITAGYETTS

GALAWAIYALLTVPGTWESAASEVARVLGGRVPAADDLSALTYLNGVVHETLRLYSPGVI

SARRVLRDLWFDGHRIRAGRLLIFSAYVTHRLPEIWPEPTEFRPLRWDPNAADYRKPAPH

EFIPFSGGLHRCIGAVMATTEMTVILARLVARAMLQLPAQRTHRIRAANFAALRPWPGLT

VEIRKSAPAQ

>CYP139A1(2582415442)*Mycobacterium africanum* MAL010074

MRYPLGEALLALYRWRGPLINAGVGGHGYTYLLGAEANRFVFANADAFSWSQTFESLVPV

DGPTALIVSDGADHRRRRSVVAPGLRHHHVQRYVATMVSNIDTVIDGWQPGQRLDIYQEL

RSAVRRSTAESLFGQRLAVHSDFLGEQLQPLLDLTRRPPQVMRLQQRVNSPGWRRAMAAR

KRIDDLIDAQIADARTAPRPDDHMLTTLISGCSEEGTTLSDNEIRDSIVSLITAGYETTS

GALAWAIYALLTVPGTWESAASEVARVLGGRVPAADDLSALTYLNGVVHETLRLYSPGVI

SARRVLRDLWFDGHRIRAGRLLIFSAYVTHRLPEIWPEPTEFRPLRWDPNAADYRKPAPH

EFIPFSGGLHRCIGAVMATTEMTVILARLVARAMLQLPAQRTHRIRAANFAALRPWPGLT

VEIRKSAPAQ

>CYP139A1(2584812089)Mycobacterium tuberculosis MD17615

MRYPLGEALLALYRWRGPLINAGVGGHGYTYLLGAEANRFVFANADAFSWSQTFESLVPV

DGPTALIVSDGADHRRRRSVVAPGLRHHHVQRYVATMVSNIDTVIDGWQPGQRLDIYQEL

RSAVRRSTAESLFGQRLAVHSDFLGEQLQPLLDLTRRPPQVMRLQQRVNSPGWRRAMAAR

KRIDDLIDAQIADARTAPRPDDHMLTTLISGCSEEGTTLSDNEIRDSIVSLITAGYETTS

GALAWAIYALLTVPGTWESAASEVARVLGGRVPAADDLSALTYLNGVVHETLRLYSPGVI

SARRVLRDLWFDGHRIRAGRLLIFSAYVTHRLPEIWPEPTEFRPLRWDPNAADYRKPAPH

EFIPFSGGLHRCIGAVMATTEMTVILARLVARAMLQLPAQRTHRIRAANFAALRPWPGLT

VEIRKSAPAQ

>CYP139A1(2590125975)Mycobacterium tuberculosis MAL020172

MRYPLGEALLALYRWRGPLINAGVGGHGYTYLLGAEANRFVFANADAFSWSQTFESLVPV

DGPTALIVSDGADHRRRRSVVAPGLRHHHVQRYVATMVSNIDTVIDGWQPGQRLDIYQEL

RSAVRRSTAESLFGQRLAVHSDFLGEQLQPLLDLTRRPPQVMRLQQRVNSPGWRRAMAAR

KRIDDLIDAQIADARTAPRPDDHMLTTLISGCSEEGTTLSDNEIRDSIVSLITAGYETTS

GALAWAIYALLTVPGTWESAASEVARVLGGRVPAADDLSALTYLNGVVHETLRLYSPGVI

SARRVLRDLWFDGHRIRAGRLLIFSAYVTHRLPEIWPEPTEFRPLRWDPNAADYRKPAPH

EFIPFSGGLHRCIGAVMATTEMTVILARLVARAMLQLPAQRTHRIRAANFAALRPWPGLT

VEIRKSAPAQ

>CYP139A1(2590219901)Mycobacterium tuberculosis KT-0053

MRYPLGEALLALYRWRGPLINAGVGGHGYTYLLGAEANRFVFANADAFSWSQTFESLVPV

DGPTALIVSDGADHRRRRSVVAPGLRHHHVQRYVATMVSNIDTVIDGWQPGQRLDIYQEL

RSAVRRSTAESLFGQRLAVHSDFLGEQLQPLLDLTRRPPQVMRLQQRVNSPGWRRAMAAR

KRIDDLIDAQIADARTAPRPDDHMLTTLISGCSEEGTTLSDNEIRDSIVSLITAGYETTS

GALAWAIYALLTVPGTWESAASEVARVLGGRVPAADDLSALTYLNGVVHETLRLYSPGVI

SARRVLRDLWFDGHRIRAGRLLIFSAYVTHRLPEIWPEPTEFRPLRWDPNAADYRKPAPH

EFIPFSGGLHRCIGAVMATTEMTVILARLVARAMLQLPAQRTHRIRAANFAALRPWPGLT

VEIRKSAPAQ

>CYP139A1(2590227570)Mycobacterium tuberculosis KT-0047

MRYPLGEALLALYRWRGPLINAGVGGHGYTYLLGAEANRFVFANADAFSWSQTFESLVPV

DGPTALIVSDGADHRRRRSVVAPGLRHHHVQRYVATMVSNIDTVIDGWQPGQRLDIYQEL

RSAVRRSTAESLFGQRLAVHSDFLGEQLQPLLDLTRRPPQVMRLQQRVNSPGWRRAMAAR

KRIDDLIDAQIADARTAPRPDDHMLTTLISGCSEEGTTLSDNEIRDSIVSLITAGYETTS

GALAWAIYALLTVPGTWESAASEVARVLGGRVPAADDLSALTYLNGVVHETLRLYSPGVI

SARRVLRDLWFDGHRIRAGRLLIFSAYVTHRLPEIWPEPTEFRPLRWDPNAADYRKPAPH

EFIPFSGGLHRCIGAVMATTEMTVILARLVARAMLQLPAQRTHRIRAANFAALRPWPGLT

VEIRKSAPAQ

>CYP139A1(2590317674)Mycobacterium tuberculosis Erdman ATCC 35801

MRYPLGEALLALYRWRGPLINAGVGGHGYTYLLGAEANRFVFANADAFSWSQTFESLVPV

DGPTALIVSDGADHRRRRSVVAPGLRHHHVQRYVATMVSNIDTVIDGWQPGQRLDIYQEL

RSAVRRSTAESLFGQRLAVHSDFLGEQLQPLLDLTRRPPQVMRLQQRVNSPGWRRAMAAR

KRIDDLIDAQIADARTAPRPDDHMLTTLISGCSEEGTTLSDNEIRDSIVSLITAGYETTS

GALAWAIYALLTVPGTWESAASEVARVLGGRVPAADDLSALTYLNGVVHETLRLYSPGVI

SARRVLRDLWFDGHRIRAGRLLIFSAYVTHRLPEIWPEPTEFRPLRWDPNAADYRKPAPH

EFIPFSGGLHRCIGAVMATTEMTVILARLVARAMLQLPAQRTHRIRAANFAALRPWPGLT

VEIRKSAPAQ

>CYP139A1(641814886)Mycobacterium tuberculosis H37Ra

MRYPLGEALLALYRWRGPLINAGVGGHGYTYLLGAEANRFVFANADAFSWSQTFESLVPV

DGPTALIVSDGADHRRRRSVVAPGLRHHHVQRYVATMVSNIDTVIDGWQPGQRLDIYQEL

RSAVRRSTAESLFGQRLAVHSDFLGEQLQPLLDLTRRPPQVMRLQQRVNSPGWRRAMAAR

KRIDDLIDAQIADARTAPRPDDHMLTTLISGCSEEGTTLSDNEIRDSIVSLITAGYETTS

GALAWAIYALLTVPGTWESAASEVARVLGGRVPAADDLSALTYLNGVVHETLRLYSPGVI

SARRVLRDLWFDGHRIRAGRLLIFSAYVTHRLPEIWPEPTEFRPLRWDPNAADYRKPAPH

EFIPFSGGLHRCIGAVMATTEMTVILARLVARAMLQLPAQRTHRIRAANFAALRPWPGLT

VEIRKSAPAQ

>CYP139A1(2574738070)Mycobacterium tuberculosis TB_RSA96

MRYPLGEALLALYRWRGPLINAGVGGHGYTYLLGAEANRFVFANADAFSWSQTFESLVPV

DGPTALIVSDGADHRRRRSVVAPGLRHHHVQRYVATMVSNIDTVIDGWQPGQRLDIYQEL

RSAVRRSTAESLFGQRLAVHSDFLGEQLQPLLDLTRRPPQVMRLQQRVNSPGWRRAMAAR

KRIDDLIDAQIADARTAPRPDDHMLTTLISGCSEEGTTLSDNEIRDSIVSLITAGYETTS

GALAWAIYALLTVPGTWESAASEVARVLGGRVPAADDLSALTYLNGVVHETLRLYSPGVI

SARRVLRDLWFDGHRIRAGRLLIFSAYVTHRLPEIWPEPTEFRPLRWDPNAADYRKPAPH

EFIPFSGGLHRCIGAVMATTEMTVILARLVARAMLQLPAQRTHRIRAANFAALRPWPGLT

VEIRKSAPAQ

>CYP139A1(2575365848)Mycobacterium tuberculosis XTB13-127

MRYPLGEALLALYRWRGPLINAGVGGHGYTYLLGAEANRFVFANADAFSWSQTFESLVPV

DGPTALIVSDGADHRRRRSVVAPGLRHHHVQRYVATMVSNIDTVIDGWQPGQRLDIYQEL

RSAVRRSTAESLFGQRLAVHSDFLGEQLQPLLDLTRRPPQVMRLQQRVNSPGWRRAMAAR

KRIDDLIDAQIADARTAPRPDDHMLTTLISGCSEEGTTLSDNEIRDSIVSLITAGYETTS

GALAWAIYALLTVPGTWESAASEVARVLGGRVPAADDLSALTYLNGVVHETLRLYSPGVI

SARRVLRDLWFDGHRIRAGRLLIFSAYVTHRLPEIWPEPTEFRPLRWDPNAADYRKPAPH

EFIPFSGGLHRCIGAVMATTEMTVILARLVARAMLQLPAQRTHRIRAANFAALRPWPGLT

VEIRKSAPAQ

>CYP139A1(2575674521)Mycobacterium tuberculosis TKK_05MA_0025

MRYPLGEALLALYRWRGPLINAGVGGHGYTYLLGAEANRFVFANADAFSWSQTFESLVPV

DGPTALIVSDGADHRRRRSVVAPGLRHHHVQRYVATMVSNIDTVIDGWQPGQRLDIYQEL

RSAVRRSTAESLFGQRLAVHSDFLGEQLQPLLDLTRRPPQVMRLQQRVNSPGWRRAMAAR

KRIDDLIDAQIADARTAPRPDDHMLTTLISGCSEEGTTLSDNEIRDSIVSLITAGYETTS

GALAWAIYALLTVPGTWESAASEVARVLGGRVPAADDLSALTYLNGVVHETLRLYSPGVI

SARRVLRDLWFDGHRIRAGRLLIFSAYVTHRLPEIWPEPTEFRPLRWDPNAADYRKPAPH

EFIPFSGGLHRCIGAVMATTEMTVILARLVARAMLQLPAQRTHRIRAANFAALRPWPGLT

VEIRKSAPAQ

>CYP139A1(2575772716)Mycobacterium tuberculosis TB_RSA15

MRYPLGEALLALYRWRGPLINAGVGGHGYTYLLGAEANRFVFANADAFSWSQTFESLVPV

DGPTALIVSDGADHRRRRSVVAPGLRHHHVQRYVATMVSNIDTVIDGWQPGQRLDIYQEL

RSAVRRSTAESLFGQRLAVHSDFLGEQLQPLLDLTRRPPQVMRLQQRVNSPGWRRAMAAR

KRIDDLIDAQIADARTAPRPDDHMLTTLISGCSEEGTTLSDNEIRDSIVSLITAGYETTS

GALAWAIYALLTVPGTWESAASEVARVLGGRVPAADDLSALTYLNGVVHETLRLYSPGVI

SARRVLRDLWFDGHRIRAGRLLIFSAYVTHRLPEIWPEPTEFRPLRWDPNAADYRKPAPH

EFIPFSGGLHRCIGAVMATTEMTVILARLVARAMLQLPAQRTHRIRAANFAALRPWPGLT

VEIRKSAPAQ

>CYP139A1(2576544659)Mycobacterium tuberculosis XTB13-241

MRYPLGEALLALYRWRGPLINAGVGGHGYTYLLGAEANRFVFANADAFSWSQTFESLVPV

DGPTALIVSDGADHRRRRSVVAPGLRHHHVQRYVATMVSNIDTVIDGWQPGQRLDIYQEL

RSAVRRSTAESLFGQRLAVHSDFLGEQLQPLLDLTRRPPQVMRLQQRVNSPGWRRAMAAR

KRIDDLIDAQIADARTAPRPDDHMLTTLISGCSEEGTTLSDNEIRDSIVSLITAGYETTS

GALAWAIYALLTVPGTWESAASEVARVLGGRVPAADDLSALTYLNGVVHETLRLYSPGVI

SARRVLRDLWFDGHRIRAGRLLIFSAYVTHRLPEIWPEPTEFRPLRWDPNAADYRKPAPH

EFIPFSGGLHRCIGAVMATTEMTVILARLVARAMLQLPAQRTHRIRAANFAALRPWPGLT

VEIRKSAPAQ

>CYP139A1(2577168470)Mycobacterium tuberculosis TKK_03_0024

MRYPLGEALLALYRWRGPLINAGVGGHGYTYLLGAEANRFVFANADAFSWSQTFESLVPV

DGPTALIVSDGADHRRRRSVVAPGLRHHHVQRYVATMVSNIDTVIDGWQPGQRLDIYQEL

RSAVRRSTAESLFGQRLAVHSDFLGEQLQPLLDLTRRPPQVMRLQQRVNSPGWRRAMAAR

KRIDDLIDAQIADARTAPRPDDHMLTTLISGCSEEGTTLSDNEIRDSIVSLITAGYETTS

GALAWAIYALLTVPGTWESAASEVARVLGGRVPAADDLSALTYLNGVVHETLRLYSPGVI

SARRVLRDLWFDGHRIRAGRLLIFSAYVTHRLPEIWPEPTEFRPLRWDPNAADYRKPAPH

EFIPFSGGLHRCIGAVMATTEMTVILARLVARAMLQLPAQRTHRIRAANFAALRPWPGLT

VEIRKSAPAQ

>CYP139A1(2577390930)Mycobacterium tuberculosis XTB13-167

MRYPLGEALLALYRWRGPLINAGVGGHGYTYLLGAEANRFVFANADAFSWSQTFESLVPV

DGPTALIVSDGADHRRRRSVVAPGLRHHHVQRYVATMVSNIDTVIDGWQPGQRLDIYQEL

RSAVRRSTAESLFGQRLAVHSDFLGEQLQPLLDLTRRPPQVMRLQQRVNSPGWRRAMAAR

KRIDDLIDAQIADARTAPRPDDHMLTTLISGCSEEGTTLSDNEIRDSIVSLITAGYETTS

GALAWAIYALLTVPGTWESAASEVARVLGGRVPAADDLSALTYLNGVVHETLRLYSPGVI

SARRVLRDLWFDGHRIRAGRLLIFSAYVTHRLPEIWPEPTEFRPLRWDPNAADYRKPAPH

EFIPFSGGLHRCIGAVMATTEMTVILARLVARAMLQLPAQRTHRIRAANFAALRPWPGLT

VEIRKSAPAQ

>CYP139A1(2577651582)Mycobacterium tuberculosis TKK_02_0038

MRYPLGEALLALYRWRGPLINAGVGGHGYTYLLGAEANRFVFANADAFSWSQTFESLVPV

DGPTALIVSDGADHRRRRSVVAPGLRHHHVQRYVATMVSNIDTVIDGWQPGQRLDIYQEL

RSAVRRSTAESLFGQRLAVHSDFLGEQLQPLLDLTRRPPQVMRLQQRVNSPGWRRAMAAR

KRIDDLIDAQIADARTAPRPDDHMLTTLISGCSEEGTTLSDNEIRDSIVSLITAGYETTS

GALAWAIYALLTVPGTWESAASEVARVLGGRVPAADDLSALTYLNGVVHETLRLYSPGVI

SARRVLRDLWFDGHRIRAGRLLIFSAYVTHRLPEIWPEPTEFRPLRWDPNAADYRKPAPH

EFIPFSGGLHRCIGAVMATTEMTVILARLVARAMLQLPAQRTHRIRAANFAALRPWPGLT

VEIRKSAPAQ

>CYP139A1(2578002537)Mycobacterium tuberculosis TB_RSA123

MRYPLGEALLALYRWRGPLINAGVGGHGYTYLLGAEANRFVFANADAFSWSQTFESLVPV

DGPTALIVSDGADHRRRRSVVAPGLRHHHVQRYVATMVSNIDTVIDGWQPGQRLDIYQEL

RSAVRRSTAESLFGQRLAVHSDFLGEQLQPLLDLTRRPPQVMRLQQRVNSPGWRRAMAAR

KRIDDLIDAQIADARTAPRPDDHMLTTLISGCSEEGTTLSDNEIRDSIVSLITAGYETTS

GALAWAIYALLTVPGTWESAASEVARVLGGRVPAADDLSALTYLNGVVHETLRLYSPGVI

SARRVLRDLWFDGHRIRAGRLLIFSAYVTHRLPEIWPEPTEFRPLRWDPNAADYRKPAPH

EFIPFSGGLHRCIGAVMATTEMTVILARLVARAMLQLPAQRTHRIRAANFAALRPWPGLT

VEIRKSAPAQ

>CYP139A1(2578230674)Mycobacterium tuberculosis OFXR-10

MRYPLGEALLALYRWRGPLINAGVGGHGYTYLLGAEANRFVFANADAFSWSQTFESLVPV

DGPTALIVSDGADHRRRRSVVAPGLRHHHVQRYVATMVSNIDTVIDGWQPGQRLDIYQEL

RSAVRRSTAESLFGQRLAVHSDFLGEQLQPLLDLTRRPPQVMRLQQRVNSPGWRRAMAAR

KRIDDLIDAQIADARTAPRPDDHMLTTLISGCSEEGTTLSDNEIRDSIVSLITAGYETTS

GALAWAIYALLTVPGTWESAASEVARVLGGRVPAADDLSALTYLNGVVHETLRLYSPGVI

SARRVLRDLWFDGHRIRAGRLLIFSAYVTHRLPEIWPEPTEFRPLRWDPNAADYRKPAPH

EFIPFSGGLHRCIGAVMATTEMTVILARLVARAMLQLPAQRTHRIRAANFAALRPWPGLT

VEIRKSAPAQ

>CYP139A1(2579825786)Mycobacterium tuberculosis TKK-01-0035

MRYPLGEALLALYRWRGPLINAGVGGHGYTYLLGAEANRFVFANADAFSWSQTFESLVPV

DGPTALIVSDGADHRRRRSVVAPGLRHHHVQRYVATMVSNIDTVIDGWQPGQRLDIYQEL

RSAVRRSTAESLFGQRLAVHSDFLGEQLQPLLDLTRRPPQVMRLQQRVNSPGWRRAMAAR

KRIDDLIDAQIADARTAPRPDDHMLTTLISGCSEEGTTLSDNEIRDSIVSLITAGYETTS

GALAWAIYALLTVPGTWESAASEVARVLGGRVPAADDLSALTYLNGVVHETLRLYSPGVI

SARRVLRDLWFDGHRIRAGRLLIFSAYVTHRLPEIWPEPTEFRPLRWDPNAADYRKPAPH

EFIPFSGGLHRCIGAVMATTEMTVILARLVARAMLQLPAQRTHRIRAANFAALRPWPGLT

VEIRKSAPAQ

>CYP139A1(2584923296)Mycobacterium tuberculosis TB_RSA79

MRYPLGEALLALYRWRGPLINAGVGGHGYTYLLGAEANRFVFANADAFSWSQTFESLVPV

DGPTALIVSDGADHRRRRSVVAPGLRHHHVQRYVATMVSNIDTVIDGWQPGQRLDIYQEL

RSAVRRSTAESLFGQRLAVHSDFLGEQLQPLLDLTRRPPQVMRLQQRVNSPGWRRAMAAR

KRIDDLIDAQIADARTAPRPDDHMLTTLISGCSEEGTTLSDNEIRDSIVSLITAGYETTS

GALAWAIYALLTVPGTWESAASEVARVLGGRVPAADDLSALTYLNGVVHETLRLYSPGVI

SARRVLRDLWFDGHRIRAGRLLIFSAYVTHRLPEIWPEPTEFRPLRWDPNAADYRKPAPH

EFIPFSGGLHRCIGAVMATTEMTVILARLVARAMLQLPAQRTHRIRAANFAALRPWPGLT

VEIRKSAPAQ

>CYP139A1(2584954771)Mycobacterium tuberculosis TRUG0095

MRYPLGEALLALYRWRGPLINAGVGGHGYTYLLGAEANRFVFANADAFSWSQTFESLVPV

DGPTALIVSDGADHRRRRSVVAPGLRHHHVQRYVATMVSNIDTVIDGWQPGQRLDIYQEL

RSAVRRSTAESLFGQRLAVHSDFLGEQLQPLLDLTRRPPQVMRLQQRVNSPGWRRAMAAR

KRIDDLIDAQIADARTAPRPDDHMLTTLISGCSEEGTTLSDNEIRDSIVSLITAGYETTS

GALAWAIYALLTVPGTWESAASEVARVLGGRVPAADDLSALTYLNGVVHETLRLYSPGVI

SARRVLRDLWFDGHRIRAGRLLIFSAYVTHRLPEIWPEPTEFRPLRWDPNAADYRKPAPH

EFIPFSGGLHRCIGAVMATTEMTVILARLVARAMLQLPAQRTHRIRAANFAALRPWPGLT

VEIRKSAPAQ

>CYP139A1(2588999278)Mycobacterium tuberculosis TKK-01-0072

MRYPLGEALLALYRWRGPLINAGVGGHGYTYLLGAEANRFVFANADAFSWSQTFESLVPV

DGPTALIVSDGADHRRRRSVVAPGLRHHHVQRYVATMVSNIDTVIDGWQPGQRLDIYQEL

RSAVRRSTAESLFGQRLAVHSDFLGEQLQPLLDLTRRPPQVMRLQQRVNSPGWRRAMAAR

KRIDDLIDAQIADARTAPRPDDHMLTTLISGCSEEGTTLSDNEIRDSIVSLITAGYETTS

GALAWAIYALLTVPGTWESAASEVARVLGGRVPAADDLSALTYLNGVVHETLRLYSPGVI

SARRVLRDLWFDGHRIRAGRLLIFSAYVTHRLPEIWPEPTEFRPLRWDPNAADYRKPAPH

EFIPFSGGLHRCIGAVMATTEMTVILARLVARAMLQLPAQRTHRIRAANFAALRPWPGLT

VEIRKSAPAQ

>CYP139A1(2589044237)Mycobacterium tuberculosis TBR9

MRYPLGEALLALYRWRGPLINAGVGGHGYTYLLGAEANRFVFANADAFSWSQTFESLVPV

DGPTALIVSDGADHRRRRSVVAPGLRHHHVQRYVATMVSNIDTVIDGWQPGQRLDIYQEL

RSAVRRSTAESLFGQRLAVHSDFLGEQLQPLLDLTRRPPQVMRLQQRVNSPGWRRAMAAR

KRIDDLIDAQIADARTAPRPDDHMLTTLISGCSEEGTTLSDNEIRDSIVSLITAGYETTS

GALAWAIYALLTVPGTWESAASEVARVLGGRVPAADDLSALTYLNGVVHETLRLYSPGVI

SARRVLRDLWFDGHRIRAGRLLIFSAYVTHRLPEIWPEPTEFRPLRWDPNAADYRKPAPH

EFIPFSGGLHRCIGAVMATTEMTVILARLVARAMLQLPAQRTHRIRAANFAALRPWPGLT

VEIRKSAPAQ

>CYP139A1(2589555384)Mycobacterium tuberculosis TKK-01-0030

MRYPLGEALLALYRWRGPLINAGVGGHGYTYLLGAEANRFVFANADAFSWSQTFESLVPV

DGPTALIVSDGADHRRRRSVVAPGLRHHHVQRYVATMVSNIDTVIDGWQPGQRLDIYQEL

RSAVRRSTAESLFGQRLAVHSDFLGEQLQPLLDLTRRPPQVMRLQQRVNSPGWRRAMAAR

KRIDDLIDAQIADARTAPRPDDHMLTTLISGCSEEGTTLSDNEIRDSIVSLITAGYETTS

GALAWAIYALLTVPGTWESAASEVARVLGGRVPAADDLSALTYLNGVVHETLRLYSPGVI

SARRVLRDLWFDGHRIRAGRLLIFSAYVTHRLPEIWPEPTEFRPLRWDPNAADYRKPAPH

EFIPFSGGLHRCIGAVMATTEMTVILARLVARAMLQLPAQRTHRIRAANFAALRPWPGLT

VEIRKSAPAQ

>CYP139A1(2589568736)Mycobacterium tuberculosis TKK-01-0033

MRYPLGEALLALYRWRGPLINAGVGGHGYTYLLGAEANRFVFANADAFSWSQTFESLVPV

DGPTALIVSDGADHRRRRSVVAPGLRHHHVQRYVATMVSNIDTVIDGWQPGQRLDIYQEL

RSAVRRSTAESLFGQRLAVHSDFLGEQLQPLLDLTRRPPQVMRLQQRVNSPGWRRAMAAR

KRIDDLIDAQIADARTAPRPDDHMLTTLISGCSEEGTTLSDNEIRDSIVSLITAGYETTS

GALAWAIYALLTVPGTWESAASEVARVLGGRVPAADDLSALTYLNGVVHETLRLYSPGVI

SARRVLRDLWFDGHRIRAGRLLIFSAYVTHRLPEIWPEPTEFRPLRWDPNAADYRKPAPH

EFIPFSGGLHRCIGAVMATTEMTVILARLVARAMLQLPAQRTHRIRAANFAALRPWPGLT

VEIRKSAPAQ

>CYP139A1(2589715710)Mycobacterium tuberculosis TKK-01-0084

MRYPLGEALLALYRWRGPLINAGVGGHGYTYLLGAEANRFVFANADAFSWSQTFESLVPV

DGPTALIVSDGADHRRRRSVVAPGLRHHHVQRYVATMVSNIDTVIDGWQPGQRLDIYQEL

RSAVRRSTAESLFGQRLAVHSDFLGEQLQPLLDLTRRPPQVMRLQQRVNSPGWRRAMAAR

KRIDDLIDAQIADARTAPRPDDHMLTTLISGCSEEGTTLSDNEIRDSIVSLITAGYETTS

GALAWAIYALLTVPGTWESAASEVARVLGGRVPAADDLSALTYLNGVVHETLRLYSPGVI

SARRVLRDLWFDGHRIRAGRLLIFSAYVTHRLPEIWPEPTEFRPLRWDPNAADYRKPAPH

EFIPFSGGLHRCIGAVMATTEMTVILARLVARAMLQLPAQRTHRIRAANFAALRPWPGLT

VEIRKSAPAQ

>CYP139A1(2592287696)Mycobacterium tuberculosis TKK_03_0043

MRYPLGEALLALYRWRGPLINAGVGGHGYTYLLGAEANRFVFANADAFSWSQTFESLVPV

DGPTALIVSDGADHRRRRSVVAPGLRHHHVQRYVATMVSNIDTVIDGWQPGQRLDIYQEL

RSAVRRSTAESLFGQRLAVHSDFLGEQLQPLLDLTRRPPQVMRLQQRVNSPGWRRAMAAR

KRIDDLIDAQIADARTAPRPDDHMLTTLISGCSEEGTTLSDNEIRDSIVSLITAGYETTS

GALAWAIYALLTVPGTWESAASEVARVLGGRVPAADDLSALTYLNGVVHETLRLYSPGVI

SARRVLRDLWFDGHRIRAGRLLIFSAYVTHRLPEIWPEPTEFRPLRWDPNAADYRKPAPH

EFIPFSGGLHRCIGAVMATTEMTVILARLVARAMLQLPAQRTHRIRAANFAALRPWPGLT

VEIRKSAPAQ

>CYP139A1(2592291479)Mycobacterium tuberculosis TKK_03_0036

MRYPLGEALLALYRWRGPLINAGVGGHGYTYLLGAEANRFVFANADAFSWSQTFESLVPV

DGPTALIVSDGADHRRRRSVVAPGLRHHHVQRYVATMVSNIDTVIDGWQPGQRLDIYQEL

RSAVRRSTAESLFGQRLAVHSDFLGEQLQPLLDLTRRPPQVMRLQQRVNSPGWRRAMAAR

KRIDDLIDAQIADARTAPRPDDHMLTTLISGCSEEGTTLSDNEIRDSIVSLITAGYETTS

GALAWAIYALLTVPGTWESAASEVARVLGGRVPAADDLSALTYLNGVVHETLRLYSPGVI

SARRVLRDLWFDGHRIRAGRLLIFSAYVTHRLPEIWPEPTEFRPLRWDPNAADYRKPAPH

EFIPFSGGLHRCIGAVMATTEMTVILARLVARAMLQLPAQRTHRIRAANFAALRPWPGLT

VEIRKSAPAQ

>CYP139A1(2592430392)Mycobacterium tuberculosis TKK_02_0012

MRYPLGEALLALYRWRGPLINAGVGGHGYTYLLGAEANRFVFANADAFSWSQTFESLVPV

DGPTALIVSDGADHRRRRSVVAPGLRHHHVQRYVATMVSNIDTVIDGWQPGQRLDIYQEL

RSAVRRSTAESLFGQRLAVHSDFLGEQLQPLLDLTRRPPQVMRLQQRVNSPGWRRAMAAR

KRIDDLIDAQIADARTAPRPDDHMLTTLISGCSEEGTTLSDNEIRDSIVSLITAGYETTS

GALAWAIYALLTVPGTWESAASEVARVLGGRVPAADDLSALTYLNGVVHETLRLYSPGVI

SARRVLRDLWFDGHRIRAGRLLIFSAYVTHRLPEIWPEPTEFRPLRWDPNAADYRKPAPH

EFIPFSGGLHRCIGAVMATTEMTVILARLVARAMLQLPAQRTHRIRAANFAALRPWPGLT

VEIRKSAPAQ

>CYP139A1(2574790496)Mycobacterium tuberculosis H1578

MRYPLGEALLALYRWRGPLINAGVGGHGYTYLLGAEANRFVFANADAFSWSQTFESLVPV

DGPTALIVSDGADHRRRRSVVAPGLRHHHVQRYVATMVSNIDTVIDGWQPGQRLDIYQEL

RSAVRRSTAESLFGQRLAVHSDFLGEQLQPLLDLTRRPPQVMRLQQRVNSPGWRRAMAAR

KRIDDLIDAQIADARTAPRPDDHMLTTLISGCSEEGTTLSDNEIRDSIVSLITAGYETTS

GALAWAIYALLTVPGTWESAASEVARVLGGRVPAADDLSALTYLNGVVHETLRLYSPGVI

SARRVLRDLWFDGHRIRAGRLLIFSAYVTHRLPEIWPEPTEFRPLRWDPNAADYRKPAPH

EFIPFSGGLHRCIGAVMATTEMTVILARLVARAMLQLPAQRTHRIRAANFAALRPWPGLT

VEIRKSAPAQ

>CYP139A1(2574834868)Mycobacterium tuberculosis KT-0087

MRYPLGEALLALYRWRGPLINAGVGGHGYTYLLGAEANRFVFANADAFSWSQTFESLVPV

DGPTALIVSDGADHRRRRSVVAPGLRHHHVQRYVATMVSNIDTVIDGWQPGQRLDIYQEL

RSAVRRSTAESLFGQRLAVHSDFLGEQLQPLLDLTRRPPQVMRLQQRVNSPGWRRAMAAR

KRIDDLIDAQIADARTAPRPDDHMLTTLISGCSEEGTTLSDNEIRDSIVSLITAGYETTS

GALAWAIYALLTVPGTWESAASEVARVLGGRVPAADDLSALTYLNGVVHETLRLYSPGVI

SARRVLRDLWFDGHRIRAGRLLIFSAYVTHRLPEIWPEPTEFRPLRWDPNAADYRKPAPH

EFIPFSGGLHRCIGAVMATTEMTVILARLVARAMLQLPAQRTHRIRAANFAALRPWPGLT

VEIRKSAPAQ

>CYP139A1(2576151818)Mycobacterium tuberculosis MAL010103

MRYPLGEALLALYRWRGPLINAGVGGHGYTYLLGAEANRFVFANADAFSWSQTFESLVPV

DGPTALIVSDGADHRRRRSVVAPGLRHHHVQRYVATMVSNIDTVIDGWQPGQRLDIYQEL

RSAVRRSTAESLFGQRLAVHSDFLGEQLQPLLDLTRRPPQVMRLQQRVNSPGWRRAMAAR

KRIDDLIDAQIADARTAPRPDDHMLTTLISGCSEEGTTLSDNEIRDSIVSLITAGYETTS

GALAWAIYALLTVPGTWESAASEVARVLGGRVPAADDLSALTYLNGVVHETLRLYSPGVI

SARRVLRDLWFDGHRIRAGRLLIFSAYVTHRLPEIWPEPTEFRPLRWDPNAADYRKPAPH

EFIPFSGGLHRCIGAVMATTEMTVILARLVARAMLQLPAQRTHRIRAANFAALRPWPGLT

VEIRKSAPAQ

>CYP139A1(2578111269)Mycobacterium tuberculosis BTB09-058

MRYPLGEALLALYRWRGPLINAGVGGHGYTYLLGAEANRFVFANADAFSWSQTFESLVPV

DGPTALIVSDGADHRRRRSVVAPGLRHHHVQRYVATMVSNIDTVIDGWQPGQRLDIYQEL

RSAVRRSTAESLFGQRLAVHSDFLGEQLQPLLDLTRRPPQVMRLQQRVNSPGWRRAMAAR

KRIDDLIDAQIADARTAPRPDDHMLTTLISGCSEEGTTLSDNEIRDSIVSLITAGYETTS

GALAWAIYALLTVPGTWESAASEVARVLGGRVPAADDLSALTYLNGVVHETLRLYSPGVI

SARRVLRDLWFDGHRIRAGRLLIFSAYVTHRLPEIWPEPTEFRPLRWDPNAADYRKPAPH

EFIPFSGGLHRCIGAVMATTEMTVILARLVARAMLQLPAQRTHRIRAANFAALRPWPGLT

VEIRKSAPAQ

>CYP139A1(2583723841)*Mycobacterium africanum* MAL010099

MRYPLGEALLALYRWRGPLINAGVGGHGYTYLLGAEANRFVFANADAFSWSQTFESLVPV

DGPTALIVSDGADHRRRRSVVAPGLRHHHVQRYVATMVSNIDTVIDGWQPGQRLDIYQEL

RSAVRRSTAESLFGQRLAVHSDFLGEQLQPLLDLTRRPPQVMRLQQRVNSPGWRRAMAAR

KRIDDLIDAQIADARTAPRPDDHMLTTLISGCSEEGTTLSDNEIRDSIVSLITAGYETTS

GALAWAIYALLTVPGTWESAASEVARVLGGRVPAADDLSALTYLNGVVHETLRLYSPGVI

SARRVLRDLWFDGHRIRAGRLLIFSAYVTHRLPEIWPEPTEFRPLRWDPNAADYRKPAPH

EFIPFSGGLHRCIGAVMATTEMTVILARLVARAMLQLPAQRTHRIRAANFAALRPWPGLT

VEIRKSAPAQ

>CYP139A1(2584631822)Mycobacterium tuberculosis BTB10-357

MRYPLGEALLALYRWRGPLINAGVGGHGYTYLLGAEANRFVFANADAFSWSQTFESLVPV

DGPTALIVSDGADHRRRRSVVAPGLRHHHVQRYVATMVSNIDTVIDGWQPGQRLDIYQEL

RSAVRRSTAESLFGQRLAVHSDFLGEQLQPLLDLTRRPPQVMRLQQRVNSPGWRRAMAAR

KRIDDLIDAQIADARTAPRPDDHMLTTLISGCSEEGTTLSDNEIRDSIVSLITAGYETTS

GALAWAIYALLTVPGTWESAASEVARVLGGRVPAADDLSALTYLNGVVHETLRLYSPGVI

SARRVLRDLWFDGHRIRAGRLLIFSAYVTHRLPEIWPEPTEFRPLRWDPNAADYRKPAPH

EFIPFSGGLHRCIGAVMATTEMTVILARLVARAMLQLPAQRTHRIRAANFAALRPWPGLT

VEIRKSAPAQ

>CYP139A1(2584721998)Mycobacterium tuberculosis KT-0057

MRYPLGEALLALYRWRGPLINAGVGGHGYTYLLGAEANRFVFANADAFSWSQTFESLVPV

DGPTALIVSDGADHRRRRSVVAPGLRHHHVQRYVATMVSNIDTVIDGWQPGQRLDIYQEL

RSAVRRSTAESLFGQRLAVHSDFLGEQLQPLLDLTRRPPQVMRLQQRVNSPGWRRAMAAR

KRIDDLIDAQIADARTAPRPDDHMLTTLISGCSEEGTTLSDNEIRDSIVSLITAGYETTS

GALAWAIYALLTVPGTWESAASEVARVLGGRVPAADDLSALTYLNGVVHETLRLYSPGVI

SARRVLRDLWFDGHRIRAGRLLIFSAYVTHRLPEIWPEPTEFRPLRWDPNAADYRKPAPH

EFIPFSGGLHRCIGAVMATTEMTVILARLVARAMLQLPAQRTHRIRAANFAALRPWPGLT

VEIRKSAPAQ

>CYP139A1(2584846770)Mycobacterium tuberculosis KT-0019

MRYPLGEALLALYRWRGPLINAGVGGHGYTYLLGAEANRFVFANADAFSWSQTFESLVPV

DGPTALIVSDGADHRRRRSVVAPGLRHHHVQRYVATMVSNIDTVIDGWQPGQRLDIYQEL

RSAVRRSTAESLFGQRLAVHSDFLGEQLQPLLDLTRRPPQVMRLQQRVNSPGWRRAMAAR

KRIDDLIDAQIADARTAPRPDDHMLTTLISGCSEEGTTLSDNEIRDSIVSLITAGYETTS

GALAWAIYALLTVPGTWESAASEVARVLGGRVPAADDLSALTYLNGVVHETLRLYSPGVI

SARRVLRDLWFDGHRIRAGRLLIFSAYVTHRLPEIWPEPTEFRPLRWDPNAADYRKPAPH

EFIPFSGGLHRCIGAVMATTEMTVILARLVARAMLQLPAQRTHRIRAANFAALRPWPGLT

VEIRKSAPAQ

>CYP139A1(2588591267)Mycobacterium tuberculosis Korean KIT87190

MRYPLGEALLALYRWRGPLINAGVGGHGYTYLLGAEANRFVFANADAFSWSQTFESLVPV

DGPTALIVSDGADHRRRRSVVAPGLRHHHVQRYVATMVSNIDTVIDGWQPGQRLDIYQEL

RSAVRRSTAESLFGQRLAVHSDFLGEQLQPLLDLTRRPPQVMRLQQRVNSPGWRRAMAAR

KRIDDLIDAQIADARTAPRPDDHMLTTLISGCSEEGTTLSDNEIRDSIVSLITAGYETTS

GALAWAIYALLTVPGTWESAASEVARVLGGRVPAADDLSALTYLNGVVHETLRLYSPGVI

SARRVLRDLWFDGHRIRAGRLLIFSAYVTHRLPEIWPEPTEFRPLRWDPNAADYRKPAPH

EFIPFSGGLHRCIGAVMATTEMTVILARLVARAMLQLPAQRTHRIRAANFAALRPWPGLT

VEIRKSAPAQ

>CYP139A1(2590007914)Mycobacterium tuberculosis MAL010087

MRYPLGEALLALYRWRGPLINAGVGGHGYTYLLGAEANRFVFANADAFSWSQTFESLVPV

DGPTALIVSDGADHRRRRSVVAPGLRHHHVQRYVATMVSNIDTVIDGWQPGQRLDIYQEL

RSAVRRSTAESLFGQRLAVHSDFLGEQLQPLLDLTRRPPQVMRLQQRVNSPGWRRAMAAR

KRIDDLIDAQIADARTAPRPDDHMLTTLISGCSEEGTTLSDNEIRDSIVSLITAGYETTS

GALAWAIYALLTVPGTWESAASEVARVLGGRVPAADDLSALTYLNGVVHETLRLYSPGVI

SARRVLRDLWFDGHRIRAGRLLIFSAYVTHRLPEIWPEPTEFRPLRWDPNAADYRKPAPH

EFIPFSGGLHRCIGAVMATTEMTVILARLVARAMLQLPAQRTHRIRAANFAALRPWPGLT

VEIRKSAPAQ

>CYP139A1(2590057066)Mycobacterium tuberculosis MAL020138

MRYPLGEALLALYRWRGPLINAGVGGHGYTYLLGAEANRFVFANADAFSWSQTFESLVPV

DGPTALIVSDGADHRRRRSVVAPGLRHHHVQRYVATMVSNIDTVIDGWQPGQRLDIYQEL

RSAVRRSTAESLFGQRLAVHSDFLGEQLQPLLDLTRRPPQVMRLQQRVNSPGWRRAMAAR

KRIDDLIDAQIADARTAPRPDDHMLTTLISGCSEEGTTLSDNEIRDSIVSLITAGYETTS

GALAWAIYALLTVPGTWESAASEVARVLGGRVPAADDLSALTYLNGVVHETLRLYSPGVI

SARRVLRDLWFDGHRIRAGRLLIFSAYVTHRLPEIWPEPTEFRPLRWDPNAADYRKPAPH

EFIPFSGGLHRCIGAVMATTEMTVILARLVARAMLQLPAQRTHRIRAANFAALRPWPGLT

VEIRKSAPAQ

>CYP139A1(2590061112)Mycobacterium tuberculosis MAL020136

MRYPLGEALLALYRWRGPLINAGVGGHGYTYLLGAEANRFVFANADAFSWSQTFESLVPV

DGPTALIVSDGADHRRRRSVVAPGLRHHHVQRYVATMVSNIDTVIDGWQPGQRLDIYQEL

RSAVRRSTAESLFGQRLAVHSDFLGEQLQPLLDLTRRPPQVMRLQQRVNSPGWRRAMAAR

KRIDDLIDAQIADARTAPRPDDHMLTTLISGCSEEGTTLSDNEIRDSIVSLITAGYETTS

GALAWAIYALLTVPGTWESAASEVARVLGGRVPAADDLSALTYLNGVVHETLRLYSPGVI

SARRVLRDLWFDGHRIRAGRLLIFSAYVTHRLPEIWPEPTEFRPLRWDPNAADYRKPAPH

EFIPFSGGLHRCIGAVMATTEMTVILARLVARAMLQLPAQRTHRIRAANFAALRPWPGLT

VEIRKSAPAQ

>CYP139A1(2590252317)Mycobacterium tuberculosis KT-0026

MRYPLGEALLALYRWRGPLINAGVGGHGYTYLLGAEANRFVFANADAFSWSQTFESLVPV

DGPTALIVSDGADHRRRRSVVAPGLRHHHVQRYVATMVSNIDTVIDGWQPGQRLDIYQEL

RSAVRRSTAESLFGQRLAVHSDFLGEQLQPLLDLTRRPPQVMRLQQRVNSPGWRRAMAAR

KRIDDLIDAQIADARTAPRPDDHMLTTLISGCSEEGTTLSDNEIRDSIVSLITAGYETTS

GALAWAIYALLTVPGTWESAASEVARVLGGRVPAADDLSALTYLNGVVHETLRLYSPGVI

SARRVLRDLWFDGHRIRAGRLLIFSAYVTHRLPEIWPEPTEFRPLRWDPNAADYRKPAPH

EFIPFSGGLHRCIGAVMATTEMTVILARLVARAMLQLPAQRTHRIRAANFAALRPWPGLT

VEIRKSAPAQ

>CYP139A1(2590543931)Mycobacterium tuberculosis KT-0079

MRYPLGEALLALYRWRGPLINAGVGGHGYTYLLGAEANRFVFANADAFSWSQTFESLVPV

DGPTALIVSDGADHRRRRSVVAPGLRHHHVQRYVATMVSNIDTVIDGWQPGQRLDIYQEL

RSAVRRSTAESLFGQRLAVHSDFLGEQLQPLLDLTRRPPQVMRLQQRVNSPGWRRAMAAR

KRIDDLIDAQIADARTAPRPDDHMLTTLISGCSEEGTTLSDNEIRDSIVSLITAGYETTS

GALAWAIYALLTVPGTWESAASEVARVLGGRVPAADDLSALTYLNGVVHETLRLYSPGVI

SARRVLRDLWFDGHRIRAGRLLIFSAYVTHRLPEIWPEPTEFRPLRWDPNAADYRKPAPH

EFIPFSGGLHRCIGAVMATTEMTVILARLVARAMLQLPAQRTHRIRAANFAALRPWPGLT

VEIRKSAPAQ

>CYP139A1(2574560432)Mycobacterium tuberculosis MD15977

MRYPLGEALLALYRWRGPLINAGVGGHGYTYLLGAEANRFVFANADAFSWSQTFESLVPV

DGPTALIVSDGADHRRRRSVVAPGLRHHHVQRYVATMVSNIDTVIDGWQPGQRLDIYQEL

RSAVRRSTAESLFGQRLAVHSDFLGEQLQPLLDLTRRPPQVMRLQQRVNSPGWRRAMAAR

KRIDDLIDAQIADARTAPRPDDHMLTTLISGCSEEGTTLSDNEIRDSIVSLITAGYETTS

GALAWAIYALLTVPGTWESAASEVARVLGGRVPAADDLSALTYLNGVVHETLRLYSPGVI

SARRVLRDLWFDGHRIRAGRLLIFSAYVTHRLPEIWPEPTEFRPLRWDPNAADYRKPAPH

EFIPFSGGLHRCIGAVMATTEMTVILARLVARAMLQLPAQRTHRIRAANFAALRPWPGLT

VEIRKSAPAQ

>CYP139A1(2575495357)Mycobacterium tuberculosis TKK-01-0010

MRYPLGEALLALYRWRGPLINAGVGGHGYTYLLGAEANRFVFANADAFSWSQTFESLVPV

DGPTALIVSDGADHRRRRSVVAPGLRHHHVQRYVATMVSNIDTVIDGWQPGQRLDIYQEL

RSAVRRSTAESLFGQRLAVHSDFLGEQLQPLLDLTRRPPQVMRLQQRVNSPGWRRAMAAR

KRIDDLIDAQIADARTAPRPDDHMLTTLISGCSEEGTTLSDNEIRDSIVSLITAGYETTS

GALAWAIYALLTVPGTWESAASEVARVLGGRVPAADDLSALTYLNGVVHETLRLYSPGVI

SARRVLRDLWFDGHRIRAGRLLIFSAYVTHRLPEIWPEPTEFRPLRWDPNAADYRKPAPH

EFIPFSGGLHRCIGAVMATTEMTVILARLVARAMLQLPAQRTHRIRAANFAALRPWPGLT

VEIRKSAPAQ

>CYP139A1(2575638157)Mycobacterium tuberculosis TRUG0088

MRYPLGEALLALYRWRGPLINAGVGGHGYTYLLGAEANRFVFANADAFSWSQTFESLVPV

DGPTALIVSDGADHRRRRSVVAPGLRHHHVQRYVATMVSNIDTVIDGWQPGQRLDIYQEL

RSAVRRSTAESLFGQRLAVHSDFLGEQLQPLLDLTRRPPQVMRLQQRVNSPGWRRAMAAR

KRIDDLIDAQIADARTAPRPDDHMLTTLISGCSEEGTTLSDNEIRDSIVSLITAGYETTS

GALAWAIYALLTVPGTWESAASEVARVLGGRVPAADDLSALTYLNGVVHETLRLYSPGVI

SARRVLRDLWFDGHRIRAGRLLIFSAYVTHRLPEIWPEPTEFRPLRWDPNAADYRKPAPH

EFIPFSGGLHRCIGAVMATTEMTVILARLVARAMLQLPAQRTHRIRAANFAALRPWPGLT

VEIRKSAPAQ

>CYP139A1(2575697737)Mycobacterium tuberculosis XTB13-131

MRYPLGEALLALYRWRGPLINAGVGGHGYTYLLGAEANRFVFANADAFSWSQTFESLVPV

DGPTALIVSDGADHRRRRSVVAPGLRHHHVQRYVATMVSNIDTVIDGWQPGQRLDIYQEL

RSAVRRSTAESLFGQRLAVHSDFLGEQLQPLLDLTRRPPQVMRLQQRVNSPGWRRAMAAR

KRIDDLIDAQIADARTAPRPDDHMLTTLISGCSEEGTTLSDNEIRDSIVSLITAGYETTS

GALAWAIYALLTVPGTWESAASEVARVLGGRVPAADDLSALTYLNGVVHETLRLYSPGVI

SARRVLRDLWFDGHRIRAGRLLIFSAYVTHRLPEIWPEPTEFRPLRWDPNAADYRKPAPH

EFIPFSGGLHRCIGAVMATTEMTVILARLVARAMLQLPAQRTHRIRAANFAALRPWPGLT

VEIRKSAPAQ

>CYP139A1(2575988382)Mycobacterium tuberculosis MD16775

MRYPLGEALLALYRWRGPLINAGVGGHGYTYLLGAEANRFVFANADAFSWSQTFESLVPV

DGPTALIVSDGADHRRRRSVVAPGLRHHHVQRYVATMVSNIDTVIDGWQPGQRLDIYQEL

RSAVRRSTAESLFGQRLAVHSDFLGEQLQPLLDLTRRPPQVMRLQQRVNSPGWRRAMAAR

KRIDDLIDAQIADARTAPRPDDHMLTTLISGCSEEGTTLSDNEIRDSIVSLITAGYETTS

GALAWAIYALLTVPGTWESAASEVARVLGGRVPAADDLSALTYLNGVVHETLRLYSPGVI

SARRVLRDLWFDGHRIRAGRLLIFSAYVTHRLPEIWPEPTEFRPLRWDPNAADYRKPAPH

EFIPFSGGLHRCIGAVMATTEMTVILARLVARAMLQLPAQRTHRIRAANFAALRPWPGLT

VEIRKSAPAQ

>CYP139A1(2576070324)Mycobacterium tuberculosis TRUG0076

MRYPLGEALLALYRWRGPLINAGVGGHGYTYLLGAEANRFVFANADAFSWSQTFESLVPV

DGPTALIVSDGADHRRRRSVVAPGLRHHHVQRYVATMVSNIDTVIDGWQPGQRLDIYQEL

RSAVRRSTAESLFGQRLAVHSDFLGEQLQPLLDLTRRPPQVMRLQQRVNSPGWRRAMAAR

KRIDDLIDAQIADARTAPRPDDHMLTTLISGCSEEGTTLSDNEIRDSIVSLITAGYETTS

GALAWAIYALLTVPGTWESAASEVARVLGGRVPAADDLSALTYLNGVVHETLRLYSPGVI

SARRVLRDLWFDGHRIRAGRLLIFSAYVTHRLPEIWPEPTEFRPLRWDPNAADYRKPAPH

EFIPFSGGLHRCIGAVMATTEMTVILARLVARAMLQLPAQRTHRIRAANFAALRPWPGLT

VEIRKSAPAQ

>CYP139A1(2576613004)Mycobacterium tuberculosis TB_RSA66

MRYPLGEALLALYRWRGPLINAGVGGHGYTYLLGAEANRFVFANADAFSWSQTFESLVPV

DGPTALIVSDGADHRRRRSVVAPGLRHHHVQRYVATMVSNIDTVIDGWQPGQRLDIYQEL

RSAVRRSTAESLFGQRLAVHSDFLGEQLQPLLDLTRRPPQVMRLQQRVNSPGWRRAMAAR

KRIDDLIDAQIADARTAPRPDDHMLTTLISGCSEEGTTLSDNEIRDSIVSLITAGYETTS

GALAWAIYALLTVPGTWESAASEVARVLGGRVPAADDLSALTYLNGVVHETLRLYSPGVI

SARRVLRDLWFDGHRIRAGRLLIFSAYVTHRLPEIWPEPTEFRPLRWDPNAADYRKPAPH

EFIPFSGGLHRCIGAVMATTEMTVILARLVARAMLQLPAQRTHRIRAANFAALRPWPGLT

VEIRKSAPAQ

>CYP139A1(2577091164)Mycobacterium tuberculosis TB_RSA12

MRYPLGEALLALYRWRGPLINAGVGGHGYTYLLGAEANRFVFANADAFSWSQTFESLVPV

DGPTALIVSDGADHRRRRSVVAPGLRHHHVQRYVATMVSNIDTVIDGWQPGQRLDIYQEL

RSAVRRSTAESLFGQRLAVHSDFLGEQLQPLLDLTRRPPQVMRLQQRVNSPGWRRAMAAR

KRIDDLIDAQIADARTAPRPDDHMLTTLISGCSEEGTTLSDNEIRDSIVSLITAGYETTS

GALAWAIYALLTVPGTWESAASEVARVLGGRVPAADDLSALTYLNGVVHETLRLYSPGVI

SARRVLRDLWFDGHRIRAGRLLIFSAYVTHRLPEIWPEPTEFRPLRWDPNAADYRKPAPH

EFIPFSGGLHRCIGAVMATTEMTVILARLVARAMLQLPAQRTHRIRAANFAALRPWPGLT

VEIRKSAPAQ

>CYP139A1(2577569748)Mycobacterium tuberculosis TB_RSA161

MRYPLGEALLALYRWRGPLINAGVGGHGYTYLLGAEANRFVFANADAFSWSQTFESLVPV

DGPTALIVSDGADHRRRRSVVAPGLRHHHVQRYVATMVSNIDTVIDGWQPGQRLDIYQEL

RSAVRRSTAESLFGQRLAVHSDFLGEQLQPLLDLTRRPPQVMRLQQRVNSPGWRRAMAAR

KRIDDLIDAQIADARTAPRPDDHMLTTLISGCSEEGTTLSDNEIRDSIVSLITAGYETTS

GALAWAIYALLTVPGTWESAASEVARVLGGRVPAADDLSALTYLNGVVHETLRLYSPGVI

SARRVLRDLWFDGHRIRAGRLLIFSAYVTHRLPEIWPEPTEFRPLRWDPNAADYRKPAPH

EFIPFSGGLHRCIGAVMATTEMTVILARLVARAMLQLPAQRTHRIRAANFAALRPWPGLT

VEIRKSAPAQ

>CYP139A1(2577697067)Mycobacterium tuberculosis MD16728

MRYPLGEALLALYRWRGPLINAGVGGHGYTYLLGAEANRFVFANADAFSWSQTFESLVPV

DGPTALIVSDGADHRRRRSVVAPGLRHHHVQRYVATMVSNIDTVIDGWQPGQRLDIYQEL

RSAVRRSTAESLFGQRLAVHSDFLGEQLQPLLDLTRRPPQVMRLQQRVNSPGWRRAMAAR

KRIDDLIDAQIADARTAPRPDDHMLTTLISGCSEEGTTLSDNEIRDSIVSLITAGYETTS

GALAWAIYALLTVPGTWESAASEVARVLGGRVPAADDLSALTYLNGVVHETLRLYSPGVI

SARRVLRDLWFDGHRIRAGRLLIFSAYVTHRLPEIWPEPTEFRPLRWDPNAADYRKPAPH

EFIPFSGGLHRCIGAVMATTEMTVILARLVARAMLQLPAQRTHRIRAANFAALRPWPGLT

VEIRKSAPAQ

>CYP139A1(2584795634)Mycobacterium tuberculosis UG-D

MRYPLGEALLALYRWRGPLINAGVGGHGYTYLLGAEANRFVFANADAFSWSQTFESLVPV

DGPTALIVSDGADHRRRRSVVAPGLRHHHVQRYVATMVSNIDTVIDGWQPGQRLDIYQEL

RSAVRRSTAESLFGQRLAVHSDFLGEQLQPLLDLTRRPPQVMRLQQRVNSPGWRRAMAAR

KRIDDLIDAQIADARTAPRPDDHMLTTLISGCSEEGTTLSDNEIRDSIVSLITAGYETTS

GALAWAIYALLTVPGTWESAASEVARVLGGRVPAADDLSALTYLNGVVHETLRLYSPGVI

SARRVLRDLWFDGHRIRAGRLLIFSAYVTHRLPEIWPEPTEFRPLRWDPNAADYRKPAPH

EFIPFSGGLHRCIGAVMATTEMTVILARLVARAMLQLPAQRTHRIRAANFAALRPWPGLT

VEIRKSAPAQ

>CYP139A1(2589158600)Mycobacterium tuberculosis OFXR-12

MRYPLGEALLALYRWRGPLINAGVGGHGYTYLLGAEANRFVFANADAFSWSQTFESLVPV

DGPTALIVSDGADHRRRRSVVAPGLRHHHVQRYVATMVSNIDTVIDGWQPGQRLDIYQEL

RSAVRRSTAESLFGQRLAVHSDFLGEQLQPLLDLTRRPPQVMRLQQRVNSPGWRRAMAAR

KRIDDLIDAQIADARTAPRPDDHMLTTLISGCSEEGTTLSDNEIRDSIVSLITAGYETTS

GALAWAIYALLTVPGTWESAASEVARVLGGRVPAADDLSALTYLNGVVHETLRLYSPGVI

SARRVLRDLWFDGHRIRAGRLLIFSAYVTHRLPEIWPEPTEFRPLRWDPNAADYRKPAPH

EFIPFSGGLHRCIGAVMATTEMTVILARLVARAMLQLPAQRTHRIRAANFAALRPWPGLT

VEIRKSAPAQ

>CYP139A1(2589491285)Mycobacterium tuberculosis TKK-01-0001

MRYPLGEALLALYRWRGPLINAGVGGHGYTYLLGAEANRFVFANADAFSWSQTFESLVPV

DGPTALIVSDGADHRRRRSVVAPGLRHHHVQRYVATMVSNIDTVIDGWQPGQRLDIYQEL

RSAVRRSTAESLFGQRLAVHSDFLGEQLQPLLDLTRRPPQVMRLQQRVNSPGWRRAMAAR

KRIDDLIDAQIADARTAPRPDDHMLTTLISGCSEEGTTLSDNEIRDSIVSLITAGYETTS

GALAWAIYALLTVPGTWESAASEVARVLGGRVPAADDLSALTYLNGVVHETLRLYSPGVI

SARRVLRDLWFDGHRIRAGRLLIFSAYVTHRLPEIWPEPTEFRPLRWDPNAADYRKPAPH

EFIPFSGGLHRCIGAVMATTEMTVILARLVARAMLQLPAQRTHRIRAANFAALRPWPGLT

VEIRKSAPAQ

>CYP139A1(2589613612)Mycobacterium tuberculosis TKK-01-0048

MRYPLGEALLALYRWRGPLINAGVGGHGYTYLLGAEANRFVFANADAFSWSQTFESLVPV

DGPTALIVSDGADHRRRRSVVAPGLRHHHVQRYVATMVSNIDTVIDGWQPGQRLDIYQEL

RSAVRRSTAESLFGQRLAVHSDFLGEQLQPLLDLTRRPPQVMRLQQRVNSPGWRRAMAAR

KRIDDLIDAQIADARTAPRPDDHMLTTLISGCSEEGTTLSDNEIRDSIVSLITAGYETTS

GALAWAIYALLTVPGTWESAASEVARVLGGRVPAADDLSALTYLNGVVHETLRLYSPGVI

SARRVLRDLWFDGHRIRAGRLLIFSAYVTHRLPEIWPEPTEFRPLRWDPNAADYRKPAPH

EFIPFSGGLHRCIGAVMATTEMTVILARLVARAMLQLPAQRTHRIRAANFAALRPWPGLT

VEIRKSAPAQ

>CYP139A1(2589650472)Mycobacterium tuberculosis TKK-01-0055

MRYPLGEALLALYRWRGPLINAGVGGHGYTYLLGAEANRFVFANADAFSWSQTFESLVPV

DGPTALIVSDGADHRRRRSVVAPGLRHHHVQRYVATMVSNIDTVIDGWQPGQRLDIYQEL

RSAVRRSTAESLFGQRLAVHSDFLGEQLQPLLDLTRRPPQVMRLQQRVNSPGWRRAMAAR

KRIDDLIDAQIADARTAPRPDDHMLTTLISGCSEEGTTLSDNEIRDSIVSLITAGYETTS

GALAWAIYALLTVPGTWESAASEVARVLGGRVPAADDLSALTYLNGVVHETLRLYSPGVI

SARRVLRDLWFDGHRIRAGRLLIFSAYVTHRLPEIWPEPTEFRPLRWDPNAADYRKPAPH

EFIPFSGGLHRCIGAVMATTEMTVILARLVARAMLQLPAQRTHRIRAANFAALRPWPGLT

VEIRKSAPAQ

>CYP139A1(2589699410)Mycobacterium tuberculosis TKK-01-0078

MRYPLGEALLALYRWRGPLINAGVGGHGYTYLLGAEANRFVFANADAFSWSQTFESLVPV

DGPTALIVSDGADHRRRRSVVAPGLRHHHVQRYVATMVSNIDTVIDGWQPGQRLDIYQEL

RSAVRRSTAESLFGQRLAVHSDFLGEQLQPLLDLTRRPPQVMRLQQRVNSPGWRRAMAAR

KRIDDLIDAQIADARTAPRPDDHMLTTLISGCSEEGTTLSDNEIRDSIVSLITAGYETTS

GALAWAIYALLTVPGTWESAASEVARVLGGRVPAADDLSALTYLNGVVHETLRLYSPGVI

SARRVLRDLWFDGHRIRAGRLLIFSAYVTHRLPEIWPEPTEFRPLRWDPNAADYRKPAPH

EFIPFSGGLHRCIGAVMATTEMTVILARLVARAMLQLPAQRTHRIRAANFAALRPWPGLT

VEIRKSAPAQ

>CYP139A1(2590366449)Mycobacterium tuberculosis OFXR-23

MRYPLGEALLALYRWRGPLINAGVGGHGYTYLLGAEANRFVFANADAFSWSQTFESLVPV

DGPTALIVSDGADHRRRRSVVAPGLRHHHVQRYVATMVSNIDTVIDGWQPGQRLDIYQEL

RSAVRRSTAESLFGQRLAVHSDFLGEQLQPLLDLTRRPPQVMRLQQRVNSPGWRRAMAAR

KRIDDLIDAQIADARTAPRPDDHMLTTLISGCSEEGTTLSDNEIRDSIVSLITAGYETTS

GALAWAIYALLTVPGTWESAASEVARVLGGRVPAADDLSALTYLNGVVHETLRLYSPGVI

SARRVLRDLWFDGHRIRAGRLLIFSAYVTHRLPEIWPEPTEFRPLRWDPNAADYRKPAPH

EFIPFSGGLHRCIGAVMATTEMTVILARLVARAMLQLPAQRTHRIRAANFAALRPWPGLT

VEIRKSAPAQ

>CYP139A1(2592226489)Mycobacterium tuberculosis TKK_04_0022

MRYPLGEALLALYRWRGPLINAGVGGHGYTYLLGAEANRFVFANADAFSWSQTFESLVPV

DGPTALIVSDGADHRRRRSVVAPGLRHHHVQRYVATMVSNIDTVIDGWQPGQRLDIYQEL

RSAVRRSTAESLFGQRLAVHSDFLGEQLQPLLDLTRRPPQVMRLQQRVNSPGWRRAMAAR

KRIDDLIDAQIADARTAPRPDDHMLTTLISGCSEEGTTLSDNEIRDSIVSLITAGYETTS

GALAWAIYALLTVPGTWESAASEVARVLGGRVPAADDLSALTYLNGVVHETLRLYSPGVI

SARRVLRDLWFDGHRIRAGRLLIFSAYVTHRLPEIWPEPTEFRPLRWDPNAADYRKPAPH

EFIPFSGGLHRCIGAVMATTEMTVILARLVARAMLQLPAQRTHRIRAANFAALRPWPGLT

VEIRKSAPAQ

>CYP139A1(2592574645)Mycobacterium tuberculosis TKK_04_0030

MRYPLGEALLALYRWRGPLINAGVGGHGYTYLLGAEANRFVFANADAFSWSQTFESLVPV

DGPTALIVSDGADHRRRRSVVAPGLRHHHVQRYVATMVSNIDTVIDGWQPGQRLDIYQEL

RSAVRRSTAESLFGQRLAVHSDFLGEQLQPLLDLTRRPPQVMRLQQRVNSPGWRRAMAAR

KRIDDLIDAQIADARTAPRPDDHMLTTLISGCSEEGTTLSDNEIRDSIVSLITAGYETTS

GALAWAIYALLTVPGTWESAASEVARVLGGRVPAADDLSALTYLNGVVHETLRLYSPGVI

SARRVLRDLWFDGHRIRAGRLLIFSAYVTHRLPEIWPEPTEFRPLRWDPNAADYRKPAPH

EFIPFSGGLHRCIGAVMATTEMTVILARLVARAMLQLPAQRTHRIRAANFAALRPWPGLT

VEIRKSAPAQ

>CYP139A1(648473432)Mycobacterium tuberculosis SUMu008

MRYPLGEALLALYRWRGPLINAGVGGHGYTYLLGAEANRFVFANADAFSWSQTFESLVPV

DGPTALIVSDGADHRRRRSVVAPGLRHHHVQRYVATMVSNIDTVIDGWQPGQRLDIYQEL

RSAVRRSTAESLFGQRLAVHSDFLGEQLQPLLDLTRRPPQVMRLQQRVNSPGWRRAMAAR

KRIDDLIDAQIADARTAPRPDDHMLTTLISGCSEEGTTLSDNEIRDSIVSLITAGYETTS

GALAWAIYALLTVPGTWESAASEVARVLGGRVPAADDLSALTYLNGVVHETLRLYSPGVI

SARRVLRDLWFDGHRIRAGRLLIFSAYVTHRLPEIWPEPTEFRPLRWDPNAADYRKPAPH

EFIPFSGGLHRCIGAVMATTEMTVILARLVARAMLQLPAQRTHRIRAANFAALRPWPGLT

VEIRKSAPAQ

>CYP139A1(2574703562)Mycobacterium tuberculosis KT-0067

MRYPLGEALLALYRWRGPLINAGVGGHGYTYLLGAEANRFVFANADAFSWSQTFESLVPV

DGPTALIVSDGADHRRRRSVVAPGLRHHHVQRYVATMVSNIDTVIDGWQPGQRLDIYQEL

RSAVRRSTAESLFGQRLAVHSDFLGEQLQPLLDLTRRPPQVMRLQQRVNSPGWRRAMAAR

KRIDDLIDAQIADARTAPRPDDHMLTTLISGCSEEGTTLSDNEIRDSIVSLITAGYETTS

GALAWAIYALLTVPGTWESAASEVARVLGGRVPAADDLSALTYLNGVVHETLRLYSPGVI

SARRVLRDLWFDGHRIRAGRLLIFSAYVTHRLPEIWPEPTEFRPLRWDPNAADYRKPAPH

EFIPFSGGLHRCIGAVMATTEMTVILARLVARAMLQLPAQRTHRIRAANFAALRPWPGLT

VEIRKSAPAQ

>CYP139A1(2575794202)Mycobacterium tuberculosis M1559

MRYPLGEALLALYRWRGPLINAGVGGHGYTYLLGAEANRFVFANADAFSWSQTFESLVPV

DGPTALIVSDGADHRRRRSVVAPGLRHHHVQRYVATMVSNIDTVIDGWQPGQRLDIYQEL

RSAVRRSTAESLFGQRLAVHSDFLGEQLQPLLDLTRRPPQVMRLQQRVNSPGWRRAMAAR

KRIDDLIDAQIADARTAPRPDDHMLTTLISGCSEEGTTLSDNEIRDSIVSLITAGYETTS

GALAWAIYALLTVPGTWESAASEVARVLGGRVPAADDLSALTYLNGVVHETLRLYSPGVI

SARRVLRDLWFDGHRIRAGRLLIFSAYVTHRLPEIWPEPTEFRPLRWDPNAADYRKPAPH

EFIPFSGGLHRCIGAVMATTEMTVILARLVARAMLQLPAQRTHRIRAANFAALRPWPGLT

VEIRKSAPAQ

>CYP139A1(2576967330)Mycobacterium tuberculosis BTB10-487

MRYPLGEALLALYRWRGPLINAGVGGHGYTYLLGAEANRFVFANADAFSWSQTFESLVPV

DGPTALIVSDGADHRRRRSVVAPGLRHHHVQRYVATMVSNIDTVIDGWQPGQRLDIYQEL

RSAVRRSTAESLFGQRLAVHSDFLGEQLQPLLDLTRRPPQVMRLQQRVNSPGWRRAMAAR

KRIDDLIDAQIADARTAPRPDDHMLTTLISGCSEEGTTLSDNEIRDSIVSLITAGYETTS

GALAWAIYALLTVPGTWESAASEVARVLGGRVPAADDLSALTYLNGVVHETLRLYSPGVI

SARRVLRDLWFDGHRIRAGRLLIFSAYVTHRLPEIWPEPTEFRPLRWDPNAADYRKPAPH

EFIPFSGGLHRCIGAVMATTEMTVILARLVARAMLQLPAQRTHRIRAANFAALRPWPGLT

VEIRKSAPAQ

>CYP139A1(2577659800)Mycobacterium tuberculosis BTB08-022

MRYPLGEALLALYRWRGPLINAGVGGHGYTYLLGAEANRFVFANADAFSWSQTFESLVPV

DGPTALIVSDGADHRRRRSVVAPGLRHHHVQRYVATMVSNIDTVIDGWQPGQRLDIYQEL

RSAVRRSTAESLFGQRLAVHSDFLGEQLQPLLDLTRRPPQVMRLQQRVNSPGWRRAMAAR

KRIDDLIDAQIADARTAPRPDDHMLTTLISGCSEEGTTLSDNEIRDSIVSLITAGYETTS

GALAWAIYALLTVPGTWESAASEVARVLGGRVPAADDLSALTYLNGVVHETLRLYSPGVI

SARRVLRDLWFDGHRIRAGRLLIFSAYVTHRLPEIWPEPTEFRPLRWDPNAADYRKPAPH

EFIPFSGGLHRCIGAVMATTEMTVILARLVARAMLQLPAQRTHRIRAANFAALRPWPGLT

VEIRKSAPAQ

>CYP139A1(2577918576)Mycobacterium tuberculosis M2249

MRYPLGEALLALYRWRGPLINAGVGGHGYTYLLGAEANRFVFANADAFSWSQTFESLVPV

DGPTALIVSDGADHRRRRSVVAPGLRHHHVQRYVATMVSNIDTVIDGWQPGQRLDIYQEL

RSAVRRSTAESLFGQRLAVHSDFLGEQLQPLLDLTRRPPQVMRLQQRVNSPGWRRAMAAR

KRIDDLIDAQIADARTAPRPDDHMLTTLISGCSEEGTTLSDNEIRDSIVSLITAGYETTS

GALAWAIYALLTVPGTWESAASEVARVLGGRVPAADDLSALTYLNGVVHETLRLYSPGVI

SARRVLRDLWFDGHRIRAGRLLIFSAYVTHRLPEIWPEPTEFRPLRWDPNAADYRKPAPH

EFIPFSGGLHRCIGAVMATTEMTVILARLVARAMLQLPAQRTHRIRAANFAALRPWPGLT

VEIRKSAPAQ

>CYP139A1(2578033540)Mycobacterium tuberculosis M2116

MRYPLGEALLALYRWRGPLINAGVGGHGYTYLLGAEANRFVFANADAFSWSQTFESLVPV

DGPTALIVSDGADHRRRRSVVAPGLRHHHVQRYVATMVSNIDTVIDGWQPGQRLDIYQEL

RSAVRRSTAESLFGQRLAVHSDFLGEQLQPLLDLTRRPPQVMRLQQRVNSPGWRRAMAAR

KRIDDLIDAQIADARTAPRPDDHMLTTLISGCSEEGTTLSDNEIRDSIVSLITAGYETTS

GALAWAIYALLTVPGTWESAASEVARVLGGRVPAADDLSALTYLNGVVHETLRLYSPGVI

SARRVLRDLWFDGHRIRAGRLLIFSAYVTHRLPEIWPEPTEFRPLRWDPNAADYRKPAPH

EFIPFSGGLHRCIGAVMATTEMTVILARLVARAMLQLPAQRTHRIRAANFAALRPWPGLT

VEIRKSAPAQ

>CYP139A1(2581869776)*Mycobacterium africanum* MAL010118

MRYPLGEALLALYRWRGPLINAGVGGHGYTYLLGAEANRFVFANADAFSWSQTFESLVPV

DGPTALIVSDGADHRRRRSVVAPGLRHHHVQRYVATMVSNIDTVIDGWQPGQRLDIYQEL

RSAVRRSTAESLFGQRLAVHSDFLGEQLQPLLDLTRRPPQVMRLQQRVNSPGWRRAMAAR

KRIDDLIDAQIADARTAPRPDDHMLTTLISGCSEEGTTLSDNEIRDSIVSLITAGYETTS

GALAWAIYALLTVPGTWESAASEVARVLGGRVPAADDLSALTYLNGVVHETLRLYSPGVI

SARRVLRDLWFDGHRIRAGRLLIFSAYVTHRLPEIWPEPTEFRPLRWDPNAADYRKPAPH

EFIPFSGGLHRCIGAVMATTEMTVILARLVARAMLQLPAQRTHRIRAANFAALRPWPGLT

VEIRKSAPAQ

>CYP139A1(2583745288)*Mycobacterium bovis*MAL010093

MRYPLGEALLALYRWRGPLINAGVGGHGYTYLLGAEANRFVFANADAFSWSQTFESLVPV

DGPTALIVSDGADHRRRRSVVAPGLRHHHVQRYVATMVSNIDTVIDGWQPGQRLDIYQEL

RSAVRRSTAESLFGQRLAVHSDFLGEQLQPLLDLTRRPPQVMRLQQRVNSPGWRRAMAAR

KRIDDLIDAQIADARTAPRPDDHMLTTLISGCSEEGTTLSDNEIRDSIVSLITAGYETTS

GALAWAIYALLTVPGTWESAASEVARVLGGRVPAADDLSALTYLNGVVHETLRLYSPGVI

SARRVLRDLWFDGHRIRAGRLLIFSAYVTHRLPEIWPEPTEFRPLRWDPNAADYRKPAPH

EFIPFSGGLHRCIGAVMATTEMTVILARLVARAMLQLPAQRTHRIRAANFAALRPWPGLT

VEIRKSAPAQ

>CYP139A1(2590086658)Mycobacterium tuberculosis MAL020167

MRYPLGEALLALYRWRGPLINAGVGGHGYTYLLGAEANRFVFANADAFSWSQTFESLVPV

DGPTALIVSDGADHRRRRSVVAPGLRHHHVQRYVATMVSNIDTVIDGWQPGQRLDIYQEL

RSAVRRSTAESLFGQRLAVHSDFLGEQLQPLLDLTRRPPQVMRLQQRVNSPGWRRAMAAR

KRIDDLIDAQIADARTAPRPDDHMLTTLISGCSEEGTTLSDNEIRDSIVSLITAGYETTS

GALAWAIYALLTVPGTWESAASEVARVLGGRVPAADDLSALTYLNGVVHETLRLYSPGVI

SARRVLRDLWFDGHRIRAGRLLIFSAYVTHRLPEIWPEPTEFRPLRWDPNAADYRKPAPH

EFIPFSGGLHRCIGAVMATTEMTVILARLVARAMLQLPAQRTHRIRAANFAALRPWPGLT

VEIRKSAPAQ

>CYP139A1(2590104693)Mycobacterium tuberculosis MAL020150

MRYPLGEALLALYRWRGPLINAGVGGHGYTYLLGAEANRFVFANADAFSWSQTFESLVPV

DGPTALIVSDGADHRRRRSVVAPGLRHHHVQRYVATMVSNIDTVIDGWQPGQRLDIYQEL

RSAVRRSTAESLFGQRLAVHSDFLGEQLQPLLDLTRRPPQVMRLQQRVNSPGWRRAMAAR

KRIDDLIDAQIADARTAPRPDDHMLTTLISGCSEEGTTLSDNEIRDSIVSLITAGYETTS

GALAWAIYALLTVPGTWESAASEVARVLGGRVPAADDLSALTYLNGVVHETLRLYSPGVI

SARRVLRDLWFDGHRIRAGRLLIFSAYVTHRLPEIWPEPTEFRPLRWDPNAADYRKPAPH

EFIPFSGGLHRCIGAVMATTEMTVILARLVARAMLQLPAQRTHRIRAANFAALRPWPGLT

VEIRKSAPAQ

>CYP139A1(2590187116)Mycobacterium tuberculosis MAL020206

MRYPLGEALLALYRWRGPLINAGVGGHGYTYLLGAEANRFVFANADAFSWSQTFESLVPV

DGPTALIVSDGADHRRRRSVVAPGLRHHHVQRYVATMVSNIDTVIDGWQPGQRLDIYQEL

RSAVRRSTAESLFGQRLAVHSDFLGEQLQPLLDLTRRPPQVMRLQQRVNSPGWRRAMAAR

KRIDDLIDAQIADARTAPRPDDHMLTTLISGCSEEGTTLSDNEIRDSIVSLITAGYETTS

GALAWAIYALLTVPGTWESAASEVARVLGGRVPAADDLSALTYLNGVVHETLRLYSPGVI

SARRVLRDLWFDGHRIRAGRLLIFSAYVTHRLPEIWPEPTEFRPLRWDPNAADYRKPAPH

EFIPFSGGLHRCIGAVMATTEMTVILARLVARAMLQLPAQRTHRIRAANFAALRPWPGLT

VEIRKSAPAQ

>CYP139A1(2590382529)Mycobacterium tuberculosis OFXR-18

MRYPLGEALLALYRWRGPLINAGVGGHGYTYLLGAEANRFVFANADAFSWSQTFESLVPV

DGPTALIVSDGADHRRRRSVVAPGLRHHHVQRYVATMVSNIDTVIDGWQPGQRLDIYQEL

RSAVRRSTAESLFGQRLAVHSDFLGEQLQPLLDLTRRPPQVMRLQQRVNSPGWRRAMAAR

KRIDDLIDAQIADARTAPRPDDHMLTTLISGCSEEGTTLSDNEIRDSIVSLITAGYETTS

GALAWAIYALLTVPGTWESAASEVARVLGGRVPAADDLSALTYLNGVVHETLRLYSPGVI

SARRVLRDLWFDGHRIRAGRLLIFSAYVTHRLPEIWPEPTEFRPLRWDPNAADYRKPAPH

EFIPFSGGLHRCIGAVMATTEMTVILARLVARAMLQLPAQRTHRIRAANFAALRPWPGLT

VEIRKSAPAQ

>CYP139A1(2590510255)Mycobacterium tuberculosis KT-0099

MRYPLGEALLALYRWRGPLINAGVGGHGYTYLLGAEANRFVFANADAFSWSQTFESLVPV

DGPTALIVSDGADHRRRRSVVAPGLRHHHVQRYVATMVSNIDTVIDGWQPGQRLDIYQEL

RSAVRRSTAESLFGQRLAVHSDFLGEQLQPLLDLTRRPPQVMRLQQRVNSPGWRRAMAAR

KRIDDLIDAQIADARTAPRPDDHMLTTLISGCSEEGTTLSDNEIRDSIVSLITAGYETTS

GALAWAIYALLTVPGTWESAASEVARVLGGRVPAADDLSALTYLNGVVHETLRLYSPGVI

SARRVLRDLWFDGHRIRAGRLLIFSAYVTHRLPEIWPEPTEFRPLRWDPNAADYRKPAPH

EFIPFSGGLHRCIGAVMATTEMTVILARLVARAMLQLPAQRTHRIRAANFAALRPWPGLT

VEIRKSAPAQ

>CYP139A1(2574635031)Mycobacterium tuberculosis TBR31

MRYPLGEALLALYRWRGPLINAGVGGHGYTYLLGAEANRFVFANADAFSWSQTFESLVPV

DGPTALIVSDGADHRRRRSVVAPGLRHHHVQRYVATMVSNIDTVIDGWQPGQRLDIYQEL

RSAVRRSTAESLFGQRLAVHSDFLGEQLQPLLDLTRRPPQVMRLQQRVNSPGWRRAMAAR

KRIDDLIDAQIADARTAPRPDDHMLTTLISGCSEEGTTLSDNEIRDSIVSLITAGYETTS

GALAWAIYALLTVPGTWESAASEVARVLGGRVPAADDLSALTYLNGVVHETLRLYSPGVI

SARRVLRDLWFDGHRIRAGRLLIFSAYVTHRLPEIWPEPTEFRPLRWDPNAADYRKPAPH

EFIPFSGGLHRCIGAVMATTEMTVILARLVARAMLQLPAQRTHRIRAANFAALRPWPGLT

VEIRKSAPAQ

>CYP139A1(2576207497)Mycobacterium tuberculosis XTB13-096

MRYPLGEALLALYRWRGPLINAGVGGHGYTYLLGAEANRFVFANADAFSWSQTFESLVPV

DGPTALIVSDGADHRRRRSVVAPGLRHHHVQRYVATMVSNIDTVIDGWQPGQRLDIYQEL

RSAVRRSTAESLFGQRLAVHSDFLGEQLQPLLDLTRRPPQVMRLQQRVNSPGWRRAMAAR

KRIDDLIDAQIADARTAPRPDDHMLTTLISGCSEEGTTLSDNEIRDSIVSLITAGYETTS

GALAWAIYALLTVPGTWESAASEVARVLGGRVPAADDLSALTYLNGVVHETLRLYSPGVI

SARRVLRDLWFDGHRIRAGRLLIFSAYVTHRLPEIWPEPTEFRPLRWDPNAADYRKPAPH

EFIPFSGGLHRCIGAVMATTEMTVILARLVARAMLQLPAQRTHRIRAANFAALRPWPGLT

VEIRKSAPAQ

>CYP139A1(2576443603)Mycobacterium tuberculosis TB_RSA136

MRYPLGEALLALYRWRGPLINAGVGGHGYTYLLGAEANRFVFANADAFSWSQTFESLVPV

DGPTALIVSDGADHRRRRSVVAPGLRHHHVQRYVATMVSNIDTVIDGWQPGQRLDIYQEL

RSAVRRSTAESLFGQRLAVHSDFLGEQLQPLLDLTRRPPQVMRLQQRVNSPGWRRAMAAR

KRIDDLIDAQIADARTAPRPDDHMLTTLISGCSEEGTTLSDNEIRDSIVSLITAGYETTS

GALAWAIYALLTVPGTWESAASEVARVLGGRVPAADDLSALTYLNGVVHETLRLYSPGVI

SARRVLRDLWFDGHRIRAGRLLIFSAYVTHRLPEIWPEPTEFRPLRWDPNAADYRKPAPH

EFIPFSGGLHRCIGAVMATTEMTVILARLVARAMLQLPAQRTHRIRAANFAALRPWPGLT

VEIRKSAPAQ

>CYP139A1(2577241442)Mycobacterium tuberculosis TKK-01-0006

MRYPLGEALLALYRWRGPLINAGVGGHGYTYLLGAEANRFVFANADAFSWSQTFESLVPV

DGPTALIVSDGADHRRRRSVVAPGLRHHHVQRYVATMVSNIDTVIDGWQPGQRLDIYQEL

RSAVRRSTAESLFGQRLAVHSDFLGEQLQPLLDLTRRPPQVMRLQQRVNSPGWRRAMAAR

KRIDDLIDAQIADARTAPRPDDHMLTTLISGCSEEGTTLSDNEIRDSIVSLITAGYETTS

GALAWAIYALLTVPGTWESAASEVARVLGGRVPAADDLSALTYLNGVVHETLRLYSPGVI

SARRVLRDLWFDGHRIRAGRLLIFSAYVTHRLPEIWPEPTEFRPLRWDPNAADYRKPAPH

EFIPFSGGLHRCIGAVMATTEMTVILARLVARAMLQLPAQRTHRIRAANFAALRPWPGLT

VEIRKSAPAQ

>CYP139A1(2578177805)Mycobacterium tuberculosis TKK_04_0033

MRYPLGEALLALYRWRGPLINAGVGGHGYTYLLGAEANRFVFANADAFSWSQTFESLVPV

DGPTALIVSDGADHRRRRSVVAPGLRHHHVQRYVATMVSNIDTVIDGWQPGQRLDIYQEL

RSAVRRSTAESLFGQRLAVHSDFLGEQLQPLLDLTRRPPQVMRLQQRVNSPGWRRAMAAR

KRIDDLIDAQIADARTAPRPDDHMLTTLISGCSEEGTTLSDNEIRDSIVSLITAGYETTS

GALAWAIYALLTVPGTWESAASEVARVLGGRVPAADDLSALTYLNGVVHETLRLYSPGVI

SARRVLRDLWFDGHRIRAGRLLIFSAYVTHRLPEIWPEPTEFRPLRWDPNAADYRKPAPH

EFIPFSGGLHRCIGAVMATTEMTVILARLVARAMLQLPAQRTHRIRAANFAALRPWPGLT

VEIRKSAPAQ

>CYP139A1(2578195506)Mycobacterium tuberculosis TKK_03_0096

MRYPLGEALLALYRWRGPLINAGVGGHGYTYLLGAEANRFVFANADAFSWSQTFESLVPV

DGPTALIVSDGADHRRRRSVVAPGLRHHHVQRYVATMVSNIDTVIDGWQPGQRLDIYQEL

RSAVRRSTAESLFGQRLAVHSDFLGEQLQPLLDLTRRPPQVMRLQQRVNSPGWRRAMAAR

KRIDDLIDAQIADARTAPRPDDHMLTTLISGCSEEGTTLSDNEIRDSIVSLITAGYETTS

GALAWAIYALLTVPGTWESAASEVARVLGGRVPAADDLSALTYLNGVVHETLRLYSPGVI

SARRVLRDLWFDGHRIRAGRLLIFSAYVTHRLPEIWPEPTEFRPLRWDPNAADYRKPAPH

EFIPFSGGLHRCIGAVMATTEMTVILARLVARAMLQLPAQRTHRIRAANFAALRPWPGLT

VEIRKSAPAQ

>CYP139A1(2578246558)Mycobacterium tuberculosis TKK_03_0094

MRYPLGEALLALYRWRGPLINAGVGGHGYTYLLGAEANRFVFANADAFSWSQTFESLVPV

DGPTALIVSDGADHRRRRSVVAPGLRHHHVQRYVATMVSNIDTVIDGWQPGQRLDIYQEL

RSAVRRSTAESLFGQRLAVHSDFLGEQLQPLLDLTRRPPQVMRLQQRVNSPGWRRAMAAR

KRIDDLIDAQIADARTAPRPDDHMLTTLISGCSEEGTTLSDNEIRDSIVSLITAGYETTS

GALAWAIYALLTVPGTWESAASEVARVLGGRVPAADDLSALTYLNGVVHETLRLYSPGVI

SARRVLRDLWFDGHRIRAGRLLIFSAYVTHRLPEIWPEPTEFRPLRWDPNAADYRKPAPH

EFIPFSGGLHRCIGAVMATTEMTVILARLVARAMLQLPAQRTHRIRAANFAALRPWPGLT

VEIRKSAPAQ

>CYP139A1(2584652546)Mycobacterium tuberculosis SK-E

MRYPLGEALLALYRWRGPLINAGVGGHGYTYLLGAEANRFVFANADAFSWSQTFESLVPV

DGPTALIVSDGADHRRRRSVVAPGLRHHHVQRYVATMVSNIDTVIDGWQPGQRLDIYQEL

RSAVRRSTAESLFGQRLAVHSDFLGEQLQPLLDLTRRPPQVMRLQQRVNSPGWRRAMAAR

KRIDDLIDAQIADARTAPRPDDHMLTTLISGCSEEGTTLSDNEIRDSIVSLITAGYETTS

GALAWAIYALLTVPGTWESAASEVARVLGGRVPAADDLSALTYLNGVVHETLRLYSPGVI

SARRVLRDLWFDGHRIRAGRLLIFSAYVTHRLPEIWPEPTEFRPLRWDPNAADYRKPAPH

EFIPFSGGLHRCIGAVMATTEMTVILARLVARAMLQLPAQRTHRIRAANFAALRPWPGLT

VEIRKSAPAQ

>CYP139A1(2584737038)Mycobacterium tuberculosis TB_RSA25

MRYPLGEALLALYRWRGPLINAGVGGHGYTYLLGAEANRFVFANADAFSWSQTFESLVPV

DGPTALIVSDGADHRRRRSVVAPGLRHHHVQRYVATMVSNIDTVIDGWQPGQRLDIYQEL

RSAVRRSTAESLFGQRLAVHSDFLGEQLQPLLDLTRRPPQVMRLQQRVNSPGWRRAMAAR

KRIDDLIDAQIADARTAPRPDDHMLTTLISGCSEEGTTLSDNEIRDSIVSLITAGYETTS

GALAWAIYALLTVPGTWESAASEVARVLGGRVPAADDLSALTYLNGVVHETLRLYSPGVI

SARRVLRDLWFDGHRIRAGRLLIFSAYVTHRLPEIWPEPTEFRPLRWDPNAADYRKPAPH

EFIPFSGGLHRCIGAVMATTEMTVILARLVARAMLQLPAQRTHRIRAANFAALRPWPGLT

VEIRKSAPAQ

>CYP139A1(2584918375)Mycobacterium tuberculosis XTB13-100

MRYPLGEALLALYRWRGPLINAGVGGHGYTYLLGAEANRFVFANADAFSWSQTFESLVPV

DGPTALIVSDGADHRRRRSVVAPGLRHHHVQRYVATMVSNIDTVIDGWQPGQRLDIYQEL

RSAVRRSTAESLFGQRLAVHSDFLGEQLQPLLDLTRRPPQVMRLQQRVNSPGWRRAMAAR

KRIDDLIDAQIADARTAPRPDDHMLTTLISGCSEEGTTLSDNEIRDSIVSLITAGYETTS

GALAWAIYALLTVPGTWESAASEVARVLGGRVPAADDLSALTYLNGVVHETLRLYSPGVI

SARRVLRDLWFDGHRIRAGRLLIFSAYVTHRLPEIWPEPTEFRPLRWDPNAADYRKPAPH

EFIPFSGGLHRCIGAVMATTEMTVILARLVARAMLQLPAQRTHRIRAANFAALRPWPGLT

VEIRKSAPAQ

>CYP139A1(2584974532)Mycobacterium tuberculosis TKK_04_0094

MRYPLGEALLALYRWRGPLINAGVGGHGYTYLLGAEANRFVFANADAFSWSQTFESLVPV

DGPTALIVSDGADHRRRRSVVAPGLRHHHVQRYVATMVSNIDTVIDGWQPGQRLDIYQEL

RSAVRRSTAESLFGQRLAVHSDFLGEQLQPLLDLTRRPPQVMRLQQRVNSPGWRRAMAAR

KRIDDLIDAQIADARTAPRPDDHMLTTLISGCSEEGTTLSDNEIRDSIVSLITAGYETTS

GALAWAIYALLTVPGTWESAASEVARVLGGRVPAADDLSALTYLNGVVHETLRLYSPGVI

SARRVLRDLWFDGHRIRAGRLLIFSAYVTHRLPEIWPEPTEFRPLRWDPNAADYRKPAPH

EFIPFSGGLHRCIGAVMATTEMTVILARLVARAMLQLPAQRTHRIRAANFAALRPWPGLT

VEIRKSAPAQ

>CYP139A1(2588987052)Mycobacterium tuberculosis TKK-01-0020

MRYPLGEALLALYRWRGPLINAGVGGHGYTYLLGAEANRFVFANADAFSWSQTFESLVPV

DGPTALIVSDGADHRRRRSVVAPGLRHHHVQRYVATMVSNIDTVIDGWQPGQRLDIYQEL

RSAVRRSTAESLFGQRLAVHSDFLGEQLQPLLDLTRRPPQVMRLQQRVNSPGWRRAMAAR

KRIDDLIDAQIADARTAPRPDDHMLTTLISGCSEEGTTLSDNEIRDSIVSLITAGYETTS

GALAWAIYALLTVPGTWESAASEVARVLGGRVPAADDLSALTYLNGVVHETLRLYSPGVI

SARRVLRDLWFDGHRIRAGRLLIFSAYVTHRLPEIWPEPTEFRPLRWDPNAADYRKPAPH

EFIPFSGGLHRCIGAVMATTEMTVILARLVARAMLQLPAQRTHRIRAANFAALRPWPGLT

VEIRKSAPAQ

>CYP139A1(2589098383)Mycobacterium tuberculosis TBR57

MRYPLGEALLALYRWRGPLINAGVGGHGYTYLLGAEANRFVFANADAFSWSQTFESLVPV

DGPTALIVSDGADHRRRRSVVAPGLRHHHVQRYVATMVSNIDTVIDGWQPGQRLDIYQEL

RSAVRRSTAESLFGQRLAVHSDFLGEQLQPLLDLTRRPPQVMRLQQRVNSPGWRRAMAAR

KRIDDLIDAQIADARTAPRPDDHMLTTLISGCSEEGTTLSDNEIRDSIVSLITAGYETTS

GALAWAIYALLTVPGTWESAASEVARVLGGRVPAADDLSALTYLNGVVHETLRLYSPGVI

SARRVLRDLWFDGHRIRAGRLLIFSAYVTHRLPEIWPEPTEFRPLRWDPNAADYRKPAPH

EFIPFSGGLHRCIGAVMATTEMTVILARLVARAMLQLPAQRTHRIRAANFAALRPWPGLT

VEIRKSAPAQ

>CYP139A1(2589486069)Mycobacterium tuberculosis TKK-01-0002

MRYPLGEALLALYRWRGPLINAGVGGHGYTYLLGAEANRFVFANADAFSWSQTFESLVPV

DGPTALIVSDGADHRRRRSVVAPGLRHHHVQRYVATMVSNIDTVIDGWQPGQRLDIYQEL

RSAVRRSTAESLFGQRLAVHSDFLGEQLQPLLDLTRRPPQVMRLQQRVNSPGWRRAMAAR

KRIDDLIDAQIADARTAPRPDDHMLTTLISGCSEEGTTLSDNEIRDSIVSLITAGYETTS

GALAWAIYALLTVPGTWESAASEVARVLGGRVPAADDLSALTYLNGVVHETLRLYSPGVI

SARRVLRDLWFDGHRIRAGRLLIFSAYVTHRLPEIWPEPTEFRPLRWDPNAADYRKPAPH

EFIPFSGGLHRCIGAVMATTEMTVILARLVARAMLQLPAQRTHRIRAANFAALRPWPGLT

VEIRKSAPAQ

>CYP139A1(2589522796)Mycobacterium tuberculosis TKK-01-0014

MRYPLGEALLALYRWRGPLINAGVGGHGYTYLLGAEANRFVFANADAFSWSQTFESLVPV

DGPTALIVSDGADHRRRRSVVAPGLRHHHVQRYVATMVSNIDTVIDGWQPGQRLDIYQEL

RSAVRRSTAESLFGQRLAVHSDFLGEQLQPLLDLTRRPPQVMRLQQRVNSPGWRRAMAAR

KRIDDLIDAQIADARTAPRPDDHMLTTLISGCSEEGTTLSDNEIRDSIVSLITAGYETTS

GALAWAIYALLTVPGTWESAASEVARVLGGRVPAADDLSALTYLNGVVHETLRLYSPGVI

SARRVLRDLWFDGHRIRAGRLLIFSAYVTHRLPEIWPEPTEFRPLRWDPNAADYRKPAPH

EFIPFSGGLHRCIGAVMATTEMTVILARLVARAMLQLPAQRTHRIRAANFAALRPWPGLT

VEIRKSAPAQ

>CYP139A1(2589616566)Mycobacterium tuberculosis TKK-01-0046

MRYPLGEALLALYRWRGPLINAGVGGHGYTYLLGAEANRFVFANADAFSWSQTFESLVPV

DGPTALIVSDGADHRRRRSVVAPGLRHHHVQRYVATMVSNIDTVIDGWQPGQRLDIYQEL

RSAVRRSTAESLFGQRLAVHSDFLGEQLQPLLDLTRRPPQVMRLQQRVNSPGWRRAMAAR

KRIDDLIDAQIADARTAPRPDDHMLTTLISGCSEEGTTLSDNEIRDSIVSLITAGYETTS

GALAWAIYALLTVPGTWESAASEVARVLGGRVPAADDLSALTYLNGVVHETLRLYSPGVI

SARRVLRDLWFDGHRIRAGRLLIFSAYVTHRLPEIWPEPTEFRPLRWDPNAADYRKPAPH

EFIPFSGGLHRCIGAVMATTEMTVILARLVARAMLQLPAQRTHRIRAANFAALRPWPGLT

VEIRKSAPAQ

>CYP139A1(2592250930)Mycobacterium tuberculosis TKK_04_0013

MRYPLGEALLALYRWRGPLINAGVGGHGYTYLLGAEANRFVFANADAFSWSQTFESLVPV

DGPTALIVSDGADHRRRRSVVAPGLRHHHVQRYVATMVSNIDTVIDGWQPGQRLDIYQEL

RSAVRRSTAESLFGQRLAVHSDFLGEQLQPLLDLTRRPPQVMRLQQRVNSPGWRRAMAAR

KRIDDLIDAQIADARTAPRPDDHMLTTLISGCSEEGTTLSDNEIRDSIVSLITAGYETTS

GALAWAIYALLTVPGTWESAASEVARVLGGRVPAADDLSALTYLNGVVHETLRLYSPGVI

SARRVLRDLWFDGHRIRAGRLLIFSAYVTHRLPEIWPEPTEFRPLRWDPNAADYRKPAPH

EFIPFSGGLHRCIGAVMATTEMTVILARLVARAMLQLPAQRTHRIRAANFAALRPWPGLT

VEIRKSAPAQ

>CYP139A1(2592303254)Mycobacterium tuberculosis TKK_03_0030

MRYPLGEALLALYRWRGPLINAGVGGHGYTYLLGAEANRFVFANADAFSWSQTFESLVPV

DGPTALIVSDGADHRRRRSVVAPGLRHHHVQRYVATMVSNIDTVIDGWQPGQRLDIYQEL

RSAVRRSTAESLFGQRLAVHSDFLGEQLQPLLDLTRRPPQVMRLQQRVNSPGWRRAMAAR

KRIDDLIDAQIADARTAPRPDDHMLTTLISGCSEEGTTLSDNEIRDSIVSLITAGYETTS

GALAWAIYALLTVPGTWESAASEVARVLGGRVPAADDLSALTYLNGVVHETLRLYSPGVI

SARRVLRDLWFDGHRIRAGRLLIFSAYVTHRLPEIWPEPTEFRPLRWDPNAADYRKPAPH

EFIPFSGGLHRCIGAVMATTEMTVILARLVARAMLQLPAQRTHRIRAANFAALRPWPGLT

VEIRKSAPAQ

>CYP139A1(2592308116)Mycobacterium tuberculosis TKK_03_0029

MRYPLGEALLALYRWRGPLINAGVGGHGYTYLLGAEANRFVFANADAFSWSQTFESLVPV

DGPTALIVSDGADHRRRRSVVAPGLRHHHVQRYVATMVSNIDTVIDGWQPGQRLDIYQEL

RSAVRRSTAESLFGQRLAVHSDFLGEQLQPLLDLTRRPPQVMRLQQRVNSPGWRRAMAAR

KRIDDLIDAQIADARTAPRPDDHMLTTLISGCSEEGTTLSDNEIRDSIVSLITAGYETTS

GALAWAIYALLTVPGTWESAASEVARVLGGRVPAADDLSALTYLNGVVHETLRLYSPGVI

SARRVLRDLWFDGHRIRAGRLLIFSAYVTHRLPEIWPEPTEFRPLRWDPNAADYRKPAPH

EFIPFSGGLHRCIGAVMATTEMTVILARLVARAMLQLPAQRTHRIRAANFAALRPWPGLT

VEIRKSAPAQ

>CYP139A1(2592385592)Mycobacterium tuberculosis TKK_02_0034

MRYPLGEALLALYRWRGPLINAGVGGHGYTYLLGAEANRFVFANADAFSWSQTFESLVPV

DGPTALIVSDGADHRRRRSVVAPGLRHHHVQRYVATMVSNIDTVIDGWQPGQRLDIYQEL

RSAVRRSTAESLFGQRLAVHSDFLGEQLQPLLDLTRRPPQVMRLQQRVNSPGWRRAMAAR

KRIDDLIDAQIADARTAPRPDDHMLTTLISGCSEEGTTLSDNEIRDSIVSLITAGYETTS

GALAWAIYALLTVPGTWESAASEVARVLGGRVPAADDLSALTYLNGVVHETLRLYSPGVI

SARRVLRDLWFDGHRIRAGRLLIFSAYVTHRLPEIWPEPTEFRPLRWDPNAADYRKPAPH

EFIPFSGGLHRCIGAVMATTEMTVILARLVARAMLQLPAQRTHRIRAANFAALRPWPGLT

VEIRKSAPAQ

>CYP139A1(2592417392)Mycobacterium tuberculosis TKK_02_0017

MRYPLGEALLALYRWRGPLINAGVGGHGYTYLLGAEANRFVFANADAFSWSQTFESLVPV

DGPTALIVSDGADHRRRRSVVAPGLRHHHVQRYVATMVSNIDTVIDGWQPGQRLDIYQEL

RSAVRRSTAESLFGQRLAVHSDFLGEQLQPLLDLTRRPPQVMRLQQRVNSPGWRRAMAAR

KRIDDLIDAQIADARTAPRPDDHMLTTLISGCSEEGTTLSDNEIRDSIVSLITAGYETTS

GALAWAIYALLTVPGTWESAASEVARVLGGRVPAADDLSALTYLNGVVHETLRLYSPGVI

SARRVLRDLWFDGHRIRAGRLLIFSAYVTHRLPEIWPEPTEFRPLRWDPNAADYRKPAPH

EFIPFSGGLHRCIGAVMATTEMTVILARLVARAMLQLPAQRTHRIRAANFAALRPWPGLT

VEIRKSAPAQ

>CYP139A1(643049582)Mycobacterium tuberculosis T17

MRYPLGEALLALYRWRGPLINAGVGGHGYTYLLGAEANRFVFANADAFSWSQTFESLVPV

DGPTALIVSDGADHRRRRSVVAPGLRHHHVQRYVATMVSNIDTVIDGWQPGQRLDIYQEL

RSAVRRSTAESLFGQRLAVHSDFLGEQLQPLLDLTRRPPQVMRLQQRVNSPGWRRAMAAR

KRIDDLIDAQIADARTAPRPDDHMLTTLISGCSEEGTTLSDNEIRDSIVSLITAGYETTS

GALAWAIYALLTVPGTWESAASEVARVLGGRVPAADDLSALTYLNGVVHETLRLYSPGVI

SARRVLRDLWFDGHRIRAGRLLIFSAYVTHRLPEIWPEPTEFRPLRWDPNAADYRKPAPH

EFIPFSGGLHRCIGAVMATTEMTVILARLVARAMLQLPAQRTHRIRAANFAALRPWPGLT

VEIRKSAPAQ

>CYP139A1(648460473)Mycobacterium tuberculosis SUMu005

MRYPLGEALLALYRWRGPLINAGVGGHGYTYLLGAEANRFVFANADAFSWSQTFESLVPV

DGPTALIVSDGADHRRRRSVVAPGLRHHHVQRYVATMVSNIDTVIDGWQPGQRLDIYQEL

RSAVRRSTAESLFGQRLAVHSDFLGEQLQPLLDLTRRPPQVMRLQQRVNSPGWRRAMAAR

KRIDDLIDAQIADARTAPRPDDHMLTTLISGCSEEGTTLSDNEIRDSIVSLITAGYETTS

GALAWAIYALLTVPGTWESAASEVARVLGGRVPAADDLSALTYLNGVVHETLRLYSPGVI

SARRVLRDLWFDGHRIRAGRLLIFSAYVTHRLPEIWPEPTEFRPLRWDPNAADYRKPAPH

EFIPFSGGLHRCIGAVMATTEMTVILARLVARAMLQLPAQRTHRIRAANFAALRPWPGLT

VEIRKSAPAQ

>CYP139A1(2574901332)Mycobacterium tuberculosis M1762

MRYPLGEALLALYRWRGPLINAGVGGHGYTYLLGAEANRFVFANADAFSWSQTFESLVPV

DGPTALIVSDGADHRRRRSVVAPGLRHHHVQRYVATMVSNIDTVIDGWQPGQRLDIYQEL

RSAVRRSTAESLFGQRLAVHSDFLGEQLQPLLDLTRRPPQVMRLQQRVNSPGWRRAMAAR

KRIDDLIDAQIADARTAPRPDDHMLTTLISGCSEEGTTLSDNEIRDSIVSLITAGYETTS

GALAWAIYALLTVPGTWESAASEVARVLGGRVPAADDLSALTYLNGVVHETLRLYSPGVI

SARRVLRDLWFDGHRIRAGRLLIFSAYVTHRLPEIWPEPTEFRPLRWDPNAADYRKPAPH

EFIPFSGGLHRCIGAVMATTEMTVILARLVARAMLQLPAQRTHRIRAANFAALRPWPGLT

VEIRKSAPAQ

>CYP139A1(2575325434)Mycobacterium tuberculosis OFXR-6

MRYPLGEALLALYRWRGPLINAGVGGHGYTYLLGAEANRFVFANADAFSWSQTFESLVPV

DGPTALIVSDGADHRRRRSVVAPGLRHHHVQRYVATMVSNIDTVIDGWQPGQRLDIYQEL

RSAVRRSTAESLFGQRLAVHSDFLGEQLQPLLDLTRRPPQVMRLQQRVNSPGWRRAMAAR

KRIDDLIDAQIADARTAPRPDDHMLTTLISGCSEEGTTLSDNEIRDSIVSLITAGYETTS

GALAWAIYALLTVPGTWESAASEVARVLGGRVPAADDLSALTYLNGVVHETLRLYSPGVI

SARRVLRDLWFDGHRIRAGRLLIFSAYVTHRLPEIWPEPTEFRPLRWDPNAADYRKPAPH

EFIPFSGGLHRCIGAVMATTEMTVILARLVARAMLQLPAQRTHRIRAANFAALRPWPGLT

VEIRKSAPAQ

>CYP139A1(2576274603)Mycobacterium tuberculosis MD18096

MRYPLGEALLALYRWRGPLINAGVGGHGYTYLLGAEANRFVFANADAFSWSQTFESLVPV

DGPTALIVSDGADHRRRRSVVAPGLRHHHVQRYVATMVSNIDTVIDGWQPGQRLDIYQEL

RSAVRRSTAESLFGQRLAVHSDFLGEQLQPLLDLTRRPPQVMRLQQRVNSPGWRRAMAAR

KRIDDLIDAQIADARTAPRPDDHMLTTLISGCSEEGTTLSDNEIRDSIVSLITAGYETTS

GALAWAIYALLTVPGTWESAASEVARVLGGRVPAADDLSALTYLNGVVHETLRLYSPGVI

SARRVLRDLWFDGHRIRAGRLLIFSAYVTHRLPEIWPEPTEFRPLRWDPNAADYRKPAPH

EFIPFSGGLHRCIGAVMATTEMTVILARLVARAMLQLPAQRTHRIRAANFAALRPWPGLT

VEIRKSAPAQ

>CYP139A1(2583719805)*Mycobacterium africanum* MAL010070

MRYPLGEALLALYRWRGPLINAGVGGHGYTYLLGAEANRFVFANADAFSWSQTFESLVPV

DGPTALIVSDGADHRRRRSVVAPGLRHHHVQRYVATMVSNIDTVIDGWQPGQRLDIYQEL

RSAVRRSTAESLFGQRLAVHSDFLGEQLQPLLDLTRRPPQVMRLQQRVNSPGWRRAMAAR

KRIDDLIDAQIADARTAPRPDDHMLTTLISGCSEEGTTLSDNEIRDSIVSLITAGYETTS

GALAWAIYALLTVPGTWESAASEVARVLGGRVPAADDLSALTYLNGVVHETLRLYSPGVI

SARRVLRDLWFDGHRIRAGRLLIFSAYVTHRLPEIWPEPTEFRPLRWDPNAADYRKPAPH

EFIPFSGGLHRCIGAVMATTEMTVILARLVARAMLQLPAQRTHRIRAANFAALRPWPGLT

VEIRKSAPAQ

>CYP139A1(2590012031)Mycobacterium tuberculosis MAL010086

MRYPLGEALLALYRWRGPLINAGVGGHGYTYLLGAEANRFVFANADAFSWSQTFESLVPV

DGPTALIVSDGADHRRRRSVVAPGLRHHHVQRYVATMVSNIDTVIDGWQPGQRLDIYQEL

RSAVRRSTAESLFGQRLAVHSDFLGEQLQPLLDLTRRPPQVMRLQQRVNSPGWRRAMAAR

KRIDDLIDAQIADARTAPRPDDHMLTTLISGCSEEGTTLSDNEIRDSIVSLITAGYETTS

GALAWAIYALLTVPGTWESAASEVARVLGGRVPAADDLSALTYLNGVVHETLRLYSPGVI

SARRVLRDLWFDGHRIRAGRLLIFSAYVTHRLPEIWPEPTEFRPLRWDPNAADYRKPAPH

EFIPFSGGLHRCIGAVMATTEMTVILARLVARAMLQLPAQRTHRIRAANFAALRPWPGLT

VEIRKSAPAQ

>CYP139A(2590028529)Mycobacterium tuberculosis MAL010110

MRYPLGEALLALYRWRGPLINAGVGGHGYTYLLGAEANRFVFANADAFSWSQTFESLVPV

DGPTALIVSDGADHRRRRSVVAPGLRHHHVQRYVATMVSNIDTVIDGWQPGQRLDIYQEL

RSAVRRSTAESLFGQRLAVHSDFLGEQLQPLLDLTRRPPQVMRLQQRVNSPGWRRAMAAR

KRIDDLIDAQIADARTAPRPDDHMLTTLISGCSEEGTTLSDNEIRDSIVSLITAGYETTS

GALAWAIYALLTVPGTWESAASEVARVLGGRVPAADDLSALTYLNGVVHETLRLYSPGVI

SARRVLRDLWFDGHRIRAGRLLIFSAYVTHRLPEIWPEPTEFRPLRWDPNAADYRKPAPH

EFIPFSGGLHRCIGAVMATTEMTVILARLVARAMLQLPAQRTHRIRAANFAALRPWPGLT

VEIRKSAPAQ

>CYP139A1(2590146409)Mycobacterium tuberculosis MAL020193

MRYPLGEALLALYRWRGPLINAGVGGHGYTYLLGAEANRFVFANADAFSWSQTFESLVPV

DGPTALIVSDGADHRRRRSVVAPGLRHHHVQRYVATMVSNIDTVIDGWQPGQRLDIYQEL

RSAVRRSTAESLFGQRLAVHSDFLGEQLQPLLDLTRRPPQVMRLQQRVNSPGWRRAMAAR

KRIDDLIDAQIADARTAPRPDDHMLTTLISGCSEEGTTLSDNEIRDSIVSLITAGYETTS

GALAWAIYALLTVPGTWESAASEVARVLGGRVPAADDLSALTYLNGVVHETLRLYSPGVI

SARRVLRDLWFDGHRIRAGRLLIFSAYVTHRLPEIWPEPTEFRPLRWDPNAADYRKPAPH

EFIPFSGGLHRCIGAVMATTEMTVILARLVARAMLQLPAQRTHRIRAANFAALRPWPGLT

VEIRKSAPAQ

>CYP139A1(2590203420)Mycobacterium tuberculosis OFXR-9

MRYPLGEALLALYRWRGPLINAGVGGHGYTYLLGAEANRFVFANADAFSWSQTFESLVPV

DGPTALIVSDGADHRRRRSVVAPGLRHHHVQRYVATMVSNIDTVIDGWQPGQRLDIYQEL

RSAVRRSTAESLFGQRLAVHSDFLGEQLQPLLDLTRRPPQVMRLQQRVNSPGWRRAMAAR

KRIDDLIDAQIADARTAPRPDDHMLTTLISGCSEEGTTLSDNEIRDSIVSLITAGYETTS

GALAWAIYALLTVPGTWESAASEVARVLGGRVPAADDLSALTYLNGVVHETLRLYSPGVI

SARRVLRDLWFDGHRIRAGRLLIFSAYVTHRLPEIWPEPTEFRPLRWDPNAADYRKPAPH

EFIPFSGGLHRCIGAVMATTEMTVILARLVARAMLQLPAQRTHRIRAANFAALRPWPGLT

VEIRKSAPAQ

>CYP139A1(2590272661)Mycobacterium tuberculosis KT-0015

MRYPLGEALLALYRWRGPLINAGVGGHGYTYLLGAEANRFVFANADAFSWSQTFESLVPV

DGPTALIVSDGADHRRRRSVVAPGLRHHHVQRYVATMVSNIDTVIDGWQPGQRLDIYQEL

RSAVRRSTAESLFGQRLAVHSDFLGEQLQPLLDLTRRPPQVMRLQQRVNSPGWRRAMAAR

KRIDDLIDAQIADARTAPRPDDHMLTTLISGCSEEGTTLSDNEIRDSIVSLITAGYETTS

GALAWAIYALLTVPGTWESAASEVARVLGGRVPAADDLSALTYLNGVVHETLRLYSPGVI

SARRVLRDLWFDGHRIRAGRLLIFSAYVTHRLPEIWPEPTEFRPLRWDPNAADYRKPAPH

EFIPFSGGLHRCIGAVMATTEMTVILARLVARAMLQLPAQRTHRIRAANFAALRPWPGLT

VEIRKSAPAQ

>CYP139A1(2590535240)Mycobacterium tuberculosis KT-0083

MRYPLGEALLALYRWRGPLINAGVGGHGYTYLLGAEANRFVFANADAFSWSQTFESLVPV

DGPTALIVSDGADHRRRRSVVAPGLRHHHVQRYVATMVSNIDTVIDGWQPGQRLDIYQEL

RSAVRRSTAESLFGQRLAVHSDFLGEQLQPLLDLTRRPPQVMRLQQRVNSPGWRRAMAAR

KRIDDLIDAQIADARTAPRPDDHMLTTLISGCSEEGTTLSDNEIRDSIVSLITAGYETTS

GALAWAIYALLTVPGTWESAASEVARVLGGRVPAADDLSALTYLNGVVHETLRLYSPGVI

SARRVLRDLWFDGHRIRAGRLLIFSAYVTHRLPEIWPEPTEFRPLRWDPNAADYRKPAPH

EFIPFSGGLHRCIGAVMATTEMTVILARLVARAMLQLPAQRTHRIRAANFAALRPWPGLT

VEIRKSAPAQ

>CYP139A1(647090515)Mycobacterium tuberculosis KZN V2475

MRYPLGEALLALYRWRGPLINAGVGGHGYTYLLGAEANRFVFANADAFSWSQTFESLVPV

DGPTALIVSDGADHRRRRSVVAPGLRHHHVQRYVATMVSNIDTVIDGWQPGQRLDIYQEL

RSAVRRSTAESLFGQRLAVHSDFLGEQLQPLLDLTRRPPQVMRLQQRVNSPGWRRAMAAR

KRIDDLIDAQIADARTAPRPDDHMLTTLISGCSEEGTTLSDNEIRDSIVSLITAGYETTS

GALAWAIYALLTVPGTWESAASEVARVLGGRVPAADDLSALTYLNGVVHETLRLYSPGVI

SARRVLRDLWFDGHRIRAGRLLIFSAYVTHRLPEIWPEPTEFRPLRWDPNAADYRKPAPH

EFIPFSGGLHRCIGAVMATTEMTVILARLVARAMLQLPAQRTHRIRAANFAALRPWPGLT

VEIRKSAPAQ

>CYP139A1(2512786915)Mycobacterium tuberculosis UT205

MRYPLGEALLALYRWRGPLINAGVGGHGYTYLLGAEANRFVFANADAFSWSQTFESLVPV

DGPTALIVSDGADHRRRRSVVAPGLRHHHVQRYVATMVSNIDTVIDGWQPGQRLDIYQEL

RSAVRRSTAESLFGQRLAVHSDFLGEQLQPLLDLTRRPPQVMRLQQRVNSPGWRRAMAAR

KRIDDLIDAQIADARTAPRPDDHMLTTLISGCSEEGTTLSDNEIRDSIVSLITAGYETTS

GALAWAIYALLTVPGTWESAASEVARVLGGRVPAADDLSALTYLNGVVHETLRLYSPGVI

SARRVLRDLWFDGHRIRAGRLLIFSAYVTHRLPEIWPEPTEFRPLRWDPNAADYRKPAPH

EFIPFSGGLHRCIGAVMATTEMTVILARLVARAMLQLPAQRTHRIRAANFAALRPWPGLT

VEIRKSAPAQ

>CYP139A1(2575329342)Mycobacterium tuberculosis TKK_02_0019

MRYPLGEALLALYRWRGPLINAGVGGHGYTYLLGAEANRFVFANADAFSWSQTFESLVPV

DGPTALIVSDGADHRRRRSVVAPGLRHHHVQRYVATMVSNIDTVIDGWQPGQRLDIYQEL

RSAVRRSTAESLFGQRLAVHSDFLGEQLQPLLDLTRRPPQVMRLQQRVNSPGWRRAMAAR

KRIDDLIDAQIADARTAPRPDDHMLTTLISGCSEEGTTLSDNEIRDSIVSLITAGYETTS

GALAWAIYALLTVPGTWESAASEVARVLGGRVPAADDLSALTYLNGVVHETLRLYSPGVI

SARRVLRDLWFDGHRIRAGRLLIFSAYVTHRLPEIWPEPTEFRPLRWDPNAADYRKPAPH

EFIPFSGGLHRCIGAVMATTEMTVILARLVARAMLQLPAQRTHRIRAANFAALRPWPGLT

VEIRKSAPAQ

>CYP139A1(2575409751)Mycobacterium tuberculosis TB_RSA165

MRYPLGEALLALYRWRGPLINAGVGGHGYTYLLGAEANRFVFANADAFSWSQTFESLVPV

DGPTALIVSDGADHRRRRSVVAPGLRHHHVQRYVATMVSNIDTVIDGWQPGQRLDIYQEL

RSAVRRSTAESLFGQRLAVHSDFLGEQLQPLLDLTRRPPQVMRLQQRVNSPGWRRAMAAR

KRIDDLIDAQIADARTAPRPDDHMLTTLISGCSEEGTTLSDNEIRDSIVSLITAGYETTS

GALAWAIYALLTVPGTWESAASEVARVLGGRVPAADDLSALTYLNGVVHETLRLYSPGVI

SARRVLRDLWFDGHRIRAGRLLIFSAYVTHRLPEIWPEPTEFRPLRWDPNAADYRKPAPH

EFIPFSGGLHRCIGAVMATTEMTVILARLVARAMLQLPAQRTHRIRAANFAALRPWPGLT

VEIRKSAPAQ

>CYP139A1(2575862603)Mycobacterium tuberculosis TB_RSA102

MRYPLGEALLALYRWRGPLINAGVGGHGYTYLLGAEANRFVFANADAFSWSQTFESLVPV

DGPTALIVSDGADHRRRRSVVAPGLRHHHVQRYVATMVSNIDTVIDGWQPGQRLDIYQEL

RSAVRRSTAESLFGQRLAVHSDFLGEQLQPLLDLTRRPPQVMRLQQRVNSPGWRRAMAAR

KRIDDLIDAQIADARTAPRPDDHMLTTLISGCSEEGTTLSDNEIRDSIVSLITAGYETTS

GALAWAIYALLTVPGTWESAASEVARVLGGRVPAADDLSALTYLNGVVHETLRLYSPGVI

SARRVLRDLWFDGHRIRAGRLLIFSAYVTHRLPEIWPEPTEFRPLRWDPNAADYRKPAPH

EFIPFSGGLHRCIGAVMATTEMTVILARLVARAMLQLPAQRTHRIRAANFAALRPWPGLT

VEIRKSAPAQ

>CYP139A1(2576589126)Mycobacterium tuberculosis TB_RSA18

MRYPLGEALLALYRWRGPLINAGVGGHGYTYLLGAEANRFVFANADAFSWSQTFESLVPV

DGPTALIVSDGADHRRRRSVVAPGLRHHHVQRYVATMVSNIDTVIDGWQPGQRLDIYQEL

RSAVRRSTAESLFGQRLAVHSDFLGEQLQPLLDLTRRPPQVMRLQQRVNSPGWRRAMAAR

KRIDDLIDAQIADARTAPRPDDHMLTTLISGCSEEGTTLSDNEIRDSIVSLITAGYETTS

GALAWAIYALLTVPGTWESAASEVARVLGGRVPAADDLSALTYLNGVVHETLRLYSPGVI

SARRVLRDLWFDGHRIRAGRLLIFSAYVTHRLPEIWPEPTEFRPLRWDPNAADYRKPAPH

EFIPFSGGLHRCIGAVMATTEMTVILARLVARAMLQLPAQRTHRIRAANFAALRPWPGLT

VEIRKSAPAQ

>CYP139A1(2576649037)Mycobacterium tuberculosis XTB13-156

MRYPLGEALLALYRWRGPLINAGVGGHGYTYLLGAEANRFVFANADAFSWSQTFESLVPV

DGPTALIVSDGADHRRRRSVVAPGLRHHHVQRYVATMVSNIDTVIDGWQPGQRLDIYQEL

RSAVRRSTAESLFGQRLAVHSDFLGEQLQPLLDLTRRPPQVMRLQQRVNSPGWRRAMAAR

KRIDDLIDAQIADARTAPRPDDHMLTTLISGCSEEGTTLSDNEIRDSIVSLITAGYETTS

GALAWAIYALLTVPGTWESAASEVARVLGGRVPAADDLSALTYLNGVVHETLRLYSPGVI

SARRVLRDLWFDGHRIRAGRLLIFSAYVTHRLPEIWPEPTEFRPLRWDPNAADYRKPAPH

EFIPFSGGLHRCIGAVMATTEMTVILARLVARAMLQLPAQRTHRIRAANFAALRPWPGLT

VEIRKSAPAQ

>CYP139A1(2576746511)Mycobacterium tuberculosis XTB13-136

MRYPLGEALLALYRWRGPLINAGVGGHGYTYLLGAEANRFVFANADAFSWSQTFESLVPV

DGPTALIVSDGADHRRRRSVVAPGLRHHHVQRYVATMVSNIDTVIDGWQPGQRLDIYQEL

RSAVRRSTAESLFGQRLAVHSDFLGEQLQPLLDLTRRPPQVMRLQQRVNSPGWRRAMAAR

KRIDDLIDAQIADARTAPRPDDHMLTTLISGCSEEGTTLSDNEIRDSIVSLITAGYETTS

GALAWAIYALLTVPGTWESAASEVARVLGGRVPAADDLSALTYLNGVVHETLRLYSPGVI

SARRVLRDLWFDGHRIRAGRLLIFSAYVTHRLPEIWPEPTEFRPLRWDPNAADYRKPAPH

EFIPFSGGLHRCIGAVMATTEMTVILARLVARAMLQLPAQRTHRIRAANFAALRPWPGLT

VEIRKSAPAQ

>CYP139A1(2577129296)Mycobacterium tuberculosis TKK_05MA_0052

MRYPLGEALLALYRWRGPLINAGVGGHGYTYLLGAEANRFVFANADAFSWSQTFESLVPV

DGPTALIVSDGADHRRRRSVVAPGLRHHHVQRYVATMVSNIDTVIDGWQPGQRLDIYQEL

RSAVRRSTAESLFGQRLAVHSDFLGEQLQPLLDLTRRPPQVMRLQQRVNSPGWRRAMAAR

KRIDDLIDAQIADARTAPRPDDHMLTTLISGCSEEGTTLSDNEIRDSIVSLITAGYETTS

GALAWAIYALLTVPGTWESAASEVARVLGGRVPAADDLSALTYLNGVVHETLRLYSPGVI

SARRVLRDLWFDGHRIRAGRLLIFSAYVTHRLPEIWPEPTEFRPLRWDPNAADYRKPAPH

EFIPFSGGLHRCIGAVMATTEMTVILARLVARAMLQLPAQRTHRIRAANFAALRPWPGLT

VEIRKSAPAQ

>CYP139A1(2578200424)Mycobacterium tuberculosis TB_RSA124

MRYPLGEALLALYRWRGPLINAGVGGHGYTYLLGAEANRFVFANADAFSWSQTFESLVPV

DGPTALIVSDGADHRRRRSVVAPGLRHHHVQRYVATMVSNIDTVIDGWQPGQRLDIYQEL

RSAVRRSTAESLFGQRLAVHSDFLGEQLQPLLDLTRRPPQVMRLQQRVNSPGWRRAMAAR

KRIDDLIDAQIADARTAPRPDDHMLTTLISGCSEEGTTLSDNEIRDSIVSLITAGYETTS

GALAWAIYALLTVPGTWESAASEVARVLGGRVPAADDLSALTYLNGVVHETLRLYSPGVI

SARRVLRDLWFDGHRIRAGRLLIFSAYVTHRLPEIWPEPTEFRPLRWDPNAADYRKPAPH

EFIPFSGGLHRCIGAVMATTEMTVILARLVARAMLQLPAQRTHRIRAANFAALRPWPGLT

VEIRKSAPAQ

>CYP139A1(2584666311)Mycobacterium tuberculosis TKK_03_0078

MRYPLGEALLALYRWRGPLINAGVGGHGYTYLLGAEANRFVFANADAFSWSQTFESLVPV

DGPTALIVSDGADHRRRRSVVAPGLRHHHVQRYVATMVSNIDTVIDGWQPGQRLDIYQEL

RSAVRRSTAESLFGQRLAVHSDFLGEQLQPLLDLTRRPPQVMRLQQRVNSPGWRRAMAAR

KRIDDLIDAQIADARTAPRPDDHMLTTLISGCSEEGTTLSDNEIRDSIVSLITAGYETTS

GALAWAIYALLTVPGTWESAASEVARVLGGRVPAADDLSALTYLNGVVHETLRLYSPGVI

SARRVLRDLWFDGHRIRAGRLLIFSAYVTHRLPEIWPEPTEFRPLRWDPNAADYRKPAPH

EFIPFSGGLHRCIGAVMATTEMTVILARLVARAMLQLPAQRTHRIRAANFAALRPWPGLT

VEIRKSAPAQ

>CYP139A1(2584743940)Mycobacterium tuberculosis TB_RSA45

MRYPLGEALLALYRWRGPLINAGVGGHGYTYLLGAEANRFVFANADAFSWSQTFESLVPV

DGPTALIVSDGADHRRRRSVVAPGLRHHHVQRYVATMVSNIDTVIDGWQPGQRLDIYQEL

RSAVRRSTAESLFGQRLAVHSDFLGEQLQPLLDLTRRPPQVMRLQQRVNSPGWRRAMAAR

KRIDDLIDAQIADARTAPRPDDHMLTTLISGCSEEGTTLSDNEIRDSIVSLITAGYETTS

GALAWAIYALLTVPGTWESAASEVARVLGGRVPAADDLSALTYLNGVVHETLRLYSPGVI

SARRVLRDLWFDGHRIRAGRLLIFSAYVTHRLPEIWPEPTEFRPLRWDPNAADYRKPAPH

EFIPFSGGLHRCIGAVMATTEMTVILARLVARAMLQLPAQRTHRIRAANFAALRPWPGLT

VEIRKSAPAQ

>CYP139A1(2584781205)Mycobacterium tuberculosis TKK_05SA_0020

MRYPLGEALLALYRWRGPLINAGVGGHGYTYLLGAEANRFVFANADAFSWSQTFESLVPV

DGPTALIVSDGADHRRRRSVVAPGLRHHHVQRYVATMVSNIDTVIDGWQPGQRLDIYQEL

RSAVRRSTAESLFGQRLAVHSDFLGEQLQPLLDLTRRPPQVMRLQQRVNSPGWRRAMAAR

KRIDDLIDAQIADARTAPRPDDHMLTTLISGCSEEGTTLSDNEIRDSIVSLITAGYETTS

GALAWAIYALLTVPGTWESAASEVARVLGGRVPAADDLSALTYLNGVVHETLRLYSPGVI

SARRVLRDLWFDGHRIRAGRLLIFSAYVTHRLPEIWPEPTEFRPLRWDPNAADYRKPAPH

EFIPFSGGLHRCIGAVMATTEMTVILARLVARAMLQLPAQRTHRIRAANFAALRPWPGLT

VEIRKSAPAQ

>CYP139A1(2584833644)Mycobacterium tuberculosis TKK-01-0021

MRYPLGEALLALYRWRGPLINAGVGGHGYTYLLGAEANRFVFANADAFSWSQTFESLVPV

DGPTALIVSDGADHRRRRSVVAPGLRHHHVQRYVATMVSNIDTVIDGWQPGQRLDIYQEL

RSAVRRSTAESLFGQRLAVHSDFLGEQLQPLLDLTRRPPQVMRLQQRVNSPGWRRAMAAR

KRIDDLIDAQIADARTAPRPDDHMLTTLISGCSEEGTTLSDNEIRDSIVSLITAGYETTS

GALAWAIYALLTVPGTWESAASEVARVLGGRVPAADDLSALTYLNGVVHETLRLYSPGVI

SARRVLRDLWFDGHRIRAGRLLIFSAYVTHRLPEIWPEPTEFRPLRWDPNAADYRKPAPH

EFIPFSGGLHRCIGAVMATTEMTVILARLVARAMLQLPAQRTHRIRAANFAALRPWPGLT

VEIRKSAPAQ

>CYP139A1(2589073023)Mycobacterium tuberculosis TBR42

MRYPLGEALLALYRWRGPLINAGVGGHGYTYLLGAEANRFVFANADAFSWSQTFESLVPV

DGPTALIVSDGADHRRRRSVVAPGLRHHHVQRYVATMVSNIDTVIDGWQPGQRLDIYQEL

RSAVRRSTAESLFGQRLAVHSDFLGEQLQPLLDLTRRPPQVMRLQQRVNSPGWRRAMAAR

KRIDDLIDAQIADARTAPRPDDHMLTTLISGCSEEGTTLSDNEIRDSIVSLITAGYETTS

GALAWAIYALLTVPGTWESAASEVARVLGGRVPAADDLSALTYLNGVVHETLRLYSPGVI

SARRVLRDLWFDGHRIRAGRLLIFSAYVTHRLPEIWPEPTEFRPLRWDPNAADYRKPAPH

EFIPFSGGLHRCIGAVMATTEMTVILARLVARAMLQLPAQRTHRIRAANFAALRPWPGLT

VEIRKSAPAQ

>CYP139A1(2589509569)Mycobacterium tuberculosis TKK-01-0012

MRYPLGEALLALYRWRGPLINAGVGGHGYTYLLGAEANRFVFANADAFSWSQTFESLVPV

DGPTALIVSDGADHRRRRSVVAPGLRHHHVQRYVATMVSNIDTVIDGWQPGQRLDIYQEL

RSAVRRSTAESLFGQRLAVHSDFLGEQLQPLLDLTRRPPQVMRLQQRVNSPGWRRAMAAR

KRIDDLIDAQIADARTAPRPDDHMLTTLISGCSEEGTTLSDNEIRDSIVSLITAGYETTS

GALAWAIYALLTVPGTWESAASEVARVLGGRVPAADDLSALTYLNGVVHETLRLYSPGVI

SARRVLRDLWFDGHRIRAGRLLIFSAYVTHRLPEIWPEPTEFRPLRWDPNAADYRKPAPH

EFIPFSGGLHRCIGAVMATTEMTVILARLVARAMLQLPAQRTHRIRAANFAALRPWPGLT

VEIRKSAPAQ

>CYP139A1(2589551314)Mycobacterium tuberculosis TKK-01-0031

MRYPLGEALLALYRWRGPLINAGVGGHGYTYLLGAEANRFVFANADAFSWSQTFESLVPV

DGPTALIVSDGADHRRRRSVVAPGLRHHHVQRYVATMVSNIDTVIDGWQPGQRLDIYQEL

RSAVRRSTAESLFGQRLAVHSDFLGEQLQPLLDLTRRPPQVMRLQQRVNSPGWRRAMAAR

KRIDDLIDAQIADARTAPRPDDHMLTTLISGCSEEGTTLSDNEIRDSIVSLITAGYETTS

GALAWAIYALLTVPGTWESAASEVARVLGGRVPAADDLSALTYLNGVVHETLRLYSPGVI

SARRVLRDLWFDGHRIRAGRLLIFSAYVTHRLPEIWPEPTEFRPLRWDPNAADYRKPAPH

EFIPFSGGLHRCIGAVMATTEMTVILARLVARAMLQLPAQRTHRIRAANFAALRPWPGLT

VEIRKSAPAQ

>CYP139A1(2589580598)Mycobacterium tuberculosis TKK-01-0040

MRYPLGEALLALYRWRGPLINAGVGGHGYTYLLGAEANRFVFANADAFSWSQTFESLVPV

DGPTALIVSDGADHRRRRSVVAPGLRHHHVQRYVATMVSNIDTVIDGWQPGQRLDIYQEL

RSAVRRSTAESLFGQRLAVHSDFLGEQLQPLLDLTRRPPQVMRLQQRVNSPGWRRAMAAR

KRIDDLIDAQIADARTAPRPDDHMLTTLISGCSEEGTTLSDNEIRDSIVSLITAGYETTS

GALAWAIYALLTVPGTWESAASEVARVLGGRVPAADDLSALTYLNGVVHETLRLYSPGVI

SARRVLRDLWFDGHRIRAGRLLIFSAYVTHRLPEIWPEPTEFRPLRWDPNAADYRKPAPH

EFIPFSGGLHRCIGAVMATTEMTVILARLVARAMLQLPAQRTHRIRAANFAALRPWPGLT

VEIRKSAPAQ

>CYP139A1(2589629003)Mycobacterium tuberculosis TKK-01-0058

MRYPLGEALLALYRWRGPLINAGVGGHGYTYLLGAEANRFVFANADAFSWSQTFESLVPV

DGPTALIVSDGADHRRRRSVVAPGLRHHHVQRYVATMVSNIDTVIDGWQPGQRLDIYQEL

RSAVRRSTAESLFGQRLAVHSDFLGEQLQPLLDLTRRPPQVMRLQQRVNSPGWRRAMAAR

KRIDDLIDAQIADARTAPRPDDHMLTTLISGCSEEGTTLSDNEIRDSIVSLITAGYETTS

GALAWAIYALLTVPGTWESAASEVARVLGGRVPAADDLSALTYLNGVVHETLRLYSPGVI

SARRVLRDLWFDGHRIRAGRLLIFSAYVTHRLPEIWPEPTEFRPLRWDPNAADYRKPAPH

EFIPFSGGLHRCIGAVMATTEMTVILARLVARAMLQLPAQRTHRIRAANFAALRPWPGLT

VEIRKSAPAQ

>CYP139A1(2589676017)Mycobacterium tuberculosis TKK-01-0071

MRYPLGEALLALYRWRGPLINAGVGGHGYTYLLGAEANRFVFANADAFSWSQTFESLVPV

DGPTALIVSDGADHRRRRSVVAPGLRHHHVQRYVATMVSNIDTVIDGWQPGQRLDIYQEL

RSAVRRSTAESLFGQRLAVHSDFLGEQLQPLLDLTRRPPQVMRLQQRVNSPGWRRAMAAR

KRIDDLIDAQIADARTAPRPDDHMLTTLISGCSEEGTTLSDNEIRDSIVSLITAGYETTS

GALAWAIYALLTVPGTWESAASEVARVLGGRVPAADDLSALTYLNGVVHETLRLYSPGVI

SARRVLRDLWFDGHRIRAGRLLIFSAYVTHRLPEIWPEPTEFRPLRWDPNAADYRKPAPH

EFIPFSGGLHRCIGAVMATTEMTVILARLVARAMLQLPAQRTHRIRAANFAALRPWPGLT

VEIRKSAPAQ

>CYP139A1(2592332604)Mycobacterium tuberculosis TKK_02_0077

MRYPLGEALLALYRWRGPLINAGVGGHGYTYLLGAEANRFVFANADAFSWSQTFESLVPV

DGPTALIVSDGADHRRRRSVVAPGLRHHHVQRYVATMVSNIDTVIDGWQPGQRLDIYQEL

RSAVRRSTAESLFGQRLAVHSDFLGEQLQPLLDLTRRPPQVMRLQQRVNSPGWRRAMAAR

KRIDDLIDAQIADARTAPRPDDHMLTTLISGCSEEGTTLSDNEIRDSIVSLITAGYETTS

GALAWAIYALLTVPGTWESAASEVARVLGGRVPAADDLSALTYLNGVVHETLRLYSPGVI

SARRVLRDLWFDGHRIRAGRLLIFSAYVTHRLPEIWPEPTEFRPLRWDPNAADYRKPAPH

EFIPFSGGLHRCIGAVMATTEMTVILARLVARAMLQLPAQRTHRIRAANFAALRPWPGLT

VEIRKSAPAQ

>CYP139A1(2592542016)Mycobacterium tuberculosis TKK_04_0047

MRYPLGEALLALYRWRGPLINAGVGGHGYTYLLGAEANRFVFANADAFSWSQTFESLVPV

DGPTALIVSDGADHRRRRSVVAPGLRHHHVQRYVATMVSNIDTVIDGWQPGQRLDIYQEL

RSAVRRSTAESLFGQRLAVHSDFLGEQLQPLLDLTRRPPQVMRLQQRVNSPGWRRAMAAR

KRIDDLIDAQIADARTAPRPDDHMLTTLISGCSEEGTTLSDNEIRDSIVSLITAGYETTS

GALAWAIYALLTVPGTWESAASEVARVLGGRVPAADDLSALTYLNGVVHETLRLYSPGVI

SARRVLRDLWFDGHRIRAGRLLIFSAYVTHRLPEIWPEPTEFRPLRWDPNAADYRKPAPH

EFIPFSGGLHRCIGAVMATTEMTVILARLVARAMLQLPAQRTHRIRAANFAALRPWPGLT

VEIRKSAPAQ

>CYP139A1(2592562396)Mycobacterium tuberculosis TKK_04_0036

MRYPLGEALLALYRWRGPLINAGVGGHGYTYLLGAEANRFVFANADAFSWSQTFESLVPV

DGPTALIVSDGADHRRRRSVVAPGLRHHHVQRYVATMVSNIDTVIDGWQPGQRLDIYQEL

RSAVRRSTAESLFGQRLAVHSDFLGEQLQPLLDLTRRPPQVMRLQQRVNSPGWRRAMAAR

KRIDDLIDAQIADARTAPRPDDHMLTTLISGCSEEGTTLSDNEIRDSIVSLITAGYETTS

GALAWAIYALLTVPGTWESAASEVARVLGGRVPAADDLSALTYLNGVVHETLRLYSPGVI

SARRVLRDLWFDGHRIRAGRLLIFSAYVTHRLPEIWPEPTEFRPLRWDPNAADYRKPAPH

EFIPFSGGLHRCIGAVMATTEMTVILARLVARAMLQLPAQRTHRIRAANFAALRPWPGLT

VEIRKSAPAQ

>CYP139A1(2541578033)*Mycobacterium canettii* CIPT 140060008

MRYPLGEALLALYRWRGPLINAGVGGHGYTYLLGAEANRFVFANADAFSWSQTFESLVPV

DGPTALIVSDGADHRRRRSVVAPGLRHHHVQRYVATMVSNIDTVIDGWQPGQRLDIYQEL

RSAVRRSTAESLFGQRLAVHSDFLGEQLQPLLDLTRRPPQVMRLQQRVNSPGWRRAMAAR

KRIDDLIDAQIADARTAPRPDDHMLTTLISGCSEEGTTLSDNEIRDSIVSLITAGYETTS

GALAWAIYALLTVPGTWESAASEVARVLGGRVPAADDLSALTYLNGVVHETLRLYSPGVI

SARRVLRDLWFDGHRIRAGRLLIFSAYVTHRLPEIWPEPTEFRPLRWDPNAADYRKPAPH

EFIPFSGGLHRCIGAVMATTEMTVILARLVARAMLQLPAQRTHRIRAANFAALRPWPGLT

VEIRKSAPAQ

>CYP139A1(2574761421)Mycobacterium tuberculosis MD19043

MRYPLGEALLALYRWRGPLINAGVGGHGYTYLLGAEANRFVFANADAFSWSQTFESLVPV

DGPTALIVSDGADHRRRRSVVAPGLRHHHVQRYVATMVSNIDTVIDGWQPGQRLDIYQEL

RSAVRRSTAESLFGQRLAVHSDFLGEQLQPLLDLTRRPPQVMRLQQRVNSPGWRRAMAAR

KRIDDLIDAQIADARTAPRPDDHMLTTLISGCSEEGTTLSDNEIRDSIVSLITAGYETTS

GALAWAIYALLTVPGTWESAASEVARVLGGRVPAADDLSALTYLNGVVHETLRLYSPGVI

SARRVLRDLWFDGHRIRAGRLLIFSAYVTHRLPEIWPEPTEFRPLRWDPNAADYRKPAPH

EFIPFSGGLHRCIGAVMATTEMTVILARLVARAMLQLPAQRTHRIRAANFAALRPWPGLT

VEIRKSAPAQ

>CYP139A1(2575611823)Mycobacterium tuberculosis TB_RSA99

MRYPLGEALLALYRWRGPLINAGVGGHGYTYLLGAEANRFVFANADAFSWSQTFESLVPV

DGPTALIVSDGADHRRRRSVVAPGLRHHHVQRYVATMVSNIDTVIDGWQPGQRLDIYQEL

RSAVRRSTAESLFGQRLAVHSDFLGEQLQPLLDLTRRPPQVMRLQQRVNSPGWRRAMAAR

KRIDDLIDAQIADARTAPRPDDHMLTTLISGCSEEGTTLSDNEIRDSIVSLITAGYETTS

GALAWAIYALLTVPGTWESAASEVARVLGGRVPAADDLSALTYLNGVVHETLRLYSPGVI

SARRVLRDLWFDGHRIRAGRLLIFSAYVTHRLPEIWPEPTEFRPLRWDPNAADYRKPAPH

EFIPFSGGLHRCIGAVMATTEMTVILARLVARAMLQLPAQRTHRIRAANFAALRPWPGLT

VEIRKSAPAQ

>CYP139A1(2576564801)Mycobacterium tuberculosis BTB05-013

MRYPLGEALLALYRWRGPLINAGVGGHGYTYLLGAEANRFVFANADAFSWSQTFESLVPV

DGPTALIVSDGADHRRRRSVVAPGLRHHHVQRYVATMVSNIDTVIDGWQPGQRLDIYQEL

RSAVRRSTAESLFGQRLAVHSDFLGEQLQPLLDLTRRPPQVMRLQQRVNSPGWRRAMAAR

KRIDDLIDAQIADARTAPRPDDHMLTTLISGCSEEGTTLSDNEIRDSIVSLITAGYETTS

GALAWAIYALLTVPGTWESAASEVARVLGGRVPAADDLSALTYLNGVVHETLRLYSPGVI

SARRVLRDLWFDGHRIRAGRLLIFSAYVTHRLPEIWPEPTEFRPLRWDPNAADYRKPAPH

EFIPFSGGLHRCIGAVMATTEMTVILARLVARAMLQLPAQRTHRIRAANFAALRPWPGLT

VEIRKSAPAQ

>CYP139A1(2577821955)Mycobacterium tuberculosis MD14435

MRYPLGEALLALYRWRGPLINAGVGGHGYTYLLGAEANRFVFANADAFSWSQTFESLVPV

DGPTALIVSDGADHRRRRSVVAPGLRHHHVQRYVATMVSNIDTVIDGWQPGQRLDIYQEL

RSAVRRSTAESLFGQRLAVHSDFLGEQLQPLLDLTRRPPQVMRLQQRVNSPGWRRAMAAR

KRIDDLIDAQIADARTAPRPDDHMLTTLISGCSEEGTTLSDNEIRDSIVSLITAGYETTS

GALAWAIYALLTVPGTWESAASEVARVLGGRVPAADDLSALTYLNGVVHETLRLYSPGVI

SARRVLRDLWFDGHRIRAGRLLIFSAYVTHRLPEIWPEPTEFRPLRWDPNAADYRKPAPH

EFIPFSGGLHRCIGAVMATTEMTVILARLVARAMLQLPAQRTHRIRAANFAALRPWPGLT

VEIRKSAPAQ

>CYP139A1(2578086064)Mycobacterium tuberculosis M1978

MRYPLGEALLALYRWRGPLINAGVGGHGYTYLLGAEANRFVFANADAFSWSQTFESLVPV

DGPTALIVSDGADHRRRRSVVAPGLRHHHVQRYVATMVSNIDTVIDGWQPGQRLDIYQEL

RSAVRRSTAESLFGQRLAVHSDFLGEQLQPLLDLTRRPPQVMRLQQRVNSPGWRRAMAAR

KRIDDLIDAQIADARTAPRPDDHMLTTLISGCSEEGTTLSDNEIRDSIVSLITAGYETTS

GALAWAIYALLTVPGTWESAASEVARVLGGRVPAADDLSALTYLNGVVHETLRLYSPGVI

SARRVLRDLWFDGHRIRAGRLLIFSAYVTHRLPEIWPEPTEFRPLRWDPNAADYRKPAPH

EFIPFSGGLHRCIGAVMATTEMTVILARLVARAMLQLPAQRTHRIRAANFAALRPWPGLT

VEIRKSAPAQ

>CYP139A1(2580669105)*Mycobacterium africanum* MAL010081

MRYPLGEALLALYRWRGPLINAGVGGHGYTYLLGAEANRFVFANADAFSWSQTFESLVPV

DGPTALIVSDGADHRRRRSVVAPGLRHHHVQRYVATMVSNIDTVIDGWQPGQRLDIYQEL

RSAVRRSTAESLFGQRLAVHSDFLGEQLQPLLDLTRRPPQVMRLQQRVNSPGWRRAMAAR

KRIDDLIDAQIADARTAPRPDDHMLTTLISGCSEEGTTLSDNEIRDSIVSLITAGYETTS

GALAWAIYALLTVPGTWESAASEVARVLGGRVPAADDLSALTYLNGVVHETLRLYSPGVI

SARRVLRDLWFDGHRIRAGRLLIFSAYVTHRLPEIWPEPTEFRPLRWDPNAADYRKPAPH

EFIPFSGGLHRCIGAVMATTEMTVILARLVARAMLQLPAQRTHRIRAANFAALRPWPGLT

VEIRKSAPAQ

>CYP139A1(2583740670)*Mycobacterium africanum* MAL020176

MRYPLGEALLALYRWRGPLINAGVGGHGYTYLLGAEANRFVFANADAFSWSQTFESLVPV

DGPTALIVSDGADHRRRRSVVAPGLRHHHVQRYVATMVSNIDTVIDGWQPGQRLDIYQEL

RSAVRRSTAESLFGQRLAVHSDFLGEQLQPLLDLTRRPPQVMRLQQRVNSPGWRRAMAAR

KRIDDLIDAQIADARTAPRPDDHMLTTLISGCSEEGTTLSDNEIRDSIVSLITAGYETTS

GALAWAIYALLTVPGTWESAASEVARVLGGRVPAADDLSALTYLNGVVHETLRLYSPGVI

SARRVLRDLWFDGHRIRAGRLLIFSAYVTHRLPEIWPEPTEFRPLRWDPNAADYRKPAPH

EFIPFSGGLHRCIGAVMATTEMTVILARLVARAMLQLPAQRTHRIRAANFAALRPWPGLT

VEIRKSAPAQ

>CYP139A1(2584831832)Mycobacterium tuberculosis NRITLD09

MRYPLGEALLALYRWRGPLINAGVGGHGYTYLLGAEANRFVFANADAFSWSQTFESLVPV

DGPTALIVSDGADHRRRRSVVAPGLRHHHVQRYVATMVSNIDTVIDGWQPGQRLDIYQEL

RSAVRRSTAESLFGQRLAVHSDFLGEQLQPLLDLTRRPPQVMRLQQRVNSPGWRRAMAAR

KRIDDLIDAQIADARTAPRPDDHMLTTLISGCSEEGTTLSDNEIRDSIVSLITAGYETTS

GALAWAIYALLTVPGTWESAASEVARVLGGRVPAADDLSALTYLNGVVHETLRLYSPGVI

SARRVLRDLWFDGHRIRAGRLLIFSAYVTHRLPEIWPEPTEFRPLRWDPNAADYRKPAPH

EFIPFSGGLHRCIGAVMATTEMTVILARLVARAMLQLPAQRTHRIRAANFAALRPWPGLT

VEIRKSAPAQ

>CYP139A1(2584875064)Mycobacterium tuberculosis 3499MM

MRYPLGEALLALYRWRGPLINAGVGGHGYTYLLGAEANRFVFANADAFSWSQTFESLVPV

DGPTALIVSDGADHRRRRSVVAPGLRHHHVQRYVATMVSNIDTVIDGWQPGQRLDIYQEL

RSAVRRSTAESLFGQRLAVHSDFLGEQLQPLLDLTRRPPQVMRLQQRVNSPGWRRAMAAR

KRIDDLIDAQIADARTAPRPDDHMLTTLISGCSEEGTTLSDNEIRDSIVSLITAGYETTS

GALAWAIYALLTVPGTWESAASEVARVLGGRVPAADDLSALTYLNGVVHETLRLYSPGVI

SARRVLRDLWFDGHRIRAGRLLIFSAYVTHRLPEIWPEPTEFRPLRWDPNAADYRKPAPH

EFIPFSGGLHRCIGAVMATTEMTVILARLVARAMLQLPAQRTHRIRAANFAALRPWPGLT

VEIRKSAPAQ

>CYP139A1(2590158810)Mycobacterium tuberculosis MAL020197

MRYPLGEALLALYRWRGPLINAGVGGHGYTYLLGAEANRFVFANADAFSWSQTFESLVPV

DGPTALIVSDGADHRRRRSVVAPGLRHHHVQRYVATMVSNIDTVIDGWQPGQRLDIYQEL

RSAVRRSTAESLFGQRLAVHSDFLGEQLQPLLDLTRRPPQVMRLQQRVNSPGWRRAMAAR

KRIDDLIDAQIADARTAPRPDDHMLTTLISGCSEEGTTLSDNEIRDSIVSLITAGYETTS

GALAWAIYALLTVPGTWESAASEVARVLGGRVPAADDLSALTYLNGVVHETLRLYSPGVI

SARRVLRDLWFDGHRIRAGRLLIFSAYVTHRLPEIWPEPTEFRPLRWDPNAADYRKPAPH

EFIPFSGGLHRCIGAVMATTEMTVILARLVARAMLQLPAQRTHRIRAANFAALRPWPGLT

VEIRKSAPAQ

>CYP139A1(2590240089)Mycobacterium tuberculosis KT-0039

MRYPLGEALLALYRWRGPLINAGVGGHGYTYLLGAEANRFVFANADAFSWSQTFESLVPV

DGPTALIVSDGADHRRRRSVVAPGLRHHHVQRYVATMVSNIDTVIDGWQPGQRLDIYQEL

RSAVRRSTAESLFGQRLAVHSDFLGEQLQPLLDLTRRPPQVMRLQQRVNSPGWRRAMAAR

KRIDDLIDAQIADARTAPRPDDHMLTTLISGCSEEGTTLSDNEIRDSIVSLITAGYETTS

GALAWAIYALLTVPGTWESAASEVARVLGGRVPAADDLSALTYLNGVVHETLRLYSPGVI

SARRVLRDLWFDGHRIRAGRLLIFSAYVTHRLPEIWPEPTEFRPLRWDPNAADYRKPAPH

EFIPFSGGLHRCIGAVMATTEMTVILARLVARAMLQLPAQRTHRIRAANFAALRPWPGLT

VEIRKSAPAQ

>CYP139A1(2590350138)Mycobacterium tuberculosis OFXR-31

MRYPLGEALLALYRWRGPLINAGVGGHGYTYLLGAEANRFVFANADAFSWSQTFESLVPV

DGPTALIVSDGADHRRRRSVVAPGLRHHHVQRYVATMVSNIDTVIDGWQPGQRLDIYQEL

RSAVRRSTAESLFGQRLAVHSDFLGEQLQPLLDLTRRPPQVMRLQQRVNSPGWRRAMAAR

KRIDDLIDAQIADARTAPRPDDHMLTTLISGCSEEGTTLSDNEIRDSIVSLITAGYETTS

GALAWAIYALLTVPGTWESAASEVARVLGGRVPAADDLSALTYLNGVVHETLRLYSPGVI

SARRVLRDLWFDGHRIRAGRLLIFSAYVTHRLPEIWPEPTEFRPLRWDPNAADYRKPAPH

EFIPFSGGLHRCIGAVMATTEMTVILARLVARAMLQLPAQRTHRIRAANFAALRPWPGLT

VEIRKSAPAQ

>CYP139A1(2590493738)Mycobacterium tuberculosis KT-0107

MRYPLGEALLALYRWRGPLINAGVGGHGYTYLLGAEANRFVFANADAFSWSQTFESLVPV

DGPTALIVSDGADHRRRRSVVAPGLRHHHVQRYVATMVSNIDTVIDGWQPGQRLDIYQEL

RSAVRRSTAESLFGQRLAVHSDFLGEQLQPLLDLTRRPPQVMRLQQRVNSPGWRRAMAAR

KRIDDLIDAQIADARTAPRPDDHMLTTLISGCSEEGTTLSDNEIRDSIVSLITAGYETTS

GALAWAIYALLTVPGTWESAASEVARVLGGRVPAADDLSALTYLNGVVHETLRLYSPGVI

SARRVLRDLWFDGHRIRAGRLLIFSAYVTHRLPEIWPEPTEFRPLRWDPNAADYRKPAPH

EFIPFSGGLHRCIGAVMATTEMTVILARLVARAMLQLPAQRTHRIRAANFAALRPWPGLT

VEIRKSAPAQ

>CYP139A1(2590554696)Mycobacterium tuberculosis KT-0075

MRYPLGEALLALYRWRGPLINAGVGGHGYTYLLGAEANRFVFANADAFSWSQTFESLVPV

DGPTALIVSDGADHRRRRSVVAPGLRHHHVQRYVATMVSNIDTVIDGWQPGQRLDIYQEL

RSAVRRSTAESLFGQRLAVHSDFLGEQLQPLLDLTRRPPQVMRLQQRVNSPGWRRAMAAR

KRIDDLIDAQIADARTAPRPDDHMLTTLISGCSEEGTTLSDNEIRDSIVSLITAGYETTS

GALAWAIYALLTVPGTWESAASEVARVLGGRVPAADDLSALTYLNGVVHETLRLYSPGVI

SARRVLRDLWFDGHRIRAGRLLIFSAYVTHRLPEIWPEPTEFRPLRWDPNAADYRKPAPH

EFIPFSGGLHRCIGAVMATTEMTVILARLVARAMLQLPAQRTHRIRAANFAALRPWPGLT

VEIRKSAPAQ

>CYP139A1(2576084283)Mycobacterium tuberculosis XTB13-195

MRYPLGEALLALYRWRGPLINAGVGGHGYTYLLGAEANRFVFANADAFSWSQTFESLVPV

DGPTALIVSDGADHRRRRSVVAPGLRHHHVQRYVATMVSNIDTVIDGWQPGQRLDIYQEL

RSAVRRSTAESLFGQRLAVHSDFLGEQLQPLLDLTRRPPQVMRLQQRVNSPGWRRAMAAR

KRIDDLIDAQIADARTAPRPDDHMLTTLISGCSEEGTTLSDNEIRDSIVSLITAGYETTS

GALAWAIYALLTVPGTWESAASEVARVLGGRVPAADDLSALTYLNGVVHETLRLYSPGVI

SARRVLRDLWFDGHRIRAGRLLIFSAYVTHRLPEIWPEPTEFRPLRWDPNAADYRKPAPH

EFIPFSGGLHRCIGAVMATTEMTVILARLVARAMLQLPAQRTHRIRAANFAALRPWPGLT

VEIRKSAPAQ

>CYP139A1(2576366402)Mycobacterium tuberculosis TKK_02_0045

MRYPLGEALLALYRWRGPLINAGVGGHGYTYLLGAEANRFVFANADAFSWSQTFESLVPV

DGPTALIVSDGADHRRRRSVVAPGLRHHHVQRYVATMVSNIDTVIDGWQPGQRLDIYQEL

RSAVRRSTAESLFGQRLAVHSDFLGEQLQPLLDLTRRPPQVMRLQQRVNSPGWRRAMAAR

KRIDDLIDAQIADARTAPRPDDHMLTTLISGCSEEGTTLSDNEIRDSIVSLITAGYETTS

GALAWAIYALLTVPGTWESAASEVARVLGGRVPAADDLSALTYLNGVVHETLRLYSPGVI

SARRVLRDLWFDGHRIRAGRLLIFSAYVTHRLPEIWPEPTEFRPLRWDPNAADYRKPAPH

EFIPFSGGLHRCIGAVMATTEMTVILARLVARAMLQLPAQRTHRIRAANFAALRPWPGLT

VEIRKSAPAQ

>CYP139A1(2576513411)Mycobacterium tuberculosis XTB13-093

MRYPLGEALLALYRWRGPLINAGVGGHGYTYLLGAEANRFVFANADAFSWSQTFESLVPV

DGPTALIVSDGADHRRRRSVVAPGLRHHHVQRYVATMVSNIDTVIDGWQPGQRLDIYQEL

RSAVRRSTAESLFGQRLAVHSDFLGEQLQPLLDLTRRPPQVMRLQQRVNSPGWRRAMAAR

KRIDDLIDAQIADARTAPRPDDHMLTTLISGCSEEGTTLSDNEIRDSIVSLITAGYETTS

GALAWAIYALLTVPGTWESAASEVARVLGGRVPAADDLSALTYLNGVVHETLRLYSPGVI

SARRVLRDLWFDGHRIRAGRLLIFSAYVTHRLPEIWPEPTEFRPLRWDPNAADYRKPAPH

EFIPFSGGLHRCIGAVMATTEMTVILARLVARAMLQLPAQRTHRIRAANFAALRPWPGLT

VEIRKSAPAQ

>CYP139A1(2576671439)Mycobacterium tuberculosis TKK_04_0108

MRYPLGEALLALYRWRGPLINAGVGGHGYTYLLGAEANRFVFANADAFSWSQTFESLVPV

DGPTALIVSDGADHRRRRSVVAPGLRHHHVQRYVATMVSNIDTVIDGWQPGQRLDIYQEL

RSAVRRSTAESLFGQRLAVHSDFLGEQLQPLLDLTRRPPQVMRLQQRVNSPGWRRAMAAR

KRIDDLIDAQIADARTAPRPDDHMLTTLISGCSEEGTTLSDNEIRDSIVSLITAGYETTS

GALAWAIYALLTVPGTWESAASEVARVLGGRVPAADDLSALTYLNGVVHETLRLYSPGVI

SARRVLRDLWFDGHRIRAGRLLIFSAYVTHRLPEIWPEPTEFRPLRWDPNAADYRKPAPH

EFIPFSGGLHRCIGAVMATTEMTVILARLVARAMLQLPAQRTHRIRAANFAALRPWPGLT

VEIRKSAPAQ

>CYP139A1(2577792721)Mycobacterium tuberculosis TBR26

MRYPLGEALLALYRWRGPLINAGVGGHGYTYLLGAEANRFVFANADAFSWSQTFESLVPV

DGPTALIVSDGADHRRRRSVVAPGLRHHHVQRYVATMVSNIDTVIDGWQPGQRLDIYQEL

RSAVRRSTAESLFGQRLAVHSDFLGEQLQPLLDLTRRPPQVMRLQQRVNSPGWRRAMAAR

KRIDDLIDAQIADARTAPRPDDHMLTTLISGCSEEGTTLSDNEIRDSIVSLITAGYETTS

GALAWAIYALLTVPGTWESAASEVARVLGGRVPAADDLSALTYLNGVVHETLRLYSPGVI

SARRVLRDLWFDGHRIRAGRLLIFSAYVTHRLPEIWPEPTEFRPLRWDPNAADYRKPAPH

EFIPFSGGLHRCIGAVMATTEMTVILARLVARAMLQLPAQRTHRIRAANFAALRPWPGLT

VEIRKSAPAQ

>CYP139A1(2577796885)Mycobacterium tuberculosis TKK_03_0103

MRYPLGEALLALYRWRGPLINAGVGGHGYTYLLGAEANRFVFANADAFSWSQTFESLVPV

DGPTALIVSDGADHRRRRSVVAPGLRHHHVQRYVATMVSNIDTVIDGWQPGQRLDIYQEL

RSAVRRSTAESLFGQRLAVHSDFLGEQLQPLLDLTRRPPQVMRLQQRVNSPGWRRAMAAR

KRIDDLIDAQIADARTAPRPDDHMLTTLISGCSEEGTTLSDNEIRDSIVSLITAGYETTS

GALAWAIYALLTVPGTWESAASEVARVLGGRVPAADDLSALTYLNGVVHETLRLYSPGVI

SARRVLRDLWFDGHRIRAGRLLIFSAYVTHRLPEIWPEPTEFRPLRWDPNAADYRKPAPH

EFIPFSGGLHRCIGAVMATTEMTVILARLVARAMLQLPAQRTHRIRAANFAALRPWPGLT

VEIRKSAPAQ

>CYP139A1(2584686721)Mycobacterium tuberculosis XTB13-175

MRYPLGEALLALYRWRGPLINAGVGGHGYTYLLGAEANRFVFANADAFSWSQTFESLVPV

DGPTALIVSDGADHRRRRSVVAPGLRHHHVQRYVATMVSNIDTVIDGWQPGQRLDIYQEL

RSAVRRSTAESLFGQRLAVHSDFLGEQLQPLLDLTRRPPQVMRLQQRVNSPGWRRAMAAR

KRIDDLIDAQIADARTAPRPDDHMLTTLISGCSEEGTTLSDNEIRDSIVSLITAGYETTS

GALAWAIYALLTVPGTWESAASEVARVLGGRVPAADDLSALTYLNGVVHETLRLYSPGVI

SARRVLRDLWFDGHRIRAGRLLIFSAYVTHRLPEIWPEPTEFRPLRWDPNAADYRKPAPH

EFIPFSGGLHRCIGAVMATTEMTVILARLVARAMLQLPAQRTHRIRAANFAALRPWPGLT

VEIRKSAPAQ

>CYP139A1(2584752944)Mycobacterium tuberculosis TKK_05MA_0040

MRYPLGEALLALYRWRGPLINAGVGGHGYTYLLGAEANRFVFANADAFSWSQTFESLVPV

DGPTALIVSDGADHRRRRSVVAPGLRHHHVQRYVATMVSNIDTVIDGWQPGQRLDIYQEL

RSAVRRSTAESLFGQRLAVHSDFLGEQLQPLLDLTRRPPQVMRLQQRVNSPGWRRAMAAR

KRIDDLIDAQIADARTAPRPDDHMLTTLISGCSEEGTTLSDNEIRDSIVSLITAGYETTS

GALAWAIYALLTVPGTWESAASEVARVLGGRVPAADDLSALTYLNGVVHETLRLYSPGVI

SARRVLRDLWFDGHRIRAGRLLIFSAYVTHRLPEIWPEPTEFRPLRWDPNAADYRKPAPH

EFIPFSGGLHRCIGAVMATTEMTVILARLVARAMLQLPAQRTHRIRAANFAALRPWPGLT

VEIRKSAPAQ

>CYP139A1(2584808933)Mycobacterium tuberculosis XTB13-203

MRYPLGEALLALYRWRGPLINAGVGGHGYTYLLGAEANRFVFANADAFSWSQTFESLVPV

DGPTALIVSDGADHRRRRSVVAPGLRHHHVQRYVATMVSNIDTVIDGWQPGQRLDIYQEL

RSAVRRSTAESLFGQRLAVHSDFLGEQLQPLLDLTRRPPQVMRLQQRVNSPGWRRAMAAR

KRIDDLIDAQIADARTAPRPDDHMLTTLISGCSEEGTTLSDNEIRDSIVSLITAGYETTS

GALAWAIYALLTVPGTWESAASEVARVLGGRVPAADDLSALTYLNGVVHETLRLYSPGVI

SARRVLRDLWFDGHRIRAGRLLIFSAYVTHRLPEIWPEPTEFRPLRWDPNAADYRKPAPH

EFIPFSGGLHRCIGAVMATTEMTVILARLVARAMLQLPAQRTHRIRAANFAALRPWPGLT

VEIRKSAPAQ

>CYP139A1(2589134136)Mycobacterium tuberculosis TBR79

MRYPLGEALLALYRWRGPLINAGVGGHGYTYLLGAEANRFVFANADAFSWSQTFESLVPV

DGPTALIVSDGADHRRRRSVVAPGLRHHHVQRYVATMVSNIDTVIDGWQPGQRLDIYQEL

RSAVRRSTAESLFGQRLAVHSDFLGEQLQPLLDLTRRPPQVMRLQQRVNSPGWRRAMAAR

KRIDDLIDAQIADARTAPRPDDHMLTTLISGCSEEGTTLSDNEIRDSIVSLITAGYETTS

GALAWAIYALLTVPGTWESAASEVARVLGGRVPAADDLSALTYLNGVVHETLRLYSPGVI

SARRVLRDLWFDGHRIRAGRLLIFSAYVTHRLPEIWPEPTEFRPLRWDPNAADYRKPAPH

EFIPFSGGLHRCIGAVMATTEMTVILARLVARAMLQLPAQRTHRIRAANFAALRPWPGLT

VEIRKSAPAQ

>CYP139A1(2589539130)Mycobacterium tuberculosis TKK-01-0015

MRYPLGEALLALYRWRGPLINAGVGGHGYTYLLGAEANRFVFANADAFSWSQTFESLVPV

DGPTALIVSDGADHRRRRSVVAPGLRHHHVQRYVATMVSNIDTVIDGWQPGQRLDIYQEL

RSAVRRSTAESLFGQRLAVHSDFLGEQLQPLLDLTRRPPQVMRLQQRVNSPGWRRAMAAR

KRIDDLIDAQIADARTAPRPDDHMLTTLISGCSEEGTTLSDNEIRDSIVSLITAGYETTS

GALAWAIYALLTVPGTWESAASEVARVLGGRVPAADDLSALTYLNGVVHETLRLYSPGVI

SARRVLRDLWFDGHRIRAGRLLIFSAYVTHRLPEIWPEPTEFRPLRWDPNAADYRKPAPH

EFIPFSGGLHRCIGAVMATTEMTVILARLVARAMLQLPAQRTHRIRAANFAALRPWPGLT

VEIRKSAPAQ

>CYP139A1(2589598915)Mycobacterium tuberculosis TKK-01-0052

MRYPLGEALLALYRWRGPLINAGVGGHGYTYLLGAEANRFVFANADAFSWSQTFESLVPV

DGPTALIVSDGADHRRRRSVVAPGLRHHHVQRYVATMVSNIDTVIDGWQPGQRLDIYQEL

RSAVRRSTAESLFGQRLAVHSDFLGEQLQPLLDLTRRPPQVMRLQQRVNSPGWRRAMAAR

KRIDDLIDAQIADARTAPRPDDHMLTTLISGCSEEGTTLSDNEIRDSIVSLITAGYETTS

GALAWAIYALLTVPGTWESAASEVARVLGGRVPAADDLSALTYLNGVVHETLRLYSPGVI

SARRVLRDLWFDGHRIRAGRLLIFSAYVTHRLPEIWPEPTEFRPLRWDPNAADYRKPAPH

EFIPFSGGLHRCIGAVMATTEMTVILARLVARAMLQLPAQRTHRIRAANFAALRPWPGLT

VEIRKSAPAQ

>CYP139A1(2589679190)Mycobacterium tuberculosis TKK-01-0070

MRYPLGEALLALYRWRGPLINAGVGGHGYTYLLGAEANRFVFANADAFSWSQTFESLVPV

DGPTALIVSDGADHRRRRSVVAPGLRHHHVQRYVATMVSNIDTVIDGWQPGQRLDIYQEL

RSAVRRSTAESLFGQRLAVHSDFLGEQLQPLLDLTRRPPQVMRLQQRVNSPGWRRAMAAR

KRIDDLIDAQIADARTAPRPDDHMLTTLISGCSEEGTTLSDNEIRDSIVSLITAGYETTS

GALAWAIYALLTVPGTWESAASEVARVLGGRVPAADDLSALTYLNGVVHETLRLYSPGVI

SARRVLRDLWFDGHRIRAGRLLIFSAYVTHRLPEIWPEPTEFRPLRWDPNAADYRKPAPH

EFIPFSGGLHRCIGAVMATTEMTVILARLVARAMLQLPAQRTHRIRAANFAALRPWPGLT

VEIRKSAPAQ

>CYP139A1(2589727979)Mycobacterium tuberculosis TKK-01-0094

MRYPLGEALLALYRWRGPLINAGVGGHGYTYLLGAEANRFVFANADAFSWSQTFESLVPV

DGPTALIVSDGADHRRRRSVVAPGLRHHHVQRYVATMVSNIDTVIDGWQPGQRLDIYQEL

RSAVRRSTAESLFGQRLAVHSDFLGEQLQPLLDLTRRPPQVMRLQQRVNSPGWRRAMAAR

KRIDDLIDAQIADARTAPRPDDHMLTTLISGCSEEGTTLSDNEIRDSIVSLITAGYETTS

GALAWAIYALLTVPGTWESAASEVARVLGGRVPAADDLSALTYLNGVVHETLRLYSPGVI

SARRVLRDLWFDGHRIRAGRLLIFSAYVTHRLPEIWPEPTEFRPLRWDPNAADYRKPAPH

EFIPFSGGLHRCIGAVMATTEMTVILARLVARAMLQLPAQRTHRIRAANFAALRPWPGLT

VEIRKSAPAQ

>CYP139A1(2592361217)Mycobacterium tuberculosis TKK_02_0062

MRYPLGEALLALYRWRGPLINAGVGGHGYTYLLGAEANRFVFANADAFSWSQTFESLVPV

DGPTALIVSDGADHRRRRSVVAPGLRHHHVQRYVATMVSNIDTVIDGWQPGQRLDIYQEL

RSAVRRSTAESLFGQRLAVHSDFLGEQLQPLLDLTRRPPQVMRLQQRVNSPGWRRAMAAR

KRIDDLIDAQIADARTAPRPDDHMLTTLISGCSEEGTTLSDNEIRDSIVSLITAGYETTS

GALAWAIYALLTVPGTWESAASEVARVLGGRVPAADDLSALTYLNGVVHETLRLYSPGVI

SARRVLRDLWFDGHRIRAGRLLIFSAYVTHRLPEIWPEPTEFRPLRWDPNAADYRKPAPH

EFIPFSGGLHRCIGAVMATTEMTVILARLVARAMLQLPAQRTHRIRAANFAALRPWPGLT

VEIRKSAPAQ

>CYP139A1(2592442711)Mycobacterium tuberculosis TKK_02_0001

MRYPLGEALLALYRWRGPLINAGVGGHGYTYLLGAEANRFVFANADAFSWSQTFESLVPV

DGPTALIVSDGADHRRRRSVVAPGLRHHHVQRYVATMVSNIDTVIDGWQPGQRLDIYQEL

RSAVRRSTAESLFGQRLAVHSDFLGEQLQPLLDLTRRPPQVMRLQQRVNSPGWRRAMAAR

KRIDDLIDAQIADARTAPRPDDHMLTTLISGCSEEGTTLSDNEIRDSIVSLITAGYETTS

GALAWAIYALLTVPGTWESAASEVARVLGGRVPAADDLSALTYLNGVVHETLRLYSPGVI

SARRVLRDLWFDGHRIRAGRLLIFSAYVTHRLPEIWPEPTEFRPLRWDPNAADYRKPAPH

EFIPFSGGLHRCIGAVMATTEMTVILARLVARAMLQLPAQRTHRIRAANFAALRPWPGLT

VEIRKSAPAQ

>CYP139A1(2537735281)Mycobacterium tuberculosis CDC1551A

MRTYRTVRYPLGEALLALYRWRGPLINAGVGGHGYTYLLGAEANRFVFANADAFSWSQTF

ESLVPVDGPTALIVSDGADHRRRRSVVAPGLRHHHVQRYVATMVSNIDTVIDGWQPGQRL

DIYQELRSAVRRSTAESLFGQRLAVHSDFLGEQLQPLLDLTRRPPQVMRLQQRVNSPGWR

RAMAARKRIDDLIDAQIADARTAPRPDDHMLTTLISGCSEEGTTLSDNEIRDSIVSLITA

GYETTSGALAWAIYALLTVPGTWESAASEVARVLGGRVPAADDLSALTYLNGVVHETLRL

YSPGVISARRVLRDLWFDGHRIRAGRLLIFSAYVTHRLPEIWPEPTEFRPLRWDPNAADY

RKPAPHEFIPFSGGLHRCIGAVMATTEMTVILARLVARAMLQLPAQRTHRIRAANFAALR

PWPGLTVEIRKSAPAQ

>CYP139A1(2555325132)Mycobacterium tuberculosis PanR0604

MRTYRTVRYPLGEALLALYRWRGPLINAGVGGHGYTYLLGAEANRFVFANADAFSWSQTF

ESLVPVDGPTALIVSDGADHRRRRSVVAPGLRHHHVQRYVATMVSNIDTVIDGWQPGQRL

DIYQELRSAVRRSTAESLFGQRLAVHSDFLGEQLQPLLDLTRRPPQVMRLQQRVNSPGWR

RAMAARKRIDDLIDAQIADARTAPRPDDHMLTTLISGCSEEGTTLSDNEIRDSIVSLITA

GYETTSGALAWAIYALLTVPGTWESAASEVARVLGGRVPAADDLSALTYLNGVVHETLRL

YSPGVISARRVLRDLWFDGHRIRAGRLLIFSAYVTHRLPEIWPEPTEFRPLRWDPNAADY

RKPAPHEFIPFSGGLHRCIGAVMATTEMTVILARLVARAMLQLPAQRTHRIRAANFAALR

PWPGLTVEIRKSAPAQ

>CYP139A1(2566259019)Mycobacterium tuberculosis BT2

MRTYRTVRYPLGEALLALYRWRGPLINAGVGGHGYTYLLGAEANRFVFANADAFSWSQTF

ESLVPVDGPTALIVSDGADHRRRRSVVAPGLRHHHVQRYVATMVSNIDTVIDGWQPGQRL

DIYQELRSAVRRSTAESLFGQRLAVHSDFLGEQLQPLLDLTRRPPQVMRLQQRVNSPGWR

RAMAARKRIDDLIDAQIADARTAPRPDDHMLTTLISGCSEEGTTLSDNEIRDSIVSLITA

GYETTSGALAWAIYALLTVPGTWESAASEVARVLGGRVPAADDLSALTYLNGVVHETLRL

YSPGVISARRVLRDLWFDGHRIRAGRLLIFSAYVTHRLPEIWPEPTEFRPLRWDPNAADY

RKPAPHEFIPFSGGLHRCIGAVMATTEMTVILARLVARAMLQLPAQRTHRIRAANFAALR

PWPGLTVEIRKSAPAQ

>CYP139A1(2555164435)Mycobacterium tuberculosis PanR0209

MRTYRTVRYPLGEALLALYRWRGPLINAGVGGHGYTYLLGAEANRFVFANADAFSWSQTF

ESLVPVDGPTALIVSDGADHRRRRSVVAPGLRHHHVQRYVATMVSNIDTVIDGWQPGQRL

DIYQELRSAVRRSTAESLFGQRLAVHSDFLGEQLQPLLDLTRRPPQVMRLQQRVNSPGWR

RAMAARKRIDDLIDAQIADARTAPRPDDHMLTTLISGCSEEGTTLSDNEIRDSIVSLITA

GYETTSGALAWAIYALLTVPGTWESAASEVARVLGGRVPAADDLSALTYLNGVVHETLRL

YSPGVISARRVLRDLWFDGHRIRAGRLLIFSAYVTHRLPEIWPEPTEFRPLRWDPNAADY

RKPAPHEFIPFSGGLHRCIGAVMATTEMTVILARLVARAMLQLPAQRTHRIRAANFAALR

PWPGLTVEIRKSAPAQ

>CYP139A1(2555583039)Mycobacterium tuberculosis PanR0410

MRTYRTVRYPLGEALLALYRWRGPLINAGVGGHGYTYLLGAEANRFVFANADAFSWSQTF

ESLVPVDGPTALIVSDGADHRRRRSVVAPGLRHHHVQRYVATMVSNIDTVIDGWQPGQRL

DIYQELRSAVRRSTAESLFGQRLAVHSDFLGEQLQPLLDLTRRPPQVMRLQQRVNSPGWR

RAMAARKRIDDLIDAQIADARTAPRPDDHMLTTLISGCSEEGTTLSDNEIRDSIVSLITA

GYETTSGALAWAIYALLTVPGTWESAASEVARVLGGRVPAADDLSALTYLNGVVHETLRL

YSPGVISARRVLRDLWFDGHRIRAGRLLIFSAYVTHRLPEIWPEPTEFRPLRWDPNAADY

RKPAPHEFIPFSGGLHRCIGAVMATTEMTVILARLVARAMLQLPAQRTHRIRAANFAALR

PWPGLTVEIRKSAPAQ

>CYP139A1(2555295099)Mycobacterium tuberculosis PanR0309

MRTYRTVRYPLGEALLALYRWRGPLINAGVGGHGYTYLLGAEANRFVFANADAFSWSQTF

ESLVPVDGPTALIVSDGADHRRRRSVVAPGLRHHHVQRYVATMVSNIDTVIDGWQPGQRL

DIYQELRSAVRRSTAESLFGQRLAVHSDFLGEQLQPLLDLTRRPPQVMRLQQRVNSPGWR

RAMAARKRIDDLIDAQIADARTAPRPDDHMLTTLISGCSEEGTTLSDNEIRDSIVSLITA

GYETTSGALAWAIYALLTVPGTWESAASEVARVLGGRVPAADDLSALTYLNGVVHETLRL

YSPGVISARRVLRDLWFDGHRIRAGRLLIFSAYVTHRLPEIWPEPTEFRPLRWDPNAADY

RKPAPHEFIPFSGGLHRCIGAVMATTEMTVILARLVARAMLQLPAQRTHRIRAANFAALR

PWPGLTVKIRKSAPAQ

>CYP139A1(2555337752)Mycobacterium tuberculosis PanR0602

MRTYRTVRYPLGEALLALYRWRGPLINAGVGGHGYTYLLGAEANRFVFANADAFSWSQTF

ESLVPVDGPTALIVSDGADHRRRRSVVAPGLRHHHVQRYVATMVSNIDTVIDGWQPGQRL

DIYQELRSAVRRSTAESLFGQRLAVHSDFLGEQLQPLLDLTRRPPQVMRLQQRVNSPGWR

RAMAARKRIDDLIDAQIADARTAPRPDDHMLTTLISGCSEEGTTLSDNEIRDSIVSLITA

GYETTSGALAWAIYALLTVPGTWESAASEVARVLGGRVPAADDLSALTYLNGVVHETLRL

YSPGVISARRVLRDLWFDGHRIRAGRLLIFSAYVTHRLPEIWPEPTEFRPLRWDPNAADY

RKPAPHEFIPFSGGLHRCIGAVMATTEMTVILARLVARAMLQLPAQRTHRIRAANFAALR

PWPGLTVEIRKSAPAQ

>CYP139A1(2555516018)Mycobacterium tuberculosis PanR0405

MRTYRTVRYPLGEALLALYRWRGPLINAGVGGHGYTYLLGAEANRFVFANADAFSWSQTF

ESLVPVDGPTALIVSDGADHRRRRSVVAPGLRHHHVQRYVATMVSNIDTVIDGWQPGQRL

DIYQELRSAVRRSTAESLFGQRLAVHSDFLGEQLQPLLDLTRRPPQVMRLQQRVNSPGWR

RAMAARKRIDDLIDAQIADARTAPRPDDHMLTTLISGCSEEGTTLSDNEIRDSIVSLITA

GYETTSGALAWAIYALLTVPGTWESAASEVARVLGGRVPAADDLSALTYLNGVVHETLRL

YSPGVISARRVLRDLWFDGHRIRAGRLLIFSAYVTHRLPEIWPEPTEFRPLRWDPNAADY

RKPAPHEFIPFSGGLHRCIGAVMATTEMTVILARLVARAMLQLPAQRTHRIRAANFAALR

PWPGLTVEIRKSAPAQ

>CYP139A1(2576196505)Mycobacterium tuberculosis BTB13-063

MRYPLGEALLALYRWRGPLINAGVGGHGYTYLLGAEANRFVFANADAFSWSQTFESLVPV

DGPTALIVSDGADHRRRRSVVAPGLRHHHVQRYVATMVSNIDTVIDGWQPGQRLDIYQEL

RSAVRRSTAESLFGQRLAVHSDFLGEQLQPLLDLTRRPPQVMRLQQRVNSPGWRRAMAAR

KRIDDLIDAQIADARTAPRPDDHMLTTLISGCSEEGTTLSDNEIRDSIVSLITAGYETTS

GALAWAIYALLTVPGTWESAASEVARVLGGRVPAADDLSTLTYLNGVVHETLRLYSPGVI

SARRVLRDLWFDGHRIRAGRLLIFSAYVTHRLPEIWPEPTEFRPLRWDPNAADYRKPAPH

EFIPFSGGLHRCIGAVMATTEMTVILARLVARAMLQLPAQRTHRIRAANFAALRPWPGLT

VEIRKSAPAQ

>CYP139A1(2555417623)Mycobacterium tuberculosis PanR0702

MRTYRTVRYPLGEALLALYRWRGPLINAGVGGHGYTYLLGAEANRFVFANADAFSWSQTF

ESLVPVDGPTALIVSDGADHRRRRSVVAPGLRHHHVQRYVATMVSNIDTVIDGWQPGQRL

DIYQELRSAVRRSTAESLFGQRLAVHSDFLGEQLQPLLDLTRRPPQVMRLQQRVNSPGWR

RAMAARKRIDDLIDAQIADARTAPRPDDHMLTTLISGCSEEGTTLSDNEIRDSIVSLITA

GYETTSGALAWAIYALLTVPGTWESAASEVARVLGGRVPAADDLSALTYLNGVVHETLRL

YSPGVISARRVLRDLWFDGHRIRAGRLLIFSAYVTHRLPEIWPEPTEFRPLRWDPNAADY

RKPAPHEFIPFSGGLHRCIGAVMATTEMTVILARLVARAMLQLPAQRTHRIRAANFAALR

PWPGLTVEIRKSAPAQ

>CYP139A1(2555430098)Mycobacterium tuberculosis PanR0909

MRTYRTVRYPLGEALLALYRWRGPLINAGVGGHGYTYLLGAEANRFVFANADAFSWSQTF

ESLVPVDGPTALIVSDGADHRRRRSVVAPGLRHHHVQRYVATMVSNIDTVIDGWQPGQRL

DIYQELRSAVRRSTAESLFGQRLAVHSDFLGEQLQPLLDLTRRPPQVMRLQQRVNSPGWR

RAMAARKRIDDLIDAQIADARTAPRPDDHMLTTLISGCSEEGTTLSDNEIRDSIVSLITA

GYETTSGALAWAIYALLTVPGTWESAASEVARVLGGRVPAADDLSALTYLNGVVHETLRL

YSPGVISARRVLRDLWFDGHRIRAGRLLIFSAYVTHRLPEIWPEPTEFRPLRWDPNAADY

RKPAPHEFIPFSGGLHRCIGAVMATTEMTVILARLVARAMLQLPAQRTHRIRAANFAALR

PWPGLTVEIRKSAPAQ

>CYP139A1(2547880750)Mycobacterium tuberculosis CTRI-4

MRTYRTVRYPLGEALLALYRWRGPLINAGVGGHGYTYLLGAEANRFVFANADAFSWSQTF

ESLVPVDGPTALIVSDGADHRRRRSVVAPGLRHHHVQRYVATMVSNIDTVIDGWQPGQRL

DIYQELRSAVRRSTAESLFGQRLAVHSDFLGEQLQPLLDLTRRPPQVMRLQQRVNSPGWR

RAMAARKRIDDLIDAQIADARTAPRPDDHMLTTLISGCSEEGTTLSDNEIRDSIVSLITA

GYETTSGALAWAIYALLTVPGTWESAASEVARVLGGRVPAADDLSALTYLNGVVHETLRL

YSPGVISARRVLRDLWFDGHRIRAGRLLIFSAYVTHRLPEIWPEPTEFRPLRWDPNAADY

RKPAPHEFIPFSGGLHRCIGAVMATTEMTVILARLVARAMLQLPAQRTHRIRAANFAALR

PWPGLTVEIRKSAPAQ

>CYP139A1(2559163499)Mycobacterium tuberculosis BS1

MRTYRTVRYPLGEALLALYRWRGPLINAGVGGHGYTYLLGAEANRFVFANADAFSWSQTF

ESLVPVDGPTALIVSDGADHRRRRSVVAPGLRHHHVQRYVATMVSNIDTVIDGWQPGQRL

DIYQELRSAVRRSTAESLFGQRLAVHSDFLGEQLQPLLDLTRRPPQVMRLQQRVNSPGWR

RAMAARKRIDDLIDAQIADARTAPRPDDHMLTTLISGCSEEGTTLSDNEIRDSIVSLITA

GYETTSGALAWAIYALLTVPGTWESAASEVARVLGGRVPAADDLSALTYLNGVVHETLRL

YSPGVISARRVLRDLWFDGHRIRAGRLLIFSAYVTHRLPEIWPEPTEFRPLRWDPNAADY

RKPAPHEFIPFSGGLHRCIGAVMATTEMTVILARLVARAMLQLPAQRTHRIRAANFAALR

PWPGLTVEIRKSAPAQ

>CYP139A1(2527056892)Mycobacterium tuberculosis H37Rv

MRYPLGEALLALYRWRGPLINAGVGGHGYTYLLGAEANRFVFANADAFSWSQTFESLVPV

DGPTALIVSDGADHRRRRSVVAPGLRHHHVQRYVATMVSNIDTVIDGWQPGQRLDIYQEL

RSAVRRSTAESLFGQRLAVHSDFLGEQLQPLLDLTRRPPQVMRLQQRVNSPGWRRAMAAR

KRIDDLIDAQIADARTAPRPDDHMLTTLISGCSEEGTTLSDNEIRDSIVSLITAGYETTS

GALAWAIYALLTVPGTWESAASEVARVLGGRVPAADDLSALTYLNGVVHETLRLYSPGVI

SARRVLRDLWFDGHRIRAGRLLIFSAYVTHRLPEIWPEPTEFRPLRWDPNAADYRKPAPH

EFIPFSGGLHRCIGAVMATTEMTVILARLVARAMLQLPAQRTHRIRAANFAALRPWPGLT

VEIRKSAPAQ

>CYP139A1(2574675630)Mycobacterium tuberculosis TRUG0004

MRYPLGEALLALYRWRGPLINAGVGGHGYTYLLGAEANRFVFANADAFSWSQTFESLVPV

DGPTALIVSDGADHRRRRSVVAPGLRHHHVQRYVATMVSNIDTVIDGWQPGQRLDIYQEL

RSAVRRSTAESLFGQRLAVHSDFLGEQLQPLLDLTRRPPQVMRLQQRVNSPGWRRAMAAR

KRIDDLIDAQIADARTAPRPDDHMLTTLISGCSEEGTTLSDNEIRDSIVSLITAGYETTS

GALAWAIYALLTVPGTWESAASEVARVLGGRVPAADDLSALTYLNGVVHETLRLYSPGVI

SARRVLRDLWFDGHRIRAGRLLIFSAYVTHRLPEIWPEPTEFRPLRWDPNAADYRKPAPH

EFIPFSGGLHRCIGAVMATTEMTVILARLVARAMLQLPAQRTHRIRAANFAALRPWPGLT

VEIRKSAPAQ

>CYP139A1(2574700588)Mycobacterium tuberculosis TB_RSA03

MRYPLGEALLALYRWRGPLINAGVGGHGYTYLLGAEANRFVFANADAFSWSQTFESLVPV

DGPTALIVSDGADHRRRRSVVAPGLRHHHVQRYVATMVSNIDTVIDGWQPGQRLDIYQEL

RSAVRRSTAESLFGQRLAVHSDFLGEQLQPLLDLTRRPPQVMRLQQRVNSPGWRRAMAAR

KRIDDLIDAQIADARTAPRPDDHMLTTLISGCSEEGTTLSDNEIRDSIVSLITAGYETTS

GALAWAIYALLTVPGTWESAASEVARVLGGRVPAADDLSALTYLNGVVHETLRLYSPGVI

SARRVLRDLWFDGHRIRAGRLLIFSAYVTHRLPEIWPEPTEFRPLRWDPNAADYRKPAPH

EFIPFSGGLHRCIGAVMATTEMTVILARLVARAMLQLPAQRTHRIRAANFAALRPWPGLT

VEIRKSAPAQ

>CYP139A1(2574830606)Mycobacterium tuberculosis M2416

MRYPLGEALLALYRWRGPLINAGVGGHGYTYLLGAEANRFVFANADAFSWSQTFESLVPV

DGPTALIVSDGADHRRRRSVVAPGLRHHHVQRYVATMVSNIDTVIDGWQPGQRLDIYQEL

RSAVRRSTAESLFGQRLAVHSDFLGEQLQPLLDLTRRPPQVMRLQQRVNSPGWRRAMAAR

KRIDDLIDAQIADARTAPRPDDHMLTTLISGCSEEGTTLSDNEIRDSIVSLITAGYETTS

GALAWAIYALLTVPGTWESAASEVARVLGGRVPAADDLSALTYLNGVVHETLRLYSPGVI

SARRVLRDLWFDGHRIRAGRLLIFSAYVTHRLPEIWPEPTEFRPLRWDPNAADYRKPAPH

EFIPFSGGLHRCIGAVMATTEMTVILARLVARAMLQLPAQRTHRIRAANFAALRPWPGLT

VEIRKSAPAQ

>CYP139A1(2574928549)Mycobacterium tuberculosis TKK_04_0075

MRYPLGEALLALYRWRGPLINAGVGGHGYTYLLGAEANRFVFANADAFSWSQTFESLVPV

DGPTALIVSDGADHRRRRSVVAPGLRHHHVQRYVATMVSNIDTVIDGWQPGQRLDIYQEL

RSAVRRSTAESLFGQRLAVHSDFLGEQLQPLLDLTRRPPQVMRLQQRVNSPGWRRAMAAR

KRIDDLIDAQIADARTAPRPDDHMLTTLISGCSEEGTTLSDNEIRDSIVSLITAGYETTS

GALAWAIYALLTVPGTWESAASEVARVLGGRVPAADDLSALTYLNGVVHETLRLYSPGVI

SARRVLRDLWFDGHRIRAGRLLIFSAYVTHRLPEIWPEPTEFRPLRWDPNAADYRKPAPH

EFIPFSGGLHRCIGAVMATTEMTVILARLVARAMLQLPAQRTHRIRAANFAALRPWPGLT

VEIRKSAPAQ

>CYP139A1(2575381682)Mycobacterium tuberculosis TBR28

MRYPLGEALLALYRWRGPLINAGVGGHGYTYLLGAEANRFVFANADAFSWSQTFESLVPV

DGPTALIVSDGADHRRRRSVVAPGLRHHHVQRYVATMVSNIDTVIDGWQPGQRLDIYQEL

RSAVRRSTAESLFGQRLAVHSDFLGEQLQPLLDLTRRPPQVMRLQQRVNSPGWRRAMAAR

KRIDDLIDAQIADARTAPRPDDHMLTTLISGCSEEGTTLSDNEIRDSIVSLITAGYETTS

GALAWAIYALLTVPGTWESAASEVARVLGGRVPAADDLSALTYLNGVVHETLRLYSPGVI

SARRVLRDLWFDGHRIRAGRLLIFSAYVTHRLPEIWPEPTEFRPLRWDPNAADYRKPAPH

EFIPFSGGLHRCIGAVMATTEMTVILARLVARAMLQLPAQRTHRIRAANFAALRPWPGLT

VEIRKSAPAQ

>CYP139A1(2575663406)Mycobacterium tuberculosis M1233

MRYPLGEALLALYRWRGPLINAGVGGHGYTYLLGAEANRFVFANADAFSWSQTFESLVPV

DGPTALIVSDGADHRRRRSVVAPGLRHHHVQRYVATMVSNIDTVIDGWQPGQRLDIYQEL

RSAVRRSTAESLFGQRLAVHSDFLGEQLQPLLDLTRRPPQVMRLQQRVNSPGWRRAMAAR

KRIDDLIDAQIADARTAPRPDDHMLTTLISGCSEEGTTLSDNEIRDSIVSLITAGYETTS

GALAWAIYALLTVPGTWESAASEVARVLGGRVPAADDLSALTYLNGVVHETLRLYSPGVI

SARRVLRDLWFDGHRIRAGRLLIFSAYVTHRLPEIWPEPTEFRPLRWDPNAADYRKPAPH

EFIPFSGGLHRCIGAVMATTEMTVILARLVARAMLQLPAQRTHRIRAANFAALRPWPGLT

VEIRKSAPAQ

>CYP139A1(2576431117)Mycobacterium tuberculosis BTB13-222

MRYPLGEALLALYRWRGPLINAGVGGHGYTYLLGAEANRFVFANADAFSWSQTFESLVPV

DGPTALIVSDGADHRRRRSVVAPGLRHHHVQRYVATMVSNIDTVIDGWQPGQRLDIYQEL

RSAVRRSTAESLFGQRLAVHSDFLGEQLQPLLDLTRRPPQVMRLQQRVNSPGWRRAMAAR

KRIDDLIDAQIADARTAPRPDDHMLTTLISGCSEEGTTLSDNEIRDSIVSLITAGYETTS

GALAWAIYALLTVPGTWESAASEVARVLGGRVPAADDLSALTYLNGVVHETLRLYSPGVI

SARRVLRDLWFDGHRIRAGRLLIFSAYVTHRLPEIWPEPTEFRPLRWDPNAADYRKPAPH

EFIPFSGGLHRCIGAVMATTEMTVILARLVARAMLQLPAQRTHRIRAANFAALRPWPGLT

VEIRKSAPAQ

>CYP139A1(2576553939)Mycobacterium tuberculosis M1007

MRYPLGEALLALYRWRGPLINAGVGGHGYTYLLGAEANRFVFANADAFSWSQTFESLVPV

DGPTALIVSDGADHRRRRSVVAPGLRHHHVQRYVATMVSNIDTVIDGWQPGQRLDIYQEL

RSAVRRSTAESLFGQRLAVHSDFLGEQLQPLLDLTRRPPQVMRLQQRVNSPGWRRAMAAR

KRIDDLIDAQIADARTAPRPDDHMLTTLISGCSEEGTTLSDNEIRDSIVSLITAGYETTS

GALAWAIYALLTVPGTWESAASEVARVLGGRVPAADDLSALTYLNGVVHETLRLYSPGVI

SARRVLRDLWFDGHRIRAGRLLIFSAYVTHRLPEIWPEPTEFRPLRWDPNAADYRKPAPH

EFIPFSGGLHRCIGAVMATTEMTVILARLVARAMLQLPAQRTHRIRAANFAALRPWPGLT

VEIRKSAPAQ

>CYP139A1(2577269438)Mycobacterium tuberculosis M1004

MRYPLGEALLALYRWRGPLINAGVGGHGYTYLLGAEANRFVFANADAFSWSQTFESLVPV

DGPTALIVSDGADHRRRRSVVAPGLRHHHVQRYVATMVSNIDTVIDGWQPGQRLDIYQEL

RSAVRRSTAESLFGQRLAVHSDFLGEQLQPLLDLTRRPPQVMRLQQRVNSPGWRRAMAAR

KRIDDLIDAQIADARTAPRPDDHMLTTLISGCSEEGTTLSDNEIRDSIVSLITAGYETTS

GALAWAIYALLTVPGTWESAASEVARVLGGRVPAADDLSALTYLNGVVHETLRLYSPGVI

SARRVLRDLWFDGHRIRAGRLLIFSAYVTHRLPEIWPEPTEFRPLRWDPNAADYRKPAPH

EFIPFSGGLHRCIGAVMATTEMTVILARLVARAMLQLPAQRTHRIRAANFAALRPWPGLT

VEIRKSAPAQ

>CYP139A1(2584870715)Mycobacterium tuberculosis MD16555

MRYPLGEALLALYRWRGPLINAGVGGHGYTYLLGAEANRFVFANADAFSWSQTFESLVPV

DGPTALIVSDGADHRRRRSVVAPGLRHHHVQRYVATMVSNIDTVIDGWQPGQRLDIYQEL

RSAVRRSTAESLFGQRLAVHSDFLGEQLQPLLDLTRRPPQVMRLQQRVNSPGWRRAMAAR

KRIDDLIDAQIADARTAPRPDDHMLTTLISGCSEEGTTLSDNEIRDSIVSLITAGYETTS

GALAWAIYALLTVPGTWESAASEVARVLGGRVPAADDLSALTYLNGVVHETLRLYSPGVI

SARRVLRDLWFDGHRIRAGRLLIFSAYVTHRLPEIWPEPTEFRPLRWDPNAADYRKPAPH

EFIPFSGGLHRCIGAVMATTEMTVILARLVARAMLQLPAQRTHRIRAANFAALRPWPGLT

VEIRKSAPAQ

>CYP139A1(2589036086)Mycobacterium tuberculosis TBR4

MRYPLGEALLALYRWRGPLINAGVGGHGYTYLLGAEANRFVFANADAFSWSQTFESLVPV

DGPTALIVSDGADHRRRRSVVAPGLRHHHVQRYVATMVSNIDTVIDGWQPGQRLDIYQEL

RSAVRRSTAESLFGQRLAVHSDFLGEQLQPLLDLTRRPPQVMRLQQRVNSPGWRRAMAAR

KRIDDLIDAQIADARTAPRPDDHMLTTLISGCSEEGTTLSDNEIRDSIVSLITAGYETTS

GALAWAIYALLTVPGTWESAASEVARVLGGRVPAADDLSALTYLNGVVHETLRLYSPGVI

SARRVLRDLWFDGHRIRAGRLLIFSAYVTHRLPEIWPEPTEFRPLRWDPNAADYRKPAPH

EFIPFSGGLHRCIGAVMATTEMTVILARLVARAMLQLPAQRTHRIRAANFAALRPWPGLT

VEIRKSAPAQ

>CYP139A1(2590101605)Mycobacterium tuberculosis MAL020147

MRYPLGEALLALYRWRGPLINAGVGGHGYTYLLGAEANRFVFANADAFSWSQTFESLVPV

DGPTALIVSDGADHRRRRSVVAPGLRHHHVQRYVATMVSNIDTVIDGWQPGQRLDIYQEL

RSAVRRSTAESLFGQRLAVHSDFLGEQLQPLLDLTRRPPQVMRLQQRVNSPGWRRAMAAR

KRIDDLIDAQIADARTAPRPDDHMLTTLISGCSEEGTTLSDNEIRDSIVSLITAGYETTS

GALAWAIYALLTVPGTWESAASEVARVLGGRVPAADDLSALTYLNGVVHETLRLYSPGVI

SARRVLRDLWFDGHRIRAGRLLIFSAYVTHRLPEIWPEPTEFRPLRWDPNAADYRKPAPH

EFIPFSGGLHRCIGAVMATTEMTVILARLVARAMLQLPAQRTHRIRAANFAALRPWPGLT

VEIRKSAPAQ

>CYP139A1(2590354422)Mycobacterium tuberculosis OFXR-30

MRYPLGEALLALYRWRGPLINAGVGGHGYTYLLGAEANRFVFANADAFSWSQTFESLVPV

DGPTALIVSDGADHRRRRSVVAPGLRHHHVQRYVATMVSNIDTVIDGWQPGQRLDIYQEL

RSAVRRSTAESLFGQRLAVHSDFLGEQLQPLLDLTRRPPQVMRLQQRVNSPGWRRAMAAR

KRIDDLIDAQIADARTAPRPDDHMLTTLISGCSEEGTTLSDNEIRDSIVSLITAGYETTS

GALAWAIYALLTVPGTWESAASEVARVLGGRVPAADDLSALTYLNGVVHETLRLYSPGVI

SARRVLRDLWFDGHRIRAGRLLIFSAYVTHRLPEIWPEPTEFRPLRWDPNAADYRKPAPH

EFIPFSGGLHRCIGAVMATTEMTVILARLVARAMLQLPAQRTHRIRAANFAALRPWPGLT

VEIRKSAPAQ

>CYP139A1(648490022)Mycobacterium tuberculosis SUMu012

MRYPLGEALLALYRWRGPLINAGVGGHGYTYLLGAEANRFVFANADAFSWSQTFESLVPV

DGPTALIVSDGADHRRRRSVVAPGLRHHHVQRYVATMVSNIDTVIDGWQPGQRLDIYQEL

RSAVRRSTAESLFGQRLAVHSDFLGEQLQPLLDLTRRPPQVMRLQQRVNSPGWRRAMAAR

KRIDDLIDAQIADARTAPRPDDHMLTTLISGCSEEGTTLSDNEIRDSIVSLITAGYETTS

GALAWAIYALLTVPGTWESAASEVARVLGGRVPAADDLSALTYLNGVVHETLRLYSPGVI

SARRVLRDLWFDGHRIRAGRLLIFSAYVTHRLPEIWPEPTEFRPLRWDPNAADYRKPAPH

EFIPFSGGLHRCIGAVMATTEMTVILARLVARAMLQLPAQRTHRIRAANFAALRPWPGLT

VEIRKSAPAQ

>CYP139A1(2547317188)*Mycobacterium bovis*BCG-Russia TMC 1022 ATCC 35740

MRTYRTVRYPLGEALLALYRWRGPLINAGVGGHGYTYLLGAEANRFVFANADAFSWSQTF

ESLVPVDGPTALIVSDGADHRRRRSVVAPGLRHHHVQRYVATMVSNIDTVIDGWQPGQRL

DIYQELRSAVRRSTAESLFGQRLAVHSDFLGEQLQPLLDLTRRPPQVMRLQQRVNSPGWR

RAMAARKRIDDLIDAQIADARTAPRPDDHMLTTLISGCSEEGTTLSDNEIRDSIVSLITA

GYETTSGALAWAIYALLTVPGTWESAASEVARVLGGRVPAADDLSALTYLNGVVHETLRL

YSPGVISARRVLRDLWFDGHRIRAGRLLIFSAYVTHRLPEIWPEPTEFRPLRWDPNAADY

RKPAPHEFIPFSGGLHRCIGAVMATTEMTVILARLVARAMLQLPAQRTHRIRAANFAALR

PWPGLTVEIRKSAPAQ

>CYP139A1(2547959756)Mycobacterium tuberculosis HN878

MRTYRTVRYPLGEALLALYRWRGPLINAGVGGHGYTYLLGAEANRFVFANADAFSWSQTF

ESLVPVDGPTALIVSDGADHRRRRSVVAPGLRHHHVQRYVATMVSNIDTVIDGWQPGQRL

DIYQELRSAVRRSTAESLFGQRLAVHSDFLGEQLQPLLDLTRRPPQVMRLQQRVNSPGWR

RAMAARKRIDDLIDAQIADARTAPRPDDHMLTTLISGCSEEGTTLSDNEIRDSIVSLITA

GYETTSGALAWAIYALLTVPGTWESAASEVARVLGGRVPAADDLSALTYLNGVVHETLRL

YSPGVISARRVLRDLWFDGHRIRAGRLLIFSAYVTHRLPEIWPEPTEFRPLRWDPNAADY

RKPAPHEFIPFSGGLHRCIGAVMATTEMTVILARLVARAMLQLPAQRTHRIRAANFAALR

PWPGLTVEIRKSAPAQ

>CYP139A1(2555303475)Mycobacterium tuberculosis PanR0306

MRTYRTVRYPLGEALLALYRWRGPLINAGVGGHGYTYLLGAEANRFVFANADAFSWSQTF

ESLVPVDGPTALIVSDGADHRRRRSVVAPGLRHHHVQRYVATMVSNIDTVIDGWQPGQRL

DIYQELRSAVRRSTAESLFGQRLAVHSDFLGEQLQPLLDLTRRPPQVMRLQQRVNSPGWR

RAMAARKRIDDLIDAQIADARTAPRPDDHMLTTLISGCSEEGTTLSDNEIRDSIVSLITA

GYETTSGALAWAIYALLTVPGTWESAASEVARVLGGRVPAADDLSALTYLNGVVHETLRL

YSPGVISARRVLRDLWFDGHRIRAGRLLIFSAYVTHRLPEIWPEPTEFRPLRWDPNAADY

RKPAPHEFIPFSGGLHRCIGAVMATTEMTVILARLVARAMLQLPAQRTHRIRAANFAALR

PWPGLTVEIRKSAPAQ

>CYP139A1(2555392373)Mycobacterium tuberculosis PanR0804

MRTYRTVRYPLGEALLALYRWRGPLINAGVGGHGYTYLLGAEANRFVFANADAFSWSQTF

ESLVPVDGPTALIVSDGADHRRRRSVVAPGLRHHHVQRYVATMVSNIDTVIDGWQPGQRL

DIYQELRSAVRRSTAESLFGQRLAVHSDFLGEQLQPLLDLTRRPPQVMRLQQRVNSPGWR

RAMAARKRIDDLIDAQIADARTAPRPDDHMLTTLISGCSEEGTTLSDNEIRDSIVSLITA

GYETTSGALAWAIYALLTVPGTWESAASEVARVLGGRVPAADDLSALTYLNGVVHETLRL

YSPGVISARRVLRDLWFDGHRIRAGRLLIFSAYVTHRLPEIWPEPTEFRPLRWDPNAADY

RKPAPHEFIPFSGGLHRCIGAVMATTEMTVILARLVARAMLQLPAQRTHRIRAANFAALR

PWPGLTVEIRKSAPAQ

>CYP139A1(2566985178)*Mycobacterium canettii* CIPT 140070005

MRTYRTVRYPLGEALLALYRWRGPLINAGVGGHGYTYLLGAEANRFVFANADAFSWSQTF

ESLVPVDGPTALIVSDGADHRRRRSVVAPGLRHHHVQRYVATMVSNIDTVIDGWQPGQRL

DIYQELRSAVRRSTAESLFGQRLAVHSDFLGEQLQPLLDLTRRPPQVMRLQQRVNSPGWR

RAMAARKRIDDLIDAQIADARTAPKPDDHMLTTLISGCSEEGTTLSDNEIRDSIVSLITA

GYETTSGALAWAIYALLTVPGTWESAASEVARVLGGRVPAADDLSALTYLNGVVHETLRL

YSPGVISARRVLRDLWFDGHRIRAGRLLIFSAYVTHRLPEIWPEPTEFRPLRWDPNAADY

RKPAPHEFIPFSGGLHRCIGAVMATTEMTVILARLVARAMLQLPAQRTHRIRAANFAALR

PWPGLTVEIRKSAPAQ

>CYP139A1(2555362970)Mycobacterium tuberculosis PanR0805

MRTYRTVRYPLGEALLALYRWRGPLINAGVGGHGYTYLLGAEANRFVFANADAFSWSQTF

ESLVPVDGPTALIVSDGADHRRRRSVVAPGLRHHHVQRYVATMVSNIDTVIDGWQPGQRL

DIYQELRSAVRRSTAESLFGQRLAVHSDFLGEQLQPLLDLTRRPPQVMRLQQRVNSPGWR

RAMAARKRIDDLIDAQIADARTAPRPDDHMLTTLISGCSEEGTTLSDNEIRDSIVSLITA

GYETTSGALAWAIYALLTVPGTWESAASEVARVLGGRVPAADDLSALTYLNGVVHETLRL

YSPGVISARRVLRDLWFDGHRIRAGRLLIFSAYVTHRLPEIWPEPTEFRPLRWDPNAADY

RKPAPHEFIPFSGGLHRCIGAVMATTEMTVILARLVARAMLQLPAQRTHRIRAANFAALR

PWPGLTVEIRKSAPAQ

>CYP139A1(2560449536)Mycobacterium tuberculosis XDR1219

MRTYRTVRYPLGEALLALYRWRGPLINAGVGGHGYTYLLGAEANRFVFANADAFSWSQTF

ESLVPVDGPTALIVSDGADHRRRRSVVAPGLRHHHVQRYVATMVSNIDTVIDGWQPGQRL

DIYQELRSAVRRSTAESLFGQRLAVHSDFLGEQLQPLLDLTRRPPQVMRLQQRVNSPGWR

RAMAARKRIDDLIDAQIADARTAPRPDDHMLTTLISGCSEEGTTLSDNEIRDSIVSLITA

GYETTSGALAWAIYALLTVPGTWESAASEVARVLGGRVPAADDLSALTYLNGVVHETLRL

YSPGVISARRVLRDLWFDGHRIRAGRLLIFSAYVTHRLPEIWPEPTEFRPLRWDPNAADY

RKPAPHEFIPFSGGLHRCIGAVMATTEMTVILARLVARAMLQLPAQRTHRIRAANFAALR

PWPGLTVEIRKSAPAQ

>CYP139A1(2555152706)Mycobacterium tuberculosis PanR0208

MRTYRTVRYPLGEALLALYRWRGPLINAGVGGHGYTYLLGAEANRFVFANADAFSWSQTF

ESLVPVDGPTALIVSDGADHRRRRSVVAPGLRHHHVQRYVATMVSNIDTVIDGWQPGQRL

DIYQELRSAVRRSTAESLFGQRLAVHSDFLGEQLQPLLDLTRRPPQVMRLQQRVNSPGWR

RAMAARKRIDDLIDAQIADARTAPRPDDHMLTTLISGCSEEGTTLSDNEIRDSIVSLITA

GYETTSGALAWAIYALLTVPGTWESAASEVARVLGGRVPAADDLSALTYLNGVVHETLRL

YSPGVISARRVLRDLWFDGHRIRAGRLLIFSAYVTHRLPEIWPEPTEFRPLRWDPNAADY

RKPAPHEFIPFSGGLHRCIGAVMATTEMTVILARLVARAMLQLPAQRTHRIRAANFAALR

PWPGLTVKIRKSAPAQ

>CYP139A1(2555543565)Mycobacterium tuberculosis PanR0403

MRTYRTVRYPLGEALLALYRWRGPLINAGVGGHGYTYLLGAEANRFVFANADAFSWSQTF

ESLVPVDGPTALIVSDGADHRRRRSVVAPGLRHHHVQRYVATMVSNIDTVIDGWQPGQRL

DIYQELRSAVRRSTAESLFGQRLAVHSDFLGEQLQPLLDLTRRPPQVMRLQQRVNSPGWR

RAMAARKRIDDLIDAQIADARTAPRPDDHMLTTLISGCSEEGTTLSDNEIRDSIVSLITA

GYETTSGALAWAIYALLTVPGTWESAASEVARVLGGRVPAADDLSALTYLNGVVHETLRL

YSPGVISARRVLRDLWFDGHRIRAGRLLIFSAYVTHRLPEIWPEPTEFRPLRWDPNAADY

RKPAPHEFIPFSGGLHRCIGAVMATTEMTVILARLVARAMLQLPAQRTHRIRAANFAALR

PWPGLTVEIRKSAPAQ

>CYP139A1(2590081259)Mycobacterium tuberculosis MAL020160

MRYPLGEALLALYRWRGPLINAGVGGHGYTYLLGAEANRFVFANADAFSWSQTFESLVPV

DGPTALIVSDGADHRRRRSVVAPGLRHHHVQRYVATMVSNIDTVIDGWQPGQRLDIYQEL

RSAVRRSTAESLFGQRLAVHSDFLGEQLQPLLDLTRRPPQVMRLQQRVNSPGWRRAMAAR

KRIDDLIDAQIADARTAPRPDDHMLTTLISGCSEEGTTLSDNEIRDSIVSLITAGYETTS

GALAWAIYALLTVPGTWESAASEVARVLGGRVPAADDLSALTYLNGVVHETLRLYSPGVI

SARRVLRDLWFDGHRIRAGRLLIFSAYVTHRLPEIWPEPTEFRPLRWDPNAADYRKPAPH

EFIPFSGGLHRFIGAVMATTEMTVILARLVARAMLQLPAQRTHRIRAANFAALRPWPGLT

VEIRKSAPAQ

>CYP139A1(2547311116)*Mycobacterium bovis*BCG-Denmark TMC 1010, ATCC 35733

MRTYRTVRYPLGEALLALYRWRGPLINAGVGGHGYTYLLGAEANRFVFANADAFSWSQTF

ESLVPVDGPTALIVSDGADHRRRRSVVAPGLRHHHVQRYVATMVSNIDTVIDGWQPGQRL

DIYQELRSAVRRSTAESLFGQRLAVHSDFLGEQLQPLLDLTRRPPQVMRLQQRVNSPGWR

RAMAARKRIDDLIDAQIADARTAPRPDDHMLTTLISGCSEEGTTLSDNEIRDSIVSLITA

GYETTSGALAWAIYALLTVPGTWESAASEVARVLGGRVPAADDLSALTYLNGVVHETLRL

YSPGVISARRVLRDLWFDGHRIRAGRLLIFSAYVTHRLPEIWPEPTEFRPLRWDPNAADY

RKPAPHEFIPFSGGLHRCIGAVMATTEMTVILARLVARAMLQLPAQRTHRIRAANFAALR

PWPGLTVEIRKSAPAQ

>CYP139A1(2548033169)Mycobacterium tuberculosis BTB05-552

MRTYRTVRYPLGEALLALYRWRGPLINAGVGGHGYTYLLGAEANRFVFANADAFSWSQTF

ESLVPVDGPTALIVSDGADHRRRRSVVAPGLRHHHVQRYVATMVSNIDTVIDGWQPGQRL

DIYQELRSAVRRSTAESLFGQRLAVHSDFLGEQLQPLLDLTRRPPQVMRLQQRVNSPGWR

RAMAARKRIDDLIDAQIADARTAPRPDDHMLTTLISGCSEEGTTLSDNEIRDSIVSLITA

GYETTSGALAWAIYALLTVPGTWESAASEVARVLGGRVPAADDLSALTYLNGVVHETLRL

YSPGVISARRVLRDLWFDGHRIRAGRLLIFSAYVTHRLPEIWPEPTEFRPLRWDPNAADY

RKPAPHEFIPFSGGLHRCIGAVMATTEMTVILARLVARAMLQLPAQRTHRIRAANFAALR

PWPGLTVEIRKSAPAQ

>CYP139A1(2548037418)Mycobacterium tuberculosis BTB05-559

MRTYRTVRYPLGEALLALYRWRGPLINAGVGGHGYTYLLGAEANRFVFANADAFSWSQTF

ESLVPVDGPTALIVSDGADHRRRRSVVAPGLRHHHVQRYVATMVSNIDTVIDGWQPGQRL

DIYQELRSAVRRSTAESLFGQRLAVHSDFLGEQLQPLLDLTRRPPQVMRLQQRVNSPGWR

RAMAARKRIDDLIDAQIADARTAPRPDDHMLTTLISGCSEEGTTLSDNEIRDSIVSLITA

GYETTSGALAWAIYALLTVPGTWESAASEVARVLGGRVPAADDLSALTYLNGVVHETLRL

YSPGVISARRVLRDLWFDGHRIRAGRLLIFSAYVTHRLPEIWPEPTEFRPLRWDPNAADY

RKPAPHEFIPFSGGLHRCIGAVMATTEMTVILARLVARAMLQLPAQRTHRIRAANFAALR

PWPGLTVEIRKSAPAQ

>CYP139A1(2555282371)Mycobacterium tuberculosis PanR0301

MRTYRTVRYPLGEALLALYRWRGPLINAGVGGHGYTYLLGAEANRFVFANADAFSWSQTF

ESLVPVDGPTALIVSDGADHRRRRSVVAPGLRHHHVQRYVATMVSNIDTVIDGWQPGQRL

DIYQELRSAVRRSTAESLFGQRLAVHSDFLGEQLQPLLDLTRRPPQVMRLQQRVNSPGWR

RAMAARKRIDDLIDAQIADARTAPRPDDHMLTTLISGCSEEGTTLSDNEIRDSIVSLITA

GYETTSGALAWAIYALLTVPGTWESAASEVARVLGGRVPAADDLSALTYLNGVVHETLRL

YSPGVISARRVLRDLWFDGHRIRAGRLLIFSAYVTHRLPEIWPEPTEFRPLRWDPNAADY

RKPAPHEFIPFSGGLHRCIGAVMATTEMTVILARLVARAMLQLPAQRTHRIRAANFAALR

PWPGLTVEIRKSAPAQ

>CYP139A1(2541569776)*Mycobacterium canettii* CIPT 140070008

MRYPLGEALLALYRWRGPLINAGVGGHGYTYLLGAEANRFVFANADAFSWSQTFESLVPV

DGPTALIVSDGADHRRRRSVVAPGLRHHHVQRYVATMVSNIDTVIDGWQPGQRLDIYQEL

RSAVRRSTAESLFGQRLAVHSDFLGEQLQPLLDLTRRPPQVMRLQQRVNSPGWRRAMAAR

KRIDDLIDAQIADARTAPKPDDHMLTTLISGCSEEGTTLSDNEIRDSIVSLITAGYETTS

GALAWAIYALLTVPGTWESAASEVARVLGGRVPAADDLSALTYLNGVVHETLRLYSPGVI

SARRVLRDLWFDGHRIRAGRLLIFSAYVTHRLPEIWPEPTEFRPLRWDPNAADYRKPAPH

EFIPFSGGLHRCIGAVMATTEMTVILARLVARAMLQLPAQRTHRIRAANFAALRPWPGLT

VEIRKSAPAQ

>CYP139A1(2555299255)Mycobacterium tuberculosis PanR0305

MRTYRTVRYPLGEALLALYRWRGPLINAGVGGHGYTYLLGAEANRFVFANADAFSWSQTF

ESLVPVDGPTALIVSDGADHRRRRSVVAPGLRHHHVQRYVATMVSNIDTVIDGWQPGQRL

DIYQELRSAVRRSTAESLFGQRLAVHSDFLGEQLQPLLDLTRRPPQVMRLQQRVNSPGWR

RAMAARKRIDDLIDAQIADARTAPRPDDHMLTTLISGCSEEGTTLSDNEIRDSIVSLITA

GYETTSGALAWAIYALLTVPGTWESAASEVARVLGGRVPAADDLSALTYLNGVVHETLRL

YSPGVISARRVLRDLWFDGHRIRAGRLLIFSAYVTHRLPEIWPEPTEFRPLRWDPNAADY

RKPAPHEFIPFSGGLHRCIGAVMATTEMTVILARLVARAMLQLPAQRTHRIRAANFAALR

PWPGLTVEIRKSAPAQ

>CYP139A1(2555379694)Mycobacterium tuberculosis PanR0801

MRTYRTVRYPLGEALLALYRWRGPLINAGVGGHGYTYLLGAEANRFVFANADAFSWSQTF

ESLVPVDGPTALIVSDGADHRRRRSVVAPGLRHHHVQRYVATMVSNIDTVIDGWQPGQRL

DIYQELRSAVRRSTAESLFGQRLAVHSDFLGEQLQPLLDLTRRPPQVMRLQQRVNSPGWR

RAMAARKRIDDLIDAQIADARTAPRPDDHMLTTLISGCSEEGTTLSDNEIRDSIVSLITA

GYETTSGALAWAIYALLTVPGTWESAASEVARVLGGRVPAADDLSALTYLNGVVHETLRL

YSPGVISARRVLRDLWFDGHRIRAGRLLIFSAYVTHRLPEIWPEPTEFRPLRWDPNAADY

RKPAPHEFIPFSGGLHRCIGAVMATTEMTVILARLVARAMLQLPAQRTHRIRAANFAALR

PWPGLTVEIRKSAPAQ

>CYP139A1(2555413387)Mycobacterium tuberculosis PanR0611

MRTYRTVRYPLGEALLALYRWRGPLINAGVGGHGYTYLLGAEANRFVFANADAFSWSQTF

ESLVPVDGPTALIVSDGADHRRRRSVVAPGLRHHHVQRYVATMVSNIDTVIDGWQPGQRL

DIYQELRSAVRRSTAESLFGQRLAVHSDFLGEQLQPLLDLTRRPPQVMRLQQRVNSPGWR

RAMAARKRIDDLIDAQIADARTAPRPDDHMLTTLISGCSEEGTTLSDNEIRDSIVSLITA

GYETTSGALAWAIYALLTVPGTWESAASEVARVLGGRVPAADDLSALTYLNGVVHETLRL

YSPGVISARRVLRDLWFDGHRIRAGRLLIFSAYVTHRLPEIWPEPTEFRPLRWDPNAADY

RKPAPHEFIPFSGGLHRCIGAVMATTEMTVILARLVARAMLQLPAQRTHRIRAANFAALR

PWPGLTVEIRKSAPAQ

>CYP139A1(2555587223)Mycobacterium tuberculosis PanR0409

MRTYRTVRYPLGEALLALYRWRGPLINAGVGGHGYTYLLGAEANRFVFANADAFSWSQTF

ESLVPVDGPTALIVSDGADHRRRRSVVAPGLRHHHVQRYVATMVSNIDTVIDGWQPGQRL

DIYQELRSAVRRSTAESLFGQRLAVHSDFLGEQLQPLLDLTRRPPQVMRLQQRVNSPGWR

RAMAARKRIDDLIDAQIADARTAPRPDDHMLTTLISGCSEEGTTLSDNEIRDSIVSLITA

GYETTSGALAWAIYALLTVPGTWESAASEVARVLGGRVPAADDLSALTYLNGVVHETLRL

YSPGVISARRVLRDLWFDGHRIRAGRLLIFSAYVTHRLPEIWPEPTEFRPLRWDPNAADY

RKPAPHEFIPFSGGLHRCIGAVMATTEMTVILARLVARAMLQLPAQRTHRIRAANFAALR

PWPGLTVEIRKSAPAQ

>CYP139A1(2555591399)Mycobacterium tuberculosis PanR0412

MRTYRTVRYPLGEALLALYRWRGPLINAGVGGHGYTYLLGAEANRFVFANADAFSWSQTF

ESLVPVDGPTALIVSDGADHRRRRSVVAPGLRHHHVQRYVATMVSNIDTVIDGWQPGQRL

DIYQELRSAVRRSTAESLFGQRLAVHSDFLGEQLQPLLDLTRRPPQVMRLQQRVNSPGWR

RAMAARKRIDDLIDAQIADARTAPRPDDHMLTTLISGCSEEGTTLSDNEIRDSIVSLITA

GYETTSGALAWAIYALLTVPGTWESAASEVARVLGGRVPAADDLSALTYLNGVVHETLRL

YSPGVISARRVLRDLWFDGHRIRAGRLLIFSAYVTHRLPEIWPEPTEFRPLRWDPNAADY

RKPAPHEFIPFSGGLHRCIGAVMATTEMTVILARLVARAMLQLPAQRTHRIRAANFAALR

PWPGLTVEIRKSAPAQ

>CYP139A1(2547306531)*Mycobacterium bovis*BCG China

MRTYRTVRYPLGEALLALYRWRGPLINAGVGGHGYTYLLGAEANRFVFANADAFSWSQTF

ESLVPVDGPTALIVSDGADHRRRRSVVAPGLRHHHVQRYVATMVSNIDTVIDGWQPGQRL

DIYQELRSAVRRSTAESLFGQRLAVHSDFLGEQLQPLLDLTRRPPQVMRLQQRVNSPGWR

RAMAARKRIDDLIDAQIADARTAPRPDDHMLTTLISGCSEEGTTLSDNEIRDSIVSLITA

GYETTSGALAWAIYALLTVPGTWESAASEVARVLGGRVPAADDLSALTYLNGVVHETLRL

YSPGVISARRVLRDLWFDGHRIRAGRLLIFSAYVTHRLPEIWPEPTEFRPLRWDPNAADY

RKPAPHEFIPFSGGLHRCIGAVMATTEMTVILARLVARAMLQLPAQRTHRIRAANFAALR

PWPGLTVEIRKSAPAQ

>CYP139A1(651088108)Mycobacterium tuberculosis CCDC5180

MRTYRTVRYPLGEALLALYRWRGPLINAGVGGHGYTYLLGAEANRFVFANADAFSWSQTF

ESLVPVDGPTALIVSDGADHRRRRSVVAPGLRHHHVQRYVATMVSNIDTVIDGWQPGQRL

DIYQELRSAVRRSTAESLFGQRLAVHSDFLGEQLQPLLDLTRRPPQVMRLQQRVNSPGWR

RAMAARKRIDDLIDAQIADARTAPRPDDHMLTTLISGCSEEGTTLSDNEIRDSIVSLITA

GYETTSGALAWAIYALLTVPGTWESAASEVARVLGGRVPAADDLSALTYLNGVVHETLRL

YSPGVISARRVLRDLWFDGHRIRAGRLLIFSAYVTHRLPEIWPEPTEFRPLRWDPNAADY

RKPAPHEFIPFSGGLHRCIGAVMATTEMTVILARLVARAMLQLPAQRTHRIRAANFAALR

PWPGLTVEIRKSAPAQ

>CYP139A1(2598067418)Mycobacterium tuberculosis PanR0202

MRTYRTVRYPLGEALLALYRWRGPLINAGVGGHGYTYLLGAEANRFVFANADAFSWSQTF

ESLVPVDGPTALIVSDGADHRRRRSVVAPGLRHHHVQRYVATMVSNIDTVIDGWQPGQRL

DIYQELRSAVRRSTAESLFGQRLAVHSDFLGEQLQPLLDLTRRPPQVMRLQQRVNSPGWR

RAMAARKRIDDLIDAQIADARTAPRPDDHMLTTLISGCSEEGTTLSDNEIRDSIVSLITA

GYETTSGALAWAIYALLTVPGTWESAASEVARVLGGRVPAADDLSALTYLNGVVHETLRL

YSPGVISARRVLRDLWFDGHRIRAGRLLIFSAYVTHRLPEIWPEPTEFRPLRWDPNAADY

RKPAPHEFIPFSGGLHRCIGAVMATTEMTVILARLVARAMLQLPAQRTHRIRAANFAALR

PWPGLTVKIRKSAPAQ

>CYP139A1(2555341950)Mycobacterium tuberculosis PanR0605

MRTYRTVRYPLGEALLALYRWRGPLINAGVGGHGYTYLLGAEANRFVFANADAFSWSQTF

ESLVPVDGPTALIVSDGADHRRRRSVVAPGLRHHHVQRYVATMVSNIDTVIDGWQPGQRL

DIYQELRSAVRRSTAESLFGQRLAVHSDFLGEQLQPLLDLTRRPPQVMRLQQRVNSPGWR

RAMAARKRIDDLIDAQIADARTAPRPDDHMLTTLISGCSEEGTTLSDNEIRDSIVSLITA

GYETTSGALAWAIYALLTVPGTWESAASEVARVLGGRVPAADDLSALTYLNGVVHETLRL

YSPGVISARRVLRDLWFDGHRIRAGRLLIFSAYVTHRLPEIWPEPTEFRPLRWDPNAADY

RKPAPHEFIPFSGGLHRCIGAVMATTEMTVILARLVARAMLQLPAQRTHRIRAANFAALR

PWPGLTVEIRKSAPAQ

>CYP139A1(2555446824)Mycobacterium tuberculosis PanR0906

MRTYRTVRYPLGEALLALYRWRGPLINAGVGGHGYTYLLGAEANRFVFANADAFSWSQTF

ESLVPVDGPTALIVSDGADHRRRRSVVAPGLRHHHVQRYVATMVSNIDTVIDGWQPGQRL

DIYQELRSAVRRSTAESLFGQRLAVHSDFLGEQLQPLLDLTRRPPQVMRLQQRVNSPGWR

RAMAARKRIDDLIDAQIADARTAPRPDDHMLTTLISGCSEEGTTLSDNEIRDSIVSLITA

GYETTSGALAWAIYALLTVPGTWESAASEVARVLGGRVPAADDLSALTYLNGVVHETLRL

YSPGVISARRVLRDLWFDGHRIRAGRLLIFSAYVTHRLPEIWPEPTEFRPLRWDPNAADY

RKPAPHEFIPFSGGLHRCIGAVMATTEMTVILARLVARAMLQLPAQRTHRIRAANFAALR

PWPGLTVEIRKSAPAQ

>CYP139A1(2555556540)Mycobacterium tuberculosis PanR0404

MRTYRTVRYPLGEALLALYRWRGPLINAGVGGHGYTYLLGAEANRFVFANADAFSWSQTF

ESLVPVDGPTALIVSDGADHRRRRSVVAPGLRHHHVQRYVATMVSNIDTVIDGWQPGQRL

DIYQELRSAVRRSTAESLFGQRLAVHSDFLGEQLQPLLDLTRRPPQVMRLQQRVNSPGWR

RAMAARKRIDDLIDAQIADARTAPRPDDHMLTTLISGCSEEGTTLSDNEIRDSIVSLITA

GYETTSGALAWAIYALLTVPGTWESAASEVARVLGGRVPAADDLSALTYLNGVVHETLRL

YSPGVISARRVLRDLWFDGHRIRAGRLLIFSAYVTHRLPEIWPEPTEFRPLRWDPNAADY

RKPAPHEFIPFSGGLHRCIGAVMATTEMTVILARLVARAMLQLPAQRTHRIRAANFAALR

PWPGLTVEIRKSAPAQ

>CYP139A1(2560451827)Mycobacterium tuberculosis WX1

MRTYRTVRYPLGEALLALYRWRGPLINAGVGGHGYTYLLGAEANRFVFANADAFSWSQTF

ESLVPVDGPTALIVSDGADHRRRRSVVAPGLRHHHVQRYVATMVSNIDTVIDGWQPGQRL

DIYQELRSAVRRSTAESLFGQRLAVHSDFLGEQLQPLLDLTRRPPQVMRLQQRVNSPGWR

RAMAARKRIDDLIDAQIADARTAPRPDDHMLTTLISGCSEEGTTLSDNEIRDSIVSLITA

GYETTSGALAWAIYALLTVPGTWESAASEVARVLGGRVPAADDLSALTYLNGVVHETLRL

YSPGVISARRVLRDLWFDGHRIRAGRLLIFSAYVTHRLPEIWPEPTEFRPLRWDPNAADY

RKPAPHEFIPFSGGLHRCIGAVMATTEMTVILARLVARAMLQLPAQRTHRIRAANFAALR

PWPGLTVEIRKSAPAQ

>CYP139A1(646010237)Mycobacterium tuberculosis T46

MFANADAFSWSQTFESLVPVDGPTALIVSDGADHRRRRSVVAPGLRHHHVQRYVATMVSN

IDTVIDGWQPGQRLDIYQELRSAVRRSTAESLFGQRLAVHSDFLGEQLQPLLDLTRRPPQ

VMRLQQRVNSPGWRRAMAARKRIDDLIDAQIADARTAPRPDDHMLTTLISGCSEEGTTLS

DNEIRDSIVSLITAGYETTSGALAWAIYALLTVPGTWESAASEVARVLGGRVPAADDLSA

LTYLNGVVHETLRLYSPGVISARRVLRDLWFDGHRIRAGRLLIFSAYVTHRLPEIWPEPT

EFRPLRWDPNAADYRKPAPHEFIPFSGGLHRCIGAVMATTEMTVILARLVARAMLQLPAQ

RTHRIRAANFAALRPWPGLTVEIRKSAPAQ

>CYP139A1(2540619998)Mycobacterium tuberculosis Erdman

MRTYRTVRYPLGEALLALYRWRGPLINAGVGGHGYTYLLGAEANRFVFANADAFSWSQTF

ESLVPVDGPTALIVSDGADHRRRRSVVAPGLRHHHVQRYVATMVSNIDTVIDGWQPGQRL

DIYQELRSAVRRSTAESLFGQRLAVHSDFLGEQLQPLLDLTRRPPQVMRLQQRVNSPGWR

RAMAARKRIDDLIDAQIADARTAPRPDDHMLTTLISGCSEEGTTLSDNEIRDSIVSLITA

GYETTSGALAWAIYALLTVPGTWESAASEVARVLGGRVPAADDLSALTYLNGVVHETLRL

YSPGVISARRVLRDLWFDGHRIRAGRLLIFSAYVTHRLPEIWPEPTEFRPLRWDPNAADY

RKPAPHEFIPFSGGLHRCIGAVMATTEMTVILARLVARAMLQLPAQRTHRIRAANFAALR

PWPGLTVEIRKSAPAQ

>CYP139A1(2554692349)Mycobacterium tuberculosis EAI/OSDD271

MRTYRTVRYPLGEALLALYRWRGPLINAGVGGHGYTYLLGAEANRFVFANADAFSWSQTF

ESLVPVDGPTALIVSDGADHRRRRSVVAPGLRHHHVQRYVATMVSNIDTVIDGWQPGQRL

DIYQELRSAVRRSTAESLFGQRLAVHSDFLGEQLQPLLDLTRRPPQVMRLQQRVNSPGWR

RAMAARKRIDDLIDAQIADARTAPRPDDHMLTTLISGCSEEGTTLSDNEIRDSIVSLITA

GYETTSGALAWAIYALLTVPGTWESAASEVARVLGGRVPAADDLSALTYLNGVVHETLRL

YSPGVISARRVLRDLWFDGHRIRAGRLLIFSAYVTHRLPEIWPEPTEFRPLRWDPNAADY

RKPAPHEFIPFSGGLHRCIGAVMATTEMTVILARLVARAMLQLPAQRTHRIRAANFAALR

PWPGLTVEIRKSAPAQ

>CYP139A1(637096038)Mycobacterium tuberculosis CDC1551

MRTYRTVRYPLGEALLALYRWRGPLINAGVGGHGYTYLLGAEANRFVFANADAFSWSQTF

ESLVPVDGPTALIVSDGADHRRRRSVVAPGLRHHHVQRYVATMVSNIDTVIDGWQPGQRL

DIYQELRSAVRRSTAESLFGQRLAVHSDFLGEQLQPLLDLTRRPPQVMRLQQRVNSPGWR

RAMAARKRIDDLIDAQIADARTAPRPDDHMLTTLISGCSEEGTTLSDNEIRDSIVSLITA

GYETTSGALAWAIYALLTVPGTWESAASEVARVLGGRVPAADDLSALTYLNGVVHETLRL

YSPGVISARRVLRDLWFDGHRIRAGRLLIFSAYVTHRLPEIWPEPTEFRPLRWDPNAADY

RKPAPHEFIPFSGGLHRCIGAVMATTEMTVILARLVARAMLQLPAQRTHRIRAANFAALR

PWPGLTVEIRKSAPAQ

>CYP139A1(2555396597)Mycobacterium tuberculosis PanR0610

MRTYRTVRYPLGEALLALYRWRGPLINAGVGGHGYTYLLGAEANRFVFANADAFSWSQTF

ESLVPVDGPTALIVSDGADHRRRRSVVAPGLRHHHVQRYVATMVSNIDTVIDGWQPGQRL

DIYQELRSAVRRSTAESLFGQRLAVHSDFLGEQLQPLLDLTRRPPQVMRLQQRVNSPGWR

RAMAARKRIDDLIDAQIADARTAPRPDDHMLTTLISGCSEEGTTLSDNEIRDSIVSLITA

GYETTSGALAWAIYALLTVPGTWESAASEVARVLGGRVPAADDLSALTYLNGVVHETLRL

YSPGVISARRVLRDLWFDGHRIRAGRLLIFSAYVTHRLPEIWPEPTEFRPLRWDPNAADY

RKPAPHEFIPFSGGLHRCIGAVMATTEMTVILARLVARAMLQLPAQRTHRIRAANFAALR

PWPGLTVEIRKSAPAQ

>CYP139A1(2555520186)Mycobacterium tuberculosis PanR0411

MRTYRTVRYPLGEALLALYRWRGPLINAGVGGHGYTYLLGAEANRFVFANADAFSWSQTF

ESLVPVDGPTALIVSDGADHRRRRSVVAPGLRHHHVQRYVATMVSNIDTVIDGWQPGQRL

DIYQELRSAVRRSTAESLFGQRLAVHSDFLGEQLQPLLDLTRRPPQVMRLQQRVNSPGWR

RAMAARKRIDDLIDAQIADARTAPRPDDHMLTTLISGCSEEGTTLSDNEIRDSIVSLITA

GYETTSGALAWAIYALLTVPGTWESAASEVARVLGGRVPAADDLSALTYLNGVVHETLRL

YSPGVISARRVLRDLWFDGHRIRAGRLLIFSAYVTHRLPEIWPEPTEFRPLRWDPNAADY

RKPAPHEFIPFSGGLHRCIGAVMATTEMTVILARLVARAMLQLPAQRTHRIRAANFAALR

PWPGLTVEIRKSAPAQ

>CYP139A1(2555599804)Mycobacterium tuberculosis PanR0401

MRTYRTVRYPLGEALLALYRWRGPLINAGVGGHGYTYLLGAEANRFVFANADAFSWSQTF

ESLVPVDGPTALIVSDGADHRRRRSVVAPGLRHHHVQRYVATMVSNIDTVIDGWQPGQRL

DIYQELRSAVRRSTAESLFGQRLAVHSDFLGEQLQPLLDLTRRPPQVMRLQQRVNSPGWR

RAMAARKRIDDLIDAQIADARTAPRPDDHMLTTLISGCSEEGTTLSDNEIRDSIVSLITA

GYETTSGALAWAIYALLTVPGTWESAASEVARVLGGRVPAADDLSALTYLNGVVHETLRL

YSPGVISARRVLRDLWFDGHRIRAGRLLIFSAYVTHRLPEIWPEPTEFRPLRWDPNAADY

RKPAPHEFIPFSGGLHRCIGAVMATTEMTVILARLVARAMLQLPAQRTHRIRAANFAALR

PWPGLTVEIRKSAPAQ

>CYP139A1(2547314995)*Mycobacterium bovis*BCG-Tice TMC 1028, ATCC 35743

MRTYRTVRYPLGEALLALYRWRGPLINAGVGGHGYTYLLGAEANRFVFANADAFSWSQTF

ESLVPVDGPTALIVSDGADHRRRRSVVAPGLRHHHVQRYVATMVSNIDTVIDGWQPGQRL

DIYQELRSAVRRSTAESLFGQRLAVHSDFLGEQLQPLLDLTRRPPQVMRLQQRVNSPGWR

RAMAARKRIDDLIDAQIADARTAPRPDDHMLTTLISGCSEEGTTLSDNEIRDSIVSLITA

GYETTSGALAWAIYALLTVPGTWESAASEVARVLGGRVPAADDLSALTYLNGVVHETLRL

YSPGVISARRVLRDLWFDGHRIRAGRLLIFSAYVTHRLPEIWPEPTEFRPLRWDPNAADY

RKPAPHEFIPFSGGLHRCIGAVMATTEMTVILARLVARAMLQLPAQRTHRIRAANFAALR

PWPGLTVEIRKSAPAQ

>CYP139A1(2555160272)Mycobacterium tuberculosis PanR0207

MRTYRTVRYPLGEALLALYRWRGPLINAGVGGHGYTYLLGAEANRFVFANADAFSWSQTF

ESLVPVDGPTALIVSDGADHRRRRSVVAPGLRHHHVQRYVATMVSNIDTVIDGWQPGQRL

DIYQELRSAVRRSTAESLFGQRLAVHSDFLGEQLQPLLDLTRRPPQVMRLQQRVNSPGWR

RAMAARKRIDDLIDAQIADARTAPRPDDHMLTTLISGCSEEGTTLSDNEIRDSIVSLITA

GYETTSGALAWAIYALLTVPGTWESAASEVARVLGGRVPAADDLSALTYLNGVVHETLRL

YSPGVISARRVLRDLWFDGHRIRAGRLLIFSAYVTHRLPEIWPEPTEFRPLRWDPNAADY

RKPAPHEFIPFSGGLHRCIGAVMATTEMTVILARLVARAMLQLPAQRTHRIRAANFAALR

PWPGLTVEIRKSAPAQ

>CYP139A1(2555367201)Mycobacterium tuberculosis PanR0803

MRTYRTVRYPLGEALLALYRWRGPLINAGVGGHGYTYLLGAEANRFVFANADAFSWSQTF

ESLVPVDGPTALIVSDGADHRRRRSVVAPGLRHHHVQRYVATMVSNIDTVIDGWQPGQRL

DIYQELRSAVRRSTAESLFGQRLAVHSDFLGEQLQPLLDLTRRPPQVMRLQQRVNSPGWR

RAMAARKRIDDLIDAQIADARTAPRPDDHMLTTLISGCSEEGTTLSDNEIRDSIVSLITA

GYETTSGALAWAIYALLTVPGTWESAASEVARVLGGRVPAADDLSALTYLNGVVHETLRL

YSPGVISARRVLRDLWFDGHRIRAGRLLIFSAYVTHRLPEIWPEPTEFRPLRWDPNAADY

RKPAPHEFIPFSGGLHRCIGAVMATTEMTVILARLVARAMLQLPAQRTHRIRAANFAALR

PWPGLTVEIRKSAPAQ

>CYP139A1(2555547894)Mycobacterium tuberculosis PanR0407

MRTYRTVRYPLGEALLALYRWRGPLINAGVGGHGYTYLLGAEANRFVFANADAFSWSQTF

ESLVPVDGPTALIVSDGADHRRRRSVVAPGLRHHHVQRYVATMVSNIDTVIDGWQPGQRL

DIYQELRSAVRRSTAESLFGQRLAVHSDFLGEQLQPLLDLTRRPPQVMRLQQRVNSPGWR

RAMAARKRIDDLIDAQIADARTAPRPDDHMLTTLISGCSEEGTTLSDNEIRDSIVSLITA

GYETTSGALAWAIYALLTVPGTWESAASEVARVLGGRVPAADDLSALTYLNGVVHETLRL

YSPGVISARRVLRDLWFDGHRIRAGRLLIFSAYVTHRLPEIWPEPTEFRPLRWDPNAADY

RKPAPHEFIPFSGGLHRCIGAVMATTEMTVILARLVARAMLQLPAQRTHRIRAANFAALR

PWPGLTVEIRKSAPAQ

>CYP139A1(2560454644)Mycobacterium tuberculosis XDR1221

MRTYRTVRYPLGEALLALYRWRGPLINAGVGGHGYTYLLGAEANRFVFANADAFSWSQTF

ESLVPVDGPTALIVSDGADHRRRRSVVAPGLRHHHVQRYVATMVSNIDTVIDGWQPGQRL

DIYQELRSAVRRSTAESLFGQRLAVHSDFLGEQLQPLLDLTRRPPQVMRLQQRVNSPGWR

RAMAARKRIDDLIDAQIADARTAPRPDDHMLTTLISGCSEEGTTLSDNEIRDSIVSLITA

GYETTSGALAWAIYALLTVPGTWESAASEVARVLGGRVPAADDLSALTYLNGVVHETLRL

YSPGVISARRVLRDLWFDGHRIRAGRLLIFSAYVTHRLPEIWPEPTEFRPLRWDPNAADY

RKPAPHEFIPFSGGLHRCIGAVMATTEMTVILARLVARAMLQLPAQRTHRIRAANFAALR

PWPGLTVEIRKSAPAQ

>CYP139A1(2555148489)Mycobacterium tuberculosis PanR0203

MRTYRTVRYPLGEALLALYRWRGPLINAGVGGHGYTYLLGAEANRFVFANADAFSWSQTF

ESLVPVDGPTALIVSDGADHRRRRSVVAPGLRHHHVQRYVATMVSNIDTVIDGWQPGQRL

DIYQELRSAVRRSTAESLFGQRLAVHSDFLGEQLQPLLDLTRRPPQVMRLQQRVNSPGWR

RAMAARKRIDDLIDAQIADARTAPRPDDHMLTTLISGCSEEGTTLSDNEIRDSIVSLITA

GYETTSGALAWAIYALLTVPGTWESAASEVARVLGGRVPAADDLSALTYLNGVVHETLRL

YSPGVISARRVLRDLWFDGHRIRAGRLLIFSAYVTHRLPEIWPEPTEFRPLRWDPNAADY

RKPAPHEFIPFSGGLHRCIGAVMATYRDDRDSRSAGRQGHAAVARSADSPHSGGQLCRAA

PLAGIDR

>CYP139A1(2546436155)Mycobacterium orygis 112400015

MRYPLGEALLALYRWRGPLINAGVGGHGYTYLLGAEANRFVFANADAFSWSQTFESLVPV

DGPTALIVSDGADHRRRRSVVAPGLRHHHVQRYVATMVSNIDTVIDGWQPGQRLDIYQEL

RSAVRRSTAESLFGQRLAVHSDFLGEQLQPLLDLTRRPPQVMRLQQRVNSPGWRRAMAAR

KRIDDLIDAQIADARTAPRPDDHMLTTLISGCSEEGTTLSDNEIRDSIVSLITAGYETTS

GALAWAIYALLTVPGTWESAASEVARVLGGRVPAANDLSALTYLNGVVHETLRLYSPGVI

SARRVLRDLWFDGHRIRAGRLLIFSAYVTHRLPEIWPEPTEFRPLRWDPNAADYRKPAPH

EFIPFSGGLHRCIGAVMATTEMTVILARLVARAMLQLPAQRTHRIRAANFAALRPWPGLT

VEIRKSAPAQ

>CYP139A1(2555307696)Mycobacterium tuberculosis PanR0314

MRTYRTVRYPLGEALLALYRWRGPLINAGVGGHGYTYLLGAEANRFVFANADAFSWSQTF

ESLVPVDGPTALIVSDGADHRRRRSVVAPGLRHHHVQRYVATMVSNIDTVIDGWQPGQRL

DIYQELRSAVRRSTAESLFGQRLAVHSDFLGEQLQPLLDLTRRPPQVMRLQQRVNSPGWR

RAMAARKRIDDLIDAQIADARTAPRPDDHMLTTLISGCSEEGTTLSDNEIRDSIVSLITA

GYETTSGALAWAIYALLTVPGTWESAASEVARVLGGRVPAADDLSALTYLNGVVHETLRL

YSPGVISARRVLRDLWFDGHRIRAGRLLIFSAYVTHRLPEIWPEPTEFRPLRWDPNAADY

RKPAPHEFIPFSGGLHRCIGAVMATTEMTVILARLVARAMLQLPAQRTHRIRAANFAALR

PWPGLTVEIRKSAPAQ

>CYP139A1(2555524372)Mycobacterium tuberculosis PanR0802

MRTYRTVRYPLGEALLALYRWRGPLINAGVGGHGYTYLLGAEANRFVFANADAFSWSQTF

ESLVPVDGPTALIVSDGADHRRRRSVVAPGLRHHHVQRYVATMVSNIDTVIDGWQPGQRL

DIYQELRSAVRRSTAESLFGQRLAVHSDFLGEQLQPLLDLTRRPPQVMRLQQRVNSPGWR

RAMAARKRIDDLIDAQIADARTAPRPDDHMLTTLISGCSEEGTTLSDNEIRDSIVSLITA

GYETTSGALAWAIYALLTVPGTWESAASEVARVLGGRVPAADDLSALTYLNGVVHETLRL

YSPGVISARRVLRDLWFDGHRIRAGRLLIFSAYVTHRLPEIWPEPTEFRPLRWDPNAADY

RKPAPHEFIPFSGGLHRCIGAVMATTEMTVILARLVARAMLQLPAQRTHRIRAANFAALR

PWPGLTVEIRKSAPAQ

>CYP139A1(2546192085)Mycobacterium tuberculosis Haarlem3/NITR202

MRYPLGEALLALYRWRGPLINAGVGGHGYTYLLGAEANRFVFANADAFSWSQTFESLVPV

DGPTALIVSDGADHRRRRSVVAPGLRHHHVQRYVATMVSNIDTVIDGWQPGQRLDIYQEL

RSAVRRSTAESLFGQRLAVHSDFLGEQLQPLLDLXRRPPQVMRLQQRVNSPGWRRAMAAR

KRIDDLIDAQIADARTAPRPDDHMLTTLISGXXXEGTTLSDNEIRDSIVSLITAGYETTS

GALAWAIYALLTVPGTWESAASEVARVLGGRVPAADDLSALTYLNGVVHETLRLYSPGVI

SARRVLRDLWFDGHRIRAGRLLIFSAYVTHRLPEIWPEPTEFRPLRWDPNAADYRKPAPH

EFIPFSGGLHRCIGAVMATTEMTVILARLVARAMLQLPAQRTHRIRAANFAALRPWPGLT

VEIRKSAPAQ

>CYP139A1(2553261414)Mycobacterium tuberculosis UM 1072388579

MRTYRTVRYPLGEALLALYRWRGPLINAGVGGHGYTYLLGAEANRFVFANADAFSWSQTF

ESLVPVDGPTALIVSDGADHRRRRSVVAPGLRHHHVQRYVATMVSNIDTVIDGWQPGQRL

DIYQELRSAVRRSTAESLFGQRLAVHSDFLGEQLQPLLDLTRRPPQVMRLQQRVNSPGWR

RAMAARKRIDDLIDAQIADARTAPRPDDHMLTTLISGCSEEGTTLSDNEIRDSIVSLITA

GYETTSGALAWAIYALLTVPGTWESAASEVARVLGGRVPAADDLSALTYLNGVVHETLRL

YSPGVISARRVLRDLWFDGHRIRAGRLLIFSAYVTHRLPEIWPEPTEFRPLRWDPNAADY

RKPAPHEFIPFSGGLHRCIGAVMATTEMTVILARLVARAMLQLPAQRTHRIRAANFAALR

PWPGLTVEIRKSAPAQ

>CYP139A1(2555442648)Mycobacterium tuberculosis PanR0904

MRTYRTVRYPLGEALLALYRWRGPLINAGVGGHGYTYLLGAEANRFVFANADAFSWSQTF

ESLVPVDGPTALIVSDGADHRRRRSVVAPGLRHHHVQRYVATMVSNIDTVIDGWQPGQRL

DIYQELRSAVRRSTAESLFGQRLAVHSDFLGEQLQPLLDLTRRPPQVMRLQQRVNSPGWR

RAMAARKRIDDLIDAQIADARTAPRPDDHMLTTLISGCSEEGTTLSDNEIRDSIVSLITA

GYETTSGALAWAIYALLTVPGTWESAASEVARVLGGRVPAADDLSALTYLNGVVHETLRL

YSPGVISARRVLRDLWFDGHRIRAGRLLIFSAYVTHRLPEIWPEPTEFRPLRWDPNAADY

RKPAPHEFIPFSGGLHRCIGAVMATTEMTVILARLVARAMLQLPAQRTHRIRAANFAALR

PWPGLTVEIRKSAPAQ

>CYP139A1(2555455224)Mycobacterium tuberculosis PanR0907

MRTYRTVRYPLGEALLALYRWRGPLINAGVGGHGYTYLLGAEANRFVFANADAFSWSQTF

ESLVPVDGPTALIVSDGADHRRRRSVVAPGLRHHHVQRYVATMVSNIDTVIDGWQPGQRL

DIYQELRSAVRRSTAESLFGQRLAVHSDFLGEQLQPLLDLTRRPPQVMRLQQRVNSPGWR

RAMAARKRIDDLIDAQIADARTAPRPDDHMLTTLISGCSEEGTTLSDNEIRDSIVSLITA

GYETTSGALAWAIYALLTVPGTWESAASEVARVLGGRVPAADDLSALTYLNGVVHETLRL

YSPGVISARRVLRDLWFDGHRIRAGRLLIFSAYVTHRLPEIWPEPTEFRPLRWDPNAADY

RKPAPHEFIPFSGGLHRCIGAVMATTEMTVILARLVARAMLQLPAQRTHRIRAANFAALR

PWPGLTVEIRKSAPAQ

>CYP139A1(651084428)Mycobacterium tuberculosis CCDC5079

MRTYRTVRYPLGEALLALYRWRGPLINAGVGGHGYTYLLGAEANRFVFANADAFSWSQTF

ESLVPVDGPTALIVSDGADHRRRRSVVAPGLRHHHVQRYVATMVSNIDTVIDGWQPGQRL

DIYQELRSAVRRSTAESLFGQRLAVHSDFLGEQLQPLLDLTRRPPQVMRLQQRVNSPGWR

RAMAARKRIDDLIDAQIADARTAPRPDDHMLTTLISGCSEEGTTLSDNEIRDSIVSLITA
[truncated: 53,153 more chars]
