# Supplementary material for: Cytochrome P450 Monooxygenase CYP139 Family Involved in the Synthesis of Secondary Metabolites in 824 Mycobacterial Species
Source: Int J Mol Sci. 2019 May 31;20(11):2690. doi: 10.3390/ijms20112690 (PMC6600245; doi:10.3390/ijms20112690)
Supplement: Supplementary file 1 [file ijms-20-02690-s001.zip › Supplementary Information/Supplementary Dataset 3.docx]

*Article*

**Cytochrome P450 monooxygenase CYP139 family involved in the synthesis of secondary metabolites in 824 mycobacterial species**

**Puleng Rosinah Syed ^1^, Wanping Chen ^2^, David R Nelson ^3^, Abidemi Paul Kappo ^4^, Jae-Hyuk Yu ^5,6^, Rajshekhar Karpoormath ^1^*, Khajamohiddin Syed ^4,^***

^1^ Department of Pharmaceutical Chemistry, College of Health Sciences, University of KwaZulu-Natal, Durban 4000, South Africa; prosinah@gmail.com (P.R.S.); Karpoormath@ukzn.ac.za (R.K.)

^2^ College of Food Science and Technology, Huazhong Agricultural University, Wuhan 430070, Hubei Province, China; chenwanping@mail.hzau.edu.cn

^3^ Department of Microbiology, Immunology and Biochemistry, University of Tennessee Health Science Center, Memphis, TN, 38163; drnelson1@gmail.com

^4^ Department of Biochemistry and Microbiology, Faculty of Science and Agriculture, University of Zululand, KwaDlangezwa 3886, South Africa; KappoA@unizulu.ac.za (A.P.K.); khajamohiddinsyed@gmail.com (K.S.)

^5^ Department of Bacteriology, University of Wisconsin-Madison, 3155 MSB, 1550 Linden Drive, Madison, WI 53706, USA; [jyu1@wisc.edu](mailto:jyu1@wisc.edu)

^6^ Department of Systems Biotechnology, Konkuk University, Seoul, 05029, Republic of Korea

**Supplementary Dataset 3:** PROMALS3D analysis of CYP139A P450s.

[**Colored**](http://prodata.swmed.edu/promals3d/info/promals_output.html)**PROMALS3D alignment (sequences in aligned order)**

Conservation: 9 9 6966966669 996 669 9 6 6 6 666 6 669669969 696 96

CYP139A_2567124714_Mycoba 1 M-----NAYRTVPYLPGEALLALYRHRGAVVDAGVG-RHGFVYLLGPEANKFVFANADAFSWRETFESLV 64

CYP139A_650873455_Mycobac 1 -----------MRYRPGDALATLHRRRGPMVDAGVG-RYGYVYLFGAEANKFVFANSDAFNWWDAFQVLV 58

CYP139A_2549393401_Mycoba 1 M-------TPPARYRPGEALLALYRRRGPMVDAGIG-RHGYIYLLGAEANKFVFANSDAFSWWDAFQVLV 62

CYP139A_2548535921_Mycoba 1 M--------RPVRYRPGEALLALYRRRGPVIDAGAG-RHGYTLLLGAEANKFVFANADAFSWRATFENLA 61

CYP139A_2576976958_Mycoba 1 -----------MRYRPGEALLALYRRRGPVIDAGAG-RHGYTLLLGAEANKFVFANADAFSWRATFENLA 58

CYP139A_2581913245_Mycoba 1 -----------MRYRPGEALLALYRRRGPVIDAGAG-RRGYTLLLGAEANKFVFANADAFSWRATFENLA 58

CYP139A_2580974538_Mycoba 1 -----------MRYRPGEALLALYRRRGPVIDAGAG-RHGYTLLLGAEANKFVFANADAFSWRATFENLA 58

CYP139A_2582181025_Mycoba 1 -----------MRYRPGEALLALYRRRGPVIDAGAG-RRGYTLLLGAEANKFVFANADAFSWRATFENLA 58

CYP139A_2580742569_Mycoba 1 -----------MRYRPGEALLALYRRRGPVIDAGAG-RHGYTLLLGAEANKFVFANADAFSWRATFENLA 58

CYP139A_2582203743_Mycoba 1 -----------MRYRPGEALLALYRRRGPVIDAGAG-RHGYTLLLGAEANKFVFANADAFSWRATFENLA 58

CYP139A_2567079276_Mycoba 1 -----------------------------MIDAGAG-RRGYTLLLGAEANKFVFANADAFSWRATFENLA 40

CYP139A_2543326887_Mycoba 1 -----------MRYRPGEALLALYRRRGPVIDAGAG-RHGYTLLLGAEANKFVFANADAFSWRATFENLA 58

CYP139A_2570865822_Mycoba 1 -----------MRYRPGEALLALYRRRGPVIDAGAG-RHGYTLLLGAEANKFVFANADAFSWRATFENLA 58

CYP139A_2592485489_Mycoba 1 -----------MRYRPGEALLALYRRRGPVIDAGAG-RHGYTLLLGAEANKFVFANADAFSWRATFENLA 58

CYP139A_2550738610_Mycoba 1 M--------RPVRYRPGEALLALYRRRGPVIDAGAG-RHGYTLLLGAEANKFVFANADAFSWRATFENLA 61

CYP139A_2580783837__Mycob 1 -----------MRYRPGEALLALYRRRGPVIDAGAG-RRGYTLLLGAEANKFVFANADAFSWRATFENLA 58

CYP139A_2569618768_Mycoba 1 -----------MRYRPGEALLALYRRRGPVIDAGAG-RRGYTLLLGAEANKFVFANADAFSWRATFENLA 58

CYP139A_2547368463_Mycoba 1 M--------RPVRYRPGEALLALYRRRGPVIDAGAG-RHGYTLLLGAEANKFVFANADAFSWRATFENLA 61

CYP139A_2572767979_Mycoba 1 -----------MRYRPGEALLALYRRRGPVIDAGAG-RRGYTALLGAEANKFVFANADAFSWRATFENLA 58

CYP139A_2582391300_Mycoba 1 -----------MRYRPGEALLALYRRRGPVIDAGAG-RRGYTLLLGAEANKFVFANADAFSWRATFENLA 58

CYP139A_2548515815_Mycoba 1 M--------RPVRYRPGEALLALYRRRGPVIDAGAG-RHGYTLLLGAEANKFVFANADAFSWRATFENLA 61

CYP139A_2548530385_Mycoba 1 M--------RPVRYRPGEALLALYRRRGPVIDAGAG-RHGYTLLLGAEANKFVFANADAFSWRATFENLA 61

CYP139A_2581397788_Mycoba 1 -----------MRYRPGEALLALYRRRGPVIDAGAG-RRGYTLLLGAEANKFVFANADAFSWRATFENLA 58

CYP139A_2549377452_Mycoba 1 M--------RPVRYRPGEALLALYRRRGPVIDAGAG-RHGYTLLLGAEANKFVFANADAFSWRATFENLA 61

CYP139A_2549389164_Mycoba 1 M--------RPVRYRPGEALLALYRRRGPVIDAGAG-RRGYTLLLGAEANKFVFANADAFSWRATFENLA 61

CYP139A_645425415_Mycobac 1 -----------MRYRPGEALLALYRRRGPVIDAGAG-RRGYTLLLGAEANKFVFANADAFSWRATFENLA 58

CYP139A_2581110378_Mycoba 1 M--------RPVRYRPGEALLALYRRRGPVIDAGAG-RRGYTLLLGAEANKFVFANADAFSWRATFENLA 61

CYP139A_2573433552_Mycoba 1 -----------MRYRPGEALLALYRRRGPVIDAGAG-RRGYTLLLGAEANKFVFANADAFSWRATFENLA 58

CYP139A_2580006443__Mycob 1 -----------MRYRPGEALLALYRRRGPVIDAGAG-RRGYTLLLGAEANKFVFANADAFSWRATFENLA 58

CYP139A_637134331_Mycobac 1 -----------MRYRPGEALLALYRRRGPVIDAGAG-RHGYTLLLGAEANKFVFANADAFSWRATFENLA 58

CYP139A_2548578292_Mycoba 1 M--------RPVRYRPGEALLALYRRRGPVIDAGAG-RHGYTLLLGAEANKFVFANADAFSWRATFENLA 61

CYP139A_2548547272_Mycoba 1 M--------RPVRYRPGEALLALYRRRGPVIDAGAG-RHGYTLLLGAEANKFVFANADAFSWRATFENLA 61

CYP139A_2549383420_Mycoba 1 M--------RPVRYRPGEALLALYRRRGPVIDAGAG-RHGYTLLLGAEANKFVFANADAFSWRATFENLA 61

CYP139A_639736419_Mycobac 1 -----------MRYRPGEALLALYRRRGPVIDAGAG-RRGYTLLLGAEANKFVFANADAFSWRATFENLA 58

CYP139A_2555735619_Mycoba 1 -----------MRYRPGEALLALYRRRGPVIDAGAG-RHGYTLLLGAEANKFVFANADAFSWRATFENLA 58

CYP139A_2555481387_Mycoba 1 MVAVGLKTYRPVRYPPGEALLAMYRWRGPVLNSGVG-RHGYTYLLGPEANKFVFANADAFSWRETFENLA 69

CYP139A_2545768030_Mycoba 1 M------RNRPIRYLPGEGLLALYRLRGPVINSGVG-RRGYTYRLGPEANKFVFANADAFSWAQTFESLA 63

CYP139A_2567131988_Mycoba 1 M-----NGYRTVPYLPGEALLALYRHRGAVIDAGVG-RHGFVYLLGAEANKFVFANADAFSWRETFESLV 64

CYP139A_2587480388_Mycoba 1 M-----NGYRTVPYLPGEALLALYRHRGAVIDAGVG-RHGFVYLLGAEANKFVFANADAFSWRETFESLV 64

CYP139A_2563577345_Mycoba 1 M-----NGYRTVPYLPGEALLALYRHRGAVIDAGVG-RHGFVYLLGAEANKFVFANADAFSWRETFESLV 64

CYP139A_2543277028_Mycoba 1 M-----STYRTVPYRPGEALLALYRRRGAFIDAGVG-RHGFVYLLGPEANRFVFANADAFSWRETFESLV 64

CYP139A_2563569217_Mycoba 1 M-----STYRTVPYRPGEALLALYRRRGAFIDAGVG-RHGFVYLLGPEANRFVFANADAFSWRETFESLV 64

CYP139A_641717750_Mycobac 1 M-----STYRTVPYRPGEALLALYRRRGAFIDAGVG-RHGFVYLLGPEANRFVFANADAFSWRETFESLV 64

CYP139A_2588629254_Mycoba 1 M-----STYRTVPYRPGEALLALYRRRGAFIDAGVG-RHGFVYLLGPEANRFVFANADAFSWRETFESLV 64

CYP139A_2546369014_Mycoba 1 M-----STYRTVPYRPGEALLALYRRRGAFIDAGVG-RHGFVYLLGPEANRFVFANADAFSWRETFESLV 64

CYP139A1_2555148489_Mycob 1 M-----RTYRTVRYPLGEALLALYRWRGPLINAGVG-GHGYTYLLGAEANRFVFANADAFSWSQTFESLV 64

CYP139A1_646010237_Mycoba 1 ----------------------------------------------------MFANADAFSWSQTFESLV 18

CYP139A1_2581377024_Mycob 1 ----------------------------------------------------MFANADAFSWSQTFESLV 18

CYP139A1_647209603_Mycoba 1 ----------------------------------------------------MFANADAFSWSQTFESLV 18

CYP139A1_2537735281_Mycob 1 M-----RTYRTVRYPLGEALLALYRWRGPLINAGVG-GHGYTYLLGAEANRFVFANADAFSWSQTFESLV 64

CYP139A1_2576388909_Mycob 1 M-----R------YPLGEALLALYRWRGPLINAGVG-GHGYTYLLGAEANRFVFANADAFSWSQTFESLV 58

CYP139A1_2577593438_Mycob 1 M-----R------YPLGEALLALYRWRGPLINAGVG-GHGYTYLLGAEANRFVFANADAFSWSQTFESLV 58

CYP139A1_2577803488_Mycob 1 M-----R------YPLGEALLALYRWRGPLINAGVG-GHGYTYLLGAEANRFVFANADAFSWSQTFESLV 58

CYP139A1_2581355094_Mycob 1 M-----R------YPLGEALLALYRWRGPLINAGVG-GHGYTYLLGAEANRFVFANADAFSWSQTFESLV 58

CYP139A1_2584983051_Mycob 1 M-----R------YPLGEALLALYRWRGPLINAGVG-GHGYTYLLGAEANRFVFANADAFSWSQTFESLV 58

CYP139A1_2584987406_Mycob 1 M-----R------YPLGEALLALYRWRGPLINAGVG-GHGYTYLLGAEANRFVFANADAFSWSQTFESLV 58

CYP139A1_2589032800_Mycob 1 M-----R------YPLGEALLALYRWRGPLINAGVG-GHGYTYLLGAEANRFVFANADAFSWSQTFESLV 58

CYP139A1_2592403099_Mycob 1 M-----R------YPLGEALLALYRWRGPLINAGVG-GHGYTYLLGAEANRFVFANADAFSWSQTFESLV 58

CYP139A1_2592422247_Mycob 1 M-----R------YPLGEALLALYRWRGPLINAGVG-GHGYTYLLGAEANRFVFANADAFSWSQTFESLV 58

CYP139A1_643734506_Mycoba 1 M-----R------YPLGEALLALYRWRGPLINAGVG-GHGYTYLLGAEANRFVFANADAFSWSQTFESLV 58

CYP139A1_648335985_Mycoba 1 M-----R------YPLGEALLALYRWRGPLINAGVG-GHGYTYLLGAEANRFVFANADAFSWSQTFESLV 58

CYP139A1_2574754194_Mycob 1 M-----R------YPLGEALLALYRWRGPLINAGVG-GHGYTYLLGAEANRFVFANADAFSWSQTFESLV 58

CYP139A1_2575447433_Mycob 1 M-----R------YPLGEALLALYRWRGPLINAGVG-GHGYTYLLGAEANRFVFANADAFSWSQTFESLV 58

CYP139A1_2575938969_Mycob 1 M-----R------YPLGEALLALYRWRGPLINAGVG-GHGYTYLLGAEANRFVFANADAFSWSQTFESLV 58

CYP139A1_2576477081_Mycob 1 M-----R------YPLGEALLALYRWRGPLINAGVG-GHGYTYLLGAEANRFVFANADAFSWSQTFESLV 58

CYP139A1_2576601719_Mycob 1 M-----R------YPLGEALLALYRWRGPLINAGVG-GHGYTYLLGAEANRFVFANADAFSWSQTFESLV 58

CYP139A1_2577098384_Mycob 1 M-----R------YPLGEALLALYRWRGPLINAGVG-GHGYTYLLGAEANRFVFANADAFSWSQTFESLV 58

CYP139A1_2578107196_Mycob 1 M-----R------YPLGEALLALYRWRGPLINAGVG-GHGYTYLLGAEANRFVFANADAFSWSQTFESLV 58

CYP139A1_2584883084_Mycob 1 M-----R------YPLGEALLALYRWRGPLINAGVG-GHGYTYLLGAEANRFVFANADAFSWSQTFESLV 58

CYP139A1_2588974834_Mycob 1 M-----R------YPLGEALLALYRWRGPLINAGVG-GHGYTYLLGAEANRFVFANADAFSWSQTFESLV 58

CYP139A1_2589056454_Mycob 1 M-----R------YPLGEALLALYRWRGPLINAGVG-GHGYTYLLGAEANRFVFANADAFSWSQTFESLV 58

CYP139A1_2589161189_Mycob 1 M-----R------YPLGEALLALYRWRGPLINAGVG-GHGYTYLLGAEANRFVFANADAFSWSQTFESLV 58

CYP139A1_2590374347_Mycob 1 M-----R------YPLGEALLALYRWRGPLINAGVG-GHGYTYLLGAEANRFVFANADAFSWSQTFESLV 58

CYP139A1_646018681_Mycoba 1 M-----R------YPLGEALLALYRWRGPLINAGVG-GHGYTYLLGAEANRFVFANADAFSWSQTFESLV 58

CYP139A1_2575060404_Mycob 1 M-----R------YPLGEALLALYRWRGPLINAGVG-GHGYTYLLGAEANRFVFANADAFSWSQTFESLV 58

CYP139A1_2576105631_Mycob 1 M-----R------YPLGEALLALYRWRGPLINAGVG-GHGYTYLLGAEANRFVFANADAFSWSQTFESLV 58

CYP139A1_2576247251_Mycob 1 M-----R------YPLGEALLALYRWRGPLINAGVG-GHGYTYLLGAEANRFVFANADAFSWSQTFESLV 58

CYP139A1_2576981010_Mycob 1 M-----R------YPLGEALLALYRWRGPLINAGVG-GHGYTYLLGAEANRFVFANADAFSWSQTFESLV 58

CYP139A1_2577093117_Mycob 1 M-----R------YPLGEALLALYRWRGPLINAGVG-GHGYTYLLGAEANRFVFANADAFSWSQTFESLV 58

CYP139A1_2577198903_Mycob 1 M-----R------YPLGEALLALYRWRGPLINAGVG-GHGYTYLLGAEANRFVFANADAFSWSQTFESLV 58

CYP139A1_2577516047_Mycob 1 M-----R------YPLGEALLALYRWRGPLINAGVG-GHGYTYLLGAEANRFVFANADAFSWSQTFESLV 58

CYP139A1_2578213104_Mycob 1 M-----R------YPLGEALLALYRWRGPLINAGVG-GHGYTYLLGAEANRFVFANADAFSWSQTFESLV 58

CYP139A1_2584711251_Mycob 1 M-----R------YPLGEALLALYRWRGPLINAGVG-GHGYTYLLGAEANRFVFANADAFSWSQTFESLV 58

CYP139A1_2584816678_Mycob 1 M-----R------YPLGEALLALYRWRGPLINAGVG-GHGYTYLLGAEANRFVFANADAFSWSQTFESLV 58

CYP139A1_2589068752_Mycob 1 M-----R------YPLGEALLALYRWRGPLINAGVG-GHGYTYLLGAEANRFVFANADAFSWSQTFESLV 58

CYP139A1_2589604293_Mycob 1 M-----R------YPLGEALLALYRWRGPLINAGVG-GHGYTYLLGAEANRFVFANADAFSWSQTFESLV 58

CYP139A1_643028176_Mycoba 1 M-----R------YPLGEALLALYRWRGPLINAGVG-GHGYTYLLGAEANRFVFANADAFSWSQTFESLV 58

CYP139A1_648476944_Mycoba 1 M-----R------YPLGEALLALYRWRGPLINAGVG-GHGYTYLLGAEANRFVFANADAFSWSQTFESLV 58

CYP139A1_2511736071_Mycob 1 M-----R------YPLGEALLALYRWRGPLINAGVG-GHGYTYLLGAEANRFVFANADAFSWSQTFESLV 58

CYP139A1_2546206123_Mycob 1 M-----R------YPLGEALLALYRWRGPLINAGVG-GHGYTYLLGAEANRFVFANADAFSWSQTFESLV 58

CYP139A1_2574780327_Mycob 1 M-----R------YPLGEALLALYRWRGPLINAGVG-GHGYTYLLGAEANRFVFANADAFSWSQTFESLV 58

CYP139A1_2575978404_Mycob 1 M-----R------YPLGEALLALYRWRGPLINAGVG-GHGYTYLLGAEANRFVFANADAFSWSQTFESLV 58

CYP139A1_2576675825_Mycob 1 M-----R------YPLGEALLALYRWRGPLINAGVG-GHGYTYLLGAEANRFVFANADAFSWSQTFESLV 58

CYP139A1_2576947708_Mycob 1 M-----R------YPLGEALLALYRWRGPLINAGVG-GHGYTYLLGAEANRFVFANADAFSWSQTFESLV 58

CYP139A1_2577400922_Mycob 1 M-----R------YPLGEALLALYRWRGPLINAGVG-GHGYTYLLGAEANRFVFANADAFSWSQTFESLV 58

CYP139A1_2577893113_Mycob 1 M-----R------YPLGEALLALYRWRGPLINAGVG-GHGYTYLLGAEANRFVFANADAFSWSQTFESLV 58

CYP139A1_2584759228_Mycob 1 M-----R------YPLGEALLALYRWRGPLINAGVG-GHGYTYLLGAEANRFVFANADAFSWSQTFESLV 58

CYP139A1_2584801008_Mycob 1 M-----R------YPLGEALLALYRWRGPLINAGVG-GHGYTYLLGAEANRFVFANADAFSWSQTFESLV 58

CYP139A1_2584946269_Mycob 1 M-----R------YPLGEALLALYRWRGPLINAGVG-GHGYTYLLGAEANRFVFANADAFSWSQTFESLV 58

CYP139A1_2589125802_Mycob 1 M-----R------YPLGEALLALYRWRGPLINAGVG-GHGYTYLLGAEANRFVFANADAFSWSQTFESLV 58

CYP139A1_2589654545_Mycob 1 M-----R------YPLGEALLALYRWRGPLINAGVG-GHGYTYLLGAEANRFVFANADAFSWSQTFESLV 58

CYP139A1_2590190898_Mycob 1 M-----R------YPLGEALLALYRWRGPLINAGVG-GHGYTYLLGAEANRFVFANADAFSWSQTFESLV 58

CYP139A1_2592267285_Mycob 1 M-----R------YPLGEALLALYRWRGPLINAGVG-GHGYTYLLGAEANRFVFANADAFSWSQTFESLV 58

CYP139A1_2592579018_Mycob 1 M-----R------YPLGEALLALYRWRGPLINAGVG-GHGYTYLLGAEANRFVFANADAFSWSQTFESLV 58

CYP139A1_2574803240_Mycob 1 M-----R------YPLGEALLALYRWRGPLINAGVG-GHGYTYLLGAEANRFVFANADAFSWSQTFESLV 58

CYP139A1_2575138339_Mycob 1 M-----R------YPLGEALLALYRWRGPLINAGVG-GHGYTYLLGAEANRFVFANADAFSWSQTFESLV 58

CYP139A1_2575935659_Mycob 1 M-----R------YPLGEALLALYRWRGPLINAGVG-GHGYTYLLGAEANRFVFANADAFSWSQTFESLV 58

CYP139A1_2576703024_Mycob 1 M-----R------YPLGEALLALYRWRGPLINAGVG-GHGYTYLLGAEANRFVFANADAFSWSQTFESLV 58

CYP139A1_2577143911_Mycob 1 M-----R------YPLGEALLALYRWRGPLINAGVG-GHGYTYLLGAEANRFVFANADAFSWSQTFESLV 58

CYP139A1_2577175183_Mycob 1 M-----R------YPLGEALLALYRWRGPLINAGVG-GHGYTYLLGAEANRFVFANADAFSWSQTFESLV 58

CYP139A1_2577879644_Mycob 1 M-----R------YPLGEALLALYRWRGPLINAGVG-GHGYTYLLGAEANRFVFANADAFSWSQTFESLV 58

CYP139A1_2577954418_Mycob 1 M-----R------YPLGEALLALYRWRGPLINAGVG-GHGYTYLLGAEANRFVFANADAFSWSQTFESLV 58

CYP139A1_2584625495_Mycob 1 M-----R------YPLGEALLALYRWRGPLINAGVG-GHGYTYLLGAEANRFVFANADAFSWSQTFESLV 58

CYP139A1_2589526877_Mycob 1 M-----R------YPLGEALLALYRWRGPLINAGVG-GHGYTYLLGAEANRFVFANADAFSWSQTFESLV 58

CYP139A1_2589711853_Mycob 1 M-----R------YPLGEALLALYRWRGPLINAGVG-GHGYTYLLGAEANRFVFANADAFSWSQTFESLV 58

CYP139A1_2590113796_Mycob 1 M-----R------YPLGEALLALYRWRGPLINAGVG-GHGYTYLLGAEANRFVFANADAFSWSQTFESLV 58

CYP139A1_2592324422_Mycob 1 M-----R------YPLGEALLALYRWRGPLINAGVG-GHGYTYLLGAEANRFVFANADAFSWSQTFESLV 58

CYP139A1_2592337997_Mycob 1 M-----R------YPLGEALLALYRWRGPLINAGVG-GHGYTYLLGAEANRFVFANADAFSWSQTFESLV 58

CYP139A1_2592373455_Mycob 1 M-----R------YPLGEALLALYRWRGPLINAGVG-GHGYTYLLGAEANRFVFANADAFSWSQTFESLV 58

CYP139A1_2592445915_Mycob 1 M-----R------YPLGEALLALYRWRGPLINAGVG-GHGYTYLLGAEANRFVFANADAFSWSQTFESLV 58

CYP139A1_2575023271_Mycob 1 M-----R------YPLGEALLALYRWRGPLINAGVG-GHGYTYLLGAEANRFVFANADAFSWSQTFESLV 58

CYP139A1_2575619239_Mycob 1 M-----R------YPLGEALLALYRWRGPLINAGVG-GHGYTYLLGAEANRFVFANADAFSWSQTFESLV 58

CYP139A1_2575786887_Mycob 1 M-----R------YPLGEALLALYRWRGPLINAGVG-GHGYTYLLGAEANRFVFANADAFSWSQTFESLV 58

CYP139A1_2576882264_Mycob 1 M-----R------YPLGEALLALYRWRGPLINAGVG-GHGYTYLLGAEANRFVFANADAFSWSQTFESLV 58

CYP139A1_2577215885_Mycob 1 M-----R------YPLGEALLALYRWRGPLINAGVG-GHGYTYLLGAEANRFVFANADAFSWSQTFESLV 58

CYP139A1_2577627248_Mycob 1 M-----R------YPLGEALLALYRWRGPLINAGVG-GHGYTYLLGAEANRFVFANADAFSWSQTFESLV 58

CYP139A1_2577923998_Mycob 1 M-----R------YPLGEALLALYRWRGPLINAGVG-GHGYTYLLGAEANRFVFANADAFSWSQTFESLV 58

CYP139A1_2583735989_Mycob 1 M-----R------YPLGEALLALYRWRGPLINAGVG-GHGYTYLLGAEANRFVFANADAFSWSQTFESLV 58

CYP139A1_2584003651_Mycob 1 M-----R------YPLGEALLALYRWRGPLINAGVG-GHGYTYLLGAEANRFVFANADAFSWSQTFESLV 58

CYP139A1_2584623655_Mycob 1 M-----R------YPLGEALLALYRWRGPLINAGVG-GHGYTYLLGAEANRFVFANADAFSWSQTFESLV 58

CYP139A1_2584739857_Mycob 1 M-----R------YPLGEALLALYRWRGPLINAGVG-GHGYTYLLGAEANRFVFANADAFSWSQTFESLV 58

CYP139A1_2584858071_Mycob 1 M-----R------YPLGEALLALYRWRGPLINAGVG-GHGYTYLLGAEANRFVFANADAFSWSQTFESLV 58

CYP139A1_2584928246_Mycob 1 M-----R------YPLGEALLALYRWRGPLINAGVG-GHGYTYLLGAEANRFVFANADAFSWSQTFESLV 58

CYP139A1_2589040293_Mycob 1 M-----R------YPLGEALLALYRWRGPLINAGVG-GHGYTYLLGAEANRFVFANADAFSWSQTFESLV 58

CYP139A1_2589658610_Mycob 1 M-----R------YPLGEALLALYRWRGPLINAGVG-GHGYTYLLGAEANRFVFANADAFSWSQTFESLV 58

CYP139A1_2590162679_Mycob 1 M-----R------YPLGEALLALYRWRGPLINAGVG-GHGYTYLLGAEANRFVFANADAFSWSQTFESLV 58

CYP139A1_2590243884_Mycob 1 M-----R------YPLGEALLALYRWRGPLINAGVG-GHGYTYLLGAEANRFVFANADAFSWSQTFESLV 58

CYP139A1_2590531681_Mycob 1 M-----R------YPLGEALLALYRWRGPLINAGVG-GHGYTYLLGAEANRFVFANADAFSWSQTFESLV 58

CYP139A1_2574614619_Mycob 1 M-----R------YPLGEALLALYRWRGPLINAGVG-GHGYTYLLGAEANRFVFANADAFSWSQTFESLV 58

CYP139A1_2575016195_Mycob 1 M-----R------YPLGEALLALYRWRGPLINAGVG-GHGYTYLLGAEANRFVFANADAFSWSQTFESLV 58

CYP139A1_2575426848_Mycob 1 M-----R------YPLGEALLALYRWRGPLINAGVG-GHGYTYLLGAEANRFVFANADAFSWSQTFESLV 58

CYP139A1_2576630848_Mycob 1 M-----R------YPLGEALLALYRWRGPLINAGVG-GHGYTYLLGAEANRFVFANADAFSWSQTFESLV 58

CYP139A1_2577468911_Mycob 1 M-----R------YPLGEALLALYRWRGPLINAGVG-GHGYTYLLGAEANRFVFANADAFSWSQTFESLV 58

CYP139A1_2577974906_Mycob 1 M-----R------YPLGEALLALYRWRGPLINAGVG-GHGYTYLLGAEANRFVFANADAFSWSQTFESLV 58

CYP139A1_2578062326_Mycob 1 M-----R------YPLGEALLALYRWRGPLINAGVG-GHGYTYLLGAEANRFVFANADAFSWSQTFESLV 58

CYP139A1_2584836956_Mycob 1 M-----R------YPLGEALLALYRWRGPLINAGVG-GHGYTYLLGAEANRFVFANADAFSWSQTFESLV 58

CYP139A1_2584998642_Mycob 1 M-----R------YPLGEALLALYRWRGPLINAGVG-GHGYTYLLGAEANRFVFANADAFSWSQTFESLV 58

CYP139A1_2589130082_Mycob 1 M-----R------YPLGEALLALYRWRGPLINAGVG-GHGYTYLLGAEANRFVFANADAFSWSQTFESLV 58

CYP139A1_2589592089_Mycob 1 M-----R------YPLGEALLALYRWRGPLINAGVG-GHGYTYLLGAEANRFVFANADAFSWSQTFESLV 58

CYP139A1_2590052832_Mycob 1 M-----R------YPLGEALLALYRWRGPLINAGVG-GHGYTYLLGAEANRFVFANADAFSWSQTFESLV 58

CYP139A1_2590505688_Mycob 1 M-----R------YPLGEALLALYRWRGPLINAGVG-GHGYTYLLGAEANRFVFANADAFSWSQTFESLV 58

CYP139A1_2592353030_Mycob 1 M-----R------YPLGEALLALYRWRGPLINAGVG-GHGYTYLLGAEANRFVFANADAFSWSQTFESLV 58

CYP139A1_637026884_Mycoba 1 M-----R------YPLGEALLALYRWRGPLINAGVG-GHGYTYLLGAEANRFVFANADAFSWSQTFESLV 58

CYP139A1_2574886309_Mycob 1 M-----R------YPLGEALLALYRWRGPLINAGVG-GHGYTYLLGAEANRFVFANADAFSWSQTFESLV 58

CYP139A1_2575295342_Mycob 1 M-----R------YPLGEALLALYRWRGPLINAGVG-GHGYTYLLGAEANRFVFANADAFSWSQTFESLV 58

CYP139A1_2575942274_Mycob 1 M-----R------YPLGEALLALYRWRGPLINAGVG-GHGYTYLLGAEANRFVFANADAFSWSQTFESLV 58

CYP139A1_2576123248_Mycob 1 M-----R------YPLGEALLALYRWRGPLINAGVG-GHGYTYLLGAEANRFVFANADAFSWSQTFESLV 58

CYP139A1_2576712596_Mycob 1 M-----R------YPLGEALLALYRWRGPLINAGVG-GHGYTYLLGAEANRFVFANADAFSWSQTFESLV 58

CYP139A1_2577856904_Mycob 1 M-----R------YPLGEALLALYRWRGPLINAGVG-GHGYTYLLGAEANRFVFANADAFSWSQTFESLV 58

CYP139A1_2578013153_Mycob 1 M-----R------YPLGEALLALYRWRGPLINAGVG-GHGYTYLLGAEANRFVFANADAFSWSQTFESLV 58

CYP139A1_2578182623_Mycob 1 M-----R------YPLGEALLALYRWRGPLINAGVG-GHGYTYLLGAEANRFVFANADAFSWSQTFESLV 58

CYP139A1_2580771058_Mycob 1 M-----R------YPLGEALLALYRWRGPLINAGVG-GHGYTYLLGAEANRFVFANADAFSWSQTFESLV 58

CYP139A1_2584641128_Mycob 1 M-----R------YPLGEALLALYRWRGPLINAGVG-GHGYTYLLGAEANRFVFANADAFSWSQTFESLV 58

CYP139A1_2584649146_Mycob 1 M-----R------YPLGEALLALYRWRGPLINAGVG-GHGYTYLLGAEANRFVFANADAFSWSQTFESLV 58

CYP139A1_2584660713_Mycob 1 M-----R------YPLGEALLALYRWRGPLINAGVG-GHGYTYLLGAEANRFVFANADAFSWSQTFESLV 58

CYP139A1_2584967006_Mycob 1 M-----R------YPLGEALLALYRWRGPLINAGVG-GHGYTYLLGAEANRFVFANADAFSWSQTFESLV 58

CYP139A1_2584970624_Mycob 1 M-----R------YPLGEALLALYRWRGPLINAGVG-GHGYTYLLGAEANRFVFANADAFSWSQTFESLV 58

CYP139A1_2589053585_Mycob 1 M-----R------YPLGEALLALYRWRGPLINAGVG-GHGYTYLLGAEANRFVFANADAFSWSQTFESLV 58

CYP139A1_2589498327_Mycob 1 M-----R------YPLGEALLALYRWRGPLINAGVG-GHGYTYLLGAEANRFVFANADAFSWSQTFESLV 58

CYP139A1_2589563529_Mycob 1 M-----R------YPLGEALLALYRWRGPLINAGVG-GHGYTYLLGAEANRFVFANADAFSWSQTFESLV 58

CYP139A1_2590377218_Mycob 1 M-----R------YPLGEALLALYRWRGPLINAGVG-GHGYTYLLGAEANRFVFANADAFSWSQTFESLV 58

CYP139A1_2592283614_Mycob 1 M-----R------YPLGEALLALYRWRGPLINAGVG-GHGYTYLLGAEANRFVFANADAFSWSQTFESLV 58

CYP139A1_2592319784_Mycob 1 M-----R------YPLGEALLALYRWRGPLINAGVG-GHGYTYLLGAEANRFVFANADAFSWSQTFESLV 58

CYP139A1_2575106637_Mycob 1 M-----R------YPLGEALLALYRWRGPLINAGVG-GHGYTYLLGAEANRFVFANADAFSWSQTFESLV 58

CYP139A1_2575157076_Mycob 1 M-----R------YPLGEALLALYRWRGPLINAGVG-GHGYTYLLGAEANRFVFANADAFSWSQTFESLV 58

CYP139A1_2575361778_Mycob 1 M-----R------YPLGEALLALYRWRGPLINAGVG-GHGYTYLLGAEANRFVFANADAFSWSQTFESLV 58

CYP139A1_2576009184_Mycob 1 M-----R------YPLGEALLALYRWRGPLINAGVG-GHGYTYLLGAEANRFVFANADAFSWSQTFESLV 58

CYP139A1_2576566954_Mycob 1 M-----R------YPLGEALLALYRWRGPLINAGVG-GHGYTYLLGAEANRFVFANADAFSWSQTFESLV 58

CYP139A1_2576731741_Mycob 1 M-----R------YPLGEALLALYRWRGPLINAGVG-GHGYTYLLGAEANRFVFANADAFSWSQTFESLV 58

CYP139A1_2581562358_Mycob 1 M-----R------YPLGEALLALYRWRGPLINAGVG-GHGYTYLLGAEANRFVFANADAFSWSQTFESLV 58

CYP139A1_2584107430_Mycob 1 M-----R------YPLGEALLALYRWRGPLINAGVG-GHGYTYLLGAEANRFVFANADAFSWSQTFESLV 58

CYP139A1_2590025444_Mycob 1 M-----R------YPLGEALLALYRWRGPLINAGVG-GHGYTYLLGAEANRFVFANADAFSWSQTFESLV 58

CYP139A1_2590040558_Mycob 1 M-----R------YPLGEALLALYRWRGPLINAGVG-GHGYTYLLGAEANRFVFANADAFSWSQTFESLV 58

CYP139A1_2590214646_Mycob 1 M-----R------YPLGEALLALYRWRGPLINAGVG-GHGYTYLLGAEANRFVFANADAFSWSQTFESLV 58

CYP139A1_2590223019_Mycob 1 M-----R------YPLGEALLALYRWRGPLINAGVG-GHGYTYLLGAEANRFVFANADAFSWSQTFESLV 58

CYP139A1_2590266966_Mycob 1 M-----R------YPLGEALLALYRWRGPLINAGVG-GHGYTYLLGAEANRFVFANADAFSWSQTFESLV 58

CYP139A1_647086307_Mycoba 1 M-----R------YPLGEALLALYRWRGPLINAGVG-GHGYTYLLGAEANRFVFANADAFSWSQTFESLV 58

CYP139A1_2574726119_Mycob 1 M-----R------YPLGEALLALYRWRGPLINAGVG-GHGYTYLLGAEANRFVFANADAFSWSQTFESLV 58

CYP139A1_2574757270_Mycob 1 M-----R------YPLGEALLALYRWRGPLINAGVG-GHGYTYLLGAEANRFVFANADAFSWSQTFESLV 58

CYP139A1_2575280304_Mycob 1 M-----R------YPLGEALLALYRWRGPLINAGVG-GHGYTYLLGAEANRFVFANADAFSWSQTFESLV 58

CYP139A1_2575601683_Mycob 1 M-----R------YPLGEALLALYRWRGPLINAGVG-GHGYTYLLGAEANRFVFANADAFSWSQTFESLV 58

CYP139A1_2576158036_Mycob 1 M-----R------YPLGEALLALYRWRGPLINAGVG-GHGYTYLLGAEANRFVFANADAFSWSQTFESLV 58

CYP139A1_2577689111_Mycob 1 M-----R------YPLGEALLALYRWRGPLINAGVG-GHGYTYLLGAEANRFVFANADAFSWSQTFESLV 58

CYP139A1_2577751179_Mycob 1 M-----R------YPLGEALLALYRWRGPLINAGVG-GHGYTYLLGAEANRFVFANADAFSWSQTFESLV 58

CYP139A1_2577845812_Mycob 1 M-----R------YPLGEALLALYRWRGPLINAGVG-GHGYTYLLGAEANRFVFANADAFSWSQTFESLV 58

CYP139A1_2577900964_Mycob 1 M-----R------YPLGEALLALYRWRGPLINAGVG-GHGYTYLLGAEANRFVFANADAFSWSQTFESLV 58

CYP139A1_2577988240_Mycob 1 M-----R------YPLGEALLALYRWRGPLINAGVG-GHGYTYLLGAEANRFVFANADAFSWSQTFESLV 58

CYP139A1_2578237814_Mycob 1 M-----R------YPLGEALLALYRWRGPLINAGVG-GHGYTYLLGAEANRFVFANADAFSWSQTFESLV 58

CYP139A1_2584703088_Mycob 1 M-----R------YPLGEALLALYRWRGPLINAGVG-GHGYTYLLGAEANRFVFANADAFSWSQTFESLV 58

CYP139A1_2584776403_Mycob 1 M-----R------YPLGEALLALYRWRGPLINAGVG-GHGYTYLLGAEANRFVFANADAFSWSQTFESLV 58

CYP139A1_2584785697_Mycob 1 M-----R------YPLGEALLALYRWRGPLINAGVG-GHGYTYLLGAEANRFVFANADAFSWSQTFESLV 58

CYP139A1_2584898862_Mycob 1 M-----R------YPLGEALLALYRWRGPLINAGVG-GHGYTYLLGAEANRFVFANADAFSWSQTFESLV 58

CYP139A1_2584906709_Mycob 1 M-----R------YPLGEALLALYRWRGPLINAGVG-GHGYTYLLGAEANRFVFANADAFSWSQTFESLV 58

CYP139A1_2589082097_Mycob 1 M-----R------YPLGEALLALYRWRGPLINAGVG-GHGYTYLLGAEANRFVFANADAFSWSQTFESLV 58

CYP139A1_2589142105_Mycob 1 M-----R------YPLGEALLALYRWRGPLINAGVG-GHGYTYLLGAEANRFVFANADAFSWSQTFESLV 58

CYP139A1_2589707360_Mycob 1 M-----R------YPLGEALLALYRWRGPLINAGVG-GHGYTYLLGAEANRFVFANADAFSWSQTFESLV 58

CYP139A1_2590142346_Mycob 1 M-----R------YPLGEALLALYRWRGPLINAGVG-GHGYTYLLGAEANRFVFANADAFSWSQTFESLV 58

CYP139A1_2592254166_Mycob 1 M-----R------YPLGEALLALYRWRGPLINAGVG-GHGYTYLLGAEANRFVFANADAFSWSQTFESLV 58

CYP139A1_2592348241_Mycob 1 M-----R------YPLGEALLALYRWRGPLINAGVG-GHGYTYLLGAEANRFVFANADAFSWSQTFESLV 58

CYP139A1_2592364277_Mycob 1 M-----R------YPLGEALLALYRWRGPLINAGVG-GHGYTYLLGAEANRFVFANADAFSWSQTFESLV 58

CYP139A1_2592537861_Mycob 1 M-----R------YPLGEALLALYRWRGPLINAGVG-GHGYTYLLGAEANRFVFANADAFSWSQTFESLV 58

CYP139A1_2546454904_Mycob 1 M-----R------YPLGEALLALYRWRGPLINAGVG-GHGYTYLLGAEANRFVFANADAFSWSQTFESLV 58

CYP139A1_2577218717_Mycob 1 M-----R------YPLGEALLALYRWRGPLINAGVG-GHGYTYLLGAEANRFVFANADAFSWSQTFESLV 58

CYP139A1_2577720788_Mycob 1 M-----R------YPLGEALLALYRWRGPLINAGVG-GHGYTYLLGAEANRFVFANADAFSWSQTFESLV 58

CYP139A1_2581366557_Mycob 1 M-----R------YPLGEALLALYRWRGPLINAGVG-GHGYTYLLGAEANRFVFANADAFSWSQTFESLV 58

CYP139A1_2584638962_Mycob 1 M-----R------YPLGEALLALYRWRGPLINAGVG-GHGYTYLLGAEANRFVFANADAFSWSQTFESLV 58

CYP139A1_2590154524_Mycob 1 M-----R------YPLGEALLALYRWRGPLINAGVG-GHGYTYLLGAEANRFVFANADAFSWSQTFESLV 58

CYP139A1_2590260165_Mycob 1 M-----R------YPLGEALLALYRWRGPLINAGVG-GHGYTYLLGAEANRFVFANADAFSWSQTFESLV 58

CYP139A1_2590539845_Mycob 1 M-----R------YPLGEALLALYRWRGPLINAGVG-GHGYTYLLGAEANRFVFANADAFSWSQTFESLV 58

CYP139A1_641783198_Mycoba 1 M-----R------YPLGEALLALYRWRGPLINAGVG-GHGYTYLLGAEANRFVFANADAFSWSQTFESLV 58

CYP139A1_643031783_Mycoba 1 M-----R------YPLGEALLALYRWRGPLINAGVG-GHGYTYLLGAEANRFVFANADAFSWSQTFESLV 58

CYP139A1_651039004_Mycoba 1 M-----R------YPLGEALLALYRWRGPLINAGVG-GHGYTYLLGAEANRFVFANADAFSWSQTFESLV 58

CYP139A1_2574843285_Mycob 1 M-----R------YPLGEALLALYRWRGPLINAGVG-GHGYTYLLGAEANRFVFANADAFSWSQTFESLV 58

CYP139A1_2575542837_Mycob 1 M-----R------YPLGEALLALYRWRGPLINAGVG-GHGYTYLLGAEANRFVFANADAFSWSQTFESLV 58

CYP139A1_2575709449_Mycob 1 M-----R------YPLGEALLALYRWRGPLINAGVG-GHGYTYLLGAEANRFVFANADAFSWSQTFESLV 58

CYP139A1_2576051820_Mycob 1 M-----R------YPLGEALLALYRWRGPLINAGVG-GHGYTYLLGAEANRFVFANADAFSWSQTFESLV 58

CYP139A1_2578094092_Mycob 1 M-----R------YPLGEALLALYRWRGPLINAGVG-GHGYTYLLGAEANRFVFANADAFSWSQTFESLV 58

CYP139A1_2584617586_Mycob 1 M-----R------YPLGEALLALYRWRGPLINAGVG-GHGYTYLLGAEANRFVFANADAFSWSQTFESLV 58

CYP139A1_2584694835_Mycob 1 M-----R------YPLGEALLALYRWRGPLINAGVG-GHGYTYLLGAEANRFVFANADAFSWSQTFESLV 58

CYP139A1_2584822720_Mycob 1 M-----R------YPLGEALLALYRWRGPLINAGVG-GHGYTYLLGAEANRFVFANADAFSWSQTFESLV 58

CYP139A1_2584931720_Mycob 1 M-----R------YPLGEALLALYRWRGPLINAGVG-GHGYTYLLGAEANRFVFANADAFSWSQTFESLV 58

CYP139A1_2589165303_Mycob 1 M-----R------YPLGEALLALYRWRGPLINAGVG-GHGYTYLLGAEANRFVFANADAFSWSQTFESLV 58

CYP139A1_2589608377_Mycob 1 M-----R------YPLGEALLALYRWRGPLINAGVG-GHGYTYLLGAEANRFVFANADAFSWSQTFESLV 58

CYP139A1_2589638476_Mycob 1 M-----R------YPLGEALLALYRWRGPLINAGVG-GHGYTYLLGAEANRFVFANADAFSWSQTFESLV 58

CYP139A1_2589687166_Mycob 1 M-----R------YPLGEALLALYRWRGPLINAGVG-GHGYTYLLGAEANRFVFANADAFSWSQTFESLV 58

CYP139A1_2590174897_Mycob 1 M-----R------YPLGEALLALYRWRGPLINAGVG-GHGYTYLLGAEANRFVFANADAFSWSQTFESLV 58

CYP139A1_2590370532_Mycob 1 M-----R------YPLGEALLALYRWRGPLINAGVG-GHGYTYLLGAEANRFVFANADAFSWSQTFESLV 58

CYP139A1_2592328029_Mycob 1 M-----R------YPLGEALLALYRWRGPLINAGVG-GHGYTYLLGAEANRFVFANADAFSWSQTFESLV 58

CYP139A1_2592426321_Mycob 1 M-----R------YPLGEALLALYRWRGPLINAGVG-GHGYTYLLGAEANRFVFANADAFSWSQTFESLV 58

CYP139A1_2575094331_Mycob 1 M-----R------YPLGEALLALYRWRGPLINAGVG-GHGYTYLLGAEANRFVFANADAFSWSQTFESLV 58

CYP139A1_2576101264_Mycob 1 M-----R------YPLGEALLALYRWRGPLINAGVG-GHGYTYLLGAEANRFVFANADAFSWSQTFESLV 58

CYP139A1_2576927477_Mycob 1 M-----R------YPLGEALLALYRWRGPLINAGVG-GHGYTYLLGAEANRFVFANADAFSWSQTFESLV 58

CYP139A1_2577009596_Mycob 1 M-----R------YPLGEALLALYRWRGPLINAGVG-GHGYTYLLGAEANRFVFANADAFSWSQTFESLV 58

CYP139A1_2577884547_Mycob 1 M-----R------YPLGEALLALYRWRGPLINAGVG-GHGYTYLLGAEANRFVFANADAFSWSQTFESLV 58

CYP139A1_2577997123_Mycob 1 M-----R------YPLGEALLALYRWRGPLINAGVG-GHGYTYLLGAEANRFVFANADAFSWSQTFESLV 58

CYP139A1_2578155745_Mycob 1 M-----R------YPLGEALLALYRWRGPLINAGVG-GHGYTYLLGAEANRFVFANADAFSWSQTFESLV 58

CYP139A1_2581807696_Mycob 1 M-----R------YPLGEALLALYRWRGPLINAGVG-GHGYTYLLGAEANRFVFANADAFSWSQTFESLV 58

CYP139A1_2582018155_Mycob 1 M-----R------YPLGEALLALYRWRGPLINAGVG-GHGYTYLLGAEANRFVFANADAFSWSQTFESLV 58

CYP139A1_2589154518_Mycob 1 M-----R------YPLGEALLALYRWRGPLINAGVG-GHGYTYLLGAEANRFVFANADAFSWSQTFESLV 58

CYP139A1_2590109466_Mycob 1 M-----R------YPLGEALLALYRWRGPLINAGVG-GHGYTYLLGAEANRFVFANADAFSWSQTFESLV 58

CYP139A1_2590195268_Mycob 1 M-----R------YPLGEALLALYRWRGPLINAGVG-GHGYTYLLGAEANRFVFANADAFSWSQTFESLV 58

CYP139A1_2590198349_Mycob 1 M-----R------YPLGEALLALYRWRGPLINAGVG-GHGYTYLLGAEANRFVFANADAFSWSQTFESLV 58

CYP139A1_2574799840_Mycob 1 M-----R------YPLGEALLALYRWRGPLINAGVG-GHGYTYLLGAEANRFVFANADAFSWSQTFESLV 58

CYP139A1_2576399693_Mycob 1 M-----R------YPLGEALLALYRWRGPLINAGVG-GHGYTYLLGAEANRFVFANADAFSWSQTFESLV 58

CYP139A1_2577024745_Mycob 1 M-----R------YPLGEALLALYRWRGPLINAGVG-GHGYTYLLGAEANRFVFANADAFSWSQTFESLV 58

CYP139A1_2577872587_Mycob 1 M-----R------YPLGEALLALYRWRGPLINAGVG-GHGYTYLLGAEANRFVFANADAFSWSQTFESLV 58

CYP139A1_2579813772_Mycob 1 M-----R------YPLGEALLALYRWRGPLINAGVG-GHGYTYLLGAEANRFVFANADAFSWSQTFESLV 58

CYP139A1_2584772421_Mycob 1 M-----R------YPLGEALLALYRWRGPLINAGVG-GHGYTYLLGAEANRFVFANADAFSWSQTFESLV 58

CYP139A1_2584893836_Mycob 1 M-----R------YPLGEALLALYRWRGPLINAGVG-GHGYTYLLGAEANRFVFANADAFSWSQTFESLV 58

CYP139A1_2589026697_Mycob 1 M-----R------YPLGEALLALYRWRGPLINAGVG-GHGYTYLLGAEANRFVFANADAFSWSQTFESLV 58

CYP139A1_2589514641_Mycob 1 M-----R------YPLGEALLALYRWRGPLINAGVG-GHGYTYLLGAEANRFVFANADAFSWSQTFESLV 58

CYP139A1_2589547247_Mycob 1 M-----R------YPLGEALLALYRWRGPLINAGVG-GHGYTYLLGAEANRFVFANADAFSWSQTFESLV 58

CYP139A1_2589620637_Mycob 1 M-----R------YPLGEALLALYRWRGPLINAGVG-GHGYTYLLGAEANRFVFANADAFSWSQTFESLV 58

CYP139A1_2592230560_Mycob 1 M-----R------YPLGEALLALYRWRGPLINAGVG-GHGYTYLLGAEANRFVFANADAFSWSQTFESLV 58

CYP139A1_2592242791_Mycob 1 M-----R------YPLGEALLALYRWRGPLINAGVG-GHGYTYLLGAEANRFVFANADAFSWSQTFESLV 58

CYP139A1_2592299949_Mycob 1 M-----R------YPLGEALLALYRWRGPLINAGVG-GHGYTYLLGAEANRFVFANADAFSWSQTFESLV 58

CYP139A1_2592377452_Mycob 1 M-----R------YPLGEALLALYRWRGPLINAGVG-GHGYTYLLGAEANRFVFANADAFSWSQTFESLV 58

CYP139A1_2592405959_Mycob 1 M-----R------YPLGEALLALYRWRGPLINAGVG-GHGYTYLLGAEANRFVFANADAFSWSQTFESLV 58

CYP139A1_2592558321_Mycob 1 M-----R------YPLGEALLALYRWRGPLINAGVG-GHGYTYLLGAEANRFVFANADAFSWSQTFESLV 58

CYP139A1_648456112_Mycoba 1 M-----R------YPLGEALLALYRWRGPLINAGVG-GHGYTYLLGAEANRFVFANADAFSWSQTFESLV 58

CYP139A1_2575561335_Mycob 1 M-----R------YPLGEALLALYRWRGPLINAGVG-GHGYTYLLGAEANRFVFANADAFSWSQTFESLV 58

CYP139A1_2575869049_Mycob 1 M-----R------YPLGEALLALYRWRGPLINAGVG-GHGYTYLLGAEANRFVFANADAFSWSQTFESLV 58

CYP139A1_2576250927_Mycob 1 M-----R------YPLGEALLALYRWRGPLINAGVG-GHGYTYLLGAEANRFVFANADAFSWSQTFESLV 58

CYP139A1_2577075963_Mycob 1 M-----R------YPLGEALLALYRWRGPLINAGVG-GHGYTYLLGAEANRFVFANADAFSWSQTFESLV 58

CYP139A1_2577655387_Mycob 1 M-----R------YPLGEALLALYRWRGPLINAGVG-GHGYTYLLGAEANRFVFANADAFSWSQTFESLV 58

CYP139A1_2579808474_Mycob 1 M-----R------YPLGEALLALYRWRGPLINAGVG-GHGYTYLLGAEANRFVFANADAFSWSQTFESLV 58

CYP139A1_2580939152_Mycob 1 M-----R------YPLGEALLALYRWRGPLINAGVG-GHGYTYLLGAEANRFVFANADAFSWSQTFESLV 58

CYP139A1_2581510874_Mycob 1 M-----R------YPLGEALLALYRWRGPLINAGVG-GHGYTYLLGAEANRFVFANADAFSWSQTFESLV 58

CYP139A1_2590048710_Mycob 1 M-----R------YPLGEALLALYRWRGPLINAGVG-GHGYTYLLGAEANRFVFANADAFSWSQTFESLV 58

CYP139A1_2590237321_Mycob 1 M-----R------YPLGEALLALYRWRGPLINAGVG-GHGYTYLLGAEANRFVFANADAFSWSQTFESLV 58

CYP139A1_2574784397_Mycob 1 M-----R------YPLGEALLALYRWRGPLINAGVG-GHGYTYLLGAEANRFVFANADAFSWSQTFESLV 58

CYP139A1_2574860198_Mycob 1 M-----R------YPLGEALLALYRWRGPLINAGVG-GHGYTYLLGAEANRFVFANADAFSWSQTFESLV 58

CYP139A1_2574872651_Mycob 1 M-----R------YPLGEALLALYRWRGPLINAGVG-GHGYTYLLGAEANRFVFANADAFSWSQTFESLV 58

CYP139A1_2575185207_Mycob 1 M-----R------YPLGEALLALYRWRGPLINAGVG-GHGYTYLLGAEANRFVFANADAFSWSQTFESLV 58

CYP139A1_2576316581_Mycob 1 M-----R------YPLGEALLALYRWRGPLINAGVG-GHGYTYLLGAEANRFVFANADAFSWSQTFESLV 58

CYP139A1_2576932272_Mycob 1 M-----R------YPLGEALLALYRWRGPLINAGVG-GHGYTYLLGAEANRFVFANADAFSWSQTFESLV 58

CYP139A1_2577632297_Mycob 1 M-----R------YPLGEALLALYRWRGPLINAGVG-GHGYTYLLGAEANRFVFANADAFSWSQTFESLV 58

CYP139A1_2577641910_Mycob 1 M-----R------YPLGEALLALYRWRGPLINAGVG-GHGYTYLLGAEANRFVFANADAFSWSQTFESLV 58

CYP139A1_2584609065_Mycob 1 M-----R------YPLGEALLALYRWRGPLINAGVG-GHGYTYLLGAEANRFVFANADAFSWSQTFESLV 58

CYP139A1_2584674477_Mycob 1 M-----R------YPLGEALLALYRWRGPLINAGVG-GHGYTYLLGAEANRFVFANADAFSWSQTFESLV 58

CYP139A1_2584806904_Mycob 1 M-----R------YPLGEALLALYRWRGPLINAGVG-GHGYTYLLGAEANRFVFANADAFSWSQTFESLV 58

CYP139A1_2584849650_Mycob 1 M-----R------YPLGEALLALYRWRGPLINAGVG-GHGYTYLLGAEANRFVFANADAFSWSQTFESLV 58

CYP139A1_2584854188_Mycob 1 M-----R------YPLGEALLALYRWRGPLINAGVG-GHGYTYLLGAEANRFVFANADAFSWSQTFESLV 58

CYP139A1_2589100068_Mycob 1 M-----R------YPLGEALLALYRWRGPLINAGVG-GHGYTYLLGAEANRFVFANADAFSWSQTFESLV 58

CYP139A1_2589666655_Mycob 1 M-----R------YPLGEALLALYRWRGPLINAGVG-GHGYTYLLGAEANRFVFANADAFSWSQTFESLV 58

CYP139A1_2589703490_Mycob 1 M-----R------YPLGEALLALYRWRGPLINAGVG-GHGYTYLLGAEANRFVFANADAFSWSQTFESLV 58

CYP139A1_2589724111_Mycob 1 M-----R------YPLGEALLALYRWRGPLINAGVG-GHGYTYLLGAEANRFVFANADAFSWSQTFESLV 58

CYP139A1_2590117900_Mycob 1 M-----R------YPLGEALLALYRWRGPLINAGVG-GHGYTYLLGAEANRFVFANADAFSWSQTFESLV 58

CYP139A1_2590134192_Mycob 1 M-----R------YPLGEALLALYRWRGPLINAGVG-GHGYTYLLGAEANRFVFANADAFSWSQTFESLV 58

CYP139A1_2590166763_Mycob 1 M-----R------YPLGEALLALYRWRGPLINAGVG-GHGYTYLLGAEANRFVFANADAFSWSQTFESLV 58

CYP139A1_2590356674_Mycob 1 M-----R------YPLGEALLALYRWRGPLINAGVG-GHGYTYLLGAEANRFVFANADAFSWSQTFESLV 58

CYP139A1_2549410800_Mycob 1 --------------------------RGPLINAGVG-GHGYTYLLGAEANRFVFANADAFSWSQTFESLV 43

CYP139A1_2575655283_Mycob 1 M-----R------YPLGEALLALYRWRGPLINAGVG-GHGYTYLLGAEANRFVFANADAFSWSQTFESLV 58

CYP139A1_2576373809_Mycob 1 M-----R------YPLGEALLALYRWRGPLINAGVG-GHGYTYLLGAEANRFVFANADAFSWSQTFESLV 58

CYP139A1_2576609942_Mycob 1 M-----R------YPLGEALLALYRWRGPLINAGVG-GHGYTYLLGAEANRFVFANADAFSWSQTFESLV 58

CYP139A1_2576684060_Mycob 1 M-----R------YPLGEALLALYRWRGPLINAGVG-GHGYTYLLGAEANRFVFANADAFSWSQTFESLV 58

CYP139A1_2577110129_Mycob 1 M-----R------YPLGEALLALYRWRGPLINAGVG-GHGYTYLLGAEANRFVFANADAFSWSQTFESLV 58

CYP139A1_2577733463_Mycob 1 M-----R------YPLGEALLALYRWRGPLINAGVG-GHGYTYLLGAEANRFVFANADAFSWSQTFESLV 58

CYP139A1_2578170996_Mycob 1 M-----R------YPLGEALLALYRWRGPLINAGVG-GHGYTYLLGAEANRFVFANADAFSWSQTFESLV 58

CYP139A1_2583731958_Mycob 1 M-----R------YPLGEALLALYRWRGPLINAGVG-GHGYTYLLGAEANRFVFANADAFSWSQTFESLV 58

CYP139A1_2590523531_Mycob 1 M-----R------YPLGEALLALYRWRGPLINAGVG-GHGYTYLLGAEANRFVFANADAFSWSQTFESLV 58

CYP139A1_2590526411_Mycob 1 M-----R------YPLGEALLALYRWRGPLINAGVG-GHGYTYLLGAEANRFVFANADAFSWSQTFESLV 58

CYP139A1_2590552289_Mycob 1 M-----R------YPLGEALLALYRWRGPLINAGVG-GHGYTYLLGAEANRFVFANADAFSWSQTFESLV 58

CYP139A1_2575205306_Mycob 1 M-----R------YPLGEALLALYRWRGPLINAGVG-GHGYTYLLGAEANRFVFANADAFSWSQTFESLV 58

CYP139A1_2575230562_Mycob 1 M-----R------YPLGEALLALYRWRGPLINAGVG-GHGYTYLLGAEANRFVFANADAFSWSQTFESLV 58

CYP139A1_2575304538_Mycob 1 M-----R------YPLGEALLALYRWRGPLINAGVG-GHGYTYLLGAEANRFVFANADAFSWSQTFESLV 58

CYP139A1_2575476916_Mycob 1 M-----R------YPLGEALLALYRWRGPLINAGVG-GHGYTYLLGAEANRFVFANADAFSWSQTFESLV 58

CYP139A1_2575790354_Mycob 1 M-----R------YPLGEALLALYRWRGPLINAGVG-GHGYTYLLGAEANRFVFANADAFSWSQTFESLV 58

CYP139A1_2576077839_Mycob 1 M-----R------YPLGEALLALYRWRGPLINAGVG-GHGYTYLLGAEANRFVFANADAFSWSQTFESLV 58

CYP139A1_2576163842_Mycob 1 M-----R------YPLGEALLALYRWRGPLINAGVG-GHGYTYLLGAEANRFVFANADAFSWSQTFESLV 58

CYP139A1_2577313382_Mycob 1 M-----R------YPLGEALLALYRWRGPLINAGVG-GHGYTYLLGAEANRFVFANADAFSWSQTFESLV 58

CYP139A1_2577519304_Mycob 1 M-----R------YPLGEALLALYRWRGPLINAGVG-GHGYTYLLGAEANRFVFANADAFSWSQTFESLV 58

CYP139A1_2577816400_Mycob 1 M-----R------YPLGEALLALYRWRGPLINAGVG-GHGYTYLLGAEANRFVFANADAFSWSQTFESLV 58

CYP139A1_2584670398_Mycob 1 M-----R------YPLGEALLALYRWRGPLINAGVG-GHGYTYLLGAEANRFVFANADAFSWSQTFESLV 58

CYP139A1_2584841030_Mycob 1 M-----R------YPLGEALLALYRWRGPLINAGVG-GHGYTYLLGAEANRFVFANADAFSWSQTFESLV 58

CYP139A1_2584865918_Mycob 1 M-----R------YPLGEALLALYRWRGPLINAGVG-GHGYTYLLGAEANRFVFANADAFSWSQTFESLV 58

CYP139A1_2589588224_Mycob 1 M-----R------YPLGEALLALYRWRGPLINAGVG-GHGYTYLLGAEANRFVFANADAFSWSQTFESLV 58

CYP139A1_2589624713_Mycob 1 M-----R------YPLGEALLALYRWRGPLINAGVG-GHGYTYLLGAEANRFVFANADAFSWSQTFESLV 58

CYP139A1_2589736180_Mycob 1 M-----R------YPLGEALLALYRWRGPLINAGVG-GHGYTYLLGAEANRFVFANADAFSWSQTFESLV 58

CYP139A1_2590032388_Mycob 1 M-----R------YPLGEALLALYRWRGPLINAGVG-GHGYTYLLGAEANRFVFANADAFSWSQTFESLV 58

CYP139A1_2590501611_Mycob 1 M-----R------YPLGEALLALYRWRGPLINAGVG-GHGYTYLLGAEANRFVFANADAFSWSQTFESLV 58

CYP139A1_2592312197_Mycob 1 M-----R------YPLGEALLALYRWRGPLINAGVG-GHGYTYLLGAEANRFVFANADAFSWSQTFESLV 58

CYP139A1_2592435284_Mycob 1 M-----R------YPLGEALLALYRWRGPLINAGVG-GHGYTYLLGAEANRFVFANADAFSWSQTFESLV 58

CYP139A1_2511811274_Mycob 1 M-----R------YPLGEALLALYRWRGPLINAGVG-GHGYTYLLGAEANRFVFANADAFSWSQTFESLV 58

CYP139A1_2546202077_Mycob 1 M-----R------YPLGEALLALYRWRGPLINAGVG-GHGYTYLLGAEANRFVFANADAFSWSQTFESLV 58

CYP139A1_2574693694_Mycob 1 M-----R------YPLGEALLALYRWRGPLINAGVG-GHGYTYLLGAEANRFVFANADAFSWSQTFESLV 58

CYP139A1_2574968392_Mycob 1 M-----R------YPLGEALLALYRWRGPLINAGVG-GHGYTYLLGAEANRFVFANADAFSWSQTFESLV 58

CYP139A1_2577060340_Mycob 1 M-----R------YPLGEALLALYRWRGPLINAGVG-GHGYTYLLGAEANRFVFANADAFSWSQTFESLV 58

CYP139A1_2577454945_Mycob 1 M-----R------YPLGEALLALYRWRGPLINAGVG-GHGYTYLLGAEANRFVFANADAFSWSQTFESLV 58

CYP139A1_2580123929_Mycob 1 M-----R------YPLGEALLALYRWRGPLINAGVG-GHGYTYLLGAEANRFVFANADAFSWSQTFESLV 58

CYP139A1_2581930746_Mycob 1 M-----R------YPLGEALLALYRWRGPLINAGVG-GHGYTYLLGAEANRFVFANADAFSWSQTFESLV 58

CYP139A1_2584196432_Mycob 1 M-----R------YPLGEALLALYRWRGPLINAGVG-GHGYTYLLGAEANRFVFANADAFSWSQTFESLV 58

CYP139A1_2584726832_Mycob 1 M-----R------YPLGEALLALYRWRGPLINAGVG-GHGYTYLLGAEANRFVFANADAFSWSQTFESLV 58

CYP139A1_2584911080_Mycob 1 M-----R------YPLGEALLALYRWRGPLINAGVG-GHGYTYLLGAEANRFVFANADAFSWSQTFESLV 58

CYP139A1_2584962435_Mycob 1 M-----R------YPLGEALLALYRWRGPLINAGVG-GHGYTYLLGAEANRFVFANADAFSWSQTFESLV 58

CYP139A1_2584995097_Mycob 1 M-----R------YPLGEALLALYRWRGPLINAGVG-GHGYTYLLGAEANRFVFANADAFSWSQTFESLV 58

CYP139A1_640602381_Mycoba 1 M-----R------YPLGEALLALYRWRGPLINAGVG-GHGYTYLLGAEANRFVFANADAFSWSQTFESLV 58

CYP139A1_643019022_Mycoba 1 M-----R------YPLGEALLALYRWRGPLINAGVG-GHGYTYLLGAEANRFVFANADAFSWSQTFESLV 58

CYP139A1_2573562450_Mycob 1 M-----R------YPLGEALLALYRWRGPLINAGVG-GHGYTYLLGAEANRFVFANADAFSWSQTFESLV 58

CYP139A1_2575255926_Mycob 1 M-----R------YPLGEALLALYRWRGPLINAGVG-GHGYTYLLGAEANRFVFANADAFSWSQTFESLV 58

CYP139A1_2577905307_Mycob 1 M-----R------YPLGEALLALYRWRGPLINAGVG-GHGYTYLLGAEANRFVFANADAFSWSQTFESLV 58

CYP139A1_2579818167_Mycob 1 M-----R------YPLGEALLALYRWRGPLINAGVG-GHGYTYLLGAEANRFVFANADAFSWSQTFESLV 58

CYP139A1_2582001898_Mycob 1 M-----R------YPLGEALLALYRWRGPLINAGVG-GHGYTYLLGAEANRFVFANADAFSWSQTFESLV 58

CYP139A1_2588538927_Mycob 1 M-----R------YPLGEALLALYRWRGPLINAGVG-GHGYTYLLGAEANRFVFANADAFSWSQTFESLV 58

CYP139A1_2590073317_Mycob 1 M-----R------YPLGEALLALYRWRGPLINAGVG-GHGYTYLLGAEANRFVFANADAFSWSQTFESLV 58

CYP139A1_2590075971_Mycob 1 M-----R------YPLGEALLALYRWRGPLINAGVG-GHGYTYLLGAEANRFVFANADAFSWSQTFESLV 58

CYP139A1_2590211264_Mycob 1 M-----R------YPLGEALLALYRWRGPLINAGVG-GHGYTYLLGAEANRFVFANADAFSWSQTFESLV 58

CYP139A1_2590249475_Mycob 1 M-----R------YPLGEALLALYRWRGPLINAGVG-GHGYTYLLGAEANRFVFANADAFSWSQTFESLV 58

CYP139A1_2590279314_Mycob 1 M-----R------YPLGEALLALYRWRGPLINAGVG-GHGYTYLLGAEANRFVFANADAFSWSQTFESLV 58

CYP139A1_2590559932_Mycob 1 M-----R------YPLGEALLALYRWRGPLINAGVG-GHGYTYLLGAEANRFVFANADAFSWSQTFESLV 58

CYP139A1_640606444_Mycoba 1 M-----R------YPLGEALLALYRWRGPLINAGVG-GHGYTYLLGAEANRFVFANADAFSWSQTFESLV 58

CYP139A1_651025167_Mycoba 1 M-----R------YPLGEALLALYRWRGPLINAGVG-GHGYTYLLGAEANRFVFANADAFSWSQTFESLV 58

CYP139A1_2574876573_Mycob 1 M-----R------YPLGEALLALYRWRGPLINAGVG-GHGYTYLLGAEANRFVFANADAFSWSQTFESLV 58

CYP139A1_2575028161_Mycob 1 M-----R------YPLGEALLALYRWRGPLINAGVG-GHGYTYLLGAEANRFVFANADAFSWSQTFESLV 58

CYP139A1_2575888575_Mycob 1 M-----R------YPLGEALLALYRWRGPLINAGVG-GHGYTYLLGAEANRFVFANADAFSWSQTFESLV 58

CYP139A1_2576708817_Mycob 1 M-----R------YPLGEALLALYRWRGPLINAGVG-GHGYTYLLGAEANRFVFANADAFSWSQTFESLV 58

CYP139A1_2577386815_Mycob 1 M-----R------YPLGEALLALYRWRGPLINAGVG-GHGYTYLLGAEANRFVFANADAFSWSQTFESLV 58

CYP139A1_2578040087_Mycob 1 M-----R------YPLGEALLALYRWRGPLINAGVG-GHGYTYLLGAEANRFVFANADAFSWSQTFESLV 58

CYP139A1_2584645880_Mycob 1 M-----R------YPLGEALLALYRWRGPLINAGVG-GHGYTYLLGAEANRFVFANADAFSWSQTFESLV 58

CYP139A1_2584659187_Mycob 1 M-----R------YPLGEALLALYRWRGPLINAGVG-GHGYTYLLGAEANRFVFANADAFSWSQTFESLV 58

CYP139A1_2584718757_Mycob 1 M-----R------YPLGEALLALYRWRGPLINAGVG-GHGYTYLLGAEANRFVFANADAFSWSQTFESLV 58

CYP139A1_2589064595_Mycob 1 M-----R------YPLGEALLALYRWRGPLINAGVG-GHGYTYLLGAEANRFVFANADAFSWSQTFESLV 58

CYP139A1_2589113575_Mycob 1 M-----R------YPLGEALLALYRWRGPLINAGVG-GHGYTYLLGAEANRFVFANADAFSWSQTFESLV 58

CYP139A1_2589559459_Mycob 1 M-----R------YPLGEALLALYRWRGPLINAGVG-GHGYTYLLGAEANRFVFANADAFSWSQTFESLV 58

CYP139A1_2589575466_Mycob 1 M-----R------YPLGEALLALYRWRGPLINAGVG-GHGYTYLLGAEANRFVFANADAFSWSQTFESLV 58

CYP139A1_2589595990_Mycob 1 M-----R------YPLGEALLALYRWRGPLINAGVG-GHGYTYLLGAEANRFVFANADAFSWSQTFESLV 58

CYP139A1_2592259141_Mycob 1 M-----R------YPLGEALLALYRWRGPLINAGVG-GHGYTYLLGAEANRFVFANADAFSWSQTFESLV 58

CYP139A1_2592280664_Mycob 1 M-----R------YPLGEALLALYRWRGPLINAGVG-GHGYTYLLGAEANRFVFANADAFSWSQTFESLV 58

CYP139A1_2592389699_Mycob 1 M-----R------YPLGEALLALYRWRGPLINAGVG-GHGYTYLLGAEANRFVFANADAFSWSQTFESLV 58

CYP139A1_2592438630_Mycob 1 M-----R------YPLGEALLALYRWRGPLINAGVG-GHGYTYLLGAEANRFVFANADAFSWSQTFESLV 58

CYP139A1_2592553482_Mycob 1 M-----R------YPLGEALLALYRWRGPLINAGVG-GHGYTYLLGAEANRFVFANADAFSWSQTFESLV 58

CYP139A1_2592570555_Mycob 1 M-----R------YPLGEALLALYRWRGPLINAGVG-GHGYTYLLGAEANRFVFANADAFSWSQTFESLV 58

CYP139A1_2575252379_Mycob 1 M-----R------YPLGEALLALYRWRGPLINAGVG-GHGYTYLLGAEANRFVFANADAFSWSQTFESLV 58

CYP139A1_2575520720_Mycob 1 M-----R------YPLGEALLALYRWRGPLINAGVG-GHGYTYLLGAEANRFVFANADAFSWSQTFESLV 58

CYP139A1_2575705222_Mycob 1 M-----R------YPLGEALLALYRWRGPLINAGVG-GHGYTYLLGAEANRFVFANADAFSWSQTFESLV 58

CYP139A1_2575964273_Mycob 1 M-----R------YPLGEALLALYRWRGPLINAGVG-GHGYTYLLGAEANRFVFANADAFSWSQTFESLV 58

CYP139A1_2577195360_Mycob 1 M-----R------YPLGEALLALYRWRGPLINAGVG-GHGYTYLLGAEANRFVFANADAFSWSQTFESLV 58

CYP139A1_2581587072_Mycob 1 M-----R------YPLGEALLALYRWRGPLINAGVG-GHGYTYLLGAEANRFVFANADAFSWSQTFESLV 58

CYP139A1_2584633235_Mycob 1 M-----R------YPLGEALLALYRWRGPLINAGVG-GHGYTYLLGAEANRFVFANADAFSWSQTFESLV 58

CYP139A1_2584732271_Mycob 1 M-----R------YPLGEALLALYRWRGPLINAGVG-GHGYTYLLGAEANRFVFANADAFSWSQTFESLV 58

CYP139A1_2584791771_Mycob 1 M-----R------YPLGEALLALYRWRGPLINAGVG-GHGYTYLLGAEANRFVFANADAFSWSQTFESLV 58

CYP139A1_2589148607_Mycob 1 M-----R------YPLGEALLALYRWRGPLINAGVG-GHGYTYLLGAEANRFVFANADAFSWSQTFESLV 58

CYP139A1_2590150448_Mycob 1 M-----R------YPLGEALLALYRWRGPLINAGVG-GHGYTYLLGAEANRFVFANADAFSWSQTFESLV 58

CYP139A1_2590231653_Mycob 1 M-----R------YPLGEALLALYRWRGPLINAGVG-GHGYTYLLGAEANRFVFANADAFSWSQTFESLV 58

CYP139A1_2590283895_Mycob 1 M-----R------YPLGEALLALYRWRGPLINAGVG-GHGYTYLLGAEANRFVFANADAFSWSQTFESLV 58

CYP139A1_645120373_Mycoba 1 M-----R------YPLGEALLALYRWRGPLINAGVG-GHGYTYLLGAEANRFVFANADAFSWSQTFESLV 58

CYP139A1_2574734007_Mycob 1 M-----R------YPLGEALLALYRWRGPLINAGVG-GHGYTYLLGAEANRFVFANADAFSWSQTFESLV 58

CYP139A1_2575099885_Mycob 1 M-----R------YPLGEALLALYRWRGPLINAGVG-GHGYTYLLGAEANRFVFANADAFSWSQTFESLV 58

CYP139A1_2575132657_Mycob 1 M-----R------YPLGEALLALYRWRGPLINAGVG-GHGYTYLLGAEANRFVFANADAFSWSQTFESLV 58

CYP139A1_2575465536_Mycob 1 M-----R------YPLGEALLALYRWRGPLINAGVG-GHGYTYLLGAEANRFVFANADAFSWSQTFESLV 58

CYP139A1_2576665707_Mycob 1 M-----R------YPLGEALLALYRWRGPLINAGVG-GHGYTYLLGAEANRFVFANADAFSWSQTFESLV 58

CYP139A1_2576698280_Mycob 1 M-----R------YPLGEALLALYRWRGPLINAGVG-GHGYTYLLGAEANRFVFANADAFSWSQTFESLV 58

CYP139A1_2576759003_Mycob 1 M-----R------YPLGEALLALYRWRGPLINAGVG-GHGYTYLLGAEANRFVFANADAFSWSQTFESLV 58

CYP139A1_2577673179_Mycob 1 M-----R------YPLGEALLALYRWRGPLINAGVG-GHGYTYLLGAEANRFVFANADAFSWSQTFESLV 58

CYP139A1_2577936335_Mycob 1 M-----R------YPLGEALLALYRWRGPLINAGVG-GHGYTYLLGAEANRFVFANADAFSWSQTFESLV 58

CYP139A1_2578008273_Mycob 1 M-----R------YPLGEALLALYRWRGPLINAGVG-GHGYTYLLGAEANRFVFANADAFSWSQTFESLV 58

CYP139A1_2578073775_Mycob 1 M-----R------YPLGEALLALYRWRGPLINAGVG-GHGYTYLLGAEANRFVFANADAFSWSQTFESLV 58

CYP139A1_2584960744_Mycob 1 M-----R------YPLGEALLALYRWRGPLINAGVG-GHGYTYLLGAEANRFVFANADAFSWSQTFESLV 58

CYP139A1_2588992251_Mycob 1 M-----R------YPLGEALLALYRWRGPLINAGVG-GHGYTYLLGAEANRFVFANADAFSWSQTFESLV 58

CYP139A1_2589077872_Mycob 1 M-----R------YPLGEALLALYRWRGPLINAGVG-GHGYTYLLGAEANRFVFANADAFSWSQTFESLV 58

CYP139A1_2589518744_Mycob 1 M-----R------YPLGEALLALYRWRGPLINAGVG-GHGYTYLLGAEANRFVFANADAFSWSQTFESLV 58

CYP139A1_2589583955_Mycob 1 M-----R------YPLGEALLALYRWRGPLINAGVG-GHGYTYLLGAEANRFVFANADAFSWSQTFESLV 58

CYP139A1_2589646609_Mycob 1 M-----R------YPLGEALLALYRWRGPLINAGVG-GHGYTYLLGAEANRFVFANADAFSWSQTFESLV 58

CYP139A1_2592316279_Mycob 1 M-----R------YPLGEALLALYRWRGPLINAGVG-GHGYTYLLGAEANRFVFANADAFSWSQTFESLV 58

CYP139A1_2592546111_Mycob 1 M-----R------YPLGEALLALYRWRGPLINAGVG-GHGYTYLLGAEANRFVFANADAFSWSQTFESLV 58

CYP139A1_2511553315_Mycob 1 M-----R------YPLGEALLALYRWRGPLINAGVG-GHGYTYLLGAEANRFVFANADAFSWSQTFESLV 58

CYP139A1_2574949227_Mycob 1 M-----R------YPLGEALLALYRWRGPLINAGVG-GHGYTYLLGAEANRFVFANADAFSWSQTFESLV 58

CYP139A1_2575533703_Mycob 1 M-----R------YPLGEALLALYRWRGPLINAGVG-GHGYTYLLGAEANRFVFANADAFSWSQTFESLV 58

CYP139A1_2576015739_Mycob 1 M-----R------YPLGEALLALYRWRGPLINAGVG-GHGYTYLLGAEANRFVFANADAFSWSQTFESLV 58

CYP139A1_2576644886_Mycob 1 M-----R------YPLGEALLALYRWRGPLINAGVG-GHGYTYLLGAEANRFVFANADAFSWSQTFESLV 58

CYP139A1_2577497204_Mycob 1 M-----R------YPLGEALLALYRWRGPLINAGVG-GHGYTYLLGAEANRFVFANADAFSWSQTFESLV 58

CYP139A1_2580744928_Mycob 1 M-----R------YPLGEALLALYRWRGPLINAGVG-GHGYTYLLGAEANRFVFANADAFSWSQTFESLV 58

CYP139A1_2583727889_Mycob 1 M-----R------YPLGEALLALYRWRGPLINAGVG-GHGYTYLLGAEANRFVFANADAFSWSQTFESLV 58

CYP139A1_2590064965_Mycob 1 M-----R------YPLGEALLALYRWRGPLINAGVG-GHGYTYLLGAEANRFVFANADAFSWSQTFESLV 58

CYP139A1_2590121989_Mycob 1 M-----R------YPLGEALLALYRWRGPLINAGVG-GHGYTYLLGAEANRFVFANADAFSWSQTFESLV 58

CYP139A1_2590294228_Mycob 1 M-----R------YPLGEALLALYRWRGPLINAGVG-GHGYTYLLGAEANRFVFANADAFSWSQTFESLV 58

CYP139A1_2590499050_Mycob 1 M-----R------YPLGEALLALYRWRGPLINAGVG-GHGYTYLLGAEANRFVFANADAFSWSQTFESLV 58

CYP139A1_2590519464_Mycob 1 M-----R------YPLGEALLALYRWRGPLINAGVG-GHGYTYLLGAEANRFVFANADAFSWSQTFESLV 58

CYP139A1_2590548005_Mycob 1 M-----R------YPLGEALLALYRWRGPLINAGVG-GHGYTYLLGAEANRFVFANADAFSWSQTFESLV 58

CYP139A1_637139034_Mycoba 1 M-----R------YPLGEALLALYRWRGPLINAGVG-GHGYTYLLGAEANRFVFANADAFSWSQTFESLV 58

CYP139A1_639830617_Mycoba 1 M-----R------YPLGEALLALYRWRGPLINAGVG-GHGYTYLLGAEANRFVFANADAFSWSQTFESLV 58

CYP139A1_2574640348_Mycob 1 M-----R------YPLGEALLALYRWRGPLINAGVG-GHGYTYLLGAEANRFVFANADAFSWSQTFESLV 58

CYP139A1_2574794413_Mycob 1 M-----R------YPLGEALLALYRWRGPLINAGVG-GHGYTYLLGAEANRFVFANADAFSWSQTFESLV 58

CYP139A1_2574987296_Mycob 1 M-----R------YPLGEALLALYRWRGPLINAGVG-GHGYTYLLGAEANRFVFANADAFSWSQTFESLV 58

CYP139A1_2575084753_Mycob 1 M-----R------YPLGEALLALYRWRGPLINAGVG-GHGYTYLLGAEANRFVFANADAFSWSQTFESLV 58

CYP139A1_2575627312_Mycob 1 M-----R------YPLGEALLALYRWRGPLINAGVG-GHGYTYLLGAEANRFVFANADAFSWSQTFESLV 58

CYP139A1_2576321347_Mycob 1 M-----R------YPLGEALLALYRWRGPLINAGVG-GHGYTYLLGAEANRFVFANADAFSWSQTFESLV 58

CYP139A1_2576378950_Mycob 1 M-----R------YPLGEALLALYRWRGPLINAGVG-GHGYTYLLGAEANRFVFANADAFSWSQTFESLV 58

CYP139A1_2576735552_Mycob 1 M-----R------YPLGEALLALYRWRGPLINAGVG-GHGYTYLLGAEANRFVFANADAFSWSQTFESLV 58

CYP139A1_2576940434_Mycob 1 M-----R------YPLGEALLALYRWRGPLINAGVG-GHGYTYLLGAEANRFVFANADAFSWSQTFESLV 58

CYP139A1_2577812038_Mycob 1 M-----R------YPLGEALLALYRWRGPLINAGVG-GHGYTYLLGAEANRFVFANADAFSWSQTFESLV 58

CYP139A1_2577861791_Mycob 1 M-----R------YPLGEALLALYRWRGPLINAGVG-GHGYTYLLGAEANRFVFANADAFSWSQTFESLV 58

CYP139A1_2578053379_Mycob 1 M-----R------YPLGEALLALYRWRGPLINAGVG-GHGYTYLLGAEANRFVFANADAFSWSQTFESLV 58

CYP139A1_2584860925_Mycob 1 M-----R------YPLGEALLALYRWRGPLINAGVG-GHGYTYLLGAEANRFVFANADAFSWSQTFESLV 58

CYP139A1_2584936325_Mycob 1 M-----R------YPLGEALLALYRWRGPLINAGVG-GHGYTYLLGAEANRFVFANADAFSWSQTFESLV 58

CYP139A1_2584939524_Mycob 1 M-----R------YPLGEALLALYRWRGPLINAGVG-GHGYTYLLGAEANRFVFANADAFSWSQTFESLV 58

CYP139A1_2589106145_Mycob 1 M-----R------YPLGEALLALYRWRGPLINAGVG-GHGYTYLLGAEANRFVFANADAFSWSQTFESLV 58

CYP139A1_2589543202_Mycob 1 M-----R------YPLGEALLALYRWRGPLINAGVG-GHGYTYLLGAEANRFVFANADAFSWSQTFESLV 58

CYP139A1_2589642534_Mycob 1 M-----R------YPLGEALLALYRWRGPLINAGVG-GHGYTYLLGAEANRFVFANADAFSWSQTFESLV 58

CYP139A1_2589691243_Mycob 1 M-----R------YPLGEALLALYRWRGPLINAGVG-GHGYTYLLGAEANRFVFANADAFSWSQTFESLV 58

CYP139A1_2589695547_Mycob 1 M-----R------YPLGEALLALYRWRGPLINAGVG-GHGYTYLLGAEANRFVFANADAFSWSQTFESLV 58

CYP139A1_2592222422_Mycob 1 M-----R------YPLGEALLALYRWRGPLINAGVG-GHGYTYLLGAEANRFVFANADAFSWSQTFESLV 58

CYP139A1_648446923_Mycoba 1 M-----R------YPLGEALLALYRWRGPLINAGVG-GHGYTYLLGAEANRFVFANADAFSWSQTFESLV 58

CYP139A1_648469578_Mycoba 1 M-----R------YPLGEALLALYRWRGPLINAGVG-GHGYTYLLGAEANRFVFANADAFSWSQTFESLV 58

CYP139A1_2546188127_Mycob 1 M-----R------YPLGEALLALYRWRGPLINAGVG-GHGYTYLLGAEANRFVFANADAFSWSQTFESLV 58

CYP139A1_2574773054_Mycob 1 M-----R------YPLGEALLALYRWRGPLINAGVG-GHGYTYLLGAEANRFVFANADAFSWSQTFESLV 58

CYP139A1_2574854728_Mycob 1 M-----R------YPLGEALLALYRWRGPLINAGVG-GHGYTYLLGAEANRFVFANADAFSWSQTFESLV 58

CYP139A1_2575984510_Mycob 1 M-----R------YPLGEALLALYRWRGPLINAGVG-GHGYTYLLGAEANRFVFANADAFSWSQTFESLV 58

CYP139A1_2576293562_Mycob 1 M-----R------YPLGEALLALYRWRGPLINAGVG-GHGYTYLLGAEANRFVFANADAFSWSQTFESLV 58

CYP139A1_2576717872_Mycob 1 M-----R------YPLGEALLALYRWRGPLINAGVG-GHGYTYLLGAEANRFVFANADAFSWSQTFESLV 58

CYP139A1_2578189802_Mycob 1 M-----R------YPLGEALLALYRWRGPLINAGVG-GHGYTYLLGAEANRFVFANADAFSWSQTFESLV 58

CYP139A1_2584890677_Mycob 1 M-----R------YPLGEALLALYRWRGPLINAGVG-GHGYTYLLGAEANRFVFANADAFSWSQTFESLV 58

CYP139A1_2590070071_Mycob 1 M-----R------YPLGEALLALYRWRGPLINAGVG-GHGYTYLLGAEANRFVFANADAFSWSQTFESLV 58

CYP139A1_2590169218_Mycob 1 M-----R------YPLGEALLALYRWRGPLINAGVG-GHGYTYLLGAEANRFVFANADAFSWSQTFESLV 58

CYP139A1_644880084_Mycoba 1 M-----R------YPLGEALLALYRWRGPLINAGVG-GHGYTYLLGAEANRFVFANADAFSWSQTFESLV 58

CYP139A1_2574905673_Mycob 1 M-----R------YPLGEALLALYRWRGPLINAGVG-GHGYTYLLGAEANRFVFANADAFSWSQTFESLV 58

CYP139A1_2575285636_Mycob 1 M-----R------YPLGEALLALYRWRGPLINAGVG-GHGYTYLLGAEANRFVFANADAFSWSQTFESLV 58

CYP139A1_2575508785_Mycob 1 M-----R------YPLGEALLALYRWRGPLINAGVG-GHGYTYLLGAEANRFVFANADAFSWSQTFESLV 58

CYP139A1_2576585830_Mycob 1 M-----R------YPLGEALLALYRWRGPLINAGVG-GHGYTYLLGAEANRFVFANADAFSWSQTFESLV 58

CYP139A1_2584979253_Mycob 1 M-----R------YPLGEALLALYRWRGPLINAGVG-GHGYTYLLGAEANRFVFANADAFSWSQTFESLV 58

CYP139A1_2588982991_Mycob 1 M-----R------YPLGEALLALYRWRGPLINAGVG-GHGYTYLLGAEANRFVFANADAFSWSQTFESLV 58

CYP139A1_2589048312_Mycob 1 M-----R------YPLGEALLALYRWRGPLINAGVG-GHGYTYLLGAEANRFVFANADAFSWSQTFESLV 58

CYP139A1_2589060525_Mycob 1 M-----R------YPLGEALLALYRWRGPLINAGVG-GHGYTYLLGAEANRFVFANADAFSWSQTFESLV 58

CYP139A1_2589502409_Mycob 1 M-----R------YPLGEALLALYRWRGPLINAGVG-GHGYTYLLGAEANRFVFANADAFSWSQTFESLV 58

CYP139A1_2589572815_Mycob 1 M-----R------YPLGEALLALYRWRGPLINAGVG-GHGYTYLLGAEANRFVFANADAFSWSQTFESLV 58

CYP139A1_2590181544_Mycob 1 M-----R------YPLGEALLALYRWRGPLINAGVG-GHGYTYLLGAEANRFVFANADAFSWSQTFESLV 58

CYP139A1_2592234638_Mycob 1 M-----R------YPLGEALLALYRWRGPLINAGVG-GHGYTYLLGAEANRFVFANADAFSWSQTFESLV 58

CYP139A1_2592271382_Mycob 1 M-----R------YPLGEALLALYRWRGPLINAGVG-GHGYTYLLGAEANRFVFANADAFSWSQTFESLV 58

CYP139A1_2592295853_Mycob 1 M-----R------YPLGEALLALYRWRGPLINAGVG-GHGYTYLLGAEANRFVFANADAFSWSQTFESLV 58

CYP139A1_2592344179_Mycob 1 M-----R------YPLGEALLALYRWRGPLINAGVG-GHGYTYLLGAEANRFVFANADAFSWSQTFESLV 58

CYP139A1_2592357110_Mycob 1 M-----R------YPLGEALLALYRWRGPLINAGVG-GHGYTYLLGAEANRFVFANADAFSWSQTFESLV 58

CYP139A1_643035957_Mycoba 1 M-----R------YPLGEALLALYRWRGPLINAGVG-GHGYTYLLGAEANRFVFANADAFSWSQTFESLV 58

CYP139A1_2575162612_Mycob 1 M-----R------YPLGEALLALYRWRGPLINAGVG-GHGYTYLLGAEANRFVFANADAFSWSQTFESLV 58

CYP139A1_2575198589_Mycob 1 M-----R------YPLGEALLALYRWRGPLINAGVG-GHGYTYLLGAEANRFVFANADAFSWSQTFESLV 58

CYP139A1_2575418829_Mycob 1 M-----R------YPLGEALLALYRWRGPLINAGVG-GHGYTYLLGAEANRFVFANADAFSWSQTFESLV 58

CYP139A1_2576172216_Mycob 1 M-----R------YPLGEALLALYRWRGPLINAGVG-GHGYTYLLGAEANRFVFANADAFSWSQTFESLV 58

CYP139A1_2576534922_Mycob 1 M-----R------YPLGEALLALYRWRGPLINAGVG-GHGYTYLLGAEANRFVFANADAFSWSQTFESLV 58

CYP139A1_2577185436_Mycob 1 M-----R------YPLGEALLALYRWRGPLINAGVG-GHGYTYLLGAEANRFVFANADAFSWSQTFESLV 58

CYP139A1_2577236838_Mycob 1 M-----R------YPLGEALLALYRWRGPLINAGVG-GHGYTYLLGAEANRFVFANADAFSWSQTFESLV 58

CYP139A1_2577373328_Mycob 1 M-----R------YPLGEALLALYRWRGPLINAGVG-GHGYTYLLGAEANRFVFANADAFSWSQTFESLV 58

CYP139A1_2578099005_Mycob 1 M-----R------YPLGEALLALYRWRGPLINAGVG-GHGYTYLLGAEANRFVFANADAFSWSQTFESLV 58

CYP139A1_2580366102_Mycob 1 M-----R------YPLGEALLALYRWRGPLINAGVG-GHGYTYLLGAEANRFVFANADAFSWSQTFESLV 58

CYP139A1_2580467795_Mycob 1 M-----R------YPLGEALLALYRWRGPLINAGVG-GHGYTYLLGAEANRFVFANADAFSWSQTFESLV 58

CYP139A1_2584613792_Mycob 1 M-----R------YPLGEALLALYRWRGPLINAGVG-GHGYTYLLGAEANRFVFANADAFSWSQTFESLV 58

CYP139A1_2584681396_Mycob 1 M-----R------YPLGEALLALYRWRGPLINAGVG-GHGYTYLLGAEANRFVFANADAFSWSQTFESLV 58

CYP139A1_2584913651_Mycob 1 M-----R------YPLGEALLALYRWRGPLINAGVG-GHGYTYLLGAEANRFVFANADAFSWSQTFESLV 58

CYP139A1_2584942354_Mycob 1 M-----R------YPLGEALLALYRWRGPLINAGVG-GHGYTYLLGAEANRFVFANADAFSWSQTFESLV 58

CYP139A1_2590093450_Mycob 1 M-----R------YPLGEALLALYRWRGPLINAGVG-GHGYTYLLGAEANRFVFANADAFSWSQTFESLV 58

CYP139A1_2590207004_Mycob 1 M-----R------YPLGEALLALYRWRGPLINAGVG-GHGYTYLLGAEANRFVFANADAFSWSQTFESLV 58

CYP139A1_2590276448_Mycob 1 M-----R------YPLGEALLALYRWRGPLINAGVG-GHGYTYLLGAEANRFVFANADAFSWSQTFESLV 58

CYP139A1_2575455196_Mycob 1 M-----R------YPLGEALLALYRWRGPLINAGVG-GHGYTYLLGAEANRFVFANADAFSWSQTFESLV 58

CYP139A1_2575920420_Mycob 1 M-----R------YPLGEALLALYRWRGPLINAGVG-GHGYTYLLGAEANRFVFANADAFSWSQTFESLV 58

CYP139A1_2575998368_Mycob 1 M-----R------YPLGEALLALYRWRGPLINAGVG-GHGYTYLLGAEANRFVFANADAFSWSQTFESLV 58

CYP139A1_2576583109_Mycob 1 M-----R------YPLGEALLALYRWRGPLINAGVG-GHGYTYLLGAEANRFVFANADAFSWSQTFESLV 58

CYP139A1_2576886343_Mycob 1 M-----R------YPLGEALLALYRWRGPLINAGVG-GHGYTYLLGAEANRFVFANADAFSWSQTFESLV 58

CYP139A1_2576986616_Mycob 1 M-----R------YPLGEALLALYRWRGPLINAGVG-GHGYTYLLGAEANRFVFANADAFSWSQTFESLV 58

CYP139A1_2577256179_Mycob 1 M-----R------YPLGEALLALYRWRGPLINAGVG-GHGYTYLLGAEANRFVFANADAFSWSQTFESLV 58

CYP139A1_2577876445_Mycob 1 M-----R------YPLGEALLALYRWRGPLINAGVG-GHGYTYLLGAEANRFVFANADAFSWSQTFESLV 58

CYP139A1_2579798813_Mycob 1 M-----R------YPLGEALLALYRWRGPLINAGVG-GHGYTYLLGAEANRFVFANADAFSWSQTFESLV 58

CYP139A1_2584715344_Mycob 1 M-----R------YPLGEALLALYRWRGPLINAGVG-GHGYTYLLGAEANRFVFANADAFSWSQTFESLV 58

CYP139A1_2588659602_Mycob 1 M-----R------YPLGEALLALYRWRGPLINAGVG-GHGYTYLLGAEANRFVFANADAFSWSQTFESLV 58

CYP139A1_2589089021_Mycob 1 M-----R------YPLGEALLALYRWRGPLINAGVG-GHGYTYLLGAEANRFVFANADAFSWSQTFESLV 58

CYP139A1_2589093094_Mycob 1 M-----R------YPLGEALLALYRWRGPLINAGVG-GHGYTYLLGAEANRFVFANADAFSWSQTFESLV 58

CYP139A1_2589535085_Mycob 1 M-----R------YPLGEALLALYRWRGPLINAGVG-GHGYTYLLGAEANRFVFANADAFSWSQTFESLV 58

CYP139A1_2589663797_Mycob 1 M-----R------YPLGEALLALYRWRGPLINAGVG-GHGYTYLLGAEANRFVFANADAFSWSQTFESLV 58

CYP139A1_2589671952_Mycob 1 M-----R------YPLGEALLALYRWRGPLINAGVG-GHGYTYLLGAEANRFVFANADAFSWSQTFESLV 58

CYP139A1_2589719807_Mycob 1 M-----R------YPLGEALLALYRWRGPLINAGVG-GHGYTYLLGAEANRFVFANADAFSWSQTFESLV 58

CYP139A1_2592246876_Mycob 1 M-----R------YPLGEALLALYRWRGPLINAGVG-GHGYTYLLGAEANRFVFANADAFSWSQTFESLV 58

CYP139A1_2592381635_Mycob 1 M-----R------YPLGEALLALYRWRGPLINAGVG-GHGYTYLLGAEANRFVFANADAFSWSQTFESLV 58

CYP139A1_2592410032_Mycob 1 M-----R------YPLGEALLALYRWRGPLINAGVG-GHGYTYLLGAEANRFVFANADAFSWSQTFESLV 58

CYP139A1_2592414205_Mycob 1 M-----R------YPLGEALLALYRWRGPLINAGVG-GHGYTYLLGAEANRFVFANADAFSWSQTFESLV 58

CYP139A1_2592566477_Mycob 1 M-----R------YPLGEALLALYRWRGPLINAGVG-GHGYTYLLGAEANRFVFANADAFSWSQTFESLV 58

CYP139A1_2575647889_Mycob 1 M-----R------YPLGEALLALYRWRGPLINAGVG-GHGYTYLLGAEANRFVFANADAFSWSQTFESLV 58

CYP139A1_2577429481_Mycob 1 M-----R------YPLGEALLALYRWRGPLINAGVG-GHGYTYLLGAEANRFVFANADAFSWSQTFESLV 58

CYP139A1_2578084434_Mycob 1 M-----R------YPLGEALLALYRWRGPLINAGVG-GHGYTYLLGAEANRFVFANADAFSWSQTFESLV 58

CYP139A1_2580301723_Mycob 1 M-----R------YPLGEALLALYRWRGPLINAGVG-GHGYTYLLGAEANRFVFANADAFSWSQTFESLV 58

CYP139A1_2581901399_Mycob 1 M-----R------YPLGEALLALYRWRGPLINAGVG-GHGYTYLLGAEANRFVFANADAFSWSQTFESLV 58

CYP139A1_2584689961_Mycob 1 M-----R------YPLGEALLALYRWRGPLINAGVG-GHGYTYLLGAEANRFVFANADAFSWSQTFESLV 58

CYP139A1_2584748012_Mycob 1 M-----R------YPLGEALLALYRWRGPLINAGVG-GHGYTYLLGAEANRFVFANADAFSWSQTFESLV 58

CYP139A1_2584878425_Mycob 1 M-----R------YPLGEALLALYRWRGPLINAGVG-GHGYTYLLGAEANRFVFANADAFSWSQTFESLV 58

CYP139A1_2584990570_Mycob 1 M-----R------YPLGEALLALYRWRGPLINAGVG-GHGYTYLLGAEANRFVFANADAFSWSQTFESLV 58

CYP139A1_2590014899_Mycob 1 M-----R------YPLGEALLALYRWRGPLINAGVG-GHGYTYLLGAEANRFVFANADAFSWSQTFESLV 58

CYP139A1_2590313607_Mycob 1 M-----R------YPLGEALLALYRWRGPLINAGVG-GHGYTYLLGAEANRFVFANADAFSWSQTFESLV 58

CYP139A1_2590563570_Mycob 1 M-----R------YPLGEALLALYRWRGPLINAGVG-GHGYTYLLGAEANRFVFANADAFSWSQTFESLV 58

CYP139A1_2574682482_Mycob 1 M-----R------YPLGEALLALYRWRGPLINAGVG-GHGYTYLLGAEANRFVFANADAFSWSQTFESLV 58

CYP139A1_2574937709_Mycob 1 M-----R------YPLGEALLALYRWRGPLINAGVG-GHGYTYLLGAEANRFVFANADAFSWSQTFESLV 58

CYP139A1_2575051511_Mycob 1 M-----R------YPLGEALLALYRWRGPLINAGVG-GHGYTYLLGAEANRFVFANADAFSWSQTFESLV 58

CYP139A1_2575142685_Mycob 1 M-----R------YPLGEALLALYRWRGPLINAGVG-GHGYTYLLGAEANRFVFANADAFSWSQTFESLV 58

CYP139A1_2575538950_Mycob 1 M-----R------YPLGEALLALYRWRGPLINAGVG-GHGYTYLLGAEANRFVFANADAFSWSQTFESLV 58

CYP139A1_2575858812_Mycob 1 M-----R------YPLGEALLALYRWRGPLINAGVG-GHGYTYLLGAEANRFVFANADAFSWSQTFESLV 58

CYP139A1_2576059958_Mycob 1 M-----R------YPLGEALLALYRWRGPLINAGVG-GHGYTYLLGAEANRFVFANADAFSWSQTFESLV 58

CYP139A1_2576471218_Mycob 1 M-----R------YPLGEALLALYRWRGPLINAGVG-GHGYTYLLGAEANRFVFANADAFSWSQTFESLV 58

CYP139A1_2576497080_Mycob 1 M-----R------YPLGEALLALYRWRGPLINAGVG-GHGYTYLLGAEANRFVFANADAFSWSQTFESLV 58

CYP139A1_2577038023_Mycob 1 M-----R------YPLGEALLALYRWRGPLINAGVG-GHGYTYLLGAEANRFVFANADAFSWSQTFESLV 58

CYP139A1_2577203844_Mycob 1 M-----R------YPLGEALLALYRWRGPLINAGVG-GHGYTYLLGAEANRFVFANADAFSWSQTFESLV 58

CYP139A1_2577322951_Mycob 1 M-----R------YPLGEALLALYRWRGPLINAGVG-GHGYTYLLGAEANRFVFANADAFSWSQTFESLV 58

CYP139A1_2577422627_Mycob 1 M-----R------YPLGEALLALYRWRGPLINAGVG-GHGYTYLLGAEANRFVFANADAFSWSQTFESLV 58

CYP139A1_2577551091_Mycob 1 M-----R------YPLGEALLALYRWRGPLINAGVG-GHGYTYLLGAEANRFVFANADAFSWSQTFESLV 58

CYP139A1_2584755863_Mycob 1 M-----R------YPLGEALLALYRWRGPLINAGVG-GHGYTYLLGAEANRFVFANADAFSWSQTFESLV 58

CYP139A1_2589117553_Mycob 1 M-----R------YPLGEALLALYRWRGPLINAGVG-GHGYTYLLGAEANRFVFANADAFSWSQTFESLV 58

CYP139A1_2589121735_Mycob 1 M-----R------YPLGEALLALYRWRGPLINAGVG-GHGYTYLLGAEANRFVFANADAFSWSQTFESLV 58

CYP139A1_2589506393_Mycob 1 M-----R------YPLGEALLALYRWRGPLINAGVG-GHGYTYLLGAEANRFVFANADAFSWSQTFESLV 58

CYP139A1_2590044637_Mycob 1 M-----R------YPLGEALLALYRWRGPLINAGVG-GHGYTYLLGAEANRFVFANADAFSWSQTFESLV 58

CYP139A1_2590137275_Mycob 1 M-----R------YPLGEALLALYRWRGPLINAGVG-GHGYTYLLGAEANRFVFANADAFSWSQTFESLV 58

CYP139A1_2590345757_Mycob 1 M-----R------YPLGEALLALYRWRGPLINAGVG-GHGYTYLLGAEANRFVFANADAFSWSQTFESLV 58

CYP139A1_2592275464_Mycob 1 M-----R------YPLGEALLALYRWRGPLINAGVG-GHGYTYLLGAEANRFVFANADAFSWSQTFESLV 58

CYP139A1_2575515927_Mycob 1 M-----R------YPLGEALLALYRWRGPLINAGVG-GHGYTYLLGAEANRFVFANADAFSWSQTFESLV 58

CYP139A1_2576459372_Mycob 1 M-----R------YPLGEALLALYRWRGPLINAGVG-GHGYTYLLGAEANRFVFANADAFSWSQTFESLV 58

CYP139A1_2576522568_Mycob 1 M-----R------YPLGEALLALYRWRGPLINAGVG-GHGYTYLLGAEANRFVFANADAFSWSQTFESLV 58

CYP139A1_2576559538_Mycob 1 M-----R------YPLGEALLALYRWRGPLINAGVG-GHGYTYLLGAEANRFVFANADAFSWSQTFESLV 58

CYP139A1_2577302440_Mycob 1 M-----R------YPLGEALLALYRWRGPLINAGVG-GHGYTYLLGAEANRFVFANADAFSWSQTFESLV 58

CYP139A1_2577448440_Mycob 1 M-----R------YPLGEALLALYRWRGPLINAGVG-GHGYTYLLGAEANRFVFANADAFSWSQTFESLV 58

CYP139A1_2577462548_Mycob 1 M-----R------YPLGEALLALYRWRGPLINAGVG-GHGYTYLLGAEANRFVFANADAFSWSQTFESLV 58

CYP139A1_2584678567_Mycob 1 M-----R------YPLGEALLALYRWRGPLINAGVG-GHGYTYLLGAEANRFVFANADAFSWSQTFESLV 58

CYP139A1_2584763258_Mycob 1 M-----R------YPLGEALLALYRWRGPLINAGVG-GHGYTYLLGAEANRFVFANADAFSWSQTFESLV 58

CYP139A1_2584949783_Mycob 1 M-----R------YPLGEALLALYRWRGPLINAGVG-GHGYTYLLGAEANRFVFANADAFSWSQTFESLV 58

CYP139A1_2589146274_Mycob 1 M-----R------YPLGEALLALYRWRGPLINAGVG-GHGYTYLLGAEANRFVFANADAFSWSQTFESLV 58

CYP139A1_2590089378_Mycob 1 M-----R------YPLGEALLALYRWRGPLINAGVG-GHGYTYLLGAEANRFVFANADAFSWSQTFESLV 58

CYP139A1_2590288661_Mycob 1 M-----R------YPLGEALLALYRWRGPLINAGVG-GHGYTYLLGAEANRFVFANADAFSWSQTFESLV 58

CYP139A1_2590513765_Mycob 1 M-----R------YPLGEALLALYRWRGPLINAGVG-GHGYTYLLGAEANRFVFANADAFSWSQTFESLV 58

CYP139A1_643045086_Mycoba 1 M-----R------YPLGEALLALYRWRGPLINAGVG-GHGYTYLLGAEANRFVFANADAFSWSQTFESLV 58

CYP139A1_646014426_Mycoba 1 M-----R------YPLGEALLALYRWRGPLINAGVG-GHGYTYLLGAEANRFVFANADAFSWSQTFESLV 58

CYP139A1_2574985446_Mycob 1 M-----R------YPLGEALLALYRWRGPLINAGVG-GHGYTYLLGAEANRFVFANADAFSWSQTFESLV 58

CYP139A1_2575103505_Mycob 1 M-----R------YPLGEALLALYRWRGPLINAGVG-GHGYTYLLGAEANRFVFANADAFSWSQTFESLV 58

CYP139A1_2575806159_Mycob 1 M-----R------YPLGEALLALYRWRGPLINAGVG-GHGYTYLLGAEANRFVFANADAFSWSQTFESLV 58

CYP139A1_2576156796_Mycob 1 M-----R------YPLGEALLALYRWRGPLINAGVG-GHGYTYLLGAEANRFVFANADAFSWSQTFESLV 58

CYP139A1_2576936333_Mycob 1 M-----R------YPLGEALLALYRWRGPLINAGVG-GHGYTYLLGAEANRFVFANADAFSWSQTFESLV 58

CYP139A1_2577030827_Mycob 1 M-----R------YPLGEALLALYRWRGPLINAGVG-GHGYTYLLGAEANRFVFANADAFSWSQTFESLV 58

CYP139A1_2577381729_Mycob 1 M-----R------YPLGEALLALYRWRGPLINAGVG-GHGYTYLLGAEANRFVFANADAFSWSQTFESLV 58

CYP139A1_2584698776_Mycob 1 M-----R------YPLGEALLALYRWRGPLINAGVG-GHGYTYLLGAEANRFVFANADAFSWSQTFESLV 58

CYP139A1_2584825730_Mycob 1 M-----R------YPLGEALLALYRWRGPLINAGVG-GHGYTYLLGAEANRFVFANADAFSWSQTFESLV 58

CYP139A1_2584885951_Mycob 1 M-----R------YPLGEALLALYRWRGPLINAGVG-GHGYTYLLGAEANRFVFANADAFSWSQTFESLV 58

CYP139A1_2584902930_Mycob 1 M-----R------YPLGEALLALYRWRGPLINAGVG-GHGYTYLLGAEANRFVFANADAFSWSQTFESLV 58

CYP139A1_2588995218_Mycob 1 M-----R------YPLGEALLALYRWRGPLINAGVG-GHGYTYLLGAEANRFVFANADAFSWSQTFESLV 58

CYP139A1_2589139119_Mycob 1 M-----R------YPLGEALLALYRWRGPLINAGVG-GHGYTYLLGAEANRFVFANADAFSWSQTFESLV 58

CYP139A1_2592238712_Mycob 1 M-----R------YPLGEALLALYRWRGPLINAGVG-GHGYTYLLGAEANRFVFANADAFSWSQTFESLV 58

CYP139A1_2592261699_Mycob 1 M-----R------YPLGEALLALYRWRGPLINAGVG-GHGYTYLLGAEANRFVFANADAFSWSQTFESLV 58

CYP139A1_2592393538_Mycob 1 M-----R------YPLGEALLALYRWRGPLINAGVG-GHGYTYLLGAEANRFVFANADAFSWSQTFESLV 58

CYP139A1_2592397895_Mycob 1 M-----R------YPLGEALLALYRWRGPLINAGVG-GHGYTYLLGAEANRFVFANADAFSWSQTFESLV 58

CYP139A1_2592549349_Mycob 1 M-----R------YPLGEALLALYRWRGPLINAGVG-GHGYTYLLGAEANRFVFANADAFSWSQTFESLV 58

CYP139A1_648464907_Mycoba 1 M-----R------YPLGEALLALYRWRGPLINAGVG-GHGYTYLLGAEANRFVFANADAFSWSQTFESLV 58

CYP139A1_648481186_Mycoba 1 M-----R------YPLGEALLALYRWRGPLINAGVG-GHGYTYLLGAEANRFVFANADAFSWSQTFESLV 58

CYP139A1_2574586310_Mycob 1 M-----R------YPLGEALLALYRWRGPLINAGVG-GHGYTYLLGAEANRFVFANADAFSWSQTFESLV 58

CYP139A1_2574663269_Mycob 1 M-----R------YPLGEALLALYRWRGPLINAGVG-GHGYTYLLGAEANRFVFANADAFSWSQTFESLV 58

CYP139A1_2574880930_Mycob 1 M-----R------YPLGEALLALYRWRGPLINAGVG-GHGYTYLLGAEANRFVFANADAFSWSQTFESLV 58

CYP139A1_2574911407_Mycob 1 M-----R------YPLGEALLALYRWRGPLINAGVG-GHGYTYLLGAEANRFVFANADAFSWSQTFESLV 58

CYP139A1_2575345775_Mycob 1 M-----R------YPLGEALLALYRWRGPLINAGVG-GHGYTYLLGAEANRFVFANADAFSWSQTFESLV 58

CYP139A1_2575468742_Mycob 1 M-----R------YPLGEALLALYRWRGPLINAGVG-GHGYTYLLGAEANRFVFANADAFSWSQTFESLV 58

CYP139A1_2576199760_Mycob 1 M-----R------YPLGEALLALYRWRGPLINAGVG-GHGYTYLLGAEANRFVFANADAFSWSQTFESLV 58

CYP139A1_2577069926_Mycob 1 M-----R------YPLGEALLALYRWRGPLINAGVG-GHGYTYLLGAEANRFVFANADAFSWSQTFESLV 58

CYP139A1_2577613318_Mycob 1 M-----R------YPLGEALLALYRWRGPLINAGVG-GHGYTYLLGAEANRFVFANADAFSWSQTFESLV 58

CYP139A1_2577684900_Mycob 1 M-----R------YPLGEALLALYRWRGPLINAGVG-GHGYTYLLGAEANRFVFANADAFSWSQTFESLV 58

CYP139A1_2582415442_Mycob 1 M-----R------YPLGEALLALYRWRGPLINAGVG-GHGYTYLLGAEANRFVFANADAFSWSQTFESLV 58

CYP139A1_2584812089_Mycob 1 M-----R------YPLGEALLALYRWRGPLINAGVG-GHGYTYLLGAEANRFVFANADAFSWSQTFESLV 58

CYP139A1_2590125975_Mycob 1 M-----R------YPLGEALLALYRWRGPLINAGVG-GHGYTYLLGAEANRFVFANADAFSWSQTFESLV 58

CYP139A1_2590219901_Mycob 1 M-----R------YPLGEALLALYRWRGPLINAGVG-GHGYTYLLGAEANRFVFANADAFSWSQTFESLV 58

CYP139A1_2590227570_Mycob 1 M-----R------YPLGEALLALYRWRGPLINAGVG-GHGYTYLLGAEANRFVFANADAFSWSQTFESLV 58

CYP139A1_2590317674_Mycob 1 M-----R------YPLGEALLALYRWRGPLINAGVG-GHGYTYLLGAEANRFVFANADAFSWSQTFESLV 58

CYP139A1_641814886_Mycoba 1 M-----R------YPLGEALLALYRWRGPLINAGVG-GHGYTYLLGAEANRFVFANADAFSWSQTFESLV 58

CYP139A1_2574738070_Mycob 1 M-----R------YPLGEALLALYRWRGPLINAGVG-GHGYTYLLGAEANRFVFANADAFSWSQTFESLV 58

CYP139A1_2575365848_Mycob 1 M-----R------YPLGEALLALYRWRGPLINAGVG-GHGYTYLLGAEANRFVFANADAFSWSQTFESLV 58

CYP139A1_2575674521_Mycob 1 M-----R------YPLGEALLALYRWRGPLINAGVG-GHGYTYLLGAEANRFVFANADAFSWSQTFESLV 58

CYP139A1_2575772716_Mycob 1 M-----R------YPLGEALLALYRWRGPLINAGVG-GHGYTYLLGAEANRFVFANADAFSWSQTFESLV 58

CYP139A1_2576544659_Mycob 1 M-----R------YPLGEALLALYRWRGPLINAGVG-GHGYTYLLGAEANRFVFANADAFSWSQTFESLV 58

CYP139A1_2577168470_Mycob 1 M-----R------YPLGEALLALYRWRGPLINAGVG-GHGYTYLLGAEANRFVFANADAFSWSQTFESLV 58

CYP139A1_2577390930_Mycob 1 M-----R------YPLGEALLALYRWRGPLINAGVG-GHGYTYLLGAEANRFVFANADAFSWSQTFESLV 58

CYP139A1_2577651582_Mycob 1 M-----R------YPLGEALLALYRWRGPLINAGVG-GHGYTYLLGAEANRFVFANADAFSWSQTFESLV 58

CYP139A1_2578002537_Mycob 1 M-----R------YPLGEALLALYRWRGPLINAGVG-GHGYTYLLGAEANRFVFANADAFSWSQTFESLV 58

CYP139A1_2578230674_Mycob 1 M-----R------YPLGEALLALYRWRGPLINAGVG-GHGYTYLLGAEANRFVFANADAFSWSQTFESLV 58

CYP139A1_2579825786_Mycob 1 M-----R------YPLGEALLALYRWRGPLINAGVG-GHGYTYLLGAEANRFVFANADAFSWSQTFESLV 58

CYP139A1_2584923296_Mycob 1 M-----R------YPLGEALLALYRWRGPLINAGVG-GHGYTYLLGAEANRFVFANADAFSWSQTFESLV 58

CYP139A1_2584954771_Mycob 1 M-----R------YPLGEALLALYRWRGPLINAGVG-GHGYTYLLGAEANRFVFANADAFSWSQTFESLV 58

CYP139A1_2588999278_Mycob 1 M-----R------YPLGEALLALYRWRGPLINAGVG-GHGYTYLLGAEANRFVFANADAFSWSQTFESLV 58

CYP139A1_2589044237_Mycob 1 M-----R------YPLGEALLALYRWRGPLINAGVG-GHGYTYLLGAEANRFVFANADAFSWSQTFESLV 58

CYP139A1_2589555384_Mycob 1 M-----R------YPLGEALLALYRWRGPLINAGVG-GHGYTYLLGAEANRFVFANADAFSWSQTFESLV 58

CYP139A1_2589568736_Mycob 1 M-----R------YPLGEALLALYRWRGPLINAGVG-GHGYTYLLGAEANRFVFANADAFSWSQTFESLV 58

CYP139A1_2589715710_Mycob 1 M-----R------YPLGEALLALYRWRGPLINAGVG-GHGYTYLLGAEANRFVFANADAFSWSQTFESLV 58

CYP139A1_2592287696_Mycob 1 M-----R------YPLGEALLALYRWRGPLINAGVG-GHGYTYLLGAEANRFVFANADAFSWSQTFESLV 58

CYP139A1_2592291479_Mycob 1 M-----R------YPLGEALLALYRWRGPLINAGVG-GHGYTYLLGAEANRFVFANADAFSWSQTFESLV 58

CYP139A1_2592430392_Mycob 1 M-----R------YPLGEALLALYRWRGPLINAGVG-GHGYTYLLGAEANRFVFANADAFSWSQTFESLV 58

CYP139A1_2574790496_Mycob 1 M-----R------YPLGEALLALYRWRGPLINAGVG-GHGYTYLLGAEANRFVFANADAFSWSQTFESLV 58

CYP139A1_2574834868_Mycob 1 M-----R------YPLGEALLALYRWRGPLINAGVG-GHGYTYLLGAEANRFVFANADAFSWSQTFESLV 58

CYP139A1_2576151818_Mycob 1 M-----R------YPLGEALLALYRWRGPLINAGVG-GHGYTYLLGAEANRFVFANADAFSWSQTFESLV 58

CYP139A1_2578111269_Mycob 1 M-----R------YPLGEALLALYRWRGPLINAGVG-GHGYTYLLGAEANRFVFANADAFSWSQTFESLV 58

CYP139A1_2583723841_Mycob 1 M-----R------YPLGEALLALYRWRGPLINAGVG-GHGYTYLLGAEANRFVFANADAFSWSQTFESLV 58

CYP139A1_2584631822_Mycob 1 M-----R------YPLGEALLALYRWRGPLINAGVG-GHGYTYLLGAEANRFVFANADAFSWSQTFESLV 58

CYP139A1_2584721998_Mycob 1 M-----R------YPLGEALLALYRWRGPLINAGVG-GHGYTYLLGAEANRFVFANADAFSWSQTFESLV 58

CYP139A1_2584846770_Mycob 1 M-----R------YPLGEALLALYRWRGPLINAGVG-GHGYTYLLGAEANRFVFANADAFSWSQTFESLV 58

CYP139A1_2588591267_Mycob 1 M-----R------YPLGEALLALYRWRGPLINAGVG-GHGYTYLLGAEANRFVFANADAFSWSQTFESLV 58

CYP139A1_2590007914_Mycob 1 M-----R------YPLGEALLALYRWRGPLINAGVG-GHGYTYLLGAEANRFVFANADAFSWSQTFESLV 58

CYP139A1_2590057066_Mycob 1 M-----R------YPLGEALLALYRWRGPLINAGVG-GHGYTYLLGAEANRFVFANADAFSWSQTFESLV 58

CYP139A1_2590061112_Mycob 1 M-----R------YPLGEALLALYRWRGPLINAGVG-GHGYTYLLGAEANRFVFANADAFSWSQTFESLV 58

CYP139A1_2590252317_Mycob 1 M-----R------YPLGEALLALYRWRGPLINAGVG-GHGYTYLLGAEANRFVFANADAFSWSQTFESLV 58

CYP139A1_2590543931_Mycob 1 M-----R------YPLGEALLALYRWRGPLINAGVG-GHGYTYLLGAEANRFVFANADAFSWSQTFESLV 58

CYP139A1_2574560432_Mycob 1 M-----R------YPLGEALLALYRWRGPLINAGVG-GHGYTYLLGAEANRFVFANADAFSWSQTFESLV 58

CYP139A1_2575495357_Mycob 1 M-----R------YPLGEALLALYRWRGPLINAGVG-GHGYTYLLGAEANRFVFANADAFSWSQTFESLV 58

CYP139A1_2575638157_Mycob 1 M-----R------YPLGEALLALYRWRGPLINAGVG-GHGYTYLLGAEANRFVFANADAFSWSQTFESLV 58

CYP139A1_2575697737_Mycob 1 M-----R------YPLGEALLALYRWRGPLINAGVG-GHGYTYLLGAEANRFVFANADAFSWSQTFESLV 58

CYP139A1_2575988382_Mycob 1 M-----R------YPLGEALLALYRWRGPLINAGVG-GHGYTYLLGAEANRFVFANADAFSWSQTFESLV 58

CYP139A1_2576070324_Mycob 1 M-----R------YPLGEALLALYRWRGPLINAGVG-GHGYTYLLGAEANRFVFANADAFSWSQTFESLV 58

CYP139A1_2576613004_Mycob 1 M-----R------YPLGEALLALYRWRGPLINAGVG-GHGYTYLLGAEANRFVFANADAFSWSQTFESLV 58

CYP139A1_2577091164_Mycob 1 M-----R------YPLGEALLALYRWRGPLINAGVG-GHGYTYLLGAEANRFVFANADAFSWSQTFESLV 58

CYP139A1_2577569748_Mycob 1 M-----R------YPLGEALLALYRWRGPLINAGVG-GHGYTYLLGAEANRFVFANADAFSWSQTFESLV 58

CYP139A1_2577697067_Mycob 1 M-----R------YPLGEALLALYRWRGPLINAGVG-GHGYTYLLGAEANRFVFANADAFSWSQTFESLV 58

CYP139A1_2584795634_Mycob 1 M-----R------YPLGEALLALYRWRGPLINAGVG-GHGYTYLLGAEANRFVFANADAFSWSQTFESLV 58

CYP139A1_2589158600_Mycob 1 M-----R------YPLGEALLALYRWRGPLINAGVG-GHGYTYLLGAEANRFVFANADAFSWSQTFESLV 58

CYP139A1_2589491285_Mycob 1 M-----R------YPLGEALLALYRWRGPLINAGVG-GHGYTYLLGAEANRFVFANADAFSWSQTFESLV 58

CYP139A1_2589613612_Mycob 1 M-----R------YPLGEALLALYRWRGPLINAGVG-GHGYTYLLGAEANRFVFANADAFSWSQTFESLV 58

CYP139A1_2589650472_Mycob 1 M-----R------YPLGEALLALYRWRGPLINAGVG-GHGYTYLLGAEANRFVFANADAFSWSQTFESLV 58

CYP139A1_2589699410_Mycob 1 M-----R------YPLGEALLALYRWRGPLINAGVG-GHGYTYLLGAEANRFVFANADAFSWSQTFESLV 58

CYP139A1_2590366449_Mycob 1 M-----R------YPLGEALLALYRWRGPLINAGVG-GHGYTYLLGAEANRFVFANADAFSWSQTFESLV 58

CYP139A1_2592226489_Mycob 1 M-----R------YPLGEALLALYRWRGPLINAGVG-GHGYTYLLGAEANRFVFANADAFSWSQTFESLV 58

CYP139A1_2592574645_Mycob 1 M-----R------YPLGEALLALYRWRGPLINAGVG-GHGYTYLLGAEANRFVFANADAFSWSQTFESLV 58

CYP139A1_648473432_Mycoba 1 M-----R------YPLGEALLALYRWRGPLINAGVG-GHGYTYLLGAEANRFVFANADAFSWSQTFESLV 58

CYP139A1_2574703562_Mycob 1 M-----R------YPLGEALLALYRWRGPLINAGVG-GHGYTYLLGAEANRFVFANADAFSWSQTFESLV 58

CYP139A1_2575794202_Mycob 1 M-----R------YPLGEALLALYRWRGPLINAGVG-GHGYTYLLGAEANRFVFANADAFSWSQTFESLV 58

CYP139A1_2576967330_Mycob 1 M-----R------YPLGEALLALYRWRGPLINAGVG-GHGYTYLLGAEANRFVFANADAFSWSQTFESLV 58

CYP139A1_2577659800_Mycob 1 M-----R------YPLGEALLALYRWRGPLINAGVG-GHGYTYLLGAEANRFVFANADAFSWSQTFESLV 58

CYP139A1_2577918576_Mycob 1 M-----R------YPLGEALLALYRWRGPLINAGVG-GHGYTYLLGAEANRFVFANADAFSWSQTFESLV 58

CYP139A1_2578033540_Mycob 1 M-----R------YPLGEALLALYRWRGPLINAGVG-GHGYTYLLGAEANRFVFANADAFSWSQTFESLV 58

CYP139A1_2581869776_Mycob 1 M-----R------YPLGEALLALYRWRGPLINAGVG-GHGYTYLLGAEANRFVFANADAFSWSQTFESLV 58

CYP139A1_2583745288_Mycob 1 M-----R------YPLGEALLALYRWRGPLINAGVG-GHGYTYLLGAEANRFVFANADAFSWSQTFESLV 58

CYP139A1_2590086658_Mycob 1 M-----R------YPLGEALLALYRWRGPLINAGVG-GHGYTYLLGAEANRFVFANADAFSWSQTFESLV 58

CYP139A1_2590104693_Mycob 1 M-----R------YPLGEALLALYRWRGPLINAGVG-GHGYTYLLGAEANRFVFANADAFSWSQTFESLV 58

CYP139A1_2590187116_Mycob 1 M-----R------YPLGEALLALYRWRGPLINAGVG-GHGYTYLLGAEANRFVFANADAFSWSQTFESLV 58

CYP139A1_2590382529_Mycob 1 M-----R------YPLGEALLALYRWRGPLINAGVG-GHGYTYLLGAEANRFVFANADAFSWSQTFESLV 58

CYP139A1_2590510255_Mycob 1 M-----R------YPLGEALLALYRWRGPLINAGVG-GHGYTYLLGAEANRFVFANADAFSWSQTFESLV 58

CYP139A1_2574635031_Mycob 1 M-----R------YPLGEALLALYRWRGPLINAGVG-GHGYTYLLGAEANRFVFANADAFSWSQTFESLV 58

CYP139A1_2576207497_Mycob 1 M-----R------YPLGEALLALYRWRGPLINAGVG-GHGYTYLLGAEANRFVFANADAFSWSQTFESLV 58

CYP139A1_2576443603_Mycob 1 M-----R------YPLGEALLALYRWRGPLINAGVG-GHGYTYLLGAEANRFVFANADAFSWSQTFESLV 58

CYP139A1_2577241442_Mycob 1 M-----R------YPLGEALLALYRWRGPLINAGVG-GHGYTYLLGAEANRFVFANADAFSWSQTFESLV 58

CYP139A1_2578177805_Mycob 1 M-----R------YPLGEALLALYRWRGPLINAGVG-GHGYTYLLGAEANRFVFANADAFSWSQTFESLV 58

CYP139A1_2578195506_Mycob 1 M-----R------YPLGEALLALYRWRGPLINAGVG-GHGYTYLLGAEANRFVFANADAFSWSQTFESLV 58

CYP139A1_2578246558_Mycob 1 M-----R------YPLGEALLALYRWRGPLINAGVG-GHGYTYLLGAEANRFVFANADAFSWSQTFESLV 58

CYP139A1_2584652546_Mycob 1 M-----R------YPLGEALLALYRWRGPLINAGVG-GHGYTYLLGAEANRFVFANADAFSWSQTFESLV 58

CYP139A1_2584737038_Mycob 1 M-----R------YPLGEALLALYRWRGPLINAGVG-GHGYTYLLGAEANRFVFANADAFSWSQTFESLV 58

CYP139A1_2584918375_Mycob 1 M-----R------YPLGEALLALYRWRGPLINAGVG-GHGYTYLLGAEANRFVFANADAFSWSQTFESLV 58

CYP139A1_2584974532_Mycob 1 M-----R------YPLGEALLALYRWRGPLINAGVG-GHGYTYLLGAEANRFVFANADAFSWSQTFESLV 58

CYP139A1_2588987052_Mycob 1 M-----R------YPLGEALLALYRWRGPLINAGVG-GHGYTYLLGAEANRFVFANADAFSWSQTFESLV 58

CYP139A1_2589098383_Mycob 1 M-----R------YPLGEALLALYRWRGPLINAGVG-GHGYTYLLGAEANRFVFANADAFSWSQTFESLV 58

CYP139A1_2589486069_Mycob 1 M-----R------YPLGEALLALYRWRGPLINAGVG-GHGYTYLLGAEANRFVFANADAFSWSQTFESLV 58

CYP139A1_2589522796_Mycob 1 M-----R------YPLGEALLALYRWRGPLINAGVG-GHGYTYLLGAEANRFVFANADAFSWSQTFESLV 58

CYP139A1_2589616566_Mycob 1 M-----R------YPLGEALLALYRWRGPLINAGVG-GHGYTYLLGAEANRFVFANADAFSWSQTFESLV 58

CYP139A1_2592250930_Mycob 1 M-----R------YPLGEALLALYRWRGPLINAGVG-GHGYTYLLGAEANRFVFANADAFSWSQTFESLV 58

CYP139A1_2592303254_Mycob 1 M-----R------YPLGEALLALYRWRGPLINAGVG-GHGYTYLLGAEANRFVFANADAFSWSQTFESLV 58

CYP139A1_2592308116_Mycob 1 M-----R------YPLGEALLALYRWRGPLINAGVG-GHGYTYLLGAEANRFVFANADAFSWSQTFESLV 58

CYP139A1_2592385592_Mycob 1 M-----R------YPLGEALLALYRWRGPLINAGVG-GHGYTYLLGAEANRFVFANADAFSWSQTFESLV 58

CYP139A1_2592417392_Mycob 1 M-----R------YPLGEALLALYRWRGPLINAGVG-GHGYTYLLGAEANRFVFANADAFSWSQTFESLV 58

CYP139A1_643049582_Mycoba 1 M-----R------YPLGEALLALYRWRGPLINAGVG-GHGYTYLLGAEANRFVFANADAFSWSQTFESLV 58

CYP139A1_648460473_Mycoba 1 M-----R------YPLGEALLALYRWRGPLINAGVG-GHGYTYLLGAEANRFVFANADAFSWSQTFESLV 58

CYP139A1_2574901332_Mycob 1 M-----R------YPLGEALLALYRWRGPLINAGVG-GHGYTYLLGAEANRFVFANADAFSWSQTFESLV 58

CYP139A1_2575325434_Mycob 1 M-----R------YPLGEALLALYRWRGPLINAGVG-GHGYTYLLGAEANRFVFANADAFSWSQTFESLV 58

CYP139A1_2576274603_Mycob 1 M-----R------YPLGEALLALYRWRGPLINAGVG-GHGYTYLLGAEANRFVFANADAFSWSQTFESLV 58

CYP139A1_2583719805_Mycob 1 M-----R------YPLGEALLALYRWRGPLINAGVG-GHGYTYLLGAEANRFVFANADAFSWSQTFESLV 58

CYP139A1_2590012031_Mycob 1 M-----R------YPLGEALLALYRWRGPLINAGVG-GHGYTYLLGAEANRFVFANADAFSWSQTFESLV 58

CYP139A_2590028529_Mycoba 1 M-----R------YPLGEALLALYRWRGPLINAGVG-GHGYTYLLGAEANRFVFANADAFSWSQTFESLV 58

CYP139A1_2590146409_Mycob 1 M-----R------YPLGEALLALYRWRGPLINAGVG-GHGYTYLLGAEANRFVFANADAFSWSQTFESLV 58

CYP139A1_2590203420_Mycob 1 M-----R------YPLGEALLALYRWRGPLINAGVG-GHGYTYLLGAEANRFVFANADAFSWSQTFESLV 58

CYP139A1_2590272661_Mycob 1 M-----R------YPLGEALLALYRWRGPLINAGVG-GHGYTYLLGAEANRFVFANADAFSWSQTFESLV 58

CYP139A1_2590535240_Mycob 1 M-----R------YPLGEALLALYRWRGPLINAGVG-GHGYTYLLGAEANRFVFANADAFSWSQTFESLV 58

CYP139A1_647090515_Mycoba 1 M-----R------YPLGEALLALYRWRGPLINAGVG-GHGYTYLLGAEANRFVFANADAFSWSQTFESLV 58

CYP139A1_2512786915_Mycob 1 M-----R------YPLGEALLALYRWRGPLINAGVG-GHGYTYLLGAEANRFVFANADAFSWSQTFESLV 58

CYP139A1_2575329342_Mycob 1 M-----R------YPLGEALLALYRWRGPLINAGVG-GHGYTYLLGAEANRFVFANADAFSWSQTFESLV 58

CYP139A1_2575409751_Mycob 1 M-----R------YPLGEALLALYRWRGPLINAGVG-GHGYTYLLGAEANRFVFANADAFSWSQTFESLV 58

CYP139A1_2575862603_Mycob 1 M-----R------YPLGEALLALYRWRGPLINAGVG-GHGYTYLLGAEANRFVFANADAFSWSQTFESLV 58

CYP139A1_2576589126_Mycob 1 M-----R------YPLGEALLALYRWRGPLINAGVG-GHGYTYLLGAEANRFVFANADAFSWSQTFESLV 58

CYP139A1_2576649037_Mycob 1 M-----R------YPLGEALLALYRWRGPLINAGVG-GHGYTYLLGAEANRFVFANADAFSWSQTFESLV 58

CYP139A1_2576746511_Mycob 1 M-----R------YPLGEALLALYRWRGPLINAGVG-GHGYTYLLGAEANRFVFANADAFSWSQTFESLV 58

CYP139A1_2577129296_Mycob 1 M-----R------YPLGEALLALYRWRGPLINAGVG-GHGYTYLLGAEANRFVFANADAFSWSQTFESLV 58

CYP139A1_2578200424_Mycob 1 M-----R------YPLGEALLALYRWRGPLINAGVG-GHGYTYLLGAEANRFVFANADAFSWSQTFESLV 58

CYP139A1_2584666311_Mycob 1 M-----R------YPLGEALLALYRWRGPLINAGVG-GHGYTYLLGAEANRFVFANADAFSWSQTFESLV 58

CYP139A1_2584743940_Mycob 1 M-----R------YPLGEALLALYRWRGPLINAGVG-GHGYTYLLGAEANRFVFANADAFSWSQTFESLV 58

CYP139A1_2584781205_Mycob 1 M-----R------YPLGEALLALYRWRGPLINAGVG-GHGYTYLLGAEANRFVFANADAFSWSQTFESLV 58

CYP139A1_2584833644_Mycob 1 M-----R------YPLGEALLALYRWRGPLINAGVG-GHGYTYLLGAEANRFVFANADAFSWSQTFESLV 58

CYP139A1_2589073023_Mycob 1 M-----R------YPLGEALLALYRWRGPLINAGVG-GHGYTYLLGAEANRFVFANADAFSWSQTFESLV 58

CYP139A1_2589509569_Mycob 1 M-----R------YPLGEALLALYRWRGPLINAGVG-GHGYTYLLGAEANRFVFANADAFSWSQTFESLV 58

CYP139A1_2589551314_Mycob 1 M-----R------YPLGEALLALYRWRGPLINAGVG-GHGYTYLLGAEANRFVFANADAFSWSQTFESLV 58

CYP139A1_2589580598_Mycob 1 M-----R------YPLGEALLALYRWRGPLINAGVG-GHGYTYLLGAEANRFVFANADAFSWSQTFESLV 58

CYP139A1_2589629003_Mycob 1 M-----R------YPLGEALLALYRWRGPLINAGVG-GHGYTYLLGAEANRFVFANADAFSWSQTFESLV 58

CYP139A1_2589676017_Mycob 1 M-----R------YPLGEALLALYRWRGPLINAGVG-GHGYTYLLGAEANRFVFANADAFSWSQTFESLV 58

CYP139A1_2592332604_Mycob 1 M-----R------YPLGEALLALYRWRGPLINAGVG-GHGYTYLLGAEANRFVFANADAFSWSQTFESLV 58

CYP139A1_2592542016_Mycob 1 M-----R------YPLGEALLALYRWRGPLINAGVG-GHGYTYLLGAEANRFVFANADAFSWSQTFESLV 58

CYP139A1_2592562396_Mycob 1 M-----R------YPLGEALLALYRWRGPLINAGVG-GHGYTYLLGAEANRFVFANADAFSWSQTFESLV 58

CYP139A1_2541578033_Mycob 1 M-----R------YPLGEALLALYRWRGPLINAGVG-GHGYTYLLGAEANRFVFANADAFSWSQTFESLV 58

CYP139A1_2574761421_Mycob 1 M-----R------YPLGEALLALYRWRGPLINAGVG-GHGYTYLLGAEANRFVFANADAFSWSQTFESLV 58

CYP139A1_2575611823_Mycob 1 M-----R------YPLGEALLALYRWRGPLINAGVG-GHGYTYLLGAEANRFVFANADAFSWSQTFESLV 58

CYP139A1_2576564801_Mycob 1 M-----R------YPLGEALLALYRWRGPLINAGVG-GHGYTYLLGAEANRFVFANADAFSWSQTFESLV 58

CYP139A1_2577821955_Mycob 1 M-----R------YPLGEALLALYRWRGPLINAGVG-GHGYTYLLGAEANRFVFANADAFSWSQTFESLV 58

CYP139A1_2578086064_Mycob 1 M-----R------YPLGEALLALYRWRGPLINAGVG-GHGYTYLLGAEANRFVFANADAFSWSQTFESLV 58

CYP139A1_2580669105_Mycob 1 M-----R------YPLGEALLALYRWRGPLINAGVG-GHGYTYLLGAEANRFVFANADAFSWSQTFESLV 58

CYP139A1_2583740670_Mycob 1 M-----R------YPLGEALLALYRWRGPLINAGVG-GHGYTYLLGAEANRFVFANADAFSWSQTFESLV 58

CYP139A1_2584831832_Mycob 1 M-----R------YPLGEALLALYRWRGPLINAGVG-GHGYTYLLGAEANRFVFANADAFSWSQTFESLV 58

CYP139A1_2584875064_Mycob 1 M-----R------YPLGEALLALYRWRGPLINAGVG-GHGYTYLLGAEANRFVFANADAFSWSQTFESLV 58

CYP139A1_2590158810_Mycob 1 M-----R------YPLGEALLALYRWRGPLINAGVG-GHGYTYLLGAEANRFVFANADAFSWSQTFESLV 58

CYP139A1_2590240089_Mycob 1 M-----R------YPLGEALLALYRWRGPLINAGVG-GHGYTYLLGAEANRFVFANADAFSWSQTFESLV 58

CYP139A1_2590350138_Mycob 1 M-----R------YPLGEALLALYRWRGPLINAGVG-GHGYTYLLGAEANRFVFANADAFSWSQTFESLV 58

CYP139A1_2590493738_Mycob 1 M-----R------YPLGEALLALYRWRGPLINAGVG-GHGYTYLLGAEANRFVFANADAFSWSQTFESLV 58

CYP139A1_2590554696_Mycob 1 M-----R------YPLGEALLALYRWRGPLINAGVG-GHGYTYLLGAEANRFVFANADAFSWSQTFESLV 58

CYP139A1_2576084283_Mycob 1 M-----R------YPLGEALLALYRWRGPLINAGVG-GHGYTYLLGAEANRFVFANADAFSWSQTFESLV 58

CYP139A1_2576366402_Mycob 1 M-----R------YPLGEALLALYRWRGPLINAGVG-GHGYTYLLGAEANRFVFANADAFSWSQTFESLV 58

CYP139A1_2576513411_Mycob 1 M-----R------YPLGEALLALYRWRGPLINAGVG-GHGYTYLLGAEANRFVFANADAFSWSQTFESLV 58

CYP139A1_2576671439_Mycob 1 M-----R------YPLGEALLALYRWRGPLINAGVG-GHGYTYLLGAEANRFVFANADAFSWSQTFESLV 58

CYP139A1_2577792721_Mycob 1 M-----R------YPLGEALLALYRWRGPLINAGVG-GHGYTYLLGAEANRFVFANADAFSWSQTFESLV 58

CYP139A1_2577796885_Mycob 1 M-----R------YPLGEALLALYRWRGPLINAGVG-GHGYTYLLGAEANRFVFANADAFSWSQTFESLV 58

CYP139A1_2584686721_Mycob 1 M-----R------YPLGEALLALYRWRGPLINAGVG-GHGYTYLLGAEANRFVFANADAFSWSQTFESLV 58

CYP139A1_2584752944_Mycob 1 M-----R------YPLGEALLALYRWRGPLINAGVG-GHGYTYLLGAEANRFVFANADAFSWSQTFESLV 58

CYP139A1_2584808933_Mycob 1 M-----R------YPLGEALLALYRWRGPLINAGVG-GHGYTYLLGAEANRFVFANADAFSWSQTFESLV 58

CYP139A1_2589134136_Mycob 1 M-----R------YPLGEALLALYRWRGPLINAGVG-GHGYTYLLGAEANRFVFANADAFSWSQTFESLV 58

CYP139A1_2589539130_Mycob 1 M-----R------YPLGEALLALYRWRGPLINAGVG-GHGYTYLLGAEANRFVFANADAFSWSQTFESLV 58

CYP139A1_2589598915_Mycob 1 M-----R------YPLGEALLALYRWRGPLINAGVG-GHGYTYLLGAEANRFVFANADAFSWSQTFESLV 58

CYP139A1_2589679190_Mycob 1 M-----R------YPLGEALLALYRWRGPLINAGVG-GHGYTYLLGAEANRFVFANADAFSWSQTFESLV 58

CYP139A1_2589727979_Mycob 1 M-----R------YPLGEALLALYRWRGPLINAGVG-GHGYTYLLGAEANRFVFANADAFSWSQTFESLV 58

CYP139A1_2592361217_Mycob 1 M-----R------YPLGEALLALYRWRGPLINAGVG-GHGYTYLLGAEANRFVFANADAFSWSQTFESLV 58

CYP139A1_2592442711_Mycob 1 M-----R------YPLGEALLALYRWRGPLINAGVG-GHGYTYLLGAEANRFVFANADAFSWSQTFESLV 58

CYP139A1_2555325132_Mycob 1 M-----RTYRTVRYPLGEALLALYRWRGPLINAGVG-GHGYTYLLGAEANRFVFANADAFSWSQTFESLV 64

CYP139A1_2566259019_Mycob 1 M-----RTYRTVRYPLGEALLALYRWRGPLINAGVG-GHGYTYLLGAEANRFVFANADAFSWSQTFESLV 64

CYP139A1_2555164435_Mycob 1 M-----RTYRTVRYPLGEALLALYRWRGPLINAGVG-GHGYTYLLGAEANRFVFANADAFSWSQTFESLV 64

CYP139A1_2555583039_Mycob 1 M-----RTYRTVRYPLGEALLALYRWRGPLINAGVG-GHGYTYLLGAEANRFVFANADAFSWSQTFESLV 64

CYP139A1_2555295099_Mycob 1 M-----RTYRTVRYPLGEALLALYRWRGPLINAGVG-GHGYTYLLGAEANRFVFANADAFSWSQTFESLV 64

CYP139A1_2555337752_Mycob 1 M-----RTYRTVRYPLGEALLALYRWRGPLINAGVG-GHGYTYLLGAEANRFVFANADAFSWSQTFESLV 64

CYP139A1_2555516018_Mycob 1 M-----RTYRTVRYPLGEALLALYRWRGPLINAGVG-GHGYTYLLGAEANRFVFANADAFSWSQTFESLV 64

CYP139A1_2576196505_Mycob 1 M-----R------YPLGEALLALYRWRGPLINAGVG-GHGYTYLLGAEANRFVFANADAFSWSQTFESLV 58

CYP139A1_2555417623_Mycob 1 M-----RTYRTVRYPLGEALLALYRWRGPLINAGVG-GHGYTYLLGAEANRFVFANADAFSWSQTFESLV 64

CYP139A1_2555430098_Mycob 1 M-----RTYRTVRYPLGEALLALYRWRGPLINAGVG-GHGYTYLLGAEANRFVFANADAFSWSQTFESLV 64

CYP139A1_2547880750_Mycob 1 M-----RTYRTVRYPLGEALLALYRWRGPLINAGVG-GHGYTYLLGAEANRFVFANADAFSWSQTFESLV 64

CYP139A1_2559163499_Mycob 1 M-----RTYRTVRYPLGEALLALYRWRGPLINAGVG-GHGYTYLLGAEANRFVFANADAFSWSQTFESLV 64

CYP139A1_2527056892_Mycob 1 M-----R------YPLGEALLALYRWRGPLINAGVG-GHGYTYLLGAEANRFVFANADAFSWSQTFESLV 58

CYP139A1_2574675630_Mycob 1 M-----R------YPLGEALLALYRWRGPLINAGVG-GHGYTYLLGAEANRFVFANADAFSWSQTFESLV 58

CYP139A1_2574700588_Mycob 1 M-----R------YPLGEALLALYRWRGPLINAGVG-GHGYTYLLGAEANRFVFANADAFSWSQTFESLV 58

CYP139A1_2574830606_Mycob 1 M-----R------YPLGEALLALYRWRGPLINAGVG-GHGYTYLLGAEANRFVFANADAFSWSQTFESLV 58

CYP139A1_2574928549_Mycob 1 M-----R------YPLGEALLALYRWRGPLINAGVG-GHGYTYLLGAEANRFVFANADAFSWSQTFESLV 58

CYP139A1_2575381682_Mycob 1 M-----R------YPLGEALLALYRWRGPLINAGVG-GHGYTYLLGAEANRFVFANADAFSWSQTFESLV 58

CYP139A1_2575663406_Mycob 1 M-----R------YPLGEALLALYRWRGPLINAGVG-GHGYTYLLGAEANRFVFANADAFSWSQTFESLV 58

CYP139A1_2576431117_Mycob 1 M-----R------YPLGEALLALYRWRGPLINAGVG-GHGYTYLLGAEANRFVFANADAFSWSQTFESLV 58

CYP139A1_2576553939_Mycob 1 M-----R------YPLGEALLALYRWRGPLINAGVG-GHGYTYLLGAEANRFVFANADAFSWSQTFESLV 58

CYP139A1_2577269438_Mycob 1 M-----R------YPLGEALLALYRWRGPLINAGVG-GHGYTYLLGAEANRFVFANADAFSWSQTFESLV 58

CYP139A1_2584870715_Mycob 1 M-----R------YPLGEALLALYRWRGPLINAGVG-GHGYTYLLGAEANRFVFANADAFSWSQTFESLV 58

CYP139A1_2589036086_Mycob 1 M-----R------YPLGEALLALYRWRGPLINAGVG-GHGYTYLLGAEANRFVFANADAFSWSQTFESLV 58

CYP139A1_2590101605_Mycob 1 M-----R------YPLGEALLALYRWRGPLINAGVG-GHGYTYLLGAEANRFVFANADAFSWSQTFESLV 58

CYP139A1_2590354422_Mycob 1 M-----R------YPLGEALLALYRWRGPLINAGVG-GHGYTYLLGAEANRFVFANADAFSWSQTFESLV 58

CYP139A1_648490022_Mycoba 1 M-----R------YPLGEALLALYRWRGPLINAGVG-GHGYTYLLGAEANRFVFANADAFSWSQTFESLV 58

CYP139A1_2547317188_Mycob 1 M-----RTYRTVRYPLGEALLALYRWRGPLINAGVG-GHGYTYLLGAEANRFVFANADAFSWSQTFESLV 64

CYP139A1_2547959756_Mycob 1 M-----RTYRTVRYPLGEALLALYRWRGPLINAGVG-GHGYTYLLGAEANRFVFANADAFSWSQTFESLV 64

CYP139A1_2555303475_Mycob 1 M-----RTYRTVRYPLGEALLALYRWRGPLINAGVG-GHGYTYLLGAEANRFVFANADAFSWSQTFESLV 64

CYP139A1_2555392373_Mycob 1 M-----RTYRTVRYPLGEALLALYRWRGPLINAGVG-GHGYTYLLGAEANRFVFANADAFSWSQTFESLV 64

CYP139A1_2566985178_Mycob 1 M-----RTYRTVRYPLGEALLALYRWRGPLINAGVG-GHGYTYLLGAEANRFVFANADAFSWSQTFESLV 64

CYP139A1_2555362970_Mycob 1 M-----RTYRTVRYPLGEALLALYRWRGPLINAGVG-GHGYTYLLGAEANRFVFANADAFSWSQTFESLV 64

CYP139A1_2560449536_Mycob 1 M-----RTYRTVRYPLGEALLALYRWRGPLINAGVG-GHGYTYLLGAEANRFVFANADAFSWSQTFESLV 64

CYP139A1_2555152706_Mycob 1 M-----RTYRTVRYPLGEALLALYRWRGPLINAGVG-GHGYTYLLGAEANRFVFANADAFSWSQTFESLV 64

CYP139A1_2555543565_Mycob 1 M-----RTYRTVRYPLGEALLALYRWRGPLINAGVG-GHGYTYLLGAEANRFVFANADAFSWSQTFESLV 64

CYP139A1_2590081259_Mycob 1 M-----R------YPLGEALLALYRWRGPLINAGVG-GHGYTYLLGAEANRFVFANADAFSWSQTFESLV 58

CYP139A1_2547311116_Mycob 1 M-----RTYRTVRYPLGEALLALYRWRGPLINAGVG-GHGYTYLLGAEANRFVFANADAFSWSQTFESLV 64

CYP139A1_2548033169_Mycob 1 M-----RTYRTVRYPLGEALLALYRWRGPLINAGVG-GHGYTYLLGAEANRFVFANADAFSWSQTFESLV 64

CYP139A1_2548037418_Mycob 1 M-----RTYRTVRYPLGEALLALYRWRGPLINAGVG-GHGYTYLLGAEANRFVFANADAFSWSQTFESLV 64

CYP139A1_2555282371_Mycob 1 M-----RTYRTVRYPLGEALLALYRWRGPLINAGVG-GHGYTYLLGAEANRFVFANADAFSWSQTFESLV 64

CYP139A1_2541569776_Mycob 1 M-----R------YPLGEALLALYRWRGPLINAGVG-GHGYTYLLGAEANRFVFANADAFSWSQTFESLV 58

CYP139A1_2555299255_Mycob 1 M-----RTYRTVRYPLGEALLALYRWRGPLINAGVG-GHGYTYLLGAEANRFVFANADAFSWSQTFESLV 64

CYP139A1_2555379694_Mycob 1 M-----RTYRTVRYPLGEALLALYRWRGPLINAGVG-GHGYTYLLGAEANRFVFANADAFSWSQTFESLV 64

CYP139A1_2555413387_Mycob 1 M-----RTYRTVRYPLGEALLALYRWRGPLINAGVG-GHGYTYLLGAEANRFVFANADAFSWSQTFESLV 64

CYP139A1_2555587223_Mycob 1 M-----RTYRTVRYPLGEALLALYRWRGPLINAGVG-GHGYTYLLGAEANRFVFANADAFSWSQTFESLV 64

CYP139A1_2555591399_Mycob 1 M-----RTYRTVRYPLGEALLALYRWRGPLINAGVG-GHGYTYLLGAEANRFVFANADAFSWSQTFESLV 64

CYP139A1_2547306531_Mycob 1 M-----RTYRTVRYPLGEALLALYRWRGPLINAGVG-GHGYTYLLGAEANRFVFANADAFSWSQTFESLV 64

CYP139A1_651088108_Mycoba 1 M-----RTYRTVRYPLGEALLALYRWRGPLINAGVG-GHGYTYLLGAEANRFVFANADAFSWSQTFESLV 64

CYP139A1_2598067418_Mycob 1 M-----RTYRTVRYPLGEALLALYRWRGPLINAGVG-GHGYTYLLGAEANRFVFANADAFSWSQTFESLV 64

CYP139A1_2555341950_Mycob 1 M-----RTYRTVRYPLGEALLALYRWRGPLINAGVG-GHGYTYLLGAEANRFVFANADAFSWSQTFESLV 64

CYP139A1_2555446824_Mycob 1 M-----RTYRTVRYPLGEALLALYRWRGPLINAGVG-GHGYTYLLGAEANRFVFANADAFSWSQTFESLV 64

CYP139A1_2555556540_Mycob 1 M-----RTYRTVRYPLGEALLALYRWRGPLINAGVG-GHGYTYLLGAEANRFVFANADAFSWSQTFESLV 64

CYP139A1_2560451827_Mycob 1 M-----RTYRTVRYPLGEALLALYRWRGPLINAGVG-GHGYTYLLGAEANRFVFANADAFSWSQTFESLV 64

CYP139A1_2540619998_Mycob 1 M-----RTYRTVRYPLGEALLALYRWRGPLINAGVG-GHGYTYLLGAEANRFVFANADAFSWSQTFESLV 64

CYP139A1_2554692349_Mycob 1 M-----RTYRTVRYPLGEALLALYRWRGPLINAGVG-GHGYTYLLGAEANRFVFANADAFSWSQTFESLV 64

CYP139A1_637096038_Mycoba 1 M-----RTYRTVRYPLGEALLALYRWRGPLINAGVG-GHGYTYLLGAEANRFVFANADAFSWSQTFESLV 64

CYP139A1_2555396597_Mycob 1 M-----RTYRTVRYPLGEALLALYRWRGPLINAGVG-GHGYTYLLGAEANRFVFANADAFSWSQTFESLV 64

CYP139A1_2555520186_Mycob 1 M-----RTYRTVRYPLGEALLALYRWRGPLINAGVG-GHGYTYLLGAEANRFVFANADAFSWSQTFESLV 64

CYP139A1_2555599804_Mycob 1 M-----RTYRTVRYPLGEALLALYRWRGPLINAGVG-GHGYTYLLGAEANRFVFANADAFSWSQTFESLV 64

CYP139A1_2547314995_Mycob 1 M-----RTYRTVRYPLGEALLALYRWRGPLINAGVG-GHGYTYLLGAEANRFVFANADAFSWSQTFESLV 64

CYP139A1_2555160272_Mycob 1 M-----RTYRTVRYPLGEALLALYRWRGPLINAGVG-GHGYTYLLGAEANRFVFANADAFSWSQTFESLV 64

CYP139A1_2555367201_Mycob 1 M-----RTYRTVRYPLGEALLALYRWRGPLINAGVG-GHGYTYLLGAEANRFVFANADAFSWSQTFESLV 64

CYP139A1_2555547894_Mycob 1 M-----RTYRTVRYPLGEALLALYRWRGPLINAGVG-GHGYTYLLGAEANRFVFANADAFSWSQTFESLV 64

CYP139A1_2560454644_Mycob 1 M-----RTYRTVRYPLGEALLALYRWRGPLINAGVG-GHGYTYLLGAEANRFVFANADAFSWSQTFESLV 64

CYP139A1_2546436155_Mycob 1 M-----R------YPLGEALLALYRWRGPLINAGVG-GHGYTYLLGAEANRFVFANADAFSWSQTFESLV 58

CYP139A1_2555307696_Mycob 1 M-----RTYRTVRYPLGEALLALYRWRGPLINAGVG-GHGYTYLLGAEANRFVFANADAFSWSQTFESLV 64

CYP139A1_2555524372_Mycob 1 M-----RTYRTVRYPLGEALLALYRWRGPLINAGVG-GHGYTYLLGAEANRFVFANADAFSWSQTFESLV 64

CYP139A1_2546192085_Mycob 1 M-----R------YPLGEALLALYRWRGPLINAGVG-GHGYTYLLGAEANRFVFANADAFSWSQTFESLV 58

CYP139A1_2553261414_Mycob 1 M-----RTYRTVRYPLGEALLALYRWRGPLINAGVG-GHGYTYLLGAEANRFVFANADAFSWSQTFESLV 64

CYP139A1_2555442648_Mycob 1 M-----RTYRTVRYPLGEALLALYRWRGPLINAGVG-GHGYTYLLGAEANRFVFANADAFSWSQTFESLV 64

CYP139A1_2555455224_Mycob 1 M-----RTYRTVRYPLGEALLALYRWRGPLINAGVG-GHGYTYLLGAEANRFVFANADAFSWSQTFESLV 64

CYP139A1_651084428_Mycoba 1 M-----RTYRTVRYPLGEALLALYRWRGPLINAGVG-GHGYTYLLGAEANRFVFANADAFSWSQTFESLV 64

CYP139A1_2555273883_Mycob 1 M-----RTYRTVRYPLGEALLALYRWRGPLINAGVG-GHGYTYLLGAEANRFVFANADAFSWSQTFESLV 64

CYP139A1_2555286609_Mycob 1 M-----RTYRTVRYPLGEALLALYRWRGPLINAGVG-GHGYTYLLGAEANRFVFANADAFSWSQTFESLV 64

CYP139A1_2555329342_Mycob 1 M-----RTYRTVRYPLGEALLALYRWRGPLINAGVG-GHGYTYLLGAEANRFVFANADAFSWSQTFESLV 64

CYP139A1_2555438445_Mycob 1 M-----RTYRTVRYPLGEALLALYRWRGPLINAGVG-GHGYTYLLGAEANRFVFANADAFSWSQTFESLV 64

CYP139A1_2577281457_Mycob 1 M-----RTYRTVRYPLGEALLALYRWRGPLINAGVG-GHGYTYLLGAEANRFVFANADAFSWSQTFESLV 64

CYP139A1_2590256393_Mycob 1 M-----R------YPLGEALLALYRWRGPLINAGVG-GHGYTYLLGAEANRFVFANADAFSWSQTFESLV 58

CYP139A1_2549407735_Mycob 1 M-----RTYRTVRYPLGEALLALYRWRGPLINAGVG-GHGYTYLLGAEANRFVFANADAFSWSQTFESLV 64

CYP139A1_2573574061_Mycob 1 M-----RTYRTVRYPLGEALLALYRWRGPLINAGVG-GHGYTYLLGAEANRFVFANADAFSWSQTFESLV 64

CYP139A1_2540803840_Mycob 1 M-----RTYRTVRYPLGEALLALYRWRGPLINAGVG-GHGYTYLLGAEANRFVFANADAFSWSQTFESLV 64

CYP139A1_2584769363_Mycob 1 M-----R------YPLGEALLALYRWRGPLINAGVG-GHGYTYLLGAEANRFVFANADAFSWSQTFESLV 58

CYP139A1_2555311951_Mycob 1 M-----RTYRTVRYPLGEALLALYRWRGPLINAGVG-GHGYTYLLGAEANRFVFANADAFSWSQTFESLV 64

CYP139A1_2555346108_Mycob 1 M-----RTYRTVRYPLGEALLALYRWRGPLINAGVG-GHGYTYLLGAEANRFVFANADAFSWSQTFESLV 64

CYP139A1_2555383921_Mycob 1 M-----RTYRTVRYPLGEALLALYRWRGPLINAGVG-GHGYTYLLGAEANRFVFANADAFSWSQTFESLV 64

CYP139A1_2555400811_Mycob 1 M-----RTYRTVRYPLGEALLALYRWRGPLINAGVG-GHGYTYLLGAEANRFVFANADAFSWSQTFESLV 64

CYP139A1_2555560826_Mycob 1 M-----RTYRTVRYPLGEALLALYRWRGPLINAGVG-GHGYTYLLGAEANRFVFANADAFSWSQTFESLV 64

CYP139A1_2598813154_Mycob 1 M-----RTYRTVRYPLGEALLALYRWRGPLINAGVG-GHGYTYLLGAEANRFVFANADAFSWSQTFESLV 64

CYP139A1_2547164190_Mycob 1 M-----RTYRTVRYPLGEALLALYRWRGPLINAGVG-GHGYTYLLGAEANRFVFANADAFSWSQTFESLV 64

CYP139A1_2555409181_Mycob 1 M-----RTYRTVRYPLGEALLALYRWRGPLINAGVG-GHGYTYLLGAEANRFVFANADAFSWSQTFESLV 64

CYP139A1_2547759833_Mycob 1 M-----RTYRTVRYPLGEALLALYRWRGPLINAGVG-GHGYTYLLGAEANRFVFANADAFSWSQTFESLV 64

CYP139A1_2547955540_Mycob 1 M-----RTYRTVRYPLGEALLALYRWRGPLINAGVG-GHGYTYLLGAEANRFVFANADAFSWSQTFESLV 64

CYP139A1_2560461324_Mycob 1 M-----RTYRTVRYPLGEALLALYRWRGPLINAGVG-GHGYTYLLGAEANRFVFANADAFSWSQTFESLV 64

CYP139A1_2576392640_Mycob 1 M-----R------YPLGEALLALYRWRGPLINAGVG-GHGYTYLLGAEANRFVFANADAFSWSQTFESLV 58

CYP139A1_2541573920_Mycob 1 M-----R------YPLGEALLALYRWRGPLINAGVG-GHGYTYLLGAEANRFVFANADAFSWSQTFESLV 58

CYP139A1_2549413118_Mycob 1 M-----RTYRTVRYPLGEALLALYRWRGPLINAGVG-GHGYTYLLGAEANRFVFANADAFSWSQTFESLV 64

CYP139A1_2555333539_Mycob 1 M-----RTYRTVRYPLGEALLALYRWRGPLINAGVG-GHGYTYLLGAEANRFVFANADAFSWSQTFESLV 64

CYP139A1_2555371358_Mycob 1 M-----RTYRTVRYPLGEALLALYRWRGPLINAGVG-GHGYTYLLGAEANRFVFANADAFSWSQTFESLV 64

CYP139A1_2545499027_Mycob 1 M-----RTYRTVRYPLGEALLALYRWRGPLINAGVG-GHGYTYLLGAEANRFVFANADAFSWSQTFESLV 64

CYP139A1_2551812688_Mycob 1 M-----RTYRTVRYPLGEALLALYRWRGPLINAGVG-GHGYTYLLGAEANRFVFANADAFSWSQTFESLV 64

CYP139A1_2620699696_Mycob 1 M-----RTYRTVRYPLGEALLALYRWRGPLINAGVG-GHGYTYLLGAEANRFVFANADAFSWSQTFESLV 64

CYP139A1_2555278104_Mycob 1 M-----RTYRTVRYPLGEALLALYRWRGPLINAGVG-GHGYTYLLGAEANRFVFANADAFSWSQTFESLV 64

CYP139A1_2555320646_Mycob 1 M-----RTYRTVRYPLGEALLALYRWRGPLINAGVG-GHGYTYLLGAEANRFVFANADAFSWSQTFESLV 64

CYP139A1_2555354513_Mycob 1 M-----RTYRTVRYPLGEALLALYRWRGPLINAGVG-GHGYTYLLGAEANRFVFANADAFSWSQTFESLV 64

CYP139A1_2549401785_Mycob 1 M-----RTYRTVRYPLGEALLALYRWRGPLINAGVG-GHGYTYLLGAEANRFVFANADAFSWSQTFESLV 64

CYP139A1_2555434304_Mycob 1 M-----RTYRTVRYPLGEALLALYRWRGPLINAGVG-GHGYTYLLGAEANRFVFANADAFSWSQTFESLV 64

CYP139A1_2555528546_Mycob 1 M-----RTYRTVRYPLGEALLALYRWRGPLINAGVG-GHGYTYLLGAEANRFVFANADAFSWSQTFESLV 64

CYP139A1_2555595572_Mycob 1 M-----RTYRTVRYPLGEALLALYRWRGPLINAGVG-GHGYTYLLGAEANRFVFANADAFSWSQTFESLV 64

CYP139A1_2555144317_Mycob 1 M-----RTYRTVRYPLGEALLALYRWRGPLINAGVG-GHGYTYLLGAEANRFVFANADAFSWSQTFESLV 64

CYP139A1_2555290904_Mycob 1 M-----RTYRTVRYPLGEALLALYRWRGPLINAGVG-GHGYTYLLGAEANRFVFANADAFSWSQTFESLV 64

CYP139A1_2555316223_Mycob 1 M-----RTYRTVRYPLGEALLALYRWRGPLINAGVG-GHGYTYLLGAEANRFVFANADAFSWSQTFESLV 64

CYP139A1_2555388140_Mycob 1 M-----RTYRTVRYPLGEALLALYRWRGPLINAGVG-GHGYTYLLGAEANRFVFANADAFSWSQTFESLV 64

CYP139A1_2555350295_Mycob 1 M-----RTYRTVRYPLGEALLALYRWRGPLINAGVG-GHGYTYLLGAEANRFVFANADAFSWSQTFESLV 64

CYP139A1_2555960457_Mycob 1 M-----RTYRTVRYPLGEALLALYRWRGPLINAGVG-GHGYTYLLGAEANRFVFANADAFSWSQTFESLV 64

CYP139A1_2547951333_Mycob 1 M-----RTYRTVRYPLGEALLALYRWRGPLINAGVG-GHGYTYLLGAEANRFVFANADAFSWSQTFESLV 64

CYP139A1_2555451004_Mycob 1 M-----RTYRTVRYPLGEALLALYRWRGPLINAGVG-GHGYTYLLGAEANRFVFANADAFSWSQTFESLV 64

CYP139A1_2555140094_Mycob 1 M-----RTYRTVRYPLGEALLALYRWRGPLINAGVG-GHGYTYLLGAEANRFVFANADAFSWSQTFESLV 64

CYP139A1_2555358731_Mycob 1 M-----RTYRTVRYPLGEALLALYRWRGPLINAGVG-GHGYTYLLGAEANRFVFANADAFSWSQTFESLV 64

CYP139A1_2555375511_Mycob 1 M-----RTYRTVRYPLGEALLALYRWRGPLINAGVG-GHGYTYLLGAEANRFVFANADAFSWSQTFESLV 64

CYP139A1_2555578867_Mycob 1 M-----RTYRTVRYPLGEALLALYRWRGPLINAGVG-GHGYTYLLGAEANRFVFANADAFSWSQTFESLV 64

CYP139A1_2554700949_Mycob 1 M-----RTYRTVRYPLGEALLALYRWRGPLINAGVG-GHGYTYLLGAEANRFVFANADAFSWSQTFESLV 64

CYP139A1_2575403392_Mycob 1 M-----RTYRTVRYPLGEALLALYRWRGPLINAGVG-GHGYTYLLGAEANRFVFANADAFSWSQTFESLV 64

CYP139A1_638726892_Mycoba 1 M-----RTYRTVRYPLGEALLALYRWRGPLINAGVG-GHGYTYLLGAEANRFVFANADAFSWSQTFESLV 64

CYP139A1_648443266_Mycoba 1 M-----RTYRTVRYPLGEALLALYRWRGPLINAGVG-GHGYTYLLGAEANRFVFANADAFSWSQTFESLV 64

CYP139A1_2540554561_Mycob 1 M-----R------YPLGEALLALYRWRGPLINAGVG-GHGYTYLLGAEANRFVFANAAAFSWRQTFESLV 58

CYP139A1_2566980890_Mycob 1 M-----RTYRTVRYPLGEALLALYRWRGPLINAGVG-GHGYTYLLGAEANRFVFANAAAFSWRQTFESLV 64

CYP139A1_2566976623_Mycob 1 M-----RTYRTVRYPLGEALLALYRWRGPLINAGVG-GHGYTYLLGAEANRFVFANAAAFSWRQTFESLV 64

CYP139A1_2566972350_Mycob 1 M-----RTYRTVRYPLGEALLALYRWRGPLINAGVG-GHGYTYLLGAEANRFVFANAAAFSWRQTFESLV 64

CYP139A1_2514118145_Mycob 1 M-----R------YPLGEALLALYRWRGPLINAGVGVGMATPISWEQRPTDSCSPNADAFSWSQTFESLV 59

[Consensus_aa:](http://prodata.swmed.edu/promals3d/info/consensus.html) **M**.....p......**Y**..**GEALLALYR**.**RGP***hl*s**AG***l***G**..**+GY***h***YLLGAEAN+FVFANADAFSW**.p**TFE**s**L***h*

[Consensus_ss:](http://prodata.swmed.edu/promals3d/info/consensus_ss.html) hhhhhhhhhhhh eeeeee - eeeeee hhhhhhhhh hh hhhh

Conservation: 996999999999 9999999 9 966696 96 966 9 69 96996 9 9 969966

CYP139A_2567124714_Mycoba 65 PVDGPTALIVSDGEDHRRRRSVVAPGLRHRQVQDYVQTMVSTIDEVINTWRPGQRLDLYQQFRSAVRRST 134

CYP139A_650873455_Mycobac 59 PVDGPTALIVSDGADHRRRRSLVQPAFHHRHLGNYLQVMAANADAVIDTWRPGDTIDIVAQLRSAIRRSA 128

CYP139A_2549393401_Mycoba 63 PVDGPTALIVSDGADHRRRRSLVQPAFHHRHIANYLQIMAANADAVIDSWRPGDTVDIFAQLRSAIRRSA 132

CYP139A_2548535921_Mycoba 62 LVDGPTALIVSDGDDHRRRRSVVAPGLRHRQIQDYVTTMVSCIDRVIDGWRPGQRLDVYQHCRAAVRRST 131

CYP139A_2576976958_Mycoba 59 LVDGPTALIVSDGDDHRRRRSVVVPGLRHRQIQDYVTTMVSCIDRVIHGWRPGQRLDVYQHCRAAVRRST 128

CYP139A_2581913245_Mycoba 59 LVDGPTALIVSDGDDHRRRRSVVAPGLRHRQIQDYVTTMVSCIDRVIDGWRPGQRLDVYQHCRAAVRRST 128

CYP139A_2580974538_Mycoba 59 LVDGPTALIVSDGDDHRRRRSVVVPGLRHRQIQDYVTTMVSCIDRVIHGWRPGQRLDVYQHCRAAVRRST 128

CYP139A_2582181025_Mycoba 59 LVDGPTALIVSDGDDHRRRRSVVAPGLRHRQIQDYVTTMVSCIDRVIDGWRPGQRLDVYQHCRAAVRRST 128

CYP139A_2580742569_Mycoba 59 LVDGPTALIVSDGDDHRRRRSVVVPGLRHRQIQDYVTTMVSCIDRVIHGWRPGQRLDVYQHCRAAVRRST 128

CYP139A_2582203743_Mycoba 59 LVDGPTALIVSDGDDHRRRRSVVVPGLRHRQIQDYVTTMVSCIDRVIHGWRPGQRLDVYQHCRAAVRRST 128

CYP139A_2567079276_Mycoba 41 LVDGPTALIVSDGDDHRRRRSVVAPGLRHRQIQDYVTTMVSCIDRVIDGWRPGQRLDVYQHCRAAVRRST 110

CYP139A_2543326887_Mycoba 59 LVDGPTALIVSDGDDHRRRRSVVAPGLRHRQIQDYVTTMVSCIDRVIDGWRPGQRLDVYQHCRAAVRRST 128

CYP139A_2570865822_Mycoba 59 LVDGPTALIVSDGDDHRRRRSVVAPGLRHRQIQDYVTTMVSCIDRVIDGWRPGQRLDVYQHCRAAVRRST 128

CYP139A_2592485489_Mycoba 59 LVDGPTALIVSDGDDHRRRRSVVAPGLRHRQIQDYVTTMVSCIDRVIDGWRPGQRLDVYQHCRAAVRRST 128

CYP139A_2550738610_Mycoba 62 LVDGPTALIVSDGDDHRRRRSVVAPGLRHRQIQDYVTTMVSCIDRVIDGWRPGQRLDVYQHCRAAVRRST 131

CYP139A_2580783837__Mycob 59 LVDGPTALIVSDGDDHRRRRSVVAPGLRHRQIQDYVTTMVSCIDRVIDGWRPGQRLDVYQHCRAAVRRST 128

CYP139A_2569618768_Mycoba 59 LVDGPTALIVSDGDDHRRRRSVVAPGLRHRQIQDYVTTMVSCIDRVIDGWRPGQRLDVYQHCRAAVRRST 128

CYP139A_2547368463_Mycoba 62 LVDGPTALIVSDGDDHRRRRSVVAPGLRHRQIQDYVTTMVSCIDRVIDGWRPGQRLDVYQHCRAAVRRST 131

CYP139A_2572767979_Mycoba 59 LVDGPTALIVSDGDDHRRRRSVVAPGLRHRQIQDYLTTMVSCIDRVIDGWRPGQRLDVYQHCRAAVRRST 128

CYP139A_2582391300_Mycoba 59 LVDGPTALIVSDGDDHRRRRSVVAPGLRHRQIQDYVTTMVSCIDRVIDGWRPGQRLDVYQHCRAAVRRST 128

CYP139A_2548515815_Mycoba 62 LVDGPTALIVSDGDDHRRRRSVVAPGLRHRQIQDYVTTMVSCIDRVIDGWRPGQRLDVYQHCRAAVRRST 131

CYP139A_2548530385_Mycoba 62 LVDGPTALIVSDGDDHRRRRSVVAPGLRHRQIQDYVTTMVSCIDRVIDGWRPGQRLDVYQHCRAAVRRST 131

CYP139A_2581397788_Mycoba 59 LVDGPTALIVSDGDDHRRRRSVVAPGLRHRQIQDYVTTMVSCIDRVIDGWRPGQRLDVYQHCRAAVRRST 128

CYP139A_2549377452_Mycoba 62 LVDGPTALIVSDGDDHRRRRSVVAPGLRHRQIQDYVTTMVSCIDRVIDGWRPGQRLDVYQHCRAAVRRST 131

CYP139A_2549389164_Mycoba 62 LVDGPTALIVSDGDDHRRRRSVVAPGLRHRQIQDYVTTMVSCIDRVIDGWRPGQRLDVYQHCRAAVRRST 131

CYP139A_645425415_Mycobac 59 LVDGPTALIVSDGDDHRRRRSVVAPGLRHRQIQDYVTTMVSCIDRVIDGWRPGQRLDVYQHCRAAVRRST 128

CYP139A_2581110378_Mycoba 62 LVDGPTALIVSDGDDHRRRRSVVAPGLRHRQIQDYVTTMVSCIDRVIDGWRPGQRLDVYQHCRAAVRRST 131

CYP139A_2573433552_Mycoba 59 LVDGPTALIVSDGDDHRRRRSVVAPGLRHRQIQDYVTTMVSCIDRVIDGWRPGQRLDVYQHCRAAVRRST 128

CYP139A_2580006443__Mycob 59 LVDGPTALIVSDGDDHRRRRSVVAPGLRHRQIQDYVTTMVSCIDRVIDGWRPGQRLDVYQHCRAAVRRST 128

CYP139A_637134331_Mycobac 59 LVDGPTALIVSDGDDHRRRRSVVAPGLRHRQIQDYVTTMVSCIDRVIDGWRPGQRLDVYQHCRAAVRRST 128

CYP139A_2548578292_Mycoba 62 LVDGPTALIVSDGDDHRRRRSVVAPGLRHRQIQDYVTTMVSCIDRVIDGWRPGQRLDVYQHCRAAVRRST 131

CYP139A_2548547272_Mycoba 62 LVDGPTALIVSDGDDHRRRRSVVAPGLRHRQIQDYVTTMVSCIDRVIDGWRPGQRLDVYQHCRAAVRRST 131

CYP139A_2549383420_Mycoba 62 LVDGPTALIVSDGDDHRRRRSVVAPGLRHRQIQDYVTTMVSCIDRVIDGWRPGQRLDVYQHCRAAVRRST 131

CYP139A_639736419_Mycobac 59 LVDGPTALIVSDGDDHRRRRSVVAPGLRHRQIQDYVTTMVSCIDRVIDGWRPGQRLDVYQHCRAAVRRST 128

CYP139A_2555735619_Mycoba 59 LVDGPTALIVSDGDDHRRRRSVVAPGLRHRQIQDYVTTMVSCIDRVIDGWRPGQRLDVYQHCRAAVRRST 128

CYP139A_2555481387_Mycoba 70 IVDGPTALIVSDGDDHRRRRSVVAPGLRHRQIQDYVQTMVANVDAVIDDWRPGQRLDIYRQCRSAVRRST 139

CYP139A_2545768030_Mycoba 64 WVDEPTALIVSDGDDHRRRRSVVAPGLRHRQIQDYVQTMVSNIDAAIDGWRPGQRLDIYRQCRSAVRRAT 133

CYP139A_2567131988_Mycoba 65 PVDGPTALIVSDGEDHRRRRSVVAPGLHHRRVQDYVQTMVSTIDAVIDAWRPGQRLDLYQEFRSAVRRST 134

CYP139A_2587480388_Mycoba 65 PVDGPTALIVSDGEDHRRRRSVVAPGLHHRRVQDYVQTMVSTIDAVIDAWRPGQRLDLYQEFRSAVRRST 134

CYP139A_2563577345_Mycoba 65 PVDGPTALIVSDGEDHRRRRSVVAPGLHHRRVQDYVQTMVSTIDAVIDAWRPGQRLDLYQEFRSAVRRST 134

CYP139A_2543277028_Mycoba 65 PVDGPTALIVSDGEDHRRRRSIVAPGLRHRSVQDYVATMVSTIDNVIDSWRPGQPLDIYQEFRCAVRRST 134

CYP139A_2563569217_Mycoba 65 PVDGPTALIVSDGEDHRRRRSIVAPGLRHRSVQDYVATMVSTIDNVIDSWRPGQPLDIYQEFRCAVRRST 134

CYP139A_641717750_Mycobac 65 PVDGPTALIVSDGEDHRRRRSIVAPGLRHRSVQDYVATMVSTIDNVIDSWRPGQPLDIYQEFRCAVRRST 134

CYP139A_2588629254_Mycoba 65 PVDGPTALIVSDGEDHRRRRSIVAPGLRHRSVQDYVATMVSTIDNVIDSWRPGQPLDIYQEFRCAVRRST 134

CYP139A_2546369014_Mycoba 65 PVDGPTALIVSDGEDHRRRRSIVAPGLRHRSVQDYVATMVSTIDNVIDSWRPGQPLDIYQEFRCAVRRST 134

CYP139A1_2555148489_Mycob 65 PVDGPTALIVSDGADHRRRRSVVAPGLRHHHVQRYVATMVSNIDTVIDGWQPGQRLDIYQELRSAVRRST 134

CYP139A1_646010237_Mycoba 19 PVDGPTALIVSDGADHRRRRSVVAPGLRHHHVQRYVATMVSNIDTVIDGWQPGQRLDIYQELRSAVRRST 88

CYP139A1_2581377024_Mycob 19 PVDGPTALIVSDGADHRRRRSVVAPGLRHHHVQRYVATMVSNIDTVIDGWQPGQRLDIYQELRSAVRRST 88

CYP139A1_647209603_Mycoba 19 PVDGPTALIVSDGADHRRRRSVVAPGLRHHHVQRYVATMVSNIDTVIDGWQPGQRLDIYQELRSAVRRST 88

CYP139A1_2537735281_Mycob 65 PVDGPTALIVSDGADHRRRRSVVAPGLRHHHVQRYVATMVSNIDTVIDGWQPGQRLDIYQELRSAVRRST 134

CYP139A1_2576388909_Mycob 59 PVDGPTALIVSDGADHRRRRSVVAPGLRHHHVQRYVATMVSNIDTVIDGWQPGQRLDIYQELRSAVRRST 128

CYP139A1_2577593438_Mycob 59 PVDGPTALIVSDGADHRRRRSVVAPGLRHHHVQRYVATMVSNIDTVIDGWQPGQRLDIYQELRSAVRRST 128

CYP139A1_2577803488_Mycob 59 PVDGPTALIVSDGADHRRRRSVVAPGLRHHHVQRYVATMVSNIDTVIDGWQPGQRLDIYQELRSAVRRST 128

CYP139A1_2581355094_Mycob 59 PVDGPTALIVSDGADHRRRRSVVAPGLRHHHVQRYVATMVSNIDTVIDGWQPGQRLDIYQELRSAVRRST 128

CYP139A1_2584983051_Mycob 59 PVDGPTALIVSDGADHRRRRSVVAPGLRHHHVQRYVATMVSNIDTVIDGWQPGQRLDIYQELRSAVRRST 128

CYP139A1_2584987406_Mycob 59 PVDGPTALIVSDGADHRRRRSVVAPGLRHHHVQRYVATMVSNIDTVIDGWQPGQRLDIYQELRSAVRRST 128

CYP139A1_2589032800_Mycob 59 PVDGPTALIVSDGADHRRRRSVVAPGLRHHHVQRYVATMVSNIDTVIDGWQPGQRLDIYQELRSAVRRST 128

CYP139A1_2592403099_Mycob 59 PVDGPTALIVSDGADHRRRRSVVAPGLRHHHVQRYVATMVSNIDTVIDGWQPGQRLDIYQELRSAVRRST 128

CYP139A1_2592422247_Mycob 59 PVDGPTALIVSDGADHRRRRSVVAPGLRHHHVQRYVATMVSNIDTVIDGWQPGQRLDIYQELRSAVRRST 128

CYP139A1_643734506_Mycoba 59 PVDGPTALIVSDGADHRRRRSVVAPGLRHHHVQRYVATMVSNIDTVIDGWQPGQRLDIYQELRSAVRRST 128

CYP139A1_648335985_Mycoba 59 PVDGPTALIVSDGADHRRRRSVVAPGLRHHHVQRYVATMVSNIDTVIDGWQPGQRLDIYQELRSAVRRST 128

CYP139A1_2574754194_Mycob 59 PVDGPTALIVSDGADHRRRRSVVAPGLRHHHVQRYVATMVSNIDTVIDGWQPGQRLDIYQELRSAVRRST 128

CYP139A1_2575447433_Mycob 59 PVDGPTALIVSDGADHRRRRSVVAPGLRHHHVQRYVATMVSNIDTVIDGWQPGQRLDIYQELRSAVRRST 128

CYP139A1_2575938969_Mycob 59 PVDGPTALIVSDGADHRRRRSVVAPGLRHHHVQRYVATMVSNIDTVIDGWQPGQRLDIYQELRSAVRRST 128

CYP139A1_2576477081_Mycob 59 PVDGPTALIVSDGADHRRRRSVVAPGLRHHHVQRYVATMVSNIDTVIDGWQPGQRLDIYQELRSAVRRST 128

CYP139A1_2576601719_Mycob 59 PVDGPTALIVSDGADHRRRRSVVAPGLRHHHVQRYVATMVSNIDTVIDGWQPGQRLDIYQELRSAVRRST 128

CYP139A1_2577098384_Mycob 59 PVDGPTALIVSDGADHRRRRSVVAPGLRHHHVQRYVATMVSNIDTVIDGWQPGQRLDIYQELRSAVRRST 128

CYP139A1_2578107196_Mycob 59 PVDGPTALIVSDGADHRRRRSVVAPGLRHHHVQRYVATMVSNIDTVIDGWQPGQRLDIYQELRSAVRRST 128

CYP139A1_2584883084_Mycob 59 PVDGPTALIVSDGADHRRRRSVVAPGLRHHHVQRYVATMVSNIDTVIDGWQPGQRLDIYQELRSAVRRST 128

CYP139A1_2588974834_Mycob 59 PVDGPTALIVSDGADHRRRRSVVAPGLRHHHVQRYVATMVSNIDTVIDGWQPGQRLDIYQELRSAVRRST 128

CYP139A1_2589056454_Mycob 59 PVDGPTALIVSDGADHRRRRSVVAPGLRHHHVQRYVATMVSNIDTVIDGWQPGQRLDIYQELRSAVRRST 128

CYP139A1_2589161189_Mycob 59 PVDGPTALIVSDGADHRRRRSVVAPGLRHHHVQRYVATMVSNIDTVIDGWQPGQRLDIYQELRSAVRRST 128

CYP139A1_2590374347_Mycob 59 PVDGPTALIVSDGADHRRRRSVVAPGLRHHHVQRYVATMVSNIDTVIDGWQPGQRLDIYQELRSAVRRST 128

CYP139A1_646018681_Mycoba 59 PVDGPTALIVSDGADHRRRRSVVAPGLRHHHVQRYVATMVSNIDTVIDGWQPGQRLDIYQELRSAVRRST 128

CYP139A1_2575060404_Mycob 59 PVDGPTALIVSDGADHRRRRSVVAPGLRHHHVQRYVATMVSNIDTVIDGWQPGQRLDIYQELRSAVRRST 128

CYP139A1_2576105631_Mycob 59 PVDGPTALIVSDGADHRRRRSVVAPGLRHHHVQRYVATMVSNIDTVIDGWQPGQRLDIYQELRSAVRRST 128

CYP139A1_2576247251_Mycob 59 PVDGPTALIVSDGADHRRRRSVVAPGLRHHHVQRYVATMVSNIDTVIDGWQPGQRLDIYQELRSAVRRST 128

CYP139A1_2576981010_Mycob 59 PVDGPTALIVSDGADHRRRRSVVAPGLRHHHVQRYVATMVSNIDTVIDGWQPGQRLDIYQELRSAVRRST 128

CYP139A1_2577093117_Mycob 59 PVDGPTALIVSDGADHRRRRSVVAPGLRHHHVQRYVATMVSNIDTVIDGWQPGQRLDIYQELRSAVRRST 128

CYP139A1_2577198903_Mycob 59 PVDGPTALIVSDGADHRRRRSVVAPGLRHHHVQRYVATMVSNIDTVIDGWQPGQRLDIYQELRSAVRRST 128

CYP139A1_2577516047_Mycob 59 PVDGPTALIVSDGADHRRRRSVVAPGLRHHHVQRYVATMVSNIDTVIDGWQPGQRLDIYQELRSAVRRST 128

CYP139A1_2578213104_Mycob 59 PVDGPTALIVSDGADHRRRRSVVAPGLRHHHVQRYVATMVSNIDTVIDGWQPGQRLDIYQELRSAVRRST 128

CYP139A1_2584711251_Mycob 59 PVDGPTALIVSDGADHRRRRSVVAPGLRHHHVQRYVATMVSNIDTVIDGWQPGQRLDIYQELRSAVRRST 128

CYP139A1_2584816678_Mycob 59 PVDGPTALIVSDGADHRRRRSVVAPGLRHHHVQRYVATMVSNIDTVIDGWQPGQRLDIYQELRSAVRRST 128

CYP139A1_2589068752_Mycob 59 PVDGPTALIVSDGADHRRRRSVVAPGLRHHHVQRYVATMVSNIDTVIDGWQPGQRLDIYQELRSAVRRST 128

CYP139A1_2589604293_Mycob 59 PVDGPTALIVSDGADHRRRRSVVAPGLRHHHVQRYVATMVSNIDTVIDGWQPGQRLDIYQELRSAVRRST 128

CYP139A1_643028176_Mycoba 59 PVDGPTALIVSDGADHRRRRSVVAPGLRHHHVQRYVATMVSNIDTVIDGWQPGQRLDIYQELRSAVRRST 128

CYP139A1_648476944_Mycoba 59 PVDGPTALIVSDGADHRRRRSVVAPGLRHHHVQRYVATMVSNIDTVIDGWQPGQRLDIYQELRSAVRRST 128

CYP139A1_2511736071_Mycob 59 PVDGPTALIVSDGADHRRRRSVVAPGLRHHHVQRYVATMVSNIDTVIDGWQPGQRLDIYQELRSAVRRST 128

CYP139A1_2546206123_Mycob 59 PVDGPTALIVSDGADHRRRRSVVAPGLRHHHVQRYVATMVSNIDTVIDGWQPGQRLDIYQELRSAVRRST 128

CYP139A1_2574780327_Mycob 59 PVDGPTALIVSDGADHRRRRSVVAPGLRHHHVQRYVATMVSNIDTVIDGWQPGQRLDIYQELRSAVRRST 128

CYP139A1_2575978404_Mycob 59 PVDGPTALIVSDGADHRRRRSVVAPGLRHHHVQRYVATMVSNIDTVIDGWQPGQRLDIYQELRSAVRRST 128

CYP139A1_2576675825_Mycob 59 PVDGPTALIVSDGADHRRRRSVVAPGLRHHHVQRYVATMVSNIDTVIDGWQPGQRLDIYQELRSAVRRST 128

CYP139A1_2576947708_Mycob 59 PVDGPTALIVSDGADHRRRRSVVAPGLRHHHVQRYVATMVSNIDTVIDGWQPGQRLDIYQELRSAVRRST 128

CYP139A1_2577400922_Mycob 59 PVDGPTALIVSDGADHRRRRSVVAPGLRHHHVQRYVATMVSNIDTVIDGWQPGQRLDIYQELRSAVRRST 128

CYP139A1_2577893113_Mycob 59 PVDGPTALIVSDGADHRRRRSVVAPGLRHHHVQRYVATMVSNIDTVIDGWQPGQRLDIYQELRSAVRRST 128

CYP139A1_2584759228_Mycob 59 PVDGPTALIVSDGADHRRRRSVVAPGLRHHHVQRYVATMVSNIDTVIDGWQPGQRLDIYQELRSAVRRST 128

CYP139A1_2584801008_Mycob 59 PVDGPTALIVSDGADHRRRRSVVAPGLRHHHVQRYVATMVSNIDTVIDGWQPGQRLDIYQELRSAVRRST 128

CYP139A1_2584946269_Mycob 59 PVDGPTALIVSDGADHRRRRSVVAPGLRHHHVQRYVATMVSNIDTVIDGWQPGQRLDIYQELRSAVRRST 128

CYP139A1_2589125802_Mycob 59 PVDGPTALIVSDGADHRRRRSVVAPGLRHHHVQRYVATMVSNIDTVIDGWQPGQRLDIYQELRSAVRRST 128

CYP139A1_2589654545_Mycob 59 PVDGPTALIVSDGADHRRRRSVVAPGLRHHHVQRYVATMVSNIDTVIDGWQPGQRLDIYQELRSAVRRST 128

CYP139A1_2590190898_Mycob 59 PVDGPTALIVSDGADHRRRRSVVAPGLRHHHVQRYVATMVSNIDTVIDGWQPGQRLDIYQELRSAVRRST 128

CYP139A1_2592267285_Mycob 59 PVDGPTALIVSDGADHRRRRSVVAPGLRHHHVQRYVATMVSNIDTVIDGWQPGQRLDIYQELRSAVRRST 128

CYP139A1_2592579018_Mycob 59 PVDGPTALIVSDGADHRRRRSVVAPGLRHHHVQRYVATMVSNIDTVIDGWQPGQRLDIYQELRSAVRRST 128

CYP139A1_2574803240_Mycob 59 PVDGPTALIVSDGADHRRRRSVVAPGLRHHHVQRYVATMVSNIDTVIDGWQPGQRLDIYQELRSAVRRST 128

CYP139A1_2575138339_Mycob 59 PVDGPTALIVSDGADHRRRRSVVAPGLRHHHVQRYVATMVSNIDTVIDGWQPGQRLDIYQELRSAVRRST 128

CYP139A1_2575935659_Mycob 59 PVDGPTALIVSDGADHRRRRSVVAPGLRHHHVQRYVATMVSNIDTVIDGWQPGQRLDIYQELRSAVRRST 128

CYP139A1_2576703024_Mycob 59 PVDGPTALIVSDGADHRRRRSVVAPGLRHHHVQRYVATMVSNIDTVIDGWQPGQRLDIYQELRSAVRRST 128

CYP139A1_2577143911_Mycob 59 PVDGPTALIVSDGADHRRRRSVVAPGLRHHHVQRYVATMVSNIDTVIDGWQPGQRLDIYQELRSAVRRST 128

CYP139A1_2577175183_Mycob 59 PVDGPTALIVSDGADHRRRRSVVAPGLRHHHVQRYVATMVSNIDTVIDGWQPGQRLDIYQELRSAVRRST 128

CYP139A1_2577879644_Mycob 59 PVDGPTALIVSDGADHRRRRSVVAPGLRHHHVQRYVATMVSNIDTVIDGWQPGQRLDIYQELRSAVRRST 128

CYP139A1_2577954418_Mycob 59 PVDGPTALIVSDGADHRRRRSVVAPGLRHHHVQRYVATMVSNIDTVIDGWQPGQRLDIYQELRSAVRRST 128

CYP139A1_2584625495_Mycob 59 PVDGPTALIVSDGADHRRRRSVVAPGLRHHHVQRYVATMVSNIDTVIDGWQPGQRLDIYQELRSAVRRST 128

CYP139A1_2589526877_Mycob 59 PVDGPTALIVSDGADHRRRRSVVAPGLRHHHVQRYVATMVSNIDTVIDGWQPGQRLDIYQELRSAVRRST 128

CYP139A1_2589711853_Mycob 59 PVDGPTALIVSDGADHRRRRSVVAPGLRHHHVQRYVATMVSNIDTVIDGWQPGQRLDIYQELRSAVRRST 128

CYP139A1_2590113796_Mycob 59 PVDGPTALIVSDGADHRRRRSVVAPGLRHHHVQRYVATMVSNIDTVIDGWQPGQRLDIYQELRSAVRRST 128

CYP139A1_2592324422_Mycob 59 PVDGPTALIVSDGADHRRRRSVVAPGLRHHHVQRYVATMVSNIDTVIDGWQPGQRLDIYQELRSAVRRST 128

CYP139A1_2592337997_Mycob 59 PVDGPTALIVSDGADHRRRRSVVAPGLRHHHVQRYVATMVSNIDTVIDGWQPGQRLDIYQELRSAVRRST 128

CYP139A1_2592373455_Mycob 59 PVDGPTALIVSDGADHRRRRSVVAPGLRHHHVQRYVATMVSNIDTVIDGWQPGQRLDIYQELRSAVRRST 128

CYP139A1_2592445915_Mycob 59 PVDGPTALIVSDGADHRRRRSVVAPGLRHHHVQRYVATMVSNIDTVIDGWQPGQRLDIYQELRSAVRRST 128

CYP139A1_2575023271_Mycob 59 PVDGPTALIVSDGADHRRRRSVVAPGLRHHHVQRYVATMVSNIDTVIDGWQPGQRLDIYQELRSAVRRST 128

CYP139A1_2575619239_Mycob 59 PVDGPTALIVSDGADHRRRRSVVAPGLRHHHVQRYVATMVSNIDTVIDGWQPGQRLDIYQELRSAVRRST 128

CYP139A1_2575786887_Mycob 59 PVDGPTALIVSDGADHRRRRSVVAPGLRHHHVQRYVATMVSNIDTVIDGWQPGQRLDIYQELRSAVRRST 128

CYP139A1_2576882264_Mycob 59 PVDGPTALIVSDGADHRRRRSVVAPGLRHHHVQRYVATMVSNIDTVIDGWQPGQRLDIYQELRSAVRRST 128

CYP139A1_2577215885_Mycob 59 PVDGPTALIVSDGADHRRRRSVVAPGLRHHHVQRYVATMVSNIDTVIDGWQPGQRLDIYQELRSAVRRST 128

CYP139A1_2577627248_Mycob 59 PVDGPTALIVSDGADHRRRRSVVAPGLRHHHVQRYVATMVSNIDTVIDGWQPGQRLDIYQELRSAVRRST 128

CYP139A1_2577923998_Mycob 59 PVDGPTALIVSDGADHRRRRSVVAPGLRHHHVQRYVATMVSNIDTVIDGWQPGQRLDIYQELRSAVRRST 128

CYP139A1_2583735989_Mycob 59 PVDGPTALIVSDGADHRRRRSVVAPGLRHHHVQRYVATMVSNIDTVIDGWQPGQRLDIYQELRSAVRRST 128

CYP139A1_2584003651_Mycob 59 PVDGPTALIVSDGADHRRRRSVVAPGLRHHHVQRYVATMVSNIDTVIDGWQPGQRLDIYQELRSAVRRST 128

CYP139A1_2584623655_Mycob 59 PVDGPTALIVSDGADHRRRRSVVAPGLRHHHVQRYVATMVSNIDTVIDGWQPGQRLDIYQELRSAVRRST 128

CYP139A1_2584739857_Mycob 59 PVDGPTALIVSDGADHRRRRSVVAPGLRHHHVQRYVATMVSNIDTVIDGWQPGQRLDIYQELRSAVRRST 128

CYP139A1_2584858071_Mycob 59 PVDGPTALIVSDGADHRRRRSVVAPGLRHHHVQRYVATMVSNIDTVIDGWQPGQRLDIYQELRSAVRRST 128

CYP139A1_2584928246_Mycob 59 PVDGPTALIVSDGADHRRRRSVVAPGLRHHHVQRYVATMVSNIDTVIDGWQPGQRLDIYQELRSAVRRST 128

CYP139A1_2589040293_Mycob 59 PVDGPTALIVSDGADHRRRRSVVAPGLRHHHVQRYVATMVSNIDTVIDGWQPGQRLDIYQELRSAVRRST 128

CYP139A1_2589658610_Mycob 59 PVDGPTALIVSDGADHRRRRSVVAPGLRHHHVQRYVATMVSNIDTVIDGWQPGQRLDIYQELRSAVRRST 128

CYP139A1_2590162679_Mycob 59 PVDGPTALIVSDGADHRRRRSVVAPGLRHHHVQRYVATMVSNIDTVIDGWQPGQRLDIYQELRSAVRRST 128

CYP139A1_2590243884_Mycob 59 PVDGPTALIVSDGADHRRRRSVVAPGLRHHHVQRYVATMVSNIDTVIDGWQPGQRLDIYQELRSAVRRST 128

CYP139A1_2590531681_Mycob 59 PVDGPTALIVSDGADHRRRRSVVAPGLRHHHVQRYVATMVSNIDTVIDGWQPGQRLDIYQELRSAVRRST 128

CYP139A1_2574614619_Mycob 59 PVDGPTALIVSDGADHRRRRSVVAPGLRHHHVQRYVATMVSNIDTVIDGWQPGQRLDIYQELRSAVRRST 128

CYP139A1_2575016195_Mycob 59 PVDGPTALIVSDGADHRRRRSVVAPGLRHHHVQRYVATMVSNIDTVIDGWQPGQRLDIYQELRSAVRRST 128

CYP139A1_2575426848_Mycob 59 PVDGPTALIVSDGADHRRRRSVVAPGLRHHHVQRYVATMVSNIDTVIDGWQPGQRLDIYQELRSAVRRST 128

CYP139A1_2576630848_Mycob 59 PVDGPTALIVSDGADHRRRRSVVAPGLRHHHVQRYVATMVSNIDTVIDGWQPGQRLDIYQELRSAVRRST 128

CYP139A1_2577468911_Mycob 59 PVDGPTALIVSDGADHRRRRSVVAPGLRHHHVQRYVATMVSNIDTVIDGWQPGQRLDIYQELRSAVRRST 128

CYP139A1_2577974906_Mycob 59 PVDGPTALIVSDGADHRRRRSVVAPGLRHHHVQRYVATMVSNIDTVIDGWQPGQRLDIYQELRSAVRRST 128

CYP139A1_2578062326_Mycob 59 PVDGPTALIVSDGADHRRRRSVVAPGLRHHHVQRYVATMVSNIDTVIDGWQPGQRLDIYQELRSAVRRST 128

CYP139A1_2584836956_Mycob 59 PVDGPTALIVSDGADHRRRRSVVAPGLRHHHVQRYVATMVSNIDTVIDGWQPGQRLDIYQELRSAVRRST 128

CYP139A1_2584998642_Mycob 59 PVDGPTALIVSDGADHRRRRSVVAPGLRHHHVQRYVATMVSNIDTVIDGWQPGQRLDIYQELRSAVRRST 128

CYP139A1_2589130082_Mycob 59 PVDGPTALIVSDGADHRRRRSVVAPGLRHHHVQRYVATMVSNIDTVIDGWQPGQRLDIYQELRSAVRRST 128

CYP139A1_2589592089_Mycob 59 PVDGPTALIVSDGADHRRRRSVVAPGLRHHHVQRYVATMVSNIDTVIDGWQPGQRLDIYQELRSAVRRST 128

CYP139A1_2590052832_Mycob 59 PVDGPTALIVSDGADHRRRRSVVAPGLRHHHVQRYVATMVSNIDTVIDGWQPGQRLDIYQELRSAVRRST 128

CYP139A1_2590505688_Mycob 59 PVDGPTALIVSDGADHRRRRSVVAPGLRHHHVQRYVATMVSNIDTVIDGWQPGQRLDIYQELRSAVRRST 128

CYP139A1_2592353030_Mycob 59 PVDGPTALIVSDGADHRRRRSVVAPGLRHHHVQRYVATMVSNIDTVIDGWQPGQRLDIYQELRSAVRRST 128

CYP139A1_637026884_Mycoba 59 PVDGPTALIVSDGADHRRRRSVVAPGLRHHHVQRYVATMVSNIDTVIDGWQPGQRLDIYQELRSAVRRST 128

CYP139A1_2574886309_Mycob 59 PVDGPTALIVSDGADHRRRRSVVAPGLRHHHVQRYVATMVSNIDTVIDGWQPGQRLDIYQELRSAVRRST 128

CYP139A1_2575295342_Mycob 59 PVDGPTALIVSDGADHRRRRSVVAPGLRHHHVQRYVATMVSNIDTVIDGWQPGQRLDIYQELRSAVRRST 128

CYP139A1_2575942274_Mycob 59 PVDGPTALIVSDGADHRRRRSVVAPGLRHHHVQRYVATMVSNIDTVIDGWQPGQRLDIYQELRSAVRRST 128

CYP139A1_2576123248_Mycob 59 PVDGPTALIVSDGADHRRRRSVVAPGLRHHHVQRYVATMVSNIDTVIDGWQPGQRLDIYQELRSAVRRST 128

CYP139A1_2576712596_Mycob 59 PVDGPTALIVSDGADHRRRRSVVAPGLRHHHVQRYVATMVSNIDTVIDGWQPGQRLDIYQELRSAVRRST 128

CYP139A1_2577856904_Mycob 59 PVDGPTALIVSDGADHRRRRSVVAPGLRHHHVQRYVATMVSNIDTVIDGWQPGQRLDIYQELRSAVRRST 128

CYP139A1_2578013153_Mycob 59 PVDGPTALIVSDGADHRRRRSVVAPGLRHHHVQRYVATMVSNIDTVIDGWQPGQRLDIYQELRSAVRRST 128

CYP139A1_2578182623_Mycob 59 PVDGPTALIVSDGADHRRRRSVVAPGLRHHHVQRYVATMVSNIDTVIDGWQPGQRLDIYQELRSAVRRST 128

CYP139A1_2580771058_Mycob 59 PVDGPTALIVSDGADHRRRRSVVAPGLRHHHVQRYVATMVSNIDTVIDGWQPGQRLDIYQELRSAVRRST 128

CYP139A1_2584641128_Mycob 59 PVDGPTALIVSDGADHRRRRSVVAPGLRHHHVQRYVATMVSNIDTVIDGWQPGQRLDIYQELRSAVRRST 128

CYP139A1_2584649146_Mycob 59 PVDGPTALIVSDGADHRRRRSVVAPGLRHHHVQRYVATMVSNIDTVIDGWQPGQRLDIYQELRSAVRRST 128

CYP139A1_2584660713_Mycob 59 PVDGPTALIVSDGADHRRRRSVVAPGLRHHHVQRYVATMVSNIDTVIDGWQPGQRLDIYQELRSAVRRST 128

CYP139A1_2584967006_Mycob 59 PVDGPTALIVSDGADHRRRRSVVAPGLRHHHVQRYVATMVSNIDTVIDGWQPGQRLDIYQELRSAVRRST 128

CYP139A1_2584970624_Mycob 59 PVDGPTALIVSDGADHRRRRSVVAPGLRHHHVQRYVATMVSNIDTVIDGWQPGQRLDIYQELRSAVRRST 128

CYP139A1_2589053585_Mycob 59 PVDGPTALIVSDGADHRRRRSVVAPGLRHHHVQRYVATMVSNIDTVIDGWQPGQRLDIYQELRSAVRRST 128

CYP139A1_2589498327_Mycob 59 PVDGPTALIVSDGADHRRRRSVVAPGLRHHHVQRYVATMVSNIDTVIDGWQPGQRLDIYQELRSAVRRST 128

CYP139A1_2589563529_Mycob 59 PVDGPTALIVSDGADHRRRRSVVAPGLRHHHVQRYVATMVSNIDTVIDGWQPGQRLDIYQELRSAVRRST 128

CYP139A1_2590377218_Mycob 59 PVDGPTALIVSDGADHRRRRSVVAPGLRHHHVQRYVATMVSNIDTVIDGWQPGQRLDIYQELRSAVRRST 128

CYP139A1_2592283614_Mycob 59 PVDGPTALIVSDGADHRRRRSVVAPGLRHHHVQRYVATMVSNIDTVIDGWQPGQRLDIYQELRSAVRRST 128

CYP139A1_2592319784_Mycob 59 PVDGPTALIVSDGADHRRRRSVVAPGLRHHHVQRYVATMVSNIDTVIDGWQPGQRLDIYQELRSAVRRST 128

CYP139A1_2575106637_Mycob 59 PVDGPTALIVSDGADHRRRRSVVAPGLRHHHVQRYVATMVSNIDTVIDGWQPGQRLDIYQELRSAVRRST 128

CYP139A1_2575157076_Mycob 59 PVDGPTALIVSDGADHRRRRSVVAPGLRHHHVQRYVATMVSNIDTVIDGWQPGQRLDIYQELRSAVRRST 128

CYP139A1_2575361778_Mycob 59 PVDGPTALIVSDGADHRRRRSVVAPGLRHHHVQRYVATMVSNIDTVIDGWQPGQRLDIYQELRSAVRRST 128

CYP139A1_2576009184_Mycob 59 PVDGPTALIVSDGADHRRRRSVVAPGLRHHHVQRYVATMVSNIDTVIDGWQPGQRLDIYQELRSAVRRST 128

CYP139A1_2576566954_Mycob 59 PVDGPTALIVSDGADHRRRRSVVAPGLRHHHVQRYVATMVSNIDTVIDGWQPGQRLDIYQELRSAVRRST 128

CYP139A1_2576731741_Mycob 59 PVDGPTALIVSDGADHRRRRSVVAPGLRHHHVQRYVATMVSNIDTVIDGWQPGQRLDIYQELRSAVRRST 128

CYP139A1_2581562358_Mycob 59 PVDGPTALIVSDGADHRRRRSVVAPGLRHHHVQRYVATMVSNIDTVIDGWQPGQRLDIYQELRSAVRRST 128

CYP139A1_2584107430_Mycob 59 PVDGPTALIVSDGADHRRRRSVVAPGLRHHHVQRYVATMVSNIDTVIDGWQPGQRLDIYQELRSAVRRST 128

CYP139A1_2590025444_Mycob 59 PVDGPTALIVSDGADHRRRRSVVAPGLRHHHVQRYVATMVSNIDTVIDGWQPGQRLDIYQELRSAVRRST 128

CYP139A1_2590040558_Mycob 59 PVDGPTALIVSDGADHRRRRSVVAPGLRHHHVQRYVATMVSNIDTVIDGWQPGQRLDIYQELRSAVRRST 128

CYP139A1_2590214646_Mycob 59 PVDGPTALIVSDGADHRRRRSVVAPGLRHHHVQRYVATMVSNIDTVIDGWQPGQRLDIYQELRSAVRRST 128

CYP139A1_2590223019_Mycob 59 PVDGPTALIVSDGADHRRRRSVVAPGLRHHHVQRYVATMVSNIDTVIDGWQPGQRLDIYQELRSAVRRST 128

CYP139A1_2590266966_Mycob 59 PVDGPTALIVSDGADHRRRRSVVAPGLRHHHVQRYVATMVSNIDTVIDGWQPGQRLDIYQELRSAVRRST 128

CYP139A1_647086307_Mycoba 59 PVDGPTALIVSDGADHRRRRSVVAPGLRHHHVQRYVATMVSNIDTVIDGWQPGQRLDIYQELRSAVRRST 128

CYP139A1_2574726119_Mycob 59 PVDGPTALIVSDGADHRRRRSVVAPGLRHHHVQRYVATMVSNIDTVIDGWQPGQRLDIYQELRSAVRRST 128

CYP139A1_2574757270_Mycob 59 PVDGPTALIVSDGADHRRRRSVVAPGLRHHHVQRYVATMVSNIDTVIDGWQPGQRLDIYQELRSAVRRST 128

CYP139A1_2575280304_Mycob 59 PVDGPTALIVSDGADHRRRRSVVAPGLRHHHVQRYVATMVSNIDTVIDGWQPGQRLDIYQELRSAVRRST 128

CYP139A1_2575601683_Mycob 59 PVDGPTALIVSDGADHRRRRSVVAPGLRHHHVQRYVATMVSNIDTVIDGWQPGQRLDIYQELRSAVRRST 128

CYP139A1_2576158036_Mycob 59 PVDGPTALIVSDGADHRRRRSVVAPGLRHHHVQRYVATMVSNIDTVIDGWQPGQRLDIYQELRSAVRRST 128

CYP139A1_2577689111_Mycob 59 PVDGPTALIVSDGADHRRRRSVVAPGLRHHHVQRYVATMVSNIDTVIDGWQPGQRLDIYQELRSAVRRST 128

CYP139A1_2577751179_Mycob 59 PVDGPTALIVSDGADHRRRRSVVAPGLRHHHVQRYVATMVSNIDTVIDGWQPGQRLDIYQELRSAVRRST 128

CYP139A1_2577845812_Mycob 59 PVDGPTALIVSDGADHRRRRSVVAPGLRHHHVQRYVATMVSNIDTVIDGWQPGQRLDIYQELRSAVRRST 128

CYP139A1_2577900964_Mycob 59 PVDGPTALIVSDGADHRRRRSVVAPGLRHHHVQRYVATMVSNIDTVIDGWQPGQRLDIYQELRSAVRRST 128

CYP139A1_2577988240_Mycob 59 PVDGPTALIVSDGADHRRRRSVVAPGLRHHHVQRYVATMVSNIDTVIDGWQPGQRLDIYQELRSAVRRST 128

CYP139A1_2578237814_Mycob 59 PVDGPTALIVSDGADHRRRRSVVAPGLRHHHVQRYVATMVSNIDTVIDGWQPGQRLDIYQELRSAVRRST 128

CYP139A1_2584703088_Mycob 59 PVDGPTALIVSDGADHRRRRSVVAPGLRHHHVQRYVATMVSNIDTVIDGWQPGQRLDIYQELRSAVRRST 128

CYP139A1_2584776403_Mycob 59 PVDGPTALIVSDGADHRRRRSVVAPGLRHHHVQRYVATMVSNIDTVIDGWQPGQRLDIYQELRSAVRRST 128

CYP139A1_2584785697_Mycob 59 PVDGPTALIVSDGADHRRRRSVVAPGLRHHHVQRYVATMVSNIDTVIDGWQPGQRLDIYQELRSAVRRST 128

CYP139A1_2584898862_Mycob 59 PVDGPTALIVSDGADHRRRRSVVAPGLRHHHVQRYVATMVSNIDTVIDGWQPGQRLDIYQELRSAVRRST 128

CYP139A1_2584906709_Mycob 59 PVDGPTALIVSDGADHRRRRSVVAPGLRHHHVQRYVATMVSNIDTVIDGWQPGQRLDIYQELRSAVRRST 128

CYP139A1_2589082097_Mycob 59 PVDGPTALIVSDGADHRRRRSVVAPGLRHHHVQRYVATMVSNIDTVIDGWQPGQRLDIYQELRSAVRRST 128

CYP139A1_2589142105_Mycob 59 PVDGPTALIVSDGADHRRRRSVVAPGLRHHHVQRYVATMVSNIDTVIDGWQPGQRLDIYQELRSAVRRST 128

CYP139A1_2589707360_Mycob 59 PVDGPTALIVSDGADHRRRRSVVAPGLRHHHVQRYVATMVSNIDTVIDGWQPGQRLDIYQELRSAVRRST 128

CYP139A1_2590142346_Mycob 59 PVDGPTALIVSDGADHRRRRSVVAPGLRHHHVQRYVATMVSNIDTVIDGWQPGQRLDIYQELRSAVRRST 128

CYP139A1_2592254166_Mycob 59 PVDGPTALIVSDGADHRRRRSVVAPGLRHHHVQRYVATMVSNIDTVIDGWQPGQRLDIYQELRSAVRRST 128

CYP139A1_2592348241_Mycob 59 PVDGPTALIVSDGADHRRRRSVVAPGLRHHHVQRYVATMVSNIDTVIDGWQPGQRLDIYQELRSAVRRST 128

CYP139A1_2592364277_Mycob 59 PVDGPTALIVSDGADHRRRRSVVAPGLRHHHVQRYVATMVSNIDTVIDGWQPGQRLDIYQELRSAVRRST 128

CYP139A1_2592537861_Mycob 59 PVDGPTALIVSDGADHRRRRSVVAPGLRHHHVQRYVATMVSNIDTVIDGWQPGQRLDIYQELRSAVRRST 128

CYP139A1_2546454904_Mycob 59 PVDGPTALIVSDGADHRRRRSVVAPGLRHHHVQRYVATMVSNIDTVIDGWQPGQRLDIYQELRSAVRRST 128

CYP139A1_2577218717_Mycob 59 PVDGPTALIVSDGADHRRRRSVVAPGLRHHHVQRYVATMVSNIDTVIDGWQPGQRLDIYQELRSAVRRST 128

CYP139A1_2577720788_Mycob 59 PVDGPTALIVSDGADHRRRRSVVAPGLRHHHVQRYVATMVSNIDTVIDGWQPGQRLDIYQELRSAVRRST 128

CYP139A1_2581366557_Mycob 59 PVDGPTALIVSDGADHRRRRSVVAPGLRHHHVQRYVATMVSNIDTVIDGWQPGQRLDIYQELRSAVRRST 128

CYP139A1_2584638962_Mycob 59 PVDGPTALIVSDGADHRRRRSVVAPGLRHHHVQRYVATMVSNIDTVIDGWQPGQRLDIYQELRSAVRRST 128

CYP139A1_2590154524_Mycob 59 PVDGPTALIVSDGADHRRRRSVVAPGLRHHHVQRYVATMVSNIDTVIDGWQPGQRLDIYQELRSAVRRST 128

CYP139A1_2590260165_Mycob 59 PVDGPTALIVSDGADHRRRRSVVAPGLRHHHVQRYVATMVSNIDTVIDGWQPGQRLDIYQELRSAVRRST 128

CYP139A1_2590539845_Mycob 59 PVDGPTALIVSDGADHRRRRSVVAPGLRHHHVQRYVATMVSNIDTVIDGWQPGQRLDIYQELRSAVRRST 128

CYP139A1_641783198_Mycoba 59 PVDGPTALIVSDGADHRRRRSVVAPGLRHHHVQRYVATMVSNIDTVIDGWQPGQRLDIYQELRSAVRRST 128

CYP139A1_643031783_Mycoba 59 PVDGPTALIVSDGADHRRRRSVVAPGLRHHHVQRYVATMVSNIDTVIDGWQPGQRLDIYQELRSAVRRST 128

CYP139A1_651039004_Mycoba 59 PVDGPTALIVSDGADHRRRRSVVAPGLRHHHVQRYVATMVSNIDTVIDGWQPGQRLDIYQELRSAVRRST 128

CYP139A1_2574843285_Mycob 59 PVDGPTALIVSDGADHRRRRSVVAPGLRHHHVQRYVATMVSNIDTVIDGWQPGQRLDIYQELRSAVRRST 128

CYP139A1_2575542837_Mycob 59 PVDGPTALIVSDGADHRRRRSVVAPGLRHHHVQRYVATMVSNIDTVIDGWQPGQRLDIYQELRSAVRRST 128

CYP139A1_2575709449_Mycob 59 PVDGPTALIVSDGADHRRRRSVVAPGLRHHHVQRYVATMVSNIDTVIDGWQPGQRLDIYQELRSAVRRST 128

CYP139A1_2576051820_Mycob 59 PVDGPTALIVSDGADHRRRRSVVAPGLRHHHVQRYVATMVSNIDTVIDGWQPGQRLDIYQELRSAVRRST 128

CYP139A1_2578094092_Mycob 59 PVDGPTALIVSDGADHRRRRSVVAPGLRHHHVQRYVATMVSNIDTVIDGWQPGQRLDIYQELRSAVRRST 128

CYP139A1_2584617586_Mycob 59 PVDGPTALIVSDGADHRRRRSVVAPGLRHHHVQRYVATMVSNIDTVIDGWQPGQRLDIYQELRSAVRRST 128

CYP139A1_2584694835_Mycob 59 PVDGPTALIVSDGADHRRRRSVVAPGLRHHHVQRYVATMVSNIDTVIDGWQPGQRLDIYQELRSAVRRST 128

CYP139A1_2584822720_Mycob 59 PVDGPTALIVSDGADHRRRRSVVAPGLRHHHVQRYVATMVSNIDTVIDGWQPGQRLDIYQELRSAVRRST 128

CYP139A1_2584931720_Mycob 59 PVDGPTALIVSDGADHRRRRSVVAPGLRHHHVQRYVATMVSNIDTVIDGWQPGQRLDIYQELRSAVRRST 128

CYP139A1_2589165303_Mycob 59 PVDGPTALIVSDGADHRRRRSVVAPGLRHHHVQRYVATMVSNIDTVIDGWQPGQRLDIYQELRSAVRRST 128

CYP139A1_2589608377_Mycob 59 PVDGPTALIVSDGADHRRRRSVVAPGLRHHHVQRYVATMVSNIDTVIDGWQPGQRLDIYQELRSAVRRST 128

CYP139A1_2589638476_Mycob 59 PVDGPTALIVSDGADHRRRRSVVAPGLRHHHVQRYVATMVSNIDTVIDGWQPGQRLDIYQELRSAVRRST 128

CYP139A1_2589687166_Mycob 59 PVDGPTALIVSDGADHRRRRSVVAPGLRHHHVQRYVATMVSNIDTVIDGWQPGQRLDIYQELRSAVRRST 128

CYP139A1_2590174897_Mycob 59 PVDGPTALIVSDGADHRRRRSVVAPGLRHHHVQRYVATMVSNIDTVIDGWQPGQRLDIYQELRSAVRRST 128

CYP139A1_2590370532_Mycob 59 PVDGPTALIVSDGADHRRRRSVVAPGLRHHHVQRYVATMVSNIDTVIDGWQPGQRLDIYQELRSAVRRST 128

CYP139A1_2592328029_Mycob 59 PVDGPTALIVSDGADHRRRRSVVAPGLRHHHVQRYVATMVSNIDTVIDGWQPGQRLDIYQELRSAVRRST 128

CYP139A1_2592426321_Mycob 59 PVDGPTALIVSDGADHRRRRSVVAPGLRHHHVQRYVATMVSNIDTVIDGWQPGQRLDIYQELRSAVRRST 128

CYP139A1_2575094331_Mycob 59 PVDGPTALIVSDGADHRRRRSVVAPGLRHHHVQRYVATMVSNIDTVIDGWQPGQRLDIYQELRSAVRRST 128

CYP139A1_2576101264_Mycob 59 PVDGPTALIVSDGADHRRRRSVVAPGLRHHHVQRYVATMVSNIDTVIDGWQPGQRLDIYQELRSAVRRST 128

CYP139A1_2576927477_Mycob 59 PVDGPTALIVSDGADHRRRRSVVAPGLRHHHVQRYVATMVSNIDTVIDGWQPGQRLDIYQELRSAVRRST 128

CYP139A1_2577009596_Mycob 59 PVDGPTALIVSDGADHRRRRSVVAPGLRHHHVQRYVATMVSNIDTVIDGWQPGQRLDIYQELRSAVRRST 128

CYP139A1_2577884547_Mycob 59 PVDGPTALIVSDGADHRRRRSVVAPGLRHHHVQRYVATMVSNIDTVIDGWQPGQRLDIYQELRSAVRRST 128

CYP139A1_2577997123_Mycob 59 PVDGPTALIVSDGADHRRRRSVVAPGLRHHHVQRYVATMVSNIDTVIDGWQPGQRLDIYQELRSAVRRST 128

CYP139A1_2578155745_Mycob 59 PVDGPTALIVSDGADHRRRRSVVAPGLRHHHVQRYVATMVSNIDTVIDGWQPGQRLDIYQELRSAVRRST 128

CYP139A1_2581807696_Mycob 59 PVDGPTALIVSDGADHRRRRSVVAPGLRHHHVQRYVATMVSNIDTVIDGWQPGQRLDIYQELRSAVRRST 128

CYP139A1_2582018155_Mycob 59 PVDGPTALIVSDGADHRRRRSVVAPGLRHHHVQRYVATMVSNIDTVIDGWQPGQRLDIYQELRSAVRRST 128

CYP139A1_2589154518_Mycob 59 PVDGPTALIVSDGADHRRRRSVVAPGLRHHHVQRYVATMVSNIDTVIDGWQPGQRLDIYQELRSAVRRST 128

CYP139A1_2590109466_Mycob 59 PVDGPTALIVSDGADHRRRRSVVAPGLRHHHVQRYVATMVSNIDTVIDGWQPGQRLDIYQELRSAVRRST 128

CYP139A1_2590195268_Mycob 59 PVDGPTALIVSDGADHRRRRSVVAPGLRHHHVQRYVATMVSNIDTVIDGWQPGQRLDIYQELRSAVRRST 128

CYP139A1_2590198349_Mycob 59 PVDGPTALIVSDGADHRRRRSVVAPGLRHHHVQRYVATMVSNIDTVIDGWQPGQRLDIYQELRSAVRRST 128

CYP139A1_2574799840_Mycob 59 PVDGPTALIVSDGADHRRRRSVVAPGLRHHHVQRYVATMVSNIDTVIDGWQPGQRLDIYQELRSAVRRST 128

CYP139A1_2576399693_Mycob 59 PVDGPTALIVSDGADHRRRRSVVAPGLRHHHVQRYVATMVSNIDTVIDGWQPGQRLDIYQELRSAVRRST 128

CYP139A1_2577024745_Mycob 59 PVDGPTALIVSDGADHRRRRSVVAPGLRHHHVQRYVATMVSNIDTVIDGWQPGQRLDIYQELRSAVRRST 128

CYP139A1_2577872587_Mycob 59 PVDGPTALIVSDGADHRRRRSVVAPGLRHHHVQRYVATMVSNIDTVIDGWQPGQRLDIYQELRSAVRRST 128

CYP139A1_2579813772_Mycob 59 PVDGPTALIVSDGADHRRRRSVVAPGLRHHHVQRYVATMVSNIDTVIDGWQPGQRLDIYQELRSAVRRST 128

CYP139A1_2584772421_Mycob 59 PVDGPTALIVSDGADHRRRRSVVAPGLRHHHVQRYVATMVSNIDTVIDGWQPGQRLDIYQELRSAVRRST 128

CYP139A1_2584893836_Mycob 59 PVDGPTALIVSDGADHRRRRSVVAPGLRHHHVQRYVATMVSNIDTVIDGWQPGQRLDIYQELRSAVRRST 128

CYP139A1_2589026697_Mycob 59 PVDGPTALIVSDGADHRRRRSVVAPGLRHHHVQRYVATMVSNIDTVIDGWQPGQRLDIYQELRSAVRRST 128

CYP139A1_2589514641_Mycob 59 PVDGPTALIVSDGADHRRRRSVVAPGLRHHHVQRYVATMVSNIDTVIDGWQPGQRLDIYQELRSAVRRST 128

CYP139A1_2589547247_Mycob 59 PVDGPTALIVSDGADHRRRRSVVAPGLRHHHVQRYVATMVSNIDTVIDGWQPGQRLDIYQELRSAVRRST 128

CYP139A1_2589620637_Mycob 59 PVDGPTALIVSDGADHRRRRSVVAPGLRHHHVQRYVATMVSNIDTVIDGWQPGQRLDIYQELRSAVRRST 128

CYP139A1_2592230560_Mycob 59 PVDGPTALIVSDGADHRRRRSVVAPGLRHHHVQRYVATMVSNIDTVIDGWQPGQRLDIYQELRSAVRRST 128

CYP139A1_2592242791_Mycob 59 PVDGPTALIVSDGADHRRRRSVVAPGLRHHHVQRYVATMVSNIDTVIDGWQPGQRLDIYQELRSAVRRST 128

CYP139A1_2592299949_Mycob 59 PVDGPTALIVSDGADHRRRRSVVAPGLRHHHVQRYVATMVSNIDTVIDGWQPGQRLDIYQELRSAVRRST 128

CYP139A1_2592377452_Mycob 59 PVDGPTALIVSDGADHRRRRSVVAPGLRHHHVQRYVATMVSNIDTVIDGWQPGQRLDIYQELRSAVRRST 128

CYP139A1_2592405959_Mycob 59 PVDGPTALIVSDGADHRRRRSVVAPGLRHHHVQRYVATMVSNIDTVIDGWQPGQRLDIYQELRSAVRRST 128

CYP139A1_2592558321_Mycob 59 PVDGPTALIVSDGADHRRRRSVVAPGLRHHHVQRYVATMVSNIDTVIDGWQPGQRLDIYQELRSAVRRST 128

CYP139A1_648456112_Mycoba 59 PVDGPTALIVSDGADHRRRRSVVAPGLRHHHVQRYVATMVSNIDTVIDGWQPGQRLDIYQELRSAVRRST 128

CYP139A1_2575561335_Mycob 59 PVDGPTALIVSDGADHRRRRSVVAPGLRHHHVQRYVATMVSNIDTVIDGWQPGQRLDIYQELRSAVRRST 128

CYP139A1_2575869049_Mycob 59 PVDGPTALIVSDGADHRRRRSVVAPGLRHHHVQRYVATMVSNIDTVIDGWQPGQRLDIYQELRSAVRRST 128

CYP139A1_2576250927_Mycob 59 PVDGPTALIVSDGADHRRRRSVVAPGLRHHHVQRYVATMVSNIDTVIDGWQPGQRLDIYQELRSAVRRST 128

CYP139A1_2577075963_Mycob 59 PVDGPTALIVSDGADHRRRRSVVAPGLRHHHVQRYVATMVSNIDTVIDGWQPGQRLDIYQELRSAVRRST 128

CYP139A1_2577655387_Mycob 59 PVDGPTALIVSDGADHRRRRSVVAPGLRHHHVQRYVATMVSNIDTVIDGWQPGQRLDIYQELRSAVRRST 128

CYP139A1_2579808474_Mycob 59 PVDGPTALIVSDGADHRRRRSVVAPGLRHHHVQRYVATMVSNIDTVIDGWQPGQRLDIYQELRSAVRRST 128

CYP139A1_2580939152_Mycob 59 PVDGPTALIVSDGADHRRRRSVVAPGLRHHHVQRYVATMVSNIDTVIDGWQPGQRLDIYQELRSAVRRST 128

CYP139A1_2581510874_Mycob 59 PVDGPTALIVSDGADHRRRRSVVAPGLRHHHVQRYVATMVSNIDTVIDGWQPGQRLDIYQELRSAVRRST 128

CYP139A1_2590048710_Mycob 59 PVDGPTALIVSDGADHRRRRSVVAPGLRHHHVQRYVATMVSNIDTVIDGWQPGQRLDIYQELRSAVRRST 128

CYP139A1_2590237321_Mycob 59 PVDGPTALIVSDGADHRRRRSVVAPGLRHHHVQRYVATMVSNIDTVIDGWQPGQRLDIYQELRSAVRRST 128

CYP139A1_2574784397_Mycob 59 PVDGPTALIVSDGADHRRRRSVVAPGLRHHHVQRYVATMVSNIDTVIDGWQPGQRLDIYQELRSAVRRST 128

CYP139A1_2574860198_Mycob 59 PVDGPTALIVSDGADHRRRRSVVAPGLRHHHVQRYVATMVSNIDTVIDGWQPGQRLDIYQELRSAVRRST 128

CYP139A1_2574872651_Mycob 59 PVDGPTALIVSDGADHRRRRSVVAPGLRHHHVQRYVATMVSNIDTVIDGWQPGQRLDIYQELRSAVRRST 128

CYP139A1_2575185207_Mycob 59 PVDGPTALIVSDGADHRRRRSVVAPGLRHHHVQRYVATMVSNIDTVIDGWQPGQRLDIYQELRSAVRRST 128

CYP139A1_2576316581_Mycob 59 PVDGPTALIVSDGADHRRRRSVVAPGLRHHHVQRYVATMVSNIDTVIDGWQPGQRLDIYQELRSAVRRST 128

CYP139A1_2576932272_Mycob 59 PVDGPTALIVSDGADHRRRRSVVAPGLRHHHVQRYVATMVSNIDTVIDGWQPGQRLDIYQELRSAVRRST 128

CYP139A1_2577632297_Mycob 59 PVDGPTALIVSDGADHRRRRSVVAPGLRHHHVQRYVATMVSNIDTVIDGWQPGQRLDIYQELRSAVRRST 128

CYP139A1_2577641910_Mycob 59 PVDGPTALIVSDGADHRRRRSVVAPGLRHHHVQRYVATMVSNIDTVIDGWQPGQRLDIYQELRSAVRRST 128

CYP139A1_2584609065_Mycob 59 PVDGPTALIVSDGADHRRRRSVVAPGLRHHHVQRYVATMVSNIDTVIDGWQPGQRLDIYQELRSAVRRST 128

CYP139A1_2584674477_Mycob 59 PVDGPTALIVSDGADHRRRRSVVAPGLRHHHVQRYVATMVSNIDTVIDGWQPGQRLDIYQELRSAVRRST 128

CYP139A1_2584806904_Mycob 59 PVDGPTALIVSDGADHRRRRSVVAPGLRHHHVQRYVATMVSNIDTVIDGWQPGQRLDIYQELRSAVRRST 128

CYP139A1_2584849650_Mycob 59 PVDGPTALIVSDGADHRRRRSVVAPGLRHHHVQRYVATMVSNIDTVIDGWQPGQRLDIYQELRSAVRRST 128

CYP139A1_2584854188_Mycob 59 PVDGPTALIVSDGADHRRRRSVVAPGLRHHHVQRYVATMVSNIDTVIDGWQPGQRLDIYQELRSAVRRST 128

CYP139A1_2589100068_Mycob 59 PVDGPTALIVSDGADHRRRRSVVAPGLRHHHVQRYVATMVSNIDTVIDGWQPGQRLDIYQELRSAVRRST 128

CYP139A1_2589666655_Mycob 59 PVDGPTALIVSDGADHRRRRSVVAPGLRHHHVQRYVATMVSNIDTVIDGWQPGQRLDIYQELRSAVRRST 128

CYP139A1_2589703490_Mycob 59 PVDGPTALIVSDGADHRRRRSVVAPGLRHHHVQRYVATMVSNIDTVIDGWQPGQRLDIYQELRSAVRRST 128

CYP139A1_2589724111_Mycob 59 PVDGPTALIVSDGADHRRRRSVVAPGLRHHHVQRYVATMVSNIDTVIDGWQPGQRLDIYQELRSAVRRST 128

CYP139A1_2590117900_Mycob 59 PVDGPTALIVSDGADHRRRRSVVAPGLRHHHVQRYVATMVSNIDTVIDGWQPGQRLDIYQELRSAVRRST 128

CYP139A1_2590134192_Mycob 59 PVDGPTALIVSDGADHRRRRSVVAPGLRHHHVQRYVATMVSNIDTVIDGWQPGQRLDIYQELRSAVRRST 128

CYP139A1_2590166763_Mycob 59 PVDGPTALIVSDGADHRRRRSVVAPGLRHHHVQRYVATMVSNIDTVIDGWQPGQRLDIYQELRSAVRRST 128

CYP139A1_2590356674_Mycob 59 PVDGPTALIVSDGADHRRRRSVVAPGLRHHHVQRYVATMVSNIDTVIDGWQPGQRLDIYQELRSAVRRST 128

CYP139A1_2549410800_Mycob 44 PVDGPTALIVSDGADHRRRRSVVAPGLRHHHVQRYVATMVSNIDTVIDGWQPGQRLDIYQELRSAVRRST 113

CYP139A1_2575655283_Mycob 59 PVDGPTALIVSDGADHRRRRSVVAPGLRHHHVQRYVATMVSNIDTVIDGWQPGQRLDIYQELRSAVRRST 128

CYP139A1_2576373809_Mycob 59 PVDGPTALIVSDGADHRRRRSVVAPGLRHHHVQRYVATMVSNIDTVIDGWQPGQRLDIYQELRSAVRRST 128

CYP139A1_2576609942_Mycob 59 PVDGPTALIVSDGADHRRRRSVVAPGLRHHHVQRYVATMVSNIDTVIDGWQPGQRLDIYQELRSAVRRST 128

CYP139A1_2576684060_Mycob 59 PVDGPTALIVSDGADHRRRRSVVAPGLRHHHVQRYVATMVSNIDTVIDGWQPGQRLDIYQELRSAVRRST 128

CYP139A1_2577110129_Mycob 59 PVDGPTALIVSDGADHRRRRSVVAPGLRHHHVQRYVATMVSNIDTVIDGWQPGQRLDIYQELRSAVRRST 128

CYP139A1_2577733463_Mycob 59 PVDGPTALIVSDGADHRRRRSVVAPGLRHHHVQRYVATMVSNIDTVIDGWQPGQRLDIYQELRSAVRRST 128

CYP139A1_2578170996_Mycob 59 PVDGPTALIVSDGADHRRRRSVVAPGLRHHHVQRYVATMVSNIDTVIDGWQPGQRLDIYQELRSAVRRST 128

CYP139A1_2583731958_Mycob 59 PVDGPTALIVSDGADHRRRRSVVAPGLRHHHVQRYVATMVSNIDTVIDGWQPGQRLDIYQELRSAVRRST 128

CYP139A1_2590523531_Mycob 59 PVDGPTALIVSDGADHRRRRSVVAPGLRHHHVQRYVATMVSNIDTVIDGWQPGQRLDIYQELRSAVRRST 128

CYP139A1_2590526411_Mycob 59 PVDGPTALIVSDGADHRRRRSVVAPGLRHHHVQRYVATMVSNIDTVIDGWQPGQRLDIYQELRSAVRRST 128

CYP139A1_2590552289_Mycob 59 PVDGPTALIVSDGADHRRRRSVVAPGLRHHHVQRYVATMVSNIDTVIDGWQPGQRLDIYQELRSAVRRST 128

CYP139A1_2575205306_Mycob 59 PVDGPTALIVSDGADHRRRRSVVAPGLRHHHVQRYVATMVSNIDTVIDGWQPGQRLDIYQELRSAVRRST 128

CYP139A1_2575230562_Mycob 59 PVDGPTALIVSDGADHRRRRSVVAPGLRHHHVQRYVATMVSNIDTVIDGWQPGQRLDIYQELRSAVRRST 128

CYP139A1_2575304538_Mycob 59 PVDGPTALIVSDGADHRRRRSVVAPGLRHHHVQRYVATMVSNIDTVIDGWQPGQRLDIYQELRSAVRRST 128

CYP139A1_2575476916_Mycob 59 PVDGPTALIVSDGADHRRRRSVVAPGLRHHHVQRYVATMVSNIDTVIDGWQPGQRLDIYQELRSAVRRST 128

CYP139A1_2575790354_Mycob 59 PVDGPTALIVSDGADHRRRRSVVAPGLRHHHVQRYVATMVSNIDTVIDGWQPGQRLDIYQELRSAVRRST 128

CYP139A1_2576077839_Mycob 59 PVDGPTALIVSDGADHRRRRSVVAPGLRHHHVQRYVATMVSNIDTVIDGWQPGQRLDIYQELRSAVRRST 128

CYP139A1_2576163842_Mycob 59 PVDGPTALIVSDGADHRRRRSVVAPGLRHHHVQRYVATMVSNIDTVIDGWQPGQRLDIYQELRSAVRRST 128

CYP139A1_2577313382_Mycob 59 PVDGPTALIVSDGADHRRRRSVVAPGLRHHHVQRYVATMVSNIDTVIDGWQPGQRLDIYQELRSAVRRST 128

CYP139A1_2577519304_Mycob 59 PVDGPTALIVSDGADHRRRRSVVAPGLRHHHVQRYVATMVSNIDTVIDGWQPGQRLDIYQELRSAVRRST 128

CYP139A1_2577816400_Mycob 59 PVDGPTALIVSDGADHRRRRSVVAPGLRHHHVQRYVATMVSNIDTVIDGWQPGQRLDIYQELRSAVRRST 128

CYP139A1_2584670398_Mycob 59 PVDGPTALIVSDGADHRRRRSVVAPGLRHHHVQRYVATMVSNIDTVIDGWQPGQRLDIYQELRSAVRRST 128

CYP139A1_2584841030_Mycob 59 PVDGPTALIVSDGADHRRRRSVVAPGLRHHHVQRYVATMVSNIDTVIDGWQPGQRLDIYQELRSAVRRST 128

CYP139A1_2584865918_Mycob 59 PVDGPTALIVSDGADHRRRRSVVAPGLRHHHVQRYVATMVSNIDTVIDGWQPGQRLDIYQELRSAVRRST 128

CYP139A1_2589588224_Mycob 59 PVDGPTALIVSDGADHRRRRSVVAPGLRHHHVQRYVATMVSNIDTVIDGWQPGQRLDIYQELRSAVRRST 128

CYP139A1_2589624713_Mycob 59 PVDGPTALIVSDGADHRRRRSVVAPGLRHHHVQRYVATMVSNIDTVIDGWQPGQRLDIYQELRSAVRRST 128

CYP139A1_2589736180_Mycob 59 PVDGPTALIVSDGADHRRRRSVVAPGLRHHHVQRYVATMVSNIDTVIDGWQPGQRLDIYQELRSAVRRST 128

CYP139A1_2590032388_Mycob 59 PVDGPTALIVSDGADHRRRRSVVAPGLRHHHVQRYVATMVSNIDTVIDGWQPGQRLDIYQELRSAVRRST 128

CYP139A1_2590501611_Mycob 59 PVDGPTALIVSDGADHRRRRSVVAPGLRHHHVQRYVATMVSNIDTVIDGWQPGQRLDIYQELRSAVRRST 128

CYP139A1_2592312197_Mycob 59 PVDGPTALIVSDGADHRRRRSVVAPGLRHHHVQRYVATMVSNIDTVIDGWQPGQRLDIYQELRSAVRRST 128

CYP139A1_2592435284_Mycob 59 PVDGPTALIVSDGADHRRRRSVVAPGLRHHHVQRYVATMVSNIDTVIDGWQPGQRLDIYQELRSAVRRST 128

CYP139A1_2511811274_Mycob 59 PVDGPTALIVSDGADHRRRRSVVAPGLRHHHVQRYVATMVSNIDTVIDGWQPGQRLDIYQELRSAVRRST 128

CYP139A1_2546202077_Mycob 59 PVDGPTALIVSDGADHRRRRSVVAPGLRHHHVQRYVATMVSNIDTVIDGWQPGQRLDIYQELRSAVRRST 128

CYP139A1_2574693694_Mycob 59 PVDGPTALIVSDGADHRRRRSVVAPGLRHHHVQRYVATMVSNIDTVIDGWQPGQRLDIYQELRSAVRRST 128

CYP139A1_2574968392_Mycob 59 PVDGPTALIVSDGADHRRRRSVVAPGLRHHHVQRYVATMVSNIDTVIDGWQPGQRLDIYQELRSAVRRST 128

CYP139A1_2577060340_Mycob 59 PVDGPTALIVSDGADHRRRRSVVAPGLRHHHVQRYVATMVSNIDTVIDGWQPGQRLDIYQELRSAVRRST 128

CYP139A1_2577454945_Mycob 59 PVDGPTALIVSDGADHRRRRSVVAPGLRHHHVQRYVATMVSNIDTVIDGWQPGQRLDIYQELRSAVRRST 128

CYP139A1_2580123929_Mycob 59 PVDGPTALIVSDGADHRRRRSVVAPGLRHHHVQRYVATMVSNIDTVIDGWQPGQRLDIYQELRSAVRRST 128

CYP139A1_2581930746_Mycob 59 PVDGPTALIVSDGADHRRRRSVVAPGLRHHHVQRYVATMVSNIDTVIDGWQPGQRLDIYQELRSAVRRST 128

CYP139A1_2584196432_Mycob 59 PVDGPTALIVSDGADHRRRRSVVAPGLRHHHVQRYVATMVSNIDTVIDGWQPGQRLDIYQELRSAVRRST 128

CYP139A1_2584726832_Mycob 59 PVDGPTALIVSDGADHRRRRSVVAPGLRHHHVQRYVATMVSNIDTVIDGWQPGQRLDIYQELRSAVRRST 128

CYP139A1_2584911080_Mycob 59 PVDGPTALIVSDGADHRRRRSVVAPGLRHHHVQRYVATMVSNIDTVIDGWQPGQRLDIYQELRSAVRRST 128

CYP139A1_2584962435_Mycob 59 PVDGPTALIVSDGADHRRRRSVVAPGLRHHHVQRYVATMVSNIDTVIDGWQPGQRLDIYQELRSAVRRST 128

CYP139A1_2584995097_Mycob 59 PVDGPTALIVSDGADHRRRRSVVAPGLRHHHVQRYVATMVSNIDTVIDGWQPGQRLDIYQELRSAVRRST 128

CYP139A1_640602381_Mycoba 59 PVDGPTALIVSDGADHRRRRSVVAPGLRHHHVQRYVATMVSNIDTVIDGWQPGQRLDIYQELRSAVRRST 128

CYP139A1_643019022_Mycoba 59 PVDGPTALIVSDGADHRRRRSVVAPGLRHHHVQRYVATMVSNIDTVIDGWQPGQRLDIYQELRSAVRRST 128

CYP139A1_2573562450_Mycob 59 PVDGPTALIVSDGADHRRRRSVVAPGLRHHHVQRYVATMVSNIDTVIDGWQPGQRLDIYQELRSAVRRST 128

CYP139A1_2575255926_Mycob 59 PVDGPTALIVSDGADHRRRRSVVAPGLRHHHVQRYVATMVSNIDTVIDGWQPGQRLDIYQELRSAVRRST 128

CYP139A1_2577905307_Mycob 59 PVDGPTALIVSDGADHRRRRSVVAPGLRHHHVQRYVATMVSNIDTVIDGWQPGQRLDIYQELRSAVRRST 128

CYP139A1_2579818167_Mycob 59 PVDGPTALIVSDGADHRRRRSVVAPGLRHHHVQRYVATMVSNIDTVIDGWQPGQRLDIYQELRSAVRRST 128

CYP139A1_2582001898_Mycob 59 PVDGPTALIVSDGADHRRRRSVVAPGLRHHHVQRYVATMVSNIDTVIDGWQPGQRLDIYQELRSAVRRST 128

CYP139A1_2588538927_Mycob 59 PVDGPTALIVSDGADHRRRRSVVAPGLRHHHVQRYVATMVSNIDTVIDGWQPGQRLDIYQELRSAVRRST 128

CYP139A1_2590073317_Mycob 59 PVDGPTALIVSDGADHRRRRSVVAPGLRHHHVQRYVATMVSNIDTVIDGWQPGQRLDIYQELRSAVRRST 128

CYP139A1_2590075971_Mycob 59 PVDGPTALIVSDGADHRRRRSVVAPGLRHHHVQRYVATMVSNIDTVIDGWQPGQRLDIYQELRSAVRRST 128

CYP139A1_2590211264_Mycob 59 PVDGPTALIVSDGADHRRRRSVVAPGLRHHHVQRYVATMVSNIDTVIDGWQPGQRLDIYQELRSAVRRST 128

CYP139A1_2590249475_Mycob 59 PVDGPTALIVSDGADHRRRRSVVAPGLRHHHVQRYVATMVSNIDTVIDGWQPGQRLDIYQELRSAVRRST 128

CYP139A1_2590279314_Mycob 59 PVDGPTALIVSDGADHRRRRSVVAPGLRHHHVQRYVATMVSNIDTVIDGWQPGQRLDIYQELRSAVRRST 128

CYP139A1_2590559932_Mycob 59 PVDGPTALIVSDGADHRRRRSVVAPGLRHHHVQRYVATMVSNIDTVIDGWQPGQRLDIYQELRSAVRRST 128

CYP139A1_640606444_Mycoba 59 PVDGPTALIVSDGADHRRRRSVVAPGLRHHHVQRYVATMVSNIDTVIDGWQPGQRLDIYQELRSAVRRST 128

CYP139A1_651025167_Mycoba 59 PVDGPTALIVSDGADHRRRRSVVAPGLRHHHVQRYVATMVSNIDTVIDGWQPGQRLDIYQELRSAVRRST 128

CYP139A1_2574876573_Mycob 59 PVDGPTALIVSDGADHRRRRSVVAPGLRHHHVQRYVATMVSNIDTVIDGWQPGQRLDIYQELRSAVRRST 128

CYP139A1_2575028161_Mycob 59 PVDGPTALIVSDGADHRRRRSVVAPGLRHHHVQRYVATMVSNIDTVIDGWQPGQRLDIYQELRSAVRRST 128

CYP139A1_2575888575_Mycob 59 PVDGPTALIVSDGADHRRRRSVVAPGLRHHHVQRYVATMVSNIDTVIDGWQPGQRLDIYQELRSAVRRST 128

CYP139A1_2576708817_Mycob 59 PVDGPTALIVSDGADHRRRRSVVAPGLRHHHVQRYVATMVSNIDTVIDGWQPGQRLDIYQELRSAVRRST 128

CYP139A1_2577386815_Mycob 59 PVDGPTALIVSDGADHRRRRSVVAPGLRHHHVQRYVATMVSNIDTVIDGWQPGQRLDIYQELRSAVRRST 128

CYP139A1_2578040087_Mycob 59 PVDGPTALIVSDGADHRRRRSVVAPGLRHHHVQRYVATMVSNIDTVIDGWQPGQRLDIYQELRSAVRRST 128

CYP139A1_2584645880_Mycob 59 PVDGPTALIVSDGADHRRRRSVVAPGLRHHHVQRYVATMVSNIDTVIDGWQPGQRLDIYQELRSAVRRST 128

CYP139A1_2584659187_Mycob 59 PVDGPTALIVSDGADHRRRRSVVAPGLRHHHVQRYVATMVSNIDTVIDGWQPGQRLDIYQELRSAVRRST 128

CYP139A1_2584718757_Mycob 59 PVDGPTALIVSDGADHRRRRSVVAPGLRHHHVQRYVATMVSNIDTVIDGWQPGQRLDIYQELRSAVRRST 128

CYP139A1_2589064595_Mycob 59 PVDGPTALIVSDGADHRRRRSVVAPGLRHHHVQRYVATMVSNIDTVIDGWQPGQRLDIYQELRSAVRRST 128

CYP139A1_2589113575_Mycob 59 PVDGPTALIVSDGADHRRRRSVVAPGLRHHHVQRYVATMVSNIDTVIDGWQPGQRLDIYQELRSAVRRST 128

CYP139A1_2589559459_Mycob 59 PVDGPTALIVSDGADHRRRRSVVAPGLRHHHVQRYVATMVSNIDTVIDGWQPGQRLDIYQELRSAVRRST 128

CYP139A1_2589575466_Mycob 59 PVDGPTALIVSDGADHRRRRSVVAPGLRHHHVQRYVATMVSNIDTVIDGWQPGQRLDIYQELRSAVRRST 128

CYP139A1_2589595990_Mycob 59 PVDGPTALIVSDGADHRRRRSVVAPGLRHHHVQRYVATMVSNIDTVIDGWQPGQRLDIYQELRSAVRRST 128

CYP139A1_2592259141_Mycob 59 PVDGPTALIVSDGADHRRRRSVVAPGLRHHHVQRYVATMVSNIDTVIDGWQPGQRLDIYQELRSAVRRST 128

CYP139A1_2592280664_Mycob 59 PVDGPTALIVSDGADHRRRRSVVAPGLRHHHVQRYVATMVSNIDTVIDGWQPGQRLDIYQELRSAVRRST 128

CYP139A1_2592389699_Mycob 59 PVDGPTALIVSDGADHRRRRSVVAPGLRHHHVQRYVATMVSNIDTVIDGWQPGQRLDIYQELRSAVRRST 128

CYP139A1_2592438630_Mycob 59 PVDGPTALIVSDGADHRRRRSVVAPGLRHHHVQRYVATMVSNIDTVIDGWQPGQRLDIYQELRSAVRRST 128

CYP139A1_2592553482_Mycob 59 PVDGPTALIVSDGADHRRRRSVVAPGLRHHHVQRYVATMVSNIDTVIDGWQPGQRLDIYQELRSAVRRST 128

CYP139A1_2592570555_Mycob 59 PVDGPTALIVSDGADHRRRRSVVAPGLRHHHVQRYVATMVSNIDTVIDGWQPGQRLDIYQELRSAVRRST 128

CYP139A1_2575252379_Mycob 59 PVDGPTALIVSDGADHRRRRSVVAPGLRHHHVQRYVATMVSNIDTVIDGWQPGQRLDIYQELRSAVRRST 128

CYP139A1_2575520720_Mycob 59 PVDGPTALIVSDGADHRRRRSVVAPGLRHHHVQRYVATMVSNIDTVIDGWQPGQRLDIYQELRSAVRRST 128

CYP139A1_2575705222_Mycob 59 PVDGPTALIVSDGADHRRRRSVVAPGLRHHHVQRYVATMVSNIDTVIDGWQPGQRLDIYQELRSAVRRST 128

CYP139A1_2575964273_Mycob 59 PVDGPTALIVSDGADHRRRRSVVAPGLRHHHVQRYVATMVSNIDTVIDGWQPGQRLDIYQELRSAVRRST 128

CYP139A1_2577195360_Mycob 59 PVDGPTALIVSDGADHRRRRSVVAPGLRHHHVQRYVATMVSNIDTVIDGWQPGQRLDIYQELRSAVRRST 128

CYP139A1_2581587072_Mycob 59 PVDGPTALIVSDGADHRRRRSVVAPGLRHHHVQRYVATMVSNIDTVIDGWQPGQRLDIYQELRSAVRRST 128

CYP139A1_2584633235_Mycob 59 PVDGPTALIVSDGADHRRRRSVVAPGLRHHHVQRYVATMVSNIDTVIDGWQPGQRLDIYQELRSAVRRST 128

CYP139A1_2584732271_Mycob 59 PVDGPTALIVSDGADHRRRRSVVAPGLRHHHVQRYVATMVSNIDTVIDGWQPGQRLDIYQELRSAVRRST 128

CYP139A1_2584791771_Mycob 59 PVDGPTALIVSDGADHRRRRSVVAPGLRHHHVQRYVATMVSNIDTVIDGWQPGQRLDIYQELRSAVRRST 128

CYP139A1_2589148607_Mycob 59 PVDGPTALIVSDGADHRRRRSVVAPGLRHHHVQRYVATMVSNIDTVIDGWQPGQRLDIYQELRSAVRRST 128

CYP139A1_2590150448_Mycob 59 PVDGPTALIVSDGADHRRRRSVVAPGLRHHHVQRYVATMVSNIDTVIDGWQPGQRLDIYQELRSAVRRST 128

CYP139A1_2590231653_Mycob 59 PVDGPTALIVSDGADHRRRRSVVAPGLRHHHVQRYVATMVSNIDTVIDGWQPGQRLDIYQELRSAVRRST 128

CYP139A1_2590283895_Mycob 59 PVDGPTALIVSDGADHRRRRSVVAPGLRHHHVQRYVATMVSNIDTVIDGWQPGQRLDIYQELRSAVRRST 128

CYP139A1_645120373_Mycoba 59 PVDGPTALIVSDGADHRRRRSVVAPGLRHHHVQRYVATMVSNIDTVIDGWQPGQRLDIYQELRSAVRRST 128

CYP139A1_2574734007_Mycob 59 PVDGPTALIVSDGADHRRRRSVVAPGLRHHHVQRYVATMVSNIDTVIDGWQPGQRLDIYQELRSAVRRST 128

CYP139A1_2575099885_Mycob 59 PVDGPTALIVSDGADHRRRRSVVAPGLRHHHVQRYVATMVSNIDTVIDGWQPGQRLDIYQELRSAVRRST 128

CYP139A1_2575132657_Mycob 59 PVDGPTALIVSDGADHRRRRSVVAPGLRHHHVQRYVATMVSNIDTVIDGWQPGQRLDIYQELRSAVRRST 128

CYP139A1_2575465536_Mycob 59 PVDGPTALIVSDGADHRRRRSVVAPGLRHHHVQRYVATMVSNIDTVIDGWQPGQRLDIYQELRSAVRRST 128

CYP139A1_2576665707_Mycob 59 PVDGPTALIVSDGADHRRRRSVVAPGLRHHHVQRYVATMVSNIDTVIDGWQPGQRLDIYQELRSAVRRST 128

CYP139A1_2576698280_Mycob 59 PVDGPTALIVSDGADHRRRRSVVAPGLRHHHVQRYVATMVSNIDTVIDGWQPGQRLDIYQELRSAVRRST 128

CYP139A1_2576759003_Mycob 59 PVDGPTALIVSDGADHRRRRSVVAPGLRHHHVQRYVATMVSNIDTVIDGWQPGQRLDIYQELRSAVRRST 128

CYP139A1_2577673179_Mycob 59 PVDGPTALIVSDGADHRRRRSVVAPGLRHHHVQRYVATMVSNIDTVIDGWQPGQRLDIYQELRSAVRRST 128

CYP139A1_2577936335_Mycob 59 PVDGPTALIVSDGADHRRRRSVVAPGLRHHHVQRYVATMVSNIDTVIDGWQPGQRLDIYQELRSAVRRST 128

CYP139A1_2578008273_Mycob 59 PVDGPTALIVSDGADHRRRRSVVAPGLRHHHVQRYVATMVSNIDTVIDGWQPGQRLDIYQELRSAVRRST 128

CYP139A1_2578073775_Mycob 59 PVDGPTALIVSDGADHRRRRSVVAPGLRHHHVQRYVATMVSNIDTVIDGWQPGQRLDIYQELRSAVRRST 128

CYP139A1_2584960744_Mycob 59 PVDGPTALIVSDGADHRRRRSVVAPGLRHHHVQRYVATMVSNIDTVIDGWQPGQRLDIYQELRSAVRRST 128

CYP139A1_2588992251_Mycob 59 PVDGPTALIVSDGADHRRRRSVVAPGLRHHHVQRYVATMVSNIDTVIDGWQPGQRLDIYQELRSAVRRST 128

CYP139A1_2589077872_Mycob 59 PVDGPTALIVSDGADHRRRRSVVAPGLRHHHVQRYVATMVSNIDTVIDGWQPGQRLDIYQELRSAVRRST 128

CYP139A1_2589518744_Mycob 59 PVDGPTALIVSDGADHRRRRSVVAPGLRHHHVQRYVATMVSNIDTVIDGWQPGQRLDIYQELRSAVRRST 128

CYP139A1_2589583955_Mycob 59 PVDGPTALIVSDGADHRRRRSVVAPGLRHHHVQRYVATMVSNIDTVIDGWQPGQRLDIYQELRSAVRRST 128

CYP139A1_2589646609_Mycob 59 PVDGPTALIVSDGADHRRRRSVVAPGLRHHHVQRYVATMVSNIDTVIDGWQPGQRLDIYQELRSAVRRST 128

CYP139A1_2592316279_Mycob 59 PVDGPTALIVSDGADHRRRRSVVAPGLRHHHVQRYVATMVSNIDTVIDGWQPGQRLDIYQELRSAVRRST 128

CYP139A1_2592546111_Mycob 59 PVDGPTALIVSDGADHRRRRSVVAPGLRHHHVQRYVATMVSNIDTVIDGWQPGQRLDIYQELRSAVRRST 128

CYP139A1_2511553315_Mycob 59 PVDGPTALIVSDGADHRRRRSVVAPGLRHHHVQRYVATMVSNIDTVIDGWQPGQRLDIYQELRSAVRRST 128

CYP139A1_2574949227_Mycob 59 PVDGPTALIVSDGADHRRRRSVVAPGLRHHHVQRYVATMVSNIDTVIDGWQPGQRLDIYQELRSAVRRST 128

CYP139A1_2575533703_Mycob 59 PVDGPTALIVSDGADHRRRRSVVAPGLRHHHVQRYVATMVSNIDTVIDGWQPGQRLDIYQELRSAVRRST 128

CYP139A1_2576015739_Mycob 59 PVDGPTALIVSDGADHRRRRSVVAPGLRHHHVQRYVATMVSNIDTVIDGWQPGQRLDIYQELRSAVRRST 128

CYP139A1_2576644886_Mycob 59 PVDGPTALIVSDGADHRRRRSVVAPGLRHHHVQRYVATMVSNIDTVIDGWQPGQRLDIYQELRSAVRRST 128

CYP139A1_2577497204_Mycob 59 PVDGPTALIVSDGADHRRRRSVVAPGLRHHHVQRYVATMVSNIDTVIDGWQPGQRLDIYQELRSAVRRST 128

CYP139A1_2580744928_Mycob 59 PVDGPTALIVSDGADHRRRRSVVAPGLRHHHVQRYVATMVSNIDTVIDGWQPGQRLDIYQELRSAVRRST 128

CYP139A1_2583727889_Mycob 59 PVDGPTALIVSDGADHRRRRSVVAPGLRHHHVQRYVATMVSNIDTVIDGWQPGQRLDIYQELRSAVRRST 128

CYP139A1_2590064965_Mycob 59 PVDGPTALIVSDGADHRRRRSVVAPGLRHHHVQRYVATMVSNIDTVIDGWQPGQRLDIYQELRSAVRRST 128

CYP139A1_2590121989_Mycob 59 PVDGPTALIVSDGADHRRRRSVVAPGLRHHHVQRYVATMVSNIDTVIDGWQPGQRLDIYQELRSAVRRST 128

CYP139A1_2590294228_Mycob 59 PVDGPTALIVSDGADHRRRRSVVAPGLRHHHVQRYVATMVSNIDTVIDGWQPGQRLDIYQELRSAVRRST 128

CYP139A1_2590499050_Mycob 59 PVDGPTALIVSDGADHRRRRSVVAPGLRHHHVQRYVATMVSNIDTVIDGWQPGQRLDIYQELRSAVRRST 128

CYP139A1_2590519464_Mycob 59 PVDGPTALIVSDGADHRRRRSVVAPGLRHHHVQRYVATMVSNIDTVIDGWQPGQRLDIYQELRSAVRRST 128

CYP139A1_2590548005_Mycob 59 PVDGPTALIVSDGADHRRRRSVVAPGLRHHHVQRYVATMVSNIDTVIDGWQPGQRLDIYQELRSAVRRST 128

CYP139A1_637139034_Mycoba 59 PVDGPTALIVSDGADHRRRRSVVAPGLRHHHVQRYVATMVSNIDTVIDGWQPGQRLDIYQELRSAVRRST 128

CYP139A1_639830617_Mycoba 59 PVDGPTALIVSDGADHRRRRSVVAPGLRHHHVQRYVATMVSNIDTVIDGWQPGQRLDIYQELRSAVRRST 128

CYP139A1_2574640348_Mycob 59 PVDGPTALIVSDGADHRRRRSVVAPGLRHHHVQRYVATMVSNIDTVIDGWQPGQRLDIYQELRSAVRRST 128

CYP139A1_2574794413_Mycob 59 PVDGPTALIVSDGADHRRRRSVVAPGLRHHHVQRYVATMVSNIDTVIDGWQPGQRLDIYQELRSAVRRST 128

CYP139A1_2574987296_Mycob 59 PVDGPTALIVSDGADHRRRRSVVAPGLRHHHVQRYVATMVSNIDTVIDGWQPGQRLDIYQELRSAVRRST 128

CYP139A1_2575084753_Mycob 59 PVDGPTALIVSDGADHRRRRSVVAPGLRHHHVQRYVATMVSNIDTVIDGWQPGQRLDIYQELRSAVRRST 128

CYP139A1_2575627312_Mycob 59 PVDGPTALIVSDGADHRRRRSVVAPGLRHHHVQRYVATMVSNIDTVIDGWQPGQRLDIYQELRSAVRRST 128

CYP139A1_2576321347_Mycob 59 PVDGPTALIVSDGADHRRRRSVVAPGLRHHHVQRYVATMVSNIDTVIDGWQPGQRLDIYQELRSAVRRST 128

CYP139A1_2576378950_Mycob 59 PVDGPTALIVSDGADHRRRRSVVAPGLRHHHVQRYVATMVSNIDTVIDGWQPGQRLDIYQELRSAVRRST 128

CYP139A1_2576735552_Mycob 59 PVDGPTALIVSDGADHRRRRSVVAPGLRHHHVQRYVATMVSNIDTVIDGWQPGQRLDIYQELRSAVRRST 128

CYP139A1_2576940434_Mycob 59 PVDGPTALIVSDGADHRRRRSVVAPGLRHHHVQRYVATMVSNIDTVIDGWQPGQRLDIYQELRSAVRRST 128

CYP139A1_2577812038_Mycob 59 PVDGPTALIVSDGADHRRRRSVVAPGLRHHHVQRYVATMVSNIDTVIDGWQPGQRLDIYQELRSAVRRST 128

CYP139A1_2577861791_Mycob 59 PVDGPTALIVSDGADHRRRRSVVAPGLRHHHVQRYVATMVSNIDTVIDGWQPGQRLDIYQELRSAVRRST 128

CYP139A1_2578053379_Mycob 59 PVDGPTALIVSDGADHRRRRSVVAPGLRHHHVQRYVATMVSNIDTVIDGWQPGQRLDIYQELRSAVRRST 128

CYP139A1_2584860925_Mycob 59 PVDGPTALIVSDGADHRRRRSVVAPGLRHHHVQRYVATMVSNIDTVIDGWQPGQRLDIYQELRSAVRRST 128

CYP139A1_2584936325_Mycob 59 PVDGPTALIVSDGADHRRRRSVVAPGLRHHHVQRYVATMVSNIDTVIDGWQPGQRLDIYQELRSAVRRST 128

CYP139A1_2584939524_Mycob 59 PVDGPTALIVSDGADHRRRRSVVAPGLRHHHVQRYVATMVSNIDTVIDGWQPGQRLDIYQELRSAVRRST 128

CYP139A1_2589106145_Mycob 59 PVDGPTALIVSDGADHRRRRSVVAPGLRHHHVQRYVATMVSNIDTVIDGWQPGQRLDIYQELRSAVRRST 128

CYP139A1_2589543202_Mycob 59 PVDGPTALIVSDGADHRRRRSVVAPGLRHHHVQRYVATMVSNIDTVIDGWQPGQRLDIYQELRSAVRRST 128

CYP139A1_2589642534_Mycob 59 PVDGPTALIVSDGADHRRRRSVVAPGLRHHHVQRYVATMVSNIDTVIDGWQPGQRLDIYQELRSAVRRST 128

CYP139A1_2589691243_Mycob 59 PVDGPTALIVSDGADHRRRRSVVAPGLRHHHVQRYVATMVSNIDTVIDGWQPGQRLDIYQELRSAVRRST 128

CYP139A1_2589695547_Mycob 59 PVDGPTALIVSDGADHRRRRSVVAPGLRHHHVQRYVATMVSNIDTVIDGWQPGQRLDIYQELRSAVRRST 128

CYP139A1_2592222422_Mycob 59 PVDGPTALIVSDGADHRRRRSVVAPGLRHHHVQRYVATMVSNIDTVIDGWQPGQRLDIYQELRSAVRRST 128

CYP139A1_648446923_Mycoba 59 PVDGPTALIVSDGADHRRRRSVVAPGLRHHHVQRYVATMVSNIDTVIDGWQPGQRLDIYQELRSAVRRST 128

CYP139A1_648469578_Mycoba 59 PVDGPTALIVSDGADHRRRRSVVAPGLRHHHVQRYVATMVSNIDTVIDGWQPGQRLDIYQELRSAVRRST 128

CYP139A1_2546188127_Mycob 59 PVDGPTALIVSDGADHRRRRSVVAPGLRHHHVQRYVATMVSNIDTVIDGWQPGQRLDIYQELRSAVRRST 128

CYP139A1_2574773054_Mycob 59 PVDGPTALIVSDGADHRRRRSVVAPGLRHHHVQRYVATMVSNIDTVIDGWQPGQRLDIYQELRSAVRRST 128

CYP139A1_2574854728_Mycob 59 PVDGPTALIVSDGADHRRRRSVVAPGLRHHHVQRYVATMVSNIDTVIDGWQPGQRLDIYQELRSAVRRST 128

CYP139A1_2575984510_Mycob 59 PVDGPTALIVSDGADHRRRRSVVAPGLRHHHVQRYVATMVSNIDTVIDGWQPGQRLDIYQELRSAVRRST 128

CYP139A1_2576293562_Mycob 59 PVDGPTALIVSDGADHRRRRSVVAPGLRHHHVQRYVATMVSNIDTVIDGWQPGQRLDIYQELRSAVRRST 128

CYP139A1_2576717872_Mycob 59 PVDGPTALIVSDGADHRRRRSVVAPGLRHHHVQRYVATMVSNIDTVIDGWQPGQRLDIYQELRSAVRRST 128

CYP139A1_2578189802_Mycob 59 PVDGPTALIVSDGADHRRRRSVVAPGLRHHHVQRYVATMVSNIDTVIDGWQPGQRLDIYQELRSAVRRST 128

CYP139A1_2584890677_Mycob 59 PVDGPTALIVSDGADHRRRRSVVAPGLRHHHVQRYVATMVSNIDTVIDGWQPGQRLDIYQELRSAVRRST 128

CYP139A1_2590070071_Mycob 59 PVDGPTALIVSDGADHRRRRSVVAPGLRHHHVQRYVATMVSNIDTVIDGWQPGQRLDIYQELRSAVRRST 128

CYP139A1_2590169218_Mycob 59 PVDGPTALIVSDGADHRRRRSVVAPGLRHHHVQRYVATMVSNIDTVIDGWQPGQRLDIYQELRSAVRRST 128

CYP139A1_644880084_Mycoba 59 PVDGPTALIVSDGADHRRRRSVVAPGLRHHHVQRYVATMVSNIDTVIDGWQPGQRLDIYQELRSAVRRST 128

CYP139A1_2574905673_Mycob 59 PVDGPTALIVSDGADHRRRRSVVAPGLRHHHVQRYVATMVSNIDTVIDGWQPGQRLDIYQELRSAVRRST 128

CYP139A1_2575285636_Mycob 59 PVDGPTALIVSDGADHRRRRSVVAPGLRHHHVQRYVATMVSNIDTVIDGWQPGQRLDIYQELRSAVRRST 128

CYP139A1_2575508785_Mycob 59 PVDGPTALIVSDGADHRRRRSVVAPGLRHHHVQRYVATMVSNIDTVIDGWQPGQRLDIYQELRSAVRRST 128

CYP139A1_2576585830_Mycob 59 PVDGPTALIVSDGADHRRRRSVVAPGLRHHHVQRYVATMVSNIDTVIDGWQPGQRLDIYQELRSAVRRST 128

CYP139A1_2584979253_Mycob 59 PVDGPTALIVSDGADHRRRRSVVAPGLRHHHVQRYVATMVSNIDTVIDGWQPGQRLDIYQELRSAVRRST 128

CYP139A1_2588982991_Mycob 59 PVDGPTALIVSDGADHRRRRSVVAPGLRHHHVQRYVATMVSNIDTVIDGWQPGQRLDIYQELRSAVRRST 128

CYP139A1_2589048312_Mycob 59 PVDGPTALIVSDGADHRRRRSVVAPGLRHHHVQRYVATMVSNIDTVIDGWQPGQRLDIYQELRSAVRRST 128

CYP139A1_2589060525_Mycob 59 PVDGPTALIVSDGADHRRRRSVVAPGLRHHHVQRYVATMVSNIDTVIDGWQPGQRLDIYQELRSAVRRST 128

CYP139A1_2589502409_Mycob 59 PVDGPTALIVSDGADHRRRRSVVAPGLRHHHVQRYVATMVSNIDTVIDGWQPGQRLDIYQELRSAVRRST 128

CYP139A1_2589572815_Mycob 59 PVDGPTALIVSDGADHRRRRSVVAPGLRHHHVQRYVATMVSNIDTVIDGWQPGQRLDIYQELRSAVRRST 128

CYP139A1_2590181544_Mycob 59 PVDGPTALIVSDGADHRRRRSVVAPGLRHHHVQRYVATMVSNIDTVIDGWQPGQRLDIYQELRSAVRRST 128

CYP139A1_2592234638_Mycob 59 PVDGPTALIVSDGADHRRRRSVVAPGLRHHHVQRYVATMVSNIDTVIDGWQPGQRLDIYQELRSAVRRST 128

CYP139A1_2592271382_Mycob 59 PVDGPTALIVSDGADHRRRRSVVAPGLRHHHVQRYVATMVSNIDTVIDGWQPGQRLDIYQELRSAVRRST 128

CYP139A1_2592295853_Mycob 59 PVDGPTALIVSDGADHRRRRSVVAPGLRHHHVQRYVATMVSNIDTVIDGWQPGQRLDIYQELRSAVRRST 128

CYP139A1_2592344179_Mycob 59 PVDGPTALIVSDGADHRRRRSVVAPGLRHHHVQRYVATMVSNIDTVIDGWQPGQRLDIYQELRSAVRRST 128

CYP139A1_2592357110_Mycob 59 PVDGPTALIVSDGADHRRRRSVVAPGLRHHHVQRYVATMVSNIDTVIDGWQPGQRLDIYQELRSAVRRST 128

CYP139A1_643035957_Mycoba 59 PVDGPTALIVSDGADHRRRRSVVAPGLRHHHVQRYVATMVSNIDTVIDGWQPGQRLDIYQELRSAVRRST 128

CYP139A1_2575162612_Mycob 59 PVDGPTALIVSDGADHRRRRSVVAPGLRHHHVQRYVATMVSNIDTVIDGWQPGQRLDIYQELRSAVRRST 128

CYP139A1_2575198589_Mycob 59 PVDGPTALIVSDGADHRRRRSVVAPGLRHHHVQRYVATMVSNIDTVIDGWQPGQRLDIYQELRSAVRRST 128

CYP139A1_2575418829_Mycob 59 PVDGPTALIVSDGADHRRRRSVVAPGLRHHHVQRYVATMVSNIDTVIDGWQPGQRLDIYQELRSAVRRST 128

CYP139A1_2576172216_Mycob 59 PVDGPTALIVSDGADHRRRRSVVAPGLRHHHVQRYVATMVSNIDTVIDGWQPGQRLDIYQELRSAVRRST 128

CYP139A1_2576534922_Mycob 59 PVDGPTALIVSDGADHRRRRSVVAPGLRHHHVQRYVATMVSNIDTVIDGWQPGQRLDIYQELRSAVRRST 128

CYP139A1_2577185436_Mycob 59 PVDGPTALIVSDGADHRRRRSVVAPGLRHHHVQRYVATMVSNIDTVIDGWQPGQRLDIYQELRSAVRRST 128

CYP139A1_2577236838_Mycob 59 PVDGPTALIVSDGADHRRRRSVVAPGLRHHHVQRYVATMVSNIDTVIDGWQPGQRLDIYQELRSAVRRST 128

CYP139A1_2577373328_Mycob 59 PVDGPTALIVSDGADHRRRRSVVAPGLRHHHVQRYVATMVSNIDTVIDGWQPGQRLDIYQELRSAVRRST 128

CYP139A1_2578099005_Mycob 59 PVDGPTALIVSDGADHRRRRSVVAPGLRHHHVQRYVATMVSNIDTVIDGWQPGQRLDIYQELRSAVRRST 128

CYP139A1_2580366102_Mycob 59 PVDGPTALIVSDGADHRRRRSVVAPGLRHHHVQRYVATMVSNIDTVIDGWQPGQRLDIYQELRSAVRRST 128

CYP139A1_2580467795_Mycob 59 PVDGPTALIVSDGADHRRRRSVVAPGLRHHHVQRYVATMVSNIDTVIDGWQPGQRLDIYQELRSAVRRST 128

CYP139A1_2584613792_Mycob 59 PVDGPTALIVSDGADHRRRRSVVAPGLRHHHVQRYVATMVSNIDTVIDGWQPGQRLDIYQELRSAVRRST 128

CYP139A1_2584681396_Mycob 59 PVDGPTALIVSDGADHRRRRSVVAPGLRHHHVQRYVATMVSNIDTVIDGWQPGQRLDIYQELRSAVRRST 128

CYP139A1_2584913651_Mycob 59 PVDGPTALIVSDGADHRRRRSVVAPGLRHHHVQRYVATMVSNIDTVIDGWQPGQRLDIYQELRSAVRRST 128

CYP139A1_2584942354_Mycob 59 PVDGPTALIVSDGADHRRRRSVVAPGLRHHHVQRYVATMVSNIDTVIDGWQPGQRLDIYQELRSAVRRST 128

CYP139A1_2590093450_Mycob 59 PVDGPTALIVSDGADHRRRRSVVAPGLRHHHVQRYVATMVSNIDTVIDGWQPGQRLDIYQELRSAVRRST 128

CYP139A1_2590207004_Mycob 59 PVDGPTALIVSDGADHRRRRSVVAPGLRHHHVQRYVATMVSNIDTVIDGWQPGQRLDIYQELRSAVRRST 128

CYP139A1_2590276448_Mycob 59 PVDGPTALIVSDGADHRRRRSVVAPGLRHHHVQRYVATMVSNIDTVIDGWQPGQRLDIYQELRSAVRRST 128

CYP139A1_2575455196_Mycob 59 PVDGPTALIVSDGADHRRRRSVVAPGLRHHHVQRYVATMVSNIDTVIDGWQPGQRLDIYQELRSAVRRST 128

CYP139A1_2575920420_Mycob 59 PVDGPTALIVSDGADHRRRRSVVAPGLRHHHVQRYVATMVSNIDTVIDGWQPGQRLDIYQELRSAVRRST 128

CYP139A1_2575998368_Mycob 59 PVDGPTALIVSDGADHRRRRSVVAPGLRHHHVQRYVATMVSNIDTVIDGWQPGQRLDIYQELRSAVRRST 128

CYP139A1_2576583109_Mycob 59 PVDGPTALIVSDGADHRRRRSVVAPGLRHHHVQRYVATMVSNIDTVIDGWQPGQRLDIYQELRSAVRRST 128

CYP139A1_2576886343_Mycob 59 PVDGPTALIVSDGADHRRRRSVVAPGLRHHHVQRYVATMVSNIDTVIDGWQPGQRLDIYQELRSAVRRST 128

CYP139A1_2576986616_Mycob 59 PVDGPTALIVSDGADHRRRRSVVAPGLRHHHVQRYVATMVSNIDTVIDGWQPGQRLDIYQELRSAVRRST 128

CYP139A1_2577256179_Mycob 59 PVDGPTALIVSDGADHRRRRSVVAPGLRHHHVQRYVATMVSNIDTVIDGWQPGQRLDIYQELRSAVRRST 128

CYP139A1_2577876445_Mycob 59 PVDGPTALIVSDGADHRRRRSVVAPGLRHHHVQRYVATMVSNIDTVIDGWQPGQRLDIYQELRSAVRRST 128

CYP139A1_2579798813_Mycob 59 PVDGPTALIVSDGADHRRRRSVVAPGLRHHHVQRYVATMVSNIDTVIDGWQPGQRLDIYQELRSAVRRST 128

CYP139A1_2584715344_Mycob 59 PVDGPTALIVSDGADHRRRRSVVAPGLRHHHVQRYVATMVSNIDTVIDGWQPGQRLDIYQELRSAVRRST 128

CYP139A1_2588659602_Mycob 59 PVDGPTALIVSDGADHRRRRSVVAPGLRHHHVQRYVATMVSNIDTVIDGWQPGQRLDIYQELRSAVRRST 128

CYP139A1_2589089021_Mycob 59 PVDGPTALIVSDGADHRRRRSVVAPGLRHHHVQRYVATMVSNIDTVIDGWQPGQRLDIYQELRSAVRRST 128

CYP139A1_2589093094_Mycob 59 PVDGPTALIVSDGADHRRRRSVVAPGLRHHHVQRYVATMVSNIDTVIDGWQPGQRLDIYQELRSAVRRST 128

CYP139A1_2589535085_Mycob 59 PVDGPTALIVSDGADHRRRRSVVAPGLRHHHVQRYVATMVSNIDTVIDGWQPGQRLDIYQELRSAVRRST 128

CYP139A1_2589663797_Mycob 59 PVDGPTALIVSDGADHRRRRSVVAPGLRHHHVQRYVATMVSNIDTVIDGWQPGQRLDIYQELRSAVRRST 128

CYP139A1_2589671952_Mycob 59 PVDGPTALIVSDGADHRRRRSVVAPGLRHHHVQRYVATMVSNIDTVIDGWQPGQRLDIYQELRSAVRRST 128

CYP139A1_2589719807_Mycob 59 PVDGPTALIVSDGADHRRRRSVVAPGLRHHHVQRYVATMVSNIDTVIDGWQPGQRLDIYQELRSAVRRST 128

CYP139A1_2592246876_Mycob 59 PVDGPTALIVSDGADHRRRRSVVAPGLRHHHVQRYVATMVSNIDTVIDGWQPGQRLDIYQELRSAVRRST 128

CYP139A1_2592381635_Mycob 59 PVDGPTALIVSDGADHRRRRSVVAPGLRHHHVQRYVATMVSNIDTVIDGWQPGQRLDIYQELRSAVRRST 128

CYP139A1_2592410032_Mycob 59 PVDGPTALIVSDGADHRRRRSVVAPGLRHHHVQRYVATMVSNIDTVIDGWQPGQRLDIYQELRSAVRRST 128

CYP139A1_2592414205_Mycob 59 PVDGPTALIVSDGADHRRRRSVVAPGLRHHHVQRYVATMVSNIDTVIDGWQPGQRLDIYQELRSAVRRST 128

CYP139A1_2592566477_Mycob 59 PVDGPTALIVSDGADHRRRRSVVAPGLRHHHVQRYVATMVSNIDTVIDGWQPGQRLDIYQELRSAVRRST 128

CYP139A1_2575647889_Mycob 59 PVDGPTALIVSDGADHRRRRSVVAPGLRHHHVQRYVATMVSNIDTVIDGWQPGQRLDIYQELRSAVRRST 128

CYP139A1_2577429481_Mycob 59 PVDGPTALIVSDGADHRRRRSVVAPGLRHHHVQRYVATMVSNIDTVIDGWQPGQRLDIYQELRSAVRRST 128

CYP139A1_2578084434_Mycob 59 PVDGPTALIVSDGADHRRRRSVVAPGLRHHHVQRYVATMVSNIDTVIDGWQPGQRLDIYQELRSAVRRST 128

CYP139A1_2580301723_Mycob 59 PVDGPTALIVSDGADHRRRRSVVAPGLRHHHVQRYVATMVSNIDTVIDGWQPGQRLDIYQELRSAVRRST 128

CYP139A1_2581901399_Mycob 59 PVDGPTALIVSDGADHRRRRSVVAPGLRHHHVQRYVATMVSNIDTVIDGWQPGQRLDIYQELRSAVRRST 128

CYP139A1_2584689961_Mycob 59 PVDGPTALIVSDGADHRRRRSVVAPGLRHHHVQRYVATMVSNIDTVIDGWQPGQRLDIYQELRSAVRRST 128

CYP139A1_2584748012_Mycob 59 PVDGPTALIVSDGADHRRRRSVVAPGLRHHHVQRYVATMVSNIDTVIDGWQPGQRLDIYQELRSAVRRST 128

CYP139A1_2584878425_Mycob 59 PVDGPTALIVSDGADHRRRRSVVAPGLRHHHVQRYVATMVSNIDTVIDGWQPGQRLDIYQELRSAVRRST 128

CYP139A1_2584990570_Mycob 59 PVDGPTALIVSDGADHRRRRSVVAPGLRHHHVQRYVATMVSNIDTVIDGWQPGQRLDIYQELRSAVRRST 128

CYP139A1_2590014899_Mycob 59 PVDGPTALIVSDGADHRRRRSVVAPGLRHHHVQRYVATMVSNIDTVIDGWQPGQRLDIYQELRSAVRRST 128

CYP139A1_2590313607_Mycob 59 PVDGPTALIVSDGADHRRRRSVVAPGLRHHHVQRYVATMVSNIDTVIDGWQPGQRLDIYQELRSAVRRST 128

CYP139A1_2590563570_Mycob 59 PVDGPTALIVSDGADHRRRRSVVAPGLRHHHVQRYVATMVSNIDTVIDGWQPGQRLDIYQELRSAVRRST 128

CYP139A1_2574682482_Mycob 59 PVDGPTALIVSDGADHRRRRSVVAPGLRHHHVQRYVATMVSNIDTVIDGWQPGQRLDIYQELRSAVRRST 128

CYP139A1_2574937709_Mycob 59 PVDGPTALIVSDGADHRRRRSVVAPGLRHHHVQRYVATMVSNIDTVIDGWQPGQRLDIYQELRSAVRRST 128

CYP139A1_2575051511_Mycob 59 PVDGPTALIVSDGADHRRRRSVVAPGLRHHHVQRYVATMVSNIDTVIDGWQPGQRLDIYQELRSAVRRST 128

CYP139A1_2575142685_Mycob 59 PVDGPTALIVSDGADHRRRRSVVAPGLRHHHVQRYVATMVSNIDTVIDGWQPGQRLDIYQELRSAVRRST 128

CYP139A1_2575538950_Mycob 59 PVDGPTALIVSDGADHRRRRSVVAPGLRHHHVQRYVATMVSNIDTVIDGWQPGQRLDIYQELRSAVRRST 128

CYP139A1_2575858812_Mycob 59 PVDGPTALIVSDGADHRRRRSVVAPGLRHHHVQRYVATMVSNIDTVIDGWQPGQRLDIYQELRSAVRRST 128

CYP139A1_2576059958_Mycob 59 PVDGPTALIVSDGADHRRRRSVVAPGLRHHHVQRYVATMVSNIDTVIDGWQPGQRLDIYQELRSAVRRST 128

CYP139A1_2576471218_Mycob 59 PVDGPTALIVSDGADHRRRRSVVAPGLRHHHVQRYVATMVSNIDTVIDGWQPGQRLDIYQELRSAVRRST 128

CYP139A1_2576497080_Mycob 59 PVDGPTALIVSDGADHRRRRSVVAPGLRHHHVQRYVATMVSNIDTVIDGWQPGQRLDIYQELRSAVRRST 128

CYP139A1_2577038023_Mycob 59 PVDGPTALIVSDGADHRRRRSVVAPGLRHHHVQRYVATMVSNIDTVIDGWQPGQRLDIYQELRSAVRRST 128

CYP139A1_2577203844_Mycob 59 PVDGPTALIVSDGADHRRRRSVVAPGLRHHHVQRYVATMVSNIDTVIDGWQPGQRLDIYQELRSAVRRST 128

CYP139A1_2577322951_Mycob 59 PVDGPTALIVSDGADHRRRRSVVAPGLRHHHVQRYVATMVSNIDTVIDGWQPGQRLDIYQELRSAVRRST 128

CYP139A1_2577422627_Mycob 59 PVDGPTALIVSDGADHRRRRSVVAPGLRHHHVQRYVATMVSNIDTVIDGWQPGQRLDIYQELRSAVRRST 128

CYP139A1_2577551091_Mycob 59 PVDGPTALIVSDGADHRRRRSVVAPGLRHHHVQRYVATMVSNIDTVIDGWQPGQRLDIYQELRSAVRRST 128

CYP139A1_2584755863_Mycob 59 PVDGPTALIVSDGADHRRRRSVVAPGLRHHHVQRYVATMVSNIDTVIDGWQPGQRLDIYQELRSAVRRST 128

CYP139A1_2589117553_Mycob 59 PVDGPTALIVSDGADHRRRRSVVAPGLRHHHVQRYVATMVSNIDTVIDGWQPGQRLDIYQELRSAVRRST 128

CYP139A1_2589121735_Mycob 59 PVDGPTALIVSDGADHRRRRSVVAPGLRHHHVQRYVATMVSNIDTVIDGWQPGQRLDIYQELRSAVRRST 128

CYP139A1_2589506393_Mycob 59 PVDGPTALIVSDGADHRRRRSVVAPGLRHHHVQRYVATMVSNIDTVIDGWQPGQRLDIYQELRSAVRRST 128

CYP139A1_2590044637_Mycob 59 PVDGPTALIVSDGADHRRRRSVVAPGLRHHHVQRYVATMVSNIDTVIDGWQPGQRLDIYQELRSAVRRST 128

CYP139A1_2590137275_Mycob 59 PVDGPTALIVSDGADHRRRRSVVAPGLRHHHVQRYVATMVSNIDTVIDGWQPGQRLDIYQELRSAVRRST 128

CYP139A1_2590345757_Mycob 59 PVDGPTALIVSDGADHRRRRSVVAPGLRHHHVQRYVATMVSNIDTVIDGWQPGQRLDIYQELRSAVRRST 128

CYP139A1_2592275464_Mycob 59 PVDGPTALIVSDGADHRRRRSVVAPGLRHHHVQRYVATMVSNIDTVIDGWQPGQRLDIYQELRSAVRRST 128

CYP139A1_2575515927_Mycob 59 PVDGPTALIVSDGADHRRRRSVVAPGLRHHHVQRYVATMVSNIDTVIDGWQPGQRLDIYQELRSAVRRST 128

CYP139A1_2576459372_Mycob 59 PVDGPTALIVSDGADHRRRRSVVAPGLRHHHVQRYVATMVSNIDTVIDGWQPGQRLDIYQELRSAVRRST 128

CYP139A1_2576522568_Mycob 59 PVDGPTALIVSDGADHRRRRSVVAPGLRHHHVQRYVATMVSNIDTVIDGWQPGQRLDIYQELRSAVRRST 128

CYP139A1_2576559538_Mycob 59 PVDGPTALIVSDGADHRRRRSVVAPGLRHHHVQRYVATMVSNIDTVIDGWQPGQRLDIYQELRSAVRRST 128

CYP139A1_2577302440_Mycob 59 PVDGPTALIVSDGADHRRRRSVVAPGLRHHHVQRYVATMVSNIDTVIDGWQPGQRLDIYQELRSAVRRST 128

CYP139A1_2577448440_Mycob 59 PVDGPTALIVSDGADHRRRRSVVAPGLRHHHVQRYVATMVSNIDTVIDGWQPGQRLDIYQELRSAVRRST 128

CYP139A1_2577462548_Mycob 59 PVDGPTALIVSDGADHRRRRSVVAPGLRHHHVQRYVATMVSNIDTVIDGWQPGQRLDIYQELRSAVRRST 128

CYP139A1_2584678567_Mycob 59 PVDGPTALIVSDGADHRRRRSVVAPGLRHHHVQRYVATMVSNIDTVIDGWQPGQRLDIYQELRSAVRRST 128

CYP139A1_2584763258_Mycob 59 PVDGPTALIVSDGADHRRRRSVVAPGLRHHHVQRYVATMVSNIDTVIDGWQPGQRLDIYQELRSAVRRST 128

CYP139A1_2584949783_Mycob 59 PVDGPTALIVSDGADHRRRRSVVAPGLRHHHVQRYVATMVSNIDTVIDGWQPGQRLDIYQELRSAVRRST 128

CYP139A1_2589146274_Mycob 59 PVDGPTALIVSDGADHRRRRSVVAPGLRHHHVQRYVATMVSNIDTVIDGWQPGQRLDIYQELRSAVRRST 128

CYP139A1_2590089378_Mycob 59 PVDGPTALIVSDGADHRRRRSVVAPGLRHHHVQRYVATMVSNIDTVIDGWQPGQRLDIYQELRSAVRRST 128

CYP139A1_2590288661_Mycob 59 PVDGPTALIVSDGADHRRRRSVVAPGLRHHHVQRYVATMVSNIDTVIDGWQPGQRLDIYQELRSAVRRST 128

CYP139A1_2590513765_Mycob 59 PVDGPTALIVSDGADHRRRRSVVAPGLRHHHVQRYVATMVSNIDTVIDGWQPGQRLDIYQELRSAVRRST 128

CYP139A1_643045086_Mycoba 59 PVDGPTALIVSDGADHRRRRSVVAPGLRHHHVQRYVATMVSNIDTVIDGWQPGQRLDIYQELRSAVRRST 128

CYP139A1_646014426_Mycoba 59 PVDGPTALIVSDGADHRRRRSVVAPGLRHHHVQRYVATMVSNIDTVIDGWQPGQRLDIYQELRSAVRRST 128

CYP139A1_2574985446_Mycob 59 PVDGPTALIVSDGADHRRRRSVVAPGLRHHHVQRYVATMVSNIDTVIDGWQPGQRLDIYQELRSAVRRST 128

CYP139A1_2575103505_Mycob 59 PVDGPTALIVSDGADHRRRRSVVAPGLRHHHVQRYVATMVSNIDTVIDGWQPGQRLDIYQELRSAVRRST 128

CYP139A1_2575806159_Mycob 59 PVDGPTALIVSDGADHRRRRSVVAPGLRHHHVQRYVATMVSNIDTVIDGWQPGQRLDIYQELRSAVRRST 128

CYP139A1_2576156796_Mycob 59 PVDGPTALIVSDGADHRRRRSVVAPGLRHHHVQRYVATMVSNIDTVIDGWQPGQRLDIYQELRSAVRRST 128

CYP139A1_2576936333_Mycob 59 PVDGPTALIVSDGADHRRRRSVVAPGLRHHHVQRYVATMVSNIDTVIDGWQPGQRLDIYQELRSAVRRST 128

CYP139A1_2577030827_Mycob 59 PVDGPTALIVSDGADHRRRRSVVAPGLRHHHVQRYVATMVSNIDTVIDGWQPGQRLDIYQELRSAVRRST 128

CYP139A1_2577381729_Mycob 59 PVDGPTALIVSDGADHRRRRSVVAPGLRHHHVQRYVATMVSNIDTVIDGWQPGQRLDIYQELRSAVRRST 128

CYP139A1_2584698776_Mycob 59 PVDGPTALIVSDGADHRRRRSVVAPGLRHHHVQRYVATMVSNIDTVIDGWQPGQRLDIYQELRSAVRRST 128

CYP139A1_2584825730_Mycob 59 PVDGPTALIVSDGADHRRRRSVVAPGLRHHHVQRYVATMVSNIDTVIDGWQPGQRLDIYQELRSAVRRST 128

CYP139A1_2584885951_Mycob 59 PVDGPTALIVSDGADHRRRRSVVAPGLRHHHVQRYVATMVSNIDTVIDGWQPGQRLDIYQELRSAVRRST 128

CYP139A1_2584902930_Mycob 59 PVDGPTALIVSDGADHRRRRSVVAPGLRHHHVQRYVATMVSNIDTVIDGWQPGQRLDIYQELRSAVRRST 128

CYP139A1_2588995218_Mycob 59 PVDGPTALIVSDGADHRRRRSVVAPGLRHHHVQRYVATMVSNIDTVIDGWQPGQRLDIYQELRSAVRRST 128

CYP139A1_2589139119_Mycob 59 PVDGPTALIVSDGADHRRRRSVVAPGLRHHHVQRYVATMVSNIDTVIDGWQPGQRLDIYQELRSAVRRST 128

CYP139A1_2592238712_Mycob 59 PVDGPTALIVSDGADHRRRRSVVAPGLRHHHVQRYVATMVSNIDTVIDGWQPGQRLDIYQELRSAVRRST 128

CYP139A1_2592261699_Mycob 59 PVDGPTALIVSDGADHRRRRSVVAPGLRHHHVQRYVATMVSNIDTVIDGWQPGQRLDIYQELRSAVRRST 128

CYP139A1_2592393538_Mycob 59 PVDGPTALIVSDGADHRRRRSVVAPGLRHHHVQRYVATMVSNIDTVIDGWQPGQRLDIYQELRSAVRRST 128

CYP139A1_2592397895_Mycob 59 PVDGPTALIVSDGADHRRRRSVVAPGLRHHHVQRYVATMVSNIDTVIDGWQPGQRLDIYQELRSAVRRST 128

CYP139A1_2592549349_Mycob 59 PVDGPTALIVSDGADHRRRRSVVAPGLRHHHVQRYVATMVSNIDTVIDGWQPGQRLDIYQELRSAVRRST 128

CYP139A1_648464907_Mycoba 59 PVDGPTALIVSDGADHRRRRSVVAPGLRHHHVQRYVATMVSNIDTVIDGWQPGQRLDIYQELRSAVRRST 128

CYP139A1_648481186_Mycoba 59 PVDGPTALIVSDGADHRRRRSVVAPGLRHHHVQRYVATMVSNIDTVIDGWQPGQRLDIYQELRSAVRRST 128

CYP139A1_2574586310_Mycob 59 PVDGPTALIVSDGADHRRRRSVVAPGLRHHHVQRYVATMVSNIDTVIDGWQPGQRLDIYQELRSAVRRST 128

CYP139A1_2574663269_Mycob 59 PVDGPTALIVSDGADHRRRRSVVAPGLRHHHVQRYVATMVSNIDTVIDGWQPGQRLDIYQELRSAVRRST 128

CYP139A1_2574880930_Mycob 59 PVDGPTALIVSDGADHRRRRSVVAPGLRHHHVQRYVATMVSNIDTVIDGWQPGQRLDIYQELRSAVRRST 128

CYP139A1_2574911407_Mycob 59 PVDGPTALIVSDGADHRRRRSVVAPGLRHHHVQRYVATMVSNIDTVIDGWQPGQRLDIYQELRSAVRRST 128

CYP139A1_2575345775_Mycob 59 PVDGPTALIVSDGADHRRRRSVVAPGLRHHHVQRYVATMVSNIDTVIDGWQPGQRLDIYQELRSAVRRST 128

CYP139A1_2575468742_Mycob 59 PVDGPTALIVSDGADHRRRRSVVAPGLRHHHVQRYVATMVSNIDTVIDGWQPGQRLDIYQELRSAVRRST 128

CYP139A1_2576199760_Mycob 59 PVDGPTALIVSDGADHRRRRSVVAPGLRHHHVQRYVATMVSNIDTVIDGWQPGQRLDIYQELRSAVRRST 128

CYP139A1_2577069926_Mycob 59 PVDGPTALIVSDGADHRRRRSVVAPGLRHHHVQRYVATMVSNIDTVIDGWQPGQRLDIYQELRSAVRRST 128

CYP139A1_2577613318_Mycob 59 PVDGPTALIVSDGADHRRRRSVVAPGLRHHHVQRYVATMVSNIDTVIDGWQPGQRLDIYQELRSAVRRST 128

CYP139A1_2577684900_Mycob 59 PVDGPTALIVSDGADHRRRRSVVAPGLRHHHVQRYVATMVSNIDTVIDGWQPGQRLDIYQELRSAVRRST 128

CYP139A1_2582415442_Mycob 59 PVDGPTALIVSDGADHRRRRSVVAPGLRHHHVQRYVATMVSNIDTVIDGWQPGQRLDIYQELRSAVRRST 128

CYP139A1_2584812089_Mycob 59 PVDGPTALIVSDGADHRRRRSVVAPGLRHHHVQRYVATMVSNIDTVIDGWQPGQRLDIYQELRSAVRRST 128

CYP139A1_2590125975_Mycob 59 PVDGPTALIVSDGADHRRRRSVVAPGLRHHHVQRYVATMVSNIDTVIDGWQPGQRLDIYQELRSAVRRST 128

CYP139A1_2590219901_Mycob 59 PVDGPTALIVSDGADHRRRRSVVAPGLRHHHVQRYVATMVSNIDTVIDGWQPGQRLDIYQELRSAVRRST 128

CYP139A1_2590227570_Mycob 59 PVDGPTALIVSDGADHRRRRSVVAPGLRHHHVQRYVATMVSNIDTVIDGWQPGQRLDIYQELRSAVRRST 128

CYP139A1_2590317674_Mycob 59 PVDGPTALIVSDGADHRRRRSVVAPGLRHHHVQRYVATMVSNIDTVIDGWQPGQRLDIYQELRSAVRRST 128

CYP139A1_641814886_Mycoba 59 PVDGPTALIVSDGADHRRRRSVVAPGLRHHHVQRYVATMVSNIDTVIDGWQPGQRLDIYQELRSAVRRST 128

CYP139A1_2574738070_Mycob 59 PVDGPTALIVSDGADHRRRRSVVAPGLRHHHVQRYVATMVSNIDTVIDGWQPGQRLDIYQELRSAVRRST 128

CYP139A1_2575365848_Mycob 59 PVDGPTALIVSDGADHRRRRSVVAPGLRHHHVQRYVATMVSNIDTVIDGWQPGQRLDIYQELRSAVRRST 128

CYP139A1_2575674521_Mycob 59 PVDGPTALIVSDGADHRRRRSVVAPGLRHHHVQRYVATMVSNIDTVIDGWQPGQRLDIYQELRSAVRRST 128

CYP139A1_2575772716_Mycob 59 PVDGPTALIVSDGADHRRRRSVVAPGLRHHHVQRYVATMVSNIDTVIDGWQPGQRLDIYQELRSAVRRST 128

CYP139A1_2576544659_Mycob 59 PVDGPTALIVSDGADHRRRRSVVAPGLRHHHVQRYVATMVSNIDTVIDGWQPGQRLDIYQELRSAVRRST 128

CYP139A1_2577168470_Mycob 59 PVDGPTALIVSDGADHRRRRSVVAPGLRHHHVQRYVATMVSNIDTVIDGWQPGQRLDIYQELRSAVRRST 128

CYP139A1_2577390930_Mycob 59 PVDGPTALIVSDGADHRRRRSVVAPGLRHHHVQRYVATMVSNIDTVIDGWQPGQRLDIYQELRSAVRRST 128

CYP139A1_2577651582_Mycob 59 PVDGPTALIVSDGADHRRRRSVVAPGLRHHHVQRYVATMVSNIDTVIDGWQPGQRLDIYQELRSAVRRST 128

CYP139A1_2578002537_Mycob 59 PVDGPTALIVSDGADHRRRRSVVAPGLRHHHVQRYVATMVSNIDTVIDGWQPGQRLDIYQELRSAVRRST 128

CYP139A1_2578230674_Mycob 59 PVDGPTALIVSDGADHRRRRSVVAPGLRHHHVQRYVATMVSNIDTVIDGWQPGQRLDIYQELRSAVRRST 128

CYP139A1_2579825786_Mycob 59 PVDGPTALIVSDGADHRRRRSVVAPGLRHHHVQRYVATMVSNIDTVIDGWQPGQRLDIYQELRSAVRRST 128

CYP139A1_2584923296_Mycob 59 PVDGPTALIVSDGADHRRRRSVVAPGLRHHHVQRYVATMVSNIDTVIDGWQPGQRLDIYQELRSAVRRST 128

CYP139A1_2584954771_Mycob 59 PVDGPTALIVSDGADHRRRRSVVAPGLRHHHVQRYVATMVSNIDTVIDGWQPGQRLDIYQELRSAVRRST 128

CYP139A1_2588999278_Mycob 59 PVDGPTALIVSDGADHRRRRSVVAPGLRHHHVQRYVATMVSNIDTVIDGWQPGQRLDIYQELRSAVRRST 128

CYP139A1_2589044237_Mycob 59 PVDGPTALIVSDGADHRRRRSVVAPGLRHHHVQRYVATMVSNIDTVIDGWQPGQRLDIYQELRSAVRRST 128

CYP139A1_2589555384_Mycob 59 PVDGPTALIVSDGADHRRRRSVVAPGLRHHHVQRYVATMVSNIDTVIDGWQPGQRLDIYQELRSAVRRST 128

CYP139A1_2589568736_Mycob 59 PVDGPTALIVSDGADHRRRRSVVAPGLRHHHVQRYVATMVSNIDTVIDGWQPGQRLDIYQELRSAVRRST 128

CYP139A1_2589715710_Mycob 59 PVDGPTALIVSDGADHRRRRSVVAPGLRHHHVQRYVATMVSNIDTVIDGWQPGQRLDIYQELRSAVRRST 128

CYP139A1_2592287696_Mycob 59 PVDGPTALIVSDGADHRRRRSVVAPGLRHHHVQRYVATMVSNIDTVIDGWQPGQRLDIYQELRSAVRRST 128

CYP139A1_2592291479_Mycob 59 PVDGPTALIVSDGADHRRRRSVVAPGLRHHHVQRYVATMVSNIDTVIDGWQPGQRLDIYQELRSAVRRST 128

CYP139A1_2592430392_Mycob 59 PVDGPTALIVSDGADHRRRRSVVAPGLRHHHVQRYVATMVSNIDTVIDGWQPGQRLDIYQELRSAVRRST 128

CYP139A1_2574790496_Mycob 59 PVDGPTALIVSDGADHRRRRSVVAPGLRHHHVQRYVATMVSNIDTVIDGWQPGQRLDIYQELRSAVRRST 128

CYP139A1_2574834868_Mycob 59 PVDGPTALIVSDGADHRRRRSVVAPGLRHHHVQRYVATMVSNIDTVIDGWQPGQRLDIYQELRSAVRRST 128

CYP139A1_2576151818_Mycob 59 PVDGPTALIVSDGADHRRRRSVVAPGLRHHHVQRYVATMVSNIDTVIDGWQPGQRLDIYQELRSAVRRST 128

CYP139A1_2578111269_Mycob 59 PVDGPTALIVSDGADHRRRRSVVAPGLRHHHVQRYVATMVSNIDTVIDGWQPGQRLDIYQELRSAVRRST 128

CYP139A1_2583723841_Mycob 59 PVDGPTALIVSDGADHRRRRSVVAPGLRHHHVQRYVATMVSNIDTVIDGWQPGQRLDIYQELRSAVRRST 128

CYP139A1_2584631822_Mycob 59 PVDGPTALIVSDGADHRRRRSVVAPGLRHHHVQRYVATMVSNIDTVIDGWQPGQRLDIYQELRSAVRRST 128

CYP139A1_2584721998_Mycob 59 PVDGPTALIVSDGADHRRRRSVVAPGLRHHHVQRYVATMVSNIDTVIDGWQPGQRLDIYQELRSAVRRST 128

CYP139A1_2584846770_Mycob 59 PVDGPTALIVSDGADHRRRRSVVAPGLRHHHVQRYVATMVSNIDTVIDGWQPGQRLDIYQELRSAVRRST 128

CYP139A1_2588591267_Mycob 59 PVDGPTALIVSDGADHRRRRSVVAPGLRHHHVQRYVATMVSNIDTVIDGWQPGQRLDIYQELRSAVRRST 128

CYP139A1_2590007914_Mycob 59 PVDGPTALIVSDGADHRRRRSVVAPGLRHHHVQRYVATMVSNIDTVIDGWQPGQRLDIYQELRSAVRRST 128

CYP139A1_2590057066_Mycob 59 PVDGPTALIVSDGADHRRRRSVVAPGLRHHHVQRYVATMVSNIDTVIDGWQPGQRLDIYQELRSAVRRST 128

CYP139A1_2590061112_Mycob 59 PVDGPTALIVSDGADHRRRRSVVAPGLRHHHVQRYVATMVSNIDTVIDGWQPGQRLDIYQELRSAVRRST 128

CYP139A1_2590252317_Mycob 59 PVDGPTALIVSDGADHRRRRSVVAPGLRHHHVQRYVATMVSNIDTVIDGWQPGQRLDIYQELRSAVRRST 128

CYP139A1_2590543931_Mycob 59 PVDGPTALIVSDGADHRRRRSVVAPGLRHHHVQRYVATMVSNIDTVIDGWQPGQRLDIYQELRSAVRRST 128

CYP139A1_2574560432_Mycob 59 PVDGPTALIVSDGADHRRRRSVVAPGLRHHHVQRYVATMVSNIDTVIDGWQPGQRLDIYQELRSAVRRST 128

CYP139A1_2575495357_Mycob 59 PVDGPTALIVSDGADHRRRRSVVAPGLRHHHVQRYVATMVSNIDTVIDGWQPGQRLDIYQELRSAVRRST 128

CYP139A1_2575638157_Mycob 59 PVDGPTALIVSDGADHRRRRSVVAPGLRHHHVQRYVATMVSNIDTVIDGWQPGQRLDIYQELRSAVRRST 128

CYP139A1_2575697737_Mycob 59 PVDGPTALIVSDGADHRRRRSVVAPGLRHHHVQRYVATMVSNIDTVIDGWQPGQRLDIYQELRSAVRRST 128

CYP139A1_2575988382_Mycob 59 PVDGPTALIVSDGADHRRRRSVVAPGLRHHHVQRYVATMVSNIDTVIDGWQPGQRLDIYQELRSAVRRST 128

CYP139A1_2576070324_Mycob 59 PVDGPTALIVSDGADHRRRRSVVAPGLRHHHVQRYVATMVSNIDTVIDGWQPGQRLDIYQELRSAVRRST 128

CYP139A1_2576613004_Mycob 59 PVDGPTALIVSDGADHRRRRSVVAPGLRHHHVQRYVATMVSNIDTVIDGWQPGQRLDIYQELRSAVRRST 128

CYP139A1_2577091164_Mycob 59 PVDGPTALIVSDGADHRRRRSVVAPGLRHHHVQRYVATMVSNIDTVIDGWQPGQRLDIYQELRSAVRRST 128

CYP139A1_2577569748_Mycob 59 PVDGPTALIVSDGADHRRRRSVVAPGLRHHHVQRYVATMVSNIDTVIDGWQPGQRLDIYQELRSAVRRST 128

CYP139A1_2577697067_Mycob 59 PVDGPTALIVSDGADHRRRRSVVAPGLRHHHVQRYVATMVSNIDTVIDGWQPGQRLDIYQELRSAVRRST 128

CYP139A1_2584795634_Mycob 59 PVDGPTALIVSDGADHRRRRSVVAPGLRHHHVQRYVATMVSNIDTVIDGWQPGQRLDIYQELRSAVRRST 128

CYP139A1_2589158600_Mycob 59 PVDGPTALIVSDGADHRRRRSVVAPGLRHHHVQRYVATMVSNIDTVIDGWQPGQRLDIYQELRSAVRRST 128

CYP139A1_2589491285_Mycob 59 PVDGPTALIVSDGADHRRRRSVVAPGLRHHHVQRYVATMVSNIDTVIDGWQPGQRLDIYQELRSAVRRST 128

CYP139A1_2589613612_Mycob 59 PVDGPTALIVSDGADHRRRRSVVAPGLRHHHVQRYVATMVSNIDTVIDGWQPGQRLDIYQELRSAVRRST 128

CYP139A1_2589650472_Mycob 59 PVDGPTALIVSDGADHRRRRSVVAPGLRHHHVQRYVATMVSNIDTVIDGWQPGQRLDIYQELRSAVRRST 128

CYP139A1_2589699410_Mycob 59 PVDGPTALIVSDGADHRRRRSVVAPGLRHHHVQRYVATMVSNIDTVIDGWQPGQRLDIYQELRSAVRRST 128

CYP139A1_2590366449_Mycob 59 PVDGPTALIVSDGADHRRRRSVVAPGLRHHHVQRYVATMVSNIDTVIDGWQPGQRLDIYQELRSAVRRST 128

CYP139A1_2592226489_Mycob 59 PVDGPTALIVSDGADHRRRRSVVAPGLRHHHVQRYVATMVSNIDTVIDGWQPGQRLDIYQELRSAVRRST 128

CYP139A1_2592574645_Mycob 59 PVDGPTALIVSDGADHRRRRSVVAPGLRHHHVQRYVATMVSNIDTVIDGWQPGQRLDIYQELRSAVRRST 128

CYP139A1_648473432_Mycoba 59 PVDGPTALIVSDGADHRRRRSVVAPGLRHHHVQRYVATMVSNIDTVIDGWQPGQRLDIYQELRSAVRRST 128

CYP139A1_2574703562_Mycob 59 PVDGPTALIVSDGADHRRRRSVVAPGLRHHHVQRYVATMVSNIDTVIDGWQPGQRLDIYQELRSAVRRST 128

CYP139A1_2575794202_Mycob 59 PVDGPTALIVSDGADHRRRRSVVAPGLRHHHVQRYVATMVSNIDTVIDGWQPGQRLDIYQELRSAVRRST 128

CYP139A1_2576967330_Mycob 59 PVDGPTALIVSDGADHRRRRSVVAPGLRHHHVQRYVATMVSNIDTVIDGWQPGQRLDIYQELRSAVRRST 128

CYP139A1_2577659800_Mycob 59 PVDGPTALIVSDGADHRRRRSVVAPGLRHHHVQRYVATMVSNIDTVIDGWQPGQRLDIYQELRSAVRRST 128

CYP139A1_2577918576_Mycob 59 PVDGPTALIVSDGADHRRRRSVVAPGLRHHHVQRYVATMVSNIDTVIDGWQPGQRLDIYQELRSAVRRST 128

CYP139A1_2578033540_Mycob 59 PVDGPTALIVSDGADHRRRRSVVAPGLRHHHVQRYVATMVSNIDTVIDGWQPGQRLDIYQELRSAVRRST 128

CYP139A1_2581869776_Mycob 59 PVDGPTALIVSDGADHRRRRSVVAPGLRHHHVQRYVATMVSNIDTVIDGWQPGQRLDIYQELRSAVRRST 128

CYP139A1_2583745288_Mycob 59 PVDGPTALIVSDGADHRRRRSVVAPGLRHHHVQRYVATMVSNIDTVIDGWQPGQRLDIYQELRSAVRRST 128

CYP139A1_2590086658_Mycob 59 PVDGPTALIVSDGADHRRRRSVVAPGLRHHHVQRYVATMVSNIDTVIDGWQPGQRLDIYQELRSAVRRST 128

CYP139A1_2590104693_Mycob 59 PVDGPTALIVSDGADHRRRRSVVAPGLRHHHVQRYVATMVSNIDTVIDGWQPGQRLDIYQELRSAVRRST 128

CYP139A1_2590187116_Mycob 59 PVDGPTALIVSDGADHRRRRSVVAPGLRHHHVQRYVATMVSNIDTVIDGWQPGQRLDIYQELRSAVRRST 128

CYP139A1_2590382529_Mycob 59 PVDGPTALIVSDGADHRRRRSVVAPGLRHHHVQRYVATMVSNIDTVIDGWQPGQRLDIYQELRSAVRRST 128

CYP139A1_2590510255_Mycob 59 PVDGPTALIVSDGADHRRRRSVVAPGLRHHHVQRYVATMVSNIDTVIDGWQPGQRLDIYQELRSAVRRST 128

CYP139A1_2574635031_Mycob 59 PVDGPTALIVSDGADHRRRRSVVAPGLRHHHVQRYVATMVSNIDTVIDGWQPGQRLDIYQELRSAVRRST 128

CYP139A1_2576207497_Mycob 59 PVDGPTALIVSDGADHRRRRSVVAPGLRHHHVQRYVATMVSNIDTVIDGWQPGQRLDIYQELRSAVRRST 128

CYP139A1_2576443603_Mycob 59 PVDGPTALIVSDGADHRRRRSVVAPGLRHHHVQRYVATMVSNIDTVIDGWQPGQRLDIYQELRSAVRRST 128

CYP139A1_2577241442_Mycob 59 PVDGPTALIVSDGADHRRRRSVVAPGLRHHHVQRYVATMVSNIDTVIDGWQPGQRLDIYQELRSAVRRST 128

CYP139A1_2578177805_Mycob 59 PVDGPTALIVSDGADHRRRRSVVAPGLRHHHVQRYVATMVSNIDTVIDGWQPGQRLDIYQELRSAVRRST 128

CYP139A1_2578195506_Mycob 59 PVDGPTALIVSDGADHRRRRSVVAPGLRHHHVQRYVATMVSNIDTVIDGWQPGQRLDIYQELRSAVRRST 128

CYP139A1_2578246558_Mycob 59 PVDGPTALIVSDGADHRRRRSVVAPGLRHHHVQRYVATMVSNIDTVIDGWQPGQRLDIYQELRSAVRRST 128

CYP139A1_2584652546_Mycob 59 PVDGPTALIVSDGADHRRRRSVVAPGLRHHHVQRYVATMVSNIDTVIDGWQPGQRLDIYQELRSAVRRST 128

CYP139A1_2584737038_Mycob 59 PVDGPTALIVSDGADHRRRRSVVAPGLRHHHVQRYVATMVSNIDTVIDGWQPGQRLDIYQELRSAVRRST 128

CYP139A1_2584918375_Mycob 59 PVDGPTALIVSDGADHRRRRSVVAPGLRHHHVQRYVATMVSNIDTVIDGWQPGQRLDIYQELRSAVRRST 128

CYP139A1_2584974532_Mycob 59 PVDGPTALIVSDGADHRRRRSVVAPGLRHHHVQRYVATMVSNIDTVIDGWQPGQRLDIYQELRSAVRRST 128

CYP139A1_2588987052_Mycob 59 PVDGPTALIVSDGADHRRRRSVVAPGLRHHHVQRYVATMVSNIDTVIDGWQPGQRLDIYQELRSAVRRST 128

CYP139A1_2589098383_Mycob 59 PVDGPTALIVSDGADHRRRRSVVAPGLRHHHVQRYVATMVSNIDTVIDGWQPGQRLDIYQELRSAVRRST 128

CYP139A1_2589486069_Mycob 59 PVDGPTALIVSDGADHRRRRSVVAPGLRHHHVQRYVATMVSNIDTVIDGWQPGQRLDIYQELRSAVRRST 128

CYP139A1_2589522796_Mycob 59 PVDGPTALIVSDGADHRRRRSVVAPGLRHHHVQRYVATMVSNIDTVIDGWQPGQRLDIYQELRSAVRRST 128

CYP139A1_2589616566_Mycob 59 PVDGPTALIVSDGADHRRRRSVVAPGLRHHHVQRYVATMVSNIDTVIDGWQPGQRLDIYQELRSAVRRST 128

CYP139A1_2592250930_Mycob 59 PVDGPTALIVSDGADHRRRRSVVAPGLRHHHVQRYVATMVSNIDTVIDGWQPGQRLDIYQELRSAVRRST 128

CYP139A1_2592303254_Mycob 59 PVDGPTALIVSDGADHRRRRSVVAPGLRHHHVQRYVATMVSNIDTVIDGWQPGQRLDIYQELRSAVRRST 128

CYP139A1_2592308116_Mycob 59 PVDGPTALIVSDGADHRRRRSVVAPGLRHHHVQRYVATMVSNIDTVIDGWQPGQRLDIYQELRSAVRRST 128

CYP139A1_2592385592_Mycob 59 PVDGPTALIVSDGADHRRRRSVVAPGLRHHHVQRYVATMVSNIDTVIDGWQPGQRLDIYQELRSAVRRST 128

CYP139A1_2592417392_Mycob 59 PVDGPTALIVSDGADHRRRRSVVAPGLRHHHVQRYVATMVSNIDTVIDGWQPGQRLDIYQELRSAVRRST 128

CYP139A1_643049582_Mycoba 59 PVDGPTALIVSDGADHRRRRSVVAPGLRHHHVQRYVATMVSNIDTVIDGWQPGQRLDIYQELRSAVRRST 128

CYP139A1_648460473_Mycoba 59 PVDGPTALIVSDGADHRRRRSVVAPGLRHHHVQRYVATMVSNIDTVIDGWQPGQRLDIYQELRSAVRRST 128

CYP139A1_2574901332_Mycob 59 PVDGPTALIVSDGADHRRRRSVVAPGLRHHHVQRYVATMVSNIDTVIDGWQPGQRLDIYQELRSAVRRST 128

CYP139A1_2575325434_Mycob 59 PVDGPTALIVSDGADHRRRRSVVAPGLRHHHVQRYVATMVSNIDTVIDGWQPGQRLDIYQELRSAVRRST 128

CYP139A1_2576274603_Mycob 59 PVDGPTALIVSDGADHRRRRSVVAPGLRHHHVQRYVATMVSNIDTVIDGWQPGQRLDIYQELRSAVRRST 128

CYP139A1_2583719805_Mycob 59 PVDGPTALIVSDGADHRRRRSVVAPGLRHHHVQRYVATMVSNIDTVIDGWQPGQRLDIYQELRSAVRRST 128

CYP139A1_2590012031_Mycob 59 PVDGPTALIVSDGADHRRRRSVVAPGLRHHHVQRYVATMVSNIDTVIDGWQPGQRLDIYQELRSAVRRST 128

CYP139A_2590028529_Mycoba 59 PVDGPTALIVSDGADHRRRRSVVAPGLRHHHVQRYVATMVSNIDTVIDGWQPGQRLDIYQELRSAVRRST 128

CYP139A1_2590146409_Mycob 59 PVDGPTALIVSDGADHRRRRSVVAPGLRHHHVQRYVATMVSNIDTVIDGWQPGQRLDIYQELRSAVRRST 128

CYP139A1_2590203420_Mycob 59 PVDGPTALIVSDGADHRRRRSVVAPGLRHHHVQRYVATMVSNIDTVIDGWQPGQRLDIYQELRSAVRRST 128

CYP139A1_2590272661_Mycob 59 PVDGPTALIVSDGADHRRRRSVVAPGLRHHHVQRYVATMVSNIDTVIDGWQPGQRLDIYQELRSAVRRST 128

CYP139A1_2590535240_Mycob 59 PVDGPTALIVSDGADHRRRRSVVAPGLRHHHVQRYVATMVSNIDTVIDGWQPGQRLDIYQELRSAVRRST 128

CYP139A1_647090515_Mycoba 59 PVDGPTALIVSDGADHRRRRSVVAPGLRHHHVQRYVATMVSNIDTVIDGWQPGQRLDIYQELRSAVRRST 128

CYP139A1_2512786915_Mycob 59 PVDGPTALIVSDGADHRRRRSVVAPGLRHHHVQRYVATMVSNIDTVIDGWQPGQRLDIYQELRSAVRRST 128

CYP139A1_2575329342_Mycob 59 PVDGPTALIVSDGADHRRRRSVVAPGLRHHHVQRYVATMVSNIDTVIDGWQPGQRLDIYQELRSAVRRST 128

CYP139A1_2575409751_Mycob 59 PVDGPTALIVSDGADHRRRRSVVAPGLRHHHVQRYVATMVSNIDTVIDGWQPGQRLDIYQELRSAVRRST 128

CYP139A1_2575862603_Mycob 59 PVDGPTALIVSDGADHRRRRSVVAPGLRHHHVQRYVATMVSNIDTVIDGWQPGQRLDIYQELRSAVRRST 128

CYP139A1_2576589126_Mycob 59 PVDGPTALIVSDGADHRRRRSVVAPGLRHHHVQRYVATMVSNIDTVIDGWQPGQRLDIYQELRSAVRRST 128

CYP139A1_2576649037_Mycob 59 PVDGPTALIVSDGADHRRRRSVVAPGLRHHHVQRYVATMVSNIDTVIDGWQPGQRLDIYQELRSAVRRST 128

CYP139A1_2576746511_Mycob 59 PVDGPTALIVSDGADHRRRRSVVAPGLRHHHVQRYVATMVSNIDTVIDGWQPGQRLDIYQELRSAVRRST 128

CYP139A1_2577129296_Mycob 59 PVDGPTALIVSDGADHRRRRSVVAPGLRHHHVQRYVATMVSNIDTVIDGWQPGQRLDIYQELRSAVRRST 128

CYP139A1_2578200424_Mycob 59 PVDGPTALIVSDGADHRRRRSVVAPGLRHHHVQRYVATMVSNIDTVIDGWQPGQRLDIYQELRSAVRRST 128

CYP139A1_2584666311_Mycob 59 PVDGPTALIVSDGADHRRRRSVVAPGLRHHHVQRYVATMVSNIDTVIDGWQPGQRLDIYQELRSAVRRST 128

CYP139A1_2584743940_Mycob 59 PVDGPTALIVSDGADHRRRRSVVAPGLRHHHVQRYVATMVSNIDTVIDGWQPGQRLDIYQELRSAVRRST 128

CYP139A1_2584781205_Mycob 59 PVDGPTALIVSDGADHRRRRSVVAPGLRHHHVQRYVATMVSNIDTVIDGWQPGQRLDIYQELRSAVRRST 128

CYP139A1_2584833644_Mycob 59 PVDGPTALIVSDGADHRRRRSVVAPGLRHHHVQRYVATMVSNIDTVIDGWQPGQRLDIYQELRSAVRRST 128

CYP139A1_2589073023_Mycob 59 PVDGPTALIVSDGADHRRRRSVVAPGLRHHHVQRYVATMVSNIDTVIDGWQPGQRLDIYQELRSAVRRST 128

CYP139A1_2589509569_Mycob 59 PVDGPTALIVSDGADHRRRRSVVAPGLRHHHVQRYVATMVSNIDTVIDGWQPGQRLDIYQELRSAVRRST 128

CYP139A1_2589551314_Mycob 59 PVDGPTALIVSDGADHRRRRSVVAPGLRHHHVQRYVATMVSNIDTVIDGWQPGQRLDIYQELRSAVRRST 128

CYP139A1_2589580598_Mycob 59 PVDGPTALIVSDGADHRRRRSVVAPGLRHHHVQRYVATMVSNIDTVIDGWQPGQRLDIYQELRSAVRRST 128

CYP139A1_2589629003_Mycob 59 PVDGPTALIVSDGADHRRRRSVVAPGLRHHHVQRYVATMVSNIDTVIDGWQPGQRLDIYQELRSAVRRST 128

CYP139A1_2589676017_Mycob 59 PVDGPTALIVSDGADHRRRRSVVAPGLRHHHVQRYVATMVSNIDTVIDGWQPGQRLDIYQELRSAVRRST 128

CYP139A1_2592332604_Mycob 59 PVDGPTALIVSDGADHRRRRSVVAPGLRHHHVQRYVATMVSNIDTVIDGWQPGQRLDIYQELRSAVRRST 128

CYP139A1_2592542016_Mycob 59 PVDGPTALIVSDGADHRRRRSVVAPGLRHHHVQRYVATMVSNIDTVIDGWQPGQRLDIYQELRSAVRRST 128

CYP139A1_2592562396_Mycob 59 PVDGPTALIVSDGADHRRRRSVVAPGLRHHHVQRYVATMVSNIDTVIDGWQPGQRLDIYQELRSAVRRST 128

CYP139A1_2541578033_Mycob 59 PVDGPTALIVSDGADHRRRRSVVAPGLRHHHVQRYVATMVSNIDTVIDGWQPGQRLDIYQELRSAVRRST 128

CYP139A1_2574761421_Mycob 59 PVDGPTALIVSDGADHRRRRSVVAPGLRHHHVQRYVATMVSNIDTVIDGWQPGQRLDIYQELRSAVRRST 128

CYP139A1_2575611823_Mycob 59 PVDGPTALIVSDGADHRRRRSVVAPGLRHHHVQRYVATMVSNIDTVIDGWQPGQRLDIYQELRSAVRRST 128

CYP139A1_2576564801_Mycob 59 PVDGPTALIVSDGADHRRRRSVVAPGLRHHHVQRYVATMVSNIDTVIDGWQPGQRLDIYQELRSAVRRST 128

CYP139A1_2577821955_Mycob 59 PVDGPTALIVSDGADHRRRRSVVAPGLRHHHVQRYVATMVSNIDTVIDGWQPGQRLDIYQELRSAVRRST 128

CYP139A1_2578086064_Mycob 59 PVDGPTALIVSDGADHRRRRSVVAPGLRHHHVQRYVATMVSNIDTVIDGWQPGQRLDIYQELRSAVRRST 128

CYP139A1_2580669105_Mycob 59 PVDGPTALIVSDGADHRRRRSVVAPGLRHHHVQRYVATMVSNIDTVIDGWQPGQRLDIYQELRSAVRRST 128

CYP139A1_2583740670_Mycob 59 PVDGPTALIVSDGADHRRRRSVVAPGLRHHHVQRYVATMVSNIDTVIDGWQPGQRLDIYQELRSAVRRST 128

CYP139A1_2584831832_Mycob 59 PVDGPTALIVSDGADHRRRRSVVAPGLRHHHVQRYVATMVSNIDTVIDGWQPGQRLDIYQELRSAVRRST 128

CYP139A1_2584875064_Mycob 59 PVDGPTALIVSDGADHRRRRSVVAPGLRHHHVQRYVATMVSNIDTVIDGWQPGQRLDIYQELRSAVRRST 128

CYP139A1_2590158810_Mycob 59 PVDGPTALIVSDGADHRRRRSVVAPGLRHHHVQRYVATMVSNIDTVIDGWQPGQRLDIYQELRSAVRRST 128

CYP139A1_2590240089_Mycob 59 PVDGPTALIVSDGADHRRRRSVVAPGLRHHHVQRYVATMVSNIDTVIDGWQPGQRLDIYQELRSAVRRST 128

CYP139A1_2590350138_Mycob 59 PVDGPTALIVSDGADHRRRRSVVAPGLRHHHVQRYVATMVSNIDTVIDGWQPGQRLDIYQELRSAVRRST 128

CYP139A1_2590493738_Mycob 59 PVDGPTALIVSDGADHRRRRSVVAPGLRHHHVQRYVATMVSNIDTVIDGWQPGQRLDIYQELRSAVRRST 128

CYP139A1_2590554696_Mycob 59 PVDGPTALIVSDGADHRRRRSVVAPGLRHHHVQRYVATMVSNIDTVIDGWQPGQRLDIYQELRSAVRRST 128

CYP139A1_2576084283_Mycob 59 PVDGPTALIVSDGADHRRRRSVVAPGLRHHHVQRYVATMVSNIDTVIDGWQPGQRLDIYQELRSAVRRST 128

CYP139A1_2576366402_Mycob 59 PVDGPTALIVSDGADHRRRRSVVAPGLRHHHVQRYVATMVSNIDTVIDGWQPGQRLDIYQELRSAVRRST 128

CYP139A1_2576513411_Mycob 59 PVDGPTALIVSDGADHRRRRSVVAPGLRHHHVQRYVATMVSNIDTVIDGWQPGQRLDIYQELRSAVRRST 128

CYP139A1_2576671439_Mycob 59 PVDGPTALIVSDGADHRRRRSVVAPGLRHHHVQRYVATMVSNIDTVIDGWQPGQRLDIYQELRSAVRRST 128

CYP139A1_2577792721_Mycob 59 PVDGPTALIVSDGADHRRRRSVVAPGLRHHHVQRYVATMVSNIDTVIDGWQPGQRLDIYQELRSAVRRST 128

CYP139A1_2577796885_Mycob 59 PVDGPTALIVSDGADHRRRRSVVAPGLRHHHVQRYVATMVSNIDTVIDGWQPGQRLDIYQELRSAVRRST 128

CYP139A1_2584686721_Mycob 59 PVDGPTALIVSDGADHRRRRSVVAPGLRHHHVQRYVATMVSNIDTVIDGWQPGQRLDIYQELRSAVRRST 128

CYP139A1_2584752944_Mycob 59 PVDGPTALIVSDGADHRRRRSVVAPGLRHHHVQRYVATMVSNIDTVIDGWQPGQRLDIYQELRSAVRRST 128

CYP139A1_2584808933_Mycob 59 PVDGPTALIVSDGADHRRRRSVVAPGLRHHHVQRYVATMVSNIDTVIDGWQPGQRLDIYQELRSAVRRST 128

CYP139A1_2589134136_Mycob 59 PVDGPTALIVSDGADHRRRRSVVAPGLRHHHVQRYVATMVSNIDTVIDGWQPGQRLDIYQELRSAVRRST 128

CYP139A1_2589539130_Mycob 59 PVDGPTALIVSDGADHRRRRSVVAPGLRHHHVQRYVATMVSNIDTVIDGWQPGQRLDIYQELRSAVRRST 128

CYP139A1_2589598915_Mycob 59 PVDGPTALIVSDGADHRRRRSVVAPGLRHHHVQRYVATMVSNIDTVIDGWQPGQRLDIYQELRSAVRRST 128

CYP139A1_2589679190_Mycob 59 PVDGPTALIVSDGADHRRRRSVVAPGLRHHHVQRYVATMVSNIDTVIDGWQPGQRLDIYQELRSAVRRST 128

CYP139A1_2589727979_Mycob 59 PVDGPTALIVSDGADHRRRRSVVAPGLRHHHVQRYVATMVSNIDTVIDGWQPGQRLDIYQELRSAVRRST 128

CYP139A1_2592361217_Mycob 59 PVDGPTALIVSDGADHRRRRSVVAPGLRHHHVQRYVATMVSNIDTVIDGWQPGQRLDIYQELRSAVRRST 128

CYP139A1_2592442711_Mycob 59 PVDGPTALIVSDGADHRRRRSVVAPGLRHHHVQRYVATMVSNIDTVIDGWQPGQRLDIYQELRSAVRRST 128

CYP139A1_2555325132_Mycob 65 PVDGPTALIVSDGADHRRRRSVVAPGLRHHHVQRYVATMVSNIDTVIDGWQPGQRLDIYQELRSAVRRST 134

CYP139A1_2566259019_Mycob 65 PVDGPTALIVSDGADHRRRRSVVAPGLRHHHVQRYVATMVSNIDTVIDGWQPGQRLDIYQELRSAVRRST 134

CYP139A1_2555164435_Mycob 65 PVDGPTALIVSDGADHRRRRSVVAPGLRHHHVQRYVATMVSNIDTVIDGWQPGQRLDIYQELRSAVRRST 134

CYP139A1_2555583039_Mycob 65 PVDGPTALIVSDGADHRRRRSVVAPGLRHHHVQRYVATMVSNIDTVIDGWQPGQRLDIYQELRSAVRRST 134

CYP139A1_2555295099_Mycob 65 PVDGPTALIVSDGADHRRRRSVVAPGLRHHHVQRYVATMVSNIDTVIDGWQPGQRLDIYQELRSAVRRST 134

CYP139A1_2555337752_Mycob 65 PVDGPTALIVSDGADHRRRRSVVAPGLRHHHVQRYVATMVSNIDTVIDGWQPGQRLDIYQELRSAVRRST 134

CYP139A1_2555516018_Mycob 65 PVDGPTALIVSDGADHRRRRSVVAPGLRHHHVQRYVATMVSNIDTVIDGWQPGQRLDIYQELRSAVRRST 134

CYP139A1_2576196505_Mycob 59 PVDGPTALIVSDGADHRRRRSVVAPGLRHHHVQRYVATMVSNIDTVIDGWQPGQRLDIYQELRSAVRRST 128

CYP139A1_2555417623_Mycob 65 PVDGPTALIVSDGADHRRRRSVVAPGLRHHHVQRYVATMVSNIDTVIDGWQPGQRLDIYQELRSAVRRST 134

CYP139A1_2555430098_Mycob 65 PVDGPTALIVSDGADHRRRRSVVAPGLRHHHVQRYVATMVSNIDTVIDGWQPGQRLDIYQELRSAVRRST 134

CYP139A1_2547880750_Mycob 65 PVDGPTALIVSDGADHRRRRSVVAPGLRHHHVQRYVATMVSNIDTVIDGWQPGQRLDIYQELRSAVRRST 134

CYP139A1_2559163499_Mycob 65 PVDGPTALIVSDGADHRRRRSVVAPGLRHHHVQRYVATMVSNIDTVIDGWQPGQRLDIYQELRSAVRRST 134

CYP139A1_2527056892_Mycob 59 PVDGPTALIVSDGADHRRRRSVVAPGLRHHHVQRYVATMVSNIDTVIDGWQPGQRLDIYQELRSAVRRST 128

CYP139A1_2574675630_Mycob 59 PVDGPTALIVSDGADHRRRRSVVAPGLRHHHVQRYVATMVSNIDTVIDGWQPGQRLDIYQELRSAVRRST 128

CYP139A1_2574700588_Mycob 59 PVDGPTALIVSDGADHRRRRSVVAPGLRHHHVQRYVATMVSNIDTVIDGWQPGQRLDIYQELRSAVRRST 128

CYP139A1_2574830606_Mycob 59 PVDGPTALIVSDGADHRRRRSVVAPGLRHHHVQRYVATMVSNIDTVIDGWQPGQRLDIYQELRSAVRRST 128

CYP139A1_2574928549_Mycob 59 PVDGPTALIVSDGADHRRRRSVVAPGLRHHHVQRYVATMVSNIDTVIDGWQPGQRLDIYQELRSAVRRST 128

CYP139A1_2575381682_Mycob 59 PVDGPTALIVSDGADHRRRRSVVAPGLRHHHVQRYVATMVSNIDTVIDGWQPGQRLDIYQELRSAVRRST 128

CYP139A1_2575663406_Mycob 59 PVDGPTALIVSDGADHRRRRSVVAPGLRHHHVQRYVATMVSNIDTVIDGWQPGQRLDIYQELRSAVRRST 128

CYP139A1_2576431117_Mycob 59 PVDGPTALIVSDGADHRRRRSVVAPGLRHHHVQRYVATMVSNIDTVIDGWQPGQRLDIYQELRSAVRRST 128

CYP139A1_2576553939_Mycob 59 PVDGPTALIVSDGADHRRRRSVVAPGLRHHHVQRYVATMVSNIDTVIDGWQPGQRLDIYQELRSAVRRST 128

CYP139A1_2577269438_Mycob 59 PVDGPTALIVSDGADHRRRRSVVAPGLRHHHVQRYVATMVSNIDTVIDGWQPGQRLDIYQELRSAVRRST 128

CYP139A1_2584870715_Mycob 59 PVDGPTALIVSDGADHRRRRSVVAPGLRHHHVQRYVATMVSNIDTVIDGWQPGQRLDIYQELRSAVRRST 128

CYP139A1_2589036086_Mycob 59 PVDGPTALIVSDGADHRRRRSVVAPGLRHHHVQRYVATMVSNIDTVIDGWQPGQRLDIYQELRSAVRRST 128

CYP139A1_2590101605_Mycob 59 PVDGPTALIVSDGADHRRRRSVVAPGLRHHHVQRYVATMVSNIDTVIDGWQPGQRLDIYQELRSAVRRST 128

CYP139A1_2590354422_Mycob 59 PVDGPTALIVSDGADHRRRRSVVAPGLRHHHVQRYVATMVSNIDTVIDGWQPGQRLDIYQELRSAVRRST 128

CYP139A1_648490022_Mycoba 59 PVDGPTALIVSDGADHRRRRSVVAPGLRHHHVQRYVATMVSNIDTVIDGWQPGQRLDIYQELRSAVRRST 128

CYP139A1_2547317188_Mycob 65 PVDGPTALIVSDGADHRRRRSVVAPGLRHHHVQRYVATMVSNIDTVIDGWQPGQRLDIYQELRSAVRRST 134

CYP139A1_2547959756_Mycob 65 PVDGPTALIVSDGADHRRRRSVVAPGLRHHHVQRYVATMVSNIDTVIDGWQPGQRLDIYQELRSAVRRST 134

CYP139A1_2555303475_Mycob 65 PVDGPTALIVSDGADHRRRRSVVAPGLRHHHVQRYVATMVSNIDTVIDGWQPGQRLDIYQELRSAVRRST 134

CYP139A1_2555392373_Mycob 65 PVDGPTALIVSDGADHRRRRSVVAPGLRHHHVQRYVATMVSNIDTVIDGWQPGQRLDIYQELRSAVRRST 134

CYP139A1_2566985178_Mycob 65 PVDGPTALIVSDGADHRRRRSVVAPGLRHHHVQRYVATMVSNIDTVIDGWQPGQRLDIYQELRSAVRRST 134

CYP139A1_2555362970_Mycob 65 PVDGPTALIVSDGADHRRRRSVVAPGLRHHHVQRYVATMVSNIDTVIDGWQPGQRLDIYQELRSAVRRST 134

CYP139A1_2560449536_Mycob 65 PVDGPTALIVSDGADHRRRRSVVAPGLRHHHVQRYVATMVSNIDTVIDGWQPGQRLDIYQELRSAVRRST 134

CYP139A1_2555152706_Mycob 65 PVDGPTALIVSDGADHRRRRSVVAPGLRHHHVQRYVATMVSNIDTVIDGWQPGQRLDIYQELRSAVRRST 134

CYP139A1_2555543565_Mycob 65 PVDGPTALIVSDGADHRRRRSVVAPGLRHHHVQRYVATMVSNIDTVIDGWQPGQRLDIYQELRSAVRRST 134

CYP139A1_2590081259_Mycob 59 PVDGPTALIVSDGADHRRRRSVVAPGLRHHHVQRYVATMVSNIDTVIDGWQPGQRLDIYQELRSAVRRST 128

CYP139A1_2547311116_Mycob 65 PVDGPTALIVSDGADHRRRRSVVAPGLRHHHVQRYVATMVSNIDTVIDGWQPGQRLDIYQELRSAVRRST 134

CYP139A1_2548033169_Mycob 65 PVDGPTALIVSDGADHRRRRSVVAPGLRHHHVQRYVATMVSNIDTVIDGWQPGQRLDIYQELRSAVRRST 134

CYP139A1_2548037418_Mycob 65 PVDGPTALIVSDGADHRRRRSVVAPGLRHHHVQRYVATMVSNIDTVIDGWQPGQRLDIYQELRSAVRRST 134

CYP139A1_2555282371_Mycob 65 PVDGPTALIVSDGADHRRRRSVVAPGLRHHHVQRYVATMVSNIDTVIDGWQPGQRLDIYQELRSAVRRST 134

CYP139A1_2541569776_Mycob 59 PVDGPTALIVSDGADHRRRRSVVAPGLRHHHVQRYVATMVSNIDTVIDGWQPGQRLDIYQELRSAVRRST 128

CYP139A1_2555299255_Mycob 65 PVDGPTALIVSDGADHRRRRSVVAPGLRHHHVQRYVATMVSNIDTVIDGWQPGQRLDIYQELRSAVRRST 134

CYP139A1_2555379694_Mycob 65 PVDGPTALIVSDGADHRRRRSVVAPGLRHHHVQRYVATMVSNIDTVIDGWQPGQRLDIYQELRSAVRRST 134

CYP139A1_2555413387_Mycob 65 PVDGPTALIVSDGADHRRRRSVVAPGLRHHHVQRYVATMVSNIDTVIDGWQPGQRLDIYQELRSAVRRST 134

CYP139A1_2555587223_Mycob 65 PVDGPTALIVSDGADHRRRRSVVAPGLRHHHVQRYVATMVSNIDTVIDGWQPGQRLDIYQELRSAVRRST 134

CYP139A1_2555591399_Mycob 65 PVDGPTALIVSDGADHRRRRSVVAPGLRHHHVQRYVATMVSNIDTVIDGWQPGQRLDIYQELRSAVRRST 134

CYP139A1_2547306531_Mycob 65 PVDGPTALIVSDGADHRRRRSVVAPGLRHHHVQRYVATMVSNIDTVIDGWQPGQRLDIYQELRSAVRRST 134

CYP139A1_651088108_Mycoba 65 PVDGPTALIVSDGADHRRRRSVVAPGLRHHHVQRYVATMVSNIDTVIDGWQPGQRLDIYQELRSAVRRST 134

CYP139A1_2598067418_Mycob 65 PVDGPTALIVSDGADHRRRRSVVAPGLRHHHVQRYVATMVSNIDTVIDGWQPGQRLDIYQELRSAVRRST 134

CYP139A1_2555341950_Mycob 65 PVDGPTALIVSDGADHRRRRSVVAPGLRHHHVQRYVATMVSNIDTVIDGWQPGQRLDIYQELRSAVRRST 134

CYP139A1_2555446824_Mycob 65 PVDGPTALIVSDGADHRRRRSVVAPGLRHHHVQRYVATMVSNIDTVIDGWQPGQRLDIYQELRSAVRRST 134

CYP139A1_2555556540_Mycob 65 PVDGPTALIVSDGADHRRRRSVVAPGLRHHHVQRYVATMVSNIDTVIDGWQPGQRLDIYQELRSAVRRST 134

CYP139A1_2560451827_Mycob 65 PVDGPTALIVSDGADHRRRRSVVAPGLRHHHVQRYVATMVSNIDTVIDGWQPGQRLDIYQELRSAVRRST 134

CYP139A1_2540619998_Mycob 65 PVDGPTALIVSDGADHRRRRSVVAPGLRHHHVQRYVATMVSNIDTVIDGWQPGQRLDIYQELRSAVRRST 134

CYP139A1_2554692349_Mycob 65 PVDGPTALIVSDGADHRRRRSVVAPGLRHHHVQRYVATMVSNIDTVIDGWQPGQRLDIYQELRSAVRRST 134

CYP139A1_637096038_Mycoba 65 PVDGPTALIVSDGADHRRRRSVVAPGLRHHHVQRYVATMVSNIDTVIDGWQPGQRLDIYQELRSAVRRST 134

CYP139A1_2555396597_Mycob 65 PVDGPTALIVSDGADHRRRRSVVAPGLRHHHVQRYVATMVSNIDTVIDGWQPGQRLDIYQELRSAVRRST 134

CYP139A1_2555520186_Mycob 65 PVDGPTALIVSDGADHRRRRSVVAPGLRHHHVQRYVATMVSNIDTVIDGWQPGQRLDIYQELRSAVRRST 134

CYP139A1_2555599804_Mycob 65 PVDGPTALIVSDGADHRRRRSVVAPGLRHHHVQRYVATMVSNIDTVIDGWQPGQRLDIYQELRSAVRRST 134

CYP139A1_2547314995_Mycob 65 PVDGPTALIVSDGADHRRRRSVVAPGLRHHHVQRYVATMVSNIDTVIDGWQPGQRLDIYQELRSAVRRST 134

CYP139A1_2555160272_Mycob 65 PVDGPTALIVSDGADHRRRRSVVAPGLRHHHVQRYVATMVSNIDTVIDGWQPGQRLDIYQELRSAVRRST 134

CYP139A1_2555367201_Mycob 65 PVDGPTALIVSDGADHRRRRSVVAPGLRHHHVQRYVATMVSNIDTVIDGWQPGQRLDIYQELRSAVRRST 134

CYP139A1_2555547894_Mycob 65 PVDGPTALIVSDGADHRRRRSVVAPGLRHHHVQRYVATMVSNIDTVIDGWQPGQRLDIYQELRSAVRRST 134

CYP139A1_2560454644_Mycob 65 PVDGPTALIVSDGADHRRRRSVVAPGLRHHHVQRYVATMVSNIDTVIDGWQPGQRLDIYQELRSAVRRST 134

CYP139A1_2546436155_Mycob 59 PVDGPTALIVSDGADHRRRRSVVAPGLRHHHVQRYVATMVSNIDTVIDGWQPGQRLDIYQELRSAVRRST 128

CYP139A1_2555307696_Mycob 65 PVDGPTALIVSDGADHRRRRSVVAPGLRHHHVQRYVATMVSNIDTVIDGWQPGQRLDIYQELRSAVRRST 134

CYP139A1_2555524372_Mycob 65 PVDGPTALIVSDGADHRRRRSVVAPGLRHHHVQRYVATMVSNIDTVIDGWQPGQRLDIYQELRSAVRRST 134

CYP139A1_2546192085_Mycob 59 PVDGPTALIVSDGADHRRRRSVVAPGLRHHHVQRYVATMVSNIDTVIDGWQPGQRLDIYQELRSAVRRST 128

CYP139A1_2553261414_Mycob 65 PVDGPTALIVSDGADHRRRRSVVAPGLRHHHVQRYVATMVSNIDTVIDGWQPGQRLDIYQELRSAVRRST 134

CYP139A1_2555442648_Mycob 65 PVDGPTALIVSDGADHRRRRSVVAPGLRHHHVQRYVATMVSNIDTVIDGWQPGQRLDIYQELRSAVRRST 134

CYP139A1_2555455224_Mycob 65 PVDGPTALIVSDGADHRRRRSVVAPGLRHHHVQRYVATMVSNIDTVIDGWQPGQRLDIYQELRSAVRRST 134

CYP139A1_651084428_Mycoba 65 PVDGPTALIVSDGADHRRRRSVVAPGLRHHHVQRYVATMVSNIDTVIDGWQPGQRLDIYQELRSAVRRST 134

CYP139A1_2555273883_Mycob 65 PVDGPTALIVSDGADHRRRRSVVAPGLRHHHVQRYVATMVSNIDTVIDGWQPGQRLDIYQELRSAVRRST 134

CYP139A1_2555286609_Mycob 65 PVDGPTALIVSDGADHRRRRSVVAPGLRHHHVQRYVATMVSNIDTVIDGWQPGQRLDIYQELRSAVRRST 134

CYP139A1_2555329342_Mycob 65 PVDGPTALIVSDGADHRRRRSVVAPGLRHHHVQRYVATMVSNIDTVIDGWQPGQRLDIYQELRSAVRRST 134

CYP139A1_2555438445_Mycob 65 PVDGPTALIVSDGADHRRRRSVVAPGLRHHHVQRYVATMVSNIDTVIDGWQPGQRLDIYQELRSAVRRST 134

CYP139A1_2577281457_Mycob 65 PVDGPTALIVSDGADHRRRRSVVAPGLRHHHVQRYVATMVSNIDTVIDGWQPGQRLDIYQELRSAVRRST 134

CYP139A1_2590256393_Mycob 59 PVDGPTALIVSDGADHRRRRSVVAPGLRHHHVQRYVATMVSNIDTVIDGWQPGQRLDIYQELRSAVRRST 128

CYP139A1_2549407735_Mycob 65 PVDGPTALIVSDGADHRRRRSVVAPGLRHHHVQRYVATMVSNIDTVIDGWQPGQRLDIYQELRSAVRRST 134

CYP139A1_2573574061_Mycob 65 PVDGPTALIVSDGADHRRRRSVVAPGLRHHHVQRYVATMVSNIDTVIDGWQPGQRLDIYQELRSAVRRST 134

CYP139A1_2540803840_Mycob 65 PVDGPTALIVSDGADHRRRRSVVAPGLRHHHVQRYVATMVSNIDTVIDGWQPGQRLDIYQELRSAVRRST 134

CYP139A1_2584769363_Mycob 59 PVDGPTALIVSDGADHRRRRSVVAPGLRHHHFQRYVATMVSNIDTVIDGWQPGQRLDIYQELRSAVRRST 128

CYP139A1_2555311951_Mycob 65 PVDGPTALIVSDGADHRRRRSVVAPGLRHHHVQRYVATMVSNIDTVIDGWQPGQRLDIYQELRSAVRRST 134

CYP139A1_2555346108_Mycob 65 PVDGPTALIVSDGADHRRRRSVVAPGLRHHHVQRYVATMVSNIDTVIDGWQPGQRLDIYQELRSAVRRST 134

CYP139A1_2555383921_Mycob 65 PVDGPTALIVSDGADHRRRRSVVAPGLRHHHVQRYVATMVSNIDTVIDGWQPGQRLDIYQELRSAVRRST 134

CYP139A1_2555400811_Mycob 65 PVDGPTALIVSDGADHRRRRSVVAPGLRHHHVQRYVATMVSNIDTVIDGWQPGQRLDIYQELRSAVRRST 134

CYP139A1_2555560826_Mycob 65 PVDGPTALIVSDGADHRRRRSVVAPGLRHHHVQRYVATMVSNIDTVIDGWQPGQRLDIYQELRSAVRRST 134

CYP139A1_2598813154_Mycob 65 PVDGPTALIVSDGADHRRRRSVVAPGLRHHHVQRYVATMVSNIDTVIDGWQPGQRLDIYQELRSAVRRST 134

CYP139A1_2547164190_Mycob 65 PVDGPTALIVSDGADHRRRRSVVAPGLRHHHVQRYVATMVSNIDTVIDGWQPGQRLDIYQELRSAVRRST 134

CYP139A1_2555409181_Mycob 65 PVDGPTALIVSDGADHRRRRSVVAPGLRHHHVQRYVATMVSNIDTVIDGWQPGQRLDIYQELRSAVRRST 134

CYP139A1_2547759833_Mycob 65 PVDGPTALIVSDGADHRRRRSVVAPGLRHHHVQRYVATMVSNIDTVIDGWQPGQRLDIYQELRSAVRRST 134

CYP139A1_2547955540_Mycob 65 PVDGPTALIVSDGADHRRRRSVVAPGLRHHHVQRYVATMVSNIDTVIDGWQPGQRLDIYQELRSAVRRST 134

CYP139A1_2560461324_Mycob 65 PVDGPTALIVSDGADHRRRRSVVAPGLRHHHVQRYVATMVSNIDTVIDGWQPGQRLDIYQELRSAVRRST 134

CYP139A1_2576392640_Mycob 59 PVDGPTALIVSDGADHRRRRSAVAPGLRHHHVQRYVATMVSNIDTVIDGWQPGQRLDIYQELRSAVRRST 128

CYP139A1_2541573920_Mycob 59 PVDGPTALIVSDGADHRRRRSVVAPGLRHHHVQRYVATMVSNIDTVIDGWQPGQRLDIYQELRSAVRRST 128

CYP139A1_2549413118_Mycob 65 PVDGPTALIVSDGADHRRRRSVVAPGLRHHHVQRYVATMVSNIDTVIDGWQPGQRLDIYQELRSAVRRST 134

CYP139A1_2555333539_Mycob 65 PVDGPTALIVSDGADHRRRRSVVAPGLRHHHVQRYVATMVSNIDTVIDGWQPGQRLDIYQELRSAVRRST 134

CYP139A1_2555371358_Mycob 65 PVDGPTALIVSDGADHRRRRSVVAPGLRHHHVQRYVATMVSNIDTVIDGWQPGQRLDIYQELRSAVRRST 134

CYP139A1_2545499027_Mycob 65 PVDGPTALIVSDGADHRRRRSVVAPGLRHHHVQRYVATMVSNIDTVIDGWQPGQRLDIYQELRSAVRRST 134

CYP139A1_2551812688_Mycob 65 PVDGPTALIVSDGADHRRRRSVVAPGLRHHHVQRYVATMVSNIDTVIDGWQPGQRLDIYQELRSAVRRST 134

CYP139A1_2620699696_Mycob 65 PVDGPTALIVSDGADHRRRRSVVAPGLRHHHVQRYVATMVSNIDTVIDGWQPGQRLDIYQELRSAVRRST 134

CYP139A1_2555278104_Mycob 65 PVDGPTALIVSDGADHRRRRSVVAPGLRHHHVQRYVATMVSNIDTVIDGWQPGQRLDIYQELRSAVRRST 134

CYP139A1_2555320646_Mycob 65 PVDGPTALIVSDGADHRRRRSVVAPGLRHHHVQRYVATMVSNIDTVIDGWQPGQRLDIYQELRSAVRRST 134

CYP139A1_2555354513_Mycob 65 PVDGPTALIVSDGADHRRRRSVVAPGLRHHHVQRYVATMVSNIDTVIDGWQPGQRLDIYQELRSAVRRST 134

CYP139A1_2549401785_Mycob 65 PVDGPTALIVSDGADHRRRRSVVAPGLRHHHVQRYVATMVSNIDTVIDGWQPGQRLDIYQELRSAVRRST 134

CYP139A1_2555434304_Mycob 65 PVDGPTALIVSDGADHRRRRSVVAPGLRHHHVQRYVATMVSNIDTVIDGWQPGQRLDIYQELRSAVRRST 134

CYP139A1_2555528546_Mycob 65 PVDGPTALIVSDGADHRRRRSVVAPGLRHHHVQRYVATMVSNIDTVIDGWQPGQRLDIYQELRSAVRRST 134

CYP139A1_2555595572_Mycob 65 PVDGPTALIVSDGADHRRRRSVVAPGLRHHHVQRYVATMVSNIDTVIDGWQPGQRLDIYQELRSAVRRST 134

CYP139A1_2555144317_Mycob 65 PVDGPTALIVSDGADHRRRRSVVAPGLRHHHVQRYVATMVSNIDTVIDGWQPGQRLDIYQELRSAVRRST 134

CYP139A1_2555290904_Mycob 65 PVDGPTALIVSDGADHRRRRSVVAPGLRHHHVQRYVATMVSNIDTVIDGWQPGQRLDIYQELRSAVRRST 134

CYP139A1_2555316223_Mycob 65 PVDGPTALIVSDGADHRRRRSVVAPGLRHHHVQRYVATMVSNIDTVIDGWQPGQRLDIYQELRSAVRRST 134

CYP139A1_2555388140_Mycob 65 PVDGPTALIVSDGADHRRRRSVVAPGLRHHHVQRYVATMVSNIDTVIDGWQPGQRLDIYQELRSAVRRST 134

CYP139A1_2555350295_Mycob 65 PVDGPTALIVSDGADHRRRRSVVAPGLRHHHVQRYVATMVSNIDTVIDGWQPGQRLDIYQELRSAVRRST 134

CYP139A1_2555960457_Mycob 65 PVDGPTALIVSDGADHRRRRSVVAPGLRHHHVQRYVATMVSNIDTVIDGWQPGQRLDIYQELRSAVRRST 134

CYP139A1_2547951333_Mycob 65 PVDGPTALIVSDGADHRRRRSVVAPGLRHHHVQRYVATMVSNIDTVIDGWQPGQRLDIYQELRSAVRRST 134

CYP139A1_2555451004_Mycob 65 PVDGPTALIVSDGADHRRRRSVVAPGLRHHHVQRYVATMVSNIDTVIDGWQPGQRLDIYQELRSAVRRST 134

CYP139A1_2555140094_Mycob 65 PVDGPTALIVSDGADHRRRRSVVAPGLRHHHVQRYVATMVSNIDTVIDGWQPGQRLDIYQELRSAVRRST 134

CYP139A1_2555358731_Mycob 65 PVDGPTALIVSDGADHRRRRSVVAPGLRHHHVQRYVATMVSNIDTVIDGWQPGQRLDIYQELRSAVRRST 134

CYP139A1_2555375511_Mycob 65 PVDGPTALIVSDGADHRRRRSVVAPGLRHHHVQRYVATMVSNIDTVIDGWQPGQRLDIYQELRSAVRRST 134

CYP139A1_2555578867_Mycob 65 PVDGPTALIVSDGADHRRRRSVVAPGLRHHHVQRYVATMVSNIDTVIDGWQPGQRLDIYQELRSAVRRST 134

CYP139A1_2554700949_Mycob 65 PVDGPTALIVSDGADHRRRRSVVAPGLRHHHVQRYVATMVSNIDTVIDGWQPGQRLDIYQELRSAVRRST 134

CYP139A1_2575403392_Mycob 65 PVDGPTALIVSDGADHRRRRSVVAPGLRHHHVQRYVATMVSNIDTVIDGWQPGQRLDIYQELRSAVRRST 134

CYP139A1_638726892_Mycoba 65 PVDGPTALIVSDGADHRRRRSVVAPGLRHHHVQRYVATMVSNIDTVIDGWQPGQRLDIYQELRSAVRRST 134

CYP139A1_648443266_Mycoba 65 PVDGPTALIVSDGADHRRRRSVVAPGLRHHHVQRYVATMVSNIDTVIDGWQPGQRLDIYQELRSAVRRST 134

CYP139A1_2540554561_Mycob 59 PVDGPTALIVSDGADHRRRRSVVAPGLRHHHVQRYVATMVSNIDTVIDGWQPGQRLDIYQELRSAVRRST 128

CYP139A1_2566980890_Mycob 65 PVDGPTALIVSDGADHRRRRSVVAPGLRHHHVQRYVATMVSNIDTVIDGWQPGQRLDIYQELRSAVRRST 134

CYP139A1_2566976623_Mycob 65 PVDGPTALIVSDGADHRRRRSVVAPGLRHHHVQRYVATMVSNIDTVIDGWQPGQRLDIYQELRSAVRRST 134

CYP139A1_2566972350_Mycob 65 PVDGPTALIVSDGADHRRRRSVVAPGLRHHHVQRYVATMVSNIDTVIDGWQPGQRLDIYQELRSAVRRST 134

CYP139A1_2514118145_Mycob 60 PVDGPTALIVSDGADHRRRRSVVAPGLRHHHVQRYVATMVSNIDTVIDGWQPGQRLDIYQELRSAVRRST 129

[Consensus_aa:](http://prodata.swmed.edu/promals3d/info/consensus.html) .**VDGPTALIVSDG**s**DHRRRRS***l***V***h***PGL+H+**p*l***Q**c**YV**.**TMV**ts*l***D**s**VID**t**W**p**PGQ**p**LD***l***Y**pp*h***R**t**AVRRST**

[Consensus_ss:](http://prodata.swmed.edu/promals3d/info/consensus_ss.html) hh eee hhhhhhhhhhhh hhhhhhhhhhhhhhhhhhhhhhh hhhhhhhhhhhhh

Conservation: 96999 69 969 996 9996 999 696 66 9 6699696699 9 6 66 6 6 99 9

CYP139A_2567124714_Mycoba 135 AESLFGPRLAAHSDFLGEQLQPLLDLTRRPPQLMRLQRRLNSPGWRRAMAARHRVDDLVGAVISDARPRP 204

CYP139A_650873455_Mycobac 129 IEALFGQSMAGHADFLGEQLQPLMDLTQRLPQVLKAEARLKTPAWRRAMAARARVDELIYAEIARARTHP 198

CYP139A_2549393401_Mycoba 133 IESLFGQTMAVHADFLGEQLQPLMDLTHRLPQVLKAEARLKTPAWRRAMAARTRVDELIYAEIARARTHP 202

CYP139A_2548535921_Mycoba 132 AESLFGPRLAVHSDALGEHLQPLLDLTHQPPQLVGLQRRINAPAWRRAMAARQRINNLVDTLIADARAAP 201

CYP139A_2576976958_Mycoba 129 AESLFGPRLAVHSDALGEYLQPLLDLTHQPPQLVGLQRRINAPAWRRAMAARQRINNLVDTLIAEARAAP 198

CYP139A_2581913245_Mycoba 129 AESLFGPRLAVHSDALGEHLQPLLDLTHQPPQLVGLQRRINAPAWRRAMAARQRINNLVDTLIAEARAAP 198

CYP139A_2580974538_Mycoba 129 AESLFGPRLAVHSDALGEYLQPLLDLTHQPPQLVGLQRRINAPAWRRAMAARQRINNLVDTLIAEARAAP 198

CYP139A_2582181025_Mycoba 129 AESLFGPRLAVHSDALGEHLQPLLDLTHQPPQLVGLQRRINAPAWRRAMAARQRINNLVDTLIAEARAAP 198

CYP139A_2580742569_Mycoba 129 AESLFGPRLAVHSDALGEYLQPLLDLTHQPPQLVGLQRRINAPAWRRAMAARQRINNLVDTLIAEARAAP 198

CYP139A_2582203743_Mycoba 129 AESLFGPRLAVHSDALGEYLQPLLDLTHQPPQLVGLQRRINAPAWRRAMAARQRINNLVDTLIAEARAAP 198

CYP139A_2567079276_Mycoba 111 AESLFGRRLAVHSDALGEYLQPLLDLTHQPPQLVGLQRRINAPAWRRAMAARQRINNLVDTLIAEARAAP 180

CYP139A_2543326887_Mycoba 129 AESLFGPRLAVHSDALGEHLQPLLDLTHQPPQLVGLQRRINAPAWRRAMAARQRINNLVDTLIADARAAP 198

CYP139A_2570865822_Mycoba 129 AESLFGPRLAVHSDALGEHLQPLLDLTHQPPQLVGLQRRINAPAWRRAMAARQRINNLVDTLIADARAAP 198

CYP139A_2592485489_Mycoba 129 AESLFGPRLAVHSDALGEHLQPLLDLTHQPPQLVGLQRRINAPAWRRAMAARQRINNLVDTLIADARAAP 198

CYP139A_2550738610_Mycoba 132 AESLFGPRLAVHSDALGEHLQPLLDLTHQPPQLVGLQRRINAPAWRRAMAARQRINNLVDTLIADARAAP 201

CYP139A_2580783837__Mycob 129 AESLFGPRLAVHSDALGEYLQPLLDLTHQPPQLVGLQRRINAPAWRRAMAARQRINNLVDTLIADARAAP 198

CYP139A_2569618768_Mycoba 129 AESLFGRRLAVHSDALGEYLQPLLDLTHQPPQLVGLQRRINAPAWRRAMAARQRINNLVDTLIAEARAAP 198

CYP139A_2547368463_Mycoba 132 AESLFGPRLAVHSDALGEHLQPLLDLTHQPPQLVGLQRRINAPAWRRAMAARQRINNLVDTLIADARAAP 201

CYP139A_2572767979_Mycoba 129 AESLFGRRLAVHSDALGEYLQPLLDLTHQPPQLVGLQRRINAPAWRRAMAARQRINNLVDTLIAEARAAP 198

CYP139A_2582391300_Mycoba 129 AESLFGRRLAVHSDALGEYLQPLLDLTHQPPQLVGLQRRINAPAWRRAMAARQRINNLVDTLIAEARAAP 198

CYP139A_2548515815_Mycoba 132 AESLFGPRLAVHSDALGEHLQPLLDLTHQPPQLVGLQRRINAPAWRRAMAARQRINNLVDTLIADARAAP 201

CYP139A_2548530385_Mycoba 132 AESLFGPRLAVHSDALGEHLQPLLDLTHQPPQLVGLQRRINAPAWRRAMAARQRINNLVDTLIADARAAP 201

CYP139A_2581397788_Mycoba 129 AESLFGRRLAVHSDALGEYLQPLLDLTHQPPQLVGLQRRINAPAWRRAMAARQRINNLVDTLIAEARAAP 198

CYP139A_2549377452_Mycoba 132 AESLFGPRLAVHSDALGEHLQPLLDLTHQPPQLVGLQRRINAPAWRRAMAARQRINNLVDTLIADARAAP 201

CYP139A_2549389164_Mycoba 132 AESLFGRRLAVHSDALGEYLQPLLDLTHQPPQLVGLQRRINAPAWRRAMAARQRINNLVDTLIAEARAAP 201

CYP139A_645425415_Mycobac 129 AESLFGRRLAVHSDALGEYLQPLLDLTHQPPQLVGLQRRINAPAWRRAMAARQRINNLVDTLIAEARAAP 198

CYP139A_2581110378_Mycoba 132 AESLFGRRLAVHSDALGEYLQPLLDLTHQPPQLVGLQRRINAPAWRRAMAARQRINNLVDTLIAEARAAP 201

CYP139A_2573433552_Mycoba 129 AESLFGRRLAVHSDALGEYLQPLLDLTHQPPQLVGLQRRINAPAWRRAMAARQRINNLVDTLIAEARAAP 198

CYP139A_2580006443__Mycob 129 AESLFGRRLAVHSDALGEYLQPLLDLTHQPPQLVGLQRRINAPAWRRAMAARQRINNLVDTLIAEARAAP 198

CYP139A_637134331_Mycobac 129 AESLFGPRLAVHSDALGEHLQPLLDLTHQPPQLVGLQRRINAPAWRRAMAARQRINNLVDTLIADARAAP 198

CYP139A_2548578292_Mycoba 132 AESLFGPRLAVHSDALGEHLQPLLDLTHQPPQLVGLQRRINAPAWRRAMAARQRINNLVDTLIADARAAP 201

CYP139A_2548547272_Mycoba 132 AESLFGPRLAVHSDALGEHLQPLLDLTHQPPQLVGLQRRINAPAWRRAMAARQRINNLVDTLIADARAAP 201

CYP139A_2549383420_Mycoba 132 AESLFGPRLAVHSDALGEHLQPLLDLTHQPPQLVGLQRRINAPAWRRAMAARQRINNLVDTLIADARAAP 201

CYP139A_639736419_Mycobac 129 AESLFGRRLAVHSDALGEYLQPLLDLTHQPPQLVGLQRRINAPAWRRAMAARQRINNLVDTLIAEARAAP 198

CYP139A_2555735619_Mycoba 129 AESLFGPRLAVHSDALGEHLQPLLDLTHQPPQLVGLQRRINAPAWRRAMAARQRINNLVDTLIADARAAP 198

CYP139A_2555481387_Mycoba 140 AESLFGQRMAVHSDFLGEQLQPLLDLTHMLPQAVALQRRFRAPGWRRAEAARKRIDDLVDAQITAARADP 209

CYP139A_2545768030_Mycoba 134 AESLFGSRLAAHSDFLGEQLQPLLDLTHQLPEMVALQRRLNASGWRRAMAARQRLNDFVDALVADARTAP 203

CYP139A_2567131988_Mycoba 135 AESLFGPRLAAHSDFLGEQLQPLLDLTHRPPQLMRLQRRLNSPGWRSAMAARHRIDDLVNTVISDARARP 204

CYP139A_2587480388_Mycoba 135 AESLFGPRLAAHSDFLGEQLQPLLDLTHRPPQLMRLQRRLNSPGWRSAMAARHRIDDLVNTVISDARARP 204

CYP139A_2563577345_Mycoba 135 AESLFGPRLAAHSDFLGEQLQPLLDLTHRPPQLMRLQRRLNSPGWRSAMAARHRIDDLVNTVISDARARP 204

CYP139A_2543277028_Mycoba 135 TESLFGLRLASHSDYLGRQLQPLIDLTHRLPQVMQLQQRLNSPGWRRAMAARTRIDELIDAEIANARAGP 204

CYP139A_2563569217_Mycoba 135 TESLFGPRLASHSDYLGRQLQPMIDLTHRLPQVMQLQQRLNSPGWRRAMAARTRIDELIDAEIANARAEP 204

CYP139A_641717750_Mycobac 135 TESLFGPRLASHSDYLGRQLQPLIDLTHRLPQVMQLQQRLNSPGWRRAMAARTRIDELIDAEIANARAEP 204

CYP139A_2588629254_Mycoba 135 TESLFGLRLASHSDYLGRQLQPLIDLTHRLPQVMQLQQRLNSPGWRRAMAARTRIDELIDAEIANARAGP 204

CYP139A_2546369014_Mycoba 135 TESLFGPRLASHSDYLGRQLQPLIDLTHRLPQVMQLQQRLNSPGWRRAMAARTRIDELIDAEIANARAEP 204

CYP139A1_2555148489_Mycob 135 AESLFGQRLAVHSDFLGEQLQPLLDLTRRPPQVMRLQQRVNSPGWRRAMAARKRIDDLIDAQIADARTAP 204

CYP139A1_646010237_Mycoba 89 AESLFGQRLAVHSDFLGEQLQPLLDLTRRPPQVMRLQQRVNSPGWRRAMAARKRIDDLIDAQIADARTAP 158

CYP139A1_2581377024_Mycob 89 AESLFGQRLAVHSDFLGEQLQPLLDLTRRPPQVMRLQQRVNSPGWRRAMAARKRIDDLIDAQIADARTAP 158

CYP139A1_647209603_Mycoba 89 AESLFGQRLAVHSDFLGEQLQPLLDLTRRPPQVMRLQQRVNSPGWRRAMAARKRIDDLIDAQIADARTAP 158

CYP139A1_2537735281_Mycob 135 AESLFGQRLAVHSDFLGEQLQPLLDLTRRPPQVMRLQQRVNSPGWRRAMAARKRIDDLIDAQIADARTAP 204

CYP139A1_2576388909_Mycob 129 AESLFGQRLAVHSDFLGEQLQPLLDLTRRPPQVMRLQQRVNSPGWRRAMAARKRIDDLIDAQIADARTAP 198

CYP139A1_2577593438_Mycob 129 AESLFGQRLAVHSDFLGEQLQPLLDLTRRPPQVMRLQQRVNSPGWRRAMAARKRIDDLIDAQIADARTAP 198

CYP139A1_2577803488_Mycob 129 AESLFGQRLAVHSDFLGEQLQPLLDLTRRPPQVMRLQQRVNSPGWRRAMAARKRIDDLIDAQIADARTAP 198

CYP139A1_2581355094_Mycob 129 AESLFGQRLAVHSDFLGEQLQPLLDLTRRPPQVMRLQQRVNSPGWRRAMAARKRIDDLIDAQIADARTAP 198

CYP139A1_2584983051_Mycob 129 AESLFGQRLAVHSDFLGEQLQPLLDLTRRPPQVMRLQQRVNSPGWRRAMAARKRIDDLIDAQIADARTAP 198

CYP139A1_2584987406_Mycob 129 AESLFGQRLAVHSDFLGEQLQPLLDLTRRPPQVMRLQQRVNSPGWRRAMAARKRIDDLIDAQIADARTAP 198

CYP139A1_2589032800_Mycob 129 AESLFGQRLAVHSDFLGEQLQPLLDLTRRPPQVMRLQQRVNSPGWRRAMAARKRIDDLIDAQIADARTAP 198

CYP139A1_2592403099_Mycob 129 AESLFGQRLAVHSDFLGEQLQPLLDLTRRPPQVMRLQQRVNSPGWRRAMAARKRIDDLIDAQIADARTAP 198

CYP139A1_2592422247_Mycob 129 AESLFGQRLAVHSDFLGEQLQPLLDLTRRPPQVMRLQQRVNSPGWRRAMAARKRIDDLIDAQIADARTAP 198

CYP139A1_643734506_Mycoba 129 AESLFGQRLAVHSDFLGEQLQPLLDLTRRPPQVMRLQQRVNSPGWRRAMAARKRIDDLIDAQIADARTAP 198

CYP139A1_648335985_Mycoba 129 AESLFGQRLAVHSDFLGEQLQPLLDLTRRPPQVMRLQQRVNSPGWRRAMAARKRIDDLIDAQIADARTAP 198

CYP139A1_2574754194_Mycob 129 AESLFGQRLAVHSDFLGEQLQPLLDLTRRPPQVMRLQQRVNSPGWRRAMAARKRIDDLIDAQIADARTAP 198

CYP139A1_2575447433_Mycob 129 AESLFGQRLAVHSDFLGEQLQPLLDLTRRPPQVMRLQQRVNSPGWRRAMAARKRIDDLIDAQIADARTAP 198

CYP139A1_2575938969_Mycob 129 AESLFGQRLAVHSDFLGEQLQPLLDLTRRPPQVMRLQQRVNSPGWRRAMAARKRIDDLIDAQIADARTAP 198

CYP139A1_2576477081_Mycob 129 AESLFGQRLAVHSDFLGEQLQPLLDLTRRPPQVMRLQQRVNSPGWRRAMAARKRIDDLIDAQIADARTAP 198

CYP139A1_2576601719_Mycob 129 AESLFGQRLAVHSDFLGEQLQPLLDLTRRPPQVMRLQQRVNSPGWRRAMAARKRIDDLIDAQIADARTAP 198

CYP139A1_2577098384_Mycob 129 AESLFGQRLAVHSDFLGEQLQPLLDLTRRPPQVMRLQQRVNSPGWRRAMAARKRIDDLIDAQIADARTAP 198

CYP139A1_2578107196_Mycob 129 AESLFGQRLAVHSDFLGEQLQPLLDLTRRPPQVMRLQQRVNSPGWRRAMAARKRIDDLIDAQIADARTAP 198

CYP139A1_2584883084_Mycob 129 AESLFGQRLAVHSDFLGEQLQPLLDLTRRPPQVMRLQQRVNSPGWRRAMAARKRIDDLIDAQIADARTAP 198

CYP139A1_2588974834_Mycob 129 AESLFGQRLAVHSDFLGEQLQPLLDLTRRPPQVMRLQQRVNSPGWRRAMAARKRIDDLIDAQIADARTAP 198

CYP139A1_2589056454_Mycob 129 AESLFGQRLAVHSDFLGEQLQPLLDLTRRPPQVMRLQQRVNSPGWRRAMAARKRIDDLIDAQIADARTAP 198

CYP139A1_2589161189_Mycob 129 AESLFGQRLAVHSDFLGEQLQPLLDLTRRPPQVMRLQQRVNSPGWRRAMAARKRIDDLIDAQIADARTAP 198

CYP139A1_2590374347_Mycob 129 AESLFGQRLAVHSDFLGEQLQPLLDLTRRPPQVMRLQQRVNSPGWRRAMAARKRIDDLIDAQIADARTAP 198

CYP139A1_646018681_Mycoba 129 AESLFGQRLAVHSDFLGEQLQPLLDLTRRPPQVMRLQQRVNSPGWRRAMAARKRIDDLIDAQIADARTAP 198

CYP139A1_2575060404_Mycob 129 AESLFGQRLAVHSDFLGEQLQPLLDLTRRPPQVMRLQQRVNSPGWRRAMAARKRIDDLIDAQIADARTAP 198

CYP139A1_2576105631_Mycob 129 AESLFGQRLAVHSDFLGEQLQPLLDLTRRPPQVMRLQQRVNSPGWRRAMAARKRIDDLIDAQIADARTAP 198

CYP139A1_2576247251_Mycob 129 AESLFGQRLAVHSDFLGEQLQPLLDLTRRPPQVMRLQQRVNSPGWRRAMAARKRIDDLIDAQIADARTAP 198

CYP139A1_2576981010_Mycob 129 AESLFGQRLAVHSDFLGEQLQPLLDLTRRPPQVMRLQQRVNSPGWRRAMAARKRIDDLIDAQIADARTAP 198

CYP139A1_2577093117_Mycob 129 AESLFGQRLAVHSDFLGEQLQPLLDLTRRPPQVMRLQQRVNSPGWRRAMAARKRIDDLIDAQIADARTAP 198

CYP139A1_2577198903_Mycob 129 AESLFGQRLAVHSDFLGEQLQPLLDLTRRPPQVMRLQQRVNSPGWRRAMAARKRIDDLIDAQIADARTAP 198

CYP139A1_2577516047_Mycob 129 AESLFGQRLAVHSDFLGEQLQPLLDLTRRPPQVMRLQQRVNSPGWRRAMAARKRIDDLIDAQIADARTAP 198

CYP139A1_2578213104_Mycob 129 AESLFGQRLAVHSDFLGEQLQPLLDLTRRPPQVMRLQQRVNSPGWRRAMAARKRIDDLIDAQIADARTAP 198

CYP139A1_2584711251_Mycob 129 AESLFGQRLAVHSDFLGEQLQPLLDLTRRPPQVMRLQQRVNSPGWRRAMAARKRIDDLIDAQIADARTAP 198

CYP139A1_2584816678_Mycob 129 AESLFGQRLAVHSDFLGEQLQPLLDLTRRPPQVMRLQQRVNSPGWRRAMAARKRIDDLIDAQIADARTAP 198

CYP139A1_2589068752_Mycob 129 AESLFGQRLAVHSDFLGEQLQPLLDLTRRPPQVMRLQQRVNSPGWRRAMAARKRIDDLIDAQIADARTAP 198

CYP139A1_2589604293_Mycob 129 AESLFGQRLAVHSDFLGEQLQPLLDLTRRPPQVMRLQQRVNSPGWRRAMAARKRIDDLIDAQIADARTAP 198

CYP139A1_643028176_Mycoba 129 AESLFGQRLAVHSDFLGEQLQPLLDLTRRPPQVMRLQQRVNSPGWRRAMAARKRIDDLIDAQIADARTAP 198

CYP139A1_648476944_Mycoba 129 AESLFGQRLAVHSDFLGEQLQPLLDLTRRPPQVMRLQQRVNSPGWRRAMAARKRIDDLIDAQIADARTAP 198

CYP139A1_2511736071_Mycob 129 AESLFGQRLAVHSDFLGEQLQPLLDLTRRPPQVMRLQQRVNSPGWRRAMAARKRIDDLIDAQIADARTAP 198

CYP139A1_2546206123_Mycob 129 AESLFGQRLAVHSDFLGEQLQPLLDLTRRPPQVMRLQQRVNSPGWRRAMAARKRIDDLIDAQIADARTAP 198

CYP139A1_2574780327_Mycob 129 AESLFGQRLAVHSDFLGEQLQPLLDLTRRPPQVMRLQQRVNSPGWRRAMAARKRIDDLIDAQIADARTAP 198

CYP139A1_2575978404_Mycob 129 AESLFGQRLAVHSDFLGEQLQPLLDLTRRPPQVMRLQQRVNSPGWRRAMAARKRIDDLIDAQIADARTAP 198

CYP139A1_2576675825_Mycob 129 AESLFGQRLAVHSDFLGEQLQPLLDLTRRPPQVMRLQQRVNSPGWRRAMAARKRIDDLIDAQIADARTAP 198

CYP139A1_2576947708_Mycob 129 AESLFGQRLAVHSDFLGEQLQPLLDLTRRPPQVMRLQQRVNSPGWRRAMAARKRIDDLIDAQIADARTAP 198

CYP139A1_2577400922_Mycob 129 AESLFGQRLAVHSDFLGEQLQPLLDLTRRPPQVMRLQQRVNSPGWRRAMAARKRIDDLIDAQIADARTAP 198

CYP139A1_2577893113_Mycob 129 AESLFGQRLAVHSDFLGEQLQPLLDLTRRPPQVMRLQQRVNSPGWRRAMAARKRIDDLIDAQIADARTAP 198

CYP139A1_2584759228_Mycob 129 AESLFGQRLAVHSDFLGEQLQPLLDLTRRPPQVMRLQQRVNSPGWRRAMAARKRIDDLIDAQIADARTAP 198

CYP139A1_2584801008_Mycob 129 AESLFGQRLAVHSDFLGEQLQPLLDLTRRPPQVMRLQQRVNSPGWRRAMAARKRIDDLIDAQIADARTAP 198

CYP139A1_2584946269_Mycob 129 AESLFGQRLAVHSDFLGEQLQPLLDLTRRPPQVMRLQQRVNSPGWRRAMAARKRIDDLIDAQIADARTAP 198

CYP139A1_2589125802_Mycob 129 AESLFGQRLAVHSDFLGEQLQPLLDLTRRPPQVMRLQQRVNSPGWRRAMAARKRIDDLIDAQIADARTAP 198

CYP139A1_2589654545_Mycob 129 AESLFGQRLAVHSDFLGEQLQPLLDLTRRPPQVMRLQQRVNSPGWRRAMAARKRIDDLIDAQIADARTAP 198

CYP139A1_2590190898_Mycob 129 AESLFGQRLAVHSDFLGEQLQPLLDLTRRPPQVMRLQQRVNSPGWRRAMAARKRIDDLIDAQIADARTAP 198

CYP139A1_2592267285_Mycob 129 AESLFGQRLAVHSDFLGEQLQPLLDLTRRPPQVMRLQQRVNSPGWRRAMAARKRIDDLIDAQIADARTAP 198

CYP139A1_2592579018_Mycob 129 AESLFGQRLAVHSDFLGEQLQPLLDLTRRPPQVMRLQQRVNSPGWRRAMAARKRIDDLIDAQIADARTAP 198

CYP139A1_2574803240_Mycob 129 AESLFGQRLAVHSDFLGEQLQPLLDLTRRPPQVMRLQQRVNSPGWRRAMAARKRIDDLIDAQIADARTAP 198

CYP139A1_2575138339_Mycob 129 AESLFGQRLAVHSDFLGEQLQPLLDLTRRPPQVMRLQQRVNSPGWRRAMAARKRIDDLIDAQIADARTAP 198

CYP139A1_2575935659_Mycob 129 AESLFGQRLAVHSDFLGEQLQPLLDLTRRPPQVMRLQQRVNSPGWRRAMAARKRIDDLIDAQIADARTAP 198

CYP139A1_2576703024_Mycob 129 AESLFGQRLAVHSDFLGEQLQPLLDLTRRPPQVMRLQQRVNSPGWRRAMAARKRIDDLIDAQIADARTAP 198

CYP139A1_2577143911_Mycob 129 AESLFGQRLAVHSDFLGEQLQPLLDLTRRPPQVMRLQQRVNSPGWRRAMAARKRIDDLIDAQIADARTAP 198

CYP139A1_2577175183_Mycob 129 AESLFGQRLAVHSDFLGEQLQPLLDLTRRPPQVMRLQQRVNSPGWRRAMAARKRIDDLIDAQIADARTAP 198

CYP139A1_2577879644_Mycob 129 AESLFGQRLAVHSDFLGEQLQPLLDLTRRPPQVMRLQQRVNSPGWRRAMAARKRIDDLIDAQIADARTAP 198

CYP139A1_2577954418_Mycob 129 AESLFGQRLAVHSDFLGEQLQPLLDLTRRPPQVMRLQQRVNSPGWRRAMAARKRIDDLIDAQIADARTAP 198

CYP139A1_2584625495_Mycob 129 AESLFGQRLAVHSDFLGEQLQPLLDLTRRPPQVMRLQQRVNSPGWRRAMAARKRIDDLIDAQIADARTAP 198

CYP139A1_2589526877_Mycob 129 AESLFGQRLAVHSDFLGEQLQPLLDLTRRPPQVMRLQQRVNSPGWRRAMAARKRIDDLIDAQIADARTAP 198

CYP139A1_2589711853_Mycob 129 AESLFGQRLAVHSDFLGEQLQPLLDLTRRPPQVMRLQQRVNSPGWRRAMAARKRIDDLIDAQIADARTAP 198

CYP139A1_2590113796_Mycob 129 AESLFGQRLAVHSDFLGEQLQPLLDLTRRPPQVMRLQQRVNSPGWRRAMAARKRIDDLIDAQIADARTAP 198

CYP139A1_2592324422_Mycob 129 AESLFGQRLAVHSDFLGEQLQPLLDLTRRPPQVMRLQQRVNSPGWRRAMAARKRIDDLIDAQIADARTAP 198

CYP139A1_2592337997_Mycob 129 AESLFGQRLAVHSDFLGEQLQPLLDLTRRPPQVMRLQQRVNSPGWRRAMAARKRIDDLIDAQIADARTAP 198

CYP139A1_2592373455_Mycob 129 AESLFGQRLAVHSDFLGEQLQPLLDLTRRPPQVMRLQQRVNSPGWRRAMAARKRIDDLIDAQIADARTAP 198

CYP139A1_2592445915_Mycob 129 AESLFGQRLAVHSDFLGEQLQPLLDLTRRPPQVMRLQQRVNSPGWRRAMAARKRIDDLIDAQIADARTAP 198

CYP139A1_2575023271_Mycob 129 AESLFGQRLAVHSDFLGEQLQPLLDLTRRPPQVMRLQQRVNSPGWRRAMAARKRIDDLIDAQIADARTAP 198

CYP139A1_2575619239_Mycob 129 AESLFGQRLAVHSDFLGEQLQPLLDLTRRPPQVMRLQQRVNSPGWRRAMAARKRIDDLIDAQIADARTAP 198

CYP139A1_2575786887_Mycob 129 AESLFGQRLAVHSDFLGEQLQPLLDLTRRPPQVMRLQQRVNSPGWRRAMAARKRIDDLIDAQIADARTAP 198

CYP139A1_2576882264_Mycob 129 AESLFGQRLAVHSDFLGEQLQPLLDLTRRPPQVMRLQQRVNSPGWRRAMAARKRIDDLIDAQIADARTAP 198

CYP139A1_2577215885_Mycob 129 AESLFGQRLAVHSDFLGEQLQPLLDLTRRPPQVMRLQQRVNSPGWRRAMAARKRIDDLIDAQIADARTAP 198

CYP139A1_2577627248_Mycob 129 AESLFGQRLAVHSDFLGEQLQPLLDLTRRPPQVMRLQQRVNSPGWRRAMAARKRIDDLIDAQIADARTAP 198

CYP139A1_2577923998_Mycob 129 AESLFGQRLAVHSDFLGEQLQPLLDLTRRPPQVMRLQQRVNSPGWRRAMAARKRIDDLIDAQIADARTAP 198

CYP139A1_2583735989_Mycob 129 AESLFGQRLAVHSDFLGEQLQPLLDLTRRPPQVMRLQQRVNSPGWRRAMAARKRIDDLIDAQIADARTAP 198

CYP139A1_2584003651_Mycob 129 AESLFGQRLAVHSDFLGEQLQPLLDLTRRPPQVMRLQQRVNSPGWRRAMAARKRIDDLIDAQIADARTAP 198

CYP139A1_2584623655_Mycob 129 AESLFGQRLAVHSDFLGEQLQPLLDLTRRPPQVMRLQQRVNSPGWRRAMAARKRIDDLIDAQIADARTAP 198

CYP139A1_2584739857_Mycob 129 AESLFGQRLAVHSDFLGEQLQPLLDLTRRPPQVMRLQQRVNSPGWRRAMAARKRIDDLIDAQIADARTAP 198

CYP139A1_2584858071_Mycob 129 AESLFGQRLAVHSDFLGEQLQPLLDLTRRPPQVMRLQQRVNSPGWRRAMAARKRIDDLIDAQIADARTAP 198

CYP139A1_2584928246_Mycob 129 AESLFGQRLAVHSDFLGEQLQPLLDLTRRPPQVMRLQQRVNSPGWRRAMAARKRIDDLIDAQIADARTAP 198

CYP139A1_2589040293_Mycob 129 AESLFGQRLAVHSDFLGEQLQPLLDLTRRPPQVMRLQQRVNSPGWRRAMAARKRIDDLIDAQIADARTAP 198

CYP139A1_2589658610_Mycob 129 AESLFGQRLAVHSDFLGEQLQPLLDLTRRPPQVMRLQQRVNSPGWRRAMAARKRIDDLIDAQIADARTAP 198

CYP139A1_2590162679_Mycob 129 AESLFGQRLAVHSDFLGEQLQPLLDLTRRPPQVMRLQQRVNSPGWRRAMAARKRIDDLIDAQIADARTAP 198

CYP139A1_2590243884_Mycob 129 AESLFGQRLAVHSDFLGEQLQPLLDLTRRPPQVMRLQQRVNSPGWRRAMAARKRIDDLIDAQIADARTAP 198

CYP139A1_2590531681_Mycob 129 AESLFGQRLAVHSDFLGEQLQPLLDLTRRPPQVMRLQQRVNSPGWRRAMAARKRIDDLIDAQIADARTAP 198

CYP139A1_2574614619_Mycob 129 AESLFGQRLAVHSDFLGEQLQPLLDLTRRPPQVMRLQQRVNSPGWRRAMAARKRIDDLIDAQIADARTAP 198

CYP139A1_2575016195_Mycob 129 AESLFGQRLAVHSDFLGEQLQPLLDLTRRPPQVMRLQQRVNSPGWRRAMAARKRIDDLIDAQIADARTAP 198

CYP139A1_2575426848_Mycob 129 AESLFGQRLAVHSDFLGEQLQPLLDLTRRPPQVMRLQQRVNSPGWRRAMAARKRIDDLIDAQIADARTAP 198

CYP139A1_2576630848_Mycob 129 AESLFGQRLAVHSDFLGEQLQPLLDLTRRPPQVMRLQQRVNSPGWRRAMAARKRIDDLIDAQIADARTAP 198

CYP139A1_2577468911_Mycob 129 AESLFGQRLAVHSDFLGEQLQPLLDLTRRPPQVMRLQQRVNSPGWRRAMAARKRIDDLIDAQIADARTAP 198

CYP139A1_2577974906_Mycob 129 AESLFGQRLAVHSDFLGEQLQPLLDLTRRPPQVMRLQQRVNSPGWRRAMAARKRIDDLIDAQIADARTAP 198

CYP139A1_2578062326_Mycob 129 AESLFGQRLAVHSDFLGEQLQPLLDLTRRPPQVMRLQQRVNSPGWRRAMAARKRIDDLIDAQIADARTAP 198

CYP139A1_2584836956_Mycob 129 AESLFGQRLAVHSDFLGEQLQPLLDLTRRPPQVMRLQQRVNSPGWRRAMAARKRIDDLIDAQIADARTAP 198

CYP139A1_2584998642_Mycob 129 AESLFGQRLAVHSDFLGEQLQPLLDLTRRPPQVMRLQQRVNSPGWRRAMAARKRIDDLIDAQIADARTAP 198

CYP139A1_2589130082_Mycob 129 AESLFGQRLAVHSDFLGEQLQPLLDLTRRPPQVMRLQQRVNSPGWRRAMAARKRIDDLIDAQIADARTAP 198

CYP139A1_2589592089_Mycob 129 AESLFGQRLAVHSDFLGEQLQPLLDLTRRPPQVMRLQQRVNSPGWRRAMAARKRIDDLIDAQIADARTAP 198

CYP139A1_2590052832_Mycob 129 AESLFGQRLAVHSDFLGEQLQPLLDLTRRPPQVMRLQQRVNSPGWRRAMAARKRIDDLIDAQIADARTAP 198

CYP139A1_2590505688_Mycob 129 AESLFGQRLAVHSDFLGEQLQPLLDLTRRPPQVMRLQQRVNSPGWRRAMAARKRIDDLIDAQIADARTAP 198

CYP139A1_2592353030_Mycob 129 AESLFGQRLAVHSDFLGEQLQPLLDLTRRPPQVMRLQQRVNSPGWRRAMAARKRIDDLIDAQIADARTAP 198

CYP139A1_637026884_Mycoba 129 AESLFGQRLAVHSDFLGEQLQPLLDLTRRPPQVMRLQQRVNSPGWRRAMAARKRIDDLIDAQIADARTAP 198

CYP139A1_2574886309_Mycob 129 AESLFGQRLAVHSDFLGEQLQPLLDLTRRPPQVMRLQQRVNSPGWRRAMAARKRIDDLIDAQIADARTAP 198

CYP139A1_2575295342_Mycob 129 AESLFGQRLAVHSDFLGEQLQPLLDLTRRPPQVMRLQQRVNSPGWRRAMAARKRIDDLIDAQIADARTAP 198

CYP139A1_2575942274_Mycob 129 AESLFGQRLAVHSDFLGEQLQPLLDLTRRPPQVMRLQQRVNSPGWRRAMAARKRIDDLIDAQIADARTAP 198

CYP139A1_2576123248_Mycob 129 AESLFGQRLAVHSDFLGEQLQPLLDLTRRPPQVMRLQQRVNSPGWRRAMAARKRIDDLIDAQIADARTAP 198

CYP139A1_2576712596_Mycob 129 AESLFGQRLAVHSDFLGEQLQPLLDLTRRPPQVMRLQQRVNSPGWRRAMAARKRIDDLIDAQIADARTAP 198

CYP139A1_2577856904_Mycob 129 AESLFGQRLAVHSDFLGEQLQPLLDLTRRPPQVMRLQQRVNSPGWRRAMAARKRIDDLIDAQIADARTAP 198

CYP139A1_2578013153_Mycob 129 AESLFGQRLAVHSDFLGEQLQPLLDLTRRPPQVMRLQQRVNSPGWRRAMAARKRIDDLIDAQIADARTAP 198

CYP139A1_2578182623_Mycob 129 AESLFGQRLAVHSDFLGEQLQPLLDLTRRPPQVMRLQQRVNSPGWRRAMAARKRIDDLIDAQIADARTAP 198

CYP139A1_2580771058_Mycob 129 AESLFGQRLAVHSDFLGEQLQPLLDLTRRPPQVMRLQQRVNSPGWRRAMAARKRIDDLIDAQIADARTAP 198

CYP139A1_2584641128_Mycob 129 AESLFGQRLAVHSDFLGEQLQPLLDLTRRPPQVMRLQQRVNSPGWRRAMAARKRIDDLIDAQIADARTAP 198

CYP139A1_2584649146_Mycob 129 AESLFGQRLAVHSDFLGEQLQPLLDLTRRPPQVMRLQQRVNSPGWRRAMAARKRIDDLIDAQIADARTAP 198

CYP139A1_2584660713_Mycob 129 AESLFGQRLAVHSDFLGEQLQPLLDLTRRPPQVMRLQQRVNSPGWRRAMAARKRIDDLIDAQIADARTAP 198

CYP139A1_2584967006_Mycob 129 AESLFGQRLAVHSDFLGEQLQPLLDLTRRPPQVMRLQQRVNSPGWRRAMAARKRIDDLIDAQIADARTAP 198

CYP139A1_2584970624_Mycob 129 AESLFGQRLAVHSDFLGEQLQPLLDLTRRPPQVMRLQQRVNSPGWRRAMAARKRIDDLIDAQIADARTAP 198

CYP139A1_2589053585_Mycob 129 AESLFGQRLAVHSDFLGEQLQPLLDLTRRPPQVMRLQQRVNSPGWRRAMAARKRIDDLIDAQIADARTAP 198

CYP139A1_2589498327_Mycob 129 AESLFGQRLAVHSDFLGEQLQPLLDLTRRPPQVMRLQQRVNSPGWRRAMAARKRIDDLIDAQIADARTAP 198

CYP139A1_2589563529_Mycob 129 AESLFGQRLAVHSDFLGEQLQPLLDLTRRPPQVMRLQQRVNSPGWRRAMAARKRIDDLIDAQIADARTAP 198

CYP139A1_2590377218_Mycob 129 AESLFGQRLAVHSDFLGEQLQPLLDLTRRPPQVMRLQQRVNSPGWRRAMAARKRIDDLIDAQIADARTAP 198

CYP139A1_2592283614_Mycob 129 AESLFGQRLAVHSDFLGEQLQPLLDLTRRPPQVMRLQQRVNSPGWRRAMAARKRIDDLIDAQIADARTAP 198

CYP139A1_2592319784_Mycob 129 AESLFGQRLAVHSDFLGEQLQPLLDLTRRPPQVMRLQQRVNSPGWRRAMAARKRIDDLIDAQIADARTAP 198

CYP139A1_2575106637_Mycob 129 AESLFGQRLAVHSDFLGEQLQPLLDLTRRPPQVMRLQQRVNSPGWRRAMAARKRIDDLIDAQIADARTAP 198

CYP139A1_2575157076_Mycob 129 AESLFGQRLAVHSDFLGEQLQPLLDLTRRPPQVMRLQQRVNSPGWRRAMAARKRIDDLIDAQIADARTAP 198

CYP139A1_2575361778_Mycob 129 AESLFGQRLAVHSDFLGEQLQPLLDLTRRPPQVMRLQQRVNSPGWRRAMAARKRIDDLIDAQIADARTAP 198

CYP139A1_2576009184_Mycob 129 AESLFGQRLAVHSDFLGEQLQPLLDLTRRPPQVMRLQQRVNSPGWRRAMAARKRIDDLIDAQIADARTAP 198

CYP139A1_2576566954_Mycob 129 AESLFGQRLAVHSDFLGEQLQPLLDLTRRPPQVMRLQQRVNSPGWRRAMAARKRIDDLIDAQIADARTAP 198

CYP139A1_2576731741_Mycob 129 AESLFGQRLAVHSDFLGEQLQPLLDLTRRPPQVMRLQQRVNSPGWRRAMAARKRIDDLIDAQIADARTAP 198

CYP139A1_2581562358_Mycob 129 AESLFGQRLAVHSDFLGEQLQPLLDLTRRPPQVMRLQQRVNSPGWRRAMAARKRIDDLIDAQIADARTAP 198

CYP139A1_2584107430_Mycob 129 AESLFGQRLAVHSDFLGEQLQPLLDLTRRPPQVMRLQQRVNSPGWRRAMAARKRIDDLIDAQIADARTAP 198

CYP139A1_2590025444_Mycob 129 AESLFGQRLAVHSDFLGEQLQPLLDLTRRPPQVMRLQQRVNSPGWRRAMAARKRIDDLIDAQIADARTAP 198

CYP139A1_2590040558_Mycob 129 AESLFGQRLAVHSDFLGEQLQPLLDLTRRPPQVMRLQQRVNSPGWRRAMAARKRIDDLIDAQIADARTAP 198

CYP139A1_2590214646_Mycob 129 AESLFGQRLAVHSDFLGEQLQPLLDLTRRPPQVMRLQQRVNSPGWRRAMAARKRIDDLIDAQIADARTAP 198

CYP139A1_2590223019_Mycob 129 AESLFGQRLAVHSDFLGEQLQPLLDLTRRPPQVMRLQQRVNSPGWRRAMAARKRIDDLIDAQIADARTAP 198

CYP139A1_2590266966_Mycob 129 AESLFGQRLAVHSDFLGEQLQPLLDLTRRPPQVMRLQQRVNSPGWRRAMAARKRIDDLIDAQIADARTAP 198

CYP139A1_647086307_Mycoba 129 AESLFGQRLAVHSDFLGEQLQPLLDLTRRPPQVMRLQQRVNSPGWRRAMAARKRIDDLIDAQIADARTAP 198

CYP139A1_2574726119_Mycob 129 AESLFGQRLAVHSDFLGEQLQPLLDLTRRPPQVMRLQQRVNSPGWRRAMAARKRIDDLIDAQIADARTAP 198

CYP139A1_2574757270_Mycob 129 AESLFGQRLAVHSDFLGEQLQPLLDLTRRPPQVMRLQQRVNSPGWRRAMAARKRIDDLIDAQIADARTAP 198

CYP139A1_2575280304_Mycob 129 AESLFGQRLAVHSDFLGEQLQPLLDLTRRPPQVMRLQQRVNSPGWRRAMAARKRIDDLIDAQIADARTAP 198

CYP139A1_2575601683_Mycob 129 AESLFGQRLAVHSDFLGEQLQPLLDLTRRPPQVMRLQQRVNSPGWRRAMAARKRIDDLIDAQIADARTAP 198

CYP139A1_2576158036_Mycob 129 AESLFGQRLAVHSDFLGEQLQPLLDLTRRPPQVMRLQQRVNSPGWRRAMAARKRIDDLIDAQIADARTAP 198

CYP139A1_2577689111_Mycob 129 AESLFGQRLAVHSDFLGEQLQPLLDLTRRPPQVMRLQQRVNSPGWRRAMAARKRIDDLIDAQIADARTAP 198

CYP139A1_2577751179_Mycob 129 AESLFGQRLAVHSDFLGEQLQPLLDLTRRPPQVMRLQQRVNSPGWRRAMAARKRIDDLIDAQIADARTAP 198

CYP139A1_2577845812_Mycob 129 AESLFGQRLAVHSDFLGEQLQPLLDLTRRPPQVMRLQQRVNSPGWRRAMAARKRIDDLIDAQIADARTAP 198

CYP139A1_2577900964_Mycob 129 AESLFGQRLAVHSDFLGEQLQPLLDLTRRPPQVMRLQQRVNSPGWRRAMAARKRIDDLIDAQIADARTAP 198

CYP139A1_2577988240_Mycob 129 AESLFGQRLAVHSDFLGEQLQPLLDLTRRPPQVMRLQQRVNSPGWRRAMAARKRIDDLIDAQIADARTAP 198

CYP139A1_2578237814_Mycob 129 AESLFGQRLAVHSDFLGEQLQPLLDLTRRPPQVMRLQQRVNSPGWRRAMAARKRIDDLIDAQIADARTAP 198

CYP139A1_2584703088_Mycob 129 AESLFGQRLAVHSDFLGEQLQPLLDLTRRPPQVMRLQQRVNSPGWRRAMAARKRIDDLIDAQIADARTAP 198

CYP139A1_2584776403_Mycob 129 AESLFGQRLAVHSDFLGEQLQPLLDLTRRPPQVMRLQQRVNSPGWRRAMAARKRIDDLIDAQIADARTAP 198

CYP139A1_2584785697_Mycob 129 AESLFGQRLAVHSDFLGEQLQPLLDLTRRPPQVMRLQQRVNSPGWRRAMAARKRIDDLIDAQIADARTAP 198

CYP139A1_2584898862_Mycob 129 AESLFGQRLAVHSDFLGEQLQPLLDLTRRPPQVMRLQQRVNSPGWRRAMAARKRIDDLIDAQIADARTAP 198

CYP139A1_2584906709_Mycob 129 AESLFGQRLAVHSDFLGEQLQPLLDLTRRPPQVMRLQQRVNSPGWRRAMAARKRIDDLIDAQIADARTAP 198

CYP139A1_2589082097_Mycob 129 AESLFGQRLAVHSDFLGEQLQPLLDLTRRPPQVMRLQQRVNSPGWRRAMAARKRIDDLIDAQIADARTAP 198

CYP139A1_2589142105_Mycob 129 AESLFGQRLAVHSDFLGEQLQPLLDLTRRPPQVMRLQQRVNSPGWRRAMAARKRIDDLIDAQIADARTAP 198

CYP139A1_2589707360_Mycob 129 AESLFGQRLAVHSDFLGEQLQPLLDLTRRPPQVMRLQQRVNSPGWRRAMAARKRIDDLIDAQIADARTAP 198

CYP139A1_2590142346_Mycob 129 AESLFGQRLAVHSDFLGEQLQPLLDLTRRPPQVMRLQQRVNSPGWRRAMAARKRIDDLIDAQIADARTAP 198

CYP139A1_2592254166_Mycob 129 AESLFGQRLAVHSDFLGEQLQPLLDLTRRPPQVMRLQQRVNSPGWRRAMAARKRIDDLIDAQIADARTAP 198

CYP139A1_2592348241_Mycob 129 AESLFGQRLAVHSDFLGEQLQPLLDLTRRPPQVMRLQQRVNSPGWRRAMAARKRIDDLIDAQIADARTAP 198

CYP139A1_2592364277_Mycob 129 AESLFGQRLAVHSDFLGEQLQPLLDLTRRPPQVMRLQQRVNSPGWRRAMAARKRIDDLIDAQIADARTAP 198

CYP139A1_2592537861_Mycob 129 AESLFGQRLAVHSDFLGEQLQPLLDLTRRPPQVMRLQQRVNSPGWRRAMAARKRIDDLIDAQIADARTAP 198

CYP139A1_2546454904_Mycob 129 AESLFGQRLAVHSDFLGEQLQPLLDLTRRPPQVMRLQQRVNSPGWRRAMAARKRIDDLIDAQIADARTAP 198

CYP139A1_2577218717_Mycob 129 AESLFGQRLAVHSDFLGEQLQPLLDLTRRPPQVMRLQQRVNSPGWRRAMAARKRIDDLIDAQIADARTAP 198

CYP139A1_2577720788_Mycob 129 AESLFGQRLAVHSDFLGEQLQPLLDLTRRPPQVMRLQQRVNSPGWRRAMAARKRIDDLIDAQIADARTAP 198

CYP139A1_2581366557_Mycob 129 AESLFGQRLAVHSDFLGEQLQPLLDLTRRPPQVMRLQQRVNSPGWRRAMAARKRIDDLIDAQIADARTAP 198

CYP139A1_2584638962_Mycob 129 AESLFGQRLAVHSDFLGEQLQPLLDLTRRPPQVMRLQQRVNSPGWRRAMAARKRIDDLIDAQIADARTAP 198

CYP139A1_2590154524_Mycob 129 AESLFGQRLAVHSDFLGEQLQPLLDLTRRPPQVMRLQQRVNSPGWRRAMAARKRIDDLIDAQIADARTAP 198

CYP139A1_2590260165_Mycob 129 AESLFGQRLAVHSDFLGEQLQPLLDLTRRPPQVMRLQQRVNSPGWRRAMAARKRIDDLIDAQIADARTAP 198

CYP139A1_2590539845_Mycob 129 AESLFGQRLAVHSDFLGEQLQPLLDLTRRPPQVMRLQQRVNSPGWRRAMAARKRIDDLIDAQIADARTAP 198

CYP139A1_641783198_Mycoba 129 AESLFGQRLAVHSDFLGEQLQPLLDLTRRPPQVMRLQQRVNSPGWRRAMAARKRIDDLIDAQIADARTAP 198

CYP139A1_643031783_Mycoba 129 AESLFGQRLAVHSDFLGEQLQPLLDLTRRPPQVMRLQQRVNSPGWRRAMAARKRIDDLIDAQIADARTAP 198

CYP139A1_651039004_Mycoba 129 AESLFGQRLAVHSDFLGEQLQPLLDLTRRPPQVMRLQQRVNSPGWRRAMAARKRIDDLIDAQIADARTAP 198

CYP139A1_2574843285_Mycob 129 AESLFGQRLAVHSDFLGEQLQPLLDLTRRPPQVMRLQQRVNSPGWRRAMAARKRIDDLIDAQIADARTAP 198

CYP139A1_2575542837_Mycob 129 AESLFGQRLAVHSDFLGEQLQPLLDLTRRPPQVMRLQQRVNSPGWRRAMAARKRIDDLIDAQIADARTAP 198

CYP139A1_2575709449_Mycob 129 AESLFGQRLAVHSDFLGEQLQPLLDLTRRPPQVMRLQQRVNSPGWRRAMAARKRIDDLIDAQIADARTAP 198

CYP139A1_2576051820_Mycob 129 AESLFGQRLAVHSDFLGEQLQPLLDLTRRPPQVMRLQQRVNSPGWRRAMAARKRIDDLIDAQIADARTAP 198

CYP139A1_2578094092_Mycob 129 AESLFGQRLAVHSDFLGEQLQPLLDLTRRPPQVMRLQQRVNSPGWRRAMAARKRIDDLIDAQIADARTAP 198

CYP139A1_2584617586_Mycob 129 AESLFGQRLAVHSDFLGEQLQPLLDLTRRPPQVMRLQQRVNSPGWRRAMAARKRIDDLIDAQIADARTAP 198

CYP139A1_2584694835_Mycob 129 AESLFGQRLAVHSDFLGEQLQPLLDLTRRPPQVMRLQQRVNSPGWRRAMAARKRIDDLIDAQIADARTAP 198

CYP139A1_2584822720_Mycob 129 AESLFGQRLAVHSDFLGEQLQPLLDLTRRPPQVMRLQQRVNSPGWRRAMAARKRIDDLIDAQIADARTAP 198

CYP139A1_2584931720_Mycob 129 AESLFGQRLAVHSDFLGEQLQPLLDLTRRPPQVMRLQQRVNSPGWRRAMAARKRIDDLIDAQIADARTAP 198

CYP139A1_2589165303_Mycob 129 AESLFGQRLAVHSDFLGEQLQPLLDLTRRPPQVMRLQQRVNSPGWRRAMAARKRIDDLIDAQIADARTAP 198

CYP139A1_2589608377_Mycob 129 AESLFGQRLAVHSDFLGEQLQPLLDLTRRPPQVMRLQQRVNSPGWRRAMAARKRIDDLIDAQIADARTAP 198

CYP139A1_2589638476_Mycob 129 AESLFGQRLAVHSDFLGEQLQPLLDLTRRPPQVMRLQQRVNSPGWRRAMAARKRIDDLIDAQIADARTAP 198

CYP139A1_2589687166_Mycob 129 AESLFGQRLAVHSDFLGEQLQPLLDLTRRPPQVMRLQQRVNSPGWRRAMAARKRIDDLIDAQIADARTAP 198

CYP139A1_2590174897_Mycob 129 AESLFGQRLAVHSDFLGEQLQPLLDLTRRPPQVMRLQQRVNSPGWRRAMAARKRIDDLIDAQIADARTAP 198

CYP139A1_2590370532_Mycob 129 AESLFGQRLAVHSDFLGEQLQPLLDLTRRPPQVMRLQQRVNSPGWRRAMAARKRIDDLIDAQIADARTAP 198

CYP139A1_2592328029_Mycob 129 AESLFGQRLAVHSDFLGEQLQPLLDLTRRPPQVMRLQQRVNSPGWRRAMAARKRIDDLIDAQIADARTAP 198

CYP139A1_2592426321_Mycob 129 AESLFGQRLAVHSDFLGEQLQPLLDLTRRPPQVMRLQQRVNSPGWRRAMAARKRIDDLIDAQIADARTAP 198

CYP139A1_2575094331_Mycob 129 AESLFGQRLAVHSDFLGEQLQPLLDLTRRPPQVMRLQQRVNSPGWRRAMAARKRIDDLIDAQIADARTAP 198

CYP139A1_2576101264_Mycob 129 AESLFGQRLAVHSDFLGEQLQPLLDLTRRPPQVMRLQQRVNSPGWRRAMAARKRIDDLIDAQIADARTAP 198

CYP139A1_2576927477_Mycob 129 AESLFGQRLAVHSDFLGEQLQPLLDLTRRPPQVMRLQQRVNSPGWRRAMAARKRIDDLIDAQIADARTAP 198

CYP139A1_2577009596_Mycob 129 AESLFGQRLAVHSDFLGEQLQPLLDLTRRPPQVMRLQQRVNSPGWRRAMAARKRIDDLIDAQIADARTAP 198

CYP139A1_2577884547_Mycob 129 AESLFGQRLAVHSDFLGEQLQPLLDLTRRPPQVMRLQQRVNSPGWRRAMAARKRIDDLIDAQIADARTAP 198

CYP139A1_2577997123_Mycob 129 AESLFGQRLAVHSDFLGEQLQPLLDLTRRPPQVMRLQQRVNSPGWRRAMAARKRIDDLIDAQIADARTAP 198

CYP139A1_2578155745_Mycob 129 AESLFGQRLAVHSDFLGEQLQPLLDLTRRPPQVMRLQQRVNSPGWRRAMAARKRIDDLIDAQIADARTAP 198

CYP139A1_2581807696_Mycob 129 AESLFGQRLAVHSDFLGEQLQPLLDLTRRPPQVMRLQQRVNSPGWRRAMAARKRIDDLIDAQIADARTAP 198

CYP139A1_2582018155_Mycob 129 AESLFGQRLAVHSDFLGEQLQPLLDLTRRPPQVMRLQQRVNSPGWRRAMAARKRIDDLIDAQIADARTAP 198

CYP139A1_2589154518_Mycob 129 AESLFGQRLAVHSDFLGEQLQPLLDLTRRPPQVMRLQQRVNSPGWRRAMAARKRIDDLIDAQIADARTAP 198

CYP139A1_2590109466_Mycob 129 AESLFGQRLAVHSDFLGEQLQPLLDLTRRPPQVMRLQQRVNSPGWRRAMAARKRIDDLIDAQIADARTAP 198

CYP139A1_2590195268_Mycob 129 AESLFGQRLAVHSDFLGEQLQPLLDLTRRPPQVMRLQQRVNSPGWRRAMAARKRIDDLIDAQIADARTAP 198

CYP139A1_2590198349_Mycob 129 AESLFGQRLAVHSDFLGEQLQPLLDLTRRPPQVMRLQQRVNSPGWRRAMAARKRIDDLIDAQIADARTAP 198

CYP139A1_2574799840_Mycob 129 AESLFGQRLAVHSDFLGEQLQPLLDLTRRPPQVMRLQQRVNSPGWRRAMAARKRIDDLIDAQIADARTAP 198

CYP139A1_2576399693_Mycob 129 AESLFGQRLAVHSDFLGEQLQPLLDLTRRPPQVMRLQQRVNSPGWRRAMAARKRIDDLIDAQIADARTAP 198

CYP139A1_2577024745_Mycob 129 AESLFGQRLAVHSDFLGEQLQPLLDLTRRPPQVMRLQQRVNSPGWRRAMAARKRIDDLIDAQIADARTAP 198

CYP139A1_2577872587_Mycob 129 AESLFGQRLAVHSDFLGEQLQPLLDLTRRPPQVMRLQQRVNSPGWRRAMAARKRIDDLIDAQIADARTAP 198

CYP139A1_2579813772_Mycob 129 AESLFGQRLAVHSDFLGEQLQPLLDLTRRPPQVMRLQQRVNSPGWRRAMAARKRIDDLIDAQIADARTAP 198

CYP139A1_2584772421_Mycob 129 AESLFGQRLAVHSDFLGEQLQPLLDLTRRPPQVMRLQQRVNSPGWRRAMAARKRIDDLIDAQIADARTAP 198

CYP139A1_2584893836_Mycob 129 AESLFGQRLAVHSDFLGEQLQPLLDLTRRPPQVMRLQQRVNSPGWRRAMAARKRIDDLIDAQIADARTAP 198

CYP139A1_2589026697_Mycob 129 AESLFGQRLAVHSDFLGEQLQPLLDLTRRPPQVMRLQQRVNSPGWRRAMAARKRIDDLIDAQIADARTAP 198

CYP139A1_2589514641_Mycob 129 AESLFGQRLAVHSDFLGEQLQPLLDLTRRPPQVMRLQQRVNSPGWRRAMAARKRIDDLIDAQIADARTAP 198

CYP139A1_2589547247_Mycob 129 AESLFGQRLAVHSDFLGEQLQPLLDLTRRPPQVMRLQQRVNSPGWRRAMAARKRIDDLIDAQIADARTAP 198

CYP139A1_2589620637_Mycob 129 AESLFGQRLAVHSDFLGEQLQPLLDLTRRPPQVMRLQQRVNSPGWRRAMAARKRIDDLIDAQIADARTAP 198

CYP139A1_2592230560_Mycob 129 AESLFGQRLAVHSDFLGEQLQPLLDLTRRPPQVMRLQQRVNSPGWRRAMAARKRIDDLIDAQIADARTAP 198

CYP139A1_2592242791_Mycob 129 AESLFGQRLAVHSDFLGEQLQPLLDLTRRPPQVMRLQQRVNSPGWRRAMAARKRIDDLIDAQIADARTAP 198

CYP139A1_2592299949_Mycob 129 AESLFGQRLAVHSDFLGEQLQPLLDLTRRPPQVMRLQQRVNSPGWRRAMAARKRIDDLIDAQIADARTAP 198

CYP139A1_2592377452_Mycob 129 AESLFGQRLAVHSDFLGEQLQPLLDLTRRPPQVMRLQQRVNSPGWRRAMAARKRIDDLIDAQIADARTAP 198

CYP139A1_2592405959_Mycob 129 AESLFGQRLAVHSDFLGEQLQPLLDLTRRPPQVMRLQQRVNSPGWRRAMAARKRIDDLIDAQIADARTAP 198

CYP139A1_2592558321_Mycob 129 AESLFGQRLAVHSDFLGEQLQPLLDLTRRPPQVMRLQQRVNSPGWRRAMAARKRIDDLIDAQIADARTAP 198

CYP139A1_648456112_Mycoba 129 AESLFGQRLAVHSDFLGEQLQPLLDLTRRPPQVMRLQQRVNSPGWRRAMAARKRIDDLIDAQIADARTAP 198

CYP139A1_2575561335_Mycob 129 AESLFGQRLAVHSDFLGEQLQPLLDLTRRPPQVMRLQQRVNSPGWRRAMAARKRIDDLIDAQIADARTAP 198

CYP139A1_2575869049_Mycob 129 AESLFGQRLAVHSDFLGEQLQPLLDLTRRPPQVMRLQQRVNSPGWRRAMAARKRIDDLIDAQIADARTAP 198

CYP139A1_2576250927_Mycob 129 AESLFGQRLAVHSDFLGEQLQPLLDLTRRPPQVMRLQQRVNSPGWRRAMAARKRIDDLIDAQIADARTAP 198

CYP139A1_2577075963_Mycob 129 AESLFGQRLAVHSDFLGEQLQPLLDLTRRPPQVMRLQQRVNSPGWRRAMAARKRIDDLIDAQIADARTAP 198

CYP139A1_2577655387_Mycob 129 AESLFGQRLAVHSDFLGEQLQPLLDLTRRPPQVMRLQQRVNSPGWRRAMAARKRIDDLIDAQIADARTAP 198

CYP139A1_2579808474_Mycob 129 AESLFGQRLAVHSDFLGEQLQPLLDLTRRPPQVMRLQQRVNSPGWRRAMAARKRIDDLIDAQIADARTAP 198

CYP139A1_2580939152_Mycob 129 AESLFGQRLAVHSDFLGEQLQPLLDLTRRPPQVMRLQQRVNSPGWRRAMAARKRIDDLIDAQIADARTAP 198

CYP139A1_2581510874_Mycob 129 AESLFGQRLAVHSDFLGEQLQPLLDLTRRPPQVMRLQQRVNSPGWRRAMAARKRIDDLIDAQIADARTAP 198

CYP139A1_2590048710_Mycob 129 AESLFGQRLAVHSDFLGEQLQPLLDLTRRPPQVMRLQQRVNSPGWRRAMAARKRIDDLIDAQIADARTAP 198

CYP139A1_2590237321_Mycob 129 AESLFGQRLAVHSDFLGEQLQPLLDLTRRPPQVMRLQQRVNSPGWRRAMAARKRIDDLIDAQIADARTAP 198

CYP139A1_2574784397_Mycob 129 AESLFGQRLAVHSDFLGEQLQPLLDLTRRPPQVMRLQQRVNSPGWRRAMAARKRIDDLIDAQIADARTAP 198

CYP139A1_2574860198_Mycob 129 AESLFGQRLAVHSDFLGEQLQPLLDLTRRPPQVMRLQQRVNSPGWRRAMAARKRIDDLIDAQIADARTAP 198

CYP139A1_2574872651_Mycob 129 AESLFGQRLAVHSDFLGEQLQPLLDLTRRPPQVMRLQQRVNSPGWRRAMAARKRIDDLIDAQIADARTAP 198

CYP139A1_2575185207_Mycob 129 AESLFGQRLAVHSDFLGEQLQPLLDLTRRPPQVMRLQQRVNSPGWRRAMAARKRIDDLIDAQIADARTAP 198

CYP139A1_2576316581_Mycob 129 AESLFGQRLAVHSDFLGEQLQPLLDLTRRPPQVMRLQQRVNSPGWRRAMAARKRIDDLIDAQIADARTAP 198

CYP139A1_2576932272_Mycob 129 AESLFGQRLAVHSDFLGEQLQPLLDLTRRPPQVMRLQQRVNSPGWRRAMAARKRIDDLIDAQIADARTAP 198

CYP139A1_2577632297_Mycob 129 AESLFGQRLAVHSDFLGEQLQPLLDLTRRPPQVMRLQQRVNSPGWRRAMAARKRIDDLIDAQIADARTAP 198

CYP139A1_2577641910_Mycob 129 AESLFGQRLAVHSDFLGEQLQPLLDLTRRPPQVMRLQQRVNSPGWRRAMAARKRIDDLIDAQIADARTAP 198

CYP139A1_2584609065_Mycob 129 AESLFGQRLAVHSDFLGEQLQPLLDLTRRPPQVMRLQQRVNSPGWRRAMAARKRIDDLIDAQIADARTAP 198

CYP139A1_2584674477_Mycob 129 AESLFGQRLAVHSDFLGEQLQPLLDLTRRPPQVMRLQQRVNSPGWRRAMAARKRIDDLIDAQIADARTAP 198

CYP139A1_2584806904_Mycob 129 AESLFGQRLAVHSDFLGEQLQPLLDLTRRPPQVMRLQQRVNSPGWRRAMAARKRIDDLIDAQIADARTAP 198

CYP139A1_2584849650_Mycob 129 AESLFGQRLAVHSDFLGEQLQPLLDLTRRPPQVMRLQQRVNSPGWRRAMAARKRIDDLIDAQIADARTAP 198

CYP139A1_2584854188_Mycob 129 AESLFGQRLAVHSDFLGEQLQPLLDLTRRPPQVMRLQQRVNSPGWRRAMAARKRIDDLIDAQIADARTAP 198

CYP139A1_2589100068_Mycob 129 AESLFGQRLAVHSDFLGEQLQPLLDLTRRPPQVMRLQQRVNSPGWRRAMAARKRIDDLIDAQIADARTAP 198

CYP139A1_2589666655_Mycob 129 AESLFGQRLAVHSDFLGEQLQPLLDLTRRPPQVMRLQQRVNSPGWRRAMAARKRIDDLIDAQIADARTAP 198

CYP139A1_2589703490_Mycob 129 AESLFGQRLAVHSDFLGEQLQPLLDLTRRPPQVMRLQQRVNSPGWRRAMAARKRIDDLIDAQIADARTAP 198

CYP139A1_2589724111_Mycob 129 AESLFGQRLAVHSDFLGEQLQPLLDLTRRPPQVMRLQQRVNSPGWRRAMAARKRIDDLIDAQIADARTAP 198

CYP139A1_2590117900_Mycob 129 AESLFGQRLAVHSDFLGEQLQPLLDLTRRPPQVMRLQQRVNSPGWRRAMAARKRIDDLIDAQIADARTAP 198

CYP139A1_2590134192_Mycob 129 AESLFGQRLAVHSDFLGEQLQPLLDLTRRPPQVMRLQQRVNSPGWRRAMAARKRIDDLIDAQIADARTAP 198

CYP139A1_2590166763_Mycob 129 AESLFGQRLAVHSDFLGEQLQPLLDLTRRPPQVMRLQQRVNSPGWRRAMAARKRIDDLIDAQIADARTAP 198

CYP139A1_2590356674_Mycob 129 AESLFGQRLAVHSDFLGEQLQPLLDLTRRPPQVMRLQQRVNSPGWRRAMAARKRIDDLIDAQIADARTAP 198

CYP139A1_2549410800_Mycob 114 AESLFGQRLAVHSDFLGEQLQPLLDLTRRPPQVMRLQQRVNSPGWRRAMAARKRIDDLIDAQIADARTAP 183

CYP139A1_2575655283_Mycob 129 AESLFGQRLAVHSDFLGEQLQPLLDLTRRPPQVMRLQQRVNSPGWRRAMAARKRIDDLIDAQIADARTAP 198

CYP139A1_2576373809_Mycob 129 AESLFGQRLAVHSDFLGEQLQPLLDLTRRPPQVMRLQQRVNSPGWRRAMAARKRIDDLIDAQIADARTAP 198

CYP139A1_2576609942_Mycob 129 AESLFGQRLAVHSDFLGEQLQPLLDLTRRPPQVMRLQQRVNSPGWRRAMAARKRIDDLIDAQIADARTAP 198

CYP139A1_2576684060_Mycob 129 AESLFGQRLAVHSDFLGEQLQPLLDLTRRPPQVMRLQQRVNSPGWRRAMAARKRIDDLIDAQIADARTAP 198

CYP139A1_2577110129_Mycob 129 AESLFGQRLAVHSDFLGEQLQPLLDLTRRPPQVMRLQQRVNSPGWRRAMAARKRIDDLIDAQIADARTAP 198

CYP139A1_2577733463_Mycob 129 AESLFGQRLAVHSDFLGEQLQPLLDLTRRPPQVMRLQQRVNSPGWRRAMAARKRIDDLIDAQIADARTAP 198

CYP139A1_2578170996_Mycob 129 AESLFGQRLAVHSDFLGEQLQPLLDLTRRPPQVMRLQQRVNSPGWRRAMAARKRIDDLIDAQIADARTAP 198

CYP139A1_2583731958_Mycob 129 AESLFGQRLAVHSDFLGEQLQPLLDLTRRPPQVMRLQQRVNSPGWRRAMAARKRIDDLIDAQIADARTAP 198

CYP139A1_2590523531_Mycob 129 AESLFGQRLAVHSDFLGEQLQPLLDLTRRPPQVMRLQQRVNSPGWRRAMAARKRIDDLIDAQIADARTAP 198

CYP139A1_2590526411_Mycob 129 AESLFGQRLAVHSDFLGEQLQPLLDLTRRPPQVMRLQQRVNSPGWRRAMAARKRIDDLIDAQIADARTAP 198

CYP139A1_2590552289_Mycob 129 AESLFGQRLAVHSDFLGEQLQPLLDLTRRPPQVMRLQQRVNSPGWRRAMAARKRIDDLIDAQIADARTAP 198

CYP139A1_2575205306_Mycob 129 AESLFGQRLAVHSDFLGEQLQPLLDLTRRPPQVMRLQQRVNSPGWRRAMAARKRIDDLIDAQIADARTAP 198

CYP139A1_2575230562_Mycob 129 AESLFGQRLAVHSDFLGEQLQPLLDLTRRPPQVMRLQQRVNSPGWRRAMAARKRIDDLIDAQIADARTAP 198

CYP139A1_2575304538_Mycob 129 AESLFGQRLAVHSDFLGEQLQPLLDLTRRPPQVMRLQQRVNSPGWRRAMAARKRIDDLIDAQIADARTAP 198

CYP139A1_2575476916_Mycob 129 AESLFGQRLAVHSDFLGEQLQPLLDLTRRPPQVMRLQQRVNSPGWRRAMAARKRIDDLIDAQIADARTAP 198

CYP139A1_2575790354_Mycob 129 AESLFGQRLAVHSDFLGEQLQPLLDLTRRPPQVMRLQQRVNSPGWRRAMAARKRIDDLIDAQIADARTAP 198

CYP139A1_2576077839_Mycob 129 AESLFGQRLAVHSDFLGEQLQPLLDLTRRPPQVMRLQQRVNSPGWRRAMAARKRIDDLIDAQIADARTAP 198

CYP139A1_2576163842_Mycob 129 AESLFGQRLAVHSDFLGEQLQPLLDLTRRPPQVMRLQQRVNSPGWRRAMAARKRIDDLIDAQIADARTAP 198

CYP139A1_2577313382_Mycob 129 AESLFGQRLAVHSDFLGEQLQPLLDLTRRPPQVMRLQQRVNSPGWRRAMAARKRIDDLIDAQIADARTAP 198

CYP139A1_2577519304_Mycob 129 AESLFGQRLAVHSDFLGEQLQPLLDLTRRPPQVMRLQQRVNSPGWRRAMAARKRIDDLIDAQIADARTAP 198

CYP139A1_2577816400_Mycob 129 AESLFGQRLAVHSDFLGEQLQPLLDLTRRPPQVMRLQQRVNSPGWRRAMAARKRIDDLIDAQIADARTAP 198

CYP139A1_2584670398_Mycob 129 AESLFGQRLAVHSDFLGEQLQPLLDLTRRPPQVMRLQQRVNSPGWRRAMAARKRIDDLIDAQIADARTAP 198

CYP139A1_2584841030_Mycob 129 AESLFGQRLAVHSDFLGEQLQPLLDLTRRPPQVMRLQQRVNSPGWRRAMAARKRIDDLIDAQIADARTAP 198

CYP139A1_2584865918_Mycob 129 AESLFGQRLAVHSDFLGEQLQPLLDLTRRPPQVMRLQQRVNSPGWRRAMAARKRIDDLIDAQIADARTAP 198

CYP139A1_2589588224_Mycob 129 AESLFGQRLAVHSDFLGEQLQPLLDLTRRPPQVMRLQQRVNSPGWRRAMAARKRIDDLIDAQIADARTAP 198

CYP139A1_2589624713_Mycob 129 AESLFGQRLAVHSDFLGEQLQPLLDLTRRPPQVMRLQQRVNSPGWRRAMAARKRIDDLIDAQIADARTAP 198

CYP139A1_2589736180_Mycob 129 AESLFGQRLAVHSDFLGEQLQPLLDLTRRPPQVMRLQQRVNSPGWRRAMAARKRIDDLIDAQIADARTAP 198

CYP139A1_2590032388_Mycob 129 AESLFGQRLAVHSDFLGEQLQPLLDLTRRPPQVMRLQQRVNSPGWRRAMAARKRIDDLIDAQIADARTAP 198

CYP139A1_2590501611_Mycob 129 AESLFGQRLAVHSDFLGEQLQPLLDLTRRPPQVMRLQQRVNSPGWRRAMAARKRIDDLIDAQIADARTAP 198

CYP139A1_2592312197_Mycob 129 AESLFGQRLAVHSDFLGEQLQPLLDLTRRPPQVMRLQQRVNSPGWRRAMAARKRIDDLIDAQIADARTAP 198

CYP139A1_2592435284_Mycob 129 AESLFGQRLAVHSDFLGEQLQPLLDLTRRPPQVMRLQQRVNSPGWRRAMAARKRIDDLIDAQIADARTAP 198

CYP139A1_2511811274_Mycob 129 AESLFGQRLAVHSDFLGEQLQPLLDLTRRPPQVMRLQQRVNSPGWRRAMAARKRIDDLIDAQIADARTAP 198

CYP139A1_2546202077_Mycob 129 AESLFGQRLAVHSDFLGEQLQPLLDLTRRPPQVMRLQQRVNSPGWRRAMAARKRIDDLIDAQIADARTAP 198

CYP139A1_2574693694_Mycob 129 AESLFGQRLAVHSDFLGEQLQPLLDLTRRPPQVMRLQQRVNSPGWRRAMAARKRIDDLIDAQIADARTAP 198

CYP139A1_2574968392_Mycob 129 AESLFGQRLAVHSDFLGEQLQPLLDLTRRPPQVMRLQQRVNSPGWRRAMAARKRIDDLIDAQIADARTAP 198

CYP139A1_2577060340_Mycob 129 AESLFGQRLAVHSDFLGEQLQPLLDLTRRPPQVMRLQQRVNSPGWRRAMAARKRIDDLIDAQIADARTAP 198

CYP139A1_2577454945_Mycob 129 AESLFGQRLAVHSDFLGEQLQPLLDLTRRPPQVMRLQQRVNSPGWRRAMAARKRIDDLIDAQIADARTAP 198

CYP139A1_2580123929_Mycob 129 AESLFGQRLAVHSDFLGEQLQPLLDLTRRPPQVMRLQQRVNSPGWRRAMAARKRIDDLIDAQIADARTAP 198

CYP139A1_2581930746_Mycob 129 AESLFGQRLAVHSDFLGEQLQPLLDLTRRPPQVMRLQQRVNSPGWRRAMAARKRIDDLIDAQIADARTAP 198

CYP139A1_2584196432_Mycob 129 AESLFGQRLAVHSDFLGEQLQPLLDLTRRPPQVMRLQQRVNSPGWRRAMAARKRIDDLIDAQIADARTAP 198

CYP139A1_2584726832_Mycob 129 AESLFGQRLAVHSDFLGEQLQPLLDLTRRPPQVMRLQQRVNSPGWRRAMAARKRIDDLIDAQIADARTAP 198

CYP139A1_2584911080_Mycob 129 AESLFGQRLAVHSDFLGEQLQPLLDLTRRPPQVMRLQQRVNSPGWRRAMAARKRIDDLIDAQIADARTAP 198

CYP139A1_2584962435_Mycob 129 AESLFGQRLAVHSDFLGEQLQPLLDLTRRPPQVMRLQQRVNSPGWRRAMAARKRIDDLIDAQIADARTAP 198

CYP139A1_2584995097_Mycob 129 AESLFGQRLAVHSDFLGEQLQPLLDLTRRPPQVMRLQQRVNSPGWRRAMAARKRIDDLIDAQIADARTAP 198

CYP139A1_640602381_Mycoba 129 AESLFGQRLAVHSDFLGEQLQPLLDLTRRPPQVMRLQQRVNSPGWRRAMAARKRIDDLIDAQIADARTAP 198

CYP139A1_643019022_Mycoba 129 AESLFGQRLAVHSDFLGEQLQPLLDLTRRPPQVMRLQQRVNSPGWRRAMAARKRIDDLIDAQIADARTAP 198

CYP139A1_2573562450_Mycob 129 AESLFGQRLAVHSDFLGEQLQPLLDLTRRPPQVMRLQQRVNSPGWRRAMAARKRIDDLIDAQIADARTAP 198

CYP139A1_2575255926_Mycob 129 AESLFGQRLAVHSDFLGEQLQPLLDLTRRPPQVMRLQQRVNSPGWRRAMAARKRIDDLIDAQIADARTAP 198

CYP139A1_2577905307_Mycob 129 AESLFGQRLAVHSDFLGEQLQPLLDLTRRPPQVMRLQQRVNSPGWRRAMAARKRIDDLIDAQIADARTAP 198

CYP139A1_2579818167_Mycob 129 AESLFGQRLAVHSDFLGEQLQPLLDLTRRPPQVMRLQQRVNSPGWRRAMAARKRIDDLIDAQIADARTAP 198

CYP139A1_2582001898_Mycob 129 AESLFGQRLAVHSDFLGEQLQPLLDLTRRPPQVMRLQQRVNSPGWRRAMAARKRIDDLIDAQIADARTAP 198

CYP139A1_2588538927_Mycob 129 AESLFGQRLAVHSDFLGEQLQPLLDLTRRPPQVMRLQQRVNSPGWRRAMAARKRIDDLIDAQIADARTAP 198

CYP139A1_2590073317_Mycob 129 AESLFGQRLAVHSDFLGEQLQPLLDLTRRPPQVMRLQQRVNSPGWRRAMAARKRIDDLIDAQIADARTAP 198

CYP139A1_2590075971_Mycob 129 AESLFGQRLAVHSDFLGEQLQPLLDLTRRPPQVMRLQQRVNSPGWRRAMAARKRIDDLIDAQIADARTAP 198

CYP139A1_2590211264_Mycob 129 AESLFGQRLAVHSDFLGEQLQPLLDLTRRPPQVMRLQQRVNSPGWRRAMAARKRIDDLIDAQIADARTAP 198

CYP139A1_2590249475_Mycob 129 AESLFGQRLAVHSDFLGEQLQPLLDLTRRPPQVMRLQQRVNSPGWRRAMAARKRIDDLIDAQIADARTAP 198

CYP139A1_2590279314_Mycob 129 AESLFGQRLAVHSDFLGEQLQPLLDLTRRPPQVMRLQQRVNSPGWRRAMAARKRIDDLIDAQIADARTAP 198

CYP139A1_2590559932_Mycob 129 AESLFGQRLAVHSDFLGEQLQPLLDLTRRPPQVMRLQQRVNSPGWRRAMAARKRIDDLIDAQIADARTAP 198

CYP139A1_640606444_Mycoba 129 AESLFGQRLAVHSDFLGEQLQPLLDLTRRPPQVMRLQQRVNSPGWRRAMAARKRIDDLIDAQIADARTAP 198

CYP139A1_651025167_Mycoba 129 AESLFGQRLAVHSDFLGEQLQPLLDLTRRPPQVMRLQQRVNSPGWRRAMAARKRIDDLIDAQIADARTAP 198

CYP139A1_2574876573_Mycob 129 AESLFGQRLAVHSDFLGEQLQPLLDLTRRPPQVMRLQQRVNSPGWRRAMAARKRIDDLIDAQIADARTAP 198

CYP139A1_2575028161_Mycob 129 AESLFGQRLAVHSDFLGEQLQPLLDLTRRPPQVMRLQQRVNSPGWRRAMAARKRIDDLIDAQIADARTAP 198

CYP139A1_2575888575_Mycob 129 AESLFGQRLAVHSDFLGEQLQPLLDLTRRPPQVMRLQQRVNSPGWRRAMAARKRIDDLIDAQIADARTAP 198

CYP139A1_2576708817_Mycob 129 AESLFGQRLAVHSDFLGEQLQPLLDLTRRPPQVMRLQQRVNSPGWRRAMAARKRIDDLIDAQIADARTAP 198

CYP139A1_2577386815_Mycob 129 AESLFGQRLAVHSDFLGEQLQPLLDLTRRPPQVMRLQQRVNSPGWRRAMAARKRIDDLIDAQIADARTAP 198

CYP139A1_2578040087_Mycob 129 AESLFGQRLAVHSDFLGEQLQPLLDLTRRPPQVMRLQQRVNSPGWRRAMAARKRIDDLIDAQIADARTAP 198

CYP139A1_2584645880_Mycob 129 AESLFGQRLAVHSDFLGEQLQPLLDLTRRPPQVMRLQQRVNSPGWRRAMAARKRIDDLIDAQIADARTAP 198

CYP139A1_2584659187_Mycob 129 AESLFGQRLAVHSDFLGEQLQPLLDLTRRPPQVMRLQQRVNSPGWRRAMAARKRIDDLIDAQIADARTAP 198

CYP139A1_2584718757_Mycob 129 AESLFGQRLAVHSDFLGEQLQPLLDLTRRPPQVMRLQQRVNSPGWRRAMAARKRIDDLIDAQIADARTAP 198

CYP139A1_2589064595_Mycob 129 AESLFGQRLAVHSDFLGEQLQPLLDLTRRPPQVMRLQQRVNSPGWRRAMAARKRIDDLIDAQIADARTAP 198

CYP139A1_2589113575_Mycob 129 AESLFGQRLAVHSDFLGEQLQPLLDLTRRPPQVMRLQQRVNSPGWRRAMAARKRIDDLIDAQIADARTAP 198

CYP139A1_2589559459_Mycob 129 AESLFGQRLAVHSDFLGEQLQPLLDLTRRPPQVMRLQQRVNSPGWRRAMAARKRIDDLIDAQIADARTAP 198

CYP139A1_2589575466_Mycob 129 AESLFGQRLAVHSDFLGEQLQPLLDLTRRPPQVMRLQQRVNSPGWRRAMAARKRIDDLIDAQIADARTAP 198

CYP139A1_2589595990_Mycob 129 AESLFGQRLAVHSDFLGEQLQPLLDLTRRPPQVMRLQQRVNSPGWRRAMAARKRIDDLIDAQIADARTAP 198

CYP139A1_2592259141_Mycob 129 AESLFGQRLAVHSDFLGEQLQPLLDLTRRPPQVMRLQQRVNSPGWRRAMAARKRIDDLIDAQIADARTAP 198

CYP139A1_2592280664_Mycob 129 AESLFGQRLAVHSDFLGEQLQPLLDLTRRPPQVMRLQQRVNSPGWRRAMAARKRIDDLIDAQIADARTAP 198

CYP139A1_2592389699_Mycob 129 AESLFGQRLAVHSDFLGEQLQPLLDLTRRPPQVMRLQQRVNSPGWRRAMAARKRIDDLIDAQIADARTAP 198

CYP139A1_2592438630_Mycob 129 AESLFGQRLAVHSDFLGEQLQPLLDLTRRPPQVMRLQQRVNSPGWRRAMAARKRIDDLIDAQIADARTAP 198

CYP139A1_2592553482_Mycob 129 AESLFGQRLAVHSDFLGEQLQPLLDLTRRPPQVMRLQQRVNSPGWRRAMAARKRIDDLIDAQIADARTAP 198

CYP139A1_2592570555_Mycob 129 AESLFGQRLAVHSDFLGEQLQPLLDLTRRPPQVMRLQQRVNSPGWRRAMAARKRIDDLIDAQIADARTAP 198

CYP139A1_2575252379_Mycob 129 AESLFGQRLAVHSDFLGEQLQPLLDLTRRPPQVMRLQQRVNSPGWRRAMAARKRIDDLIDAQIADARTAP 198

CYP139A1_2575520720_Mycob 129 AESLFGQRLAVHSDFLGEQLQPLLDLTRRPPQVMRLQQRVNSPGWRRAMAARKRIDDLIDAQIADARTAP 198

CYP139A1_2575705222_Mycob 129 AESLFGQRLAVHSDFLGEQLQPLLDLTRRPPQVMRLQQRVNSPGWRRAMAARKRIDDLIDAQIADARTAP 198

CYP139A1_2575964273_Mycob 129 AESLFGQRLAVHSDFLGEQLQPLLDLTRRPPQVMRLQQRVNSPGWRRAMAARKRIDDLIDAQIADARTAP 198

CYP139A1_2577195360_Mycob 129 AESLFGQRLAVHSDFLGEQLQPLLDLTRRPPQVMRLQQRVNSPGWRRAMAARKRIDDLIDAQIADARTAP 198

CYP139A1_2581587072_Mycob 129 AESLFGQRLAVHSDFLGEQLQPLLDLTRRPPQVMRLQQRVNSPGWRRAMAARKRIDDLIDAQIADARTAP 198

CYP139A1_2584633235_Mycob 129 AESLFGQRLAVHSDFLGEQLQPLLDLTRRPPQVMRLQQRVNSPGWRRAMAARKRIDDLIDAQIADARTAP 198

CYP139A1_2584732271_Mycob 129 AESLFGQRLAVHSDFLGEQLQPLLDLTRRPPQVMRLQQRVNSPGWRRAMAARKRIDDLIDAQIADARTAP 198

CYP139A1_2584791771_Mycob 129 AESLFGQRLAVHSDFLGEQLQPLLDLTRRPPQVMRLQQRVNSPGWRRAMAARKRIDDLIDAQIADARTAP 198

CYP139A1_2589148607_Mycob 129 AESLFGQRLAVHSDFLGEQLQPLLDLTRRPPQVMRLQQRVNSPGWRRAMAARKRIDDLIDAQIADARTAP 198

CYP139A1_2590150448_Mycob 129 AESLFGQRLAVHSDFLGEQLQPLLDLTRRPPQVMRLQQRVNSPGWRRAMAARKRIDDLIDAQIADARTAP 198

CYP139A1_2590231653_Mycob 129 AESLFGQRLAVHSDFLGEQLQPLLDLTRRPPQVMRLQQRVNSPGWRRAMAARKRIDDLIDAQIADARTAP 198

CYP139A1_2590283895_Mycob 129 AESLFGQRLAVHSDFLGEQLQPLLDLTRRPPQVMRLQQRVNSPGWRRAMAARKRIDDLIDAQIADARTAP 198

CYP139A1_645120373_Mycoba 129 AESLFGQRLAVHSDFLGEQLQPLLDLTRRPPQVMRLQQRVNSPGWRRAMAARKRIDDLIDAQIADARTAP 198

CYP139A1_2574734007_Mycob 129 AESLFGQRLAVHSDFLGEQLQPLLDLTRRPPQVMRLQQRVNSPGWRRAMAARKRIDDLIDAQIADARTAP 198

CYP139A1_2575099885_Mycob 129 AESLFGQRLAVHSDFLGEQLQPLLDLTRRPPQVMRLQQRVNSPGWRRAMAARKRIDDLIDAQIADARTAP 198

CYP139A1_2575132657_Mycob 129 AESLFGQRLAVHSDFLGEQLQPLLDLTRRPPQVMRLQQRVNSPGWRRAMAARKRIDDLIDAQIADARTAP 198

CYP139A1_2575465536_Mycob 129 AESLFGQRLAVHSDFLGEQLQPLLDLTRRPPQVMRLQQRVNSPGWRRAMAARKRIDDLIDAQIADARTAP 198

CYP139A1_2576665707_Mycob 129 AESLFGQRLAVHSDFLGEQLQPLLDLTRRPPQVMRLQQRVNSPGWRRAMAARKRIDDLIDAQIADARTAP 198

CYP139A1_2576698280_Mycob 129 AESLFGQRLAVHSDFLGEQLQPLLDLTRRPPQVMRLQQRVNSPGWRRAMAARKRIDDLIDAQIADARTAP 198

CYP139A1_2576759003_Mycob 129 AESLFGQRLAVHSDFLGEQLQPLLDLTRRPPQVMRLQQRVNSPGWRRAMAARKRIDDLIDAQIADARTAP 198

CYP139A1_2577673179_Mycob 129 AESLFGQRLAVHSDFLGEQLQPLLDLTRRPPQVMRLQQRVNSPGWRRAMAARKRIDDLIDAQIADARTAP 198

CYP139A1_2577936335_Mycob 129 AESLFGQRLAVHSDFLGEQLQPLLDLTRRPPQVMRLQQRVNSPGWRRAMAARKRIDDLIDAQIADARTAP 198

CYP139A1_2578008273_Mycob 129 AESLFGQRLAVHSDFLGEQLQPLLDLTRRPPQVMRLQQRVNSPGWRRAMAARKRIDDLIDAQIADARTAP 198

CYP139A1_2578073775_Mycob 129 AESLFGQRLAVHSDFLGEQLQPLLDLTRRPPQVMRLQQRVNSPGWRRAMAARKRIDDLIDAQIADARTAP 198

CYP139A1_2584960744_Mycob 129 AESLFGQRLAVHSDFLGEQLQPLLDLTRRPPQVMRLQQRVNSPGWRRAMAARKRIDDLIDAQIADARTAP 198

CYP139A1_2588992251_Mycob 129 AESLFGQRLAVHSDFLGEQLQPLLDLTRRPPQVMRLQQRVNSPGWRRAMAARKRIDDLIDAQIADARTAP 198

CYP139A1_2589077872_Mycob 129 AESLFGQRLAVHSDFLGEQLQPLLDLTRRPPQVMRLQQRVNSPGWRRAMAARKRIDDLIDAQIADARTAP 198

CYP139A1_2589518744_Mycob 129 AESLFGQRLAVHSDFLGEQLQPLLDLTRRPPQVMRLQQRVNSPGWRRAMAARKRIDDLIDAQIADARTAP 198

CYP139A1_2589583955_Mycob 129 AESLFGQRLAVHSDFLGEQLQPLLDLTRRPPQVMRLQQRVNSPGWRRAMAARKRIDDLIDAQIADARTAP 198

CYP139A1_2589646609_Mycob 129 AESLFGQRLAVHSDFLGEQLQPLLDLTRRPPQVMRLQQRVNSPGWRRAMAARKRIDDLIDAQIADARTAP 198

CYP139A1_2592316279_Mycob 129 AESLFGQRLAVHSDFLGEQLQPLLDLTRRPPQVMRLQQRVNSPGWRRAMAARKRIDDLIDAQIADARTAP 198

CYP139A1_2592546111_Mycob 129 AESLFGQRLAVHSDFLGEQLQPLLDLTRRPPQVMRLQQRVNSPGWRRAMAARKRIDDLIDAQIADARTAP 198

CYP139A1_2511553315_Mycob 129 AESLFGQRLAVHSDFLGEQLQPLLDLTRRPPQVMRLQQRVNSPGWRRAMAARKRIDDLIDAQIADARTAP 198

CYP139A1_2574949227_Mycob 129 AESLFGQRLAVHSDFLGEQLQPLLDLTRRPPQVMRLQQRVNSPGWRRAMAARKRIDDLIDAQIADARTAP 198

CYP139A1_2575533703_Mycob 129 AESLFGQRLAVHSDFLGEQLQPLLDLTRRPPQVMRLQQRVNSPGWRRAMAARKRIDDLIDAQIADARTAP 198

CYP139A1_2576015739_Mycob 129 AESLFGQRLAVHSDFLGEQLQPLLDLTRRPPQVMRLQQRVNSPGWRRAMAARKRIDDLIDAQIADARTAP 198

CYP139A1_2576644886_Mycob 129 AESLFGQRLAVHSDFLGEQLQPLLDLTRRPPQVMRLQQRVNSPGWRRAMAARKRIDDLIDAQIADARTAP 198

CYP139A1_2577497204_Mycob 129 AESLFGQRLAVHSDFLGEQLQPLLDLTRRPPQVMRLQQRVNSPGWRRAMAARKRIDDLIDAQIADARTAP 198

CYP139A1_2580744928_Mycob 129 AESLFGQRLAVHSDFLGEQLQPLLDLTRRPPQVMRLQQRVNSPGWRRAMAARKRIDDLIDAQIADARTAP 198

CYP139A1_2583727889_Mycob 129 AESLFGQRLAVHSDFLGEQLQPLLDLTRRPPQVMRLQQRVNSPGWRRAMAARKRIDDLIDAQIADARTAP 198

CYP139A1_2590064965_Mycob 129 AESLFGQRLAVHSDFLGEQLQPLLDLTRRPPQVMRLQQRVNSPGWRRAMAARKRIDDLIDAQIADARTAP 198

CYP139A1_2590121989_Mycob 129 AESLFGQRLAVHSDFLGEQLQPLLDLTRRPPQVMRLQQRVNSPGWRRAMAARKRIDDLIDAQIADARTAP 198

CYP139A1_2590294228_Mycob 129 AESLFGQRLAVHSDFLGEQLQPLLDLTRRPPQVMRLQQRVNSPGWRRAMAARKRIDDLIDAQIADARTAP 198

CYP139A1_2590499050_Mycob 129 AESLFGQRLAVHSDFLGEQLQPLLDLTRRPPQVMRLQQRVNSPGWRRAMAARKRIDDLIDAQIADARTAP 198

CYP139A1_2590519464_Mycob 129 AESLFGQRLAVHSDFLGEQLQPLLDLTRRPPQVMRLQQRVNSPGWRRAMAARKRIDDLIDAQIADARTAP 198

CYP139A1_2590548005_Mycob 129 AESLFGQRLAVHSDFLGEQLQPLLDLTRRPPQVMRLQQRVNSPGWRRAMAARKRIDDLIDAQIADARTAP 198

CYP139A1_637139034_Mycoba 129 AESLFGQRLAVHSDFLGEQLQPLLDLTRRPPQVMRLQQRVNSPGWRRAMAARKRIDDLIDAQIADARTAP 198

CYP139A1_639830617_Mycoba 129 AESLFGQRLAVHSDFLGEQLQPLLDLTRRPPQVMRLQQRVNSPGWRRAMAARKRIDDLIDAQIADARTAP 198

CYP139A1_2574640348_Mycob 129 AESLFGQRLAVHSDFLGEQLQPLLDLTRRPPQVMRLQQRVNSPGWRRAMAARKRIDDLIDAQIADARTAP 198

CYP139A1_2574794413_Mycob 129 AESLFGQRLAVHSDFLGEQLQPLLDLTRRPPQVMRLQQRVNSPGWRRAMAARKRIDDLIDAQIADARTAP 198

CYP139A1_2574987296_Mycob 129 AESLFGQRLAVHSDFLGEQLQPLLDLTRRPPQVMRLQQRVNSPGWRRAMAARKRIDDLIDAQIADARTAP 198

CYP139A1_2575084753_Mycob 129 AESLFGQRLAVHSDFLGEQLQPLLDLTRRPPQVMRLQQRVNSPGWRRAMAARKRIDDLIDAQIADARTAP 198

CYP139A1_2575627312_Mycob 129 AESLFGQRLAVHSDFLGEQLQPLLDLTRRPPQVMRLQQRVNSPGWRRAMAARKRIDDLIDAQIADARTAP 198

CYP139A1_2576321347_Mycob 129 AESLFGQRLAVHSDFLGEQLQPLLDLTRRPPQVMRLQQRVNSPGWRRAMAARKRIDDLIDAQIADARTAP 198

CYP139A1_2576378950_Mycob 129 AESLFGQRLAVHSDFLGEQLQPLLDLTRRPPQVMRLQQRVNSPGWRRAMAARKRIDDLIDAQIADARTAP 198

CYP139A1_2576735552_Mycob 129 AESLFGQRLAVHSDFLGEQLQPLLDLTRRPPQVMRLQQRVNSPGWRRAMAARKRIDDLIDAQIADARTAP 198

CYP139A1_2576940434_Mycob 129 AESLFGQRLAVHSDFLGEQLQPLLDLTRRPPQVMRLQQRVNSPGWRRAMAARKRIDDLIDAQIADARTAP 198

CYP139A1_2577812038_Mycob 129 AESLFGQRLAVHSDFLGEQLQPLLDLTRRPPQVMRLQQRVNSPGWRRAMAARKRIDDLIDAQIADARTAP 198

CYP139A1_2577861791_Mycob 129 AESLFGQRLAVHSDFLGEQLQPLLDLTRRPPQVMRLQQRVNSPGWRRAMAARKRIDDLIDAQIADARTAP 198

CYP139A1_2578053379_Mycob 129 AESLFGQRLAVHSDFLGEQLQPLLDLTRRPPQVMRLQQRVNSPGWRRAMAARKRIDDLIDAQIADARTAP 198

CYP139A1_2584860925_Mycob 129 AESLFGQRLAVHSDFLGEQLQPLLDLTRRPPQVMRLQQRVNSPGWRRAMAARKRIDDLIDAQIADARTAP 198

CYP139A1_2584936325_Mycob 129 AESLFGQRLAVHSDFLGEQLQPLLDLTRRPPQVMRLQQRVNSPGWRRAMAARKRIDDLIDAQIADARTAP 198

CYP139A1_2584939524_Mycob 129 AESLFGQRLAVHSDFLGEQLQPLLDLTRRPPQVMRLQQRVNSPGWRRAMAARKRIDDLIDAQIADARTAP 198

CYP139A1_2589106145_Mycob 129 AESLFGQRLAVHSDFLGEQLQPLLDLTRRPPQVMRLQQRVNSPGWRRAMAARKRIDDLIDAQIADARTAP 198

CYP139A1_2589543202_Mycob 129 AESLFGQRLAVHSDFLGEQLQPLLDLTRRPPQVMRLQQRVNSPGWRRAMAARKRIDDLIDAQIADARTAP 198

CYP139A1_2589642534_Mycob 129 AESLFGQRLAVHSDFLGEQLQPLLDLTRRPPQVMRLQQRVNSPGWRRAMAARKRIDDLIDAQIADARTAP 198

CYP139A1_2589691243_Mycob 129 AESLFGQRLAVHSDFLGEQLQPLLDLTRRPPQVMRLQQRVNSPGWRRAMAARKRIDDLIDAQIADARTAP 198

CYP139A1_2589695547_Mycob 129 AESLFGQRLAVHSDFLGEQLQPLLDLTRRPPQVMRLQQRVNSPGWRRAMAARKRIDDLIDAQIADARTAP 198

CYP139A1_2592222422_Mycob 129 AESLFGQRLAVHSDFLGEQLQPLLDLTRRPPQVMRLQQRVNSPGWRRAMAARKRIDDLIDAQIADARTAP 198

CYP139A1_648446923_Mycoba 129 AESLFGQRLAVHSDFLGEQLQPLLDLTRRPPQVMRLQQRVNSPGWRRAMAARKRIDDLIDAQIADARTAP 198

CYP139A1_648469578_Mycoba 129 AESLFGQRLAVHSDFLGEQLQPLLDLTRRPPQVMRLQQRVNSPGWRRAMAARKRIDDLIDAQIADARTAP 198

CYP139A1_2546188127_Mycob 129 AESLFGQRLAVHSDFLGEQLQPLLDLTRRPPQVMRLQQRVNSPGWRRAMAARKRIDDLIDAQIADARTAP 198

CYP139A1_2574773054_Mycob 129 AESLFGQRLAVHSDFLGEQLQPLLDLTRRPPQVMRLQQRVNSPGWRRAMAARKRIDDLIDAQIADARTAP 198

CYP139A1_2574854728_Mycob 129 AESLFGQRLAVHSDFLGEQLQPLLDLTRRPPQVMRLQQRVNSPGWRRAMAARKRIDDLIDAQIADARTAP 198

CYP139A1_2575984510_Mycob 129 AESLFGQRLAVHSDFLGEQLQPLLDLTRRPPQVMRLQQRVNSPGWRRAMAARKRIDDLIDAQIADARTAP 198

CYP139A1_2576293562_Mycob 129 AESLFGQRLAVHSDFLGEQLQPLLDLTRRPPQVMRLQQRVNSPGWRRAMAARKRIDDLIDAQIADARTAP 198

CYP139A1_2576717872_Mycob 129 AESLFGQRLAVHSDFLGEQLQPLLDLTRRPPQVMRLQQRVNSPGWRRAMAARKRIDDLIDAQIADARTAP 198

CYP139A1_2578189802_Mycob 129 AESLFGQRLAVHSDFLGEQLQPLLDLTRRPPQVMRLQQRVNSPGWRRAMAARKRIDDLIDAQIADARTAP 198

CYP139A1_2584890677_Mycob 129 AESLFGQRLAVHSDFLGEQLQPLLDLTRRPPQVMRLQQRVNSPGWRRAMAARKRIDDLIDAQIADARTAP 198

CYP139A1_2590070071_Mycob 129 AESLFGQRLAVHSDFLGEQLQPLLDLTRRPPQVMRLQQRVNSPGWRRAMAARKRIDDLIDAQIADARTAP 198

CYP139A1_2590169218_Mycob 129 AESLFGQRLAVHSDFLGEQLQPLLDLTRRPPQVMRLQQRVNSPGWRRAMAARKRIDDLIDAQIADARTAP 198

CYP139A1_644880084_Mycoba 129 AESLFGQRLAVHSDFLGEQLQPLLDLTRRPPQVMRLQQRVNSPGWRRAMAARKRIDDLIDAQIADARTAP 198

CYP139A1_2574905673_Mycob 129 AESLFGQRLAVHSDFLGEQLQPLLDLTRRPPQVMRLQQRVNSPGWRRAMAARKRIDDLIDAQIADARTAP 198

CYP139A1_2575285636_Mycob 129 AESLFGQRLAVHSDFLGEQLQPLLDLTRRPPQVMRLQQRVNSPGWRRAMAARKRIDDLIDAQIADARTAP 198

CYP139A1_2575508785_Mycob 129 AESLFGQRLAVHSDFLGEQLQPLLDLTRRPPQVMRLQQRVNSPGWRRAMAARKRIDDLIDAQIADARTAP 198

CYP139A1_2576585830_Mycob 129 AESLFGQRLAVHSDFLGEQLQPLLDLTRRPPQVMRLQQRVNSPGWRRAMAARKRIDDLIDAQIADARTAP 198

CYP139A1_2584979253_Mycob 129 AESLFGQRLAVHSDFLGEQLQPLLDLTRRPPQVMRLQQRVNSPGWRRAMAARKRIDDLIDAQIADARTAP 198

CYP139A1_2588982991_Mycob 129 AESLFGQRLAVHSDFLGEQLQPLLDLTRRPPQVMRLQQRVNSPGWRRAMAARKRIDDLIDAQIADARTAP 198

CYP139A1_2589048312_Mycob 129 AESLFGQRLAVHSDFLGEQLQPLLDLTRRPPQVMRLQQRVNSPGWRRAMAARKRIDDLIDAQIADARTAP 198

CYP139A1_2589060525_Mycob 129 AESLFGQRLAVHSDFLGEQLQPLLDLTRRPPQVMRLQQRVNSPGWRRAMAARKRIDDLIDAQIADARTAP 198

CYP139A1_2589502409_Mycob 129 AESLFGQRLAVHSDFLGEQLQPLLDLTRRPPQVMRLQQRVNSPGWRRAMAARKRIDDLIDAQIADARTAP 198

CYP139A1_2589572815_Mycob 129 AESLFGQRLAVHSDFLGEQLQPLLDLTRRPPQVMRLQQRVNSPGWRRAMAARKRIDDLIDAQIADARTAP 198

CYP139A1_2590181544_Mycob 129 AESLFGQRLAVHSDFLGEQLQPLLDLTRRPPQVMRLQQRVNSPGWRRAMAARKRIDDLIDAQIADARTAP 198

CYP139A1_2592234638_Mycob 129 AESLFGQRLAVHSDFLGEQLQPLLDLTRRPPQVMRLQQRVNSPGWRRAMAARKRIDDLIDAQIADARTAP 198

CYP139A1_2592271382_Mycob 129 AESLFGQRLAVHSDFLGEQLQPLLDLTRRPPQVMRLQQRVNSPGWRRAMAARKRIDDLIDAQIADARTAP 198

CYP139A1_2592295853_Mycob 129 AESLFGQRLAVHSDFLGEQLQPLLDLTRRPPQVMRLQQRVNSPGWRRAMAARKRIDDLIDAQIADARTAP 198

CYP139A1_2592344179_Mycob 129 AESLFGQRLAVHSDFLGEQLQPLLDLTRRPPQVMRLQQRVNSPGWRRAMAARKRIDDLIDAQIADARTAP 198

CYP139A1_2592357110_Mycob 129 AESLFGQRLAVHSDFLGEQLQPLLDLTRRPPQVMRLQQRVNSPGWRRAMAARKRIDDLIDAQIADARTAP 198

CYP139A1_643035957_Mycoba 129 AESLFGQRLAVHSDFLGEQLQPLLDLTRRPPQVMRLQQRVNSPGWRRAMAARKRIDDLIDAQIADARTAP 198

CYP139A1_2575162612_Mycob 129 AESLFGQRLAVHSDFLGEQLQPLLDLTRRPPQVMRLQQRVNSPGWRRAMAARKRIDDLIDAQIADARTAP 198

CYP139A1_2575198589_Mycob 129 AESLFGQRLAVHSDFLGEQLQPLLDLTRRPPQVMRLQQRVNSPGWRRAMAARKRIDDLIDAQIADARTAP 198

CYP139A1_2575418829_Mycob 129 AESLFGQRLAVHSDFLGEQLQPLLDLTRRPPQVMRLQQRVNSPGWRRAMAARKRIDDLIDAQIADARTAP 198

CYP139A1_2576172216_Mycob 129 AESLFGQRLAVHSDFLGEQLQPLLDLTRRPPQVMRLQQRVNSPGWRRAMAARKRIDDLIDAQIADARTAP 198

CYP139A1_2576534922_Mycob 129 AESLFGQRLAVHSDFLGEQLQPLLDLTRRPPQVMRLQQRVNSPGWRRAMAARKRIDDLIDAQIADARTAP 198

CYP139A1_2577185436_Mycob 129 AESLFGQRLAVHSDFLGEQLQPLLDLTRRPPQVMRLQQRVNSPGWRRAMAARKRIDDLIDAQIADARTAP 198

CYP139A1_2577236838_Mycob 129 AESLFGQRLAVHSDFLGEQLQPLLDLTRRPPQVMRLQQRVNSPGWRRAMAARKRIDDLIDAQIADARTAP 198

CYP139A1_2577373328_Mycob 129 AESLFGQRLAVHSDFLGEQLQPLLDLTRRPPQVMRLQQRVNSPGWRRAMAARKRIDDLIDAQIADARTAP 198

CYP139A1_2578099005_Mycob 129 AESLFGQRLAVHSDFLGEQLQPLLDLTRRPPQVMRLQQRVNSPGWRRAMAARKRIDDLIDAQIADARTAP 198

CYP139A1_2580366102_Mycob 129 AESLFGQRLAVHSDFLGEQLQPLLDLTRRPPQVMRLQQRVNSPGWRRAMAARKRIDDLIDAQIADARTAP 198

CYP139A1_2580467795_Mycob 129 AESLFGQRLAVHSDFLGEQLQPLLDLTRRPPQVMRLQQRVNSPGWRRAMAARKRIDDLIDAQIADARTAP 198

CYP139A1_2584613792_Mycob 129 AESLFGQRLAVHSDFLGEQLQPLLDLTRRPPQVMRLQQRVNSPGWRRAMAARKRIDDLIDAQIADARTAP 198

CYP139A1_2584681396_Mycob 129 AESLFGQRLAVHSDFLGEQLQPLLDLTRRPPQVMRLQQRVNSPGWRRAMAARKRIDDLIDAQIADARTAP 198

CYP139A1_2584913651_Mycob 129 AESLFGQRLAVHSDFLGEQLQPLLDLTRRPPQVMRLQQRVNSPGWRRAMAARKRIDDLIDAQIADARTAP 198

CYP139A1_2584942354_Mycob 129 AESLFGQRLAVHSDFLGEQLQPLLDLTRRPPQVMRLQQRVNSPGWRRAMAARKRIDDLIDAQIADARTAP 198

CYP139A1_2590093450_Mycob 129 AESLFGQRLAVHSDFLGEQLQPLLDLTRRPPQVMRLQQRVNSPGWRRAMAARKRIDDLIDAQIADARTAP 198

CYP139A1_2590207004_Mycob 129 AESLFGQRLAVHSDFLGEQLQPLLDLTRRPPQVMRLQQRVNSPGWRRAMAARKRIDDLIDAQIADARTAP 198

CYP139A1_2590276448_Mycob 129 AESLFGQRLAVHSDFLGEQLQPLLDLTRRPPQVMRLQQRVNSPGWRRAMAARKRIDDLIDAQIADARTAP 198

CYP139A1_2575455196_Mycob 129 AESLFGQRLAVHSDFLGEQLQPLLDLTRRPPQVMRLQQRVNSPGWRRAMAARKRIDDLIDAQIADARTAP 198

CYP139A1_2575920420_Mycob 129 AESLFGQRLAVHSDFLGEQLQPLLDLTRRPPQVMRLQQRVNSPGWRRAMAARKRIDDLIDAQIADARTAP 198

CYP139A1_2575998368_Mycob 129 AESLFGQRLAVHSDFLGEQLQPLLDLTRRPPQVMRLQQRVNSPGWRRAMAARKRIDDLIDAQIADARTAP 198

CYP139A1_2576583109_Mycob 129 AESLFGQRLAVHSDFLGEQLQPLLDLTRRPPQVMRLQQRVNSPGWRRAMAARKRIDDLIDAQIADARTAP 198

CYP139A1_2576886343_Mycob 129 AESLFGQRLAVHSDFLGEQLQPLLDLTRRPPQVMRLQQRVNSPGWRRAMAARKRIDDLIDAQIADARTAP 198

CYP139A1_2576986616_Mycob 129 AESLFGQRLAVHSDFLGEQLQPLLDLTRRPPQVMRLQQRVNSPGWRRAMAARKRIDDLIDAQIADARTAP 198

CYP139A1_2577256179_Mycob 129 AESLFGQRLAVHSDFLGEQLQPLLDLTRRPPQVMRLQQRVNSPGWRRAMAARKRIDDLIDAQIADARTAP 198

CYP139A1_2577876445_Mycob 129 AESLFGQRLAVHSDFLGEQLQPLLDLTRRPPQVMRLQQRVNSPGWRRAMAARKRIDDLIDAQIADARTAP 198

CYP139A1_2579798813_Mycob 129 AESLFGQRLAVHSDFLGEQLQPLLDLTRRPPQVMRLQQRVNSPGWRRAMAARKRIDDLIDAQIADARTAP 198

CYP139A1_2584715344_Mycob 129 AESLFGQRLAVHSDFLGEQLQPLLDLTRRPPQVMRLQQRVNSPGWRRAMAARKRIDDLIDAQIADARTAP 198

CYP139A1_2588659602_Mycob 129 AESLFGQRLAVHSDFLGEQLQPLLDLTRRPPQVMRLQQRVNSPGWRRAMAARKRIDDLIDAQIADARTAP 198

CYP139A1_2589089021_Mycob 129 AESLFGQRLAVHSDFLGEQLQPLLDLTRRPPQVMRLQQRVNSPGWRRAMAARKRIDDLIDAQIADARTAP 198

CYP139A1_2589093094_Mycob 129 AESLFGQRLAVHSDFLGEQLQPLLDLTRRPPQVMRLQQRVNSPGWRRAMAARKRIDDLIDAQIADARTAP 198

CYP139A1_2589535085_Mycob 129 AESLFGQRLAVHSDFLGEQLQPLLDLTRRPPQVMRLQQRVNSPGWRRAMAARKRIDDLIDAQIADARTAP 198

CYP139A1_2589663797_Mycob 129 AESLFGQRLAVHSDFLGEQLQPLLDLTRRPPQVMRLQQRVNSPGWRRAMAARKRIDDLIDAQIADARTAP 198

CYP139A1_2589671952_Mycob 129 AESLFGQRLAVHSDFLGEQLQPLLDLTRRPPQVMRLQQRVNSPGWRRAMAARKRIDDLIDAQIADARTAP 198

CYP139A1_2589719807_Mycob 129 AESLFGQRLAVHSDFLGEQLQPLLDLTRRPPQVMRLQQRVNSPGWRRAMAARKRIDDLIDAQIADARTAP 198

CYP139A1_2592246876_Mycob 129 AESLFGQRLAVHSDFLGEQLQPLLDLTRRPPQVMRLQQRVNSPGWRRAMAARKRIDDLIDAQIADARTAP 198

CYP139A1_2592381635_Mycob 129 AESLFGQRLAVHSDFLGEQLQPLLDLTRRPPQVMRLQQRVNSPGWRRAMAARKRIDDLIDAQIADARTAP 198

CYP139A1_2592410032_Mycob 129 AESLFGQRLAVHSDFLGEQLQPLLDLTRRPPQVMRLQQRVNSPGWRRAMAARKRIDDLIDAQIADARTAP 198

CYP139A1_2592414205_Mycob 129 AESLFGQRLAVHSDFLGEQLQPLLDLTRRPPQVMRLQQRVNSPGWRRAMAARKRIDDLIDAQIADARTAP 198

CYP139A1_2592566477_Mycob 129 AESLFGQRLAVHSDFLGEQLQPLLDLTRRPPQVMRLQQRVNSPGWRRAMAARKRIDDLIDAQIADARTAP 198

CYP139A1_2575647889_Mycob 129 AESLFGQRLAVHSDFLGEQLQPLLDLTRRPPQVMRLQQRVNSPGWRRAMAARKRIDDLIDAQIADARTAP 198

CYP139A1_2577429481_Mycob 129 AESLFGQRLAVHSDFLGEQLQPLLDLTRRPPQVMRLQQRVNSPGWRRAMAARKRIDDLIDAQIADARTAP 198

CYP139A1_2578084434_Mycob 129 AESLFGQRLAVHSDFLGEQLQPLLDLTRRPPQVMRLQQRVNSPGWRRAMAARKRIDDLIDAQIADARTAP 198

CYP139A1_2580301723_Mycob 129 AESLFGQRLAVHSDFLGEQLQPLLDLTRRPPQVMRLQQRVNSPGWRRAMAARKRIDDLIDAQIADARTAP 198

CYP139A1_2581901399_Mycob 129 AESLFGQRLAVHSDFLGEQLQPLLDLTRRPPQVMRLQQRVNSPGWRRAMAARKRIDDLIDAQIADARTAP 198

CYP139A1_2584689961_Mycob 129 AESLFGQRLAVHSDFLGEQLQPLLDLTRRPPQVMRLQQRVNSPGWRRAMAARKRIDDLIDAQIADARTAP 198

CYP139A1_2584748012_Mycob 129 AESLFGQRLAVHSDFLGEQLQPLLDLTRRPPQVMRLQQRVNSPGWRRAMAARKRIDDLIDAQIADARTAP 198

CYP139A1_2584878425_Mycob 129 AESLFGQRLAVHSDFLGEQLQPLLDLTRRPPQVMRLQQRVNSPGWRRAMAARKRIDDLIDAQIADARTAP 198

CYP139A1_2584990570_Mycob 129 AESLFGQRLAVHSDFLGEQLQPLLDLTRRPPQVMRLQQRVNSPGWRRAMAARKRIDDLIDAQIADARTAP 198

CYP139A1_2590014899_Mycob 129 AESLFGQRLAVHSDFLGEQLQPLLDLTRRPPQVMRLQQRVNSPGWRRAMAARKRIDDLIDAQIADARTAP 198

CYP139A1_2590313607_Mycob 129 AESLFGQRLAVHSDFLGEQLQPLLDLTRRPPQVMRLQQRVNSPGWRRAMAARKRIDDLIDAQIADARTAP 198

CYP139A1_2590563570_Mycob 129 AESLFGQRLAVHSDFLGEQLQPLLDLTRRPPQVMRLQQRVNSPGWRRAMAARKRIDDLIDAQIADARTAP 198

CYP139A1_2574682482_Mycob 129 AESLFGQRLAVHSDFLGEQLQPLLDLTRRPPQVMRLQQRVNSPGWRRAMAARKRIDDLIDAQIADARTAP 198

CYP139A1_2574937709_Mycob 129 AESLFGQRLAVHSDFLGEQLQPLLDLTRRPPQVMRLQQRVNSPGWRRAMAARKRIDDLIDAQIADARTAP 198

CYP139A1_2575051511_Mycob 129 AESLFGQRLAVHSDFLGEQLQPLLDLTRRPPQVMRLQQRVNSPGWRRAMAARKRIDDLIDAQIADARTAP 198

CYP139A1_2575142685_Mycob 129 AESLFGQRLAVHSDFLGEQLQPLLDLTRRPPQVMRLQQRVNSPGWRRAMAARKRIDDLIDAQIADARTAP 198

CYP139A1_2575538950_Mycob 129 AESLFGQRLAVHSDFLGEQLQPLLDLTRRPPQVMRLQQRVNSPGWRRAMAARKRIDDLIDAQIADARTAP 198

CYP139A1_2575858812_Mycob 129 AESLFGQRLAVHSDFLGEQLQPLLDLTRRPPQVMRLQQRVNSPGWRRAMAARKRIDDLIDAQIADARTAP 198

CYP139A1_2576059958_Mycob 129 AESLFGQRLAVHSDFLGEQLQPLLDLTRRPPQVMRLQQRVNSPGWRRAMAARKRIDDLIDAQIADARTAP 198

CYP139A1_2576471218_Mycob 129 AESLFGQRLAVHSDFLGEQLQPLLDLTRRPPQVMRLQQRVNSPGWRRAMAARKRIDDLIDAQIADARTAP 198

CYP139A1_2576497080_Mycob 129 AESLFGQRLAVHSDFLGEQLQPLLDLTRRPPQVMRLQQRVNSPGWRRAMAARKRIDDLIDAQIADARTAP 198

CYP139A1_2577038023_Mycob 129 AESLFGQRLAVHSDFLGEQLQPLLDLTRRPPQVMRLQQRVNSPGWRRAMAARKRIDDLIDAQIADARTAP 198

CYP139A1_2577203844_Mycob 129 AESLFGQRLAVHSDFLGEQLQPLLDLTRRPPQVMRLQQRVNSPGWRRAMAARKRIDDLIDAQIADARTAP 198

CYP139A1_2577322951_Mycob 129 AESLFGQRLAVHSDFLGEQLQPLLDLTRRPPQVMRLQQRVNSPGWRRAMAARKRIDDLIDAQIADARTAP 198

CYP139A1_2577422627_Mycob 129 AESLFGQRLAVHSDFLGEQLQPLLDLTRRPPQVMRLQQRVNSPGWRRAMAARKRIDDLIDAQIADARTAP 198

CYP139A1_2577551091_Mycob 129 AESLFGQRLAVHSDFLGEQLQPLLDLTRRPPQVMRLQQRVNSPGWRRAMAARKRIDDLIDAQIADARTAP 198

CYP139A1_2584755863_Mycob 129 AESLFGQRLAVHSDFLGEQLQPLLDLTRRPPQVMRLQQRVNSPGWRRAMAARKRIDDLIDAQIADARTAP 198

CYP139A1_2589117553_Mycob 129 AESLFGQRLAVHSDFLGEQLQPLLDLTRRPPQVMRLQQRVNSPGWRRAMAARKRIDDLIDAQIADARTAP 198

CYP139A1_2589121735_Mycob 129 AESLFGQRLAVHSDFLGEQLQPLLDLTRRPPQVMRLQQRVNSPGWRRAMAARKRIDDLIDAQIADARTAP 198

CYP139A1_2589506393_Mycob 129 AESLFGQRLAVHSDFLGEQLQPLLDLTRRPPQVMRLQQRVNSPGWRRAMAARKRIDDLIDAQIADARTAP 198

CYP139A1_2590044637_Mycob 129 AESLFGQRLAVHSDFLGEQLQPLLDLTRRPPQVMRLQQRVNSPGWRRAMAARKRIDDLIDAQIADARTAP 198

CYP139A1_2590137275_Mycob 129 AESLFGQRLAVHSDFLGEQLQPLLDLTRRPPQVMRLQQRVNSPGWRRAMAARKRIDDLIDAQIADARTAP 198

CYP139A1_2590345757_Mycob 129 AESLFGQRLAVHSDFLGEQLQPLLDLTRRPPQVMRLQQRVNSPGWRRAMAARKRIDDLIDAQIADARTAP 198

CYP139A1_2592275464_Mycob 129 AESLFGQRLAVHSDFLGEQLQPLLDLTRRPPQVMRLQQRVNSPGWRRAMAARKRIDDLIDAQIADARTAP 198

CYP139A1_2575515927_Mycob 129 AESLFGQRLAVHSDFLGEQLQPLLDLTRRPPQVMRLQQRVNSPGWRRAMAARKRIDDLIDAQIADARTAP 198

CYP139A1_2576459372_Mycob 129 AESLFGQRLAVHSDFLGEQLQPLLDLTRRPPQVMRLQQRVNSPGWRRAMAARKRIDDLIDAQIADARTAP 198

CYP139A1_2576522568_Mycob 129 AESLFGQRLAVHSDFLGEQLQPLLDLTRRPPQVMRLQQRVNSPGWRRAMAARKRIDDLIDAQIADARTAP 198

CYP139A1_2576559538_Mycob 129 AESLFGQRLAVHSDFLGEQLQPLLDLTRRPPQVMRLQQRVNSPGWRRAMAARKRIDDLIDAQIADARTAP 198

CYP139A1_2577302440_Mycob 129 AESLFGQRLAVHSDFLGEQLQPLLDLTRRPPQVMRLQQRVNSPGWRRAMAARKRIDDLIDAQIADARTAP 198

CYP139A1_2577448440_Mycob 129 AESLFGQRLAVHSDFLGEQLQPLLDLTRRPPQVMRLQQRVNSPGWRRAMAARKRIDDLIDAQIADARTAP 198

CYP139A1_2577462548_Mycob 129 AESLFGQRLAVHSDFLGEQLQPLLDLTRRPPQVMRLQQRVNSPGWRRAMAARKRIDDLIDAQIADARTAP 198

CYP139A1_2584678567_Mycob 129 AESLFGQRLAVHSDFLGEQLQPLLDLTRRPPQVMRLQQRVNSPGWRRAMAARKRIDDLIDAQIADARTAP 198

CYP139A1_2584763258_Mycob 129 AESLFGQRLAVHSDFLGEQLQPLLDLTRRPPQVMRLQQRVNSPGWRRAMAARKRIDDLIDAQIADARTAP 198

CYP139A1_2584949783_Mycob 129 AESLFGQRLAVHSDFLGEQLQPLLDLTRRPPQVMRLQQRVNSPGWRRAMAARKRIDDLIDAQIADARTAP 198

CYP139A1_2589146274_Mycob 129 AESLFGQRLAVHSDFLGEQLQPLLDLTRRPPQVMRLQQRVNSPGWRRAMAARKRIDDLIDAQIADARTAP 198

CYP139A1_2590089378_Mycob 129 AESLFGQRLAVHSDFLGEQLQPLLDLTRRPPQVMRLQQRVNSPGWRRAMAARKRIDDLIDAQIADARTAP 198

CYP139A1_2590288661_Mycob 129 AESLFGQRLAVHSDFLGEQLQPLLDLTRRPPQVMRLQQRVNSPGWRRAMAARKRIDDLIDAQIADARTAP 198

CYP139A1_2590513765_Mycob 129 AESLFGQRLAVHSDFLGEQLQPLLDLTRRPPQVMRLQQRVNSPGWRRAMAARKRIDDLIDAQIADARTAP 198

CYP139A1_643045086_Mycoba 129 AESLFGQRLAVHSDFLGEQLQPLLDLTRRPPQVMRLQQRVNSPGWRRAMAARKRIDDLIDAQIADARTAP 198

CYP139A1_646014426_Mycoba 129 AESLFGQRLAVHSDFLGEQLQPLLDLTRRPPQVMRLQQRVNSPGWRRAMAARKRIDDLIDAQIADARTAP 198

CYP139A1_2574985446_Mycob 129 AESLFGQRLAVHSDFLGEQLQPLLDLTRRPPQVMRLQQRVNSPGWRRAMAARKRIDDLIDAQIADARTAP 198

CYP139A1_2575103505_Mycob 129 AESLFGQRLAVHSDFLGEQLQPLLDLTRRPPQVMRLQQRVNSPGWRRAMAARKRIDDLIDAQIADARTAP 198

CYP139A1_2575806159_Mycob 129 AESLFGQRLAVHSDFLGEQLQPLLDLTRRPPQVMRLQQRVNSPGWRRAMAARKRIDDLIDAQIADARTAP 198

CYP139A1_2576156796_Mycob 129 AESLFGQRLAVHSDFLGEQLQPLLDLTRRPPQVMRLQQRVNSPGWRRAMAARKRIDDLIDAQIADARTAP 198

CYP139A1_2576936333_Mycob 129 AESLFGQRLAVHSDFLGEQLQPLLDLTRRPPQVMRLQQRVNSPGWRRAMAARKRIDDLIDAQIADARTAP 198

CYP139A1_2577030827_Mycob 129 AESLFGQRLAVHSDFLGEQLQPLLDLTRRPPQVMRLQQRVNSPGWRRAMAARKRIDDLIDAQIADARTAP 198

CYP139A1_2577381729_Mycob 129 AESLFGQRLAVHSDFLGEQLQPLLDLTRRPPQVMRLQQRVNSPGWRRAMAARKRIDDLIDAQIADARTAP 198

CYP139A1_2584698776_Mycob 129 AESLFGQRLAVHSDFLGEQLQPLLDLTRRPPQVMRLQQRVNSPGWRRAMAARKRIDDLIDAQIADARTAP 198

CYP139A1_2584825730_Mycob 129 AESLFGQRLAVHSDFLGEQLQPLLDLTRRPPQVMRLQQRVNSPGWRRAMAARKRIDDLIDAQIADARTAP 198

CYP139A1_2584885951_Mycob 129 AESLFGQRLAVHSDFLGEQLQPLLDLTRRPPQVMRLQQRVNSPGWRRAMAARKRIDDLIDAQIADARTAP 198

CYP139A1_2584902930_Mycob 129 AESLFGQRLAVHSDFLGEQLQPLLDLTRRPPQVMRLQQRVNSPGWRRAMAARKRIDDLIDAQIADARTAP 198

CYP139A1_2588995218_Mycob 129 AESLFGQRLAVHSDFLGEQLQPLLDLTRRPPQVMRLQQRVNSPGWRRAMAARKRIDDLIDAQIADARTAP 198

CYP139A1_2589139119_Mycob 129 AESLFGQRLAVHSDFLGEQLQPLLDLTRRPPQVMRLQQRVNSPGWRRAMAARKRIDDLIDAQIADARTAP 198

CYP139A1_2592238712_Mycob 129 AESLFGQRLAVHSDFLGEQLQPLLDLTRRPPQVMRLQQRVNSPGWRRAMAARKRIDDLIDAQIADARTAP 198

CYP139A1_2592261699_Mycob 129 AESLFGQRLAVHSDFLGEQLQPLLDLTRRPPQVMRLQQRVNSPGWRRAMAARKRIDDLIDAQIADARTAP 198

CYP139A1_2592393538_Mycob 129 AESLFGQRLAVHSDFLGEQLQPLLDLTRRPPQVMRLQQRVNSPGWRRAMAARKRIDDLIDAQIADARTAP 198

CYP139A1_2592397895_Mycob 129 AESLFGQRLAVHSDFLGEQLQPLLDLTRRPPQVMRLQQRVNSPGWRRAMAARKRIDDLIDAQIADARTAP 198

CYP139A1_2592549349_Mycob 129 AESLFGQRLAVHSDFLGEQLQPLLDLTRRPPQVMRLQQRVNSPGWRRAMAARKRIDDLIDAQIADARTAP 198

CYP139A1_648464907_Mycoba 129 AESLFGQRLAVHSDFLGEQLQPLLDLTRRPPQVMRLQQRVNSPGWRRAMAARKRIDDLIDAQIADARTAP 198

CYP139A1_648481186_Mycoba 129 AESLFGQRLAVHSDFLGEQLQPLLDLTRRPPQVMRLQQRVNSPGWRRAMAARKRIDDLIDAQIADARTAP 198

CYP139A1_2574586310_Mycob 129 AESLFGQRLAVHSDFLGEQLQPLLDLTRRPPQVMRLQQRVNSPGWRRAMAARKRIDDLIDAQIADARTAP 198

CYP139A1_2574663269_Mycob 129 AESLFGQRLAVHSDFLGEQLQPLLDLTRRPPQVMRLQQRVNSPGWRRAMAARKRIDDLIDAQIADARTAP 198

CYP139A1_2574880930_Mycob 129 AESLFGQRLAVHSDFLGEQLQPLLDLTRRPPQVMRLQQRVNSPGWRRAMAARKRIDDLIDAQIADARTAP 198

CYP139A1_2574911407_Mycob 129 AESLFGQRLAVHSDFLGEQLQPLLDLTRRPPQVMRLQQRVNSPGWRRAMAARKRIDDLIDAQIADARTAP 198

CYP139A1_2575345775_Mycob 129 AESLFGQRLAVHSDFLGEQLQPLLDLTRRPPQVMRLQQRVNSPGWRRAMAARKRIDDLIDAQIADARTAP 198

CYP139A1_2575468742_Mycob 129 AESLFGQRLAVHSDFLGEQLQPLLDLTRRPPQVMRLQQRVNSPGWRRAMAARKRIDDLIDAQIADARTAP 198

CYP139A1_2576199760_Mycob 129 AESLFGQRLAVHSDFLGEQLQPLLDLTRRPPQVMRLQQRVNSPGWRRAMAARKRIDDLIDAQIADARTAP 198

CYP139A1_2577069926_Mycob 129 AESLFGQRLAVHSDFLGEQLQPLLDLTRRPPQVMRLQQRVNSPGWRRAMAARKRIDDLIDAQIADARTAP 198

CYP139A1_2577613318_Mycob 129 AESLFGQRLAVHSDFLGEQLQPLLDLTRRPPQVMRLQQRVNSPGWRRAMAARKRIDDLIDAQIADARTAP 198

CYP139A1_2577684900_Mycob 129 AESLFGQRLAVHSDFLGEQLQPLLDLTRRPPQVMRLQQRVNSPGWRRAMAARKRIDDLIDAQIADARTAP 198

CYP139A1_2582415442_Mycob 129 AESLFGQRLAVHSDFLGEQLQPLLDLTRRPPQVMRLQQRVNSPGWRRAMAARKRIDDLIDAQIADARTAP 198

CYP139A1_2584812089_Mycob 129 AESLFGQRLAVHSDFLGEQLQPLLDLTRRPPQVMRLQQRVNSPGWRRAMAARKRIDDLIDAQIADARTAP 198

CYP139A1_2590125975_Mycob 129 AESLFGQRLAVHSDFLGEQLQPLLDLTRRPPQVMRLQQRVNSPGWRRAMAARKRIDDLIDAQIADARTAP 198

CYP139A1_2590219901_Mycob 129 AESLFGQRLAVHSDFLGEQLQPLLDLTRRPPQVMRLQQRVNSPGWRRAMAARKRIDDLIDAQIADARTAP 198

CYP139A1_2590227570_Mycob 129 AESLFGQRLAVHSDFLGEQLQPLLDLTRRPPQVMRLQQRVNSPGWRRAMAARKRIDDLIDAQIADARTAP 198

CYP139A1_2590317674_Mycob 129 AESLFGQRLAVHSDFLGEQLQPLLDLTRRPPQVMRLQQRVNSPGWRRAMAARKRIDDLIDAQIADARTAP 198

CYP139A1_641814886_Mycoba 129 AESLFGQRLAVHSDFLGEQLQPLLDLTRRPPQVMRLQQRVNSPGWRRAMAARKRIDDLIDAQIADARTAP 198

CYP139A1_2574738070_Mycob 129 AESLFGQRLAVHSDFLGEQLQPLLDLTRRPPQVMRLQQRVNSPGWRRAMAARKRIDDLIDAQIADARTAP 198

CYP139A1_2575365848_Mycob 129 AESLFGQRLAVHSDFLGEQLQPLLDLTRRPPQVMRLQQRVNSPGWRRAMAARKRIDDLIDAQIADARTAP 198

CYP139A1_2575674521_Mycob 129 AESLFGQRLAVHSDFLGEQLQPLLDLTRRPPQVMRLQQRVNSPGWRRAMAARKRIDDLIDAQIADARTAP 198

CYP139A1_2575772716_Mycob 129 AESLFGQRLAVHSDFLGEQLQPLLDLTRRPPQVMRLQQRVNSPGWRRAMAARKRIDDLIDAQIADARTAP 198

CYP139A1_2576544659_Mycob 129 AESLFGQRLAVHSDFLGEQLQPLLDLTRRPPQVMRLQQRVNSPGWRRAMAARKRIDDLIDAQIADARTAP 198

CYP139A1_2577168470_Mycob 129 AESLFGQRLAVHSDFLGEQLQPLLDLTRRPPQVMRLQQRVNSPGWRRAMAARKRIDDLIDAQIADARTAP 198

CYP139A1_2577390930_Mycob 129 AESLFGQRLAVHSDFLGEQLQPLLDLTRRPPQVMRLQQRVNSPGWRRAMAARKRIDDLIDAQIADARTAP 198

CYP139A1_2577651582_Mycob 129 AESLFGQRLAVHSDFLGEQLQPLLDLTRRPPQVMRLQQRVNSPGWRRAMAARKRIDDLIDAQIADARTAP 198

CYP139A1_2578002537_Mycob 129 AESLFGQRLAVHSDFLGEQLQPLLDLTRRPPQVMRLQQRVNSPGWRRAMAARKRIDDLIDAQIADARTAP 198

CYP139A1_2578230674_Mycob 129 AESLFGQRLAVHSDFLGEQLQPLLDLTRRPPQVMRLQQRVNSPGWRRAMAARKRIDDLIDAQIADARTAP 198

CYP139A1_2579825786_Mycob 129 AESLFGQRLAVHSDFLGEQLQPLLDLTRRPPQVMRLQQRVNSPGWRRAMAARKRIDDLIDAQIADARTAP 198

CYP139A1_2584923296_Mycob 129 AESLFGQRLAVHSDFLGEQLQPLLDLTRRPPQVMRLQQRVNSPGWRRAMAARKRIDDLIDAQIADARTAP 198

CYP139A1_2584954771_Mycob 129 AESLFGQRLAVHSDFLGEQLQPLLDLTRRPPQVMRLQQRVNSPGWRRAMAARKRIDDLIDAQIADARTAP 198

CYP139A1_2588999278_Mycob 129 AESLFGQRLAVHSDFLGEQLQPLLDLTRRPPQVMRLQQRVNSPGWRRAMAARKRIDDLIDAQIADARTAP 198

CYP139A1_2589044237_Mycob 129 AESLFGQRLAVHSDFLGEQLQPLLDLTRRPPQVMRLQQRVNSPGWRRAMAARKRIDDLIDAQIADARTAP 198

CYP139A1_2589555384_Mycob 129 AESLFGQRLAVHSDFLGEQLQPLLDLTRRPPQVMRLQQRVNSPGWRRAMAARKRIDDLIDAQIADARTAP 198

CYP139A1_2589568736_Mycob 129 AESLFGQRLAVHSDFLGEQLQPLLDLTRRPPQVMRLQQRVNSPGWRRAMAARKRIDDLIDAQIADARTAP 198

CYP139A1_2589715710_Mycob 129 AESLFGQRLAVHSDFLGEQLQPLLDLTRRPPQVMRLQQRVNSPGWRRAMAARKRIDDLIDAQIADARTAP 198

CYP139A1_2592287696_Mycob 129 AESLFGQRLAVHSDFLGEQLQPLLDLTRRPPQVMRLQQRVNSPGWRRAMAARKRIDDLIDAQIADARTAP 198

CYP139A1_2592291479_Mycob 129 AESLFGQRLAVHSDFLGEQLQPLLDLTRRPPQVMRLQQRVNSPGWRRAMAARKRIDDLIDAQIADARTAP 198

CYP139A1_2592430392_Mycob 129 AESLFGQRLAVHSDFLGEQLQPLLDLTRRPPQVMRLQQRVNSPGWRRAMAARKRIDDLIDAQIADARTAP 198

CYP139A1_2574790496_Mycob 129 AESLFGQRLAVHSDFLGEQLQPLLDLTRRPPQVMRLQQRVNSPGWRRAMAARKRIDDLIDAQIADARTAP 198

CYP139A1_2574834868_Mycob 129 AESLFGQRLAVHSDFLGEQLQPLLDLTRRPPQVMRLQQRVNSPGWRRAMAARKRIDDLIDAQIADARTAP 198

CYP139A1_2576151818_Mycob 129 AESLFGQRLAVHSDFLGEQLQPLLDLTRRPPQVMRLQQRVNSPGWRRAMAARKRIDDLIDAQIADARTAP 198

CYP139A1_2578111269_Mycob 129 AESLFGQRLAVHSDFLGEQLQPLLDLTRRPPQVMRLQQRVNSPGWRRAMAARKRIDDLIDAQIADARTAP 198

CYP139A1_2583723841_Mycob 129 AESLFGQRLAVHSDFLGEQLQPLLDLTRRPPQVMRLQQRVNSPGWRRAMAARKRIDDLIDAQIADARTAP 198

CYP139A1_2584631822_Mycob 129 AESLFGQRLAVHSDFLGEQLQPLLDLTRRPPQVMRLQQRVNSPGWRRAMAARKRIDDLIDAQIADARTAP 198

CYP139A1_2584721998_Mycob 129 AESLFGQRLAVHSDFLGEQLQPLLDLTRRPPQVMRLQQRVNSPGWRRAMAARKRIDDLIDAQIADARTAP 198

CYP139A1_2584846770_Mycob 129 AESLFGQRLAVHSDFLGEQLQPLLDLTRRPPQVMRLQQRVNSPGWRRAMAARKRIDDLIDAQIADARTAP 198

CYP139A1_2588591267_Mycob 129 AESLFGQRLAVHSDFLGEQLQPLLDLTRRPPQVMRLQQRVNSPGWRRAMAARKRIDDLIDAQIADARTAP 198

CYP139A1_2590007914_Mycob 129 AESLFGQRLAVHSDFLGEQLQPLLDLTRRPPQVMRLQQRVNSPGWRRAMAARKRIDDLIDAQIADARTAP 198

CYP139A1_2590057066_Mycob 129 AESLFGQRLAVHSDFLGEQLQPLLDLTRRPPQVMRLQQRVNSPGWRRAMAARKRIDDLIDAQIADARTAP 198

CYP139A1_2590061112_Mycob 129 AESLFGQRLAVHSDFLGEQLQPLLDLTRRPPQVMRLQQRVNSPGWRRAMAARKRIDDLIDAQIADARTAP 198

CYP139A1_2590252317_Mycob 129 AESLFGQRLAVHSDFLGEQLQPLLDLTRRPPQVMRLQQRVNSPGWRRAMAARKRIDDLIDAQIADARTAP 198

CYP139A1_2590543931_Mycob 129 AESLFGQRLAVHSDFLGEQLQPLLDLTRRPPQVMRLQQRVNSPGWRRAMAARKRIDDLIDAQIADARTAP 198

CYP139A1_2574560432_Mycob 129 AESLFGQRLAVHSDFLGEQLQPLLDLTRRPPQVMRLQQRVNSPGWRRAMAARKRIDDLIDAQIADARTAP 198

CYP139A1_2575495357_Mycob 129 AESLFGQRLAVHSDFLGEQLQPLLDLTRRPPQVMRLQQRVNSPGWRRAMAARKRIDDLIDAQIADARTAP 198

CYP139A1_2575638157_Mycob 129 AESLFGQRLAVHSDFLGEQLQPLLDLTRRPPQVMRLQQRVNSPGWRRAMAARKRIDDLIDAQIADARTAP 198

CYP139A1_2575697737_Mycob 129 AESLFGQRLAVHSDFLGEQLQPLLDLTRRPPQVMRLQQRVNSPGWRRAMAARKRIDDLIDAQIADARTAP 198

CYP139A1_2575988382_Mycob 129 AESLFGQRLAVHSDFLGEQLQPLLDLTRRPPQVMRLQQRVNSPGWRRAMAARKRIDDLIDAQIADARTAP 198

CYP139A1_2576070324_Mycob 129 AESLFGQRLAVHSDFLGEQLQPLLDLTRRPPQVMRLQQRVNSPGWRRAMAARKRIDDLIDAQIADARTAP 198

CYP139A1_2576613004_Mycob 129 AESLFGQRLAVHSDFLGEQLQPLLDLTRRPPQVMRLQQRVNSPGWRRAMAARKRIDDLIDAQIADARTAP 198

CYP139A1_2577091164_Mycob 129 AESLFGQRLAVHSDFLGEQLQPLLDLTRRPPQVMRLQQRVNSPGWRRAMAARKRIDDLIDAQIADARTAP 198

CYP139A1_2577569748_Mycob 129 AESLFGQRLAVHSDFLGEQLQPLLDLTRRPPQVMRLQQRVNSPGWRRAMAARKRIDDLIDAQIADARTAP 198

CYP139A1_2577697067_Mycob 129 AESLFGQRLAVHSDFLGEQLQPLLDLTRRPPQVMRLQQRVNSPGWRRAMAARKRIDDLIDAQIADARTAP 198

CYP139A1_2584795634_Mycob 129 AESLFGQRLAVHSDFLGEQLQPLLDLTRRPPQVMRLQQRVNSPGWRRAMAARKRIDDLIDAQIADARTAP 198

CYP139A1_2589158600_Mycob 129 AESLFGQRLAVHSDFLGEQLQPLLDLTRRPPQVMRLQQRVNSPGWRRAMAARKRIDDLIDAQIADARTAP 198

CYP139A1_2589491285_Mycob 129 AESLFGQRLAVHSDFLGEQLQPLLDLTRRPPQVMRLQQRVNSPGWRRAMAARKRIDDLIDAQIADARTAP 198

CYP139A1_2589613612_Mycob 129 AESLFGQRLAVHSDFLGEQLQPLLDLTRRPPQVMRLQQRVNSPGWRRAMAARKRIDDLIDAQIADARTAP 198

CYP139A1_2589650472_Mycob 129 AESLFGQRLAVHSDFLGEQLQPLLDLTRRPPQVMRLQQRVNSPGWRRAMAARKRIDDLIDAQIADARTAP 198

CYP139A1_2589699410_Mycob 129 AESLFGQRLAVHSDFLGEQLQPLLDLTRRPPQVMRLQQRVNSPGWRRAMAARKRIDDLIDAQIADARTAP 198

CYP139A1_2590366449_Mycob 129 AESLFGQRLAVHSDFLGEQLQPLLDLTRRPPQVMRLQQRVNSPGWRRAMAARKRIDDLIDAQIADARTAP 198

CYP139A1_2592226489_Mycob 129 AESLFGQRLAVHSDFLGEQLQPLLDLTRRPPQVMRLQQRVNSPGWRRAMAARKRIDDLIDAQIADARTAP 198

CYP139A1_2592574645_Mycob 129 AESLFGQRLAVHSDFLGEQLQPLLDLTRRPPQVMRLQQRVNSPGWRRAMAARKRIDDLIDAQIADARTAP 198

CYP139A1_648473432_Mycoba 129 AESLFGQRLAVHSDFLGEQLQPLLDLTRRPPQVMRLQQRVNSPGWRRAMAARKRIDDLIDAQIADARTAP 198

CYP139A1_2574703562_Mycob 129 AESLFGQRLAVHSDFLGEQLQPLLDLTRRPPQVMRLQQRVNSPGWRRAMAARKRIDDLIDAQIADARTAP 198

CYP139A1_2575794202_Mycob 129 AESLFGQRLAVHSDFLGEQLQPLLDLTRRPPQVMRLQQRVNSPGWRRAMAARKRIDDLIDAQIADARTAP 198

CYP139A1_2576967330_Mycob 129 AESLFGQRLAVHSDFLGEQLQPLLDLTRRPPQVMRLQQRVNSPGWRRAMAARKRIDDLIDAQIADARTAP 198

CYP139A1_2577659800_Mycob 129 AESLFGQRLAVHSDFLGEQLQPLLDLTRRPPQVMRLQQRVNSPGWRRAMAARKRIDDLIDAQIADARTAP 198

CYP139A1_2577918576_Mycob 129 AESLFGQRLAVHSDFLGEQLQPLLDLTRRPPQVMRLQQRVNSPGWRRAMAARKRIDDLIDAQIADARTAP 198

CYP139A1_2578033540_Mycob 129 AESLFGQRLAVHSDFLGEQLQPLLDLTRRPPQVMRLQQRVNSPGWRRAMAARKRIDDLIDAQIADARTAP 198

CYP139A1_2581869776_Mycob 129 AESLFGQRLAVHSDFLGEQLQPLLDLTRRPPQVMRLQQRVNSPGWRRAMAARKRIDDLIDAQIADARTAP 198

CYP139A1_2583745288_Mycob 129 AESLFGQRLAVHSDFLGEQLQPLLDLTRRPPQVMRLQQRVNSPGWRRAMAARKRIDDLIDAQIADARTAP 198

CYP139A1_2590086658_Mycob 129 AESLFGQRLAVHSDFLGEQLQPLLDLTRRPPQVMRLQQRVNSPGWRRAMAARKRIDDLIDAQIADARTAP 198

CYP139A1_2590104693_Mycob 129 AESLFGQRLAVHSDFLGEQLQPLLDLTRRPPQVMRLQQRVNSPGWRRAMAARKRIDDLIDAQIADARTAP 198

CYP139A1_2590187116_Mycob 129 AESLFGQRLAVHSDFLGEQLQPLLDLTRRPPQVMRLQQRVNSPGWRRAMAARKRIDDLIDAQIADARTAP 198

CYP139A1_2590382529_Mycob 129 AESLFGQRLAVHSDFLGEQLQPLLDLTRRPPQVMRLQQRVNSPGWRRAMAARKRIDDLIDAQIADARTAP 198

CYP139A1_2590510255_Mycob 129 AESLFGQRLAVHSDFLGEQLQPLLDLTRRPPQVMRLQQRVNSPGWRRAMAARKRIDDLIDAQIADARTAP 198

CYP139A1_2574635031_Mycob 129 AESLFGQRLAVHSDFLGEQLQPLLDLTRRPPQVMRLQQRVNSPGWRRAMAARKRIDDLIDAQIADARTAP 198

CYP139A1_2576207497_Mycob 129 AESLFGQRLAVHSDFLGEQLQPLLDLTRRPPQVMRLQQRVNSPGWRRAMAARKRIDDLIDAQIADARTAP 198

CYP139A1_2576443603_Mycob 129 AESLFGQRLAVHSDFLGEQLQPLLDLTRRPPQVMRLQQRVNSPGWRRAMAARKRIDDLIDAQIADARTAP 198

CYP139A1_2577241442_Mycob 129 AESLFGQRLAVHSDFLGEQLQPLLDLTRRPPQVMRLQQRVNSPGWRRAMAARKRIDDLIDAQIADARTAP 198

CYP139A1_2578177805_Mycob 129 AESLFGQRLAVHSDFLGEQLQPLLDLTRRPPQVMRLQQRVNSPGWRRAMAARKRIDDLIDAQIADARTAP 198

CYP139A1_2578195506_Mycob 129 AESLFGQRLAVHSDFLGEQLQPLLDLTRRPPQVMRLQQRVNSPGWRRAMAARKRIDDLIDAQIADARTAP 198

CYP139A1_2578246558_Mycob 129 AESLFGQRLAVHSDFLGEQLQPLLDLTRRPPQVMRLQQRVNSPGWRRAMAARKRIDDLIDAQIADARTAP 198

CYP139A1_2584652546_Mycob 129 AESLFGQRLAVHSDFLGEQLQPLLDLTRRPPQVMRLQQRVNSPGWRRAMAARKRIDDLIDAQIADARTAP 198

CYP139A1_2584737038_Mycob 129 AESLFGQRLAVHSDFLGEQLQPLLDLTRRPPQVMRLQQRVNSPGWRRAMAARKRIDDLIDAQIADARTAP 198

CYP139A1_2584918375_Mycob 129 AESLFGQRLAVHSDFLGEQLQPLLDLTRRPPQVMRLQQRVNSPGWRRAMAARKRIDDLIDAQIADARTAP 198

CYP139A1_2584974532_Mycob 129 AESLFGQRLAVHSDFLGEQLQPLLDLTRRPPQVMRLQQRVNSPGWRRAMAARKRIDDLIDAQIADARTAP 198

CYP139A1_2588987052_Mycob 129 AESLFGQRLAVHSDFLGEQLQPLLDLTRRPPQVMRLQQRVNSPGWRRAMAARKRIDDLIDAQIADARTAP 198

CYP139A1_2589098383_Mycob 129 AESLFGQRLAVHSDFLGEQLQPLLDLTRRPPQVMRLQQRVNSPGWRRAMAARKRIDDLIDAQIADARTAP 198

CYP139A1_2589486069_Mycob 129 AESLFGQRLAVHSDFLGEQLQPLLDLTRRPPQVMRLQQRVNSPGWRRAMAARKRIDDLIDAQIADARTAP 198

CYP139A1_2589522796_Mycob 129 AESLFGQRLAVHSDFLGEQLQPLLDLTRRPPQVMRLQQRVNSPGWRRAMAARKRIDDLIDAQIADARTAP 198

CYP139A1_2589616566_Mycob 129 AESLFGQRLAVHSDFLGEQLQPLLDLTRRPPQVMRLQQRVNSPGWRRAMAARKRIDDLIDAQIADARTAP 198

CYP139A1_2592250930_Mycob 129 AESLFGQRLAVHSDFLGEQLQPLLDLTRRPPQVMRLQQRVNSPGWRRAMAARKRIDDLIDAQIADARTAP 198

CYP139A1_2592303254_Mycob 129 AESLFGQRLAVHSDFLGEQLQPLLDLTRRPPQVMRLQQRVNSPGWRRAMAARKRIDDLIDAQIADARTAP 198

CYP139A1_2592308116_Mycob 129 AESLFGQRLAVHSDFLGEQLQPLLDLTRRPPQVMRLQQRVNSPGWRRAMAARKRIDDLIDAQIADARTAP 198

CYP139A1_2592385592_Mycob 129 AESLFGQRLAVHSDFLGEQLQPLLDLTRRPPQVMRLQQRVNSPGWRRAMAARKRIDDLIDAQIADARTAP 198

CYP139A1_2592417392_Mycob 129 AESLFGQRLAVHSDFLGEQLQPLLDLTRRPPQVMRLQQRVNSPGWRRAMAARKRIDDLIDAQIADARTAP 198

CYP139A1_643049582_Mycoba 129 AESLFGQRLAVHSDFLGEQLQPLLDLTRRPPQVMRLQQRVNSPGWRRAMAARKRIDDLIDAQIADARTAP 198

CYP139A1_648460473_Mycoba 129 AESLFGQRLAVHSDFLGEQLQPLLDLTRRPPQVMRLQQRVNSPGWRRAMAARKRIDDLIDAQIADARTAP 198

CYP139A1_2574901332_Mycob 129 AESLFGQRLAVHSDFLGEQLQPLLDLTRRPPQVMRLQQRVNSPGWRRAMAARKRIDDLIDAQIADARTAP 198

CYP139A1_2575325434_Mycob 129 AESLFGQRLAVHSDFLGEQLQPLLDLTRRPPQVMRLQQRVNSPGWRRAMAARKRIDDLIDAQIADARTAP 198

CYP139A1_2576274603_Mycob 129 AESLFGQRLAVHSDFLGEQLQPLLDLTRRPPQVMRLQQRVNSPGWRRAMAARKRIDDLIDAQIADARTAP 198

CYP139A1_2583719805_Mycob 129 AESLFGQRLAVHSDFLGEQLQPLLDLTRRPPQVMRLQQRVNSPGWRRAMAARKRIDDLIDAQIADARTAP 198

CYP139A1_2590012031_Mycob 129 AESLFGQRLAVHSDFLGEQLQPLLDLTRRPPQVMRLQQRVNSPGWRRAMAARKRIDDLIDAQIADARTAP 198

CYP139A_2590028529_Mycoba 129 AESLFGQRLAVHSDFLGEQLQPLLDLTRRPPQVMRLQQRVNSPGWRRAMAARKRIDDLIDAQIADARTAP 198

CYP139A1_2590146409_Mycob 129 AESLFGQRLAVHSDFLGEQLQPLLDLTRRPPQVMRLQQRVNSPGWRRAMAARKRIDDLIDAQIADARTAP 198

CYP139A1_2590203420_Mycob 129 AESLFGQRLAVHSDFLGEQLQPLLDLTRRPPQVMRLQQRVNSPGWRRAMAARKRIDDLIDAQIADARTAP 198

CYP139A1_2590272661_Mycob 129 AESLFGQRLAVHSDFLGEQLQPLLDLTRRPPQVMRLQQRVNSPGWRRAMAARKRIDDLIDAQIADARTAP 198

CYP139A1_2590535240_Mycob 129 AESLFGQRLAVHSDFLGEQLQPLLDLTRRPPQVMRLQQRVNSPGWRRAMAARKRIDDLIDAQIADARTAP 198

CYP139A1_647090515_Mycoba 129 AESLFGQRLAVHSDFLGEQLQPLLDLTRRPPQVMRLQQRVNSPGWRRAMAARKRIDDLIDAQIADARTAP 198

CYP139A1_2512786915_Mycob 129 AESLFGQRLAVHSDFLGEQLQPLLDLTRRPPQVMRLQQRVNSPGWRRAMAARKRIDDLIDAQIADARTAP 198

CYP139A1_2575329342_Mycob 129 AESLFGQRLAVHSDFLGEQLQPLLDLTRRPPQVMRLQQRVNSPGWRRAMAARKRIDDLIDAQIADARTAP 198

CYP139A1_2575409751_Mycob 129 AESLFGQRLAVHSDFLGEQLQPLLDLTRRPPQVMRLQQRVNSPGWRRAMAARKRIDDLIDAQIADARTAP 198

CYP139A1_2575862603_Mycob 129 AESLFGQRLAVHSDFLGEQLQPLLDLTRRPPQVMRLQQRVNSPGWRRAMAARKRIDDLIDAQIADARTAP 198

CYP139A1_2576589126_Mycob 129 AESLFGQRLAVHSDFLGEQLQPLLDLTRRPPQVMRLQQRVNSPGWRRAMAARKRIDDLIDAQIADARTAP 198

CYP139A1_2576649037_Mycob 129 AESLFGQRLAVHSDFLGEQLQPLLDLTRRPPQVMRLQQRVNSPGWRRAMAARKRIDDLIDAQIADARTAP 198

CYP139A1_2576746511_Mycob 129 AESLFGQRLAVHSDFLGEQLQPLLDLTRRPPQVMRLQQRVNSPGWRRAMAARKRIDDLIDAQIADARTAP 198

CYP139A1_2577129296_Mycob 129 AESLFGQRLAVHSDFLGEQLQPLLDLTRRPPQVMRLQQRVNSPGWRRAMAARKRIDDLIDAQIADARTAP 198

CYP139A1_2578200424_Mycob 129 AESLFGQRLAVHSDFLGEQLQPLLDLTRRPPQVMRLQQRVNSPGWRRAMAARKRIDDLIDAQIADARTAP 198

CYP139A1_2584666311_Mycob 129 AESLFGQRLAVHSDFLGEQLQPLLDLTRRPPQVMRLQQRVNSPGWRRAMAARKRIDDLIDAQIADARTAP 198

CYP139A1_2584743940_Mycob 129 AESLFGQRLAVHSDFLGEQLQPLLDLTRRPPQVMRLQQRVNSPGWRRAMAARKRIDDLIDAQIADARTAP 198

CYP139A1_2584781205_Mycob 129 AESLFGQRLAVHSDFLGEQLQPLLDLTRRPPQVMRLQQRVNSPGWRRAMAARKRIDDLIDAQIADARTAP 198

CYP139A1_2584833644_Mycob 129 AESLFGQRLAVHSDFLGEQLQPLLDLTRRPPQVMRLQQRVNSPGWRRAMAARKRIDDLIDAQIADARTAP 198

CYP139A1_2589073023_Mycob 129 AESLFGQRLAVHSDFLGEQLQPLLDLTRRPPQVMRLQQRVNSPGWRRAMAARKRIDDLIDAQIADARTAP 198

CYP139A1_2589509569_Mycob 129 AESLFGQRLAVHSDFLGEQLQPLLDLTRRPPQVMRLQQRVNSPGWRRAMAARKRIDDLIDAQIADARTAP 198

CYP139A1_2589551314_Mycob 129 AESLFGQRLAVHSDFLGEQLQPLLDLTRRPPQVMRLQQRVNSPGWRRAMAARKRIDDLIDAQIADARTAP 198

CYP139A1_2589580598_Mycob 129 AESLFGQRLAVHSDFLGEQLQPLLDLTRRPPQVMRLQQRVNSPGWRRAMAARKRIDDLIDAQIADARTAP 198

CYP139A1_2589629003_Mycob 129 AESLFGQRLAVHSDFLGEQLQPLLDLTRRPPQVMRLQQRVNSPGWRRAMAARKRIDDLIDAQIADARTAP 198

CYP139A1_2589676017_Mycob 129 AESLFGQRLAVHSDFLGEQLQPLLDLTRRPPQVMRLQQRVNSPGWRRAMAARKRIDDLIDAQIADARTAP 198

CYP139A1_2592332604_Mycob 129 AESLFGQRLAVHSDFLGEQLQPLLDLTRRPPQVMRLQQRVNSPGWRRAMAARKRIDDLIDAQIADARTAP 198

CYP139A1_2592542016_Mycob 129 AESLFGQRLAVHSDFLGEQLQPLLDLTRRPPQVMRLQQRVNSPGWRRAMAARKRIDDLIDAQIADARTAP 198

CYP139A1_2592562396_Mycob 129 AESLFGQRLAVHSDFLGEQLQPLLDLTRRPPQVMRLQQRVNSPGWRRAMAARKRIDDLIDAQIADARTAP 198

CYP139A1_2541578033_Mycob 129 AESLFGQRLAVHSDFLGEQLQPLLDLTRRPPQVMRLQQRVNSPGWRRAMAARKRIDDLIDAQIADARTAP 198

CYP139A1_2574761421_Mycob 129 AESLFGQRLAVHSDFLGEQLQPLLDLTRRPPQVMRLQQRVNSPGWRRAMAARKRIDDLIDAQIADARTAP 198

CYP139A1_2575611823_Mycob 129 AESLFGQRLAVHSDFLGEQLQPLLDLTRRPPQVMRLQQRVNSPGWRRAMAARKRIDDLIDAQIADARTAP 198

CYP139A1_2576564801_Mycob 129 AESLFGQRLAVHSDFLGEQLQPLLDLTRRPPQVMRLQQRVNSPGWRRAMAARKRIDDLIDAQIADARTAP 198

CYP139A1_2577821955_Mycob 129 AESLFGQRLAVHSDFLGEQLQPLLDLTRRPPQVMRLQQRVNSPGWRRAMAARKRIDDLIDAQIADARTAP 198

CYP139A1_2578086064_Mycob 129 AESLFGQRLAVHSDFLGEQLQPLLDLTRRPPQVMRLQQRVNSPGWRRAMAARKRIDDLIDAQIADARTAP 198

CYP139A1_2580669105_Mycob 129 AESLFGQRLAVHSDFLGEQLQPLLDLTRRPPQVMRLQQRVNSPGWRRAMAARKRIDDLIDAQIADARTAP 198

CYP139A1_2583740670_Mycob 129 AESLFGQRLAVHSDFLGEQLQPLLDLTRRPPQVMRLQQRVNSPGWRRAMAARKRIDDLIDAQIADARTAP 198

CYP139A1_2584831832_Mycob 129 AESLFGQRLAVHSDFLGEQLQPLLDLTRRPPQVMRLQQRVNSPGWRRAMAARKRIDDLIDAQIADARTAP 198

CYP139A1_2584875064_Mycob 129 AESLFGQRLAVHSDFLGEQLQPLLDLTRRPPQVMRLQQRVNSPGWRRAMAARKRIDDLIDAQIADARTAP 198

CYP139A1_2590158810_Mycob 129 AESLFGQRLAVHSDFLGEQLQPLLDLTRRPPQVMRLQQRVNSPGWRRAMAARKRIDDLIDAQIADARTAP 198

CYP139A1_2590240089_Mycob 129 AESLFGQRLAVHSDFLGEQLQPLLDLTRRPPQVMRLQQRVNSPGWRRAMAARKRIDDLIDAQIADARTAP 198

CYP139A1_2590350138_Mycob 129 AESLFGQRLAVHSDFLGEQLQPLLDLTRRPPQVMRLQQRVNSPGWRRAMAARKRIDDLIDAQIADARTAP 198

CYP139A1_2590493738_Mycob 129 AESLFGQRLAVHSDFLGEQLQPLLDLTRRPPQVMRLQQRVNSPGWRRAMAARKRIDDLIDAQIADARTAP 198

CYP139A1_2590554696_Mycob 129 AESLFGQRLAVHSDFLGEQLQPLLDLTRRPPQVMRLQQRVNSPGWRRAMAARKRIDDLIDAQIADARTAP 198

CYP139A1_2576084283_Mycob 129 AESLFGQRLAVHSDFLGEQLQPLLDLTRRPPQVMRLQQRVNSPGWRRAMAARKRIDDLIDAQIADARTAP 198

CYP139A1_2576366402_Mycob 129 AESLFGQRLAVHSDFLGEQLQPLLDLTRRPPQVMRLQQRVNSPGWRRAMAARKRIDDLIDAQIADARTAP 198

CYP139A1_2576513411_Mycob 129 AESLFGQRLAVHSDFLGEQLQPLLDLTRRPPQVMRLQQRVNSPGWRRAMAARKRIDDLIDAQIADARTAP 198

CYP139A1_2576671439_Mycob 129 AESLFGQRLAVHSDFLGEQLQPLLDLTRRPPQVMRLQQRVNSPGWRRAMAARKRIDDLIDAQIADARTAP 198

CYP139A1_2577792721_Mycob 129 AESLFGQRLAVHSDFLGEQLQPLLDLTRRPPQVMRLQQRVNSPGWRRAMAARKRIDDLIDAQIADARTAP 198

CYP139A1_2577796885_Mycob 129 AESLFGQRLAVHSDFLGEQLQPLLDLTRRPPQVMRLQQRVNSPGWRRAMAARKRIDDLIDAQIADARTAP 198

CYP139A1_2584686721_Mycob 129 AESLFGQRLAVHSDFLGEQLQPLLDLTRRPPQVMRLQQRVNSPGWRRAMAARKRIDDLIDAQIADARTAP 198

CYP139A1_2584752944_Mycob 129 AESLFGQRLAVHSDFLGEQLQPLLDLTRRPPQVMRLQQRVNSPGWRRAMAARKRIDDLIDAQIADARTAP 198

CYP139A1_2584808933_Mycob 129 AESLFGQRLAVHSDFLGEQLQPLLDLTRRPPQVMRLQQRVNSPGWRRAMAARKRIDDLIDAQIADARTAP 198

CYP139A1_2589134136_Mycob 129 AESLFGQRLAVHSDFLGEQLQPLLDLTRRPPQVMRLQQRVNSPGWRRAMAARKRIDDLIDAQIADARTAP 198

CYP139A1_2589539130_Mycob 129 AESLFGQRLAVHSDFLGEQLQPLLDLTRRPPQVMRLQQRVNSPGWRRAMAARKRIDDLIDAQIADARTAP 198

CYP139A1_2589598915_Mycob 129 AESLFGQRLAVHSDFLGEQLQPLLDLTRRPPQVMRLQQRVNSPGWRRAMAARKRIDDLIDAQIADARTAP 198

CYP139A1_2589679190_Mycob 129 AESLFGQRLAVHSDFLGEQLQPLLDLTRRPPQVMRLQQRVNSPGWRRAMAARKRIDDLIDAQIADARTAP 198

CYP139A1_2589727979_Mycob 129 AESLFGQRLAVHSDFLGEQLQPLLDLTRRPPQVMRLQQRVNSPGWRRAMAARKRIDDLIDAQIADARTAP 198

CYP139A1_2592361217_Mycob 129 AESLFGQRLAVHSDFLGEQLQPLLDLTRRPPQVMRLQQRVNSPGWRRAMAARKRIDDLIDAQIADARTAP 198

CYP139A1_2592442711_Mycob 129 AESLFGQRLAVHSDFLGEQLQPLLDLTRRPPQVMRLQQRVNSPGWRRAMAARKRIDDLIDAQIADARTAP 198

CYP139A1_2555325132_Mycob 135 AESLFGQRLAVHSDFLGEQLQPLLDLTRRPPQVMRLQQRVNSPGWRRAMAARKRIDDLIDAQIADARTAP 204

CYP139A1_2566259019_Mycob 135 AESLFGQRLAVHSDFLGEQLQPLLDLTRRPPQVMRLQQRVNSPGWRRAMAARKRIDDLIDAQIADARTAP 204

CYP139A1_2555164435_Mycob 135 AESLFGQRLAVHSDFLGEQLQPLLDLTRRPPQVMRLQQRVNSPGWRRAMAARKRIDDLIDAQIADARTAP 204

CYP139A1_2555583039_Mycob 135 AESLFGQRLAVHSDFLGEQLQPLLDLTRRPPQVMRLQQRVNSPGWRRAMAARKRIDDLIDAQIADARTAP 204

CYP139A1_2555295099_Mycob 135 AESLFGQRLAVHSDFLGEQLQPLLDLTRRPPQVMRLQQRVNSPGWRRAMAARKRIDDLIDAQIADARTAP 204

CYP139A1_2555337752_Mycob 135 AESLFGQRLAVHSDFLGEQLQPLLDLTRRPPQVMRLQQRVNSPGWRRAMAARKRIDDLIDAQIADARTAP 204

CYP139A1_2555516018_Mycob 135 AESLFGQRLAVHSDFLGEQLQPLLDLTRRPPQVMRLQQRVNSPGWRRAMAARKRIDDLIDAQIADARTAP 204

CYP139A1_2576196505_Mycob 129 AESLFGQRLAVHSDFLGEQLQPLLDLTRRPPQVMRLQQRVNSPGWRRAMAARKRIDDLIDAQIADARTAP 198

CYP139A1_2555417623_Mycob 135 AESLFGQRLAVHSDFLGEQLQPLLDLTRRPPQVMRLQQRVNSPGWRRAMAARKRIDDLIDAQIADARTAP 204

CYP139A1_2555430098_Mycob 135 AESLFGQRLAVHSDFLGEQLQPLLDLTRRPPQVMRLQQRVNSPGWRRAMAARKRIDDLIDAQIADARTAP 204

CYP139A1_2547880750_Mycob 135 AESLFGQRLAVHSDFLGEQLQPLLDLTRRPPQVMRLQQRVNSPGWRRAMAARKRIDDLIDAQIADARTAP 204

CYP139A1_2559163499_Mycob 135 AESLFGQRLAVHSDFLGEQLQPLLDLTRRPPQVMRLQQRVNSPGWRRAMAARKRIDDLIDAQIADARTAP 204

CYP139A1_2527056892_Mycob 129 AESLFGQRLAVHSDFLGEQLQPLLDLTRRPPQVMRLQQRVNSPGWRRAMAARKRIDDLIDAQIADARTAP 198

CYP139A1_2574675630_Mycob 129 AESLFGQRLAVHSDFLGEQLQPLLDLTRRPPQVMRLQQRVNSPGWRRAMAARKRIDDLIDAQIADARTAP 198

CYP139A1_2574700588_Mycob 129 AESLFGQRLAVHSDFLGEQLQPLLDLTRRPPQVMRLQQRVNSPGWRRAMAARKRIDDLIDAQIADARTAP 198

CYP139A1_2574830606_Mycob 129 AESLFGQRLAVHSDFLGEQLQPLLDLTRRPPQVMRLQQRVNSPGWRRAMAARKRIDDLIDAQIADARTAP 198

CYP139A1_2574928549_Mycob 129 AESLFGQRLAVHSDFLGEQLQPLLDLTRRPPQVMRLQQRVNSPGWRRAMAARKRIDDLIDAQIADARTAP 198

CYP139A1_2575381682_Mycob 129 AESLFGQRLAVHSDFLGEQLQPLLDLTRRPPQVMRLQQRVNSPGWRRAMAARKRIDDLIDAQIADARTAP 198

CYP139A1_2575663406_Mycob 129 AESLFGQRLAVHSDFLGEQLQPLLDLTRRPPQVMRLQQRVNSPGWRRAMAARKRIDDLIDAQIADARTAP 198

CYP139A1_2576431117_Mycob 129 AESLFGQRLAVHSDFLGEQLQPLLDLTRRPPQVMRLQQRVNSPGWRRAMAARKRIDDLIDAQIADARTAP 198

CYP139A1_2576553939_Mycob 129 AESLFGQRLAVHSDFLGEQLQPLLDLTRRPPQVMRLQQRVNSPGWRRAMAARKRIDDLIDAQIADARTAP 198

CYP139A1_2577269438_Mycob 129 AESLFGQRLAVHSDFLGEQLQPLLDLTRRPPQVMRLQQRVNSPGWRRAMAARKRIDDLIDAQIADARTAP 198

CYP139A1_2584870715_Mycob 129 AESLFGQRLAVHSDFLGEQLQPLLDLTRRPPQVMRLQQRVNSPGWRRAMAARKRIDDLIDAQIADARTAP 198

CYP139A1_2589036086_Mycob 129 AESLFGQRLAVHSDFLGEQLQPLLDLTRRPPQVMRLQQRVNSPGWRRAMAARKRIDDLIDAQIADARTAP 198

CYP139A1_2590101605_Mycob 129 AESLFGQRLAVHSDFLGEQLQPLLDLTRRPPQVMRLQQRVNSPGWRRAMAARKRIDDLIDAQIADARTAP 198

CYP139A1_2590354422_Mycob 129 AESLFGQRLAVHSDFLGEQLQPLLDLTRRPPQVMRLQQRVNSPGWRRAMAARKRIDDLIDAQIADARTAP 198

CYP139A1_648490022_Mycoba 129 AESLFGQRLAVHSDFLGEQLQPLLDLTRRPPQVMRLQQRVNSPGWRRAMAARKRIDDLIDAQIADARTAP 198

CYP139A1_2547317188_Mycob 135 AESLFGQRLAVHSDFLGEQLQPLLDLTRRPPQVMRLQQRVNSPGWRRAMAARKRIDDLIDAQIADARTAP 204

CYP139A1_2547959756_Mycob 135 AESLFGQRLAVHSDFLGEQLQPLLDLTRRPPQVMRLQQRVNSPGWRRAMAARKRIDDLIDAQIADARTAP 204

CYP139A1_2555303475_Mycob 135 AESLFGQRLAVHSDFLGEQLQPLLDLTRRPPQVMRLQQRVNSPGWRRAMAARKRIDDLIDAQIADARTAP 204

CYP139A1_2555392373_Mycob 135 AESLFGQRLAVHSDFLGEQLQPLLDLTRRPPQVMRLQQRVNSPGWRRAMAARKRIDDLIDAQIADARTAP 204

CYP139A1_2566985178_Mycob 135 AESLFGQRLAVHSDFLGEQLQPLLDLTRRPPQVMRLQQRVNSPGWRRAMAARKRIDDLIDAQIADARTAP 204

CYP139A1_2555362970_Mycob 135 AESLFGQRLAVHSDFLGEQLQPLLDLTRRPPQVMRLQQRVNSPGWRRAMAARKRIDDLIDAQIADARTAP 204

CYP139A1_2560449536_Mycob 135 AESLFGQRLAVHSDFLGEQLQPLLDLTRRPPQVMRLQQRVNSPGWRRAMAARKRIDDLIDAQIADARTAP 204

CYP139A1_2555152706_Mycob 135 AESLFGQRLAVHSDFLGEQLQPLLDLTRRPPQVMRLQQRVNSPGWRRAMAARKRIDDLIDAQIADARTAP 204

CYP139A1_2555543565_Mycob 135 AESLFGQRLAVHSDFLGEQLQPLLDLTRRPPQVMRLQQRVNSPGWRRAMAARKRIDDLIDAQIADARTAP 204

CYP139A1_2590081259_Mycob 129 AESLFGQRLAVHSDFLGEQLQPLLDLTRRPPQVMRLQQRVNSPGWRRAMAARKRIDDLIDAQIADARTAP 198

CYP139A1_2547311116_Mycob 135 AESLFGQRLAVHSDFLGEQLQPLLDLTRRPPQVMRLQQRVNSPGWRRAMAARKRIDDLIDAQIADARTAP 204

CYP139A1_2548033169_Mycob 135 AESLFGQRLAVHSDFLGEQLQPLLDLTRRPPQVMRLQQRVNSPGWRRAMAARKRIDDLIDAQIADARTAP 204

CYP139A1_2548037418_Mycob 135 AESLFGQRLAVHSDFLGEQLQPLLDLTRRPPQVMRLQQRVNSPGWRRAMAARKRIDDLIDAQIADARTAP 204

CYP139A1_2555282371_Mycob 135 AESLFGQRLAVHSDFLGEQLQPLLDLTRRPPQVMRLQQRVNSPGWRRAMAARKRIDDLIDAQIADARTAP 204

CYP139A1_2541569776_Mycob 129 AESLFGQRLAVHSDFLGEQLQPLLDLTRRPPQVMRLQQRVNSPGWRRAMAARKRIDDLIDAQIADARTAP 198

CYP139A1_2555299255_Mycob 135 AESLFGQRLAVHSDFLGEQLQPLLDLTRRPPQVMRLQQRVNSPGWRRAMAARKRIDDLIDAQIADARTAP 204

CYP139A1_2555379694_Mycob 135 AESLFGQRLAVHSDFLGEQLQPLLDLTRRPPQVMRLQQRVNSPGWRRAMAARKRIDDLIDAQIADARTAP 204

CYP139A1_2555413387_Mycob 135 AESLFGQRLAVHSDFLGEQLQPLLDLTRRPPQVMRLQQRVNSPGWRRAMAARKRIDDLIDAQIADARTAP 204

CYP139A1_2555587223_Mycob 135 AESLFGQRLAVHSDFLGEQLQPLLDLTRRPPQVMRLQQRVNSPGWRRAMAARKRIDDLIDAQIADARTAP 204

CYP139A1_2555591399_Mycob 135 AESLFGQRLAVHSDFLGEQLQPLLDLTRRPPQVMRLQQRVNSPGWRRAMAARKRIDDLIDAQIADARTAP 204

CYP139A1_2547306531_Mycob 135 AESLFGQRLAVHSDFLGEQLQPLLDLTRRPPQVMRLQQRVNSPGWRRAMAARKRIDDLIDAQIADARTAP 204

CYP139A1_651088108_Mycoba 135 AESLFGQRLAVHSDFLGEQLQPLLDLTRRPPQVMRLQQRVNSPGWRRAMAARKRIDDLIDAQIADARTAP 204

CYP139A1_2598067418_Mycob 135 AESLFGQRLAVHSDFLGEQLQPLLDLTRRPPQVMRLQQRVNSPGWRRAMAARKRIDDLIDAQIADARTAP 204

CYP139A1_2555341950_Mycob 135 AESLFGQRLAVHSDFLGEQLQPLLDLTRRPPQVMRLQQRVNSPGWRRAMAARKRIDDLIDAQIADARTAP 204

CYP139A1_2555446824_Mycob 135 AESLFGQRLAVHSDFLGEQLQPLLDLTRRPPQVMRLQQRVNSPGWRRAMAARKRIDDLIDAQIADARTAP 204

CYP139A1_2555556540_Mycob 135 AESLFGQRLAVHSDFLGEQLQPLLDLTRRPPQVMRLQQRVNSPGWRRAMAARKRIDDLIDAQIADARTAP 204

CYP139A1_2560451827_Mycob 135 AESLFGQRLAVHSDFLGEQLQPLLDLTRRPPQVMRLQQRVNSPGWRRAMAARKRIDDLIDAQIADARTAP 204

CYP139A1_2540619998_Mycob 135 AESLFGQRLAVHSDFLGEQLQPLLDLTRRPPQVMRLQQRVNSPGWRRAMAARKRIDDLIDAQIADARTAP 204

CYP139A1_2554692349_Mycob 135 AESLFGQRLAVHSDFLGEQLQPLLDLTRRPPQVMRLQQRVNSPGWRRAMAARKRIDDLIDAQIADARTAP 204

CYP139A1_637096038_Mycoba 135 AESLFGQRLAVHSDFLGEQLQPLLDLTRRPPQVMRLQQRVNSPGWRRAMAARKRIDDLIDAQIADARTAP 204

CYP139A1_2555396597_Mycob 135 AESLFGQRLAVHSDFLGEQLQPLLDLTRRPPQVMRLQQRVNSPGWRRAMAARKRIDDLIDAQIADARTAP 204

CYP139A1_2555520186_Mycob 135 AESLFGQRLAVHSDFLGEQLQPLLDLTRRPPQVMRLQQRVNSPGWRRAMAARKRIDDLIDAQIADARTAP 204

CYP139A1_2555599804_Mycob 135 AESLFGQRLAVHSDFLGEQLQPLLDLTRRPPQVMRLQQRVNSPGWRRAMAARKRIDDLIDAQIADARTAP 204

CYP139A1_2547314995_Mycob 135 AESLFGQRLAVHSDFLGEQLQPLLDLTRRPPQVMRLQQRVNSPGWRRAMAARKRIDDLIDAQIADARTAP 204

CYP139A1_2555160272_Mycob 135 AESLFGQRLAVHSDFLGEQLQPLLDLTRRPPQVMRLQQRVNSPGWRRAMAARKRIDDLIDAQIADARTAP 204

CYP139A1_2555367201_Mycob 135 AESLFGQRLAVHSDFLGEQLQPLLDLTRRPPQVMRLQQRVNSPGWRRAMAARKRIDDLIDAQIADARTAP 204

CYP139A1_2555547894_Mycob 135 AESLFGQRLAVHSDFLGEQLQPLLDLTRRPPQVMRLQQRVNSPGWRRAMAARKRIDDLIDAQIADARTAP 204

CYP139A1_2560454644_Mycob 135 AESLFGQRLAVHSDFLGEQLQPLLDLTRRPPQVMRLQQRVNSPGWRRAMAARKRIDDLIDAQIADARTAP 204

CYP139A1_2546436155_Mycob 129 AESLFGQRLAVHSDFLGEQLQPLLDLTRRPPQVMRLQQRVNSPGWRRAMAARKRIDDLIDAQIADARTAP 198

CYP139A1_2555307696_Mycob 135 AESLFGQRLAVHSDFLGEQLQPLLDLTRRPPQVMRLQQRVNSPGWRRAMAARKRIDDLIDAQIADARTAP 204

CYP139A1_2555524372_Mycob 135 AESLFGQRLAVHSDFLGEQLQPLLDLTRRPPQVMRLQQRVNSPGWRRAMAARKRIDDLIDAQIADARTAP 204

CYP139A1_2546192085_Mycob 129 AESLFGQRLAVHSDFLGEQLQPLLDLXRRPPQVMRLQQRVNSPGWRRAMAARKRIDDLIDAQIADARTAP 198

CYP139A1_2553261414_Mycob 135 AESLFGQRLAVHSDFLGEQLQPLLDLTRRPPQVMRLQQRVNSPGWRRAMAARKRIDDLIDAQIADARTAP 204

CYP139A1_2555442648_Mycob 135 AESLFGQRLAVHSDFLGEQLQPLLDLTRRPPQVMRLQQRVNSPGWRRAMAARKRIDDLIDAQIADARTAP 204

CYP139A1_2555455224_Mycob 135 AESLFGQRLAVHSDFLGEQLQPLLDLTRRPPQVMRLQQRVNSPGWRRAMAARKRIDDLIDAQIADARTAP 204

CYP139A1_651084428_Mycoba 135 AESLFGQRLAVHSDFLGEQLQPLLDLTRRPPQVMRLQQRVNSPGWRRAMAARKRIDDLIDAQIADARTAP 204

CYP139A1_2555273883_Mycob 135 AESLFGQRLAVHSDFLGEQLQPLLDLTRRPPQVMRLQQRVNSPGWRRAMAARKRIDDLIDAQIADARTAP 204

CYP139A1_2555286609_Mycob 135 AESLFGQRLAVHSDFLGEQLQPLLDLTRRPPQVMRLQQRVNSPGWRRAMAARKRIDDLIDAQIADARTAP 204

CYP139A1_2555329342_Mycob 135 AESLFGQRLAVHSDFLGEQLQPLLDLTRRPPQVMRLQQRVNSPGWRRAMAARKRIDDLIDAQIADARTAP 204

CYP139A1_2555438445_Mycob 135 AESLFGQRLAVHSDFLGEQLQPLLDLTRRPPQVMRLQQRVNSPGWRRAMAARKRIDDLIDAQIADARTAP 204

CYP139A1_2577281457_Mycob 135 AESLFGQRLAVHSDFLGEQLQPLLDLTRRPPQVMRLQQRVNSPGWRRAMAARKRIDDLIDAQIADARTAP 204

CYP139A1_2590256393_Mycob 129 AESLFGQRLAVHSDFLGEQLQPLLDLTRRPPQVMRLQQRVNSPGWRRAMAARKRIDDLIDAQIADARTAP 198

CYP139A1_2549407735_Mycob 135 AESLFGQRLAVHSDFLGEQLQPLLDLTRRPPQVMRLQQRVNSPGWRRAMAARKRIDDLIDAQIADARTAP 204

CYP139A1_2573574061_Mycob 135 AESLFGQRLAVHSDFLGEQLQPLLDLTRRPPQVMRLQQRVNSPGWRRAMAARKRIDDLIDAQIADARTTP 204

CYP139A1_2540803840_Mycob 135 AESLFGQRLAVHSDFLGEQLQPLLDLTRRPPQVMRLQQRVNSPGWRRAMAARKRIDDLIDAQIADARTAP 204

CYP139A1_2584769363_Mycob 129 AESLFGQRLAVHSDFLGEQLQPLLDLTRRPPQVMRLQQRVNSPGWRRAMAARKRIDDLIDAQIADARTAP 198

CYP139A1_2555311951_Mycob 135 AESLFGQRLAVHSDFLGEQLQPLLDLTRRPPQVMRLQQRVNSPGWRRAMAARKRIDDLIDAQIADARTAP 204

CYP139A1_2555346108_Mycob 135 AESLFGQRLAVHSDFLGEQLQPLLDLTRRPPQVMRLQQRVNSPGWRRAMAARKRIDDLIDAQIADARTAP 204

CYP139A1_2555383921_Mycob 135 AESLFGQRLAVHSDFLGEQLQPLLDLTRRPPQVMRLQQRVNSPGWRRAMAARKRIDDLIDAQIADARTAP 204

CYP139A1_2555400811_Mycob 135 AESLFGQRLAVHSDFLGEQLQPLLDLTRRPPQVMRLQQRVNSPGWRRAMAARKRIDDLIDAQIADARTAP 204

CYP139A1_2555560826_Mycob 135 AESLFGQRLAVHSDFLGEQLQPLLDLTRRPPQVMRLQQRVNSPGWRRAMAARKRIDDLIDAQIADARTAP 204

CYP139A1_2598813154_Mycob 135 AESLFGQRLAVHSDFLGEQLQPLLDLTRRPPQVMRLQQRVNSPGWRRAMAARKRIDDLIDAQIADARTAP 204

CYP139A1_2547164190_Mycob 135 AESLFGQRLAVHSDFLGEQLQPLLDLTRRPPQVMRLQQRVNSPGWRRAMAARKRIDDLIDAQIADARTAP 204

CYP139A1_2555409181_Mycob 135 AESLFGQRLAVHSDFLGEQLQPLLDLTRRPPQVMRLQQRVNSPGWRRAMAARKRIDDLIDAQIADARTAP 204

CYP139A1_2547759833_Mycob 135 AESLFGQRLAVHSDFLGEQLQPLLDLTRRPPQVMRLQQRVNSPGWRRAMAARKRIDDLIDAQIADARTAP 204

CYP139A1_2547955540_Mycob 135 AESLFGQRLAVHSDFLGEQLQPLLDLTRRPPQVMRLQQRVNSPGWRRAMAARKRIDDLIDAQIADARTAP 204

CYP139A1_2560461324_Mycob 135 AESLFGQRLAVHSDFLGEQLQPLLDLTRRPPQVMRLQQRVNSPGWRRAMAARKRIDDLIDAQIADARTAP 204

CYP139A1_2576392640_Mycob 129 AESLFGQRLAVHSDFLGEQLQPLLDLTRRPPQVMRLQQRVNSPGWRRAMAARKRIDDLIDAQIADARTAP 198

CYP139A1_2541573920_Mycob 129 AESLFGQRLAVHSDFLGEQLQPLLDLTRRPPQVMRLQQRVNSPGWRRAMAARKRIDDLIDAQIADARTAP 198

CYP139A1_2549413118_Mycob 135 AESLFGQRLAVHSDFLGEQLQPLLDLTRRPPQVMRLQQRVNSPGWRRAMAARKRIDDLIDAQIADARTAP 204

CYP139A1_2555333539_Mycob 135 AESLFGQRLAVHSDFLGEQLQPLLDLTRRPPQVMRLQQRVNSPGWRRAMAARKRIDDLIDAQIADARTAP 204

CYP139A1_2555371358_Mycob 135 AESLFGQRLAVHSDFLGEQLQPLLDLTRRPPQVMRLQQRVNSPGWRRAMAARKRIDDLIDAQIADARTAP 204

CYP139A1_2545499027_Mycob 135 AESLFGQRLAVHSDFLGEQLQPLLDLTRRPPQVMRLQQRVNSPGWRRAMAARKRIDDLIDAQIADARTAP 204

CYP139A1_2551812688_Mycob 135 AESLFGQRLAVHSDFLGEQLQPLLDLTRRPPQVMRLQQRVNSPGWRRAMAARKRIDDLIDAQIADARTAP 204

CYP139A1_2620699696_Mycob 135 AESLFGQRLAVHSDFLGEQLQPLLDLTRRPPQVMRLQQRVNSPGWRRAMAARKRIDDLIDAQIADARTAP 204

CYP139A1_2555278104_Mycob 135 AESLFGQRLAVHSDFLGEQLQPLLDLTRRPPQVMRLQQRVNSPGWRRAMAARKRIDDLIDAQIADARTAP 204

CYP139A1_2555320646_Mycob 135 AESLFGQRLAVHSDFLGEQLQPLLDLTRRPPQVMRLQQRVNSPGWRRAMAARKRIDDLIDAQIADARTAP 204

CYP139A1_2555354513_Mycob 135 AESLFGQRLAVHSDFLGEQLQPLLDLTRRPPQVMRLQQRVNSPGWRRAMAARKRIDDLIDAQIADARTAP 204

CYP139A1_2549401785_Mycob 135 AESLFGQRLAVHSDFLGEQLQPLLDLTRRPPQVMRLQQRVNSPGWRRAMAARKRIDDLIDAQIADARTAP 204

CYP139A1_2555434304_Mycob 135 AESLFGQRLAVHSDFLGEQLQPLLDLTRRPPQVMRLQQRVNSPGWRRAMAARKRIDDLIDAQIADARTAP 204

CYP139A1_2555528546_Mycob 135 AESLFGQRLAVHSDFLGEQLQPLLDLTRRPPQVMRLQQRVNSPGWRRAMAARKRIDDLIDAQIADARTAP 204

CYP139A1_2555595572_Mycob 135 AESLFGQRLAVHSDFLGEQLQPLLDLTRRPPQVMRLQQRVNSPGWRRAMAARKRIDDLIDAQIADARTAP 204

CYP139A1_2555144317_Mycob 135 AESLFGQRLAVHSDFLGEQLQPLLDLTRRPPQVMRLQQRVNSPGWRRAMAARKRIDDLIDAQIADARTAP 204

CYP139A1_2555290904_Mycob 135 AESLFGQRLAVHSDFLGEQLQPLLDLTRRPPQVMRLQQRVNSPGWRRAMAARKRIDDLIDAQIADARTAP 204

CYP139A1_2555316223_Mycob 135 AESLFGQRLAVHSDFLGEQLQPLLDLTRRPPQVMRLQQRVNSPGWRRAMAARKRIDDLIDAQIADARTAP 204

CYP139A1_2555388140_Mycob 135 AESLFGQRLAVHSDFLGEQLQPLLDLTRRPPQVMRLQQRVNSPGWRRAMAARKRIDDLIDAQIADARTAP 204

CYP139A1_2555350295_Mycob 135 AESLFGQRLAVHSDFLGEQLQPLLDLTRRPPQVMRLQQRVNSPGWRRAMAARKRIDDLIDAQIADARTAP 204

CYP139A1_2555960457_Mycob 135 AESLFGQRLAVHSDFLGEQLQPLLDLTRRPPQVMRLQQRVNSPGWRRAMAARKRIDDLIDAQIADARTAP 204

CYP139A1_2547951333_Mycob 135 AESLFGQRLAVHSDFLGEQLQPLLDLTRRPPQVMRLQQRVNSPGWRRAMAARKRIDDLIDAQIADARTAP 204

CYP139A1_2555451004_Mycob 135 AESLFGQRLAVHSDFLGEQLQPLLDLTRRPPQVMRLQQRVNSPGWRRAMAARKRIDDLIDAQIADARTAP 204

CYP139A1_2555140094_Mycob 135 AESLFGQRLAVHSDFLGEQLQPLLDLTRRPPQVMRLQQRVNSPGWRRAMAARKRIDDLIDAQIADARTAP 204

CYP139A1_2555358731_Mycob 135 AESLFGQRLAVHSDFLGEQLQPLLDLTRRPPQVMRLQQRVNSPGWRRAMAARKRIDDLIDAQIADARTAP 204

CYP139A1_2555375511_Mycob 135 AESLFGQRLAVHSDFLGEQLQPLLDLTRRPPQVMRLQQRVNSPGWRRAMAARKRIDDLIDAQIADARTAP 204

CYP139A1_2555578867_Mycob 135 AESLFGQRLAVHSDFLGEQLQPLLDLTRRPPQVMRLQQRVNSPGWRRAMAARKRIDDLIDAQIADARTAP 204

CYP139A1_2554700949_Mycob 135 AESLFGQRLAVHSDFLGEQLQPLLDLTRRPPQVMRLQQRVNSPGWRRAMAARKRIDDLIDAQIADARTAP 204

CYP139A1_2575403392_Mycob 135 AESLFGQRLAVHSDFLGEQLQPLLDLTRRPPQVMRLQQRVNSPGWRRAMAARKRIDDLIDAQIADARTAP 204

CYP139A1_638726892_Mycoba 135 AESLFGQRLAVHSDFLGEQLQPLLDLTRRPPQVMRLQQRVNSPGWRRAMAARKRIDDLIDAQIADARTAP 204

CYP139A1_648443266_Mycoba 135 AESLFGQRLAVHSDFLGEQLQPLLDLTRRPPQVMRLQQRVNSPGWRRAMAARKRIDDLIDAQIADARTAP 204

CYP139A1_2540554561_Mycob 129 AESLFGQRLAVHSDFLGEQLQPLLDLTRRPPQVMRLQQRVNSPGWRRAMAARKRIDDLIDAQIADARTAP 198

CYP139A1_2566980890_Mycob 135 AESLFGRRLAVHSDFLGEQLQPLLDLTRRPPQVMRLQQRVNSPGWRRAMTARKRIDDLIDAQIADARTAP 204

CYP139A1_2566976623_Mycob 135 AESLFGRRLAVHSDFLGEQLQPLLDLTRRPPQVMRLQQRVNSPGWRRAMTARKRIDDLIDAQIADARTAP 204

CYP139A1_2566972350_Mycob 135 AESLFGRRLAVHSDFLGEQLQPLLDLTRRPPQVMRLQQRVNSPGWRRAMTARKRIDDLIDAQIADARTAP 204

CYP139A1_2514118145_Mycob 130 AESLFGQRLAVHSDFLGEQLQPLLDLTRRPPQVMRLQQRVNSPGWRRAMAARKRIDDLIDAQIADARTAP 199

[Consensus_aa:](http://prodata.swmed.edu/promals3d/info/consensus.html) *h***ESLFG**.**R***h***A***h***HSD***@***LGEQLQPL***l***DLT+**p.**PQ***lh*.**LQ**p**R***l*pt**P**t**WRRAMAAR**p**R***l***D-L***l*s**A**b**IA**c**AR***hh***P**

[Consensus_ss:](http://prodata.swmed.edu/promals3d/info/consensus_ss.html) hhhh hhhhhhhhhhhhhh hhhhhhhhh hhhhhhhhhhhhhhhhhhhhhhhhh

Conservation: 666 696 96 96 69 9 969696 6 99669999699696699 6 996 99 9 66 69 96

CYP139A_2567124714_Mycoba 205 GPDDHMLTTLID-------TLSDNEIRDAIVSLITAGYETTSGALAWAAHALLTLPGAWETVAREVDRVL 267

CYP139A_650873455_Mycobac 199 SADDNVLATLIGGRTEVGESLRDDEIRDQIVSLIAAGYETTSAAMGWAVYALLSTPGVWQTAAAEVGDVT 268

CYP139A_2549393401_Mycoba 203 AADDNVLTTLINGRTDEGEALRDDEIRDQVVSLIAAGYETTSAAMGWAVYALLSTPGVWETAAAEVASVT 272

CYP139A_2548535921_Mycoba 202 NPNDHMLTMLIDGRGDEGYTLSDNEIRDAIVSLVTAGYETTSGALAWAVYLLLSQPGAWATAAGEVRRVL 271

CYP139A_2576976958_Mycoba 199 NPNDHMLTMLIDGRGDEGYTLSDNEIRDAIVSLVTAGYETTSGALAWAVYLLLSQPGAWATAAGEVRRVL 268

CYP139A_2581913245_Mycoba 199 NPDDHMLTMLIDGRGDEGYALSDNEIRDAIVSLVTAGYETTSGALAWAVYLLLSQPGAWAAAAGEVRRVL 268

CYP139A_2580974538_Mycoba 199 NPNDHMLTMLIDGRGDEGYTLSDNEIRDAIVSLVTAGYETTSGALAWAVYLLLSQPGAWATAAGEVRRVL 268

CYP139A_2582181025_Mycoba 199 NPDDHMLTMLIDGRGDEGYALSDNEIRDAIVSLVTAGYETTSGALAWAVYLLLSQPGAWAAAAGEVRRVL 268

CYP139A_2580742569_Mycoba 199 NPNDHMLTMLIDGRGDEGYTLSDNEIRDAIVSLVTAGYETTSGALAWAVYLLLSQPGAWATAAGEVRRVL 268

CYP139A_2582203743_Mycoba 199 NPNDHMLTMLIDGRGDEGYTLSDNEIRDAIVSLVTAGYETTSGALAWAVYLLLSQPGAWATAAGEVRRVL 268

CYP139A_2567079276_Mycoba 181 NPDDHMLTMLIDGRGDEGYALNDNEIRDAIVSLVTAGYETTSGALAWAVYLLLSQPGAWATAAGEVRRVL 250

CYP139A_2543326887_Mycoba 199 NPNDHMLTMLIDGRGDEGYTLSDNEIRDAIVSLVTAGYETTSGALAWAVYLLLSQPGAWATAAGEVRRVL 268

CYP139A_2570865822_Mycoba 199 NPNDHMLTMLIDGRGDEGYTLSDNEIRDAIVSLVTAGYETTSGALAWAVYLLLSQPGAWATAAGEVRRVL 268

CYP139A_2592485489_Mycoba 199 NPNDHMLTMLIDGRGDEGYTLSDNEIRDAIVSLVTAGYETTSGALAWAVYLLLSQPGAWATAAGEVRRVL 268

CYP139A_2550738610_Mycoba 202 NPNDHMLTMLIDGRGDEGYTLSDNEIRDAIVSLVTAGYETTSGALAWAVYLLLSQPGAWATAAGEVRRVL 271

CYP139A_2580783837__Mycob 199 NPDDHMLTMLIDGRGDEGYALNDNEIRDAIASLVTAGYETTSGALAWAVYLLLSQPGAWATAAGEVRRVL 268

CYP139A_2569618768_Mycoba 199 NPDGHMLTMLVDGRGDEGYTLSDNEIRDAIVSLVTAGYETTSGALAWAVYLLLSQPGAWAAAAGDVRRVL 268

CYP139A_2547368463_Mycoba 202 NPNDHMLTMLIDGRGDEGYTLSDNEIRDAIVSLVTAGYETTSGALAWAVYLLLSQPGAWATAAGEVRRVL 271

CYP139A_2572767979_Mycoba 199 NPNDHMLTMLINGRGDEGYALSDNEIRDAIVSLVTAGYETTSGALAWAVYLLLSQPGAWATAAGEVRRVL 268

CYP139A_2582391300_Mycoba 199 NPDDHMLTMLIDGRGDEGYALNDNEIRDAIVSLVTAGYETTSGALAWAVYLLLSQPGAWATAAGEVRRVL 268

CYP139A_2548515815_Mycoba 202 NPNDHMLTMLIDGRGDEGYTLSDNEIRDAIVSLVTAGYETTSGALAWAVYLLLSQPGAWATAAGEVRRVL 271

CYP139A_2548530385_Mycoba 202 NPNDHMLTMLIDGRGDEGYTLSDNEIRDAIVSLVTAGYETTSGALAWAVYLLLSQPGAWATAAGEVRRVL 271

CYP139A_2581397788_Mycoba 199 NPDDHMLTMLIDGRGDEGYALNDNEIRDAIVSLVTAGYETTSGALAWAVYLLLSQPGAWATAAGEVRRVL 268

CYP139A_2549377452_Mycoba 202 NPNDHMLTMLIDGRGDEGYTLSDNEIRDAIVSLVTAGYETTSGALAWAVYLLLSQPGAWATAAGEVRRVL 271

CYP139A_2549389164_Mycoba 202 NPDDHMLTMLIDGRGDEGYALNDNEIRDAIVSLVTAGYETTSGALAWAVYLLLSQPGAWATAAGEVRRVL 271

CYP139A_645425415_Mycobac 199 NPDGHMLTMLVDGRGDEGYTLSDNEIRDAIVSLVTAGYETTSGALAWAVYLLLSQPGAWAAAAGDVRRVL 268

CYP139A_2581110378_Mycoba 202 NPDDHMLTMLIDGRGDEGYALNDNEIRDAIVSLVTAGYETTSGALAWAVYLLLSQPGAWATAAGEVRRVL 271

CYP139A_2573433552_Mycoba 199 NPDDHMLTMLIDGRGDEGYALNDNEIRDAIVSLVTAGYETTSGALAWAVYLLLSQPGAWATAAGEVRRVL 268

CYP139A_2580006443__Mycob 199 NPDDHMLTMLIDGRGDEGYALNDNEIRDAIVSLVTAGYETTSGALAWAVYLLLSQPGAWATAAGEVRRVL 268

CYP139A_637134331_Mycobac 199 NPNDHMLTMLIDGRGDEGYTLSDNEIRDAIVSLVTAGYETTSGALAWAVYLLLSQPGAWATAAGEVRRVL 268

CYP139A_2548578292_Mycoba 202 NPNDHMLTMLIDGRGDEGYTLSDNEIRDAIVSLVTAGYETTSGALAWAVYLLLSQPGAWATAAGEVRRVL 271

CYP139A_2548547272_Mycoba 202 NPNDHMLTMLIDGRGDEGYTLSDNEIRDAIVSLVTAGYETTSGALAWAVYLLLSQPGAWATAAGEVRRVL 271

CYP139A_2549383420_Mycoba 202 NPNDHMLTMLIDGRGDEGYTLSDNEIRDAIVSLVTAGYETTSGALAWAVYLLLSQPGAWATAAGEVRRVL 271

CYP139A_639736419_Mycobac 199 NPDDHMLTMLIDGRGDEGYALNDNEIRDAIVSLVTAGYETTSGALAWAVYLLLSQPGAWATAAGEVRRVL 268

CYP139A_2555735619_Mycoba 199 NPNDHMLTMLIDGRGDEGYTLSDNEIRDAIVSLVTAGYETTSGALAWAVYLLLSQPGAWATAAGEVRRVL 268

CYP139A_2555481387_Mycoba 210 RPDDHMLTMLINGRGDEGYALSDDEIRDAVISLITAGYETTSGALAWAIYSLLSLPGAWDTAVDEVRRVL 279

CYP139A_2545768030_Mycoba 204 RPDDHMLTMLIDGRGDEGYALSDNEVRYSIISLITAGYEITSGALAWAIYTLLTVPGAWDRAADEVRRVL 273

CYP139A_2567131988_Mycoba 205 MPDDHMLTALID-------TLSDNEVRDAIVSLITAGYETTSGALAWAAHALLTLPGAWETVAREVDRVL 267

CYP139A_2587480388_Mycoba 205 MPDDHMLTALID-------TLSDNEVRDAIVSLITAGYETTSGALAWAAHALLTLPGAWETVAREVDRVL 267

CYP139A_2563577345_Mycoba 205 MPDDHMLTALID-------TLSDNEVRDAIVSLITAGYETTSGALAWAAHALLTLPGAWETVAREVDRVL 267

CYP139A_2543277028_Mycoba 205 SPDDRMLTALINGRSEEGCALSDNEIRDSIVSLIAAGYETTSGALAWATYTLLTLPGAWETAAREVARVL 274

CYP139A_2563569217_Mycoba 205 SPDDRMLTALINGRSEEGCALSDNEIRDSIVSLIAAGYETTSGALAWATYTLLTLPGAWETAAREVARVL 274

CYP139A_641717750_Mycobac 205 SPDDRMLTALINGRSEEGCALSDNEIRDSIVSLIAAGYETTSGALAWATYSLLTLPGAWETAAREVARVL 274

CYP139A_2588629254_Mycoba 205 SPDDRMLTALINGRSEEGCALSDNEIRDSIVSLIAAGYETTSGALAWATYTLLTLPGAWETAAREVARVL 274

CYP139A_2546369014_Mycoba 205 SPDDRMLTALINGRSEEGCVLSDNEIRDSIVSLIAAGYETTSGALAWATYTLLTLPGAWETAAREVARVL 274

CYP139A1_2555148489_Mycob 205 RPDDHMLTTLISGCSEEGTTLSDNEIRDSIVSLITAGYETTSGALAWAIYALLTVPGTWESAASEVARVL 274

CYP139A1_646010237_Mycoba 159 RPDDHMLTTLISGCSEEGTTLSDNEIRDSIVSLITAGYETTSGALAWAIYALLTVPGTWESAASEVARVL 228

CYP139A1_2581377024_Mycob 159 RPDDHMLTTLISGCSEEGTTLSDNEIRDSIVSLITAGYETTSGALAWAIYALLTVPGTWESAASEVARVL 228

CYP139A1_647209603_Mycoba 159 RPDDHMLTTLISGCSEEGTTLSDNEIRDSIVSLITAGYETTSGALAWAIYALLTVPGTWESAASEVARVL 228

CYP139A1_2537735281_Mycob 205 RPDDHMLTTLISGCSEEGTTLSDNEIRDSIVSLITAGYETTSGALAWAIYALLTVPGTWESAASEVARVL 274

CYP139A1_2576388909_Mycob 199 RPDDHMLTTLISGCSEEGTTLSDNEIRDSIVSLITAGYETTSGALAWAIYALLTVPGTWESAASEVARVL 268

CYP139A1_2577593438_Mycob 199 RPDDHMLTTLISGCSEEGTTLSDNEIRDSIVSLITAGYETTSGALAWAIYALLTVPGTWESAASEVARVL 268

CYP139A1_2577803488_Mycob 199 RPDDHMLTTLISGCSEEGTTLSDNEIRDSIVSLITAGYETTSGALAWAIYALLTVPGTWESAASEVARVL 268

CYP139A1_2581355094_Mycob 199 RPDDHMLTTLISGCSEEGTTLSDNEIRDSIVSLITAGYETTSGALAWAIYALLTVPGTWESAASEVARVL 268

CYP139A1_2584983051_Mycob 199 RPDDHMLTTLISGCSEEGTTLSDNEIRDSIVSLITAGYETTSGALAWAIYALLTVPGTWESAASEVARVL 268

CYP139A1_2584987406_Mycob 199 RPDDHMLTTLISGCSEEGTTLSDNEIRDSIVSLITAGYETTSGALAWAIYALLTVPGTWESAASEVARVL 268

CYP139A1_2589032800_Mycob 199 RPDDHMLTTLISGCSEEGTTLSDNEIRDSIVSLITAGYETTSGALAWAIYALLTVPGTWESAASEVARVL 268

CYP139A1_2592403099_Mycob 199 RPDDHMLTTLISGCSEEGTTLSDNEIRDSIVSLITAGYETTSGALAWAIYALLTVPGTWESAASEVARVL 268

CYP139A1_2592422247_Mycob 199 RPDDHMLTTLISGCSEEGTTLSDNEIRDSIVSLITAGYETTSGALAWAIYALLTVPGTWESAASEVARVL 268

CYP139A1_643734506_Mycoba 199 RPDDHMLTTLISGCSEEGTTLSDNEIRDSIVSLITAGYETTSGALAWAIYALLTVPGTWESAASEVARVL 268

CYP139A1_648335985_Mycoba 199 RPDDHMLTTLISGCSEEGTTLSDNEIRDSIVSLITAGYETTSGALAWAIYALLTVPGTWESAASEVARVL 268

CYP139A1_2574754194_Mycob 199 RPDDHMLTTLISGCSEEGTTLSDNEIRDSIVSLITAGYETTSGALAWAIYALLTVPGTWESAASEVARVL 268

CYP139A1_2575447433_Mycob 199 RPDDHMLTTLISGCSEEGTTLSDNEIRDSIVSLITAGYETTSGALAWAIYALLTVPGTWESAASEVARVL 268

CYP139A1_2575938969_Mycob 199 RPDDHMLTTLISGCSEEGTTLSDNEIRDSIVSLITAGYETTSGALAWAIYALLTVPGTWESAASEVARVL 268

CYP139A1_2576477081_Mycob 199 RPDDHMLTTLISGCSEEGTTLSDNEIRDSIVSLITAGYETTSGALAWAIYALLTVPGTWESAASEVARVL 268

CYP139A1_2576601719_Mycob 199 RPDDHMLTTLISGCSEEGTTLSDNEIRDSIVSLITAGYETTSGALAWAIYALLTVPGTWESAASEVARVL 268

CYP139A1_2577098384_Mycob 199 RPDDHMLTTLISGCSEEGTTLSDNEIRDSIVSLITAGYETTSGALAWAIYALLTVPGTWESAASEVARVL 268

CYP139A1_2578107196_Mycob 199 RPDDHMLTTLISGCSEEGTTLSDNEIRDSIVSLITAGYETTSGALAWAIYALLTVPGTWESAASEVARVL 268

CYP139A1_2584883084_Mycob 199 RPDDHMLTTLISGCSEEGTTLSDNEIRDSIVSLITAGYETTSGALAWAIYALLTVPGTWESAASEVARVL 268

CYP139A1_2588974834_Mycob 199 RPDDHMLTTLISGCSEEGTTLSDNEIRDSIVSLITAGYETTSGALAWAIYALLTVPGTWESAASEVARVL 268

CYP139A1_2589056454_Mycob 199 RPDDHMLTTLISGCSEEGTTLSDNEIRDSIVSLITAGYETTSGALAWAIYALLTVPGTWESAASEVARVL 268

CYP139A1_2589161189_Mycob 199 RPDDHMLTTLISGCSEEGTTLSDNEIRDSIVSLITAGYETTSGALAWAIYALLTVPGTWESAASEVARVL 268

CYP139A1_2590374347_Mycob 199 RPDDHMLTTLISGCSEEGTTLSDNEIRDSIVSLITAGYETTSGALAWAIYALLTVPGTWESAASEVARVL 268

CYP139A1_646018681_Mycoba 199 RPDDHMLTTLISGCSEEGTTLSDNEIRDSIVSLITAGYETTSGALAWAIYALLTVPGTWESAASEVARVL 268

CYP139A1_2575060404_Mycob 199 RPDDHMLTTLISGCSEEGTTLSDNEIRDSIVSLITAGYETTSGALAWAIYALLTVPGTWESAASEVARVL 268

CYP139A1_2576105631_Mycob 199 RPDDHMLTTLISGCSEEGTTLSDNEIRDSIVSLITAGYETTSGALAWAIYALLTVPGTWESAASEVARVL 268

CYP139A1_2576247251_Mycob 199 RPDDHMLTTLISGCSEEGTTLSDNEIRDSIVSLITAGYETTSGALAWAIYALLTVPGTWESAASEVARVL 268

CYP139A1_2576981010_Mycob 199 RPDDHMLTTLISGCSEEGTTLSDNEIRDSIVSLITAGYETTSGALAWAIYALLTVPGTWESAASEVARVL 268

CYP139A1_2577093117_Mycob 199 RPDDHMLTTLISGCSEEGTTLSDNEIRDSIVSLITAGYETTSGALAWAIYALLTVPGTWESAASEVARVL 268

CYP139A1_2577198903_Mycob 199 RPDDHMLTTLISGCSEEGTTLSDNEIRDSIVSLITAGYETTSGALAWAIYALLTVPGTWESAASEVARVL 268

CYP139A1_2577516047_Mycob 199 RPDDHMLTTLISGCSEEGTTLSDNEIRDSIVSLITAGYETTSGALAWAIYALLTVPGTWESAASEVARVL 268

CYP139A1_2578213104_Mycob 199 RPDDHMLTTLISGCSEEGTTLSDNEIRDSIVSLITAGYETTSGALAWAIYALLTVPGTWESAASEVARVL 268

CYP139A1_2584711251_Mycob 199 RPDDHMLTTLISGCSEEGTTLSDNEIRDSIVSLITAGYETTSGALAWAIYALLTVPGTWESAASEVARVL 268

CYP139A1_2584816678_Mycob 199 RPDDHMLTTLISGCSEEGTTLSDNEIRDSIVSLITAGYETTSGALAWAIYALLTVPGTWESAASEVARVL 268

CYP139A1_2589068752_Mycob 199 RPDDHMLTTLISGCSEEGTTLSDNEIRDSIVSLITAGYETTSGALAWAIYALLTVPGTWESAASEVARVL 268

CYP139A1_2589604293_Mycob 199 RPDDHMLTTLISGCSEEGTTLSDNEIRDSIVSLITAGYETTSGALAWAIYALLTVPGTWESAASEVARVL 268

CYP139A1_643028176_Mycoba 199 RPDDHMLTTLISGCSEEGTTLSDNEIRDSIVSLITAGYETTSGALAWAIYALLTVPGTWESAASEVARVL 268

CYP139A1_648476944_Mycoba 199 RPDDHMLTTLISGCSEEGTTLSDNEIRDSIVSLITAGYETTSGALAWAIYALLTVPGTWESAASEVARVL 268

CYP139A1_2511736071_Mycob 199 RPDDHMLTTLISGCSEEGTTLSDNEIRDSIVSLITAGYETTSGALAWAIYALLTVPGTWESAASEVARVL 268

CYP139A1_2546206123_Mycob 199 RPDDHMLTTLISGCSEEGTTLSDNEIRDSIVSLITAGYETTSGALAWAIYALLTVPGTWESAASEVARVL 268

CYP139A1_2574780327_Mycob 199 RPDDHMLTTLISGCSEEGTTLSDNEIRDSIVSLITAGYETTSGALAWAIYALLTVPGTWESAASEVARVL 268

CYP139A1_2575978404_Mycob 199 RPDDHMLTTLISGCSEEGTTLSDNEIRDSIVSLITAGYETTSGALAWAIYALLTVPGTWESAASEVARVL 268

CYP139A1_2576675825_Mycob 199 RPDDHMLTTLISGCSEEGTTLSDNEIRDSIVSLITAGYETTSGALAWAIYALLTVPGTWESAASEVARVL 268

CYP139A1_2576947708_Mycob 199 RPDDHMLTTLISGCSEEGTTLSDNEIRDSIVSLITAGYETTSGALAWAIYALLTVPGTWESAASEVARVL 268

CYP139A1_2577400922_Mycob 199 RPDDHMLTTLISGCSEEGTTLSDNEIRDSIVSLITAGYETTSGALAWAIYALLTVPGTWESAASEVARVL 268

CYP139A1_2577893113_Mycob 199 RPDDHMLTTLISGCSEEGTTLSDNEIRDSIVSLITAGYETTSGALAWAIYALLTVPGTWESAASEVARVL 268

CYP139A1_2584759228_Mycob 199 RPDDHMLTTLISGCSEEGTTLSDNEIRDSIVSLITAGYETTSGALAWAIYALLTVPGTWESAASEVARVL 268

CYP139A1_2584801008_Mycob 199 RPDDHMLTTLISGCSEEGTTLSDNEIRDSIVSLITAGYETTSGALAWAIYALLTVPGTWESAASEVARVL 268

CYP139A1_2584946269_Mycob 199 RPDDHMLTTLISGCSEEGTTLSDNEIRDSIVSLITAGYETTSGALAWAIYALLTVPGTWESAASEVARVL 268

CYP139A1_2589125802_Mycob 199 RPDDHMLTTLISGCSEEGTTLSDNEIRDSIVSLITAGYETTSGALAWAIYALLTVPGTWESAASEVARVL 268

CYP139A1_2589654545_Mycob 199 RPDDHMLTTLISGCSEEGTTLSDNEIRDSIVSLITAGYETTSGALAWAIYALLTVPGTWESAASEVARVL 268

CYP139A1_2590190898_Mycob 199 RPDDHMLTTLISGCSEEGTTLSDNEIRDSIVSLITAGYETTSGALAWAIYALLTVPGTWESAASEVARVL 268

CYP139A1_2592267285_Mycob 199 RPDDHMLTTLISGCSEEGTTLSDNEIRDSIVSLITAGYETTSGALAWAIYALLTVPGTWESAASEVARVL 268

CYP139A1_2592579018_Mycob 199 RPDDHMLTTLISGCSEEGTTLSDNEIRDSIVSLITAGYETTSGALAWAIYALLTVPGTWESAASEVARVL 268

CYP139A1_2574803240_Mycob 199 RPDDHMLTTLISGCSEEGTTLSDNEIRDSIVSLITAGYETTSGALAWAIYALLTVPGTWESAASEVARVL 268

CYP139A1_2575138339_Mycob 199 RPDDHMLTTLISGCSEEGTTLSDNEIRDSIVSLITAGYETTSGALAWAIYALLTVPGTWESAASEVARVL 268

CYP139A1_2575935659_Mycob 199 RPDDHMLTTLISGCSEEGTTLSDNEIRDSIVSLITAGYETTSGALAWAIYALLTVPGTWESAASEVARVL 268

CYP139A1_2576703024_Mycob 199 RPDDHMLTTLISGCSEEGTTLSDNEIRDSIVSLITAGYETTSGALAWAIYALLTVPGTWESAASEVARVL 268

CYP139A1_2577143911_Mycob 199 RPDDHMLTTLISGCSEEGTTLSDNEIRDSIVSLITAGYETTSGALAWAIYALLTVPGTWESAASEVARVL 268

CYP139A1_2577175183_Mycob 199 RPDDHMLTTLISGCSEEGTTLSDNEIRDSIVSLITAGYETTSGALAWAIYALLTVPGTWESAASEVARVL 268

CYP139A1_2577879644_Mycob 199 RPDDHMLTTLISGCSEEGTTLSDNEIRDSIVSLITAGYETTSGALAWAIYALLTVPGTWESAASEVARVL 268

CYP139A1_2577954418_Mycob 199 RPDDHMLTTLISGCSEEGTTLSDNEIRDSIVSLITAGYETTSGALAWAIYALLTVPGTWESAASEVARVL 268

CYP139A1_2584625495_Mycob 199 RPDDHMLTTLISGCSEEGTTLSDNEIRDSIVSLITAGYETTSGALAWAIYALLTVPGTWESAASEVARVL 268

CYP139A1_2589526877_Mycob 199 RPDDHMLTTLISGCSEEGTTLSDNEIRDSIVSLITAGYETTSGALAWAIYALLTVPGTWESAASEVARVL 268

CYP139A1_2589711853_Mycob 199 RPDDHMLTTLISGCSEEGTTLSDNEIRDSIVSLITAGYETTSGALAWAIYALLTVPGTWESAASEVARVL 268

CYP139A1_2590113796_Mycob 199 RPDDHMLTTLISGCSEEGTTLSDNEIRDSIVSLITAGYETTSGALAWAIYALLTVPGTWESAASEVARVL 268

CYP139A1_2592324422_Mycob 199 RPDDHMLTTLISGCSEEGTTLSDNEIRDSIVSLITAGYETTSGALAWAIYALLTVPGTWESAASEVARVL 268

CYP139A1_2592337997_Mycob 199 RPDDHMLTTLISGCSEEGTTLSDNEIRDSIVSLITAGYETTSGALAWAIYALLTVPGTWESAASEVARVL 268

CYP139A1_2592373455_Mycob 199 RPDDHMLTTLISGCSEEGTTLSDNEIRDSIVSLITAGYETTSGALAWAIYALLTVPGTWESAASEVARVL 268

CYP139A1_2592445915_Mycob 199 RPDDHMLTTLISGCSEEGTTLSDNEIRDSIVSLITAGYETTSGALAWAIYALLTVPGTWESAASEVARVL 268

CYP139A1_2575023271_Mycob 199 RPDDHMLTTLISGCSEEGTTLSDNEIRDSIVSLITAGYETTSGALAWAIYALLTVPGTWESAASEVARVL 268

CYP139A1_2575619239_Mycob 199 RPDDHMLTTLISGCSEEGTTLSDNEIRDSIVSLITAGYETTSGALAWAIYALLTVPGTWESAASEVARVL 268

CYP139A1_2575786887_Mycob 199 RPDDHMLTTLISGCSEEGTTLSDNEIRDSIVSLITAGYETTSGALAWAIYALLTVPGTWESAASEVARVL 268

CYP139A1_2576882264_Mycob 199 RPDDHMLTTLISGCSEEGTTLSDNEIRDSIVSLITAGYETTSGALAWAIYALLTVPGTWESAASEVARVL 268

CYP139A1_2577215885_Mycob 199 RPDDHMLTTLISGCSEEGTTLSDNEIRDSIVSLITAGYETTSGALAWAIYALLTVPGTWESAASEVARVL 268

CYP139A1_2577627248_Mycob 199 RPDDHMLTTLISGCSEEGTTLSDNEIRDSIVSLITAGYETTSGALAWAIYALLTVPGTWESAASEVARVL 268

CYP139A1_2577923998_Mycob 199 RPDDHMLTTLISGCSEEGTTLSDNEIRDSIVSLITAGYETTSGALAWAIYALLTVPGTWESAASEVARVL 268

CYP139A1_2583735989_Mycob 199 RPDDHMLTTLISGCSEEGTTLSDNEIRDSIVSLITAGYETTSGALAWAIYALLTVPGTWESAASEVARVL 268

CYP139A1_2584003651_Mycob 199 RPDDHMLTTLISGCSEEGTTLSDNEIRDSIVSLITAGYETTSGALAWAIYALLTVPGTWESAASEVARVL 268

CYP139A1_2584623655_Mycob 199 RPDDHMLTTLISGCSEEGTTLSDNEIRDSIVSLITAGYETTSGALAWAIYALLTVPGTWESAASEVARVL 268

CYP139A1_2584739857_Mycob 199 RPDDHMLTTLISGCSEEGTTLSDNEIRDSIVSLITAGYETTSGALAWAIYALLTVPGTWESAASEVARVL 268

CYP139A1_2584858071_Mycob 199 RPDDHMLTTLISGCSEEGTTLSDNEIRDSIVSLITAGYETTSGALAWAIYALLTVPGTWESAASEVARVL 268

CYP139A1_2584928246_Mycob 199 RPDDHMLTTLISGCSEEGTTLSDNEIRDSIVSLITAGYETTSGALAWAIYALLTVPGTWESAASEVARVL 268

CYP139A1_2589040293_Mycob 199 RPDDHMLTTLISGCSEEGTTLSDNEIRDSIVSLITAGYETTSGALAWAIYALLTVPGTWESAASEVARVL 268

CYP139A1_2589658610_Mycob 199 RPDDHMLTTLISGCSEEGTTLSDNEIRDSIVSLITAGYETTSGALAWAIYALLTVPGTWESAASEVARVL 268

CYP139A1_2590162679_Mycob 199 RPDDHMLTTLISGCSEEGTTLSDNEIRDSIVSLITAGYETTSGALAWAIYALLTVPGTWESAASEVARVL 268

CYP139A1_2590243884_Mycob 199 RPDDHMLTTLISGCSEEGTTLSDNEIRDSIVSLITAGYETTSGALAWAIYALLTVPGTWESAASEVARVL 268

CYP139A1_2590531681_Mycob 199 RPDDHMLTTLISGCSEEGTTLSDNEIRDSIVSLITAGYETTSGALAWAIYALLTVPGTWESAASEVARVL 268

CYP139A1_2574614619_Mycob 199 RPDDHMLTTLISGCSEEGTTLSDNEIRDSIVSLITAGYETTSGALAWAIYALLTVPGTWESAASEVARVL 268

CYP139A1_2575016195_Mycob 199 RPDDHMLTTLISGCSEEGTTLSDNEIRDSIVSLITAGYETTSGALAWAIYALLTVPGTWESAASEVARVL 268

CYP139A1_2575426848_Mycob 199 RPDDHMLTTLISGCSEEGTTLSDNEIRDSIVSLITAGYETTSGALAWAIYALLTVPGTWESAASEVARVL 268

CYP139A1_2576630848_Mycob 199 RPDDHMLTTLISGCSEEGTTLSDNEIRDSIVSLITAGYETTSGALAWAIYALLTVPGTWESAASEVARVL 268

CYP139A1_2577468911_Mycob 199 RPDDHMLTTLISGCSEEGTTLSDNEIRDSIVSLITAGYETTSGALAWAIYALLTVPGTWESAASEVARVL 268

CYP139A1_2577974906_Mycob 199 RPDDHMLTTLISGCSEEGTTLSDNEIRDSIVSLITAGYETTSGALAWAIYALLTVPGTWESAASEVARVL 268

CYP139A1_2578062326_Mycob 199 RPDDHMLTTLISGCSEEGTTLSDNEIRDSIVSLITAGYETTSGALAWAIYALLTVPGTWESAASEVARVL 268

CYP139A1_2584836956_Mycob 199 RPDDHMLTTLISGCSEEGTTLSDNEIRDSIVSLITAGYETTSGALAWAIYALLTVPGTWESAASEVARVL 268

CYP139A1_2584998642_Mycob 199 RPDDHMLTTLISGCSEEGTTLSDNEIRDSIVSLITAGYETTSGALAWAIYALLTVPGTWESAASEVARVL 268

CYP139A1_2589130082_Mycob 199 RPDDHMLTTLISGCSEEGTTLSDNEIRDSIVSLITAGYETTSGALAWAIYALLTVPGTWESAASEVARVL 268

CYP139A1_2589592089_Mycob 199 RPDDHMLTTLISGCSEEGTTLSDNEIRDSIVSLITAGYETTSGALAWAIYALLTVPGTWESAASEVARVL 268

CYP139A1_2590052832_Mycob 199 RPDDHMLTTLISGCSEEGTTLSDNEIRDSIVSLITAGYETTSGALAWAIYALLTVPGTWESAASEVARVL 268

CYP139A1_2590505688_Mycob 199 RPDDHMLTTLISGCSEEGTTLSDNEIRDSIVSLITAGYETTSGALAWAIYALLTVPGTWESAASEVARVL 268

CYP139A1_2592353030_Mycob 199 RPDDHMLTTLISGCSEEGTTLSDNEIRDSIVSLITAGYETTSGALAWAIYALLTVPGTWESAASEVARVL 268

CYP139A1_637026884_Mycoba 199 RPDDHMLTTLISGCSEEGTTLSDNEIRDSIVSLITAGYETTSGALAWAIYALLTVPGTWESAASEVARVL 268

CYP139A1_2574886309_Mycob 199 RPDDHMLTTLISGCSEEGTTLSDNEIRDSIVSLITAGYETTSGALAWAIYALLTVPGTWESAASEVARVL 268

CYP139A1_2575295342_Mycob 199 RPDDHMLTTLISGCSEEGTTLSDNEIRDSIVSLITAGYETTSGALAWAIYALLTVPGTWESAASEVARVL 268

CYP139A1_2575942274_Mycob 199 RPDDHMLTTLISGCSEEGTTLSDNEIRDSIVSLITAGYETTSGALAWAIYALLTVPGTWESAASEVARVL 268

CYP139A1_2576123248_Mycob 199 RPDDHMLTTLISGCSEEGTTLSDNEIRDSIVSLITAGYETTSGALAWAIYALLTVPGTWESAASEVARVL 268

CYP139A1_2576712596_Mycob 199 RPDDHMLTTLISGCSEEGTTLSDNEIRDSIVSLITAGYETTSGALAWAIYALLTVPGTWESAASEVARVL 268

CYP139A1_2577856904_Mycob 199 RPDDHMLTTLISGCSEEGTTLSDNEIRDSIVSLITAGYETTSGALAWAIYALLTVPGTWESAASEVARVL 268

CYP139A1_2578013153_Mycob 199 RPDDHMLTTLISGCSEEGTTLSDNEIRDSIVSLITAGYETTSGALAWAIYALLTVPGTWESAASEVARVL 268

CYP139A1_2578182623_Mycob 199 RPDDHMLTTLISGCSEEGTTLSDNEIRDSIVSLITAGYETTSGALAWAIYALLTVPGTWESAASEVARVL 268

CYP139A1_2580771058_Mycob 199 RPDDHMLTTLISGCSEEGTTLSDNEIRDSIVSLITAGYETTSGALAWAIYALLTVPGTWESAASEVARVL 268

CYP139A1_2584641128_Mycob 199 RPDDHMLTTLISGCSEEGTTLSDNEIRDSIVSLITAGYETTSGALAWAIYALLTVPGTWESAASEVARVL 268

CYP139A1_2584649146_Mycob 199 RPDDHMLTTLISGCSEEGTTLSDNEIRDSIVSLITAGYETTSGALAWAIYALLTVPGTWESAASEVARVL 268

CYP139A1_2584660713_Mycob 199 RPDDHMLTTLISGCSEEGTTLSDNEIRDSIVSLITAGYETTSGALAWAIYALLTVPGTWESAASEVARVL 268

CYP139A1_2584967006_Mycob 199 RPDDHMLTTLISGCSEEGTTLSDNEIRDSIVSLITAGYETTSGALAWAIYALLTVPGTWESAASEVARVL 268

CYP139A1_2584970624_Mycob 199 RPDDHMLTTLISGCSEEGTTLSDNEIRDSIVSLITAGYETTSGALAWAIYALLTVPGTWESAASEVARVL 268

CYP139A1_2589053585_Mycob 199 RPDDHMLTTLISGCSEEGTTLSDNEIRDSIVSLITAGYETTSGALAWAIYALLTVPGTWESAASEVARVL 268

CYP139A1_2589498327_Mycob 199 RPDDHMLTTLISGCSEEGTTLSDNEIRDSIVSLITAGYETTSGALAWAIYALLTVPGTWESAASEVARVL 268

CYP139A1_2589563529_Mycob 199 RPDDHMLTTLISGCSEEGTTLSDNEIRDSIVSLITAGYETTSGALAWAIYALLTVPGTWESAASEVARVL 268

CYP139A1_2590377218_Mycob 199 RPDDHMLTTLISGCSEEGTTLSDNEIRDSIVSLITAGYETTSGALAWAIYALLTVPGTWESAASEVARVL 268

CYP139A1_2592283614_Mycob 199 RPDDHMLTTLISGCSEEGTTLSDNEIRDSIVSLITAGYETTSGALAWAIYALLTVPGTWESAASEVARVL 268

CYP139A1_2592319784_Mycob 199 RPDDHMLTTLISGCSEEGTTLSDNEIRDSIVSLITAGYETTSGALAWAIYALLTVPGTWESAASEVARVL 268

CYP139A1_2575106637_Mycob 199 RPDDHMLTTLISGCSEEGTTLSDNEIRDSIVSLITAGYETTSGALAWAIYALLTVPGTWESAASEVARVL 268

CYP139A1_2575157076_Mycob 199 RPDDHMLTTLISGCSEEGTTLSDNEIRDSIVSLITAGYETTSGALAWAIYALLTVPGTWESAASEVARVL 268

CYP139A1_2575361778_Mycob 199 RPDDHMLTTLISGCSEEGTTLSDNEIRDSIVSLITAGYETTSGALAWAIYALLTVPGTWESAASEVARVL 268

CYP139A1_2576009184_Mycob 199 RPDDHMLTTLISGCSEEGTTLSDNEIRDSIVSLITAGYETTSGALAWAIYALLTVPGTWESAASEVARVL 268

CYP139A1_2576566954_Mycob 199 RPDDHMLTTLISGCSEEGTTLSDNEIRDSIVSLITAGYETTSGALAWAIYALLTVPGTWESAASEVARVL 268

CYP139A1_2576731741_Mycob 199 RPDDHMLTTLISGCSEEGTTLSDNEIRDSIVSLITAGYETTSGALAWAIYALLTVPGTWESAASEVARVL 268

CYP139A1_2581562358_Mycob 199 RPDDHMLTTLISGCSEEGTTLSDNEIRDSIVSLITAGYETTSGALAWAIYALLTVPGTWESAASEVARVL 268

CYP139A1_2584107430_Mycob 199 RPDDHMLTTLISGCSEEGTTLSDNEIRDSIVSLITAGYETTSGALAWAIYALLTVPGTWESAASEVARVL 268

CYP139A1_2590025444_Mycob 199 RPDDHMLTTLISGCSEEGTTLSDNEIRDSIVSLITAGYETTSGALAWAIYALLTVPGTWESAASEVARVL 268

CYP139A1_2590040558_Mycob 199 RPDDHMLTTLISGCSEEGTTLSDNEIRDSIVSLITAGYETTSGALAWAIYALLTVPGTWESAASEVARVL 268

CYP139A1_2590214646_Mycob 199 RPDDHMLTTLISGCSEEGTTLSDNEIRDSIVSLITAGYETTSGALAWAIYALLTVPGTWESAASEVARVL 268

CYP139A1_2590223019_Mycob 199 RPDDHMLTTLISGCSEEGTTLSDNEIRDSIVSLITAGYETTSGALAWAIYALLTVPGTWESAASEVARVL 268

CYP139A1_2590266966_Mycob 199 RPDDHMLTTLISGCSEEGTTLSDNEIRDSIVSLITAGYETTSGALAWAIYALLTVPGTWESAASEVARVL 268

CYP139A1_647086307_Mycoba 199 RPDDHMLTTLISGCSEEGTTLSDNEIRDSIVSLITAGYETTSGALAWAIYALLTVPGTWESAASEVARVL 268

CYP139A1_2574726119_Mycob 199 RPDDHMLTTLISGCSEEGTTLSDNEIRDSIVSLITAGYETTSGALAWAIYALLTVPGTWESAASEVARVL 268

CYP139A1_2574757270_Mycob 199 RPDDHMLTTLISGCSEEGTTLSDNEIRDSIVSLITAGYETTSGALAWAIYALLTVPGTWESAASEVARVL 268

CYP139A1_2575280304_Mycob 199 RPDDHMLTTLISGCSEEGTTLSDNEIRDSIVSLITAGYETTSGALAWAIYALLTVPGTWESAASEVARVL 268

CYP139A1_2575601683_Mycob 199 RPDDHMLTTLISGCSEEGTTLSDNEIRDSIVSLITAGYETTSGALAWAIYALLTVPGTWESAASEVARVL 268

CYP139A1_2576158036_Mycob 199 RPDDHMLTTLISGCSEEGTTLSDNEIRDSIVSLITAGYETTSGALAWAIYALLTVPGTWESAASEVARVL 268

CYP139A1_2577689111_Mycob 199 RPDDHMLTTLISGCSEEGTTLSDNEIRDSIVSLITAGYETTSGALAWAIYALLTVPGTWESAASEVARVL 268

CYP139A1_2577751179_Mycob 199 RPDDHMLTTLISGCSEEGTTLSDNEIRDSIVSLITAGYETTSGALAWAIYALLTVPGTWESAASEVARVL 268

CYP139A1_2577845812_Mycob 199 RPDDHMLTTLISGCSEEGTTLSDNEIRDSIVSLITAGYETTSGALAWAIYALLTVPGTWESAASEVARVL 268

CYP139A1_2577900964_Mycob 199 RPDDHMLTTLISGCSEEGTTLSDNEIRDSIVSLITAGYETTSGALAWAIYALLTVPGTWESAASEVARVL 268

CYP139A1_2577988240_Mycob 199 RPDDHMLTTLISGCSEEGTTLSDNEIRDSIVSLITAGYETTSGALAWAIYALLTVPGTWESAASEVARVL 268

CYP139A1_2578237814_Mycob 199 RPDDHMLTTLISGCSEEGTTLSDNEIRDSIVSLITAGYETTSGALAWAIYALLTVPGTWESAASEVARVL 268

CYP139A1_2584703088_Mycob 199 RPDDHMLTTLISGCSEEGTTLSDNEIRDSIVSLITAGYETTSGALAWAIYALLTVPGTWESAASEVARVL 268

CYP139A1_2584776403_Mycob 199 RPDDHMLTTLISGCSEEGTTLSDNEIRDSIVSLITAGYETTSGALAWAIYALLTVPGTWESAASEVARVL 268

CYP139A1_2584785697_Mycob 199 RPDDHMLTTLISGCSEEGTTLSDNEIRDSIVSLITAGYETTSGALAWAIYALLTVPGTWESAASEVARVL 268

CYP139A1_2584898862_Mycob 199 RPDDHMLTTLISGCSEEGTTLSDNEIRDSIVSLITAGYETTSGALAWAIYALLTVPGTWESAASEVARVL 268

CYP139A1_2584906709_Mycob 199 RPDDHMLTTLISGCSEEGTTLSDNEIRDSIVSLITAGYETTSGALAWAIYALLTVPGTWESAASEVARVL 268

CYP139A1_2589082097_Mycob 199 RPDDHMLTTLISGCSEEGTTLSDNEIRDSIVSLITAGYETTSGALAWAIYALLTVPGTWESAASEVARVL 268

CYP139A1_2589142105_Mycob 199 RPDDHMLTTLISGCSEEGTTLSDNEIRDSIVSLITAGYETTSGALAWAIYALLTVPGTWESAASEVARVL 268

CYP139A1_2589707360_Mycob 199 RPDDHMLTTLISGCSEEGTTLSDNEIRDSIVSLITAGYETTSGALAWAIYALLTVPGTWESAASEVARVL 268

CYP139A1_2590142346_Mycob 199 RPDDHMLTTLISGCSEEGTTLSDNEIRDSIVSLITAGYETTSGALAWAIYALLTVPGTWESAASEVARVL 268

CYP139A1_2592254166_Mycob 199 RPDDHMLTTLISGCSEEGTTLSDNEIRDSIVSLITAGYETTSGALAWAIYALLTVPGTWESAASEVARVL 268

CYP139A1_2592348241_Mycob 199 RPDDHMLTTLISGCSEEGTTLSDNEIRDSIVSLITAGYETTSGALAWAIYALLTVPGTWESAASEVARVL 268

CYP139A1_2592364277_Mycob 199 RPDDHMLTTLISGCSEEGTTLSDNEIRDSIVSLITAGYETTSGALAWAIYALLTVPGTWESAASEVARVL 268

CYP139A1_2592537861_Mycob 199 RPDDHMLTTLISGCSEEGTTLSDNEIRDSIVSLITAGYETTSGALAWAIYALLTVPGTWESAASEVARVL 268

CYP139A1_2546454904_Mycob 199 RPDDHMLTTLISGCSEEGTTLSDNEIRDSIVSLITAGYETTSGALAWAIYALLTVPGTWESAASEVARVL 268

CYP139A1_2577218717_Mycob 199 RPDDHMLTTLISGCSEEGTTLSDNEIRDSIVSLITAGYETTSGALAWAIYALLTVPGTWESAASEVARVL 268

CYP139A1_2577720788_Mycob 199 RPDDHMLTTLISGCSEEGTTLSDNEIRDSIVSLITAGYETTSGALAWAIYALLTVPGTWESAASEVARVL 268

CYP139A1_2581366557_Mycob 199 RPDDHMLTTLISGCSEEGTTLSDNEIRDSIVSLITAGYETTSGALAWAIYALLTVPGTWESAASEVARVL 268

CYP139A1_2584638962_Mycob 199 RPDDHMLTTLISGCSEEGTTLSDNEIRDSIVSLITAGYETTSGALAWAIYALLTVPGTWESAASEVARVL 268

CYP139A1_2590154524_Mycob 199 RPDDHMLTTLISGCSEEGTTLSDNEIRDSIVSLITAGYETTSGALAWAIYALLTVPGTWESAASEVARVL 268

CYP139A1_2590260165_Mycob 199 RPDDHMLTTLISGCSEEGTTLSDNEIRDSIVSLITAGYETTSGALAWAIYALLTVPGTWESAASEVARVL 268

CYP139A1_2590539845_Mycob 199 RPDDHMLTTLISGCSEEGTTLSDNEIRDSIVSLITAGYETTSGALAWAIYALLTVPGTWESAASEVARVL 268

CYP139A1_641783198_Mycoba 199 RPDDHMLTTLISGCSEEGTTLSDNEIRDSIVSLITAGYETTSGALAWAIYALLTVPGTWESAASEVARVL 268

CYP139A1_643031783_Mycoba 199 RPDDHMLTTLISGCSEEGTTLSDNEIRDSIVSLITAGYETTSGALAWAIYALLTVPGTWESAASEVARVL 268

CYP139A1_651039004_Mycoba 199 RPDDHMLTTLISGCSEEGTTLSDNEIRDSIVSLITAGYETTSGALAWAIYALLTVPGTWESAASEVARVL 268

CYP139A1_2574843285_Mycob 199 RPDDHMLTTLISGCSEEGTTLSDNEIRDSIVSLITAGYETTSGALAWAIYALLTVPGTWESAASEVARVL 268

CYP139A1_2575542837_Mycob 199 RPDDHMLTTLISGCSEEGTTLSDNEIRDSIVSLITAGYETTSGALAWAIYALLTVPGTWESAASEVARVL 268

CYP139A1_2575709449_Mycob 199 RPDDHMLTTLISGCSEEGTTLSDNEIRDSIVSLITAGYETTSGALAWAIYALLTVPGTWESAASEVARVL 268

CYP139A1_2576051820_Mycob 199 RPDDHMLTTLISGCSEEGTTLSDNEIRDSIVSLITAGYETTSGALAWAIYALLTVPGTWESAASEVARVL 268

CYP139A1_2578094092_Mycob 199 RPDDHMLTTLISGCSEEGTTLSDNEIRDSIVSLITAGYETTSGALAWAIYALLTVPGTWESAASEVARVL 268

CYP139A1_2584617586_Mycob 199 RPDDHMLTTLISGCSEEGTTLSDNEIRDSIVSLITAGYETTSGALAWAIYALLTVPGTWESAASEVARVL 268

CYP139A1_2584694835_Mycob 199 RPDDHMLTTLISGCSEEGTTLSDNEIRDSIVSLITAGYETTSGALAWAIYALLTVPGTWESAASEVARVL 268

CYP139A1_2584822720_Mycob 199 RPDDHMLTTLISGCSEEGTTLSDNEIRDSIVSLITAGYETTSGALAWAIYALLTVPGTWESAASEVARVL 268

CYP139A1_2584931720_Mycob 199 RPDDHMLTTLISGCSEEGTTLSDNEIRDSIVSLITAGYETTSGALAWAIYALLTVPGTWESAASEVARVL 268

CYP139A1_2589165303_Mycob 199 RPDDHMLTTLISGCSEEGTTLSDNEIRDSIVSLITAGYETTSGALAWAIYALLTVPGTWESAASEVARVL 268

CYP139A1_2589608377_Mycob 199 RPDDHMLTTLISGCSEEGTTLSDNEIRDSIVSLITAGYETTSGALAWAIYALLTVPGTWESAASEVARVL 268

CYP139A1_2589638476_Mycob 199 RPDDHMLTTLISGCSEEGTTLSDNEIRDSIVSLITAGYETTSGALAWAIYALLTVPGTWESAASEVARVL 268

CYP139A1_2589687166_Mycob 199 RPDDHMLTTLISGCSEEGTTLSDNEIRDSIVSLITAGYETTSGALAWAIYALLTVPGTWESAASEVARVL 268

CYP139A1_2590174897_Mycob 199 RPDDHMLTTLISGCSEEGTTLSDNEIRDSIVSLITAGYETTSGALAWAIYALLTVPGTWESAASEVARVL 268

CYP139A1_2590370532_Mycob 199 RPDDHMLTTLISGCSEEGTTLSDNEIRDSIVSLITAGYETTSGALAWAIYALLTVPGTWESAASEVARVL 268

CYP139A1_2592328029_Mycob 199 RPDDHMLTTLISGCSEEGTTLSDNEIRDSIVSLITAGYETTSGALAWAIYALLTVPGTWESAASEVARVL 268

CYP139A1_2592426321_Mycob 199 RPDDHMLTTLISGCSEEGTTLSDNEIRDSIVSLITAGYETTSGALAWAIYALLTVPGTWESAASEVARVL 268

CYP139A1_2575094331_Mycob 199 RPDDHMLTTLISGCSEEGTTLSDNEIRDSIVSLITAGYETTSGALAWAIYALLTVPGTWESAASEVARVL 268

CYP139A1_2576101264_Mycob 199 RPDDHMLTTLISGCSEEGTTLSDNEIRDSIVSLITAGYETTSGALAWAIYALLTVPGTWESAASEVARVL 268

CYP139A1_2576927477_Mycob 199 RPDDHMLTTLISGCSEEGTTLSDNEIRDSIVSLITAGYETTSGALAWAIYALLTVPGTWESAASEVARVL 268

CYP139A1_2577009596_Mycob 199 RPDDHMLTTLISGCSEEGTTLSDNEIRDSIVSLITAGYETTSGALAWAIYALLTVPGTWESAASEVARVL 268

CYP139A1_2577884547_Mycob 199 RPDDHMLTTLISGCSEEGTTLSDNEIRDSIVSLITAGYETTSGALAWAIYALLTVPGTWESAASEVARVL 268

CYP139A1_2577997123_Mycob 199 RPDDHMLTTLISGCSEEGTTLSDNEIRDSIVSLITAGYETTSGALAWAIYALLTVPGTWESAASEVARVL 268

CYP139A1_2578155745_Mycob 199 RPDDHMLTTLISGCSEEGTTLSDNEIRDSIVSLITAGYETTSGALAWAIYALLTVPGTWESAASEVARVL 268

CYP139A1_2581807696_Mycob 199 RPDDHMLTTLISGCSEEGTTLSDNEIRDSIVSLITAGYETTSGALAWAIYALLTVPGTWESAASEVARVL 268

CYP139A1_2582018155_Mycob 199 RPDDHMLTTLISGCSEEGTTLSDNEIRDSIVSLITAGYETTSGALAWAIYALLTVPGTWESAASEVARVL 268

CYP139A1_2589154518_Mycob 199 RPDDHMLTTLISGCSEEGTTLSDNEIRDSIVSLITAGYETTSGALAWAIYALLTVPGTWESAASEVARVL 268

CYP139A1_2590109466_Mycob 199 RPDDHMLTTLISGCSEEGTTLSDNEIRDSIVSLITAGYETTSGALAWAIYALLTVPGTWESAASEVARVL 268

CYP139A1_2590195268_Mycob 199 RPDDHMLTTLISGCSEEGTTLSDNEIRDSIVSLITAGYETTSGALAWAIYALLTVPGTWESAASEVARVL 268

CYP139A1_2590198349_Mycob 199 RPDDHMLTTLISGCSEEGTTLSDNEIRDSIVSLITAGYETTSGALAWAIYALLTVPGTWESAASEVARVL 268

CYP139A1_2574799840_Mycob 199 RPDDHMLTTLISGCSEEGTTLSDNEIRDSIVSLITAGYETTSGALAWAIYALLTVPGTWESAASEVARVL 268

CYP139A1_2576399693_Mycob 199 RPDDHMLTTLISGCSEEGTTLSDNEIRDSIVSLITAGYETTSGALAWAIYALLTVPGTWESAASEVARVL 268

CYP139A1_2577024745_Mycob 199 RPDDHMLTTLISGCSEEGTTLSDNEIRDSIVSLITAGYETTSGALAWAIYALLTVPGTWESAASEVARVL 268

CYP139A1_2577872587_Mycob 199 RPDDHMLTTLISGCSEEGTTLSDNEIRDSIVSLITAGYETTSGALAWAIYALLTVPGTWESAASEVARVL 268

CYP139A1_2579813772_Mycob 199 RPDDHMLTTLISGCSEEGTTLSDNEIRDSIVSLITAGYETTSGALAWAIYALLTVPGTWESAASEVARVL 268

CYP139A1_2584772421_Mycob 199 RPDDHMLTTLISGCSEEGTTLSDNEIRDSIVSLITAGYETTSGALAWAIYALLTVPGTWESAASEVARVL 268

CYP139A1_2584893836_Mycob 199 RPDDHMLTTLISGCSEEGTTLSDNEIRDSIVSLITAGYETTSGALAWAIYALLTVPGTWESAASEVARVL 268

CYP139A1_2589026697_Mycob 199 RPDDHMLTTLISGCSEEGTTLSDNEIRDSIVSLITAGYETTSGALAWAIYALLTVPGTWESAASEVARVL 268

CYP139A1_2589514641_Mycob 199 RPDDHMLTTLISGCSEEGTTLSDNEIRDSIVSLITAGYETTSGALAWAIYALLTVPGTWESAASEVARVL 268

CYP139A1_2589547247_Mycob 199 RPDDHMLTTLISGCSEEGTTLSDNEIRDSIVSLITAGYETTSGALAWAIYALLTVPGTWESAASEVARVL 268

CYP139A1_2589620637_Mycob 199 RPDDHMLTTLISGCSEEGTTLSDNEIRDSIVSLITAGYETTSGALAWAIYALLTVPGTWESAASEVARVL 268

CYP139A1_2592230560_Mycob 199 RPDDHMLTTLISGCSEEGTTLSDNEIRDSIVSLITAGYETTSGALAWAIYALLTVPGTWESAASEVARVL 268

CYP139A1_2592242791_Mycob 199 RPDDHMLTTLISGCSEEGTTLSDNEIRDSIVSLITAGYETTSGALAWAIYALLTVPGTWESAASEVARVL 268

CYP139A1_2592299949_Mycob 199 RPDDHMLTTLISGCSEEGTTLSDNEIRDSIVSLITAGYETTSGALAWAIYALLTVPGTWESAASEVARVL 268

CYP139A1_2592377452_Mycob 199 RPDDHMLTTLISGCSEEGTTLSDNEIRDSIVSLITAGYETTSGALAWAIYALLTVPGTWESAASEVARVL 268

CYP139A1_2592405959_Mycob 199 RPDDHMLTTLISGCSEEGTTLSDNEIRDSIVSLITAGYETTSGALAWAIYALLTVPGTWESAASEVARVL 268

CYP139A1_2592558321_Mycob 199 RPDDHMLTTLISGCSEEGTTLSDNEIRDSIVSLITAGYETTSGALAWAIYALLTVPGTWESAASEVARVL 268

CYP139A1_648456112_Mycoba 199 RPDDHMLTTLISGCSEEGTTLSDNEIRDSIVSLITAGYETTSGALAWAIYALLTVPGTWESAASEVARVL 268

CYP139A1_2575561335_Mycob 199 RPDDHMLTTLISGCSEEGTTLSDNEIRDSIVSLITAGYETTSGALAWAIYALLTVPGTWESAASEVARVL 268

CYP139A1_2575869049_Mycob 199 RPDDHMLTTLISGCSEEGTTLSDNEIRDSIVSLITAGYETTSGALAWAIYALLTVPGTWESAASEVARVL 268

CYP139A1_2576250927_Mycob 199 RPDDHMLTTLISGCSEEGTTLSDNEIRDSIVSLITAGYETTSGALAWAIYALLTVPGTWESAASEVARVL 268

CYP139A1_2577075963_Mycob 199 RPDDHMLTTLISGCSEEGTTLSDNEIRDSIVSLITAGYETTSGALAWAIYALLTVPGTWESAASEVARVL 268

CYP139A1_2577655387_Mycob 199 RPDDHMLTTLISGCSEEGTTLSDNEIRDSIVSLITAGYETTSGALAWAIYALLTVPGTWESAASEVARVL 268

CYP139A1_2579808474_Mycob 199 RPDDHMLTTLISGCSEEGTTLSDNEIRDSIVSLITAGYETTSGALAWAIYALLTVPGTWESAASEVARVL 268

CYP139A1_2580939152_Mycob 199 RPDDHMLTTLISGCSEEGTTLSDNEIRDSIVSLITAGYETTSGALAWAIYALLTVPGTWESAASEVARVL 268

CYP139A1_2581510874_Mycob 199 RPDDHMLTTLISGCSEEGTTLSDNEIRDSIVSLITAGYETTSGALAWAIYALLTVPGTWESAASEVARVL 268

CYP139A1_2590048710_Mycob 199 RPDDHMLTTLISGCSEEGTTLSDNEIRDSIVSLITAGYETTSGALAWAIYALLTVPGTWESAASEVARVL 268

CYP139A1_2590237321_Mycob 199 RPDDHMLTTLISGCSEEGTTLSDNEIRDSIVSLITAGYETTSGALAWAIYALLTVPGTWESAASEVARVL 268

CYP139A1_2574784397_Mycob 199 RPDDHMLTTLISGCSEEGTTLSDNEIRDSIVSLITAGYETTSGALAWAIYALLTVPGTWESAASEVARVL 268

CYP139A1_2574860198_Mycob 199 RPDDHMLTTLISGCSEEGTTLSDNEIRDSIVSLITAGYETTSGALAWAIYALLTVPGTWESAASEVARVL 268

CYP139A1_2574872651_Mycob 199 RPDDHMLTTLISGCSEEGTTLSDNEIRDSIVSLITAGYETTSGALAWAIYALLTVPGTWESAASEVARVL 268

CYP139A1_2575185207_Mycob 199 RPDDHMLTTLISGCSEEGTTLSDNEIRDSIVSLITAGYETTSGALAWAIYALLTVPGTWESAASEVARVL 268

CYP139A1_2576316581_Mycob 199 RPDDHMLTTLISGCSEEGTTLSDNEIRDSIVSLITAGYETTSGALAWAIYALLTVPGTWESAASEVARVL 268

CYP139A1_2576932272_Mycob 199 RPDDHMLTTLISGCSEEGTTLSDNEIRDSIVSLITAGYETTSGALAWAIYALLTVPGTWESAASEVARVL 268

CYP139A1_2577632297_Mycob 199 RPDDHMLTTLISGCSEEGTTLSDNEIRDSIVSLITAGYETTSGALAWAIYALLTVPGTWESAASEVARVL 268

CYP139A1_2577641910_Mycob 199 RPDDHMLTTLISGCSEEGTTLSDNEIRDSIVSLITAGYETTSGALAWAIYALLTVPGTWESAASEVARVL 268

CYP139A1_2584609065_Mycob 199 RPDDHMLTTLISGCSEEGTTLSDNEIRDSIVSLITAGYETTSGALAWAIYALLTVPGTWESAASEVARVL 268

CYP139A1_2584674477_Mycob 199 RPDDHMLTTLISGCSEEGTTLSDNEIRDSIVSLITAGYETTSGALAWAIYALLTVPGTWESAASEVARVL 268

CYP139A1_2584806904_Mycob 199 RPDDHMLTTLISGCSEEGTTLSDNEIRDSIVSLITAGYETTSGALAWAIYALLTVPGTWESAASEVARVL 268

CYP139A1_2584849650_Mycob 199 RPDDHMLTTLISGCSEEGTTLSDNEIRDSIVSLITAGYETTSGALAWAIYALLTVPGTWESAASEVARVL 268

CYP139A1_2584854188_Mycob 199 RPDDHMLTTLISGCSEEGTTLSDNEIRDSIVSLITAGYETTSGALAWAIYALLTVPGTWESAASEVARVL 268

CYP139A1_2589100068_Mycob 199 RPDDHMLTTLISGCSEEGTTLSDNEIRDSIVSLITAGYETTSGALAWAIYALLTVPGTWESAASEVARVL 268

CYP139A1_2589666655_Mycob 199 RPDDHMLTTLISGCSEEGTTLSDNEIRDSIVSLITAGYETTSGALAWAIYALLTVPGTWESAASEVARVL 268

CYP139A1_2589703490_Mycob 199 RPDDHMLTTLISGCSEEGTTLSDNEIRDSIVSLITAGYETTSGALAWAIYALLTVPGTWESAASEVARVL 268

CYP139A1_2589724111_Mycob 199 RPDDHMLTTLISGCSEEGTTLSDNEIRDSIVSLITAGYETTSGALAWAIYALLTVPGTWESAASEVARVL 268

CYP139A1_2590117900_Mycob 199 RPDDHMLTTLISGCSEEGTTLSDNEIRDSIVSLITAGYETTSGALAWAIYALLTVPGTWESAASEVARVL 268

CYP139A1_2590134192_Mycob 199 RPDDHMLTTLISGCSEEGTTLSDNEIRDSIVSLITAGYETTSGALAWAIYALLTVPGTWESAASEVARVL 268

CYP139A1_2590166763_Mycob 199 RPDDHMLTTLISGCSEEGTTLSDNEIRDSIVSLITAGYETTSGALAWAIYALLTVPGTWESAASEVARVL 268

CYP139A1_2590356674_Mycob 199 RPDDHMLTTLISGCSEEGTTLSDNEIRDSIVSLITAGYETTSGALAWAIYALLTVPGTWESAASEVARVL 268

CYP139A1_2549410800_Mycob 184 RPDDHMLTTLISGCSEEGTTLSDNEIRDSIVSLITAGYETTSGALAWAIYALLTVPGTWESAASEVARVL 253

CYP139A1_2575655283_Mycob 199 RPDDHMLTTLISGCSEEGTTLSDNEIRDSIVSLITAGYETTSGALAWAIYALLTVPGTWESAASEVARVL 268

CYP139A1_2576373809_Mycob 199 RPDDHMLTTLISGCSEEGTTLSDNEIRDSIVSLITAGYETTSGALAWAIYALLTVPGTWESAASEVARVL 268

CYP139A1_2576609942_Mycob 199 RPDDHMLTTLISGCSEEGTTLSDNEIRDSIVSLITAGYETTSGALAWAIYALLTVPGTWESAASEVARVL 268

CYP139A1_2576684060_Mycob 199 RPDDHMLTTLISGCSEEGTTLSDNEIRDSIVSLITAGYETTSGALAWAIYALLTVPGTWESAASEVARVL 268

CYP139A1_2577110129_Mycob 199 RPDDHMLTTLISGCSEEGTTLSDNEIRDSIVSLITAGYETTSGALAWAIYALLTVPGTWESAASEVARVL 268

CYP139A1_2577733463_Mycob 199 RPDDHMLTTLISGCSEEGTTLSDNEIRDSIVSLITAGYETTSGALAWAIYALLTVPGTWESAASEVARVL 268

CYP139A1_2578170996_Mycob 199 RPDDHMLTTLISGCSEEGTTLSDNEIRDSIVSLITAGYETTSGALAWAIYALLTVPGTWESAASEVARVL 268

CYP139A1_2583731958_Mycob 199 RPDDHMLTTLISGCSEEGTTLSDNEIRDSIVSLITAGYETTSGALAWAIYALLTVPGTWESAASEVARVL 268

CYP139A1_2590523531_Mycob 199 RPDDHMLTTLISGCSEEGTTLSDNEIRDSIVSLITAGYETTSGALAWAIYALLTVPGTWESAASEVARVL 268

CYP139A1_2590526411_Mycob 199 RPDDHMLTTLISGCSEEGTTLSDNEIRDSIVSLITAGYETTSGALAWAIYALLTVPGTWESAASEVARVL 268

CYP139A1_2590552289_Mycob 199 RPDDHMLTTLISGCSEEGTTLSDNEIRDSIVSLITAGYETTSGALAWAIYALLTVPGTWESAASEVARVL 268

CYP139A1_2575205306_Mycob 199 RPDDHMLTTLISGCSEEGTTLSDNEIRDSIVSLITAGYETTSGALAWAIYALLTVPGTWESAASEVARVL 268

CYP139A1_2575230562_Mycob 199 RPDDHMLTTLISGCSEEGTTLSDNEIRDSIVSLITAGYETTSGALAWAIYALLTVPGTWESAASEVARVL 268

CYP139A1_2575304538_Mycob 199 RPDDHMLTTLISGCSEEGTTLSDNEIRDSIVSLITAGYETTSGALAWAIYALLTVPGTWESAASEVARVL 268

CYP139A1_2575476916_Mycob 199 RPDDHMLTTLISGCSEEGTTLSDNEIRDSIVSLITAGYETTSGALAWAIYALLTVPGTWESAASEVARVL 268

CYP139A1_2575790354_Mycob 199 RPDDHMLTTLISGCSEEGTTLSDNEIRDSIVSLITAGYETTSGALAWAIYALLTVPGTWESAASEVARVL 268

CYP139A1_2576077839_Mycob 199 RPDDHMLTTLISGCSEEGTTLSDNEIRDSIVSLITAGYETTSGALAWAIYALLTVPGTWESAASEVARVL 268

CYP139A1_2576163842_Mycob 199 RPDDHMLTTLISGCSEEGTTLSDNEIRDSIVSLITAGYETTSGALAWAIYALLTVPGTWESAASEVARVL 268

CYP139A1_2577313382_Mycob 199 RPDDHMLTTLISGCSEEGTTLSDNEIRDSIVSLITAGYETTSGALAWAIYALLTVPGTWESAASEVARVL 268

CYP139A1_2577519304_Mycob 199 RPDDHMLTTLISGCSEEGTTLSDNEIRDSIVSLITAGYETTSGALAWAIYALLTVPGTWESAASEVARVL 268

CYP139A1_2577816400_Mycob 199 RPDDHMLTTLISGCSEEGTTLSDNEIRDSIVSLITAGYETTSGALAWAIYALLTVPGTWESAASEVARVL 268

CYP139A1_2584670398_Mycob 199 RPDDHMLTTLISGCSEEGTTLSDNEIRDSIVSLITAGYETTSGALAWAIYALLTVPGTWESAASEVARVL 268

CYP139A1_2584841030_Mycob 199 RPDDHMLTTLISGCSEEGTTLSDNEIRDSIVSLITAGYETTSGALAWAIYALLTVPGTWESAASEVARVL 268

CYP139A1_2584865918_Mycob 199 RPDDHMLTTLISGCSEEGTTLSDNEIRDSIVSLITAGYETTSGALAWAIYALLTVPGTWESAASEVARVL 268

CYP139A1_2589588224_Mycob 199 RPDDHMLTTLISGCSEEGTTLSDNEIRDSIVSLITAGYETTSGALAWAIYALLTVPGTWESAASEVARVL 268

CYP139A1_2589624713_Mycob 199 RPDDHMLTTLISGCSEEGTTLSDNEIRDSIVSLITAGYETTSGALAWAIYALLTVPGTWESAASEVARVL 268

CYP139A1_2589736180_Mycob 199 RPDDHMLTTLISGCSEEGTTLSDNEIRDSIVSLITAGYETTSGALAWAIYALLTVPGTWESAASEVARVL 268

CYP139A1_2590032388_Mycob 199 RPDDHMLTTLISGCSEEGTTLSDNEIRDSIVSLITAGYETTSGALAWAIYALLTVPGTWESAASEVARVL 268

CYP139A1_2590501611_Mycob 199 RPDDHMLTTLISGCSEEGTTLSDNEIRDSIVSLITAGYETTSGALAWAIYALLTVPGTWESAASEVARVL 268

CYP139A1_2592312197_Mycob 199 RPDDHMLTTLISGCSEEGTTLSDNEIRDSIVSLITAGYETTSGALAWAIYALLTVPGTWESAASEVARVL 268

CYP139A1_2592435284_Mycob 199 RPDDHMLTTLISGCSEEGTTLSDNEIRDSIVSLITAGYETTSGALAWAIYALLTVPGTWESAASEVARVL 268

CYP139A1_2511811274_Mycob 199 RPDDHMLTTLISGCSEEGTTLSDNEIRDSIVSLITAGYETTSGALAWAIYALLTVPGTWESAASEVARVL 268

CYP139A1_2546202077_Mycob 199 RPDDHMLTTLISGCSEEGTTLSDNEIRDSIVSLITAGYETTSGALAWAIYALLTVPGTWESAASEVARVL 268

CYP139A1_2574693694_Mycob 199 RPDDHMLTTLISGCSEEGTTLSDNEIRDSIVSLITAGYETTSGALAWAIYALLTVPGTWESAASEVARVL 268

CYP139A1_2574968392_Mycob 199 RPDDHMLTTLISGCSEEGTTLSDNEIRDSIVSLITAGYETTSGALAWAIYALLTVPGTWESAASEVARVL 268

CYP139A1_2577060340_Mycob 199 RPDDHMLTTLISGCSEEGTTLSDNEIRDSIVSLITAGYETTSGALAWAIYALLTVPGTWESAASEVARVL 268

CYP139A1_2577454945_Mycob 199 RPDDHMLTTLISGCSEEGTTLSDNEIRDSIVSLITAGYETTSGALAWAIYALLTVPGTWESAASEVARVL 268

CYP139A1_2580123929_Mycob 199 RPDDHMLTTLISGCSEEGTTLSDNEIRDSIVSLITAGYETTSGALAWAIYALLTVPGTWESAASEVARVL 268

CYP139A1_2581930746_Mycob 199 RPDDHMLTTLISGCSEEGTTLSDNEIRDSIVSLITAGYETTSGALAWAIYALLTVPGTWESAASEVARVL 268

CYP139A1_2584196432_Mycob 199 RPDDHMLTTLISGCSEEGTTLSDNEIRDSIVSLITAGYETTSGALAWAIYALLTVPGTWESAASEVARVL 268

CYP139A1_2584726832_Mycob 199 RPDDHMLTTLISGCSEEGTTLSDNEIRDSIVSLITAGYETTSGALAWAIYALLTVPGTWESAASEVARVL 268

CYP139A1_2584911080_Mycob 199 RPDDHMLTTLISGCSEEGTTLSDNEIRDSIVSLITAGYETTSGALAWAIYALLTVPGTWESAASEVARVL 268

CYP139A1_2584962435_Mycob 199 RPDDHMLTTLISGCSEEGTTLSDNEIRDSIVSLITAGYETTSGALAWAIYALLTVPGTWESAASEVARVL 268

CYP139A1_2584995097_Mycob 199 RPDDHMLTTLISGCSEEGTTLSDNEIRDSIVSLITAGYETTSGALAWAIYALLTVPGTWESAASEVARVL 268

CYP139A1_640602381_Mycoba 199 RPDDHMLTTLISGCSEEGTTLSDNEIRDSIVSLITAGYETTSGALAWAIYALLTVPGTWESAASEVARVL 268

CYP139A1_643019022_Mycoba 199 RPDDHMLTTLISGCSEEGTTLSDNEIRDSIVSLITAGYETTSGALAWAIYALLTVPGTWESAASEVARVL 268

CYP139A1_2573562450_Mycob 199 RPDDHMLTTLISGCSEEGTTLSDNEIRDSIVSLITAGYETTSGALAWAIYALLTVPGTWESAASEVARVL 268

CYP139A1_2575255926_Mycob 199 RPDDHMLTTLISGCSEEGTTLSDNEIRDSIVSLITAGYETTSGALAWAIYALLTVPGTWESAASEVARVL 268

CYP139A1_2577905307_Mycob 199 RPDDHMLTTLISGCSEEGTTLSDNEIRDSIVSLITAGYETTSGALAWAIYALLTVPGTWESAASEVARVL 268

CYP139A1_2579818167_Mycob 199 RPDDHMLTTLISGCSEEGTTLSDNEIRDSIVSLITAGYETTSGALAWAIYALLTVPGTWESAASEVARVL 268

CYP139A1_2582001898_Mycob 199 RPDDHMLTTLISGCSEEGTTLSDNEIRDSIVSLITAGYETTSGALAWAIYALLTVPGTWESAASEVARVL 268

CYP139A1_2588538927_Mycob 199 RPDDHMLTTLISGCSEEGTTLSDNEIRDSIVSLITAGYETTSGALAWAIYALLTVPGTWESAASEVARVL 268

CYP139A1_2590073317_Mycob 199 RPDDHMLTTLISGCSEEGTTLSDNEIRDSIVSLITAGYETTSGALAWAIYALLTVPGTWESAASEVARVL 268

CYP139A1_2590075971_Mycob 199 RPDDHMLTTLISGCSEEGTTLSDNEIRDSIVSLITAGYETTSGALAWAIYALLTVPGTWESAASEVARVL 268

CYP139A1_2590211264_Mycob 199 RPDDHMLTTLISGCSEEGTTLSDNEIRDSIVSLITAGYETTSGALAWAIYALLTVPGTWESAASEVARVL 268

CYP139A1_2590249475_Mycob 199 RPDDHMLTTLISGCSEEGTTLSDNEIRDSIVSLITAGYETTSGALAWAIYALLTVPGTWESAASEVARVL 268

CYP139A1_2590279314_Mycob 199 RPDDHMLTTLISGCSEEGTTLSDNEIRDSIVSLITAGYETTSGALAWAIYALLTVPGTWESAASEVARVL 268

CYP139A1_2590559932_Mycob 199 RPDDHMLTTLISGCSEEGTTLSDNEIRDSIVSLITAGYETTSGALAWAIYALLTVPGTWESAASEVARVL 268

CYP139A1_640606444_Mycoba 199 RPDDHMLTTLISGCSEEGTTLSDNEIRDSIVSLITAGYETTSGALAWAIYALLTVPGTWESAASEVARVL 268

CYP139A1_651025167_Mycoba 199 RPDDHMLTTLISGCSEEGTTLSDNEIRDSIVSLITAGYETTSGALAWAIYALLTVPGTWESAASEVARVL 268

CYP139A1_2574876573_Mycob 199 RPDDHMLTTLISGCSEEGTTLSDNEIRDSIVSLITAGYETTSGALAWAIYALLTVPGTWESAASEVARVL 268

CYP139A1_2575028161_Mycob 199 RPDDHMLTTLISGCSEEGTTLSDNEIRDSIVSLITAGYETTSGALAWAIYALLTVPGTWESAASEVARVL 268

CYP139A1_2575888575_Mycob 199 RPDDHMLTTLISGCSEEGTTLSDNEIRDSIVSLITAGYETTSGALAWAIYALLTVPGTWESAASEVARVL 268

CYP139A1_2576708817_Mycob 199 RPDDHMLTTLISGCSEEGTTLSDNEIRDSIVSLITAGYETTSGALAWAIYALLTVPGTWESAASEVARVL 268

CYP139A1_2577386815_Mycob 199 RPDDHMLTTLISGCSEEGTTLSDNEIRDSIVSLITAGYETTSGALAWAIYALLTVPGTWESAASEVARVL 268

CYP139A1_2578040087_Mycob 199 RPDDHMLTTLISGCSEEGTTLSDNEIRDSIVSLITAGYETTSGALAWAIYALLTVPGTWESAASEVARVL 268

CYP139A1_2584645880_Mycob 199 RPDDHMLTTLISGCSEEGTTLSDNEIRDSIVSLITAGYETTSGALAWAIYALLTVPGTWESAASEVARVL 268

CYP139A1_2584659187_Mycob 199 RPDDHMLTTLISGCSEEGTTLSDNEIRDSIVSLITAGYETTSGALAWAIYALLTVPGTWESAASEVARVL 268

CYP139A1_2584718757_Mycob 199 RPDDHMLTTLISGCSEEGTTLSDNEIRDSIVSLITAGYETTSGALAWAIYALLTVPGTWESAASEVARVL 268

CYP139A1_2589064595_Mycob 199 RPDDHMLTTLISGCSEEGTTLSDNEIRDSIVSLITAGYETTSGALAWAIYALLTVPGTWESAASEVARVL 268

CYP139A1_2589113575_Mycob 199 RPDDHMLTTLISGCSEEGTTLSDNEIRDSIVSLITAGYETTSGALAWAIYALLTVPGTWESAASEVARVL 268

CYP139A1_2589559459_Mycob 199 RPDDHMLTTLISGCSEEGTTLSDNEIRDSIVSLITAGYETTSGALAWAIYALLTVPGTWESAASEVARVL 268

CYP139A1_2589575466_Mycob 199 RPDDHMLTTLISGCSEEGTTLSDNEIRDSIVSLITAGYETTSGALAWAIYALLTVPGTWESAASEVARVL 268

CYP139A1_2589595990_Mycob 199 RPDDHMLTTLISGCSEEGTTLSDNEIRDSIVSLITAGYETTSGALAWAIYALLTVPGTWESAASEVARVL 268

CYP139A1_2592259141_Mycob 199 RPDDHMLTTLISGCSEEGTTLSDNEIRDSIVSLITAGYETTSGALAWAIYALLTVPGTWESAASEVARVL 268

CYP139A1_2592280664_Mycob 199 RPDDHMLTTLISGCSEEGTTLSDNEIRDSIVSLITAGYETTSGALAWAIYALLTVPGTWESAASEVARVL 268

CYP139A1_2592389699_Mycob 199 RPDDHMLTTLISGCSEEGTTLSDNEIRDSIVSLITAGYETTSGALAWAIYALLTVPGTWESAASEVARVL 268

CYP139A1_2592438630_Mycob 199 RPDDHMLTTLISGCSEEGTTLSDNEIRDSIVSLITAGYETTSGALAWAIYALLTVPGTWESAASEVARVL 268

CYP139A1_2592553482_Mycob 199 RPDDHMLTTLISGCSEEGTTLSDNEIRDSIVSLITAGYETTSGALAWAIYALLTVPGTWESAASEVARVL 268

CYP139A1_2592570555_Mycob 199 RPDDHMLTTLISGCSEEGTTLSDNEIRDSIVSLITAGYETTSGALAWAIYALLTVPGTWESAASEVARVL 268

CYP139A1_2575252379_Mycob 199 RPDDHMLTTLISGCSEEGTTLSDNEIRDSIVSLITAGYETTSGALAWAIYALLTVPGTWESAASEVARVL 268

CYP139A1_2575520720_Mycob 199 RPDDHMLTTLISGCSEEGTTLSDNEIRDSIVSLITAGYETTSGALAWAIYALLTVPGTWESAASEVARVL 268

CYP139A1_2575705222_Mycob 199 RPDDHMLTTLISGCSEEGTTLSDNEIRDSIVSLITAGYETTSGALAWAIYALLTVPGTWESAASEVARVL 268

CYP139A1_2575964273_Mycob 199 RPDDHMLTTLISGCSEEGTTLSDNEIRDSIVSLITAGYETTSGALAWAIYALLTVPGTWESAASEVARVL 268

CYP139A1_2577195360_Mycob 199 RPDDHMLTTLISGCSEEGTTLSDNEIRDSIVSLITAGYETTSGALAWAIYALLTVPGTWESAASEVARVL 268

CYP139A1_2581587072_Mycob 199 RPDDHMLTTLISGCSEEGTTLSDNEIRDSIVSLITAGYETTSGALAWAIYALLTVPGTWESAASEVARVL 268

CYP139A1_2584633235_Mycob 199 RPDDHMLTTLISGCSEEGTTLSDNEIRDSIVSLITAGYETTSGALAWAIYALLTVPGTWESAASEVARVL 268

CYP139A1_2584732271_Mycob 199 RPDDHMLTTLISGCSEEGTTLSDNEIRDSIVSLITAGYETTSGALAWAIYALLTVPGTWESAASEVARVL 268

CYP139A1_2584791771_Mycob 199 RPDDHMLTTLISGCSEEGTTLSDNEIRDSIVSLITAGYETTSGALAWAIYALLTVPGTWESAASEVARVL 268

CYP139A1_2589148607_Mycob 199 RPDDHMLTTLISGCSEEGTTLSDNEIRDSIVSLITAGYETTSGALAWAIYALLTVPGTWESAASEVARVL 268

CYP139A1_2590150448_Mycob 199 RPDDHMLTTLISGCSEEGTTLSDNEIRDSIVSLITAGYETTSGALAWAIYALLTVPGTWESAASEVARVL 268

CYP139A1_2590231653_Mycob 199 RPDDHMLTTLISGCSEEGTTLSDNEIRDSIVSLITAGYETTSGALAWAIYALLTVPGTWESAASEVARVL 268

CYP139A1_2590283895_Mycob 199 RPDDHMLTTLISGCSEEGTTLSDNEIRDSIVSLITAGYETTSGALAWAIYALLTVPGTWESAASEVARVL 268

CYP139A1_645120373_Mycoba 199 RPDDHMLTTLISGCSEEGTTLSDNEIRDSIVSLITAGYETTSGALAWAIYALLTVPGTWESAASEVARVL 268

CYP139A1_2574734007_Mycob 199 RPDDHMLTTLISGCSEEGTTLSDNEIRDSIVSLITAGYETTSGALAWAIYALLTVPGTWESAASEVARVL 268

CYP139A1_2575099885_Mycob 199 RPDDHMLTTLISGCSEEGTTLSDNEIRDSIVSLITAGYETTSGALAWAIYALLTVPGTWESAASEVARVL 268

CYP139A1_2575132657_Mycob 199 RPDDHMLTTLISGCSEEGTTLSDNEIRDSIVSLITAGYETTSGALAWAIYALLTVPGTWESAASEVARVL 268

CYP139A1_2575465536_Mycob 199 RPDDHMLTTLISGCSEEGTTLSDNEIRDSIVSLITAGYETTSGALAWAIYALLTVPGTWESAASEVARVL 268

CYP139A1_2576665707_Mycob 199 RPDDHMLTTLISGCSEEGTTLSDNEIRDSIVSLITAGYETTSGALAWAIYALLTVPGTWESAASEVARVL 268

CYP139A1_2576698280_Mycob 199 RPDDHMLTTLISGCSEEGTTLSDNEIRDSIVSLITAGYETTSGALAWAIYALLTVPGTWESAASEVARVL 268

CYP139A1_2576759003_Mycob 199 RPDDHMLTTLISGCSEEGTTLSDNEIRDSIVSLITAGYETTSGALAWAIYALLTVPGTWESAASEVARVL 268

CYP139A1_2577673179_Mycob 199 RPDDHMLTTLISGCSEEGTTLSDNEIRDSIVSLITAGYETTSGALAWAIYALLTVPGTWESAASEVARVL 268

CYP139A1_2577936335_Mycob 199 RPDDHMLTTLISGCSEEGTTLSDNEIRDSIVSLITAGYETTSGALAWAIYALLTVPGTWESAASEVARVL 268

CYP139A1_2578008273_Mycob 199 RPDDHMLTTLISGCSEEGTTLSDNEIRDSIVSLITAGYETTSGALAWAIYALLTVPGTWESAASEVARVL 268

CYP139A1_2578073775_Mycob 199 RPDDHMLTTLISGCSEEGTTLSDNEIRDSIVSLITAGYETTSGALAWAIYALLTVPGTWESAASEVARVL 268

CYP139A1_2584960744_Mycob 199 RPDDHMLTTLISGCSEEGTTLSDNEIRDSIVSLITAGYETTSGALAWAIYALLTVPGTWESAASEVARVL 268

CYP139A1_2588992251_Mycob 199 RPDDHMLTTLISGCSEEGTTLSDNEIRDSIVSLITAGYETTSGALAWAIYALLTVPGTWESAASEVARVL 268

CYP139A1_2589077872_Mycob 199 RPDDHMLTTLISGCSEEGTTLSDNEIRDSIVSLITAGYETTSGALAWAIYALLTVPGTWESAASEVARVL 268

CYP139A1_2589518744_Mycob 199 RPDDHMLTTLISGCSEEGTTLSDNEIRDSIVSLITAGYETTSGALAWAIYALLTVPGTWESAASEVARVL 268

CYP139A1_2589583955_Mycob 199 RPDDHMLTTLISGCSEEGTTLSDNEIRDSIVSLITAGYETTSGALAWAIYALLTVPGTWESAASEVARVL 268

CYP139A1_2589646609_Mycob 199 RPDDHMLTTLISGCSEEGTTLSDNEIRDSIVSLITAGYETTSGALAWAIYALLTVPGTWESAASEVARVL 268

CYP139A1_2592316279_Mycob 199 RPDDHMLTTLISGCSEEGTTLSDNEIRDSIVSLITAGYETTSGALAWAIYALLTVPGTWESAASEVARVL 268

CYP139A1_2592546111_Mycob 199 RPDDHMLTTLISGCSEEGTTLSDNEIRDSIVSLITAGYETTSGALAWAIYALLTVPGTWESAASEVARVL 268

CYP139A1_2511553315_Mycob 199 RPDDHMLTTLISGCSEEGTTLSDNEIRDSIVSLITAGYETTSGALAWAIYALLTVPGTWESAASEVARVL 268

CYP139A1_2574949227_Mycob 199 RPDDHMLTTLISGCSEEGTTLSDNEIRDSIVSLITAGYETTSGALAWAIYALLTVPGTWESAASEVARVL 268

CYP139A1_2575533703_Mycob 199 RPDDHMLTTLISGCSEEGTTLSDNEIRDSIVSLITAGYETTSGALAWAIYALLTVPGTWESAASEVARVL 268

CYP139A1_2576015739_Mycob 199 RPDDHMLTTLISGCSEEGTTLSDNEIRDSIVSLITAGYETTSGALAWAIYALLTVPGTWESAASEVARVL 268

CYP139A1_2576644886_Mycob 199 RPDDHMLTTLISGCSEEGTTLSDNEIRDSIVSLITAGYETTSGALAWAIYALLTVPGTWESAASEVARVL 268

CYP139A1_2577497204_Mycob 199 RPDDHMLTTLISGCSEEGTTLSDNEIRDSIVSLITAGYETTSGALAWAIYALLTVPGTWESAASEVARVL 268

CYP139A1_2580744928_Mycob 199 RPDDHMLTTLISGCSEEGTTLSDNEIRDSIVSLITAGYETTSGALAWAIYALLTVPGTWESAASEVARVL 268

CYP139A1_2583727889_Mycob 199 RPDDHMLTTLISGCSEEGTTLSDNEIRDSIVSLITAGYETTSGALAWAIYALLTVPGTWESAASEVARVL 268

CYP139A1_2590064965_Mycob 199 RPDDHMLTTLISGCSEEGTTLSDNEIRDSIVSLITAGYETTSGALAWAIYALLTVPGTWESAASEVARVL 268

CYP139A1_2590121989_Mycob 199 RPDDHMLTTLISGCSEEGTTLSDNEIRDSIVSLITAGYETTSGALAWAIYALLTVPGTWESAASEVARVL 268

CYP139A1_2590294228_Mycob 199 RPDDHMLTTLISGCSEEGTTLSDNEIRDSIVSLITAGYETTSGALAWAIYALLTVPGTWESAASEVARVL 268

CYP139A1_2590499050_Mycob 199 RPDDHMLTTLISGCSEEGTTLSDNEIRDSIVSLITAGYETTSGALAWAIYALLTVPGTWESAASEVARVL 268

CYP139A1_2590519464_Mycob 199 RPDDHMLTTLISGCSEEGTTLSDNEIRDSIVSLITAGYETTSGALAWAIYALLTVPGTWESAASEVARVL 268

CYP139A1_2590548005_Mycob 199 RPDDHMLTTLISGCSEEGTTLSDNEIRDSIVSLITAGYETTSGALAWAIYALLTVPGTWESAASEVARVL 268

CYP139A1_637139034_Mycoba 199 RPDDHMLTTLISGCSEEGTTLSDNEIRDSIVSLITAGYETTSGALAWAIYALLTVPGTWESAASEVARVL 268

CYP139A1_639830617_Mycoba 199 RPDDHMLTTLISGCSEEGTTLSDNEIRDSIVSLITAGYETTSGALAWAIYALLTVPGTWESAASEVARVL 268

CYP139A1_2574640348_Mycob 199 RPDDHMLTTLISGCSEEGTTLSDNEIRDSIVSLITAGYETTSGALAWAIYALLTVPGTWESAASEVARVL 268

CYP139A1_2574794413_Mycob 199 RPDDHMLTTLISGCSEEGTTLSDNEIRDSIVSLITAGYETTSGALAWAIYALLTVPGTWESAASEVARVL 268

CYP139A1_2574987296_Mycob 199 RPDDHMLTTLISGCSEEGTTLSDNEIRDSIVSLITAGYETTSGALAWAIYALLTVPGTWESAASEVARVL 268

CYP139A1_2575084753_Mycob 199 RPDDHMLTTLISGCSEEGTTLSDNEIRDSIVSLITAGYETTSGALAWAIYALLTVPGTWESAASEVARVL 268

CYP139A1_2575627312_Mycob 199 RPDDHMLTTLISGCSEEGTTLSDNEIRDSIVSLITAGYETTSGALAWAIYALLTVPGTWESAASEVARVL 268

CYP139A1_2576321347_Mycob 199 RPDDHMLTTLISGCSEEGTTLSDNEIRDSIVSLITAGYETTSGALAWAIYALLTVPGTWESAASEVARVL 268

CYP139A1_2576378950_Mycob 199 RPDDHMLTTLISGCSEEGTTLSDNEIRDSIVSLITAGYETTSGALAWAIYALLTVPGTWESAASEVARVL 268

CYP139A1_2576735552_Mycob 199 RPDDHMLTTLISGCSEEGTTLSDNEIRDSIVSLITAGYETTSGALAWAIYALLTVPGTWESAASEVARVL 268

CYP139A1_2576940434_Mycob 199 RPDDHMLTTLISGCSEEGTTLSDNEIRDSIVSLITAGYETTSGALAWAIYALLTVPGTWESAASEVARVL 268

CYP139A1_2577812038_Mycob 199 RPDDHMLTTLISGCSEEGTTLSDNEIRDSIVSLITAGYETTSGALAWAIYALLTVPGTWESAASEVARVL 268

CYP139A1_2577861791_Mycob 199 RPDDHMLTTLISGCSEEGTTLSDNEIRDSIVSLITAGYETTSGALAWAIYALLTVPGTWESAASEVARVL 268

CYP139A1_2578053379_Mycob 199 RPDDHMLTTLISGCSEEGTTLSDNEIRDSIVSLITAGYETTSGALAWAIYALLTVPGTWESAASEVARVL 268

CYP139A1_2584860925_Mycob 199 RPDDHMLTTLISGCSEEGTTLSDNEIRDSIVSLITAGYETTSGALAWAIYALLTVPGTWESAASEVARVL 268

CYP139A1_2584936325_Mycob 199 RPDDHMLTTLISGCSEEGTTLSDNEIRDSIVSLITAGYETTSGALAWAIYALLTVPGTWESAASEVARVL 268

CYP139A1_2584939524_Mycob 199 RPDDHMLTTLISGCSEEGTTLSDNEIRDSIVSLITAGYETTSGALAWAIYALLTVPGTWESAASEVARVL 268

CYP139A1_2589106145_Mycob 199 RPDDHMLTTLISGCSEEGTTLSDNEIRDSIVSLITAGYETTSGALAWAIYALLTVPGTWESAASEVARVL 268

CYP139A1_2589543202_Mycob 199 RPDDHMLTTLISGCSEEGTTLSDNEIRDSIVSLITAGYETTSGALAWAIYALLTVPGTWESAASEVARVL 268

CYP139A1_2589642534_Mycob 199 RPDDHMLTTLISGCSEEGTTLSDNEIRDSIVSLITAGYETTSGALAWAIYALLTVPGTWESAASEVARVL 268

CYP139A1_2589691243_Mycob 199 RPDDHMLTTLISGCSEEGTTLSDNEIRDSIVSLITAGYETTSGALAWAIYALLTVPGTWESAASEVARVL 268

CYP139A1_2589695547_Mycob 199 RPDDHMLTTLISGCSEEGTTLSDNEIRDSIVSLITAGYETTSGALAWAIYALLTVPGTWESAASEVARVL 268

CYP139A1_2592222422_Mycob 199 RPDDHMLTTLISGCSEEGTTLSDNEIRDSIVSLITAGYETTSGALAWAIYALLTVPGTWESAASEVARVL 268

CYP139A1_648446923_Mycoba 199 RPDDHMLTTLISGCSEEGTTLSDNEIRDSIVSLITAGYETTSGALAWAIYALLTVPGTWESAASEVARVL 268

CYP139A1_648469578_Mycoba 199 RPDDHMLTTLISGCSEEGTTLSDNEIRDSIVSLITAGYETTSGALAWAIYALLTVPGTWESAASEVARVL 268

CYP139A1_2546188127_Mycob 199 RPDDHMLTTLISGCSEEGTTLSDNEIRDSIVSLITAGYETTSGALAWAIYALLTVPGTWESAASEVARVL 268

CYP139A1_2574773054_Mycob 199 RPDDHMLTTLISGCSEEGTTLSDNEIRDSIVSLITAGYETTSGALAWAIYALLTVPGTWESAASEVARVL 268

CYP139A1_2574854728_Mycob 199 RPDDHMLTTLISGCSEEGTTLSDNEIRDSIVSLITAGYETTSGALAWAIYALLTVPGTWESAASEVARVL 268

CYP139A1_2575984510_Mycob 199 RPDDHMLTTLISGCSEEGTTLSDNEIRDSIVSLITAGYETTSGALAWAIYALLTVPGTWESAASEVARVL 268

CYP139A1_2576293562_Mycob 199 RPDDHMLTTLISGCSEEGTTLSDNEIRDSIVSLITAGYETTSGALAWAIYALLTVPGTWESAASEVARVL 268

CYP139A1_2576717872_Mycob 199 RPDDHMLTTLISGCSEEGTTLSDNEIRDSIVSLITAGYETTSGALAWAIYALLTVPGTWESAASEVARVL 268

CYP139A1_2578189802_Mycob 199 RPDDHMLTTLISGCSEEGTTLSDNEIRDSIVSLITAGYETTSGALAWAIYALLTVPGTWESAASEVARVL 268

CYP139A1_2584890677_Mycob 199 RPDDHMLTTLISGCSEEGTTLSDNEIRDSIVSLITAGYETTSGALAWAIYALLTVPGTWESAASEVARVL 268

CYP139A1_2590070071_Mycob 199 RPDDHMLTTLISGCSEEGTTLSDNEIRDSIVSLITAGYETTSGALAWAIYALLTVPGTWESAASEVARVL 268

CYP139A1_2590169218_Mycob 199 RPDDHMLTTLISGCSEEGTTLSDNEIRDSIVSLITAGYETTSGALAWAIYALLTVPGTWESAASEVARVL 268

CYP139A1_644880084_Mycoba 199 RPDDHMLTTLISGCSEEGTTLSDNEIRDSIVSLITAGYETTSGALAWAIYALLTVPGTWESAASEVARVL 268

CYP139A1_2574905673_Mycob 199 RPDDHMLTTLISGCSEEGTTLSDNEIRDSIVSLITAGYETTSGALAWAIYALLTVPGTWESAASEVARVL 268

CYP139A1_2575285636_Mycob 199 RPDDHMLTTLISGCSEEGTTLSDNEIRDSIVSLITAGYETTSGALAWAIYALLTVPGTWESAASEVARVL 268

CYP139A1_2575508785_Mycob 199 RPDDHMLTTLISGCSEEGTTLSDNEIRDSIVSLITAGYETTSGALAWAIYALLTVPGTWESAASEVARVL 268

CYP139A1_2576585830_Mycob 199 RPDDHMLTTLISGCSEEGTTLSDNEIRDSIVSLITAGYETTSGALAWAIYALLTVPGTWESAASEVARVL 268

CYP139A1_2584979253_Mycob 199 RPDDHMLTTLISGCSEEGTTLSDNEIRDSIVSLITAGYETTSGALAWAIYALLTVPGTWESAASEVARVL 268

CYP139A1_2588982991_Mycob 199 RPDDHMLTTLISGCSEEGTTLSDNEIRDSIVSLITAGYETTSGALAWAIYALLTVPGTWESAASEVARVL 268

CYP139A1_2589048312_Mycob 199 RPDDHMLTTLISGCSEEGTTLSDNEIRDSIVSLITAGYETTSGALAWAIYALLTVPGTWESAASEVARVL 268

CYP139A1_2589060525_Mycob 199 RPDDHMLTTLISGCSEEGTTLSDNEIRDSIVSLITAGYETTSGALAWAIYALLTVPGTWESAASEVARVL 268

CYP139A1_2589502409_Mycob 199 RPDDHMLTTLISGCSEEGTTLSDNEIRDSIVSLITAGYETTSGALAWAIYALLTVPGTWESAASEVARVL 268

CYP139A1_2589572815_Mycob 199 RPDDHMLTTLISGCSEEGTTLSDNEIRDSIVSLITAGYETTSGALAWAIYALLTVPGTWESAASEVARVL 268

CYP139A1_2590181544_Mycob 199 RPDDHMLTTLISGCSEEGTTLSDNEIRDSIVSLITAGYETTSGALAWAIYALLTVPGTWESAASEVARVL 268

CYP139A1_2592234638_Mycob 199 RPDDHMLTTLISGCSEEGTTLSDNEIRDSIVSLITAGYETTSGALAWAIYALLTVPGTWESAASEVARVL 268

CYP139A1_2592271382_Mycob 199 RPDDHMLTTLISGCSEEGTTLSDNEIRDSIVSLITAGYETTSGALAWAIYALLTVPGTWESAASEVARVL 268

CYP139A1_2592295853_Mycob 199 RPDDHMLTTLISGCSEEGTTLSDNEIRDSIVSLITAGYETTSGALAWAIYALLTVPGTWESAASEVARVL 268

CYP139A1_2592344179_Mycob 199 RPDDHMLTTLISGCSEEGTTLSDNEIRDSIVSLITAGYETTSGALAWAIYALLTVPGTWESAASEVARVL 268

CYP139A1_2592357110_Mycob 199 RPDDHMLTTLISGCSEEGTTLSDNEIRDSIVSLITAGYETTSGALAWAIYALLTVPGTWESAASEVARVL 268

CYP139A1_643035957_Mycoba 199 RPDDHMLTTLISGCSEEGTTLSDNEIRDSIVSLITAGYETTSGALAWAIYALLTVPGTWESAASEVARVL 268

CYP139A1_2575162612_Mycob 199 RPDDHMLTTLISGCSEEGTTLSDNEIRDSIVSLITAGYETTSGALAWAIYALLTVPGTWESAASEVARVL 268

CYP139A1_2575198589_Mycob 199 RPDDHMLTTLISGCSEEGTTLSDNEIRDSIVSLITAGYETTSGALAWAIYALLTVPGTWESAASEVARVL 268

CYP139A1_2575418829_Mycob 199 RPDDHMLTTLISGCSEEGTTLSDNEIRDSIVSLITAGYETTSGALAWAIYALLTVPGTWESAASEVARVL 268

CYP139A1_2576172216_Mycob 199 RPDDHMLTTLISGCSEEGTTLSDNEIRDSIVSLITAGYETTSGALAWAIYALLTVPGTWESAASEVARVL 268

CYP139A1_2576534922_Mycob 199 RPDDHMLTTLISGCSEEGTTLSDNEIRDSIVSLITAGYETTSGALAWAIYALLTVPGTWESAASEVARVL 268

CYP139A1_2577185436_Mycob 199 RPDDHMLTTLISGCSEEGTTLSDNEIRDSIVSLITAGYETTSGALAWAIYALLTVPGTWESAASEVARVL 268

CYP139A1_2577236838_Mycob 199 RPDDHMLTTLISGCSEEGTTLSDNEIRDSIVSLITAGYETTSGALAWAIYALLTVPGTWESAASEVARVL 268

CYP139A1_2577373328_Mycob 199 RPDDHMLTTLISGCSEEGTTLSDNEIRDSIVSLITAGYETTSGALAWAIYALLTVPGTWESAASEVARVL 268

CYP139A1_2578099005_Mycob 199 RPDDHMLTTLISGCSEEGTTLSDNEIRDSIVSLITAGYETTSGALAWAIYALLTVPGTWESAASEVARVL 268

CYP139A1_2580366102_Mycob 199 RPDDHMLTTLISGCSEEGTTLSDNEIRDSIVSLITAGYETTSGALAWAIYALLTVPGTWESAASEVARVL 268

CYP139A1_2580467795_Mycob 199 RPDDHMLTTLISGCSEEGTTLSDNEIRDSIVSLITAGYETTSGALAWAIYALLTVPGTWESAASEVARVL 268

CYP139A1_2584613792_Mycob 199 RPDDHMLTTLISGCSEEGTTLSDNEIRDSIVSLITAGYETTSGALAWAIYALLTVPGTWESAASEVARVL 268

CYP139A1_2584681396_Mycob 199 RPDDHMLTTLISGCSEEGTTLSDNEIRDSIVSLITAGYETTSGALAWAIYALLTVPGTWESAASEVARVL 268

CYP139A1_2584913651_Mycob 199 RPDDHMLTTLISGCSEEGTTLSDNEIRDSIVSLITAGYETTSGALAWAIYALLTVPGTWESAASEVARVL 268

CYP139A1_2584942354_Mycob 199 RPDDHMLTTLISGCSEEGTTLSDNEIRDSIVSLITAGYETTSGALAWAIYALLTVPGTWESAASEVARVL 268

CYP139A1_2590093450_Mycob 199 RPDDHMLTTLISGCSEEGTTLSDNEIRDSIVSLITAGYETTSGALAWAIYALLTVPGTWESAASEVARVL 268

CYP139A1_2590207004_Mycob 199 RPDDHMLTTLISGCSEEGTTLSDNEIRDSIVSLITAGYETTSGALAWAIYALLTVPGTWESAASEVARVL 268

CYP139A1_2590276448_Mycob 199 RPDDHMLTTLISGCSEEGTTLSDNEIRDSIVSLITAGYETTSGALAWAIYALLTVPGTWESAASEVARVL 268

CYP139A1_2575455196_Mycob 199 RPDDHMLTTLISGCSEEGTTLSDNEIRDSIVSLITAGYETTSGALAWAIYALLTVPGTWESAASEVARVL 268

CYP139A1_2575920420_Mycob 199 RPDDHMLTTLISGCSEEGTTLSDNEIRDSIVSLITAGYETTSGALAWAIYALLTVPGTWESAASEVARVL 268

CYP139A1_2575998368_Mycob 199 RPDDHMLTTLISGCSEEGTTLSDNEIRDSIVSLITAGYETTSGALAWAIYALLTVPGTWESAASEVARVL 268

CYP139A1_2576583109_Mycob 199 RPDDHMLTTLISGCSEEGTTLSDNEIRDSIVSLITAGYETTSGALAWAIYALLTVPGTWESAASEVARVL 268

CYP139A1_2576886343_Mycob 199 RPDDHMLTTLISGCSEEGTTLSDNEIRDSIVSLITAGYETTSGALAWAIYALLTVPGTWESAASEVARVL 268

CYP139A1_2576986616_Mycob 199 RPDDHMLTTLISGCSEEGTTLSDNEIRDSIVSLITAGYETTSGALAWAIYALLTVPGTWESAASEVARVL 268

CYP139A1_2577256179_Mycob 199 RPDDHMLTTLISGCSEEGTTLSDNEIRDSIVSLITAGYETTSGALAWAIYALLTVPGTWESAASEVARVL 268

CYP139A1_2577876445_Mycob 199 RPDDHMLTTLISGCSEEGTTLSDNEIRDSIVSLITAGYETTSGALAWAIYALLTVPGTWESAASEVARVL 268

CYP139A1_2579798813_Mycob 199 RPDDHMLTTLISGCSEEGTTLSDNEIRDSIVSLITAGYETTSGALAWAIYALLTVPGTWESAASEVARVL 268

CYP139A1_2584715344_Mycob 199 RPDDHMLTTLISGCSEEGTTLSDNEIRDSIVSLITAGYETTSGALAWAIYALLTVPGTWESAASEVARVL 268

CYP139A1_2588659602_Mycob 199 RPDDHMLTTLISGCSEEGTTLSDNEIRDSIVSLITAGYETTSGALAWAIYALLTVPGTWESAASEVARVL 268

CYP139A1_2589089021_Mycob 199 RPDDHMLTTLISGCSEEGTTLSDNEIRDSIVSLITAGYETTSGALAWAIYALLTVPGTWESAASEVARVL 268

CYP139A1_2589093094_Mycob 199 RPDDHMLTTLISGCSEEGTTLSDNEIRDSIVSLITAGYETTSGALAWAIYALLTVPGTWESAASEVARVL 268

CYP139A1_2589535085_Mycob 199 RPDDHMLTTLISGCSEEGTTLSDNEIRDSIVSLITAGYETTSGALAWAIYALLTVPGTWESAASEVARVL 268

CYP139A1_2589663797_Mycob 199 RPDDHMLTTLISGCSEEGTTLSDNEIRDSIVSLITAGYETTSGALAWAIYALLTVPGTWESAASEVARVL 268

CYP139A1_2589671952_Mycob 199 RPDDHMLTTLISGCSEEGTTLSDNEIRDSIVSLITAGYETTSGALAWAIYALLTVPGTWESAASEVARVL 268

CYP139A1_2589719807_Mycob 199 RPDDHMLTTLISGCSEEGTTLSDNEIRDSIVSLITAGYETTSGALAWAIYALLTVPGTWESAASEVARVL 268

CYP139A1_2592246876_Mycob 199 RPDDHMLTTLISGCSEEGTTLSDNEIRDSIVSLITAGYETTSGALAWAIYALLTVPGTWESAASEVARVL 268

CYP139A1_2592381635_Mycob 199 RPDDHMLTTLISGCSEEGTTLSDNEIRDSIVSLITAGYETTSGALAWAIYALLTVPGTWESAASEVARVL 268

CYP139A1_2592410032_Mycob 199 RPDDHMLTTLISGCSEEGTTLSDNEIRDSIVSLITAGYETTSGALAWAIYALLTVPGTWESAASEVARVL 268

CYP139A1_2592414205_Mycob 199 RPDDHMLTTLISGCSEEGTTLSDNEIRDSIVSLITAGYETTSGALAWAIYALLTVPGTWESAASEVARVL 268

CYP139A1_2592566477_Mycob 199 RPDDHMLTTLISGCSEEGTTLSDNEIRDSIVSLITAGYETTSGALAWAIYALLTVPGTWESAASEVARVL 268

CYP139A1_2575647889_Mycob 199 RPDDHMLTTLISGCSEEGTTLSDNEIRDSIVSLITAGYETTSGALAWAIYALLTVPGTWESAASEVARVL 268

CYP139A1_2577429481_Mycob 199 RPDDHMLTTLISGCSEEGTTLSDNEIRDSIVSLITAGYETTSGALAWAIYALLTVPGTWESAASEVARVL 268

CYP139A1_2578084434_Mycob 199 RPDDHMLTTLISGCSEEGTTLSDNEIRDSIVSLITAGYETTSGALAWAIYALLTVPGTWESAASEVARVL 268

CYP139A1_2580301723_Mycob 199 RPDDHMLTTLISGCSEEGTTLSDNEIRDSIVSLITAGYETTSGALAWAIYALLTVPGTWESAASEVARVL 268

CYP139A1_2581901399_Mycob 199 RPDDHMLTTLISGCSEEGTTLSDNEIRDSIVSLITAGYETTSGALAWAIYALLTVPGTWESAASEVARVL 268

CYP139A1_2584689961_Mycob 199 RPDDHMLTTLISGCSEEGTTLSDNEIRDSIVSLITAGYETTSGALAWAIYALLTVPGTWESAASEVARVL 268

CYP139A1_2584748012_Mycob 199 RPDDHMLTTLISGCSEEGTTLSDNEIRDSIVSLITAGYETTSGALAWAIYALLTVPGTWESAASEVARVL 268

CYP139A1_2584878425_Mycob 199 RPDDHMLTTLISGCSEEGTTLSDNEIRDSIVSLITAGYETTSGALAWAIYALLTVPGTWESAASEVARVL 268

CYP139A1_2584990570_Mycob 199 RPDDHMLTTLISGCSEEGTTLSDNEIRDSIVSLITAGYETTSGALAWAIYALLTVPGTWESAASEVARVL 268

CYP139A1_2590014899_Mycob 199 RPDDHMLTTLISGCSEEGTTLSDNEIRDSIVSLITAGYETTSGALAWAIYALLTVPGTWESAASEVARVL 268

CYP139A1_2590313607_Mycob 199 RPDDHMLTTLISGCSEEGTTLSDNEIRDSIVSLITAGYETTSGALAWAIYALLTVPGTWESAASEVARVL 268

CYP139A1_2590563570_Mycob 199 RPDDHMLTTLISGCSEEGTTLSDNEIRDSIVSLITAGYETTSGALAWAIYALLTVPGTWESAASEVARVL 268

CYP139A1_2574682482_Mycob 199 RPDDHMLTTLISGCSEEGTTLSDNEIRDSIVSLITAGYETTSGALAWAIYALLTVPGTWESAASEVARVL 268

CYP139A1_2574937709_Mycob 199 RPDDHMLTTLISGCSEEGTTLSDNEIRDSIVSLITAGYETTSGALAWAIYALLTVPGTWESAASEVARVL 268

CYP139A1_2575051511_Mycob 199 RPDDHMLTTLISGCSEEGTTLSDNEIRDSIVSLITAGYETTSGALAWAIYALLTVPGTWESAASEVARVL 268

CYP139A1_2575142685_Mycob 199 RPDDHMLTTLISGCSEEGTTLSDNEIRDSIVSLITAGYETTSGALAWAIYALLTVPGTWESAASEVARVL 268

CYP139A1_2575538950_Mycob 199 RPDDHMLTTLISGCSEEGTTLSDNEIRDSIVSLITAGYETTSGALAWAIYALLTVPGTWESAASEVARVL 268

CYP139A1_2575858812_Mycob 199 RPDDHMLTTLISGCSEEGTTLSDNEIRDSIVSLITAGYETTSGALAWAIYALLTVPGTWESAASEVARVL 268

CYP139A1_2576059958_Mycob 199 RPDDHMLTTLISGCSEEGTTLSDNEIRDSIVSLITAGYETTSGALAWAIYALLTVPGTWESAASEVARVL 268

CYP139A1_2576471218_Mycob 199 RPDDHMLTTLISGCSEEGTTLSDNEIRDSIVSLITAGYETTSGALAWAIYALLTVPGTWESAASEVARVL 268

CYP139A1_2576497080_Mycob 199 RPDDHMLTTLISGCSEEGTTLSDNEIRDSIVSLITAGYETTSGALAWAIYALLTVPGTWESAASEVARVL 268

CYP139A1_2577038023_Mycob 199 RPDDHMLTTLISGCSEEGTTLSDNEIRDSIVSLITAGYETTSGALAWAIYALLTVPGTWESAASEVARVL 268

CYP139A1_2577203844_Mycob 199 RPDDHMLTTLISGCSEEGTTLSDNEIRDSIVSLITAGYETTSGALAWAIYALLTVPGTWESAASEVARVL 268

CYP139A1_2577322951_Mycob 199 RPDDHMLTTLISGCSEEGTTLSDNEIRDSIVSLITAGYETTSGALAWAIYALLTVPGTWESAASEVARVL 268

CYP139A1_2577422627_Mycob 199 RPDDHMLTTLISGCSEEGTTLSDNEIRDSIVSLITAGYETTSGALAWAIYALLTVPGTWESAASEVARVL 268

CYP139A1_2577551091_Mycob 199 RPDDHMLTTLISGCSEEGTTLSDNEIRDSIVSLITAGYETTSGALAWAIYALLTVPGTWESAASEVARVL 268

CYP139A1_2584755863_Mycob 199 RPDDHMLTTLISGCSEEGTTLSDNEIRDSIVSLITAGYETTSGALAWAIYALLTVPGTWESAASEVARVL 268

CYP139A1_2589117553_Mycob 199 RPDDHMLTTLISGCSEEGTTLSDNEIRDSIVSLITAGYETTSGALAWAIYALLTVPGTWESAASEVARVL 268

CYP139A1_2589121735_Mycob 199 RPDDHMLTTLISGCSEEGTTLSDNEIRDSIVSLITAGYETTSGALAWAIYALLTVPGTWESAASEVARVL 268

CYP139A1_2589506393_Mycob 199 RPDDHMLTTLISGCSEEGTTLSDNEIRDSIVSLITAGYETTSGALAWAIYALLTVPGTWESAASEVARVL 268

CYP139A1_2590044637_Mycob 199 RPDDHMLTTLISGCSEEGTTLSDNEIRDSIVSLITAGYETTSGALAWAIYALLTVPGTWESAASEVARVL 268

CYP139A1_2590137275_Mycob 199 RPDDHMLTTLISGCSEEGTTLSDNEIRDSIVSLITAGYETTSGALAWAIYALLTVPGTWESAASEVARVL 268

CYP139A1_2590345757_Mycob 199 RPDDHMLTTLISGCSEEGTTLSDNEIRDSIVSLITAGYETTSGALAWAIYALLTVPGTWESAASEVARVL 268

CYP139A1_2592275464_Mycob 199 RPDDHMLTTLISGCSEEGTTLSDNEIRDSIVSLITAGYETTSGALAWAIYALLTVPGTWESAASEVARVL 268

CYP139A1_2575515927_Mycob 199 RPDDHMLTTLISGCSEEGTTLSDNEIRDSIVSLITAGYETTSGALAWAIYALLTVPGTWESAASEVARVL 268

CYP139A1_2576459372_Mycob 199 RPDDHMLTTLISGCSEEGTTLSDNEIRDSIVSLITAGYETTSGALAWAIYALLTVPGTWESAASEVARVL 268

CYP139A1_2576522568_Mycob 199 RPDDHMLTTLISGCSEEGTTLSDNEIRDSIVSLITAGYETTSGALAWAIYALLTVPGTWESAASEVARVL 268

CYP139A1_2576559538_Mycob 199 RPDDHMLTTLISGCSEEGTTLSDNEIRDSIVSLITAGYETTSGALAWAIYALLTVPGTWESAASEVARVL 268

CYP139A1_2577302440_Mycob 199 RPDDHMLTTLISGCSEEGTTLSDNEIRDSIVSLITAGYETTSGALAWAIYALLTVPGTWESAASEVARVL 268

CYP139A1_2577448440_Mycob 199 RPDDHMLTTLISGCSEEGTTLSDNEIRDSIVSLITAGYETTSGALAWAIYALLTVPGTWESAASEVARVL 268

CYP139A1_2577462548_Mycob 199 RPDDHMLTTLISGCSEEGTTLSDNEIRDSIVSLITAGYETTSGALAWAIYALLTVPGTWESAASEVARVL 268

CYP139A1_2584678567_Mycob 199 RPDDHMLTTLISGCSEEGTTLSDNEIRDSIVSLITAGYETTSGALAWAIYALLTVPGTWESAASEVARVL 268

CYP139A1_2584763258_Mycob 199 RPDDHMLTTLISGCSEEGTTLSDNEIRDSIVSLITAGYETTSGALAWAIYALLTVPGTWESAASEVARVL 268

CYP139A1_2584949783_Mycob 199 RPDDHMLTTLISGCSEEGTTLSDNEIRDSIVSLITAGYETTSGALAWAIYALLTVPGTWESAASEVARVL 268

CYP139A1_2589146274_Mycob 199 RPDDHMLTTLISGCSEEGTTLSDNEIRDSIVSLITAGYETTSGALAWAIYALLTVPGTWESAASEVARVL 268

CYP139A1_2590089378_Mycob 199 RPDDHMLTTLISGCSEEGTTLSDNEIRDSIVSLITAGYETTSGALAWAIYALLTVPGTWESAASEVARVL 268

CYP139A1_2590288661_Mycob 199 RPDDHMLTTLISGCSEEGTTLSDNEIRDSIVSLITAGYETTSGALAWAIYALLTVPGTWESAASEVARVL 268

CYP139A1_2590513765_Mycob 199 RPDDHMLTTLISGCSEEGTTLSDNEIRDSIVSLITAGYETTSGALAWAIYALLTVPGTWESAASEVARVL 268

CYP139A1_643045086_Mycoba 199 RPDDHMLTTLISGCSEEGTTLSDNEIRDSIVSLITAGYETTSGALAWAIYALLTVPGTWESAASEVARVL 268

CYP139A1_646014426_Mycoba 199 RPDDHMLTTLISGCSEEGTTLSDNEIRDSIVSLITAGYETTSGALAWAIYALLTVPGTWESAASEVARVL 268

CYP139A1_2574985446_Mycob 199 RPDDHMLTTLISGCSEEGTTLSDNEIRDSIVSLITAGYETTSGALAWAIYALLTVPGTWESAASEVARVL 268

CYP139A1_2575103505_Mycob 199 RPDDHMLTTLISGCSEEGTTLSDNEIRDSIVSLITAGYETTSGALAWAIYALLTVPGTWESAASEVARVL 268

CYP139A1_2575806159_Mycob 199 RPDDHMLTTLISGCSEEGTTLSDNEIRDSIVSLITAGYETTSGALAWAIYALLTVPGTWESAASEVARVL 268

CYP139A1_2576156796_Mycob 199 RPDDHMLTTLISGCSEEGTTLSDNEIRDSIVSLITAGYETTSGALAWAIYALLTVPGTWESAASEVARVL 268

CYP139A1_2576936333_Mycob 199 RPDDHMLTTLISGCSEEGTTLSDNEIRDSIVSLITAGYETTSGALAWAIYALLTVPGTWESAASEVARVL 268

CYP139A1_2577030827_Mycob 199 RPDDHMLTTLISGCSEEGTTLSDNEIRDSIVSLITAGYETTSGALAWAIYALLTVPGTWESAASEVARVL 268

CYP139A1_2577381729_Mycob 199 RPDDHMLTTLISGCSEEGTTLSDNEIRDSIVSLITAGYETTSGALAWAIYALLTVPGTWESAASEVARVL 268

CYP139A1_2584698776_Mycob 199 RPDDHMLTTLISGCSEEGTTLSDNEIRDSIVSLITAGYETTSGALAWAIYALLTVPGTWESAASEVARVL 268

CYP139A1_2584825730_Mycob 199 RPDDHMLTTLISGCSEEGTTLSDNEIRDSIVSLITAGYETTSGALAWAIYALLTVPGTWESAASEVARVL 268

CYP139A1_2584885951_Mycob 199 RPDDHMLTTLISGCSEEGTTLSDNEIRDSIVSLITAGYETTSGALAWAIYALLTVPGTWESAASEVARVL 268

CYP139A1_2584902930_Mycob 199 RPDDHMLTTLISGCSEEGTTLSDNEIRDSIVSLITAGYETTSGALAWAIYALLTVPGTWESAASEVARVL 268

CYP139A1_2588995218_Mycob 199 RPDDHMLTTLISGCSEEGTTLSDNEIRDSIVSLITAGYETTSGALAWAIYALLTVPGTWESAASEVARVL 268

CYP139A1_2589139119_Mycob 199 RPDDHMLTTLISGCSEEGTTLSDNEIRDSIVSLITAGYETTSGALAWAIYALLTVPGTWESAASEVARVL 268

CYP139A1_2592238712_Mycob 199 RPDDHMLTTLISGCSEEGTTLSDNEIRDSIVSLITAGYETTSGALAWAIYALLTVPGTWESAASEVARVL 268

CYP139A1_2592261699_Mycob 199 RPDDHMLTTLISGCSEEGTTLSDNEIRDSIVSLITAGYETTSGALAWAIYALLTVPGTWESAASEVARVL 268

CYP139A1_2592393538_Mycob 199 RPDDHMLTTLISGCSEEGTTLSDNEIRDSIVSLITAGYETTSGALAWAIYALLTVPGTWESAASEVARVL 268

CYP139A1_2592397895_Mycob 199 RPDDHMLTTLISGCSEEGTTLSDNEIRDSIVSLITAGYETTSGALAWAIYALLTVPGTWESAASEVARVL 268

CYP139A1_2592549349_Mycob 199 RPDDHMLTTLISGCSEEGTTLSDNEIRDSIVSLITAGYETTSGALAWAIYALLTVPGTWESAASEVARVL 268

CYP139A1_648464907_Mycoba 199 RPDDHMLTTLISGCSEEGTTLSDNEIRDSIVSLITAGYETTSGALAWAIYALLTVPGTWESAASEVARVL 268

CYP139A1_648481186_Mycoba 199 RPDDHMLTTLISGCSEEGTTLSDNEIRDSIVSLITAGYETTSGALAWAIYALLTVPGTWESAASEVARVL 268

CYP139A1_2574586310_Mycob 199 RPDDHMLTTLISGCSEEGTTLSDNEIRDSIVSLITAGYETTSGALAWAIYALLTVPGTWESAASEVARVL 268

CYP139A1_2574663269_Mycob 199 RPDDHMLTTLISGCSEEGTTLSDNEIRDSIVSLITAGYETTSGALAWAIYALLTVPGTWESAASEVARVL 268

CYP139A1_2574880930_Mycob 199 RPDDHMLTTLISGCSEEGTTLSDNEIRDSIVSLITAGYETTSGALAWAIYALLTVPGTWESAASEVARVL 268

CYP139A1_2574911407_Mycob 199 RPDDHMLTTLISGCSEEGTTLSDNEIRDSIVSLITAGYETTSGALAWAIYALLTVPGTWESAASEVARVL 268

CYP139A1_2575345775_Mycob 199 RPDDHMLTTLISGCSEEGTTLSDNEIRDSIVSLITAGYETTSGALAWAIYALLTVPGTWESAASEVARVL 268

CYP139A1_2575468742_Mycob 199 RPDDHMLTTLISGCSEEGTTLSDNEIRDSIVSLITAGYETTSGALAWAIYALLTVPGTWESAASEVARVL 268

CYP139A1_2576199760_Mycob 199 RPDDHMLTTLISGCSEEGTTLSDNEIRDSIVSLITAGYETTSGALAWAIYALLTVPGTWESAASEVARVL 268

CYP139A1_2577069926_Mycob 199 RPDDHMLTTLISGCSEEGTTLSDNEIRDSIVSLITAGYETTSGALAWAIYALLTVPGTWESAASEVARVL 268

CYP139A1_2577613318_Mycob 199 RPDDHMLTTLISGCSEEGTTLSDNEIRDSIVSLITAGYETTSGALAWAIYALLTVPGTWESAASEVARVL 268

CYP139A1_2577684900_Mycob 199 RPDDHMLTTLISGCSEEGTTLSDNEIRDSIVSLITAGYETTSGALAWAIYALLTVPGTWESAASEVARVL 268

CYP139A1_2582415442_Mycob 199 RPDDHMLTTLISGCSEEGTTLSDNEIRDSIVSLITAGYETTSGALAWAIYALLTVPGTWESAASEVARVL 268

CYP139A1_2584812089_Mycob 199 RPDDHMLTTLISGCSEEGTTLSDNEIRDSIVSLITAGYETTSGALAWAIYALLTVPGTWESAASEVARVL 268

CYP139A1_2590125975_Mycob 199 RPDDHMLTTLISGCSEEGTTLSDNEIRDSIVSLITAGYETTSGALAWAIYALLTVPGTWESAASEVARVL 268

CYP139A1_2590219901_Mycob 199 RPDDHMLTTLISGCSEEGTTLSDNEIRDSIVSLITAGYETTSGALAWAIYALLTVPGTWESAASEVARVL 268

CYP139A1_2590227570_Mycob 199 RPDDHMLTTLISGCSEEGTTLSDNEIRDSIVSLITAGYETTSGALAWAIYALLTVPGTWESAASEVARVL 268

CYP139A1_2590317674_Mycob 199 RPDDHMLTTLISGCSEEGTTLSDNEIRDSIVSLITAGYETTSGALAWAIYALLTVPGTWESAASEVARVL 268

CYP139A1_641814886_Mycoba 199 RPDDHMLTTLISGCSEEGTTLSDNEIRDSIVSLITAGYETTSGALAWAIYALLTVPGTWESAASEVARVL 268

CYP139A1_2574738070_Mycob 199 RPDDHMLTTLISGCSEEGTTLSDNEIRDSIVSLITAGYETTSGALAWAIYALLTVPGTWESAASEVARVL 268

CYP139A1_2575365848_Mycob 199 RPDDHMLTTLISGCSEEGTTLSDNEIRDSIVSLITAGYETTSGALAWAIYALLTVPGTWESAASEVARVL 268

CYP139A1_2575674521_Mycob 199 RPDDHMLTTLISGCSEEGTTLSDNEIRDSIVSLITAGYETTSGALAWAIYALLTVPGTWESAASEVARVL 268

CYP139A1_2575772716_Mycob 199 RPDDHMLTTLISGCSEEGTTLSDNEIRDSIVSLITAGYETTSGALAWAIYALLTVPGTWESAASEVARVL 268

CYP139A1_2576544659_Mycob 199 RPDDHMLTTLISGCSEEGTTLSDNEIRDSIVSLITAGYETTSGALAWAIYALLTVPGTWESAASEVARVL 268

CYP139A1_2577168470_Mycob 199 RPDDHMLTTLISGCSEEGTTLSDNEIRDSIVSLITAGYETTSGALAWAIYALLTVPGTWESAASEVARVL 268

CYP139A1_2577390930_Mycob 199 RPDDHMLTTLISGCSEEGTTLSDNEIRDSIVSLITAGYETTSGALAWAIYALLTVPGTWESAASEVARVL 268

CYP139A1_2577651582_Mycob 199 RPDDHMLTTLISGCSEEGTTLSDNEIRDSIVSLITAGYETTSGALAWAIYALLTVPGTWESAASEVARVL 268

CYP139A1_2578002537_Mycob 199 RPDDHMLTTLISGCSEEGTTLSDNEIRDSIVSLITAGYETTSGALAWAIYALLTVPGTWESAASEVARVL 268

CYP139A1_2578230674_Mycob 199 RPDDHMLTTLISGCSEEGTTLSDNEIRDSIVSLITAGYETTSGALAWAIYALLTVPGTWESAASEVARVL 268

CYP139A1_2579825786_Mycob 199 RPDDHMLTTLISGCSEEGTTLSDNEIRDSIVSLITAGYETTSGALAWAIYALLTVPGTWESAASEVARVL 268

CYP139A1_2584923296_Mycob 199 RPDDHMLTTLISGCSEEGTTLSDNEIRDSIVSLITAGYETTSGALAWAIYALLTVPGTWESAASEVARVL 268

CYP139A1_2584954771_Mycob 199 RPDDHMLTTLISGCSEEGTTLSDNEIRDSIVSLITAGYETTSGALAWAIYALLTVPGTWESAASEVARVL 268

CYP139A1_2588999278_Mycob 199 RPDDHMLTTLISGCSEEGTTLSDNEIRDSIVSLITAGYETTSGALAWAIYALLTVPGTWESAASEVARVL 268

CYP139A1_2589044237_Mycob 199 RPDDHMLTTLISGCSEEGTTLSDNEIRDSIVSLITAGYETTSGALAWAIYALLTVPGTWESAASEVARVL 268

CYP139A1_2589555384_Mycob 199 RPDDHMLTTLISGCSEEGTTLSDNEIRDSIVSLITAGYETTSGALAWAIYALLTVPGTWESAASEVARVL 268

CYP139A1_2589568736_Mycob 199 RPDDHMLTTLISGCSEEGTTLSDNEIRDSIVSLITAGYETTSGALAWAIYALLTVPGTWESAASEVARVL 268

CYP139A1_2589715710_Mycob 199 RPDDHMLTTLISGCSEEGTTLSDNEIRDSIVSLITAGYETTSGALAWAIYALLTVPGTWESAASEVARVL 268

CYP139A1_2592287696_Mycob 199 RPDDHMLTTLISGCSEEGTTLSDNEIRDSIVSLITAGYETTSGALAWAIYALLTVPGTWESAASEVARVL 268

CYP139A1_2592291479_Mycob 199 RPDDHMLTTLISGCSEEGTTLSDNEIRDSIVSLITAGYETTSGALAWAIYALLTVPGTWESAASEVARVL 268

CYP139A1_2592430392_Mycob 199 RPDDHMLTTLISGCSEEGTTLSDNEIRDSIVSLITAGYETTSGALAWAIYALLTVPGTWESAASEVARVL 268

CYP139A1_2574790496_Mycob 199 RPDDHMLTTLISGCSEEGTTLSDNEIRDSIVSLITAGYETTSGALAWAIYALLTVPGTWESAASEVARVL 268

CYP139A1_2574834868_Mycob 199 RPDDHMLTTLISGCSEEGTTLSDNEIRDSIVSLITAGYETTSGALAWAIYALLTVPGTWESAASEVARVL 268

CYP139A1_2576151818_Mycob 199 RPDDHMLTTLISGCSEEGTTLSDNEIRDSIVSLITAGYETTSGALAWAIYALLTVPGTWESAASEVARVL 268

CYP139A1_2578111269_Mycob 199 RPDDHMLTTLISGCSEEGTTLSDNEIRDSIVSLITAGYETTSGALAWAIYALLTVPGTWESAASEVARVL 268

CYP139A1_2583723841_Mycob 199 RPDDHMLTTLISGCSEEGTTLSDNEIRDSIVSLITAGYETTSGALAWAIYALLTVPGTWESAASEVARVL 268

CYP139A1_2584631822_Mycob 199 RPDDHMLTTLISGCSEEGTTLSDNEIRDSIVSLITAGYETTSGALAWAIYALLTVPGTWESAASEVARVL 268

CYP139A1_2584721998_Mycob 199 RPDDHMLTTLISGCSEEGTTLSDNEIRDSIVSLITAGYETTSGALAWAIYALLTVPGTWESAASEVARVL 268

CYP139A1_2584846770_Mycob 199 RPDDHMLTTLISGCSEEGTTLSDNEIRDSIVSLITAGYETTSGALAWAIYALLTVPGTWESAASEVARVL 268

CYP139A1_2588591267_Mycob 199 RPDDHMLTTLISGCSEEGTTLSDNEIRDSIVSLITAGYETTSGALAWAIYALLTVPGTWESAASEVARVL 268

CYP139A1_2590007914_Mycob 199 RPDDHMLTTLISGCSEEGTTLSDNEIRDSIVSLITAGYETTSGALAWAIYALLTVPGTWESAASEVARVL 268

CYP139A1_2590057066_Mycob 199 RPDDHMLTTLISGCSEEGTTLSDNEIRDSIVSLITAGYETTSGALAWAIYALLTVPGTWESAASEVARVL 268

CYP139A1_2590061112_Mycob 199 RPDDHMLTTLISGCSEEGTTLSDNEIRDSIVSLITAGYETTSGALAWAIYALLTVPGTWESAASEVARVL 268

CYP139A1_2590252317_Mycob 199 RPDDHMLTTLISGCSEEGTTLSDNEIRDSIVSLITAGYETTSGALAWAIYALLTVPGTWESAASEVARVL 268

CYP139A1_2590543931_Mycob 199 RPDDHMLTTLISGCSEEGTTLSDNEIRDSIVSLITAGYETTSGALAWAIYALLTVPGTWESAASEVARVL 268

CYP139A1_2574560432_Mycob 199 RPDDHMLTTLISGCSEEGTTLSDNEIRDSIVSLITAGYETTSGALAWAIYALLTVPGTWESAASEVARVL 268

CYP139A1_2575495357_Mycob 199 RPDDHMLTTLISGCSEEGTTLSDNEIRDSIVSLITAGYETTSGALAWAIYALLTVPGTWESAASEVARVL 268

CYP139A1_2575638157_Mycob 199 RPDDHMLTTLISGCSEEGTTLSDNEIRDSIVSLITAGYETTSGALAWAIYALLTVPGTWESAASEVARVL 268

CYP139A1_2575697737_Mycob 199 RPDDHMLTTLISGCSEEGTTLSDNEIRDSIVSLITAGYETTSGALAWAIYALLTVPGTWESAASEVARVL 268

CYP139A1_2575988382_Mycob 199 RPDDHMLTTLISGCSEEGTTLSDNEIRDSIVSLITAGYETTSGALAWAIYALLTVPGTWESAASEVARVL 268

CYP139A1_2576070324_Mycob 199 RPDDHMLTTLISGCSEEGTTLSDNEIRDSIVSLITAGYETTSGALAWAIYALLTVPGTWESAASEVARVL 268

CYP139A1_2576613004_Mycob 199 RPDDHMLTTLISGCSEEGTTLSDNEIRDSIVSLITAGYETTSGALAWAIYALLTVPGTWESAASEVARVL 268

CYP139A1_2577091164_Mycob 199 RPDDHMLTTLISGCSEEGTTLSDNEIRDSIVSLITAGYETTSGALAWAIYALLTVPGTWESAASEVARVL 268

CYP139A1_2577569748_Mycob 199 RPDDHMLTTLISGCSEEGTTLSDNEIRDSIVSLITAGYETTSGALAWAIYALLTVPGTWESAASEVARVL 268

CYP139A1_2577697067_Mycob 199 RPDDHMLTTLISGCSEEGTTLSDNEIRDSIVSLITAGYETTSGALAWAIYALLTVPGTWESAASEVARVL 268

CYP139A1_2584795634_Mycob 199 RPDDHMLTTLISGCSEEGTTLSDNEIRDSIVSLITAGYETTSGALAWAIYALLTVPGTWESAASEVARVL 268

CYP139A1_2589158600_Mycob 199 RPDDHMLTTLISGCSEEGTTLSDNEIRDSIVSLITAGYETTSGALAWAIYALLTVPGTWESAASEVARVL 268

CYP139A1_2589491285_Mycob 199 RPDDHMLTTLISGCSEEGTTLSDNEIRDSIVSLITAGYETTSGALAWAIYALLTVPGTWESAASEVARVL 268

CYP139A1_2589613612_Mycob 199 RPDDHMLTTLISGCSEEGTTLSDNEIRDSIVSLITAGYETTSGALAWAIYALLTVPGTWESAASEVARVL 268

CYP139A1_2589650472_Mycob 199 RPDDHMLTTLISGCSEEGTTLSDNEIRDSIVSLITAGYETTSGALAWAIYALLTVPGTWESAASEVARVL 268

CYP139A1_2589699410_Mycob 199 RPDDHMLTTLISGCSEEGTTLSDNEIRDSIVSLITAGYETTSGALAWAIYALLTVPGTWESAASEVARVL 268

CYP139A1_2590366449_Mycob 199 RPDDHMLTTLISGCSEEGTTLSDNEIRDSIVSLITAGYETTSGALAWAIYALLTVPGTWESAASEVARVL 268

CYP139A1_2592226489_Mycob 199 RPDDHMLTTLISGCSEEGTTLSDNEIRDSIVSLITAGYETTSGALAWAIYALLTVPGTWESAASEVARVL 268

CYP139A1_2592574645_Mycob 199 RPDDHMLTTLISGCSEEGTTLSDNEIRDSIVSLITAGYETTSGALAWAIYALLTVPGTWESAASEVARVL 268

CYP139A1_648473432_Mycoba 199 RPDDHMLTTLISGCSEEGTTLSDNEIRDSIVSLITAGYETTSGALAWAIYALLTVPGTWESAASEVARVL 268

CYP139A1_2574703562_Mycob 199 RPDDHMLTTLISGCSEEGTTLSDNEIRDSIVSLITAGYETTSGALAWAIYALLTVPGTWESAASEVARVL 268

CYP139A1_2575794202_Mycob 199 RPDDHMLTTLISGCSEEGTTLSDNEIRDSIVSLITAGYETTSGALAWAIYALLTVPGTWESAASEVARVL 268

CYP139A1_2576967330_Mycob 199 RPDDHMLTTLISGCSEEGTTLSDNEIRDSIVSLITAGYETTSGALAWAIYALLTVPGTWESAASEVARVL 268

CYP139A1_2577659800_Mycob 199 RPDDHMLTTLISGCSEEGTTLSDNEIRDSIVSLITAGYETTSGALAWAIYALLTVPGTWESAASEVARVL 268

CYP139A1_2577918576_Mycob 199 RPDDHMLTTLISGCSEEGTTLSDNEIRDSIVSLITAGYETTSGALAWAIYALLTVPGTWESAASEVARVL 268

CYP139A1_2578033540_Mycob 199 RPDDHMLTTLISGCSEEGTTLSDNEIRDSIVSLITAGYETTSGALAWAIYALLTVPGTWESAASEVARVL 268

CYP139A1_2581869776_Mycob 199 RPDDHMLTTLISGCSEEGTTLSDNEIRDSIVSLITAGYETTSGALAWAIYALLTVPGTWESAASEVARVL 268

CYP139A1_2583745288_Mycob 199 RPDDHMLTTLISGCSEEGTTLSDNEIRDSIVSLITAGYETTSGALAWAIYALLTVPGTWESAASEVARVL 268

CYP139A1_2590086658_Mycob 199 RPDDHMLTTLISGCSEEGTTLSDNEIRDSIVSLITAGYETTSGALAWAIYALLTVPGTWESAASEVARVL 268

CYP139A1_2590104693_Mycob 199 RPDDHMLTTLISGCSEEGTTLSDNEIRDSIVSLITAGYETTSGALAWAIYALLTVPGTWESAASEVARVL 268

CYP139A1_2590187116_Mycob 199 RPDDHMLTTLISGCSEEGTTLSDNEIRDSIVSLITAGYETTSGALAWAIYALLTVPGTWESAASEVARVL 268

CYP139A1_2590382529_Mycob 199 RPDDHMLTTLISGCSEEGTTLSDNEIRDSIVSLITAGYETTSGALAWAIYALLTVPGTWESAASEVARVL 268

CYP139A1_2590510255_Mycob 199 RPDDHMLTTLISGCSEEGTTLSDNEIRDSIVSLITAGYETTSGALAWAIYALLTVPGTWESAASEVARVL 268

CYP139A1_2574635031_Mycob 199 RPDDHMLTTLISGCSEEGTTLSDNEIRDSIVSLITAGYETTSGALAWAIYALLTVPGTWESAASEVARVL 268

CYP139A1_2576207497_Mycob 199 RPDDHMLTTLISGCSEEGTTLSDNEIRDSIVSLITAGYETTSGALAWAIYALLTVPGTWESAASEVARVL 268

CYP139A1_2576443603_Mycob 199 RPDDHMLTTLISGCSEEGTTLSDNEIRDSIVSLITAGYETTSGALAWAIYALLTVPGTWESAASEVARVL 268

CYP139A1_2577241442_Mycob 199 RPDDHMLTTLISGCSEEGTTLSDNEIRDSIVSLITAGYETTSGALAWAIYALLTVPGTWESAASEVARVL 268

CYP139A1_2578177805_Mycob 199 RPDDHMLTTLISGCSEEGTTLSDNEIRDSIVSLITAGYETTSGALAWAIYALLTVPGTWESAASEVARVL 268

CYP139A1_2578195506_Mycob 199 RPDDHMLTTLISGCSEEGTTLSDNEIRDSIVSLITAGYETTSGALAWAIYALLTVPGTWESAASEVARVL 268

CYP139A1_2578246558_Mycob 199 RPDDHMLTTLISGCSEEGTTLSDNEIRDSIVSLITAGYETTSGALAWAIYALLTVPGTWESAASEVARVL 268

CYP139A1_2584652546_Mycob 199 RPDDHMLTTLISGCSEEGTTLSDNEIRDSIVSLITAGYETTSGALAWAIYALLTVPGTWESAASEVARVL 268

CYP139A1_2584737038_Mycob 199 RPDDHMLTTLISGCSEEGTTLSDNEIRDSIVSLITAGYETTSGALAWAIYALLTVPGTWESAASEVARVL 268

CYP139A1_2584918375_Mycob 199 RPDDHMLTTLISGCSEEGTTLSDNEIRDSIVSLITAGYETTSGALAWAIYALLTVPGTWESAASEVARVL 268

CYP139A1_2584974532_Mycob 199 RPDDHMLTTLISGCSEEGTTLSDNEIRDSIVSLITAGYETTSGALAWAIYALLTVPGTWESAASEVARVL 268

CYP139A1_2588987052_Mycob 199 RPDDHMLTTLISGCSEEGTTLSDNEIRDSIVSLITAGYETTSGALAWAIYALLTVPGTWESAASEVARVL 268

CYP139A1_2589098383_Mycob 199 RPDDHMLTTLISGCSEEGTTLSDNEIRDSIVSLITAGYETTSGALAWAIYALLTVPGTWESAASEVARVL 268

CYP139A1_2589486069_Mycob 199 RPDDHMLTTLISGCSEEGTTLSDNEIRDSIVSLITAGYETTSGALAWAIYALLTVPGTWESAASEVARVL 268

CYP139A1_2589522796_Mycob 199 RPDDHMLTTLISGCSEEGTTLSDNEIRDSIVSLITAGYETTSGALAWAIYALLTVPGTWESAASEVARVL 268

CYP139A1_2589616566_Mycob 199 RPDDHMLTTLISGCSEEGTTLSDNEIRDSIVSLITAGYETTSGALAWAIYALLTVPGTWESAASEVARVL 268

CYP139A1_2592250930_Mycob 199 RPDDHMLTTLISGCSEEGTTLSDNEIRDSIVSLITAGYETTSGALAWAIYALLTVPGTWESAASEVARVL 268

CYP139A1_2592303254_Mycob 199 RPDDHMLTTLISGCSEEGTTLSDNEIRDSIVSLITAGYETTSGALAWAIYALLTVPGTWESAASEVARVL 268

CYP139A1_2592308116_Mycob 199 RPDDHMLTTLISGCSEEGTTLSDNEIRDSIVSLITAGYETTSGALAWAIYALLTVPGTWESAASEVARVL 268

CYP139A1_2592385592_Mycob 199 RPDDHMLTTLISGCSEEGTTLSDNEIRDSIVSLITAGYETTSGALAWAIYALLTVPGTWESAASEVARVL 268

CYP139A1_2592417392_Mycob 199 RPDDHMLTTLISGCSEEGTTLSDNEIRDSIVSLITAGYETTSGALAWAIYALLTVPGTWESAASEVARVL 268

CYP139A1_643049582_Mycoba 199 RPDDHMLTTLISGCSEEGTTLSDNEIRDSIVSLITAGYETTSGALAWAIYALLTVPGTWESAASEVARVL 268

CYP139A1_648460473_Mycoba 199 RPDDHMLTTLISGCSEEGTTLSDNEIRDSIVSLITAGYETTSGALAWAIYALLTVPGTWESAASEVARVL 268

CYP139A1_2574901332_Mycob 199 RPDDHMLTTLISGCSEEGTTLSDNEIRDSIVSLITAGYETTSGALAWAIYALLTVPGTWESAASEVARVL 268

CYP139A1_2575325434_Mycob 199 RPDDHMLTTLISGCSEEGTTLSDNEIRDSIVSLITAGYETTSGALAWAIYALLTVPGTWESAASEVARVL 268

CYP139A1_2576274603_Mycob 199 RPDDHMLTTLISGCSEEGTTLSDNEIRDSIVSLITAGYETTSGALAWAIYALLTVPGTWESAASEVARVL 268

CYP139A1_2583719805_Mycob 199 RPDDHMLTTLISGCSEEGTTLSDNEIRDSIVSLITAGYETTSGALAWAIYALLTVPGTWESAASEVARVL 268

CYP139A1_2590012031_Mycob 199 RPDDHMLTTLISGCSEEGTTLSDNEIRDSIVSLITAGYETTSGALAWAIYALLTVPGTWESAASEVARVL 268

CYP139A_2590028529_Mycoba 199 RPDDHMLTTLISGCSEEGTTLSDNEIRDSIVSLITAGYETTSGALAWAIYALLTVPGTWESAASEVARVL 268

CYP139A1_2590146409_Mycob 199 RPDDHMLTTLISGCSEEGTTLSDNEIRDSIVSLITAGYETTSGALAWAIYALLTVPGTWESAASEVARVL 268

CYP139A1_2590203420_Mycob 199 RPDDHMLTTLISGCSEEGTTLSDNEIRDSIVSLITAGYETTSGALAWAIYALLTVPGTWESAASEVARVL 268

CYP139A1_2590272661_Mycob 199 RPDDHMLTTLISGCSEEGTTLSDNEIRDSIVSLITAGYETTSGALAWAIYALLTVPGTWESAASEVARVL 268

CYP139A1_2590535240_Mycob 199 RPDDHMLTTLISGCSEEGTTLSDNEIRDSIVSLITAGYETTSGALAWAIYALLTVPGTWESAASEVARVL 268

CYP139A1_647090515_Mycoba 199 RPDDHMLTTLISGCSEEGTTLSDNEIRDSIVSLITAGYETTSGALAWAIYALLTVPGTWESAASEVARVL 268

CYP139A1_2512786915_Mycob 199 RPDDHMLTTLISGCSEEGTTLSDNEIRDSIVSLITAGYETTSGALAWAIYALLTVPGTWESAASEVARVL 268

CYP139A1_2575329342_Mycob 199 RPDDHMLTTLISGCSEEGTTLSDNEIRDSIVSLITAGYETTSGALAWAIYALLTVPGTWESAASEVARVL 268

CYP139A1_2575409751_Mycob 199 RPDDHMLTTLISGCSEEGTTLSDNEIRDSIVSLITAGYETTSGALAWAIYALLTVPGTWESAASEVARVL 268

CYP139A1_2575862603_Mycob 199 RPDDHMLTTLISGCSEEGTTLSDNEIRDSIVSLITAGYETTSGALAWAIYALLTVPGTWESAASEVARVL 268

CYP139A1_2576589126_Mycob 199 RPDDHMLTTLISGCSEEGTTLSDNEIRDSIVSLITAGYETTSGALAWAIYALLTVPGTWESAASEVARVL 268

CYP139A1_2576649037_Mycob 199 RPDDHMLTTLISGCSEEGTTLSDNEIRDSIVSLITAGYETTSGALAWAIYALLTVPGTWESAASEVARVL 268

CYP139A1_2576746511_Mycob 199 RPDDHMLTTLISGCSEEGTTLSDNEIRDSIVSLITAGYETTSGALAWAIYALLTVPGTWESAASEVARVL 268

CYP139A1_2577129296_Mycob 199 RPDDHMLTTLISGCSEEGTTLSDNEIRDSIVSLITAGYETTSGALAWAIYALLTVPGTWESAASEVARVL 268

CYP139A1_2578200424_Mycob 199 RPDDHMLTTLISGCSEEGTTLSDNEIRDSIVSLITAGYETTSGALAWAIYALLTVPGTWESAASEVARVL 268

CYP139A1_2584666311_Mycob 199 RPDDHMLTTLISGCSEEGTTLSDNEIRDSIVSLITAGYETTSGALAWAIYALLTVPGTWESAASEVARVL 268

CYP139A1_2584743940_Mycob 199 RPDDHMLTTLISGCSEEGTTLSDNEIRDSIVSLITAGYETTSGALAWAIYALLTVPGTWESAASEVARVL 268

CYP139A1_2584781205_Mycob 199 RPDDHMLTTLISGCSEEGTTLSDNEIRDSIVSLITAGYETTSGALAWAIYALLTVPGTWESAASEVARVL 268

CYP139A1_2584833644_Mycob 199 RPDDHMLTTLISGCSEEGTTLSDNEIRDSIVSLITAGYETTSGALAWAIYALLTVPGTWESAASEVARVL 268

CYP139A1_2589073023_Mycob 199 RPDDHMLTTLISGCSEEGTTLSDNEIRDSIVSLITAGYETTSGALAWAIYALLTVPGTWESAASEVARVL 268

CYP139A1_2589509569_Mycob 199 RPDDHMLTTLISGCSEEGTTLSDNEIRDSIVSLITAGYETTSGALAWAIYALLTVPGTWESAASEVARVL 268

CYP139A1_2589551314_Mycob 199 RPDDHMLTTLISGCSEEGTTLSDNEIRDSIVSLITAGYETTSGALAWAIYALLTVPGTWESAASEVARVL 268

CYP139A1_2589580598_Mycob 199 RPDDHMLTTLISGCSEEGTTLSDNEIRDSIVSLITAGYETTSGALAWAIYALLTVPGTWESAASEVARVL 268

CYP139A1_2589629003_Mycob 199 RPDDHMLTTLISGCSEEGTTLSDNEIRDSIVSLITAGYETTSGALAWAIYALLTVPGTWESAASEVARVL 268

CYP139A1_2589676017_Mycob 199 RPDDHMLTTLISGCSEEGTTLSDNEIRDSIVSLITAGYETTSGALAWAIYALLTVPGTWESAASEVARVL 268

CYP139A1_2592332604_Mycob 199 RPDDHMLTTLISGCSEEGTTLSDNEIRDSIVSLITAGYETTSGALAWAIYALLTVPGTWESAASEVARVL 268

CYP139A1_2592542016_Mycob 199 RPDDHMLTTLISGCSEEGTTLSDNEIRDSIVSLITAGYETTSGALAWAIYALLTVPGTWESAASEVARVL 268

CYP139A1_2592562396_Mycob 199 RPDDHMLTTLISGCSEEGTTLSDNEIRDSIVSLITAGYETTSGALAWAIYALLTVPGTWESAASEVARVL 268

CYP139A1_2541578033_Mycob 199 RPDDHMLTTLISGCSEEGTTLSDNEIRDSIVSLITAGYETTSGALAWAIYALLTVPGTWESAASEVARVL 268

CYP139A1_2574761421_Mycob 199 RPDDHMLTTLISGCSEEGTTLSDNEIRDSIVSLITAGYETTSGALAWAIYALLTVPGTWESAASEVARVL 268

CYP139A1_2575611823_Mycob 199 RPDDHMLTTLISGCSEEGTTLSDNEIRDSIVSLITAGYETTSGALAWAIYALLTVPGTWESAASEVARVL 268

CYP139A1_2576564801_Mycob 199 RPDDHMLTTLISGCSEEGTTLSDNEIRDSIVSLITAGYETTSGALAWAIYALLTVPGTWESAASEVARVL 268

CYP139A1_2577821955_Mycob 199 RPDDHMLTTLISGCSEEGTTLSDNEIRDSIVSLITAGYETTSGALAWAIYALLTVPGTWESAASEVARVL 268

CYP139A1_2578086064_Mycob 199 RPDDHMLTTLISGCSEEGTTLSDNEIRDSIVSLITAGYETTSGALAWAIYALLTVPGTWESAASEVARVL 268

CYP139A1_2580669105_Mycob 199 RPDDHMLTTLISGCSEEGTTLSDNEIRDSIVSLITAGYETTSGALAWAIYALLTVPGTWESAASEVARVL 268

CYP139A1_2583740670_Mycob 199 RPDDHMLTTLISGCSEEGTTLSDNEIRDSIVSLITAGYETTSGALAWAIYALLTVPGTWESAASEVARVL 268

CYP139A1_2584831832_Mycob 199 RPDDHMLTTLISGCSEEGTTLSDNEIRDSIVSLITAGYETTSGALAWAIYALLTVPGTWESAASEVARVL 268

CYP139A1_2584875064_Mycob 199 RPDDHMLTTLISGCSEEGTTLSDNEIRDSIVSLITAGYETTSGALAWAIYALLTVPGTWESAASEVARVL 268

CYP139A1_2590158810_Mycob 199 RPDDHMLTTLISGCSEEGTTLSDNEIRDSIVSLITAGYETTSGALAWAIYALLTVPGTWESAASEVARVL 268

CYP139A1_2590240089_Mycob 199 RPDDHMLTTLISGCSEEGTTLSDNEIRDSIVSLITAGYETTSGALAWAIYALLTVPGTWESAASEVARVL 268

CYP139A1_2590350138_Mycob 199 RPDDHMLTTLISGCSEEGTTLSDNEIRDSIVSLITAGYETTSGALAWAIYALLTVPGTWESAASEVARVL 268

CYP139A1_2590493738_Mycob 199 RPDDHMLTTLISGCSEEGTTLSDNEIRDSIVSLITAGYETTSGALAWAIYALLTVPGTWESAASEVARVL 268

CYP139A1_2590554696_Mycob 199 RPDDHMLTTLISGCSEEGTTLSDNEIRDSIVSLITAGYETTSGALAWAIYALLTVPGTWESAASEVARVL 268

CYP139A1_2576084283_Mycob 199 RPDDHMLTTLISGCSEEGTTLSDNEIRDSIVSLITAGYETTSGALAWAIYALLTVPGTWESAASEVARVL 268

CYP139A1_2576366402_Mycob 199 RPDDHMLTTLISGCSEEGTTLSDNEIRDSIVSLITAGYETTSGALAWAIYALLTVPGTWESAASEVARVL 268

CYP139A1_2576513411_Mycob 199 RPDDHMLTTLISGCSEEGTTLSDNEIRDSIVSLITAGYETTSGALAWAIYALLTVPGTWESAASEVARVL 268

CYP139A1_2576671439_Mycob 199 RPDDHMLTTLISGCSEEGTTLSDNEIRDSIVSLITAGYETTSGALAWAIYALLTVPGTWESAASEVARVL 268

CYP139A1_2577792721_Mycob 199 RPDDHMLTTLISGCSEEGTTLSDNEIRDSIVSLITAGYETTSGALAWAIYALLTVPGTWESAASEVARVL 268

CYP139A1_2577796885_Mycob 199 RPDDHMLTTLISGCSEEGTTLSDNEIRDSIVSLITAGYETTSGALAWAIYALLTVPGTWESAASEVARVL 268

CYP139A1_2584686721_Mycob 199 RPDDHMLTTLISGCSEEGTTLSDNEIRDSIVSLITAGYETTSGALAWAIYALLTVPGTWESAASEVARVL 268

CYP139A1_2584752944_Mycob 199 RPDDHMLTTLISGCSEEGTTLSDNEIRDSIVSLITAGYETTSGALAWAIYALLTVPGTWESAASEVARVL 268

CYP139A1_2584808933_Mycob 199 RPDDHMLTTLISGCSEEGTTLSDNEIRDSIVSLITAGYETTSGALAWAIYALLTVPGTWESAASEVARVL 268

CYP139A1_2589134136_Mycob 199 RPDDHMLTTLISGCSEEGTTLSDNEIRDSIVSLITAGYETTSGALAWAIYALLTVPGTWESAASEVARVL 268

CYP139A1_2589539130_Mycob 199 RPDDHMLTTLISGCSEEGTTLSDNEIRDSIVSLITAGYETTSGALAWAIYALLTVPGTWESAASEVARVL 268

CYP139A1_2589598915_Mycob 199 RPDDHMLTTLISGCSEEGTTLSDNEIRDSIVSLITAGYETTSGALAWAIYALLTVPGTWESAASEVARVL 268

CYP139A1_2589679190_Mycob 199 RPDDHMLTTLISGCSEEGTTLSDNEIRDSIVSLITAGYETTSGALAWAIYALLTVPGTWESAASEVARVL 268

CYP139A1_2589727979_Mycob 199 RPDDHMLTTLISGCSEEGTTLSDNEIRDSIVSLITAGYETTSGALAWAIYALLTVPGTWESAASEVARVL 268

CYP139A1_2592361217_Mycob 199 RPDDHMLTTLISGCSEEGTTLSDNEIRDSIVSLITAGYETTSGALAWAIYALLTVPGTWESAASEVARVL 268

CYP139A1_2592442711_Mycob 199 RPDDHMLTTLISGCSEEGTTLSDNEIRDSIVSLITAGYETTSGALAWAIYALLTVPGTWESAASEVARVL 268

CYP139A1_2555325132_Mycob 205 RPDDHMLTTLISGCSEEGTTLSDNEIRDSIVSLITAGYETTSGALAWAIYALLTVPGTWESAASEVARVL 274

CYP139A1_2566259019_Mycob 205 RPDDHMLTTLISGCSEEGTTLSDNEIRDSIVSLITAGYETTSGALAWAIYALLTVPGTWESAASEVARVL 274

CYP139A1_2555164435_Mycob 205 RPDDHMLTTLISGCSEEGTTLSDNEIRDSIVSLITAGYETTSGALAWAIYALLTVPGTWESAASEVARVL 274

CYP139A1_2555583039_Mycob 205 RPDDHMLTTLISGCSEEGTTLSDNEIRDSIVSLITAGYETTSGALAWAIYALLTVPGTWESAASEVARVL 274

CYP139A1_2555295099_Mycob 205 RPDDHMLTTLISGCSEEGTTLSDNEIRDSIVSLITAGYETTSGALAWAIYALLTVPGTWESAASEVARVL 274

CYP139A1_2555337752_Mycob 205 RPDDHMLTTLISGCSEEGTTLSDNEIRDSIVSLITAGYETTSGALAWAIYALLTVPGTWESAASEVARVL 274

CYP139A1_2555516018_Mycob 205 RPDDHMLTTLISGCSEEGTTLSDNEIRDSIVSLITAGYETTSGALAWAIYALLTVPGTWESAASEVARVL 274

CYP139A1_2576196505_Mycob 199 RPDDHMLTTLISGCSEEGTTLSDNEIRDSIVSLITAGYETTSGALAWAIYALLTVPGTWESAASEVARVL 268

CYP139A1_2555417623_Mycob 205 RPDDHMLTTLISGCSEEGTTLSDNEIRDSIVSLITAGYETTSGALAWAIYALLTVPGTWESAASEVARVL 274

CYP139A1_2555430098_Mycob 205 RPDDHMLTTLISGCSEEGTTLSDNEIRDSIVSLITAGYETTSGALAWAIYALLTVPGTWESAASEVARVL 274

CYP139A1_2547880750_Mycob 205 RPDDHMLTTLISGCSEEGTTLSDNEIRDSIVSLITAGYETTSGALAWAIYALLTVPGTWESAASEVARVL 274

CYP139A1_2559163499_Mycob 205 RPDDHMLTTLISGCSEEGTTLSDNEIRDSIVSLITAGYETTSGALAWAIYALLTVPGTWESAASEVARVL 274

CYP139A1_2527056892_Mycob 199 RPDDHMLTTLISGCSEEGTTLSDNEIRDSIVSLITAGYETTSGALAWAIYALLTVPGTWESAASEVARVL 268

CYP139A1_2574675630_Mycob 199 RPDDHMLTTLISGCSEEGTTLSDNEIRDSIVSLITAGYETTSGALAWAIYALLTVPGTWESAASEVARVL 268

CYP139A1_2574700588_Mycob 199 RPDDHMLTTLISGCSEEGTTLSDNEIRDSIVSLITAGYETTSGALAWAIYALLTVPGTWESAASEVARVL 268

CYP139A1_2574830606_Mycob 199 RPDDHMLTTLISGCSEEGTTLSDNEIRDSIVSLITAGYETTSGALAWAIYALLTVPGTWESAASEVARVL 268

CYP139A1_2574928549_Mycob 199 RPDDHMLTTLISGCSEEGTTLSDNEIRDSIVSLITAGYETTSGALAWAIYALLTVPGTWESAASEVARVL 268

CYP139A1_2575381682_Mycob 199 RPDDHMLTTLISGCSEEGTTLSDNEIRDSIVSLITAGYETTSGALAWAIYALLTVPGTWESAASEVARVL 268

CYP139A1_2575663406_Mycob 199 RPDDHMLTTLISGCSEEGTTLSDNEIRDSIVSLITAGYETTSGALAWAIYALLTVPGTWESAASEVARVL 268

CYP139A1_2576431117_Mycob 199 RPDDHMLTTLISGCSEEGTTLSDNEIRDSIVSLITAGYETTSGALAWAIYALLTVPGTWESAASEVARVL 268

CYP139A1_2576553939_Mycob 199 RPDDHMLTTLISGCSEEGTTLSDNEIRDSIVSLITAGYETTSGALAWAIYALLTVPGTWESAASEVARVL 268

CYP139A1_2577269438_Mycob 199 RPDDHMLTTLISGCSEEGTTLSDNEIRDSIVSLITAGYETTSGALAWAIYALLTVPGTWESAASEVARVL 268

CYP139A1_2584870715_Mycob 199 RPDDHMLTTLISGCSEEGTTLSDNEIRDSIVSLITAGYETTSGALAWAIYALLTVPGTWESAASEVARVL 268

CYP139A1_2589036086_Mycob 199 RPDDHMLTTLISGCSEEGTTLSDNEIRDSIVSLITAGYETTSGALAWAIYALLTVPGTWESAASEVARVL 268

CYP139A1_2590101605_Mycob 199 RPDDHMLTTLISGCSEEGTTLSDNEIRDSIVSLITAGYETTSGALAWAIYALLTVPGTWESAASEVARVL 268

CYP139A1_2590354422_Mycob 199 RPDDHMLTTLISGCSEEGTTLSDNEIRDSIVSLITAGYETTSGALAWAIYALLTVPGTWESAASEVARVL 268

CYP139A1_648490022_Mycoba 199 RPDDHMLTTLISGCSEEGTTLSDNEIRDSIVSLITAGYETTSGALAWAIYALLTVPGTWESAASEVARVL 268

CYP139A1_2547317188_Mycob 205 RPDDHMLTTLISGCSEEGTTLSDNEIRDSIVSLITAGYETTSGALAWAIYALLTVPGTWESAASEVARVL 274

CYP139A1_2547959756_Mycob 205 RPDDHMLTTLISGCSEEGTTLSDNEIRDSIVSLITAGYETTSGALAWAIYALLTVPGTWESAASEVARVL 274

CYP139A1_2555303475_Mycob 205 RPDDHMLTTLISGCSEEGTTLSDNEIRDSIVSLITAGYETTSGALAWAIYALLTVPGTWESAASEVARVL 274

CYP139A1_2555392373_Mycob 205 RPDDHMLTTLISGCSEEGTTLSDNEIRDSIVSLITAGYETTSGALAWAIYALLTVPGTWESAASEVARVL 274

CYP139A1_2566985178_Mycob 205 KPDDHMLTTLISGCSEEGTTLSDNEIRDSIVSLITAGYETTSGALAWAIYALLTVPGTWESAASEVARVL 274

CYP139A1_2555362970_Mycob 205 RPDDHMLTTLISGCSEEGTTLSDNEIRDSIVSLITAGYETTSGALAWAIYALLTVPGTWESAASEVARVL 274

CYP139A1_2560449536_Mycob 205 RPDDHMLTTLISGCSEEGTTLSDNEIRDSIVSLITAGYETTSGALAWAIYALLTVPGTWESAASEVARVL 274

CYP139A1_2555152706_Mycob 205 RPDDHMLTTLISGCSEEGTTLSDNEIRDSIVSLITAGYETTSGALAWAIYALLTVPGTWESAASEVARVL 274

CYP139A1_2555543565_Mycob 205 RPDDHMLTTLISGCSEEGTTLSDNEIRDSIVSLITAGYETTSGALAWAIYALLTVPGTWESAASEVARVL 274

CYP139A1_2590081259_Mycob 199 RPDDHMLTTLISGCSEEGTTLSDNEIRDSIVSLITAGYETTSGALAWAIYALLTVPGTWESAASEVARVL 268

CYP139A1_2547311116_Mycob 205 RPDDHMLTTLISGCSEEGTTLSDNEIRDSIVSLITAGYETTSGALAWAIYALLTVPGTWESAASEVARVL 274

CYP139A1_2548033169_Mycob 205 RPDDHMLTTLISGCSEEGTTLSDNEIRDSIVSLITAGYETTSGALAWAIYALLTVPGTWESAASEVARVL 274

CYP139A1_2548037418_Mycob 205 RPDDHMLTTLISGCSEEGTTLSDNEIRDSIVSLITAGYETTSGALAWAIYALLTVPGTWESAASEVARVL 274

CYP139A1_2555282371_Mycob 205 RPDDHMLTTLISGCSEEGTTLSDNEIRDSIVSLITAGYETTSGALAWAIYALLTVPGTWESAASEVARVL 274

CYP139A1_2541569776_Mycob 199 KPDDHMLTTLISGCSEEGTTLSDNEIRDSIVSLITAGYETTSGALAWAIYALLTVPGTWESAASEVARVL 268

CYP139A1_2555299255_Mycob 205 RPDDHMLTTLISGCSEEGTTLSDNEIRDSIVSLITAGYETTSGALAWAIYALLTVPGTWESAASEVARVL 274

CYP139A1_2555379694_Mycob 205 RPDDHMLTTLISGCSEEGTTLSDNEIRDSIVSLITAGYETTSGALAWAIYALLTVPGTWESAASEVARVL 274

CYP139A1_2555413387_Mycob 205 RPDDHMLTTLISGCSEEGTTLSDNEIRDSIVSLITAGYETTSGALAWAIYALLTVPGTWESAASEVARVL 274

CYP139A1_2555587223_Mycob 205 RPDDHMLTTLISGCSEEGTTLSDNEIRDSIVSLITAGYETTSGALAWAIYALLTVPGTWESAASEVARVL 274

CYP139A1_2555591399_Mycob 205 RPDDHMLTTLISGCSEEGTTLSDNEIRDSIVSLITAGYETTSGALAWAIYALLTVPGTWESAASEVARVL 274

CYP139A1_2547306531_Mycob 205 RPDDHMLTTLISGCSEEGTTLSDNEIRDSIVSLITAGYETTSGALAWAIYALLTVPGTWESAASEVARVL 274

CYP139A1_651088108_Mycoba 205 RPDDHMLTTLISGCSEEGTTLSDNEIRDSIVSLITAGYETTSGALAWAIYALLTVPGTWESAASEVARVL 274

CYP139A1_2598067418_Mycob 205 RPDDHMLTTLISGCSEEGTTLSDNEIRDSIVSLITAGYETTSGALAWAIYALLTVPGTWESAASEVARVL 274

CYP139A1_2555341950_Mycob 205 RPDDHMLTTLISGCSEEGTTLSDNEIRDSIVSLITAGYETTSGALAWAIYALLTVPGTWESAASEVARVL 274

CYP139A1_2555446824_Mycob 205 RPDDHMLTTLISGCSEEGTTLSDNEIRDSIVSLITAGYETTSGALAWAIYALLTVPGTWESAASEVARVL 274

CYP139A1_2555556540_Mycob 205 RPDDHMLTTLISGCSEEGTTLSDNEIRDSIVSLITAGYETTSGALAWAIYALLTVPGTWESAASEVARVL 274

CYP139A1_2560451827_Mycob 205 RPDDHMLTTLISGCSEEGTTLSDNEIRDSIVSLITAGYETTSGALAWAIYALLTVPGTWESAASEVARVL 274

CYP139A1_2540619998_Mycob 205 RPDDHMLTTLISGCSEEGTTLSDNEIRDSIVSLITAGYETTSGALAWAIYALLTVPGTWESAASEVARVL 274

CYP139A1_2554692349_Mycob 205 RPDDHMLTTLISGCSEEGTTLSDNEIRDSIVSLITAGYETTSGALAWAIYALLTVPGTWESAASEVARVL 274

CYP139A1_637096038_Mycoba 205 RPDDHMLTTLISGCSEEGTTLSDNEIRDSIVSLITAGYETTSGALAWAIYALLTVPGTWESAASEVARVL 274

CYP139A1_2555396597_Mycob 205 RPDDHMLTTLISGCSEEGTTLSDNEIRDSIVSLITAGYETTSGALAWAIYALLTVPGTWESAASEVARVL 274

CYP139A1_2555520186_Mycob 205 RPDDHMLTTLISGCSEEGTTLSDNEIRDSIVSLITAGYETTSGALAWAIYALLTVPGTWESAASEVARVL 274

CYP139A1_2555599804_Mycob 205 RPDDHMLTTLISGCSEEGTTLSDNEIRDSIVSLITAGYETTSGALAWAIYALLTVPGTWESAASEVARVL 274

CYP139A1_2547314995_Mycob 205 RPDDHMLTTLISGCSEEGTTLSDNEIRDSIVSLITAGYETTSGALAWAIYALLTVPGTWESAASEVARVL 274

CYP139A1_2555160272_Mycob 205 RPDDHMLTTLISGCSEEGTTLSDNEIRDSIVSLITAGYETTSGALAWAIYALLTVPGTWESAASEVARVL 274

CYP139A1_2555367201_Mycob 205 RPDDHMLTTLISGCSEEGTTLSDNEIRDSIVSLITAGYETTSGALAWAIYALLTVPGTWESAASEVARVL 274

CYP139A1_2555547894_Mycob 205 RPDDHMLTTLISGCSEEGTTLSDNEIRDSIVSLITAGYETTSGALAWAIYALLTVPGTWESAASEVARVL 274

CYP139A1_2560454644_Mycob 205 RPDDHMLTTLISGCSEEGTTLSDNEIRDSIVSLITAGYETTSGALAWAIYALLTVPGTWESAASEVARVL 274

CYP139A1_2546436155_Mycob 199 RPDDHMLTTLISGCSEEGTTLSDNEIRDSIVSLITAGYETTSGALAWAIYALLTVPGTWESAASEVARVL 268

CYP139A1_2555307696_Mycob 205 RPDDHMLTTLISGCSEEGTTLSDNEIRDSIVSLITAGYETTSGALAWAIYALLTVPGTWESAASEVARVL 274

CYP139A1_2555524372_Mycob 205 RPDDHMLTTLISGCSEEGTTLSDNEIRDSIVSLITAGYETTSGALAWAIYALLTVPGTWESAASEVARVL 274

CYP139A1_2546192085_Mycob 199 RPDDHMLTTLISGXXXEGTTLSDNEIRDSIVSLITAGYETTSGALAWAIYALLTVPGTWESAASEVARVL 268

CYP139A1_2553261414_Mycob 205 RPDDHMLTTLISGCSEEGTTLSDNEIRDSIVSLITAGYETTSGALAWAIYALLTVPGTWESAASEVARVL 274

CYP139A1_2555442648_Mycob 205 RPDDHMLTTLISGCSEEGTTLSDNEIRDSIVSLITAGYETTSGALAWAIYALLTVPGTWESAASEVARVL 274

CYP139A1_2555455224_Mycob 205 RPDDHMLTTLISGCSEEGTTLSDNEIRDSIVSLITAGYETTSGALAWAIYALLTVPGTWESAASEVARVL 274

CYP139A1_651084428_Mycoba 205 RPDDHMLTTLISGCSEEGTTLSDNEIRDSIVSLITAGYETTSGALAWAIYALLTVPGTWESAASEVARVL 274

CYP139A1_2555273883_Mycob 205 RPDDHMLTTLISGCSEEGTTLSDNEIRDSIVSLITAGYETTSGALAWAIYALLTVPGTWESAASEVARVL 274

CYP139A1_2555286609_Mycob 205 RPDDHMLTTLISGCSEEGTTLSDNEIRDSIVSLITAGYETTSGALAWAIYALLTVPGTWESAASEVARVL 274

CYP139A1_2555329342_Mycob 205 RPDDHMLTTLISGCSEEGTTLSDNEIRDSIVSLITAGYETTSGALAWAIYALLTVPGTWESAASEVARVL 274

CYP139A1_2555438445_Mycob 205 RPDDHMLTTLISGCSEEGTTLSDNEIRDSIVSLITAGYETTSGALAWAIYALLTVPGTWESAASEVARVL 274

CYP139A1_2577281457_Mycob 205 RPDDHMLTTLISGCSEEGTTLSDNEIRDSIVSLITAGYETTSGALAWAIYALLTVPGTWESAASEVARVL 274

CYP139A1_2590256393_Mycob 199 RPDDHMLTTLISGCSEEGTTLSDNEIRDSIVSLITAGYETTSGALAWAIYALLTVPGTWESAASEVARVL 268

CYP139A1_2549407735_Mycob 205 RPDDHMLTTLISGCSEEGTTLSDNEIRDSIVSLITAGYETTSGALAWAIYALLTVPGTWESAASEVARVL 274

CYP139A1_2573574061_Mycob 205 RPDDHMLTTLISGCSEEGTTLSDNEIRDSIVSLITAGYETTSGALAWAIYALLTVPGTWESAASEVARVL 274

CYP139A1_2540803840_Mycob 205 RPDDHMLTTLISGCSEEGTTLSDNEIRDSIVSLITAGYETTSGALAWAIYALLTVPGTWESAASEVARVL 274

CYP139A1_2584769363_Mycob 199 RPDDHMLTTLISGCSEEGTTLSDNEIRDSIVSLITAGYETTSGALAWAIYALLTVPGTWESAASEVARVL 268

CYP139A1_2555311951_Mycob 205 RPDDHMLTTLISGCSEEGTTLSDNEIRDSIVSLITAGYETTSGALAWAIYALLTVPGTWESAASEVARVL 274

CYP139A1_2555346108_Mycob 205 RPDDHMLTTLISGCSEEGTTLSDNEIRDSIVSLITAGYETTSGALAWAIYALLTVPGTWESAASEVARVL 274

CYP139A1_2555383921_Mycob 205 RPDDHMLTTLISGCSEEGTTLSDNEIRDSIVSLITAGYETTSGALAWAIYALLTVPGTWESAASEVARVL 274

CYP139A1_2555400811_Mycob 205 RPDDHMLTTLISGCSEEGTTLSDNEIRDSIVSLITAGYETTSGALAWAIYALLTVPGTWESAASEVARVL 274

CYP139A1_2555560826_Mycob 205 RPDDHMLTTLISGCSEEGTTLSDNEIRDSIVSLITAGYETTSGALAWAIYALLTVPGTWESAASEVARVL 274

CYP139A1_2598813154_Mycob 205 RPDDHMLTTLISGCSEEGTTLSDNEIRDSIVSLITAGYETTSGALAWAIYALLTVPGTWESAASEVARVL 274

CYP139A1_2547164190_Mycob 205 RPDDHMLTTLISGCSEEGTTLSDNEIRDSIVSLITAGYETTSGALAWAIYALLTVPGTWESAASEVARVL 274

CYP139A1_2555409181_Mycob 205 RPDDHMLTTLISGCSEEGTTLSDNEIRDSIVSLITAGYETTSGALAWAIYALLTVPGTWESAASEVARVL 274

CYP139A1_2547759833_Mycob 205 RPDDHMLTTLISGCSEEGTTLSDNEIRDSIVSLITAGYETTSGALAWAIYALLTVPGTWESAASEVARVL 274

CYP139A1_2547955540_Mycob 205 RPDDHMLTTLISGCSEEGTTLSDNEIRDSIVSLITAGYETTSGALAWAIYALLTVPGTWESAASEVARVL 274

CYP139A1_2560461324_Mycob 205 RPDDHMLTTLISGCSEEGTTLSDNEIRDSIVSLITAGYETTSGALAWAIYALLTVPGTWESAASEVARVL 274

CYP139A1_2576392640_Mycob 199 RPDDHMLTTLISGCSEEGTTLSDNEIRDSIVSLITAGYETTSGALAWAIYALLTVPGTWESAASEVARVL 268

CYP139A1_2541573920_Mycob 199 RPDDHMLTTLISGCSEEGTTLSDNEIRDSIVSLITAGYETTSGALAWAIYALLTVPGAWESAASEVARVL 268

CYP139A1_2549413118_Mycob 205 RPDDHMLTTLISGCSEEGTTLSDNEIRDSIVSLITAGYETTSGALAWAIYALLTVPGTWESAASEVARVL 274

CYP139A1_2555333539_Mycob 205 RPDDHMLTTLISGCSEEGTTLSDNEIRDSIVSLITAGYETTSGALAWAIYALLTVPGTWESAASEVARVL 274

CYP139A1_2555371358_Mycob 205 RPDDHMLTTLISGCSEEGTTLSDNEIRDSIVSLITAGYETTSGALAWAIYALLTVPGTWESAASEVARVL 274

CYP139A1_2545499027_Mycob 205 RPDDHMLTTLISGCSEEGTTLSDNEIRDSIVSLITAGYETTSGALAWAIYALLTVPGTWESAASEVARVL 274

CYP139A1_2551812688_Mycob 205 RPDDHMLTTLISGCSEEGTTLSDNEIRDSIVSLITAGYETTSGALAWAIYALLTVPGTWESAASEVARVL 274

CYP139A1_2620699696_Mycob 205 RPDDHMLTTLISGCSEEGTTLSDNEIRDSIVSLITAGYETTSGALAWAIYALLTVPGTWESAASEVARVL 274

CYP139A1_2555278104_Mycob 205 RPDDHMLTTLISGCSEEGTTLSDNEIRDSIVSLITAGYETTSGALAWAIYALLTVPGTWESAASEVARVL 274

CYP139A1_2555320646_Mycob 205 RPDDHMLTTLISGCSEEGTTLSDNEIRDSIVSLITAGYETTSGALAWAIYALLTVPGTWESAASEVARVL 274

CYP139A1_2555354513_Mycob 205 RPDDHMLTTLISGCSEEGTTLSDNEIRDSIVSLITAGYETTSGALAWAIYALLTVPGTWESAASEVARVL 274

CYP139A1_2549401785_Mycob 205 RPDDHMLTTLISGCSEEGTTLSDNEIRDSIVSLITAGYETTSGALAWAIYALLTVPGTWESAASEVARVL 274

CYP139A1_2555434304_Mycob 205 RPDDHMLTTLISGCSEEGTTLSDNEIRDSIVSLITAGYETTSGALAWAIYALLTVPGTWESAASEVARVL 274

CYP139A1_2555528546_Mycob 205 RPDDHMLTTLISGCSEEGTTLSDNEIRDSIVSLITAGYETTSGALAWAIYALLTVPGTWESAASEVARVL 274

CYP139A1_2555595572_Mycob 205 RPDDHMLTTLISGCSEEGTTLSDNEIRDSIVSLITAGYETTSGALAWAIYALLTVPGTWESAASEVARVL 274

CYP139A1_2555144317_Mycob 205 RPDDHMLTTLISGCSEEGTTLSDNEIRDSIVSLITAGYETTSGALAWAIYALLTVPGTWESAASEVARVL 274

CYP139A1_2555290904_Mycob 205 RPDDHMLTTLISGCSEEGTTLSDNEIRDSIVSLITAGYETTSGALAWAIYALLTVPGTWESAASEVARVL 274

CYP139A1_2555316223_Mycob 205 RPDDHMLTTLISGCSEEGTTLSDNEIRDSIVSLITAGYETTSGALAWAIYALLTVPGTWESAASEVARVL 274

CYP139A1_2555388140_Mycob 205 RPDDHMLTTLISGCSEEGTTLSDNEIRDSIVSLITAGYETTSGALAWAIYALLTVPGTWESAASEVARVL 274

CYP139A1_2555350295_Mycob 205 RPDDHMLTTLISGCSEEGTTLSDNEIRDSIVSLITAGYETTSGALAWAIYALLTVPGTWESAASEVARVL 274

CYP139A1_2555960457_Mycob 205 RPDDHMLTTLISGCSEEGTTLSDNEIRDSIVSLITAGYETTSGALAWAIYALLTVPGTWESAASEVARVL 274

CYP139A1_2547951333_Mycob 205 RPDDHMLTTLISGCSEEGTTLSDNEIRDSIVSLITAGYETTSGALAWAIYALLTVPGTWESAASEVARVL 274

CYP139A1_2555451004_Mycob 205 RPDDHMLTTLISGCSEEGTTLSDNEIRDSIVSLITAGYETTSGALAWAIYALLTVPGTWESAASEVARVL 274

CYP139A1_2555140094_Mycob 205 RPDDHMLTTLISGCSEEGTTLSDNEIRDSIVSLITAGYETTSGALAWAIYALLTVPGTWESAASEVARVL 274

CYP139A1_2555358731_Mycob 205 RPDDHMLTTLISGCSEEGTTLSDNEIRDSIVSLITAGYETTSGALAWAIYALLTVPGTWESAASEVARVL 274

CYP139A1_2555375511_Mycob 205 RPDDHMLTTLISGCSEEGTTLSDNEIRDSIVSLITAGYETTSGALAWAIYALLTVPGTWESAASEVARVL 274

CYP139A1_2555578867_Mycob 205 RPDDHMLTTLISGCSEEGTTLSDNEIRDSIVSLITAGYETTSGALAWAIYALLTVPGTWESAASEVARVL 274

CYP139A1_2554700949_Mycob 205 RPDDHMLTTLISGCSEEGTTLSDNEIRDSIVSLITAGYETTSGALAWAIYALLTVPGTWESAASEVARVL 274

CYP139A1_2575403392_Mycob 205 RPDDHMLTTLISGCSEEGTTLSDNEIRDSIVSLITAGYETTSGALAWAIYALLTVPGTWESAASEVARVL 274

CYP139A1_638726892_Mycoba 205 RPDDHMLTTLISGCSEEGTTLSDNEIRDSIVSLITAGYETTSGALAWAIYALLTVPGTWESAASEVARVL 274

CYP139A1_648443266_Mycoba 205 RPDDHMLTTLISGCSEEGTTLSDNEIRDSIVSLITAGYETTSGALAWAIYALLTVPGTWESAASEVARVL 274

CYP139A1_2540554561_Mycob 199 RPDDHMLTTLISGCPKEGTTLSDNEIRDSIVSLITAGYETTSGALAWAIYALLTVPGAWESAASEVARVL 268

CYP139A1_2566980890_Mycob 205 RPDDHMLTTLISGCPEEGTTLSDNEIRDSIVSLITAGYETTSGALAWAIYALLTVPGAWESAANEVARVL 274

CYP139A1_2566976623_Mycob 205 RPDDHMLTTLISGCPEEGTTLSDNEIRDSIVSLITAGYETTSGALAWAIYALLTVPGAWESAANEVARVL 274

CYP139A1_2566972350_Mycob 205 RPDDHMLTTLISGCPEEGTTLSDNEIRDSIVSLITAGYETTSGALAWAIYALLTVPGAWESAANEVARVL 274

CYP139A1_2514118145_Mycob 200 RPDDHMLTTLISGCSEEGTTLSDNEIRDSIVSLITAGYETTSGALAWAIYALLTVPGTWESAASEVARVL 269

[Consensus_aa:](http://prodata.swmed.edu/promals3d/info/consensus.html) p**PDD+MLT***h***LI**s**G**.s**-EG**.*h***L**s**D**s**EIRD**t**IVSLI***h***AGYETTSGALAWA***l***Y***h***LL**o*h***PG***h***W**po**AA**s**EV**.**RVL**

[Consensus_ss:](http://prodata.swmed.edu/promals3d/info/consensus_ss.html) hhhhhhh h hhhhhhhhhhhhh hhhhhhhhhhhhhhhhhhhhhhhhhhhhhhhh

Conservation: 9 6 966969696999999696969996 969 9 9696 69969699 99999669 9

CYP139A_2567124714_Mycoba 268 GGNAPAADNIGSLTYLNGLVQETLRLYSPGVISARRVMRDLVFDGHRVRSGRLLIFSAYVTHRLPEIWPA 337

CYP139A_650873455_Mycobac 269 DGRTPASADLKNLTYLNGVVHETLRLYPPAVISARKVTRELSFHGRRIHAGRTLVFSPYVTHRLPELWVD 338

CYP139A_2549393401_Mycoba 273 GERAP---DVAGLTYLNGVVHETLRLYPPGVISARKVTRELSFAGRRIRAGRTLVFSLYVTHRLPELWAD 339

CYP139A_2548535921_Mycoba 272 AGLPPAAADLSGLTYLNGVVHETLRLYPPGVISARRVMRDLRFKGRRIRSGRLLIFSPYVTHRLHEIWPE 341

CYP139A_2576976958_Mycoba 269 AGRPPAAADLSGLTYLNGVVHETLRLYPPGVISARRVMRDLRFEGRRIRSGRLLIFSPYVTHRLPEIWPE 338

CYP139A_2581913245_Mycoba 269 AGRPPAAADLSGLTYLNGVVHETLRLYPPGVISARRVMRDLRFEGRRIRSGRLLIFSPYVTHRLPEIWPE 338

CYP139A_2580974538_Mycoba 269 AGRPPAAADLSGLTYLNGVVHETLRLYPPGVISARRVMRDLRFEGRRIRSGRLLIFSPYVTHRLPEIWPE 338

CYP139A_2582181025_Mycoba 269 AGRPPAAADLSGLTYLNGVVHETLRLYPPGVISARRVMRDLRFEGRRIRSGRLLIFSPYVTHRLPEIWPE 338

CYP139A_2580742569_Mycoba 269 AGRPPAAADLSGLTYLNGVVHETLRLYPPGVISARRVMRDLRFEGRRIRSGRLLIFSPYVTHRLPEIWPE 338

CYP139A_2582203743_Mycoba 269 AGRPPAAADLSGLTYLNGVVHETLRLYPPGVISARRVMRDLRFEGRRIRSGRLLIFSPYVTHRLPEIWPE 338

CYP139A_2567079276_Mycoba 251 AGRLPAAADLSGLTYLNGVVHETLRLYPPGVISARRVMRDLRFEGRRIRSGRLLIFSPYVTHRLPEIWPE 320

CYP139A_2543326887_Mycoba 269 AGLPPAAADLSGLTYLNGVVHETLRLYPPGVISARRVMRDLRFKGRRIRSGRLLIFSPYVTHRLHEIWPE 338

CYP139A_2570865822_Mycoba 269 AGLPPAAADLSGLTYLNGVVHETLRLYPPGVISARRVMRDLRFKGRRIRSGRLLIFSPYVTHRLHEIWPE 338

CYP139A_2592485489_Mycoba 269 AGLPPAAADLSGLTYLNGVVHETLRLYPPGVISARRVMRDLRFKGRRIRSGRLLIFSPYVTHRLHEIWPE 338

CYP139A_2550738610_Mycoba 272 AGLPPAAADLSGLTYLNGVVHETLRLYPPGVISARRVMRDLRFKGRRIRSGRLLIFSPYVTHRLHEIWPE 341

CYP139A_2580783837__Mycob 269 AGRPPAAADLSGLTYLNGVVHETLRLYPPGVISARRVMRDLRFEGRRIRSGRLLIFSPYVTHRLPEIWPE 338

CYP139A_2569618768_Mycoba 269 AGRPPAAADLSGLTYLNGVVHETLRLYPPGVISARRVMRDLRFEGRRIRSGRLLIFSPYVTHRLPEIWPE 338

CYP139A_2547368463_Mycoba 272 AGLPPAAADLSGLTYLNGVVHETLRLYPPGVISARRVMRDLRFKGRRIRSGRLLIFSPYVTHRLHEIWPE 341

CYP139A_2572767979_Mycoba 269 AGRPPAAADLSGLTYLNGVVHETLRLYPPGVISARRAMRDLRFEGRRIRSGRLLIFSPYVTHRLPEIWPE 338

CYP139A_2582391300_Mycoba 269 AGRLPAAADLSGLTYLNGVVHETLRLYPPGVISARRVMRDLRFEGRRIRSGRLLIFSPYVTHRLPEIWPE 338

CYP139A_2548515815_Mycoba 272 AGLPPAAADLSGLTYLNGVVHETLRLYPPGVISARRVMRDLRFKGRRIRSGRLLIFSPYVTHRLHEIWPE 341

CYP139A_2548530385_Mycoba 272 AGLPPAAADLSGLTYLNGVVHETLRLYPPGVISARRVMRDLRFKGRRIRSGRLLIFSPYVTHRLHEIWPE 341

CYP139A_2581397788_Mycoba 269 AGRLPAAADLSGLTYLNGVVHETLRLYPPGVISARRVMRDLRFEGRRIRSGRLLIFSPYVTHRLPEIWPE 338

CYP139A_2549377452_Mycoba 272 AGLPPAAADLSGLTYLNGVVHETLRLYPPGVISARRVMRDLRFKGRRIRSGRLLIFSPYVTHRLHEIWPE 341

CYP139A_2549389164_Mycoba 272 AGRLPAAADLSGLTYLNGVVHETLRLYPPGVISARRVMRDLRFEGRRIRSGRLLIFSPYVTHRLPEIWPE 341

CYP139A_645425415_Mycobac 269 AGRPPAAADLSGLTYLNGVVHETLRLYPPGVISARRVMRDLRFEGRRIRSGRLLIFSPYVTHRLPEIWPE 338

CYP139A_2581110378_Mycoba 272 AGRLPAAADLSGLTYLNGVVHETLRLYPPGVISARRVMRDLRFEGRRIRSGRLLIFSPYVTHRLPEIWPE 341

CYP139A_2573433552_Mycoba 269 AGRLPAAADLSGLTYLNGVVHETLRLYPPGVISARRVMRDLRFEGRRIRSGRLLIFSPYVTHRLPEIWPE 338

CYP139A_2580006443__Mycob 269 AGRLPAAADLSGLTYLNGVVHETLRLYPPGVISARRVMRDLRFEGRRIRSGRLLIFSPYVTHRLPEIWPE 338

CYP139A_637134331_Mycobac 269 AGLPPAAADLSGLTYLNGVVHETLRLYPPGVISARRVMRDLRFKGRRIRSGRLLIFSPYVTHRLHEIWPE 338

CYP139A_2548578292_Mycoba 272 AGLPPAAADLSGLTYLNGVVHETLRLYPPGVISARRVMRDLRFKGRRIRSGRLLIFSPYVTHRLHEIWPE 341

CYP139A_2548547272_Mycoba 272 AGLPPAAADLSGLTYLNGVVHETLRLYPPGVISARRVMRDLRFKGRRIRSGRLLIFSPYVTHRLHEIWPE 341

CYP139A_2549383420_Mycoba 272 AGLPPAAADLSGLTYLNGVVHETLRLYPPGVISARRVMRDLRFKGRRIRSGRLLIFSPYVTHRLHEIWPE 341

CYP139A_639736419_Mycobac 269 AGRLPAAADLSGLTYLNGVVHETLRLYPPGVISARRVMRDLRFEGRRIRSGRLLIFSPYVTHRLPEIWPE 338

CYP139A_2555735619_Mycoba 269 AGLPPAAADLSGLTYLNGVVHETLRLYPPGVISARRVMRDLRFKGRRIRSGRLLIFSPYVTHRLHEIWPE 338

CYP139A_2555481387_Mycoba 280 GDRPPAAADLDALVYLDGVVHETLRLYPPGVISARKVMRDLTFDGHRIPAGRLLIFSPYVTHRLPELWPQ 349

CYP139A_2545768030_Mycoba 274 GDAPPGAADLDALTYLNGVVHETLRLYSPGVISARRVMRDLWFDGRRIRAGRLLIFSAYVTHRLPEIWPD 343

CYP139A_2567131988_Mycoba 268 GGNAPAADNIDSLTFLNGLVQETLRLYSPGVISARRVMRDLVFDGHRIRSGRLLIFSAYVTHRLPEIWPA 337

CYP139A_2587480388_Mycoba 268 GGNAPAADNIDSLTFLNGLVQETLRLYSPGVISARRVMRDLVFDGHRIRSGRLLIFSAYVTHRLPEIWPA 337

CYP139A_2563577345_Mycoba 268 GGNAPAADNIDSLTFLNGLVQETLRLYSPGVISARRVMRDLVFDGHRIRSGRLLIFSAYVTHRLPEIWPA 337

CYP139A_2543277028_Mycoba 275 GGTAPSAETLGALTYLNGVVHETLRLYSPGVVSARRLIRDLWFDGHRINSGRLLIFSAYVTHRIPEVWPN 344

CYP139A_2563569217_Mycoba 275 GGTAPSAETLGALTYLNGVVHETLRLYSPGVVSARRLIRDLWFDGHRINSGRLLIFSAYVTHRIPEVWPN 344

CYP139A_641717750_Mycobac 275 GGTAPSAETLGALTYLNGVVHETLRLYSPGVVSARRLIRDLWFDGHRINSGRLLIFSAYVTHRIPEVWPN 344

CYP139A_2588629254_Mycoba 275 GGTAPSAETLGALTYLNGVVHETLRLYSPGVVSARRLIRDLWFDGHRINSGRLLIFSAYVTHRIPEVWPN 344

CYP139A_2546369014_Mycoba 275 GGTAPSAETLGALTYLNGVVHETLRLYSPGVVSARRLIRDLWFDGHRINSGRLLIFSAYVTHRIPEVWPN 344

CYP139A1_2555148489_Mycob 275 GGRVPAADDLSALTYLNGVVHETLRLYSPGVISARRVLRDLWFDGHRIRAGRLLIFSAYVTHRLPEIWPE 344

CYP139A1_646010237_Mycoba 229 GGRVPAADDLSALTYLNGVVHETLRLYSPGVISARRVLRDLWFDGHRIRAGRLLIFSAYVTHRLPEIWPE 298

CYP139A1_2581377024_Mycob 229 GGRVPAADDLSALTYLNGVVHETLRLYSPGVISARRVLRDLWFDGHRIRAGRLLIFSAYVTHRLPEIWPE 298

CYP139A1_647209603_Mycoba 229 GGRVPAADDLSALTYLNGVVHETLRLYSPGVISARRVLRDLWFDGHRIRAGRLLIFSAYVTHRLPEIWPE 298

CYP139A1_2537735281_Mycob 275 GGRVPAADDLSALTYLNGVVHETLRLYSPGVISARRVLRDLWFDGHRIRAGRLLIFSAYVTHRLPEIWPE 344

CYP139A1_2576388909_Mycob 269 GGRVPAADDLSALTYLNGVVHETLRLYSPGVISARRVLRDLWFDGHRIRAGRLLIFSAYVTHRLPEIWPE 338

CYP139A1_2577593438_Mycob 269 GGRVPAADDLSALTYLNGVVHETLRLYSPGVISARRVLRDLWFDGHRIRAGRLLIFSAYVTHRLPEIWPE 338

CYP139A1_2577803488_Mycob 269 GGRVPAADDLSALTYLNGVVHETLRLYSPGVISARRVLRDLWFDGHRIRAGRLLIFSAYVTHRLPEIWPE 338

CYP139A1_2581355094_Mycob 269 GGRVPAADDLSALTYLNGVVHETLRLYSPGVISARRVLRDLWFDGHRIRAGRLLIFSAYVTHRLPEIWPE 338

CYP139A1_2584983051_Mycob 269 GGRVPAADDLSALTYLNGVVHETLRLYSPGVISARRVLRDLWFDGHRIRAGRLLIFSAYVTHRLPEIWPE 338

CYP139A1_2584987406_Mycob 269 GGRVPAADDLSALTYLNGVVHETLRLYSPGVISARRVLRDLWFDGHRIRAGRLLIFSAYVTHRLPEIWPE 338

CYP139A1_2589032800_Mycob 269 GGRVPAADDLSALTYLNGVVHETLRLYSPGVISARRVLRDLWFDGHRIRAGRLLIFSAYVTHRLPEIWPE 338

CYP139A1_2592403099_Mycob 269 GGRVPAADDLSALTYLNGVVHETLRLYSPGVISARRVLRDLWFDGHRIRAGRLLIFSAYVTHRLPEIWPE 338

CYP139A1_2592422247_Mycob 269 GGRVPAADDLSALTYLNGVVHETLRLYSPGVISARRVLRDLWFDGHRIRAGRLLIFSAYVTHRLPEIWPE 338

CYP139A1_643734506_Mycoba 269 GGRVPAADDLSALTYLNGVVHETLRLYSPGVISARRVLRDLWFDGHRIRAGRLLIFSAYVTHRLPEIWPE 338

CYP139A1_648335985_Mycoba 269 GGRVPAADDLSALTYLNGVVHETLRLYSPGVISARRVLRDLWFDGHRIRAGRLLIFSAYVTHRLPEIWPE 338

CYP139A1_2574754194_Mycob 269 GGRVPAADDLSALTYLNGVVHETLRLYSPGVISARRVLRDLWFDGHRIRAGRLLIFSAYVTHRLPEIWPE 338

CYP139A1_2575447433_Mycob 269 GGRVPAADDLSALTYLNGVVHETLRLYSPGVISARRVLRDLWFDGHRIRAGRLLIFSAYVTHRLPEIWPE 338

CYP139A1_2575938969_Mycob 269 GGRVPAADDLSALTYLNGVVHETLRLYSPGVISARRVLRDLWFDGHRIRAGRLLIFSAYVTHRLPEIWPE 338

CYP139A1_2576477081_Mycob 269 GGRVPAADDLSALTYLNGVVHETLRLYSPGVISARRVLRDLWFDGHRIRAGRLLIFSAYVTHRLPEIWPE 338

CYP139A1_2576601719_Mycob 269 GGRVPAADDLSALTYLNGVVHETLRLYSPGVISARRVLRDLWFDGHRIRAGRLLIFSAYVTHRLPEIWPE 338

CYP139A1_2577098384_Mycob 269 GGRVPAADDLSALTYLNGVVHETLRLYSPGVISARRVLRDLWFDGHRIRAGRLLIFSAYVTHRLPEIWPE 338

CYP139A1_2578107196_Mycob 269 GGRVPAADDLSALTYLNGVVHETLRLYSPGVISARRVLRDLWFDGHRIRAGRLLIFSAYVTHRLPEIWPE 338

CYP139A1_2584883084_Mycob 269 GGRVPAADDLSALTYLNGVVHETLRLYSPGVISARRVLRDLWFDGHRIRAGRLLIFSAYVTHRLPEIWPE 338

CYP139A1_2588974834_Mycob 269 GGRVPAADDLSALTYLNGVVHETLRLYSPGVISARRVLRDLWFDGHRIRAGRLLIFSAYVTHRLPEIWPE 338

CYP139A1_2589056454_Mycob 269 GGRVPAADDLSALTYLNGVVHETLRLYSPGVISARRVLRDLWFDGHRIRAGRLLIFSAYVTHRLPEIWPE 338

CYP139A1_2589161189_Mycob 269 GGRVPAADDLSALTYLNGVVHETLRLYSPGVISARRVLRDLWFDGHRIRAGRLLIFSAYVTHRLPEIWPE 338

CYP139A1_2590374347_Mycob 269 GGRVPAADDLSALTYLNGVVHETLRLYSPGVISARRVLRDLWFDGHRIRAGRLLIFSAYVTHRLPEIWPE 338

CYP139A1_646018681_Mycoba 269 GGRVPAADDLSALTYLNGVVHETLRLYSPGVISARRVLRDLWFDGHRIRAGRLLIFSAYVTHRLPEIWPE 338

CYP139A1_2575060404_Mycob 269 GGRVPAADDLSALTYLNGVVHETLRLYSPGVISARRVLRDLWFDGHRIRAGRLLIFSAYVTHRLPEIWPE 338

CYP139A1_2576105631_Mycob 269 GGRVPAADDLSALTYLNGVVHETLRLYSPGVISARRVLRDLWFDGHRIRAGRLLIFSAYVTHRLPEIWPE 338

CYP139A1_2576247251_Mycob 269 GGRVPAADDLSALTYLNGVVHETLRLYSPGVISARRVLRDLWFDGHRIRAGRLLIFSAYVTHRLPEIWPE 338

CYP139A1_2576981010_Mycob 269 GGRVPAADDLSALTYLNGVVHETLRLYSPGVISARRVLRDLWFDGHRIRAGRLLIFSAYVTHRLPEIWPE 338

CYP139A1_2577093117_Mycob 269 GGRVPAADDLSALTYLNGVVHETLRLYSPGVISARRVLRDLWFDGHRIRAGRLLIFSAYVTHRLPEIWPE 338

CYP139A1_2577198903_Mycob 269 GGRVPAADDLSALTYLNGVVHETLRLYSPGVISARRVLRDLWFDGHRIRAGRLLIFSAYVTHRLPEIWPE 338

CYP139A1_2577516047_Mycob 269 GGRVPAADDLSALTYLNGVVHETLRLYSPGVISARRVLRDLWFDGHRIRAGRLLIFSAYVTHRLPEIWPE 338

CYP139A1_2578213104_Mycob 269 GGRVPAADDLSALTYLNGVVHETLRLYSPGVISARRVLRDLWFDGHRIRAGRLLIFSAYVTHRLPEIWPE 338

CYP139A1_2584711251_Mycob 269 GGRVPAADDLSALTYLNGVVHETLRLYSPGVISARRVLRDLWFDGHRIRAGRLLIFSAYVTHRLPEIWPE 338

CYP139A1_2584816678_Mycob 269 GGRVPAADDLSALTYLNGVVHETLRLYSPGVISARRVLRDLWFDGHRIRAGRLLIFSAYVTHRLPEIWPE 338

CYP139A1_2589068752_Mycob 269 GGRVPAADDLSALTYLNGVVHETLRLYSPGVISARRVLRDLWFDGHRIRAGRLLIFSAYVTHRLPEIWPE 338

CYP139A1_2589604293_Mycob 269 GGRVPAADDLSALTYLNGVVHETLRLYSPGVISARRVLRDLWFDGHRIRAGRLLIFSAYVTHRLPEIWPE 338

CYP139A1_643028176_Mycoba 269 GGRVPAADDLSALTYLNGVVHETLRLYSPGVISARRVLRDLWFDGHRIRAGRLLIFSAYVTHRLPEIWPE 338

CYP139A1_648476944_Mycoba 269 GGRVPAADDLSALTYLNGVVHETLRLYSPGVISARRVLRDLWFDGHRIRAGRLLIFSAYVTHRLPEIWPE 338

CYP139A1_2511736071_Mycob 269 GGRVPAADDLSALTYLNGVVHETLRLYSPGVISARRVLRDLWFDGHRIRAGRLLIFSAYVTHRLPEIWPE 338

CYP139A1_2546206123_Mycob 269 GGRVPAADDLSALTYLNGVVHETLRLYSPGVISARRVLRDLWFDGHRIRAGRLLIFSAYVTHRLPEIWPE 338

CYP139A1_2574780327_Mycob 269 GGRVPAADDLSALTYLNGVVHETLRLYSPGVISARRVLRDLWFDGHRIRAGRLLIFSAYVTHRLPEIWPE 338

CYP139A1_2575978404_Mycob 269 GGRVPAADDLSALTYLNGVVHETLRLYSPGVISARRVLRDLWFDGHRIRAGRLLIFSAYVTHRLPEIWPE 338

CYP139A1_2576675825_Mycob 269 GGRVPAADDLSALTYLNGVVHETLRLYSPGVISARRVLRDLWFDGHRIRAGRLLIFSAYVTHRLPEIWPE 338

CYP139A1_2576947708_Mycob 269 GGRVPAADDLSALTYLNGVVHETLRLYSPGVISARRVLRDLWFDGHRIRAGRLLIFSAYVTHRLPEIWPE 338

CYP139A1_2577400922_Mycob 269 GGRVPAADDLSALTYLNGVVHETLRLYSPGVISARRVLRDLWFDGHRIRAGRLLIFSAYVTHRLPEIWPE 338

CYP139A1_2577893113_Mycob 269 GGRVPAADDLSALTYLNGVVHETLRLYSPGVISARRVLRDLWFDGHRIRAGRLLIFSAYVTHRLPEIWPE 338

CYP139A1_2584759228_Mycob 269 GGRVPAADDLSALTYLNGVVHETLRLYSPGVISARRVLRDLWFDGHRIRAGRLLIFSAYVTHRLPEIWPE 338

CYP139A1_2584801008_Mycob 269 GGRVPAADDLSALTYLNGVVHETLRLYSPGVISARRVLRDLWFDGHRIRAGRLLIFSAYVTHRLPEIWPE 338

CYP139A1_2584946269_Mycob 269 GGRVPAADDLSALTYLNGVVHETLRLYSPGVISARRVLRDLWFDGHRIRAGRLLIFSAYVTHRLPEIWPE 338

CYP139A1_2589125802_Mycob 269 GGRVPAADDLSALTYLNGVVHETLRLYSPGVISARRVLRDLWFDGHRIRAGRLLIFSAYVTHRLPEIWPE 338

CYP139A1_2589654545_Mycob 269 GGRVPAADDLSALTYLNGVVHETLRLYSPGVISARRVLRDLWFDGHRIRAGRLLIFSAYVTHRLPEIWPE 338

CYP139A1_2590190898_Mycob 269 GGRVPAADDLSALTYLNGVVHETLRLYSPGVISARRVLRDLWFDGHRIRAGRLLIFSAYVTHRLPEIWPE 338

CYP139A1_2592267285_Mycob 269 GGRVPAADDLSALTYLNGVVHETLRLYSPGVISARRVLRDLWFDGHRIRAGRLLIFSAYVTHRLPEIWPE 338

CYP139A1_2592579018_Mycob 269 GGRVPAADDLSALTYLNGVVHETLRLYSPGVISARRVLRDLWFDGHRIRAGRLLIFSAYVTHRLPEIWPE 338

CYP139A1_2574803240_Mycob 269 GGRVPAADDLSALTYLNGVVHETLRLYSPGVISARRVLRDLWFDGHRIRAGRLLIFSAYVTHRLPEIWPE 338

CYP139A1_2575138339_Mycob 269 GGRVPAADDLSALTYLNGVVHETLRLYSPGVISARRVLRDLWFDGHRIRAGRLLIFSAYVTHRLPEIWPE 338

CYP139A1_2575935659_Mycob 269 GGRVPAADDLSALTYLNGVVHETLRLYSPGVISARRVLRDLWFDGHRIRAGRLLIFSAYVTHRLPEIWPE 338

CYP139A1_2576703024_Mycob 269 GGRVPAADDLSALTYLNGVVHETLRLYSPGVISARRVLRDLWFDGHRIRAGRLLIFSAYVTHRLPEIWPE 338

CYP139A1_2577143911_Mycob 269 GGRVPAADDLSALTYLNGVVHETLRLYSPGVISARRVLRDLWFDGHRIRAGRLLIFSAYVTHRLPEIWPE 338

CYP139A1_2577175183_Mycob 269 GGRVPAADDLSALTYLNGVVHETLRLYSPGVISARRVLRDLWFDGHRIRAGRLLIFSAYVTHRLPEIWPE 338

CYP139A1_2577879644_Mycob 269 GGRVPAADDLSALTYLNGVVHETLRLYSPGVISARRVLRDLWFDGHRIRAGRLLIFSAYVTHRLPEIWPE 338

CYP139A1_2577954418_Mycob 269 GGRVPAADDLSALTYLNGVVHETLRLYSPGVISARRVLRDLWFDGHRIRAGRLLIFSAYVTHRLPEIWPE 338

CYP139A1_2584625495_Mycob 269 GGRVPAADDLSALTYLNGVVHETLRLYSPGVISARRVLRDLWFDGHRIRAGRLLIFSAYVTHRLPEIWPE 338

CYP139A1_2589526877_Mycob 269 GGRVPAADDLSALTYLNGVVHETLRLYSPGVISARRVLRDLWFDGHRIRAGRLLIFSAYVTHRLPEIWPE 338

CYP139A1_2589711853_Mycob 269 GGRVPAADDLSALTYLNGVVHETLRLYSPGVISARRVLRDLWFDGHRIRAGRLLIFSAYVTHRLPEIWPE 338

CYP139A1_2590113796_Mycob 269 GGRVPAADDLSALTYLNGVVHETLRLYSPGVISARRVLRDLWFDGHRIRAGRLLIFSAYVTHRLPEIWPE 338

CYP139A1_2592324422_Mycob 269 GGRVPAADDLSALTYLNGVVHETLRLYSPGVISARRVLRDLWFDGHRIRAGRLLIFSAYVTHRLPEIWPE 338

CYP139A1_2592337997_Mycob 269 GGRVPAADDLSALTYLNGVVHETLRLYSPGVISARRVLRDLWFDGHRIRAGRLLIFSAYVTHRLPEIWPE 338

CYP139A1_2592373455_Mycob 269 GGRVPAADDLSALTYLNGVVHETLRLYSPGVISARRVLRDLWFDGHRIRAGRLLIFSAYVTHRLPEIWPE 338

CYP139A1_2592445915_Mycob 269 GGRVPAADDLSALTYLNGVVHETLRLYSPGVISARRVLRDLWFDGHRIRAGRLLIFSAYVTHRLPEIWPE 338

CYP139A1_2575023271_Mycob 269 GGRVPAADDLSALTYLNGVVHETLRLYSPGVISARRVLRDLWFDGHRIRAGRLLIFSAYVTHRLPEIWPE 338

CYP139A1_2575619239_Mycob 269 GGRVPAADDLSALTYLNGVVHETLRLYSPGVISARRVLRDLWFDGHRIRAGRLLIFSAYVTHRLPEIWPE 338

CYP139A1_2575786887_Mycob 269 GGRVPAADDLSALTYLNGVVHETLRLYSPGVISARRVLRDLWFDGHRIRAGRLLIFSAYVTHRLPEIWPE 338

CYP139A1_2576882264_Mycob 269 GGRVPAADDLSALTYLNGVVHETLRLYSPGVISARRVLRDLWFDGHRIRAGRLLIFSAYVTHRLPEIWPE 338

CYP139A1_2577215885_Mycob 269 GGRVPAADDLSALTYLNGVVHETLRLYSPGVISARRVLRDLWFDGHRIRAGRLLIFSAYVTHRLPEIWPE 338

CYP139A1_2577627248_Mycob 269 GGRVPAADDLSALTYLNGVVHETLRLYSPGVISARRVLRDLWFDGHRIRAGRLLIFSAYVTHRLPEIWPE 338

CYP139A1_2577923998_Mycob 269 GGRVPAADDLSALTYLNGVVHETLRLYSPGVISARRVLRDLWFDGHRIRAGRLLIFSAYVTHRLPEIWPE 338

CYP139A1_2583735989_Mycob 269 GGRVPAADDLSALTYLNGVVHETLRLYSPGVISARRVLRDLWFDGHRIRAGRLLIFSAYVTHRLPEIWPE 338

CYP139A1_2584003651_Mycob 269 GGRVPAADDLSALTYLNGVVHETLRLYSPGVISARRVLRDLWFDGHRIRAGRLLIFSAYVTHRLPEIWPE 338

CYP139A1_2584623655_Mycob 269 GGRVPAADDLSALTYLNGVVHETLRLYSPGVISARRVLRDLWFDGHRIRAGRLLIFSAYVTHRLPEIWPE 338

CYP139A1_2584739857_Mycob 269 GGRVPAADDLSALTYLNGVVHETLRLYSPGVISARRVLRDLWFDGHRIRAGRLLIFSAYVTHRLPEIWPE 338

CYP139A1_2584858071_Mycob 269 GGRVPAADDLSALTYLNGVVHETLRLYSPGVISARRVLRDLWFDGHRIRAGRLLIFSAYVTHRLPEIWPE 338

CYP139A1_2584928246_Mycob 269 GGRVPAADDLSALTYLNGVVHETLRLYSPGVISARRVLRDLWFDGHRIRAGRLLIFSAYVTHRLPEIWPE 338

CYP139A1_2589040293_Mycob 269 GGRVPAADDLSALTYLNGVVHETLRLYSPGVISARRVLRDLWFDGHRIRAGRLLIFSAYVTHRLPEIWPE 338

CYP139A1_2589658610_Mycob 269 GGRVPAADDLSALTYLNGVVHETLRLYSPGVISARRVLRDLWFDGHRIRAGRLLIFSAYVTHRLPEIWPE 338

CYP139A1_2590162679_Mycob 269 GGRVPAADDLSALTYLNGVVHETLRLYSPGVISARRVLRDLWFDGHRIRAGRLLIFSAYVTHRLPEIWPE 338

CYP139A1_2590243884_Mycob 269 GGRVPAADDLSALTYLNGVVHETLRLYSPGVISARRVLRDLWFDGHRIRAGRLLIFSAYVTHRLPEIWPE 338

CYP139A1_2590531681_Mycob 269 GGRVPAADDLSALTYLNGVVHETLRLYSPGVISARRVLRDLWFDGHRIRAGRLLIFSAYVTHRLPEIWPE 338

CYP139A1_2574614619_Mycob 269 GGRVPAADDLSALTYLNGVVHETLRLYSPGVISARRVLRDLWFDGHRIRAGRLLIFSAYVTHRLPEIWPE 338

CYP139A1_2575016195_Mycob 269 GGRVPAADDLSALTYLNGVVHETLRLYSPGVISARRVLRDLWFDGHRIRAGRLLIFSAYVTHRLPEIWPE 338

CYP139A1_2575426848_Mycob 269 GGRVPAADDLSALTYLNGVVHETLRLYSPGVISARRVLRDLWFDGHRIRAGRLLIFSAYVTHRLPEIWPE 338

CYP139A1_2576630848_Mycob 269 GGRVPAADDLSALTYLNGVVHETLRLYSPGVISARRVLRDLWFDGHRIRAGRLLIFSAYVTHRLPEIWPE 338

CYP139A1_2577468911_Mycob 269 GGRVPAADDLSALTYLNGVVHETLRLYSPGVISARRVLRDLWFDGHRIRAGRLLIFSAYVTHRLPEIWPE 338

CYP139A1_2577974906_Mycob 269 GGRVPAADDLSALTYLNGVVHETLRLYSPGVISARRVLRDLWFDGHRIRAGRLLIFSAYVTHRLPEIWPE 338

CYP139A1_2578062326_Mycob 269 GGRVPAADDLSALTYLNGVVHETLRLYSPGVISARRVLRDLWFDGHRIRAGRLLIFSAYVTHRLPEIWPE 338

CYP139A1_2584836956_Mycob 269 GGRVPAADDLSALTYLNGVVHETLRLYSPGVISARRVLRDLWFDGHRIRAGRLLIFSAYVTHRLPEIWPE 338

CYP139A1_2584998642_Mycob 269 GGRVPAADDLSALTYLNGVVHETLRLYSPGVISARRVLRDLWFDGHRIRAGRLLIFSAYVTHRLPEIWPE 338

CYP139A1_2589130082_Mycob 269 GGRVPAADDLSALTYLNGVVHETLRLYSPGVISARRVLRDLWFDGHRIRAGRLLIFSAYVTHRLPEIWPE 338

CYP139A1_2589592089_Mycob 269 GGRVPAADDLSALTYLNGVVHETLRLYSPGVISARRVLRDLWFDGHRIRAGRLLIFSAYVTHRLPEIWPE 338

CYP139A1_2590052832_Mycob 269 GGRVPAADDLSALTYLNGVVHETLRLYSPGVISARRVLRDLWFDGHRIRAGRLLIFSAYVTHRLPEIWPE 338

CYP139A1_2590505688_Mycob 269 GGRVPAADDLSALTYLNGVVHETLRLYSPGVISARRVLRDLWFDGHRIRAGRLLIFSAYVTHRLPEIWPE 338

CYP139A1_2592353030_Mycob 269 GGRVPAADDLSALTYLNGVVHETLRLYSPGVISARRVLRDLWFDGHRIRAGRLLIFSAYVTHRLPEIWPE 338

CYP139A1_637026884_Mycoba 269 GGRVPAADDLSALTYLNGVVHETLRLYSPGVISARRVLRDLWFDGHRIRAGRLLIFSAYVTHRLPEIWPE 338

CYP139A1_2574886309_Mycob 269 GGRVPAADDLSALTYLNGVVHETLRLYSPGVISARRVLRDLWFDGHRIRAGRLLIFSAYVTHRLPEIWPE 338

CYP139A1_2575295342_Mycob 269 GGRVPAADDLSALTYLNGVVHETLRLYSPGVISARRVLRDLWFDGHRIRAGRLLIFSAYVTHRLPEIWPE 338

CYP139A1_2575942274_Mycob 269 GGRVPAADDLSALTYLNGVVHETLRLYSPGVISARRVLRDLWFDGHRIRAGRLLIFSAYVTHRLPEIWPE 338

CYP139A1_2576123248_Mycob 269 GGRVPAADDLSALTYLNGVVHETLRLYSPGVISARRVLRDLWFDGHRIRAGRLLIFSAYVTHRLPEIWPE 338

CYP139A1_2576712596_Mycob 269 GGRVPAADDLSALTYLNGVVHETLRLYSPGVISARRVLRDLWFDGHRIRAGRLLIFSAYVTHRLPEIWPE 338

CYP139A1_2577856904_Mycob 269 GGRVPAADDLSALTYLNGVVHETLRLYSPGVISARRVLRDLWFDGHRIRAGRLLIFSAYVTHRLPEIWPE 338

CYP139A1_2578013153_Mycob 269 GGRVPAADDLSALTYLNGVVHETLRLYSPGVISARRVLRDLWFDGHRIRAGRLLIFSAYVTHRLPEIWPE 338

CYP139A1_2578182623_Mycob 269 GGRVPAADDLSALTYLNGVVHETLRLYSPGVISARRVLRDLWFDGHRIRAGRLLIFSAYVTHRLPEIWPE 338

CYP139A1_2580771058_Mycob 269 GGRVPAADDLSALTYLNGVVHETLRLYSPGVISARRVLRDLWFDGHRIRAGRLLIFSAYVTHRLPEIWPE 338

CYP139A1_2584641128_Mycob 269 GGRVPAADDLSALTYLNGVVHETLRLYSPGVISARRVLRDLWFDGHRIRAGRLLIFSAYVTHRLPEIWPE 338

CYP139A1_2584649146_Mycob 269 GGRVPAADDLSALTYLNGVVHETLRLYSPGVISARRVLRDLWFDGHRIRAGRLLIFSAYVTHRLPEIWPE 338

CYP139A1_2584660713_Mycob 269 GGRVPAADDLSALTYLNGVVHETLRLYSPGVISARRVLRDLWFDGHRIRAGRLLIFSAYVTHRLPEIWPE 338

CYP139A1_2584967006_Mycob 269 GGRVPAADDLSALTYLNGVVHETLRLYSPGVISARRVLRDLWFDGHRIRAGRLLIFSAYVTHRLPEIWPE 338

CYP139A1_2584970624_Mycob 269 GGRVPAADDLSALTYLNGVVHETLRLYSPGVISARRVLRDLWFDGHRIRAGRLLIFSAYVTHRLPEIWPE 338

CYP139A1_2589053585_Mycob 269 GGRVPAADDLSALTYLNGVVHETLRLYSPGVISARRVLRDLWFDGHRIRAGRLLIFSAYVTHRLPEIWPE 338

CYP139A1_2589498327_Mycob 269 GGRVPAADDLSALTYLNGVVHETLRLYSPGVISARRVLRDLWFDGHRIRAGRLLIFSAYVTHRLPEIWPE 338

CYP139A1_2589563529_Mycob 269 GGRVPAADDLSALTYLNGVVHETLRLYSPGVISARRVLRDLWFDGHRIRAGRLLIFSAYVTHRLPEIWPE 338

CYP139A1_2590377218_Mycob 269 GGRVPAADDLSALTYLNGVVHETLRLYSPGVISARRVLRDLWFDGHRIRAGRLLIFSAYVTHRLPEIWPE 338

CYP139A1_2592283614_Mycob 269 GGRVPAADDLSALTYLNGVVHETLRLYSPGVISARRVLRDLWFDGHRIRAGRLLIFSAYVTHRLPEIWPE 338

CYP139A1_2592319784_Mycob 269 GGRVPAADDLSALTYLNGVVHETLRLYSPGVISARRVLRDLWFDGHRIRAGRLLIFSAYVTHRLPEIWPE 338

CYP139A1_2575106637_Mycob 269 GGRVPAADDLSALTYLNGVVHETLRLYSPGVISARRVLRDLWFDGHRIRAGRLLIFSAYVTHRLPEIWPE 338

CYP139A1_2575157076_Mycob 269 GGRVPAADDLSALTYLNGVVHETLRLYSPGVISARRVLRDLWFDGHRIRAGRLLIFSAYVTHRLPEIWPE 338

CYP139A1_2575361778_Mycob 269 GGRVPAADDLSALTYLNGVVHETLRLYSPGVISARRVLRDLWFDGHRIRAGRLLIFSAYVTHRLPEIWPE 338

CYP139A1_2576009184_Mycob 269 GGRVPAADDLSALTYLNGVVHETLRLYSPGVISARRVLRDLWFDGHRIRAGRLLIFSAYVTHRLPEIWPE 338

CYP139A1_2576566954_Mycob 269 GGRVPAADDLSALTYLNGVVHETLRLYSPGVISARRVLRDLWFDGHRIRAGRLLIFSAYVTHRLPEIWPE 338

CYP139A1_2576731741_Mycob 269 GGRVPAADDLSALTYLNGVVHETLRLYSPGVISARRVLRDLWFDGHRIRAGRLLIFSAYVTHRLPEIWPE 338

CYP139A1_2581562358_Mycob 269 GGRVPAADDLSALTYLNGVVHETLRLYSPGVISARRVLRDLWFDGHRIRAGRLLIFSAYVTHRLPEIWPE 338

CYP139A1_2584107430_Mycob 269 GGRVPAADDLSALTYLNGVVHETLRLYSPGVISARRVLRDLWFDGHRIRAGRLLIFSAYVTHRLPEIWPE 338

CYP139A1_2590025444_Mycob 269 GGRVPAADDLSALTYLNGVVHETLRLYSPGVISARRVLRDLWFDGHRIRAGRLLIFSAYVTHRLPEIWPE 338

CYP139A1_2590040558_Mycob 269 GGRVPAADDLSALTYLNGVVHETLRLYSPGVISARRVLRDLWFDGHRIRAGRLLIFSAYVTHRLPEIWPE 338

CYP139A1_2590214646_Mycob 269 GGRVPAADDLSALTYLNGVVHETLRLYSPGVISARRVLRDLWFDGHRIRAGRLLIFSAYVTHRLPEIWPE 338

CYP139A1_2590223019_Mycob 269 GGRVPAADDLSALTYLNGVVHETLRLYSPGVISARRVLRDLWFDGHRIRAGRLLIFSAYVTHRLPEIWPE 338

CYP139A1_2590266966_Mycob 269 GGRVPAADDLSALTYLNGVVHETLRLYSPGVISARRVLRDLWFDGHRIRAGRLLIFSAYVTHRLPEIWPE 338

CYP139A1_647086307_Mycoba 269 GGRVPAADDLSALTYLNGVVHETLRLYSPGVISARRVLRDLWFDGHRIRAGRLLIFSAYVTHRLPEIWPE 338

CYP139A1_2574726119_Mycob 269 GGRVPAADDLSALTYLNGVVHETLRLYSPGVISARRVLRDLWFDGHRIRAGRLLIFSAYVTHRLPEIWPE 338

CYP139A1_2574757270_Mycob 269 GGRVPAADDLSALTYLNGVVHETLRLYSPGVISARRVLRDLWFDGHRIRAGRLLIFSAYVTHRLPEIWPE 338

CYP139A1_2575280304_Mycob 269 GGRVPAADDLSALTYLNGVVHETLRLYSPGVISARRVLRDLWFDGHRIRAGRLLIFSAYVTHRLPEIWPE 338

CYP139A1_2575601683_Mycob 269 GGRVPAADDLSALTYLNGVVHETLRLYSPGVISARRVLRDLWFDGHRIRAGRLLIFSAYVTHRLPEIWPE 338

CYP139A1_2576158036_Mycob 269 GGRVPAADDLSALTYLNGVVHETLRLYSPGVISARRVLRDLWFDGHRIRAGRLLIFSAYVTHRLPEIWPE 338

CYP139A1_2577689111_Mycob 269 GGRVPAADDLSALTYLNGVVHETLRLYSPGVISARRVLRDLWFDGHRIRAGRLLIFSAYVTHRLPEIWPE 338

CYP139A1_2577751179_Mycob 269 GGRVPAADDLSALTYLNGVVHETLRLYSPGVISARRVLRDLWFDGHRIRAGRLLIFSAYVTHRLPEIWPE 338

CYP139A1_2577845812_Mycob 269 GGRVPAADDLSALTYLNGVVHETLRLYSPGVISARRVLRDLWFDGHRIRAGRLLIFSAYVTHRLPEIWPE 338

CYP139A1_2577900964_Mycob 269 GGRVPAADDLSALTYLNGVVHETLRLYSPGVISARRVLRDLWFDGHRIRAGRLLIFSAYVTHRLPEIWPE 338

CYP139A1_2577988240_Mycob 269 GGRVPAADDLSALTYLNGVVHETLRLYSPGVISARRVLRDLWFDGHRIRAGRLLIFSAYVTHRLPEIWPE 338

CYP139A1_2578237814_Mycob 269 GGRVPAADDLSALTYLNGVVHETLRLYSPGVISARRVLRDLWFDGHRIRAGRLLIFSAYVTHRLPEIWPE 338

CYP139A1_2584703088_Mycob 269 GGRVPAADDLSALTYLNGVVHETLRLYSPGVISARRVLRDLWFDGHRIRAGRLLIFSAYVTHRLPEIWPE 338

CYP139A1_2584776403_Mycob 269 GGRVPAADDLSALTYLNGVVHETLRLYSPGVISARRVLRDLWFDGHRIRAGRLLIFSAYVTHRLPEIWPE 338

CYP139A1_2584785697_Mycob 269 GGRVPAADDLSALTYLNGVVHETLRLYSPGVISARRVLRDLWFDGHRIRAGRLLIFSAYVTHRLPEIWPE 338
[truncated: 224,598 more chars]
