# Supplementary material for: Cytochrome P450 Monooxygenase CYP139 Family Involved in the Synthesis of Secondary Metabolites in 824 Mycobacterial Species
Source: Int J Mol Sci. 2019 May 31;20(11):2690. doi: 10.3390/ijms20112690 (PMC6600245; doi:10.3390/ijms20112690)
Supplement: Supplementary file 1 [file ijms-20-02690-s001.zip › Supplementary Information/Supplementary Information.docx]

*Article*

**Cytochrome P450 monooxygenase CYP139 family involved in the synthesis of secondary metabolites in 824 mycobacterial species**

**Puleng Rosinah Syed ^1^, Wanping Chen ^2^, David R Nelson ^3^, Abidemi Paul Kappo ^4^, Jae-Hyuk Yu ^5,6^, Rajshekhar Karpoormath ^1^*, Khajamohiddin Syed ^4,^***

^1^ Department of Pharmaceutical Chemistry, College of Health Sciences, University of KwaZulu-Natal, Durban 4000, South Africa; prosinah@gmail.com (P.R.S.); Karpoormath@ukzn.ac.za (R.K.)

^2^ College of Food Science and Technology, Huazhong Agricultural University, Wuhan 430070, Hubei Province, China; chenwanping@mail.hzau.edu.cn

^3^ Department of Microbiology, Immunology and Biochemistry, University of Tennessee Health Science Center, Memphis, TN, 38163; drnelson1@gmail.com

^4^ Department of Biochemistry and Microbiology, Faculty of Science and Agriculture, University of Zululand, KwaDlangezwa 3886, South Africa; KappoA@unizulu.ac.za (A.P.K.); khajamohiddinsyed@gmail.com (K.S.)

^5^ Department of Bacteriology, University of Wisconsin-Madison, 3155 MSB, 1550 Linden Drive, Madison, WI 53706, USA; [jyu1@wisc.edu](mailto:jyu1@wisc.edu)

^6^ Department of Systems Biotechnology, Konkuk University, Seoul, 05029, Republic of Korea

**Table S1.Mycobacterial species used in this study.** Species Genome ID’s and their respective genome database links were also presented in the table.

| **Number** | **Species name** | **Genome ID** | **Genome database link** |
| --- | --- | --- | --- |
| ***Mycobacterium tuberculosis* complex (MTBC)** | | | |
|  | *Mycobacterium tuberculosis* BT2 | 2565956582 | https://img.jgi.doe.gov/cgi-bin/m/main.cgi?section=TaxonDetail&page=taxonDetail&taxon_oid=2565956582 |
|  | *Mycobacterium tuberculosis* PanR0604 | 2554235249 | https://img.jgi.doe.gov/cgi-bin/m/main.cgi?section=TaxonDetail&page=taxonDetail&taxon_oid=2554235249 |
|  | *Mycobacterium tuberculosis* M1283 | 2576861262 | https://img.jgi.doe.gov/cgi-bin/m/main.cgi?section=TaxonDetail&page=taxonDetail&taxon_oid=2576861262 |
|  | *Mycobacterium tuberculosis* TKK_02_0022 | 2590828642 | https://img.jgi.doe.gov/cgi-bin/m/main.cgi?section=TaxonDetail&page=taxonDetail&taxon_oid=2590828642 |
|  | *Mycobacterium tuberculosis* CDC1551A | 2537561546 | https://img.jgi.doe.gov/cgi-bin/m/main.cgi?section=TaxonDetail&page=taxonDetail&taxon_oid=2537561546 |
|  | *Mycobacterium bovis* Wt 21419 | 2579778875 | https://img.jgi.doe.gov/cgi-bin/m/main.cgi?section=TaxonDetail&page=taxonDetail&taxon_oid=2579778875 |
|  | *Mycobacterium tuberculosis* KZN R506 | 648276692 | https://img.jgi.doe.gov/cgi-bin/m/main.cgi?section=TaxonDetail&page=taxonDetail&taxon_oid=648276692 |
|  | *Mycobacterium tuberculosis* M1415 | 2582581220 | https://img.jgi.doe.gov/cgi-bin/m/main.cgi?section=TaxonDetail&page=taxonDetail&taxon_oid=2582581220 |
|  | *Mycobacterium tuberculosis* TRUG0117 | 2582581221 | https://img.jgi.doe.gov/cgi-bin/m/main.cgi?section=TaxonDetail&page=taxonDetail&taxon_oid=2582581221 |
|  | *Mycobacterium tuberculosis* TKK_05MA_0033 | 2576861202 | https://img.jgi.doe.gov/cgi-bin/m/main.cgi?section=TaxonDetail&page=taxonDetail&taxon_oid=2576861202 |
|  | *Mycobacterium bovis* BCG Tokyo 172 | 643692028 | https://img.jgi.doe.gov/cgi-bin/m/main.cgi?section=TaxonDetail&page=taxonDetail&taxon_oid=643692028 |
|  | *Mycobacterium tuberculosis* TKK_02_0016 | 2590828647 | https://img.jgi.doe.gov/cgi-bin/m/main.cgi?section=TaxonDetail&page=taxonDetail&taxon_oid=2590828647 |
|  | *Mycobacterium tuberculosis* TBR5 | 2588253868 | https://img.jgi.doe.gov/cgi-bin/m/main.cgi?section=TaxonDetail&page=taxonDetail&taxon_oid=2588253868 |
|  | *Mycobacterium tuberculosis* TKK_04_0003 | 2574180319 | https://img.jgi.doe.gov/cgi-bin/m/main.cgi?section=TaxonDetail&page=taxonDetail&taxon_oid=2574180319 |
|  | *Mycobacterium* sp. MOTT36Y | 2518645550 | https://img.jgi.doe.gov/cgi-bin/m/main.cgi?section=TaxonDetail&page=taxonDetail&taxon_oid=2518645550 |
|  | *Mycobacterium mageritense* JR2009 | 2548876798 | https://img.jgi.doe.gov/cgi-bin/m/main.cgi?section=TaxonDetail&page=taxonDetail&taxon_oid=2548876798 |
|  | *Mycobacterium tuberculosis* PanR0402 | 2554235300 | https://img.jgi.doe.gov/cgi-bin/m/main.cgi?section=TaxonDetail&page=taxonDetail&taxon_oid=2554235300 |
|  | *Mycobacterium tuberculosis* TRUG0106 | 2582581172 | https://img.jgi.doe.gov/cgi-bin/m/main.cgi?section=TaxonDetail&page=taxonDetail&taxon_oid=2582581172 |
|  | *Mycobacterium tuberculosis* TRUG0107 | 2574180353 | https://img.jgi.doe.gov/cgi-bin/m/main.cgi?section=TaxonDetail&page=taxonDetail&taxon_oid=2574180353 |
|  | *Mycobacterium tuberculosis* XTB13-229 | 2574180114 | https://img.jgi.doe.gov/cgi-bin/m/main.cgi?section=TaxonDetail&page=taxonDetail&taxon_oid=2574180114 |
|  | *Mycobacterium tuberculosis* MAL010078 | 2588254096 | https://img.jgi.doe.gov/cgi-bin/m/main.cgi?section=TaxonDetail&page=taxonDetail&taxon_oid=2588254096 |
|  | *Mycobacterium tuberculosis* TBR59 | 2588253887 | https://img.jgi.doe.gov/cgi-bin/m/main.cgi?section=TaxonDetail&page=taxonDetail&taxon_oid=2588253887 |
|  | *Mycobacterium tuberculosis* TB_RSA06 | 2574180201 | https://img.jgi.doe.gov/cgi-bin/m/main.cgi?section=TaxonDetail&page=taxonDetail&taxon_oid=2574180201 |
|  | *Mycobacterium tuberculosis* OFXR-19 | 2576861001 | https://img.jgi.doe.gov/cgi-bin/m/main.cgi?section=TaxonDetail&page=taxonDetail&taxon_oid=2576861001 |
|  | *Mycobacterium tuberculosis* MAL020182 | 2588254123 | https://img.jgi.doe.gov/cgi-bin/m/main.cgi?section=TaxonDetail&page=taxonDetail&taxon_oid=2588254123 |
|  | *Mycobacterium tuberculosis* TKK-01-0067 | 2588254024 | https://img.jgi.doe.gov/cgi-bin/m/main.cgi?section=TaxonDetail&page=taxonDetail&taxon_oid=2588254024 |
|  | *Mycobacterium tuberculosis* TKK-01-0092 | 2588254036 | https://img.jgi.doe.gov/cgi-bin/m/main.cgi?section=TaxonDetail&page=taxonDetail&taxon_oid=2588254036 |
|  | *Mycobacterium tuberculosis* TKK_02_0052 | 2590828634 | https://img.jgi.doe.gov/cgi-bin/m/main.cgi?section=TaxonDetail&page=taxonDetail&taxon_oid=2590828634 |
|  | *Mycobacterium tuberculosis* KT-0017 | 2588254156 | https://img.jgi.doe.gov/cgi-bin/m/main.cgi?section=TaxonDetail&page=taxonDetail&taxon_oid=2588254156 |
|  | *Mycobacterium tuberculosis* OFXR-17 | 2588254187 | https://img.jgi.doe.gov/cgi-bin/m/main.cgi?section=TaxonDetail&page=taxonDetail&taxon_oid=2588254187 |
|  | *Mycobacterium tuberculosis* 7199-99 | 2540341146 | https://img.jgi.doe.gov/cgi-bin/m/main.cgi?section=TaxonDetail&page=taxonDetail&taxon_oid=2540341146 |
|  | *Mycobacterium tuberculosis* OFXR-25 | 2588254181 | https://img.jgi.doe.gov/cgi-bin/m/main.cgi?section=TaxonDetail&page=taxonDetail&taxon_oid=2588254181 |
|  | *Mycobacterium tuberculosis* MAL020131 | 2574179850 | https://img.jgi.doe.gov/cgi-bin/m/main.cgi?section=TaxonDetail&page=taxonDetail&taxon_oid=2574179850 |
|  | *Mycobacterium tuberculosis* TKK-01-0008 | 2588253844 | https://img.jgi.doe.gov/cgi-bin/m/main.cgi?section=TaxonDetail&page=taxonDetail&taxon_oid=2588253844 |
|  | *Mycobacterium tuberculosis* MAL020179 | 2574180380 | https://img.jgi.doe.gov/cgi-bin/m/main.cgi?section=TaxonDetail&page=taxonDetail&taxon_oid=2574180380 |
|  | *Mycobacterium massiliense* 2B-0626 | 2526164657 | https://img.jgi.doe.gov/cgi-bin/m/main.cgi?section=TaxonDetail&page=taxonDetail&taxon_oid=2526164657 |
|  | *Mycobacterium africanum* MAL010111 | 2574180196 | https://img.jgi.doe.gov/cgi-bin/m/main.cgi?section=TaxonDetail&page=taxonDetail&taxon_oid=2574180196 |
|  | *Mycobacterium tuberculosis* OFXR-21 | 2588254184 | https://img.jgi.doe.gov/cgi-bin/m/main.cgi?section=TaxonDetail&page=taxonDetail&taxon_oid=2588254184 |
|  | *Mycobacterium africanum* K85 | 645951854 | https://img.jgi.doe.gov/cgi-bin/m/main.cgi?section=TaxonDetail&page=taxonDetail&taxon_oid=645951854 |
|  | *Mycobacterium tuberculosis* PanR0209 | 2554235214 | https://img.jgi.doe.gov/cgi-bin/m/main.cgi?section=TaxonDetail&page=taxonDetail&taxon_oid=2554235214 |
|  | *Mycobacterium tuberculosis* XTB13-198 | 2582581195 | https://img.jgi.doe.gov/cgi-bin/m/main.cgi?section=TaxonDetail&page=taxonDetail&taxon_oid=2582581195 |
|  | *Mycobacterium tuberculosis* PanR0410 | 2554235306 | https://img.jgi.doe.gov/cgi-bin/m/main.cgi?section=TaxonDetail&page=taxonDetail&taxon_oid=2554235306 |
|  | *Mycobacterium tuberculosis* BTB12-449 | 2576861060 | https://img.jgi.doe.gov/cgi-bin/m/main.cgi?section=TaxonDetail&page=taxonDetail&taxon_oid=2576861060 |
|  | *Mycobacterium tuberculosis* OFXR-4 | 2588253900 | https://img.jgi.doe.gov/cgi-bin/m/main.cgi?section=TaxonDetail&page=taxonDetail&taxon_oid=2588253900 |
|  | *Mycobacterium tuberculosis* PanR0309 | 2554235242 | https://img.jgi.doe.gov/cgi-bin/m/main.cgi?section=TaxonDetail&page=taxonDetail&taxon_oid=2554235242 |
|  | *Mycobacterium iranicum* UM_TJL | 2585427704 | https://img.jgi.doe.gov/cgi-bin/m/main.cgi?section=TaxonDetail&page=taxonDetail&taxon_oid=2585427704 |
|  | *Mycobacterium tuberculosis* TBR29 | 2588253874 | https://img.jgi.doe.gov/cgi-bin/m/main.cgi?section=TaxonDetail&page=taxonDetail&taxon_oid=2588253874 |
|  | *Mycobacterium tuberculosis* BTB06-001 | 2576861352 | https://img.jgi.doe.gov/cgi-bin/m/main.cgi?section=TaxonDetail&page=taxonDetail&taxon_oid=2576861352 |
|  | *Mycobacterium tuberculosis* TKK-01-0077 | 2574180051 | https://img.jgi.doe.gov/cgi-bin/m/main.cgi?section=TaxonDetail&page=taxonDetail&taxon_oid=2574180051 |
|  | *Mycobacterium tuberculosis* OFXR-15 | 2574180344 | https://img.jgi.doe.gov/cgi-bin/m/main.cgi?section=TaxonDetail&page=taxonDetail&taxon_oid=2574180344 |
|  | *Mycobacterium tuberculosis* TKK-01-0051 | 2576861218 | https://img.jgi.doe.gov/cgi-bin/m/main.cgi?section=TaxonDetail&page=taxonDetail&taxon_oid=2576861218 |
|  | *Mycobacterium tuberculosis* TKK_04_0132 | 2576861181 | https://img.jgi.doe.gov/cgi-bin/m/main.cgi?section=TaxonDetail&page=taxonDetail&taxon_oid=2576861181 |
|  | *Mycobacterium tuberculosis* SUMu009 | 648276701 | https://img.jgi.doe.gov/cgi-bin/m/main.cgi?section=TaxonDetail&page=taxonDetail&taxon_oid=648276701 |
|  | *Mycobacterium tuberculosis* PanR0602 | 2554235252 | https://img.jgi.doe.gov/cgi-bin/m/main.cgi?section=TaxonDetail&page=taxonDetail&taxon_oid=2554235252 |
|  | *Mycobacterium* sp. UNC267MFSha1.1M11 | 2593339259 | https://img.jgi.doe.gov/cgi-bin/m/main.cgi?section=TaxonDetail&page=taxonDetail&taxon_oid=2593339259 |
|  | *Mycobacterium tuberculosis* PanR0405 | 2554235292 | https://img.jgi.doe.gov/cgi-bin/m/main.cgi?section=TaxonDetail&page=taxonDetail&taxon_oid=2554235292 |
|  | *Mycobacterium tuberculosis* XTB13-255 | 2576861384 | https://img.jgi.doe.gov/cgi-bin/m/main.cgi?section=TaxonDetail&page=taxonDetail&taxon_oid=2576861384 |
|  | *Mycobacterium tuberculosis* TKK-01-0050 | 2588254005 | https://img.jgi.doe.gov/cgi-bin/m/main.cgi?section=TaxonDetail&page=taxonDetail&taxon_oid=2588254005 |
|  | *Mycobacterium tuberculosis* MD15974 | 2576861090 | https://img.jgi.doe.gov/cgi-bin/m/main.cgi?section=TaxonDetail&page=taxonDetail&taxon_oid=2576861090 |
|  | *Mycobacterium tuberculosis* TKK_04_0103 | 2574180277 | https://img.jgi.doe.gov/cgi-bin/m/main.cgi?section=TaxonDetail&page=taxonDetail&taxon_oid=2574180277 |
|  | *Mycobacterium tuberculosis* TRUG0037 | 2574180240 | https://img.jgi.doe.gov/cgi-bin/m/main.cgi?section=TaxonDetail&page=taxonDetail&taxon_oid=2574180240 |
|  | *Mycobacterium tuberculosis* XTB13-209 | 2574179941 | https://img.jgi.doe.gov/cgi-bin/m/main.cgi?section=TaxonDetail&page=taxonDetail&taxon_oid=2574179941 |
|  | *Mycobacterium tuberculosis* T92 | 642979311 | https://img.jgi.doe.gov/cgi-bin/m/main.cgi?section=TaxonDetail&page=taxonDetail&taxon_oid=642979311 |
|  | *Mycobacterium tuberculosis* TB_RSA01 | 2582581153 | https://img.jgi.doe.gov/cgi-bin/m/main.cgi?section=TaxonDetail&page=taxonDetail&taxon_oid=2582581153 |
|  | *Mycobacterium tuberculosis* CPHL_A | 2576861059 | https://img.jgi.doe.gov/cgi-bin/m/main.cgi?section=TaxonDetail&page=taxonDetail&taxon_oid=2576861059 |
|  | *Mycobacterium tuberculosis* BTB13-063 | 2574180264 | https://img.jgi.doe.gov/cgi-bin/m/main.cgi?section=TaxonDetail&page=taxonDetail&taxon_oid=2574180264 |
|  | *Mycobacterium xenopi* 4042 | 2565956798 | https://img.jgi.doe.gov/cgi-bin/m/main.cgi?section=TaxonDetail&page=taxonDetail&taxon_oid=2565956798 |
|  | *Mycobacterium tuberculosis* TBR35 | 2588253877 | https://img.jgi.doe.gov/cgi-bin/m/main.cgi?section=TaxonDetail&page=taxonDetail&taxon_oid=2588253877 |
|  | *Mycobacterium tuberculosis* XTB13-113 | 2582581179 | https://img.jgi.doe.gov/cgi-bin/m/main.cgi?section=TaxonDetail&page=taxonDetail&taxon_oid=2582581179 |
|  | *Mycobacterium tuberculosis* TB_RSA149 | 2576861030 | https://img.jgi.doe.gov/cgi-bin/m/main.cgi?section=TaxonDetail&page=taxonDetail&taxon_oid=2576861030 |
|  | *Mycobacterium tuberculosis* BTB03-144 | 2576861021 | https://img.jgi.doe.gov/cgi-bin/m/main.cgi?section=TaxonDetail&page=taxonDetail&taxon_oid=2576861021 |
|  | *Mycobacterium tuberculosis* MD15050 | 2582581211 | https://img.jgi.doe.gov/cgi-bin/m/main.cgi?section=TaxonDetail&page=taxonDetail&taxon_oid=2582581211 |
|  | *Mycobacterium tuberculosis* TB_RSA120 | 2576861148 | https://img.jgi.doe.gov/cgi-bin/m/main.cgi?section=TaxonDetail&page=taxonDetail&taxon_oid=2576861148 |
|  | *Mycobacterium tuberculosis* KT-0086 | 2574179857 | https://img.jgi.doe.gov/cgi-bin/m/main.cgi?section=TaxonDetail&page=taxonDetail&taxon_oid=2574179857 |
|  | *Mycobacterium tuberculosis* TKK_04_0082 | 2582581165 | https://img.jgi.doe.gov/cgi-bin/m/main.cgi?section=TaxonDetail&page=taxonDetail&taxon_oid=2582581165 |
|  | *Mycobacterium tuberculosis* GM 1503 | 2576861289 | https://img.jgi.doe.gov/cgi-bin/m/main.cgi?section=TaxonDetail&page=taxonDetail&taxon_oid=2576861289 |
|  | *Mycobacterium tuberculosis* EAI5/NITR206 | 2545824630 | https://img.jgi.doe.gov/cgi-bin/m/main.cgi?section=TaxonDetail&page=taxonDetail&taxon_oid=2545824630 |
|  | *Mycobacterium tuberculosis* MAL020208 | 2588254138 | https://img.jgi.doe.gov/cgi-bin/m/main.cgi?section=TaxonDetail&page=taxonDetail&taxon_oid=2588254138 |
|  | *Mycobacterium tuberculosis* TKK-01-0056 | 2588254017 | https://img.jgi.doe.gov/cgi-bin/m/main.cgi?section=TaxonDetail&page=taxonDetail&taxon_oid=2588254017 |
|  | *Mycobacterium tuberculosis* CTRI-2 | 2511231130 | https://img.jgi.doe.gov/cgi-bin/m/main.cgi?section=TaxonDetail&page=taxonDetail&taxon_oid=2511231130 |
|  | *Mycobacterium tuberculosis* TKK_02_0002 | 2582581175 | https://img.jgi.doe.gov/cgi-bin/m/main.cgi?section=TaxonDetail&page=taxonDetail&taxon_oid=2582581175 |
|  | *Mycobacterium tuberculosis* TKK_04_0005 | 2590828609 | https://img.jgi.doe.gov/cgi-bin/m/main.cgi?section=TaxonDetail&page=taxonDetail&taxon_oid=2590828609 |
|  | *Mycobacterium tuberculosis* TKK_04_0029 | 2590828682 | https://img.jgi.doe.gov/cgi-bin/m/main.cgi?section=TaxonDetail&page=taxonDetail&taxon_oid=2590828682 |
|  | *Mycobacterium tuberculosis* PanR0909 | 2554235274 | https://img.jgi.doe.gov/cgi-bin/m/main.cgi?section=TaxonDetail&page=taxonDetail&taxon_oid=2554235274 |
|  | *Mycobacterium tuberculosis* MD18478 | 2576861285 | https://img.jgi.doe.gov/cgi-bin/m/main.cgi?section=TaxonDetail&page=taxonDetail&taxon_oid=2576861285 |
|  | *Mycobacterium tuberculosis* TRUG0101 | 2574180401 | https://img.jgi.doe.gov/cgi-bin/m/main.cgi?section=TaxonDetail&page=taxonDetail&taxon_oid=2574180401 |
|  | *Mycobacterium tuberculosis* TBR7 | 2574180207 | https://img.jgi.doe.gov/cgi-bin/m/main.cgi?section=TaxonDetail&page=taxonDetail&taxon_oid=2574180207 |
|  | *Mycobacterium tuberculosis* TBR75 | 2588253891 | https://img.jgi.doe.gov/cgi-bin/m/main.cgi?section=TaxonDetail&page=taxonDetail&taxon_oid=2588253891 |
|  | *Mycobacterium tuberculosis* PanR0702 | 2554235271 | https://img.jgi.doe.gov/cgi-bin/m/main.cgi?section=TaxonDetail&page=taxonDetail&taxon_oid=2554235271 |
|  | *Mycobacterium tuberculosis* MAL020144 | 2574180408 | https://img.jgi.doe.gov/cgi-bin/m/main.cgi?section=TaxonDetail&page=taxonDetail&taxon_oid=2574180408 |
|  | *Mycobacterium tuberculosis* TKK-01-0083 | 2588254031 | https://img.jgi.doe.gov/cgi-bin/m/main.cgi?section=TaxonDetail&page=taxonDetail&taxon_oid=2588254031 |
|  | *Mycobacterium tuberculosis* MD17902 | 2574179965 | https://img.jgi.doe.gov/cgi-bin/m/main.cgi?section=TaxonDetail&page=taxonDetail&taxon_oid=2574179965 |
|  | *Mycobacterium tuberculosis* TKK_02_0046 | 2590828635 | https://img.jgi.doe.gov/cgi-bin/m/main.cgi?section=TaxonDetail&page=taxonDetail&taxon_oid=2590828635 |
|  | *Mycobacterium tuberculosis* TKK-01-0059 | 2588254012 | https://img.jgi.doe.gov/cgi-bin/m/main.cgi?section=TaxonDetail&page=taxonDetail&taxon_oid=2588254012 |
|  | *Mycobacterium tuberculosis* TKK_03_0026 | 2574179863 | https://img.jgi.doe.gov/cgi-bin/m/main.cgi?section=TaxonDetail&page=taxonDetail&taxon_oid=2574179863 |
|  | *Mycobacterium tuberculosis* BTB13-276 | 2585427841 | https://img.jgi.doe.gov/cgi-bin/m/main.cgi?section=TaxonDetail&page=taxonDetail&taxon_oid=2585427841 |
|  | *Mycobacterium tuberculosis* SK-C | 2590828653 | https://img.jgi.doe.gov/cgi-bin/m/main.cgi?section=TaxonDetail&page=taxonDetail&taxon_oid=2590828653 |
|  | *Mycobacterium tuberculosis* MAL020156 | 2588254119 | https://img.jgi.doe.gov/cgi-bin/m/main.cgi?section=TaxonDetail&page=taxonDetail&taxon_oid=2588254119 |
|  | *Mycobacterium tuberculosis* TKK-01-0064 | 2582581132 | https://img.jgi.doe.gov/cgi-bin/m/main.cgi?section=TaxonDetail&page=taxonDetail&taxon_oid=2582581132 |
|  | *Mycobacterium tuberculosis* 2094HD | 2576861074 | https://img.jgi.doe.gov/cgi-bin/m/main.cgi?section=TaxonDetail&page=taxonDetail&taxon_oid=2576861074 |
|  | *Mycobacterium tuberculosis* 1615 | 2576861307 | https://img.jgi.doe.gov/cgi-bin/m/main.cgi?section=TaxonDetail&page=taxonDetail&taxon_oid=2576861307 |
|  | *Mycobacterium tuberculosis* TKK-01-0022 | 2588253986 | https://img.jgi.doe.gov/cgi-bin/m/main.cgi?section=TaxonDetail&page=taxonDetail&taxon_oid=2588253986 |
|  | *Mycobacterium tuberculosis* TKK_03_0020 | 2590828623 | https://img.jgi.doe.gov/cgi-bin/m/main.cgi?section=TaxonDetail&page=taxonDetail&taxon_oid=2590828623 |
|  | *Mycobacterium tuberculosis* TKK_02_0073 | 2590828626 | https://img.jgi.doe.gov/cgi-bin/m/main.cgi?section=TaxonDetail&page=taxonDetail&taxon_oid=2590828626 |
|  | *Mycobacterium tuberculosis* CTRI-4 | 2547132221 | https://img.jgi.doe.gov/cgi-bin/m/main.cgi?section=TaxonDetail&page=taxonDetail&taxon_oid=2547132221 |
|  | *Mycobacterium africanum* MAL010102 | 2576861083 | https://img.jgi.doe.gov/cgi-bin/m/main.cgi?section=TaxonDetail&page=taxonDetail&taxon_oid=2576861083 |
|  | *Mycobacterium tuberculosis* BS1 | 2558860211 | https://img.jgi.doe.gov/cgi-bin/m/main.cgi?section=TaxonDetail&page=taxonDetail&taxon_oid=2558860211 |
|  | *Mycobacterium tuberculosis* TKK_04_0044 | 2574180195 | https://img.jgi.doe.gov/cgi-bin/m/main.cgi?section=TaxonDetail&page=taxonDetail&taxon_oid=2574180195 |
|  | *Mycobacterium tuberculosis* M1004 | 2576861107 | https://img.jgi.doe.gov/cgi-bin/m/main.cgi?section=TaxonDetail&page=taxonDetail&taxon_oid=2576861107 |
|  | *Mycobacterium tuberculosis* MAL020147 | 2588254116 | https://img.jgi.doe.gov/cgi-bin/m/main.cgi?section=TaxonDetail&page=taxonDetail&taxon_oid=2588254116 |
|  | *Mycobacterium canettii* CIPT 140070010 | 2540341073 | https://img.jgi.doe.gov/cgi-bin/m/main.cgi?section=TaxonDetail&page=taxonDetail&taxon_oid=2540341073 |
|  | *Mycobacterium tuberculosis* TB_RSA03 | 2574179837 | https://img.jgi.doe.gov/cgi-bin/m/main.cgi?section=TaxonDetail&page=taxonDetail&taxon_oid=2574179837 |
|  | *Mycobacterium tuberculosis* M2416 | 2574179873 | https://img.jgi.doe.gov/cgi-bin/m/main.cgi?section=TaxonDetail&page=taxonDetail&taxon_oid=2574179873 |
|  | *Mycobacterium bovis* BCG-Russia TMC 1022 , ATCC 35740 | 2547132087 | https://img.jgi.doe.gov/cgi-bin/m/main.cgi?section=TaxonDetail&page=taxonDetail&taxon_oid=2547132087 |
|  | *Mycobacterium tuberculosis* TBR28 | 2574180031 | https://img.jgi.doe.gov/cgi-bin/m/main.cgi?section=TaxonDetail&page=taxonDetail&taxon_oid=2574180031 |
|  | *Mycobacterium tuberculosis* MD16555 | 2582581192 | https://img.jgi.doe.gov/cgi-bin/m/main.cgi?section=TaxonDetail&page=taxonDetail&taxon_oid=2582581192 |
|  | *Mycobacterium tuberculosis* M1007 | 2574180366 | https://img.jgi.doe.gov/cgi-bin/m/main.cgi?section=TaxonDetail&page=taxonDetail&taxon_oid=2574180366 |
|  | *Mycobacterium massiliense* 2B-0912-R | 2526164655 | https://img.jgi.doe.gov/cgi-bin/m/main.cgi?section=TaxonDetail&page=taxonDetail&taxon_oid=2526164655 |
|  | *Mycobacterium tuberculosis* SUMu012 | 648276704 | https://img.jgi.doe.gov/cgi-bin/m/main.cgi?section=TaxonDetail&page=taxonDetail&taxon_oid=648276704 |
|  | *Mycobacterium tuberculosis* PanR0306 | 2554235244 | https://img.jgi.doe.gov/cgi-bin/m/main.cgi?section=TaxonDetail&page=taxonDetail&taxon_oid=2554235244 |
|  | *Mycobacterium tuberculosis* TRUG0004 | 2574179830 | https://img.jgi.doe.gov/cgi-bin/m/main.cgi?section=TaxonDetail&page=taxonDetail&taxon_oid=2574179830 |
|  | *Mycobacterium tuberculosis* BTB13-222 | 2574180330 | https://img.jgi.doe.gov/cgi-bin/m/main.cgi?section=TaxonDetail&page=taxonDetail&taxon_oid=2574180330 |
|  | *Mycobacterium tuberculosis* OFXR-30 | 2588254179 | https://img.jgi.doe.gov/cgi-bin/m/main.cgi?section=TaxonDetail&page=taxonDetail&taxon_oid=2588254179 |
|  | *Mycobacterium tuberculosis* TKK_04_0075 | 2574179902 | https://img.jgi.doe.gov/cgi-bin/m/main.cgi?section=TaxonDetail&page=taxonDetail&taxon_oid=2574179902 |
|  | *Mycobacterium* sp. UM_WWY | 2554235288 | https://img.jgi.doe.gov/cgi-bin/m/main.cgi?section=TaxonDetail&page=taxonDetail&taxon_oid=2554235288 |
|  | *Mycobacterium tuberculosis* HN878 | 2547132240 | https://img.jgi.doe.gov/cgi-bin/m/main.cgi?section=TaxonDetail&page=taxonDetail&taxon_oid=2547132240 |
|  | *Mycobacterium tuberculosis* H37Rv | 2526164708 | https://img.jgi.doe.gov/cgi-bin/m/main.cgi?section=TaxonDetail&page=taxonDetail&taxon_oid=2526164708 |
|  | *Mycobacterium tuberculosis* M1233 | 2574180118 | https://img.jgi.doe.gov/cgi-bin/m/main.cgi?section=TaxonDetail&page=taxonDetail&taxon_oid=2574180118 |
|  | *Mycobacterium tuberculosis* TBR4 | 2588253869 | https://img.jgi.doe.gov/cgi-bin/m/main.cgi?section=TaxonDetail&page=taxonDetail&taxon_oid=2588253869 |
|  | *Mycobacterium tuberculosis* TBR11 | 2588253870 | https://img.jgi.doe.gov/cgi-bin/m/main.cgi?section=TaxonDetail&page=taxonDetail&taxon_oid=2588253870 |
|  | *Mycobacterium tuberculosis* TKK-01-0019 | 2574180154 | https://img.jgi.doe.gov/cgi-bin/m/main.cgi?section=TaxonDetail&page=taxonDetail&taxon_oid=2574180154 |
|  | *Mycobacterium tuberculosis* KT-0084 | 2588254234 | https://img.jgi.doe.gov/cgi-bin/m/main.cgi?section=TaxonDetail&page=taxonDetail&taxon_oid=2588254234 |
|  | *Mycobacterium canettii* CIPT 140070005 | 2565956759 | https://img.jgi.doe.gov/cgi-bin/m/main.cgi?section=TaxonDetail&page=taxonDetail&taxon_oid=2565956759 |
|  | *Mycobacterium africanum* MAL020130 | 2582580878 | https://img.jgi.doe.gov/cgi-bin/m/main.cgi?section=TaxonDetail&page=taxonDetail&taxon_oid=2582580878 |
|  | *Mycobacterium tuberculosis* M1340 | 2582581131 | https://img.jgi.doe.gov/cgi-bin/m/main.cgi?section=TaxonDetail&page=taxonDetail&taxon_oid=2582581131 |
|  | *Mycobacterium tuberculosis* XTB13-161 | 2576861213 | https://img.jgi.doe.gov/cgi-bin/m/main.cgi?section=TaxonDetail&page=taxonDetail&taxon_oid=2576861213 |
|  | *Mycobacterium tuberculosis* TKK_05MA_0012 | 2576861094 | https://img.jgi.doe.gov/cgi-bin/m/main.cgi?section=TaxonDetail&page=taxonDetail&taxon_oid=2576861094 |
|  | *Mycobacterium bovis* Wt 21231 | 2582580936 | https://img.jgi.doe.gov/cgi-bin/m/main.cgi?section=TaxonDetail&page=taxonDetail&taxon_oid=2582580936 |
|  | *Mycobacterium tuberculosis* M2346 | 2582581206 | https://img.jgi.doe.gov/cgi-bin/m/main.cgi?section=TaxonDetail&page=taxonDetail&taxon_oid=2582581206 |
|  | *Mycobacterium tuberculosis* TB_RSA163 | 2576861005 | https://img.jgi.doe.gov/cgi-bin/m/main.cgi?section=TaxonDetail&page=taxonDetail&taxon_oid=2576861005 |
|  | *Mycobacterium tuberculosis* OFXR-7 | 2574180104 | https://img.jgi.doe.gov/cgi-bin/m/main.cgi?section=TaxonDetail&page=taxonDetail&taxon_oid=2574180104 |
|  | *Mycobacterium tuberculosis* H3361 | 2574179930 | https://img.jgi.doe.gov/cgi-bin/m/main.cgi?section=TaxonDetail&page=taxonDetail&taxon_oid=2574179930 |
|  | *Mycobacterium tuberculosis* TKK_04_0045 | 2582581189 | https://img.jgi.doe.gov/cgi-bin/m/main.cgi?section=TaxonDetail&page=taxonDetail&taxon_oid=2582581189 |
|  | *Mycobacterium tuberculosis* TKK-01-0054 | 2588254018 | https://img.jgi.doe.gov/cgi-bin/m/main.cgi?section=TaxonDetail&page=taxonDetail&taxon_oid=2588254018 |
|  | *Mycobacterium tuberculosis* MAL020196 | 2588254131 | https://img.jgi.doe.gov/cgi-bin/m/main.cgi?section=TaxonDetail&page=taxonDetail&taxon_oid=2588254131 |
|  | *Mycobacterium tuberculosis* KT-0035 | 2588254151 | https://img.jgi.doe.gov/cgi-bin/m/main.cgi?section=TaxonDetail&page=taxonDetail&taxon_oid=2588254151 |
|  | *Mycobacterium tuberculosis* NRITLD14 | 2576861298 | https://img.jgi.doe.gov/cgi-bin/m/main.cgi?section=TaxonDetail&page=taxonDetail&taxon_oid=2576861298 |
|  | *Mycobacterium tuberculosis* PanR0804 | 2554235265 | https://img.jgi.doe.gov/cgi-bin/m/main.cgi?section=TaxonDetail&page=taxonDetail&taxon_oid=2554235265 |
|  | *Mycobacterium tuberculosis* XTB13-092 | 2582581160 | https://img.jgi.doe.gov/cgi-bin/m/main.cgi?section=TaxonDetail&page=taxonDetail&taxon_oid=2582581160 |
|  | *Mycobacterium tuberculosis* TB_RSA111 | 2574179811 | https://img.jgi.doe.gov/cgi-bin/m/main.cgi?section=TaxonDetail&page=taxonDetail&taxon_oid=2574179811 |
|  | *Mycobacterium tuberculosis* TB_RSA59 | 2574180388 | https://img.jgi.doe.gov/cgi-bin/m/main.cgi?section=TaxonDetail&page=taxonDetail&taxon_oid=2574180388 |
|  | *Mycobacterium tuberculosis* BTB12-001 | 2574179928 | https://img.jgi.doe.gov/cgi-bin/m/main.cgi?section=TaxonDetail&page=taxonDetail&taxon_oid=2574179928 |
|  | *Mycobacterium tuberculosis* KT-0102 | 2588254228 | https://img.jgi.doe.gov/cgi-bin/m/main.cgi?section=TaxonDetail&page=taxonDetail&taxon_oid=2588254228 |
|  | *Mycobacterium tuberculosis* XDR1219 | 2558860629 | https://img.jgi.doe.gov/cgi-bin/m/main.cgi?section=TaxonDetail&page=taxonDetail&taxon_oid=2558860629 |
|  | *Mycobacterium tuberculosis* NRITLD56 | 2576861167 | https://img.jgi.doe.gov/cgi-bin/m/main.cgi?section=TaxonDetail&page=taxonDetail&taxon_oid=2576861167 |
|  | *Mycobacterium tuberculosis* H37Rv | 637000173 | https://img.jgi.doe.gov/cgi-bin/m/main.cgi?section=TaxonDetail&page=taxonDetail&taxon_oid=637000173 |
|  | *Mycobacterium tuberculosis* BTB09-565 | 2576861338 | https://img.jgi.doe.gov/cgi-bin/m/main.cgi?section=TaxonDetail&page=taxonDetail&taxon_oid=2576861338 |
|  | *Mycobacterium tuberculosis* RGTB423 | 2513237185 | https://img.jgi.doe.gov/cgi-bin/m/main.cgi?section=TaxonDetail&page=taxonDetail&taxon_oid=2513237185 |
|  | *Mycobacterium tuberculosis* TKK_02_0067 | 2590828630 | https://img.jgi.doe.gov/cgi-bin/m/main.cgi?section=TaxonDetail&page=taxonDetail&taxon_oid=2590828630 |
|  | *Mycobacterium tuberculosis* TBR80 | 2588253892 | https://img.jgi.doe.gov/cgi-bin/m/main.cgi?section=TaxonDetail&page=taxonDetail&taxon_oid=2588253892 |
|  | *Mycobacterium tuberculosis* TKK-01-0034 | 2588254002 | https://img.jgi.doe.gov/cgi-bin/m/main.cgi?section=TaxonDetail&page=taxonDetail&taxon_oid=2588254002 |
|  | *Mycobacterium tuberculosis* TKK_04_0038 | 2582581184 | https://img.jgi.doe.gov/cgi-bin/m/main.cgi?section=TaxonDetail&page=taxonDetail&taxon_oid=2582581184 |
|  | *Mycobacterium tuberculosis* TB_RSA118 | 2574180046 | https://img.jgi.doe.gov/cgi-bin/m/main.cgi?section=TaxonDetail&page=taxonDetail&taxon_oid=2574180046 |
|  | *Mycobacterium tuberculosis* MAL010130 | 2588254104 | https://img.jgi.doe.gov/cgi-bin/m/main.cgi?section=TaxonDetail&page=taxonDetail&taxon_oid=2588254104 |
|  | *Mycobacterium tuberculosis* PanR0805 | 2554235258 | https://img.jgi.doe.gov/cgi-bin/m/main.cgi?section=TaxonDetail&page=taxonDetail&taxon_oid=2554235258 |
|  | *Mycobacterium tuberculosis* KT-0109 | 2582581224 | https://img.jgi.doe.gov/cgi-bin/m/main.cgi?section=TaxonDetail&page=taxonDetail&taxon_oid=2582581224 |
|  | *Mycobacterium tuberculosis* XTB13-214 | 2576861314 | https://img.jgi.doe.gov/cgi-bin/m/main.cgi?section=TaxonDetail&page=taxonDetail&taxon_oid=2576861314 |
|  | *Mycobacterium tuberculosis* PanR0208 | 2554235211 | https://img.jgi.doe.gov/cgi-bin/m/main.cgi?section=TaxonDetail&page=taxonDetail&taxon_oid=2554235211 |
|  | *Mycobacterium tuberculosis* TKK-01-0025 | 2576861375 | https://img.jgi.doe.gov/cgi-bin/m/main.cgi?section=TaxonDetail&page=taxonDetail&taxon_oid=2576861375 |
|  | *Mycobacterium tuberculosis* TBR65 | 2574179889 | https://img.jgi.doe.gov/cgi-bin/m/main.cgi?section=TaxonDetail&page=taxonDetail&taxon_oid=2574179889 |
|  | *Mycobacterium tuberculosis* TKK_03_0022 | 2590828622 | https://img.jgi.doe.gov/cgi-bin/m/main.cgi?section=TaxonDetail&page=taxonDetail&taxon_oid=2590828622 |
|  | *Mycobacterium tuberculosis* PanR0403 | 2554235298 | https://img.jgi.doe.gov/cgi-bin/m/main.cgi?section=TaxonDetail&page=taxonDetail&taxon_oid=2554235298 |
|  | *Mycobacterium bovis* Mr 4387 | 2579778734 | https://img.jgi.doe.gov/cgi-bin/m/main.cgi?section=TaxonDetail&page=taxonDetail&taxon_oid=2579778734 |
|  | *Mycobacterium tuberculosis* TKK-01-0007 | 2588253979 | https://img.jgi.doe.gov/cgi-bin/m/main.cgi?section=TaxonDetail&page=taxonDetail&taxon_oid=2588253979 |
|  | *Mycobacterium tuberculosis* TB_RSA32 | 2576861325 | https://img.jgi.doe.gov/cgi-bin/m/main.cgi?section=TaxonDetail&page=taxonDetail&taxon_oid=2576861325 |
|  | *Mycobacterium tuberculosis* OFXR-20 | 2588254185 | https://img.jgi.doe.gov/cgi-bin/m/main.cgi?section=TaxonDetail&page=taxonDetail&taxon_oid=2588254185 |
|  | *Mycobacterium tuberculosis* KT-0056 | 2574180411 | https://img.jgi.doe.gov/cgi-bin/m/main.cgi?section=TaxonDetail&page=taxonDetail&taxon_oid=2574180411 |
|  | *Mycobacterium tuberculosis* TRUG0072 | 2582581141 | https://img.jgi.doe.gov/cgi-bin/m/main.cgi?section=TaxonDetail&page=taxonDetail&taxon_oid=2582581141 |
|  | *Mycobacterium tuberculosis* M1906 | 2574180244 | https://img.jgi.doe.gov/cgi-bin/m/main.cgi?section=TaxonDetail&page=taxonDetail&taxon_oid=2574180244 |
|  | *Mycobacterium tuberculosis* BTB12-384 | 2582581217 | https://img.jgi.doe.gov/cgi-bin/m/main.cgi?section=TaxonDetail&page=taxonDetail&taxon_oid=2582581217 |
|  | *Mycobacterium tuberculosis* TBR23 | 2588253873 | https://img.jgi.doe.gov/cgi-bin/m/main.cgi?section=TaxonDetail&page=taxonDetail&taxon_oid=2588253873 |
|  | *Mycobacterium tuberculosis* TB_RSA148 | 2574180007 | https://img.jgi.doe.gov/cgi-bin/m/main.cgi?section=TaxonDetail&page=taxonDetail&taxon_oid=2574180007 |
|  | *Mycobacterium tuberculosis* M2203 | 2582581138 | https://img.jgi.doe.gov/cgi-bin/m/main.cgi?section=TaxonDetail&page=taxonDetail&taxon_oid=2582581138 |
|  | *Mycobacterium tuberculosis* TKK_05SA_0014 | 2576861279 | https://img.jgi.doe.gov/cgi-bin/m/main.cgi?section=TaxonDetail&page=taxonDetail&taxon_oid=2576861279 |
|  | *Mycobacterium tuberculosis* MAL020160 | 2588254111 | https://img.jgi.doe.gov/cgi-bin/m/main.cgi?section=TaxonDetail&page=taxonDetail&taxon_oid=2588254111 |
|  | *Mycobacterium tuberculosis* TKK_03_0045 | 2590828613 | https://img.jgi.doe.gov/cgi-bin/m/main.cgi?section=TaxonDetail&page=taxonDetail&taxon_oid=2590828613 |
|  | *Mycobacterium tuberculosis* TKK-01-0028 | 2588253995 | https://img.jgi.doe.gov/cgi-bin/m/main.cgi?section=TaxonDetail&page=taxonDetail&taxon_oid=2588253995 |
|  | *Mycobacterium tuberculosis* XTB13-200 | 2574180197 | https://img.jgi.doe.gov/cgi-bin/m/main.cgi?section=TaxonDetail&page=taxonDetail&taxon_oid=2574180197 |
|  | *Mycobacterium tuberculosis* XTB13-088 | 2582581136 | https://img.jgi.doe.gov/cgi-bin/m/main.cgi?section=TaxonDetail&page=taxonDetail&taxon_oid=2582581136 |
|  | *Mycobacterium tuberculosis* TKK_04_0002 | 2582581216 | https://img.jgi.doe.gov/cgi-bin/m/main.cgi?section=TaxonDet2ail&page=taxonDetail&taxon_oid=2582581216 |
|  | *Mycobacterium tuberculosis* TKK-01-0065 | 2582581199 | https://img.jgi.doe.gov/cgi-bin/m/main.cgi?section=TaxonDetail&page=taxonDetail&taxon_oid=2582581199 |
|  | *Mycobacterium tuberculosis* TB_RSA51 | 2576861229 | https://img.jgi.doe.gov/cgi-bin/m/main.cgi?section=TaxonDetail&page=taxonDetail&taxon_oid=2576861229 |
|  | *Mycobacterium tuberculosis* M1449 | 2574180026 | https://img.jgi.doe.gov/cgi-bin/m/main.cgi?section=TaxonDetail&page=taxonDetail&taxon_oid=2574180026 |
|  | *Mycobacterium tuberculosis* TKK_02_0068 | 2590828629 | https://img.jgi.doe.gov/cgi-bin/m/main.cgi?section=TaxonDetail&page=taxonDetail&taxon_oid=2590828629 |
|  | *Mycobacterium canettii* CIPT 140070002 | 2565956758 | https://img.jgi.doe.gov/cgi-bin/m/main.cgi?section=TaxonDetail&page=taxonDetail&taxon_oid=2565956758 |
|  | *Mycobacterium bovis* BCG-Denmark TMC 1010, ATCC 35733 | 2547132085 | https://img.jgi.doe.gov/cgi-bin/m/main.cgi?section=TaxonDetail&page=taxonDetail&taxon_oid=2547132085 |
|  | *Mycobacterium tuberculosis* TKK_02_0015 | 2574179843 | https://img.jgi.doe.gov/cgi-bin/m/main.cgi?section=TaxonDetail&page=taxonDetail&taxon_oid=2574179843 |
|  | *Mycobacterium bovis* Kc 32216 | 2582580974 | https://img.jgi.doe.gov/cgi-bin/m/main.cgi?section=TaxonDetail&page=taxonDetail&taxon_oid=2582580974 |
|  | *Mycobacterium tuberculosis* TKK-01-0068 | 2576861246 | https://img.jgi.doe.gov/cgi-bin/m/main.cgi?section=TaxonDetail&page=taxonDetail&taxon_oid=2576861246 |
|  | *Mycobacterium tuberculosis* KT-0058 | 2588254144 | https://img.jgi.doe.gov/cgi-bin/m/main.cgi?section=TaxonDetail&page=taxonDetail&taxon_oid=2588254144 |
|  | *Mycobacterium tuberculosis* XTB13-199 | 2576861274 | https://img.jgi.doe.gov/cgi-bin/m/main.cgi?section=TaxonDetail&page=taxonDetail&taxon_oid=2576861274 |
|  | *Mycobacterium tuberculosis* MAL020192 | 2588254126 | https://img.jgi.doe.gov/cgi-bin/m/main.cgi?section=TaxonDetail&page=taxonDetail&taxon_oid=2588254126 |
|  | *Mycobacterium tuberculosis* TB_RSA138 | 2576861291 | https://img.jgi.doe.gov/cgi-bin/m/main.cgi?section=TaxonDetail&page=taxonDetail&taxon_oid=2576861291 |
|  | *Mycobacterium tuberculosis* KT-0048 | 2588254146 | https://img.jgi.doe.gov/cgi-bin/m/main.cgi?section=TaxonDetail&page=taxonDetail&taxon_oid=2588254146 |
|  | *Mycobacterium tuberculosis* KZN 4207 | 647000280 | https://img.jgi.doe.gov/cgi-bin/m/main.cgi?section=TaxonDetail&page=taxonDetail&taxon_oid=647000280 |
|  | *Mycobacterium tuberculosis* TKK_04_0129 | 2582581169 | https://img.jgi.doe.gov/cgi-bin/m/main.cgi?section=TaxonDetail&page=taxonDetail&taxon_oid=2582581169 |
|  | *Mycobacterium tuberculosis* BTB08-362 | 2574179970 | https://img.jgi.doe.gov/cgi-bin/m/main.cgi?section=TaxonDetail&page=taxonDetail&taxon_oid=2574179970 |
|  | *Mycobacterium tuberculosis* TB_RSA132 | 2576861318 | https://img.jgi.doe.gov/cgi-bin/m/main.cgi?section=TaxonDetail&page=taxonDetail&taxon_oid=2576861318 |
|  | *Mycobacterium* sp. UNC280MFTsu5.1 | 2579778519 | https://img.jgi.doe.gov/cgi-bin/m/main.cgi?section=TaxonDetail&page=taxonDetail&taxon_oid=2579778519 |
|  | *Mycobacterium tuberculosis* 16955 | 2574179955 | https://img.jgi.doe.gov/cgi-bin/m/main.cgi?section=TaxonDetail&page=taxonDetail&taxon_oid=2574179955 |
|  | *Mycobacterium tuberculosis* PanR0301 | 2554235239 | https://img.jgi.doe.gov/cgi-bin/m/main.cgi?section=TaxonDetail&page=taxonDetail&taxon_oid=2554235239 |
|  | *Mycobacterium tuberculosis* TKK_04_0072 | 2582581151 | https://img.jgi.doe.gov/cgi-bin/m/main.cgi?section=TaxonDetail&page=taxonDetail&taxon_oid=2582581151 |
|  | *Mycobacterium tuberculosis* BTB04-452 | 2574180214 | https://img.jgi.doe.gov/cgi-bin/m/main.cgi?section=TaxonDetail&page=taxonDetail&taxon_oid=2574180214 |
|  | *Mycobacterium tuberculosis* M2131 | 2574180418 | https://img.jgi.doe.gov/cgi-bin/m/main.cgi?section=TaxonDetail&page=taxonDetail&taxon_oid=2574180418 |
|  | *Mycobacterium tuberculosis* KT-0016 | 2588254157 | https://img.jgi.doe.gov/cgi-bin/m/main.cgi?section=TaxonDetail&page=taxonDetail&taxon_oid=2588254157 |
|  | *Mycobacterium tuberculosis* XTB13-123 | 2576861391 | https://img.jgi.doe.gov/cgi-bin/m/main.cgi?section=TaxonDetail&page=taxonDetail&taxon_oid=2576861391 |
|  | *Mycobacterium tuberculosis* MAL010133 | 2574180004 | https://img.jgi.doe.gov/cgi-bin/m/main.cgi?section=TaxonDetail&page=taxonDetail&taxon_oid=2574180004 |
|  | *Mycobacterium tuberculosis* OFXR-2 | 2588253895 | https://img.jgi.doe.gov/cgi-bin/m/main.cgi?section=TaxonDetail&page=taxonDetail&taxon_oid=2588253895 |
|  | *Mycobacterium tuberculosis* TKK_04_0042 | 2582581201 | https://img.jgi.doe.gov/cgi-bin/m/main.cgi?section=TaxonDetail&page=taxonDetail&taxon_oid=2582581201 |
|  | *Mycobacterium africanum* MAL020148 | 2579778922 | https://img.jgi.doe.gov/cgi-bin/m/main.cgi?section=TaxonDetail&page=taxonDetail&taxon_oid=2579778922 |
|  | *Mycobacterium tuberculosis* TKK_05SA_0012 | 2574180098 | https://img.jgi.doe.gov/cgi-bin/m/main.cgi?section=TaxonDetail&page=taxonDetail&taxon_oid=2574180098 |
|  | *Mycobacterium tuberculosis* MAL010088 | 2588254097 | https://img.jgi.doe.gov/cgi-bin/m/main.cgi?section=TaxonDetail&page=taxonDetail&taxon_oid=2588254097 |
|  | *Mycobacterium tuberculosis* TKK_05MA_0009 | 2582581171 | https://img.jgi.doe.gov/cgi-bin/m/main.cgi?section=TaxonDetail&page=taxonDetail&taxon_oid=2582581171 |
|  | *Mycobacterium tuberculosis* BTB05-559 | 2547132260 | https://img.jgi.doe.gov/cgi-bin/m/main.cgi?section=TaxonDetail&page=taxonDetail&taxon_oid=2547132260 |
|  | *Mycobacterium tuberculosis* BTB05-552 | 2547132259 | https://img.jgi.doe.gov/cgi-bin/m/main.cgi?section=TaxonDetail&page=taxonDetail&taxon_oid=2547132259 |
|  | *Mycobacterium tuberculosis* M1008 | 2574180370 | https://img.jgi.doe.gov/cgi-bin/m/main.cgi?section=TaxonDetail&page=taxonDetail&taxon_oid=2574180370 |
|  | *Mycobacterium tuberculosis* MAL010105 | 2588254101 | https://img.jgi.doe.gov/cgi-bin/m/main.cgi?section=TaxonDetail&page=taxonDetail&taxon_oid=2588254101 |
|  | *Mycobacterium tuberculosis* TBR44 | 2588253880 | https://img.jgi.doe.gov/cgi-bin/m/main.cgi?section=TaxonDetail&page=taxonDetail&taxon_oid=2588253880 |
|  | *Mycobacterium tuberculosis* TKK_04_0048 | 2590828672 | https://img.jgi.doe.gov/cgi-bin/m/main.cgi?section=TaxonDetail&page=taxonDetail&taxon_oid=2590828672 |
|  | *Mycobacterium tuberculosis* TKK_02_0061 | 2590828633 | https://img.jgi.doe.gov/cgi-bin/m/main.cgi?section=TaxonDetail&page=taxonDetail&taxon_oid=2590828633 |
|  | *Mycobacterium tuberculosis* TKK_04_0008 | 2590828606 | https://img.jgi.doe.gov/cgi-bin/m/main.cgi?section=TaxonDetail&page=taxonDetail&taxon_oid=2590828606 |
|  | *Mycobacterium tuberculosis* TKK-01-0082 | 2588254030 | https://img.jgi.doe.gov/cgi-bin/m/main.cgi?section=TaxonDetail&page=taxonDetail&taxon_oid=2588254030 |
|  | *Mycobacterium tuberculosis* TB_RSA83 | 2574180255 | https://img.jgi.doe.gov/cgi-bin/m/main.cgi?section=TaxonDetail&page=taxonDetail&taxon_oid=2574180255 |
|  | *Mycobacterium tuberculosis* T46 | 2574179851 | https://img.jgi.doe.gov/cgi-bin/m/main.cgi?section=TaxonDetail&page=taxonDetail&taxon_oid=2574179851 |
|  | *Mycobacterium tuberculosis* EAS054 | 642979363 | https://img.jgi.doe.gov/cgi-bin/m/main.cgi?section=TaxonDetail&page=taxonDetail&taxon_oid=642979363 |
|  | *Mycobacterium tuberculosis* OFXR-22 | 2588254183 | https://img.jgi.doe.gov/cgi-bin/m/main.cgi?section=TaxonDetail&page=taxonDetail&taxon_oid=2588254183 |
|  | *Mycobacterium tuberculosis* TRUG0116 | 2582581130 | https://img.jgi.doe.gov/cgi-bin/m/main.cgi?section=TaxonDetail&page=taxonDetail&taxon_oid=2582581130 |
|  | *Mycobacterium tuberculosis* SP21 | 2554235011 | https://img.jgi.doe.gov/cgi-bin/m/main.cgi?section=TaxonDetail&page=taxonDetail&taxon_oid=2554235011 |
|  | *Mycobacterium tuberculosis* TKK_03_0018 | 2590828624 | https://img.jgi.doe.gov/cgi-bin/m/main.cgi?section=TaxonDetail&page=taxonDetail&taxon_oid=2590828624 |
|  | *Mycobacterium tuberculosis* NRITLD44 | 2574180131 | https://img.jgi.doe.gov/cgi-bin/m/main.cgi?section=TaxonDetail&page=taxonDetail&taxon_oid=2574180131 |
|  | *Mycobacterium tuberculosis* PanR0801 | 2554235262 | https://img.jgi.doe.gov/cgi-bin/m/main.cgi?section=TaxonDetail&page=taxonDetail&taxon_oid=2554235262 |
|  | *Mycobacterium tuberculosis* XTB13-238 | 2582581180 | https://img.jgi.doe.gov/cgi-bin/m/main.cgi?section=TaxonDetail&page=taxonDetail&taxon_oid=2582581180 |
|  | *Mycobacterium tuberculosis* M2479 | 2576861239 | https://img.jgi.doe.gov/cgi-bin/m/main.cgi?section=TaxonDetail&page=taxonDetail&taxon_oid=2576861239 |
|  | *Mycobacterium tuberculosis* PanR0305 | 2554235243 | https://img.jgi.doe.gov/cgi-bin/m/main.cgi?section=TaxonDetail&page=taxonDetail&taxon_oid=2554235243 |
|  | *Mycobacterium aromaticivorans* JS19b1 | 2558309009 | https://img.jgi.doe.gov/cgi-bin/m/main.cgi?section=TaxonDetail&page=taxonDetail&taxon_oid=2558309009 |
|  | *Mycobacterium tuberculosis* TKK_04_0157 | 2574179876 | https://img.jgi.doe.gov/cgi-bin/m/main.cgi?section=TaxonDetail&page=taxonDetail&taxon_oid=2574179876 |
|  | *Mycobacterium tuberculosis* 51628 | 2582581135 | https://img.jgi.doe.gov/cgi-bin/m/main.cgi?section=TaxonDetail&page=taxonDetail&taxon_oid=2582581135 |
|  | *Mycobacterium tuberculosis* MD16577 | 2582581207 | https://img.jgi.doe.gov/cgi-bin/m/main.cgi?section=TaxonDetail&page=taxonDetail&taxon_oid=2582581207 |
|  | *Mycobacterium tuberculosis* PanR0409 | 2554235307 | https://img.jgi.doe.gov/cgi-bin/m/main.cgi?section=TaxonDetail&page=taxonDetail&taxon_oid=2554235307 |
|  | *Mycobacterium tuberculosis* MAL020187 | 2588254129 | https://img.jgi.doe.gov/cgi-bin/m/main.cgi?section=TaxonDetail&page=taxonDetail&taxon_oid=2588254129 |
|  | *Mycobacterium tuberculosis* TKK-01-0060 | 2588254013 | https://img.jgi.doe.gov/cgi-bin/m/main.cgi?section=TaxonDetail&page=taxonDetail&taxon_oid=2588254013 |
|  | *Mycobacterium tuberculosis* TKK-01-0049 | 2588254006 | https://img.jgi.doe.gov/cgi-bin/m/main.cgi?section=TaxonDetail&page=taxonDetail&taxon_oid=2588254006 |
|  | *Mycobacterium tuberculosis* TKK_04_0021 | 2574180080 | https://img.jgi.doe.gov/cgi-bin/m/main.cgi?section=TaxonDetail&page=taxonDetail&taxon_oid=2574180080 |
|  | *Mycobacterium massiliense* 2B-0307 | 2526164658 | https://img.jgi.doe.gov/cgi-bin/m/main.cgi?section=TaxonDetail&page=taxonDetail&taxon_oid=2526164658 |
|  | *Mycobacterium canettii* CIPT 140070008 | 2541047045 | https://img.jgi.doe.gov/cgi-bin/m/main.cgi?section=TaxonDetail&page=taxonDetail&taxon_oid=2541047045 |
|  | *Mycobacterium canettii* CIPT 140010059 | 650716060 | https://img.jgi.doe.gov/cgi-bin/m/main.cgi?section=TaxonDetail&page=taxonDetail&taxon_oid=650716060 |
|  | *Mycobacterium intracellulare* MIN_052511_1280 | 2568526126 | https://img.jgi.doe.gov/cgi-bin/m/main.cgi?section=TaxonDetail&page=taxonDetail&taxon_oid=2568526126 |
|  | *Mycobacterium tuberculosis* MAL020201 | 2588254134 | https://img.jgi.doe.gov/cgi-bin/m/main.cgi?section=TaxonDetail&page=taxonDetail&taxon_oid=2588254134 |
|  | *Mycobacterium tuberculosis* KT-0022 | 2588254155 | https://img.jgi.doe.gov/cgi-bin/m/main.cgi?section=TaxonDetail&page=taxonDetail&taxon_oid=2588254155 |
|  | *Mycobacterium tuberculosis* PanR0412 | 2554235308 | https://img.jgi.doe.gov/cgi-bin/m/main.cgi?section=TaxonDetail&page=taxonDetail&taxon_oid=2554235308 |
|  | *Mycobacterium tuberculosis* TB_RSA67 | 2574180226 | https://img.jgi.doe.gov/cgi-bin/m/main.cgi?section=TaxonDetail&page=taxonDetail&taxon_oid=2574180226 |
|  | *Mycobacterium tuberculosis* TKK_02_0013 | 2590828648 | https://img.jgi.doe.gov/cgi-bin/m/main.cgi?section=TaxonDetail&page=taxonDetail&taxon_oid=2590828648 |
|  | *Mycobacterium tuberculosis* TKK_04_0066 | 2582581149 | https://img.jgi.doe.gov/cgi-bin/m/main.cgi?section=TaxonDetail&page=taxonDetail&taxon_oid=2582581149 |
|  | *Mycobacterium tuberculosis* OFXR-16 | 2588253901 | https://img.jgi.doe.gov/cgi-bin/m/main.cgi?section=TaxonDetail&page=taxonDetail&taxon_oid=2588253901 |
|  | *Mycobacterium africanum* MAL020173 | 2579778878 | https://img.jgi.doe.gov/cgi-bin/m/main.cgi?section=TaxonDetail&page=taxonDetail&taxon_oid=2579778878 |
|  | *Mycobacterium tuberculosis* Haarlem | 641736194 | https://img.jgi.doe.gov/cgi-bin/m/main.cgi?section=TaxonDetail&page=taxonDetail&taxon_oid=641736194 |
|  | *Mycobacterium tuberculosis* TKK-01-0066 | 2588254025 | https://img.jgi.doe.gov/cgi-bin/m/main.cgi?section=TaxonDetail&page=taxonDetail&taxon_oid=2588254025 |
|  | *Mycobacterium tuberculosis* NRITLD15 | 2576861348 | https://img.jgi.doe.gov/cgi-bin/m/main.cgi?section=TaxonDetail&page=taxonDetail&taxon_oid=2576861348 |
|  | *Mycobacterium tuberculosis* KT-0080 | 2588254236 | https://img.jgi.doe.gov/cgi-bin/m/main.cgi?section=TaxonDetail&page=taxonDetail&taxon_oid=2588254236 |
|  | *Mycobacterium intracellulare* MIN_061107_1834 | 2568526481 | https://img.jgi.doe.gov/cgi-bin/m/main.cgi?section=TaxonDetail&page=taxonDetail&taxon_oid=2568526481 |
|  | *Mycobacterium tuberculosis* PanR0611 | 2554235270 | https://img.jgi.doe.gov/cgi-bin/m/main.cgi?section=TaxonDetail&page=taxonDetail&taxon_oid=2554235270 |
|  | *Mycobacterium tuberculosis* GuangZ0019 | 2545824696 | https://img.jgi.doe.gov/cgi-bin/m/main.cgi?section=TaxonDetail&page=taxonDetail&taxon_oid=2545824696 |
|  | *Mycobacterium tuberculosis* M2248 | 2576861095 | https://img.jgi.doe.gov/cgi-bin/m/main.cgi?section=TaxonDetail&page=taxonDetail&taxon_oid=2576861095 |
|  | *Mycobacterium tuberculosis* TKK_03_0033 | 2590828617 | https://img.jgi.doe.gov/cgi-bin/m/main.cgi?section=TaxonDetail&page=taxonDetail&taxon_oid=2590828617 |
|  | *Mycobacterium tuberculosis* TKK_05MA_0020 | 2574179862 | https://img.jgi.doe.gov/cgi-bin/m/main.cgi?section=TaxonDetail&page=taxonDetail&taxon_oid=2574179862 |
|  | *Mycobacterium tuberculosis* TB_RSA107 | 2582581198 | https://img.jgi.doe.gov/cgi-bin/m/main.cgi?section=TaxonDetail&page=taxonDetail&taxon_oid=2582581198 |
|  | *Mycobacterium tuberculosis* TKK_02_0021 | 2590828643 | https://img.jgi.doe.gov/cgi-bin/m/main.cgi?section=TaxonDetail&page=taxonDetail&taxon_oid=2590828643 |
|  | *Mycobacterium tuberculosis* TKK_02_0039 | 2590828636 | https://img.jgi.doe.gov/cgi-bin/m/main.cgi?section=TaxonDetail&page=taxonDetail&taxon_oid=2590828636 |
|  | *Mycobacterium tuberculosis* M1025 | 2576861368 | https://img.jgi.doe.gov/cgi-bin/m/main.cgi?section=TaxonDetail&page=taxonDetail&taxon_oid=2576861368 |
|  | *Mycobacterium tuberculosis* M1017 | 2576861286 | https://img.jgi.doe.gov/cgi-bin/m/main.cgi?section=TaxonDetail&page=taxonDetail&taxon_oid=2576861286 |
|  | *Mycobacterium tuberculosis* TBR10 | 2579778510 | https://img.jgi.doe.gov/cgi-bin/m/main.cgi?section=TaxonDetail&page=taxonDetail&taxon_oid=2579778510 |
|  | *Mycobacterium tuberculosis* CCDC5180 | 651053044 | https://img.jgi.doe.gov/cgi-bin/m/main.cgi?section=TaxonDetail&page=taxonDetail&taxon_oid=651053044 |
|  | *Mycobacterium tuberculosis* OFXR-11 | 2588253898 | https://img.jgi.doe.gov/cgi-bin/m/main.cgi?section=TaxonDetail&page=taxonDetail&taxon_oid=2588253898 |
|  | *Mycobacterium tuberculosis* TRUG0080 | 2582581168 | https://img.jgi.doe.gov/cgi-bin/m/main.cgi?section=TaxonDetail&page=taxonDetail&taxon_oid=2582581168 |
|  | *Mycobacterium tuberculosis* KT-0027 | 2576861321 | https://img.jgi.doe.gov/cgi-bin/m/main.cgi?section=TaxonDetail&page=taxonDetail&taxon_oid=2576861321 |
|  | *Mycobacterium bovis* BCG China | 2547132084 | https://img.jgi.doe.gov/cgi-bin/m/main.cgi?section=TaxonDetail&page=taxonDetail&taxon_oid=2547132084 |
|  | *Mycobacterium tuberculosis* MAL020152 | 2588254118 | https://img.jgi.doe.gov/cgi-bin/m/main.cgi?section=TaxonDetail&page=taxonDetail&taxon_oid=2588254118 |
|  | *Mycobacterium* sp. 05-1390 | 2541047007 | https://img.jgi.doe.gov/cgi-bin/m/main.cgi?section=TaxonDetail&page=taxonDetail&taxon_oid=2541047007 |
|  | *Mycobacterium tuberculosis* KT-0072 | 2574180239 | https://img.jgi.doe.gov/cgi-bin/m/main.cgi?section=TaxonDetail&page=taxonDetail&taxon_oid=2574180239 |
|  | *Mycobacterium tuberculosis* T46 | 645951864 | https://img.jgi.doe.gov/cgi-bin/m/main.cgi?section=TaxonDetail&page=taxonDetail&taxon_oid=645951864 |
|  | *Mycobacterium tuberculosis* TKK_04_0015 | 2590828603 | https://img.jgi.doe.gov/cgi-bin/m/main.cgi?section=TaxonDetail&page=taxonDetail&taxon_oid=2590828603 |
|  | *Mycobacterium tuberculosis* TKK-01-0091 | 2576861042 | https://img.jgi.doe.gov/cgi-bin/m/main.cgi?section=TaxonDetail&page=taxonDetail&taxon_oid=2576861042 |
|  | *Mycobacterium xenopi* 3993 | 2565956797 | https://img.jgi.doe.gov/cgi-bin/m/main.cgi?section=TaxonDetail&page=taxonDetail&taxon_oid=2565956797 |
|  | *Mycobacterium tuberculosis* TBR8 | 2588253867 | https://img.jgi.doe.gov/cgi-bin/m/main.cgi?section=TaxonDetail&page=taxonDetail&taxon_oid=2588253867 |
|  | *Mycobacterium massiliense* 2B-1231 | 2531839069 | https://img.jgi.doe.gov/cgi-bin/m/main.cgi?section=TaxonDetail&page=taxonDetail&taxon_oid=2531839069 |
|  | *Mycobacterium africanum* MAL010123 | 2579778981 | https://img.jgi.doe.gov/cgi-bin/m/main.cgi?section=TaxonDetail&page=taxonDetail&taxon_oid=2579778981 |
|  | *Mycobacterium africanum* MAL010071 | 2579779036 | https://img.jgi.doe.gov/cgi-bin/m/main.cgi?section=TaxonDetail&page=taxonDetail&taxon_oid=2579779036 |
|  | *Mycobacterium tuberculosis* M1418 | 2576861038 | https://img.jgi.doe.gov/cgi-bin/m/main.cgi?section=TaxonDetail&page=taxonDetail&taxon_oid=2576861038 |
|  | *Mycobacterium tuberculosis* TKK_04_0020 | 2590828600 | https://img.jgi.doe.gov/cgi-bin/m/main.cgi?section=TaxonDetail&page=taxonDetail&taxon_oid=2590828600 |
|  | *Mycobacterium tuberculosis* TB_RSA07 | 2576861283 | https://img.jgi.doe.gov/cgi-bin/m/main.cgi?section=TaxonDetail&page=taxonDetail&taxon_oid=2576861283 |
|  | *Mycobacterium tuberculosis* PanR0404 | 2554235301 | https://img.jgi.doe.gov/cgi-bin/m/main.cgi?section=TaxonDetail&page=taxonDetail&taxon_oid=2554235301 |
|  | *Mycobacterium tuberculosis* WX1 | 2558860630 | https://img.jgi.doe.gov/cgi-bin/m/main.cgi?section=TaxonDetail&page=taxonDetail&taxon_oid=2558860630 |
|  | *Mycobacterium tuberculosis* TKK-01-0003 | 2588253983 | https://img.jgi.doe.gov/cgi-bin/m/main.cgi?section=TaxonDetail&page=taxonDetail&taxon_oid=2588253983 |
|  | *Mycobacterium tuberculosis* OM-V02_005 | 2568526658 | https://img.jgi.doe.gov/cgi-bin/m/main.cgi?section=TaxonDetail&page=taxonDetail&taxon_oid=2568526658 |
|  | *Mycobacterium tuberculosis* MAL020211 | 2588254140 | https://img.jgi.doe.gov/cgi-bin/m/main.cgi?section=TaxonDetail&page=taxonDetail&taxon_oid=2588254140 |
|  | *Mycobacterium tuberculosis* SUMu004 | 648276696 | https://img.jgi.doe.gov/cgi-bin/m/main.cgi?section=TaxonDetail&page=taxonDetail&taxon_oid=648276696 |
|  | *Mycobacterium tuberculosis* TKK_04_0037 | 2590828677 | https://img.jgi.doe.gov/cgi-bin/m/main.cgi?section=TaxonDetail&page=taxonDetail&taxon_oid=2590828677 |
|  | *Mycobacterium tuberculosis* TKK-01-0045 | 2588254009 | https://img.jgi.doe.gov/cgi-bin/m/main.cgi?section=TaxonDetail&page=taxonDetail&taxon_oid=2588254009 |
|  | *Mycobacterium tuberculosis* KT-0051 | 2574179952 | https://img.jgi.doe.gov/cgi-bin/m/main.cgi?section=TaxonDetail&page=taxonDetail&taxon_oid=2574179952 |
|  | *Mycobacterium tuberculosis* TKK-01-0027 | 2588253991 | https://img.jgi.doe.gov/cgi-bin/m/main.cgi?section=TaxonDetail&page=taxonDetail&taxon_oid=2588253991 |
|  | *Mycobacterium tuberculosis* PanR0906 | 2554235278 | https://img.jgi.doe.gov/cgi-bin/m/main.cgi?section=TaxonDetail&page=taxonDetail&taxon_oid=2554235278 |
|  | *Mycobacterium tuberculosis* PanR0202 | 2597489946 | https://img.jgi.doe.gov/cgi-bin/m/main.cgi?section=TaxonDetail&page=taxonDetail&taxon_oid=2597489946 |
|  | *Mycobacterium tusciae* JS617 | 2508501052 | https://img.jgi.doe.gov/cgi-bin/m/main.cgi?section=TaxonDetail&page=taxonDetail&taxon_oid=2508501052 |
|  | *Mycobacterium tuberculosis* PanR0605 | 2554235253 | https://img.jgi.doe.gov/cgi-bin/m/main.cgi?section=TaxonDetail&page=taxonDetail&taxon_oid=2554235253 |
|  | *Mycobacterium tuberculosis* MAL020209 | 2588254139 | https://img.jgi.doe.gov/cgi-bin/m/main.cgi?section=TaxonDetail&page=taxonDetail&taxon_oid=2588254139 |
|  | *Mycobacterium tuberculosis* TB_RSA195 | 2574180322 | https://img.jgi.doe.gov/cgi-bin/m/main.cgi?section=TaxonDetail&page=taxonDetail&taxon_oid=2574180322 |
|  | *Mycobacterium tuberculosis* M1913 | 2576861016 | https://img.jgi.doe.gov/cgi-bin/m/main.cgi?section=TaxonDetail&page=taxonDetail&taxon_oid=2576861016 |
|  | *Mycobacterium africanum* MAL010084 | 2579778911 | https://img.jgi.doe.gov/cgi-bin/m/main.cgi?section=TaxonDetail&page=taxonDetail&taxon_oid=2579778911 |
|  | *Mycobacterium africanum* MAL010079 | 2579778777 | https://img.jgi.doe.gov/cgi-bin/m/main.cgi?section=TaxonDetail&page=taxonDetail&taxon_oid=2579778777 |
|  | *Mycobacterium tuberculosis* TBR56 | 2588253885 | https://img.jgi.doe.gov/cgi-bin/m/main.cgi?section=TaxonDetail&page=taxonDetail&taxon_oid=2588253885 |
|  | *Mycobacterium tuberculosis* 98-R604 INH-RIF-EM | 645058721 | https://img.jgi.doe.gov/cgi-bin/m/main.cgi?section=TaxonDetail&page=taxonDetail&taxon_oid=645058721 |
|  | *Mycobacterium tuberculosis* KT-0043 | 2588254149 | https://img.jgi.doe.gov/cgi-bin/m/main.cgi?section=TaxonDetail&page=taxonDetail&taxon_oid=2588254149 |
|  | *Mycobacterium tuberculosis* TB_RSA70 | 2576861217 | https://img.jgi.doe.gov/cgi-bin/m/main.cgi?section=TaxonDetail&page=taxonDetail&taxon_oid=2576861217 |
|  | *Mycobacterium tuberculosis* PanR0411 | 2554235293 | https://img.jgi.doe.gov/cgi-bin/m/main.cgi?section=TaxonDetail&page=taxonDetail&taxon_oid=2554235293 |
|  | *Mycobacterium tuberculosis* KT-0110 | 2574180278 | https://img.jgi.doe.gov/cgi-bin/m/main.cgi?section=TaxonDetail&page=taxonDetail&taxon_oid=2574180278 |
|  | *Mycobacterium tuberculosis* M1213 | 2574180085 | https://img.jgi.doe.gov/cgi-bin/m/main.cgi?section=TaxonDetail&page=taxonDetail&taxon_oid=2574180085 |
|  | *Mycobacterium tuberculosis* BTB03-012 | 2576861055 | https://img.jgi.doe.gov/cgi-bin/m/main.cgi?section=TaxonDetail&page=taxonDetail&taxon_oid=2576861055 |
|  | *Mycobacterium tuberculosis* CDC1551 | 637000172 | https://img.jgi.doe.gov/cgi-bin/m/main.cgi?section=TaxonDetail&page=taxonDetail&taxon_oid=637000172 |
|  | *Mycobacterium tuberculosis* MD19964 | 2574179977 | https://img.jgi.doe.gov/cgi-bin/m/main.cgi?section=TaxonDetail&page=taxonDetail&taxon_oid=2574179977 |
|  | *Mycobacterium tuberculosis* TRUG0083 | 2582581187 | https://img.jgi.doe.gov/cgi-bin/m/main.cgi?section=TaxonDetail&page=taxonDetail&taxon_oid=2582581187 |
|  | *Mycobacterium tuberculosis* MD17517 | 2576861215 | https://img.jgi.doe.gov/cgi-bin/m/main.cgi?section=TaxonDetail&page=taxonDetail&taxon_oid=2576861215 |
|  | *Mycobacterium phlei* RIVM601174 | 2522572168 | https://img.jgi.doe.gov/cgi-bin/m/main.cgi?section=TaxonDetail&page=taxonDetail&taxon_oid=2522572168 |
|  | *Mycobacterium tuberculosis* 02_1987 | 642979309 | https://img.jgi.doe.gov/cgi-bin/m/main.cgi?section=TaxonDetail&page=taxonDetail&taxon_oid=642979309 |
|  | *Mycobacterium tuberculosis* MAL020157 | 2588254120 | https://img.jgi.doe.gov/cgi-bin/m/main.cgi?section=TaxonDetail&page=taxonDetail&taxon_oid=2588254120 |
|  | *Mycobacterium tuberculosis* TB_RSA104 | 2582581176 | https://img.jgi.doe.gov/cgi-bin/m/main.cgi?section=TaxonDetail&page=taxonDetail&taxon_oid=2582581176 |
|  | *Mycobacterium tuberculosis* TB_RSA194 | 2574179858 | https://img.jgi.doe.gov/cgi-bin/m/main.cgi?section=TaxonDetail&page=taxonDetail&taxon_oid=2574179858 |
|  | *Mycobacterium tuberculosis* TKK-01-0086 | 2588254034 | https://img.jgi.doe.gov/cgi-bin/m/main.cgi?section=TaxonDetail&page=taxonDetail&taxon_oid=2588254034 |
|  | *Mycobacterium tuberculosis* TB_RSA68 | 2574179886 | https://img.jgi.doe.gov/cgi-bin/m/main.cgi?section=TaxonDetail&page=taxonDetail&taxon_oid=2574179886 |
|  | *Mycobacterium tuberculosis* TKK_03_0040 | 2582581144 | https://img.jgi.doe.gov/cgi-bin/m/main.cgi?section=TaxonDetail&page=taxonDetail&taxon_oid=2582581144 |
|  | *Mycobacterium* sp. 360 MFTsu5.1 | 2521172630 | https://img.jgi.doe.gov/cgi-bin/m/main.cgi?section=TaxonDetail&page=taxonDetail&taxon_oid=2521172630 |
|  | *Mycobacterium tuberculosis* XTB13-143 | 2574180298 | https://img.jgi.doe.gov/cgi-bin/m/main.cgi?section=TaxonDetail&page=taxonDetail&taxon_oid=2574180298 |
|  | *Mycobacterium tuberculosis* EAI/OSDD271 | 2554235103 | https://img.jgi.doe.gov/cgi-bin/m/main.cgi?section=TaxonDetail&page=taxonDetail&taxon_oid=2554235103 |
|  | *Mycobacterium tuberculosis* PanR0401 | 2554235310 | https://img.jgi.doe.gov/cgi-bin/m/main.cgi?section=TaxonDetail&page=taxonDetail&taxon_oid=2554235310 |
|  | *Mycobacterium tuberculosis* KT-0034 | 2574180175 | https://img.jgi.doe.gov/cgi-bin/m/main.cgi?section=TaxonDetail&page=taxonDetail&taxon_oid=2574180175 |
|  | *Mycobacterium tuberculosis* MAL010124 | 2588254103 | https://img.jgi.doe.gov/cgi-bin/m/main.cgi?section=TaxonDetail&page=taxonDetail&taxon_oid=2588254103 |
|  | *Mycobacterium tuberculosis* M995 | 2579778509 | https://img.jgi.doe.gov/cgi-bin/m/main.cgi?section=TaxonDetail&page=taxonDetail&taxon_oid=2579778509 |
|  | *Mycobacterium tuberculosis* MAL020186 | 2588254124 | https://img.jgi.doe.gov/cgi-bin/m/main.cgi?section=TaxonDetail&page=taxonDetail&taxon_oid=2588254124 |
|  | *Mycobacterium tuberculosis* OSDD504 | 2548876690 | https://img.jgi.doe.gov/cgi-bin/m/main.cgi?section=TaxonDetail&page=taxonDetail&taxon_oid=2548876690 |
|  | *Mycobacterium tuberculosis* TKK_03_0158 | 2576861017 | https://img.jgi.doe.gov/cgi-bin/m/main.cgi?section=TaxonDetail&page=taxonDetail&taxon_oid=2576861017 |
|  | *Mycobacterium tuberculosis* TKK-01-0088 | 2588254029 | https://img.jgi.doe.gov/cgi-bin/m/main.cgi?section=TaxonDetail&page=taxonDetail&taxon_oid=2588254029 |
|  | *Mycobacterium para scrofulaceum* ATCC BAA-614 | 647000278 | https://img.jgi.doe.gov/cgi-bin/m/main.cgi?section=TaxonDetail&page=taxonDetail&taxon_oid=647000278 |
|  | *Mycobacterium tuberculosis* PanR0610 | 2554235266 | https://img.jgi.doe.gov/cgi-bin/m/main.cgi?section=TaxonDetail&page=taxonDetail&taxon_oid=2554235266 |
|  | *Mycobacterium tuberculosis* TBR51 | 2582581128 | https://img.jgi.doe.gov/cgi-bin/m/main.cgi?section=TaxonDetail&page=taxonDetail&taxon_oid=2582581128 |
|  | *Mycobacterium tuberculosis* MAL020199 | 2588254132 | https://img.jgi.doe.gov/cgi-bin/m/main.cgi?section=TaxonDetail&page=taxonDetail&taxon_oid=2588254132 |
|  | *Mycobacterium tuberculosis* OFXR-29 | 2588254180 | https://img.jgi.doe.gov/cgi-bin/m/main.cgi?section=TaxonDetail&page=taxonDetail&taxon_oid=2588254180 |
|  | *Mycobacterium tuberculosis* Erdman | 2540341098 | https://img.jgi.doe.gov/cgi-bin/m/main.cgi?section=TaxonDetail&page=taxonDetail&taxon_oid=2540341098 |
|  | *Mycobacterium tuberculosis* TKK-01-0076 | 2588254020 | https://img.jgi.doe.gov/cgi-bin/m/main.cgi?section=TaxonDetail&page=taxonDetail&taxon_oid=2588254020 |
|  | *Mycobacterium tuberculosis* TRUG0124 | 2582581188 | https://img.jgi.doe.gov/cgi-bin/m/main.cgi?section=TaxonDetail&page=taxonDetail&taxon_oid=2582581188 |
|  | *Mycobacterium tuberculosis* TKK_02_0071 | 2574179882 | https://img.jgi.doe.gov/cgi-bin/m/main.cgi?section=TaxonDetail&page=taxonDetail&taxon_oid=2574179882 |
|  | *Mycobacterium tuberculosis* KT-0070 | 2576861220 | https://img.jgi.doe.gov/cgi-bin/m/main.cgi?section=TaxonDetail&page=taxonDetail&taxon_oid=2576861220 |
|  | *Mycobacterium canettii* CIPT 140070013 | 2565956757 | https://img.jgi.doe.gov/cgi-bin/m/main.cgi?section=TaxonDetail&page=taxonDetail&taxon_oid=2565956757 |
|  | *Mycobacterium tuberculosis* TKK-01-0090 | 2574179983 | https://img.jgi.doe.gov/cgi-bin/m/main.cgi?section=TaxonDetail&page=taxonDetail&taxon_oid=2574179983 |
|  | *Mycobacterium tuberculosis* TKK-01-0043 | 2588254001 | https://img.jgi.doe.gov/cgi-bin/m/main.cgi?section=TaxonDetail&page=taxonDetail&taxon_oid=2588254001 |
|  | *Mycobacterium tuberculosis* PanR0207 | 2554235213 | https://img.jgi.doe.gov/cgi-bin/m/main.cgi?section=TaxonDetail&page=taxonDetail&taxon_oid=2554235213 |
|  | *Mycobacterium tuberculosis* TB_RSA90 | 2574180060 | https://img.jgi.doe.gov/cgi-bin/m/main.cgi?section=TaxonDetail&page=taxonDetail&taxon_oid=2574180060 |
|  | *Mycobacterium tuberculosis* TKK_04_0060 | 2582581185 | https://img.jgi.doe.gov/cgi-bin/m/main.cgi?section=TaxonDetail&page=taxonDetail&taxon_oid=2582581185 |
|  | *Mycobacterium tuberculosis* BTB11-236 | 2576861372 | https://img.jgi.doe.gov/cgi-bin/m/main.cgi?section=TaxonDetail&page=taxonDetail&taxon_oid=2576861372 |
|  | *Mycobacterium tuberculosis* PanR0407 | 2554235299 | https://img.jgi.doe.gov/cgi-bin/m/main.cgi?section=TaxonDetail&page=taxonDetail&taxon_oid=2554235299 |
|  | *Mycobacterium tuberculosis* MD15212 | 2576861182 | https://img.jgi.doe.gov/cgi-bin/m/main.cgi?section=TaxonDetail&page=taxonDetail&taxon_oid=2576861182 |
|  | *Mycobacterium tuberculosis* TB_RSA140 | 2574180256 | https://img.jgi.doe.gov/cgi-bin/m/main.cgi?section=TaxonDetail&page=taxonDetail&taxon_oid=2574180256 |
|  | *Mycobacterium tuberculosis* PanR0803 | 2554235259 | https://img.jgi.doe.gov/cgi-bin/m/main.cgi?section=TaxonDetail&page=taxonDetail&taxon_oid=2554235259 |
|  | *Mycobacterium* sp. UM_RHS | 2554235287 | https://img.jgi.doe.gov/cgi-bin/m/main.cgi?section=TaxonDetail&page=taxonDetail&taxon_oid=2554235287 |
|  | *Mycobacterium tuberculosis* BTB08-148 | 2574180382 | https://img.jgi.doe.gov/cgi-bin/m/main.cgi?section=TaxonDetail&page=taxonDetail&taxon_oid=2574180382 |
|  | *Mycobacterium tuberculosis* UG-C | 2574180010 | https://img.jgi.doe.gov/cgi-bin/m/main.cgi?section=TaxonDetail&page=taxonDetail&taxon_oid=2574180010 |
|  | *Mycobacterium massiliense* 1S-153-0915 | 2526164660 | https://img.jgi.doe.gov/cgi-bin/m/main.cgi?section=TaxonDetail&page=taxonDetail&taxon_oid=2526164660 |
|  | *Mycobacterium africanum* MAL020135 | 2582580877 | https://img.jgi.doe.gov/cgi-bin/m/main.cgi?section=TaxonDetail&page=taxonDetail&taxon_oid=2582580877 |
|  | *Mycobacterium tuberculosis* TKK_04_0024 | 2574180232 | https://img.jgi.doe.gov/cgi-bin/m/main.cgi?section=TaxonDetail&page=taxonDetail&taxon_oid=2574180232 |
|  | *Mycobacterium tuberculosis* TKK_03_0059 | 2582581143 | https://img.jgi.doe.gov/cgi-bin/m/main.cgi?section=TaxonDetail&page=taxonDetail&taxon_oid=2582581143 |
|  | *Mycobacterium tuberculosis* BTB12-400 | 2574180403 | https://img.jgi.doe.gov/cgi-bin/m/main.cgi?section=TaxonDetail&page=taxonDetail&taxon_oid=2574180403 |
|  | *Mycobacterium tuberculosis* KT-0024 | 2576861064 | https://img.jgi.doe.gov/cgi-bin/m/main.cgi?section=TaxonDetail&page=taxonDetail&taxon_oid=2576861064 |
|  | *Mycobacterium tuberculosis* KT-0104 | 2588254227 | https://img.jgi.doe.gov/cgi-bin/m/main.cgi?section=TaxonDetail&page=taxonDetail&taxon_oid=2588254227 |
|  | *Mycobacterium tuberculosis* KT-0089 | 2588254232 | https://img.jgi.doe.gov/cgi-bin/m/main.cgi?section=TaxonDetail&page=taxonDetail&taxon_oid=2588254232 |
|  | *Mycobacterium tuberculosis* TRUG0085 | 2576861121 | https://img.jgi.doe.gov/cgi-bin/m/main.cgi?section=TaxonDetail&page=taxonDetail&taxon_oid=2576861121 |
|  | *Mycobacterium tuberculosis* PanR0203 | 2554235210 | https://img.jgi.doe.gov/cgi-bin/m/main.cgi?section=TaxonDetail&page=taxonDetail&taxon_oid=2554235210 |
|  | *Mycobacterium tuberculosis* TKK_03_0031 | 2576861265 | https://img.jgi.doe.gov/cgi-bin/m/main.cgi?section=TaxonDetail&page=taxonDetail&taxon_oid=2576861265 |
|  | *Mycobacterium tuberculosis* MAL010108 | 2588254099 | https://img.jgi.doe.gov/cgi-bin/m/main.cgi?section=TaxonDetail&page=taxonDetail&taxon_oid=2588254099 |
|  | *Mycobacterium tuberculosis* TB_RSA46 | 2582581191 | https://img.jgi.doe.gov/cgi-bin/m/main.cgi?section=TaxonDetail&page=taxonDetail&taxon_oid=2582581191 |
|  | *Mycobacterium tuberculosis* TKK_03_0027 | 2590828620 | https://img.jgi.doe.gov/cgi-bin/m/main.cgi?section=TaxonDetail&page=taxonDetail&taxon_oid=2590828620 |
|  | *Mycobacterium tuberculosis* TKK_05SA_0048 | 2574180155 | https://img.jgi.doe.gov/cgi-bin/m/main.cgi?section=TaxonDetail&page=taxonDetail&taxon_oid=2574180155 |
|  | *Mycobacterium tuberculosis* KT-0100 | 2576861242 | https://img.jgi.doe.gov/cgi-bin/m/main.cgi?section=TaxonDetail&page=taxonDetail&taxon_oid=2576861242 |
|  | *Mycobacterium tuberculosis* TKK_02_0006 | 2590828650 | https://img.jgi.doe.gov/cgi-bin/m/main.cgi?section=TaxonDetail&page=taxonDetail&taxon_oid=2590828650 |
|  | *Mycobacterium tuberculosis* TKK-01-0057 | 2588254010 | https://img.jgi.doe.gov/cgi-bin/m/main.cgi?section=TaxonDetail&page=taxonDetail&taxon_oid=2588254010 |
|  | *Mycobacterium tuberculosis* XDR1221 | 2558860631 | https://img.jgi.doe.gov/cgi-bin/m/main.cgi?section=TaxonDetail&page=taxonDetail&taxon_oid=2558860631 |
|  | *Mycobacterium tuberculosis* TKK-01-0093 | 2588254037 | https://img.jgi.doe.gov/cgi-bin/m/main.cgi?section=TaxonDetail&page=taxonDetail&taxon_oid=2588254037 |
|  | *Mycobacterium massiliense* 2B-0107 | 2526164656 | https://img.jgi.doe.gov/cgi-bin/m/main.cgi?section=TaxonDetail&page=taxonDetail&taxon_oid=2526164656 |
|  | *Mycobacterium tuberculosis* 3280CJ | 2574180115 | https://img.jgi.doe.gov/cgi-bin/m/main.cgi?section=TaxonDetail&page=taxonDetail&taxon_oid=2574180115 |
|  | *Mycobacterium bovis* BCG-Tice, TMC 1028 | 2547132086 | https://img.jgi.doe.gov/cgi-bin/m/main.cgi?section=TaxonDetail&page=taxonDetail&taxon_oid=2547132086 |
|  | *Mycobacterium tuberculosis* KT-0085 | 2588254233 | https://img.jgi.doe.gov/cgi-bin/m/main.cgi?section=TaxonDetail&page=taxonDetail&taxon_oid=2588254233 |
|  | *Mycobacterium tuberculosis* KT-0077 | 2588254239 | https://img.jgi.doe.gov/cgi-bin/m/main.cgi?section=TaxonDetail&page=taxonDetail&taxon_oid=2588254239 |
|  | *Mycobacterium tuberculosis* 2541MS | 2574180315 | https://img.jgi.doe.gov/cgi-bin/m/main.cgi?section=TaxonDetail&page=taxonDetail&taxon_oid=2574180315 |
|  | *Mycobacterium tuberculosis* TBR55 | 2574179989 | https://img.jgi.doe.gov/cgi-bin/m/main.cgi?section=TaxonDetail&page=taxonDetail&taxon_oid=2574179989 |
|  | *Mycobacterium tuberculosis* PanR0908 | 2554235272 | https://img.jgi.doe.gov/cgi-bin/m/main.cgi?section=TaxonDetail&page=taxonDetail&taxon_oid=2554235272 |
|  | *Mycobacterium tuberculosis* NRITLD57 | 2574180032 | https://img.jgi.doe.gov/cgi-bin/m/main.cgi?section=TaxonDetail&page=taxonDetail&taxon_oid=2574180032 |
|  | *Mycobacterium bovis* BCG Mexico | 2511231152 | https://img.jgi.doe.gov/cgi-bin/m/main.cgi?section=TaxonDetail&page=taxonDetail&taxon_oid=2511231152 |
|  | *Mycobacterium mucogenicum* 261Sha1.1M5 | 2548877166 | https://img.jgi.doe.gov/cgi-bin/m/main.cgi?section=TaxonDetail&page=taxonDetail&taxon_oid=2548877166 |
|  | *Mycobacterium tuberculosis* BTB07-034 | 2576861163 | https://img.jgi.doe.gov/cgi-bin/m/main.cgi?section=TaxonDetail&page=taxonDetail&taxon_oid=2576861163 |
|  | *Mycobacterium tuberculosis* NRITLD12 | 2574179824 | https://img.jgi.doe.gov/cgi-bin/m/main.cgi?section=TaxonDetail&page=taxonDetail&taxon_oid=2574179824 |
|  | *Mycobacterium tuberculosis* OFXR-26 | 2576861035 | https://img.jgi.doe.gov/cgi-bin/m/main.cgi?section=TaxonDetail&page=taxonDetail&taxon_oid=2576861035 |
|  | *Mycobacterium tuberculosis* 2091HD | 2582581157 | https://img.jgi.doe.gov/cgi-bin/m/main.cgi?section=TaxonDetail&page=taxonDetail&taxon_oid=2582581157 |
|  | *Mycobacterium tuberculosis* PanR0609 | 2554235268 | https://img.jgi.doe.gov/cgi-bin/m/main.cgi?section=TaxonDetail&page=taxonDetail&taxon_oid=2554235268 |
|  | *Mycobacterium tuberculosis* MAL020146 | 2588254115 | https://img.jgi.doe.gov/cgi-bin/m/main.cgi?section=TaxonDetail&page=taxonDetail&taxon_oid=2588254115 |
|  | *Mycobacterium tuberculosis* TBR47 | 2588253881 | https://img.jgi.doe.gov/cgi-bin/m/main.cgi?section=TaxonDetail&page=taxonDetail&taxon_oid=2588253881 |
|  | *Mycobacterium tuberculosis* KT-0011 | 2582581202 | https://img.jgi.doe.gov/cgi-bin/m/main.cgi?section=TaxonDetail&page=taxonDetail&taxon_oid=2582581202 |
|  | *Mycobacterium tuberculosis* TKK-01-0085 | 2574179808 | https://img.jgi.doe.gov/cgi-bin/m/main.cgi?section=TaxonDetail&page=taxonDetail&taxon_oid=2574179808 |
|  | *Mycobacterium tuberculosis* TKK-01-0041 | 2574180165 | https://img.jgi.doe.gov/cgi-bin/m/main.cgi?section=TaxonDetail&page=taxonDetail&taxon_oid=2574180165 |
|  | *Mycobacterium tuberculosis* TKK-01-0018 | 2588253987 | https://img.jgi.doe.gov/cgi-bin/m/main.cgi?section=TaxonDetail&page=taxonDetail&taxon_oid=2588253987 |
|  | *Mycobacterium africanum* MAL010100 | 2574179914 | https://img.jgi.doe.gov/cgi-bin/m/main.cgi?section=TaxonDetail&page=taxonDetail&taxon_oid=2574179914 |
|  | *Mycobacterium bovis* Kc 9614 | 2579778584 | https://img.jgi.doe.gov/cgi-bin/m/main.cgi?section=TaxonDetail&page=taxonDetail&taxon_oid=2579778584 |
|  | *Mycobacterium tuberculosis* TKK_03_0028 | 2574180204 | https://img.jgi.doe.gov/cgi-bin/m/main.cgi?section=TaxonDetail&page=taxonDetail&taxon_oid=2574180204 |
|  | *Mycobacterium tuberculosis* SUMu003 | 648276695 | https://img.jgi.doe.gov/cgi-bin/m/main.cgi?section=TaxonDetail&page=taxonDetail&taxon_oid=648276695 |
|  | *Mycobacterium* sp. H4Y | 2531839247 | https://img.jgi.doe.gov/cgi-bin/m/main.cgi?section=TaxonDetail&page=taxonDetail&taxon_oid=2531839247 |
|  | *Mycobacterium tuberculosis* TKK-01-0017 | 2588253845 | https://img.jgi.doe.gov/cgi-bin/m/main.cgi?section=TaxonDetail&page=taxonDetail&taxon_oid=2588253845 |
|  | *Mycobacterium tuberculosis* TB_RSA121 | 2582581152 | https://img.jgi.doe.gov/cgi-bin/m/main.cgi?section=TaxonDetail&page=taxonDetail&taxon_oid=2582581152 |
|  | *Mycobacterium tuberculosis* TKK-01-0053 | 2574180042 | https://img.jgi.doe.gov/cgi-bin/m/main.cgi?section=TaxonDetail&page=taxonDetail&taxon_oid=2574180042 |
|  | *Mycobacterium tuberculosis* XTB13-121 | 2576861080 | https://img.jgi.doe.gov/cgi-bin/m/main.cgi?section=TaxonDetail&page=taxonDetail&taxon_oid=2576861080 |
|  | *Mycobacterium tuberculosis* 94_M4241A | 642979310 | https://img.jgi.doe.gov/cgi-bin/m/main.cgi?section=TaxonDetail&page=taxonDetail&taxon_oid=642979310 |
|  | *Mycobacterium tuberculosis* BTB07-206 | 2574179835 | https://img.jgi.doe.gov/cgi-bin/m/main.cgi?section=TaxonDetail&page=taxonDetail&taxon_oid=2574179835 |
|  | *Mycobacterium tuberculosis* MD20344 | 2576861179 | https://img.jgi.doe.gov/cgi-bin/m/main.cgi?section=TaxonDetail&page=taxonDetail&taxon_oid=2576861179 |
|  | *Mycobacterium tuberculosis* MAL020202 | 2588254135 | https://img.jgi.doe.gov/cgi-bin/m/main.cgi?section=TaxonDetail&page=taxonDetail&taxon_oid=2588254135 |
|  | *Mycobacterium tuberculosis* TKK_05MA_2015 | 2574180371 | https://img.jgi.doe.gov/cgi-bin/m/main.cgi?section=TaxonDetail&page=taxonDetail&taxon_oid=2574180371 |
|  | *Mycobacterium tuberculosis* CAS/NITR204 | 2545824629 | https://img.jgi.doe.gov/cgi-bin/m/main.cgi?section=TaxonDetail&page=taxonDetail&taxon_oid=2545824629 |
|  | *Mycobacterium tuberculosis* M2129 | 2582581223 | https://img.jgi.doe.gov/cgi-bin/m/main.cgi?section=TaxonDetail&page=taxonDetail&taxon_oid=2582581223 |
|  | *Mycobacterium tuberculosis* MAL010106 | 2588254100 | https://img.jgi.doe.gov/cgi-bin/m/main.cgi?section=TaxonDetail&page=taxonDetail&taxon_oid=2588254100 |
|  | *Mycobacterium africanum* MAL020107 | 2579779012 | https://img.jgi.doe.gov/cgi-bin/m/main.cgi?section=TaxonDetail&page=taxonDetail&taxon_oid=2579779012 |
|  | *Mycobacterium hassiacum* DSM 44199 | 2515154012 | https://img.jgi.doe.gov/cgi-bin/m/main.cgi?section=TaxonDetail&page=taxonDetail&taxon_oid=2515154012 |
|  | *Mycobacterium tuberculosis* TKK_02_0070 | 2590828627 | https://img.jgi.doe.gov/cgi-bin/m/main.cgi?section=TaxonDetail&page=taxonDetail&taxon_oid=2590828627 |
|  | *Mycobacterium tuberculosis* TKK-01-0009 | 2588253978 | https://img.jgi.doe.gov/cgi-bin/m/main.cgi?section=TaxonDetail&page=taxonDetail&taxon_oid=2588253978 |
|  | *Mycobacterium tuberculosis* H2438 | 2582581215 | https://img.jgi.doe.gov/cgi-bin/m/main.cgi?section=TaxonDetail&page=taxonDetail&taxon_oid=2582581215 |
|  | *Mycobacterium tuberculosis* NA-A0008 | 2551306118 | https://img.jgi.doe.gov/cgi-bin/m/main.cgi?section=TaxonDetail&page=taxonDetail&taxon_oid=2551306118 |
|  | *Mycobacterium tuberculosis* BTB12-314 | 2576861051 | https://img.jgi.doe.gov/cgi-bin/m/main.cgi?section=TaxonDetail&page=taxonDetail&taxon_oid=2576861051 |
|  | *Mycobacterium tuberculosis* S96-129 | 2547132258 | https://img.jgi.doe.gov/cgi-bin/m/main.cgi?section=TaxonDetail&page=taxonDetail&taxon_oid=2547132258 |
|  | *Mycobacterium tuberculosis* TKK-01-0061 | 2574179969 | https://img.jgi.doe.gov/cgi-bin/m/main.cgi?section=TaxonDetail&page=taxonDetail&taxon_oid=2574179969 |
|  | *Mycobacterium tuberculosis* H37Ra | 640427124 | https://img.jgi.doe.gov/cgi-bin/m/main.cgi?section=TaxonDetail&page=taxonDetail&taxon_oid=640427124 |
|  | *Mycobacterium africanum* K85 | 2582580994 | https://img.jgi.doe.gov/cgi-bin/m/main.cgi?section=TaxonDetail&page=taxonDetail&taxon_oid=2582580994 |
|  | *Mycobacterium tuberculosis* TKK_04_0007 | 2590828607 | https://img.jgi.doe.gov/cgi-bin/m/main.cgi?section=TaxonDetail&page=taxonDetail&taxon_oid=2590828607 |
|  | *Mycobacterium africanum* MAL010128 | 2579779032 | https://img.jgi.doe.gov/cgi-bin/m/main.cgi?section=TaxonDetail&page=taxonDetail&taxon_oid=2579779032 |
|  | *Mycobacterium vaccae* RIVM | 2534681942 | https://img.jgi.doe.gov/cgi-bin/m/main.cgi?section=TaxonDetail&page=taxonDetail&taxon_oid=2534681942 |
|  | *Mycobacterium tuberculosis* INS_XDR | 2571042743 | https://img.jgi.doe.gov/cgi-bin/m/main.cgi?section=TaxonDetail&page=taxonDetail&taxon_oid=2571042743 |
|  | *Mycobacterium tuberculosis* TKK-01-0039 | 2588253998 | https://img.jgi.doe.gov/cgi-bin/m/main.cgi?section=TaxonDetail&page=taxonDetail&taxon_oid=2588253998 |
|  | *Mycobacterium tuberculosis* PanR0314 | 2554235245 | https://img.jgi.doe.gov/cgi-bin/m/main.cgi?section=TaxonDetail&page=taxonDetail&taxon_oid=2554235245 |
|  | *Mycobacterium tuberculosis* TKK_02_0033 | 2590828639 | https://img.jgi.doe.gov/cgi-bin/m/main.cgi?section=TaxonDetail&page=taxonDetail&taxon_oid=2590828639 |
|  | *Mycobacterium tuberculosis* PanR0907 | 2554235280 | https://img.jgi.doe.gov/cgi-bin/m/main.cgi?section=TaxonDetail&page=taxonDetail&taxon_oid=2554235280 |
|  | *Mycobacterium tuberculosis* K | 2588253735 | https://img.jgi.doe.gov/cgi-bin/m/main.cgi?section=TaxonDetail&page=taxonDetail&taxon_oid=2588253735 |
|  | *Mycobacterium tuberculosis* TKK_03_0081 | 2590828612 | https://img.jgi.doe.gov/cgi-bin/m/main.cgi?section=TaxonDetail&page=taxonDetail&taxon_oid=2590828612 |
|  | *Mycobacterium tuberculosis* TBR41 | 2574180180 | https://img.jgi.doe.gov/cgi-bin/m/main.cgi?section=TaxonDetail&page=taxonDetail&taxon_oid=2574180180 |
|  | *Mycobacterium tuberculosis* MAL010134 | 2588254110 | https://img.jgi.doe.gov/cgi-bin/m/main.cgi?section=TaxonDetail&page=taxonDetail&taxon_oid=2588254110 |
|  | *Mycobacterium intracellulare* ATCC 13950 | 645058739 | https://img.jgi.doe.gov/cgi-bin/m/main.cgi?section=TaxonDetail&page=taxonDetail&taxon_oid=645058739 |
|  | *Mycobacterium tuberculosis* XTB13-194 | 2582581140 | https://img.jgi.doe.gov/cgi-bin/m/main.cgi?section=TaxonDetail&page=taxonDetail&taxon_oid=2582581140 |
|  | *Mycobacterium tuberculosis* TKK_03_0090 | 2574180410 | https://img.jgi.doe.gov/cgi-bin/m/main.cgi?section=TaxonDetail&page=taxonDetail&taxon_oid=2574180410 |
|  | *Mycobacterium tuberculosis* MAL020102 | 2588254109 | https://img.jgi.doe.gov/cgi-bin/m/main.cgi?section=TaxonDetail&page=taxonDetail&taxon_oid=2588254109 |
|  | *Mycobacterium tuberculosis* TKK-01-0038 | 2582581137 | https://img.jgi.doe.gov/cgi-bin/m/main.cgi?section=TaxonDetail&page=taxonDetail&taxon_oid=2582581137 |
|  | *Mycobacterium africanum* GM041182 | 650716059 | https://img.jgi.doe.gov/cgi-bin/m/main.cgi?section=TaxonDetail&page=taxonDetail&taxon_oid=650716059 |
|  | *Mycobacterium tuberculosis* TKK_05SA_0017 | 2576861332 | https://img.jgi.doe.gov/cgi-bin/m/main.cgi?section=TaxonDetail&page=taxonDetail&taxon_oid=2576861332 |
|  | *Mycobacterium tuberculosis* TBR40 | 2588253876 | https://img.jgi.doe.gov/cgi-bin/m/main.cgi?section=TaxonDetail&page=taxonDetail&taxon_oid=2588253876 |
|  | *Mycobacterium tuberculosis* TBR66 | 2588253888 | https://img.jgi.doe.gov/cgi-bin/m/main.cgi?section=TaxonDetail&page=taxonDetail&taxon_oid=2588253888 |
|  | *Mycobacterium* sp. UNCCL9 | 2576861824 | https://img.jgi.doe.gov/cgi-bin/m/main.cgi?section=TaxonDetail&page=taxonDetail&taxon_oid=2576861824 |
|  | *Mycobacterium tuberculosis* KT-0028 | 2588254152 | https://img.jgi.doe.gov/cgi-bin/m/main.cgi?section=TaxonDetail&page=taxonDetail&taxon_oid=2588254152 |
|  | *Mycobacterium tuberculosis* KT-0063 | 2588254143 | https://img.jgi.doe.gov/cgi-bin/m/main.cgi?section=TaxonDetail&page=taxonDetail&taxon_oid=2588254143 |
|  | *Mycobacterium tuberculosis* Haarlem3/NITR202 | 2545824626 | https://img.jgi.doe.gov/cgi-bin/m/main.cgi?section=TaxonDetail&page=taxonDetail&taxon_oid=2545824626 |
|  | *Mycobacterium xenopi* RIVM700367 | 2522572167 | https://img.jgi.doe.gov/cgi-bin/m/main.cgi?section=TaxonDetail&page=taxonDetail&taxon_oid=2522572167 |
|  | *Mycobacterium tuberculosis* KT-0071 | 2588254241 | https://img.jgi.doe.gov/cgi-bin/m/main.cgi?section=TaxonDetail&page=taxonDetail&taxon_oid=2588254241 |
|  | *Mycobacterium orygis* 112400015 | 2545824691 | https://img.jgi.doe.gov/cgi-bin/m/main.cgi?section=TaxonDetail&page=taxonDetail&taxon_oid=2545824691 |
|  | *Mycobacterium tuberculosis* TKK_05SA_0058 | 2574179931 | https://img.jgi.doe.gov/cgi-bin/m/main.cgi?section=TaxonDetail&page=taxonDetail&taxon_oid=2574179931 |
|  | *Mycobacterium tuberculosis* M2085 | 2576861292 | https://img.jgi.doe.gov/cgi-bin/m/main.cgi?section=TaxonDetail&page=taxonDetail&taxon_oid=2576861292 |
|  | *Mycobacterium tuberculosis* PanR0904 | 2554235277 | https://img.jgi.doe.gov/cgi-bin/m/main.cgi?section=TaxonDetail&page=taxonDetail&taxon_oid=2554235277 |
|  | *Mycobacterium tuberculosis* PanR0802 | 2554235294 | https://img.jgi.doe.gov/cgi-bin/m/main.cgi?section=TaxonDetail&page=taxonDetail&taxon_oid=2554235294 |
|  | *Mycobacterium tuberculosis* KT-0008 | 2588254160 | https://img.jgi.doe.gov/cgi-bin/m/main.cgi?section=TaxonDetail&page=taxonDetail&taxon_oid=2588254160 |
|  | *Mycobacterium tuberculosis* KT-0004 | 2574179997 | https://img.jgi.doe.gov/cgi-bin/m/main.cgi?section=TaxonDetail&page=taxonDetail&taxon_oid=2574179997 |
|  | *Mycobacterium tuberculosis* M1787 | 2579778511 | https://img.jgi.doe.gov/cgi-bin/m/main.cgi?section=TaxonDetail&page=taxonDetail&taxon_oid=2579778511 |
|  | *Mycobacterium tuberculosis* F11 (ExPEC) | 640427123 | https://img.jgi.doe.gov/cgi-bin/m/main.cgi?section=TaxonDetail&page=taxonDetail&taxon_oid=640427123 |
|  | *Mycobacterium tuberculosis* UM 1072388579 | 2551306486 | https://img.jgi.doe.gov/cgi-bin/m/main.cgi?section=TaxonDetail&page=taxonDetail&taxon_oid=2551306486 |
|  | *Mycobacterium tuberculosis* TBR58 | 2574179887 | https://img.jgi.doe.gov/cgi-bin/m/main.cgi?section=TaxonDetail&page=taxonDetail&taxon_oid=2574179887 |
|  | *Mycobacterium tuberculosis* XTB13-082 | 2582581155 | https://img.jgi.doe.gov/cgi-bin/m/main.cgi?section=TaxonDetail&page=taxonDetail&taxon_oid=2582581155 |
|  | *Mycobacterium tuberculosis* TKK_02_0003 | 2590828651 | https://img.jgi.doe.gov/cgi-bin/m/main.cgi?section=TaxonDetail&page=taxonDetail&taxon_oid=2590828651 |
|  | *Mycobacterium tuberculosis* TKK-01-0029 | 2588253994 | https://img.jgi.doe.gov/cgi-bin/m/main.cgi?section=TaxonDetail&page=taxonDetail&taxon_oid=2588253994 |
|  | *Mycobacterium tuberculosis* TKK_04_0039 | 2590828676 | https://img.jgi.doe.gov/cgi-bin/m/main.cgi?section=TaxonDetail&page=taxonDetail&taxon_oid=2590828676 |
|  | *Mycobacterium tuberculosis* TKK_04_0031 | 2590828680 | https://img.jgi.doe.gov/cgi-bin/m/main.cgi?section=TaxonDetail&page=taxonDetail&taxon_oid=2590828680 |
|  | *Mycobacterium tuberculosis* TKK-01-0044 | 2588254003 | https://img.jgi.doe.gov/cgi-bin/m/main.cgi?section=TaxonDetail&page=taxonDetail&taxon_oid=2588254003 |
|  | *Mycobacterium tuberculosis* TKK_04_0054 | 2576861144 | https://img.jgi.doe.gov/cgi-bin/m/main.cgi?section=TaxonDetail&page=taxonDetail&taxon_oid=2576861144 |
|  | *Mycobacterium tuberculosis* TKK-01-0063 | 2588254015 | https://img.jgi.doe.gov/cgi-bin/m/main.cgi?section=TaxonDetail&page=taxonDetail&taxon_oid=2588254015 |
|  | *Mycobacterium tuberculosis* XTB13-290 | 2582581214 | https://img.jgi.doe.gov/cgi-bin/m/main.cgi?section=TaxonDetail&page=taxonDetail&taxon_oid=2582581214 |
|  | *Mycobacterium tuberculosis* TKK_04_0046 | 2590828674 | https://img.jgi.doe.gov/cgi-bin/m/main.cgi?section=TaxonDetail&page=taxonDetail&taxon_oid=2590828674 |
|  | *Mycobacterium tuberculosis* M1481 | 2574179996 | https://img.jgi.doe.gov/cgi-bin/m/main.cgi?section=TaxonDetail&page=taxonDetail&taxon_oid=2574179996 |
|  | *Mycobacterium tuberculosis* 1010SM | 2582581173 | https://img.jgi.doe.gov/cgi-bin/m/main.cgi?section=TaxonDetail&page=taxonDetail&taxon_oid=2582581173 |
|  | *Mycobacterium tuberculosis* TBR43 | 2588253879 | https://img.jgi.doe.gov/cgi-bin/m/main.cgi?section=TaxonDetail&page=taxonDetail&taxon_oid=2588253879 |
|  | *Mycobacterium tuberculosis* TKK-01-0042 | 2588254000 | https://img.jgi.doe.gov/cgi-bin/m/main.cgi?section=TaxonDetail&page=taxonDetail&taxon_oid=2588254000 |
|  | *Mycobacterium bovis Bz* 31150 | 2579778928 | https://img.jgi.doe.gov/cgi-bin/m/main.cgi?section=TaxonDetail&page=taxonDetail&taxon_oid=2579778928 |
|  | *Mycobacterium tuberculosis* TKK_04_0080 | 2574179845 | https://img.jgi.doe.gov/cgi-bin/m/main.cgi?section=TaxonDetail&page=taxonDetail&taxon_oid=2574179845 |
|  | *Mycobacterium tuberculosis* TB_RSA74 | 2576861342 | https://img.jgi.doe.gov/cgi-bin/m/main.cgi?section=TaxonDetail&page=taxonDetail&taxon_oid=2576861342 |
|  | *Mycobacterium tuberculosis* XTB13-081 | 2574180407 | https://img.jgi.doe.gov/cgi-bin/m/main.cgi?section=TaxonDetail&page=taxonDetail&taxon_oid=2574180407 |
|  | *Mycobacterium tuberculosis* SUMu011 | 648276703 | https://img.jgi.doe.gov/cgi-bin/m/main.cgi?section=TaxonDetail&page=taxonDetail&taxon_oid=648276703 |
|  | *Mycobacterium tuberculosis* TKK_04_0117 | 2574180398 | https://img.jgi.doe.gov/cgi-bin/m/main.cgi?section=TaxonDetail&page=taxonDetail&taxon_oid=2574180398 |
|  | *Mycobacterium tuberculosis* TKK-01-0026 | 2576861324 | https://img.jgi.doe.gov/cgi-bin/m/main.cgi?section=TaxonDetail&page=taxonDetail&taxon_oid=2576861324 |
|  | *Mycobacterium tuberculosis* M1703 | 2582581158 | https://img.jgi.doe.gov/cgi-bin/m/main.cgi?section=TaxonDetail&page=taxonDetail&taxon_oid=2582581158 |
|  | *Mycobacterium tuberculosis* TBR37 | 2574180426 | https://img.jgi.doe.gov/cgi-bin/m/main.cgi?section=TaxonDetail&page=taxonDetail&taxon_oid=2574180426 |
|  | *Mycobacterium tuberculosis* KT-0045 | 2588254148 | https://img.jgi.doe.gov/cgi-bin/m/main.cgi?section=TaxonDetail&page=taxonDetail&taxon_oid=2588254148 |
|  | *Mycobacterium tuberculosis* TKK_03_0035 | 2585427842 | https://img.jgi.doe.gov/cgi-bin/m/main.cgi?section=TaxonDetail&page=taxonDetail&taxon_oid=2585427842 |
|  | *Mycobacterium tuberculosis* TKK_04_0019 | 2574179963 | https://img.jgi.doe.gov/cgi-bin/m/main.cgi?section=TaxonDetail&page=taxonDetail&taxon_oid=2574179963 |
|  | *Mycobacterium tuberculosis* XTB13-251 | 2574179953 | https://img.jgi.doe.gov/cgi-bin/m/main.cgi?section=TaxonDetail&page=taxonDetail&taxon_oid=2574179953 |
|  | *Mycobacterium tuberculosis* TKK_03_0118 | 2576861301 | https://img.jgi.doe.gov/cgi-bin/m/main.cgi?section=TaxonDetail&page=taxonDetail&taxon_oid=2576861301 |
|  | *Mycobacterium tuberculosis* PanR0311 | 2554235240 | https://img.jgi.doe.gov/cgi-bin/m/main.cgi?section=TaxonDetail&page=taxonDetail&taxon_oid=2554235240 |
|  | *Mycobacterium tuberculosis* MAL020120 | 2576861089 | https://img.jgi.doe.gov/cgi-bin/m/main.cgi?section=TaxonDetail&page=taxonDetail&taxon_oid=2576861089 |
|  | *Mycobacterium indicuspranii* MTCC 9506 | 2521172703 | https://img.jgi.doe.gov/cgi-bin/m/main.cgi?section=TaxonDetail&page=taxonDetail&taxon_oid=2521172703 |
|  | *Mycobacterium tuberculosis* PanR1101 | 2554235276 | https://img.jgi.doe.gov/cgi-bin/m/main.cgi?section=TaxonDetail&page=taxonDetail&taxon_oid=2554235276 |
|  | *Mycobacterium tuberculosis* CCDC5079 | 651053043 | https://img.jgi.doe.gov/cgi-bin/m/main.cgi?section=TaxonDetail&page=taxonDetail&taxon_oid=651053043 |
|  | *Mycobacterium tuberculosis* TKK_03_0025 | 2590828621 | https://img.jgi.doe.gov/cgi-bin/m/main.cgi?section=TaxonDetail&page=taxonDetail&taxon_oid=2590828621 |
|  | *Mycobacterium tuberculosis* OFXR-5 | 2588253897 | https://img.jgi.doe.gov/cgi-bin/m/main.cgi?section=TaxonDetail&page=taxonDetail&taxon_oid=2588253897 |
|  | *Mycobacterium tuberculosis* BTB11-001 | 2574180074 | https://img.jgi.doe.gov/cgi-bin/m/main.cgi?section=TaxonDetail&page=taxonDetail&taxon_oid=2574180074 |
|  | *Mycobacterium tuberculosis* M1023 | 2576861102 | https://img.jgi.doe.gov/cgi-bin/m/main.cgi?section=TaxonDetail&page=taxonDetail&taxon_oid=2576861102 |
|  | *Mycobacterium tuberculosis* OFXR-8 | 2574180057 | https://img.jgi.doe.gov/cgi-bin/m/main.cgi?section=TaxonDetail&page=taxonDetail&taxon_oid=2574180057 |
|  | *Mycobacterium africanum* MAL010137 | 2574180130 | https://img.jgi.doe.gov/cgi-bin/m/main.cgi?section=TaxonDetail&page=taxonDetail&taxon_oid=2574180130 |
|  | *Mycobacterium tuberculosis* KZN 605 (XDR) | 645058861 | https://img.jgi.doe.gov/cgi-bin/m/main.cgi?section=TaxonDetail&page=taxonDetail&taxon_oid=645058861 |
|  | *Mycobacterium tuberculosis* M1956 | 2582581134 | https://img.jgi.doe.gov/cgi-bin/m/main.cgi?section=TaxonDetail&page=taxonDetail&taxon_oid=2582581134 |
|  | *Mycobacterium tuberculosis* TKK-01-0069 | 2588253848 | https://img.jgi.doe.gov/cgi-bin/m/main.cgi?section=TaxonDetail&page=taxonDetail&taxon_oid=2588253848 |
|  | *Mycobacterium tuberculosis* KT-0007 | 2588254161 | https://img.jgi.doe.gov/cgi-bin/m/main.cgi?section=TaxonDetail&page=taxonDetail&taxon_oid=2588254161 |
|  | *Mycobacterium tuberculosis* PanR0316 | 2554235250 | https://img.jgi.doe.gov/cgi-bin/m/main.cgi?section=TaxonDetail&page=taxonDetail&taxon_oid=2554235250 |
|  | *Mycobacterium tuberculosis* KT-0040 | 2574180203 | https://img.jgi.doe.gov/cgi-bin/m/main.cgi?section=TaxonDetail&page=taxonDetail&taxon_oid=2574180203 |
|  | *Mycobacterium tuberculosis* TKK_03_0083 | 2576861224 | https://img.jgi.doe.gov/cgi-bin/m/main.cgi?section=TaxonDetail&page=taxonDetail&taxon_oid=2576861224 |
|  | *Mycobacterium tuberculosis* MAL020194 | 2588254128 | https://img.jgi.doe.gov/cgi-bin/m/main.cgi?section=TaxonDetail&page=taxonDetail&taxon_oid=2588254128 |
|  | *Mycobacterium tuberculosis* TKK-01-0013 | 2588253984 | https://img.jgi.doe.gov/cgi-bin/m/main.cgi?section=TaxonDetail&page=taxonDetail&taxon_oid=2588253984 |
|  | *Mycobacterium* sp. UNC410CL29Cvi84 | 2563366507 | https://img.jgi.doe.gov/cgi-bin/m/main.cgi?section=TaxonDetail&page=taxonDetail&taxon_oid=2563366507 |
|  | *Mycobacterium tuberculosis* PanR0308 | 2554235237 | https://img.jgi.doe.gov/cgi-bin/m/main.cgi?section=TaxonDetail&page=taxonDetail&taxon_oid=2554235237 |
|  | *Mycobacterium tuberculosis* TKK-01-0037 | 2576861280 | https://img.jgi.doe.gov/cgi-bin/m/main.cgi?section=TaxonDetail&page=taxonDetail&taxon_oid=2576861280 |
|  | *Mycobacterium tuberculosis* TKK-01-0062 | 2588254014 | https://img.jgi.doe.gov/cgi-bin/m/main.cgi?section=TaxonDetail&page=taxonDetail&taxon_oid=2588254014 |
|  | *Mycobacterium tuberculosis* KT-0002 | 2588254163 | https://img.jgi.doe.gov/cgi-bin/m/main.cgi?section=TaxonDetail&page=taxonDetail&taxon_oid=2588254163 |
|  | *Mycobacterium tuberculosis* SK-B | 2574180419 | https://img.jgi.doe.gov/cgi-bin/m/main.cgi?section=TaxonDetail&page=taxonDetail&taxon_oid=2574180419 |
|  | *Mycobacterium tuberculosis* KT-0092 | 2588254231 | https://img.jgi.doe.gov/cgi-bin/m/main.cgi?section=TaxonDetail&page=taxonDetail&taxon_oid=2588254231 |
|  | *Mycobacterium tuberculosis* TKK_04_0051 | 2574180317 | https://img.jgi.doe.gov/cgi-bin/m/main.cgi?section=TaxonDetail&page=taxonDetail&taxon_oid=2574180317 |
|  | *Mycobacterium tuberculosis* KT-0023 | 2588254154 | https://img.jgi.doe.gov/cgi-bin/m/main.cgi?section=TaxonDetail&page=taxonDetail&taxon_oid=2588254154 |
|  | *Mycobacterium tuberculosis* TKK_05SA_0042 | 2582581190 | https://img.jgi.doe.gov/cgi-bin/m/main.cgi?section=TaxonDetail&page=taxonDetail&taxon_oid=2582581190 |
|  | *Mycobacterium tuberculosis* MAL020162 | 2574180078 | https://img.jgi.doe.gov/cgi-bin/m/main.cgi?section=TaxonDetail&page=taxonDetail&taxon_oid=2574180078 |
|  | *Mycobacterium tuberculosis* TB_RSA21 | 2576861264 | https://img.jgi.doe.gov/cgi-bin/m/main.cgi?section=TaxonDetail&page=taxonDetail&taxon_oid=2576861264 |
|  | *Mycobacterium bovis* BCG Pasteur 1173P2 | 639633040 | https://img.jgi.doe.gov/cgi-bin/m/main.cgi?section=TaxonDetail&page=taxonDetail&taxon_oid=639633040 |
|  | *Mycobacterium tuberculosis* MAL020132 | 2588254107 | https://img.jgi.doe.gov/cgi-bin/m/main.cgi?section=TaxonDetail&page=taxonDetail&taxon_oid=2588254107 |
|  | *Mycobacterium tuberculosis* M1444 | 2574180216 | https://img.jgi.doe.gov/cgi-bin/m/main.cgi?section=TaxonDetail&page=taxonDetail&taxon_oid=2574180216 |
|  | *Mycobacterium tuberculosis* TBR53 | 2588253886 | https://img.jgi.doe.gov/cgi-bin/m/main.cgi?section=TaxonDetail&page=taxonDetail&taxon_oid=2588253886 |
|  | *Mycobacterium tuberculosis* MAL020174 | 2588254121 | https://img.jgi.doe.gov/cgi-bin/m/main.cgi?section=TaxonDetail&page=taxonDetail&taxon_oid=2588254121 |
|  | *Mycobacterium bovis* AF 2122/97 | 637000169 | https://img.jgi.doe.gov/cgi-bin/m/main.cgi?section=TaxonDetail&page=taxonDetail&taxon_oid=637000169 |
|  | *Mycobacterium tuberculosis* TKK_04_0040 | 2576861019 | https://img.jgi.doe.gov/cgi-bin/m/main.cgi?section=TaxonDetail&page=taxonDetail&taxon_oid=2576861019 |
|  | *Mycobacterium tuberculosis* KT-0042 | 2574179907 | https://img.jgi.doe.gov/cgi-bin/m/main.cgi?section=TaxonDetail&page=taxonDetail&taxon_oid=2574179907 |
|  | *Mycobacterium tuberculosis* TKK_02_0014 | 2582581209 | https://img.jgi.doe.gov/cgi-bin/m/main.cgi?section=TaxonDetail&page=taxonDetail&taxon_oid=2582581209 |
|  | *Mycobacterium tuberculosis* M2137 | 2576861176 | https://img.jgi.doe.gov/cgi-bin/m/main.cgi?section=TaxonDetail&page=taxonDetail&taxon_oid=2576861176 |
|  | *Mycobacterium bovis* D 4155 | 2579778729 | https://img.jgi.doe.gov/cgi-bin/m/main.cgi?section=TaxonDetail&page=taxonDetail&taxon_oid=2579778729 |
|  | *Mycobacterium tuberculosis* TKK-01-0089 | 2588254027 | https://img.jgi.doe.gov/cgi-bin/m/main.cgi?section=TaxonDetail&page=taxonDetail&taxon_oid=2588254027 |
|  | *Mycobacterium tuberculosis* TB_RSA78 | 2576861336 | https://img.jgi.doe.gov/cgi-bin/m/main.cgi?section=TaxonDetail&page=taxonDetail&taxon_oid=2576861336 |
|  | *Mycobacterium tuberculosis* SUMu002 | 648276694 | https://img.jgi.doe.gov/cgi-bin/m/main.cgi?section=TaxonDetail&page=taxonDetail&taxon_oid=648276694 |
|  | *Mycobacterium tuberculosis* MD17888 | 2574179949 | https://img.jgi.doe.gov/cgi-bin/m/main.cgi?section=TaxonDetail&page=taxonDetail&taxon_oid=2574179949 |
|  | *Mycobacterium tuberculosis* KT-0106 | 2588254226 | https://img.jgi.doe.gov/cgi-bin/m/main.cgi?section=TaxonDetail&page=taxonDetail&taxon_oid=2588254226 |
|  | *Mycobacterium tuberculosis* KT-0078 | 2588254238 | https://img.jgi.doe.gov/cgi-bin/m/main.cgi?section=TaxonDetail&page=taxonDetail&taxon_oid=2588254238 |
|  | *Mycobacterium tuberculosis* SUMu007 | 648276699 | https://img.jgi.doe.gov/cgi-bin/m/main.cgi?section=TaxonDetail&page=taxonDetail&taxon_oid=648276699 |
|  | *Mycobacterium tuberculosis* TKK-01-0081 | 2588254026 | https://img.jgi.doe.gov/cgi-bin/m/main.cgi?section=TaxonDetail&page=taxonDetail&taxon_oid=2588254026 |
|  | *Mycobacterium tuberculosis* TB_RSA76 | 2574180299 | https://img.jgi.doe.gov/cgi-bin/m/main.cgi?section=TaxonDetail&page=taxonDetail&taxon_oid=2574180299 |
|  | *Mycobacterium tuberculosis* XTB13-114 | 2574179861 | https://img.jgi.doe.gov/cgi-bin/m/main.cgi?section=TaxonDetail&page=taxonDetail&taxon_oid=2574179861 |
|  | *Mycobacterium tuberculosis* MTB-476 | 2571042747 | https://img.jgi.doe.gov/cgi-bin/m/main.cgi?section=TaxonDetail&page=taxonDetail&taxon_oid=2571042747 |
|  | *Mycobacterium tuberculosis* G-12-005 | 2576861111 | https://img.jgi.doe.gov/cgi-bin/m/main.cgi?section=TaxonDetail&page=taxonDetail&taxon_oid=2576861111 |
|  | *Mycobacterium tuberculosis* TKK_04_0120 | 2574179819 | https://img.jgi.doe.gov/cgi-bin/m/main.cgi?section=TaxonDetail&page=taxonDetail&taxon_oid=2574179819 |
|  | *Mycobacterium tuberculosis* KZN 4207 (DS) | 2511231070 | https://img.jgi.doe.gov/cgi-bin/m/main.cgi?section=TaxonDetail&page=taxonDetail&taxon_oid=2511231070 |
|  | *Mycobacterium africanum* MAL010136 | 2582580876 | https://img.jgi.doe.gov/cgi-bin/m/main.cgi?section=TaxonDetail&page=taxonDetail&taxon_oid=2582580876 |
|  | *Mycobacterium tuberculosis* BTB03-143 | 2574180392 | https://img.jgi.doe.gov/cgi-bin/m/main.cgi?section=TaxonDetail&page=taxonDetail&taxon_oid=2574180392 |
|  | *Mycobacterium tuberculosis* TKK-01-0024 | 2588253990 | https://img.jgi.doe.gov/cgi-bin/m/main.cgi?section=TaxonDetail&page=taxonDetail&taxon_oid=2588253990 |
|  | *Mycobacterium tuberculosis* MD18498 | 2574179919 | https://img.jgi.doe.gov/cgi-bin/m/main.cgi?section=TaxonDetail&page=taxonDetail&taxon_oid=2574179919 |
|  | *Mycobacterium africanum* MAL010131 | 2579778881 | https://img.jgi.doe.gov/cgi-bin/m/main.cgi?section=TaxonDetail&page=taxonDetail&taxon_oid=2579778881 |
|  | *Mycobacterium tuberculosis* TB_RSA134 | 2574180106 | https://img.jgi.doe.gov/cgi-bin/m/main.cgi?section=TaxonDetail&page=taxonDetail&taxon_oid=2574180106 |
|  | *Mycobacterium tuberculosis* TKK_04_0023 | 2590828598 | https://img.jgi.doe.gov/cgi-bin/m/main.cgi?section=TaxonDetail&page=taxonDetail&taxon_oid=2590828598 |
|  | *Mycobacterium tuberculosis* XTB13-110 | 2582581208 | https://img.jgi.doe.gov/cgi-bin/m/main.cgi?section=TaxonDetail&page=taxonDetail&taxon_oid=2582581208 |
|  | *Mycobacterium tuberculosis* OSDD071 | 2548876689 | https://img.jgi.doe.gov/cgi-bin/m/main.cgi?section=TaxonDetail&page=taxonDetail&taxon_oid=2548876689 |
|  | *Mycobacterium massiliense* 1S-151-0930 | 2526164659 | https://img.jgi.doe.gov/cgi-bin/m/main.cgi?section=TaxonDetail&page=taxonDetail&taxon_oid=2526164659 |
|  | *Mycobacterium tuberculosis* MAL020200 | 2588254133 | https://img.jgi.doe.gov/cgi-bin/m/main.cgi?section=TaxonDetail&page=taxonDetail&taxon_oid=2588254133 |
|  | *Mycobacterium tuberculosis* H3986 | 2574180065 | https://img.jgi.doe.gov/cgi-bin/m/main.cgi?section=TaxonDetail&page=taxonDetail&taxon_oid=2574180065 |
|  | *Mycobacterium tuberculosis* BTB12-206 | 2582581197 | https://img.jgi.doe.gov/cgi-bin/m/main.cgi?section=TaxonDetail&page=taxonDetail&taxon_oid=2582581197 |
|  | *Mycobacterium tuberculosis* PanR0902 | 2554235263 | https://img.jgi.doe.gov/cgi-bin/m/main.cgi?section=TaxonDetail&page=taxonDetail&taxon_oid=2554235263 |
|  | *Mycobacterium tuberculosis* TB_RSA97 | 2574180375 | https://img.jgi.doe.gov/cgi-bin/m/main.cgi?section=TaxonDetail&page=taxonDetail&taxon_oid=2574180375 |
|  | *Mycobacterium tuberculosis* TKK_03_0034 | 2590828616 | https://img.jgi.doe.gov/cgi-bin/m/main.cgi?section=TaxonDetail&page=taxonDetail&taxon_oid=2590828616 |
|  | *Mycobacterium tuberculosis* PanR0317 | 2554235302 | https://img.jgi.doe.gov/cgi-bin/m/main.cgi?section=TaxonDetail&page=taxonDetail&taxon_oid=2554235302 |
|  | *Mycobacterium tuberculosis* TRUG0070 | 2574180005 | https://img.jgi.doe.gov/cgi-bin/m/main.cgi?section=TaxonDetail&page=taxonDetail&taxon_oid=2574180005 |
|  | *Mycobacterium tuberculosis* TKK_04_0018 | 2590828601 | https://img.jgi.doe.gov/cgi-bin/m/main.cgi?section=TaxonDetail&page=taxonDetail&taxon_oid=2590828601 |
|  | *Mycobacterium tuberculosis* TKK-01-0005 | 2588253980 | https://img.jgi.doe.gov/cgi-bin/m/main.cgi?section=TaxonDetail&page=taxonDetail&taxon_oid=2588253980 |
|  | *Mycobacterium tuberculosis* PanR0315 | 2554235246 | https://img.jgi.doe.gov/cgi-bin/m/main.cgi?section=TaxonDetail&page=taxonDetail&taxon_oid=2554235246 |
|  | *Mycobacterium tuberculosis* TBR24 | 2588253872 | https://img.jgi.doe.gov/cgi-bin/m/main.cgi?section=TaxonDetail&page=taxonDetail&taxon_oid=2588253872 |
|  | *Mycobacterium tuberculosis* T85 | 642979364 | https://img.jgi.doe.gov/cgi-bin/m/main.cgi?section=TaxonDetail&page=taxonDetail&taxon_oid=642979364 |
|  | *Mycobacterium bovis* BCG Korea 1168P | 2540341164 | https://img.jgi.doe.gov/cgi-bin/m/main.cgi?section=TaxonDetail&page=taxonDetail&taxon_oid=2540341164 |
|  | *Mycobacterium tuberculosis* BTB10-308 | 2574180291 | https://img.jgi.doe.gov/cgi-bin/m/main.cgi?section=TaxonDetail&page=taxonDetail&taxon_oid=2574180291 |
|  | *Mycobacterium tuberculosis* 210 | 647000279 | https://img.jgi.doe.gov/cgi-bin/m/main.cgi?section=TaxonDetail&page=taxonDetail&taxon_oid=647000279 |
|  | *Mycobacterium tuberculosis* TKK_04_0067 | 2582581219 | https://img.jgi.doe.gov/cgi-bin/m/main.cgi?section=TaxonDetail&page=taxonDetail&taxon_oid=2582581219 |
|  | *Mycobacterium tuberculosis* TKK_02_0063 | 2590828631 | https://img.jgi.doe.gov/cgi-bin/m/main.cgi?section=TaxonDetail&page=taxonDetail&taxon_oid=2590828631 |
|  | *Mycobacterium tuberculosis* PanR0313 | 2554235254 | https://img.jgi.doe.gov/cgi-bin/m/main.cgi?section=TaxonDetail&page=taxonDetail&taxon_oid=2554235254 |
|  | *Mycobacterium* sp. 141 | 2540341250 | https://img.jgi.doe.gov/cgi-bin/m/main.cgi?section=TaxonDetail&page=taxonDetail&taxon_oid=2540341250 |
|  | *Mycobacterium massiliense* 1S-152-0914 | 2526164580 | https://img.jgi.doe.gov/cgi-bin/m/main.cgi?section=TaxonDetail&page=taxonDetail&taxon_oid=2526164580 |
|  | *Mycobacterium tuberculosis* TKK_02_0069 | 2590828628 | https://img.jgi.doe.gov/cgi-bin/m/main.cgi?section=TaxonDetail&page=taxonDetail&taxon_oid=2590828628 |
|  | *Mycobacterium septicum* DSM 44393 | 2551306423 | https://img.jgi.doe.gov/cgi-bin/m/main.cgi?section=TaxonDetail&page=taxonDetail&taxon_oid=2551306423 |
|  | *Mycobacterium tuberculosis* M1848 | 2574179855 | https://img.jgi.doe.gov/cgi-bin/m/main.cgi?section=TaxonDetail&page=taxonDetail&taxon_oid=2574179855 |
|  | *Mycobacterium tuberculosis* TKK_04_0001 | 2590828610 | https://img.jgi.doe.gov/cgi-bin/m/main.cgi?section=TaxonDetail&page=taxonDetail&taxon_oid=2590828610 |
|  | *Mycobacterium tuberculosis* VRF CWCF XDRTB 1028 | 2597490246 | https://img.jgi.doe.gov/cgi-bin/m/main.cgi?section=TaxonDetail&page=taxonDetail&taxon_oid=2597490246 |
|  | *Mycobacterium tuberculosis* M2136 | 2574180209 | https://img.jgi.doe.gov/cgi-bin/m/main.cgi?section=TaxonDetail&page=taxonDetail&taxon_oid=2574180209 |
|  | *Mycobacterium tuberculosis* M1475 | 2582581167 | https://img.jgi.doe.gov/cgi-bin/m/main.cgi?section=TaxonDetail&page=taxonDetail&taxon_oid=2582581167 |
|  | *Mycobacterium tuberculosis* TRUG0098 | 2574179895 | https://img.jgi.doe.gov/cgi-bin/m/main.cgi?section=TaxonDetail&page=taxonDetail&taxon_oid=2574179895 |
|  | *Mycobacterium tuberculosis* PanR0703 | 2554235267 | https://img.jgi.doe.gov/cgi-bin/m/main.cgi?section=TaxonDetail&page=taxonDetail&taxon_oid=2554235267 |
|  | *Mycobacterium tuberculosis* Beijing/NITR203 | 2545824625 | https://img.jgi.doe.gov/cgi-bin/m/main.cgi?section=TaxonDetail&page=taxonDetail&taxon_oid=2545824625 |
|  | *Mycobacterium tuberculosis* MAL020205 | 2588254136 | https://img.jgi.doe.gov/cgi-bin/m/main.cgi?section=TaxonDetail&page=taxonDetail&taxon_oid=2588254136 |
|  | *Mycobacterium tuberculosis* TKK-01-0075 | 2574180070 | https://img.jgi.doe.gov/cgi-bin/m/main.cgi?section=TaxonDetail&page=taxonDetail&taxon_oid=2574180070 |
|  | *Mycobacterium tuberculosis* KT-0096 | 2576861377 | https://img.jgi.doe.gov/cgi-bin/m/main.cgi?section=TaxonDetail&page=taxonDetail&taxon_oid=2576861377 |
|  | *Mycobacterium tuberculosis* TKK-01-0036 | 2588253997 | https://img.jgi.doe.gov/cgi-bin/m/main.cgi?section=TaxonDetail&page=taxonDetail&taxon_oid=2588253997 |
|  | *Mycobacterium tuberculosis* TKK-01-0023 | 2588253846 | https://img.jgi.doe.gov/cgi-bin/m/main.cgi?section=TaxonDetail&page=taxonDetail&taxon_oid=2588253846 |
|  | *Mycobacterium tuberculosis* MAL020110 | 2588254108 | https://img.jgi.doe.gov/cgi-bin/m/main.cgi?section=TaxonDetail&page=taxonDetail&taxon_oid=2588254108 |
|  | *Mycobacterium tuberculosis* KZN 1435 (MDR) | 644736391 | https://img.jgi.doe.gov/cgi-bin/m/main.cgi?section=TaxonDetail&page=taxonDetail&taxon_oid=644736391 |
|  | *Mycobacterium tuberculosis* M2402 | 2574179880 | https://img.jgi.doe.gov/cgi-bin/m/main.cgi?section=TaxonDetail&page=taxonDetail&taxon_oid=2574179880 |
|  | *Mycobacterium tuberculosis* TBR30 | 2588253875 | https://img.jgi.doe.gov/cgi-bin/m/main.cgi?section=TaxonDetail&page=taxonDetail&taxon_oid=2588253875 |
|  | *Mycobacterium intracellulare* MOTT-02 | 2512564050 | https://img.jgi.doe.gov/cgi-bin/m/main.cgi?section=TaxonDetail&page=taxonDetail&taxon_oid=2512564050 |
|  | *Mycobacterium tuberculosis* BTB11-214 | 2574180413 | https://img.jgi.doe.gov/cgi-bin/m/main.cgi?section=TaxonDetail&page=taxonDetail&taxon_oid=2574180413 |
|  | *Mycobacterium tuberculosis* OFXR-13 | 2574180360 | https://img.jgi.doe.gov/cgi-bin/m/main.cgi?section=TaxonDetail&page=taxonDetail&taxon_oid=2574180360 |
|  | *Mycobacterium tuberculosis* 2483AR | 2574179972 | https://img.jgi.doe.gov/cgi-bin/m/main.cgi?section=TaxonDetail&page=taxonDetail&taxon_oid=2574179972 |
|  | *Mycobacterium tuberculosis* TKK_04_0064 | 2574180190 | https://img.jgi.doe.gov/cgi-bin/m/main.cgi?section=TaxonDetail&page=taxonDetail&taxon_oid=2574180190 |
|  | *Mycobacterium tuberculosis* TKK_04_0034 | 2590828679 | https://img.jgi.doe.gov/cgi-bin/m/main.cgi?section=TaxonDetail&page=taxonDetail&taxon_oid=2590828679 |
|  | *Mycobacterium tuberculosis* OFXR-1 | 2588254142 | https://img.jgi.doe.gov/cgi-bin/m/main.cgi?section=TaxonDetail&page=taxonDetail&taxon_oid=2588254142 |
|  | *Mycobacterium tuberculosis* W-148 | 2547132198 | https://img.jgi.doe.gov/cgi-bin/m/main.cgi?section=TaxonDetail&page=taxonDetail&taxon_oid=2547132198 |
|  | *Mycobacterium tuberculosis* WX3 | 2558860632 | https://img.jgi.doe.gov/cgi-bin/m/main.cgi?section=TaxonDetail&page=taxonDetail&taxon_oid=2558860632 |
|  | *Mycobacterium africanum* MAL010112 | 2574179981 | https://img.jgi.doe.gov/cgi-bin/m/main.cgi?section=TaxonDetail&page=taxonDetail&taxon_oid=2574179981 |
|  | *Mycobacterium tuberculosis* TKK-01-0087 | 2588254033 | https://img.jgi.doe.gov/cgi-bin/m/main.cgi?section=TaxonDetail&page=taxonDetail&taxon_oid=2588254033 |
|  | *Mycobacterium tuberculosis* MAL020142 | 2588254114 | https://img.jgi.doe.gov/cgi-bin/m/main.cgi?section=TaxonDetail&page=taxonDetail&taxon_oid=2588254114 |
|  | *Mycobacterium tuberculosis* TKK-01-0079 | 2576861103 | https://img.jgi.doe.gov/cgi-bin/m/main.cgi?section=TaxonDetail&page=taxonDetail&taxon_oid=2576861103 |
|  | *Mycobacterium africanum* MAL020185 | 2579778664 | https://img.jgi.doe.gov/cgi-bin/m/main.cgi?section=TaxonDetail&page=taxonDetail&taxon_oid=2579778664 |
|  | *Mycobacterium tuberculosis* TKK-01-0016 | 2588253988 | https://img.jgi.doe.gov/cgi-bin/m/main.cgi?section=TaxonDetail&page=taxonDetail&taxon_oid=2588253988 |
|  | *Mycobacterium tuberculosis* TRUG0040 | 2576861031 | https://img.jgi.doe.gov/cgi-bin/m/main.cgi?section=TaxonDetail&page=taxonDetail&taxon_oid=2576861031 |
|  | *Mycobacterium tuberculosis* TKK_02_0036 | 2590828637 | https://img.jgi.doe.gov/cgi-bin/m/main.cgi?section=TaxonDetail&page=taxonDetail&taxon_oid=2590828637 |
|  | *Mycobacterium tuberculosis* TKK_02_0020 | 2590828644 | https://img.jgi.doe.gov/cgi-bin/m/main.cgi?section=TaxonDetail&page=taxonDetail&taxon_oid=2590828644 |
|  | *Mycobacterium africanum* MAL010120 | 2579778639 | https://img.jgi.doe.gov/cgi-bin/m/main.cgi?section=TaxonDetail&page=taxonDetail&taxon_oid=2579778639 |
|  | *Mycobacterium tuberculosis* TBR48 | 2588253882 | https://img.jgi.doe.gov/cgi-bin/m/main.cgi?section=TaxonDetail&page=taxonDetail&taxon_oid=2588253882 |
|  | *Mycobacterium tuberculosis* M1700 | 2582581203 | https://img.jgi.doe.gov/cgi-bin/m/main.cgi?section=TaxonDetail&page=taxonDetail&taxon_oid=2582581203 |
|  | *Mycobacterium tuberculosis* MD15956 | 2582581210 | https://img.jgi.doe.gov/cgi-bin/m/main.cgi?section=TaxonDetail&page=taxonDetail&taxon_oid=2582581210 |
|  | *Mycobacterium tuberculosis* KT-0014 | 2588254159 | https://img.jgi.doe.gov/cgi-bin/m/main.cgi?section=TaxonDetail&page=taxonDetail&taxon_oid=2588254159 |
|  | *Mycobacterium tuberculosis* TKK_03_0109 | 2574180053 | https://img.jgi.doe.gov/cgi-bin/m/main.cgi?section=TaxonDetail&page=taxonDetail&taxon_oid=2574180053 |
|  | *Mycobacterium tuberculosis* BTB07-325 | 2576861349 | https://img.jgi.doe.gov/cgi-bin/m/main.cgi?section=TaxonDetail&page=taxonDetail&taxon_oid=2576861349 |
|  | *Mycobacterium tuberculosis* X122 | 2547132239 | https://img.jgi.doe.gov/cgi-bin/m/main.cgi?section=TaxonDetail&page=taxonDetail&taxon_oid=2547132239 |
|  | *Mycobacterium tuberculosis* 44503 | 2582581129 | https://img.jgi.doe.gov/cgi-bin/m/main.cgi?section=TaxonDetail&page=taxonDetail&taxon_oid=2582581129 |
|  | *Mycobacterium tuberculosis* TB_RSA166 | 2582581154 | https://img.jgi.doe.gov/cgi-bin/m/main.cgi?section=TaxonDetail&page=taxonDetail&taxon_oid=2582581154 |
|  | *Mycobacterium tuberculosis* TKK-01-0074 | 2588254019 | https://img.jgi.doe.gov/cgi-bin/m/main.cgi?section=TaxonDetail&page=taxonDetail&taxon_oid=2588254019 |
|  | *Mycobacterium tuberculosis* TBR49 | 2588253883 | https://img.jgi.doe.gov/cgi-bin/m/main.cgi?section=TaxonDetail&page=taxonDetail&taxon_oid=2588253883 |
|  | *Mycobacterium tuberculosis* TKK-01-0073 | 2588254021 | https://img.jgi.doe.gov/cgi-bin/m/main.cgi?section=TaxonDetail&page=taxonDetail&taxon_oid=2588254021 |
|  | *Mycobacterium tuberculosis* TKK_02_0018 | 2590828645 | https://img.jgi.doe.gov/cgi-bin/m/main.cgi?section=TaxonDetail&page=taxonDetail&taxon_oid=2590828645 |
|  | *Mycobacterium tuberculosis* BTB05-285 | 2576861086 | https://img.jgi.doe.gov/cgi-bin/m/main.cgi?section=TaxonDetail&page=taxonDetail&taxon_oid=2576861086 |
|  | *Mycobacterium tuberculosis* XTB13-094 | 2574180212 | https://img.jgi.doe.gov/cgi-bin/m/main.cgi?section=TaxonDetail&page=taxonDetail&taxon_oid=2574180212 |
|  | *Mycobacterium tuberculosis* H37RvCO | 2547132039 | https://img.jgi.doe.gov/cgi-bin/m/main.cgi?section=TaxonDetail&page=taxonDetail&taxon_oid=2547132039 |
|  | *Mycobacterium tuberculosis* TB_RSA82 | 2574180374 | https://img.jgi.doe.gov/cgi-bin/m/main.cgi?section=TaxonDetail&page=taxonDetail&taxon_oid=2574180374 |
|  | *Mycobacterium tuberculosis* KT-0108 | 2574180043 | https://img.jgi.doe.gov/cgi-bin/m/main.cgi?section=TaxonDetail&page=taxonDetail&taxon_oid=2574180043 |
|  | *Mycobacterium tuberculosis* TB_RSA09 | 2576861006 | https://img.jgi.doe.gov/cgi-bin/m/main.cgi?section=TaxonDetail&page=taxonDetail&taxon_oid=2576861006 |
|  | *Mycobacterium tuberculosis* XTB13-162 | 2576861284 | https://img.jgi.doe.gov/cgi-bin/m/main.cgi?section=TaxonDetail&page=taxonDetail&taxon_oid=2576861284 |
|  | *Mycobacterium tuberculosis* PanR0607 | 2554235269 | https://img.jgi.doe.gov/cgi-bin/m/main.cgi?section=TaxonDetail&page=taxonDetail&taxon_oid=2554235269 |
|  | *Mycobacterium tuberculosis* OFXR-33 | 2576861140 | https://img.jgi.doe.gov/cgi-bin/m/main.cgi?section=TaxonDetail&page=taxonDetail&taxon_oid=2576861140 |
|  | *Mycobacterium tuberculosis* TKK_04_0014 | 2590828604 | https://img.jgi.doe.gov/cgi-bin/m/main.cgi?section=TaxonDetail&page=taxonDetail&taxon_oid=2590828604 |
|  | *Mycobacterium tuberculosis* KT-0041 | 2582581146 | https://img.jgi.doe.gov/cgi-bin/m/main.cgi?section=TaxonDetail&page=taxonDetail&taxon_oid=2582581146 |
|  | *Mycobacterium tuberculosis* TB_RSA64 | 2579778506 | https://img.jgi.doe.gov/cgi-bin/m/main.cgi?section=TaxonDetail&page=taxonDetail&taxon_oid=2579778506 |
|  | *Mycobacterium tuberculosis* M2508 | 2576861098 | https://img.jgi.doe.gov/cgi-bin/m/main.cgi?section=TaxonDetail&page=taxonDetail&taxon_oid=2576861098 |
|  | *Mycobacterium tuberculosis* T67 | 2588253770 | https://img.jgi.doe.gov/cgi-bin/m/main.cgi?section=TaxonDetail&page=taxonDetail&taxon_oid=2588253770 |
|  | *Mycobacterium tuberculosis* MD16277 | 2574180259 | https://img.jgi.doe.gov/cgi-bin/m/main.cgi?section=TaxonDetail&page=taxonDetail&taxon_oid=2574180259 |
|  | *Mycobacterium tuberculosis* XTB13-086 | 2582581164 | https://img.jgi.doe.gov/cgi-bin/m/main.cgi?section=TaxonDetail&page=taxonDetail&taxon_oid=2582581164 |
|  | *Mycobacterium tuberculosis* KT-0001 | 2576861345 | https://img.jgi.doe.gov/cgi-bin/m/main.cgi?section=TaxonDetail&page=taxonDetail&taxon_oid=2576861345 |
|  | *Mycobacterium* sp. URHD0025 | 2522572101 | https://img.jgi.doe.gov/cgi-bin/m/main.cgi?section=TaxonDetail&page=taxonDetail&taxon_oid=2522572101 |
|  | *Mycobacterium tuberculosis* TKK_03_0082 | 2590828611 | https://img.jgi.doe.gov/cgi-bin/m/main.cgi?section=TaxonDetail&page=taxonDetail&taxon_oid=2590828611 |
|  | *Mycobacterium tuberculosis* M1961 | 2582581148 | https://img.jgi.doe.gov/cgi-bin/m/main.cgi?section=TaxonDetail&page=taxonDetail&taxon_oid=2582581148 |
|  | *Mycobacterium africanum* MAL010129 | 2579779005 | https://img.jgi.doe.gov/cgi-bin/m/main.cgi?section=TaxonDetail&page=taxonDetail&taxon_oid=2579779005 |
|  | *Mycobacterium tuberculosis* MAL010080 | 2588254095 | https://img.jgi.doe.gov/cgi-bin/m/main.cgi?section=TaxonDetail&page=taxonDetail&taxon_oid=2588254095 |
|  | *Mycobacterium tuberculosis* PanR0708 | 2554235260 | https://img.jgi.doe.gov/cgi-bin/m/main.cgi?section=TaxonDetail&page=taxonDetail&taxon_oid=2554235260 |
|  | *Mycobacterium tuberculosis* TB_RSA174 | 2574179966 | https://img.jgi.doe.gov/cgi-bin/m/main.cgi?section=TaxonDetail&page=taxonDetail&taxon_oid=2574179966 |
|  | *Mycobacterium tuberculosis* PanR0501 | 2554235251 | https://img.jgi.doe.gov/cgi-bin/m/main.cgi?section=TaxonDetail&page=taxonDetail&taxon_oid=2554235251 |
|  | *Mycobacterium tuberculosis* BTB11-343 | 2574180113 | https://img.jgi.doe.gov/cgi-bin/m/main.cgi?section=TaxonDetail&page=taxonDetail&taxon_oid=2574180113 |
|  | *Mycobacterium tuberculosis* TB_RSA127 | 2576861191 | https://img.jgi.doe.gov/cgi-bin/m/main.cgi?section=TaxonDetail&page=taxonDetail&taxon_oid=2576861191 |
|  | *Mycobacterium tuberculosis* MAL020181 | 2574179832 | https://img.jgi.doe.gov/cgi-bin/m/main.cgi?section=TaxonDetail&page=taxonDetail&taxon_oid=2574179832 |
|  | *Mycobacterium tuberculosis* TKK-01-0011 | 2574180079 | https://img.jgi.doe.gov/cgi-bin/m/main.cgi?section=TaxonDetail&page=taxonDetail&taxon_oid=2574180079 |
|  | *Mycobacterium canettii* CIPT 140070017 | 2541047046 | https://img.jgi.doe.gov/cgi-bin/m/main.cgi?section=TaxonDetail&page=taxonDetail&taxon_oid=2541047046 |
|  | *Mycobacterium tuberculosis* Haarlem | 2588254170 | https://img.jgi.doe.gov/cgi-bin/m/main.cgi?section=TaxonDetail&page=taxonDetail&taxon_oid=2588254170 |
|  | *Mycobacterium tuberculosis* OFXR-32 | 2588254177 | https://img.jgi.doe.gov/cgi-bin/m/main.cgi?section=TaxonDetail&page=taxonDetail&taxon_oid=2588254177 |
|  | *Mycobacterium tuberculosis* KT-0064 | 2588254242 | https://img.jgi.doe.gov/cgi-bin/m/main.cgi?section=TaxonDetail&page=taxonDetail&taxon_oid=2588254242 |
|  | *Mycobacterium tuberculosis* TBR60 | 2588253889 | https://img.jgi.doe.gov/cgi-bin/m/main.cgi?section=TaxonDetail&page=taxonDetail&taxon_oid=2588253889 |
|  | *Mycobacterium tuberculosis* TKK_04_0140 | 2574180349 | https://img.jgi.doe.gov/cgi-bin/m/main.cgi?section=TaxonDetail&page=taxonDetail&taxon_oid=2574180349 |
|  | *Mycobacterium tuberculosis* TBR74 | 2588253890 | https://img.jgi.doe.gov/cgi-bin/m/main.cgi?section=TaxonDetail&page=taxonDetail&taxon_oid=2588253890 |
|  | *Mycobacterium tuberculosis* TB_RSA77 | 2576861091 | https://img.jgi.doe.gov/cgi-bin/m/main.cgi?section=TaxonDetail&page=taxonDetail&taxon_oid=2576861091 |
|  | *Mycobacterium tuberculosis* KT-0098 | 2582581194 | https://img.jgi.doe.gov/cgi-bin/m/main.cgi?section=TaxonDetail&page=taxonDetail&taxon_oid=2582581194 |
|  | *Mycobacterium tuberculosis* MD16265 | 2576861154 | https://img.jgi.doe.gov/cgi-bin/m/main.cgi?section=TaxonDetail&page=taxonDetail&taxon_oid=2576861154 |
|  | *Mycobacterium tuberculosis* TKK_03_0042 | 2574180172 | https://img.jgi.doe.gov/cgi-bin/m/main.cgi?section=TaxonDetail&page=taxonDetail&taxon_oid=2574180172 |
|  | *Mycobacterium tuberculosis* H2581 | 2574180320 | https://img.jgi.doe.gov/cgi-bin/m/main.cgi?section=TaxonDetail&page=taxonDetail&taxon_oid=2574180320 |
|  | *Mycobacterium tuberculosis* OFXR-3 | 2574180342 | https://img.jgi.doe.gov/cgi-bin/m/main.cgi?section=TaxonDetail&page=taxonDetail&taxon_oid=2574180342 |
|  | *Mycobacterium tuberculosis* MAL010117 | 2588254102 | https://img.jgi.doe.gov/cgi-bin/m/main.cgi?section=TaxonDetail&page=taxonDetail&taxon_oid=2588254102 |
|  | *Mycobacterium tuberculosis* OSDD518 | 2548876691 | https://img.jgi.doe.gov/cgi-bin/m/main.cgi?section=TaxonDetail&page=taxonDetail&taxon_oid=2548876691 |
|  | *Mycobacterium bovis* B2 7505 | 2579778623 | https://img.jgi.doe.gov/cgi-bin/m/main.cgi?section=TaxonDetail&page=taxonDetail&taxon_oid=2579778623 |
|  | *Mycobacterium tuberculosis* BTB07-254 | 2582581222 | https://img.jgi.doe.gov/cgi-bin/m/main.cgi?section=TaxonDetail&page=taxonDetail&taxon_oid=2582581222 |
|  | *Mycobacterium tuberculosis* BTB13-206 | 2582581162 | https://img.jgi.doe.gov/cgi-bin/m/main.cgi?section=TaxonDetail&page=taxonDetail&taxon_oid=2582581162 |
|  | *Mycobacterium tuberculosis* TKK-01-0047 | 2576861124 | https://img.jgi.doe.gov/cgi-bin/m/main.cgi?section=TaxonDetail&page=taxonDetail&taxon_oid=2576861124 |
|  | *Mycobacterium intracellulare* 1956 | 2565956792 | https://img.jgi.doe.gov/cgi-bin/m/main.cgi?section=TaxonDetail&page=taxonDetail&taxon_oid=2565956792 |
|  | *Mycobacterium tuberculosis* TB_RSA173 | 2574179905 | https://img.jgi.doe.gov/cgi-bin/m/main.cgi?section=TaxonDetail&page=taxonDetail&taxon_oid=2574179905 |
|  | *Mycobacterium tuberculosis* 49375 | 2576861156 | https://img.jgi.doe.gov/cgi-bin/m/main.cgi?section=TaxonDetail&page=taxonDetail&taxon_oid=2576861156 |
|  | *Mycobacterium tuberculosis* TB_RSA178 | 2576861046 | https://img.jgi.doe.gov/cgi-bin/m/main.cgi?section=TaxonDetail&page=taxonDetail&taxon_oid=2576861046 |
|  | *Mycobacterium tuberculosis* TB_RSA199 | 2574180228 | https://img.jgi.doe.gov/cgi-bin/m/main.cgi?section=TaxonDetail&page=taxonDetail&taxon_oid=2574180228 |
|  | *Mycobacterium tuberculosis* MAL020195 | 2588254125 | https://img.jgi.doe.gov/cgi-bin/m/main.cgi?section=TaxonDetail&page=taxonDetail&taxon_oid=2588254125 |
|  | *Mycobacterium tuberculosis* TKK-01-0004 | 2588253981 | https://img.jgi.doe.gov/cgi-bin/m/main.cgi?section=TaxonDetail&page=taxonDetail&taxon_oid=2588253981 |
|  | *Mycobacterium tuberculosis* MD13878 | 2574179939 | https://img.jgi.doe.gov/cgi-bin/m/main.cgi?section=TaxonDetail&page=taxonDetail&taxon_oid=2574179939 |
|  | *Mycobacterium tuberculosis* TBR50 | 2574180254 | https://img.jgi.doe.gov/cgi-bin/m/main.cgi?section=TaxonDetail&page=taxonDetail&taxon_oid=2574180254 |
|  | *Mycobacterium tuberculosis* 02_1987 | 2576861165 | https://img.jgi.doe.gov/cgi-bin/m/main.cgi?section=TaxonDetail&page=taxonDetail&taxon_oid=2576861165 |
|  | *Mycobacterium tuberculosis* NA-A0009 | 2551306109 | https://img.jgi.doe.gov/cgi-bin/m/main.cgi?section=TaxonDetail&page=taxonDetail&taxon_oid=2551306109 |
|  | *Mycobacterium tuberculosis* MAL020141 | 2588254113 | https://img.jgi.doe.gov/cgi-bin/m/main.cgi?section=TaxonDetail&page=taxonDetail&taxon_oid=2588254113 |
|  | *Mycobacterium tuberculosis* TKK-01-0032 | 2582581181 | https://img.jgi.doe.gov/cgi-bin/m/main.cgi?section=TaxonDetail&page=taxonDetail&taxon_oid=2582581181 |
|  | *Mycobacterium tuberculosis* KT-0037 | 2574180357 | https://img.jgi.doe.gov/cgi-bin/m/main.cgi?section=TaxonDetail&page=taxonDetail&taxon_oid=2574180357 |
|  | *Mycobacterium tuberculosis* 1173CS | 2574180368 | https://img.jgi.doe.gov/cgi-bin/m/main.cgi?section=TaxonDetail&page=taxonDetail&taxon_oid=2574180368 |
|  | *Mycobacterium intracellulare* M.i.198 | 2547132171 | https://img.jgi.doe.gov/cgi-bin/m/main.cgi?section=TaxonDetail&page=taxonDetail&taxon_oid=2547132171 |
|  | *Mycobacterium tuberculosis* PanR0505 | 2554235256 | https://img.jgi.doe.gov/cgi-bin/m/main.cgi?section=TaxonDetail&page=taxonDetail&taxon_oid=2554235256 |
|  | *Mycobacterium tuberculosis* SUMu010 | 648276702 | https://img.jgi.doe.gov/cgi-bin/m/main.cgi?section=TaxonDetail&page=taxonDetail&taxon_oid=648276702 |
|  | *Mycobacterium chelonae* 1518 | 2565956796 | https://img.jgi.doe.gov/cgi-bin/m/main.cgi?section=TaxonDetail&page=taxonDetail&taxon_oid=2565956796 |
|  | *Mycobacterium tuberculosis* TKK_05SA_0019 | 2576861044 | https://img.jgi.doe.gov/cgi-bin/m/main.cgi?section=TaxonDetail&page=taxonDetail&taxon_oid=2576861044 |
|  | *Mycobacterium tuberculosis* TBR76 | 2588253894 | https://img.jgi.doe.gov/cgi-bin/m/main.cgi?section=TaxonDetail&page=taxonDetail&taxon_oid=2588253894 |
|  | *Mycobacterium tuberculosis* TKK_03_0099 | 2574180159 | https://img.jgi.doe.gov/cgi-bin/m/main.cgi?section=TaxonDetail&page=taxonDetail&taxon_oid=2574180159 |
|  | *Mycobacterium tuberculosis* TKK_04_0006 | 2590828608 | https://img.jgi.doe.gov/cgi-bin/m/main.cgi?section=TaxonDetail&page=taxonDetail&taxon_oid=2590828608 |
|  | *Mycobacterium canettii* CIPT 140070007 | 2565956756 | https://img.jgi.doe.gov/cgi-bin/m/main.cgi?section=TaxonDetail&page=taxonDetail&taxon_oid=2565956756 |
|  | *Mycobacterium tuberculosis* PanR0601 | 2554235248 | https://img.jgi.doe.gov/cgi-bin/m/main.cgi?section=TaxonDetail&page=taxonDetail&taxon_oid=2554235248 |
|  | *Mycobacterium tuberculosis* PanR0304 | 2554235238 | https://img.jgi.doe.gov/cgi-bin/m/main.cgi?section=TaxonDetail&page=taxonDetail&taxon_oid=2554235238 |
|  | *Mycobacterium tuberculosis* MAL020145 | 2582581166 | https://img.jgi.doe.gov/cgi-bin/m/main.cgi?section=TaxonDetail&page=taxonDetail&taxon_oid=2582581166 |
|  | *Mycobacterium tuberculosis* M2006 | 2576861118 | https://img.jgi.doe.gov/cgi-bin/m/main.cgi?section=TaxonDetail&page=taxonDetail&taxon_oid=2576861118 |
|  | *Mycobacterium tuberculosis* MD15855 | 2576861161 | https://img.jgi.doe.gov/cgi-bin/m/main.cgi?section=TaxonDetail&page=taxonDetail&taxon_oid=2576861161 |
|  | *Mycobacterium tuberculosis* TKK-01-0080 | 2588253849 | https://img.jgi.doe.gov/cgi-bin/m/main.cgi?section=TaxonDetail&page=taxonDetail&taxon_oid=2588253849 |
|  | *Mycobacterium tuberculosis* TKK_03_0156 | 2574179954 | https://img.jgi.doe.gov/cgi-bin/m/main.cgi?section=TaxonDetail&page=taxonDetail&taxon_oid=2574179954 |
|  | *Mycobacterium tuberculosis* TB_RSA62 | 2582581200 | https://img.jgi.doe.gov/cgi-bin/m/main.cgi?section=TaxonDetail&page=taxonDetail&taxon_oid=2582581200 |
|  | *Mycobacterium tuberculosis* OFXR-14 | 2588253896 | https://img.jgi.doe.gov/cgi-bin/m/main.cgi?section=TaxonDetail&page=taxonDetail&taxon_oid=2588253896 |
|  | *Mycobacterium tuberculosis* TKK_05SA_0016 | 2574179918 | https://img.jgi.doe.gov/cgi-bin/m/main.cgi?section=TaxonDetail&page=taxonDetail&taxon_oid=2574179918 |
|  | *Mycobacterium bovis* BCG Moreau RDJ | 2619619103 | https://img.jgi.doe.gov/cgi-bin/m/main.cgi?section=TaxonDetail&page=taxonDetail&taxon_oid=2619619103 |
|  | *Mycobacterium tuberculosis* M1221 | 2574180073 | https://img.jgi.doe.gov/cgi-bin/m/main.cgi?section=TaxonDetail&page=taxonDetail&taxon_oid=2574180073 |
|  | *Mycobacterium tuberculosis* CPHL_A | 645951826 | https://img.jgi.doe.gov/cgi-bin/m/main.cgi?section=TaxonDetail&page=taxonDetail&taxon_oid=645951826 |
|  | *Mycobacterium tuberculosis* T92 | 2582581196 | https://img.jgi.doe.gov/cgi-bin/m/main.cgi?section=TaxonDetail&page=taxonDetail&taxon_oid=2582581196 |
|  | *Mycobacterium tuberculosis* XTB13-252 | 2582581150 | https://img.jgi.doe.gov/cgi-bin/m/main.cgi?section=TaxonDetail&page=taxonDetail&taxon_oid=2582581150 |
|  | *Mycobacterium tuberculosis* TKK_04_0017 | 2590828602 | https://img.jgi.doe.gov/cgi-bin/m/main.cgi?section=TaxonDetail&page=taxonDetail&taxon_oid=2590828602 |
|  | *Mycobacterium tuberculosis* MD15597 | 2582581212 | https://img.jgi.doe.gov/cgi-bin/m/main.cgi?section=TaxonDetail&page=taxonDetail&taxon_oid=2582581212 |
|  | *Mycobacterium tuberculosis* KT-0006 | 2588254162 | https://img.jgi.doe.gov/cgi-bin/m/main.cgi?section=TaxonDetail&page=taxonDetail&taxon_oid=2588254162 |
|  | *Mycobacterium hassiacum* DSM 44199 | 2531839488 | https://img.jgi.doe.gov/cgi-bin/m/main.cgi?section=TaxonDetail&page=taxonDetail&taxon_oid=2531839488 |
|  | *Mycobacterium tuberculosis* KT-0094 | 2588254230 | https://img.jgi.doe.gov/cgi-bin/m/main.cgi?section=TaxonDetail&page=taxonDetail&taxon_oid=2588254230 |
|  | *Mycobacterium tuberculosis* TKK_03_0116 | 2576861018 | https://img.jgi.doe.gov/cgi-bin/m/main.cgi?section=TaxonDetail&page=taxonDetail&taxon_oid=2576861018 |
|  | *Mycobacterium tuberculosis* TKK_05SA_0050 | 2576861143 | https://img.jgi.doe.gov/cgi-bin/m/main.cgi?section=TaxonDetail&page=taxonDetail&taxon_oid=2576861143 |
|  | *Mycobacterium tuberculosis* BTB07-246 | 2582581145 | https://img.jgi.doe.gov/cgi-bin/m/main.cgi?section=TaxonDetail&page=taxonDetail&taxon_oid=2582581145 |
|  | *Mycobacterium tuberculosis* TKK_02_0027 | 2590828640 | https://img.jgi.doe.gov/cgi-bin/m/main.cgi?section=TaxonDetail&page=taxonDetail&taxon_oid=2590828640 |
|  | *Mycobacterium tuberculosis* FJ05194 | 2541047993 | https://img.jgi.doe.gov/cgi-bin/m/main.cgi?section=TaxonDetail&page=taxonDetail&taxon_oid=2541047993 |
|  | *Mycobacterium tuberculosis* TKK_02_0025 | 2590828641 | https://img.jgi.doe.gov/cgi-bin/m/main.cgi?section=TaxonDetail&page=taxonDetail&taxon_oid=2590828641 |
|  | *Mycobacterium tuberculosis* TKK_04_0043 | 2590828675 | https://img.jgi.doe.gov/cgi-bin/m/main.cgi?section=TaxonDetail&page=taxonDetail&taxon_oid=2590828675 |
|  | *Mycobacterium tuberculosis* SUMu006 | 648276698 | https://img.jgi.doe.gov/cgi-bin/m/main.cgi?section=TaxonDetail&page=taxonDetail&taxon_oid=648276698 |
|  | *Mycobacterium tuberculosis* MAL010109 | 2574180338 | https://img.jgi.doe.gov/cgi-bin/m/main.cgi?section=TaxonDetail&page=taxonDetail&taxon_oid=2574180338 |
|  | *Mycobacterium tuberculosis* GM 1503 | 642979350 | https://img.jgi.doe.gov/cgi-bin/m/main.cgi?section=TaxonDetail&page=taxonDetail&taxon_oid=642979350 |
|  | *Mycobacterium tuberculosis* TBR9 | 2588253871 | https://img.jgi.doe.gov/cgi-bin/m/main.cgi?section=TaxonDetail&page=taxonDetail&taxon_oid=2588253871 |
|  | *Mycobacterium tuberculosis* TKK_05MA_0025 | 2574180121 | https://img.jgi.doe.gov/cgi-bin/m/main.cgi?section=TaxonDetail&page=taxonDetail&taxon_oid=2574180121 |
|  | *Mycobacterium tuberculosis* MD17615 | 2582581178 | https://img.jgi.doe.gov/cgi-bin/m/main.cgi?section=TaxonDetail&page=taxonDetail&taxon_oid=2582581178 |
|  | *Mycobacterium tuberculosis* H37Ra | 641736240 | https://img.jgi.doe.gov/cgi-bin/m/main.cgi?section=TaxonDetail&page=taxonDetail&taxon_oid=641736240 |
|  | *Mycobacterium tuberculosis* TKK-01-0030 | 2588253993 | https://img.jgi.doe.gov/cgi-bin/m/main.cgi?section=TaxonDetail&page=taxonDetail&taxon_oid=2588253993 |
|  | *Mycobacterium tuberculosis* PanR1005 | 2554235309 | https://img.jgi.doe.gov/cgi-bin/m/main.cgi?section=TaxonDetail&page=taxonDetail&taxon_oid=2554235309 |
|  | *Mycobacterium tuberculosis* XTB13-127 | 2574180027 | https://img.jgi.doe.gov/cgi-bin/m/main.cgi?section=TaxonDetail&page=taxonDetail&taxon_oid=2574180027 |
|  | *Mycobacterium tuberculosis* KT-0069 | 2574179897 | https://img.jgi.doe.gov/cgi-bin/m/main.cgi?section=TaxonDetail&page=taxonDetail&taxon_oid=2574179897 |
|  | *Mycobacterium tuberculosis* KT-0047 | 2588254147 | https://img.jgi.doe.gov/cgi-bin/m/main.cgi?section=TaxonDetail&page=taxonDetail&taxon_oid=2588254147 |
|  | *Mycobacterium tuberculosis* TKK-01-0033 | 2588253996 | https://img.jgi.doe.gov/cgi-bin/m/main.cgi?section=TaxonDetail&page=taxonDetail&taxon_oid=2588253996 |
|  | *Mycobacterium tuberculosis* TKK_02_0012 | 2590828649 | https://img.jgi.doe.gov/cgi-bin/m/main.cgi?section=TaxonDetail&page=taxonDetail&taxon_oid=2590828649 |
|  | *Mycobacterium tuberculosis* KT-0053 | 2588254145 | https://img.jgi.doe.gov/cgi-bin/m/main.cgi?section=TaxonDetail&page=taxonDetail&taxon_oid=2588254145 |
|  | *Mycobacterium tuberculosis* TRUG0095 | 2582581213 | https://img.jgi.doe.gov/cgi-bin/m/main.cgi?section=TaxonDetail&page=taxonDetail&taxon_oid=2582581213 |
|  | *Mycobacterium tuberculosis* PanR0704 | 2554235295 | https://img.jgi.doe.gov/cgi-bin/m/main.cgi?section=TaxonDetail&page=taxonDetail&taxon_oid=2554235295 |
|  | *Mycobacterium tuberculosis* M2113 | 2576861053 | https://img.jgi.doe.gov/cgi-bin/m/main.cgi?section=TaxonDetail&page=taxonDetail&taxon_oid=2576861053 |
|  | *Mycobacterium tuberculosis* XTB13-167 | 2576861145 | https://img.jgi.doe.gov/cgi-bin/m/main.cgi?section=TaxonDetail&page=taxonDetail&taxon_oid=2576861145 |
|  | *Mycobacterium tuberculosis* OFXR-10 | 2576861389 | https://img.jgi.doe.gov/cgi-bin/m/main.cgi?section=TaxonDetail&page=taxonDetail&taxon_oid=2576861389 |
|  | *Mycobacterium intracellulare* ATCC 35771 | 651716583 | https://img.jgi.doe.gov/cgi-bin/m/main.cgi?section=TaxonDetail&page=taxonDetail&taxon_oid=651716583 |
|  | *Mycobacterium tuberculosis* TKK_03_0024 | 2576861081 | https://img.jgi.doe.gov/cgi-bin/m/main.cgi?section=TaxonDetail&page=taxonDetail&taxon_oid=2576861081 |
|  | *Mycobacterium tuberculosis* TKK_03_0043 | 2590828614 | https://img.jgi.doe.gov/cgi-bin/m/main.cgi?section=TaxonDetail&page=taxonDetail&taxon_oid=2590828614 |
|  | *Mycobacterium tuberculosis* TKK_02_0038 | 2576861219 | https://img.jgi.doe.gov/cgi-bin/m/main.cgi?section=TaxonDetail&page=taxonDetail&taxon_oid=2576861219 |
|  | *Mycobacterium tuberculosis* M2343 | 2574180021 | https://img.jgi.doe.gov/cgi-bin/m/main.cgi?section=TaxonDetail&page=taxonDetail&taxon_oid=2574180021 |
|  | *Mycobacterium tuberculosis* KT-0003 | 2574180265 | https://img.jgi.doe.gov/cgi-bin/m/main.cgi?section=TaxonDetail&page=taxonDetail&taxon_oid=2574180265 |
|  | *Mycobacterium tuberculosis* KT-0091 | 2574180058 | https://img.jgi.doe.gov/cgi-bin/m/main.cgi?section=TaxonDetail&page=taxonDetail&taxon_oid=2574180058 |
|  | *Mycobacterium tuberculosis* TB_RSA96 | 2574179846 | https://img.jgi.doe.gov/cgi-bin/m/main.cgi?section=TaxonDetail&page=taxonDetail&taxon_oid=2574179846 |
|  | *Mycobacterium tuberculosis* TKK-01-0084 | 2588254032 | https://img.jgi.doe.gov/cgi-bin/m/main.cgi?section=TaxonDetail&page=taxonDetail&taxon_oid=2588254032 |
|  | *Mycobacterium tuberculosis* TB_RSA123 | 2576861323 | https://img.jgi.doe.gov/cgi-bin/m/main.cgi?section=TaxonDetail&page=taxonDetail&taxon_oid=2576861323 |
|  | *Mycobacterium tuberculosis* TKK_03_0036 | 2590828615 | https://img.jgi.doe.gov/cgi-bin/m/main.cgi?section=TaxonDetail&page=taxonDetail&taxon_oid=2590828615 |
|  | *Mycobacterium tuberculosis* MD16553 | 2574179804 | https://img.jgi.doe.gov/cgi-bin/m/main.cgi?section=TaxonDetail&page=taxonDetail&taxon_oid=2574179804 |
|  | *Mycobacterium tuberculosis* BTB13-128 | 2574179888 | https://img.jgi.doe.gov/cgi-bin/m/main.cgi?section=TaxonDetail&page=taxonDetail&taxon_oid=2574179888 |
|  | *Mycobacterium tuberculosis* TKK-01-0035 | 2579778513 | https://img.jgi.doe.gov/cgi-bin/m/main.cgi?section=TaxonDetail&page=taxonDetail&taxon_oid=2579778513 |
|  | *Mycobacterium tuberculosis* MAL020172 | 2588254122 | https://img.jgi.doe.gov/cgi-bin/m/main.cgi?section=TaxonDetail&page=taxonDetail&taxon_oid=2588254122 |
|  | *Mycobacterium tuberculosis* M2128 | 2576861209 | https://img.jgi.doe.gov/cgi-bin/m/main.cgi?section=TaxonDetail&page=taxonDetail&taxon_oid=2576861209 |
|  | *Mycobacterium tuberculosis* TB_RSA15 | 2574180150 | https://img.jgi.doe.gov/cgi-bin/m/main.cgi?section=TaxonDetail&page=taxonDetail&taxon_oid=2574180150 |
|  | *Mycobacterium tuberculosis* TKK-01-0072 | 2588253850 | https://img.jgi.doe.gov/cgi-bin/m/main.cgi?section=TaxonDetail&page=taxonDetail&taxon_oid=2588253850 |
|  | *Mycobacterium tuberculosis* TB_RSA79 | 2582581205 | https://img.jgi.doe.gov/cgi-bin/m/main.cgi?section=TaxonDetail&page=taxonDetail&taxon_oid=2582581205 |
|  | *Mycobacterium tuberculosis* NCGM2209 | 2548876688 | https://img.jgi.doe.gov/cgi-bin/m/main.cgi?section=TaxonDetail&page=taxonDetail&taxon_oid=2548876688 |
|  | *Mycobacterium africanum* MAL010074 | 2579779131 | https://img.jgi.doe.gov/cgi-bin/m/main.cgi?section=TaxonDetail&page=taxonDetail&taxon_oid=2579779131 |
|  | *Mycobacterium tuberculosis* XTB13-241 | 2574180363 | https://img.jgi.doe.gov/cgi-bin/m/main.cgi?section=TaxonDetail&page=taxonDetail&taxon_oid=2574180363 |
|  | *Mycobacterium tuberculosis* PanR1007 | 2554235275 | https://img.jgi.doe.gov/cgi-bin/m/main.cgi?section=TaxonDetail&page=taxonDetail&taxon_oid=2554235275 |
|  | *Mycobacterium tuberculosis* H2398 | 2576861228 | https://img.jgi.doe.gov/cgi-bin/m/main.cgi?section=TaxonDetail&page=taxonDetail&taxon_oid=2576861228 |
|  | *Mycobacterium tuberculosis* Erdman | 2588254171 | https://img.jgi.doe.gov/cgi-bin/m/main.cgi?section=TaxonDetail&page=taxonDetail&taxon_oid=2588254171 |
|  | *Mycobacterium tuberculosis* M1438 | 2574179826 | https://img.jgi.doe.gov/cgi-bin/m/main.cgi?section=TaxonDetail&page=taxonDetail&taxon_oid=2574179826 |
|  | *Mycobacterium tuberculosis* TKK-01-0010 | 2574180066 | https://img.jgi.doe.gov/cgi-bin/m/main.cgi?section=TaxonDetail&page=taxonDetail&taxon_oid=2574180066 |
|  | *Mycobacterium tuberculosis* UG-D | 2582581174 | https://img.jgi.doe.gov/cgi-bin/m/main.cgi?section=TaxonDetail&page=taxonDetail&taxon_oid=2582581174 |
|  | *Mycobacterium tuberculosis* MAL010103 | 2574180253 | https://img.jgi.doe.gov/cgi-bin/m/main.cgi?section=TaxonDetail&page=taxonDetail&taxon_oid=2574180253 |
|  | *Mycobacterium tuberculosis* PanR0707 | 2554235264 | https://img.jgi.doe.gov/cgi-bin/m/main.cgi?section=TaxonDetail&page=taxonDetail&taxon_oid=2554235264 |
|  | *Mycobacterium tuberculosis* KT-0087 | 2574179874 | https://img.jgi.doe.gov/cgi-bin/m/main.cgi?section=TaxonDetail&page=taxonDetail&taxon_oid=2574179874 |
|  | *Mycobacterium tuberculosis* OFXR-12 | 2588253899 | https://img.jgi.doe.gov/cgi-bin/m/main.cgi?section=TaxonDetail&page=taxonDetail&taxon_oid=2588253899 |
|  | *Mycobacterium tuberculosis* BTB10-357 | 2582581133 | https://img.jgi.doe.gov/cgi-bin/m/main.cgi?section=TaxonDetail&page=taxonDetail&taxon_oid=2582581133 |
|  | *Mycobacterium tuberculosis* TB_RSA161 | 2576861195 | https://img.jgi.doe.gov/cgi-bin/m/main.cgi?section=TaxonDetail&page=taxonDetail&taxon_oid=2576861195 |
|  | *Mycobacterium tuberculosis* PanR0603 | 2554235247 | https://img.jgi.doe.gov/cgi-bin/m/main.cgi?section=TaxonDetail&page=taxonDetail&taxon_oid=2554235247 |
|  | *Mycobacterium tuberculosis* MAL020138 | 2588254105 | https://img.jgi.doe.gov/cgi-bin/m/main.cgi?section=TaxonDetail&page=taxonDetail&taxon_oid=2588254105 |
|  | *Mycobacterium tuberculosis* MD15977 | 2574179798 | https://img.jgi.doe.gov/cgi-bin/m/main.cgi?section=TaxonDetail&page=taxonDetail&taxon_oid=2574179798 |
|  | *Mycobacterium tuberculosis* XTB13-131 | 2574180128 | https://img.jgi.doe.gov/cgi-bin/m/main.cgi?section=TaxonDetail&page=taxonDetail&taxon_oid=2574180128 |
|  | *Mycobacterium tuberculosis* KT-0019 | 2582581186 | https://img.jgi.doe.gov/cgi-bin/m/main.cgi?section=TaxonDetail&page=taxonDetail&taxon_oid=2582581186 |
|  | *Mycobacterium tuberculosis* TKK_04_0022 | 2590828599 | https://img.jgi.doe.gov/cgi-bin/m/main.cgi?section=TaxonDetail&page=taxonDetail&taxon_oid=2590828599 |
|  | *Mycobacterium tuberculosis* MAL010087 | 2588254093 | https://img.jgi.doe.gov/cgi-bin/m/main.cgi?section=TaxonDetail&page=taxonDetail&taxon_oid=2588254093 |
|  | *Mycobacterium tuberculosis* BTB09-058 | 2576861353 | https://img.jgi.doe.gov/cgi-bin/m/main.cgi?section=TaxonDetail&page=taxonDetail&taxon_oid=2576861353 |
|  | *Mycobacterium tuberculosis* KT-0057 | 2582581156 | https://img.jgi.doe.gov/cgi-bin/m/main.cgi?section=TaxonDetail&page=taxonDetail&taxon_oid=2582581156 |
|  | *Mycobacterium tuberculosis* TKK_04_0030 | 2590828681 | https://img.jgi.doe.gov/cgi-bin/m/main.cgi?section=TaxonDetail&page=taxonDetail&taxon_oid=2590828681 |
|  | *Mycobacterium tuberculosis* TKK-01-0078 | 2588254028 | https://img.jgi.doe.gov/cgi-bin/m/main.cgi?section=TaxonDetail&page=taxonDetail&taxon_oid=2588254028 |
|  | *Mycobacterium tuberculosis* PanR0307 | 2554235241 | https://img.jgi.doe.gov/cgi-bin/m/main.cgi?section=TaxonDetail&page=taxonDetail&taxon_oid=2554235241 |
|  | *Mycobacterium tuberculosis* MAL020136 | 2588254106 | https://img.jgi.doe.gov/cgi-bin/m/main.cgi?section=TaxonDetail&page=taxonDetail&taxon_oid=2588254106 |
|  | *Mycobacterium tuberculosis* Korean KIT87190 | 2588253748 | https://img.jgi.doe.gov/cgi-bin/m/main.cgi?section=TaxonDetail&page=taxonDetail&taxon_oid=2588253748 |
|  | *Mycobacterium tuberculosis* SUMu008 | 648276700 | https://img.jgi.doe.gov/cgi-bin/m/main.cgi?section=TaxonDetail&page=taxonDetail&taxon_oid=648276700 |
|  | *Mycobacterium tuberculosis* TRUG0076 | 2574180230 | https://img.jgi.doe.gov/cgi-bin/m/main.cgi?section=TaxonDetail&page=taxonDetail&taxon_oid=2574180230 |
|  | *Mycobacterium massiliense* 2B-0912-S | 2526164654 | https://img.jgi.doe.gov/cgi-bin/m/main.cgi?section=TaxonDetail&page=taxonDetail&taxon_oid=2526164654 |
|  | *Mycobacterium tuberculosis* PanR0206 | 2554235209 | https://img.jgi.doe.gov/cgi-bin/m/main.cgi?section=TaxonDetail&page=taxonDetail&taxon_oid=2554235209 |
|  | *Mycobacterium tuberculosis* KT-0079 | 2588254237 | https://img.jgi.doe.gov/cgi-bin/m/main.cgi?section=TaxonDetail&page=taxonDetail&taxon_oid=2588254237 |
|  | *Mycobacterium tuberculosis* H1578 | 2574179860 | https://img.jgi.doe.gov/cgi-bin/m/main.cgi?section=TaxonDetail&page=taxonDetail&taxon_oid=2574179860 |
|  | *Mycobacterium tuberculosis* TKK-01-0048 | 2588254007 | https://img.jgi.doe.gov/cgi-bin/m/main.cgi?section=TaxonDetail&page=taxonDetail&taxon_oid=2588254007 |
|  | *Mycobacterium africanum* MAL010099 | 2582580875 | https://img.jgi.doe.gov/cgi-bin/m/main.cgi?section=TaxonDetail&page=taxonDetail&taxon_oid=2582580875 |
|  | *Mycobacterium tuberculosis* MD16728 | 2576861232 | https://img.jgi.doe.gov/cgi-bin/m/main.cgi?section=TaxonDetail&page=taxonDetail&taxon_oid=2576861232 |
|  | *Mycobacterium tuberculosis* TKK-01-0055 | 2588254016 | https://img.jgi.doe.gov/cgi-bin/m/main.cgi?section=TaxonDetail&page=taxonDetail&taxon_oid=2588254016 |
|  | *Mycobacterium tuberculosis* TB_RSA12 | 2576861058 | https://img.jgi.doe.gov/cgi-bin/m/main.cgi?section=TaxonDetail&page=taxonDetail&taxon_oid=2576861058 |
|  | *Mycobacterium tuberculosis* TKK-01-0001 | 2588253977 | https://img.jgi.doe.gov/cgi-bin/m/main.cgi?section=TaxonDetail&page=taxonDetail&taxon_oid=2588253977 |
|  | *Mycobacterium tuberculosis* OFXR-23 | 2588254182 | https://img.jgi.doe.gov/cgi-bin/m/main.cgi?section=TaxonDetail&page=taxonDetail&taxon_oid=2588254182 |
|  | *Mycobacterium tuberculosis* KT-0026 | 2588254153 | https://img.jgi.doe.gov/cgi-bin/m/main.cgi?section=TaxonDetail&page=taxonDetail&taxon_oid=2588254153 |
|  | *Mycobacterium tuberculosis* TB_RSA66 | 2574180383 | https://img.jgi.doe.gov/cgi-bin/m/main.cgi?section=TaxonDetail&page=taxonDetail&taxon_oid=2574180383 |
|  | *Mycobacterium tuberculosis* MD16775 | 2574180210 | https://img.jgi.doe.gov/cgi-bin/m/main.cgi?section=TaxonDetail&page=taxonDetail&taxon_oid=2574180210 |
|  | *Mycobacterium tuberculosis* TRUG0088 | 2574180110 | https://img.jgi.doe.gov/cgi-bin/m/main.cgi?section=TaxonDetail&page=taxonDetail&taxon_oid=2574180110 |
|  | *Mycobacterium tuberculosis* TKK_03_0094 | 2576861394 | https://img.jgi.doe.gov/cgi-bin/m/main.cgi?section=TaxonDetail&page=taxonDetail&taxon_oid=2576861394 |
|  | *Mycobacterium tuberculosis* TBR31 | 2574179818 | https://img.jgi.doe.gov/cgi-bin/m/main.cgi?section=TaxonDetail&page=taxonDetail&taxon_oid=2574179818 |
|  | *Mycobacterium tuberculosis* XTB13-100 | 2582581204 | https://img.jgi.doe.gov/cgi-bin/m/main.cgi?section=TaxonDetail&page=taxonDetail&taxon_oid=2582581204 |
|  | *Mycobacterium tuberculosis* SUMu005 | 648276697 | https://img.jgi.doe.gov/cgi-bin/m/main.cgi?section=TaxonDetail&page=taxonDetail&taxon_oid=648276697 |
|  | *Mycobacterium* sp. 155 | 2516493018 | https://img.jgi.doe.gov/cgi-bin/m/main.cgi?section=TaxonDetail&page=taxonDetail&taxon_oid=2516493018 |
|  | *Mycobacterium tuberculosis* TBR57 | 2588253884 | https://img.jgi.doe.gov/cgi-bin/m/main.cgi?section=TaxonDetail&page=taxonDetail&taxon_oid=2588253884 |
|  | *Mycobacterium tuberculosis* XTB13-096 | 2574180267 | https://img.jgi.doe.gov/cgi-bin/m/main.cgi?section=TaxonDetail&page=taxonDetail&taxon_oid=2574180267 |
|  | *Mycobacterium* sp. URHB0044 | 2556921043 | https://img.jgi.doe.gov/cgi-bin/m/main.cgi?section=TaxonDetail&page=taxonDetail&taxon_oid=2556921043 |
|  | *Mycobacterium tuberculosis* TB_RSA25 | 2582581159 | https://img.jgi.doe.gov/cgi-bin/m/main.cgi?section=TaxonDetail&page=taxonDetail&taxon_oid=2582581159 |
|  | *Mycobacterium bovis* MAL010093 | 2582580880 | https://img.jgi.doe.gov/cgi-bin/m/main.cgi?section=TaxonDetail&page=taxonDetail&taxon_oid=2582580880 |
|  | *Mycobacterium tuberculosis* TKK-01-0014 | 2588253985 | https://img.jgi.doe.gov/cgi-bin/m/main.cgi?section=TaxonDetail&page=taxonDetail&taxon_oid=2588253985 |
|  | *Mycobacterium tuberculosis* TKK_02_0017 | 2590828646 | https://img.jgi.doe.gov/cgi-bin/m/main.cgi?section=TaxonDetail&page=taxonDetail&taxon_oid=2590828646 |
|  | *Mycobacterium tuberculosis* PanR1006 | 2554235279 | https://img.jgi.doe.gov/cgi-bin/m/main.cgi?section=TaxonDetail&page=taxonDetail&taxon_oid=2554235279 |
|  | *Mycobacterium tuberculosis* M1559 | 2574180156 | https://img.jgi.doe.gov/cgi-bin/m/main.cgi?section=TaxonDetail&page=taxonDetail&taxon_oid=2574180156 |
|  | *Mycobacterium tuberculosis* MAL020150 | 2588254117 | https://img.jgi.doe.gov/cgi-bin/m/main.cgi?section=TaxonDetail&page=taxonDetail&taxon_oid=2588254117 |
|  | *Mycobacterium tuberculosis* MAL020206 | 2588254137 | https://img.jgi.doe.gov/cgi-bin/m/main.cgi?section=TaxonDetail&page=taxonDetail&taxon_oid=2588254137 |
|  | *Mycobacterium tuberculosis* R1207 | 2547132238 | https://img.jgi.doe.gov/cgi-bin/m/main.cgi?section=TaxonDetail&page=taxonDetail&taxon_oid=2547132238 |
|  | *Mycobacterium tuberculosis* SK-E | 2582581139 | https://img.jgi.doe.gov/cgi-bin/m/main.cgi?section=TaxonDetail&page=taxonDetail&taxon_oid=2582581139 |
|  | *Mycobacterium africanum* MAL010118 | 2579778997 | https://img.jgi.doe.gov/cgi-bin/m/main.cgi?section=TaxonDetail&page=taxonDetail&taxon_oid=2579778997 |
|  | *Mycobacterium intracellulare* ATCC 13950 | 2519103109 | https://img.jgi.doe.gov/cgi-bin/m/main.cgi?section=TaxonDetail&page=taxonDetail&taxon_oid=2519103109 |
|  | *Mycobacterium tuberculosis* TKK-01-0046 | 2588254008 | https://img.jgi.doe.gov/cgi-bin/m/main.cgi?section=TaxonDetail&page=taxonDetail&taxon_oid=2588254008 |
|  | *Mycobacterium tuberculosis* KT-0099 | 2588254229 | https://img.jgi.doe.gov/cgi-bin/m/main.cgi?section=TaxonDetail&page=taxonDetail&taxon_oid=2588254229 |
|  | *Mycobacterium tuberculosis* BTB08-022 | 2576861221 | https://img.jgi.doe.gov/cgi-bin/m/main.cgi?section=TaxonDetail&page=taxonDetail&taxon_oid=2576861221 |
|  | *Mycobacterium tuberculosis* OFXR-18 | 2588254186 | https://img.jgi.doe.gov/cgi-bin/m/main.cgi?section=TaxonDetail&page=taxonDetail&taxon_oid=2588254186 |
|  | *Mycobacterium tuberculosis* TKK-01-0006 | 2576861099 | https://img.jgi.doe.gov/cgi-bin/m/main.cgi?section=TaxonDetail&page=taxonDetail&taxon_oid=2576861099 |
|  | *Mycobacterium tuberculosis* BTB10-487 | 2576861026 | https://img.jgi.doe.gov/cgi-bin/m/main.cgi?section=TaxonDetail&page=taxonDetail&taxon_oid=2576861026 |
|  | *Mycobacterium tuberculosis* TKK-01-0002 | 2588253976 | https://img.jgi.doe.gov/cgi-bin/m/main.cgi?section=TaxonDetail&page=taxonDetail&taxon_oid=2588253976 |
|  | *Mycobacterium tuberculosis* TKK-01-0020 | 2588253847 | https://img.jgi.doe.gov/cgi-bin/m/main.cgi?section=TaxonDetail&page=taxonDetail&taxon_oid=2588253847 |
|  | *Mycobacterium tuberculosis* TKK_03_0030 | 2590828618 | https://img.jgi.doe.gov/cgi-bin/m/main.cgi?section=TaxonDetail&page=taxonDetail&taxon_oid=2590828618 |
|  | *Mycobacterium tuberculosis* MAL020167 | 2588254112 | https://img.jgi.doe.gov/cgi-bin/m/main.cgi?section=TaxonDetail&page=taxonDetail&taxon_oid=2588254112 |
|  | *Mycobacterium tuberculosis* M2116 | 2576861330 | https://img.jgi.doe.gov/cgi-bin/m/main.cgi?section=TaxonDetail&page=taxonDetail&taxon_oid=2576861330 |
|  | *Mycobacterium tuberculosis* TKK_04_0013 | 2590828605 | https://img.jgi.doe.gov/cgi-bin/m/main.cgi?section=TaxonDetail&page=taxonDetail&taxon_oid=2590828605 |
|  | *Mycobacterium tuberculosis* PanR0503 | 2554235255 | https://img.jgi.doe.gov/cgi-bin/m/main.cgi?section=TaxonDetail&page=taxonDetail&taxon_oid=2554235255 |
|  | *Mycobacterium tuberculosis* T17 | 642979349 | https://img.jgi.doe.gov/cgi-bin/m/main.cgi?section=TaxonDetail&page=taxonDetail&taxon_oid=642979349 |
|  | *Mycobacterium tuberculosis* TKK_04_0094 | 2582581218 | https://img.jgi.doe.gov/cgi-bin/m/main.cgi?section=TaxonDetail&page=taxonDetail&taxon_oid=2582581218 |
|  | *Mycobacterium tuberculosis* TKK_03_0096 | 2576861378 | https://img.jgi.doe.gov/cgi-bin/m/main.cgi?section=TaxonDetail&page=taxonDetail&taxon_oid=2576861378 |
|  | *Mycobacterium tuberculosis* M2249 | 2576861297 | https://img.jgi.doe.gov/cgi-bin/m/main.cgi?section=TaxonDetail&page=taxonDetail&taxon_oid=2576861297 |
|  | *Mycobacterium tuberculosis* KT-0067 | 2574179838 | https://img.jgi.doe.gov/cgi-bin/m/main.cgi?section=TaxonDetail&page=taxonDetail&taxon_oid=2574179838 |
|  | *Mycobacterium tuberculosis* TKK_03_0029 | 2590828619 | https://img.jgi.doe.gov/cgi-bin/m/main.cgi?section=TaxonDetail&page=taxonDetail&taxon_oid=2590828619 |
|  | *Mycobacterium tuberculosis* TKK_04_0033 | 2576861374 | https://img.jgi.doe.gov/cgi-bin/m/main.cgi?section=TaxonDetail&page=taxonDetail&taxon_oid=2576861374 |
|  | *Mycobacterium tuberculosis* TB_RSA136 | 2574180334 | https://img.jgi.doe.gov/cgi-bin/m/main.cgi?section=TaxonDetail&page=taxonDetail&taxon_oid=2574180334 |
|  | *Mycobacterium tuberculosis* TKK_02_0034 | 2590828638 | https://img.jgi.doe.gov/cgi-bin/m/main.cgi?section=TaxonDetail&page=taxonDetail&taxon_oid=2590828638 |
|  | *Mycobacterium massiliense* 1S-154-0310 | 2526164579 | https://img.jgi.doe.gov/cgi-bin/m/main.cgi?section=TaxonDetail&page=taxonDetail&taxon_oid=2526164579 |
|  | *Mycobacterium tuberculosis* EAI5 | 2554235430 | https://img.jgi.doe.gov/cgi-bin/m/main.cgi?section=TaxonDetail&page=taxonDetail&taxon_oid=2554235430 |
|  | *Mycobacterium tuberculosis* TKK_02_0077 | 2590828625 | https://img.jgi.doe.gov/cgi-bin/m/main.cgi?section=TaxonDetail&page=taxonDetail&taxon_oid=2590828625 |
|  | *Mycobacterium tuberculosis* TBR42 | 2588253878 | https://img.jgi.doe.gov/cgi-bin/m/main.cgi?section=TaxonDetail&page=taxonDetail&taxon_oid=2588253878 |
|  | *Mycobacterium tuberculosis* OFXR-9 | 2588254141 | https://img.jgi.doe.gov/cgi-bin/m/main.cgi?section=TaxonDetail&page=taxonDetail&taxon_oid=2588254141 |
|  | *Mycobacterium tuberculosis* TB_RSA165 | 2574180040 | https://img.jgi.doe.gov/cgi-bin/m/main.cgi?section=TaxonDetail&page=taxonDetail&taxon_oid=2574180040 |
|  | *Mycobacterium tuberculosis* XTB13-136 | 2574180422 | https://img.jgi.doe.gov/cgi-bin/m/main.cgi?section=TaxonDetail&page=taxonDetail&taxon_oid=2574180422 |
|  | *Mycobacterium tuberculosis* PR05 | 2554235105 | https://img.jgi.doe.gov/cgi-bin/m/main.cgi?section=TaxonDetail&page=taxonDetail&taxon_oid=2554235105 |
|  | *Mycobacterium tuberculosis* TKK-01-0058 | 2588254011 | https://img.jgi.doe.gov/cgi-bin/m/main.cgi?section=TaxonDetail&page=taxonDetail&taxon_oid=2588254011 |
|  | *Mycobacterium tuberculosis* MD18096 | 2574180286 | https://img.jgi.doe.gov/cgi-bin/m/main.cgi?section=TaxonDetail&page=taxonDetail&taxon_oid=2574180286 |
|  | *Mycobacterium tuberculosis* KZN V2475 | 647000281 | https://img.jgi.doe.gov/cgi-bin/m/main.cgi?section=TaxonDetail&page=taxonDetail&taxon_oid=647000281 |
|  | *Mycobacterium tuberculosis* TKK-01-0040 | 2588253999 | https://img.jgi.doe.gov/cgi-bin/m/main.cgi?section=TaxonDetail&page=taxonDetail&taxon_oid=2588253999 |
|  | *Mycobacterium tuberculosis* TKK-01-0031 | 2588253992 | https://img.jgi.doe.gov/cgi-bin/m/main.cgi?section=TaxonDetail&page=taxonDetail&taxon_oid=2588253992 |
|  | *Mycobacterium tuberculosis* PanR0205 | 2554235208 | https://img.jgi.doe.gov/cgi-bin/m/main.cgi?section=TaxonDetail&page=taxonDetail&taxon_oid=2554235208 |
|  | *Mycobacterium tuberculosis* M1762 | 2574179894 | https://img.jgi.doe.gov/cgi-bin/m/main.cgi?section=TaxonDetail&page=taxonDetail&taxon_oid=2574179894 |
|  | *Mycobacterium massiliense* 1513 | 2565956791 | https://img.jgi.doe.gov/cgi-bin/m/main.cgi?section=TaxonDetail&page=taxonDetail&taxon_oid=2565956791 |
|  | *Mycobacterium tuberculosis* TKK_04_0036 | 2590828678 | https://img.jgi.doe.gov/cgi-bin/m/main.cgi?section=TaxonDetail&page=taxonDetail&taxon_oid=2590828678 |
|  | *Mycobacterium tuberculosis* MAL020193 | 2588254127 | https://img.jgi.doe.gov/cgi-bin/m/main.cgi?section=TaxonDetail&page=taxonDetail&taxon_oid=2588254127 |
|  | *Mycobacterium tuberculosis* MAL010086 | 2588254094 | https://img.jgi.doe.gov/cgi-bin/m/main.cgi?section=TaxonDetail&page=taxonDetail&taxon_oid=2588254094 |
|  | *Mycobacterium thermoresistibile* ATCC 19527 | 2519103087 | https://img.jgi.doe.gov/cgi-bin/m/main.cgi?section=TaxonDetail&page=taxonDetail&taxon_oid=2519103087 |
|  | *Mycobacterium tuberculosis* TKK_02_0019 | 2574180017 | https://img.jgi.doe.gov/cgi-bin/m/main.cgi?section=TaxonDetail&page=taxonDetail&taxon_oid=2574180017 |
|  | *Mycobacterium tuberculosis* XTB13-156 | 2574180393 | https://img.jgi.doe.gov/cgi-bin/m/main.cgi?section=TaxonDetail&page=taxonDetail&taxon_oid=2574180393 |
|  | *Mycobacterium tuberculosis* TKK-01-0012 | 2588253982 | https://img.jgi.doe.gov/cgi-bin/m/main.cgi?section=TaxonDetail&page=taxonDetail&taxon_oid=2588253982 |
|  | *Mycobacterium tuberculosis* TB_RSA102 | 2574180173 | https://img.jgi.doe.gov/cgi-bin/m/main.cgi?section=TaxonDetail&page=taxonDetail&taxon_oid=2574180173 |
|  | *Mycobacterium tuberculosis* OFXR-6 | 2574180016 | https://img.jgi.doe.gov/cgi-bin/m/main.cgi?section=TaxonDetail&page=taxonDetail&taxon_oid=2574180016 |
|  | *Mycobacterium tuberculosis* TB_RSA124 | 2576861380 | https://img.jgi.doe.gov/cgi-bin/m/main.cgi?section=TaxonDetail&page=taxonDetail&taxon_oid=2576861380 |
|  | *Mycobacterium africanum* MAL010070 | 2582580874 | https://img.jgi.doe.gov/cgi-bin/m/main.cgi?section=TaxonDetail&page=taxonDetail&taxon_oid=2582580874 |
|  | *Mycobacteroides massiliense* GO 06 | 2517093032 | https://img.jgi.doe.gov/cgi-bin/m/main.cgi?section=TaxonDetail&page=taxonDetail&taxon_oid=2517093032 |
|  | *Mycobacterium tuberculosis* TKK-01-0071 | 2588254022 | https://img.jgi.doe.gov/cgi-bin/m/main.cgi?section=TaxonDetail&page=taxonDetail&taxon_oid=2588254022 |
|  | *Mycobacterium tuberculosis* TKK_03_0078 | 2582581142 | https://img.jgi.doe.gov/cgi-bin/m/main.cgi?section=TaxonDetail&page=taxonDetail&taxon_oid=2582581142 |
|  | *Mycobacterium tuberculosis* KT-0015 | 2588254158 | https://img.jgi.doe.gov/cgi-bin/m/main.cgi?section=TaxonDetail&page=taxonDetail&taxon_oid=2588254158 |
|  | *Mycobacterium tuberculosis* TKK_05MA_0052 | 2576861070 | https://img.jgi.doe.gov/cgi-bin/m/main.cgi?section=TaxonDetail&page=taxonDetail&taxon_oid=2576861070 |
|  | *Mycobacterium tuberculosis* PanR0201 | 2554235305 | https://img.jgi.doe.gov/cgi-bin/m/main.cgi?section=TaxonDetail&page=taxonDetail&taxon_oid=2554235305 |
|  | *Mycobacterium tuberculosis* TKK_04_0047 | 2590828673 | https://img.jgi.doe.gov/cgi-bin/m/main.cgi?section=TaxonDetail&page=taxonDetail&taxon_oid=2590828673 |
|  | *Mycobacterium tuberculosis* Uganda 1 | 2574180039 | https://img.jgi.doe.gov/cgi-bin/m/main.cgi?section=TaxonDetail&page=taxonDetail&taxon_oid=2574180039 |
|  | *Mycobacterium tuberculosis* TKK-01-0021 | 2582581183 | https://img.jgi.doe.gov/cgi-bin/m/main.cgi?section=TaxonDetail&page=taxonDetail&taxon_oid=2582581183 |
|  | *Mycobacterium tuberculosis* PanR0903 | 2554235261 | https://img.jgi.doe.gov/cgi-bin/m/main.cgi?section=TaxonDetail&page=taxonDetail&taxon_oid=2554235261 |
|  | *Mycobacterium tuberculosis* KT-0083 | 2588254235 | https://img.jgi.doe.gov/cgi-bin/m/main.cgi?section=TaxonDetail&page=taxonDetail&taxon_oid=2588254235 |
|  | *Mycobacterium tuberculosis* TB_RSA45 | 2582581161 | https://img.jgi.doe.gov/cgi-bin/m/main.cgi?section=TaxonDetail&page=taxonDetail&taxon_oid=2582581161 |
|  | *Mycobacterium tuberculosis* TB_RSA18 | 2574180376 | https://img.jgi.doe.gov/cgi-bin/m/main.cgi?section=TaxonDetail&page=taxonDetail&taxon_oid=2574180376 |
|  | *Mycobacterium paraintracellulare* MOTT-64 | 2512564041 | https://img.jgi.doe.gov/cgi-bin/m/main.cgi?section=TaxonDetail&page=taxonDetail&taxon_oid=2512564041 |
|  | *Mycobacterium tuberculosis* UT205 | 2512564056 | https://img.jgi.doe.gov/cgi-bin/m/main.cgi?section=TaxonDetail&page=taxonDetail&taxon_oid=2512564056 |
|  | *Mycobacterium tuberculosis* TKK_05SA_0020 | 2582581170 | https://img.jgi.doe.gov/cgi-bin/m/main.cgi?section=TaxonDetail&page=taxonDetail&taxon_oid=2582581170 |
|  | *Mycobacterium tuberculosis* MAL010110 | 2588254098 | https://img.jgi.doe.gov/cgi-bin/m/main.cgi?section=TaxonDetail&page=taxonDetail&taxon_oid=2588254098 |
|  | *Mycobacterium tuberculosis* PanR0606 | 2554235257 | https://img.jgi.doe.gov/cgi-bin/m/main.cgi?section=TaxonDetail&page=taxonDetail&taxon_oid=2554235257 |
|  | *Mycobacterium tuberculosis* TKK_05MA_0040 | 2582581163 | https://img.jgi.doe.gov/cgi-bin/m/main.cgi?section=TaxonDetail&page=taxonDetail&taxon_oid=2582581163 |
|  | *Mycobacterium africanum* MAL010081 | 2579778712 | https://img.jgi.doe.gov/cgi-bin/m/main.cgi?section=TaxonDetail&page=taxonDetail&taxon_oid=2579778712 |
|  | *Mycobacterium tuberculosis* MD14435 | 2576861267 | https://img.jgi.doe.gov/cgi-bin/m/main.cgi?section=TaxonDetail&page=taxonDetail&taxon_oid=2576861267 |
|  | *Mycobacterium tuberculosis* TBR79 | 2588253893 | https://img.jgi.doe.gov/cgi-bin/m/main.cgi?section=TaxonDetail&page=taxonDetail&taxon_oid=2588253893 |
|  | *Mycobacterium tuberculosis* TKK_02_0062 | 2590828632 | https://img.jgi.doe.gov/cgi-bin/m/main.cgi?section=TaxonDetail&page=taxonDetail&taxon_oid=2590828632 |
|  | *Mycobacterium tuberculosis* OFXR-31 | 2588254178 | https://img.jgi.doe.gov/cgi-bin/m/main.cgi?section=TaxonDetail&page=taxonDetail&taxon_oid=2588254178 |
|  | *Mycobacterium tuberculosis* TKK-01-0052 | 2588254004 | https://img.jgi.doe.gov/cgi-bin/m/main.cgi?section=TaxonDetail&page=taxonDetail&taxon_oid=2588254004 |
|  | *Mycobacterium tuberculosis* SUMu001 | 648276693 | https://img.jgi.doe.gov/cgi-bin/m/main.cgi?section=TaxonDetail&page=taxonDetail&taxon_oid=648276693 |
|  | *Mycobacterium tuberculosis* M1978 | 2576861346 | https://img.jgi.doe.gov/cgi-bin/m/main.cgi?section=TaxonDetail&page=taxonDetail&taxon_oid=2576861346 |
|  | *Mycobacterium tuberculosis* BTB05-013 | 2574180369 | https://img.jgi.doe.gov/cgi-bin/m/main.cgi?section=TaxonDetail&page=taxonDetail&taxon_oid=2574180369 |
|  | *Mycobacterium tuberculosis* C | 638341130 | https://img.jgi.doe.gov/cgi-bin/m/main.cgi?section=TaxonDetail&page=taxonDetail&taxon_oid=638341130 |
|  | *Mycobacterium tuberculosis* TKK_02_0001 | 2590828652 | https://img.jgi.doe.gov/cgi-bin/m/main.cgi?section=TaxonDetail&page=taxonDetail&taxon_oid=2590828652 |
|  | *Mycobacterium tuberculosis* KT-0075 | 2588254240 | https://img.jgi.doe.gov/cgi-bin/m/main.cgi?section=TaxonDetail&page=taxonDetail&taxon_oid=2588254240 |
|  | *Mycobacterium africanum* MAL020176 | 2582580879 | https://img.jgi.doe.gov/cgi-bin/m/main.cgi?section=TaxonDetail&page=taxonDetail&taxon_oid=2582580879 |
|  | *Mycobacterium tuberculosis* TKK_02_0045 | 2574180313 | https://img.jgi.doe.gov/cgi-bin/m/main.cgi?section=TaxonDetail&page=taxonDetail&taxon_oid=2574180313 |
|  | *Mycobacterium tuberculosis* MD19043 | 2574179852 | https://img.jgi.doe.gov/cgi-bin/m/main.cgi?section=TaxonDetail&page=taxonDetail&taxon_oid=2574179852 |
|  | *Mycobacterium tuberculosis* TKK-01-0015 | 2588253989 | https://img.jgi.doe.gov/cgi-bin/m/main.cgi?section=TaxonDetail&page=taxonDetail&taxon_oid=2588253989 |
|  | *Mycobacterium tuberculosis* NRITLD09 | 2582581182 | https://img.jgi.doe.gov/cgi-bin/m/main.cgi?section=TaxonDetail&page=taxonDetail&taxon_oid=2582581182 |
|  | *Mycobacterium tuberculosis* XTB13-175 | 2582581147 | https://img.jgi.doe.gov/cgi-bin/m/main.cgi?section=TaxonDetail&page=taxonDetail&taxon_oid=2582581147 |
|  | *Mycobacterium tuberculosis* TKK_03_0103 | 2576861260 | https://img.jgi.doe.gov/cgi-bin/m/main.cgi?section=TaxonDetail&page=taxonDetail&taxon_oid=2576861260 |
|  | *Mycobacterium tuberculosis* XTB13-093 | 2574180354 | https://img.jgi.doe.gov/cgi-bin/m/main.cgi?section=TaxonDetail&page=taxonDetail&taxon_oid=2574180354 |
|  | *Mycobacterium tuberculosis* TKK-01-0094 | 2588254035 | https://img.jgi.doe.gov/cgi-bin/m/main.cgi?section=TaxonDetail&page=taxonDetail&taxon_oid=2588254035 |
|  | *Mycobacterium* sp. UM_WGJ | 2554235289 | https://img.jgi.doe.gov/cgi-bin/m/main.cgi?section=TaxonDetail&page=taxonDetail&taxon_oid=2554235289 |
|  | *Mycobacterium canettii* CIPT 140060008 | 2541047047 | https://img.jgi.doe.gov/cgi-bin/m/main.cgi?section=TaxonDetail&page=taxonDetail&taxon_oid=2541047047 |
|  | *Mycobacterium colombiense* CECT 3035 | 2562617111 | https://img.jgi.doe.gov/cgi-bin/m/main.cgi?section=TaxonDetail&page=taxonDetail&taxon_oid=2562617111 |
|  | *Mycobacterium tuberculosis* TBR26 | 2576861259 | https://img.jgi.doe.gov/cgi-bin/m/main.cgi?section=TaxonDetail&page=taxonDetail&taxon_oid=2576861259 |
|  | *Mycobacterium tuberculosis* TKK-01-0070 | 2588254023 | https://img.jgi.doe.gov/cgi-bin/m/main.cgi?section=TaxonDetail&page=taxonDetail&taxon_oid=2588254023 |
|  | *Mycobacterium tuberculosis* KT-0039 | 2588254150 | https://img.jgi.doe.gov/cgi-bin/m/main.cgi?section=TaxonDetail&page=taxonDetail&taxon_oid=2588254150 |
|  | *Mycobacterium tuberculosis* RGTB327 | 2512564073 | https://img.jgi.doe.gov/cgi-bin/m/main.cgi?section=TaxonDetail&page=taxonDetail&taxon_oid=2512564073 |
|  | *Mycobacterium tuberculosis* XTB13-195 | 2574180234 | https://img.jgi.doe.gov/cgi-bin/m/main.cgi?section=TaxonDetail&page=taxonDetail&taxon_oid=2574180234 |
|  | *Mycobacterium tuberculosis* TB_RSA99 | 2574180102 | https://img.jgi.doe.gov/cgi-bin/m/main.cgi?section=TaxonDetail&page=taxonDetail&taxon_oid=2574180102 |
|  | *Mycobacterium tuberculosis* XTB13-203 | 2582581177 | https://img.jgi.doe.gov/cgi-bin/m/main.cgi?section=TaxonDetail&page=taxonDetail&taxon_oid=2582581177 |
|  | *Mycobacterium tuberculosis* TKK_04_0108 | 2574180399 | https://img.jgi.doe.gov/cgi-bin/m/main.cgi?section=TaxonDetail&page=taxonDetail&taxon_oid=2574180399 |
|  | *Mycobacterium tuberculosis* KT-0107 | 2588254225 | https://img.jgi.doe.gov/cgi-bin/m/main.cgi?section=TaxonDetail&page=taxonDetail&taxon_oid=2588254225 |
|  | *Mycobacterium tuberculosis* MAL020197 | 2588254130 | https://img.jgi.doe.gov/cgi-bin/m/main.cgi?section=TaxonDetail&page=taxonDetail&taxon_oid=2588254130 |
|  | *Mycobacterium tuberculosis* 3499MM | 2582581193 | https://img.jgi.doe.gov/cgi-bin/m/main.cgi?section=TaxonDetail&page=taxonDetail&taxon_oid=2582581193 |
| **Mycobacterium causing leprosy (MCL)** | | | |
|  | *Mycobacterium leprae* TN | 637000170 | https://img.jgi.doe.gov/cgi-bin/m/main.cgi?section=TaxonDetail&page=taxonDetail&taxon_oid=637000170 |
|  | *Mycobacterium leprae* Br4923 | 643348566 | https://img.jgi.doe.gov/cgi-bin/m/main.cgi?section=TaxonDetail&page=taxonDetail&taxon_oid=643348566 |
| **Saprophytes (SAP)** | | | |
|  | *Mycobacterium smegmatis* MKD8 | 2545824527 | https://img.jgi.doe.gov/cgi-bin/m/main.cgi?section=TaxonDetail&page=taxonDetail&taxon_oid=2545824527 |
|  | *Mycobacterium smegmatis* MC2 51 | 2585427824 | https://img.jgi.doe.gov/cgi-bin/m/main.cgi?section=TaxonDetail&page=taxonDetail&taxon_oid=2585427824 |
|  | *Mycobacterium smegmatis* MC2 155 | 639633041 | https://img.jgi.doe.gov/cgi-bin/m/main.cgi?section=TaxonDetail&page=taxonDetail&taxon_oid=639633041 |
|  | *Mycobacterium smegmatis* MC2 155 | 2518645537 | https://img.jgi.doe.gov/cgi-bin/m/main.cgi?section=TaxonDetail&page=taxonDetail&taxon_oid=2518645537 |
|  | *Mycobacterium smegmatis* LR222 | 651716594 | https://img.jgi.doe.gov/cgi-bin/m/main.cgi?section=TaxonDetail&page=taxonDetail&taxon_oid=651716594 |
|  | *Mycobacterium smegmatis* MC2 155 | 651716860 | https://img.jgi.doe.gov/cgi-bin/m/main.cgi?section=TaxonDetail&page=taxonDetail&taxon_oid=651716860 |
|  | *Mycobacterium* sp. JS623 | 2506783060 | https://img.jgi.doe.gov/cgi-bin/m/main.cgi?section=TaxonDetail&page=taxonDetail&taxon_oid=2506783060 |
|  | *Mycobacterium vanbaalenii* PYR-1 | 639633044 | https://img.jgi.doe.gov/cgi-bin/m/main.cgi?section=TaxonDetail&page=taxonDetail&taxon_oid=639633044 |
|  | *Mycobacterium gilvum* Spyr1 | 649633070 | https://img.jgi.doe.gov/cgi-bin/m/main.cgi?section=TaxonDetail&page=taxonDetail&taxon_oid=649633070 |
|  | *Mycolicibacterium gilvum* PYR-GCK | 640427122 | https://img.jgi.doe.gov/cgi-bin/m/main.cgi?section=TaxonDetail&page=taxonDetail&taxon_oid=640427122 |
|  | *Mycobacterium* sp. MCS | 637000171 | https://img.jgi.doe.gov/cgi-bin/m/main.cgi?section=TaxonDetail&page=taxonDetail&taxon_oid=637000171 |
|  | *Mycobacterium* sp. KMS | 639633042 | https://img.jgi.doe.gov/cgi-bin/m/main.cgi?section=TaxonDetail&page=taxonDetail&taxon_oid=639633042 |
|  | *Mycobacterium* sp. JLS | 640069320 | https://img.jgi.doe.gov/cgi-bin/m/main.cgi?section=TaxonDetail&page=taxonDetail&taxon_oid=640069320 |
|  | *Mycobacterium rhodesiae* NBB3 | 2508501106 | https://img.jgi.doe.gov/cgi-bin/m/main.cgi?section=TaxonDetail&page=taxonDetail&taxon_oid=2508501106 |
|  | *Mycolicibacterium rhodesiae* JS60 | 2506783048 | https://img.jgi.doe.gov/cgi-bin/m/main.cgi?section=TaxonDetail&page=taxonDetail&taxon_oid=2506783048 |
|  | *Mycobacterium chubuense* NBB4 | 2506783014 | https://img.jgi.doe.gov/cgi-bin/m/main.cgi?section=TaxonDetail&page=taxonDetail&taxon_oid=2506783014 |
|  | *Mycobacterium neoaurum* VKM Ac-1815D | 2551306466 | https://img.jgi.doe.gov/cgi-bin/m/main.cgi?section=TaxonDetail&page=taxonDetail&taxon_oid=2551306466 |
|  | *Mycobacterium neoaurum* ATCC 25795 | 2582581012 | https://img.jgi.doe.gov/cgi-bin/m/main.cgi?section=TaxonDetail&page=taxonDetail&taxon_oid=2582581012 |
|  | *Mycobacterium* sp. VKM Ac-1816D | 2551306546 | https://img.jgi.doe.gov/cgi-bin/m/main.cgi?section=TaxonDetail&page=taxonDetail&taxon_oid=2551306546 |
|  | *Mycobacterium* sp. VKM Ac-1817D | 2551306544 | https://img.jgi.doe.gov/cgi-bin/m/main.cgi?section=TaxonDetail&page=taxonDetail&taxon_oid=2551306544 |
|  | *Mycobacterium fortuitum* subsp. *fortuitum* DSM 46621 | 2519899744 | https://img.jgi.doe.gov/cgi-bin/m/main.cgi?section=TaxonDetail&page=taxonDetail&taxon_oid=2519899744 |
| ***Mycobacterium chelonae-abscessus Complex (MCAC)*** | | | |
|  | *Mycobacterium abscessus* 4S-0206 | 2526164587 | https://img.jgi.doe.gov/cgi-bin/m/main.cgi?section=TaxonDetail&page=taxonDetail&taxon_oid=2526164587 |
|  | *Mycobacterium abscessus*V06705 | 2582580872 | https://img.jgi.doe.gov/cgi-bin/m/main.cgi?section=TaxonDetail&page=taxonDetail&taxon_oid=2582580872 |
|  | *Mycobacterium abscessus* 5S-0921 | 2526164682 | https://img.jgi.doe.gov/cgi-bin/m/main.cgi?section=TaxonDetail&page=taxonDetail&taxon_oid=2526164682 |
|  | *Mycobacterium abscessus* M148 | 2548877037 | https://img.jgi.doe.gov/cgi-bin/m/main.cgi?section=TaxonDetail&page=taxonDetail&taxon_oid=2548877037 |
|  | *Mycobacterium abscessus* 3A-0122-S | 2526164623 | https://img.jgi.doe.gov/cgi-bin/m/main.cgi?section=TaxonDetail&page=taxonDetail&taxon_oid=2526164623 |
|  | *Mycobacterium abscessus bolletii* Timone LB | 2531839070 | https://img.jgi.doe.gov/cgi-bin/m/main.cgi?section=TaxonDetail&page=taxonDetail&taxon_oid=2531839070 |
|  | *Mycobacterium abscessus* 5S-0708 | 2526164593 | https://img.jgi.doe.gov/cgi-bin/m/main.cgi?section=TaxonDetail&page=taxonDetail&taxon_oid=2526164593 |
|  | *Mycobacterium abscessus* ATCC 19977 | 641522641 | https://img.jgi.doe.gov/cgi-bin/m/main.cgi?section=TaxonDetail&page=taxonDetail&taxon_oid=641522641 |
|  | *Mycobacterium abscessus* MAB_110811_1470 | 2579778595 | https://img.jgi.doe.gov/cgi-bin/m/main.cgi?section=TaxonDetail&page=taxonDetail&taxon_oid=2579778595 |
|  | *Mycobacterium abscessus* 1948 | 2565956814 | https://img.jgi.doe.gov/cgi-bin/m/main.cgi?section=TaxonDetail&page=taxonDetail&taxon_oid=2565956814 |
|  | *Mycobacterium abscessus* 6G-1108 | 2526164679 | https://img.jgi.doe.gov/cgi-bin/m/main.cgi?section=TaxonDetail&page=taxonDetail&taxon_oid=2526164679 |
|  | *Mycobacterium abscessus* 4S-0116-R | 2526164627 | https://img.jgi.doe.gov/cgi-bin/m/main.cgi?section=TaxonDetail&page=taxonDetail&taxon_oid=2526164627 |
|  | *Mycobacterium abscessus* M156 | 2548877034 | https://img.jgi.doe.gov/cgi-bin/m/main.cgi?section=TaxonDetail&page=taxonDetail&taxon_oid=2548877034 |
|  | *Mycobacterium abscessus*5S-0421 | 2526164590 | https://img.jgi.doe.gov/cgi-bin/m/main.cgi?section=TaxonDetail&page=taxonDetail&taxon_oid=2526164590 |
|  | *Mycobacterium abscessus* 9808 | 2551306463 | https://img.jgi.doe.gov/cgi-bin/m/main.cgi?section=TaxonDetail&page=taxonDetail&taxon_oid=2551306463 |
|  | *Mycobacterium abscessus*MAB_082312_2258 | 2579779115 | https://img.jgi.doe.gov/cgi-bin/m/main.cgi?section=TaxonDetail&page=taxonDetail&taxon_oid=2579779115 |
|  | *Mycobacterium abscessus* MAB_082312_2272 | 2582580860 | https://img.jgi.doe.gov/cgi-bin/m/main.cgi?section=TaxonDetail&page=taxonDetail&taxon_oid=2582580860 |
|  | *Mycobacterium abscessus bolletii* BD | 2529293190 | https://img.jgi.doe.gov/cgi-bin/m/main.cgi?section=TaxonDetail&page=taxonDetail&taxon_oid=2529293190 |
|  | *Mycobacterium abscessus* 6G-0212 | 2526164687 | https://img.jgi.doe.gov/cgi-bin/m/main.cgi?section=TaxonDetail&page=taxonDetail&taxon_oid=2526164687 |
|  | *Mycobacterium abscessus*4S-0116-S | 2526164588 | https://img.jgi.doe.gov/cgi-bin/m/main.cgi?section=TaxonDetail&page=taxonDetail&taxon_oid=2526164588 |
|  | *Mycobacterium abscessus*3A-0731 | 2526164624 | https://img.jgi.doe.gov/cgi-bin/m/main.cgi?section=TaxonDetail&page=taxonDetail&taxon_oid=2526164624 |
|  | *Mycobacterium abscessus* 6G-0728-S | 2526164685 | https://img.jgi.doe.gov/cgi-bin/m/main.cgi?section=TaxonDetail&page=taxonDetail&taxon_oid=2526164685 |
|  | *Mycobacterium abscessus* 3A-0122-R | 2531839202 | https://img.jgi.doe.gov/cgi-bin/m/main.cgi?section=TaxonDetail&page=taxonDetail&taxon_oid=2531839202 |
|  | *Mycobacterium abscessus*M172 | 2548876926 | https://img.jgi.doe.gov/cgi-bin/m/main.cgi?section=TaxonDetail&page=taxonDetail&taxon_oid=2548876926 |
|  | *Mycobacteroides abscessus* 4S-0726-RB | 2526164592 | https://img.jgi.doe.gov/cgi-bin/m/main.cgi?section=TaxonDetail&page=taxonDetail&taxon_oid=2526164592 |
|  | *Mycobacterium abscessus* 6G-0125-R | 2526164681 | https://img.jgi.doe.gov/cgi-bin/m/main.cgi?section=TaxonDetail&page=taxonDetail&taxon_oid=2526164681 |
|  | *Mycobacterium abscessus*M154 | 2548877035 | https://img.jgi.doe.gov/cgi-bin/m/main.cgi?section=TaxonDetail&page=taxonDetail&taxon_oid=2548877035 |
|  | *Mycobacterium abscessus*4S-0726-RA | 2526164585 | https://img.jgi.doe.gov/cgi-bin/m/main.cgi?section=TaxonDetail&page=taxonDetail&taxon_oid=2526164585 |
|  | *Mycobacterium abscessus bolletii* M18 | 2551306144 | https://img.jgi.doe.gov/cgi-bin/m/main.cgi?section=TaxonDetail&page=taxonDetail&taxon_oid=2551306144 |
|  | *Mycobacterium abscessus*5S-0817 | 2537561552 | https://img.jgi.doe.gov/cgi-bin/m/main.cgi?section=TaxonDetail&page=taxonDetail&taxon_oid=2537561552 |
|  | *Mycobacterium abscessus*M93 | 2516143102 | https://img.jgi.doe.gov/cgi-bin/m/main.cgi?section=TaxonDetail&page=taxonDetail&taxon_oid=2516143102 |
|  | *Mycobacterium abscessus* M94 | 2516143052 | https://img.jgi.doe.gov/cgi-bin/m/main.cgi?section=TaxonDetail&page=taxonDetail&taxon_oid=2516143052 |
|  | *Mycobacterium abscessus* 6G-0728-R | 2526164684 | https://img.jgi.doe.gov/cgi-bin/m/main.cgi?section=TaxonDetail&page=taxonDetail&taxon_oid=2526164684 |
|  | *Mycobacterium abscessus* 5S-0422 | 2526164589 | https://img.jgi.doe.gov/cgi-bin/m/main.cgi?section=TaxonDetail&page=taxonDetail&taxon_oid=2526164589 |
|  | *Mycobacterium abscessus* M152 | 2548877038 | https://img.jgi.doe.gov/cgi-bin/m/main.cgi?section=TaxonDetail&page=taxonDetail&taxon_oid=2548877038 |
|  | *Mycobacterium abscessus* 3A-0930-S | 2526164626 | https://img.jgi.doe.gov/cgi-bin/m/main.cgi?section=TaxonDetail&page=taxonDetail&taxon_oid=2526164626 |
|  | *Mycobacterium abscessus* MAB_091912_2455 | 2579779023 | https://img.jgi.doe.gov/cgi-bin/m/main.cgi?section=TaxonDetail&page=taxonDetail&taxon_oid=2579779023 |
|  | *Mycobacterium abscessus* 6G-0125-S | 2526164686 | https://img.jgi.doe.gov/cgi-bin/m/main.cgi?section=TaxonDetail&page=taxonDetail&taxon_oid=2526164686 |
|  | *Mycobacterium abscessus* 5S-1215 | 2526164680 | https://img.jgi.doe.gov/cgi-bin/m/main.cgi?section=TaxonDetail&page=taxonDetail&taxon_oid=2526164680 |
|  | *Mycobacterium abscessus bolletii* INCQS 00594 | 2597489897 | https://img.jgi.doe.gov/cgi-bin/m/main.cgi?section=TaxonDetail&page=taxonDetail&taxon_oid=2597489897 |
|  | *Mycobacterium abscessus*M139 | 2548877041 | https://img.jgi.doe.gov/cgi-bin/m/main.cgi?section=TaxonDetail&page=taxonDetail&taxon_oid=2548877041 |
|  | *Mycobacterium abscessus bolletii* Timone LB | 2579778727 | https://img.jgi.doe.gov/cgi-bin/m/main.cgi?section=TaxonDetail&page=taxonDetail&taxon_oid=2579778727 |
|  | *Mycobacterium abscessus* MAB_091912_2446 | 2574180273 | https://img.jgi.doe.gov/cgi-bin/m/main.cgi?section=TaxonDetail&page=taxonDetail&taxon_oid=2574180273 |
|  | *Mycobacterium abscessus*159 | 2548876927 | https://img.jgi.doe.gov/cgi-bin/m/main.cgi?section=TaxonDetail&page=taxonDetail&taxon_oid=2548876927 |
|  | *Mycobacterium abscessus* 3A-0810-R | 2531839203 | https://img.jgi.doe.gov/cgi-bin/m/main.cgi?section=TaxonDetail&page=taxonDetail&taxon_oid=2531839203 |
|  | *Mycobacterium abscessus* MAB_082312_2273 | 2585427958 | https://img.jgi.doe.gov/cgi-bin/m/main.cgi?section=TaxonDetail&page=taxonDetail&taxon_oid=2585427958 |
|  | *Mycobacteroides abscessus bolletii* 50594 | 2561511210 | https://img.jgi.doe.gov/cgi-bin/m/main.cgi?section=TaxonDetail&page=taxonDetail&taxon_oid=2561511210 |
|  | *Mycobacterium abscessus bolletii* CRM-0020 | 2582580873 | https://img.jgi.doe.gov/cgi-bin/m/main.cgi?section=TaxonDetail&page=taxonDetail&taxon_oid=2582580873 |
|  | *Mycobacterium abscessus* 3A-0119-R | 2526164622 | https://img.jgi.doe.gov/cgi-bin/m/main.cgi?section=TaxonDetail&page=taxonDetail&taxon_oid=2526164622 |
|  | *Mycobacterium abscessus* 5S-1212 | 2526164683 | https://img.jgi.doe.gov/cgi-bin/m/main.cgi?section=TaxonDetail&page=taxonDetail&taxon_oid=2526164683 |
|  | *Mycobacterium abscessus* 47J26 | 2519899837 | https://img.jgi.doe.gov/cgi-bin/m/main.cgi?section=TaxonDetail&page=taxonDetail&taxon_oid=2519899837 |
|  | *Mycobacterium abscessus* MAB_020201_1075 | 2568526042 | https://img.jgi.doe.gov/cgi-bin/m/main.cgi?section=TaxonDetail&page=taxonDetail&taxon_oid=2568526042 |
|  | *Mycobacterium abscessus bolletii* Timone LB | 2526164611 | https://img.jgi.doe.gov/cgi-bin/m/main.cgi?section=TaxonDetail&page=taxonDetail&taxon_oid=2526164611 |
|  | *Mycobacterium abscessus*3A-0930-R | 2526164625 | https://img.jgi.doe.gov/cgi-bin/m/main.cgi?section=TaxonDetail&page=taxonDetail&taxon_oid=2526164625 |
|  | *Mycobacterium abscessus* 4S-0303 | 2526164586 | https://img.jgi.doe.gov/cgi-bin/m/main.cgi?section=TaxonDetail&page=taxonDetail&taxon_oid=2526164586 |
|  | *Mycobacterium abscessus* M24 | 2548877036 | https://img.jgi.doe.gov/cgi-bin/m/main.cgi?section=TaxonDetail&page=taxonDetail&taxon_oid=2548877036 |
|  | *Mycobacterium abscessus* 103 | 2565956816 | https://img.jgi.doe.gov/cgi-bin/m/main.cgi?section=TaxonDetail&page=taxonDetail&taxon_oid=2565956816 |
|  | *Mycobacterium abscessus*115 | 2548876928 | https://img.jgi.doe.gov/cgi-bin/m/main.cgi?section=TaxonDetail&page=taxonDetail&taxon_oid=2548876928 |
|  | *Mycobacterium abscessus* CF | 2551306647 | https://img.jgi.doe.gov/cgi-bin/m/main.cgi?section=TaxonDetail&page=taxonDetail&taxon_oid=2551306647 |
|  | *Mycobacterium abscessus* 5S-0304 | 2526164591 | https://img.jgi.doe.gov/cgi-bin/m/main.cgi?section=TaxonDetail&page=taxonDetail&taxon_oid=2526164591 |
|  | *Mycobacterium abscessus* MAB_110811_2726 | 2568526391 | https://img.jgi.doe.gov/cgi-bin/m/main.cgi?section=TaxonDetail&page=taxonDetail&taxon_oid=2568526391 |
| ***Mycobacterium avium* complex (MAC)** | | | |
|  | *Mycobacterium avium* 2285 (R) | 2565956782 | https://img.jgi.doe.gov/cgi-bin/m/main.cgi?section=TaxonDetail&page=taxonDetail&taxon_oid=2565956782 |
|  | *Mycobacterium avium paratuberculosis* JQ6 | 2548876687 | https://img.jgi.doe.gov/cgi-bin/m/main.cgi?section=TaxonDetail&page=taxonDetail&taxon_oid=2548876687 |
|  | *Mycobacterium avium paratuberculosis* Pt155 | 2547132383 | https://img.jgi.doe.gov/cgi-bin/m/main.cgi?section=TaxonDetail&page=taxonDetail&taxon_oid=2547132383 |
|  | *Mycobacterium avium paratuberculosis* S5 | 2541047521 | https://img.jgi.doe.gov/cgi-bin/m/main.cgi?section=TaxonDetail&page=taxonDetail&taxon_oid=2541047521 |
|  | *Mycobacterium avium avium* 10-9275 | 2576861178 | https://img.jgi.doe.gov/cgi-bin/m/main.cgi?section=TaxonDetail&page=taxonDetail&taxon_oid=2576861178 |
|  | *Mycobacterium avium paratuberculosis* 08-8281 | 2574179921 | https://img.jgi.doe.gov/cgi-bin/m/main.cgi?section=TaxonDetail&page=taxonDetail&taxon_oid=2574179921 |
|  | *Mycobacterium avium paratuberculosis* 1281 | 2548876681 | https://img.jgi.doe.gov/cgi-bin/m/main.cgi?section=TaxonDetail&page=taxonDetail&taxon_oid=2548876681 |
|  | *Mycobacterium avium paratuberculosis* 10-5864 | 2568526703 | https://img.jgi.doe.gov/cgi-bin/m/main.cgi?section=TaxonDetail&page=taxonDetail&taxon_oid=2568526703 |
|  | *Mycobacterium avium paratuberculosis* 10-4404 | 2590828662 | https://img.jgi.doe.gov/cgi-bin/m/main.cgi?section=TaxonDetail&page=taxonDetail&taxon_oid=2590828662 |
|  | *Mycobacterium avium paratuberculosis* Pt144 | 2547132379 | https://img.jgi.doe.gov/cgi-bin/m/main.cgi?section=TaxonDetail&page=taxonDetail&taxon_oid=2547132379 |
|  | *Mycobacterium avium* 2151 | 651716619 | https://img.jgi.doe.gov/cgi-bin/m/main.cgi?section=TaxonDetail&page=taxonDetail&taxon_oid=651716619 |
|  | *Mycobacterium avium avium* 11-4751 | 2579778835 | https://img.jgi.doe.gov/cgi-bin/m/main.cgi?section=TaxonDetail&page=taxonDetail&taxon_oid=2579778835 |
|  | *Mycobacterium avium* 05-4293 | 2579779008 | https://img.jgi.doe.gov/cgi-bin/m/main.cgi?section=TaxonDetail&page=taxonDetail&taxon_oid=2579779008 |
|  | *Mycobacterium avium avium* Env 77 | 2548876686 | https://img.jgi.doe.gov/cgi-bin/m/main.cgi?section=TaxonDetail&page=taxonDetail&taxon_oid=2548876686 |
|  | *Mycobacterium avium paratuberculosis* Pt154 | 2547132382 | https://img.jgi.doe.gov/cgi-bin/m/main.cgi?section=TaxonDetail&page=taxonDetail&taxon_oid=2547132382 |
|  | *Mycobacterium avium* 2285 (S) | 2565956783 | https://img.jgi.doe.gov/cgi-bin/m/main.cgi?section=TaxonDetail&page=taxonDetail&taxon_oid=2565956783 |
|  | *Mycobacterium avium paratuberculosis* 4B | 2548877002 | https://img.jgi.doe.gov/cgi-bin/m/main.cgi?section=TaxonDetail&page=taxonDetail&taxon_oid=2548877002 |
|  | *Mycobacterium avium paratuberculosis* 10-5975 | 2574180289 | https://img.jgi.doe.gov/cgi-bin/m/main.cgi?section=TaxonDetail&page=taxonDetail&taxon_oid=2574180289 |
|  | *Mycobacterium avium* subsp. *avium A5* | 2579778738 | https://img.jgi.doe.gov/cgi-bin/m/main.cgi?section=TaxonDetail&page=taxonDetail&taxon_oid=2579778738 |
|  | *Mycobacterium avium* 10-5560 | 2574180263 | https://img.jgi.doe.gov/cgi-bin/m/main.cgi?section=TaxonDetail&page=taxonDetail&taxon_oid=2574180263 |
|  | *Mycobacterium avium silvaticum* ATCC 49884 | 2568526319 | https://img.jgi.doe.gov/cgi-bin/m/main.cgi?section=TaxonDetail&page=taxonDetail&taxon_oid=2568526319 |
|  | *Mycobacterium avium paratuberculosis* CLIJ644 | 2547132102 | https://img.jgi.doe.gov/cgi-bin/m/main.cgi?section=TaxonDetail&page=taxonDetail&taxon_oid=2547132102 |
|  | *Mycobacterium avium paratuberculosis* 10-8425 | 2590828661 | https://img.jgi.doe.gov/cgi-bin/m/main.cgi?section=TaxonDetail&page=taxonDetail&taxon_oid=2590828661 |
|  | *Mycobacterium avium paratuberculosis* JQ5 | 2548876680 | https://img.jgi.doe.gov/cgi-bin/m/main.cgi?section=TaxonDetail&page=taxonDetail&taxon_oid=2548876680 |
|  | *Mycobacterium avium hominissuis* 100 | 2579778785 | https://img.jgi.doe.gov/cgi-bin/m/main.cgi?section=TaxonDetail&page=taxonDetail&taxon_oid=2579778785 |
|  | *Mycobacterium avium* 10-5581 | 2571042511 | https://img.jgi.doe.gov/cgi-bin/m/main.cgi?section=TaxonDetail&page=taxonDetail&taxon_oid=2571042511 |
|  | *Mycobacterium avium paratuberculosis* 11-1786 | 2590828660 | https://img.jgi.doe.gov/cgi-bin/m/main.cgi?section=TaxonDetail&page=taxonDetail&taxon_oid=2590828660 |
|  | *Mycobacterium avium* MAV_061107_1842 | 2579779075 | https://img.jgi.doe.gov/cgi-bin/m/main.cgi?section=TaxonDetail&page=taxonDetail&taxon_oid=2579779075 |
|  | *Mycobacterium avium paratuberculosis* ATCC 19698 | 2548876531 | https://img.jgi.doe.gov/cgi-bin/m/main.cgi?section=TaxonDetail&page=taxonDetail&taxon_oid=2548876531 |
|  | *Mycobacterium avium* 11-0986 | 2582580886 | https://img.jgi.doe.gov/cgi-bin/m/main.cgi?section=TaxonDetail&page=taxonDetail&taxon_oid=2582580886 |
|  | *Mycobacterium avium* MAV_120709_2344 | 2579779122 | https://img.jgi.doe.gov/cgi-bin/m/main.cgi?section=TaxonDetail&page=taxonDetail&taxon_oid=2579779122 |
|  | *Mycobacterium avium paratuberculosis* Pt146 | 2547132381 | https://img.jgi.doe.gov/cgi-bin/m/main.cgi?section=TaxonDetail&page=taxonDetail&taxon_oid=2547132381 |
|  | *Mycobacterium avium hominissuis* MAH 2721 | 2571042795 | https://img.jgi.doe.gov/cgi-bin/m/main.cgi?section=TaxonDetail&page=taxonDetail&taxon_oid=2571042795 |
|  | *Mycobacterium avium paratuberculosis* Pt139 | 2547132378 | https://img.jgi.doe.gov/cgi-bin/m/main.cgi?section=TaxonDetail&page=taxonDetail&taxon_oid=2547132378 |
|  | *Mycobacterium avium paratuberculosis* DT 3 | 2548876683 | https://img.jgi.doe.gov/cgi-bin/m/main.cgi?section=TaxonDetail&page=taxonDetail&taxon_oid=2548876683 |
|  | *Mycobacterium avium avium* 3388 | 2579778886 | https://img.jgi.doe.gov/cgi-bin/m/main.cgi?section=TaxonDetail&page=taxonDetail&taxon_oid=2579778886 |
|  | *Mycobacterium avium avium* ATCC 25291 | 645058725 | https://img.jgi.doe.gov/cgi-bin/m/main.cgi?section=TaxonDetail&page=taxonDetail&taxon_oid=645058725 |
|  | Mycobacterium avium avium DT 78 | 2548876685 | https://img.jgi.doe.gov/cgi-bin/m/main.cgi?section=TaxonDetail&page=taxonDetail&taxon_oid=2548876685 |
|  | *Mycobacterium avium hominissuis* 10-4249 | 2579778680 | https://img.jgi.doe.gov/cgi-bin/m/main.cgi?section=TaxonDetail&page=taxonDetail&taxon_oid=2579778680 |
|  | *Mycobacterium avium hominissuis* MAH 27-1 | 2579778818 | https://img.jgi.doe.gov/cgi-bin/m/main.cgi?section=TaxonDetail&page=taxonDetail&taxon_oid=2579778818 |
|  | *Mycobacterium avium* 09-5983 | 2571042704 | https://img.jgi.doe.gov/cgi-bin/m/main.cgi?section=TaxonDetail&page=taxonDetail&taxon_oid=2571042704 |
|  | *Mycobacterium avium paratuberculosis* Pt145 | 2547132380 | https://img.jgi.doe.gov/cgi-bin/m/main.cgi?section=TaxonDetail&page=taxonDetail&taxon_oid=2547132380 |
|  | *Mycobacterium avium* MAV_120809_2495 | 2579778728 | https://img.jgi.doe.gov/cgi-bin/m/main.cgi?section=TaxonDetail&page=taxonDetail&taxon_oid=2579778728 |
|  | *Mycobacterium avium paratuberculosis* CLIJ623 | 2547132392 | https://img.jgi.doe.gov/cgi-bin/m/main.cgi?section=TaxonDetail&page=taxonDetail&taxon_oid=2547132392 |
|  | *Mycobacterium avium paratuberculosis* K-10 | 637000168 | https://img.jgi.doe.gov/cgi-bin/m/main.cgi?section=TaxonDetail&page=taxonDetail&taxon_oid=637000168 |
|  | *Mycobacterium avium hominissuis* 10-5606 | 2579779061 | https://img.jgi.doe.gov/cgi-bin/m/main.cgi?section=TaxonDetail&page=taxonDetail&taxon_oid=2579779061 |
|  | *Mycobacterium avium hominissuis* 101 | 2579778556 | https://img.jgi.doe.gov/cgi-bin/m/main.cgi?section=TaxonDetail&page=taxonDetail&taxon_oid=2579778556 |
|  | *Mycobacterium avium paratuberculosis* S397 | 2534681669 | https://img.jgi.doe.gov/cgi-bin/m/main.cgi?section=TaxonDetail&page=taxonDetail&taxon_oid=2534681669 |
|  | *Mycobacterium avium paratuberculosis* Env 210 | 2548876684 | https://img.jgi.doe.gov/cgi-bin/m/main.cgi?section=TaxonDetail&page=taxonDetail&taxon_oid=2548876684 |
|  | *Mycobacterium avium paratuberculosis* Pt164 | 2547132384 | https://img.jgi.doe.gov/cgi-bin/m/main.cgi?section=TaxonDetail&page=taxonDetail&taxon_oid=2547132384 |
|  | *Mycobacterium avium* 104 | 639633039 | https://img.jgi.doe.gov/cgi-bin/m/main.cgi?section=TaxonDetail&page=taxonDetail&taxon_oid=639633039 |
|  | *Mycobacterium avium paratuberculosis* MAP4 | 2554235361 | https://img.jgi.doe.gov/cgi-bin/m/main.cgi?section=TaxonDetail&page=taxonDetail&taxon_oid=2554235361 |
|  | *Mycobacterium avium paratuberculosis* CLIJ361 | 2547132397 | https://img.jgi.doe.gov/cgi-bin/m/main.cgi?section=TaxonDetail&page=taxonDetail&taxon_oid=2547132397 |
|  | *Mycobacterium avium paratuberculosis* JTC 1285 | 2548876682 | https://img.jgi.doe.gov/cgi-bin/m/main.cgi?section=TaxonDetail&page=taxonDetail&taxon_oid=2548876682 |
|  | *Mycobacterium* sp. MAC_011194_8550 | 2579779081 | https://img.jgi.doe.gov/cgi-bin/m/main.cgi?section=TaxonDetail&page=taxonDetail&taxon_oid=2579779081 |
|  | *Mycobacterium* sp. MAC_080597_8934 | 2576861029 | https://img.jgi.doe.gov/cgi-bin/m/main.cgi?section=TaxonDetail&page=taxonDetail&taxon_oid=2576861029 |
|  | *Mycobacterium* sp. UM_CSW | 2554235286 | https://img.jgi.doe.gov/cgi-bin/m/main.cgi?section=TaxonDetail&page=taxonDetail&taxon_oid=2554235286 |
| ***Non-tuberculosis Mycobacteria (NTM)*** | | | |
|  | *Mycobacterium ulcerans* Harvey | 2565956790 | https://img.jgi.doe.gov/cgi-bin/m/main.cgi?section=TaxonDetail&page=taxonDetail&taxon_oid=2565956790 |
|  | *Mycobacterium ulcerans* Agy99 | 642555140 | https://img.jgi.doe.gov/cgi-bin/m/main.cgi?section=TaxonDetail&page=taxonDetail&taxon_oid=642555140 |
|  | *Mycolicibacter sinense* JDM601 | 650716061 | https://img.jgi.doe.gov/cgi-bin/m/main.cgi?section=TaxonDetail&page=taxonDetail&taxon_oid=650716061 |
|  | *Mycobacterium marinum* Europe | 2541047508 | https://img.jgi.doe.gov/cgi-bin/m/main.cgi?section=TaxonDetail&page=taxonDetail&taxon_oid=2541047508 |
|  | *Mycobacterium marinum* M, ATCC BAA-535 | 641522642 | https://img.jgi.doe.gov/cgi-bin/m/main.cgi?section=TaxonDetail&page=taxonDetail&taxon_oid=641522642 |
|  | *Mycobacterium marinum* E11 | 2588253759 | https://img.jgi.doe.gov/cgi-bin/m/main.cgi?section=TaxonDetail&page=taxonDetail&taxon_oid=2588253759 |
|  | *Mycobacterium marinum* MB2 | 2545824679 | https://img.jgi.doe.gov/cgi-bin/m/main.cgi?section=TaxonDetail&page=taxonDetail&taxon_oid=2545824679 |
|  | *Mycobacterium liflandii* 128FXT | 2563366549 | https://img.jgi.doe.gov/cgi-bin/m/main.cgi?section=TaxonDetail&page=taxonDetail&taxon_oid=2563366549 |
|  | *Mycobacterium kansasii* 662 | 2565956794 | https://img.jgi.doe.gov/cgi-bin/m/main.cgi?section=TaxonDetail&page=taxonDetail&taxon_oid=2565956794 |
|  | *Mycobacterium kansasii* 824 | 2565956815 | https://img.jgi.doe.gov/cgi-bin/m/main.cgi?section=TaxonDetail&page=taxonDetail&taxon_oid=2565956815 |
|  | *Mycobacterium kansasii* SMC1 | 2585427992 | https://img.jgi.doe.gov/cgi-bin/m/main.cgi?section=TaxonDetail&page=taxonDetail&taxon_oid=2585427992 |
|  | *Mycobacterium kansasii* 732 | 2565956793 | https://img.jgi.doe.gov/cgi-bin/m/main.cgi?section=TaxonDetail&page=taxonDetail&taxon_oid=2565956793 |
|  | *Mycobacterium kansasii* ATCC 12478 | 2563366550 | https://img.jgi.doe.gov/cgi-bin/m/main.cgi?section=TaxonDetail&page=taxonDetail&taxon_oid=2563366550 |
|  | *Mycobacterium genavense* ATCC 51234 | 2545555864 | https://img.jgi.doe.gov/cgi-bin/m/main.cgi?section=TaxonDetail&page=taxonDetail&taxon_oid=2545555864 |

**Table S2.** Genome-wide data mining, identification, annotation and secondary metabolite BGC analysis of CYP139 P450s in mycobacterial species. A blank space indicates CYP139A P450s that were not found to be part of BGCs. Abbreviations: MAR/MAP, Methylated alkyl-resorcinol/methylated acyl-phloroglucinol; T1pks, Type 1 polyketide synthase; T2pks, Type 2 polyketide synthase.

| **Species name** | **CYP139A Protein ID** | **Cluster type** | **Reference BGC** | |
| --- | --- | --- | --- | --- |
|  |  |  | **% similarity** | **BGC name** |
| ***Mycobacterium tuberculosis* complex (MTBC)** | | | | |
| *Mycobacterium africanum* GM041182 | 651025167 | T3pks-T1pks | 19 | Lorneic acid A |
| *Mycobacterium africanum* K85 | 2584196432 | T3pks-T1pks | 33 | ML-449 |
| *Mycobacterium africanum* K85 | 646018681 | T3pks, T1pks | 100 | MAR/MAP |
| *Mycobacterium africanum* MAL010070 | 2583719805 | T3pks-T1pks | 33 | ML-449 |
| *Mycobacterium africanum* MAL010071 | 2582018155 | T3pks-T1pks | 33 | ML-449 |
| *Mycobacterium africanum* MAL010074 | 2582415442 | T3pks-T1pks | 33 | ML-449 |
| *Mycobacterium africanum* MAL010079 | 2580939152 | T3pks-T1pks | 33 | ML-449 |
| *Mycobacterium africanum* MAL010081 | 2580669105 | T3pks-T1pks | 10 | Abyssomicin |
| *Mycobacterium africanum* MAL010084 | 2581510874 | T3pks-T1pks | 19 | Lorneic acid A |
| *Mycobacterium africanum* MAL010099 | 2583723841 | T3pks-T1pks | 33 | ML-449 |
| *Mycobacterium africanum* MAL010100 | 2574968392 |  |  |  |
| *Mycobacterium africanum* MAL010102 | 2577175183 | T3pks-T1pks | 33 | ML-449 |
| *Mycobacterium africanum* MAL010111 | 2575938969 | T3pks-T1pks | 33 | ML-449 |
| *Mycobacterium africanum* MAL010112 | 2575198589 | T3pks-T1pks | 33 | ML-449 |
| *Mycobacterium africanum* MAL010118 | 2581869776 | T3pks-T1pks | 33 | ML-449 |
| *Mycobacterium africanum* MAL010120 | 2580366102 | T3pks-T1pks | 10 | Abyssomicin |
| *Mycobacterium africanum* MAL010123 | 2581807696 | T3pks-T1pks | 33 | ML-449 |
| *Mycobacterium africanum* MAL010128 | 2582001898 | T3pks-T1pks | 10 | Abyssomicin |
| *Mycobacterium africanum* MAL010129 | 2581901399 | T3pks-T1pks | 33 | ML-449 |
| *Mycobacterium africanum* MAL010131 | 2581377024 | T3pks-T1pks | 27 | Nystatin |
| *Mycobacterium africanum* MAL010136 | 2583727889 | T3pks-T1pks | 33 | ML-449 |
| *Mycobacterium africanum* MAL010137 | 2575705222 | T3pks-T1pks | 33 | ML-449 |
| *Mycobacterium africanum* MAL020107 | 2581930746 | T3pks-T1pks | 19 | Lorneic acid A |
| *Mycobacterium africanum* MAL020130 | 2583735989 | T3pks-T1pks | 33 | ML-449 |
| *Mycobacterium africanum* MAL020135 | 2583731958 |  |  |  |
| *Mycobacterium africanum* MAL020148 | 2581562358 | T3pks-T1pks | 19 | Lorneic acid A |
| *Mycobacterium africanum* MAL020173 | 2581366557 | T3pks-T1pks | 33 | ML-449 |
| *Mycobacterium africanum* MAL020176 | 2583740670 | T3pks-T1pks | 29 | Amphotericin |
| *Mycobacterium africanum* MAL020185 | 2580467795 | T3pks-T1pks | 50 | Spirangien |
| *Mycobacterium bovis* AF 2122/97 | 637139034 | T3pks-T1pks | 33 | ML-449 |
| *Mycobacterium bovis* B2 7505 | 2580301723 |  |  |  |
| *Mycobacterium bovis* BCG China | 2547306531 | T3pks-T1pks | 33 | ML-449 |
| *Mycobacterium bovis* BCG Korea 1168P | 2540803840 | T3pks-T1pks | 33 | ML-449 |
| *Mycobacterium bovis* BCG Mexico | 2511811274 | T3pks-T1pks | 33 | ML-449 |
| *Mycobacterium bovis* BCG Moreau RDJ | 2620699696 | T3pks, T1pks | 100 | MAR/MAP |
| *Mycobacterium bovis* BCG Pasteur 1173P2 | 639830617 | T3pks-T1pks | 33 | ML-449 |
| *Mycobacterium bovis* BCG str. Tokyo 172 | 643734506 | T3pks-T1pks | 33 | ML-449 |
| *Mycobacterium bovis* BCG-Denmark TMC 1010, ATCC 35733 | 2547311116 | T3pks-T1pks | 33 | ML-449 |
| *Mycobacterium bovis* BCG-Russia TMC 1022 , ATCC 35740 | 2547317188 | T3pks-T1pks | 31 | Stenothricin |
| *Mycobacterium bovis* BCG-Tice, TMC 1028 | 2547314995 | T3pks-T1pks | 38 | Oligomycin |
| *Mycobacterium bovis* D 4155 | 2580744928 | T3pks-T1pks | 20 | Kendomycin |
| *Mycobacterium bovis* Kc 32216 | 2584107430 |  |  |  |
| *Mycobacterium bovis* Kc 9614 | 2580123929 | T3pks-T1pks | 33 | ML-449 |
| *Mycobacterium bovis* MAL010093 | 2583745288 | T3pks-T1pks | 33 | ML-449 |
| *Mycobacterium bovis Wt* 21419 | 2581355094 | T3pks-T1pks | 33 | ML-449 |
| *Mycobacterium canettii* CIPT 140070002 | 2566980890 | T1pks | 32 | ECO-02301 |
| *Mycobacterium canettii* CIPT 140070007 | 2566972350 | T3pks-T1pks | 23 | Cyclizidine |
| *Mycobacterium canettii* CIPT 140070008 | 2541569776 | T3pks-T1pks | 50 | Spirangien |
| *Mycobacterium canettii* CIPT 140070010 | 2540554561 | T3pks | 21 | ECO-02301 |
| *Mycobacterium canettii* CIPT 140010059 | 651039004 | T3pks-T1pks | 20 | JBIR-100 |
| *Mycobacterium canettii* CIPT 140060008 | 2541578033 | T1pks | 18 | Natamycin |
| *Mycobacterium canettii* CIPT 140070005 | 2566985178 | T1pks | 12 | Apoptolidin |
| *Mycobacterium canettii* CIPT 140070013 | 2566976623 | T3pks-T1pks | 23 | Cyclizidine |
| *Mycobacterium canettii* CIPT 140070017 | 2541573920 | T3pks-T1pks | 32 | ECO-02301 |
| *Mycobacterium kansasii* 662 | 2567131988 | T3pks-T1pks | 33 | Mycolactone |
| *Mycobacterium tuberculosis* 02_1987 | 2577462548 | T3pks-T1pks | 33 | ML-449 |
| *Mycobacterium tuberculosis* 1010SM | 2584791771 | T3pks-T1pks | 33 | ML-449 |
| *Mycobacterium tuberculosis* 1173CS | 2576559538 | T3pks-T1pks | 33 | ML-449 |
| *Mycobacterium tuberculosis* 1615 | 2577954418 |  |  |  |
| *Mycobacterium tuberculosis* 16955 | 2575106637 | T3pks-T1pks | 33 | ML-449 |
| *Mycobacterium tuberculosis* 2091HD | 2584726832 | T3pks-T1pks | 41 | Piericidin A1 |
| *Mycobacterium tuberculosis* 2094HD | 2577143911 | T3pks-T1pks | 31 | Stenothricin |
| *Mycobacterium tuberculosis* 210 | 647209603 | T3pks-T1pks | 33 | ML-449 |
| *Mycobacterium tuberculosis* 2483AR | 2575162612 | T3pks-T1pks | 33 | ML-449 |
| *Mycobacterium tuberculosis* 2541MS | 2576373809 | T3pks-T1pks | 23 | Jerangolid |
| *Mycobacterium tuberculosis* 3280CJ | 2575655283 | T3pks-T1pks | 23 | Jerangolid |
| *Mycobacterium tuberculosis* 3499MM | 2584875064 | T3pks-T1pks | 23 | Jerangolid |
| *Mycobacterium tuberculosis* 44503 | 2584613792 |  |  |  |
| *Mycobacterium tuberculosis* 49375 | 2577429481 | T3pks-T1pks | 33 | ML-449 |
| *Mycobacterium tuberculosis* 51628 | 2584638962 |  |  |  |
| *Mycobacterium tuberculosis* 94_M4241A | 643019022 | T3pks-T1pks | 71 | Tylactone |
| *Mycobacterium tuberculosis* Beijing/NITR203 | 2546188127 | T3pks-T1pks | 33 | ML-449 |
| *Mycobacterium tuberculosis* BS1 | 2559163499 | T3pks-T1pks | 33 | ML-449 |
| *Mycobacterium tuberculosis* BT2 | 2566259019 | T3pks-T1pks | 33 | ML-449 |
| *Mycobacterium tuberculosis* BTB03-012 | 2577075963 | T3pks-T1pks | 33 | ML-449 |
| *Mycobacterium tuberculosis* BTB03-143 | 2576644886 | T3pks-T1pks | 33 | ML-449 |
| *Mycobacterium tuberculosis* BTB03-144 | 2576947708 | T3pks-T1pks | 23 | Jerangolid |
| *Mycobacterium tuberculosis* BTB04-452 | 2576009184 | T3pks-T1pks | 33 | ML-449 |
| *Mycobacterium tuberculosis* BTB05-013 | 2576564801 | T3pks-T1pks | 41 | Piericidin A1 |
| *Mycobacterium tuberculosis* BTB05-285 | 2577185436 | T3pks-T1pks | 20 | Kendomycin |
| *Mycobacterium tuberculosis* BTB05-552 | 2548033169 | T3pks-T1pks | 33 | ML-449 |
| *Mycobacterium tuberculosis* BTB05-559 | 2548037418 | T3pks-T1pks | 33 | ML-449 |
| *Mycobacterium tuberculosis* BTB06-001 | 2578107196 | T3pks-T1pks | 33 | ML-449 |
| *Mycobacterium tuberculosis* BTB07-034 | 2577454945 | T3pks-T1pks | 41 | Piericidin A1 |
| *Mycobacterium tuberculosis* BTB07-206 | 2574693694 | T3pks-T1pks | 33 | ML-449 |
| *Mycobacterium tuberculosis* BTB07-246 | 2584678567 | T3pks-T1pks | 33 | ML-449 |
| *Mycobacterium tuberculosis* BTB07-254 | 2584990570 | T3pks-T1pks | 41 | Piericidin A1 |
| *Mycobacterium tuberculosis* BTB07-325 | 2578099005 | T3pks-T1pks | 27 | Nystatin |
| *Mycobacterium tuberculosis* BTB08-022 | 2577659800 | T3pks-T1pks | 33 | ML-449 |
| *Mycobacterium tuberculosis* BTB08-148 | 2576609942 | T3pks-T1pks | 38 | Oligomycin |
| *Mycobacterium tuberculosis* BTB08-362 | 2575157076 | T3pks-T1pks | 33 | ML-449 |
| *Mycobacterium tuberculosis* BTB09-058 | 2578111269 | T3pks-T1pks | 12 | Tiacumicin B |
| *Mycobacterium tuberculosis* BTB09-565 | 2578062326 | T3pks-T1pks | 23 | Jerangolid |
| *Mycobacterium tuberculosis* BTB10-308 | 2576293562 | T3pks-T1pks | 41 | Piericidin A1 |
| *Mycobacterium tuberculosis* BTB10-357 | 2584631822 | T3pks-T1pks | 12 | Tiacumicin B |
| *Mycobacterium tuberculosis* BTB10-487 | 2576967330 | T3pks | 32 | ECO-02301 |
| *Mycobacterium tuberculosis* BTB11-001 | 2575520720 | T3pks-T1pks | 20 | Kendomycin |
| *Mycobacterium tuberculosis* BTB11-214 | 2576717872 | T3pks-T1pks | 33 | ML-449 |
| *Mycobacterium tuberculosis* BTB11-236 | 2578170996 | T3pks-T1pks | 41 | Piericidin A1 |
| *Mycobacterium tuberculosis* BTB11-343 | 2575647889 | T3pks-T1pks | 33 | ML-449 |
| *Mycobacterium tuberculosis* BTB12-001 | 2575016195 | T3pks-T1pks | 33 | ML-449 |
| *Mycobacterium tuberculosis* BTB12-206 | 2584890677 | T3pks-T1pks | 41 | Piericidin A1 |
| *Mycobacterium tuberculosis* BTB12-314 | 2577060340 | T3pks-T1pks | 33 | ML-449 |
| *Mycobacterium tuberculosis* BTB12-384 | 2584970624 | T3pks-T1pks | 27 | Neoaureothin |
| *Mycobacterium tuberculosis* BTB12-400 | 2576684060 | T3pks-T1pks | 36 | Neoaureothin |
| *Mycobacterium tuberculosis* BTB12-449 | 2577098384 | T3pks-T1pks | 20 | Tirandamycin |
| *Mycobacterium tuberculosis* BTB13-063 | 2576196505 | T3pks-T1pks | 33 | ML-449 |
| *Mycobacterium tuberculosis* BTB13-128 | 2574880930 | T3pks-T1pks | 33 | ML-449 |
| *Mycobacterium tuberculosis* BTB13-206 | 2584748012 | T3pks-T1pks | 33 | ML-449 |
| *Mycobacterium tuberculosis* BTB13-222 | 2576431117 | T3pks-T1pks | 33 | ML-449 |
| *Mycobacterium tuberculosis* C | 638726892 | T1pks | 7 | Abyssomicin |
| *Mycobacterium tuberculosis* CAS/NITR204 | 2546202077 |  |  |  |
| *Mycobacterium tuberculosis* CCDC5079 | 651084428 | T3pks-T1pks | 33 | ML-449 |
| *Mycobacterium tuberculosis* CCDC5180 | 651088108 | T3pks-T1pks | 33 | ML-449 |
| *Mycobacterium tuberculosis* CDC1551 | 637096038 | T3pks-T1pks | 33 | ML-449 |
| *Mycobacterium tuberculosis* CDC1551A | 2537735281 | T3pks-T1pks | 33 | ML-449 |
| *Mycobacterium tuberculosis* CPHL_A | 646014426 | T1pks | 10 | Abyssomicin |
| *Mycobacterium tuberculosis* CPHL_A | 2577093117 | T3pks-T1pks | 33 | ML-449 |
| *Mycobacterium tuberculosis* CTRI-2 | 2511736071 | T3pks-T1pks | 29 | ML-449 |
| *Mycobacterium tuberculosis* CTRI-4 | 2547880750 | T3pks-T1pks | 33 | ML-449 |
| *Mycobacterium tuberculosis* EAI/OSDD271 | 2554692349 | T1pks | 33 | JBIR-100 |
| *Mycobacterium tuberculosis* EAI5 | 2555960457 |  |  |  |
| *Mycobacterium tuberculosis* EAI5/NITR206 | 2546206123 | T3pks-T1pks | 19 | Jerangolid |
| *Mycobacterium tuberculosis* EAS054 | 643031783 |  |  |  |
| *Mycobacterium tuberculosis* Erdman | 2540619998 | T3pks-T1pks | 33 | ML-449 |
| *Mycobacterium tuberculosis* Erdman, ATCC 35801 | 2590317674 | T3pks, T1pks | 100 | MAR/MAP |
| *Mycobacterium tuberculosis* F11 (ExPEC) | 640606444 | T3pks-T1pks | 33 | ML-449 |
| *Mycobacterium tuberculosis* FJ05194 | 2545499027 | T3pks-T1pks | 33 | ML-449 |
| *Mycobacterium tuberculosis* G-12-005 | 2577281457 | T3pks-T1pks | 33 | ML-449 |
| *Mycobacterium tuberculosis* GM 1503 | 643045086 | T1pks | 16 | Elaiophylin |
| *Mycobacterium tuberculosis* GM 1503 | 2577893113 | T3pks-T1pks | 33 | ML-449 |
| *Mycobacterium tuberculosis* GuangZ0019 | 2546454904 | T3pks-T1pks | 33 | ML-449 |
| *Mycobacterium tuberculosis* H1578 | 2574790496 | T3pks-T1pks | 23 | Jerangolid |
| *Mycobacterium tuberculosis* H2398 | 2577684900 | T3pks-T1pks | 23 | Jerangolid |
| *Mycobacterium tuberculosis* H2438 | 2584962435 | T3pks-T1pks | 23 | Jerangolid |
| *Mycobacterium tuberculosis* H2581 | 2576392640 | T3pks-T1pks | 33 | ML-449 |
| *Mycobacterium tuberculosis* H3361 | 2575023271 | T3pks-T1pks | 23 | Jerangolid |
| *Mycobacterium tuberculosis* H37Ra | 641814886 | T3pks-T1pks | 30 | Nanchangmycin |
| *Mycobacterium tuberculosis* H37Ra | 640602381 | T3pks-T1pks | 33 | ML-449 |
| *Mycobacterium tuberculosis* H37Rv | 637026884 (Rv1666c) | T3pks-T1pks | 33 | ML-449 |
| *Mycobacterium tuberculosis* H37Rv | 2527056892 | T3pks-T1pks | 33 | ML-449 |
| *Mycobacterium tuberculosis* H37RvCO | 2547164190 | T3pks-T1pks | 33 | ML-449 |
| *Mycobacterium tuberculosis* Haarlem | 641783198 | T3pks, T1pks | 100 | MAR/MAP |
| *Mycobacterium tuberculosis* Haarlem | 2590313607 | T3pks, T1pks | 100 | MAR/MAP |
| *Mycobacterium tuberculosis* Haarlem3/NITR202 | 2546192085 | T3pks | 62 | FD-891 |
| *Mycobacterium tuberculosis* HN878 | 2547959756 | T3pks-T1pks | 33 | ML-449 |
| *Mycobacterium tuberculosis* INS_XDR | 2573562450 |  |  |  |
| *Mycobacterium tuberculosis* K | 2588538927 | T3pks-T1pks | 33 | ML-449 |
| *Mycobacterium tuberculosis* Korean KIT87190 | 2588591267 | T3pks-T1pks | 33 | ML-449 |
| *Mycobacterium tuberculosis* KT-0001 | 2578084434 | T3pks-T1pks | 33 | ML-449 |
| *Mycobacterium tuberculosis* KT-0002 | 2590294228 | T3pks, T1pks | 100 | MAR/MAP |
| *Mycobacterium tuberculosis* KT-0003 | 2576199760 | T3pks-T1pks | 33 | ML-449 |
| *Mycobacterium tuberculosis* KT-0004 | 2575255926 | T3pks-T1pks | 50 | Spirangien |
| *Mycobacterium tuberculosis* KT-0006 | 2590288661 | T3pks, T1pks | 100 | MAR/MAP |
| *Mycobacterium tuberculosis* KT-0007 | 2590283895 | T3pks-T1pks | 27 | Nystatin |
| *Mycobacterium tuberculosis* KT-0008 | 2590279314 | T3pks-T1pks | 33 | ML-449 |
| *Mycobacterium tuberculosis* KT-0011 | 2584911080 | T3pks-T1pks | 33 | ML-449 |
| *Mycobacterium tuberculosis* KT-0014 | 2590276448 | T3pks, T1pks | 100 | MAR/MAP |
| *Mycobacterium tuberculosis* KT-0015 | 2590272661 | T3pks, T1pks | 100 | MAR/MAP |
| *Mycobacterium tuberculosis* KT-0016 | 2590266966 | T3pks-T1pks | 33 | ML-449 |
| *Mycobacterium tuberculosis* KT-0019 | 2584846770 | T3pks-T1pks | 33 | ML-449 |
| *Mycobacterium tuberculosis* KT-0022 | 2590260165 | T3pks-T1pks | 33 | ML-449 |
| *Mycobacterium tuberculosis* KT-0023 | 2590256393 | T3pks, T1pks | 100 | MAR/MAP |
| *Mycobacterium tuberculosis* KT-0024 | 2577110129 | T3pks-T1pks | 33 | ML-449 |
| *Mycobacterium tuberculosis* KT-0026 | 2590252317 | T3pks, T1pks | 100 | MAR/MAP |
| *Mycobacterium tuberculosis* KT-0027 | 2577997123 |  |  |  |
| *Mycobacterium tuberculosis* KT-0028 | 2590249475 | T3pks-T1pks | 27 | Nystatin |
| *Mycobacterium tuberculosis* KT-0034 | 2575869049 | T3pks-T1pks | 33 | ML-449 |
| *Mycobacterium tuberculosis* KT-0035 | 2590243884 | T3pks-T1pks | 29 | ML-449 |
| *Mycobacterium tuberculosis* KT-0037 | 2576522568 | T3pks-T1pks | 33 | ML-449 |
| *Mycobacterium tuberculosis* KT-0039 | 2590240089 | T3pks, T1pks | 100 | MAR/MAP |
| *Mycobacterium tuberculosis* KT-0040 | 2575964273 | T3pks-T1pks | 33 | ML-449 |
| *Mycobacterium tuberculosis* KT-0041 | 2584681396 | T3pks-T1pks | 33 | ML-449 |
| *Mycobacterium tuberculosis* KT-0042 | 2574949227 | T3pks-T1pks | 33 | ML-449 |
| *Mycobacterium tuberculosis* KT-0043 | 2590237321 | T3pks-T1pks | 33 | ML-449 |
| *Mycobacterium tuberculosis* KT-0045 | 2590231653 | T3pks-T1pks | 33 | ML-449 |
| *Mycobacterium tuberculosis* KT-0047 | 2590227570 | T3pks, T1pks | 100 | MAR/MAP |
| *Mycobacterium tuberculosis* KT-0048 | 2590223019 | T3pks-T1pks | 32 | ECO-02301 |
| *Mycobacterium tuberculosis* KT-0051 | 2575094331 | T3pks-T1pks | 33 | ML-449 |
| *Mycobacterium tuberculosis* KT-0053 | 2590219901 | T3pks, T1pks | 100 | MAR/MAP |
| *Mycobacterium tuberculosis* KT-0056 | 2576712596 | T3pks-T1pks | 27 | Neoaureothin |
| *Mycobacterium tuberculosis* KT-0057 | 2584721998 | T3pks-T1pks | 33 | ML-449 |
| *Mycobacterium tuberculosis* KT-0058 | 2590214646 | T3pks-T1pks | 27 | Nystatin |
| *Mycobacterium tuberculosis* KT-0063 | 2590211264 | T3pks-T1pks | 33 | ML-449 |
| *Mycobacterium tuberculosis* KT-0064 | 2590563570 | T3pks, T1pks | 100 | MAR/MAP |
| *Mycobacterium tuberculosis* KT-0067 | 2574703562 | T3pks-T1pks | 33 | ML-449 |
| *Mycobacterium tuberculosis* KT-0069 | 2574911407 | T3pks-T1pks | 33 | ML-449 |
| *Mycobacterium tuberculosis* KT-0070 | 2577655387 | T3pks-T1pks | 33 | ML-449 |
| *Mycobacterium tuberculosis* KT-0071 | 2590559932 | T3pks-T1pks | 16 | Akaeolide |
| *Mycobacterium tuberculosis* KT-0072 | 2576101264 | T3pks-T1pks | 33 | ML-449 |
| *Mycobacterium tuberculosis* KT-0075 | 2590554696 | T3pks, T1pks | 100 | MAR/MAP |
| *Mycobacterium tuberculosis* KT-0077 | 2590552289 | T3pks-T1pks | 27 | Nystatin |
| *Mycobacterium tuberculosis* KT-0078 | 2590548005 | T3pks, T1pks | 100 | MAR/MAP |
| *Mycobacterium tuberculosis* KT-0079 | 2590543931 | T3pks, T1pks | 100 | MAR/MAP |
| *Mycobacterium tuberculosis* KT-0080 | 2590539845 | T3pks-T1pks | 33 | ML-449 |
| *Mycobacterium tuberculosis* KT-0083 | 2590535240 | T3pks, T1pks | 100 | MAR/MAP |
| *Mycobacterium tuberculosis* KT-0084 | 2590531681 | T3pks-T1pks | 33 | ML-449 |
| *Mycobacterium tuberculosis* KT-0085 | 2590526411 | T3pks-T1pks | 33 | ML-449 |
| *Mycobacterium tuberculosis* KT-0086 | 2574780327 | T3pks-T1pks | 33 | ML-449 |
| *Mycobacterium tuberculosis* KT-0087 | 2574834868 | T3pks-T1pks | 33 | ML-449 |
| *Mycobacterium tuberculosis* KT-0089 | 2590523531 | T3pks-T1pks | 32 | ECO-02301 |
| *Mycobacterium tuberculosis* KT-0091 | 2575468742 | T3pks-T1pks | 27 | Nystatin |
| *Mycobacterium tuberculosis* KT-0092 | 2590519464 | T3pks, T1pks | 100 | MAR/MAP |
| *Mycobacterium tuberculosis* KT-0094 | 2590513765 | T3pks, T1pks | 100 | MAR/MAP |
| *Mycobacterium tuberculosis* KT-0096 | 2578189802 | T3pks-T1pks | 50 | Piericidin A1 |
| *Mycobacterium tuberculosis* KT-0098 | 2584878425 | T3pks-T1pks | 33 | ML-449 |
| *Mycobacterium tuberculosis* KT-0099 | 2590510255 | T3pks, T1pks | 100 | MAR/MAP |
| *Mycobacterium tuberculosis* KT-0100 | 2577733463 | T3pks-T1pks | 33 | ML-449 |
| *Mycobacterium tuberculosis* KT-0102 | 2590505688 | T3pks-T1pks | 33 | ML-449 |
| *Mycobacterium tuberculosis* KT-0104 | 2590501611 | T3pks-T1pks | 33 | ML-449 |
| *Mycobacterium tuberculosis* KT-0106 | 2590499050 | T3pks, T1pks | 100 | MAR/MAP |
| *Mycobacterium tuberculosis* KT-0107 | 2590493738 | T3pks, T1pks | 100 | MAR/MAP |
| *Mycobacterium tuberculosis* KT-0108 | 2575418829 | T3pks-T1pks | 50 | Spirangien |
| *Mycobacterium tuberculosis* KT-0109 | 2584998642 | T3pks-T1pks | 27 | Nystatin |
| *Mycobacterium tuberculosis* KT-0110 | 2576250927 | T3pks-T1pks | 33 | ML-449 |
| *Mycobacterium tuberculosis* KZN 1435 (MDR) | 644880084 | T3pks-T1pks | 32 | ECO-02301 |
| *Mycobacterium tuberculosis* KZN 4207 | 647086307 | T3pks-T1pks | 33 | ML-449 |
| *Mycobacterium tuberculosis* KZN 4207 (DS) | 2511553315 | T3pks-T1pks | 32 | ECO-02301 |
| *Mycobacterium tuberculosis* KZN 605 (XDR) | 645120373 | T3pks-T1pks | 11 | Borrelidin |
| *Mycobacterium tuberculosis* KZN R506 | 648335985 | T3pks-T1pks | 33 | ML-449 |
| *Mycobacterium tuberculosis* KZN V2475 | 647090515 | T3pks-T1pks | 33 | ML-449 |
| *Mycobacterium tuberculosis* M1004 | 2577269438 | T3pks-T1pks | 23 | Jerangolid |
| *Mycobacterium tuberculosis* M1007 | 2576553939 | T3pks-T1pks | 23 | Jerangolid |
| *Mycobacterium tuberculosis* M1008 | 2576566954 | T3pks-T1pks | 23 | Jerangolid |
| *Mycobacterium tuberculosis* M1017 | 2577884547 |  |  |  |
| *Mycobacterium tuberculosis* M1025 | 2578155745 | T3pks-T1pks | 23 | Jerangolid |
| *Mycobacterium tuberculosis* M1213 | 2575561335 | T3pks-T1pks | 23 | Jerangolid |
| *Mycobacterium tuberculosis* M1221 | 2575515927 | T3pks-T1pks | 23 | Jerangolid |
| *Mycobacterium tuberculosis* M1233 | 2575663406 | T3pks-T1pks | 23 | Jerangolid |
| *Mycobacterium tuberculosis* M1283 | 2577803488 | T3pks-T1pks | 23 | Jerangolid |
| *Mycobacterium tuberculosis* M1340 | 2584623655 | T3pks-T1pks | 23 | Jerangolid |
| *Mycobacterium tuberculosis* M1415 | 2584983051 | T3pks-T1pks | 33 | JBIR-100 |
| *Mycobacterium tuberculosis* M1418 | 2577009596 |  |  |  |
| *Mycobacterium tuberculosis* M1438 | 2574663269 | T3pks-T1pks | 23 | Jerangolid |
| *Mycobacterium tuberculosis* M1444 | 2576015739 | T3pks-T1pks | 23 | Jerangolid |
| *Mycobacterium tuberculosis* M1449 | 2575361778 | T3pks-T1pks | 23 | Jerangolid |
| *Mycobacterium tuberculosis* M1475 | 2584769363 | T3pks-T1pks | 23 | Jerangolid |
| *Mycobacterium tuberculosis* M1481 | 2575252379 | T3pks-T1pks | 23 | Jerangolid |
| *Mycobacterium tuberculosis* M1559 | 2575794202 | T3pks-T1pks | 13 | Lasalocid |
| *Mycobacterium tuberculosis* M1700 | 2584913651 | T3pks-T1pks | 33 | ML-449 |
| *Mycobacterium tuberculosis* M1703 | 2584732271 | T3pks-T1pks | 33 | ML-449 |
| *Mycobacterium tuberculosis* M1762 | 2574901332 | T3pks-T1pks | 20 | Kendomycin |
| *Mycobacterium tuberculosis* M1787 | 2579818167 | T3pks-T1pks | 20 | Kendomycin |
| *Mycobacterium tuberculosis* M1848 | 2574773054 | T3pks-T1pks | 33 | ML-449 |
| *Mycobacterium tuberculosis* M1906 | 2576123248 | T3pks-T1pks | 23 | Jerangolid |
| *Mycobacterium tuberculosis* M1913 | 2576927477 | T3pks-T1pks | 20 | Kendomycin |
| *Mycobacterium tuberculosis* M1956 | 2584633235 | T3pks-T1pks | 33 | ML-449 |
| *Mycobacterium tuberculosis* M1961 | 2584689961 | T3pks-T1pks | 23 | Jerangolid |
| *Mycobacterium tuberculosis* M1978 | 2578086064 | T3pks-T1pks | 23 | Jerangolid |
| *Mycobacterium tuberculosis* M2006 | 2577302440 | T3pks-T1pks | 20 | Kendomycin |
| *Mycobacterium tuberculosis* M2085 | 2577905307 | T3pks-T1pks | 33 | ML-449 |
| *Mycobacterium tuberculosis* M2113 | 2577069926 | T3pks-T1pks | 23 | Jerangolid |
| *Mycobacterium tuberculosis* M2116 | 2578033540 | T3pks-T1pks | 23 | Jerangolid |
| *Mycobacterium tuberculosis* M2128 | 2577613318 | T3pks-T1pks | 33 | Jerangolid |
| *Mycobacterium tuberculosis* M2129 | 2584995097 | T3pks-T1pks | 23 | Jerangolid |
| *Mycobacterium tuberculosis* M2131 | 2576731741 | T3pks-T1pks | 33 | ML-449 |
| *Mycobacterium tuberculosis* M2136 | 2575984510 | T3pks-T1pks | 23 | Jerangolid |
| *Mycobacterium tuberculosis* M2137 | 2577497204 | T3pks-T1pks | 23 | Jerangolid |
| *Mycobacterium tuberculosis* M2203 | 2584649146 | T3pks-T1pks | 23 | Jerangolid |
| *Mycobacterium tuberculosis* M2248 | 2577218717 | T3pks-T1pks | 20 | Kendomycin |
| *Mycobacterium tuberculosis* M2249 | 2577918576 | T3pks-T1pks | 20 | Kendomycin |
| *Mycobacterium tuberculosis* M2343 | 2575345775 | T3pks-T1pks | 14 | Ambruticin |
| *Mycobacterium tuberculosis* M2346 | 2584928246 | T3pks-T1pks | 23 | Jerangolid |
| *Mycobacterium tuberculosis* M2402 | 2574854728 |  |  |  |
| *Mycobacterium tuberculosis* M2416 | 2574830606 | T3pks-T1pks | 23 | Jerangolid |
| *Mycobacterium tuberculosis* M2479 | 2577720788 | T3pks-T1pks | 20 | Kendomycin |
| *Mycobacterium tuberculosis* M2508 | 2577236838 | T3pks-T1pks | 20 | Kendomycin |
| *Mycobacterium tuberculosis* M995 | 2579808474 | T3pks-T1pks | 33 | JBIR-100 |
| *Mycobacterium tuberculosis* MAL010080 | 2590014899 | T3pks, T1pks | 100 | MAR/MAP |
| *Mycobacterium tuberculosis* MAL010086 | 2590012031 | T3pks, T1pks | 100 | MAR/MAP |
| *Mycobacterium tuberculosis* MAL010087 | 2590007914 | T3pks, T1pks | 100 | MAR/MAP |
| *Mycobacterium tuberculosis* MAL010088 | 2590025444 | T3pks-T1pks | 33 | ML-449 |
| *Mycobacterium tuberculosis* MAL010103 | 2576151818 | T3pks-T1pks | 27 | Nystatin |
| *Mycobacterium tuberculosis* MAL010105 | 2590040558 | T3pks-T1pks | 33 | ML-449 |
| *Mycobacterium tuberculosis* MAL010108 | 2590032388 | T3pks-T1pks | 29 | ML-449 |
| *Mycobacterium tuberculosis* MAL010109 | 2576459372 | T3pks-T1pks | 33 | ML-449 |
| *Mycobacterium tuberculosis* MAL010110 | 2590028529 | T3pks, T1pks | 100 | MAR/MAP |
| *Mycobacterium tuberculosis* MAL010117 | 2590044637 | T3pks, T1pks | 100 | MAR/MAP |
| *Mycobacterium tuberculosis* MAL010124 | 2590048710 | T3pks-T1pks | 33 | ML-449 |
| *Mycobacterium tuberculosis* MAL010130 | 2590052832 | T3pks-T1pks | 27 | Nystatin |
| *Mycobacterium tuberculosis* MAL010133 | 2575280304 | T3pks-T1pks | 33 | ML-449 |
| *Mycobacterium tuberculosis* MAL010134 | 2590075971 | T3pks-T1pks | 33 | JBIR-100 |
| *Mycobacterium tuberculosis* MAL020102 | 2590073317 | T3pks-T1pks | 27 | Nystatin |
| *Mycobacterium tuberculosis* MAL020110 | 2590070071 | T3pks, T1pks | 100 | MAR/MAP |
| *Mycobacterium tuberculosis* MAL020120 | 2577195360 | T3pks-T1pks | 33 | ML-449 |
| *Mycobacterium tuberculosis* MAL020131 | 2574754194 | T3pks-T1pks | 27 | Nystatin |
| *Mycobacterium tuberculosis* MAL020132 | 2590064965 | T3pks, T1pks | 100 | MAR/MAP |
| *Mycobacterium tuberculosis* MAL020136 | 2590061112 | T3pks, T1pks | 100 | MAR/MAP |
| *Mycobacterium tuberculosis* MAL020138 | 2590057066 | T3pks, T1pks | 100 | MAR/MAP |
| *Mycobacterium tuberculosis* MAL020141 | 2590089378 | T3pks, T1pks | 100 | MAR/MAP |
| *Mycobacterium tuberculosis* MAL020142 | 2590093450 | T3pks, T1pks | 100 | MAR/MAP |
| *Mycobacterium tuberculosis* MAL020144 | 2576703024 | T3pks-T1pks | 33 | ML-449 |
| *Mycobacterium tuberculosis* MAL020145 | 2584763258 | T3pks-T1pks | 33 | ML-449 |
| *Mycobacterium tuberculosis* MAL020147 | 2590101605 | T3pks, T1pks | 100 | MAR/MAP |
| *Mycobacterium tuberculosis* MAL020150 | 2590104693 | T3pks, T1pks | 100 | MAR/MAP |
| *Mycobacterium tuberculosis* MAL020152 | 2590109466 | T3pks-T1pks | 33 | ML-449 |
| *Mycobacterium tuberculosis* MAL020156 | 2590113796 | T3pks-T1pks | 33 | ML-449 |
| *Mycobacterium tuberculosis* MAL020157 | 2590117900 | T3pks-T1pks | 33 | ML-449 |
| *Mycobacterium tuberculosis* MAL020160 | 2590081259 | T3pks, T1pks | 100 | MAR/MAP |
| *Mycobacterium tuberculosis* MAL020162 | 2575533703 | T3pks-T1pks | 27 | Nystatin |
| *Mycobacterium tuberculosis* MAL020167 | 2590086658 | T3pks, T1pks | 100 | MAR/MAP |
| *Mycobacterium tuberculosis* MAL020172 | 2590125975 | T3pks, T1pks | 100 | MAR/MAP |
| *Mycobacterium tuberculosis* MAL020174 | 2590121989 | T3pks, T1pks | 100 | MAR/MAP |
| *Mycobacterium tuberculosis* MAL020179 | 2576601719 | T3pks-T1pks | 33 | ML-449 |
| *Mycobacterium tuberculosis* MAL020181 | 2574682482 | T3pks-T1pks | 33 | ML-449 |
| *Mycobacterium tuberculosis* MAL020186 | 2590134192 | T3pks-T1pks | 33 | ML-449 |
| *Mycobacterium tuberculosis* MAL020187 | 2590154524 | T3pks-T1pks | 33 | ML-449 |
| *Mycobacterium tuberculosis* MAL020192 | 2590142346 | T3pks-T1pks | 38 | Oligomycin |
| *Mycobacterium tuberculosis* MAL020193 | 2590146409 | T3pks, T1pks | 100 | MAR/MAP |
| *Mycobacterium tuberculosis* MAL020194 | 2590150448 | T3pks-T1pks | 33 | ML-449 |
| *Mycobacterium tuberculosis* MAL020195 | 2590137275 | T3pks, T1pks | 100 | MAR/MAP |
| *Mycobacterium tuberculosis* MAL020196 | 2590162679 | T3pks-T1pks | 33 | ML-449 |
| *Mycobacterium tuberculosis* MAL020197 | 2590158810 | T3pks, T1pks | 100 | MAR/MAP |
| *Mycobacterium tuberculosis* MAL020199 | 2590166763 | T3pks-T1pks | 33 | ML-449 |
| *Mycobacterium tuberculosis* MAL020200 | 2590169218 | T3pks, T1pks | 100 | MAR/MAP |
| *Mycobacterium tuberculosis* MAL020201 | 2590174897 | T3pks-T1pks | 33 | ML-449 |
| *Mycobacterium tuberculosis* MAL020205 | 2590181544 | T3pks, T1pks | 100 | MAR/MAP |
| *Mycobacterium tuberculosis* MAL020206 | 2590187116 | T3pks, T1pks | 100 | MAR/MAP |
| *Mycobacterium tuberculosis* MAL020208 | 2590190898 | T3pks-T1pks | 33 | ML-449 |
| *Mycobacterium tuberculosis* MAL020209 | 2590195268 | T3pks-T1pks | 33 | ML-449 |
| *Mycobacterium tuberculosis* MAL020211 | 2590198349 | T3pks-T1pks | 27 | Nystatin |
| *Mycobacterium tuberculosis* MD13878 | 2575051511 | T3pks-T1pks | 33 | ML-449 |
| *Mycobacterium tuberculosis* MD14435 | 2577821955 | T3pks-T1pks | 33 | ML-449 |
| *Mycobacterium tuberculosis* MD15050 | 2584946269 | T3pks-T1pks | 33 | ML-449 |
| *Mycobacterium tuberculosis* MD15212 | 2577519304 | T3pks-T1pks | 33 | ML-449 |
| *Mycobacterium tuberculosis* MD15597 | 2584949783 | T3pks-T1pks | 33 | ML-449 |
| *Mycobacterium tuberculosis* MD15855 | 2577448440 | T3pks-T1pks | 33 | ML-449 |
| *Mycobacterium tuberculosis* MD15956 | 2584942354 | T3pks-T1pks | 41 | Piericidin A1 |
| *Mycobacterium tuberculosis* MD15974 | 2577198903 | T3pks-T1pks | 41 | Piericidin A1 |
| *Mycobacterium tuberculosis* MD15977 | 2574560432 | T3pks-T1pks | 33 | ML-449 |
| *Mycobacterium tuberculosis* MD16265 | 2577422627 | T3pks-T1pks | 41 | Piericidin A1 |
| *Mycobacterium tuberculosis* MD16277 | 2576172216 | T3pks-T1pks | 41 | Piericidin A1 |
| *Mycobacterium tuberculosis* MD16553 | 2574586310 | T3pks-T1pks | 23 | Jerangolid |
| *Mycobacterium tuberculosis* MD16555 | 2584870715 | T3pks-T1pks | 33 | ML-449 |
| *Mycobacterium tuberculosis* MD16577 | 2584931720 | T3pks-T1pks | 33 | ML-449 |
| *Mycobacterium tuberculosis* MD16728 | 2577697067 | T3pks-T1pks | 41 | Piericidin A1 |
| *Mycobacterium tuberculosis* MD16775 | 2575988382 | T3pks-T1pks | 41 | Piericidin A1 |
| *Mycobacterium tuberculosis* MD17517 | 2577632297 | T3pks-T1pks | 41 | Piericidin A1 |
| *Mycobacterium tuberculosis* MD17615 | 2584812089 | T3pks-T1pks | 41 | Piericidin A1 |
| *Mycobacterium tuberculosis* MD17888 | 2575084753 | T3pks-T1pks | 41 | Piericidin A1 |
| *Mycobacterium tuberculosis* MD17902 | 2575138339 | T3pks-T1pks | 41 | Piericidin A1 |
| *Mycobacterium tuberculosis* MD18096 | 2576274603 | T3pks-T1pks | 33 | ML-449 |
| *Mycobacterium tuberculosis* MD18478 | 2577879644 | T3pks-T1pks | 41 | Piericidin A1 |
| *Mycobacterium tuberculosis* MD18498 | 2574987296 | T3pks-T1pks | 41 | Piericidin A1 |
| *Mycobacterium tuberculosis* MD19043 | 2574761421 | T3pks-T1pks | 33 | ML-449 |
| *Mycobacterium tuberculosis* MD19964 | 2575185207 | T3pks-T1pks | 33 | ML-449 |
| *Mycobacterium tuberculosis* MTB-476 | 2573574061 | T3pks-T1pks | 27 | Nystatin |
| *Mycobacterium tuberculosis* NA-A0009 | 2551812688 | T3pks-T1pks | 100 | Leucanicidin |
| *Mycobacterium tuberculosis* NCGM2209 | 2549401785 | T3pks-T1pks | 33 | ML-449 |
| *Mycobacterium tuberculosis* NRITLD09 | 2584831832 |  |  |  |
| *Mycobacterium tuberculosis* NRITLD14 | 2577923998 | T3pks-T1pks | 23 | Jerangolid |
| *Mycobacterium tuberculosis* NRITLD15 | 2578094092 | T3pks-T1pks | 33 | ML-449 |
| *Mycobacterium tuberculosis* NRITLD44 | 2575709449 |  |  |  |
| *Mycobacterium tuberculosis* NRITLD56 | 2577468911 | T3pks-T1pks | 33 | ML-449 |
| *Mycobacterium tuberculosis* OFXR-1 | 2590207004 | T3pks, T1pks | 100 | MAR/MAP |
| *Mycobacterium tuberculosis* OFXR-10 | 2578230674 | T3pks-T1pks | 27 | Nystatin |
| *Mycobacterium tuberculosis* OFXR-11 | 2589154518 | T3pks-T1pks | 27 | Nystatin |
| *Mycobacterium tuberculosis* OFXR-12 | 2589158600 | T3pks-T1pks | 27 | Nystatin |
| *Mycobacterium tuberculosis* OFXR-13 | 2576534922 | T3pks-T1pks | 27 | Nystatin |
| *Mycobacterium tuberculosis* OFXR-14 | 2589146274 | T3pks-T1pks | 27 | Nystatin |
| *Mycobacterium tuberculosis* OFXR-15 | 2576477081 | T3pks-T1pks | 33 | ML-449 |
| *Mycobacterium tuberculosis* OFXR-16 | 2589165303 | T3pks-T1pks | 33 | ML-449 |
| *Mycobacterium tuberculosis* OFXR-18 | 2590382529 | T3pks, T1pks | 100 | MAR/MAP |
| *Mycobacterium tuberculosis* OFXR-2 | 2589142105 | T3pks-T1pks | 27 | Nystatin |
| *Mycobacterium tuberculosis* OFXR-20 | 2590377218 | T3pks-T1pks | 33 | ML-449 |
| *Mycobacterium tuberculosis* OFXR-21 | 2590374347 | T3pks-T1pks | 33 | ML-449 |
| *Mycobacterium tuberculosis* OFXR-22 | 2590370532 | T3pks-T1pks | 33 | ML-449 |
| *Mycobacterium tuberculosis* OFXR-23 | 2590366449 |  |  |  |
| *Mycobacterium tuberculosis* OFXR-29 | 2590356674 | T3pks-T1pks | 27 | Nystatin |
| *Mycobacterium tuberculosis* OFXR-3 | 2576471218 | T3pks-T1pks | 27 | Nystatin |
| *Mycobacterium tuberculosis* OFXR-30 | 2590354422 | T3pks, T1pks | 100 | MAR/MAP |
| *Mycobacterium tuberculosis* OFXR-31 | 2590350138 | T3pks, T1pks | 100 | MAR/MAP |
| *Mycobacterium tuberculosis* OFXR-32 | 2590345757 | T3pks, T1pks | 100 | MAR/MAP |
| *Mycobacterium tuberculosis* OFXR-33 | 2577373328 | T3pks-T1pks | 33 | ML-449 |
| *Mycobacterium tuberculosis* OFXR-4 | 2589161189 | T3pks-T1pks | 30 | Nanchangmycin |
| *Mycobacterium tuberculosis* OFXR-5 | 2589148607 | T3pks-T1pks | 30 | Nanchangmycin |
| *Mycobacterium tuberculosis* OFXR-6 | 2575325434 | T3pks-T1pks | 27 | Nystatin |
| *Mycobacterium tuberculosis* OFXR-7 | 2575619239 | T3pks-T1pks | 33 | ML-449 |
| *Mycobacterium tuberculosis* OFXR-8 | 2575465536 | T3pks-T1pks | 27 | Nystatin |
| *Mycobacterium tuberculosis* OFXR-9 | 2590203420 | T3pks, T1pks | 100 | MAR/MAP |
| *Mycobacterium tuberculosis* OSDD071 | 2549407735 |  |  |  |
| *Mycobacterium tuberculosis* OSDD504 | 2549410800 |  |  |  |
| *Mycobacterium tuberculosis* OSDD518 | 2549413118 |  |  |  |
| *Mycobacterium tuberculosis* PanR0201 | 2555578867 | T3pks-T1pks | 33 | ML-449 |
| *Mycobacterium tuberculosis* PanR0202 | 2598067418 | T3pks, T1pks | 100 | MAR/MAP |
| *Mycobacterium tuberculosis* PanR0203 | 2555148489 | T3pks-T1pks | 33 | ML-449 |
| *Mycobacterium tuberculosis* PanR0205 | 2555140094 | T3pks-T1pks | 33 | ML-449 |
| *Mycobacterium tuberculosis* PanR0206 | 2555144317 | T3pks-T1pks | 27 | Neoaureothin |
| *Mycobacterium tuberculosis* PanR0207 | 2555160272 | T3pks-T1pks | 33 | ML-449 |
| *Mycobacterium tuberculosis* PanR0208 | 2555152706 | T3pks-T1pks | 33 | ML-449 |
| *Mycobacterium tuberculosis* PanR0209 | 2555164435 | T3pks-T1pks | 33 | ML-449 |
| *Mycobacterium tuberculosis* PanR0301 | 2555282371 | T3pks-T1pks | 33 | ML-449 |
| *Mycobacterium tuberculosis* PanR0304 | 2555278104 | T3pks-T1pks | 33 | ML-449 |
| *Mycobacterium tuberculosis* PanR0305 | 2555299255 | T3pks-T1pks | 33 | ML-449 |
| *Mycobacterium tuberculosis* PanR0306 | 2555303475 | T3pks-T1pks | 33 | ML-449 |
| *Mycobacterium tuberculosis* PanR0307 | 2555290904 | T3pks-T1pks | 33 | ML-449 |
| *Mycobacterium tuberculosis* PanR0308 | 2555273883 | T3pks-T1pks | 33 | ML-449 |
| *Mycobacterium tuberculosis* PanR0309 | 2555295099 | T3pks-T1pks | 33 | ML-449 |
| *Mycobacterium tuberculosis* PanR0311 | 2555286609 | T3pks-T1pks | 33 | ML-449 |
| *Mycobacterium tuberculosis* PanR0313 | 2555346108 | T3pks-T1pks | 33 | ML-449 |
| *Mycobacterium tuberculosis* PanR0314 | 2555307696 | T3pks-T1pks | 33 | ML-449 |
| *Mycobacterium tuberculosis* PanR0315 | 2555311951 | T3pks-T1pks | 16 | Akaeolide |
| *Mycobacterium tuberculosis* PanR0316 | 2555329342 | T3pks-T1pks | 33 | ML-449 |
| *Mycobacterium tuberculosis* PanR0317 | 2555560826 | T3pks-T1pks | 33 | ML-449 |
| *Mycobacterium tuberculosis* PanR0401 | 2555599804 | T3pks-T1pks | 33 | ML-449 |
| *Mycobacterium tuberculosis* PanR0403 | 2555543565 | T3pks-T1pks | 33 | ML-449 |
| *Mycobacterium tuberculosis* PanR0404 | 2555556540 | T3pks-T1pks | 33 | ML-449 |
| *Mycobacterium tuberculosis* PanR0405 | 2555516018 | T3pks-T1pks | 33 | ML-449 |
| *Mycobacterium tuberculosis* PanR0407 | 2555547894 | T3pks-T1pks | 33 | ML-449 |
| *Mycobacterium tuberculosis* PanR0409 | 2555587223 | T3pks-T1pks | 33 | ML-449 |
| *Mycobacterium tuberculosis* PanR0410 | 2555583039 | T3pks-T1pks | 33 | ML-449 |
| *Mycobacterium tuberculosis* PanR0411 | 2555520186 | T3pks-T1pks | 33 | ML-449 |
| *Mycobacterium tuberculosis* PanR0412 | 2555591399 | T3pks-T1pks | 33 | ML-449 |
| *Mycobacterium tuberculosis* PanR0501 | 2555333539 | T3pks-T1pks | 33 | ML-449 |
| *Mycobacterium tuberculosis* PanR0503 | 2555350295 | T3pks-T1pks | 33 | ML-449 |
| *Mycobacterium tuberculosis* PanR0505 | 2555354513 | T3pks-T1pks | 33 | ML-449 |
| *Mycobacterium tuberculosis* PanR0601 | 2555320646 |  |  |  |
| *Mycobacterium tuberculosis* PanR0602 | 2555337752 | T3pks-T1pks | 33 | ML-449 |
| *Mycobacterium tuberculosis* PanR0603 | 2555316223 | T3pks-T1pks | 33 | ML-449 |
| *Mycobacterium tuberculosis* PanR0604 | 2555325132 | T3pks-T1pks | 33 | ML-449 |
| *Mycobacterium tuberculosis* PanR0605 | 2555341950 | T3pks-T1pks | 71 | Tylactone |
| *Mycobacterium tuberculosis* PanR0606 | 2555358731 | T3pks-T1pks | 33 | ML-449 |
| *Mycobacterium tuberculosis* PanR0607 | 2555409181 | T3pks-T1pks | 33 | ML-449 |
| *Mycobacterium tuberculosis* PanR0610 | 2555396597 | T3pks-T1pks | 33 | ML-449 |
| *Mycobacterium tuberculosis* PanR0611 | 2555413387 | T3pks-T1pks | 33 | ML-449 |
| *Mycobacterium tuberculosis* PanR0702 | 2555417623 | T3pks-T1pks | 33 | ML-449 |
| *Mycobacterium tuberculosis* PanR0703 | 2555400811 | T3pks-T1pks | 33 | ML-449 |
| *Mycobacterium tuberculosis* PanR0704 | 2555528546 | T3pks-T1pks | 33 | ML-449 |
| *Mycobacterium tuberculosis* PanR0707 | 2555388140 | T3pks-T1pks | 33 | ML-449 |
| *Mycobacterium tuberculosis* PanR0708 | 2555371358 | T3pks-T1pks | 33 | ML-449 |
| *Mycobacterium tuberculosis* PanR0801 | 2555379694 | T3pks-T1pks | 33 | ML-449 |
| *Mycobacterium tuberculosis* PanR0802 | 2555524372 | T3pks-T1pks | 33 | ML-449 |
| *Mycobacterium tuberculosis* PanR0803 | 2555367201 | T3pks-T1pks | 33 | ML-449 |
| *Mycobacterium tuberculosis* PanR0804 | 2555392373 | T3pks-T1pks | 33 | ML-449 |
| *Mycobacterium tuberculosis* PanR0805 | 2555362970 | T3pks-T1pks | 33 | ML-449 |
| *Mycobacterium tuberculosis* PanR0902 | 2555383921 | T3pks-T1pks | 33 | ML-449 |
| *Mycobacterium tuberculosis* PanR0903 | 2555375511 | T3pks-T1pks | 33 | ML-449 |
| *Mycobacterium tuberculosis* PanR0904 | 2555442648 | T3pks-T1pks | 33 | ML-449 |
| *Mycobacterium tuberculosis* PanR0906 | 2555446824 | T3pks-T1pks | 33 | ML-449 |
| *Mycobacterium tuberculosis* PanR0907 | 2555455224 | T3pks-T1pks | 27 | Neoaureothin |
| *Mycobacterium tuberculosis* PanR0909 | 2555430098 | T3pks-T1pks | 33 | ML-449 |
| *Mycobacterium tuberculosis* PanR1005 | 2555595572 | T3pks-T1pks | 33 | ML-449 |
| *Mycobacterium tuberculosis* PanR1006 | 2555451004 | T3pks-T1pks | 33 | ML-449 |
| *Mycobacterium tuberculosis* PanR1007 | 2555434304 | T3pks-T1pks | 33 | ML-449 |
| *Mycobacterium tuberculosis* PanR1101 | 2555438445 | T3pks-T1pks | 33 | ML-449 |
| *Mycobacterium tuberculosis* PR05 | 2554700949 | T3pks-T1pks | 11 | Borrelidin |
| *Mycobacterium tuberculosis* R1207 | 2547951333 | T3pks-T1pks | 33 | ML-449 |
| *Mycobacterium tuberculosis* RGTB423 | 2514118145 | T3pks-T1pks | 13 | Lasalocid |
| *Mycobacterium tuberculosis* SK-B | 2576735552 | T3pks-T1pks | 33 | ML-449 |
| *Mycobacterium tuberculosis* SK-C | 2592445915 | T3pks-T1pks | 12 | Streptomycin |
| *Mycobacterium tuberculosis* SK-E | 2584652546 | T3pks-T1pks | 33 | ML-449 |
| *Mycobacterium tuberculosis* SUMu001 | 648443266 | T3pks-T1pks | 33 | ML-449 |
| *Mycobacterium tuberculosis* SUMu002 | 648446923 | T3pks | 100 | Micromonolactam |
| *Mycobacterium tuberculosis* SUMu004 | 648456112 | T3pks |  |  |
| *Mycobacterium tuberculosis* SUMu005 | 648460473 | T3pks | 100 | Micromonolactam |
| *Mycobacterium tuberculosis* SUMu006 | 648464907 |  |  |  |
| *Mycobacterium tuberculosis* SUMu007 | 648469578 | T3pks | 100 | Micromonolactam |
| *Mycobacterium tuberculosis* SUMu008 | 648473432 | T3pks | 100 | Micromonolactam |
| *Mycobacterium tuberculosis* SUMu009 | 648476944 |  |  |  |
| *Mycobacterium tuberculosis* SUMu010 | 648481186 | T3pks-T1pks | 33 | ML-449 |
| *Mycobacterium tuberculosis* SUMu012 | 648490022 | T1pks | 10 | Abyssomicin |
| *Mycobacterium tuberculosis* T17 | 643049582 |  |  |  |
| *Mycobacterium tuberculosis* T46 | 2574757270 | T3pks-T1pks | 33 | ML-449 |
| *Mycobacterium tuberculosis* T46 | 646010237 |  |  |  |
| *Mycobacterium tuberculosis* T67 | 2588659602 | T3pks-T1pks | 27 | Nystatin |
| *Mycobacterium tuberculosis* T85 | 643035957 |  |  |  |
| *Mycobacterium tuberculosis* T92 | 2584885951 | T3pks-T1pks | 33 | ML-449 |
| *Mycobacterium tuberculosis* T92 | 643028176 |  |  |  |
| *Mycobacterium tuberculosis* TB_RSA01 | 2584711251 | T3pks-T1pks | 27 | Neoaureothin |
| *Mycobacterium tuberculosis* TB_RSA03 | 2574700588 | T3pks-T1pks | 27 | Neoaureothin |
| *Mycobacterium tuberculosis* TB_RSA07 | 2577872587 | T3pks-T1pks | 33 | ML-449 |
| *Mycobacterium tuberculosis* TB_RSA09 | 2576886343 | T3pks-T1pks | 27 | Neoaureothin |
| *Mycobacterium tuberculosis* TB_RSA102 | 2575862603 | T3pks-T1pks | 16 | Akaeolide |
| *Mycobacterium tuberculosis* TB_RSA104 | 2584806904 | T3pks-T1pks | 41 | Piericidin A1 |
| *Mycobacterium tuberculosis* TB_RSA107 | 2584893836 | T3pks-T1pks | 16 | Akaeolide |
| *Mycobacterium tuberculosis* TB_RSA111 | 2574614619 | T3pks-T1pks | 16 | Akaeolide |
| *Mycobacterium tuberculosis* TB_RSA118 | 2575426848 | T3pks-T1pks | 33 | ML-449 |
| *Mycobacterium tuberculosis* TB_RSA12 | 2577091164 | T3pks-T1pks | 27 | Neoaureothin |
| *Mycobacterium tuberculosis* TB_RSA120 | 2577400922 | T3pks-T1pks | 33 | ML-449 |
| *Mycobacterium tuberculosis* TB_RSA123 | 2578002537 |  |  |  |
| *Mycobacterium tuberculosis* TB_RSA124 | 2578200424 | T3pks-T1pks | 16 | Akaeolide |
| *Mycobacterium tuberculosis* TB_RSA127 | 2577551091 | T3pks-T1pks | 33 | ML-449 |
| *Mycobacterium tuberculosis* TB_RSA132 | 2577988240 | T3pks-T1pks | 33 | ML-449 |
| *Mycobacterium tuberculosis* TB_RSA134 | 2575627312 |  |  |  |
| *Mycobacterium tuberculosis* TB_RSA136 | 2576443603 | T3pks-T1pks | 41 | Piericidin A1 |
| *Mycobacterium tuberculosis* TB_RSA138 | 2577900964 | T3pks-T1pks | 16 | Akaeolide |
| *Mycobacterium tuberculosis* TB_RSA140 | 2576163842 | T3pks-T1pks | 16 | Akaeolide |
| *Mycobacterium tuberculosis* TB_RSA148 | 2575295342 | T3pks-T1pks | 33 | ML-449 |
| *Mycobacterium tuberculosis* TB_RSA149 | 2576981010 | T3pks-T1pks | 20 | Kendomycin |
| *Mycobacterium tuberculosis* TB_RSA15 | 2575772716 | T3pks-T1pks | 27 | Neoaureothin |
| *Mycobacterium tuberculosis* TB_RSA161 | 2577569748 | T3pks-T1pks | 33 | ML-449 |
| *Mycobacterium tuberculosis* TB_RSA163 | 2576882264 | T3pks-T1pks | 33 | ML-449 |
| *Mycobacterium tuberculosis* TB_RSA165 | 2575409751 | T3pks-T1pks | 33 | ML-449 |
| *Mycobacterium tuberculosis* TB_RSA166 | 2584715344 | T3pks-T1pks | 33 | ML-449 |
| *Mycobacterium tuberculosis* TB_RSA173 | 2574937709 | T3pks-T1pks | 27 | Neoaureothin |
| *Mycobacterium tuberculosis* TB_RSA174 | 2575142685 | T3pks-T1pks | 27 | Neoaureothin |
| *Mycobacterium tuberculosis* TB_RSA178 | 2577038023 | T3pks-T1pks | 33 | ML-449 |
| *Mycobacterium tuberculosis* TB_RSA18 | 2576589126 | T3pks-T1pks | 27 | Neoaureothin |
| *Mycobacterium tuberculosis* TB_RSA194 | 2574784397 | T3pks-T1pks | 27 | Neoaureothin |
| *Mycobacterium tuberculosis* TB_RSA195 | 2576399693 | T3pks-T1pks | 33 | ML-449 |
| *Mycobacterium tuberculosis* TB_RSA199 | 2576059958 | T3pks-T1pks | 33 | ML-449 |
| *Mycobacterium tuberculosis* TB_RSA21 | 2577812038 | T3pks-T1pks | 27 | Neoaureothin |
| *Mycobacterium tuberculosis* TB_RSA25 | 2584737038 | T3pks-T1pks | 16 | Akaeolide |
| *Mycobacterium tuberculosis* TB_RSA32 | 2578013153 | T3pks-T1pks | 27 | Neoaureothin |
| *Mycobacterium tuberculosis* TB_RSA45 | 2584743940 | T3pks-T1pks | 27 | Neoaureothin |
| *Mycobacterium tuberculosis* TB_RSA46 | 2584865918 | T3pks-T1pks | 27 | Neoaureothin |
| *Mycobacterium tuberculosis* TB_RSA51 | 2577689111 | T3pks-T1pks | 27 | Neoaureothin |
| *Mycobacterium tuberculosis* TB_RSA59 | 2576630848 | T3pks-T1pks | 33 | ML-449 |
| *Mycobacterium tuberculosis* TB_RSA62 | 2584902930 |  |  |  |
| *Mycobacterium tuberculosis* TB_RSA64 | 2579798813 | T3pks-T1pks | 27 | Nystatin |
| *Mycobacterium tuberculosis* TB_RSA66 | 2576613004 | T3pks-T1pks | 16 | Akaeolide |
| *Mycobacterium tuberculosis* TB_RSA67 | 2576051820 | T3pks-T1pks | 33 | ML-449 |
| *Mycobacterium tuberculosis* TB_RSA68 | 2574872651 | T3pks-T1pks | 33 | ML-449 |
| *Mycobacterium tuberculosis* TB_RSA70 | 2577641910 | T3pks-T1pks | 41 | Piericidin A1 |
| *Mycobacterium tuberculosis* TB_RSA74 | 2578073775 | T3pks-T1pks | 16 | Akaeolide |
| *Mycobacterium tuberculosis* TB_RSA76 | 2576321347 | T3pks-T1pks | 20 | Kendomycin |
| *Mycobacterium tuberculosis* TB_RSA77 | 2577203844 | T3pks-T1pks | 16 | Akaeolide |
| *Mycobacterium tuberculosis* TB_RSA78 | 2578053379 | T3pks-T1pks | 16 | Akaeolide |
| *Mycobacterium tuberculosis* TB_RSA79 | 2584923296 | T3pks-T1pks | 16 | Akaeolide |
| *Mycobacterium tuberculosis* TB_RSA82 | 2576583109 | T3pks, T1pks | 100 | MAR/MAP |
| *Mycobacterium tuberculosis* TB_RSA83 | 2576158036 |  |  |  |
| *Mycobacterium tuberculosis* TB_RSA90 | 2575476916 | T3pks-T1pks | 20 | Kendomycin |
| *Mycobacterium tuberculosis* TB_RSA96 | 2574738070 | T3pks-T1pks | 33 | ML-449 |
| *Mycobacterium tuberculosis* TB_RSA97 | 2576585830 | T3pks-T1pks | 33 | ML-449 |
| *Mycobacterium tuberculosis* TB_RSA99 | 2575611823 | T3pks-T1pks | 20 | Kendomycin |
| *Mycobacterium tuberculosis* TBR10 | 2579813772 | T3pks-T1pks | 33 | ML-449 |
| *Mycobacterium tuberculosis* TBR11 | 2589040293 | T3pks-T1pks | 27 | Nystatin |
| *Mycobacterium tuberculosis* TBR23 | 2589053585 | T3pks-T1pks | 27 | Nystatin |
| *Mycobacterium tuberculosis* TBR24 | 2589048312 | T3pks-T1pks | 33 | ML-449 |
| *Mycobacterium tuberculosis* TBR26 | 2577792721 | T3pks-T1pks | 33 | ML-449 |
| *Mycobacterium tuberculosis* TBR28 | 2575381682 | T3pks-T1pks | 33 | ML-449 |
| *Mycobacterium tuberculosis* TBR29 | 2589056454 | T3pks-T1pks | 33 | ML-449 |
| *Mycobacterium tuberculosis* TBR30 | 2589060525 | T3pks-T1pks | 33 | ML-449 |
| *Mycobacterium tuberculosis* TBR31 | 2574635031 | T3pks-T1pks | 33 | ML-449 |
| *Mycobacterium tuberculosis* TBR35 | 2589068752 | T3pks-T1pks | 41 | Piericidin A1 |
| *Mycobacterium tuberculosis* TBR37 | 2576759003 | T3pks-T1pks | 27 | Nystatin |
| *Mycobacterium tuberculosis* TBR4 | 2589036086 | T3pks-T1pks | 33 | ML-449 |
| *Mycobacterium tuberculosis* TBR40 | 2589064595 | T3pks-T1pks | 33 | ML-449 |
| *Mycobacterium tuberculosis* TBR41 | 2575888575 | T3pks-T1pks | 33 | ML-449 |
| *Mycobacterium tuberculosis* TBR42 | 2589073023 | T3pks-T1pks | 30 | Nanchangmycin |
| *Mycobacterium tuberculosis* TBR43 | 2589077872 | T3pks-T1pks | 27 | Nystatin |
| *Mycobacterium tuberculosis* TBR44 | 2589082097 | T3pks-T1pks | 27 | Nystatin |
| *Mycobacterium tuberculosis* TBR48 | 2589089021 | T3pks-T1pks | 33 | ML-449 |
| *Mycobacterium tuberculosis* TBR49 | 2589093094 | T3pks-T1pks | 33 | ML-449 |
| *Mycobacterium tuberculosis* TBR5 | 2589032800 | T3pks-T1pks | 9 | Stenothricin |
| *Mycobacterium tuberculosis* TBR50 | 2576156796 | T3pks-T1pks | 27 | Nystatin |
| *Mycobacterium tuberculosis* TBR51 | 2584609065 | T3pks-T1pks | 33 | ML-449 |
| *Mycobacterium tuberculosis* TBR53 | 2589106145 | T3pks-T1pks | 27 | Nystatin |
| *Mycobacterium tuberculosis* TBR55 | 2575230562 | T3pks-T1pks | 27 | Neoaureothin |
| *Mycobacterium tuberculosis* TBR56 | 2589100068 | T3pks-T1pks | 27 | Nystatin |
| *Mycobacterium tuberculosis* TBR57 | 2589098383 | T3pks-T1pks | 27 | Nystatin |
| *Mycobacterium tuberculosis* TBR58 | 2574876573 | T3pks-T1pks | 33 | ML-449 |
| *Mycobacterium tuberculosis* TBR60 | 2589117553 | T3pks-T1pks | 33 | ML-449 |
| *Mycobacterium tuberculosis* TBR65 | 2574886309 | T3pks-T1pks | 27 | Nystatin |
| *Mycobacterium tuberculosis* TBR66 | 2589113575 | T3pks-T1pks | 16 | Akaeolide |
| *Mycobacterium tuberculosis* TBR7 | 2575978404 | T3pks-T1pks | 33 | ML-449 |
| *Mycobacterium tuberculosis* TBR74 | 2589121735 | T3pks-T1pks | 16 | Akaeolide |
| *Mycobacterium tuberculosis* TBR75 | 2589125802 | T3pks-T1pks | 33 | ML-449 |
| *Mycobacterium tuberculosis* TBR76 | 2589139119 | T3pks-T1pks | 27 | Nystatin |
| *Mycobacterium tuberculosis* TBR79 | 2589134136 | T3pks-T1pks | 27 | Nystatin |
| *Mycobacterium tuberculosis* TBR8 | 2589026697 | T3pks-T1pks | 27 | Nystatin |
| *Mycobacterium tuberculosis* TBR80 | 2589130082 | T3pks-T1pks | 27 | Nystatin |
| *Mycobacterium tuberculosis* TBR9 | 2589044237 | T3pks-T1pks | 33 | ML-449 |
| *Mycobacterium tuberculosis* TKK_02_0001 | 2592442711 | T3pks, T1pks | 100 | MAR/MAP |
| *Mycobacterium tuberculosis* TKK_02_0002 | 2584801008 | T3pks-T1pks | 33 | ML-449 |
| *Mycobacterium tuberculosis* TKK_02_0003 | 2592438630 | T3pks-T1pks | 12 | Streptomycin |
| *Mycobacterium tuberculosis* TKK_02_0006 | 2592435284 | T3pks-T1pks | 12 | Streptomycin |
| *Mycobacterium tuberculosis* TKK_02_0012 | 2592430392 | T3pks, T1pks | 100 | MAR/MAP |
| *Mycobacterium tuberculosis* TKK_02_0013 | 2592426321 | T3pks-T1pks | 12 | Streptomycin |
| *Mycobacterium tuberculosis* TKK_02_0014 | 2584939524 | T3pks-T1pks | 33 | ML-449 |
| *Mycobacterium tuberculosis* TKK_02_0015 | 2574726119 | T3pks-T1pks | 33 | ML-449 |
| *Mycobacterium tuberculosis* TKK_02_0016 | 2592422247 | T3pks-T1pks | 12 | Streptomycin |
| *Mycobacterium tuberculosis* TKK_02_0017 | 2592417392 | T3pks, T1pks | 100 | MAR/MAP |
| *Mycobacterium tuberculosis* TKK_02_0018 | 2592414205 | T3pks, T1pks | 100 | MAR/MAP |
| *Mycobacterium tuberculosis* TKK_02_0019 | 2575329342 | T3pks-T1pks | 33 | ML-449 |
| *Mycobacterium tuberculosis* TKK_02_0020 | 2592410032 | T3pks, T1pks | 100 | MAR/MAP |
| *Mycobacterium tuberculosis* TKK_02_0021 | 2592405959 | T3pks-T1pks | 12 | Streptomycin |
| *Mycobacterium tuberculosis* TKK_02_0022 | 2592403099 | T3pks-T1pks | 12 | Streptomycin |
| *Mycobacterium tuberculosis* TKK_02_0025 | 2592397895 | T3pks, T1pks | 100 | MAR/MAP |
| *Mycobacterium tuberculosis* TKK_02_0027 | 2592393538 | T3pks, T1pks | 100 | MAR/MAP |
| *Mycobacterium tuberculosis* TKK_02_0033 | 2592389699 | T3pks-T1pks | 12 | Streptomycin |
| *Mycobacterium tuberculosis* TKK_02_0034 | 2592385592 | T3pks, T1pks | 100 | MAR/MAP |
| *Mycobacterium tuberculosis* TKK_02_0036 | 2592381635 | T3pks, T1pks | 100 | MAR/MAP |
| *Mycobacterium tuberculosis* TKK_02_0038 | 2577651582 | T3pks-T1pks | 33 | ML-449 |
| *Mycobacterium tuberculosis* TKK_02_0039 | 2592377452 | T3pks-T1pks | 12 | Streptomycin |
| *Mycobacterium tuberculosis* TKK_02_0045 | 2576366402 | T3pks-T1pks | 33 | ML-449 |
| *Mycobacterium tuberculosis* TKK_02_0046 | 2592373455 | T3pks-T1pks | 12 | Streptomycin |
| *Mycobacterium tuberculosis* TKK_02_0061 | 2592364277 | T3pks-T1pks | 12 | Streptomycin |
| *Mycobacterium tuberculosis* TKK_02_0062 | 2592361217 | T3pks, T1pks | 100 | MAR/MAP |
| *Mycobacterium tuberculosis* TKK_02_0063 | 2592357110 | T3pks, T1pks | 100 | MAR/MAP |
| *Mycobacterium tuberculosis* TKK_02_0067 | 2592353030 | T3pks-T1pks | 12 | Streptomycin |
| *Mycobacterium tuberculosis* TKK_02_0068 | 2592348241 | T3pks-T1pks | 12 | Streptomycin |
| *Mycobacterium tuberculosis* TKK_02_0069 | 2592344179 | T3pks, T1pks | 100 | MAR/MAP |
| *Mycobacterium tuberculosis* TKK_02_0071 | 2574860198 | T3pks-T1pks | 27 | Nystatin |
| *Mycobacterium tuberculosis* TKK_02_0073 | 2592337997 | T3pks-T1pks | 12 | Streptomycin |
| *Mycobacterium tuberculosis* TKK_02_0077 | 2592332604 | T3pks, T1pks | 100 | MAR/MAP |
| *Mycobacterium tuberculosis* TKK_03_0018 | 2592328029 | T3pks-T1pks | 12 | Streptomycin |
| *Mycobacterium tuberculosis* TKK_03_0020 | 2592324422 | T3pks-T1pks | 12 | Streptomycin |
| *Mycobacterium tuberculosis* TKK_03_0022 | 2592319784 | T3pks-T1pks | 31 | Stenothricin |
| *Mycobacterium tuberculosis* TKK_03_0024 | 2577168470 | T3pks-T1pks | 33 | ML-449 |
| *Mycobacterium tuberculosis* TKK_03_0025 | 2592316279 | T3pks, T1pks | 100 | MAR/MAP |
| *Mycobacterium tuberculosis* TKK_03_0026 | 2574803240 | T3pks-T1pks | 33 | ML-449 |
| *Mycobacterium tuberculosis* TKK_03_0027 | 2592312197 | T3pks-T1pks | 12 | Streptomycin |
| *Mycobacterium tuberculosis* TKK_03_0029 | 2592308116 | T3pks, T1pks | 100 | MAR/MAP |
| *Mycobacterium tuberculosis* TKK_03_0030 | 2592303254 | T3pks, T1pks | 100 | MAR/MAP |
| *Mycobacterium tuberculosis* TKK_03_0031 | 2577816400 | T3pks-T1pks | 33 | ML-449 |
| *Mycobacterium tuberculosis* TKK_03_0033 | 2592299949 | T3pks-T1pks | 12 | Streptomycin |
| *Mycobacterium tuberculosis* TKK_03_0034 | 2592295853 | T3pks, T1pks | 100 | MAR/MAP |
| *Mycobacterium tuberculosis* TKK_03_0036 | 2592291479 | T3pks, T1pks | 100 | MAR/MAP |
| *Mycobacterium tuberculosis* TKK_03_0040 | 2584674477 | T3pks-T1pks | 33 | ML-449 |
| *Mycobacterium tuberculosis* TKK_03_0042 | 2575858812 | T3pks-T1pks | 23 | Jerangolid |
| *Mycobacterium tuberculosis* TKK_03_0043 | 2592287696 | T3pks, T1pks | 100 | MAR/MAP |
| *Mycobacterium tuberculosis* TKK_03_0045 | 2592283614 | T3pks-T1pks | 12 | Streptomycin |
| *Mycobacterium tuberculosis* TKK_03_0059 | 2584670398 | T3pks-T1pks | 23 | Jerangolid |
| *Mycobacterium tuberculosis* TKK_03_0078 | 2584666311 | T3pks-T1pks | 33 | ML-449 |
| *Mycobacterium tuberculosis* TKK_03_0081 | 2592280664 | T3pks-T1pks | 12 | Streptomycin |
| *Mycobacterium tuberculosis* TKK_03_0082 | 2592275464 | T3pks, T1pks | 100 | MAR/MAP |
| *Mycobacterium tuberculosis* TKK_03_0083 | 2577673179 | T3pks-T1pks | 18 | Neoaureothin |
| *Mycobacterium tuberculosis* TKK_03_0090 | 2576708817 | T3pks-T1pks | 33 | ML-449 |
| *Mycobacterium tuberculosis* TKK_03_0094 | 2578246558 | T3pks-T1pks | 32 | ECO-02301 |
| *Mycobacterium tuberculosis* TKK_03_0096 | 2578195506 | T3pks-T1pks | 33 | ML-449 |
| *Mycobacterium tuberculosis* TKK_03_0099 | 2575806159 | T3pks-T1pks | 41 | Piericidin A1 |
| *Mycobacterium tuberculosis* TKK_03_0103 | 2577796885 | T3pks-T1pks | 33 | ML-449 |
| *Mycobacterium tuberculosis* TKK_03_0109 | 2575455196 | T3pks-T1pks | 33 | ML-449 |
| *Mycobacterium tuberculosis* TKK_03_0116 | 2576936333 | T3pks-T1pks | 23 | Jerangolid |
| *Mycobacterium tuberculosis* TKK_03_0118 | 2577936335 |  |  |  |
| *Mycobacterium tuberculosis* TKK_03_0156 | 2575103505 | T3pks-T1pks | 18 | Neoaureothin |
| *Mycobacterium tuberculosis* TKK_03_0158 | 2576932272 | T3pks-T1pks | 41 | Piericidin A1 |
| *Mycobacterium tuberculosis* TKK_04_0001 | 2592271382 | T3pks, T1pks | 100 | MAR/MAP |
| *Mycobacterium tuberculosis* TKK_04_0002 | 2584967006 | T3pks-T1pks | 23 | Jerangolid |
| *Mycobacterium tuberculosis* TKK_04_0003 | 2576388909 | T3pks-T1pks | 23 | Jerangolid |
| *Mycobacterium tuberculosis* TKK_04_0005 | 2592267285 | T3pks-T1pks | 12 | Streptomycin |
| *Mycobacterium tuberculosis* TKK_04_0006 | 2592261699 | T3pks, T1pks | 100 | MAR/MAP |
| *Mycobacterium tuberculosis* TKK_04_0007 | 2592259141 | T3pks-T1pks | 12 | Streptomycin |
| *Mycobacterium tuberculosis* TKK_04_0008 | 2592254166 | T3pks-T1pks | 12 | Streptomycin |
| *Mycobacterium tuberculosis* TKK_04_0013 | 2592250930 | T3pks, T1pks | 100 | MAR/MAP |
| *Mycobacterium tuberculosis* TKK_04_0014 | 2592246876 | T3pks, T1pks | 100 | MAR/MAP |
| *Mycobacterium tuberculosis* TKK_04_0015 | 2592242791 | T3pks-T1pks | 12 | Streptomycin |
| *Mycobacterium tuberculosis* TKK_04_0017 | 2592238712 | T3pks, T1pks | 100 | MAR/MAP |
| *Mycobacterium tuberculosis* TKK_04_0018 | 2592234638 | T3pks, T1pks | 100 | MAR/MAP |
| *Mycobacterium tuberculosis* TKK_04_0019 | 2575132657 | T3pks-T1pks | 33 | ML-449 |
| *Mycobacterium tuberculosis* TKK_04_0020 | 2592230560 | T3pks-T1pks | 12 | Streptomycin |
| *Mycobacterium tuberculosis* TKK_04_0021 | 2575542837 | T3pks-T1pks | 16 | Akaeolide |
| *Mycobacterium tuberculosis* TKK_04_0022 | 2592226489 | T3pks, T1pks | 100 | MAR/MAP |
| *Mycobacterium tuberculosis* TKK_04_0023 | 2592222422 | T3pks, T1pks | 100 | MAR/MAP |
| *Mycobacterium tuberculosis* TKK_04_0024 | 2576077839 | T3pks-T1pks | 16 | Akaeolide |
| *Mycobacterium tuberculosis* TKK_04_0029 | 2592579018 | T3pks-T1pks | 12 | Streptomycin |
| *Mycobacterium tuberculosis* TKK_04_0030 | 2592574645 | T3pks, T1pks | 100 | MAR/MAP |
| *Mycobacterium tuberculosis* TKK_04_0031 | 2592570555 | T3pks-T1pks | 12 | Streptomycin |
| *Mycobacterium tuberculosis* TKK_04_0033 | 2578177805 | T3pks-T1pks | 33 | ML-449 |
| *Mycobacterium tuberculosis* TKK_04_0034 | 2592566477 | T3pks, T1pks | 100 | MAR/MAP |
| *Mycobacterium tuberculosis* TKK_04_0036 | 2592562396 | T3pks, T1pks | 100 | MAR/MAP |
| *Mycobacterium tuberculosis* TKK_04_0037 | 2592558321 | T3pks-T1pks | 12 | Streptomycin |
| *Mycobacterium tuberculosis* TKK_04_0038 | 2584836956 | T3pks-T1pks | 33 | ML-449 |
| *Mycobacterium tuberculosis* TKK_04_0039 | 2592553482 | T3pks-T1pks | 12 | Streptomycin |
| *Mycobacterium tuberculosis* TKK_04_0040 | 2576940434 | T3pks-T1pks | 33 | ML-449 |
| *Mycobacterium tuberculosis* TKK_04_0042 | 2584906709 | T3pks-T1pks | 16 | Akaeolide |
| *Mycobacterium tuberculosis* TKK_04_0043 | 2592549349 | T3pks, T1pks | 100 | MAR/MAP |
| *Mycobacterium tuberculosis* TKK_04_0044 | 2575935659 | T3pks-T1pks | 33 | ML-449 |
| *Mycobacterium tuberculosis* TKK_04_0045 | 2584858071 | T3pks-T1pks | 16 | Akaeolide |
| *Mycobacterium tuberculosis* TKK_04_0046 | 2592546111 | T3pks, T1pks | 100 | MAR/MAP |
| *Mycobacterium tuberculosis* TKK_04_0047 | 2592542016 | T3pks, T1pks | 100 | MAR/MAP |
| *Mycobacterium tuberculosis* TKK_04_0048 | 2592537861 | T3pks-T1pks | 12 | Streptomycin |
| *Mycobacterium tuberculosis* TKK_04_0051 | 2576378950 | T3pks-T1pks | 33 | ML-449 |
| *Mycobacterium tuberculosis* TKK_04_0054 | 2577386815 | T3pks-T1pks | 33 | ML-449 |
| *Mycobacterium tuberculosis* TKK_04_0060 | 2584841030 | T3pks-T1pks | 33 | ML-449 |
| *Mycobacterium tuberculosis* TKK_04_0064 | 2575920420 | T3pks-T1pks | 33 | ML-449 |
| *Mycobacterium tuberculosis* TKK_04_0066 | 2584694835 | T3pks-T1pks | 33 | ML-449 |
| *Mycobacterium tuberculosis* TKK_04_0067 | 2584979253 | T3pks-T1pks | 16 | Akaeolide |
| *Mycobacterium tuberculosis* TKK_04_0072 | 2584703088 | T3pks-T1pks | 33 | ML-449 |
| *Mycobacterium tuberculosis* TKK_04_0075 | 2574928549 | T3pks-T1pks | 33 | ML-449 |
| *Mycobacterium tuberculosis* TKK_04_0080 | 2574734007 |  |  |  |
| *Mycobacterium tuberculosis* TKK_04_0082 | 2584759228 | T3pks-T1pks | 27 | Nystatin |
| *Mycobacterium tuberculosis* TKK_04_0094 | 2584974532 | T3pks-T1pks | 33 | ML-449 |
| *Mycobacterium tuberculosis* TKK_04_0103 | 2576247251 | T3pks-T1pks | 41 | Piericidin A1 |
| *Mycobacterium tuberculosis* TKK_04_0108 | 2576671439 | T3pks-T1pks | 33 | ML-449 |
| *Mycobacterium tuberculosis* TKK_04_0117 | 2576665707 | T3pks-T1pks | 33 | ML-449 |
| *Mycobacterium tuberculosis* TKK_04_0120 | 2574640348 |  |  |  |
| *Mycobacterium tuberculosis* TKK_04_0129 | 2584776403 | T3pks-T1pks | 33 | ML-449 |
| *Mycobacterium tuberculosis* TKK_04_0132 | 2577516047 | T3pks-T1pks | 33 | ML-449 |
| *Mycobacterium tuberculosis* TKK_04_0140 | 2576497080 |  |  |  |
| *Mycobacterium tuberculosis* TKK_04_0157 | 2574843285 | T3pks-T1pks | 33 | ML-449 |
| *Mycobacterium tuberculosis* TKK_05MA_0009 | 2584785697 |  |  |  |
| *Mycobacterium tuberculosis* TKK_05MA_0012 | 2577215885 |  |  |  |
| *Mycobacterium tuberculosis* TKK_05MA_0020 | 2574799840 | T3pks-T1pks | 23 | Jerangolid |
| *Mycobacterium tuberculosis* TKK_05MA_0025 | 2575674521 | T3pks-T1pks | 33 | ML-449 |
| *Mycobacterium tuberculosis* TKK_05MA_0033 | 2577593438 |  |  |  |
| *Mycobacterium tuberculosis* TKK_05MA_0040 | 2584752944 |  |  |  |
| *Mycobacterium tuberculosis* TKK_05MA_0052 | 2577129296 | T3pks-T1pks | 33 | ML-449 |
| *Mycobacterium tuberculosis* TKK_05SA_0012 | 2575601683 |  |  |  |
| *Mycobacterium tuberculosis* TKK_05SA_0014 | 2577856904 | T3pks-T1pks | 33 | ML-449 |
| *Mycobacterium tuberculosis* TKK_05SA_0016 | 2574985446 |  |  |  |
| *Mycobacterium tuberculosis* TKK_05SA_0017 | 2578040087 | T3pks-T1pks | 33 | ML-449 |
| *Mycobacterium tuberculosis* TKK_05SA_0019 | 2577030827 | T3pks-T1pks | 33 | ML-449 |
| *Mycobacterium tuberculosis* TKK_05SA_0020 | 2584781205 | T3pks-T1pks | 23 | Jerangolid |
| *Mycobacterium tuberculosis* TKK_05SA_0042 | 2584860925 | T3pks-T1pks | 33 | ML-449 |
| *Mycobacterium tuberculosis* TKK_05SA_0048 | 2575790354 | T3pks-T1pks | 33 | ML-449 |
| *Mycobacterium tuberculosis* TKK_05SA_0050 | 2577381729 | T3pks-T1pks | 20 | Kendomycin |
| *Mycobacterium tuberculosis* TKK_05SA_0058 | 2575028161 |  |  |  |
| *Mycobacterium tuberculosis* TKK-01-0001 | 2589491285 | T3pks-T1pks | 27 | Nystatin |
| *Mycobacterium tuberculosis* TKK-01-0002 | 2589486069 | T3pks-T1pks | 33 | ML-449 |
| *Mycobacterium tuberculosis* TKK-01-0003 | 2589514641 | T3pks-T1pks | 33 | ML-449 |
| *Mycobacterium tuberculosis* TKK-01-0004 | 2589506393 | T3pks-T1pks | 33 | ML-449 |
| *Mycobacterium tuberculosis* TKK-01-0005 | 2589502409 | T3pks-T1pks | 33 | ML-449 |
| *Mycobacterium tuberculosis* TKK-01-0006 | 2577241442 | T3pks-T1pks | 27 | Nystatin |
| *Mycobacterium tuberculosis* TKK-01-0007 | 2589498327 | T3pks-T1pks | 33 | ML-449 |
| *Mycobacterium tuberculosis* TKK-01-0008 | 2588974834 | T3pks-T1pks | 11 | Borrelidin |
| *Mycobacterium tuberculosis* TKK-01-0010 | 2575495357 | T3pks-T1pks | 33 | ML-449 |
| *Mycobacterium tuberculosis* TKK-01-0011 | 2575538950 | T3pks-T1pks | 27 | Nystatin |
| *Mycobacterium tuberculosis* TKK-01-0012 | 2589509569 | T3pks-T1pks | 27 | Nystatin |
| *Mycobacterium tuberculosis* TKK-01-0013 | 2589518744 | T3pks-T1pks | 27 | Nystatin |
| *Mycobacterium tuberculosis* TKK-01-0014 | 2589522796 | T3pks-T1pks | 33 | ML-449 |
| *Mycobacterium tuberculosis* TKK-01-0015 | 2589539130 | T3pks-T1pks | 23 | Jerangolid |
| *Mycobacterium tuberculosis* TKK-01-0016 | 2589535085 |  |  |  |
| *Mycobacterium tuberculosis* TKK-01-0019 | 2575786887 | T3pks-T1pks | 33 | ML-449 |
| *Mycobacterium tuberculosis* TKK-01-0020 | 2588987052 |  |  |  |
| *Mycobacterium tuberculosis* TKK-01-0021 | 2584833644 | T3pks-T1pks | 33 | ML-449 |
| *Mycobacterium tuberculosis* TKK-01-0022 | 2589526877 | T3pks-T1pks | 16 | Akaeolide |
| *Mycobacterium tuberculosis* TKK-01-0023 | 2588982991 | T3pks-T1pks | 26 | JBIR-100 |
| *Mycobacterium tuberculosis* TKK-01-0024 | 2589543202 | T3pks-T1pks | 26 | JBIR-100 |
| *Mycobacterium tuberculosis* TKK-01-0025 | 2578182623 | T3pks-T1pks | 27 | Nystatin |
| *Mycobacterium tuberculosis* TKK-01-0026 | 2578008273 | T3pks-T1pks | 33 | ML-449 |
| *Mycobacterium tuberculosis* TKK-01-0027 | 2589547247 | T3pks-T1pks | 33 | ML-449 |
| *Mycobacterium tuberculosis* TKK-01-0028 | 2589563529 | T3pks-T1pks | 33 | ML-449 |
| *Mycobacterium tuberculosis* TKK-01-0029 | 2589559459 | T3pks-T1pks | 23 | Jerangolid |
| *Mycobacterium tuberculosis* TKK-01-0030 | 2589555384 |  |  |  |
| *Mycobacterium tuberculosis* TKK-01-0031 | 2589551314 | T3pks-T1pks | 41 | Piericidin A1 |
| *Mycobacterium tuberculosis* TKK-01-0032 | 2584825730 | T3pks-T1pks | 30 | Nanchangmycin |
| *Mycobacterium tuberculosis* TKK-01-0033 | 2589568736 | T3pks-T1pks | 27 | Nystatin |
| *Mycobacterium tuberculosis* TKK-01-0034 | 2589592089 | T3pks-T1pks | 41 | Piericidin A1 |
| *Mycobacterium tuberculosis* TKK-01-0035 | 2579825786 | T3pks-T1pks | 27 | Nystatin |
| *Mycobacterium tuberculosis* TKK-01-0036 | 2589572815 | T3pks-T1pks | 27 | Nystatin |
| *Mycobacterium tuberculosis* TKK-01-0037 | 2577861791 | T3pks-T1pks | 33 | ML-449 |
| *Mycobacterium tuberculosis* TKK-01-0038 | 2584645880 |  |  |  |
| *Mycobacterium tuberculosis* TKK-01-0039 | 2589575466 | T3pks-T1pks | 27 | Nystatin |
| *Mycobacterium tuberculosis* TKK-01-0040 | 2589580598 | T3pks-T1pks | 27 | Nystatin |
| *Mycobacterium tuberculosis* TKK-01-0042 | 2589583955 | T3pks | 83 | MAR/MAP |
| *Mycobacterium tuberculosis* TKK-01-0043 | 2589588224 | T3pks-T1pks | 27 | Nystatin |
| *Mycobacterium tuberculosis* TKK-01-0044 | 2589595990 | T3pks-T1pks | 33 | ML-449 |
| *Mycobacterium tuberculosis* TKK-01-0045 | 2589620637 | T3pks-T1pks | 41 | Piericidin A1 |
| *Mycobacterium tuberculosis* TKK-01-0046 | 2589616566 | T3pks-T1pks | 33 | ML-449 |
| *Mycobacterium tuberculosis* TKK-01-0047 | 2577322951 | T3pks-T1pks | 33 | ML-449 |
| *Mycobacterium tuberculosis* TKK-01-0048 | 2589613612 | T3pks, T1pks | 100 | MAR/MAP |
| *Mycobacterium tuberculosis* TKK-01-0049 | 2589608377 | T3pks-T1pks | 33 | ML-449 |
| *Mycobacterium tuberculosis* TKK-01-0050 | 2589604293 | T3pks, T1pks | 100 | MAR/MAP |
| *Mycobacterium tuberculosis* TKK-01-0052 | 2589598915 | T3pks, T1pks | 100 | MAR/MAP |
| *Mycobacterium tuberculosis* TKK-01-0054 | 2589658610 | T3pks-T1pks | 41 | Piericidin A1 |
| *Mycobacterium tuberculosis* TKK-01-0055 | 2589650472 | T3pks-T1pks | 33 | ML-449 |
| *Mycobacterium tuberculosis* TKK-01-0056 | 2589654545 | T3pks-T1pks | 33 | ML-449 |
| *Mycobacterium tuberculosis* TKK-01-0057 | 2589624713 |  |  |  |
| *Mycobacterium tuberculosis* TKK-01-0058 | 2589629003 | T3pks-T1pks | 27 | Nystatin |
| *Mycobacterium tuberculosis* TKK-01-0060 | 2589638476 | T3pks-T1pks | 27 | Nystatin |
| *Mycobacterium tuberculosis* TKK-01-0062 | 2589642534 | T3pks-T1pks | 30 | Nanchangmycin |
| *Mycobacterium tuberculosis* TKK-01-0063 | 2589646609 | T3pks-T1pks | 27 | Nystatin |
| *Mycobacterium tuberculosis* TKK-01-0064 | 2584625495 |  |  |  |
| *Mycobacterium tuberculosis* TKK-01-0065 | 2584898862 | T3pks-T1pks | 27 | Nystatin |
| *Mycobacterium tuberculosis* TKK-01-0066 | 2589687166 | T3pks-T1pks | 33 | ML-449 |
| *Mycobacterium tuberculosis* TKK-01-0068 | 2577751179 | T3pks-T1pks | 27 | Nystatin |
| *Mycobacterium tuberculosis* TKK-01-0069 | 2588992251 | T3pks-T1pks | 27 | Nystatin |
| *Mycobacterium tuberculosis* TKK-01-0070 | 2589679190 |  |  |  |
| *Mycobacterium tuberculosis* TKK-01-0071 | 2589676017 | T3pks, T1pks | 100 | MAR/MAP |
| *Mycobacterium tuberculosis* TKK-01-0072 | 2588999278 | T3pks-T1pks | 23 | Jerangolid |
| *Mycobacterium tuberculosis* TKK-01-0073 | 2589671952 | T3pks-T1pks | 27 | Nystatin |
| *Mycobacterium tuberculosis* TKK-01-0074 | 2589663797 | T3pks-T1pks | 27 | Nystatin |
| *Mycobacterium tuberculosis* TKK-01-0075 | 2575508785 | T3pks-T1pks | 27 | Nystatin |
| *Mycobacterium tuberculosis* TKK-01-0076 | 2589666655 | T3pks-T1pks | 33 | ML-449 |
| *Mycobacterium tuberculosis* TKK-01-0077 | 2575447433 | T3pks-T1pks | 27 | Nystatin |
| *Mycobacterium tuberculosis* TKK-01-0078 | 2589699410 | T3pks, T1pks | 100 | MAR/MAP |
| *Mycobacterium tuberculosis* TKK-01-0079 | 2577256179 | T3pks-T1pks | 27 | Nystatin |
| *Mycobacterium tuberculosis* TKK-01-0080 | 2588995218 | T3pks-T1pks | 23 | Jerangolid |
| *Mycobacterium tuberculosis* TKK-01-0081 | 2589691243 | T3pks, T1pks | 100 | MAR/MAP |
| *Mycobacterium tuberculosis* TKK-01-0082 | 2589707360 | T3pks-T1pks | 27 | Nystatin |
| *Mycobacterium tuberculosis* TKK-01-0083 | 2589711853 | T3pks-T1pks | 27 | Nystatin |
| *Mycobacterium tuberculosis* TKK-01-0084 | 2589715710 | T3pks, T1pks | 100 | MAR/MAP |
| *Mycobacterium tuberculosis* TKK-01-0086 | 2589724111 | T3pks-T1pks | 27 | Nystatin |
| *Mycobacterium tuberculosis* TKK-01-0087 | 2589719807 | T3pks, T1pks | 100 | MAR/MAP |
| *Mycobacterium tuberculosis* TKK-01-0088 | 2589703490 | T3pks-T1pks | 33 | ML-449 |
| *Mycobacterium tuberculosis* TKK-01-0089 | 2589695547 | T3pks, T1pks | 100 | MAR/MAP |
| *Mycobacterium tuberculosis* TKK-01-0090 | 2575205306 | T3pks-T1pks | 33 | ML-449 |
| *Mycobacterium tuberculosis* TKK-01-0091 | 2577024745 | T3pks-T1pks | 33 | ML-449 |
| *Mycobacterium tuberculosis* TKK-01-0093 | 2589736180 | T3pks-T1pks | 27 | Nystatin |
| *Mycobacterium tuberculosis* TKK-01-0094 | 2589727979 | T3pks, T1pks | 100 | MAR/MAP |
| *Mycobacterium tuberculosis* TRUG0004 | 2574675630 | T3pks-T1pks | 33 | ML-449 |
| *Mycobacterium tuberculosis* TRUG0037 | 2576105631 | T3pks-T1pks | 33 | ML-449 |
| *Mycobacterium tuberculosis* TRUG0040 | 2576986616 | T3pks-T1pks | 23 | Apoptolidin |
| *Mycobacterium tuberculosis* TRUG0070 | 2575285636 | T3pks-T1pks | 20 | Kendomycin |
| *Mycobacterium tuberculosis* TRUG0072 | 2584660713 | T3pks-T1pks | 20 | Kendomycin |
| *Mycobacterium tuberculosis* TRUG0076 | 2576070324 | T3pks-T1pks | 20 | Kendomycin |
| *Mycobacterium tuberculosis* TRUG0080 | 2584772421 | T3pks-T1pks | 33 | ML-449 |
| *Mycobacterium tuberculosis* TRUG0083 | 2584849650 | T3pks-T1pks | 23 | Jerangolid |
| *Mycobacterium tuberculosis* TRUG0085 | 2577313382 | T3pks-T1pks | 27 | Nystatin |
| *Mycobacterium tuberculosis* TRUG0088 | 2575638157 | T3pks-T1pks | 20 | Kendomycin |
| *Mycobacterium tuberculosis* TRUG0095 | 2584954771 | T3pks-T1pks | 33 | ML-449 |
| *Mycobacterium tuberculosis* TRUG0098 | 2574905673 | T3pks-T1pks | 33 | ML-449 |
| *Mycobacterium tuberculosis* TRUG0101 | 2576675825 | T3pks-T1pks | 33 | ML-449 |
| *Mycobacterium tuberculosis* TRUG0116 | 2584617586 | T3pks-T1pks | 33 | ML-449 |
| *Mycobacterium tuberculosis* TRUG0117 | 2584987406 | T3pks-T1pks | 50 | Piericidin A1 |
| *Mycobacterium tuberculosis* TRUG0124 | 2584854188 | T3pks-T1pks | 27 | Nystatin |
| *Mycobacterium tuberculosis* Uganda 1 | 2575403392 | T3pks-T1pks | 27 | Bafilomycin |
| *Mycobacterium tuberculosis* UG-C | 2575304538 | T3pks-T1pks | 23 | Jerangolid |
| *Mycobacterium tuberculosis* UG-D | 2584795634 | T3pks-T1pks | 28 | Laidlomycin |
| *Mycobacterium tuberculosis* UM 1072388579 | 2553261414 | T3pks | 62 | FD-891 |
| *Mycobacterium tuberculosis* UT205 | 2512786915 | T3pks-T1pks | 33 | ML-449 |
| *Mycobacterium tuberculosis* VRF CWCF XDRTB 1028 | 2598813154 | T3pks, T1pks | 100 | MAR/MAP |
| *Mycobacterium tuberculosis* W-148 | 2547759833 | T3pks-T1pks | 33 | ML-449 |
| *Mycobacterium tuberculosis* WX1 | 2560451827 | T1pks | 10 | Abyssomicin |
| *Mycobacterium tuberculosis* WX3 | 2560461324 | T3pks-T1pks | 33 | ML-449 |
| *Mycobacterium tuberculosis* X122 | 2547955540 | T3pks-T1pks | 33 | ML-449 |
| *Mycobacterium tuberculosis* XDR1219 | 2560449536 | T3pks-T1pks | 32 | ECO-02301 |
| *Mycobacterium tuberculosis* XDR1221 | 2560454644 | T3pks-T1pks | 23 | Jerangolid |
| *Mycobacterium tuberculosis* XTB13-081 | 2576698280 | T3pks-T1pks | 30 | Nanchangmycin |
| *Mycobacterium tuberculosis* XTB13-082 | 2584718757 | T3pks-T1pks | 30 | Nanchangmycin |
| *Mycobacterium tuberculosis* XTB13-086 | 2584755863 | T3pks-T1pks | 7 | Streptolydigin |
| *Mycobacterium tuberculosis* XTB13-088 | 2584641128 | T3pks-T1pks | 30 | Nanchangmycin |
| *Mycobacterium tuberculosis* XTB13-092 | 2584739857 | T3pks-T1pks | 33 | ML-449 |
| *Mycobacterium tuberculosis* XTB13-093 | 2576513411 | T3pks-T1pks | 27 | Nystatin |
| *Mycobacterium tuberculosis* XTB13-094 | 2575998368 | T3pks-T1pks | 33 | ML-449 |
| *Mycobacterium tuberculosis* XTB13-096 | 2576207497 | T3pks-T1pks | 23 | Jerangolid |
| *Mycobacterium tuberculosis* XTB13-100 | 2584918375 | T3pks-T1pks | 33 | ML-449 |
| *Mycobacterium tuberculosis* XTB13-110 | 2584936325 | T3pks-T1pks | 12 | Tiacumicin B |
| *Mycobacterium tuberculosis* XTB13-113 | 2584816678 | T3pks-T1pks | 30 | Nanchangmycin |
| *Mycobacterium tuberculosis* XTB13-114 | 2574794413 | T3pks-T1pks | 30 | Nanchangmycin |
| *Mycobacterium tuberculosis* XTB13-123 | 2578237814 | T3pks-T1pks | 12 | Tiacumicin B |
| *Mycobacterium tuberculosis* XTB13-127 | 2575365848 | T3pks-T1pks | 33 | ML-449 |
| *Mycobacterium tuberculosis* XTB13-131 | 2575697737 | T3pks-T1pks | 30 | Nanchangmycin |
| *Mycobacterium tuberculosis* XTB13-136 | 2576746511 | T3pks-T1pks | 33 | ML-449 |
| *Mycobacterium tuberculosis* XTB13-143 | 2576316581 | T3pks-T1pks | 30 | Nanchangmycin |
| *Mycobacterium tuberculosis* XTB13-156 | 2576649037 | T3pks-T1pks | 30 | Nanchangmycin |
| *Mycobacterium tuberculosis* XTB13-161 | 2577627248 | T3pks-T1pks | 30 | Nanchangmycin |
| *Mycobacterium tuberculosis* XTB13-162 | 2577876445 | T3pks-T1pks | 12 | Tiacumicin B |
| *Mycobacterium tuberculosis* XTB13-167 | 2577390930 | T3pks-T1pks | 30 | Nanchangmycin |
| *Mycobacterium tuberculosis* XTB13-175 | 2584686721 | T3pks-T1pks | 33 | ML-449 |
| *Mycobacterium tuberculosis* XTB13-194 | 2584659187 | T3pks-T1pks | 12 | Tiacumicin B |
| *Mycobacterium tuberculosis* XTB13-195 | 2576084283 | T3pks-T1pks | 30 | Nanchangmycin |
| *Mycobacterium tuberculosis* XTB13-198 | 2584883084 | T3pks-T1pks | 30 | Nanchangmycin |
| *Mycobacterium tuberculosis* XTB13-199 | 2577845812 | T3pks-T1pks | 23 | Jerangolid |
| *Mycobacterium tuberculosis* XTB13-200 | 2575942274 | T3pks-T1pks | 27 | Neoaureothin |
| *Mycobacterium tuberculosis* XTB13-203 | 2584808933 | T3pks-T1pks | 12 | Tiacumicin B |
| *Mycobacterium tuberculosis* XTB13-209 | 2575060404 | T3pks-T1pks | 30 | Nanchangmycin |
| *Mycobacterium tuberculosis* XTB13-214 | 2577974906 | T3pks-T1pks | 33 | ML-449 |
| *Mycobacterium tuberculosis* XTB13-238 | 2584822720 | T3pks-T1pks | 12 | Tiacumicin B |
| *Mycobacterium tuberculosis* XTB13-241 | 2576544659 | T3pks-T1pks | 30 | Nanchangmycin |
| *Mycobacterium tuberculosis* XTB13-251 | 2575099885 | T3pks-T1pks | 30 | Nanchangmycin |
| *Mycobacterium tuberculosis* XTB13-252 | 2584698776 | T3pks-T1pks | 12 | Tiacumicin B |
| *Mycobacterium tuberculosis* XTB13-255 | 2578213104 | T3pks-T1pks | 33 | ML-449 |
| *Mycobacterium tuberculosis* XTB13-290 | 2584960744 | T3pks-T1pks | 12 | Tiacumicin B |
| *Mycobacterium bovis Bz* 31150 | 2581587072 |  |  |  |
| *Mycobacterium bovis* Mr 4387 | 2580771058 |  |  |  |
| *Mycobacterium bovis* Wt 21231 | 2584003651 | T3pks-T1pks | 23 | Jerangolid |
| *Mycobacterium orygis* 112400015 | 2546436155 |  |  |  |
| ***Non-tuberculosis Mycobacteria (NTM)*** | | | | |
| *Mycobacterium sinense* JDM601 | 650873455 |  |  |  |
| *Mycobacterium marinum* Europe | 2543277028 |  |  |  |
| *Mycobacterium marinum* M, ATCC BAA-535 | 641717750 | T3pks-T1pks | 9 | Chalcomycin |
| *Mycobacterium marinum* E11 | 2588629254 | T3pks-T1pks | 60 | Meridamycin |
| *Mycobacterium marinum* MB2 | 2546369014 | T3pks-T1pks | 26 | Nystatin |
| *Mycobacterium liflandii* 128FXT | 2563569217 | T3pks-T1pks | 17 | Apoptolidin |
| *Mycobacterium kansasii* SMC1 | 2587480388 | T1pks | 23 | FR-008 |
| *Mycobacterium kansasii* 732 | 2567124714 | T3pks-T1pks | 10 | Abyssomicin |
| *Mycobacterium kansasii* ATCC 12478 | 2563577345 | T3pks-T1pks | 38 | Oligomycin |
| *Mycobacterium genavense* ATCC 51234 | 2545768030 | T3pks-T1pks | 28 | FR-008 |
| ***Mycobacterium avium* complex (MAC)** | | | | |
| *Mycobacterium avium hominissuis* 100 | 2580974538 | T3PKS | 66 | MAR/MAP |
| *Mycobacterium avium hominissuis* 101 | 2580006443 | T3pks-T1pks | 100 | MAR/MAP |
| *Mycobacterium avium* 05-4293 | 2581913245 | T3pks-T1pks | 30 | Nanchangmycin |
| *Mycobacterium avium* 09-5983 | 2573433552 |  |  |  |
| *Mycobacterium avium* 104 | 639736419 | T3pks-T1pks | 17 | Apoptolidin |
| *Mycobacterium avium* 10-5581 | 2572767979 | T3pks |  |  |
| *Mycobacterium avium* 2285 (R) | 2567079276 | T3pks | 10 | Meridamycin |
| *Mycobacterium avium hominissuis* A5 | 2580783837 | T3pks-T1pks | 17 | Apoptolidin |
| *Mycobacterium avium hominissuis* MAH 27-1 | 2581110378 |  |  |  |
| *Mycobacterium avium* MAV_061107_1842 | 2582181025 | T3pks-T1pks | 30 | Nanchangmycin |
| *Mycobacterium avium* MAV_120709_2344 | 2582391300 | T3pks-T1pks | 17 | Apoptolidin |
| *Mycobacterium avium* MAV_120809_2495 | 2580742569 | T3pks-T1pks | 17 | Apoptolidin |
| *Mycobacterium avium paratuberculosis* 10-4404 | 2592485489 | T3PKS | 100 | MAR/MAP |
| *Mycobacterium avium paratuberculosis* 10-5864 | 2570865822 | T3pks |  |  |
| *Mycobacterium avium paratuberculosis* 4B | 2550738610 |  |  |  |
| *Mycobacterium avium paratuberculosis* CLIJ623 | 2548578292 |  |  |  |
| *Mycobacterium avium paratuberculosis* CLIJ644 | 2547368463 |  |  |  |
| *Mycobacterium avium paratuberculosis* DT 3 | 2549377452 | T3pks-T1pks | 55 | Nigericin |
| *Mycobacterium avium* subsp. *paratuberculosis* K-10 | 637134331 |  |  |  |
| *Mycobacterium avium paratuberculosis* MAP4 | 2555735619 | T3pks-T1pks | 30 | Nanchangmycin |
| *Mycobacterium avium paratuberculosis* Pt139 | 2548515815 | T3pks |  |  |
| *Mycobacterium avium paratuberculosis* Pt146 | 2548530385 |  |  |  |
| *Mycobacterium avium paratuberculosis* Pt154 | 2548535921 |  |  |  |
| *Mycobacterium avium paratuberculosis* Pt164 | 2548547272 | T1pks | 100 | Micromonolactam |
| *Mycobacterium avium paratuberculosis* S5 | 2543326887 |  |  |  |
| *Mycobacterium* sp. MAC_011194_8550 | 2582203743 | T3pks |  |  |
| *Mycobacterium* sp. MAC_080597_8934 | 2576976958 | T3pks-T1pks | 23 | Apoptolidin |
| *Mycobacterium* sp. UM_CSW | 2555481387 |  |  |  |
| *Mycobacterium avium* subsp. *avium* 3388 | 2581397788 | T3pks-T1pks | 17 | Apoptolidin |
| *Mycobacterium avium* subsp. *avium* ATCC 25291 | 645425415 |  |  |  |
| Mycobacterium *avium avium* DT 78 | 2549389164 |  |  |  |
| *Mycobacterium avium avium* Env 77 | 2549393401 |  |  |  |
| *Mycobacterium avium paratuberculosis* Env 210 | 2549383420 |  |  |  |
| *Mycobacterium avium silvaticum* ATCC 49884 | 2569618768 |  |  |  |
