# Supplementary figures and images for: Cytochrome P450 Monooxygenase CYP139 Family Involved in the Synthesis of Secondary Metabolites in 824 Mycobacterial Species
Source: Int J Mol Sci. 2019 May 31;20(11):2690. doi: 10.3390/ijms20112690 (PMC6600245; doi:10.3390/ijms20112690)

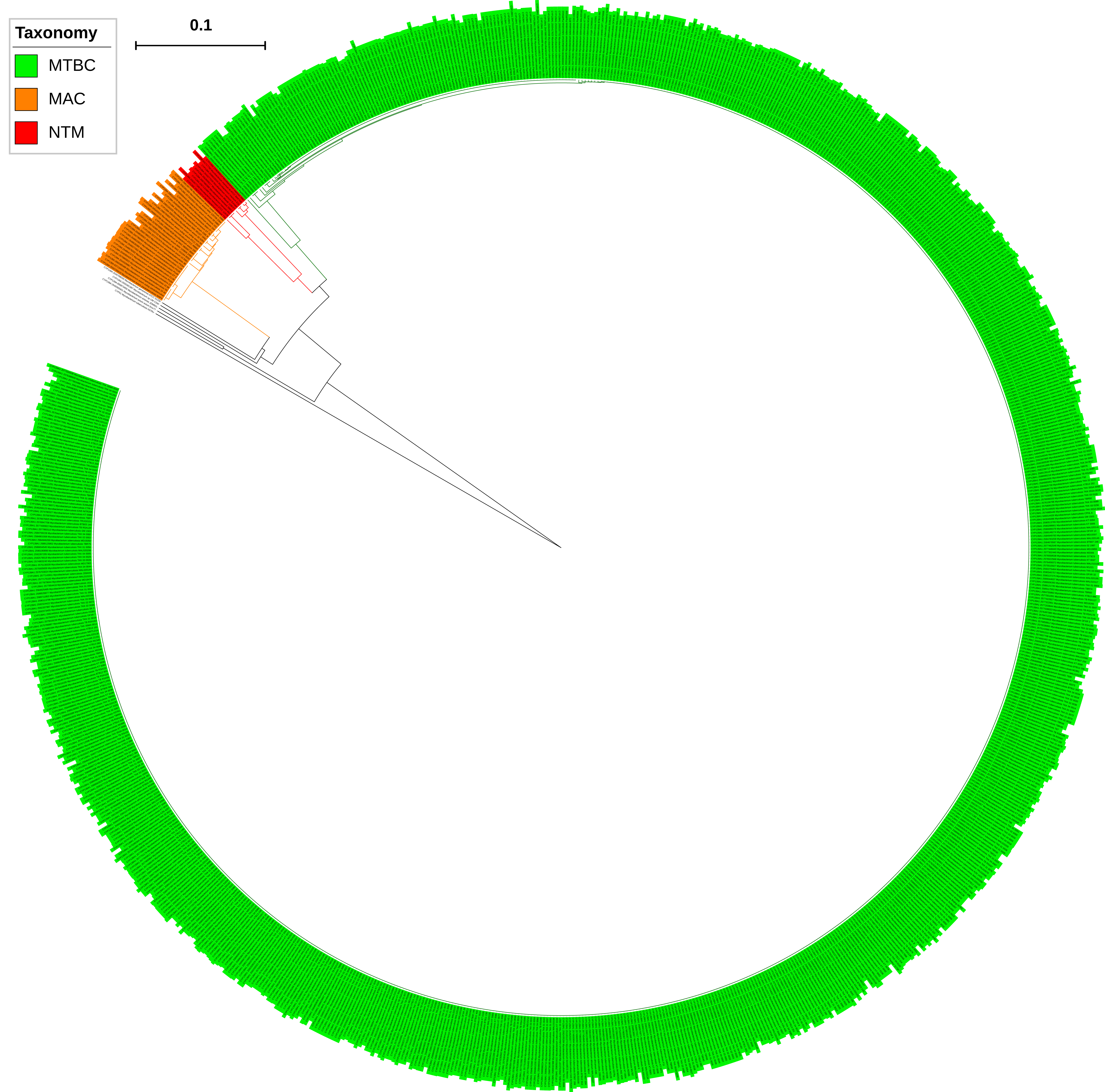

Supplement: Supplementary file 1 [file ijms-20-02690-s001.zip › Supplementary Information/Figure S1.tif]
